# Nitrate and ammonium lead to distinct global dynamic phosphorylation patterns when resupplied to nitrogen-starved Arabidopsis seedlings

**DOI:** 10.1111/j.1365-313X.2011.04848.x

**Published:** 2012-01-20

**Authors:** Wolfgang R Engelsberger, Waltraud X Schulze

**Affiliations:** Max Planck Institut für Molekulare PflanzenphysiologieAm Mühlenberg 1, 14476 Golm, Germany

**Keywords:** nitrogen, phosphorylation, membrane proteins, metabolic enzymes, proteomics, signaling

## Abstract

Nitrogen is an essential macronutrient for plant growth and development. Inorganic nitrogen and its assimilation products control various metabolic, physiological and developmental processes. Although the transcriptional responses induced by nitrogen have been extensively studied in the past, our work here focused on the discovery of candidate proteins for regulatory events that are complementary to transcriptional changes. Most signaling pathways involve modulation of protein abundance and/or activity by protein phosphorylation. Therefore, we analyzed the dynamic changes in protein phosphorylation in membrane and soluble proteins from plants exposed to rapid changes in nutrient availability over a time course of 30 min. Plants were starved of nitrogen and subsequently resupplied with nitrogen in the form of nitrate or ammonium. Proteins with maximum change in their phosphorylation level at up to 5 min after nitrogen resupply (fast responses) included GPI-anchored proteins, receptor kinases and transcription factors, while proteins with maximum change in their phosphorylation level after 10 min of nitrogen resupply (late responses) included proteins involved in protein synthesis and degradation, as well as proteins with functions in central metabolism and hormone metabolism. Resupply of nitrogen in the form of nitrate or ammonium resulted in distinct phosphorylation patterns, mainly of proteins with signaling functions, transcription factors and transporters.

## Introduction

As sessile organisms, plants need to rapidly adapt to changes in the environment such as light fluctuations and alterations in nutrient availability. Nitrogen is an important macronutrient to ensure plant growth and development as it is a component of proteins and nucleic acids, and many co-factors and secondary metabolites. Plants have the potential for adaptation to reduced nitrogen availability by increasing the capacity for nutrient acquisition and by alteration of whole-plant morphology and metabolism, such as increasing the root/shoot ratio or anthocyanin accumulation in leaves (Rubio *et al.*, 2009). Developmental adaptive mechanisms stimulate growth in organs that directly participate in nutrient acquisition, such as primary roots (Walch-Liu and Forde, 2008). To trigger these adaptive responses and to induce fast switching from starvation metabolism to nutrient assimilation, the nutrient itself or its primary assimilation products serve as signaling molecules (Crawford, 1995; Scheible *et al.*, 1997b; Schulze *et al.*, 1994; Stitt, 1999).

Nitrate starvation itself, as well as nitrate resupply after starvation, induces global changes of gene expression within 30 min (Krapp *et al.*, 2011; Scheible *et al.*, 2004). Recent studies of nitrate-induced transcriptional changes revealed very fast responses as early as 3 min after resupply (Krouk *et al.*, 2010). Supply of external nitrate directly affects its uptake and assimilation by regulating the gene expression of nitrate transporters, nitrate reductase, nitrite reductase and pathways for production of reducing equivalents, such as the pentose phosphate pathway and glycolysis. Nitrate also regulates the expression of genes involved in carbon metabolism, thereby coordinating the production of organic acids required for inorganic nitrogen assimilation (Gutierrez *et al.*, 2007).

The nitrate transporter NRT1.1 has a direct role in nitrate sensing (Ho *et al.*, 2009), and its phosphorylation status at T101 is dependent on external nitrate concentration. NRT1.1 is highly phosphorylated under low-nitrogen conditions and then rapidly dephosphorylated when nitrogen is supplied at higher concentrations. Phosphorylation at T101 is required to activate the sensing function of NRT1.1 and to switch from low-affinity transport to high-affinity uptake (Liu and Tsay, 2003). However, most studies on nitrate signaling have focused on expression changes of the nitrate transporter NRT2.1, which is a high-affinity nitrate transport system (Filleur *et al.*, 2001). NRT2.1 expression is transiently up-regulated by nitrogen deficiency (Girin *et al.*, 2007; Lejay *et al.*, 1999), and there is evidence for regulation also at the post-translational level (Wirth *et al.*, 2007). Due to the direct inhibition of NRT2.1 expression by nitrate, NRT2.1 expression has been used as means of screening for nitrate signaling mutants (Girin *et al.*, 2010). Expression of NRT1.1 and NRT2.1 is also rapidly induced by nitrate resupply after starvation (Krouk *et al.*, 2011; Scheible *et al.*, 2004).

Similarly, expression of the ammonium transporter AMT1.1 is strongly up-regulated by nitrogen starvation, and expression is reduced upon ammonium supply. AMT1.1 is responsible for cellular ammonium acquisition as well as ammonium retrieval, thus serving as the major high-affinity ammonium transporter in Arabidopsis roots (Gazzarrini *et al.*, 1999; Ninnemann *et al.*, 1994). Studies correlating transcript or protein abundance with ammonium influx or employing promoter fusions to reporter genes indicate that transcriptional control in response to nitrogen and carbon nutritional status is a major regulatory mechanism for ammonium transporters in plants (Yuan *et al.*, 2006). Recently, it was shown that ammonium uptake is also regulated at the post-transcriptional level by transporter *trans-*inactivation through phosphorylation of a C-terminal threonine (Lanquar *et al.*, 2009; Loque *et al.*, 2007). Reversible phosphorylation of nitrogen transporters thus provides a powerful fast mechanism for regulating their sensitivity according to changes in nitrogen availability (Walch-Liu and Forde, 2008).

In general, many molecular components of nitrogen-induced signaling processes remain unknown. Nitrate and ammonium can induce direct expression changes in their respective uptake systems and trigger immediate downstream responses involving kinases and transcription factors (Gojon *et al.*, 2009). However, complexity is introduced by feedback repression of nitrogen uptake and metabolism by nitrogen-containing metabolites. Furthermore, several microRNA targets have been found to be regulated by nitrate, suggesting that microRNAs are involved in systemic signaling of nitrogen status between roots and shoots (Gifford *et al.*, 2008; Liu *et al.*, 2009).

Most efforts so far to characterize signaling components of nitrogen-induced responses have made use of genetic tools and monitored changes in gene expression. However, a significant part of the immediate regulation occurs through post-translational modifications of proteins. Enzymes of nitrate assimilation are regulated by protein phosphorylation (Kaiser and Huber, 2001), and the transporters for nitrate and ammonium can also be directly regulated by phosphorylation events (Ho *et al.*, 2009; Lanquar *et al.*, 2009; Loque *et al.*, 2007). Thus, analysis of time-resolved protein phosphorylation patterns is expected to identify additional candidate proteins that are involved in nitrogen-induced sensing and signaling processes. Our work thus complements existing large-scale analyses of nitrogen-induced transcriptional changes.

Quantitative analyses of stimulus-induced protein phosphorylation patterns have been performed to study sucrose-induced phosphorylation (Niittylä*et al.*, 2007), cellular responses to elicitor treatment (Benschop *et al.*, 2007; Nühse *et al.*, 2007), cellular responses to phytohormones (Chen *et al.*, 2010; Kline *et al.*, 2010; Tang *et al.*, 2008) and to compare responses to light and dark conditions in chloroplasts (Reiland *et al.*, 2009). Label-free techniques and stable isotope labeling have been used for such quantitative comparison. Here, we present results from a systematic analysis of dynamic changes in protein phosphorylation induced by resupply of various nitrogen sources to nitrogen-starved Arabidopsis seedlings over a period of up to 30 min.

## Results

### Nitrogen starvation of seedlings

**Figure 1:**
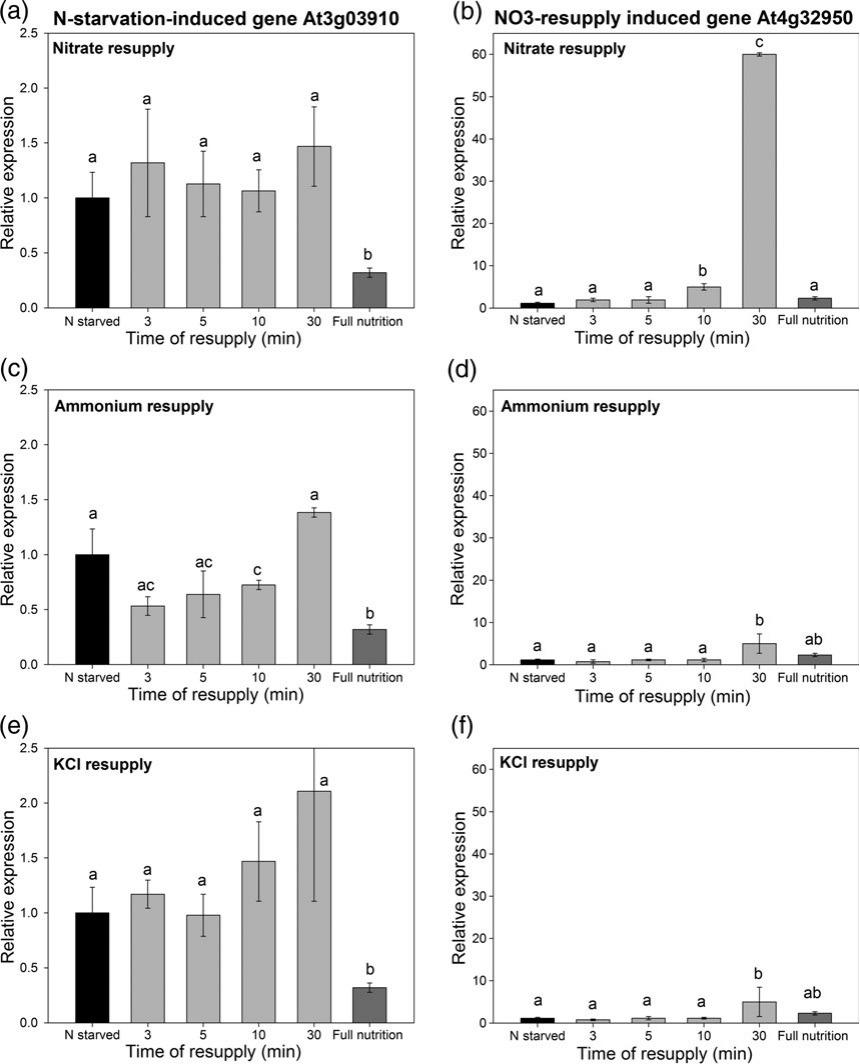
Analysis of gene expression by real-time PCR. *At3g03910* was used as an example of a nitrogen starvation-induced gene, while *At4g32950* was used as an example of a gene induced by nitrate resupply after starvation. (a–c) Expression of *At3g03910* in nitrogen-starved seedlings and after nitrate resupply (a), ammonium resupply (b) and KCl treatment (c). (d–f) Expression of *At4g32950* in seedlings subjected to nitrate resupply (a), ammonium resupply (b) and KCl treatment (c). Values are means ± standard deviation of three biological replicate experiments. Different letters indicate significant differences (*P* < 0.05, Student's *t*-test).

For nitrogen starvation and resupply experiments, seedlings were grown in liquid culture. Under these growth conditions, the nitrogen-starved seedlings displayed typical phenotypes of nitrogen starvation, such as long roots, pale leaves and anthocyanine accumulation. The nitrogen starvation status, as well as induction of nitrogen resupply, was monitored by expression of characteristic marker genes (Scheible *et al.*, 2004). Expression of *At3g03910* (a putative glutamate dehydrogenase) was up-regulated in all seedlings starved of nitrogen (Figure 1a,c,e). In contrast, expression of *At4g32950* (a putative protein phosphatase 2C) was strongly induced upon resupply of nitrate starting at 10 min of nitrate resupply and increasing over a period of 30 min (Figure 1b). Expression of this known nitrate resupply-induced gene was not induced by ammonium resupply or the addition of KCl (Figure 1d,f). This is in agreement with previously published results on nitrogen-starvation induced genes and gene expression induced by nitrate (Scheible *et al.*, 2004).

The up-regulated expression of nitrogen starvation-induced gene *At3g03910* in all treatments throughout the time course of nitrate and ammonium resupply (Figure 1a,c) suggested that nitrogen resupply for short time periods did not alter the general ‘starvation status’ of the plant, although nitrate-induced transcriptional changes have been observed as early as 3 min after resupply (Krouk *et al.*, 2010). However, particularly at the last time point (30 min of nitrogen resupply), changes in gene expression began to affect nitrogen transport and nitrogen metabolism, as indicated by the strong up-regulation of *At4g2950* upon nitrate resupply (Figure 1b) and as described previously (Krouk *et al.*, 2010). Thus, the early changes in phosphorylation most likely occur in parallel with fast transcript changes, and particularly at the later time points of 10 and 30 min, we may observe overlying effects due to onset of transcriptional changes. These possible changes in total protein abundance have been corrected for by normalizing the ion intensities of phosphopeptides to the mean ion intensities of non-phosphopeptides as described previously (Niittylä*et al.*, 2007).

### Global dynamic phosphorylation responses to nitrogen resupply

In total, from four biological replicate time-course experiments each with ammonium or nitrate resupply and from two biological replicates with KCl supply, 11 693 peptides corresponding to 6164 proteins were identified by LC-MS/MS. Of these, 1225 unique phosphopeptides were identified based on the criteria described in Experimental Procedures (Table S1). The phosphopeptides comprised 773 unique peptides identified in seedlings resupplied with nitrate, 869 peptides identified upon ammonium resupply, and 620 peptides identified upon treatment with KCl. A total of 436 peptides were identified under all three conditions (Figure S1a). The overlap of phosphopeptides identified in experiments with nitrate and ammonium resupply was significantly larger than the overlap with KCl treatment. This suggests that resupply of nitrogen as either nitrate or ammonium induces phosphorylation of a large common set of proteins that is distinct from the set of proteins induced by a non-nitrogen-related ionic treatment, such as KCl.

For analysis of time-resolved response profiles, proteins were only considered if they were quantified for at least three of the five time points after re-addition of nitrate, ammonium or KCl. Thus, of the 1225 phosphopeptides identified in all experiments, we obtained time-resolved abundance profiles for 588 unique phosphopeptides. These phosphopeptides comprised 332 peptides from the experiments with nitrate resupply, 386 peptides from experiments with ammonium resupply, and 228 peptides from experiments with KCl addition (Figure S1b).

The time-resolved abundance profiles of normalized ion intensities for individual phosphopeptides showed responses common to all three treatments, as well as responses specifically induced by nitrate, ammonium or KCl, respectively. For example, phosphopeptides quantified for all treatments included peptides from transporters, such as peptide (pT)LHGLQPK of plasma membrane ATPase AHA1 (AT2G18960) and AHA2 (AT4G30190) and peptide QTTAEGSANPEPDQIL(pS)PR of purine transporter PUP18 (AT1G57990), as well as peptides (pS)QLHELHA and ALG(pS)FR(pS)NATN from aquaporin isoforms.

**Figure 2:**
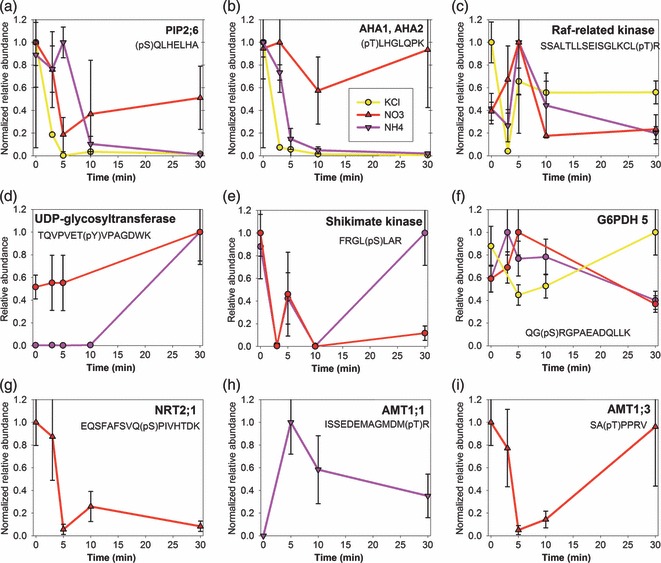
Examples of commonly quantified proteins with distinct time-response profiles upon resupply of nitrate (red), ammonium (pink) or KCl (yellow). (a) Aquaporins. (b) AHA1/AHA2. (c) Raf-related kinase (AT1G04210). (d) UDP-glucosyl transferase (AT2G36800). (e) Shikimate kinase (AT2G21940). (f) Glucose-6-phosphate dehydrogenase (AT3G27300). (g) Nitrate trans (AT2G18960/AT4G30190) porter NRT2;1 (AT1G08090). (h) Ammonium transporter AMT1;1 (AT4G13510) *trans*-inactivation site. (i) Ammonium transporter AMT1;3 (AT3G24300) C-terminal phosphorylation site.

These peptides (pS)QLHELHA, ALG(pS)FR and ALG(pS)FR(pS)NATN correspond to the conserved phosphorylation sites in aquaporins that control pore gating (Tornroth-Horsefield *et al.*, 2006). Upon phosphorylation, the pore is open; dephosphorylation leads to closing of the pore. External supply of KCl lead to very rapid dephosphorylation of these phosphorylation sites. Upon re-addition of nitrogen in the form of ammonium, aquaporins were also rapidly dephosphorylated, but with a delay of approximately 5 min. Nitrate resupply had the weakest effect on pore closing of aquaporins, with maximum dephosphorylation occurring at 5 min of nitrate supply and a subsequent tendency towards an increase in the phosphorylation level over time (i.e. re-opening of the pores) (Figure 2a).

Another example of proteins whose phosphorylation was affected by all three treatments is the plasma membrane ATPases AHA1 and AHA2, at phosphorylation site T881 (pTLHGLQPK). Phosphorylation of T881 is associated with activation of the proton pumps (Niittylä*et al.*, 2007). Rapid dephosphorylation of T881 was observed in cases of KCl addition and also upon ammonium resupply to nitrogen-starved seedlings. This could be explained as an inactivation of plasma membrane ATPase upon a strong depolarization of the membrane potential during uptake of cations. In contrast, re-addition of nitrate only induced a short transient decrease in phosphorylation status of T881, and the phosphorylation level of T881 returned to that in nitrogen-starved cells after 30 min (Figure 2b). In addition, a protein that functions as a Raf-related kinase (AT1G04210) showed similar phosphorylation profiles under both types of nitrogen resupply, but showed a clearly distinct phosphorylation time course under conditions of KCl supply (Figure 2c).

Other proteins with changes in phosphorylation status upon both nitrate and ammonium resupply were mainly found to function in primary nitrogen assimilation metabolism, amino acid synthesis, nucleotide metabolism and tetrapyrrole synthesis. For example, time-resolved phosphorylation patterns were recorded in nitrate- and ammonium-resupplied seedlings for peptides from UDP-glucosyl transferase (Figure 2d), shikimate kinase (Figure 2e), glucose-6-phosphate dehydrogenase (Figure 2f), as well as nitrate reductase and isoforms of glutamine synthase (see below).

**Figure 3:**
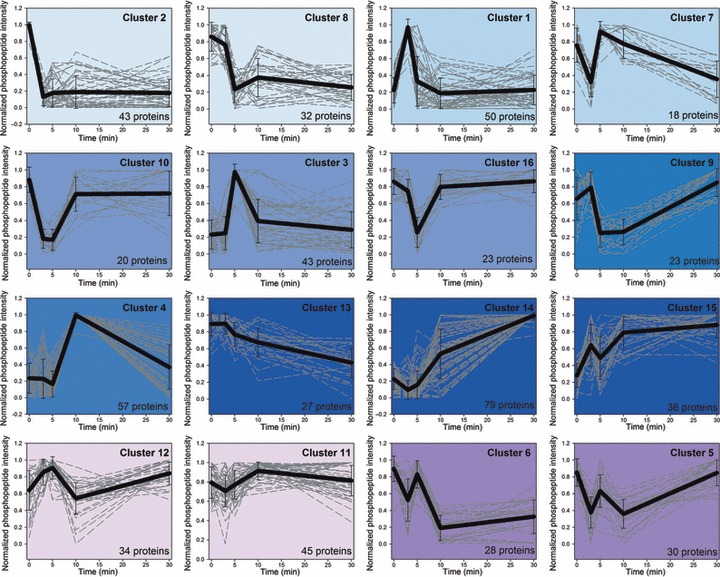
k-means clustering of time-course profiles of all phosphopeptides. A total of 16 clusters were formed using an Euclidean distance matrix. Different colors indicate response groups with maximal or minimal phosphorylation at the respective time points.

The phosphorylation profiles comprised the nitrogen-starved condition (time point 0) and various durations of resupply (3, 5, 10 and 30 min) of nitrate, ammonium or KCl. All profiles of phosphorylation change with more than four data point values (588 phosphopeptides) were grouped using k-means clustering (Figure 3). As both dephosphorylation (decrease in the phosphorylation level) and phosphorylation (increase in the phosphorylation level) may have activating or inactivating effects on downstream targets, the 16 distinct clusters were combined into larger response groups consisting of clusters with maxima or minima of normalized phosphopeptide intensity at the individual time points. Thus, two clusters showed a phosphorylation maximum at time point 0 (clusters 2 and 8), two clusters showed maximum or minimum phosphorylation changes at 3 min (clusters 1 and 7), two clusters showed phosphorylation peaks at 5 min (clusters 3 and 16), two clusters showed peaks at 10 min (clusters 4 and 9) and three clusters showed a slow increase or decrease of phosphorylation over 30 min (clusters 13, 14 and 15). Four clusters showed oscillating phosphorylation responses (clusters 5 and 6) or displayed an almost constant phosphorylation profile (clusters 11 and 12). Cluster 10 showed a phosphorylation minimum at 3 and 5 min (Figure 3).

**Figure 4:**
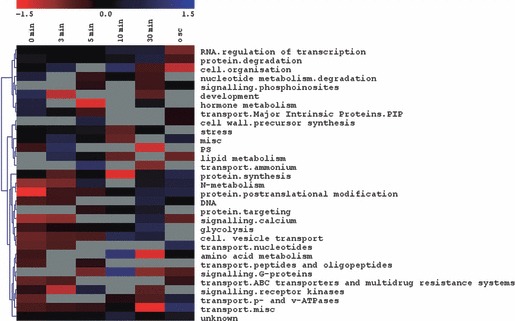
Heatmap of the relative abundance of functional categories in different response groups of phosphorylation. Nitrate and ammonium responses were analyzed together. A Euclidean distance matrix with mean linkage distance was used.

Within each response group, proteins were functionally classified according to MapMan (Thimm *et al.*, 2004), and the relative abundance of proteins within each functional bin was expressed as *z*-scores to identify patterns of over-representation of specific cellular functions within response groups (Figure 4). Proteins with a phosphorylation maximum under nitrogen-starved conditions (time point 0) included those with functions in nucleotide degradation (e.g. salvage pathway), hormone metabolism and development, as well as aquaporins. Obviously, these proteins with very fast changes in phosphorylation status are mainly involved in general adaptations to the new nutritional and osmotic conditions, such as inactivation of typical starvation responses, for example dephosphorylation of enzymes in the salvage pathways, as well as immediate adaptations to alterations in external solute concentration (e.g. regulation of aquaporin and transporter activities). The nitrate transporter NRT2.1 was also rapidly dephopsphorylated after only 3 min of nitrate supply.

Proteins with a transient change in phosphorylation after 3 min were involved in lipid metabolism, especially of ‘special lipids’, such as sterols, glycolipids, squalenes and sphingolipids (MapMan bin ‘lipid metabolism.exotics’; e.g. AT5G23450, a sphingosine kinase). The functional category ‘misc’ includes mainly proteins with as yet uncharacterized functions. Many of these proteins, such as AT1G78800, a UDP-glucosyl transferase, form part of sterol-rich membrane microdomains (Kierszniowska *et al.*, 2009). Proteins with a transient change in phosphorylation at 5 min of nitrate or ammonium resupply included receptor-like kinases (AT3G21990, AT5G41300, AT3G46330 and AT1G55200), proteins with functions in cell-wall precursor synthesis, and ammonium transporter AMT1.

In contrast, proteins with functions in vesicle transport, G-protein signaling, cell organization and transcription factors showed a peak of transient change in phosphorylation at 10 min. Proteins with a slow change in phosphorylation status over 30 min mainly included proteins involved in calcium signaling, central metabolism (nitrogen metabolism and glycolysis) and protein synthesis, and proteins affecting DNA structure and synthesis (DNA polymerases and histones, e.g. AT1G10520). Some proteins, such as ribosomal proteins (MapMan bin ‘protein.synthesis’), several kinases and phosphatases (MapMan bin ‘protein.posttranslational modification’) and ABC transporters (MapMan bin ‘transport.misc’) were over-represented among proteins with an oscillating phosphorylation pattern.

**Figure 5:**
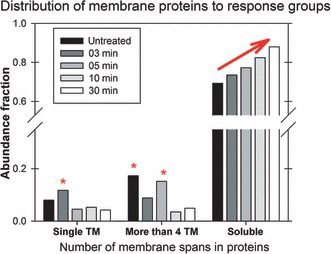
Number of proteins with one or more than four membrane spans and number of soluble proteins expressed as a proportion of all proteins identified.

In general, the protein composition of the phosphorylation response groups indicated progression of phosphorylation ‘waves’ from the membrane to the cell interior with an increasing duration of nitrogen resupply. Early transient changes in phosphorylation status within 3–5 min affected proteins with functions in membrane lipid metabolism, transporters, membrane-bound kinases and transcription factors. At later time points (after 10–30 min), phosphorylation changes affected mainly cytosolic signaling proteins, and proteins involved in protein synthesis and degradation, as well as those involved in central metabolism. This trend for progression of the phosphorylation peak time from membrane proteins to cytosolic proteins was apparent in the distribution of soluble proteins and membrane proteins to different phosphorylation response groups (Figure 5). We observed a clear increase in the proportion of soluble proteins from the response group with a phosphorylation peak at 3 min to the response group with a phosphorylation peak at 30 min. The group of proteins for which phosphorylation peaked at 3 min included the highest proportion of proteins with single transmembrane spans. These included receptor-like kinases, RING-type ubiquitin ligases (e.g. AT1G20823), proteins with functions in lipid modification and cell-wall proteins (e.g. AT1G78400 and AT1G78800). Proteins with more than four transmembrane spans, such as transporters, were more frequently observed among proteins with early (up to 5 min) transient changes in phosphorylation.

Consistently, the transporter NRT2.1 for high-affinity nitrate uptake was rapidly de-phosphorylated after 3 min of nitrate resupply (Figure 2g). We investigated the phosphorylation of the high-affinity transporter NRT2.1 in more detail under conditions of resupply of various concentrations of nitrate for 10 min. In starved cells, protein phosphorylation at S28 of NRT2.1 was high, and remained high upon resupply of 0.3 mm nitrate (Figure S2). Upon resupply of higher concentrations of nitrate (3 or 10 mm), the concentration range used in the present starvation/resupply phosphorylation screen, NRT2.1 phosphorylation decreased. Under these conditions, low-affinity transport systems are expected to contribute the majority of nitrate influx. Interaction of NRT2.1 with the protein NAR2.1 is required for NRT2.1 transporter function (Orsel *et al.*, 2006, 2007; Wirth *et al.*, 2007), and protein phosphorylation may influence this protein–protein interaction. Phosphorylation may also be involved in NRT2.1 dimerization, targeting for protein degradation or direct activity regulation. Understanding the precise role of NRT2.1 phosphorylation at S28 will require further in-depth studies of site-directed mutants.

The transporter AMT1;1 for ammonium uptake also showed rapid changes in phosphorylation at early time points of nitrate or ammonium resupply (Figure 2h,i). In addition to the known phosphorylation site in ammonium transporters with *trans-*inactivating functions (Figure 2h), we identified a number of further phosphorylation sites in the C-terminus of AMT1;1 and AMT1;3 that were altered upon nitrate addition (Figure 2i; see Table S1 for full list). The precise function of these multiple C-terminal phosphorylation sites in ammonium transporters remains to be elucidated.

### Nitrate- and ammonium-specific phosphorylation patterns

To define nitrate- and ammonium-specific phosphorylation responses, proteins were considered that (i) were specifically phosphorylated under either nitrate or ammonium resupply, (ii) were identified as non-phosphorylated peptides under the respective other nutrient condition (Figure S3), or (iii) were not identified under conditions of KCl supply or displayed a clearly different profile under KCl treatment. An over-representation analysis revealed that proteins specifically phosphorylated under nitrate resupply conditions and not phosphorylated upon ammonium resupply included those with functions in phosphatidylinositol signaling, cell-wall degradation and the Calvin–Benson cycle, and transcription factors of the zinc finger group (Table 1). In contrast, proteins specifically phosphorylated upon ammonium resupply and not phosphorylated upon nitrate resupply included aquaporins, receptor-like kinases and proteins involved in secondary metabolism and aromatic amino acid synthesis. As expected, proteins involved in nitrogen assimilation, tetrapyrrole synthesis, nucleotide synthesis and proton ATPases were over-represented among the proteins phosphorylated under both types of nitrogen resupply. We thus observed phosphorylation patterns under conditions of both nitrate and ammonium resupply mainly for central nitrogen metabolism, whereas specific phosphorylation responses affected mainly specific transporters, signaling components and transcription factors (Table 1).

**Table 1:** Over-representation analysis of protein functions specifically phosphorylated by either nitrate or ammonium resupply

NO_3_ resupply				Overlap of NO_3_ and NH_4_		NH_4_ resupply			
Specific presence and specific phosphorylation		Specific phosphorylation; presence under both conditions		Phosphorylation under both conditions		Specific phosphorylation; presence under both conditions		Specific presence and specific phosphorylation	
MapMan bin	*P-*value	MapMan bin	*P-*value	MapMan bin	*P-*value	MapMan bin	*P-*value	MapMan bin	*P-*value
Lipid metabolism.lipid degradation.lipases	0.0003	PS.calvin cycle	0.0014	Transport.p- and v-ATPases.H+-exporting ATPase	0.0023	Transport.major intrinsic proteins.PIP	0.0155	Transport.ABC transporters and multidrug resistance systems	0.0018
RNA.regulation of transcription	0.0008	Lipid metabolism.‘exotics’ (steroids.squalene etc.).*trans-*2-enoyl-CoA reductase (NADPH)	0.0091	Secondary metabolism.wax	0.0045	Secondary metabolism.isoprenoids	0.0162	RNA.regulation of transcription.Polycomb Group (PcG)	0.0060
Secondary metabolism.isoprenoids.terpenoids	0.0089	Nucleotide metabolism.synthesis.purine.GMP synthetase	0.0091	N-metabolism.ammonia metabolism.glutamine synthase	0.0109	Amino acid metabolism.synthesis.aromatic aa	0.0337	Stress.abiotic.touch/wounding	0.0060
Protein.degradation.ubiquitin.E3	0.0128	RNA.regulation of transcription.C3H zinc finger family	0.0181	Protein.targeting.nucleus	0.0151	PS.photorespiration.glycerate kinase	0.0162	Not assigned	0.0120
RNA.regulation of transcription.WRKY domain transcription factor family	0.0161	Cell wall.degradation	0.0214	Nucleotide metabolism.synthesis.pyrimidine	0.0281	Major CHO metabolism.degradation	0.0172	Protein.synthesis	0.0123
Lipid metabolism.phospholipid synthesis	0.0253	Signaling.phosphinositides.phosphatidylinositol-4-phosphate 5-kinase	0.0448	Tetrapyrrole synthesis	0.0281	RNA.regulation of transcription	0.0294	Cell wall	0.0234
RNA.regulation of transcription.unclassified	0.0282			Lipid metabolism.lipid degradation	0.0281	Secondary metabolism.N misc.betaine	0.0322	RNA.processing.RNA helicase	0.0244
Protein.postranslational modification.kinase	0.0392			N-metabolism	0.0498	Transport.major intrinsic proteins	0.0337	RNA.regulation of transcription.MYB domain transcription factor family	0.0273
RNA.regulation of transcription.bZIP transcription factor family	0.0414					Cell.organization	0.0359	Signaling.receptor kinases.misc	0.0377
						Signaling.receptor kinases.leucine rich repeat V	0.0479	DNA	0.0410
						Lipid metabolism.‘exotics’ (steroids.squalene etc.).sphingolipids	0.0479	Redox.regulation	0.0440

*P*-values were obtained using a Fisher exact test and were corrected for multiple testing (Benjamini and Hochberg, 1995).

### Pathways with altered phosphorylation

In most cases, the kinases responsible for phosphorylation of nitrogen uptake transporters remain unknown. Calcineurin-B like (CBL)-CBL-interacting protein kinase (CIPK) kinases have been identified as candidates for regulation of potassium channels through phosphorylation (Lee *et al.*, 2007), and calcium-dependent protein kinases have been studied intensively as plant-specific kinases in various stress responses, often also being membrane-associated (Ludwig *et al.*, 2004). With regard to metabolic signaling, the Snf1-related protein kinases (SnRKs) are central players linking stress responses and metabolic signaling (Fragoso *et al.*, 2009; Halford and Hey, 2009; Shin *et al.*, 2007), and the MAP kinase pathway is among the most well-studied central signaling pathway, with functions in biotic stress responses, plant defense, hormone signaling, senescence and development. Here, we identified phosphorylated members of all the above-mentioned kinase families (Table 2). For example, CIPK2 was phosphorylated in nitrate-resupplied seedlings after 5 min, while CPK27 was phosphorylated in ammonium-resupplied seedlings after 5 min. SNRK2.4 was phosphorylated after nitrate resupply at 10 and 30 min, and two phosphorylation sites in MKK2 were identified after nitrate and ammonium resupply.

**Table 2:** Known kinases and phosphorylation sites identified as phosphorylated upon nitrate or ammonium resupply after nitrogen starvation

Kinase family**RLKs**	AGI	Phosphopeptide	NH_4_ resupply	NO_3_ resupply	Description
			**23**	**18**	
	AT1G33260.1	DGSIDLEEVK(pT)MLR		x	Protein kinase family protein
	AT1G34420.1	LEV(pS)DN(pS)LSGTIPEGIK	x		Leucine-rich repeat family protein/protein kinase family protein
	AT1G51800.1	(pS)HHGFEPPVIAK		x	Leucine-rich repeat protein kinase
	AT1G55200.1	K(pS)QANWVVLDK		x	Protein kinase family protein
	AT1G70130.1	ILAI(pS)L(pS)I(pS)GV(pT)LVIVLILGV(oxM)LFLK	x		Lectin protein kinase
	AT2G01820.1	LLDV(pS)NNDF(pY)GIPPKFR	x		Leucine-rich repeat protein kinase
	AT2G07040.1	LGRLNHENLLPIVA(y)(y)YK	X	x	PRK2A (pollen receptor kinase)
	AT2G11520.1	LIITE(pY)VRNGTLR	X		CRCK3 (CALMODULIN-BINDING RECEPTOR-LIKE CYTOPLASMIC KINASE 3)
	AT2G13790.1	LE(pS)LVDAELEGK	X		SERK4 (SOMATIC EMBRYOGENESIS RECEPTOR-LIKE KINASE 4)
	AT2G19190.1	(pS)ILANGDIR		x	FRK1 (FLG22-INDUCED RECEPTOR-LIKE KINASE 1)
	AT2G23300.1	ELEVE(pT)LLK	X	x	Leucine-rich repeat transmembrane protein kinase
	AT2G23770.1	AKIG(pS)LGSAR	X	x	Protein kinase family protein/peptidoglycan-binding LysM domain-containing
	AT2G32800.1	LG(oxM)(pT)KCPALVTR			AP4.3A; protein serine/threonine kinase
	AT3G02880.1	LIEEVSHSSG(pS)PNPV(pS)D		x	Leucine-rich repeat transmembrane protein kinase
	AT3G08680.1	GI(pS)HIH(pS)ASGAKLLHGNIK	X	x	Leucine-rich repeat transmembrane protein kinase
	AT3G14350.1	DGNLLNSGPAPPPPPG(pT)PPISK	X		SRF7 (STRUBBELIG-RECEPTOR FAMILY 7)
	AT3G21990.1	VSAMVQC(pT)K	X		Receptor-like protein kinase-related
	AT3G23750.1	GGFGVV(y)AGELHDG(t)KTAVK	X	x	Leucine-rich repeat protein kinase family protein
	AT3G46330.1	SLLVINL(pS)GNK	X		MEE39 (MATERNAL EFFECT EMBRYO ARREST 39)
	AT3G47570.1	V(pT)HLELGR	X	x	Leucine-rich repeat transmembrane protein kinase
	AT4G08850.1	VTEIAI(pY)DNLL(pT)GPIPSSFGNLTK	X	x	Leucine-rich repeat receptor-like protein kinase
	AT4G23190.1	QLKLVSEGSESDQYT(pS)K	X		CRK11 (CYSTEINE-RICH RLK11)
	AT4G34220.1	STAPINPLTEKPNQ(pT)GK(pS)K	X		Leucine-rich repeat transmembrane protein kinase
	AT5G07150.1	LREI(pT)GI(pT)PEAALPSR	X	x	Leucine-rich repeat protein kinase
	AT5G10530.1	GEVI(pT)AIDEKLR	X	x	Lectin protein kinase
	AT5G37450.1	DHV(pT)TIVK	X	x	Leucine-rich repeat transmembrane protein kinase
	AT5G41300.1	SSTLS(pS)ALTPYYYLDVTR	X		Receptor-like protein kinase-related
	AT5G58150.1	LSALH(pY)LNLSR		x	Leucine-rich repeat transmembrane protein kinase
	AT5G61350.1	INIGGDLI(pS)PK	X	x	Protein kinase family protein
	AT5G65830.1	LFG(pY)PLEEMKNK	X	x	Leucine-rich repeat protein kinase
**SNRKs**			**–**	**1**	
	AT1G10940.1	(s)(t)VGTPAYIAPEVLSR		X	SNRK2.4 (SNF1-RELATED PROTEIN KINASE 2.4)
**BSKs**			**–**	**1**	
	AT4G35230.1	SY(pS)TNLAYTPPEYLR		X	BSK1 (BR-SIGNALING KINASE 1)
**CIPKs**			**1**	**1**	
	AT5G07070.1	HPNVVEL(pY)EV(oxM)ATKSR		X	CIPK2 (CBL-INTERACTING PROTEIN KINASE 2)
	AT5G35410.1	EPSEIIANIEAVAN(pS)(oxM)GFK	x		CIPK24 (CBL-INTERACTING PROTEIN KINASE 24)
**CPKs**			**3**	**1**	
	AT3G20410.1	LESNENL(pY)K	x		CPK9 (CALMODULIN-DOMAIN PROTEIN KINASE 9)
	AT3G56760.1	TAILKSS(pT)EATK	x	X	Calcium-dependent protein kinase
	AT4G04700.1	IYILGEELGRGNFGL(pT)R	x		CPK27 (CALCIUM-DEPENDENT PROTEIN KINASE 27)
**MAPK pathway**			**2**	**1**	
	AT1G09000.1	GPLGG(s)P(s)RATDAT(s)C(s)K	X		ANP1 (ARABIDOPSIS NPK1-RELATED PROTEIN KINASE 1)
	AT4G29810.1	AIPD(pS)YLSAIFR	X		ATMKK2 (ARABIDOPSIS THALIANA MAP KINASE KINASE 2)
	AT4G29810.1	IISQLEPEVL(pS)PIKPADDQLSLSDLD(oxM)VK		X	ATMKK2 (ARABIDOPSIS THALIANA MAP KINASE KINASE 2)
**Casein kinase**			**2**	**–**	
	AT2G23080.1	(pY)QLDLDPQLEALVGR	X		Casein kinase II α chain
	AT4G28860.1	NMNMPSSTSLSPAG(t)(s)KR	X		CKL4 (CASEIN KINASE I-LIKE 4)
**PI kinase**			**2**	**1**	
	AT4G33240.1	(pS)P(pT)(pS)LAKILGIYQV(pS)(pS)K	X		1-phosphatidylinositol-4-phosphate 5-kinase
	AT4G33240.1	CAAN(pS)IPSPSDETK	X	x	1-phosphatidylinositol-4-phosphate 5-kinase
**Other kinases**			**22**	**24**	
	AT1G01450.1	LFPS(pS)LLDN(pT)K	X		Protein kinase-related
	AT1G04210.1	SSALTLLSEISGLKCL(pT)R	X	x	Leucine-rich repeat protein kinase
	AT1G09600.1	IFKLCGSPSEE(pY)WK	X	x	Protein kinase family protein
	AT1G16760.1	GQ(pT)LALIHVLPK		x	Protein kinase family protein
	AT1G27070.1	HRLQQLQSEL(s)(s)VLHSLR	X	x	5′-AMP-activated protein kinase-related
	AT1G27070.1	HRLQQLQSELSSVLH(pS)LR		x	5′-AMP-activated protein kinase-related
	AT1G33770.1	(pT)VIVERPSR	X	x	Protein kinase family protein
	AT1G67470.1	KPK(pS)EIASER	X		Protein kinase family protein
	AT1G80870.1	AKI(pS)DFGLSR		x	Protein kinase family protein
	AT1G80870.1	E(oxM)NLLSPN(pS)VLDLGKGSK	X	x	Protein kinase family protein
	AT2G29000.1	IISLDL(pS)NR	X		Leucine-rich repeat protein kinase
	AT3G02810.1	LSSK(s)(s)QK	X	x	Protein kinase family protein
	AT3G13670.1	I(s)GGNDR(s)AGA(s)ILEVALK	X	x	Protein kinase family protein
	AT3G13670.1	VQVGG(pS)PLYK	X		Protein kinase family protein
	AT3G17420.1	SNATTLPVTQ(pS)PR		x	GPK1 (GLYOXYSOMAL PROTEIN KINASE 1)
	AT3G20830.1	LRTTP(pS)(pS)APPSPLR	X		Protein kinase family protein
	AT3G44200.1	RTSLIAHQ(pS)R		x	NEK6 NIMA (NEVER IN MITOSIS, GENE A)-RELATED 6
	AT3G59410.1	GALRADRP(pT)R	X	x	Protein kinase family protein
	AT3G59410.1	GQLKDHGSNADEDNELL(pS)EEI(pT)AL(pS)AIFQEDCK	X	x	Protein kinase family protein
	AT4G10730.1	(oxM)KEL(pT)EELEVEK	X	x	Protein kinase family protein
	AT4G16970.1	LLLSSGHPESVIDL(s)(s)K	X	x	Protein kinase family protein
	AT4G24740.1	I(pT)AREALR	X	x	AFC2 (ARABIDOPSIS FUS3-COMPLEMENTING GENE 2)
	AT4G33080.1	I(pS)VDDFELL(pT)IIGR		x	Protein kinase
	AT4G35600.1	VG(pS)GMIVAIK		x	CONNEXIN 32; Receptor-like cytoplasmic kinase
	AT5G01020.1	G(pY)IDDNLRVGLK		x	Protein kinase family protein
	AT5G01020.1	TTAPL(pS)WSR	X	x	Protein kinase family protein
	AT5G11400.1	GYIDET(pT)FAPSR	X	x	Protein kinase-related
	AT5G18910.1	GKQLTP(pS)(pT)R	X		Protein kinase family protein
	AT5G51770.1	G(pS)VLEVGNVVR	X	x	Protein kinase family protein
	AT5G57035.1	MTNKFFELIGGAP(s)Y(s)(s)V(s)VAVK		x	Protein kinase family protein
**Phosphatases**			**3**	**1**	
	AT2G42810.1	SHEVKDEG(pY)EVEHDGK	X		PP5.2 (PROTEIN PHOSPHATASE 5.2)
	AT5G02400.1	(pT)LFANLISNNNKPRLK		x	PLL2 (POL-LIKE 2)
	AT5G23720.1	SYDTGLM(pS)P(oxM)SDR	X		PHS1 (PROPYZAMIDE-HYPERSENSITIVE 1)
	AT5G53000.1	AIEEA(t)(t)(s)WYNDKPLR	X		TAP46 (2A PHOSPHATASE ASSOCIATED PROTEIN OF 46 KD)
**PP2Cs**			**–**	**2**	
	AT1G22280.1	TDQAILSN(pS)SDLGR		x	Protein phosphatase 2C
	AT1G34750.1	TDQAILSH(pS)SDLGR		x	Protein phosphatase 2C

The majority of the proteins in the largest plant kinase family, the family of receptor-like kinases (Shiu and Bleecker, 2001), remain functionally uncharacterized. In our study, RLK family members constituted a large proportion of the phosphorylated kinases, some of which were specifically phosphorylated under nitrate or ammonium resupply, while others were phosphorylated under both conditions (Table 2).

With regard to kinase substrates, a prominent sequence motif around the identified phosphorylation sites was the SP motif, which is indicative of a MAP kinase substrate (motifs are listed in Table S1). The phosphoproteins with the SP motif identified here were mainly transcription factors, but the identified phosphorylation site of nitrate reductase also included an SP motif and it has been suggested to be the substrate of MPK7 based on *in vitro* experiments (Feilner *et al.*, 2005; Popescu *et al.*, 2009).

### Phosphorylation and activity of metabolic enzymes

**Figure 6:**
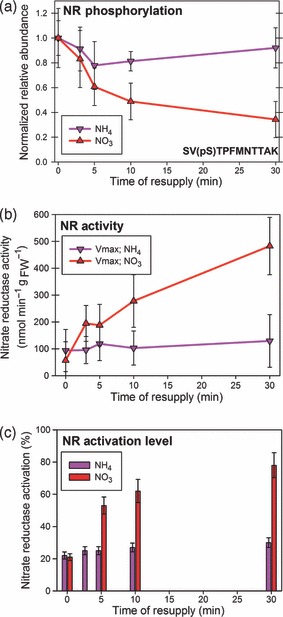
Nitrate reductase phosphorylation and activity in nitrate- and ammonium-resupplied seedlings. (a) Phosphorylation time course for S534. (b) Total nitrate reductase activity in the absence of magnesium. (c) Activation status of nitrate reductase in the presence of magnesium.

In order to link observed changes in phosphorylation patterns with changes in enzyme activity, the activities of nitrate reductase and glutamine synthase were analyzed. Nitrate reductase was found to be phosphorylated at the regulatory site S534 (Bachmann *et al.*, 1996), and phosphorylation decreased with the duration of nitrate resupply (Figure 6a). Phosphorylated nitrate reductase is known to be inactivated after 14-3-3 binding (Kaiser and Huber, 2001; Lambeck *et al.*, 2010). Some metabolic enzymes (sucrose phosphate synthase, and possibly glutamine synthase) are phosphorylated, and are targeted for degradation after binding of 14-3-3 proteins (MacKintosh, 1998), but under some conditions, 14-3-3 binding can protect proteins (e.g. nitrate reductase) from degradation (Cotelle *et al.*, 2000). We observed an increase in nitrate reductase activity (*V*_max_) over time in nitrate-supplied samples that corresponded with a decrease in nitrate reductase phosphorylation levels (Figure 6b). The activation state of nitrate reductase, calculated on the basis of the enzyme activity measured in the presence of magnesium compared with the maximal activity in the absence of magnesium in nitrate-resupplied seedlings, also increased with decreasing phosphorylation status (Figure 6c). In contrast, in ammonium-resupplied seedlings, nitrate reductase activity and activation status remained low, and higher phosphorylation was observed.

Glutamine synthase regulation is more complex. Plants have two glutamine synthase isoenzymes, plastidic GS2 and cytosolic GS1, which has multiple isoforms. Both isoenzymes can be phosphorylated (Finnemann and Schjoerring, 2000; Lima *et al.*, 2006). In *Brassica napus*, phosphorylated GS1 is active and protected against degradation upon binding of 14-3-3 proteins (Finnemann and Schjoerring, 2000). GS1 isoform 1 expression is more dominant under low-nitrate conditions, while expression of GS1 isoform 2 increases under high-nitrate conditions (Lothier *et al.*, 2011). GS1 isoform 1 has a high affinity for ammonium but GS1 isoform 2 has a low affinity for ammonium (Ishiyama *et al.*, 2004). GS1 isoform 2 has a proposed role in nitrogen remobilization and ammonium detoxification under high-nitrogen growth conditions (Ishiyama *et al.*, 2004). In our experiments, GS1 showed an increase in phosphorylation in nitrate-supplied seedlings, but the phosphorylation levels were almost unchanged in ammonium-supplied seedlings for isoforms 1 and 2. GS1 isoform 3 showed an increase in phosphorylation level in ammonium-supplied seedlings (Figure S4a–c). The total activity of glutamine synthase increased over time in both treatments, but reached higher levels in ammonium-supplied cells (Figure S4d).

## Discussion

The aim of this study was to identify candidate phosphorylation sites involved in transduction of signals related to changes in external nitrogen availability. We were especially interested in characterization of fast and slow responses in protein phosphorylation. Therefore, we used a defined common reference point in the starvation/resupply experimental design, namely the nitrogen starvation status of the untreated seedlings. The time points chosen in this study allowed identification of early responses upon changes in external conditions. The shortest time point of 3 min was the fastest possible time point that allowed reproducible harvesting of plant material. However, there are indications also studies on plant receptors that externally induced phosphorylation responses occur within seconds (Schulze *et al.*, 2010). Although a number of receptors and transporters were identified whose phosphorylation change peaked at 3 or 5 min of nitrogen resupply, some initial regulatory proteins may have been missed due to a transient response occurring in <3 min.

Under conditions of external changes in available nitrogen supply, plasma membrane proteins are expected to play a central role in recognition, uptake and transduction of this external nutrient signal. Only after nutrient uptake are effects on metabolism and transcriptional changes expected. In agreement with this hypothesis, an increasing proportion of cytosolic proteins were phosphorylated at the later time points of 10 and 30 min of nitrogen resupply. In contrast, the proportion of membrane proteins showing a transient change in phosphorylation was highest at 3 or 5 min of nitrogen resupply. We conclude that the proteins identified as showing changes in their phosphorylation status at early time points of nitrate or ammonium resupply are proteins that are either involved in regulation of nitrogen uptake, or candidates for recognition and transduction of nutritional signals. Fast phosphorylation changes for solute transporters have been reported for ammonium transporter inactivation (Lanquar *et al.*, 2009; Loque *et al.*, 2007), for regulation of pore gating by aquaporins (Tornroth-Horsefield *et al.*, 2006) and for potassium channels (Lee *et al.*, 2007). Phosphorylation has also been shown to be involved in regulation of nitrate uptake (Ho *et al.*, 2009), but regulation of the major nitrate uptake protein NRT2;1 has so far only been described in context with the interacting protein NRT3.1 (NAR2.1) (Orsel *et al.*, 2006; Yong *et al.*, 2010), and the role of the change in phosphorylation observed here remains to be functionally elucidated. We hypothesize that, similar to NRT1.1, the high-affinity transporter NRT2.1 may be inactivated by dephosphorylation under a high external nitrogen concentration (3 mm).

### Quantification error and reproducibility

The relative standard deviation between biological replicates in the label-free quantitative analysis of phosphorylation time courses was found to be approximately 20% calculated on the basis of normalized ion intensities of phosphopeptides identified in multiple replicate experiments (Figure S5). Variation was lowest for nitrogen-starved seedlings (time point 0) and greatest for the shorter durations of nitrate, ammonium or KCl addition. All relative standard deviations are shown in the full list of phosphopeptides (Table S1). A few individual peptides showed very high between-experiment variation in nutrient-induced dynamic behavior, indicating that phosphorylation of these proteins may be unrelated to the stimulus applied in the experiments. In general, the standard deviations observed here are within the expected range for label-free quantification (Schulze and Usadel, 2010).

### Quantitative coverage and amplitude of change

In the present study, over 1200 phosphorylation sites were identified with high confidence (Olsen *et al.*, 2006) in approximately 800 proteins. However, only approximately 50% of these identified phosphorylation sites could be used for further robust quantitative analysis. This is because the majority of phosphorylation sites were identified at only one time point under one experimental condition. Although retention time correlation (Andersen *et al.*, 2003; Foster *et al.*, 2006) of identified parent ions across samples was used, not all missing values could be resolved. In public databases, 83% of all identified phosphorylation sites in plants were identified only under one particular condition, and a minority (5%) were found under more than three conditions (Durek *et al.*, 2010). These observations can be explained by (i) phosphorylation is indeed specific to particular experimental conditions or tissues, (ii) due to the stochastic nature of data-dependent acquisition in proteomic experiments, the overlap between identified peptides even for technical replicates on LTQ-Orbitrap instruments is between 35 and 60% (Tabb *et al.*, 2010), and (iii) false-positive phosphorylation site identifications are more likely among ‘single’ identifications. The latter conclusion is supported by the positive correlation between high phosphorylation site prediction scores and the number of experiments identifying a particular phosphorylation site (Durek *et al.*, 2010). Furthermore, we conclude that we may have identified only a subset of the possible nitrogen-induced phosphorylation sites, as different enrichment methods result in identification of distinct subsets of the phosphoproteome (Bodenmiller *et al.*, 2007).

In the dataset presented here, we observed significant changes in the phosphorylation level of between 20 and 80%. However, due to the nature of the quantification applied here, we do not know the absolute stoichiometry of the observed phosphorylation changes. There are examples indicating that a change in phosphorylation level of approximately 20–50% can have a biological effect at overall low stoichiometry. In mammalian cells, Akt phosphorylation has a low stoichiometry, with only 1.5–5% of all molecules being phosphorylated at two different sites. Inhibition of a downstream signaling pathway leads to strong changes in the Akt phosphorylation level although the overall phosphorylation stoichiometry remains low. However, these changes at low absolute level were sufficient to trigger a cellular response (Atrih *et al.*, 2010). In contrast, responses may be induced by a low overall change in phosphorylation at a generally high phosphorylation stoichiometry. For example, the phosphorylation stoichiometry of some photosynthetic proteins in thylakoids is quite high in both dark- and light-adapted plants, and changes by only approximately 10% during a day/night cycle (Vener *et al.*, 2001).

Thus, despite the limitations in the unbiased phosphoproteomic ‘discovery’ experiments discussed above, the nitrate- and ammonium-induced dynamic phosphoproteome presented here provides important information for further in-depth study of nutrient-related signaling pathways. Known key phosphorylation sites of nitrogen assimilatory enzymes and important pathway components such as transporters and proton pumps have been reliably identified and quantified. In addition, novel candidate proteins are presented that may contribute to understanding nutrient-induced signaling. In addition, our dataset clearly goes beyond static identification of phosphorylation sites, instead providing a time-resolved phosphorylation pattern for almost 600 proteins. This information may be crucial during the task of pinpointing potential regulatory phosphorylation sites.

### Comparison with gene expression studies

Many studies have systematically investigated changes in gene expression during starvation/resupply experiments (Krouk *et al.*, 2010; Morcuende *et al.*, 2007; Osuna *et al.*, 2007; Scheible *et al.*, 1997a). Putative regulatory elements were identified and co-expression analysis led to definition of regulatory networks. Recently, very early nitrate-induced transcript changes have been defined that affect mainly the translation machinery (ribosomal proteins) or signaling components, and that are clearly distinct from later transcriptional changes affecting nitrate transport and metabolism (Krouk *et al.*, 2010).

Comparing our results on proteins with nitrogen-induced phosphorylation changes with results obtained by transcriptional analysis after short- and long-term nitrate resupply after starvation revealed a rather small overlap (Figure S6). Only five proteins showed overlap with proteins displaying very early transcriptional regulation upon nitrate resupply (3 and 6 min) (Krouk *et al.*, 2010). Among these were a protein kinase and two transcriptional regulators. Another three proteins, namely the nitrate transporter NRT2;1 (At1g08090.1), a lipase (At1g73920.1) and a receptor kinase (At2g23300.1), showed a response to nitrate resupply at the level of protein phosphorylation as well as medium-term (9–30 min) and long-term (3 h) transcriptional changes.

The generally low overlap is not surprising as the approaches focus on complementary levels of regulation (Carpentier *et al.*, 2008). The changes observed at the post-translational level may not be reflected in alterations in gene expression. Moreover, regulation at the post-translational and post-transcriptional levels does not require *de novo* protein synthesis, and thus is faster and allows adaptations to short-term environmental changes. Transcriptional regulation has been suggested to provide more long-term adaptive potential (Piques *et al.*, 2009). Our findings indicate that protein phosphorylation provides a layer of regulation parallel to early transcriptional changes, and, in some cases, specific proteins/genes may be affected at both regulatory levels. In general, our finding that early phosphorylation events particularly affect signaling proteins and transcription factors while later phosphorylation responses affect nitrogen transport and metabolism is in line with similar findings in high-resolution time-course analyses of nitrate-induced gene transcription (Krouk *et al.*, 2010). In the transcription study, genes induced at 3 and 5 min were also functionally distinct from the typical ‘nitrogen’ responses occurring at later time points.

In conclusion, our study provides important insights into the dynamics of individual phosphorylation sites under conditions of nitrogen starvation and resupply using two nitrogen sources. Our work contributes to understanding of the complex regulatory patterns with respect to very short-term changes in external nutritional conditions of the plant. We showed that there are specific and common responses for the two nitrogen sources (nitrate and ammonium). The differences in phosphorylation dynamics were most prominent with regard to signal transduction, and at later time points merged to common regulation sites of enzyme activities. Despite the limitations of unbiased phosphoproteomic analysis discussed above, our results indicate new candidate protein phosphorylation sites that are possibly involved in nitrogen sensing and regulation of transport. These candidate phosphorylation sites were defined based on their dynamic phosphorylation patterns rather than merely identification of phosphopeptides. In-depth future study of these candidates may close the gap in knowledge between sensing at the protein level and the gene expression response at the transcriptional level.

## Experimental Procedures

### Seedling liquid culture

To perform the starvation/resupply experiments, seedlings were grown in axenic liquid culture as described previously (Scheible *et al.*, 2004). Surface-sterilized seeds were incubated for 2 days at 4°C in the dark in standard growth medium containing 2 mm KNO_3_, 1 mm NH_4_NO_3_ and 1 mm glutamine (Scheible *et al.*, 2004), and then under continuous light (20°C, 80 μE sec^−1^ m^−2^) for 14 days. Then seedlings were starved for 2 days by changing the growth medium to starvation medium containing very low amounts of nitrogen (0.15 mm KNO_3_ and 5 μm NH_4_NO_3_) (Scheible *et al.*, 2004). The medium contained 0.5% sucrose under all conditions.

Resupply of nitrogen was performed by addition of KNO_3_ or NH_4_Cl for 3, 5, 10 or 30 min to a final concentration of 3 mm. Resupply of KCl served as an ionic control. Seedlings were harvested by suction over a filter plate, drained from the remaining liquid on paper towels, and frozen immediately in liquid nitrogen.

In total, four biological replicate time-course experiments were performed across the whole time series for nitrate and ammonium resupply, and two sets of experiments were performed for KCl resupply.

### Analysis of marker gene expression

RNA was extracted from 200 mg of powdered seedlings using a plant RNA mini kit (Invitek, http://www.invitek.de/) according to the manufacturer’s instructions. Removal of residual DNA, cDNA synthesis and quantitative RT-PCR using an ABI PRISM 7900 HT sequence detection system (http://www.appliedbiosystems.com/) and SYBR Green (Applied Biosystems, http://www.appliedbiosystems.com) were performed as described previously (Le *et al.*, 2008).

The following genes were used as marker genes for nitrogen responses in seedlings (Scheible *et al.*, 2004): *At3g03910*, which encodes a putative glutamate dehydrogenase, was used as a marker for nitrogen starvation, and *At4g32950*, which encodes a putative protein phosphatase 2C, was used as a marker for nitrogen resupply. Both genes are expected to be up-regulated during nitrogen starvation. Ubiquitin (UBQ) was used as a control gene. Data analysis was performed using StepOne 2.0 software (ABI-Sciex). In order to compare data between experiments, *C*_t_ values were normalized to the value for ubiquitin [*C*_t(gene of interest)_−*C*_t(UBQ)_].

### Nitrate reductase activity

Nitrate reductase activity was analyzed in a microtiter plate assay as described previously (Gibon *et al.*, 2004). Frozen plant material was powdered using a Retsch mill (http://www.retsch.com/), and protein was extracted in 250 mm HEPES/KOH buffer, pH 7.5, 0.25% Triton X-100, 100 mm KNO_3_. The extraction buffer was supplemented with phosphatase inhibitors (2 mm NaVO_3_, 50 mm NaF, 50 μm cantharidin, 2 mm leupeptin). In order to measure *V*_max_, the extraction buffer additionally contained 10 mm EDTA. To determine the nitrate reductase activation state (*V*_act_), the assay was performed in the presence of 10 mm Mg^2+^ acetate to assess the activity of the non-phosphorylated form of nitrate reductase (Botrel and Kaiser, 1997).

The assay mix contained protein extract together with 2 mm FAD, 10 mm NaMoO_4_, 500 mm dithiothreitol and 625 μm NADH. The reaction was stopped after 20 and 40 min by adding Zn acetate (200 mm) and phenylmethanesulfonylfluoride (0.2 mm). Nitrite production was detected at 540 nm by a colorimetric assay using *N*(1-naphtyl)ethylendiamine dihydrochloride.

The activation state of the enzyme was then calculated as *V*_act_/*V*_max_, expressed as a percentage (Botrel and Kaiser, 1997).

### Plasma membrane preparation

All protein extractions were performed in the presence of phosphatase inhibitors (50 mm NaF, 1 mm Na_3_VO_4_, 4 μm leupeptin, 1 mm benzamidine, 0.03 μm microcystin). Plasma membranes were purified from the microsomal pellet (100 000 ***g***) using a two-phase system of dextran and polyethylene glycol as described previously (Kierszniowska *et al.*, 2009) Plasma membrane vesicles were inverted using Brij-58 (Sigma Aldrich, http://www.sigmaaldrich.com), and intracellular protein parts were digested using trypsin (Nühse *et al.*, 2003).

### Fractionation of soluble proteins

The supernatant of the first ultra-centrifugation step during plasma membrane preparation was precipitated using four volumes of ice-cold acetone. Pellets were resolved in 6 m urea and 2 m thiourea, and the protein concentration was measured using the Bradford assay. Then 400 μg of protein was digested with trypsin and acidified with acetic acid as described previously (Niittylä*et al.*, 2007).

Modular fractionation tips using strong cation exchange (SCX) and C18 (a polymer of 18 carbon atoms) (Varian, http://www.varian.com) were used for cation-exchange fractionation of soluble proteins (Ishihama *et al.*, 2006). Two cation exchange fractions were collected after elution with 70 mm ammonium formate and 30% acetonitrile and after elution with 1 m ammonium formate and 30% acetonitrile.

### Phosphopeptide enrichment

Phosphopeptides were enriched over TiO_2_ (Larsen *et al.*, 2005). Titanium enrichment was performed in batch mode using 10 mg TiO_2_ powder with 200 μg of protein. Beads were washed using 70% acetonitrile, 20 mg ml^−1^ 2,5-dihydroxybenzoic acid and 0.1% trifluoroacetic acid (TFA). Tryptic digests were diluted in 80% acetonitrile, 2 mg ml^−1^ DHB and 0.1% TFA, and loaded onto TiO_2_ beads. After shaking, the supernatant was removed and the TiO_2_ was washed with 10% acetonitrile and 0.1% TFA and then with 80% acetonitrile and 0.1% TFA. Phosphopeptides were eluted using 70% acetonitrile, 0.5% NH_4_OH and 0.1% TFA. The eluate was acidified to pH 2 and concentrated.

### LC-MS/MS

Tryptic peptide mixtures were analyzed by LC-MS/MS using a nanoflow Easy-nLC HPLC system (Thermo Scientific, http://www.thermo.com) and an LTQ-Orbitrap hybrid mass spectrometer (Thermo Scientific) as the mass analyzer. For liquid chromatography, 0.5% acetic acid was used as aqueous phase (solution A), and 0.5% acetic acid, 80% acetonitrile (solution B) were used. Peptides were eluted from a 75 mm analytical column using a two-step gradient (71 min 5% solution B to 30% solution B, 14 min to 60% solution B, 10 min to 90% solution B), and sprayed directly into the LTQ-Orbitrap mass spectrometer. Peptides were identified by information-dependent acquisition of fragmentation spectra of multiple-charged peptides. Up to five information-dependent MS/MS spectra were acquired in the linear ion trap for each full scan spectrum acquired at 60 000 full width at half maximum (FWHM) resolution in the mass spectrometer. The overall cycle time was approximately 1 sec. The complete range for full scans was split into two segments: 300–1500 and 950–1850 *m*/*z*. For fragmentation, CID was chosen, multi-stage activation (Schroeder *et al.*, 2004) was enabled, and the collision energy was 35 eV.

### Raw data processing

Fragment MS/MS spectra from raw files were extracted as DTA files and then merged to peak lists using default settings of DTASuperCharge version 1.18 (http://msquant.alwaysdata.net) with a tolerance for precursor ion detection of 50 ppm. The DTA files for one experiment were combined into a single file that was used for the database search. Fragmentation spectra were searched against a non-redundant Arabidopsis protein database (tair8, version 2008-04; 31 921 entries; http://www.arabidopsis.org) using the Mascot algorithm (version 2.2.0; Matrix Science, http://www.matrixscience.com). The database contained the full Arabidopsis proteome and commonly observed contaminants (human keratin, trypsin and lysyl endopeptidase). The following search parameters were used: trypsin as cleaving enzyme, peptide mass tolerance 10 ppm, MS/MS tolerance 0.8 Da, one missed cleavage allowed. Methionine oxidation and threonine, serine and tyrosine phosphorylation were chosen as variable modifications. For data derived from soluble protein fractions, carbamidomethylation of cysteine was set as a fixed modification in addition to the above-described variable modifications. Only peptides with a length of more than five amino acids were considered.

Using the above criteria for protein identification, the rate of false peptide sequence assignment as determined by the ‘decoy database’ function implemented in Mascot version 2.2.0 was 2.5% at the 95% confidence level. Peptide assignment to proteins used the Mascot default settings, assigning each redundant peptide to the highest-scoring protein. For proteins identified by non-proteotypic peptides only, all possible isoforms are listed. Isoforms of protein only appear in tables as a separate protein entry if they were assigned at least one unique peptide.

### Phosphopeptide identification

For phosphopeptide scoring and determination of modification sites, post-translational modification (PTM) scores (Olsen *et al.*, 2006) were calculated using MSQuant version 1.4.3 (http://msquant.alwaysdata.net). All phosphopeptides were checked manually by comparison of experimentally identified masses with the theoretically expected masses for the fragments. Phosphopeptides were accepted if the PTM score was >40 and/or site-determining *b* or *y* ions were present. Annotated spectra of all phosphopeptides are shown in Figure S7. All phosphopeptides, including their fragment spectra, have been submitted to the phosphorylation site database PHOSPHAT (http://phosphat.mpimp-golm.mpg.de) and are publicly available.

### Label-free peptide quantification

Label-free relative quantification of phosphorylation was performed as described previously (Steen *et al.*, 2005) and as applied previously (Niittylä*et al.*, 2007). Retention time alignment of fragmented ion mass to charge ratios was also used to obtain quantitative information for these ions from samples in which no fragment spectra were acquired (Andersen *et al.*, 2005; Foster *et al.*, 2006). Ion intensity extraction from raw data files as well as retention time alignment were performed using MSQuant version 1.4.3. For ion intensities of each non-phosphopeptide, the mean ion intensity across the five time points was calculated and used for normalization. Subsequently, for each phosphopeptide sequence, the mean of normalized intensities was calculated from biological replicates based on the individual phosphopeptide *m*/*z* species, and this value was used to calculate ratios between treated (time points 3, 5, 10 and 30 min) and untreated (time point 0) samples. For each protein, only proteotypic non-phosphopeptides were used for normalization. If no unphosphorylated peptides were identified for a given protein, the mean of all identified non-phosphopeptides was used. Normalized ion intensities were standardized to values between 0 and 1 across the time series by dividing the ion intensities of each peptide species (each *m*/*z*) by the maximum value over the time series of each peptide. This allowed inclusion of peptides in the quantitative analysis for which no ion intensities were determined at reference time point 0.

Peptides conserved in multiple members of a protein family were identified using the ‘show subsets’ option in Mascot, and the non-peptides present in multiple proteins were excluded from quantitative analysis. Phosphopeptides matching multiple proteins are indicated in Table S1.

### Bioinformatic analysis

Information about gene function, structure and annotation was retrieved from the following resources: tair (Swarbreck *et al.*, 2008), aramemnon (Schwacke *et al.*, 2003) and PhosPhAt (Durek *et al.*, 2010). For functional annotation. the MapMan functional classification scheme was used (Thimm *et al.*, 2004).

K-means clustering was performed using Multi-Experiment Viewer 4.0 (http://www.tm4.org/mev/). Standardized time-course profiles of phosphopeptides were subjected to the analysis if quantitative information for at least four time points was obtained. Euclidean was used as a distance matrix, and 16 clusters were formed. Over-representation analysis was performed using a Fisher exact test, with the total number of identified proteins as a reference set. *P*-values were corrected for multiple testing (Benjamini and Hochberg, 1995).

## Supporting information

**Figure S1.** Venn diagram of the distribution phosphopeptides to the different treatments. (a) All unique phosphopeptides sequences identified in the different experiments. (b) Unique phosphopeptides that could be quantified at least at three different time points.

**Figure S2.** NRT2.1 phosphorylation and nitrateuptake (a) Targeted analysis of NRT2.1 phosphorylation underdifferent concentrations of nitrate resupply for 10 min.Normalized ion intensities of the phosphorylated form (red) andnon-phosphorylated form (gray) are plotted as average of threebiological replicates (b) ^15^N-Nitrate uptake ofseedlings resupplied with different concentrations of nitrate underconditions of high- and low-affinity nitrate uptake.

**Figure S3.** Venn diagram of the identified phosphopeptides under nitrate and ammonium resupply and the respective non-phosphorylated peptides identified under the same conditions in the same respective samples.

**Figure S4.** Phosphorylation time course profiles for phosphopeptides identified from glutamine synthase 1 isoforms and total GS enzymes activitiy. (a) Phosphorylation time course for GS1.1 (b) phosphorylation time course for GS1.2, (c) phosphorylation time course for GS1.3 (d) Total activity of glutamine synthase in nitrate and ammonium resupplied seedlings.

**Figure S5.** Boxplot of the relative standard deviation of all quantified peptides averaged over all 12 replicated experiments.

**Figure S6.** Overlap of proteins found with change in phosphorylation and with significant changes in transcript levels upon nitrate resupply after nitrogen starvation.

**Figure S7.** All phosphopeptide spectra as exported from the respective entry in the public database PhosPhAt (http://phosphat.mpimp-golm.mpg.de).

**Table S1.** List of all phosphopeptides. The first worksheet contains the mass spectrometric evidence for each phosphopeptide by listing mass to charge ratio, MASCOT identification score, PTM score. The second worksheet contains the normalized ion intensities used for quantitation as well as additional information about sequence motifs, or putative MAP-kinase substrates.

**Appendix S1.** Glutamine synthase activity; targeted analysis of protein phosphorylation by single reaction monitoring; nitrate uptake assays.

As a service to our authors and readers, this journal provides supporting information supplied by the authors. Such materials are peer-reviewed and may be re-organized for online delivery, but are not copy-edited or typeset. Technical support issues arising from supporting information (other than missing files) should be addressed to the authors.
