# Supplementary material for: Nitrate and ammonium lead to distinct global dynamic phosphorylation patterns when resupplied to nitrogen-starved Arabidopsis seedlings
Source: Plant J. 2012 Jan 20;69(6):978–95. doi: 10.1111/j.1365-313X.2011.04848.x (PMC3380553; doi:10.1111/j.1365-313X.2011.04848.x)
Supplement: Supplementary file 1 [file tpj0069-0978-SD1.rtf]

Supplementary materials and methods

Glutamine synthase acitivity
Glutamine synthase activity was determined in a microtiter plate assay as described (Gibon et al., 2004). Frozen plant material was powderized using a Retch mill and protein was extracted in 500 mM Hepes/KOH buffer pH7.5, 0.25% Triton X100, 100 mM MgCl2, 20 mM NH4Cl, 10 mM EDTA, 0.4 mM 5',5'-Diadenosinpentaphosphate. Extraction buffer was supplemented with phosphatases inhibitors (2mM NaVO3, 50 mM NaF 50µM Cantharidine, 2mM Leupeptin). Briefly, Glutamate and NADH production from glutamine synthase was analyzed by the rate of NADH to NAD conversion (absorbance at 340 nm) by pyruvate kinase added to the assay mix. 

Targeted analysis of protein phosphorylation by single reaction monitoring
Protein pellets were resuspended in 6M urea, 2M thiourea, pH 8. Protein disulfide bridges were reduced by adding DTT and subsequently, free cysteine residues were alkylated using iodacetamide. Samples (50µg of protein) were then digested using sequencing grade trypsin (Promega) and desalted over C18 tips (Rappsilber et al., 2003).
Tryptic peptide mixtures were analyzed by selected reaction monitoring using nanoflow HPLC (Easy nLC, Thermo Scientific) and a triple quadrupole mass spectrometer (TSQ Quantum Discovery Max, Thermo Scientific) as mass analyzer. Peptides were eluted from a 75 µm analytical column (Easy Columns, Thermo Scientific) on a linear gradient running from 10% to 30% acetonitrile in 50 minutes and were ionized by electrospray directly into the mass spectrometer.
Specifically, phosphorylated peptides from proteins of interest were selected as targets of analysis. The following target peptides were used: SV(pS)TPFMNTTAK (nitrate reductase), (pS)LVSDLINLNLSDSTDK (pS)LLADLVNLDISDNSEK, (pS)LLSDLVNLNLTDATGK (isoforms of glutamine synthase), as well as EQSFAFSVQ(pS)PIVHTDK (nitrate transporter NRT2.1). 
Selected reaction monitoring (SRM) was used to quantify the abundance of phosphorylated peptides within each sample. An isotopically labeled synthetic standard peptide of the same sequence was used as an internal standard and was used for normalization of ion intensities across replica samples. The quadrupole Q1 was set as a mass filter for the respective parent ion, while Q3 was set to monitor specific fragment ions. Suitable fragments were experimentally determined using an extra set of samples. For each peptide, at least three fragment ions were used. Mass width for Q1 and Q3 was 0.7 Da, scan time 5 milliseconds. 
Data analysis involving merging of fragment ion information to a parent ion average and calculation of peak area ration was also done using the Software Pinpoint v.1.0 (Thermo Scientific). Briefly, for quantitative analysis of peptide abundance, ion intensities of three to five most intense fragment ions were summed and averaged across three biological replicates. Normalization was done against the spiked-in synthetic standard peptides. In some cases, these normalized ion intensities were expressed as ratios to reference to time point 0 and displayed as log2-values. 

Nitrate uptake assays
Nitrate uptake after resupply of different concentrations of nitrate was measured using 15NO3 as described (Delhon et al., 1995). Seedlings in liquid culture were starved for nitrogen for two days, then seedlings were washed in 1mM CaSO4 for one minute and were resupplied with full nutrient solution containing different concentrations of 15NO3 for 10 minutes. The assay was ended with another wash in 1 mM CaSO4. Plant material was powderized in a Retchmill and 15N content in 2 mg dry weight was analyzed after combustion using a GC-coupled isotope ratio mass spectrometer (Delta V, Thermo Scientific).
