# Supplementary figures and images for: Nitrate and ammonium lead to distinct global dynamic phosphorylation patterns when resupplied to nitrogen-starved Arabidopsis seedlings
Source: Plant J. 2012 Jan 20;69(6):978–95. doi: 10.1111/j.1365-313X.2011.04848.x (PMC3380553; doi:10.1111/j.1365-313X.2011.04848.x)

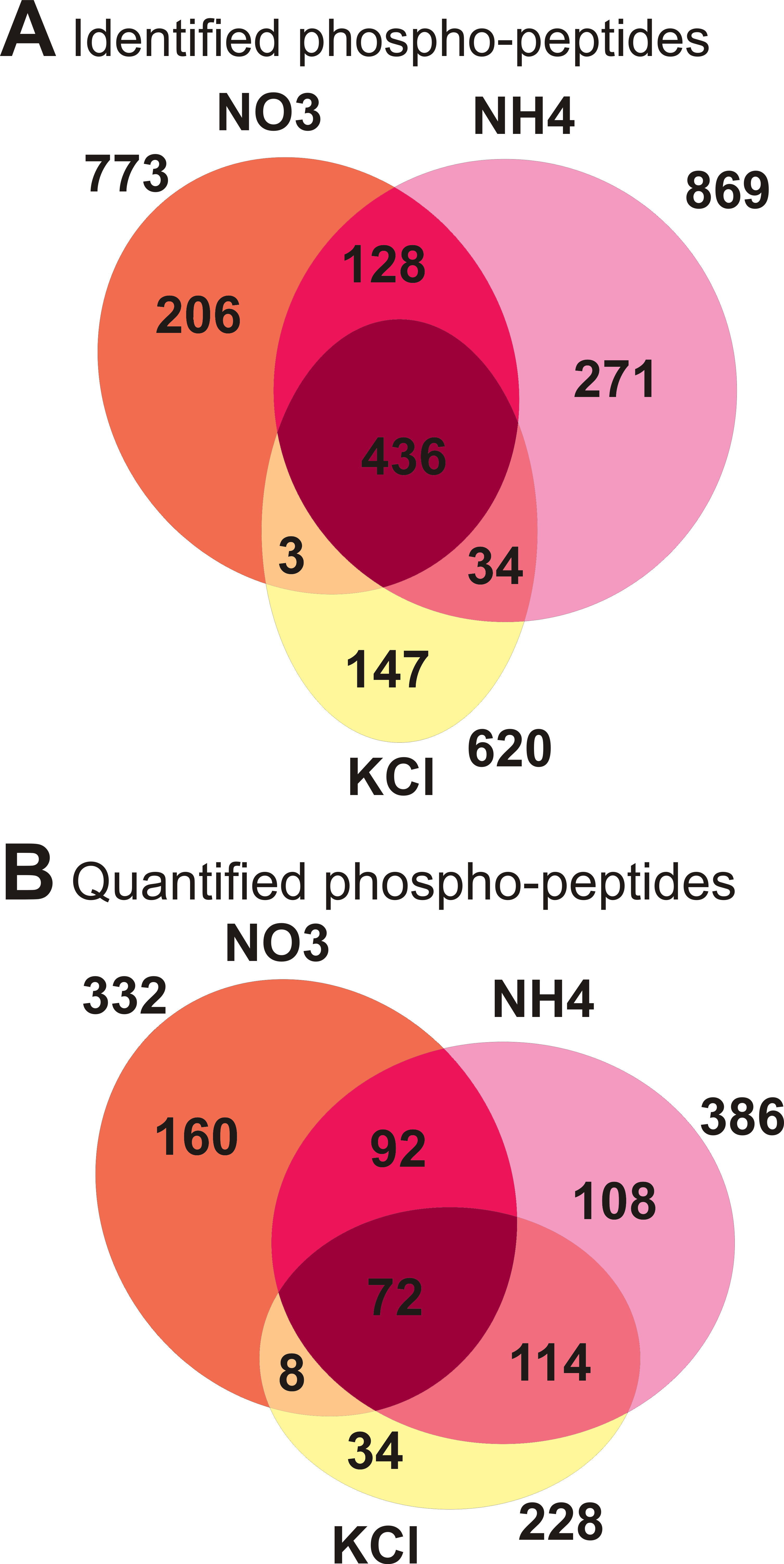

Supplement: Supplementary file 2 [file tpj0069-0978-SD2.jpg]

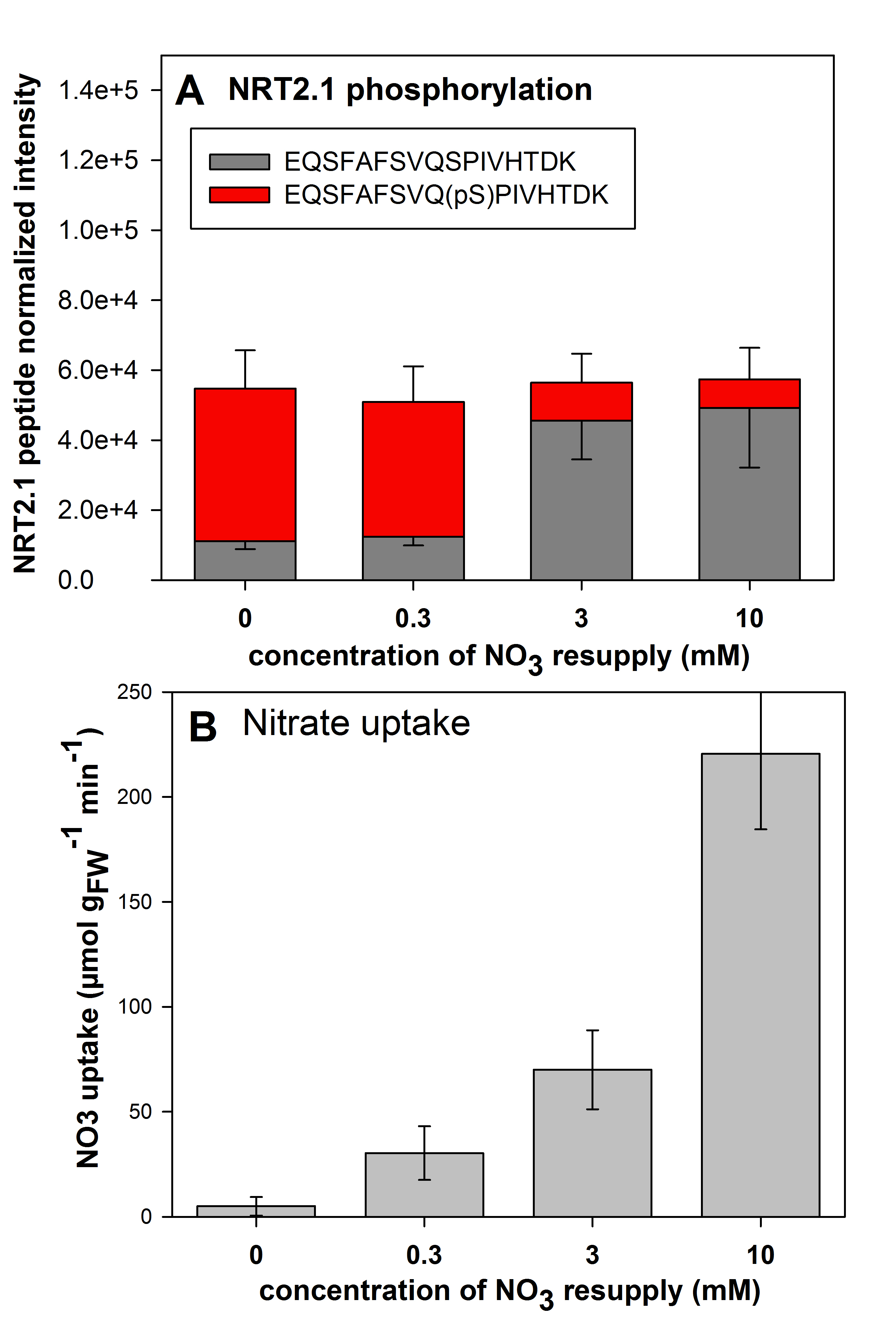

Supplement: Supplementary file 3 [file tpj0069-0978-SD3.jpg]

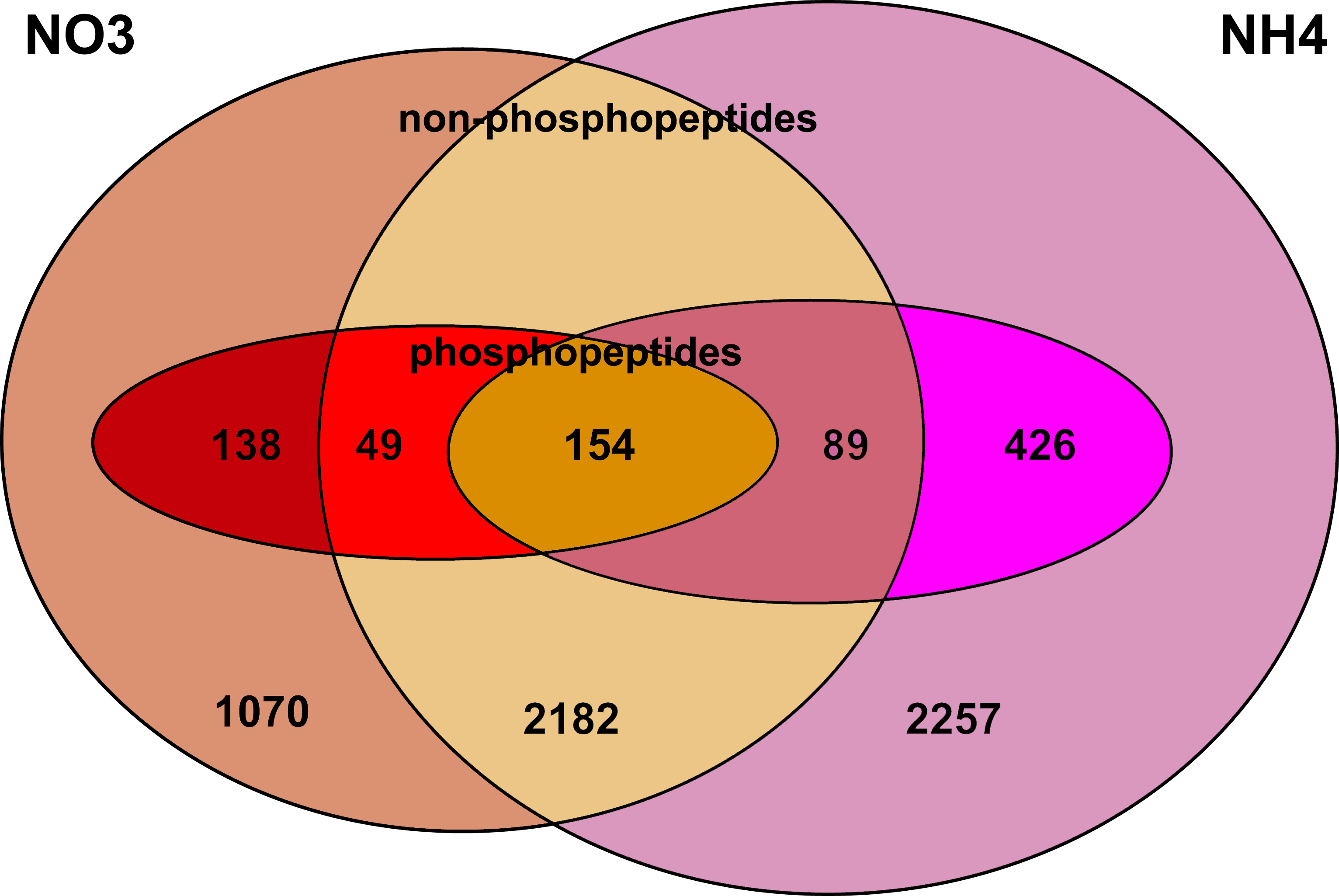

Supplement: Supplementary file 4 [file tpj0069-0978-SD4.jpg]

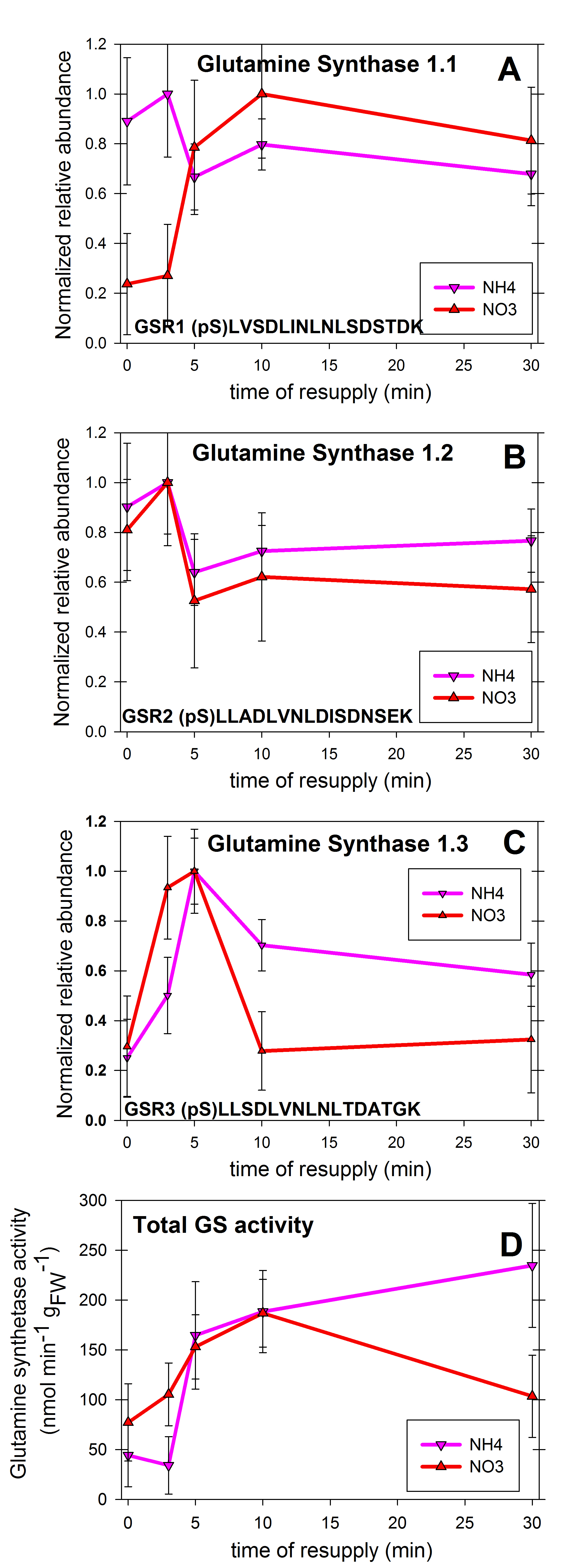

Supplement: Supplementary file 5 [file tpj0069-0978-SD5.jpg]

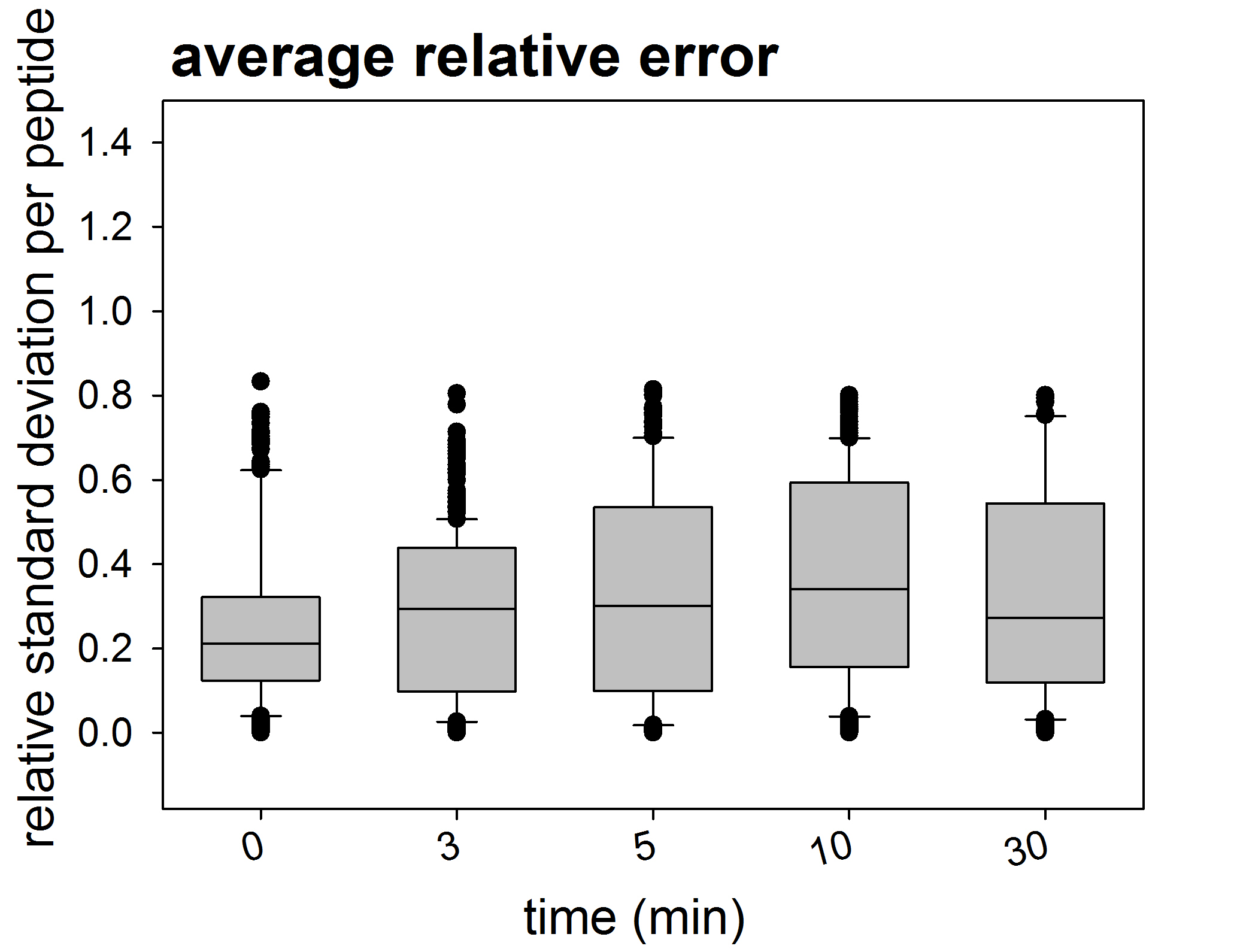

Supplement: Supplementary file 6 [file tpj0069-0978-SD6.jpg]

# Overlap with gene expression data

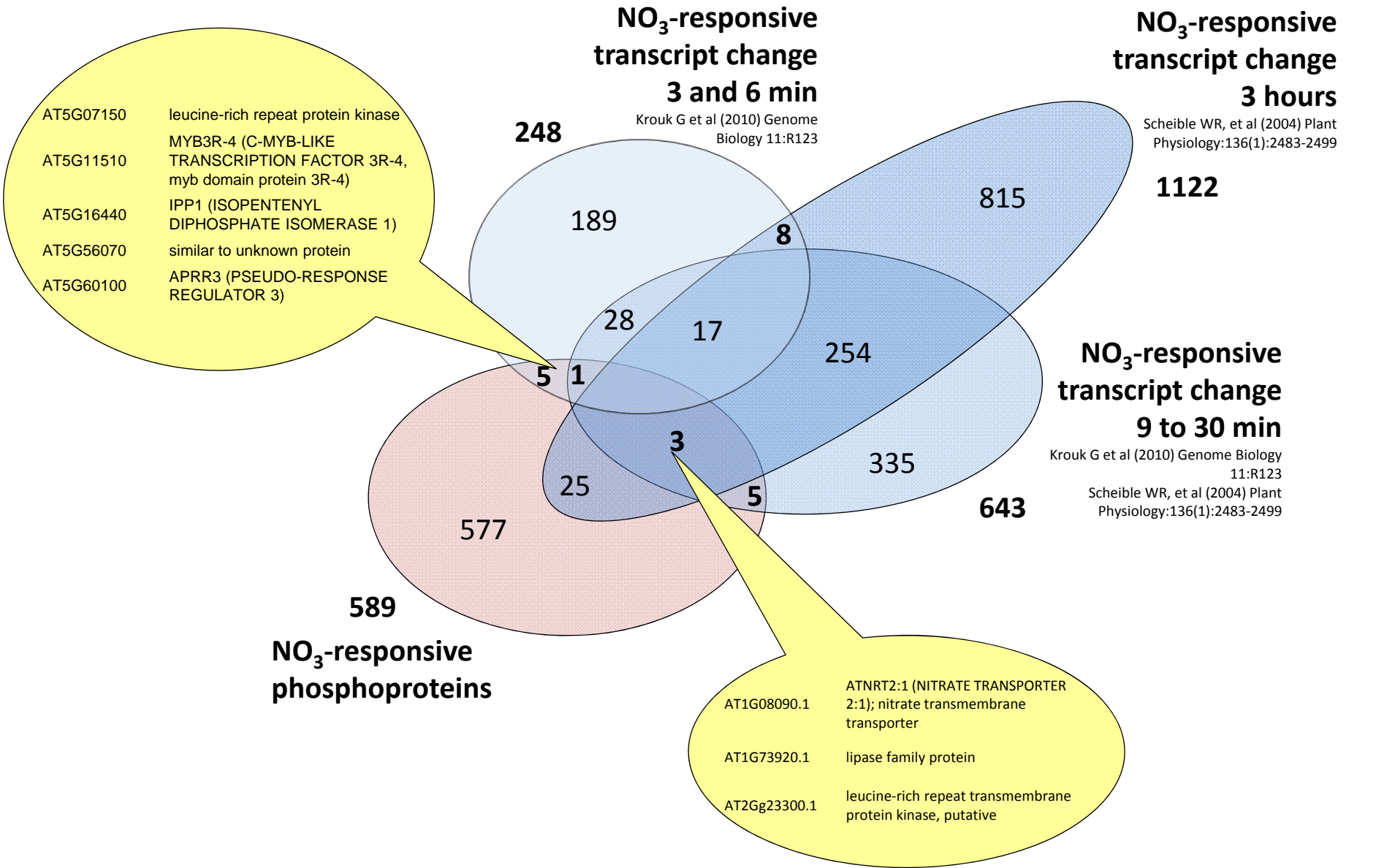

Supplement: Supplementary file 7 [file tpj0069-0978-SD7.pdf]
