# Supplementary material for: Nitrate and ammonium lead to distinct global dynamic phosphorylation patterns when resupplied to nitrogen-starved Arabidopsis seedlings
Source: Plant J. 2012 Jan 20;69(6):978–95. doi: 10.1111/j.1365-313X.2011.04848.x (PMC3380553; doi:10.1111/j.1365-313X.2011.04848.x)

AT1G17990.1 - ETKQSIPLL(oxM)P(pY)K - 822.403213 - Charge:2

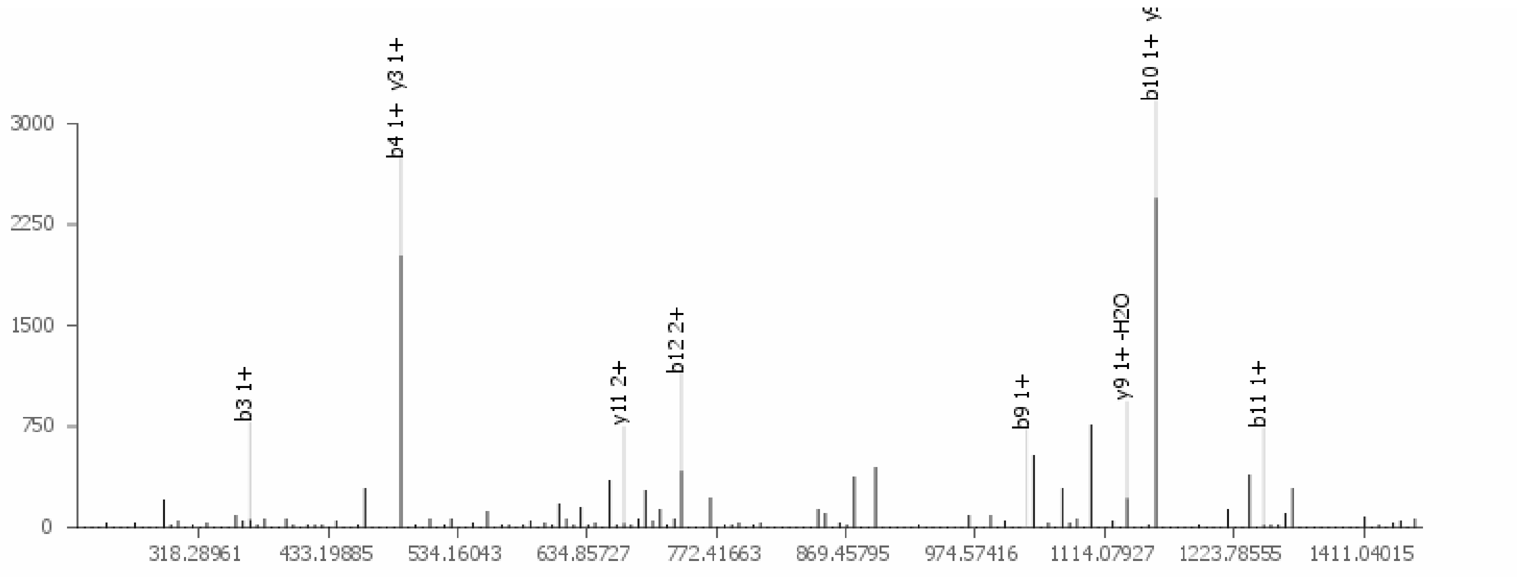

AT4G28080.1 - AFPIE(pT)AVSNARPGK(pS)K - 644.972591 - Charge:3

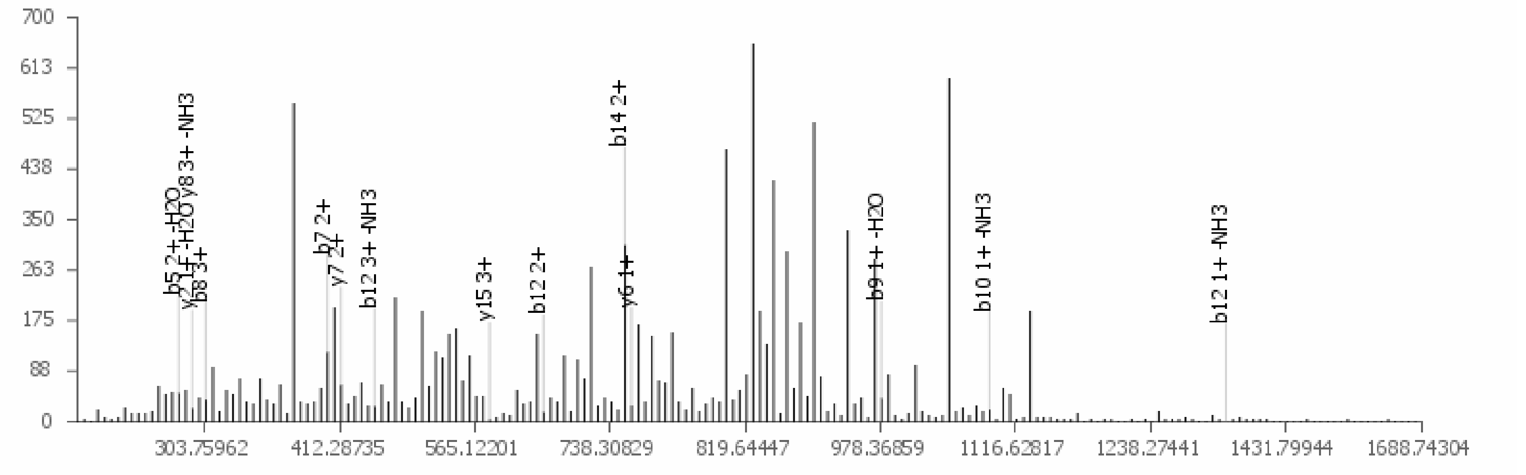

AT4G01660.1 - (pS)F(pT)RLVNGVSLVAK - 825.906846 - Charge:2

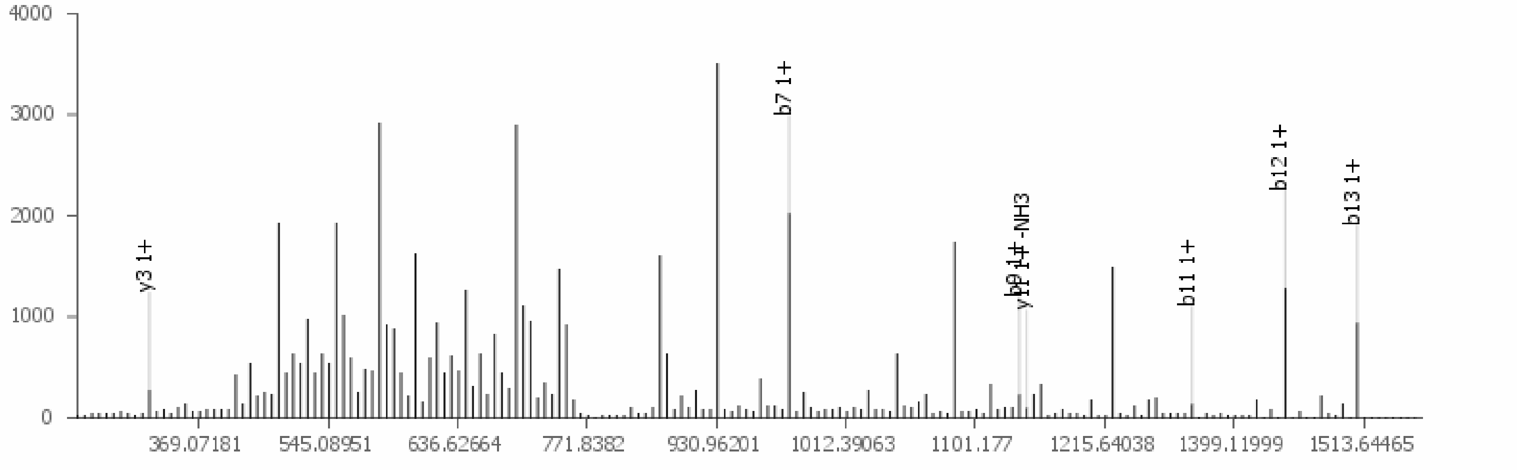

AT2G22950.1 - AHTKGA(pS)EIVLAACDK - 875.918197 - Charge:2

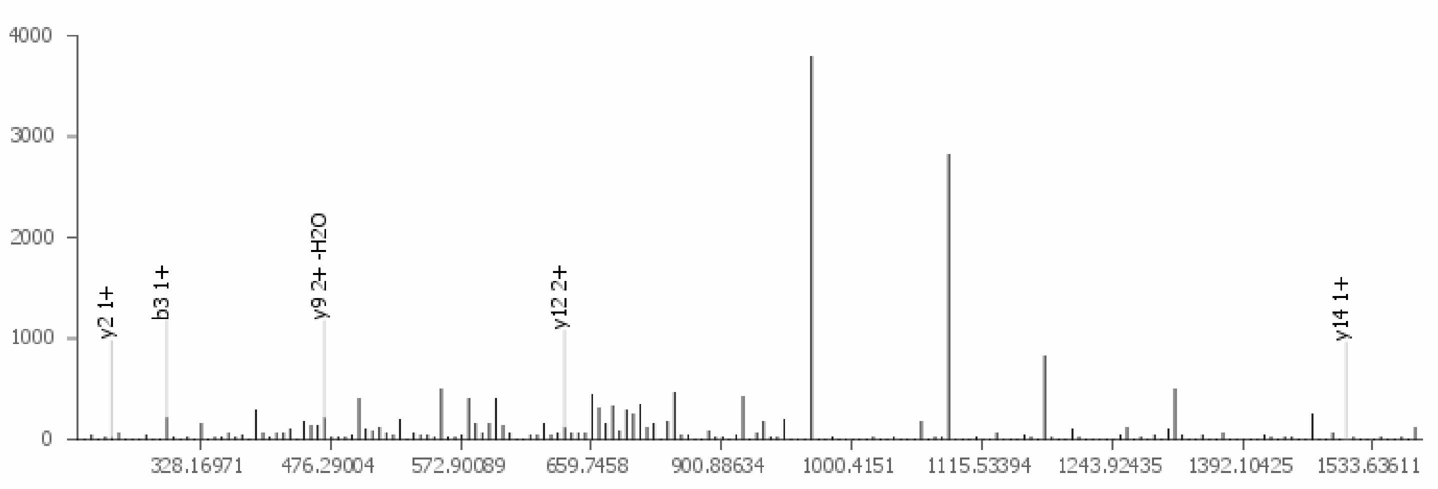

AT2G26540.1 - RSSVLYPA(pS)LK - 650.830804 - Charge:2

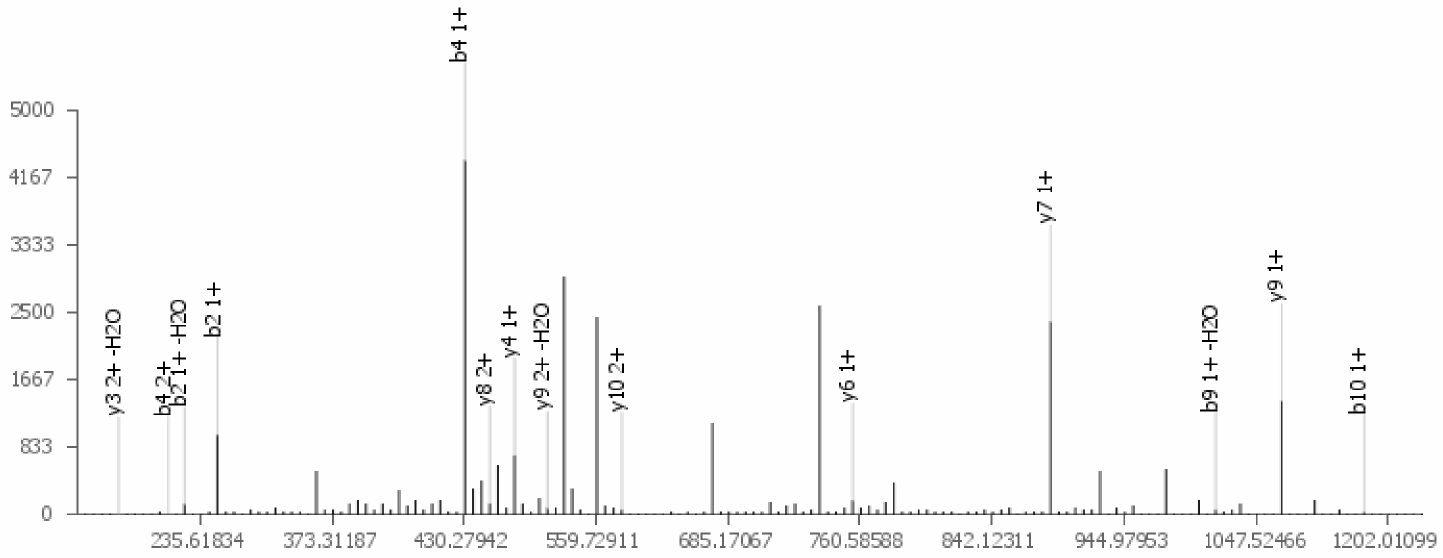

AT5G47690.1 - ILTNLLDPNTSI(pT)QA(pS)R - 1008.983361 - Charge:2

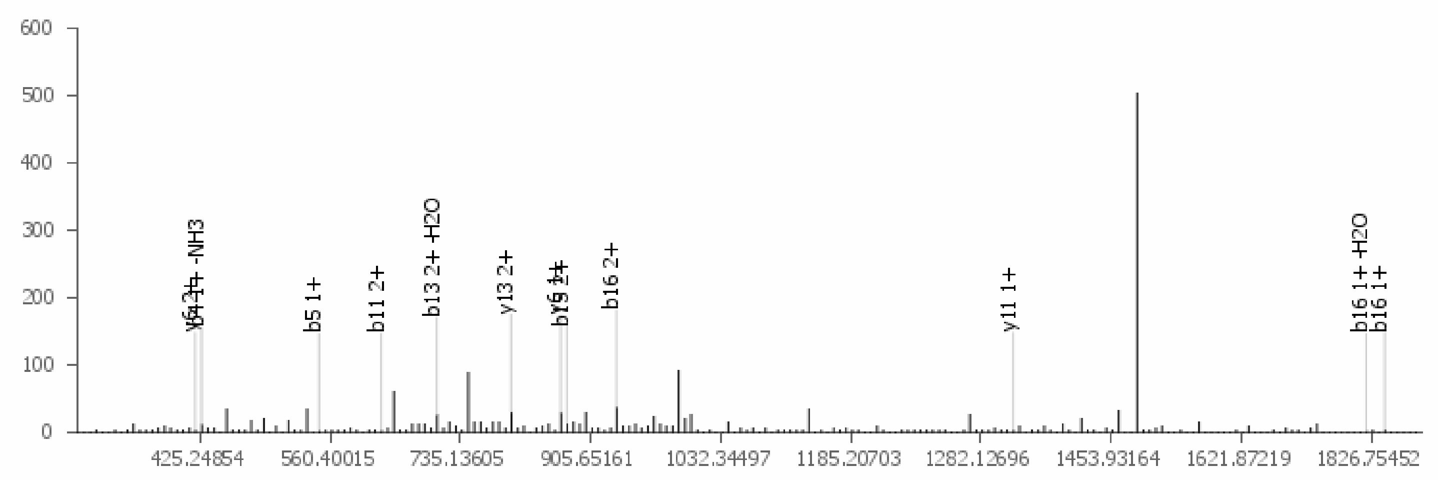

AT1G69430.1 - (pS)SGLPLLPFVRNSCQK - 941.966547 - Charge:2

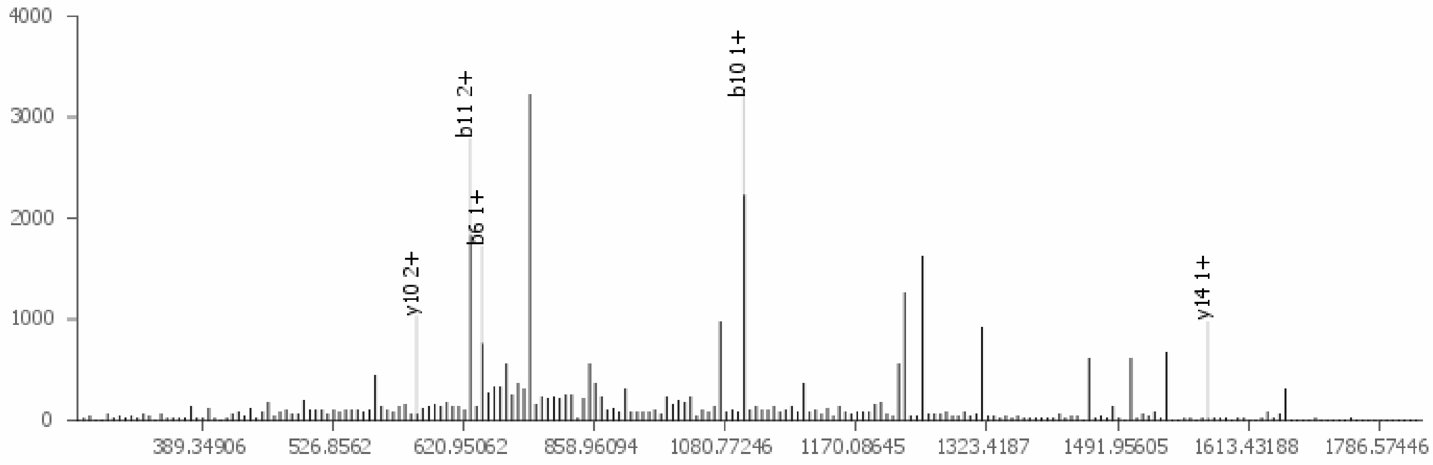

AT5G57220.1 - ICSLEILS(s)NRL(t)GFLSVR - 1123.081921 - Charge:2

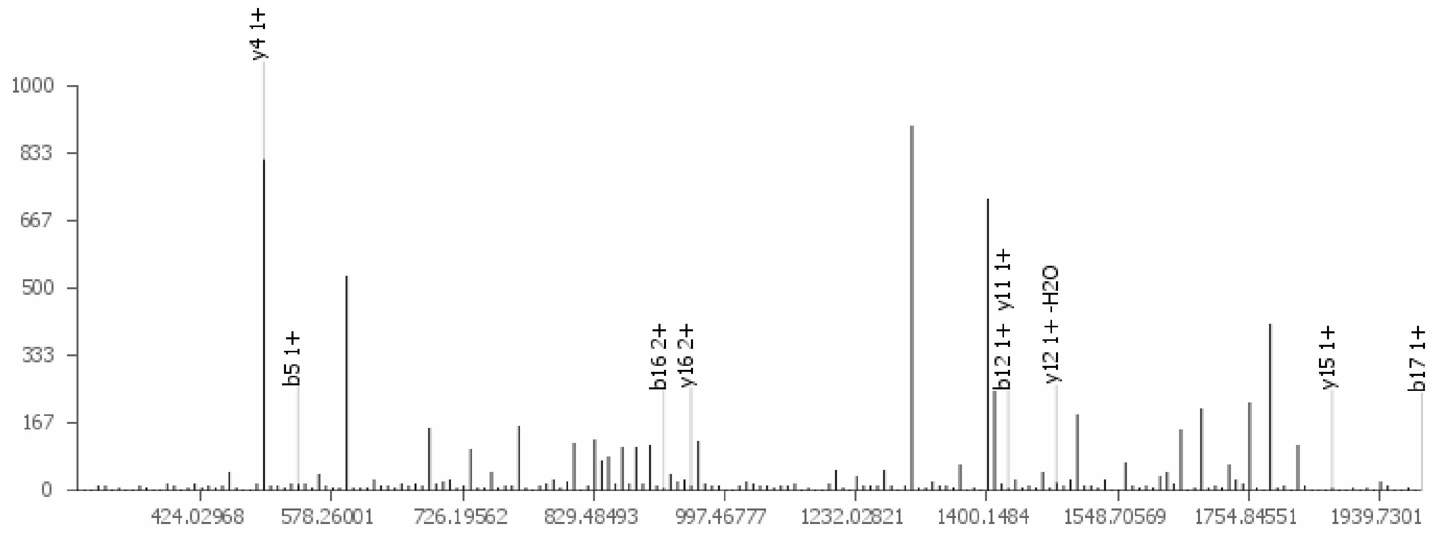

AT5G07940.1 - ETQ(pS)GHVGSRPSTSRK - 897.417433 - Charge:2

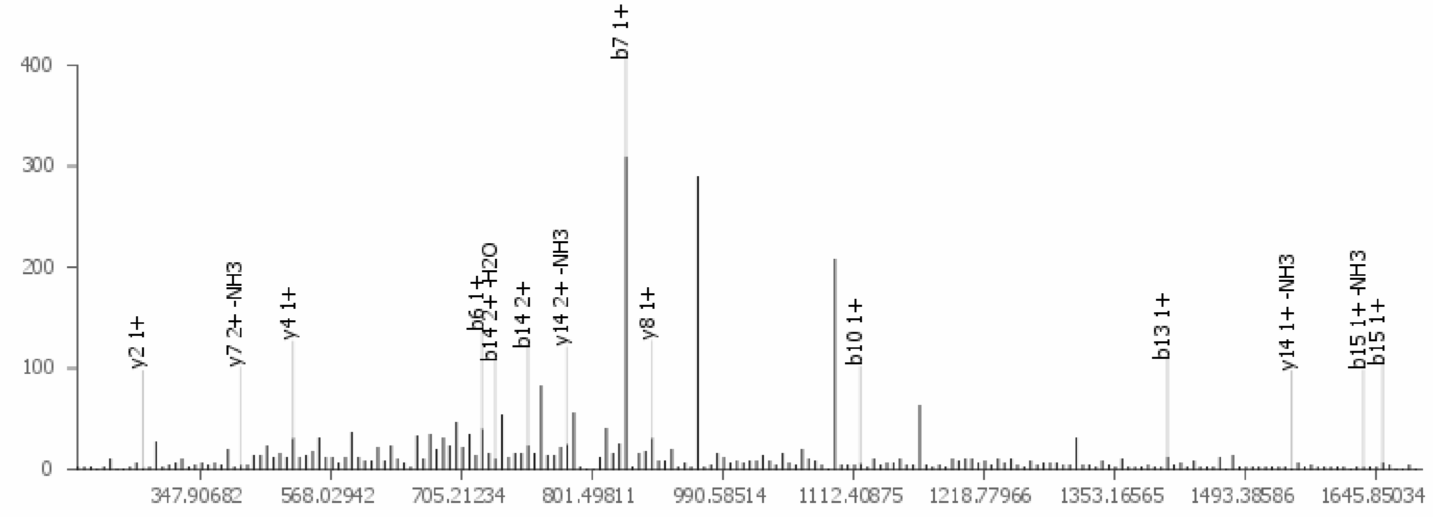

AT3G13930.1 - KLAEDNNVPL(s)(s)IK - 804.403272 - Charge:2

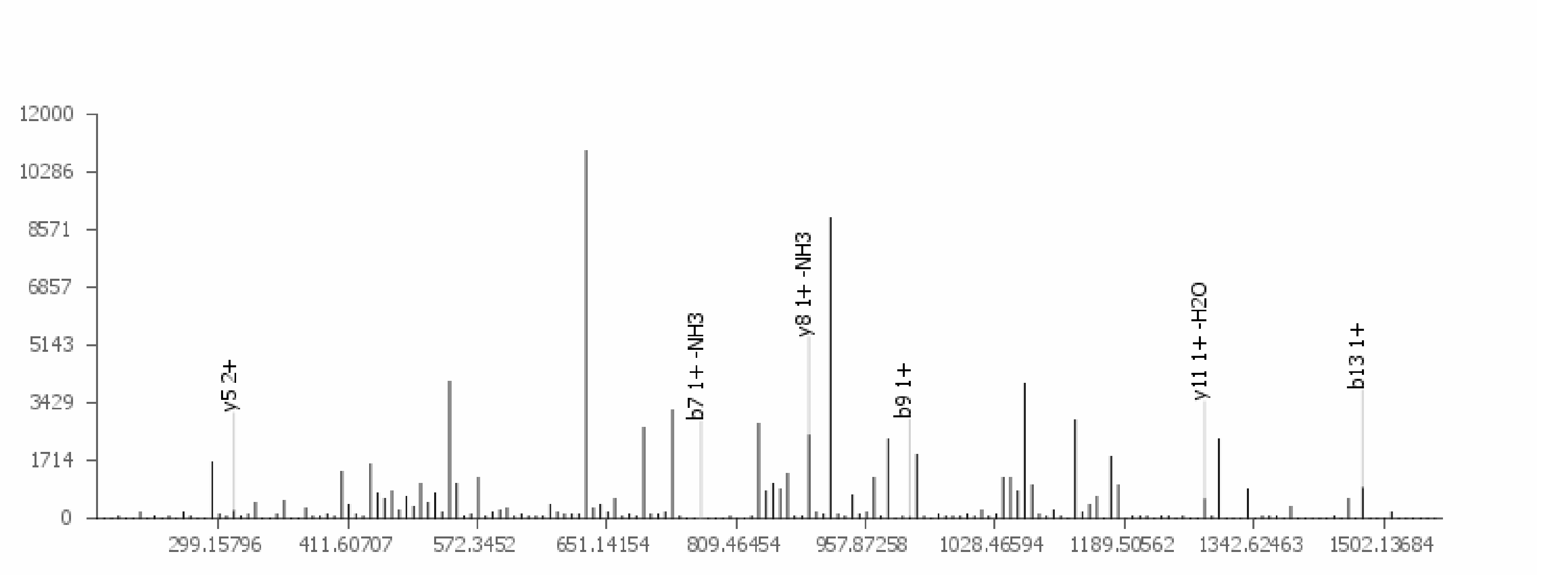

AT1G04210.1 - SSALTLLSEISGLKCL(pT)R - 1015.01981 - Charge:2

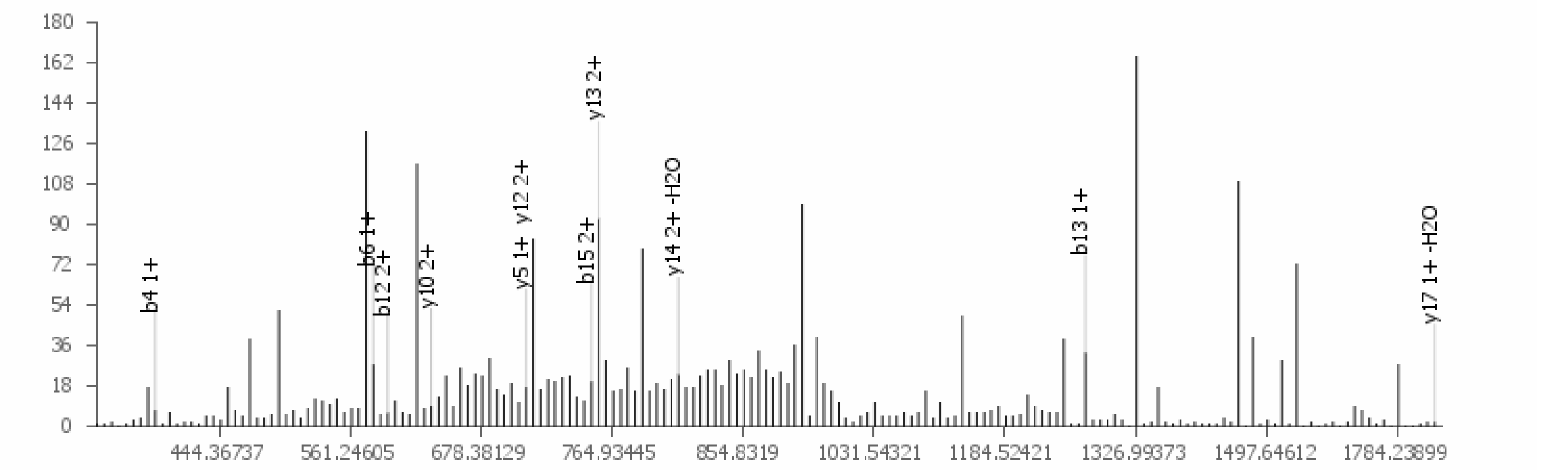

AT1G72100.1 - A(pT)(pS)VGILVGLFGHVLSR - 943.475636 - Charge:2

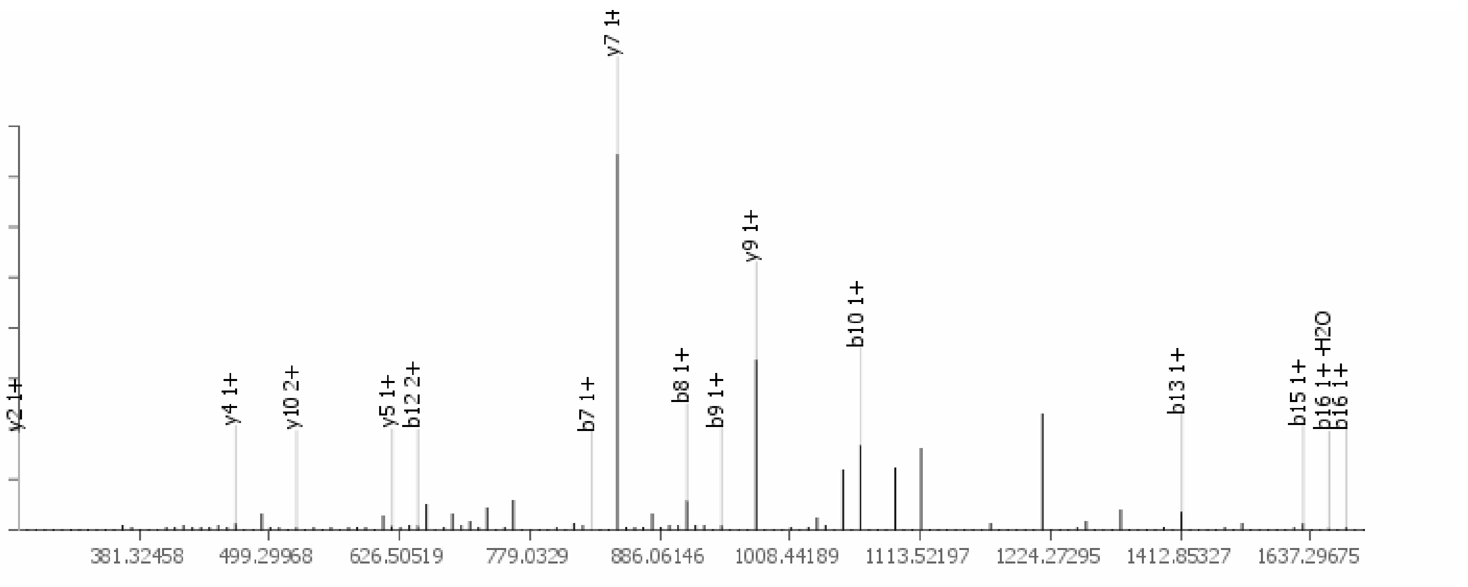

AT2G46340.1 - SNMLVAAN(pS)TGNMKLLK - 936.458482 - Charge:2

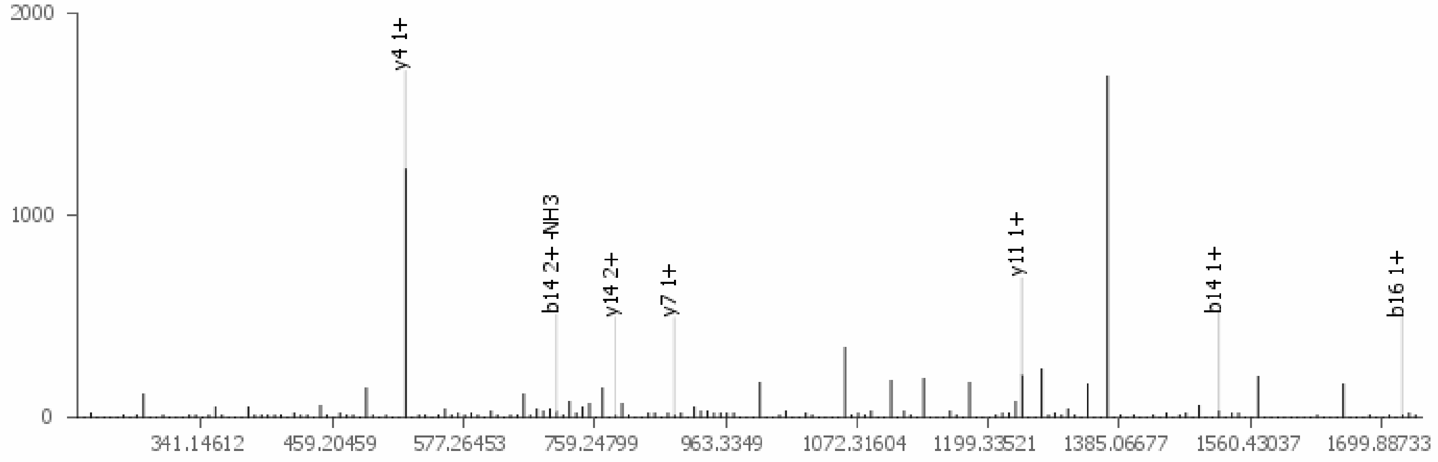

AT3G07520.1 - (pT)KVPVISSFQVPSSLSLAK - 1034.558778 - Charge:2

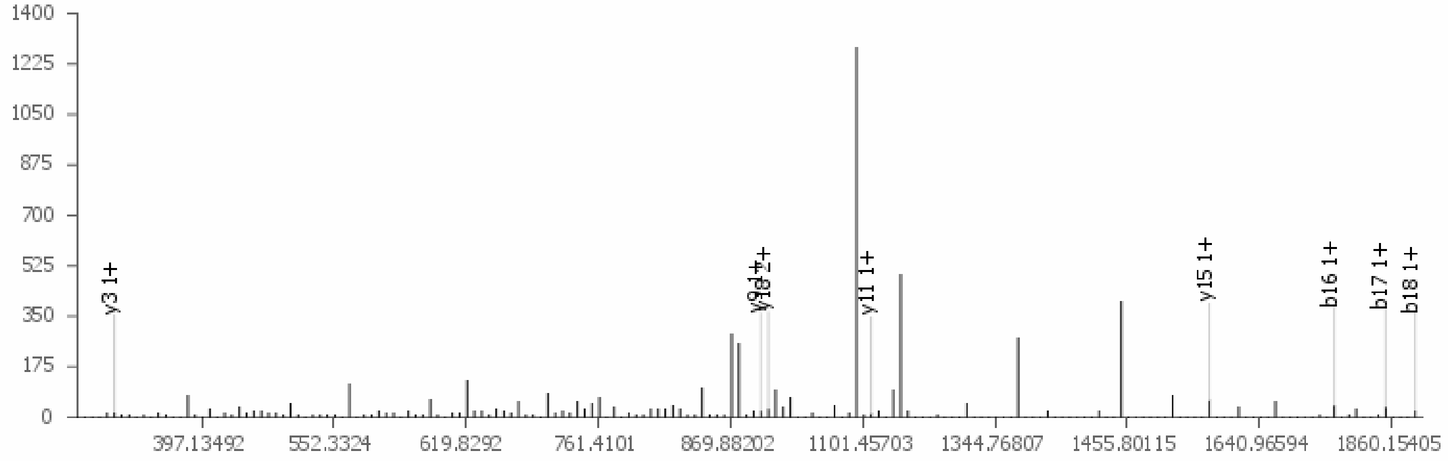

AT1G04010.1 - L(pT)FETALKLR - 636.340185 - Charge:2

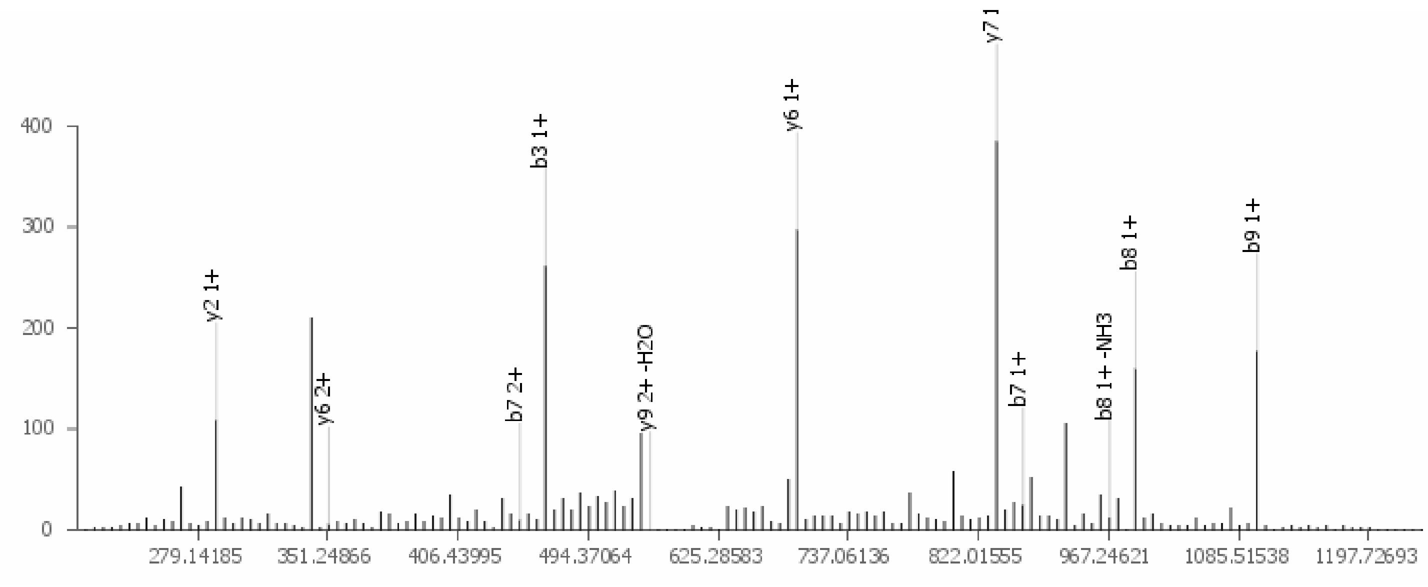

AT3G21380.1 - IFGQDTEVI(t)(s)LIFK - 895.959577 - Charge:2

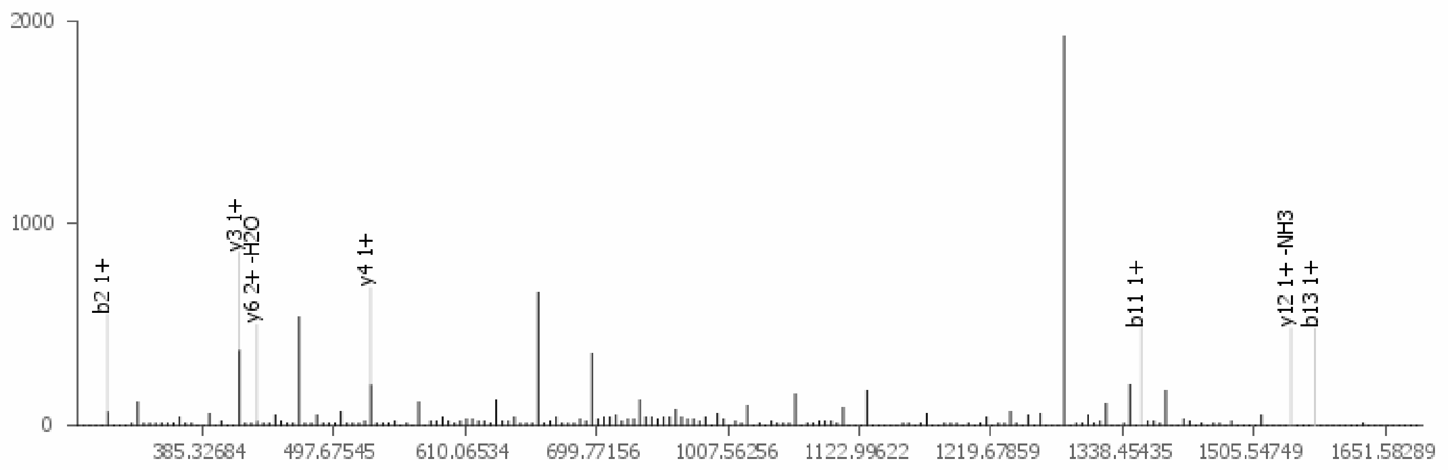

AT1G52590.1 - AILIPA(pS)FGRL(pT)ITSR - 938.486822 - Charge:2

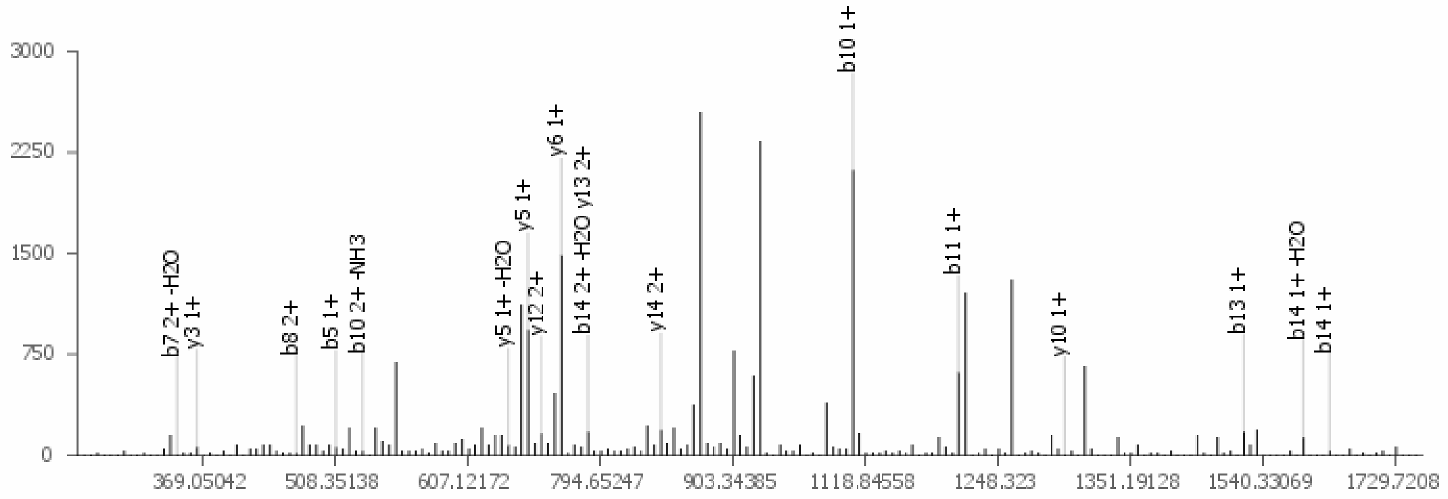

AT3G02700.1 - SGQAASIVAAA(pS)VLL(pS)SPLR - 1029.514513 - Charge:2

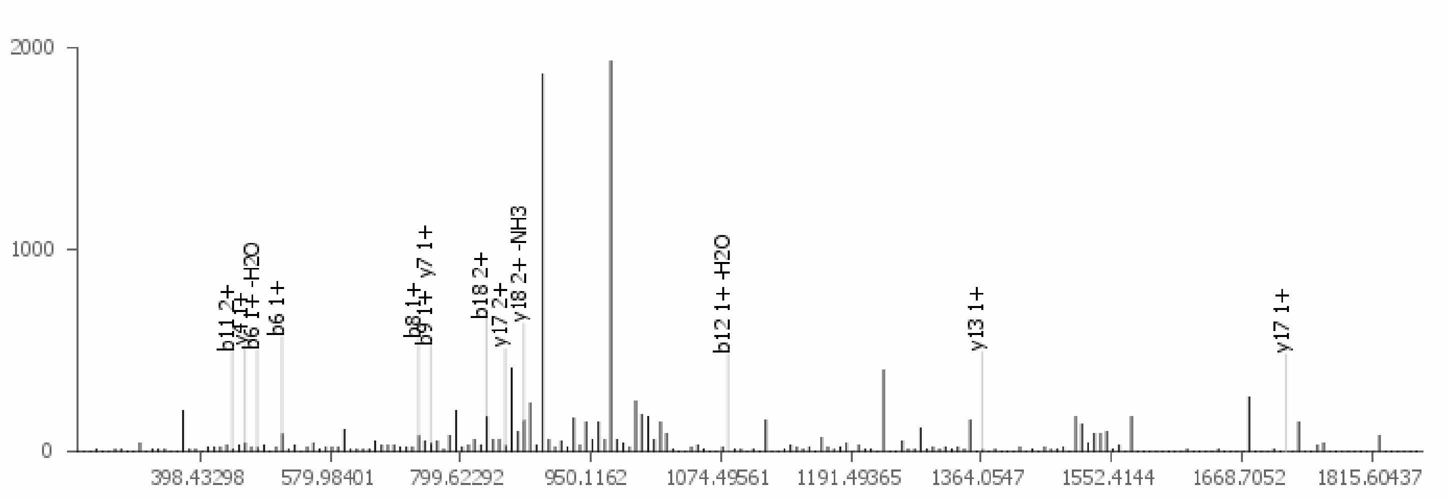

AT2G21550.1 - KIFWLGVVEEILQLI(pS)GSNNPK - 855.450218 - Charge:3

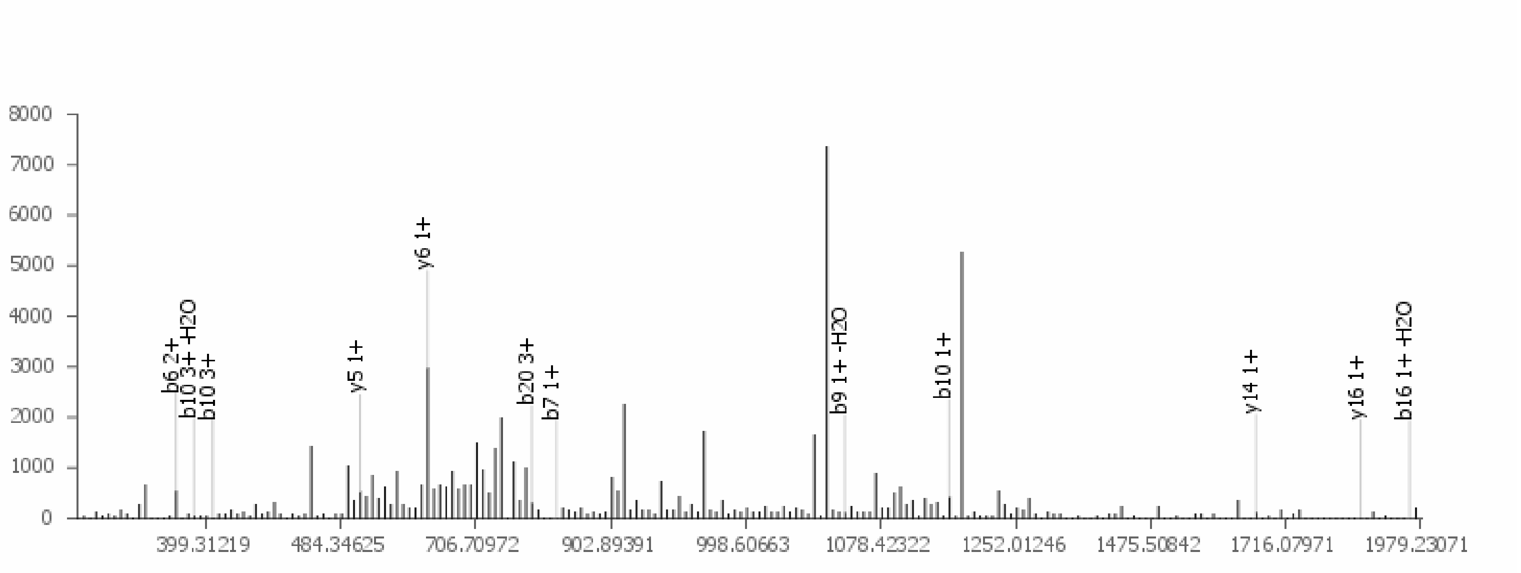

AT2G45030.1 - IVL(pT)DGA(s)HAVD(s)SELAFK - 1060.469531 - Charge:2

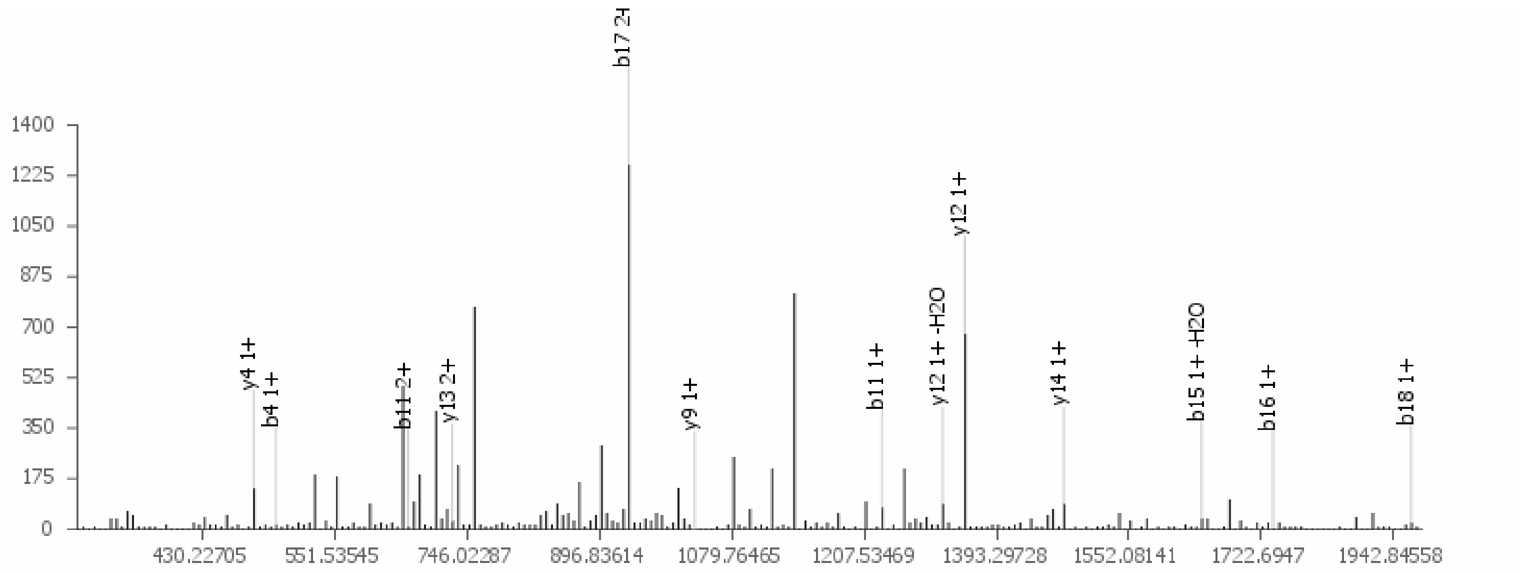

AT4G32980.1 - DISNNVVTQGF(pS)QLIFGSK - 1067.518216 - Charge:2

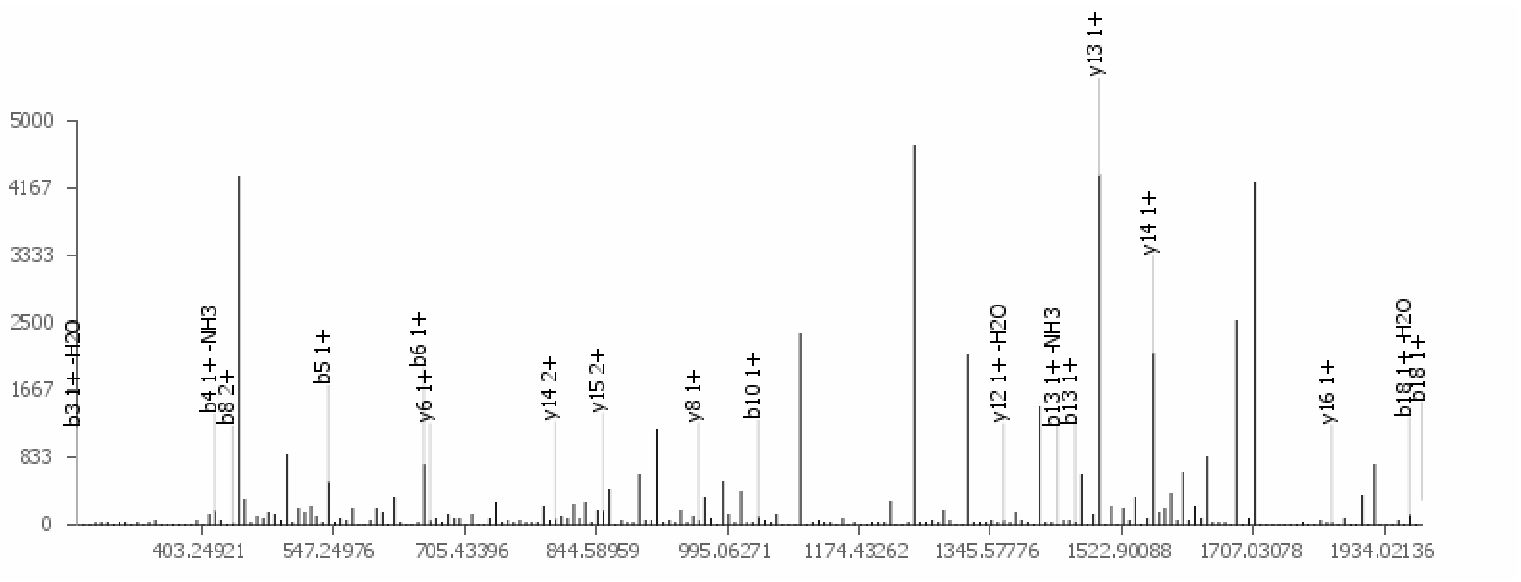

AT5G24840.1 - VTE(pY)VKER - 552.25968 - Charge:2

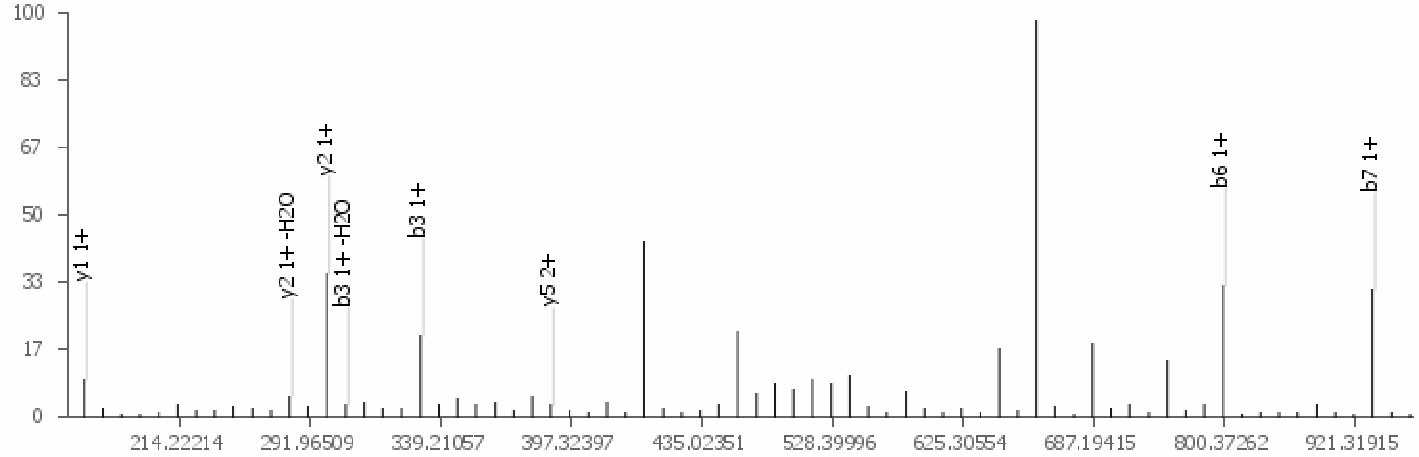

AT5G48770.1 - SLIHI(pS)KDGYIDIHSLIK - 1066.56177 - Charge:2

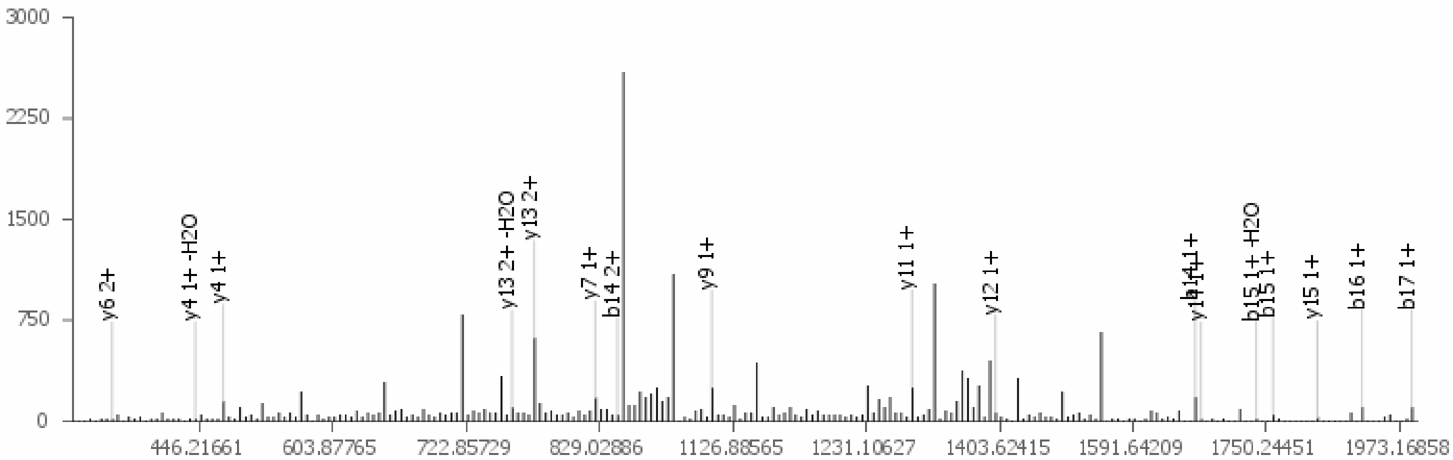

AT3G06340.1 - F(pS)H(pS)IPSFRLTGTEGR - 976.420064 - Charge:2

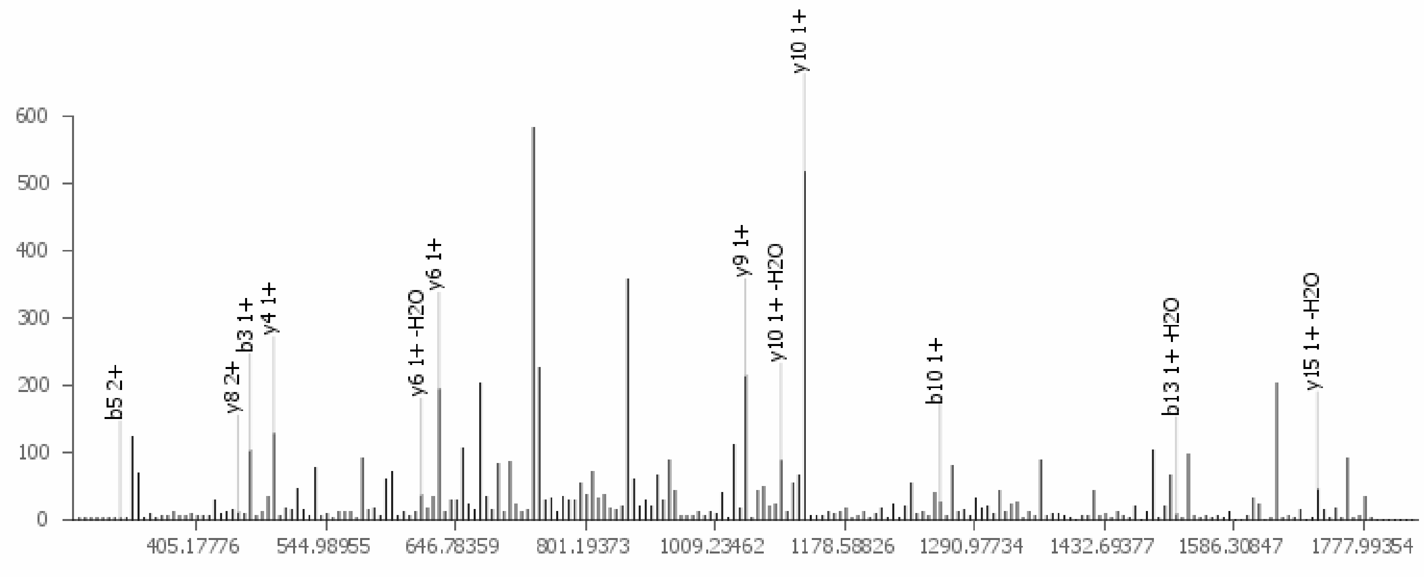

AT5G37430.1 - L(oxM)SFQLLNILE(pS)AKK - 915.977855 - Charge:2

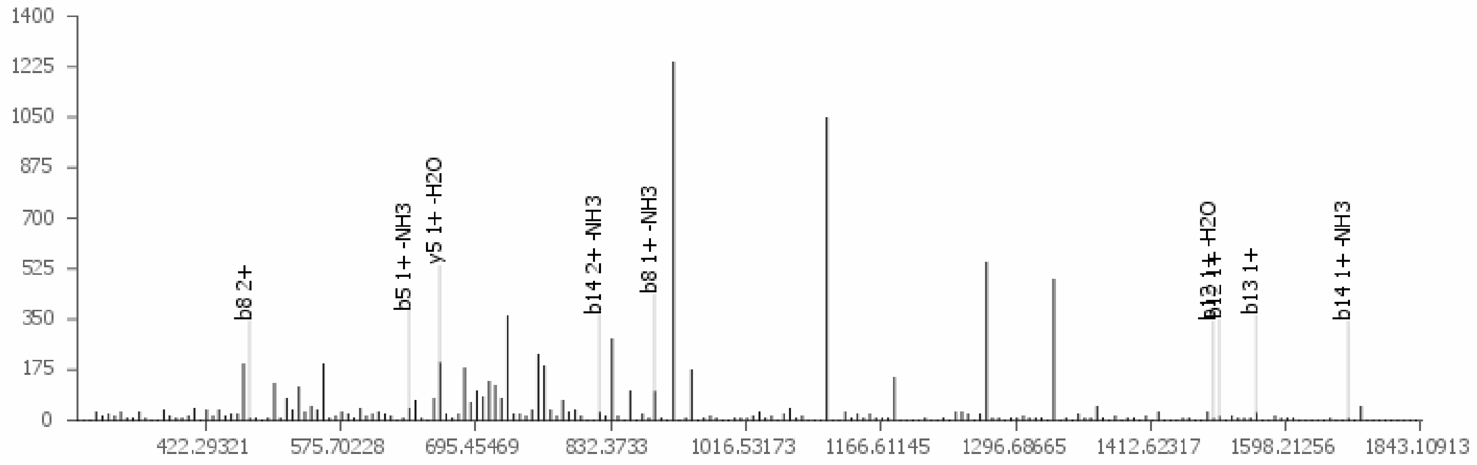

AT1G75210.1 - EPNR(pY)INEDTTIVPLIK - 1048.033088 - Charge:2

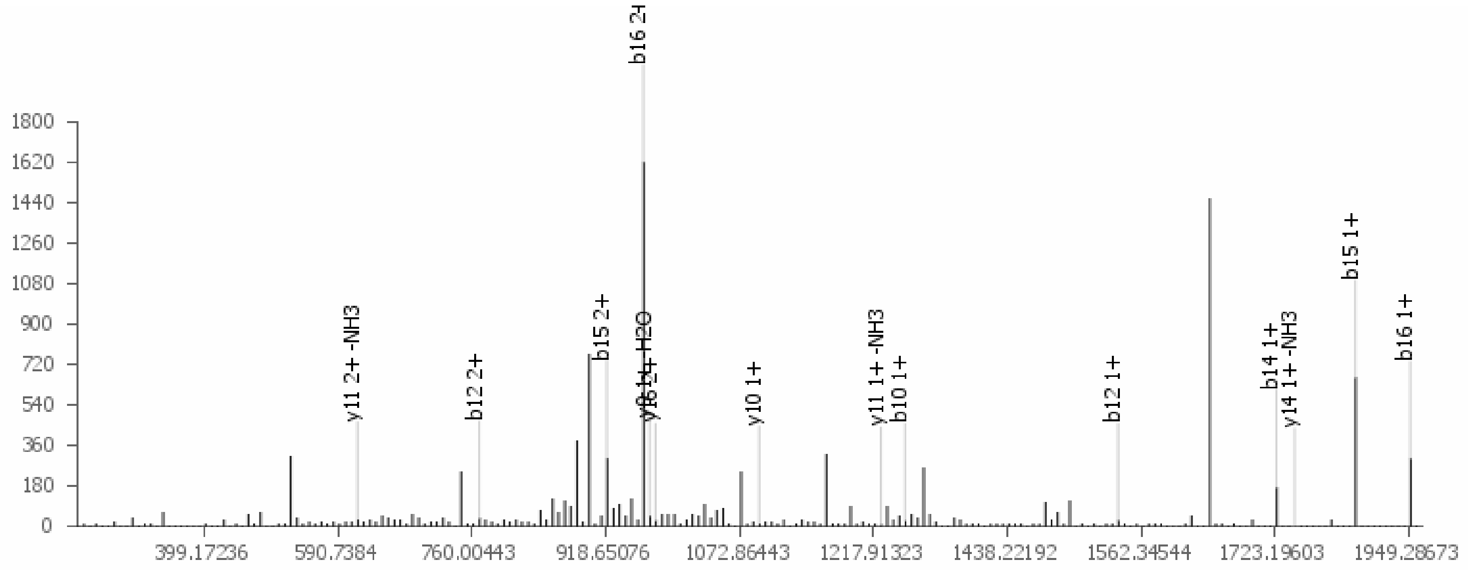

AT3G49590.1 - S(pS)QDAAVGVLVHMLK - 817.898283 - Charge:2

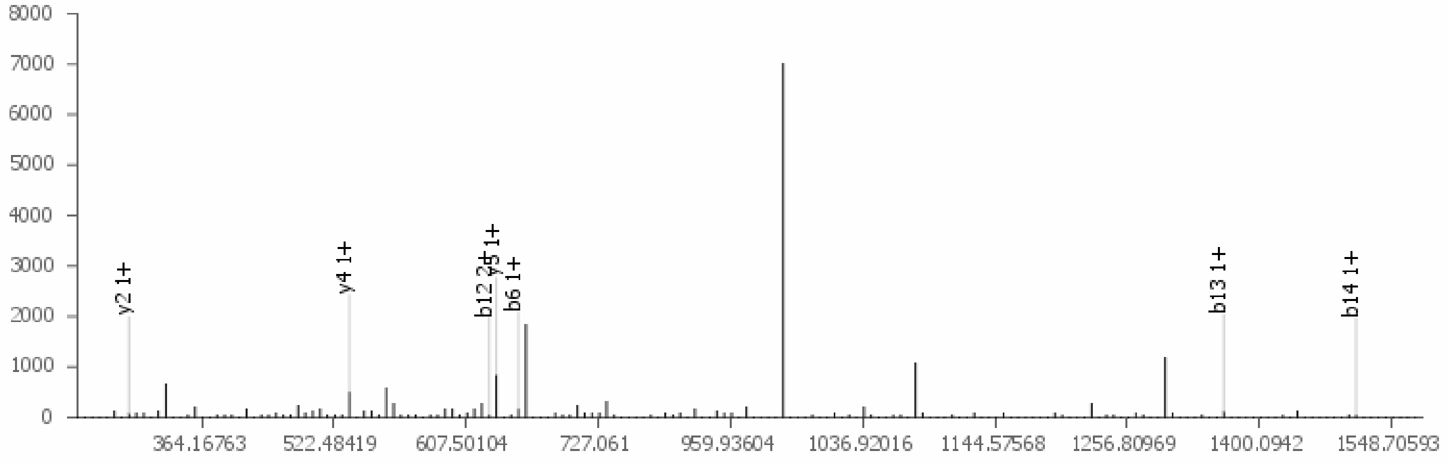

AT3G22400.1 - E(oxM)LAGLNPVVI(pS)RLQEFPPK - 1167.591795 - Charge:2

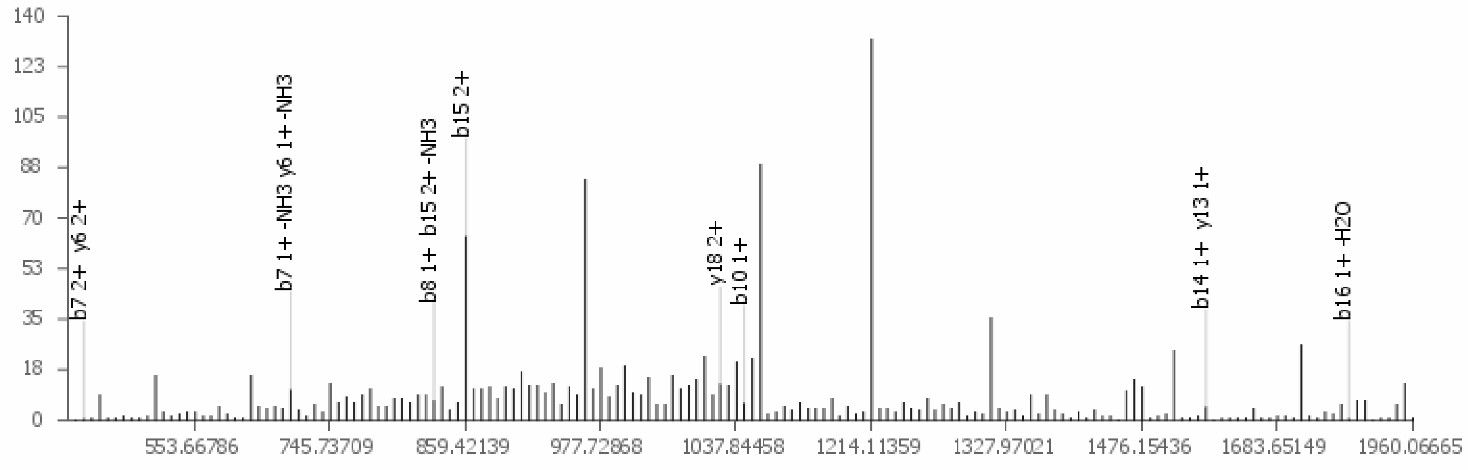

AT3G08680.1 - LDALRII(pS)LR - 625.350048 - Charge:2

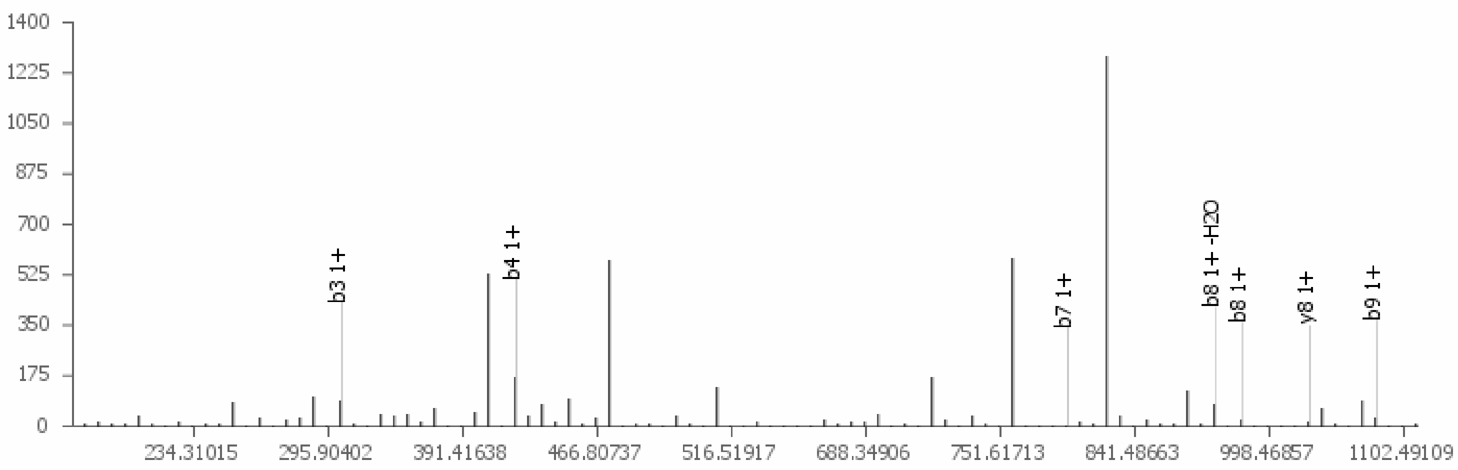

AT3G48170.1 - (pS)PIIVFDDVDIDK - 778.373646 - Charge:2

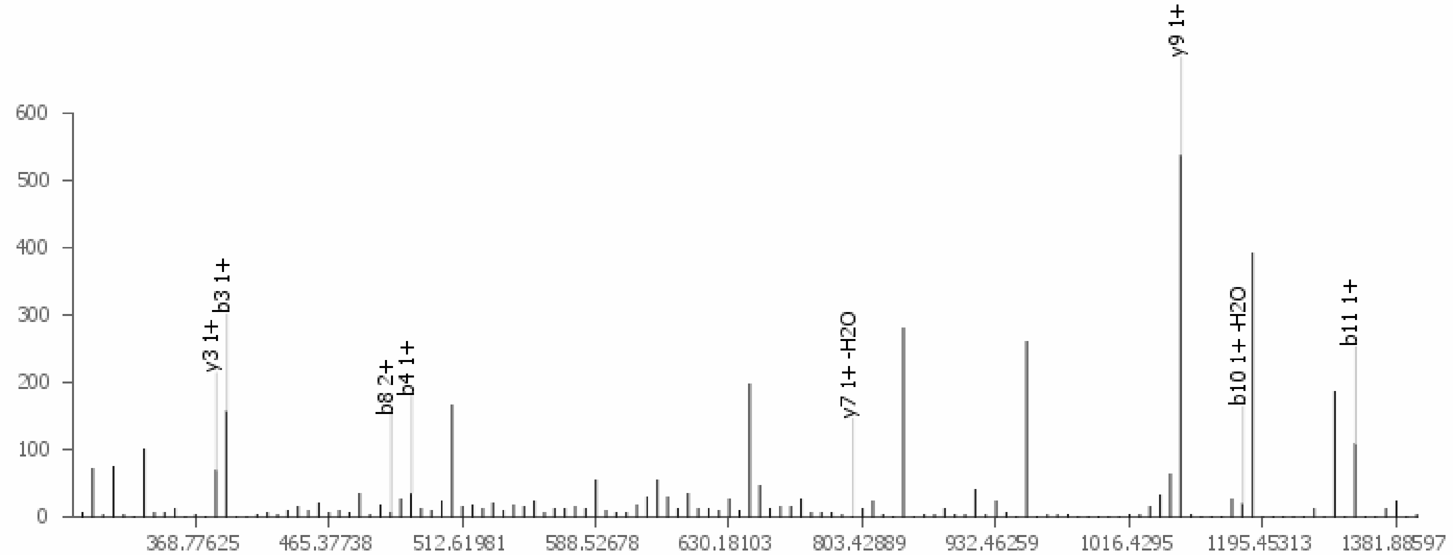

AT4G38830.1 - LISSLS(pS)LR - 528.275638 - Charge:2

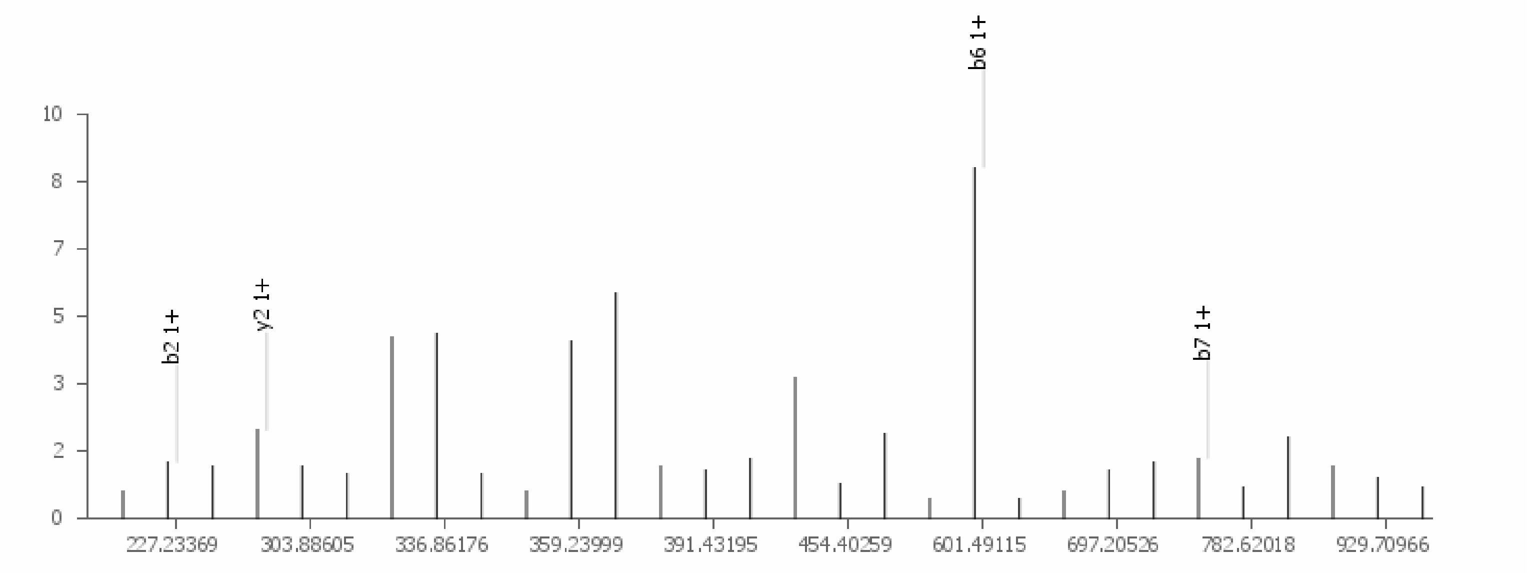

AT1G33490.1 - I(pT)GGPHFPLTSDALK - 817.40478 - Charge:2

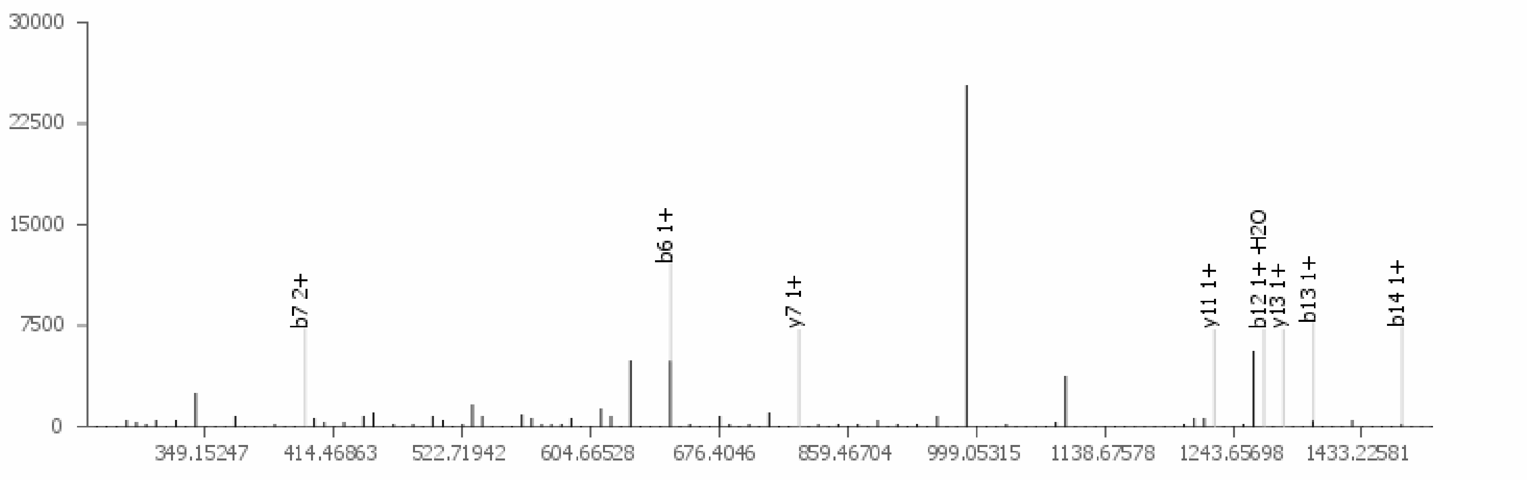

AT3G03890.1 - TGLRITLHGDAVLV(pS)EK - 944.990297 - Charge:2

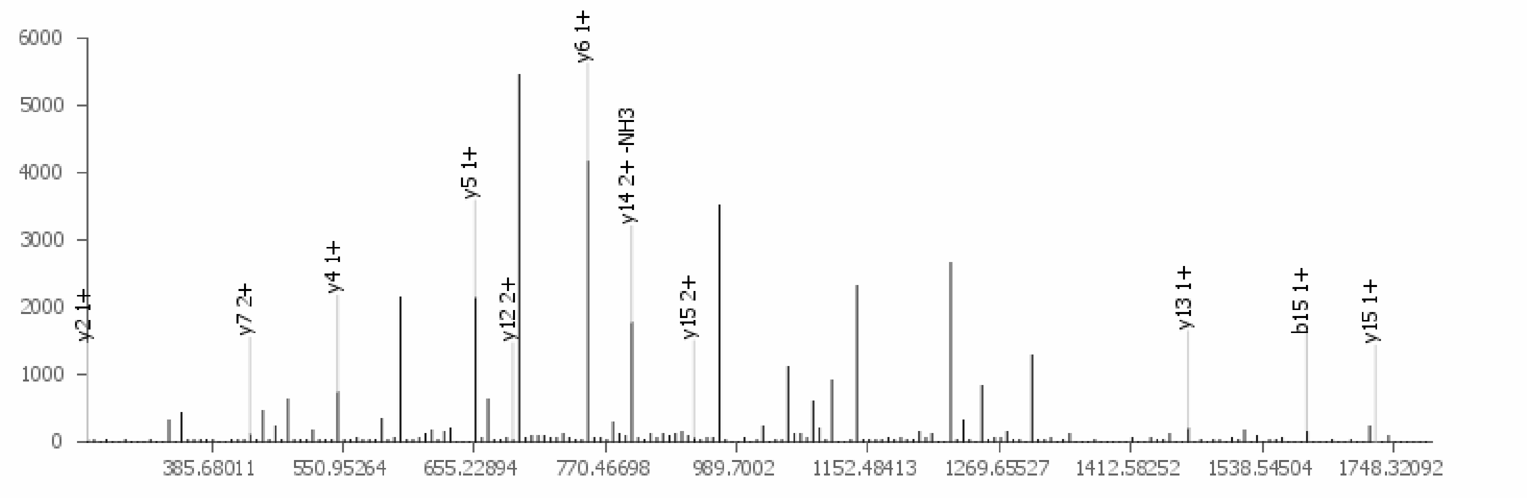

AT5G62410.1 - (pT)YDLSLFLK - 590.28746 - Charge:2

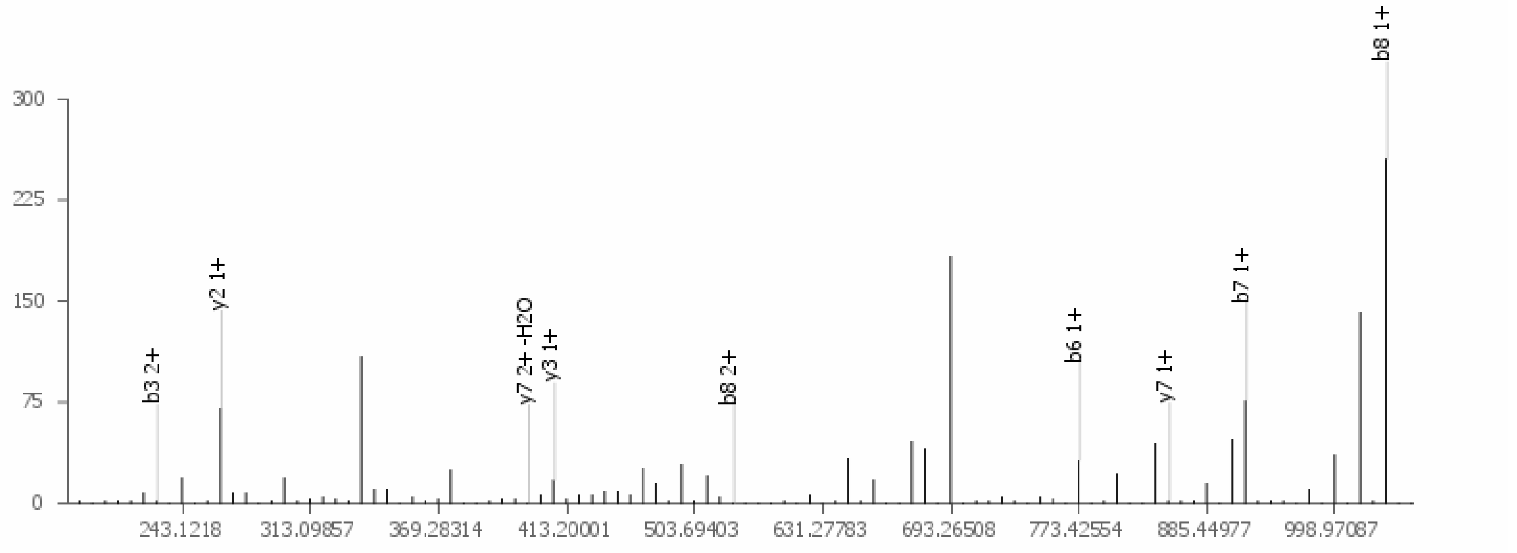

AT3G45810.1 - QIK(pT)ENGIDK - 613.299728 - Charge:2

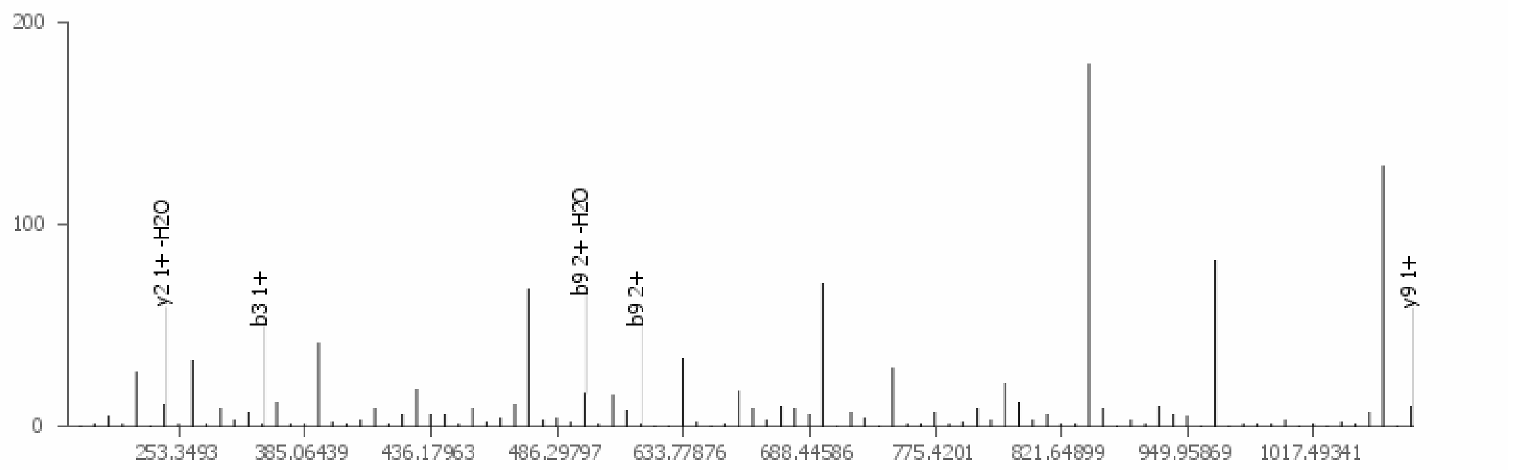

AT5G04220.2 - ELLFEP(pS)IK - 578.285057 - Charge:2

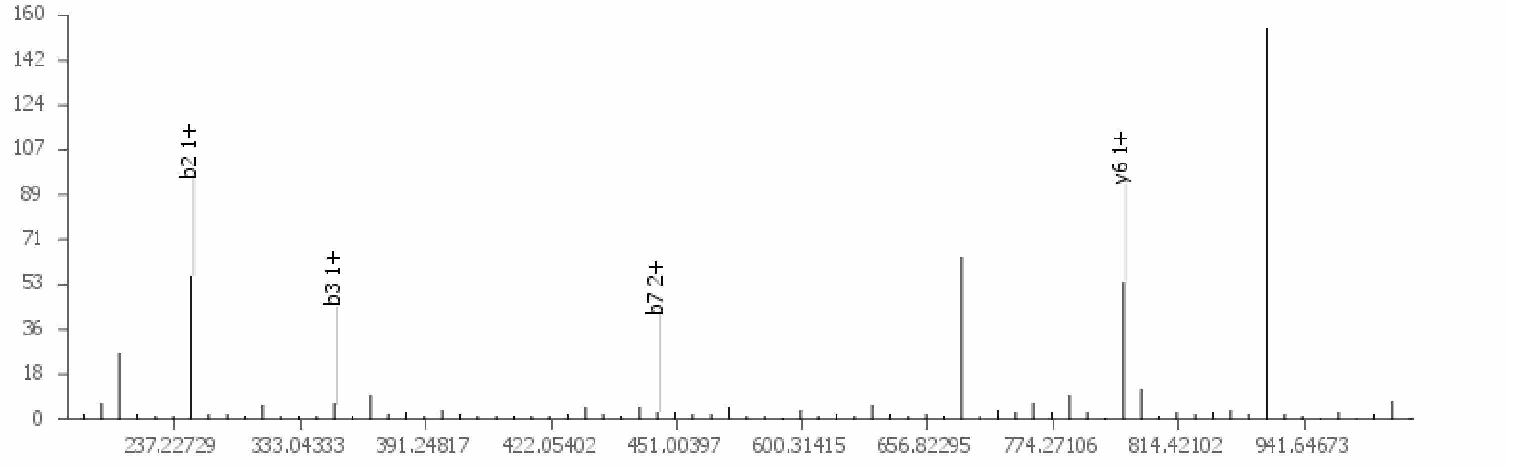

AT1G27070.1 - HRLQQLQSEL(s)(s)VLHSLR - 737.714423 - Charge:3

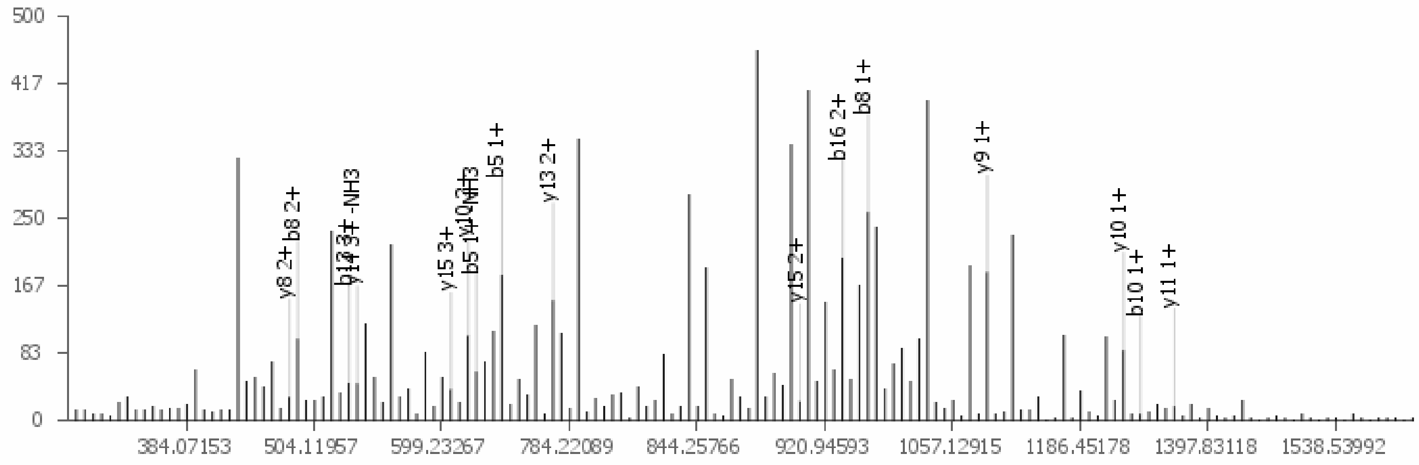

AT2G07040.1 - LE(s)F(s)QVDK - 623.293911 - Charge:2

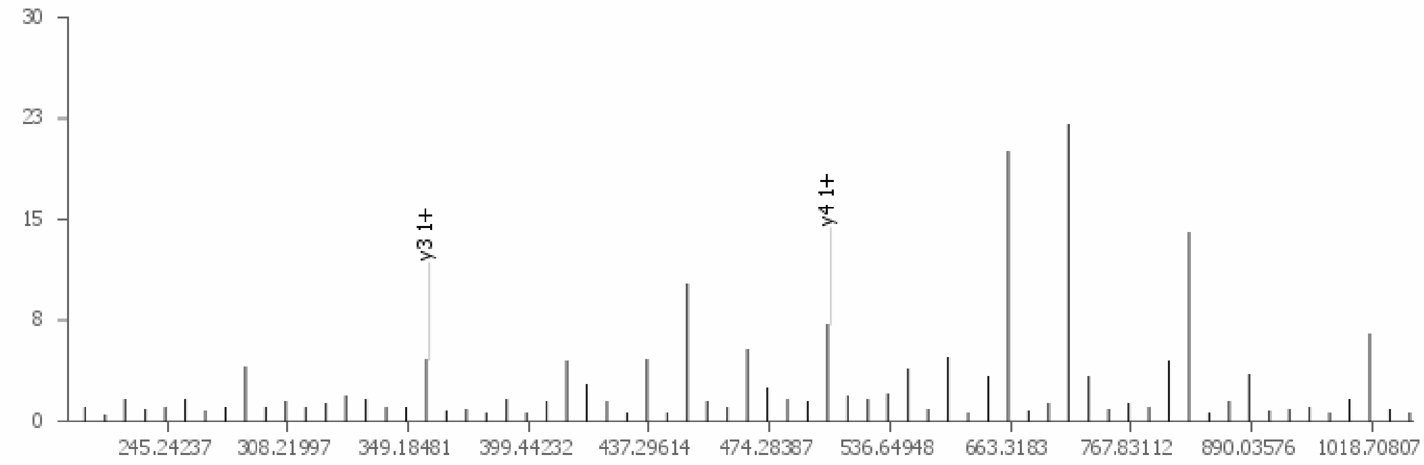

AT1G65950.1 - IATAVEGVVR(pS)SR - 712.868569 - Charge:2

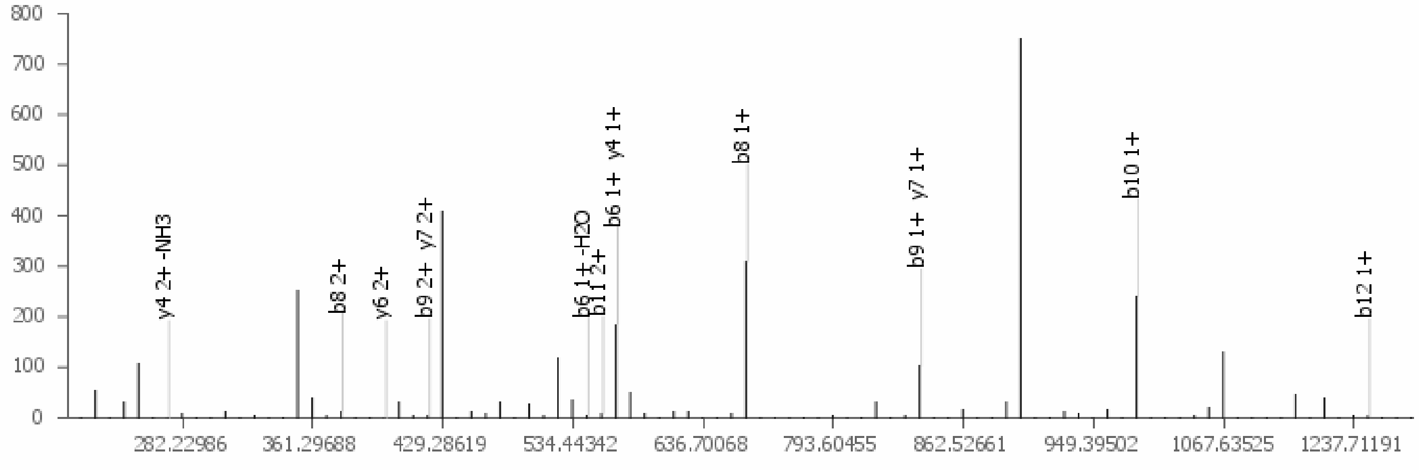

AT2G26540.1 - RSSVL(pY)PASLK - 650.831349 - Charge:2

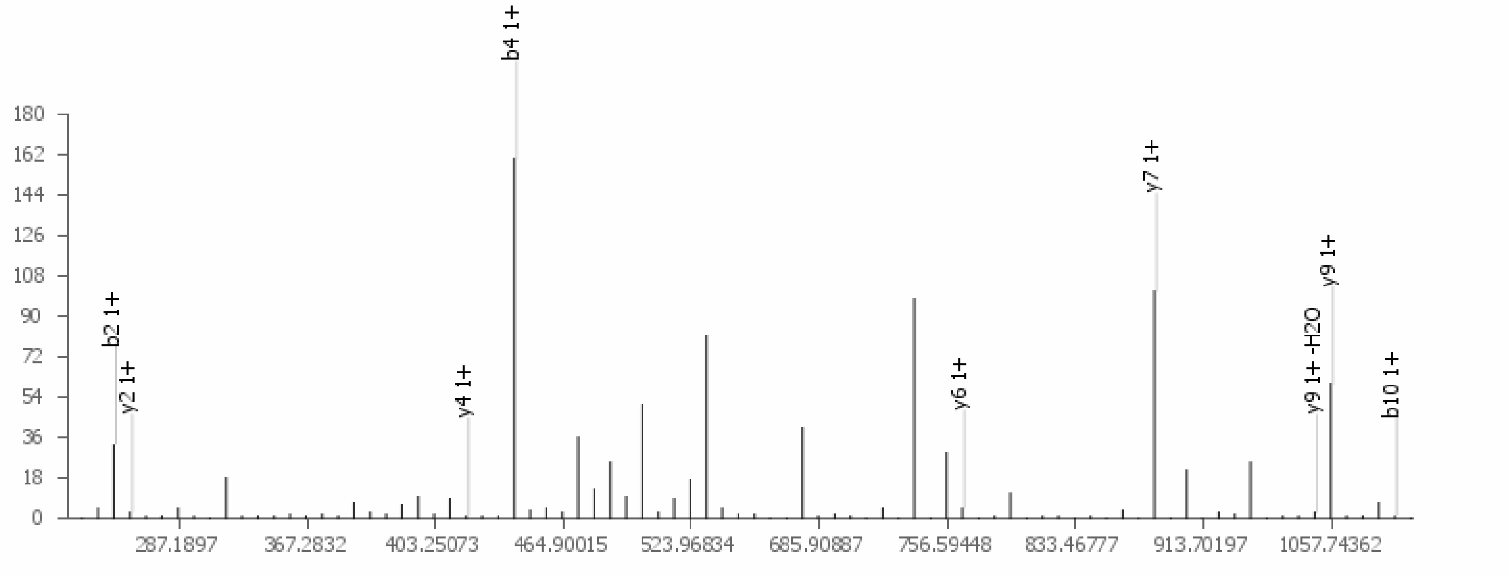

AT2G21280.1 - ALLGEGA(pT)VVLEGQK - 782.907474 - Charge:2

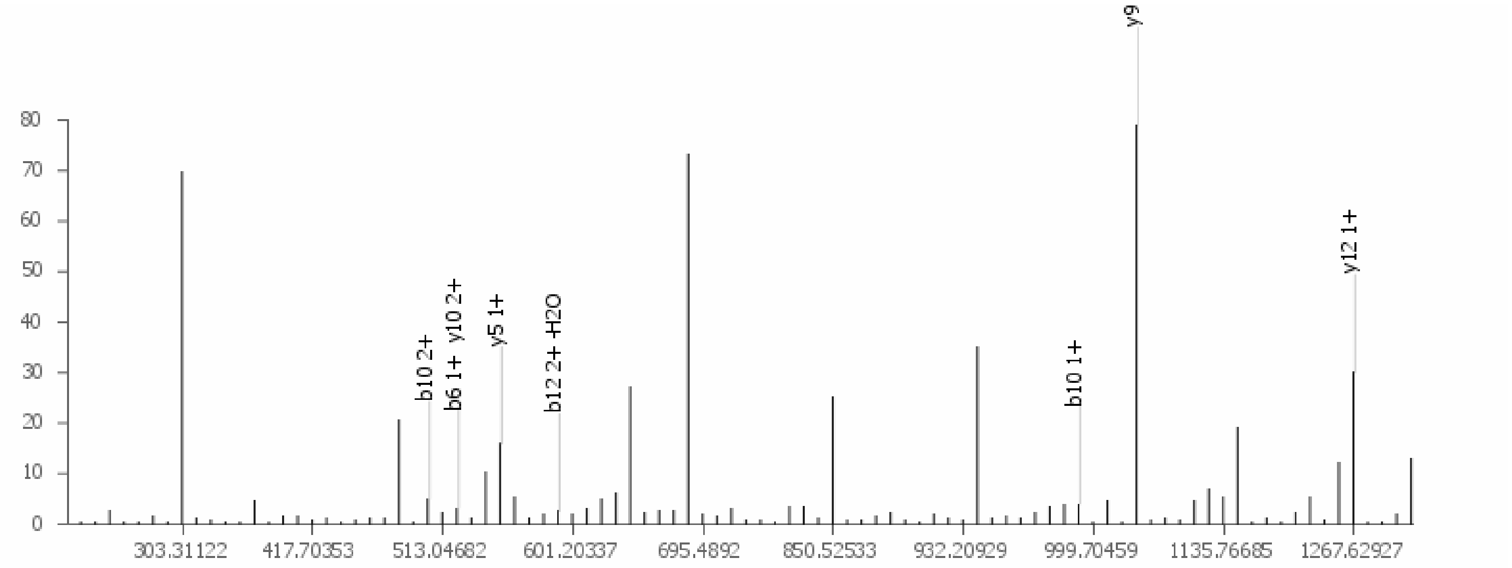

AT2G21720.1 - NFLIFSLFEEVPKGDYVLEELAEI(pS)LK - 1074.879402 - Charge:3

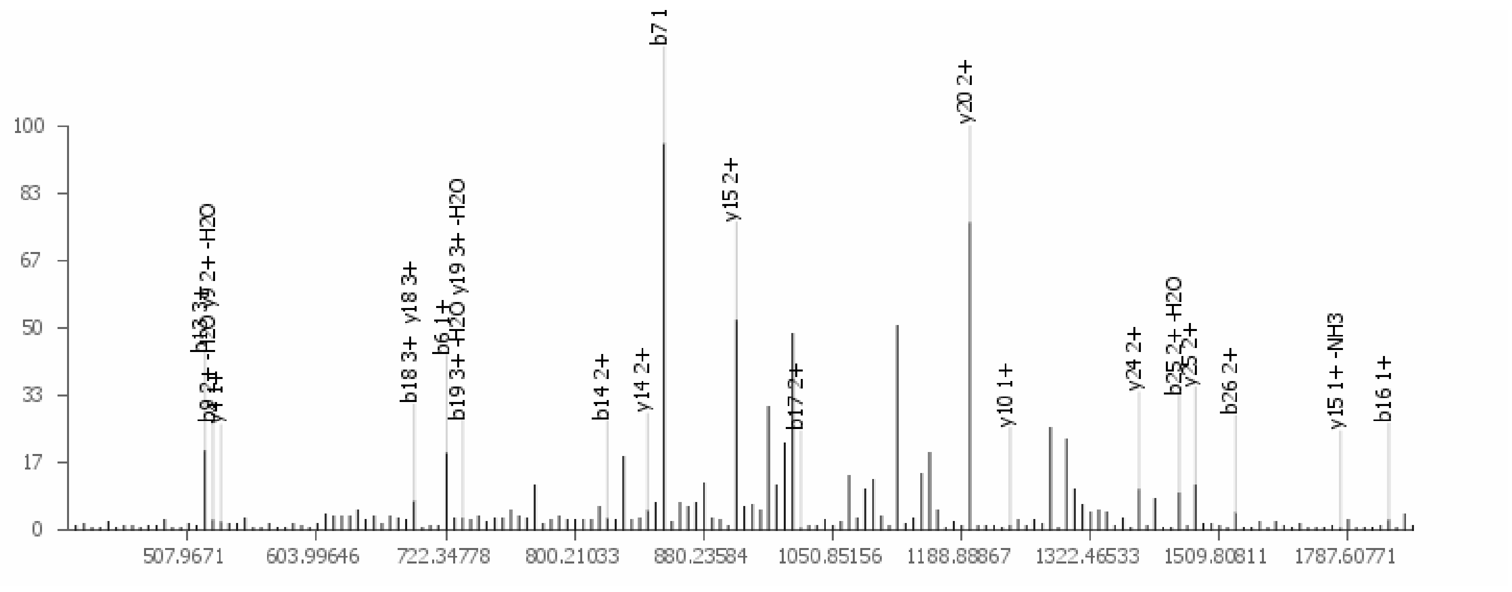

AT1G77140.1 - MQGNV(pS)KHVTLVTEMSK - 984.973875 - Charge:2

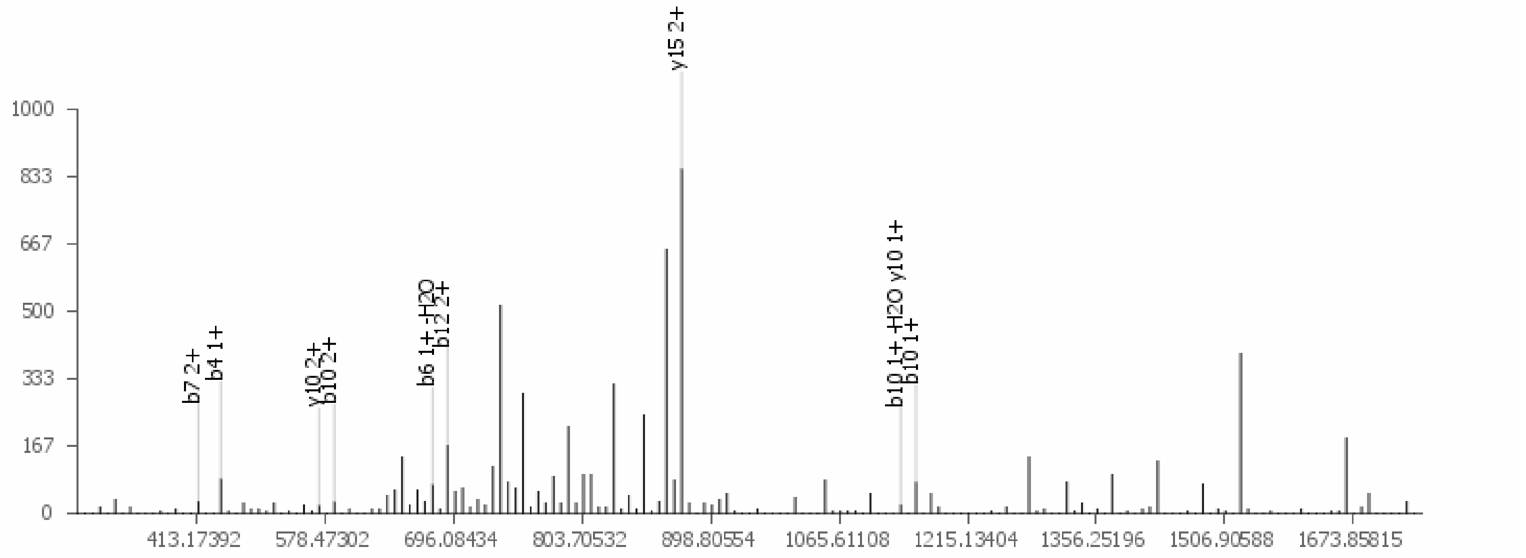

AT4G02710.1 - (pT)IGDKLTDAETENLQLK - 984.975226 - Charge:2

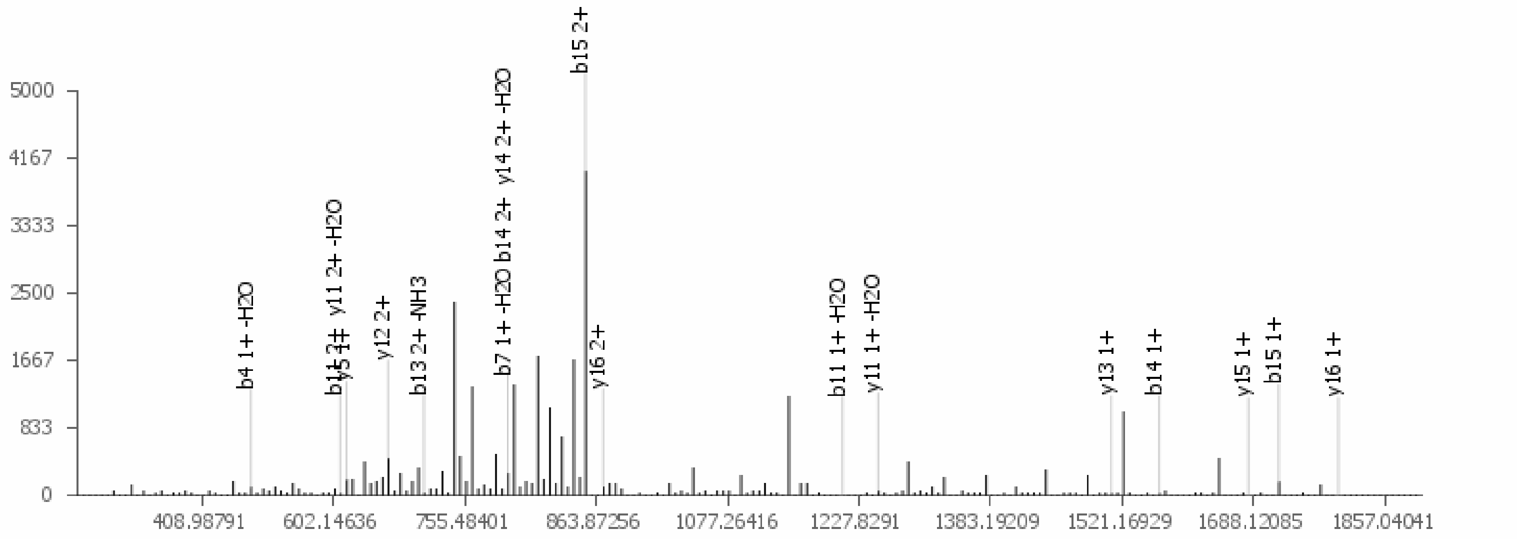

AT1G66200.1 - (pS)LLADLVNLDISDNSEK - 963.463021 - Charge:2

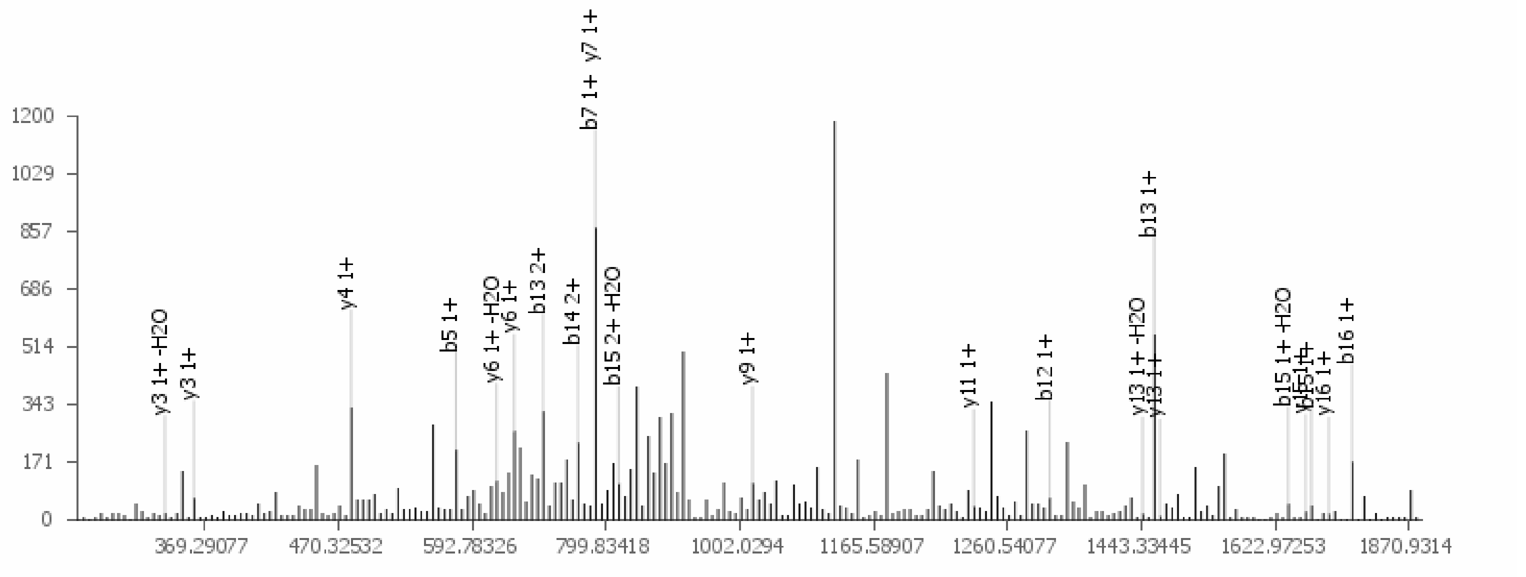

AT4G29450.1 - KGAYSGPLLP(pS)GK - 677.844196 - Charge:2

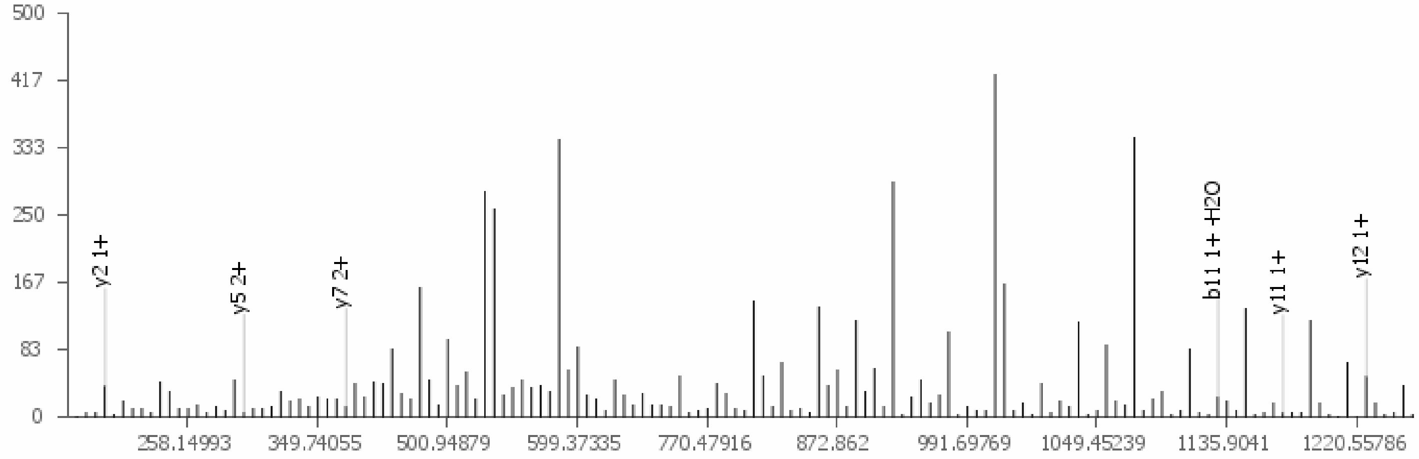

AT2G46340.1 - SNMLVAAN(s)(t)GNMKLLK - 936.456576 - Charge:2

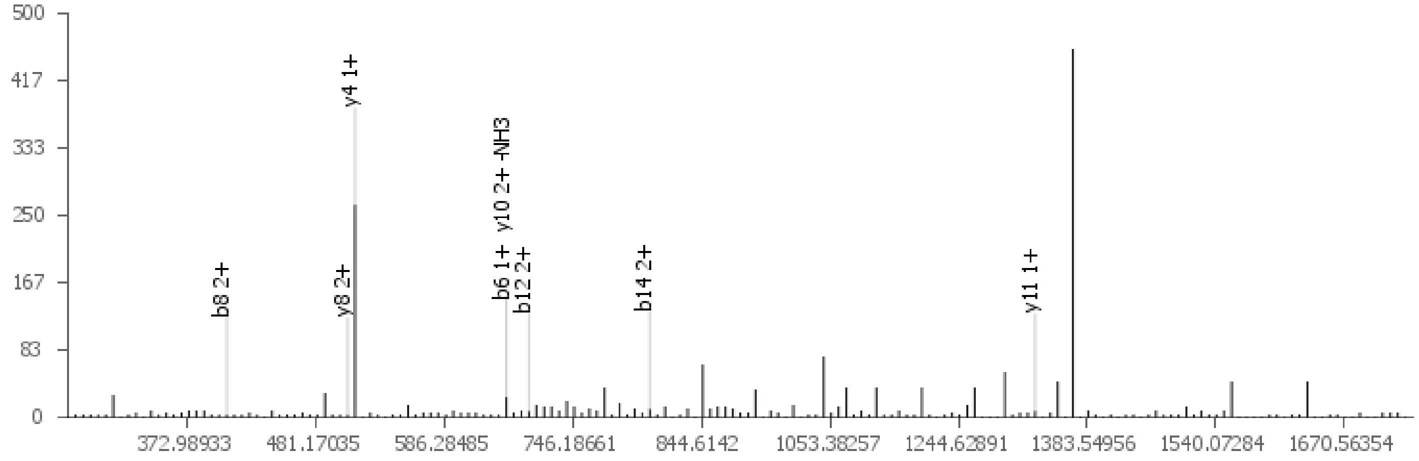

AT5G08390.1 - L(s)(s)(s)RNEPDLPTSSLLER - 1040.997359 - Charge:2

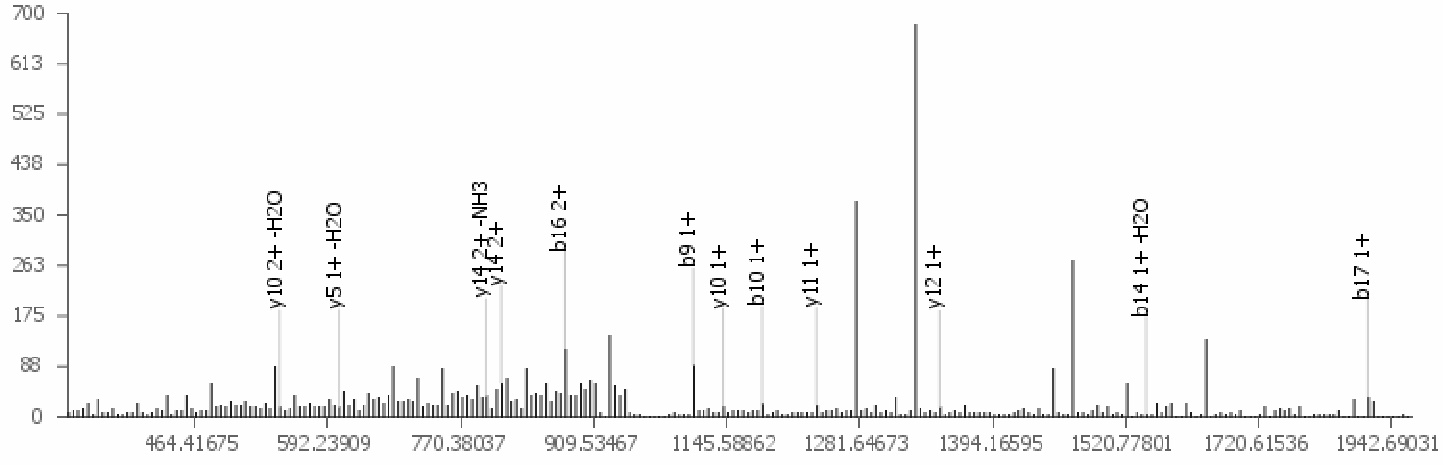

AT5G04238.1 - PV(pS)KM(pY)LPGFFK - 787.356639 - Charge:2

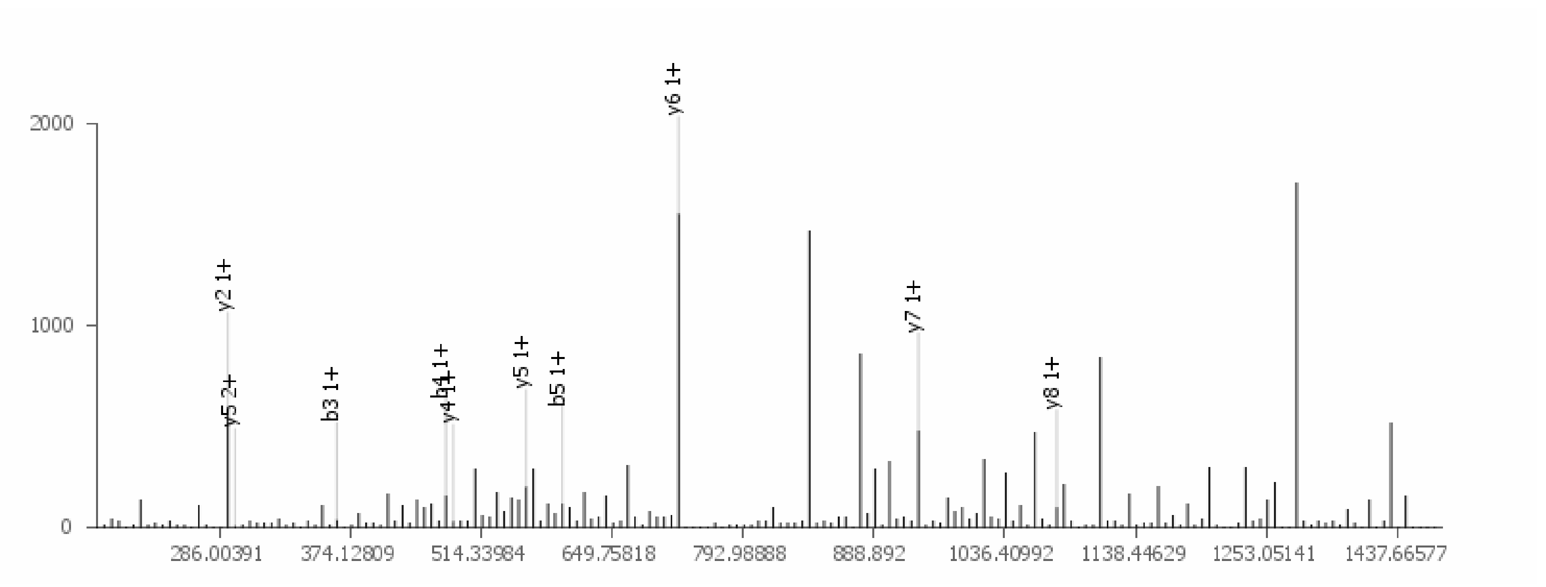

AT5G37600.1 - (pS)LVSDLINLNLSDSTDK - 957.461619 - Charge:2

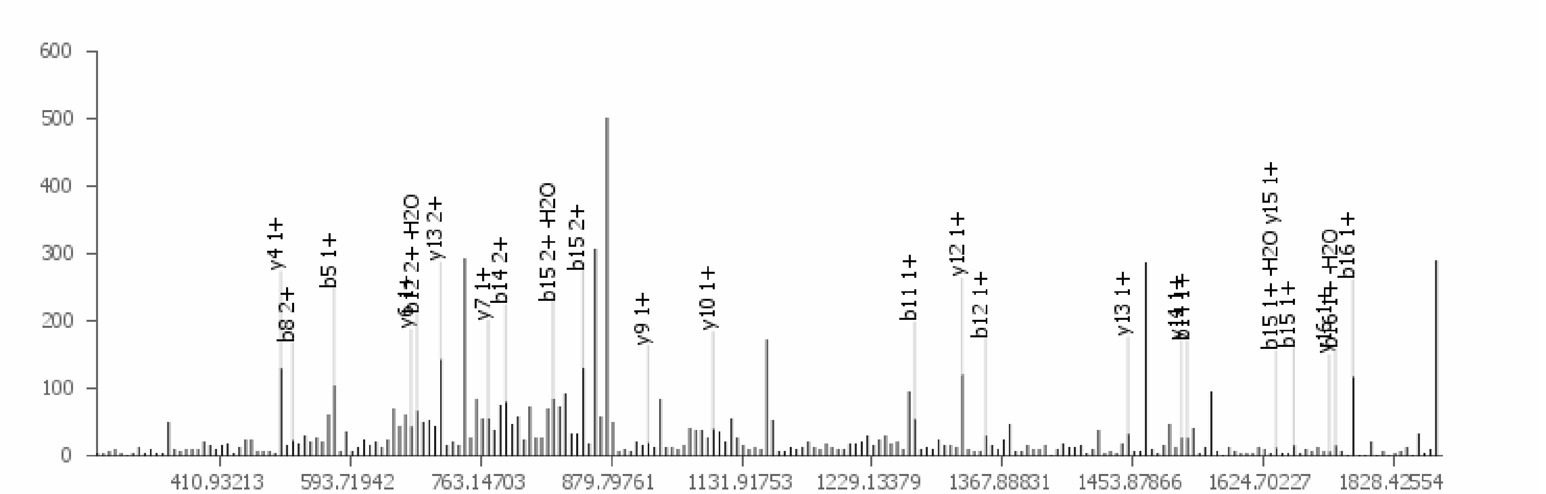

ATCG00420.1 - MQG(t)L(s)VWLAK - 657.323015 - Charge:2

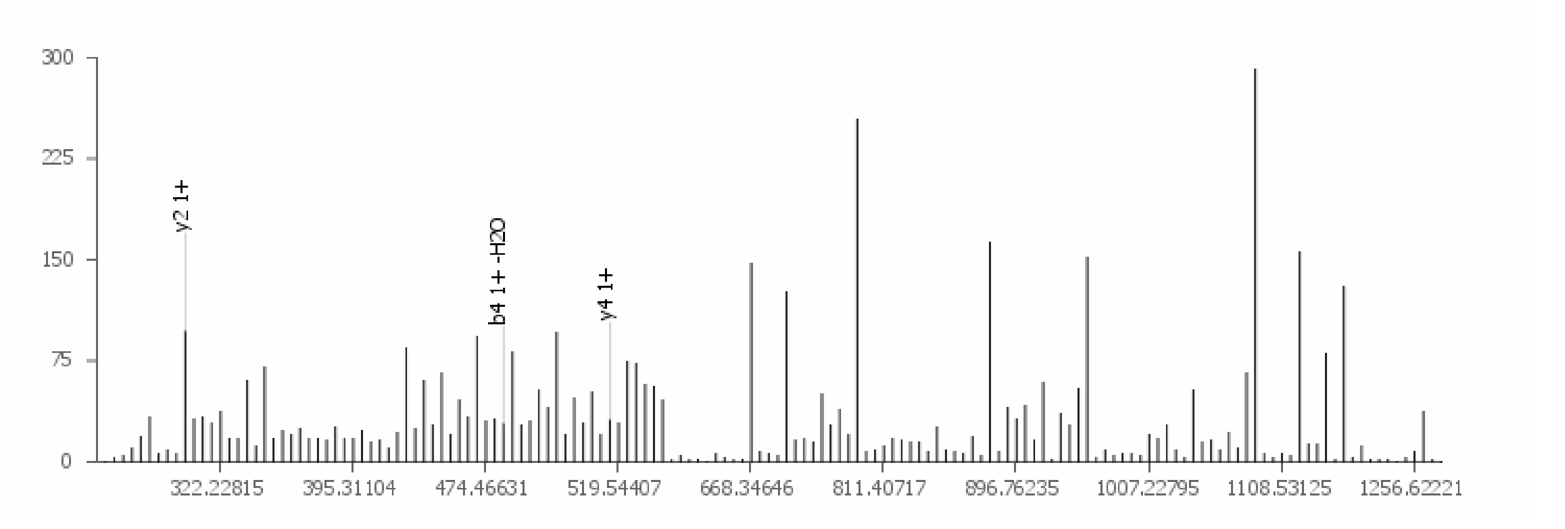

AT5G16715.1 - MLA(pS)EGIKR - 542.767734 - Charge:2

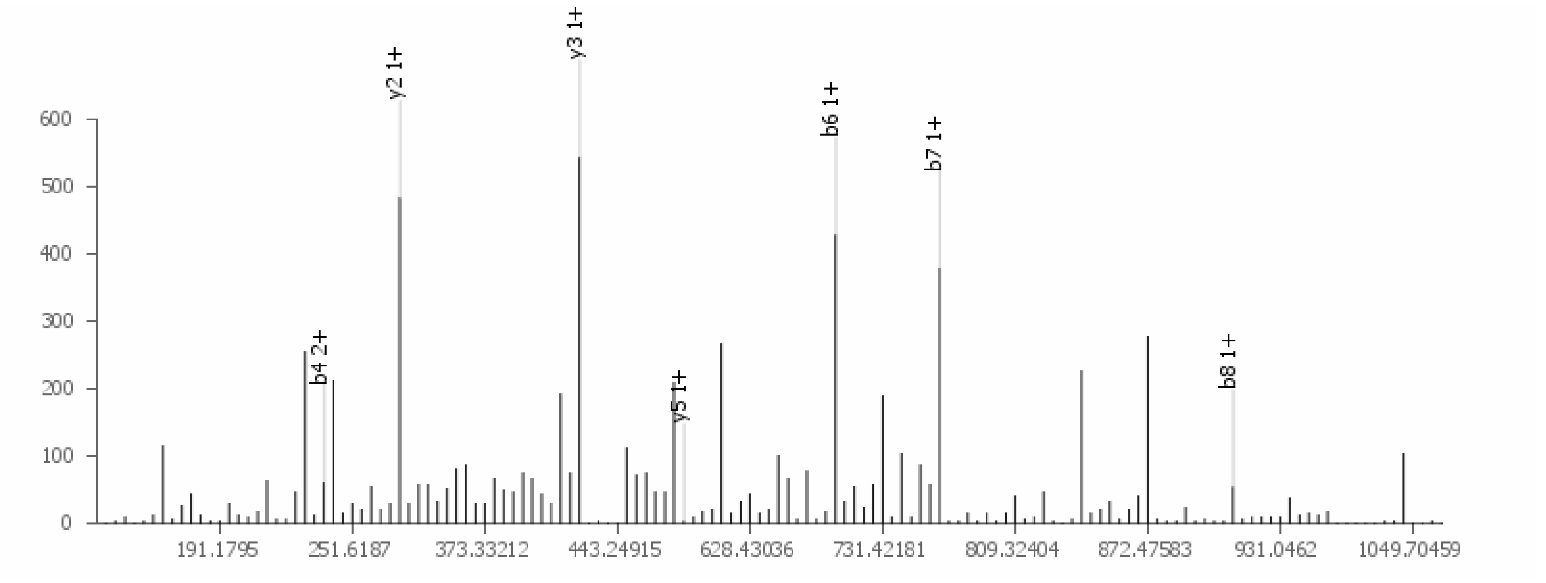

AT5G16780.1 - (pT)DEFGRTL(pT)PK - 712.794126 - Charge:2

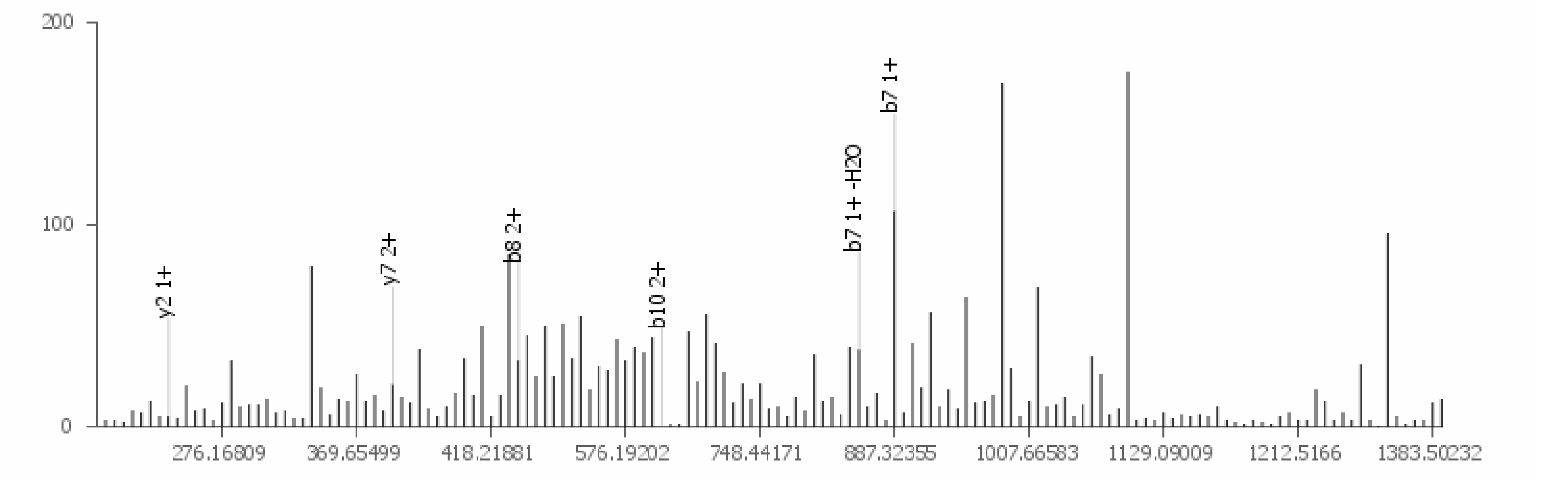

AT2G19270.1 - SSSFLSSLPPPK(pS)(pS)ISR - 968.950359 - Charge:2

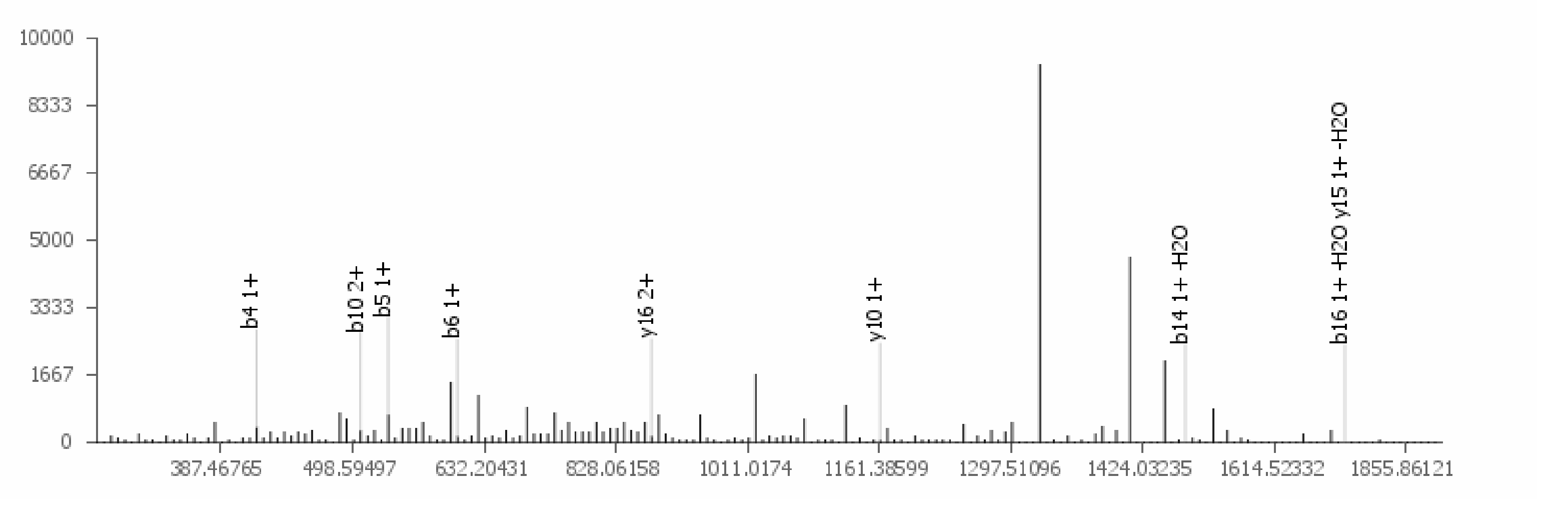

AT2G06200.1 - RSGY(oxM)DDFF(pS)IEPSGSIK - 1066.462587 - Charge:2

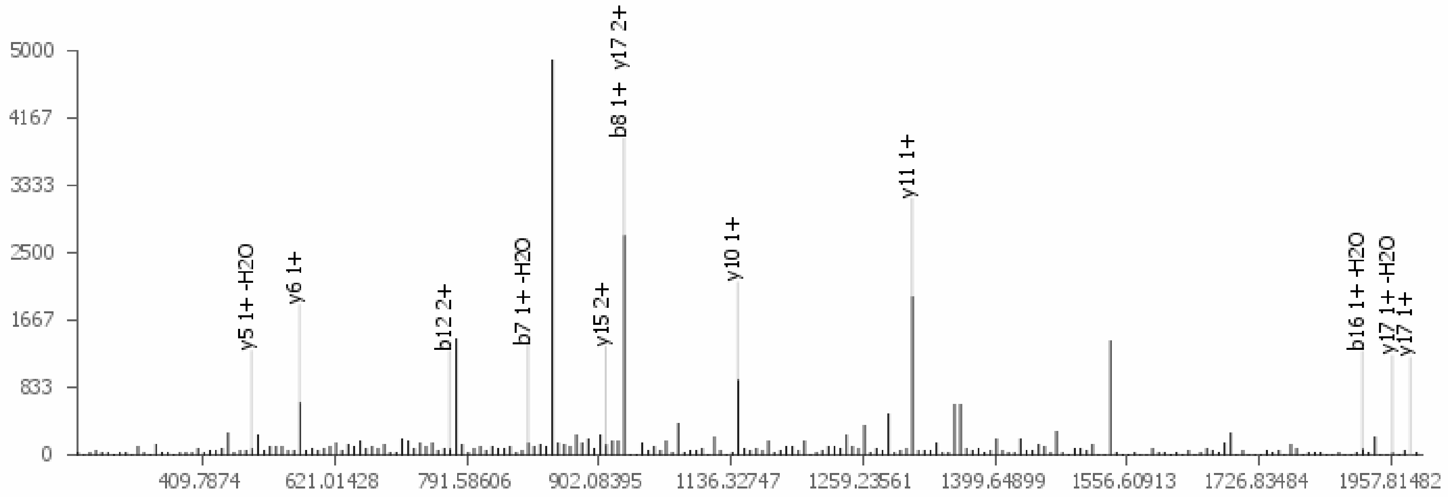

AT3G11964.1 - QMEGLAVG(pT)LK - 613.800696 - Charge:2

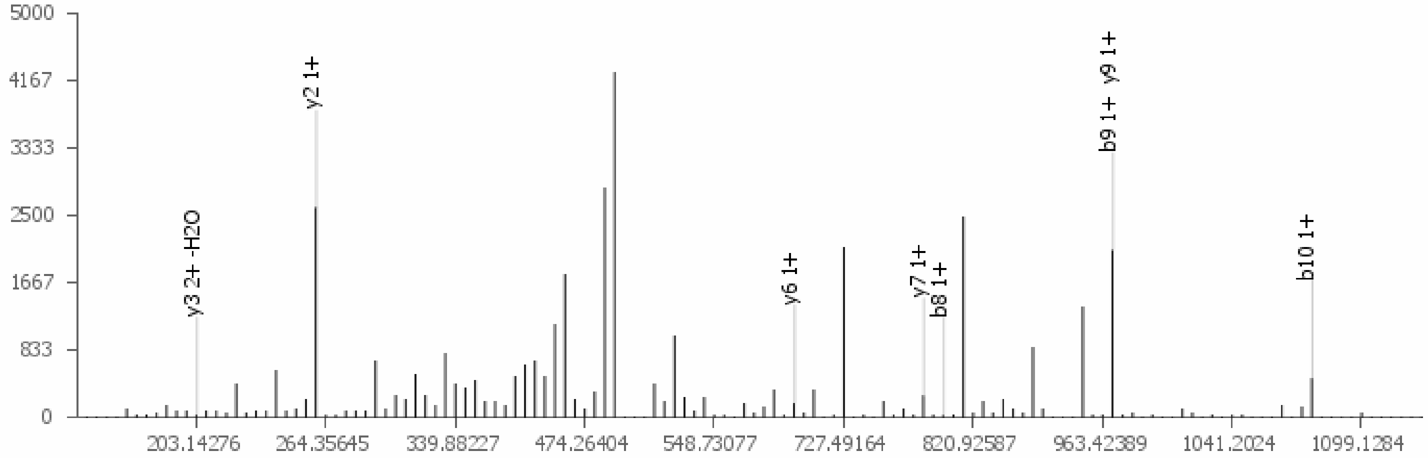

AT1G53885.1 - E(pS)Q(oxM)LIDDRK - 665.787299 - Charge:2

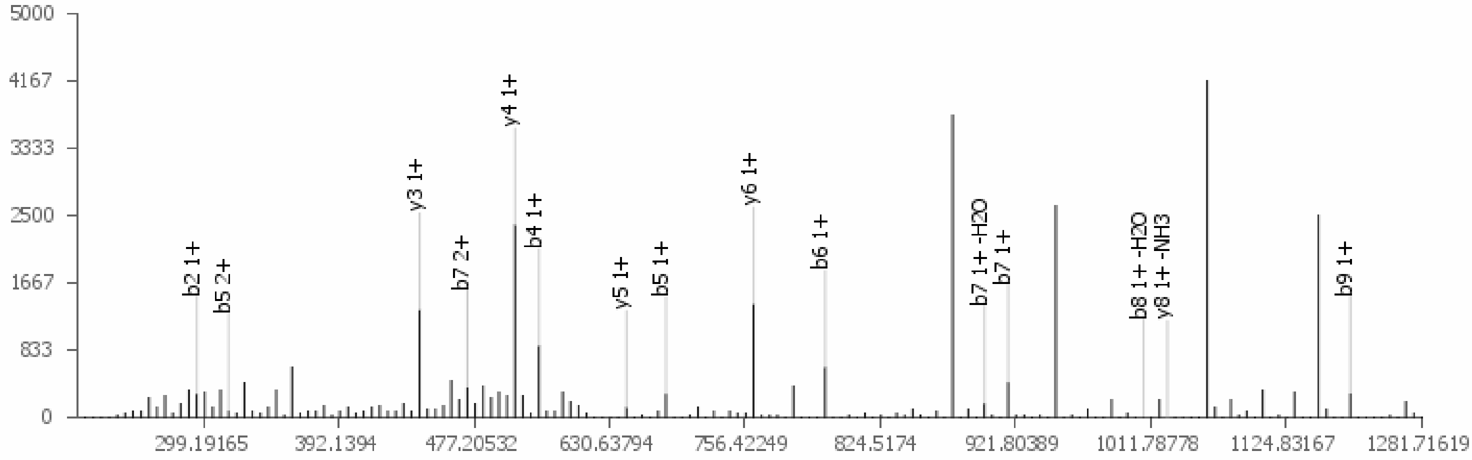

AT1G51538.1 - EDDV(s)(s)MDHK - 621.723697 - Charge:2

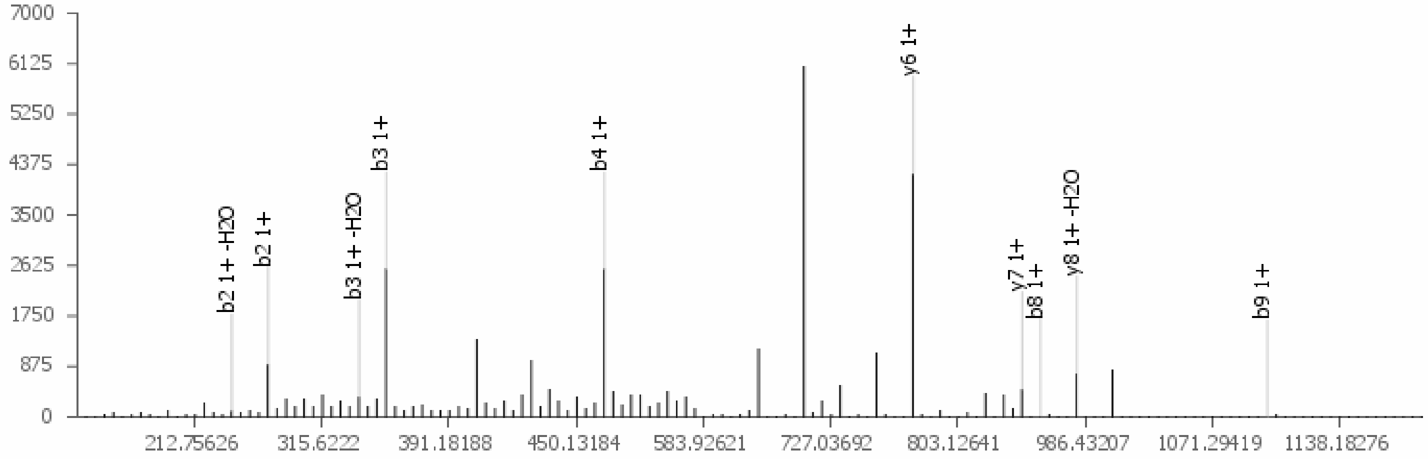

AT5G10550.1 - L(pT)FTNAM(pS)YNPK - 773.808851 - Charge:2

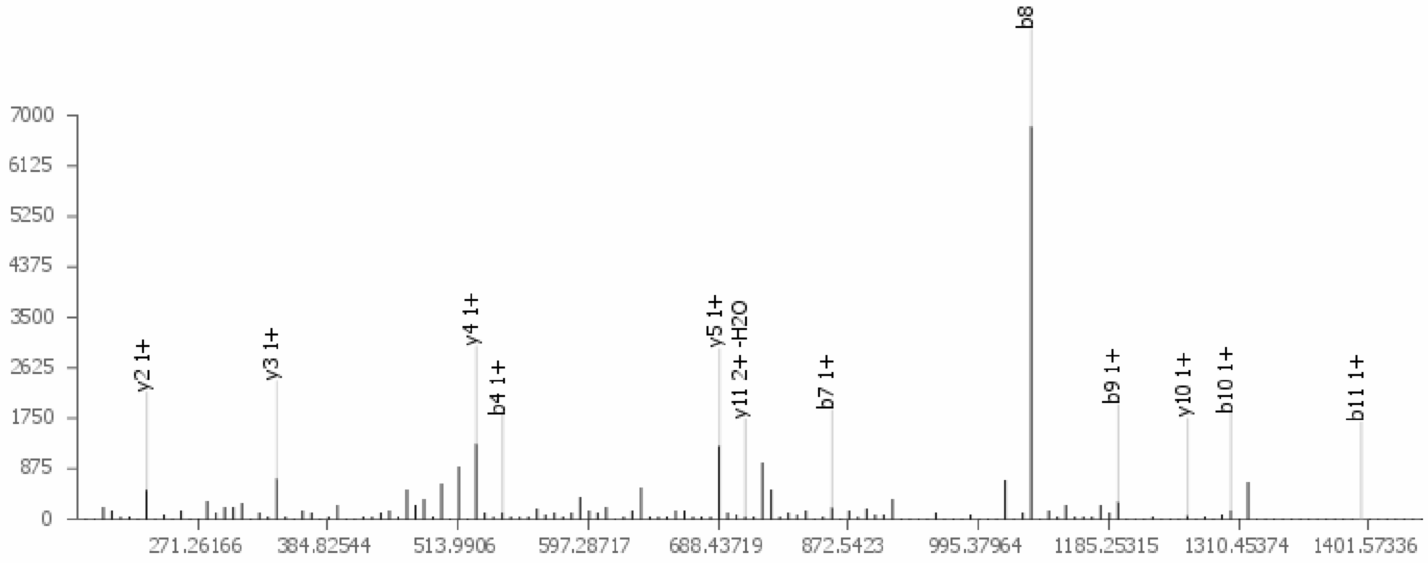

AT1G70320.1 - ASDNSVSASS(s)(t)AER - 774.811743 - Charge:2

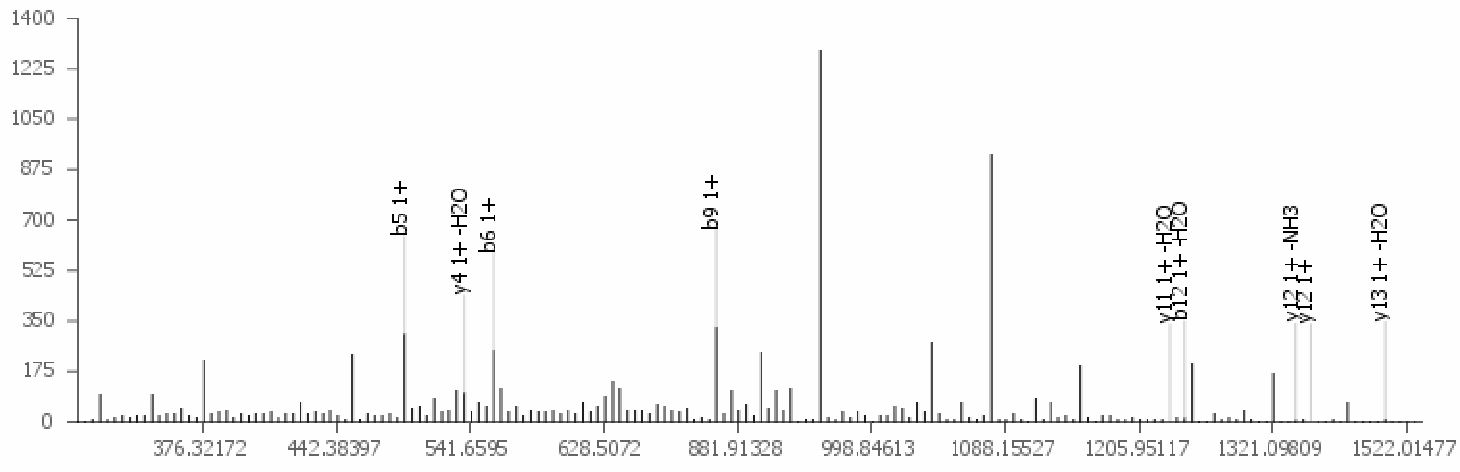

AT1G08125.1 - DEVVID(pT)DE(pT)K - 712.274442 - Charge:2

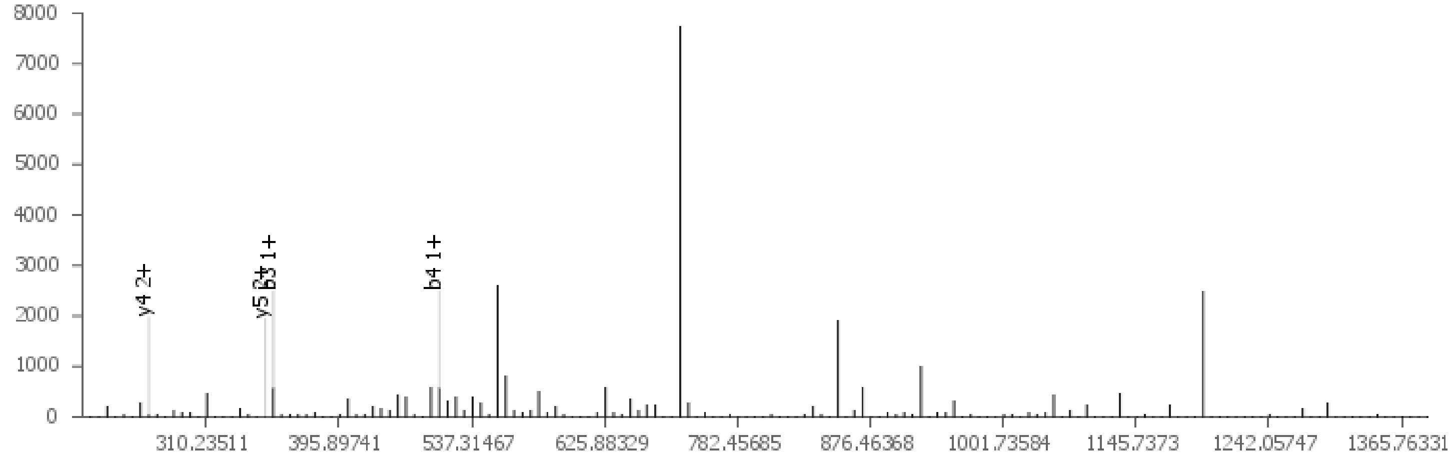

AT2G43795.1 - NY(pS)SDISR - 511.207496 - Charge:2

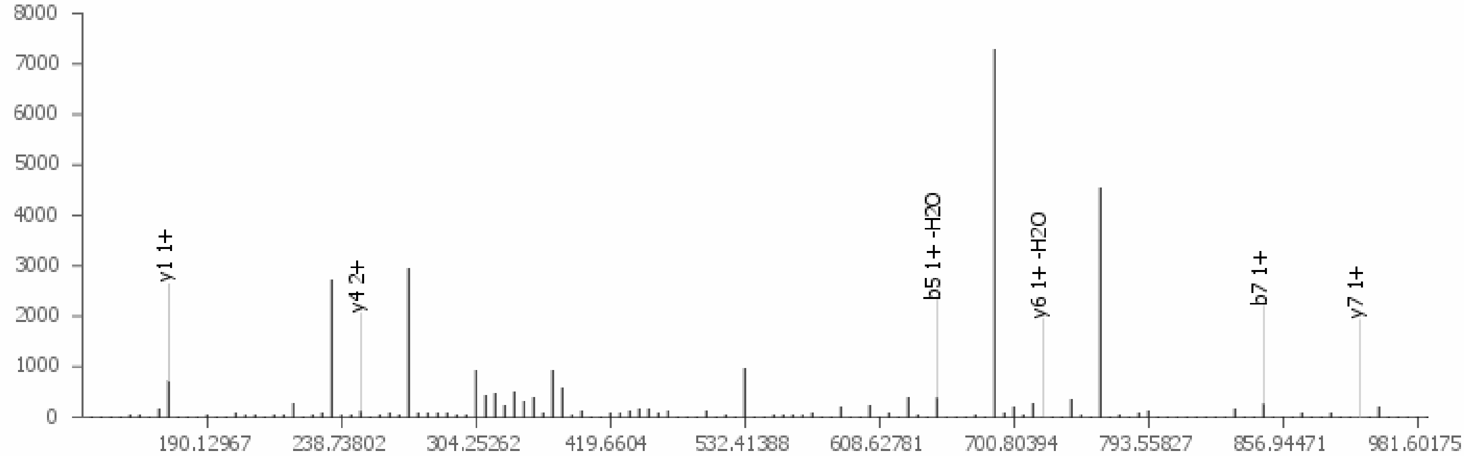

AT5G48545.1 - EASQLV(pS)CVR - 614.778718 - Charge:2

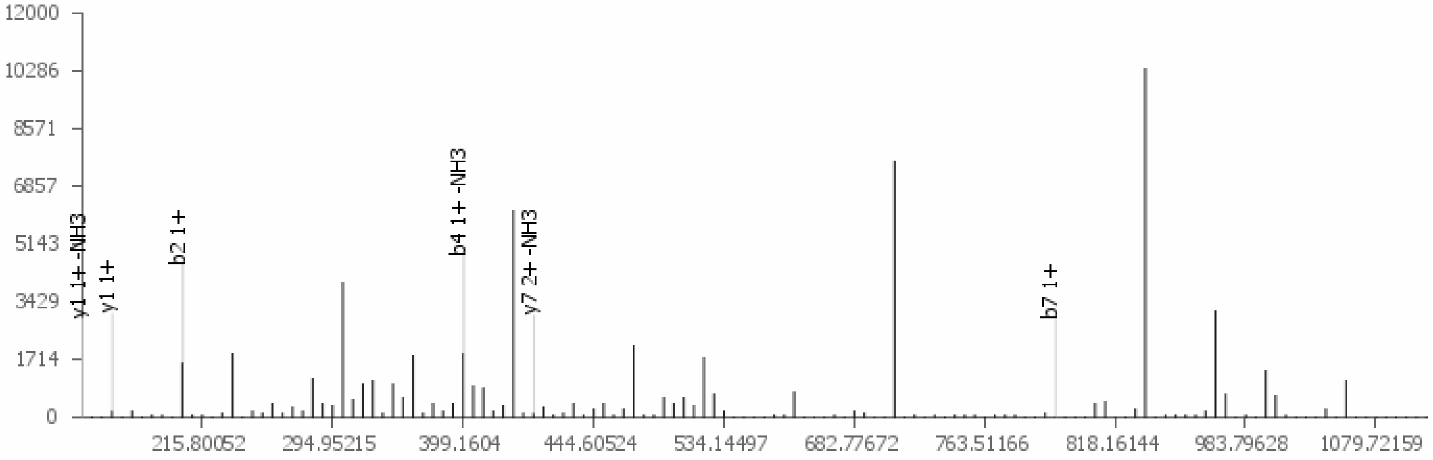

AT2G23570.1 - SFTGEG(pY)GSVTR - 670.772887 - Charge:2

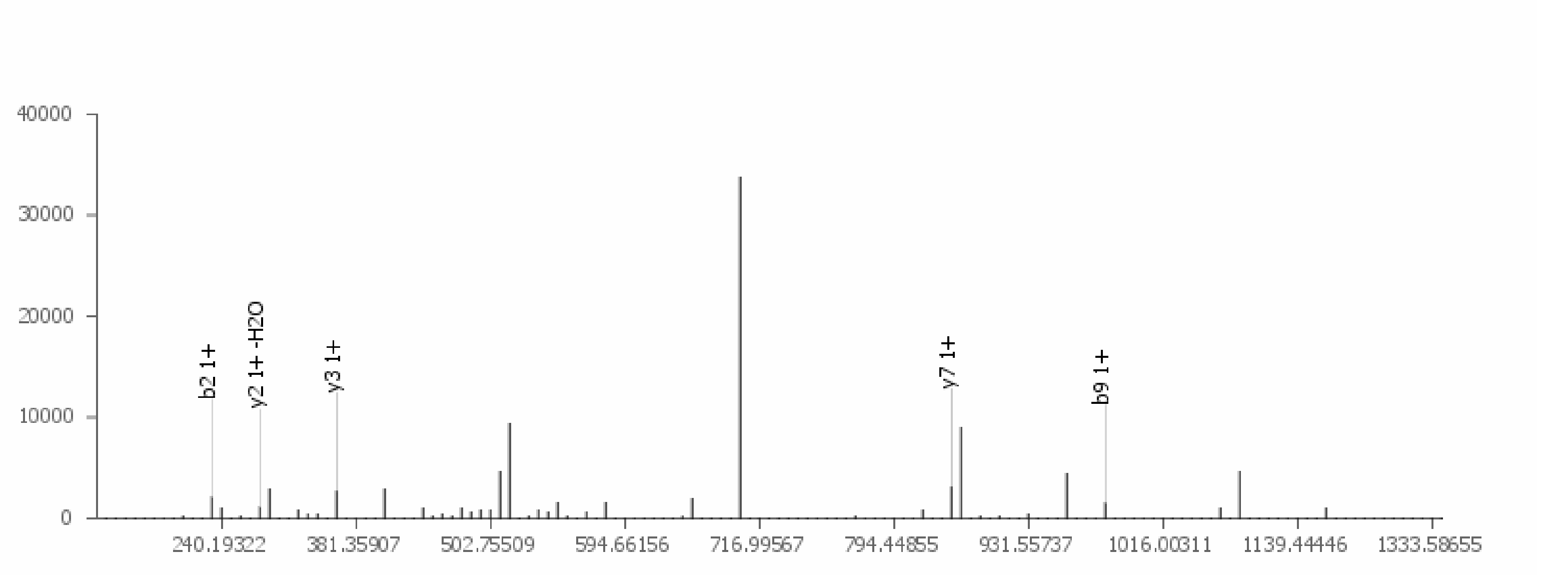

AT3G11490.1 - EE(pT)LDNFEAE(oxM)K - 776.297229 - Charge:2

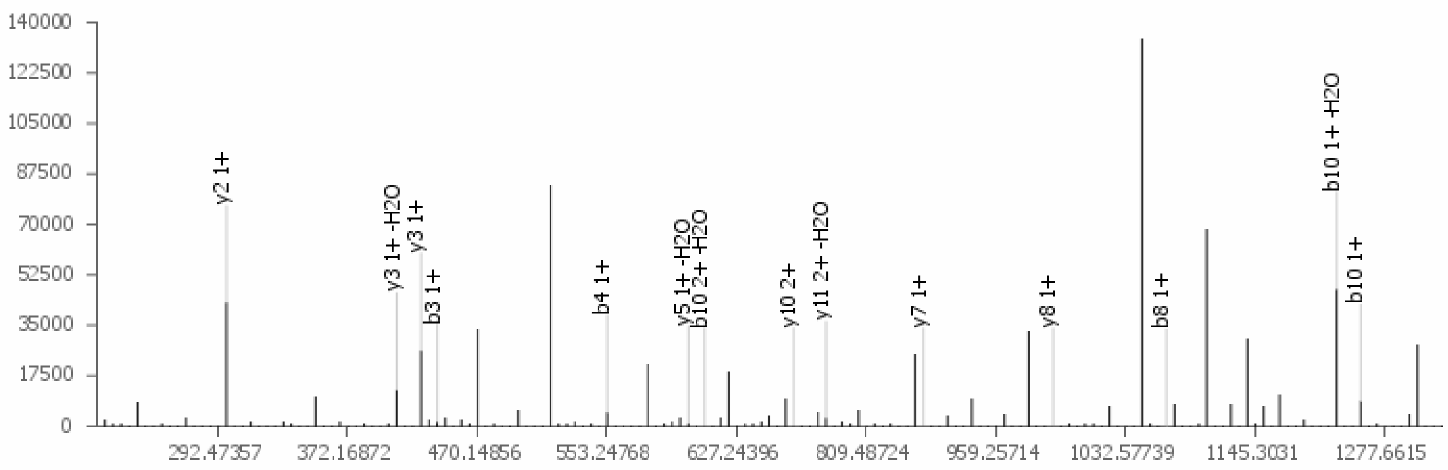

AT3G46530.1 - E(pT)YGIGGLK - 509.231345 - Charge:2

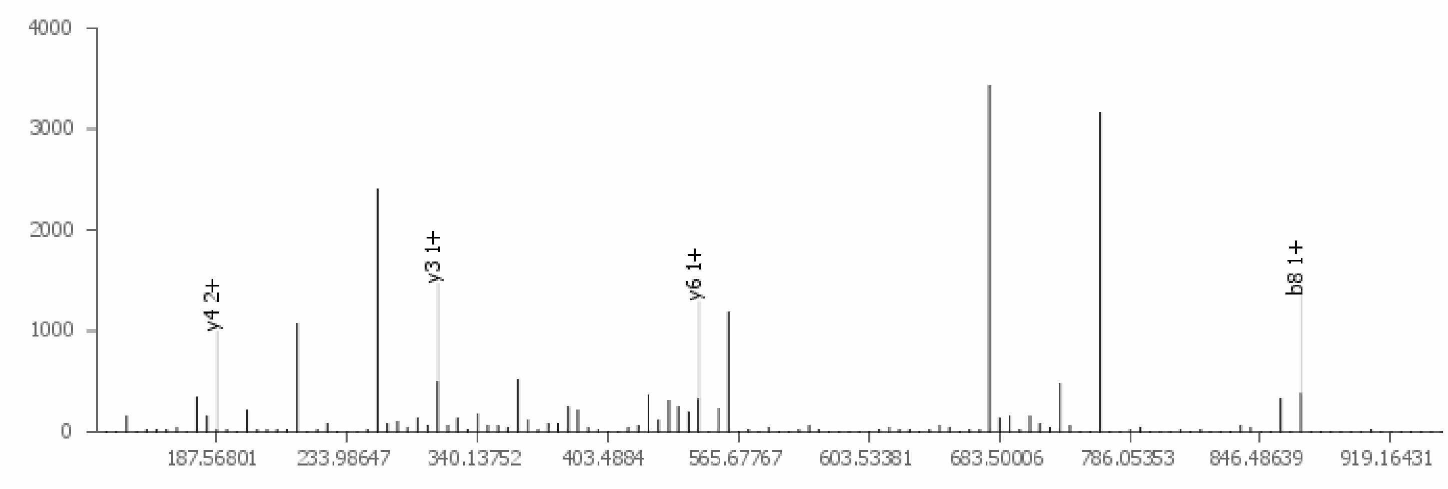

AT5G05610.1 - DGKPS(oxM)DLG(pS)K - 615.759623 - Charge:2

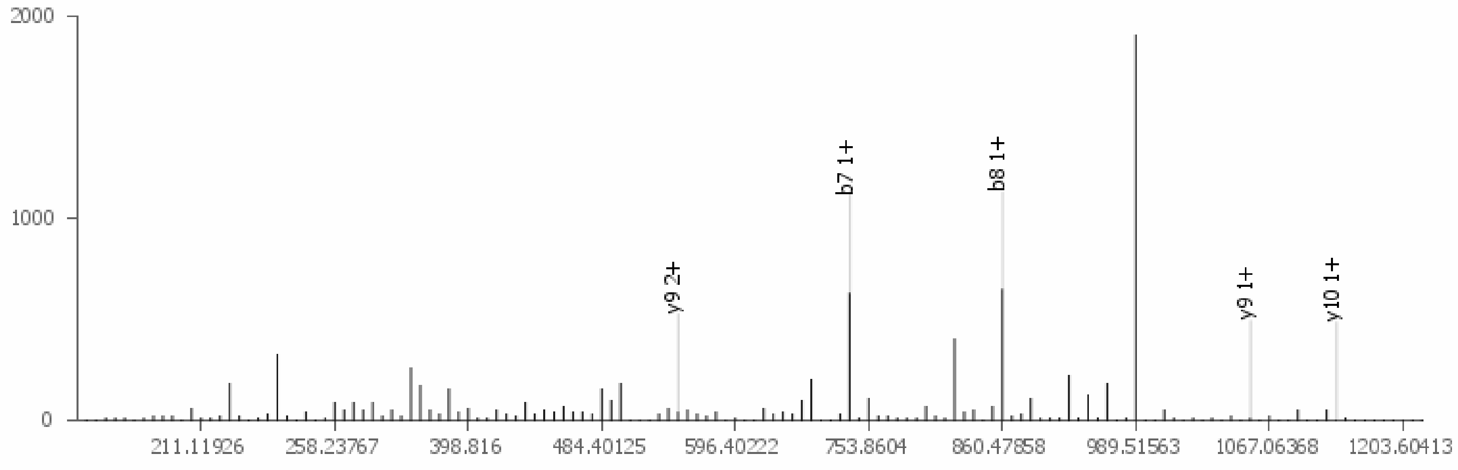

AT3G29770.1 - NQLPR(s)(s)(s)SR - 646.265309 - Charge:2

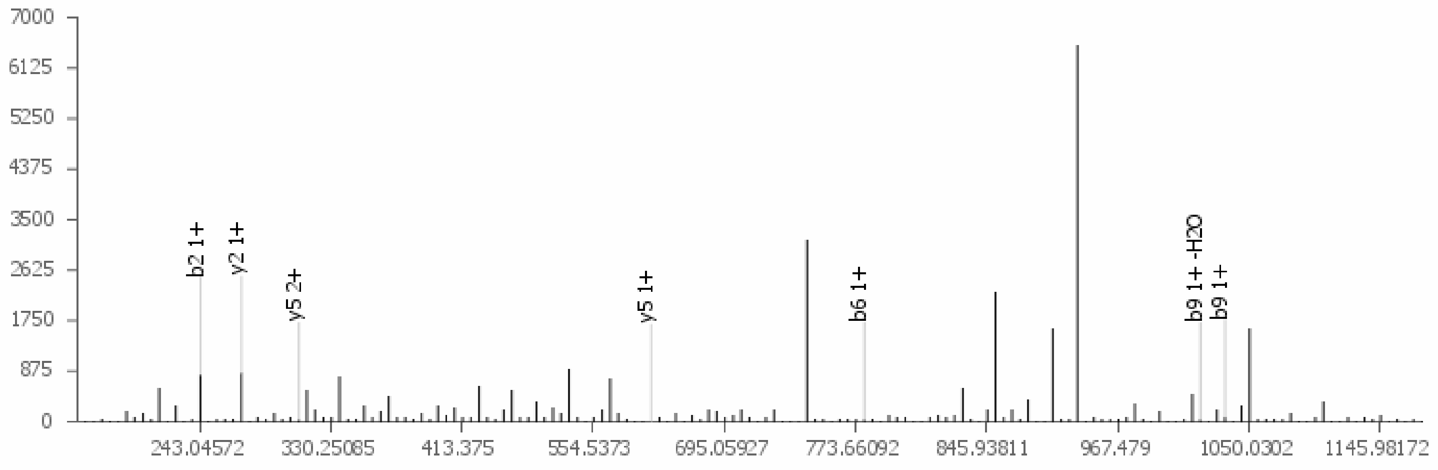

AT2G45460.1 - IS(pS)QNGASPSP(pS)LNSK - 867.357929 - Charge:2

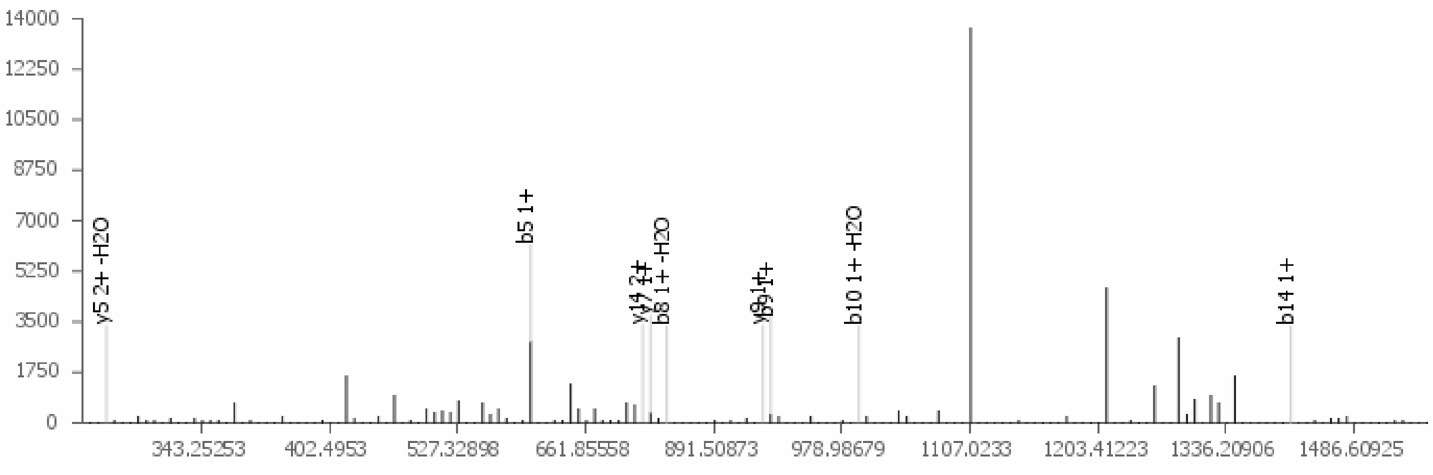

AT3G12930.1 - (pT)SSSLSCLSNR - 646.265215 - Charge:2

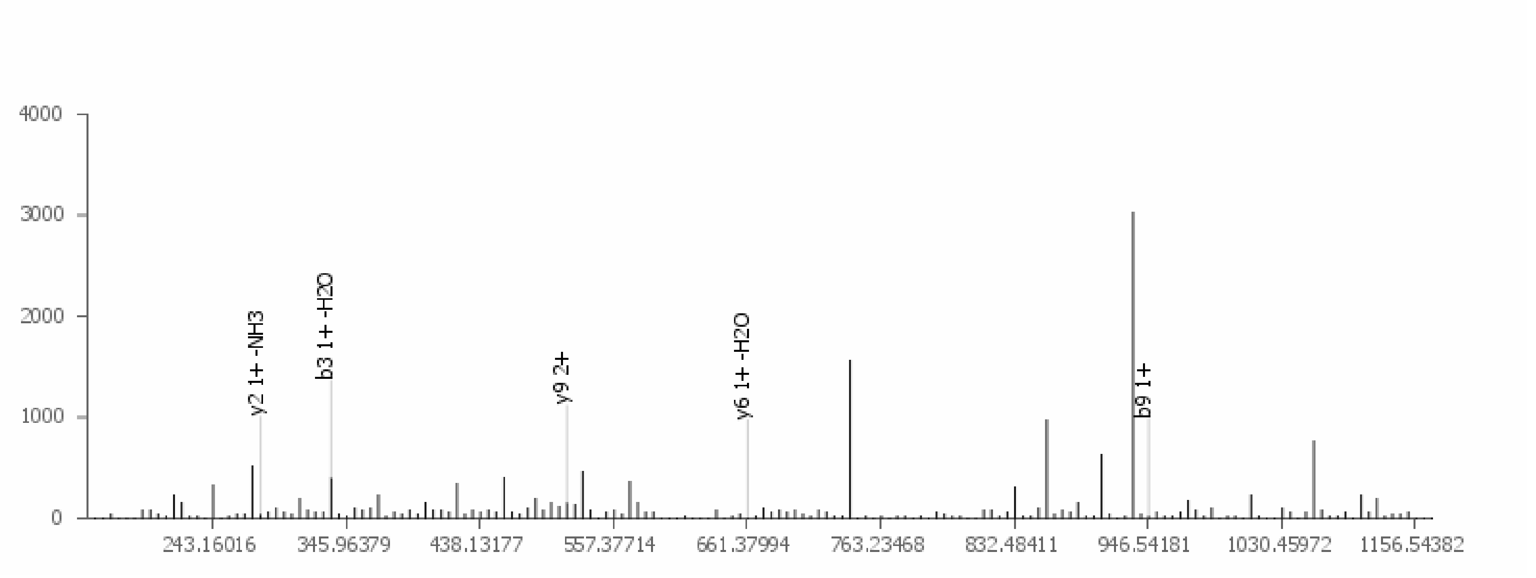

AT2G20650.1 - DCVICM(pT)(pT)IDLR - 828.822865 - Charge:2

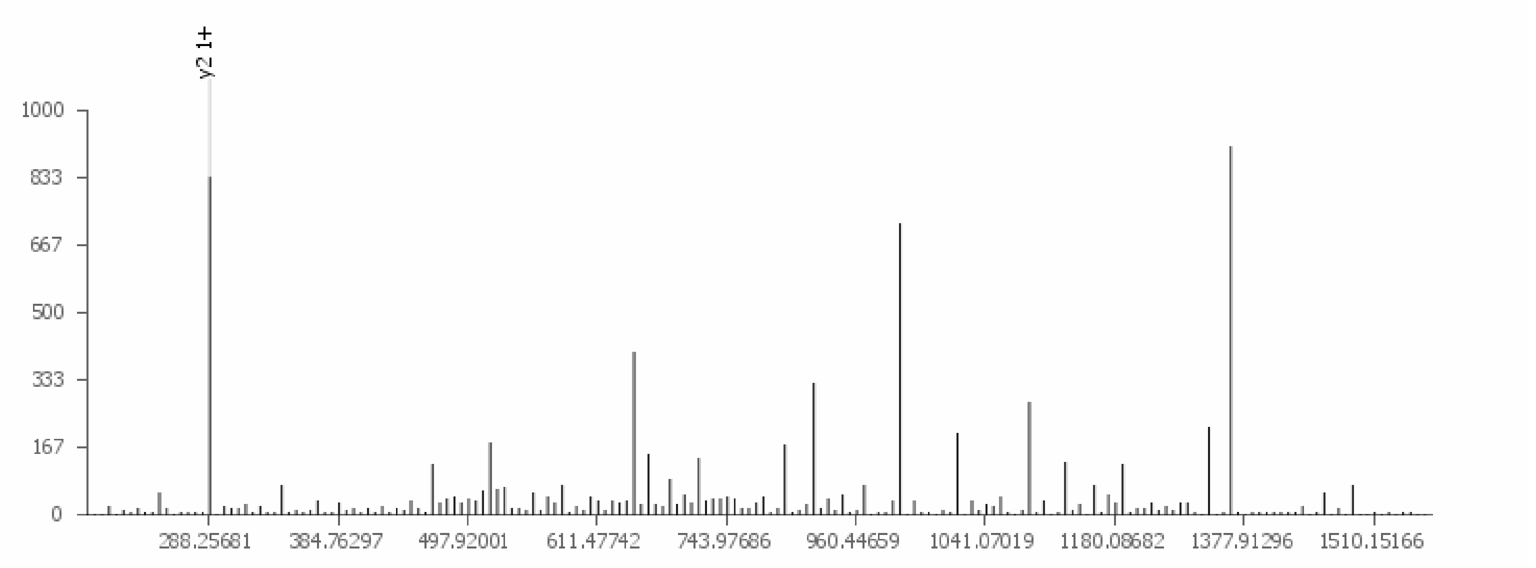

AT2G35140.1 - RAN(oxM)SN(pS)EDVAIPK - 814.361613 - Charge:2

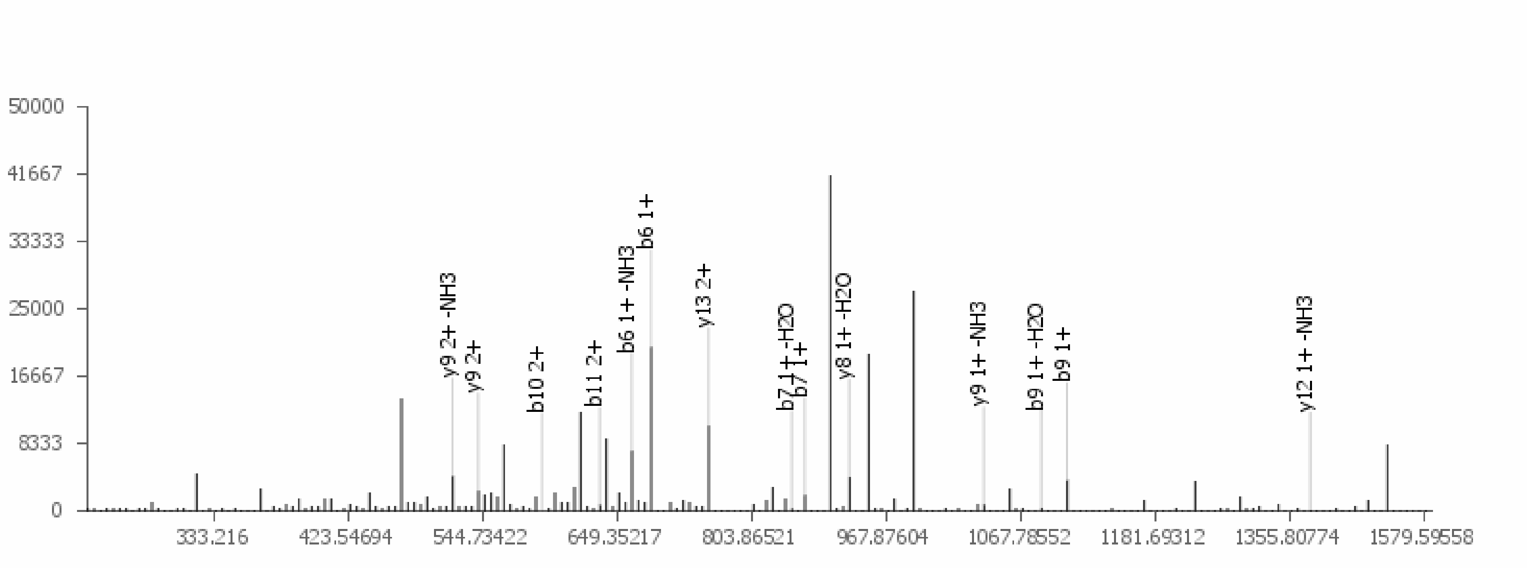

AT3G21990.1 - VSAMVQC(pT)K - 552.232265 - Charge:2

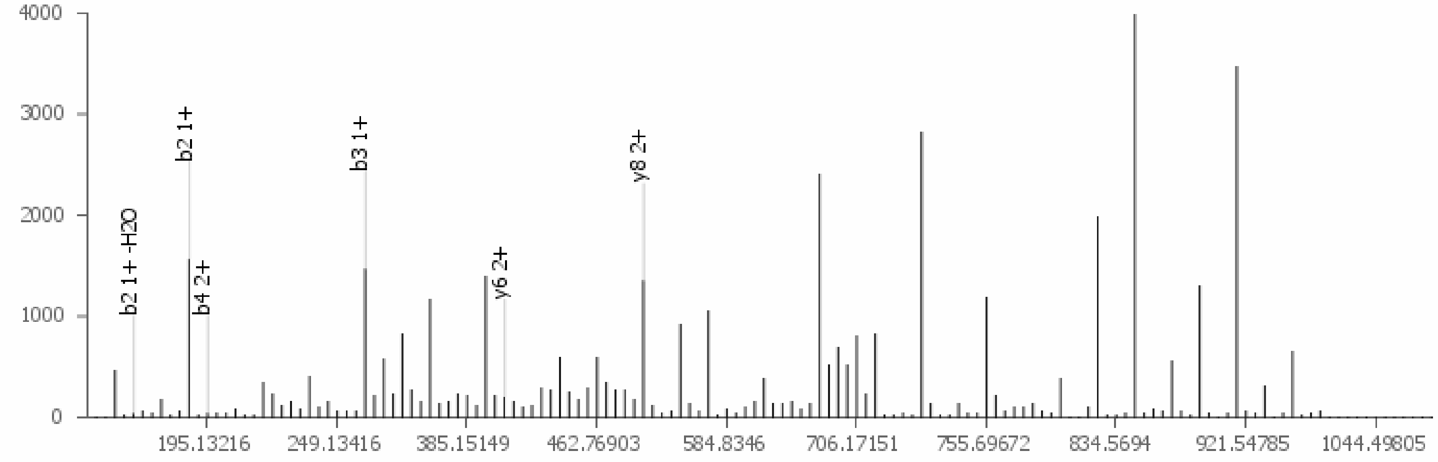

AT3G28600.1 - EKNLM(t)(s)R - 529.743837 - Charge:2

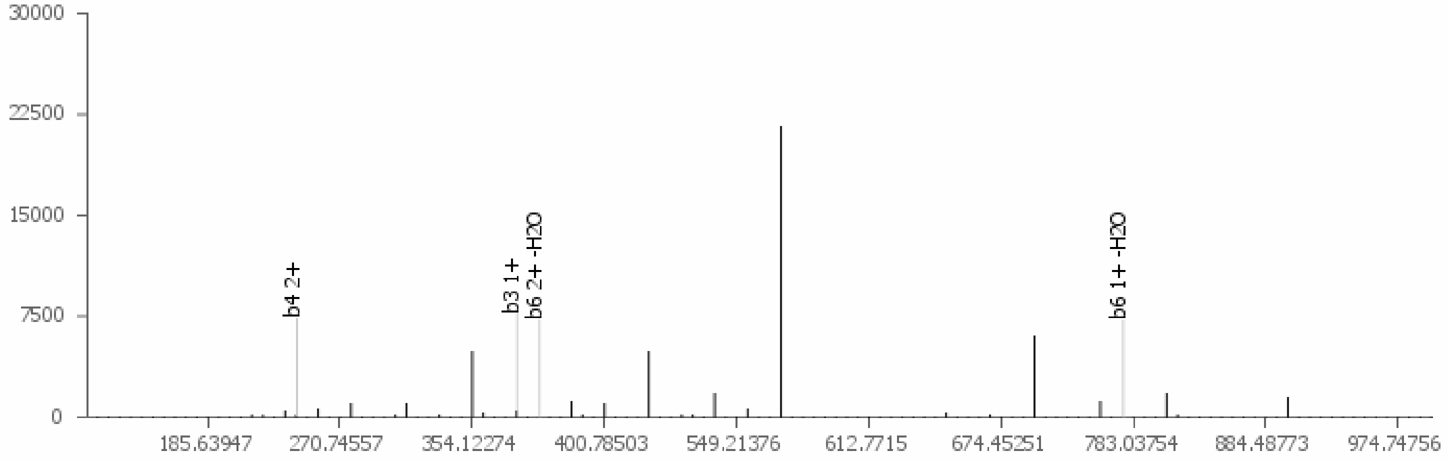

AT4G21065.1 - VSGLVEPD(pT)H(pT)(pY)PFLIK - 1078.453281 - Charge:2

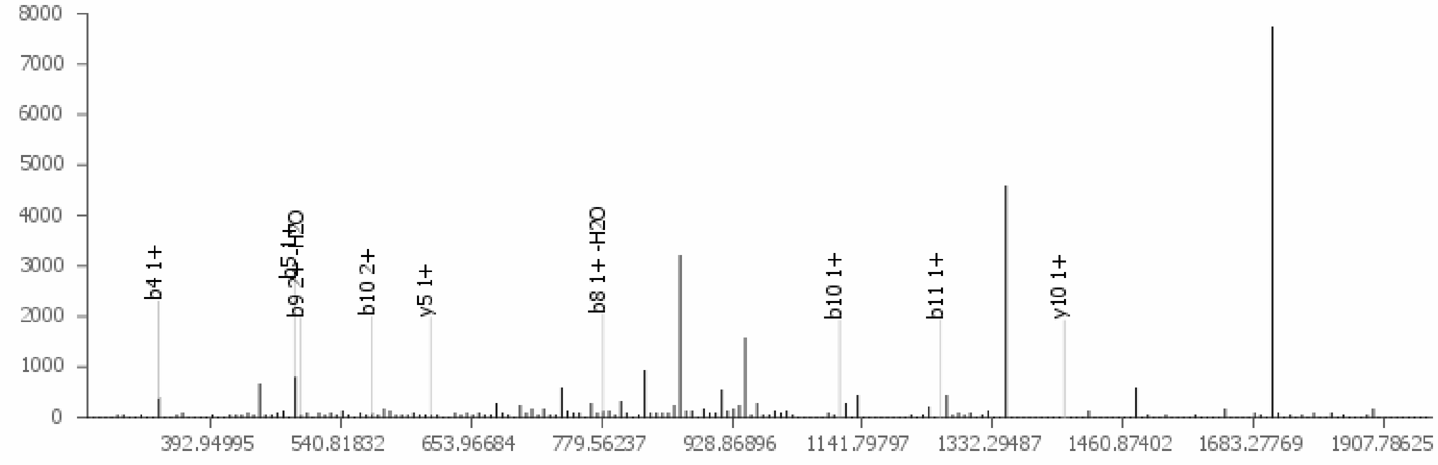

AT3G04650.1 - A(pT)(pS)LM(pS)L(pS)S(pS)FLQPLK - 1055.385125 - Charge:2

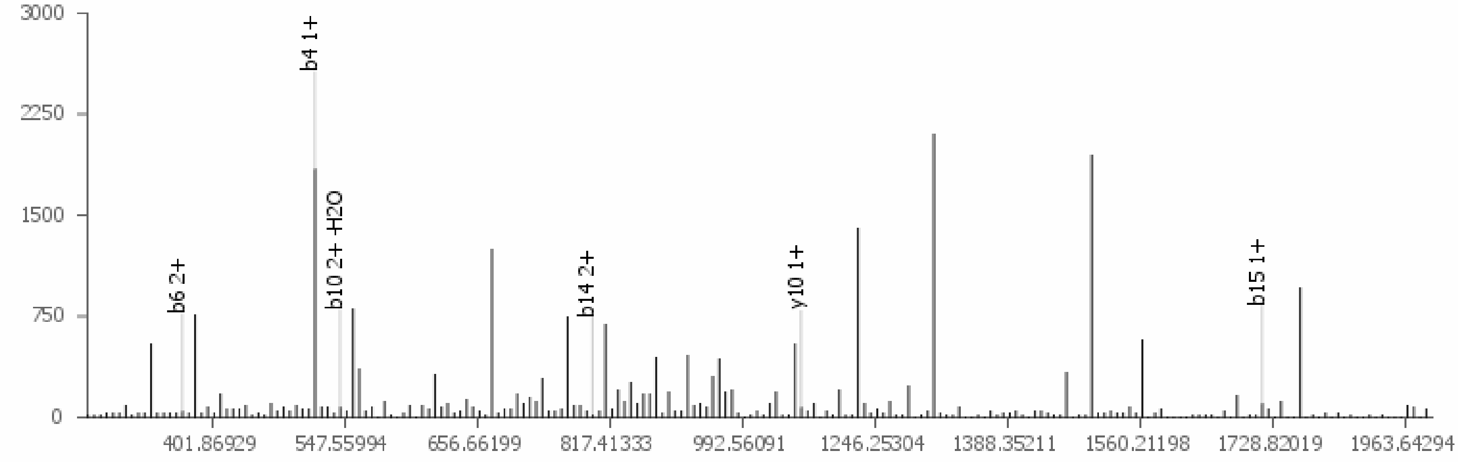

AT4G33240.1 - pS)P(pT)(pS)LAKILGIYQV(pS)(pS)K - 1096.433501 - Charge:2

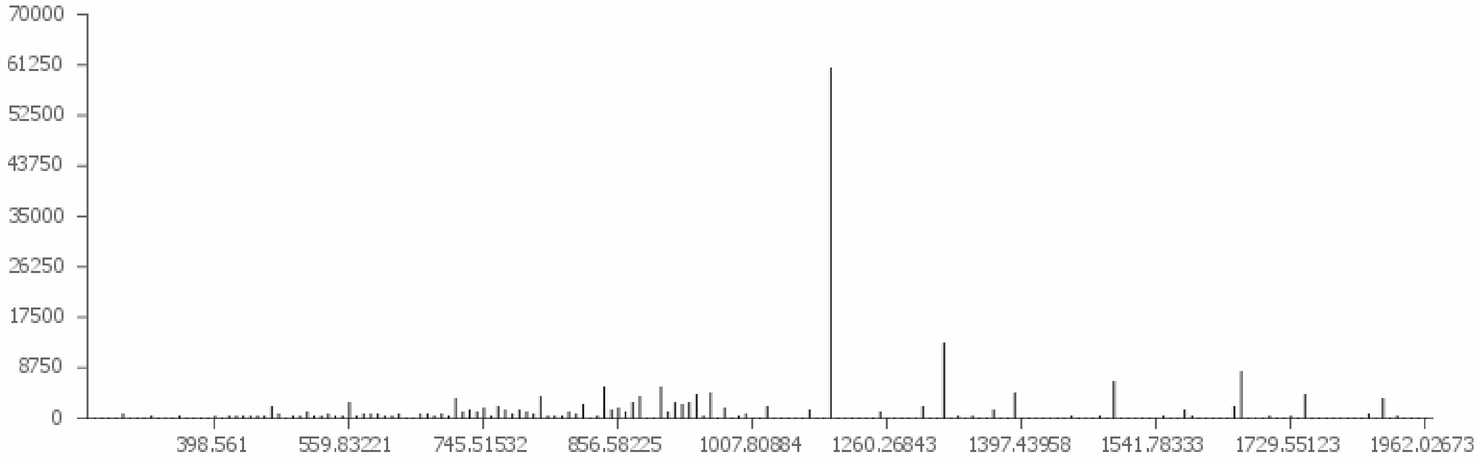

AT3G23400.1 - (pY)(pS)FSPLTTPK - 650.76767 - Charge:2

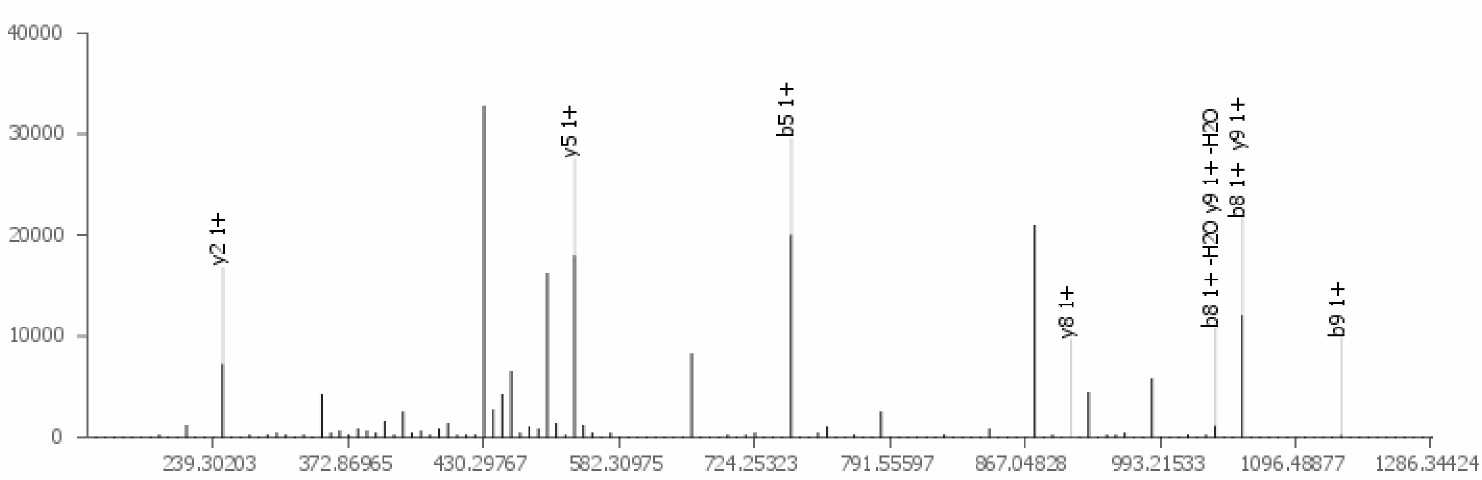

AT2G47470.1 - IEEEA(pS)(pT)LK - 590.235346 - Charge:2

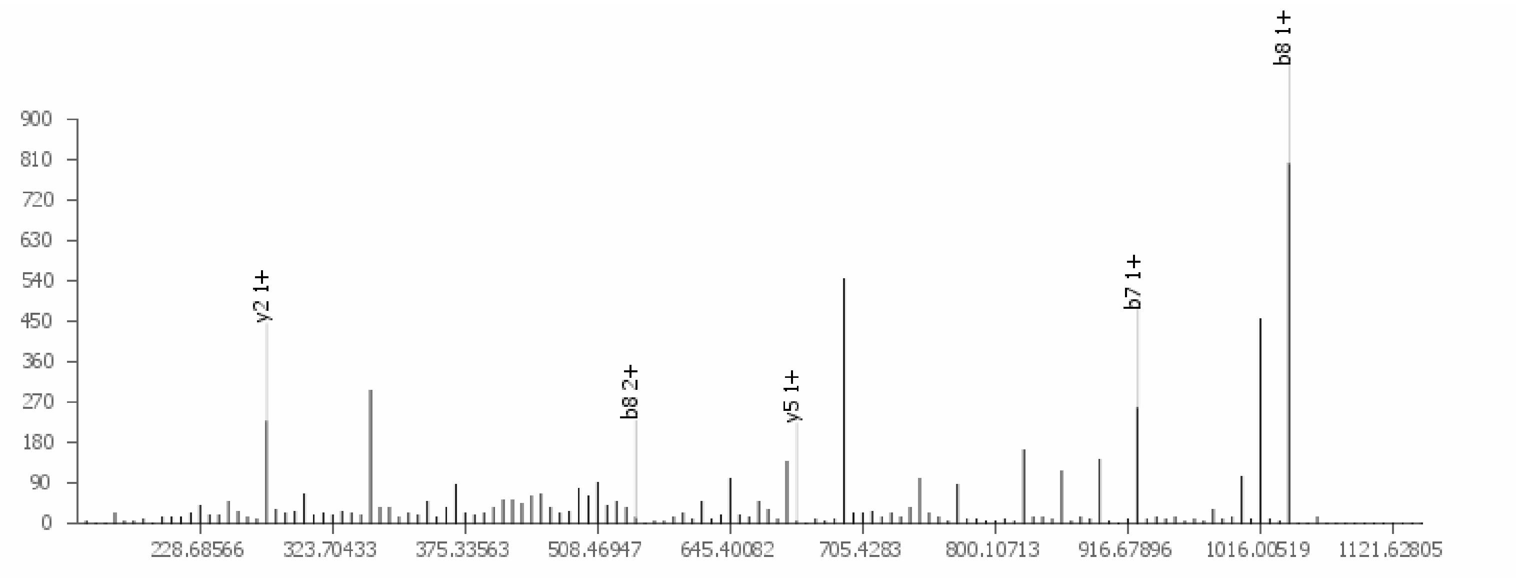

AT2G38440.1 - NSDLPSETSSISS(pT)(pS)EG(pS)R - 1090.897988 - Charge:2

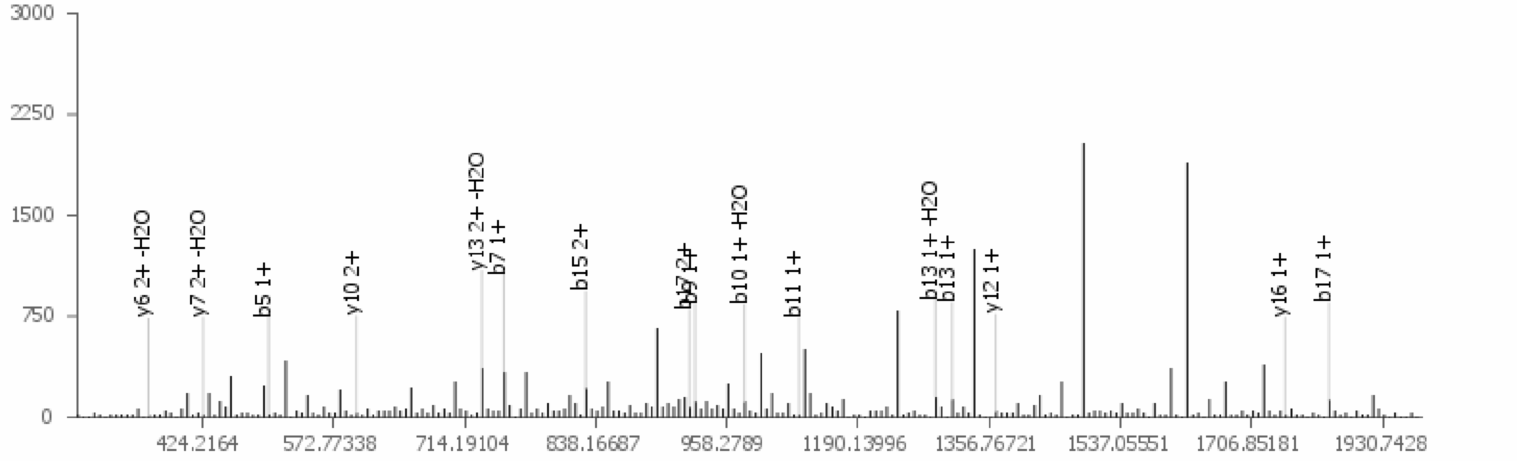

AT2G30505.1 - (pS)G(pS)FVDHIGQEDK - 789.793021 - Charge:2

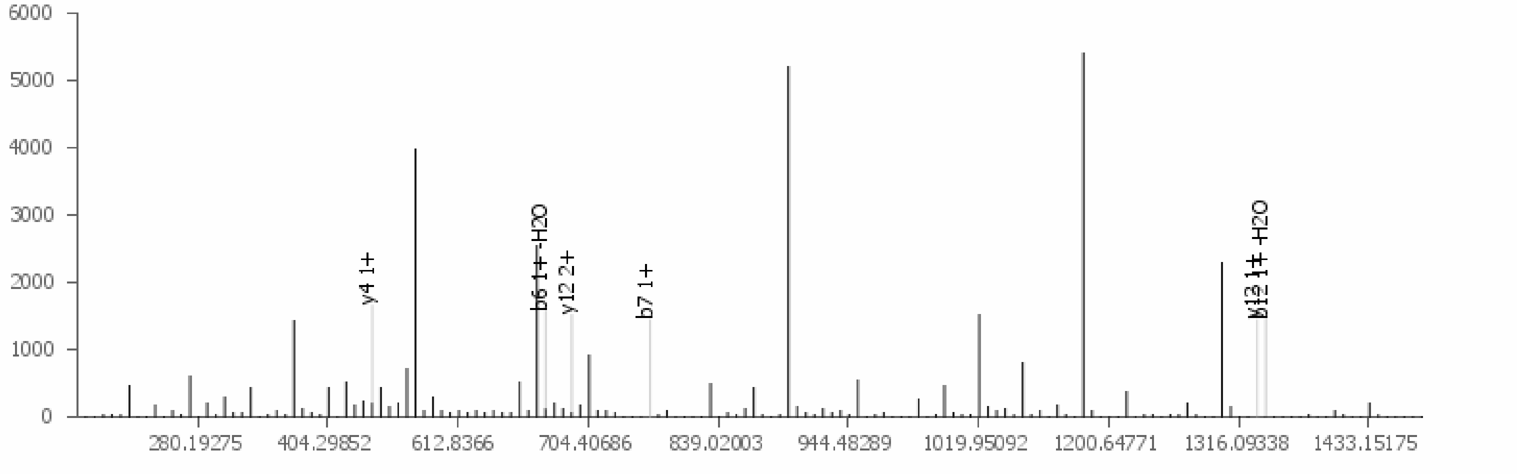

AT3G18650.1 - AS(pS)SSSSLSF(s)(t)(s)K - 761.79237 - Charge:2

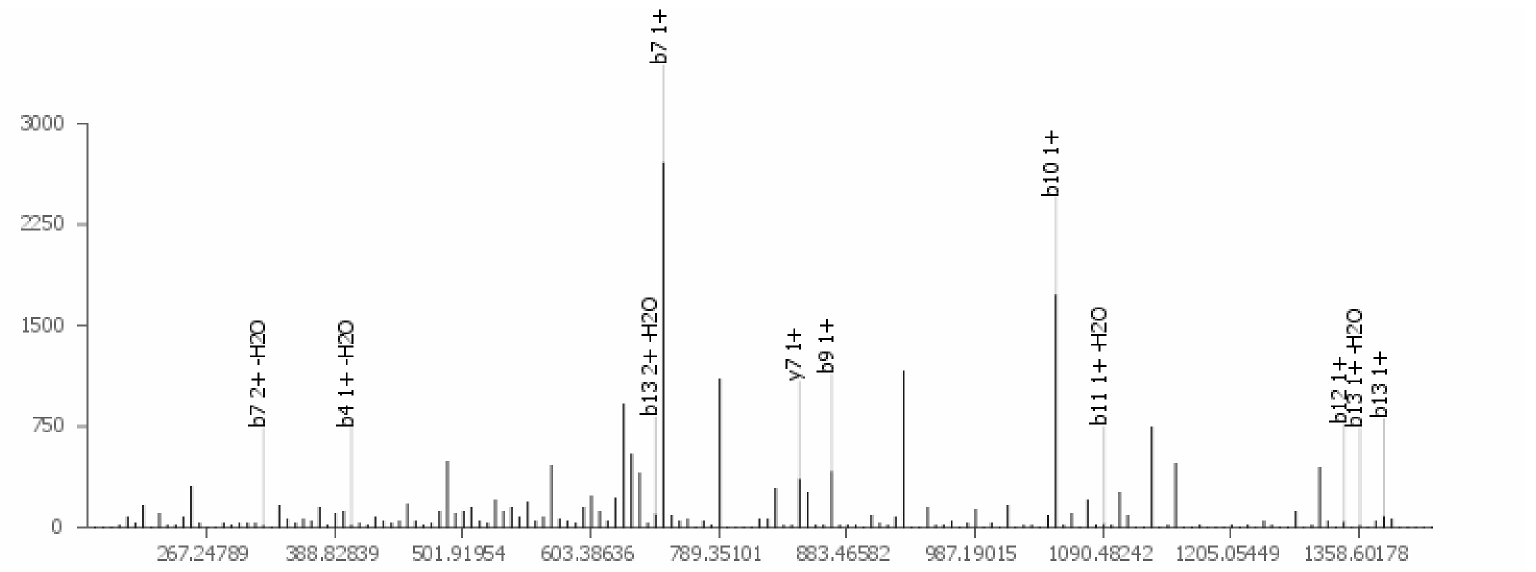

AT2G41990.1 - HAK(pT)D(pS)EATSIDAAALSPPR - 1099.484838 - Charge:2

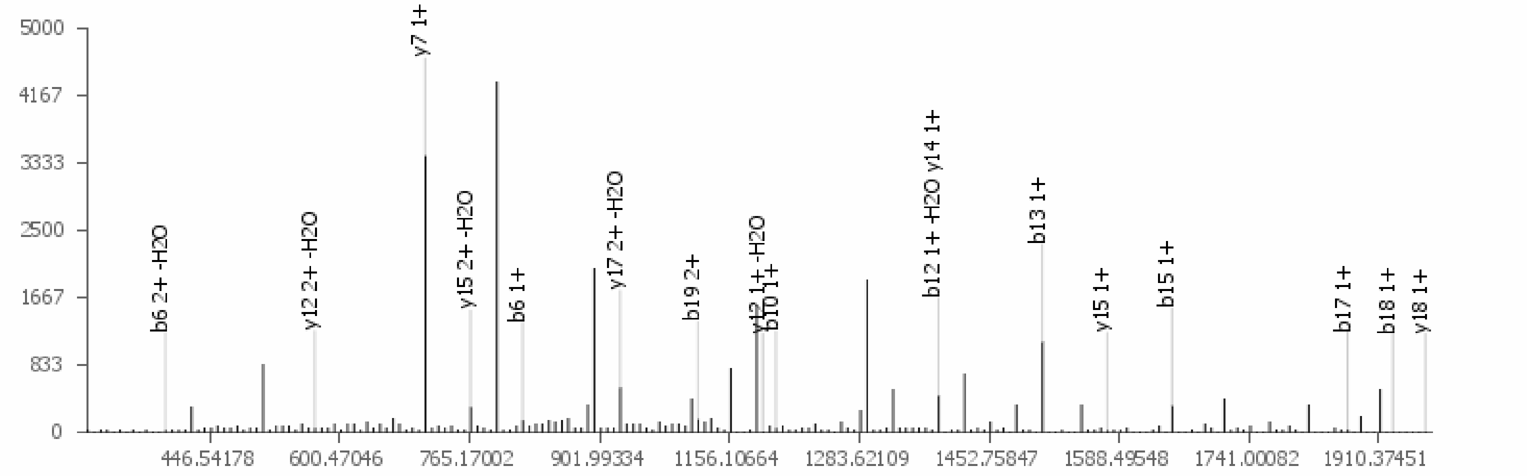

AT5G65460.1 - ILAD(pS)LGG(s)(s)K - 604.249779 - Charge:2

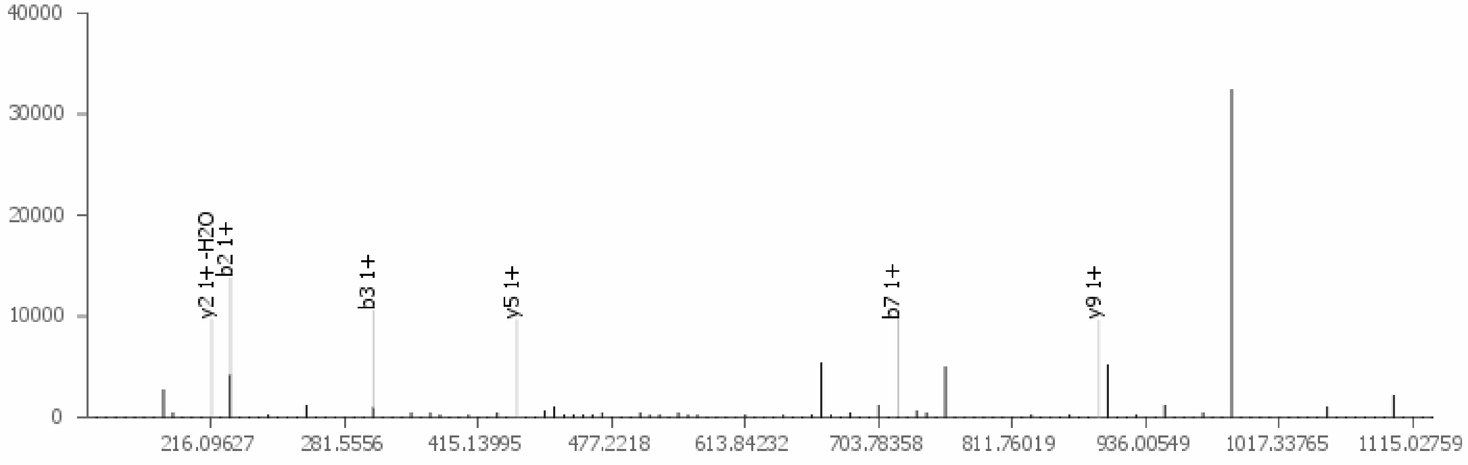

AT2G01680.1 - E(pT)AMDLADK - 537.219967 - Charge:2

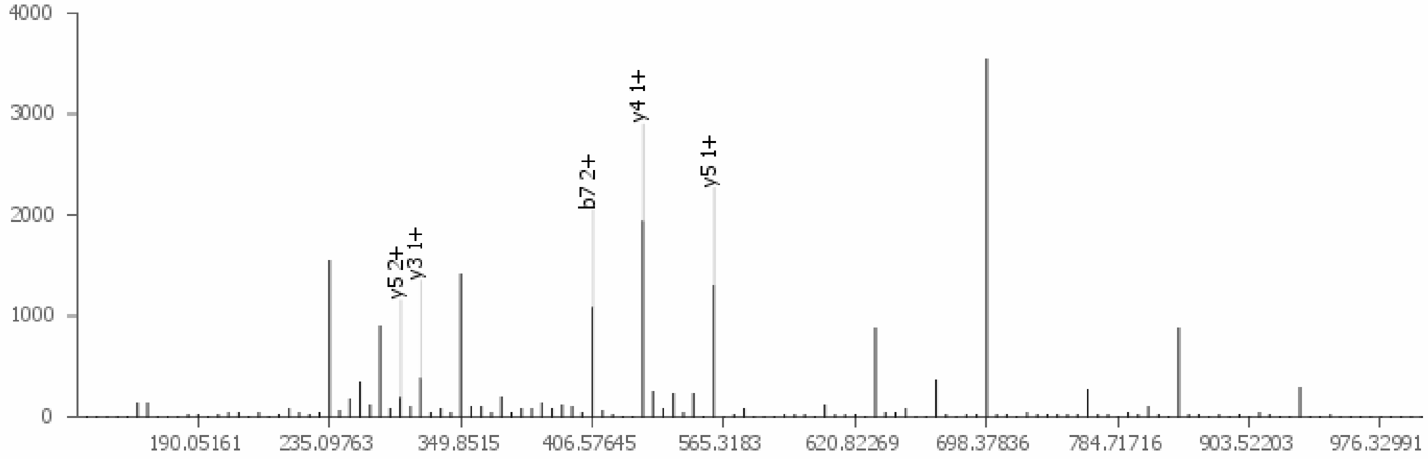

AT1G49700.1 - M(pT)YMYDHDHIPK - 815.821264 - Charge:2

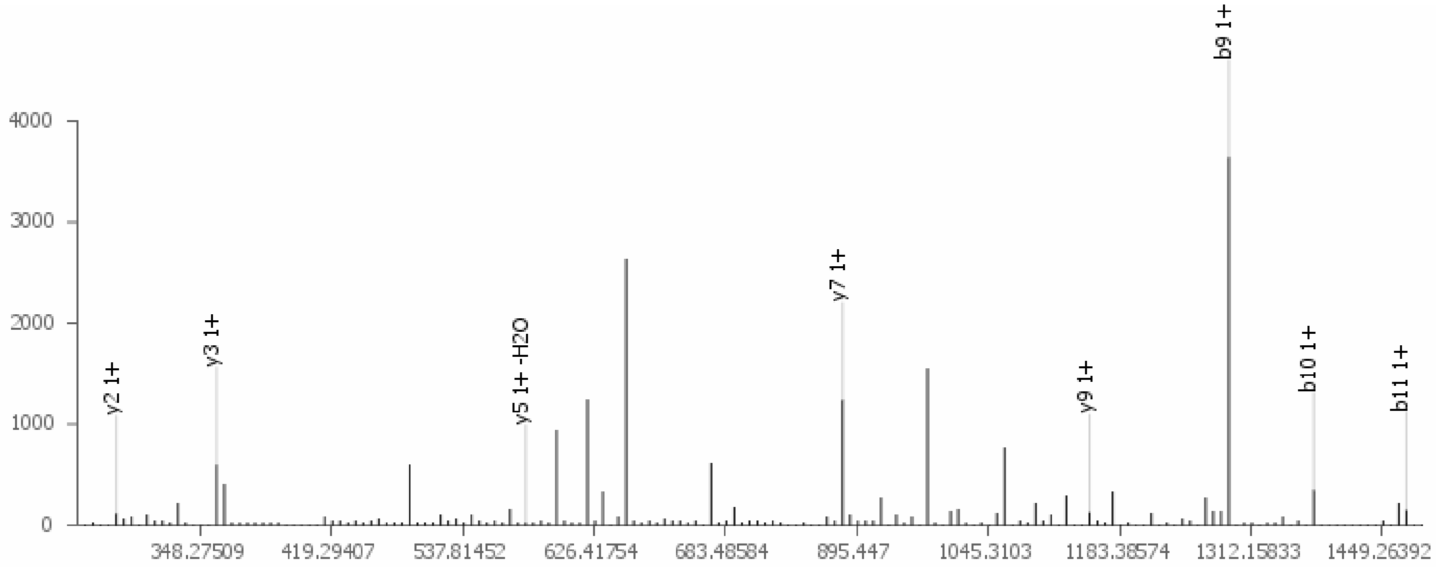

AT3G10310.1 - AYHECKLP(pS)NGNGLYK - 965.921158 - Charge:2

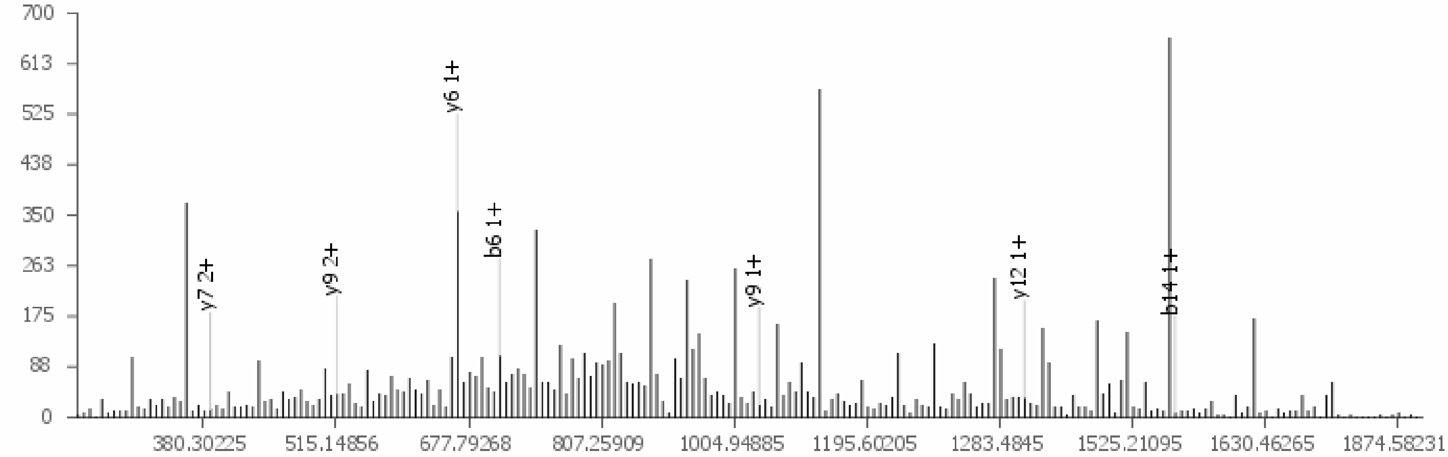

AT5G11400.1 - GYIDET(pT)FAPSR - 718.804574 - Charge:2

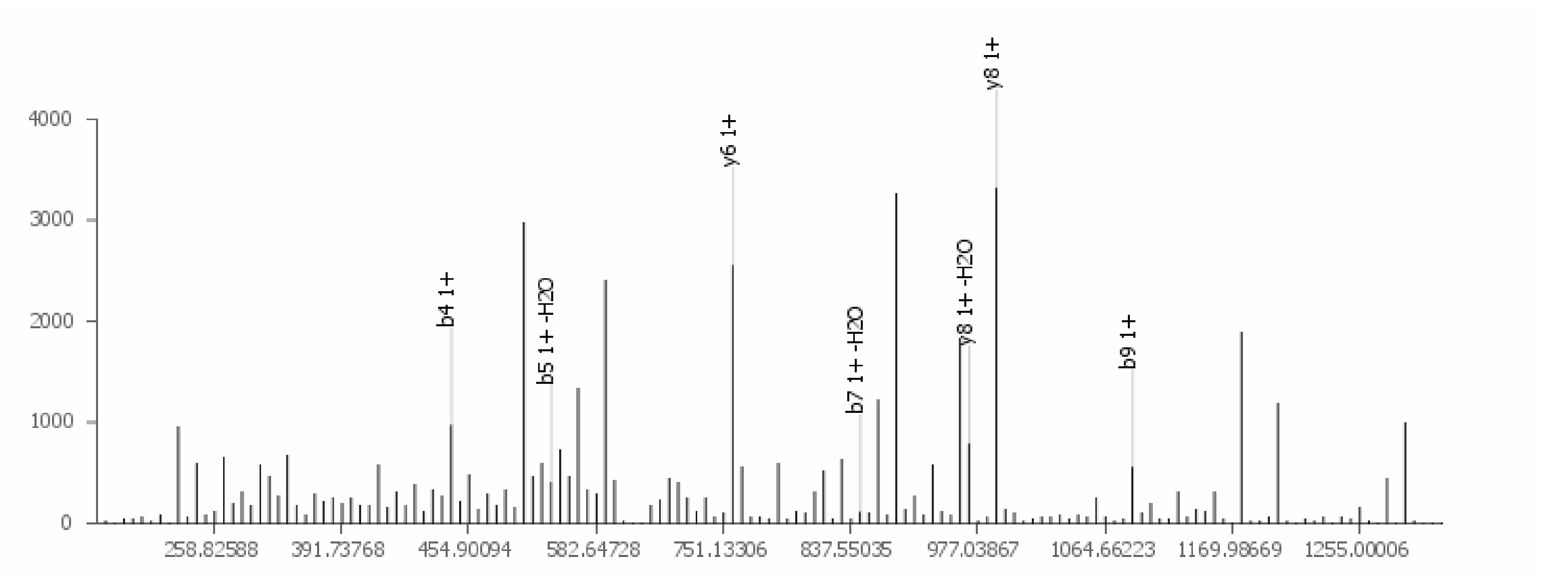

AT3G66652.1 - IQDGW(pS)(pS)P(pS)LSLR - 843.322631 - Charge:2

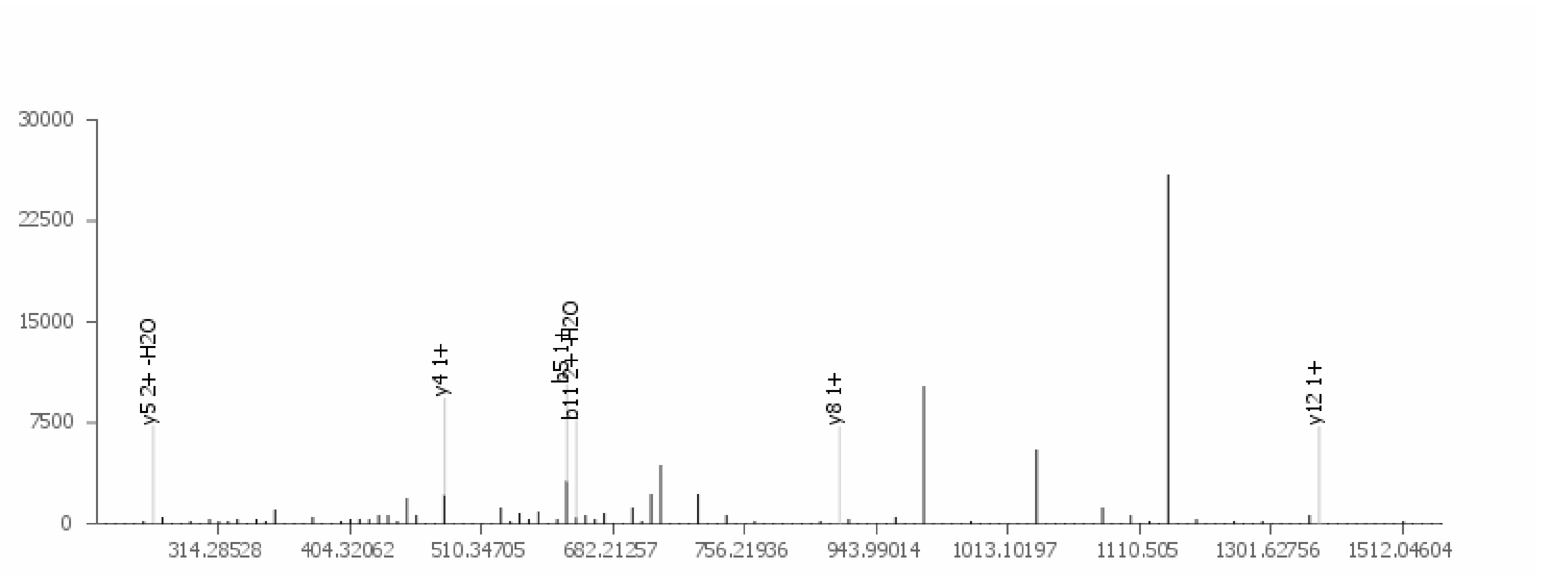

AT3G20750.1 - C(pT)NMNCNALNTP(oxM)WRR - 1067.917641 - Charge:2

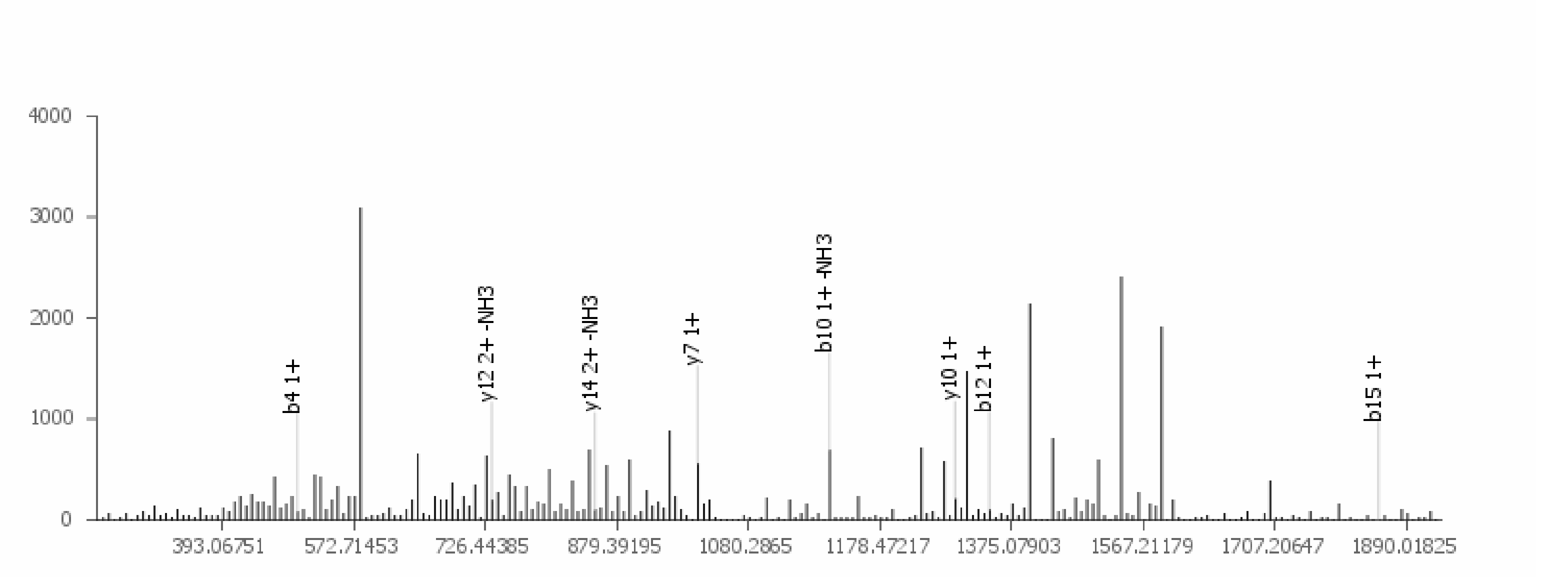

AT4G29540.1 - LSSSL(s)(pY)(s)REDSR - 823.822327 - Charge:2

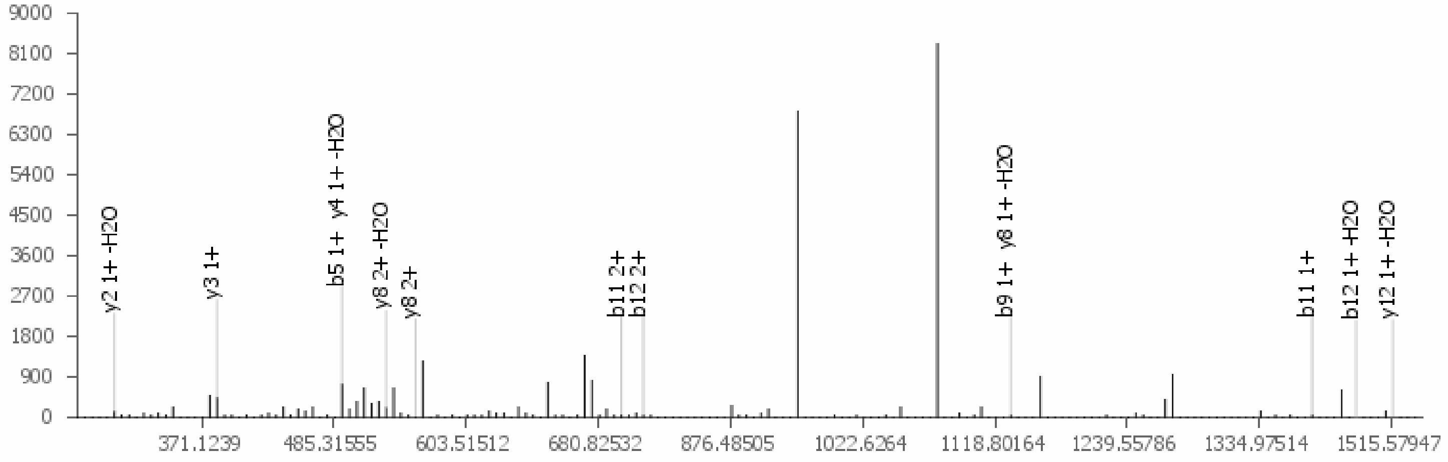

AT2G44180.1 - (pT)VLQ(pY)DDVMK - 686.265139 - Charge:2

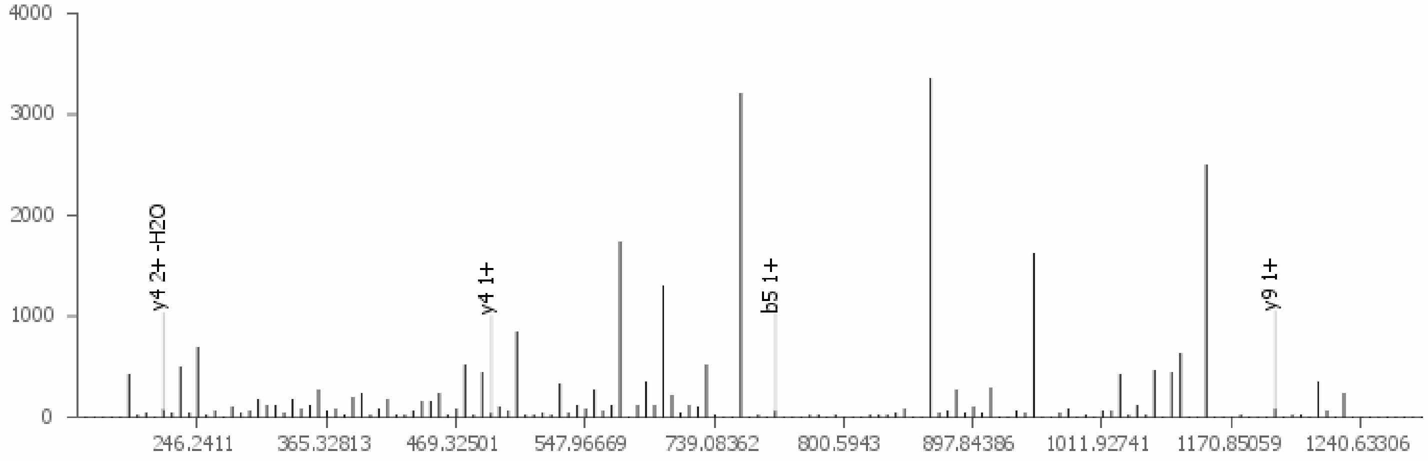

AT5G09930.1 - QSNNGA(s)(s)I(s)(s)GVR - 722.313336 - Charge:2

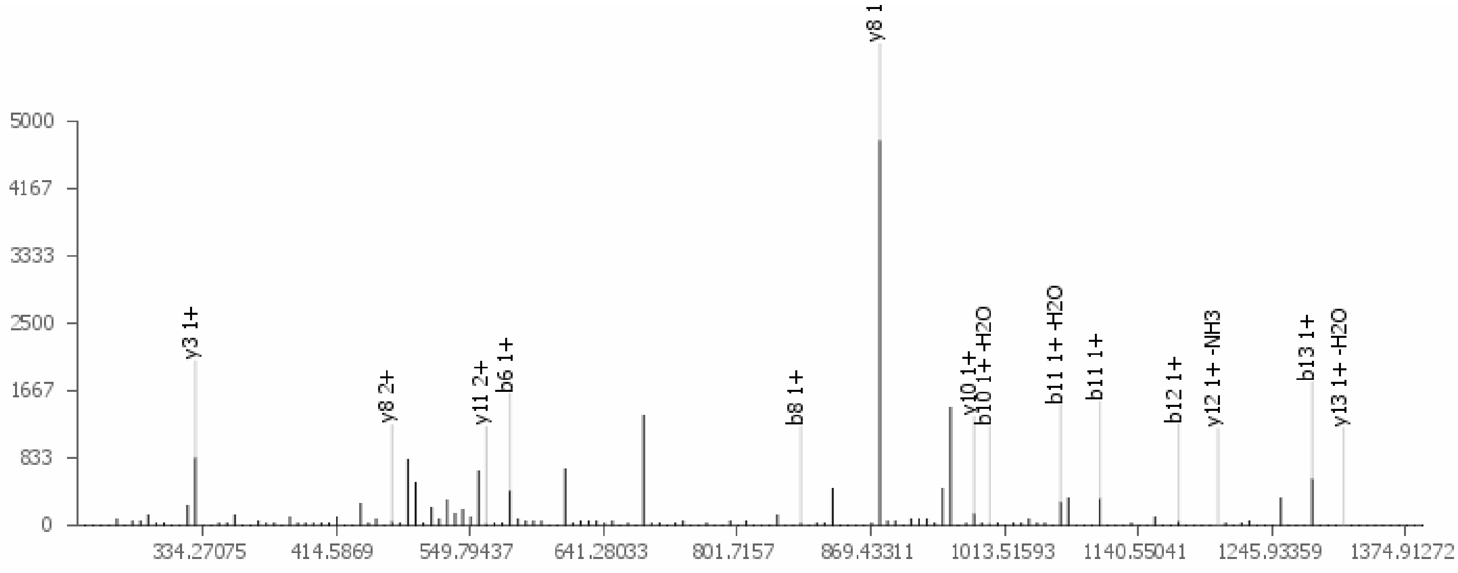

AT1G53470.1 - (pT)KTLMK - 401.203846 - Charge:2

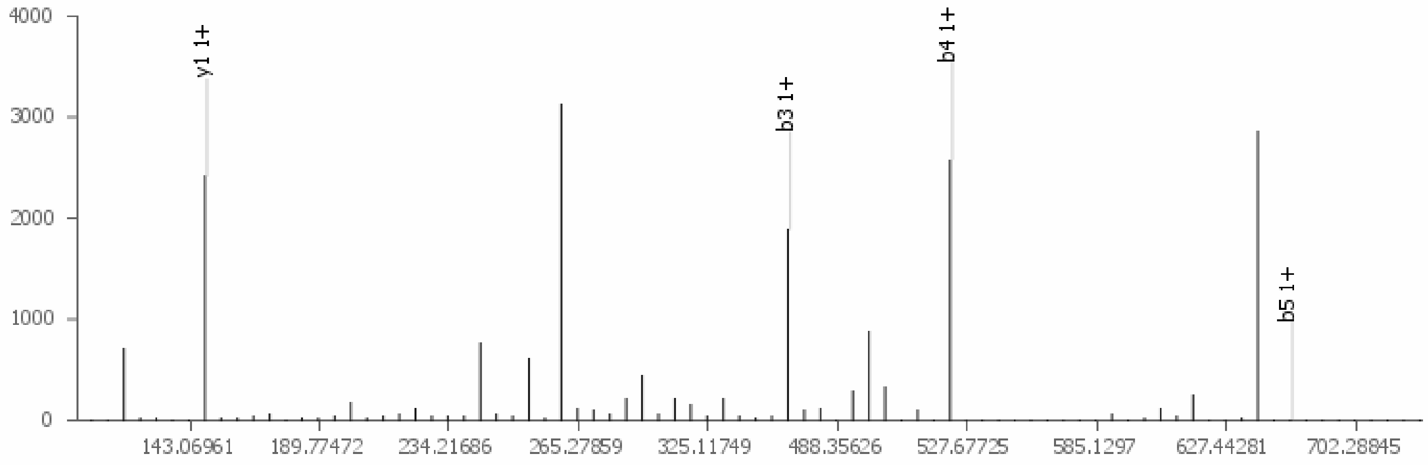

AT4G24770.1 - GFGFVTMSSVDEAE(pT)AVEK - 1042.444401 - Charge:2

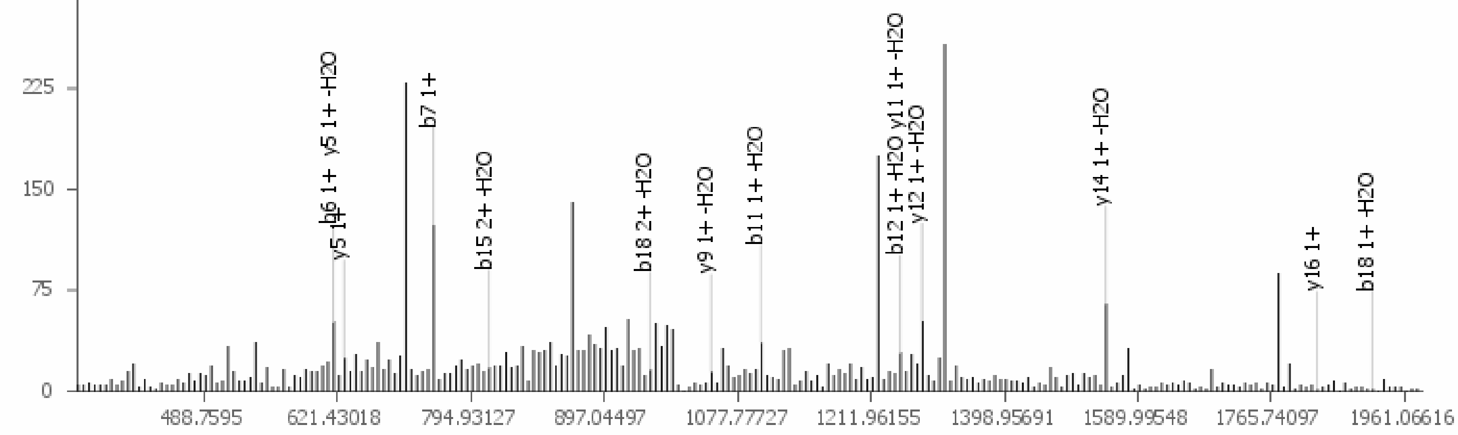

AT2G42810.1 - SHEVKDEG(pY)EVEHDGK - 969.396117 - Charge:2

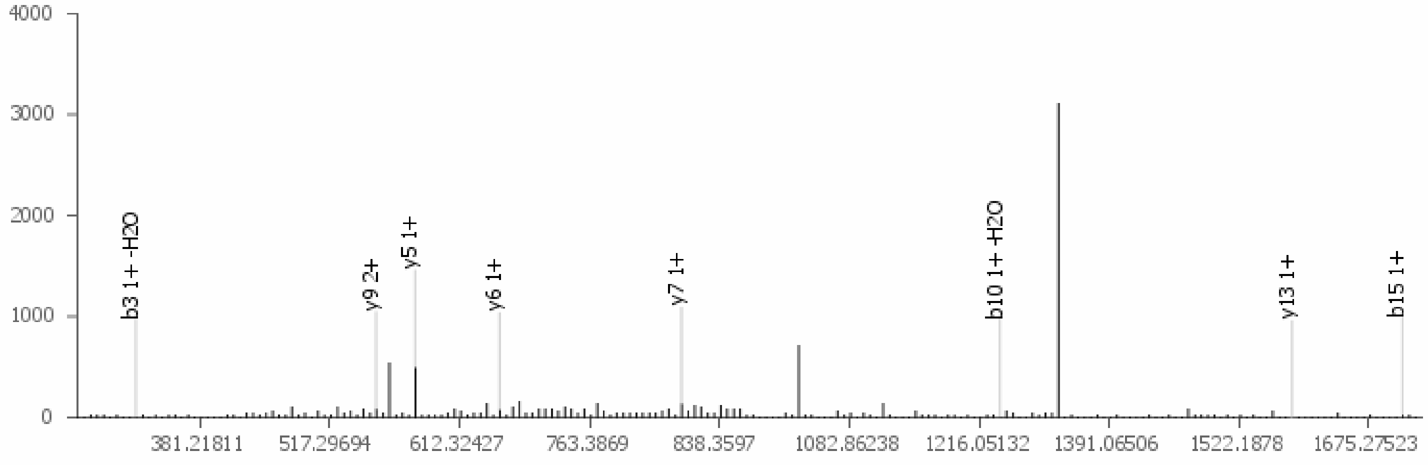

AT5G20200.1 - LMQLQND(pS)(pS)K - 662.249687 - Charge:2

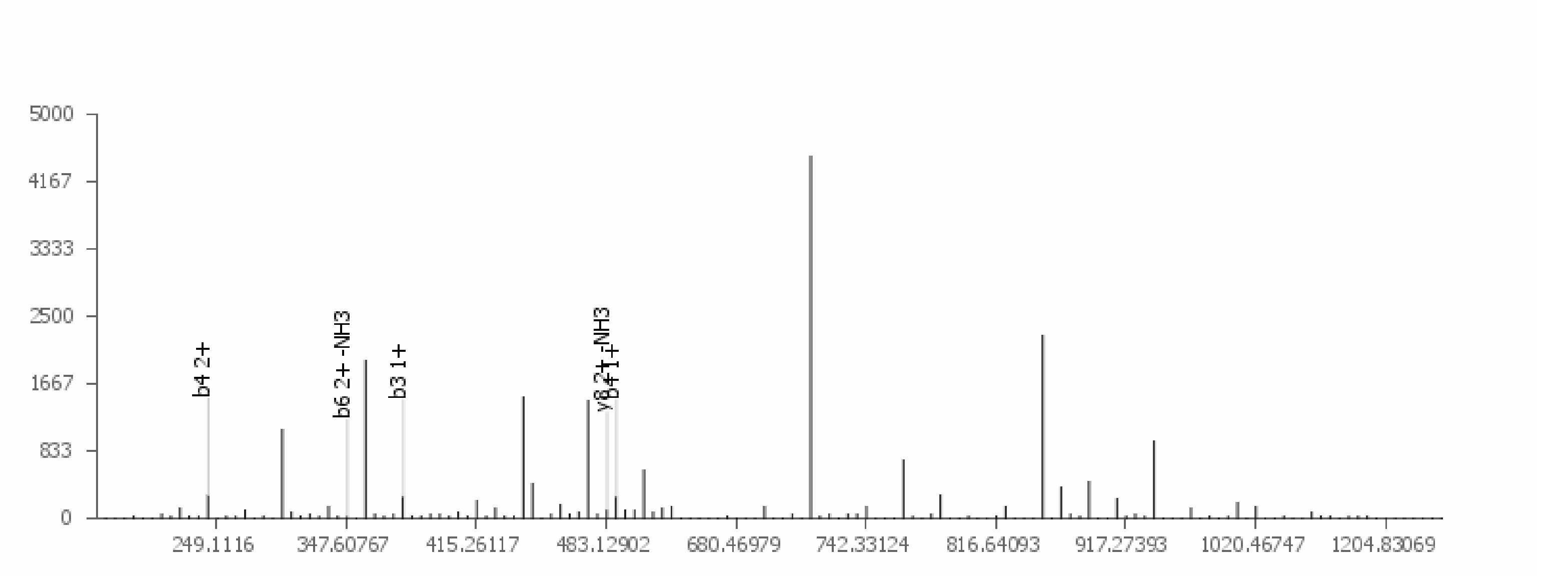

AT3G06500.1 - A(pY)GKLTGD(pY)TLQER - 887.878626 - Charge:2

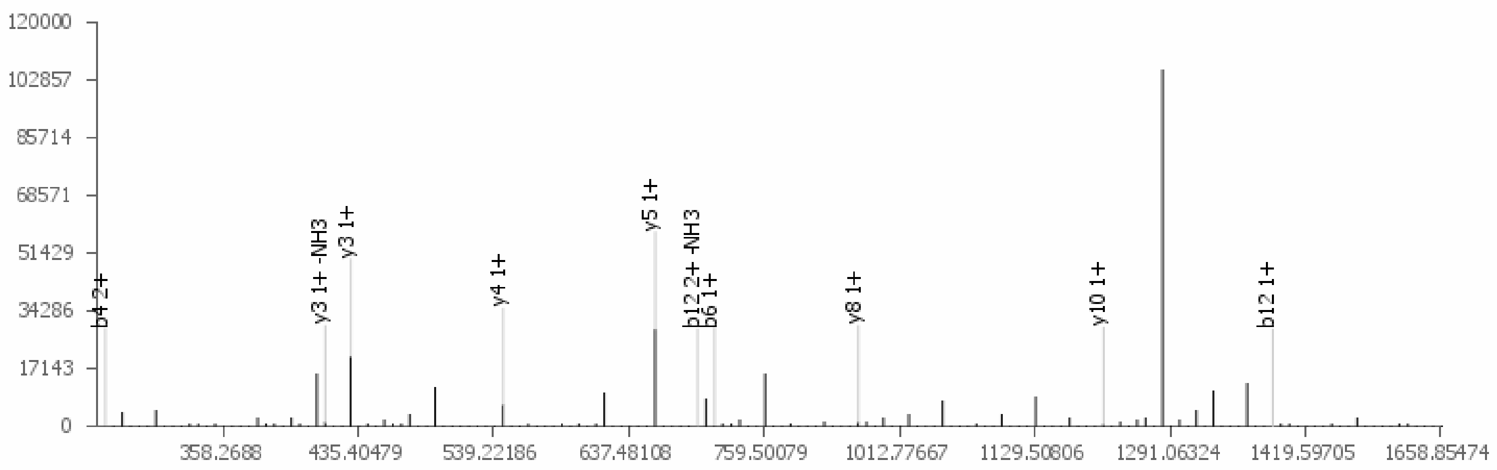

AT1G15480.1 - G(s)(s)NDI(pT)G(oxM)EQIVETMK - 1008.393353 - Charge:2

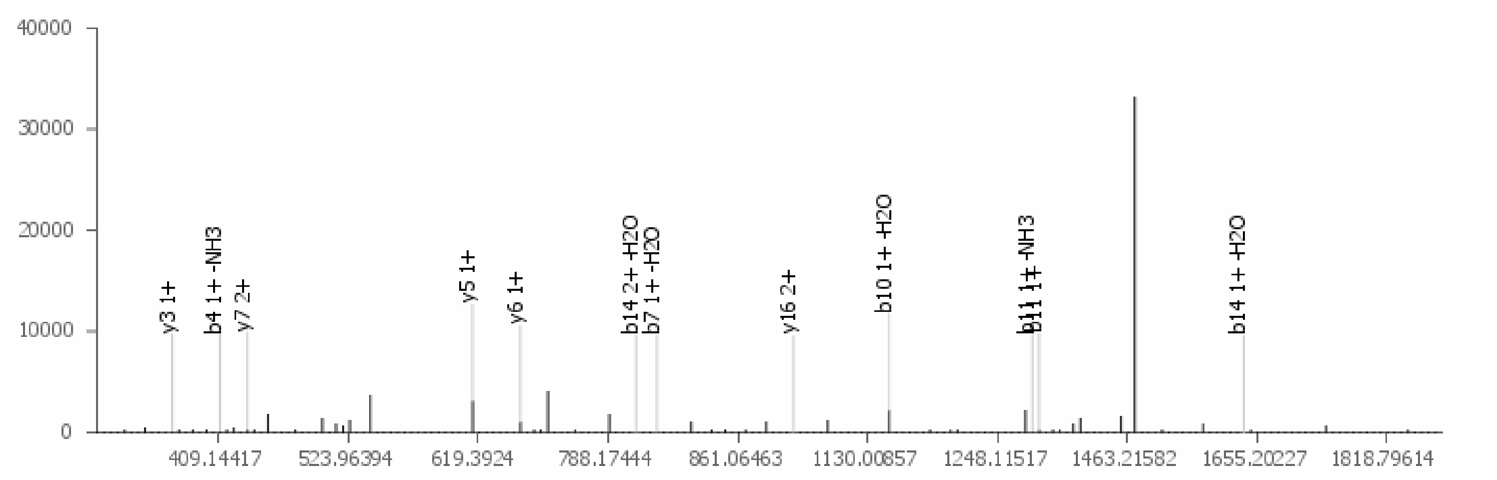

AT2G41360.1 - (pY)ISTSINT(pT)KNNNNNNK - 1050.439662 - Charge:2

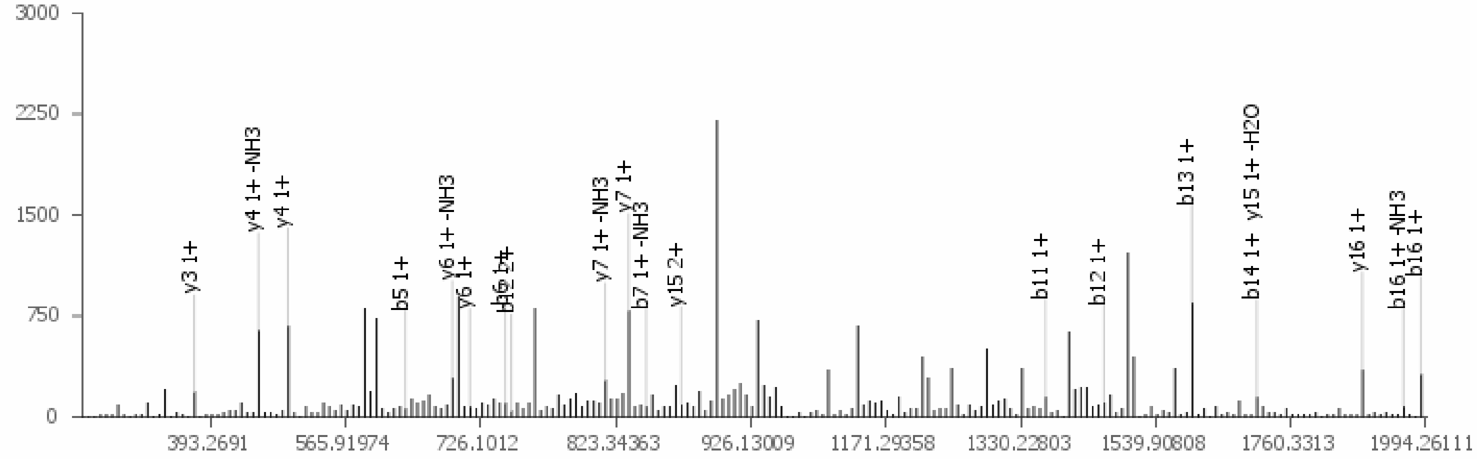

AT1G22800.1 - LI(oxM)(oxM)DT(pS)YD(oxM)IKSCR - 996.406308 - Charge:2

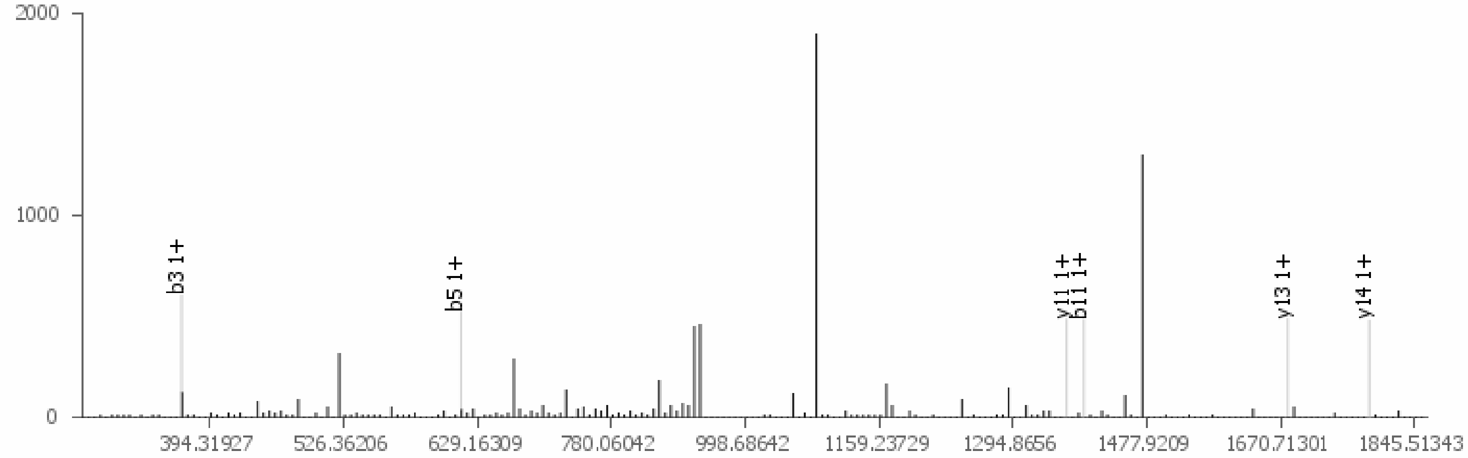

AT1G51160.1 - (pS)L(pT)AK(oxM)DPVNADK - 783.315054 - Charge:2

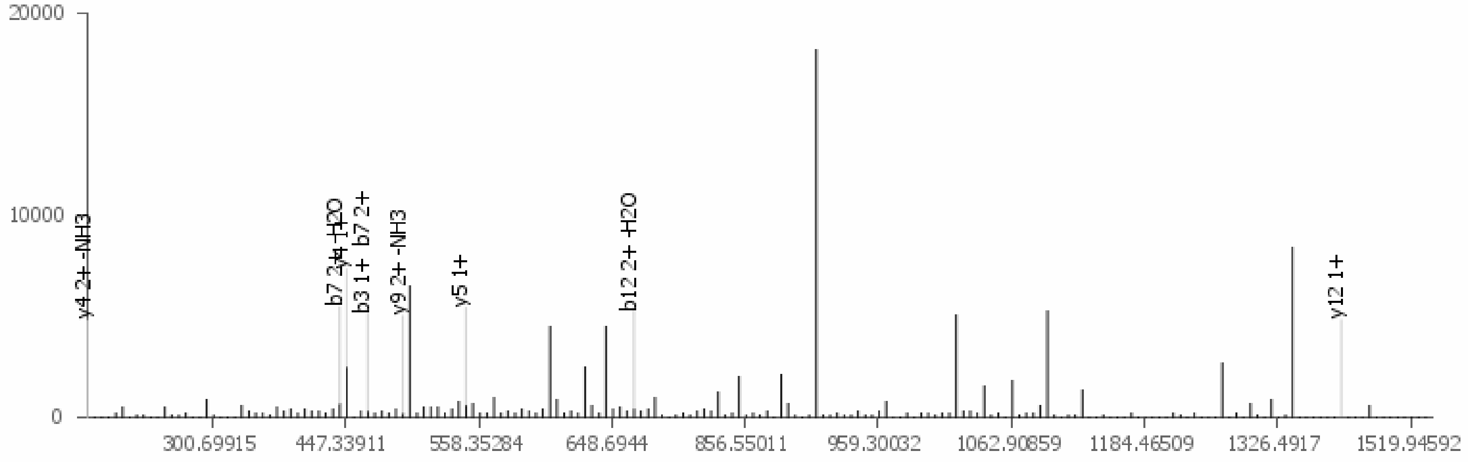

AT5G11510.1 - HNM(pS)EIP(oxM)PYTKESK - 944.404808 - Charge:2

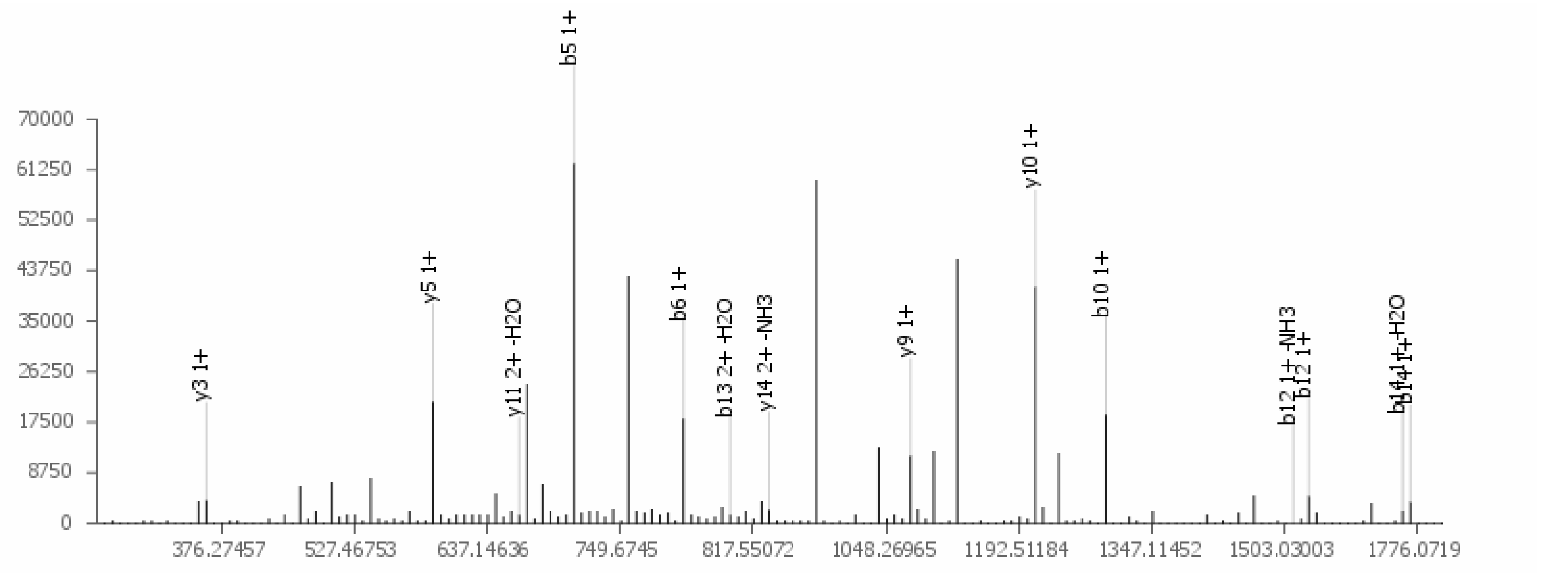

AT3G18180.1 - ML(pS)I(pT)EL(pT)(pT)NTTI(pS)K - 1026.85817 - Charge:2

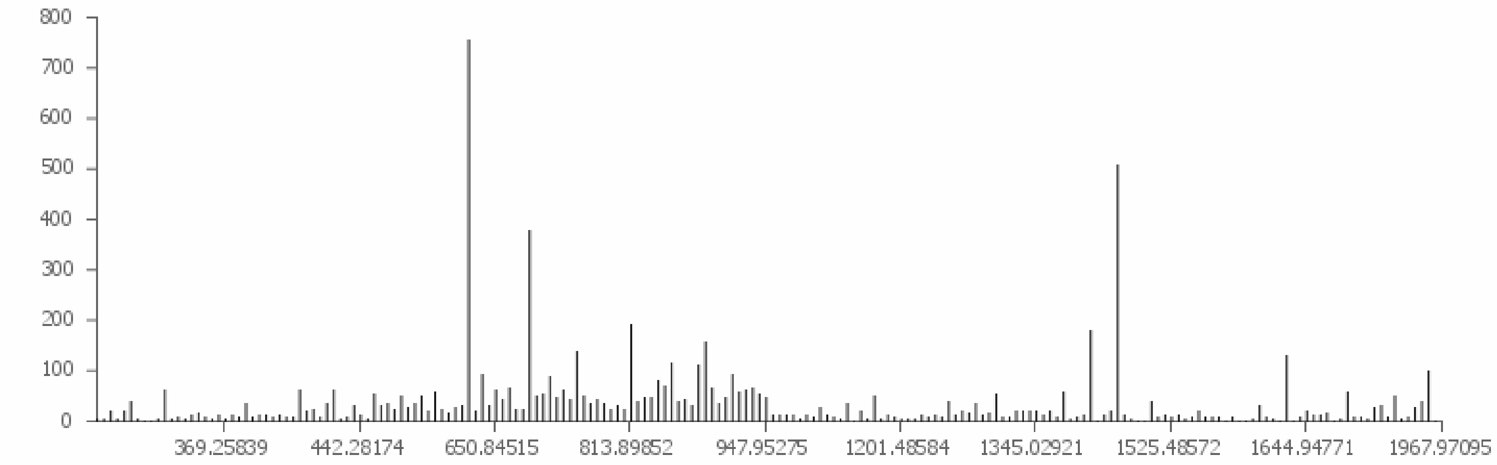

AT4G25650.1 - (pS)DANVV(pT)FR - 584.720948 - Charge:2

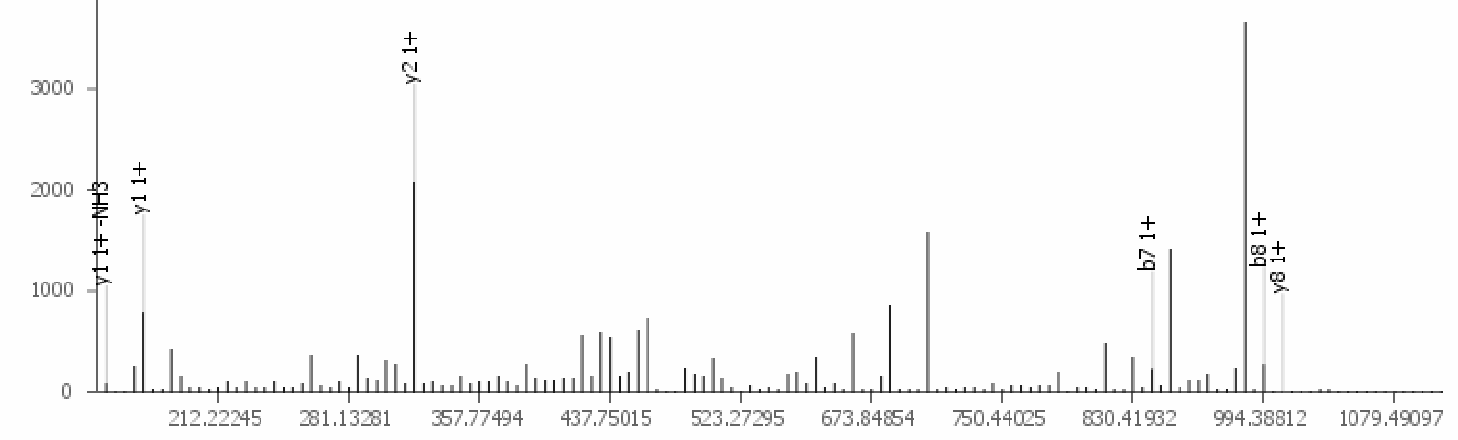

AT4G11810.1 - ERSIEEWQEYYIN(pY)K - 1065.470504 - Charge:2

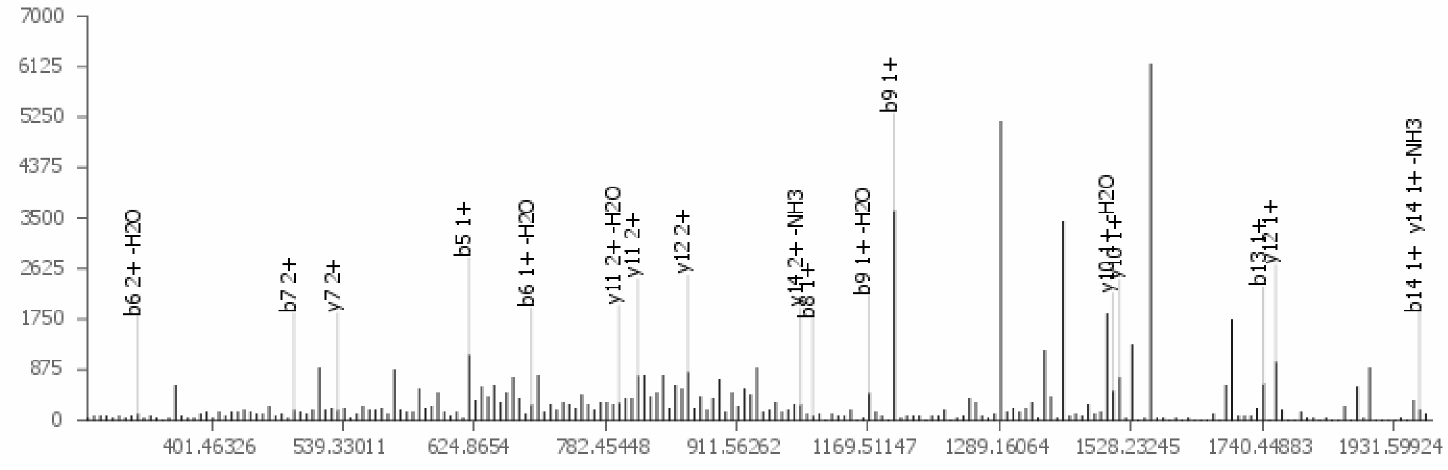

AT5G40480.1 - VLIFIA(pS)(pS)INVEENAGFVNVVQGR - 912.441484 - Charge:3

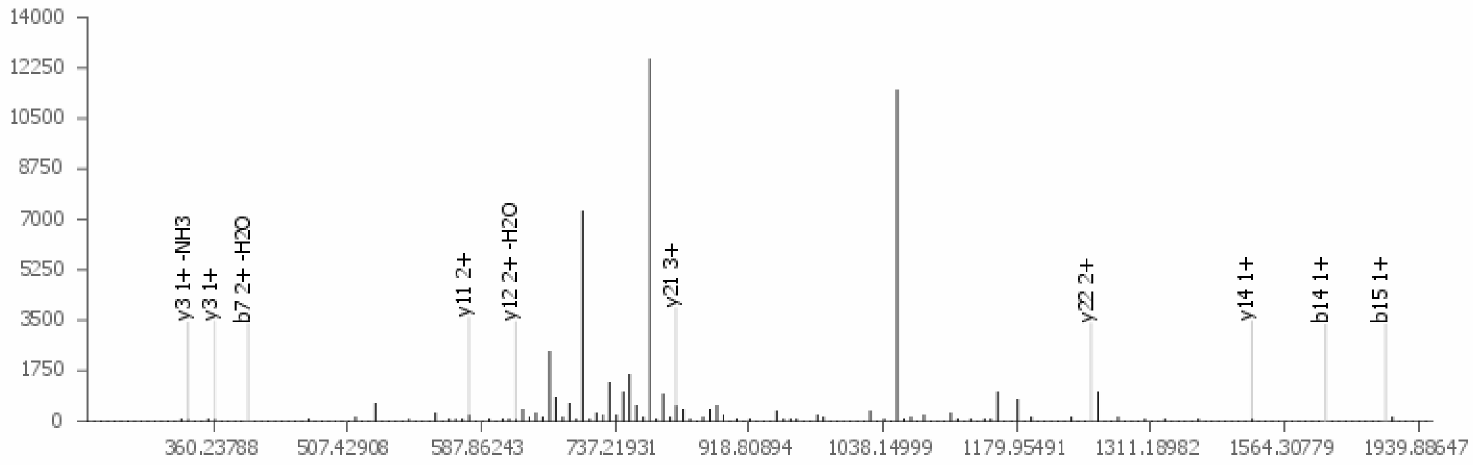

AT1G63070.1 - LRDGDEAA(pS)AELIK - 784.377794 - Charge:2

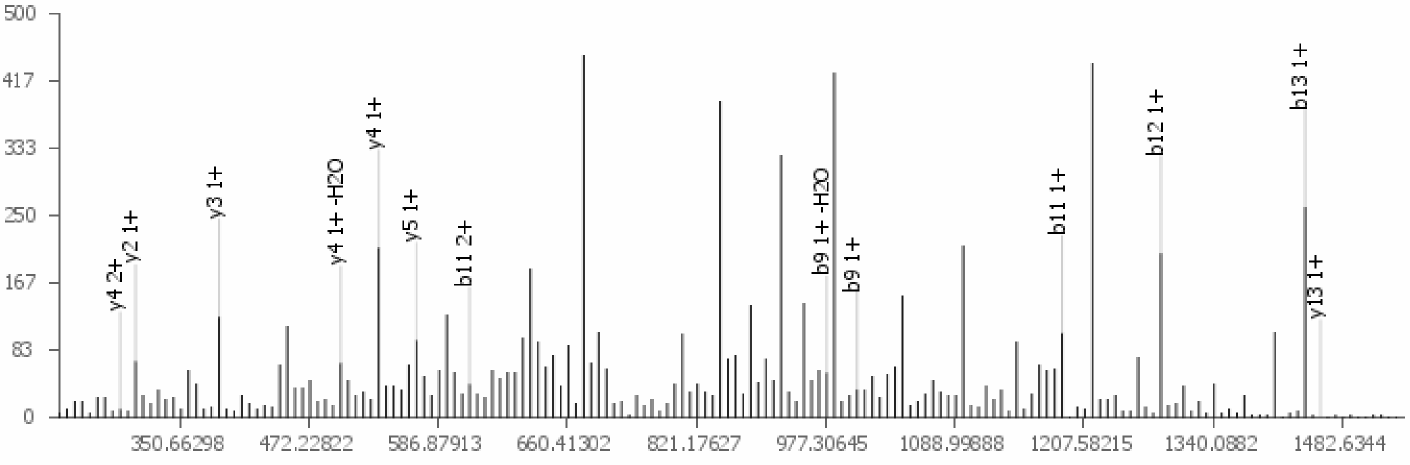

AT1G57750.1 - (oxM)VALEIIRN(pY)DFK - 854.418561 - Charge:2

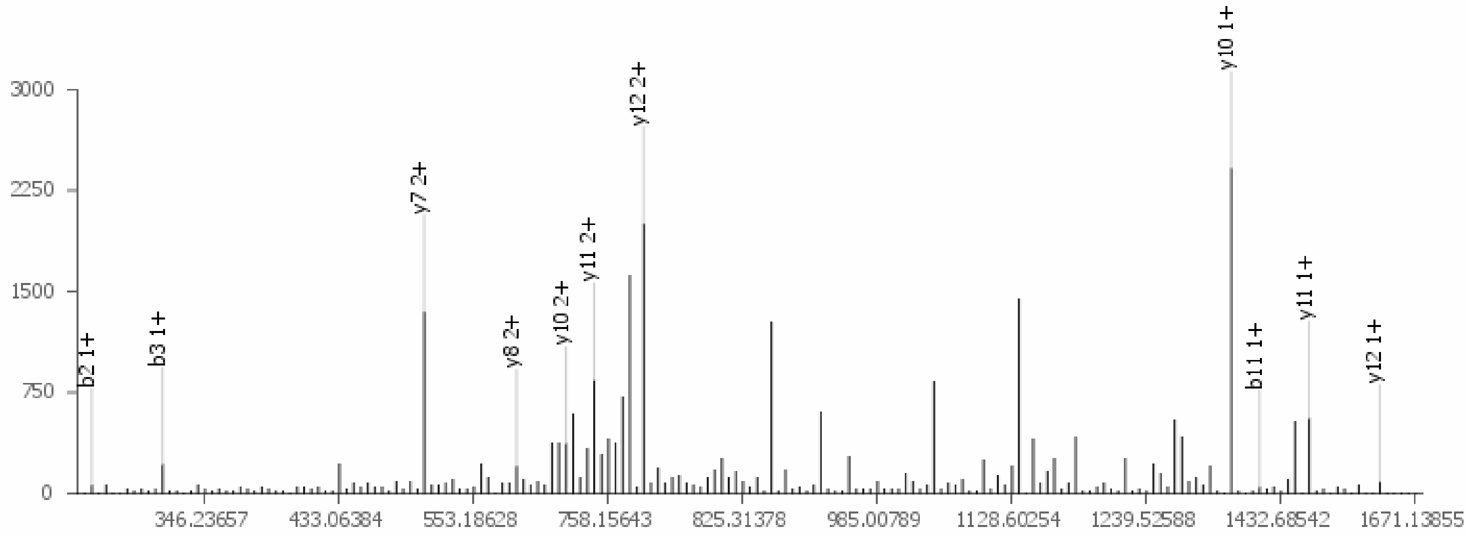

AT1G58050.1 - AALPI(pS)EVK - 504.264726 - Charge:2

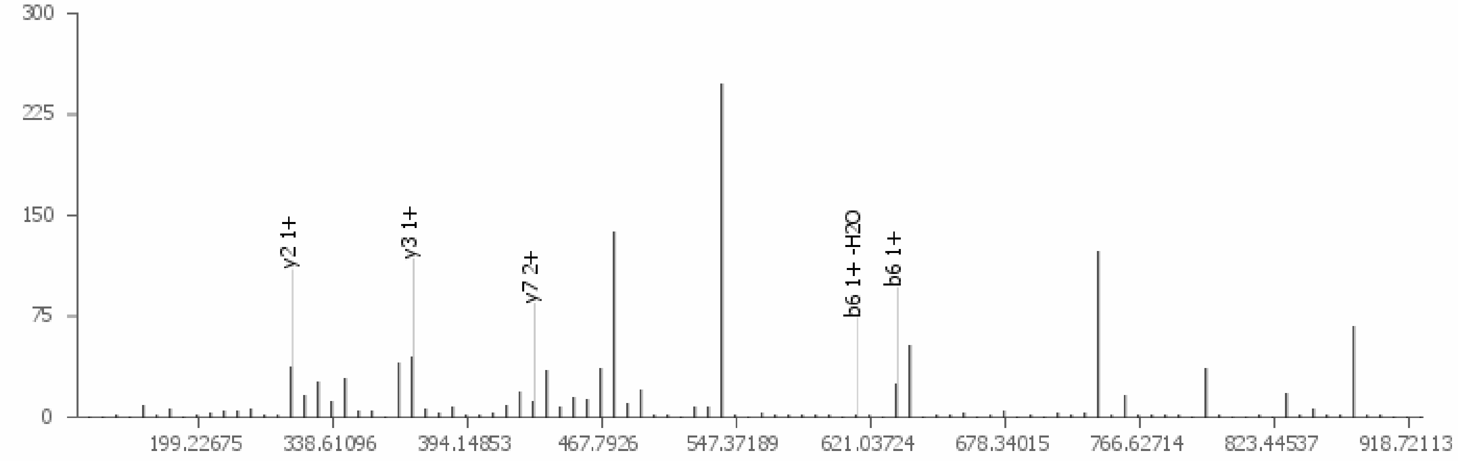

AT4G33200.1 - RAEVLDA(pS)AR - 584.280504 - Charge:2

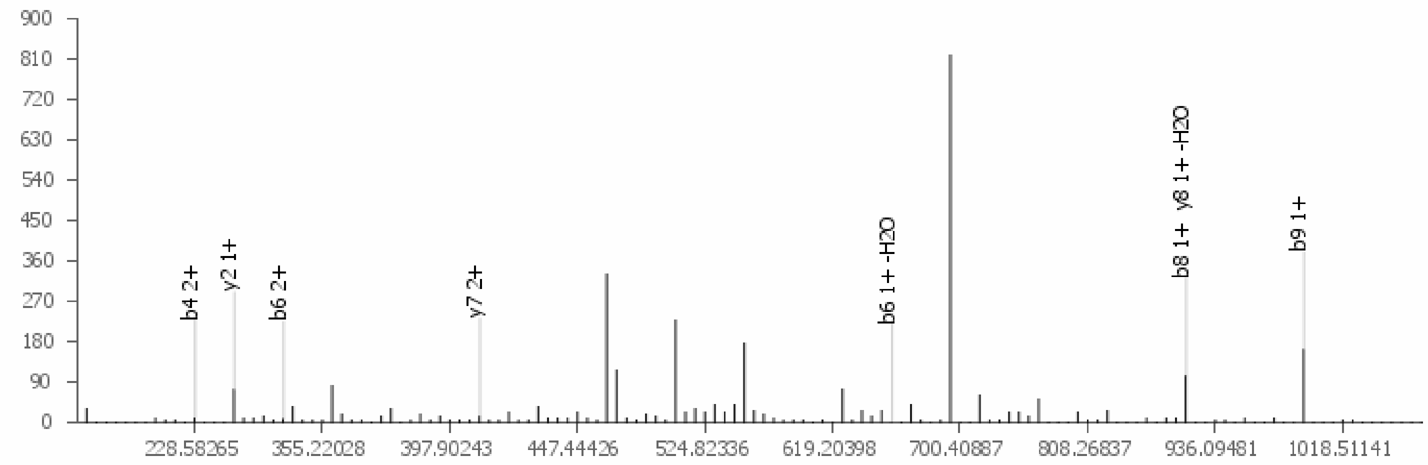

AT5G07940.1 - FAPWA(pS)NQ(pS)YQQ(s)(t)HQGPFPGILGG(s)N(oxM)TSGFPYSR - 848.53911

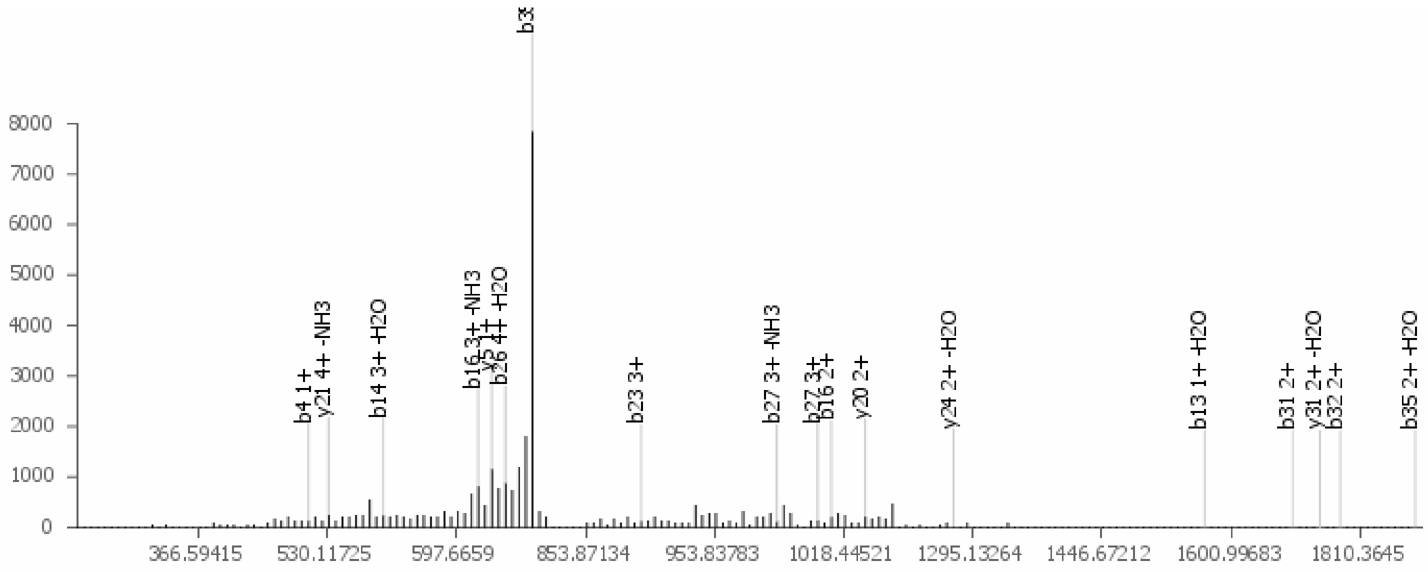

AT1G51710.1 - TALYVKE(pS)LID(pS)LPR - 932.949193 - Charge:2

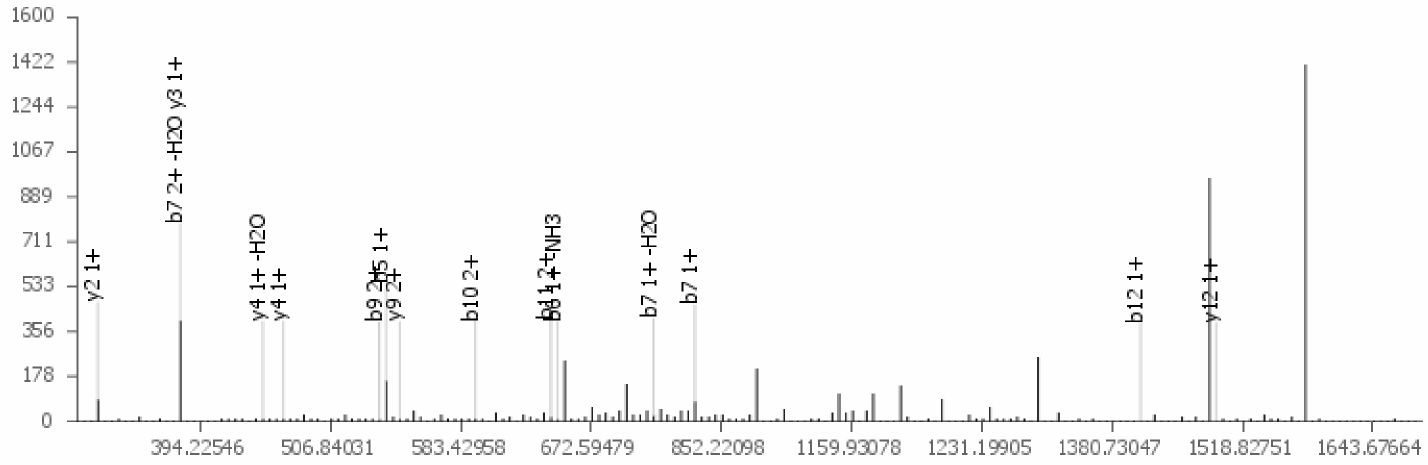

AT3G24840.1 - (pS)IEQDLQKTK - 635.30256 - Charge:2

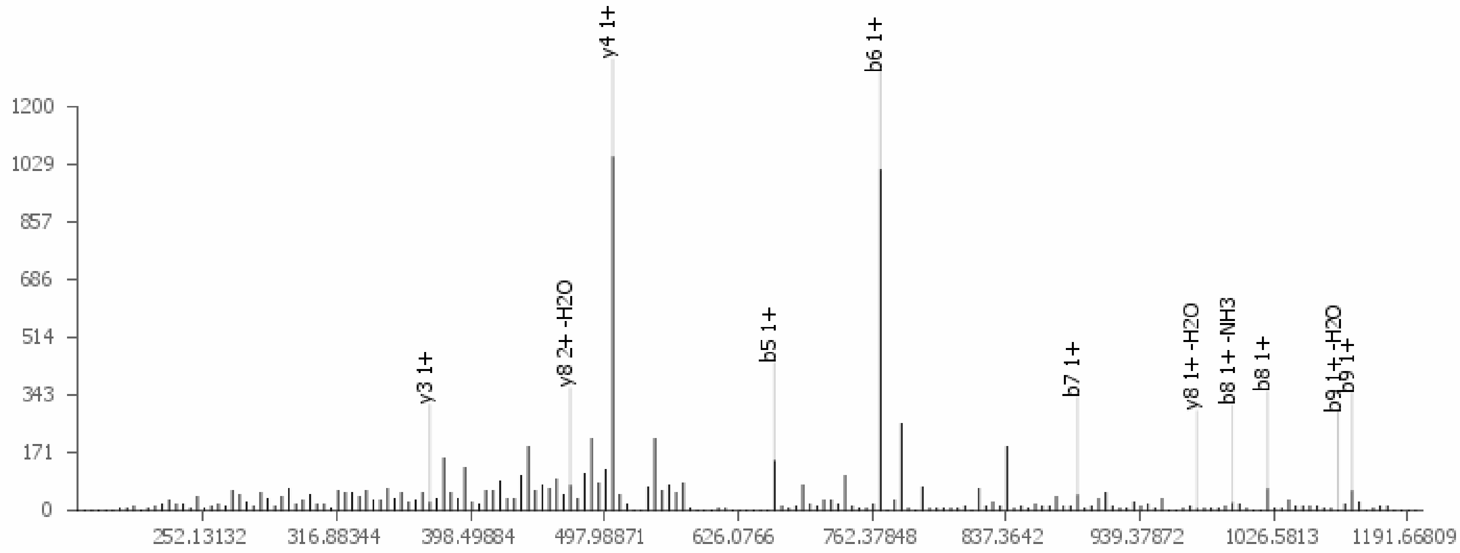

AT1G12240.1 - TGISLV(pY)DTIDFK - 776.364513 - Charge:2

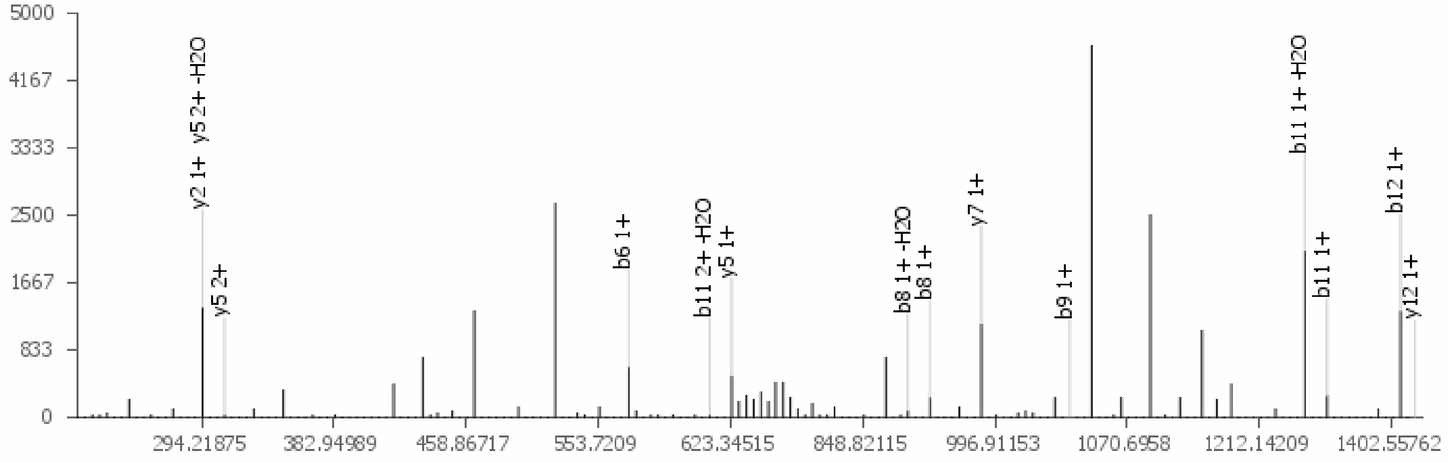

AT2G28650.1 - IFLTLDVYQ(pT)IVDLLPK - 1036.061604 - Charge:2

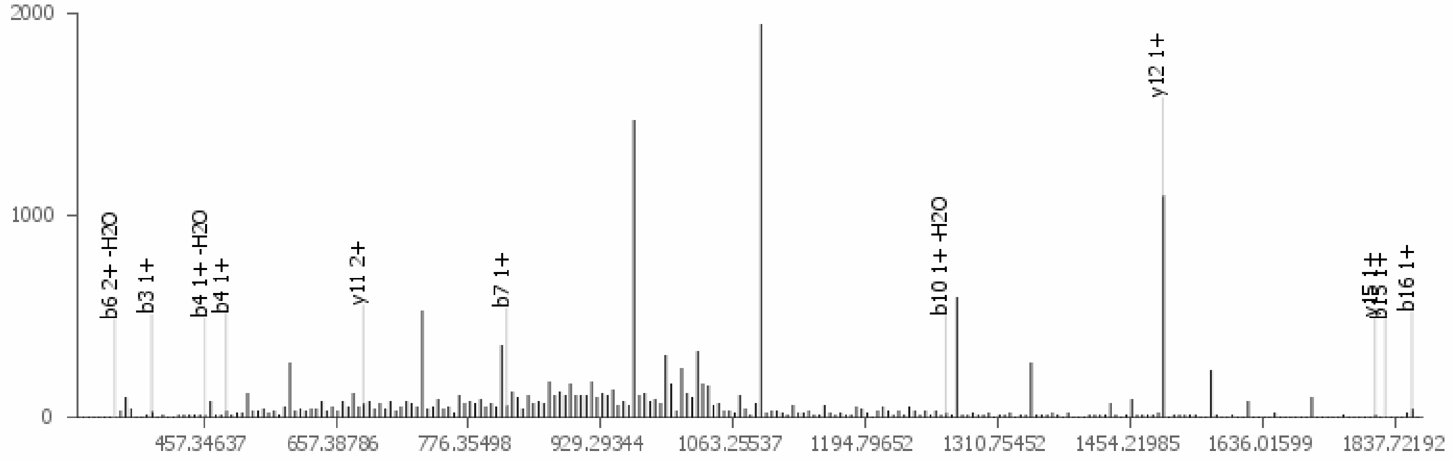

AT4G04630.1 - EV(pS)GVKQSSAPMNVPDWSK - 1063.478302 - Charge:2

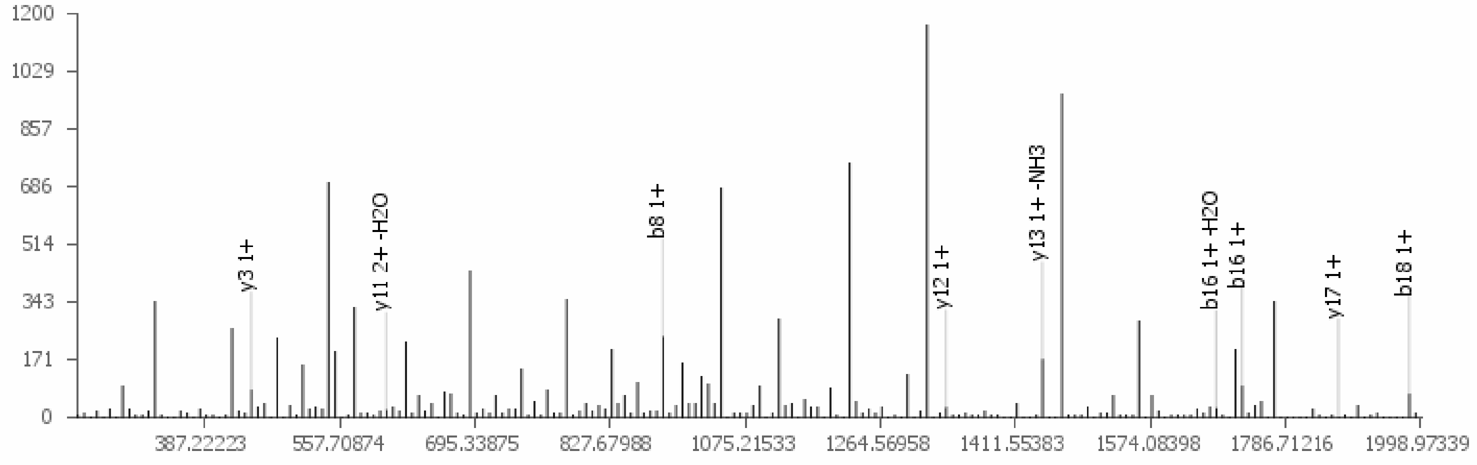

AT5G07150.1 - LREI(pT)GI(pT)PEAALPSR - 942.457519 - Charge:2

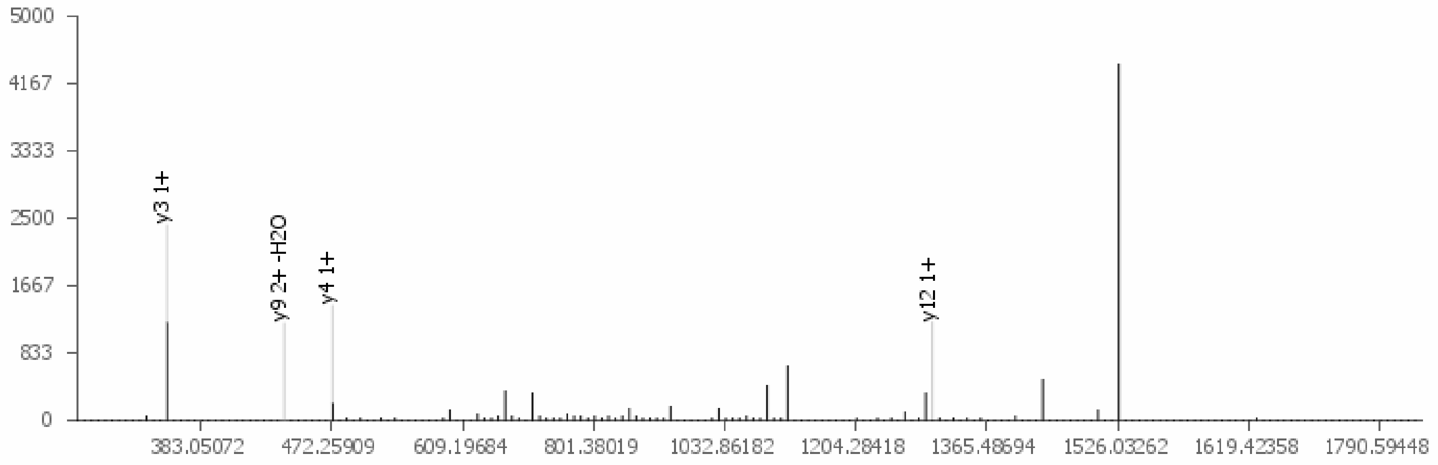

AT3G27300.1 - QG(pS)RGPAEADQLLK - 775.367121 - Charge:2

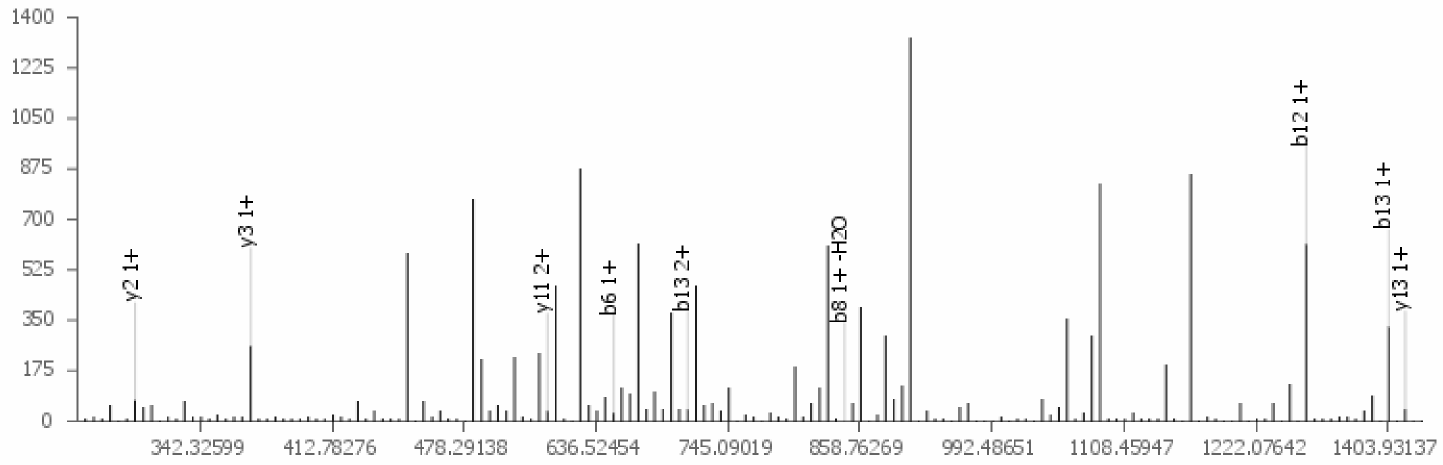

AT3G52340.1 - SLRD(pT)IDELK - 635.313219 - Charge:2

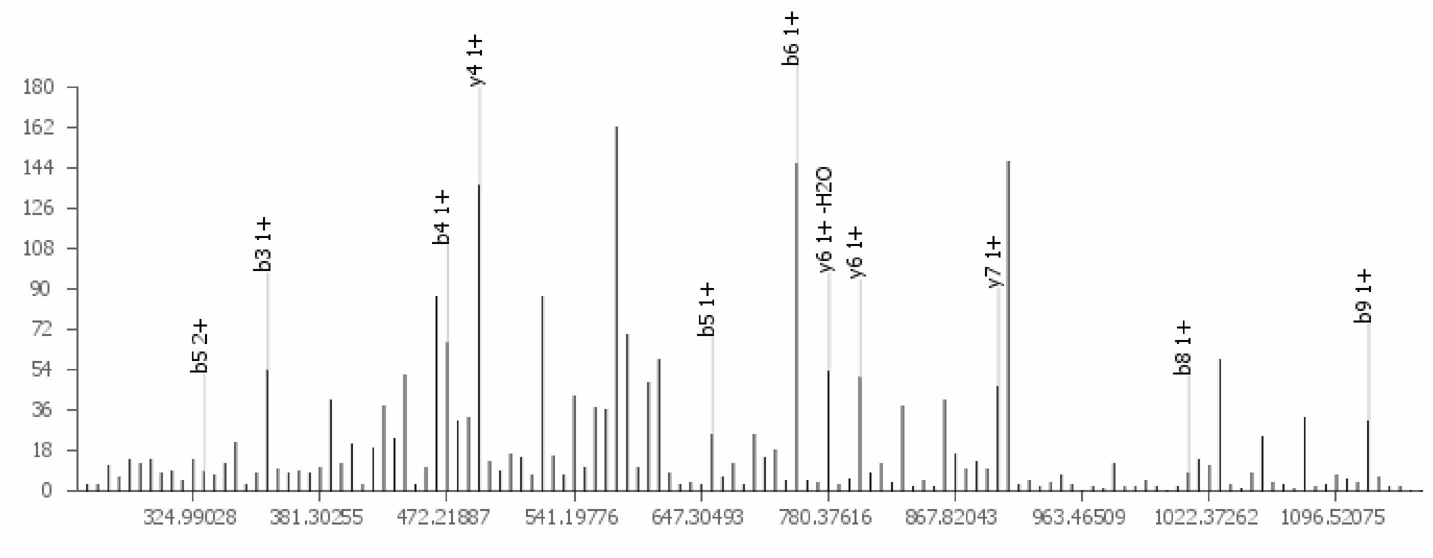

AT3G49670.1 - MGENFLNG(pS)IPKELFGLPK - 1086.039316 - Charge:2

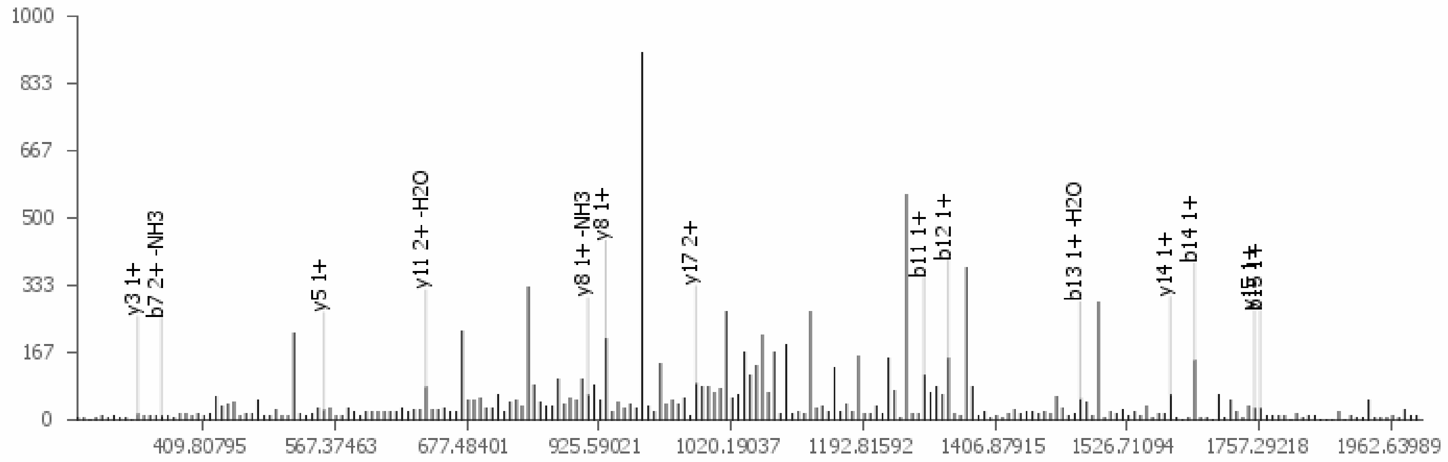

AT1G59610.1 - TS(pT)APPLKLIDLPGLDQR - 672.356837 - Charge:3

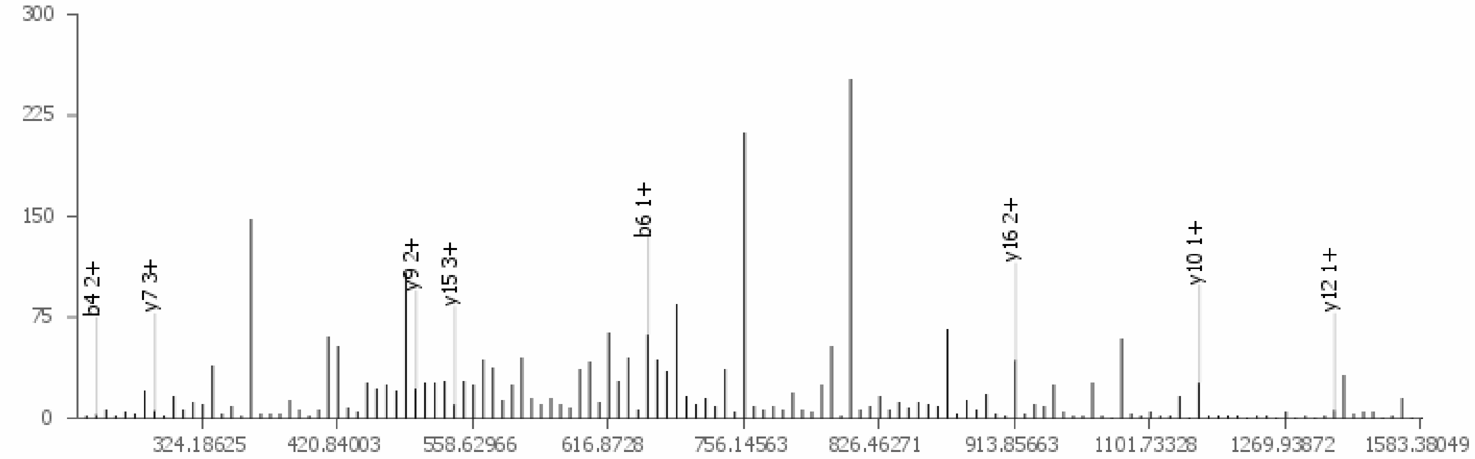

AT5G44680.1 - KSPPKPLNPIA(pS)K - 728.900731 - Charge:2

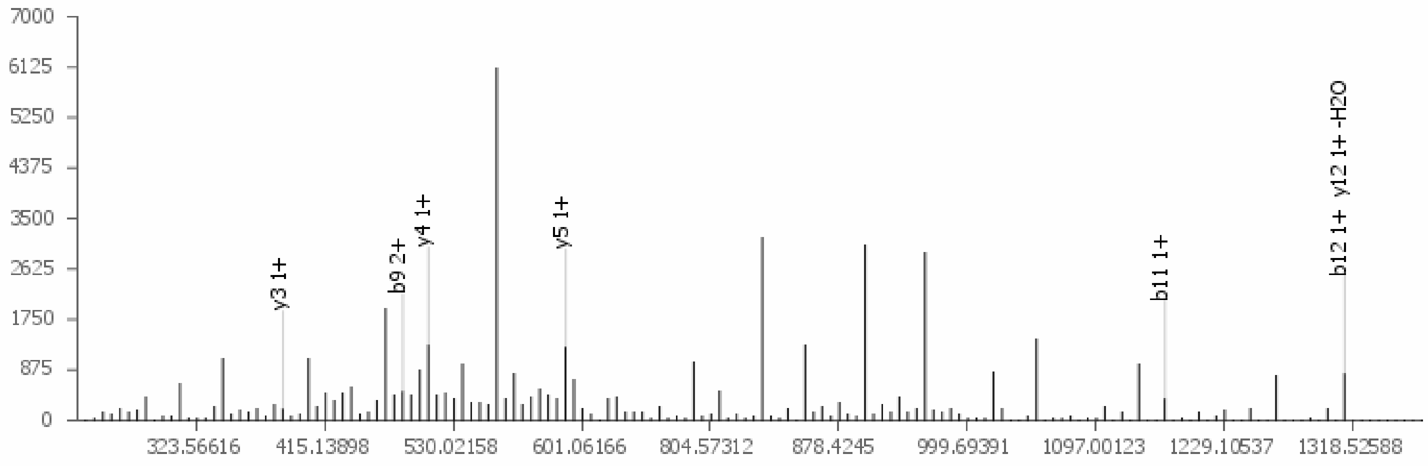

AT1G06550.1 - G(pT)GRAFSAGGDLK - 658.802989 - Charge:2

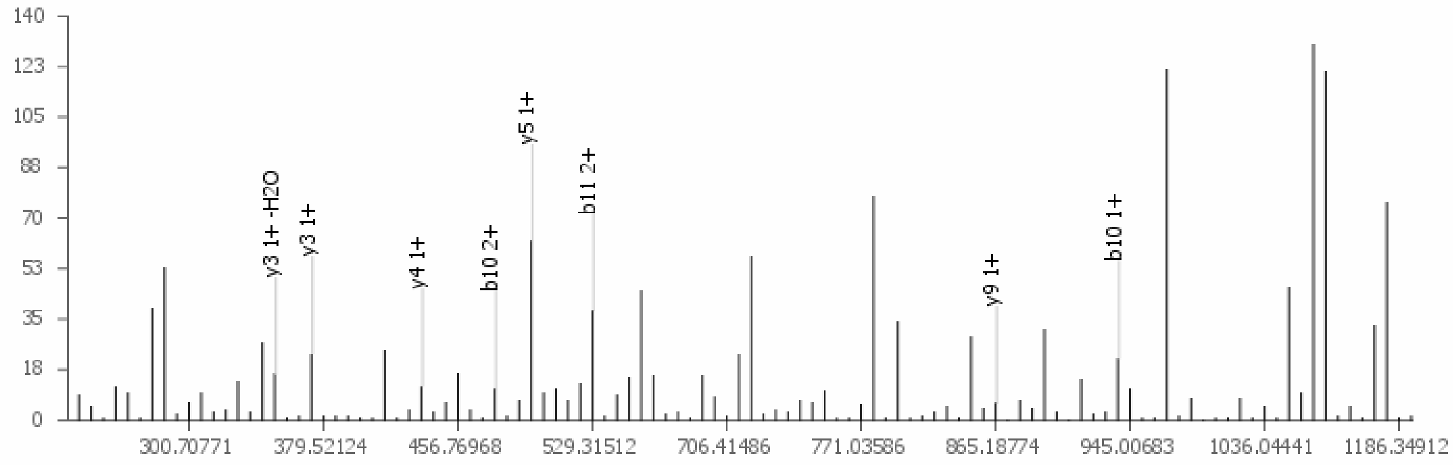

AT2G29190.1 - LKG(pS)(pS)NVLGGVGDR - 759.840334 - Charge:2

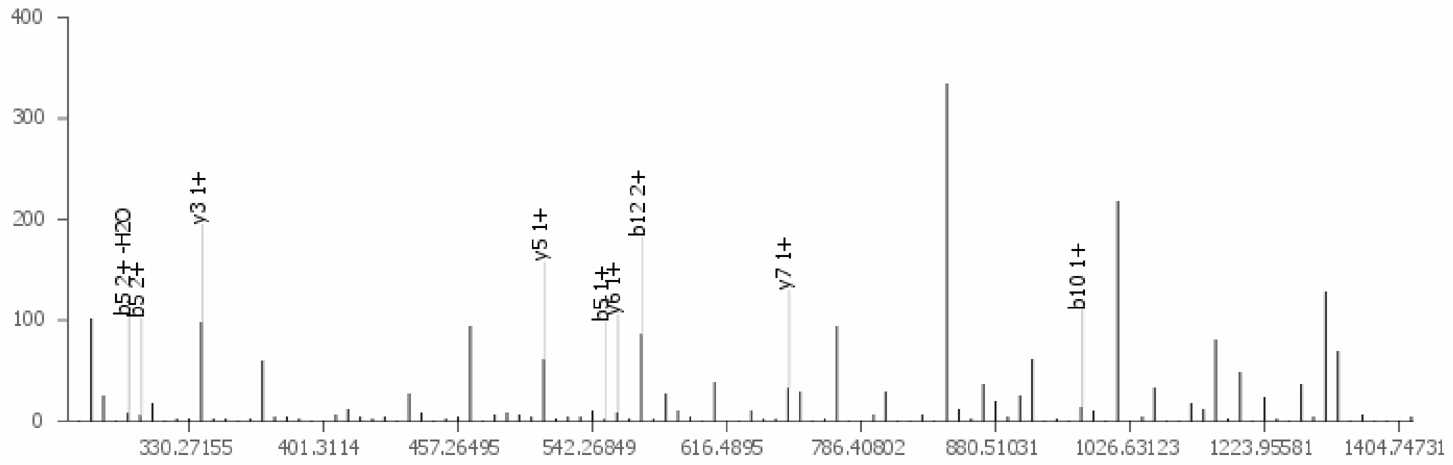

AT1G17840.1 - LAANAFL(pS)G(pT)VLLNGR - 888.934299 - Charge:2

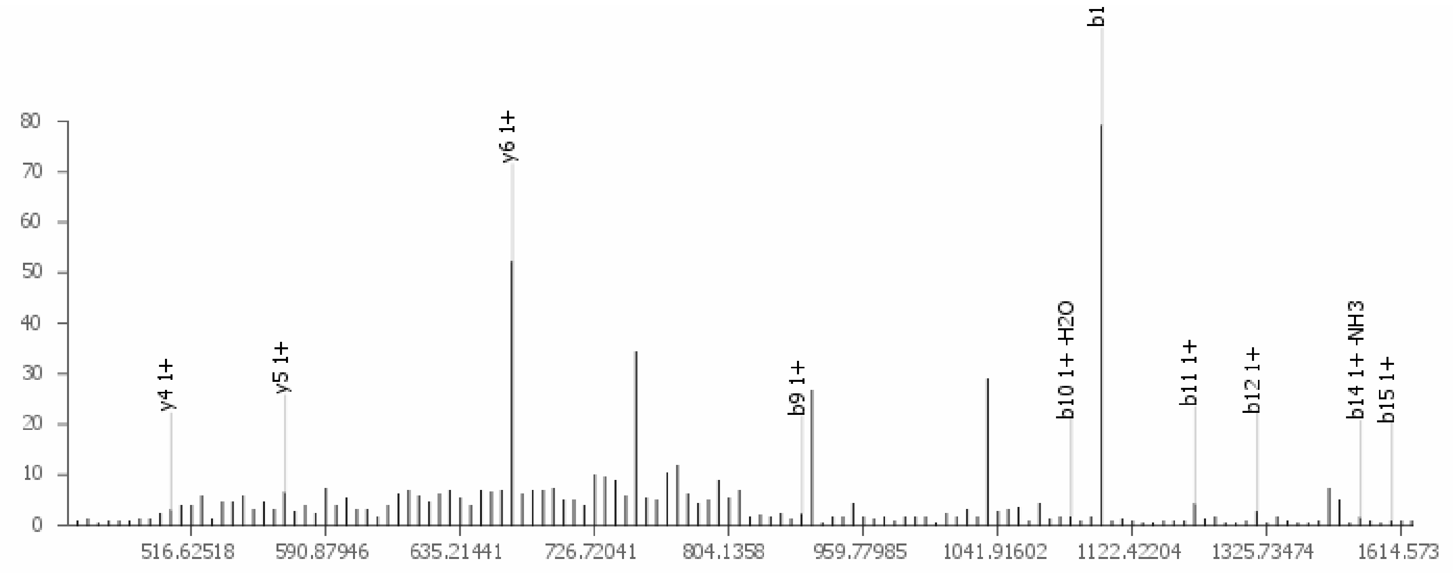

AT1G15940.1 - AEVILE(pT)VAK - 576.800706 - Charge:2

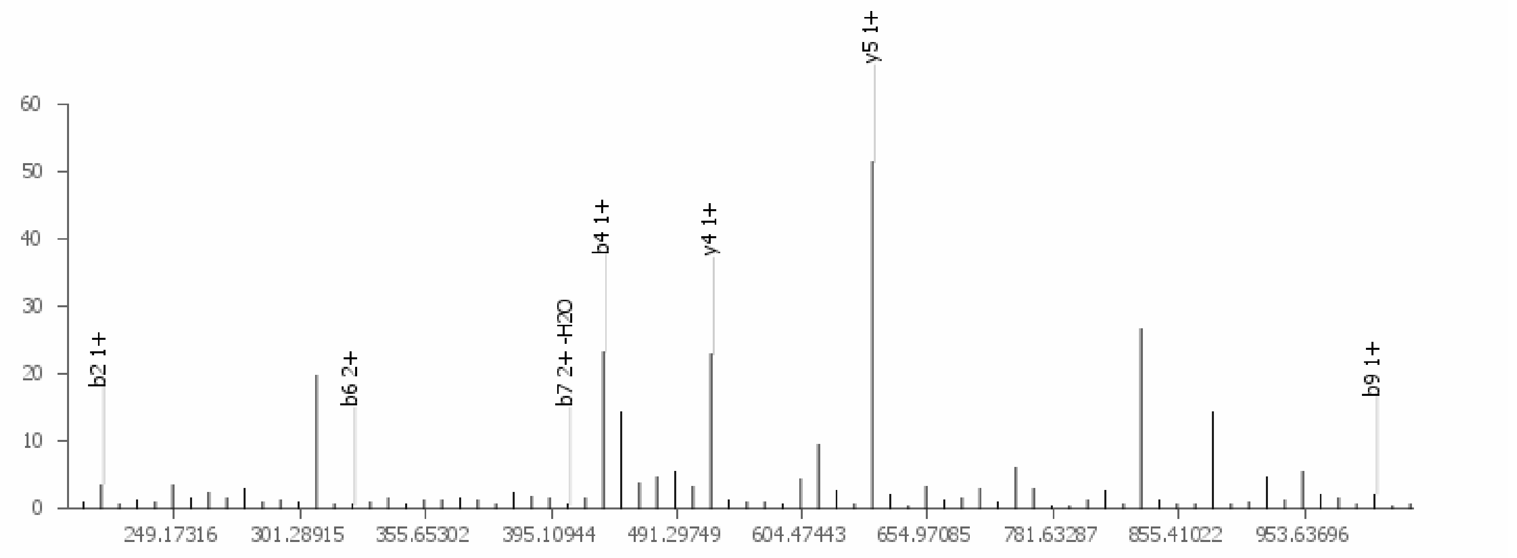

AT3G22080.1 - TPN(pS)YSLKLQNISQVEK - 1015.012558 - Charge:2

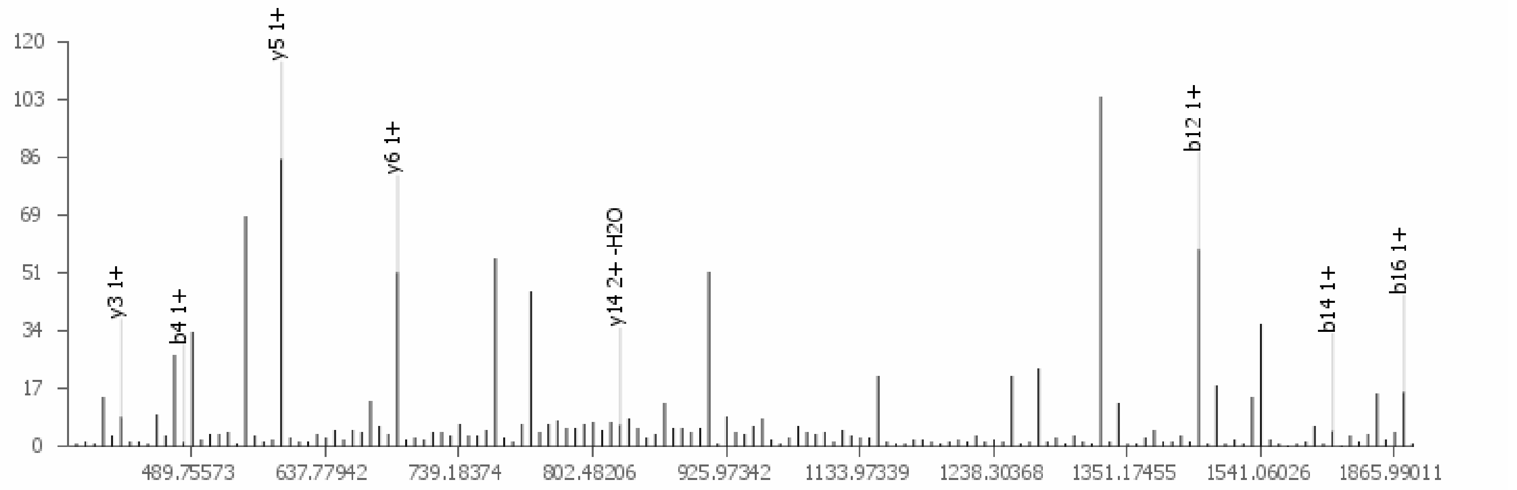

AT5G04180.1 - IVHNSTEILQT(y)(y)KPVEAILK - 847.119282 - Charge:3

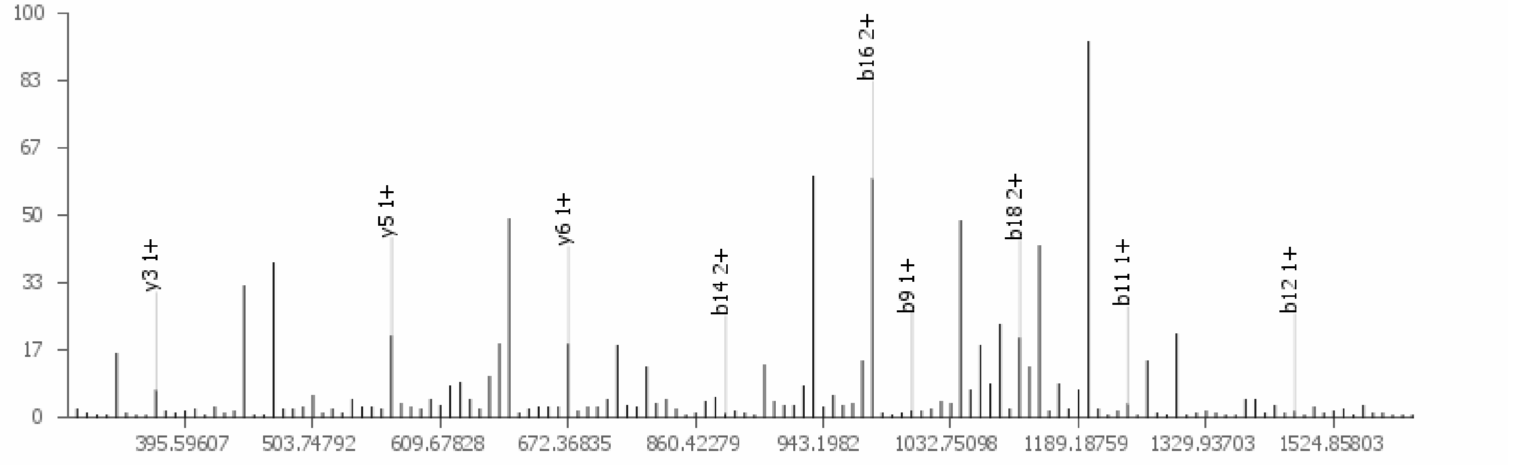

AT2G07040.1 - LGRLNHENLLPIVA(y)(y)YK - 752.72989 - Charge:3

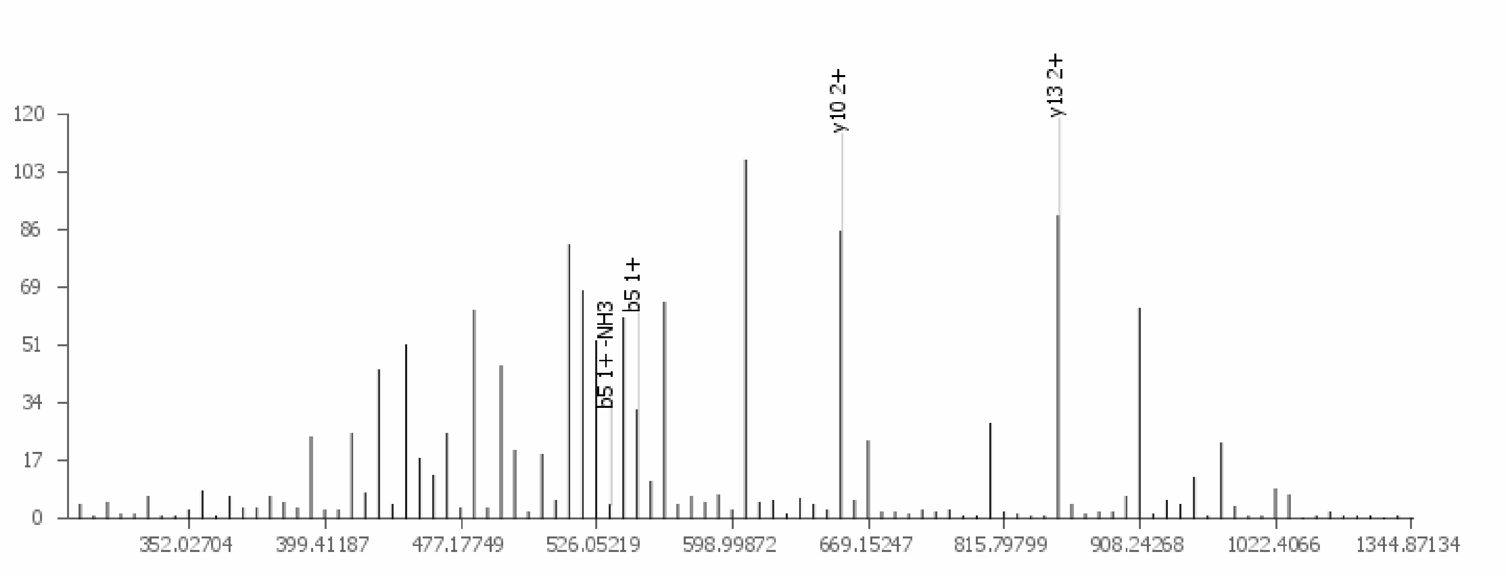

AT5G01020.1 - TTAPL(pS)WSR - 549.752715 - Charge:2

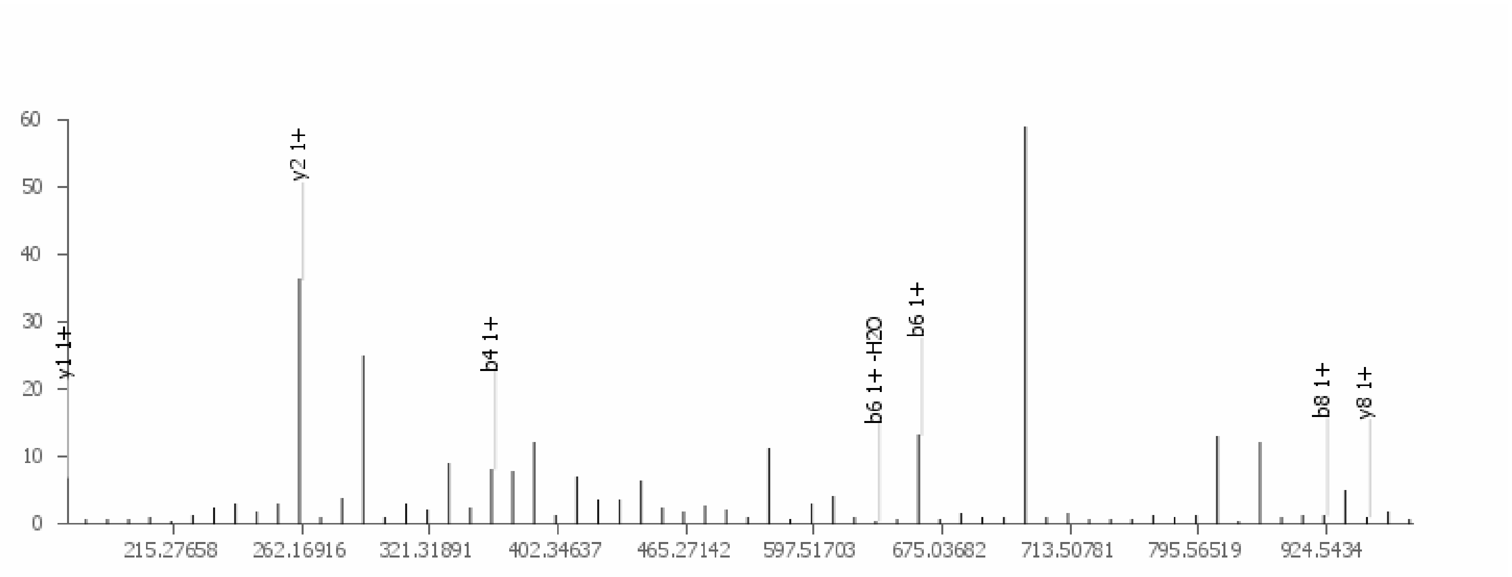

AT2G31340.1 - (pY)WWQRIIR - 650.825727 - Charge:2

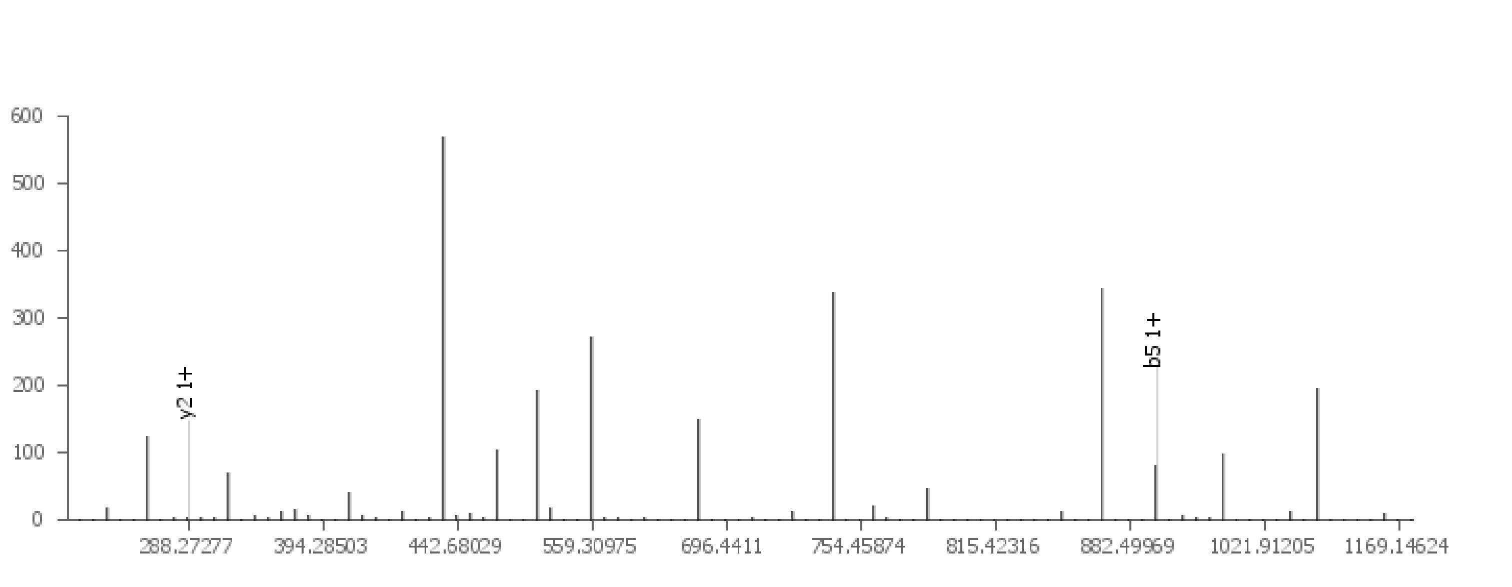

AT4G01023.1 - VAA(oxM)A(t)KAGE(t)A(t)(oxM)AAD(oxM)VK - 1048.467213 - Charge:2

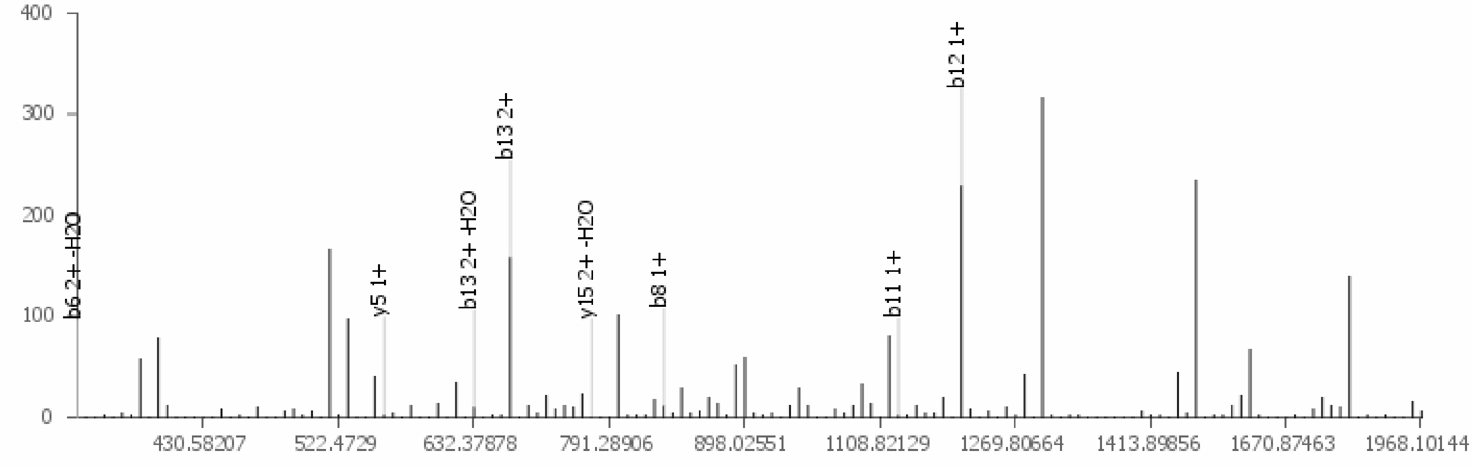

AT4G00890.1 - (pS)LPLSPL(s)PK(s)K - 707.344898 - Charge:2

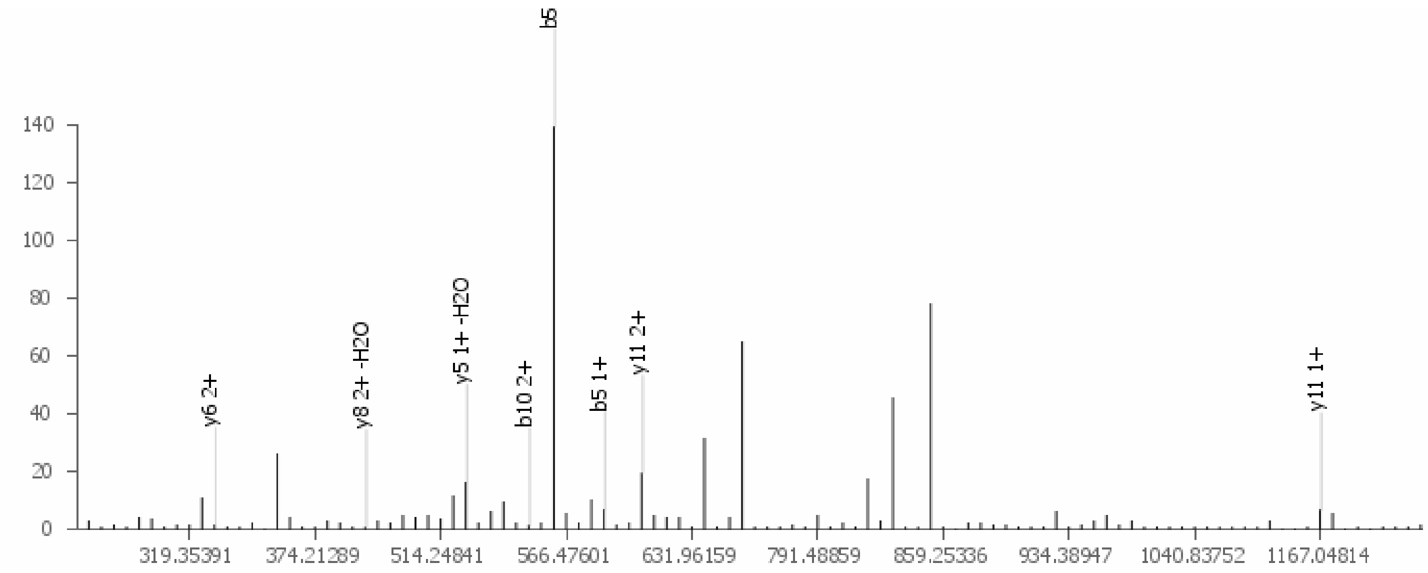

AT5G55320.1 - RWNLIV(s)A(s)LR - 697.867152 - Charge:2

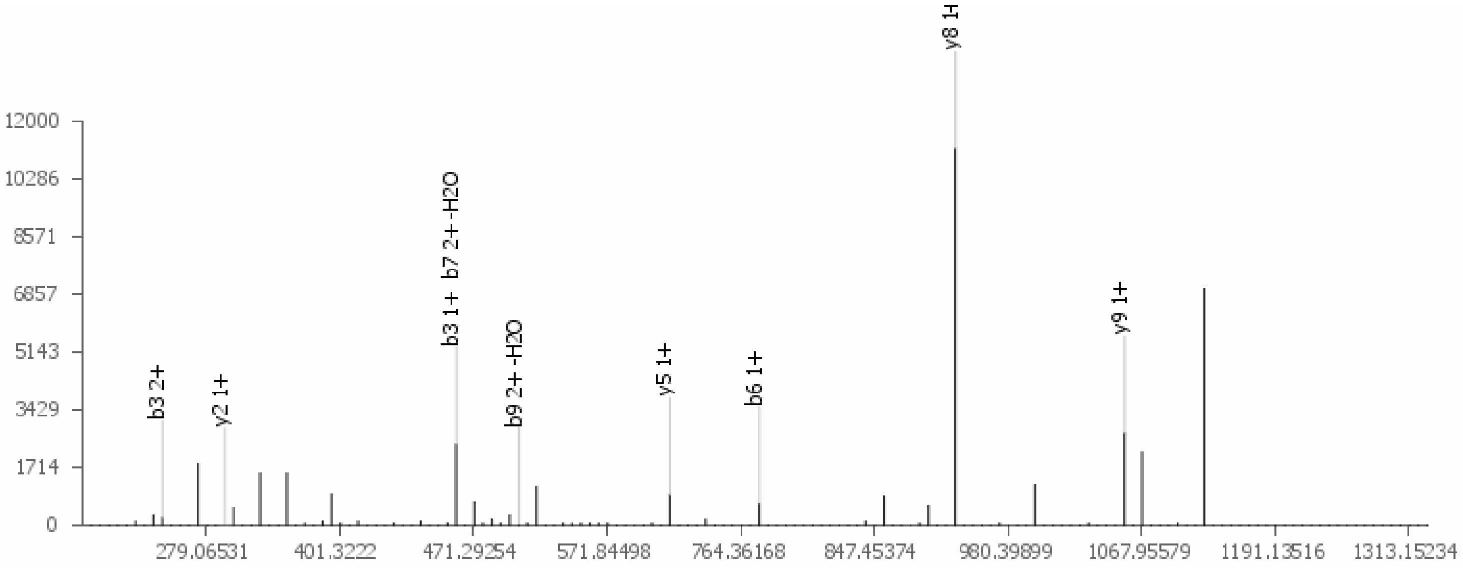

AT5G37450.1 - DHV(pT)TIVK - 496.743095 - Charge:2

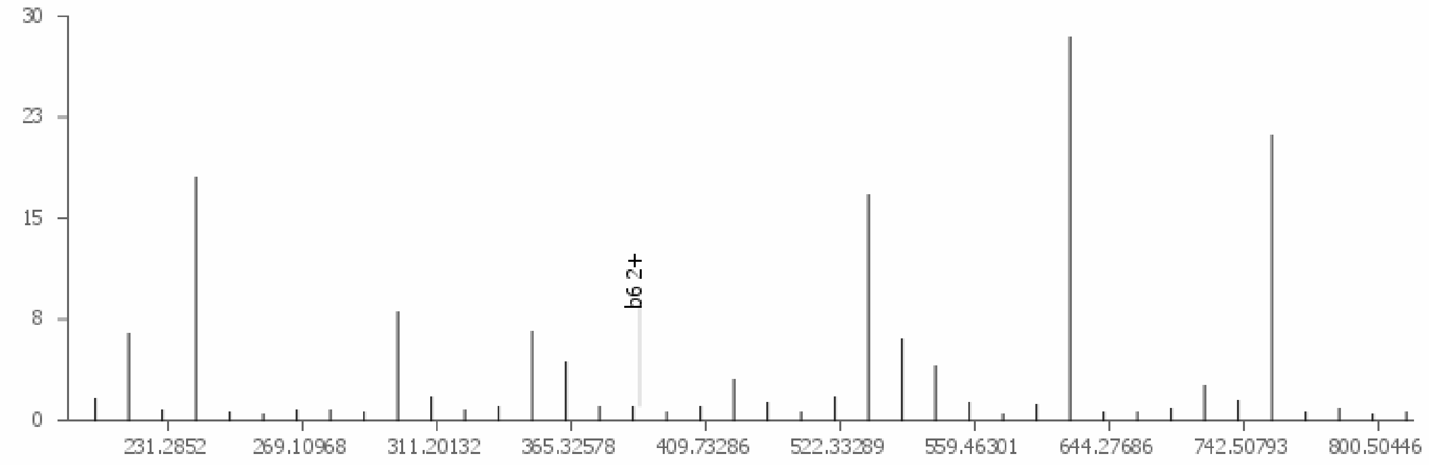

AT1G58250.1 - AAR(pS)GNLSLDK - 606.296188 - Charge:2

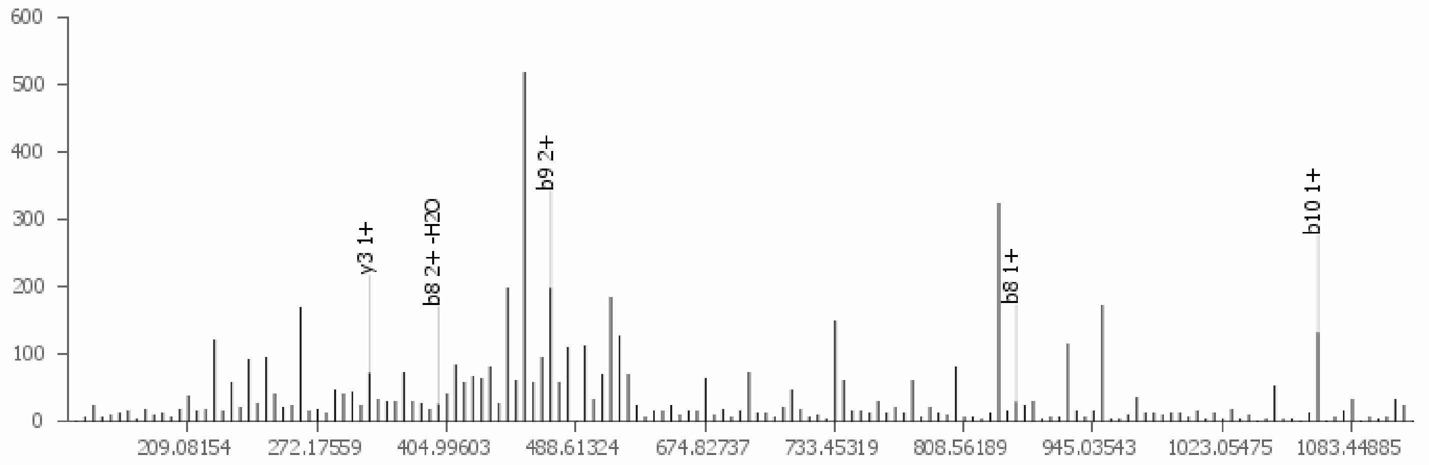

AT3G10380.1 - YVQ(pT)FLER - 568.261211 - Charge:2

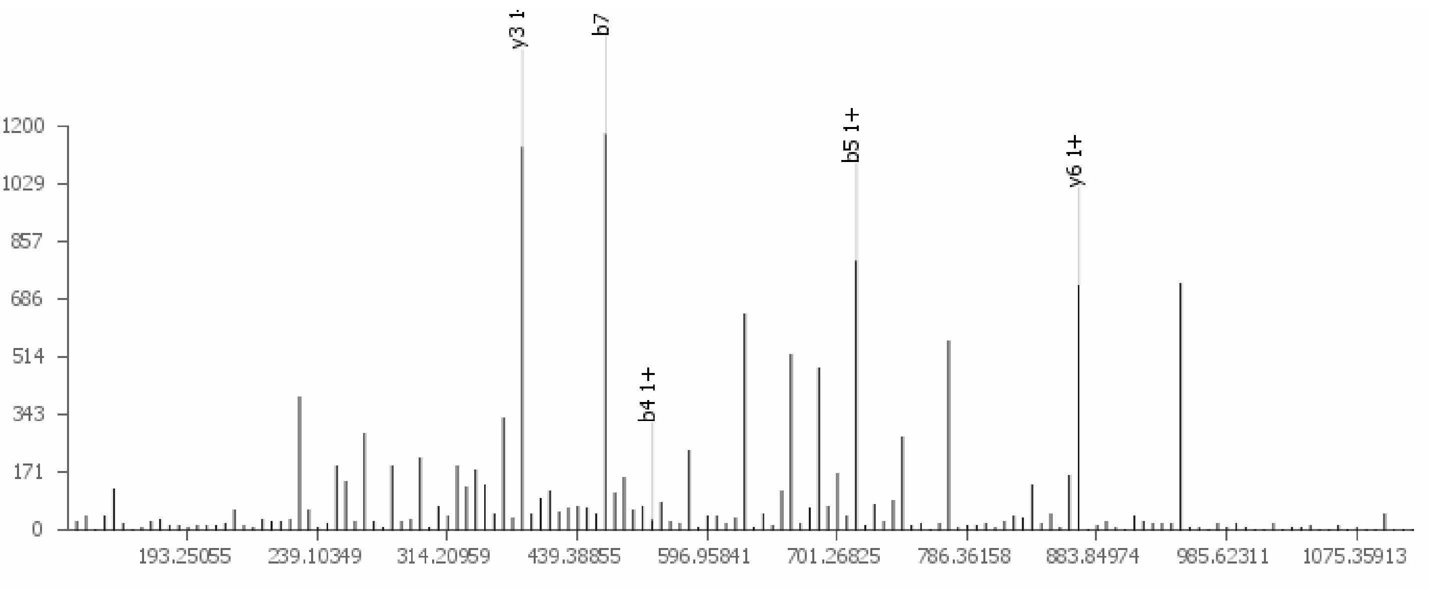

AT3G11800.1 - DSL(pT)NPFKNIF - 688.319845 - Charge:2

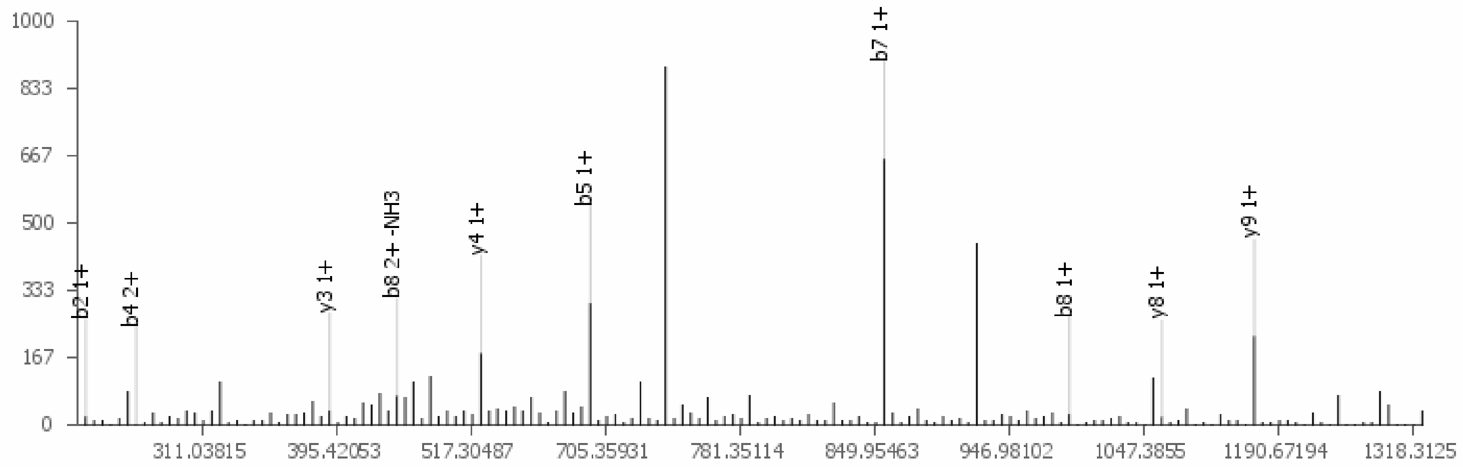

AT1G07705.1 - TLSSGGGL(pS)IP(pS)LG(pS)R - 576.908447 - Charge:3

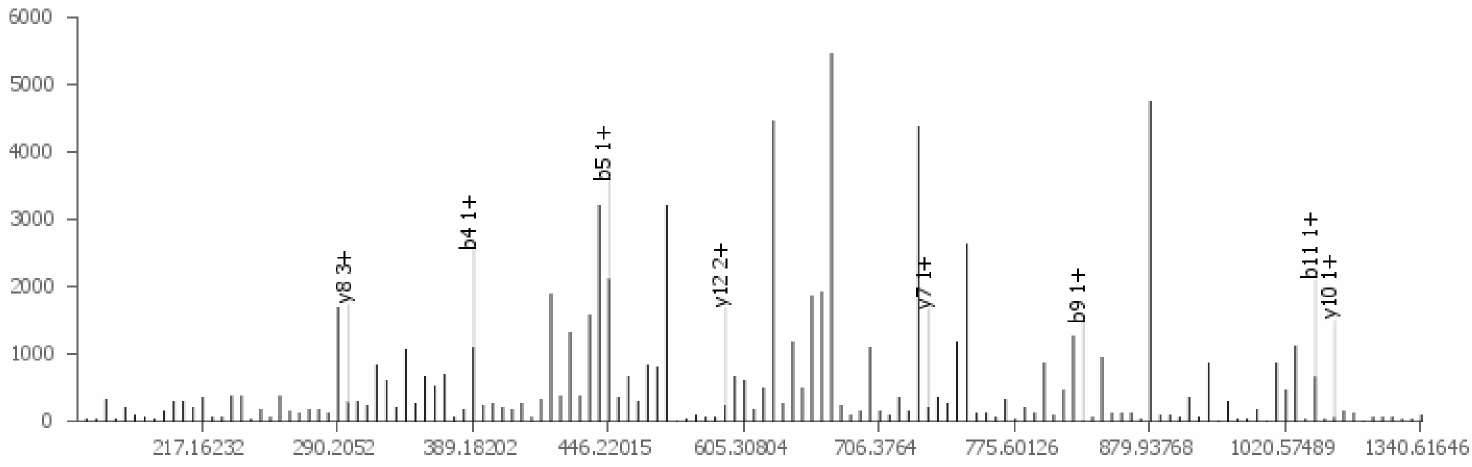

AT4G22750.1 - (t)NFEQVFG(s)DKMYWFVPLY(pT)EDDK - 1040.426351 - Charge:3

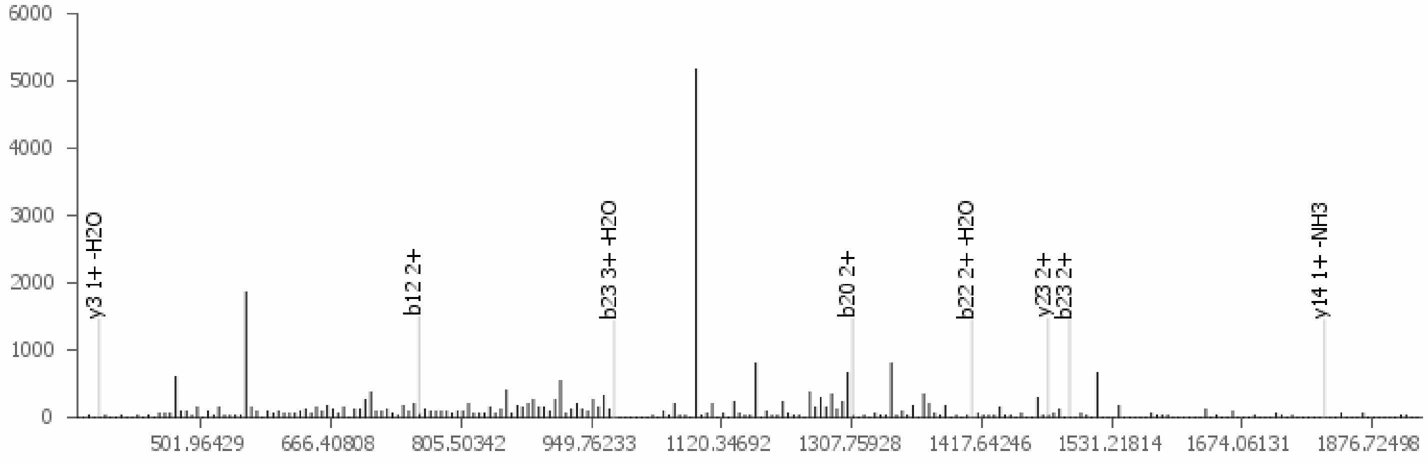

AT1G04120.1 - (pS)(pS)FISCILGEIPKI(pS)GEVR - 1166.499468 - Charge:2

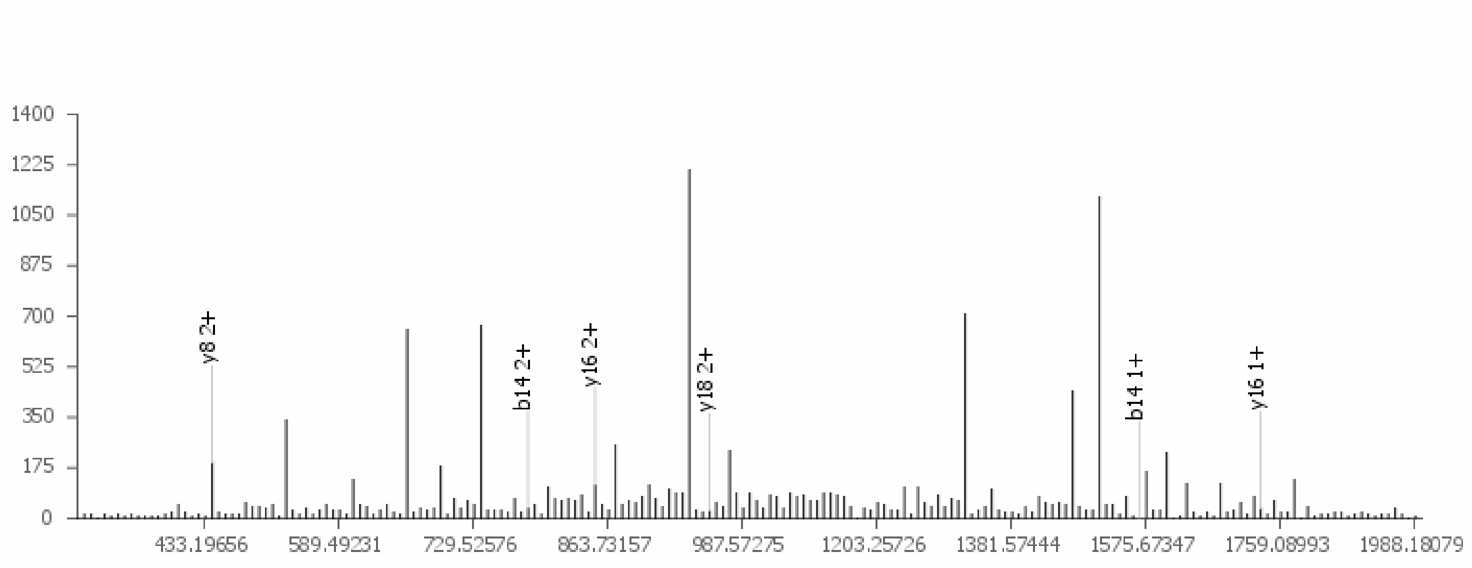

AT2G19580.1 - LEGF(pS)NWLK - 587.273876 - Charge:2

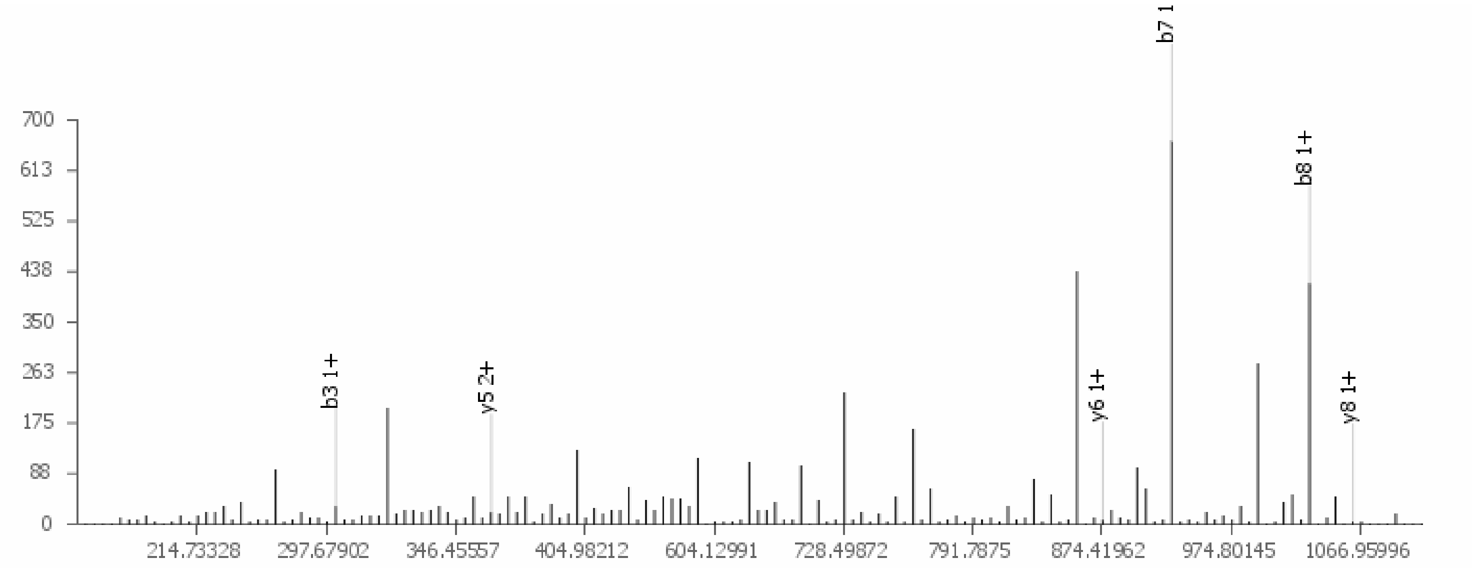

AT3G55980.1 - (pS)NLC(pS)SRTLTEIESR - 956.897925 - Charge:2

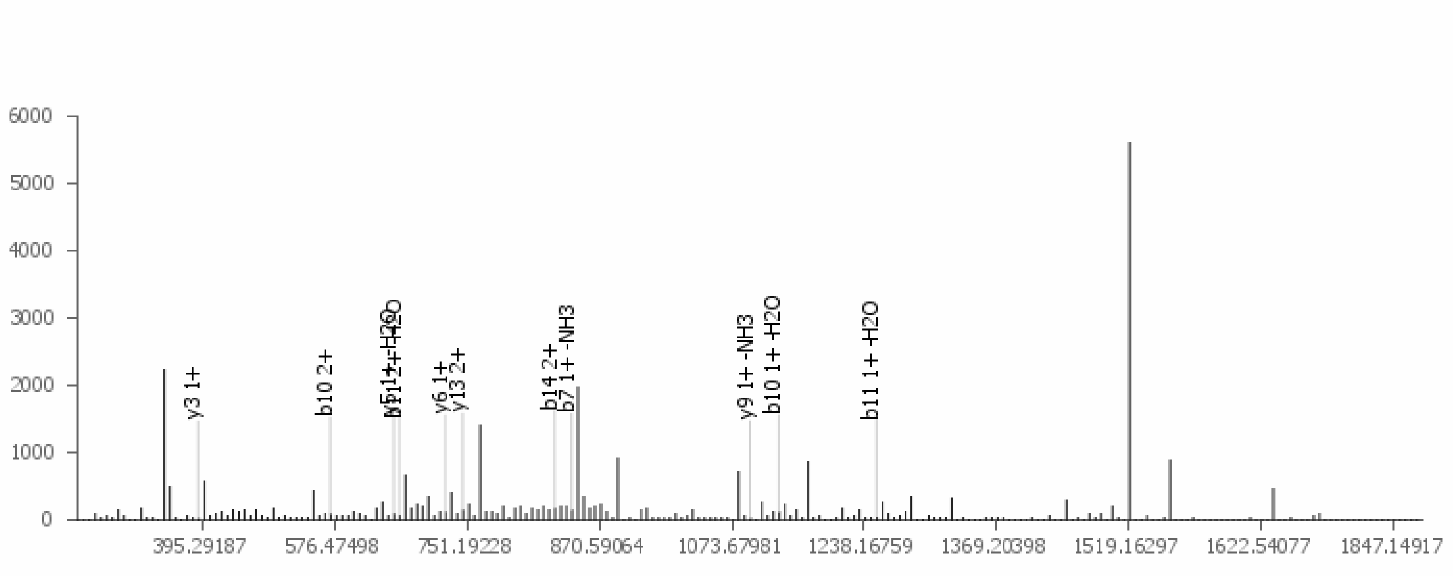

AT1G63210.1 - FQNI(pT)AEQATV(pY)LSDK - 994.430448 - Charge:2

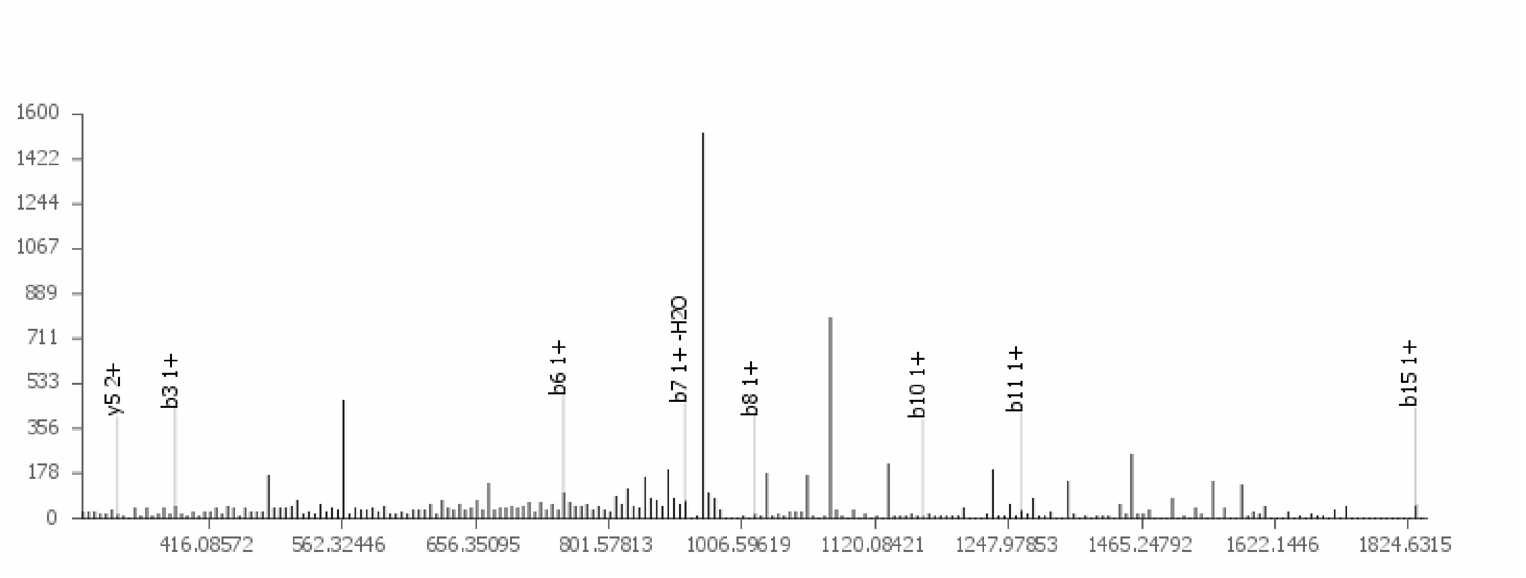

AT1G36925.1 - FPC(pT)ATSR - 510.199961 - Charge:2

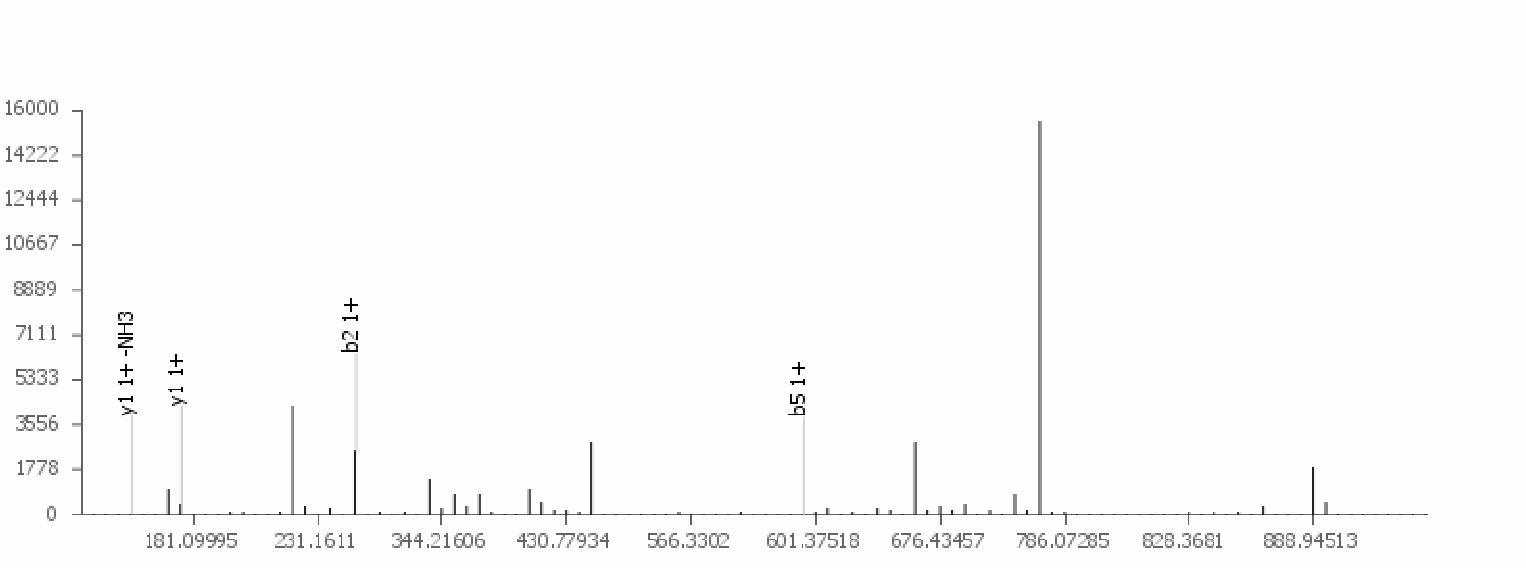

AT4G26030.1 - INKDQTGGQ(pS)CR - 722.312941 - Charge:2

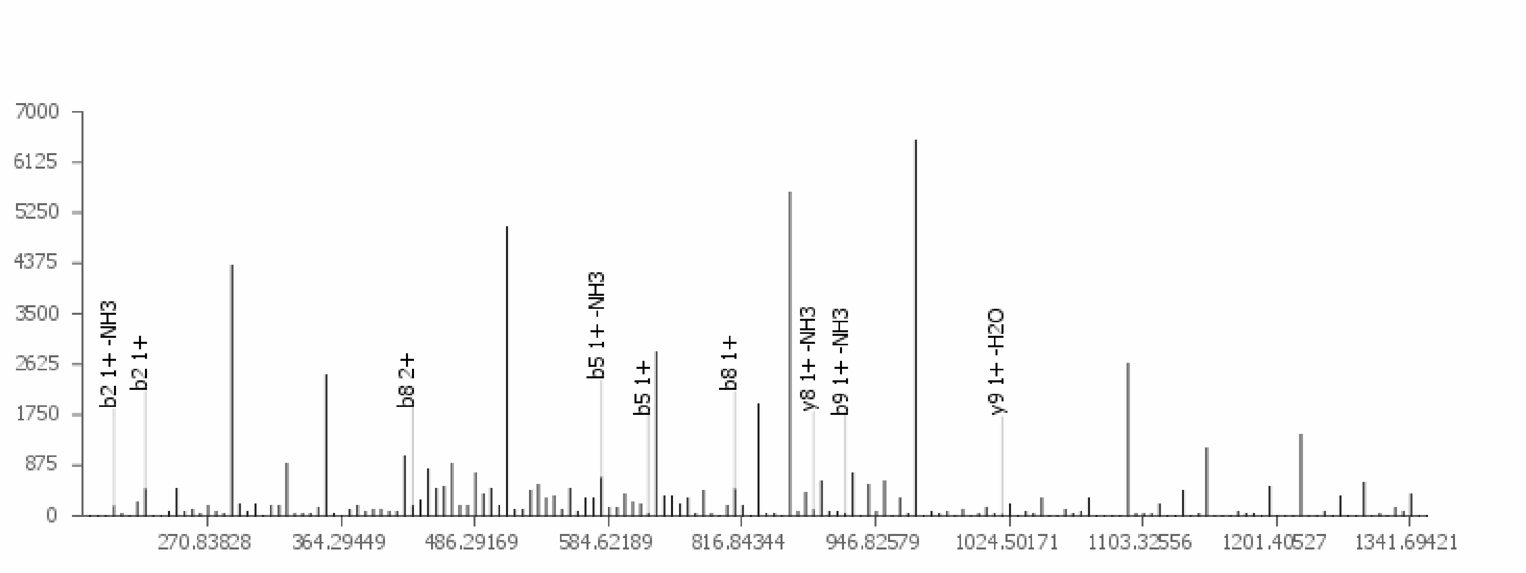

AT1G50980.1 - ILSIDN(pT)(pS)GK - 604.249646 - Charge:2

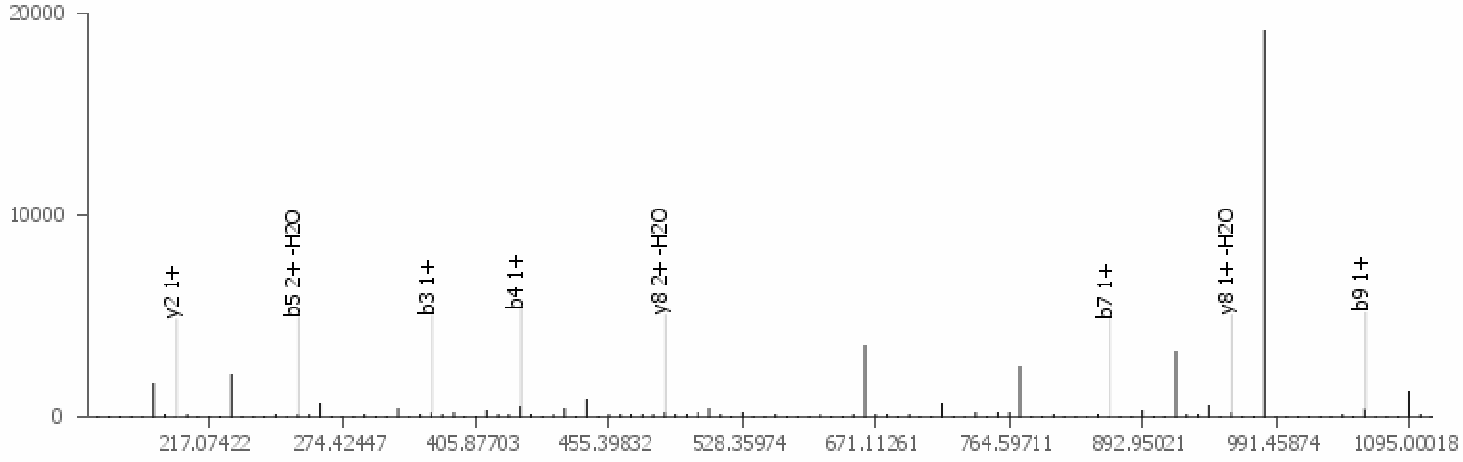

AT5G16680.1 - NT(pS)LP(pT)SNVLPR - 729.816556 - Charge:2

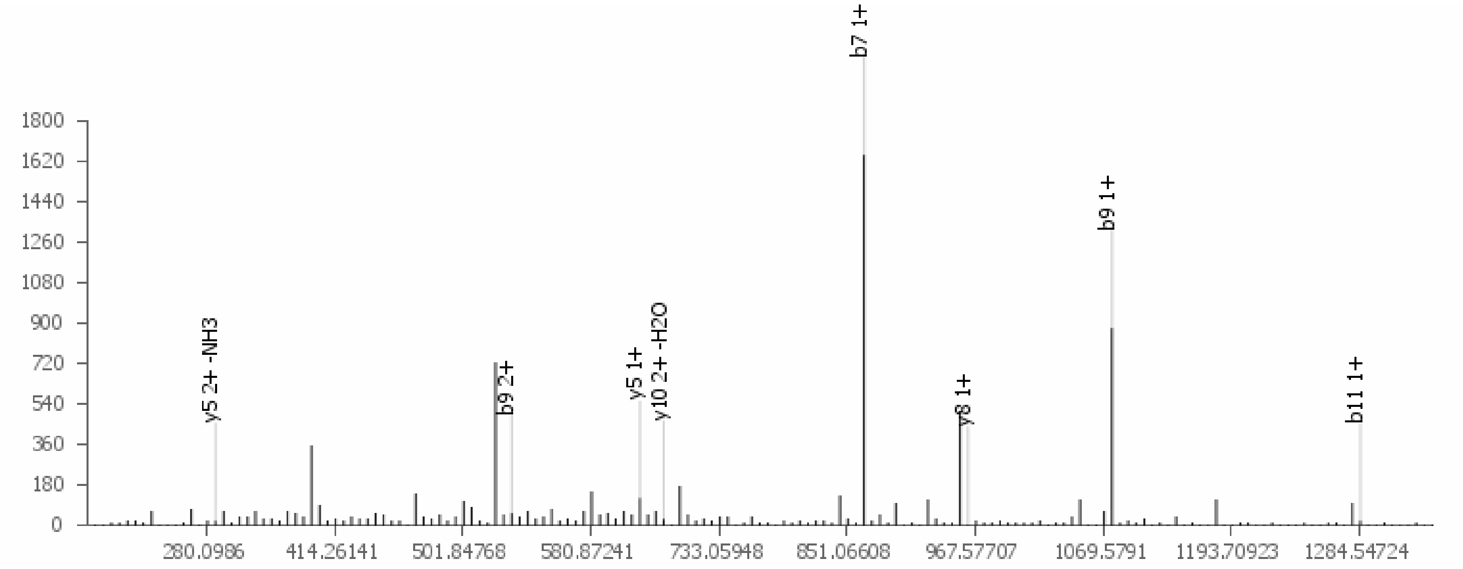

AT2G39350.1 - DGEIMAVLGA(s)G(s)GK - 736.324741 - Charge:2

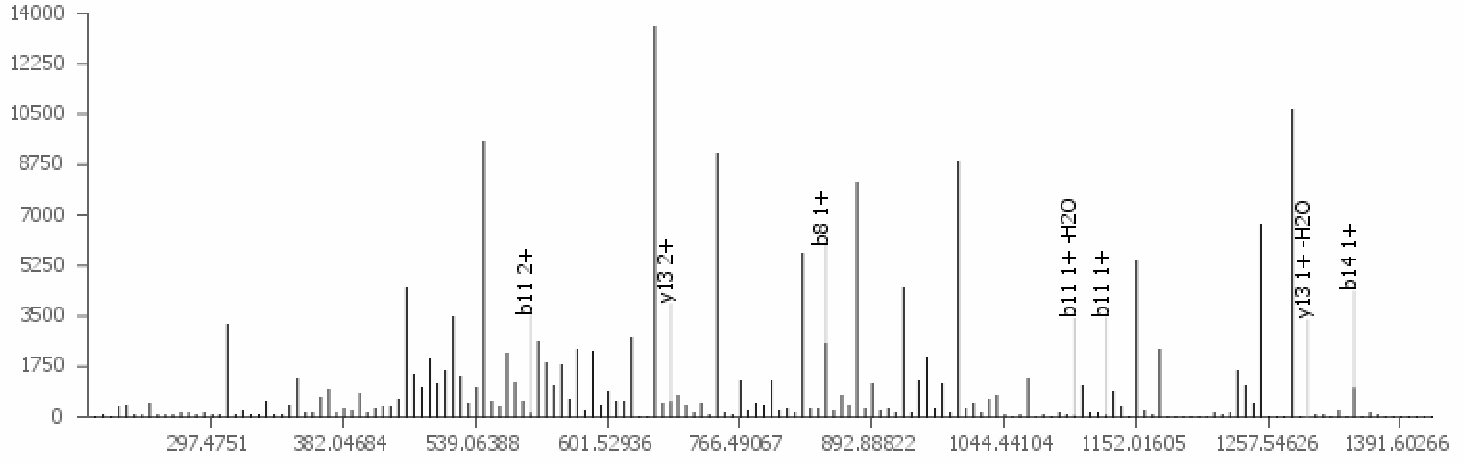

AT4G20760.1 - MRV(s)GLA(t)(s)(s)S(pS)SVPNWK - 1067.419096 - Charge:2

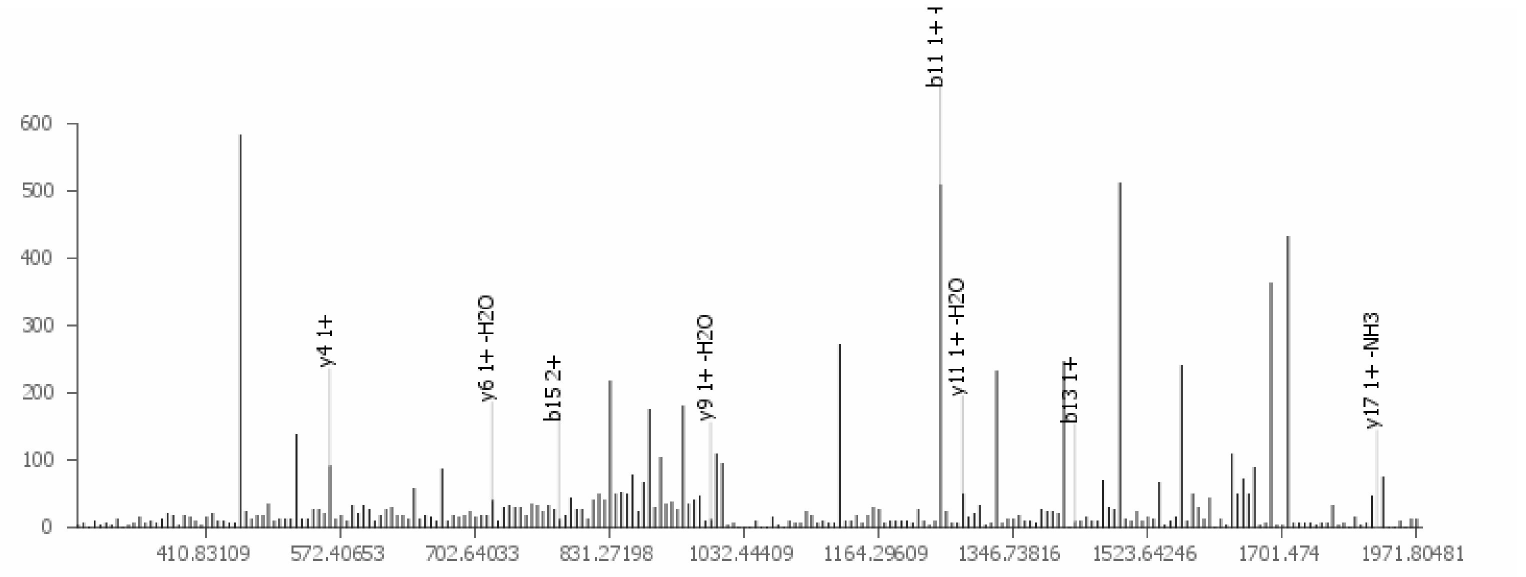

AT2G37500.1 - (oxM)H(s)C(s)H(t)HFVSFK - 890.830671 - Charge:2

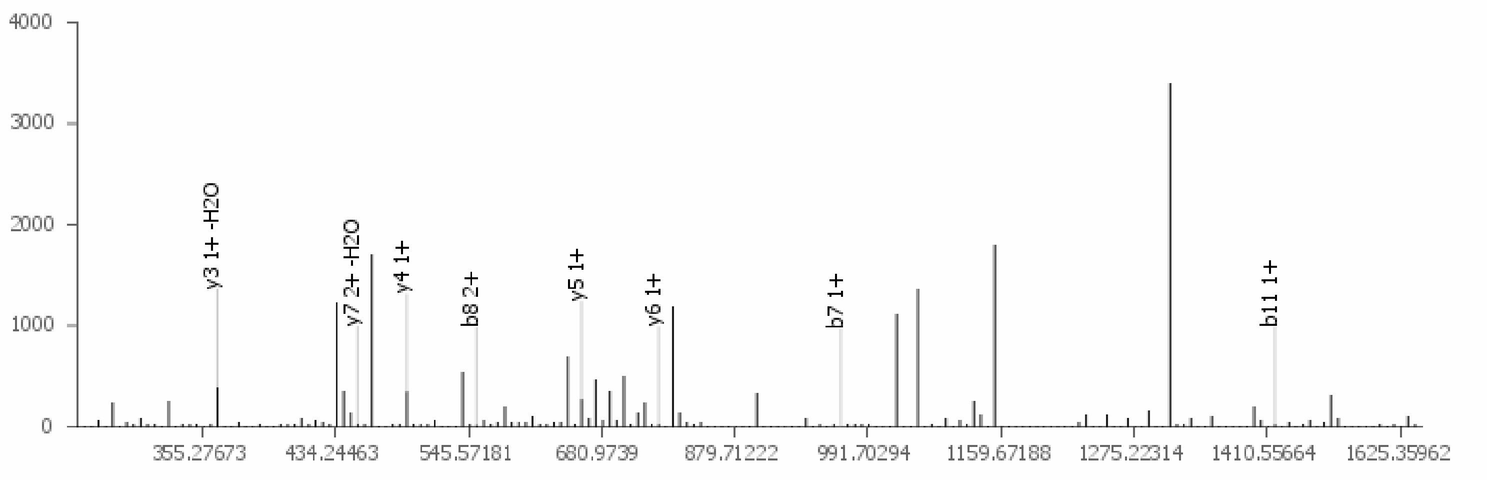

AT5G53150.1 - GQ(pS)EIFK - 444.69577 - Charge:2

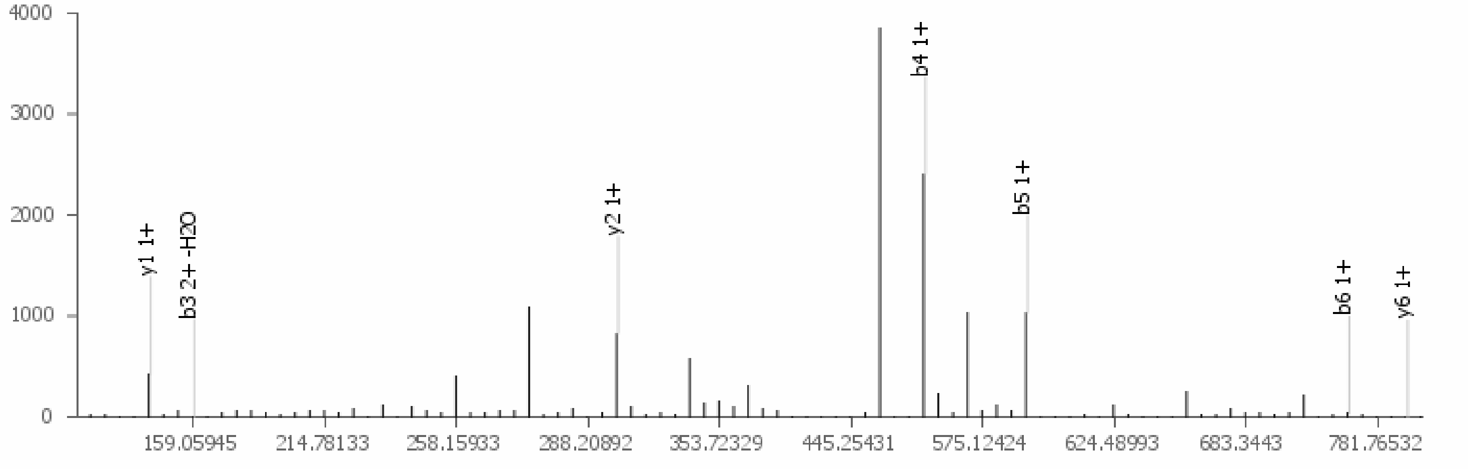

AT3G26840.1 - (pY)KIMGGVPVSHFNI(pY)K - 671.640225 - Charge:3

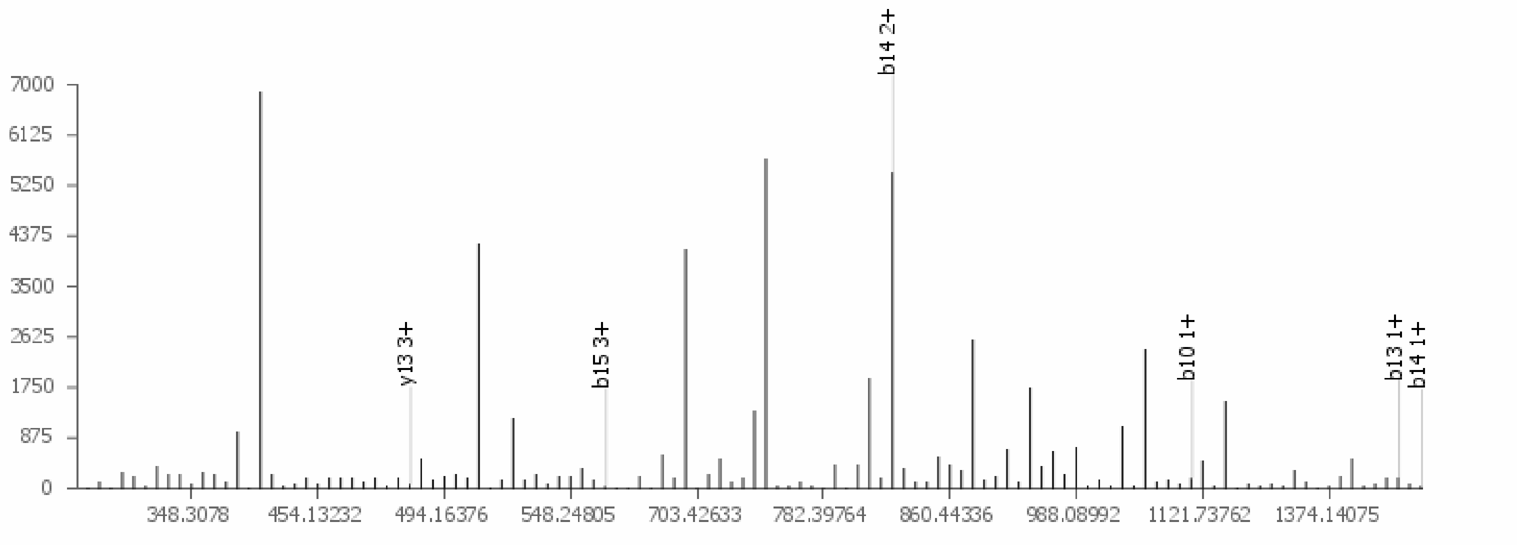

AT4G13130.1 - EIN(pS)EERFYK - 697.800773 - Charge:2

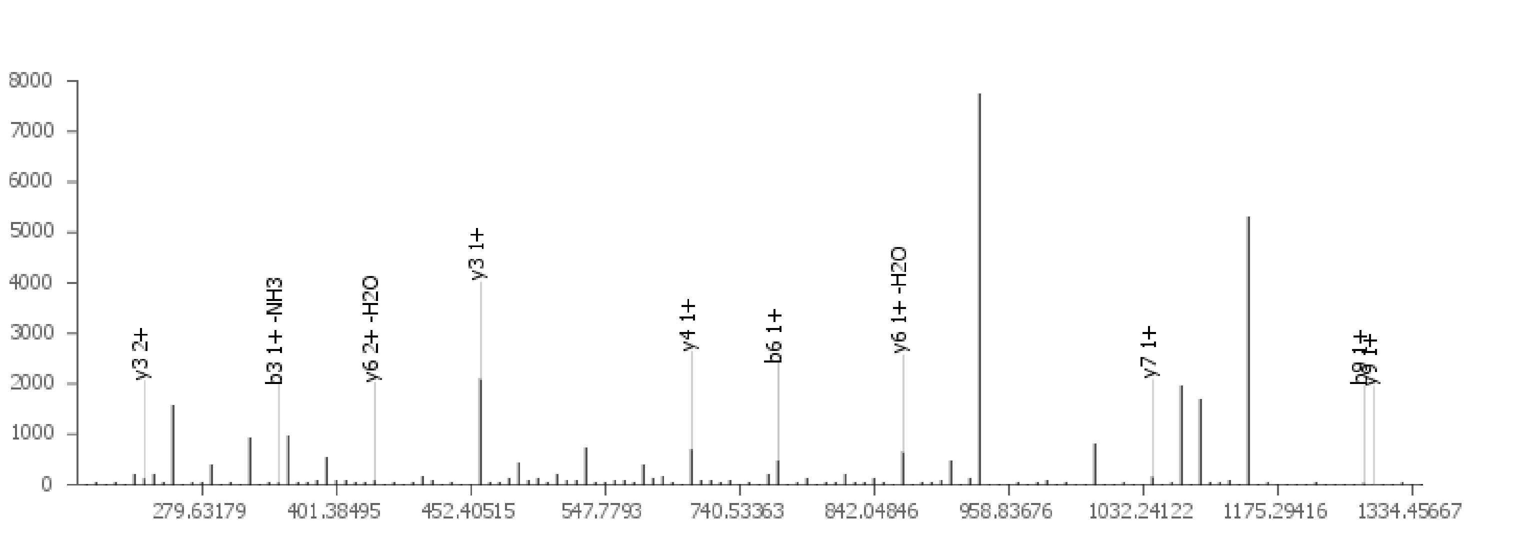

AT1G58390.1 - D(pY)E(pS)DFVGLEVNVKK - 951.401946 - Charge:2

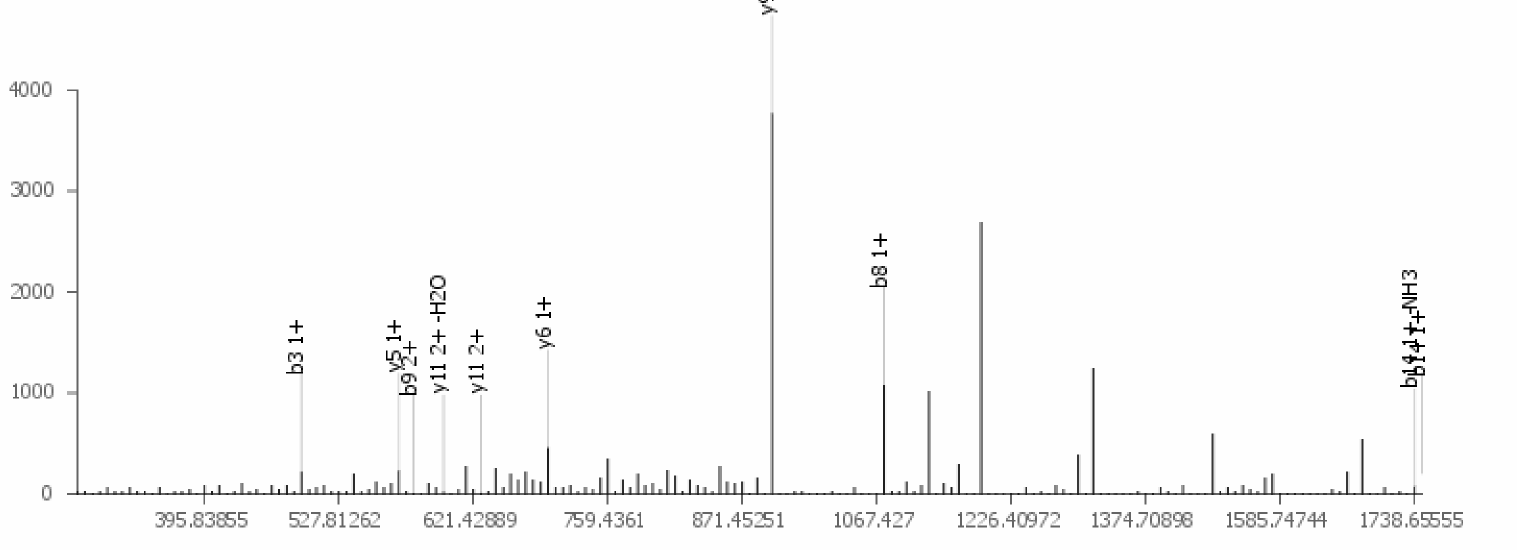

AT1G11060.1 - ED(pY)PQNGGVVR - 657.286226 - Charge:2

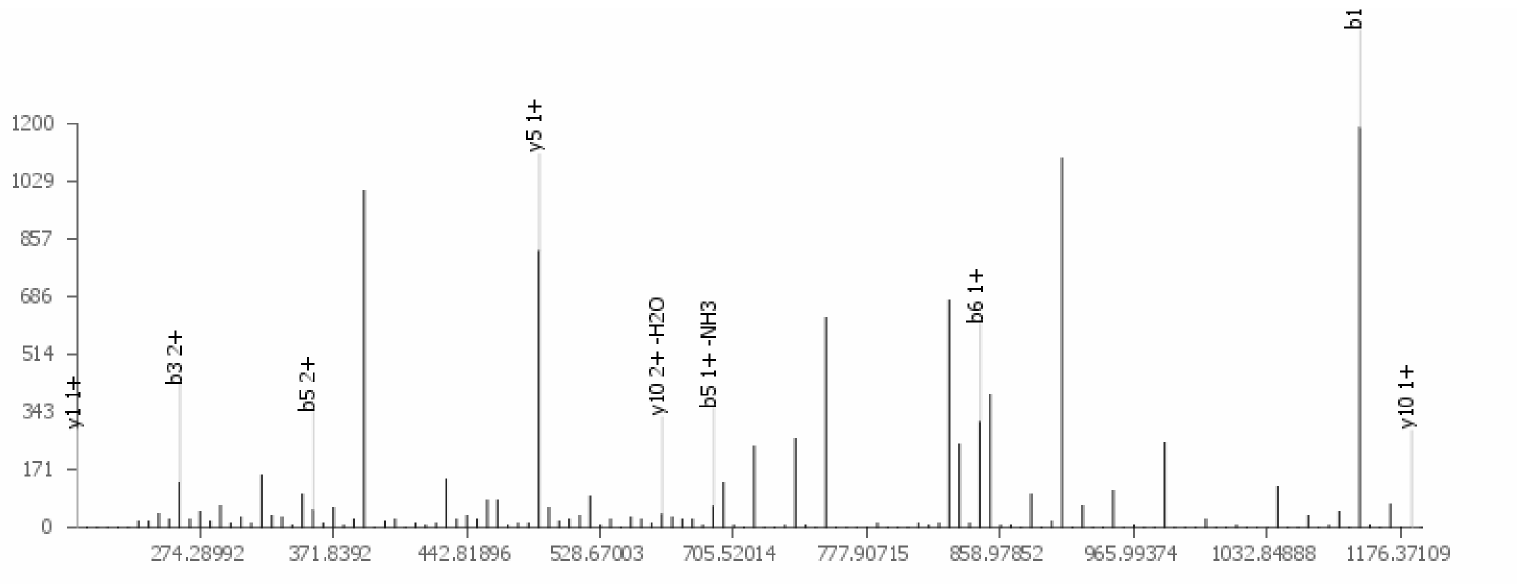

AT1G78900.1 - (oxM)PAF(pY)GGKL(pT)TFEDDEK - 1062.924166 - Charge:2

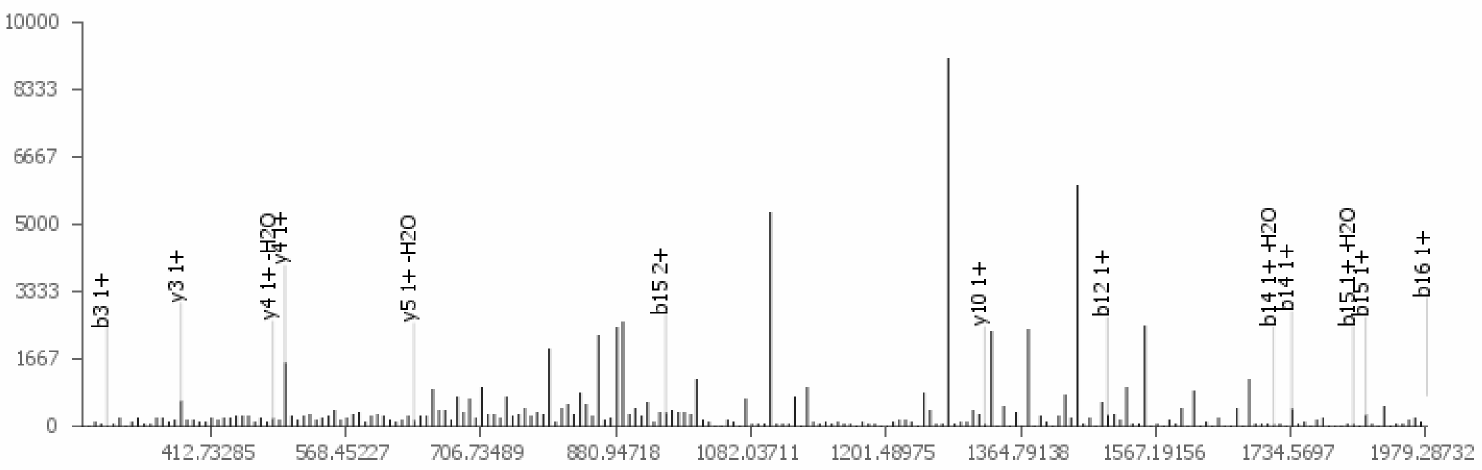

AT4G10730.1 - (oxM)KEL(pT)EELEVEK - 787.356992 - Charge:2

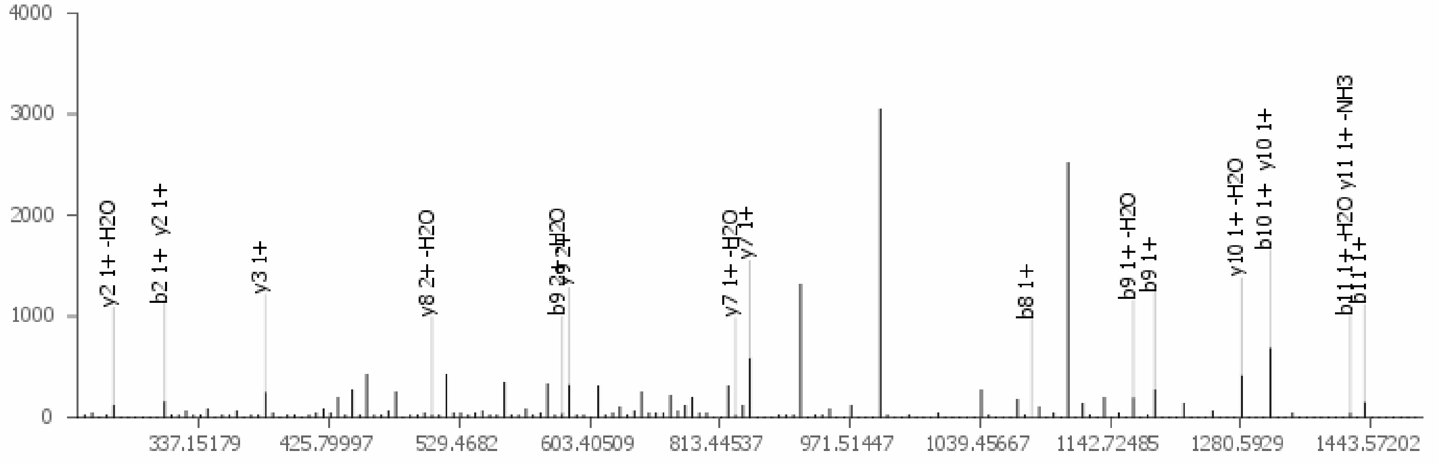

AT5G42950.1 - TGMGTGDPNQ(pY)GNHSDVVR - 1042.92957 - Charge:2

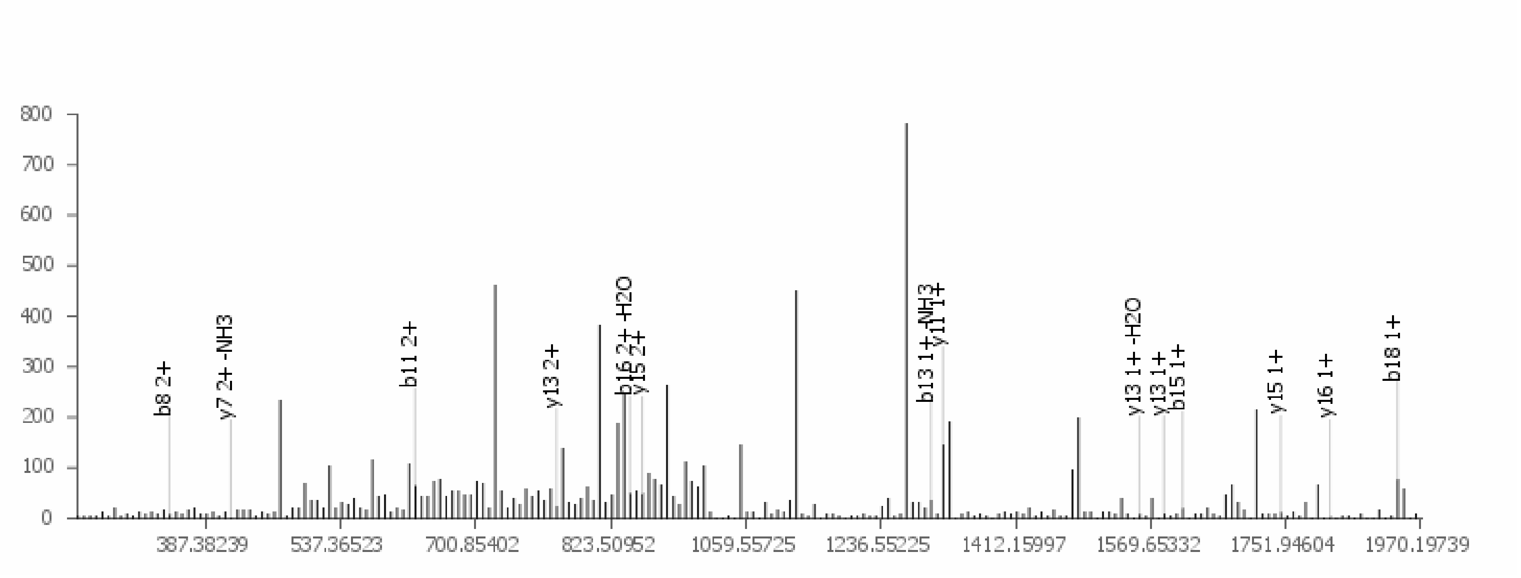

AT4G33240.1 - CAAN(pS)IPSPSDETK - 778.820339 - Charge:2

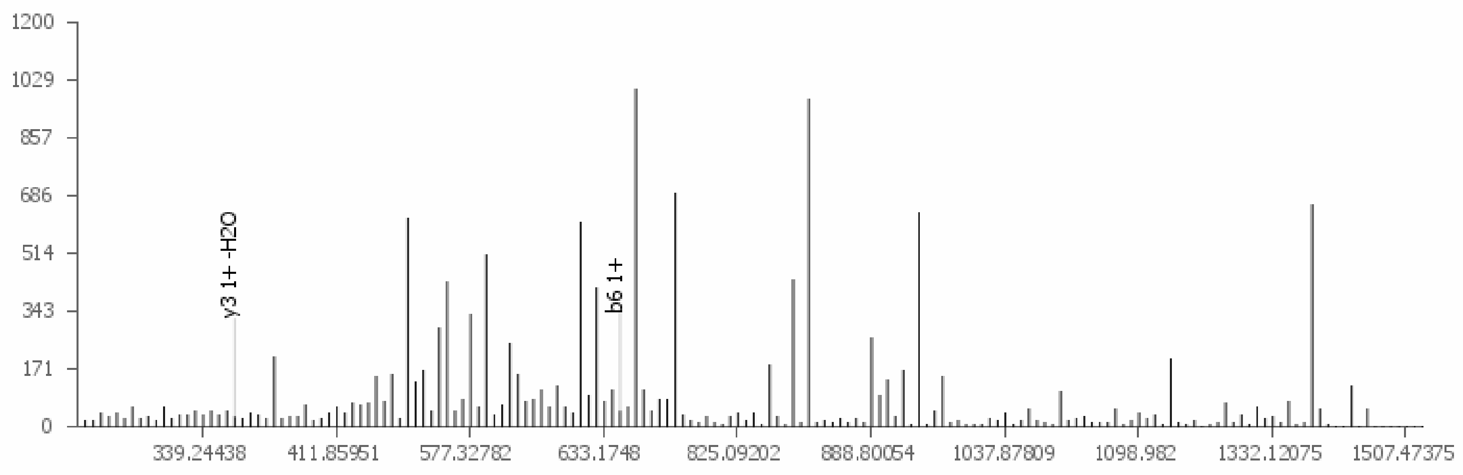

AT1G72645.1 - GFSDNAYIKNGV(pY)(s)L(s)ELS(oxM)SK - 1293.537772 - Charge:2

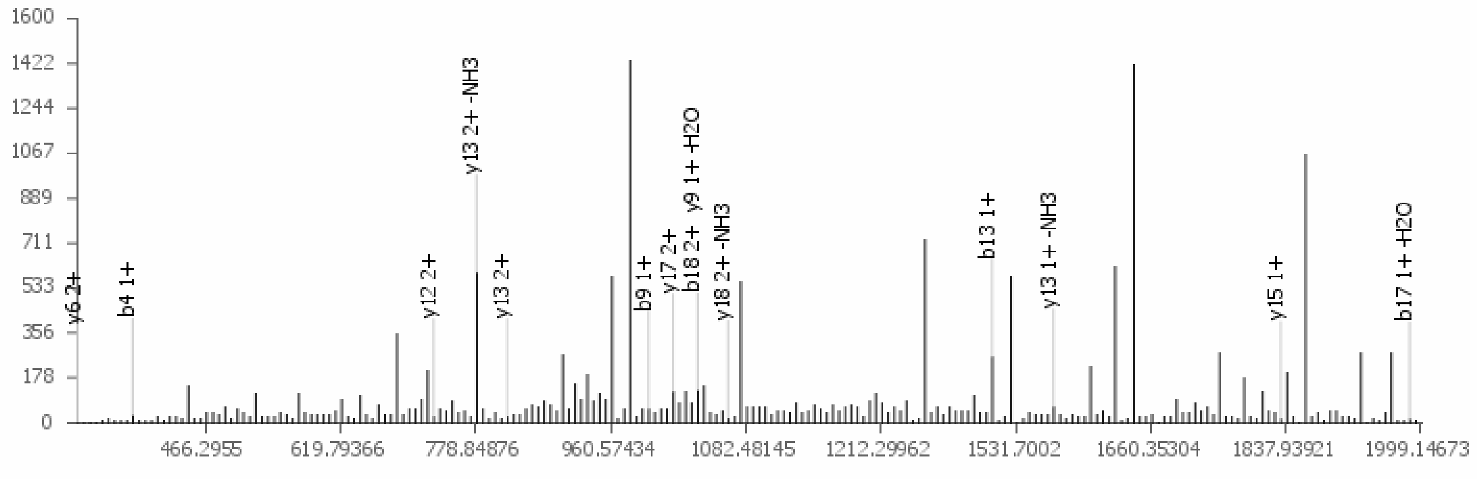

AT2G26610.1 - T(s)(s)SKDLR - 487.225776 - Charge:2

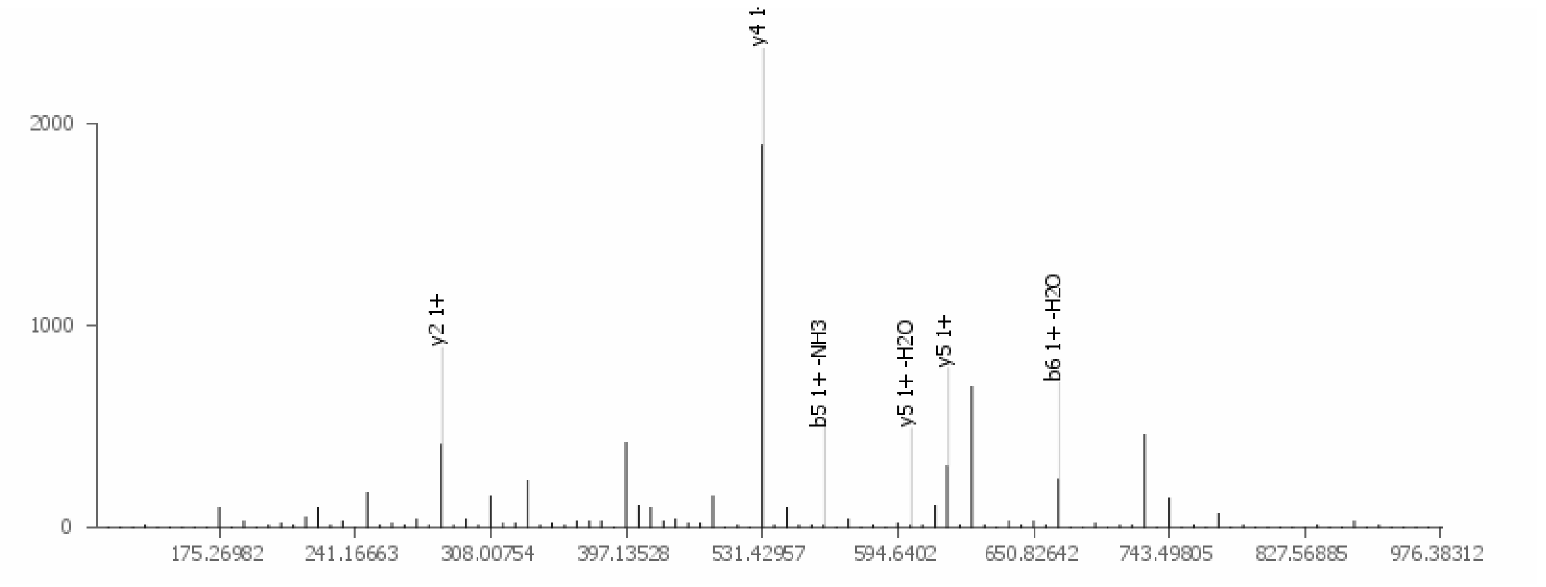

AT5G05630.1 - FS(t)(s)(s)HLPNLME - 761.79234 - Charge:2

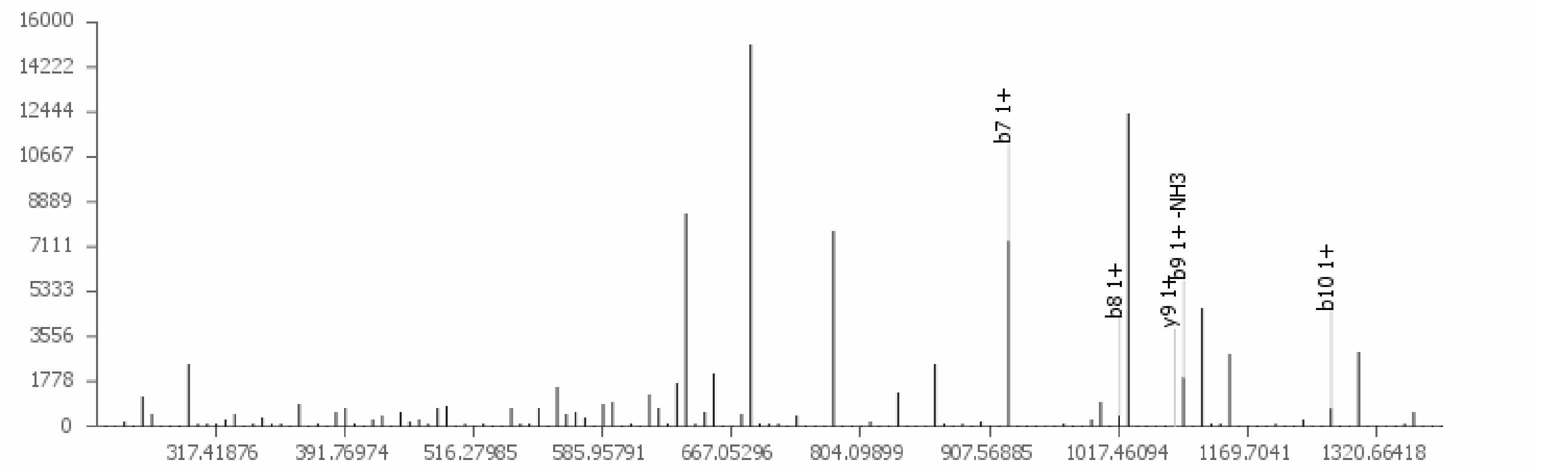

AT1G09100.1 - RFD(pS)EV(pS)GDR - 664.243922 - Charge:2

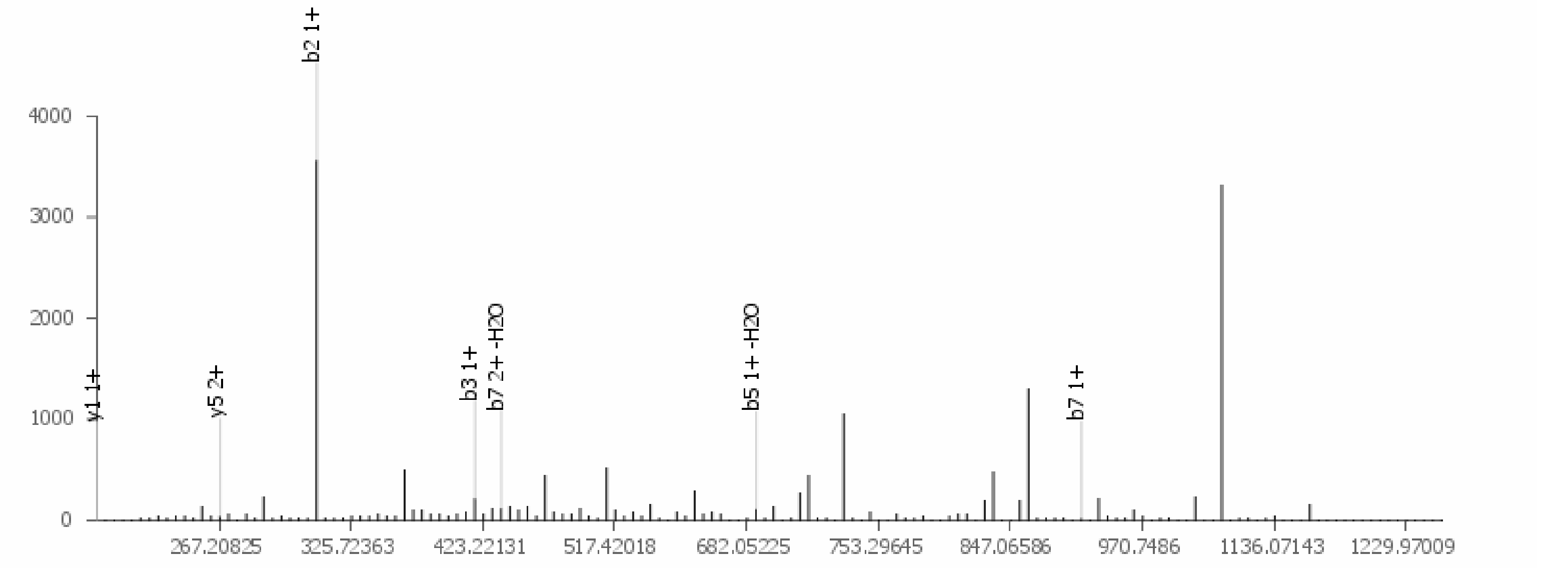

AT5G65030.1 - SSK(pS)EVVGR - 514.745527 - Charge:2

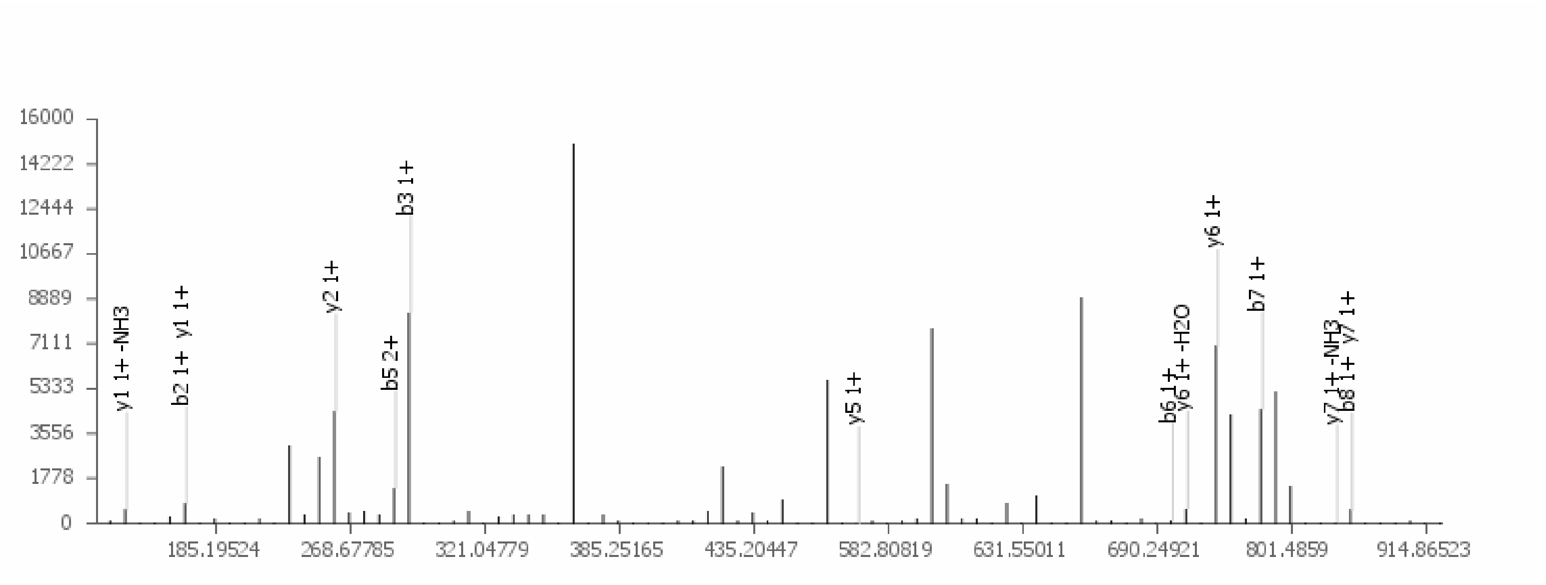

AT3G17330.1 - SNTQEATDD(pS)TPSTLK - 887.874774 - Charge:2

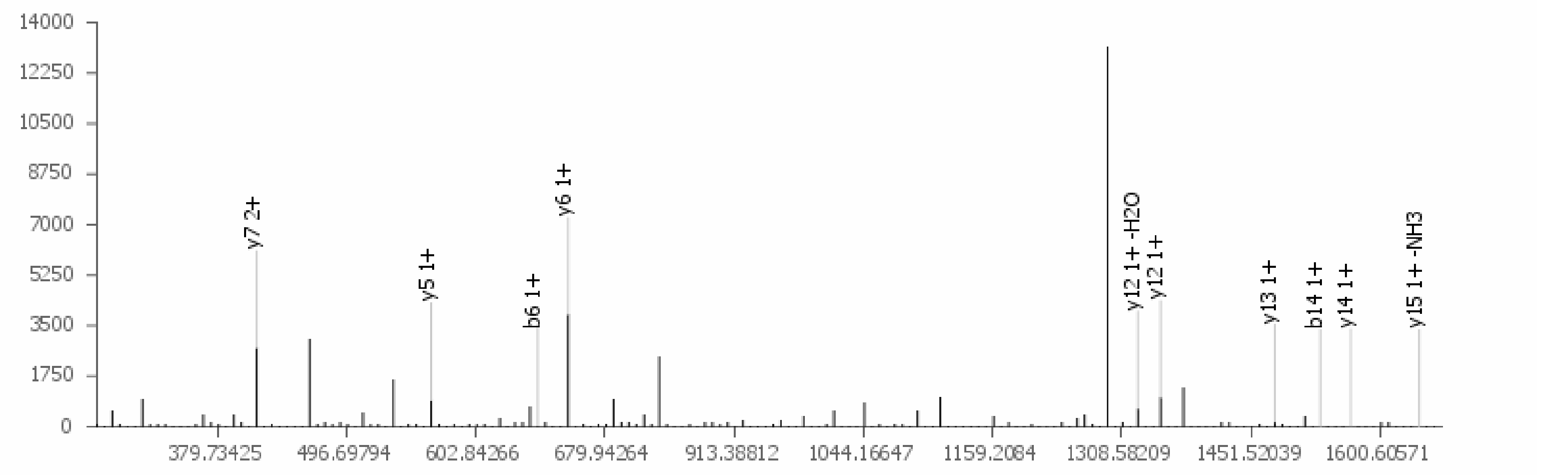

AT1G28090.1 - (pY)PQAPGSD(oxM)VFL(pS)R - 872.354513 - Charge:2

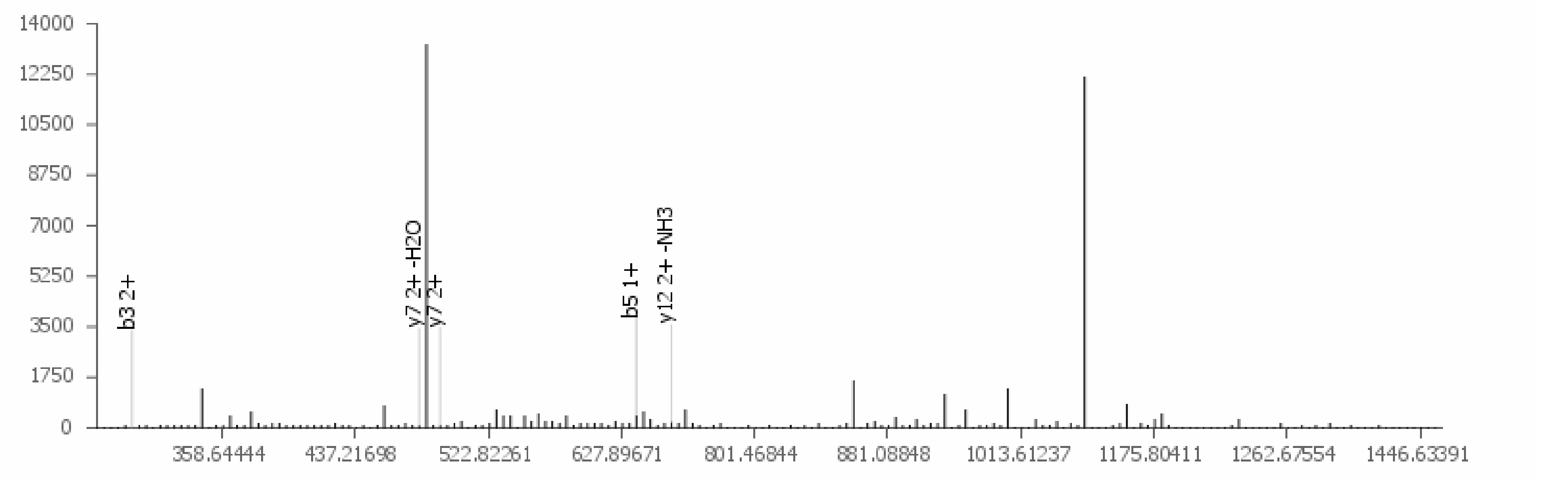

AT3G59410.1 - GQLKDHGSNADEDNELL(pS)EEI(pT)AL(pS)AIFQEDCK - 1306.200136 - Charg

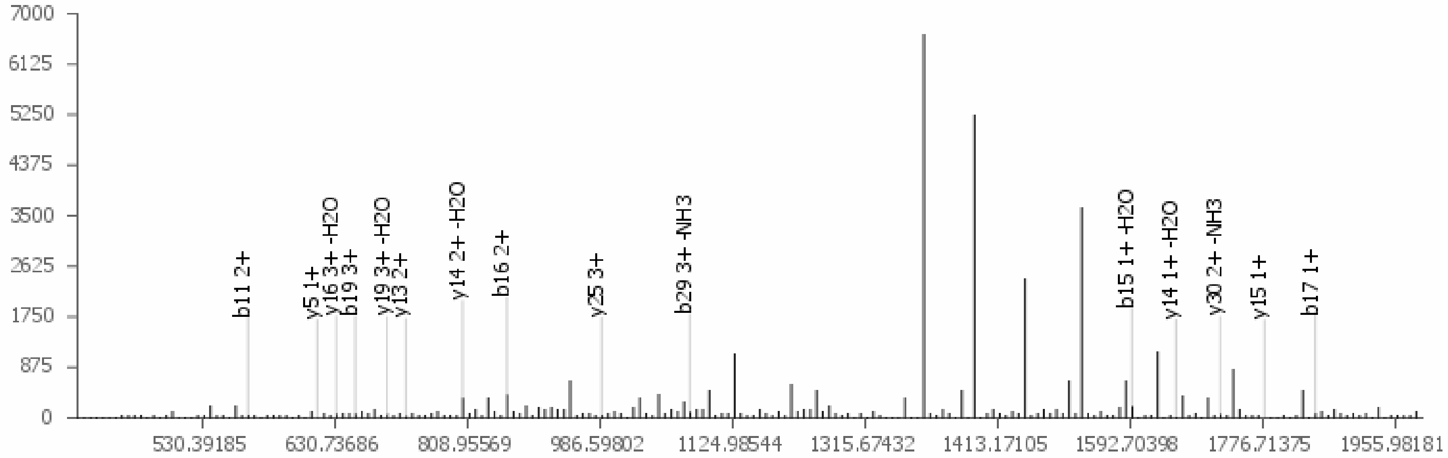

AT5G49050.1 - IKMASG(pS)ER - 529.733819 - Charge:2

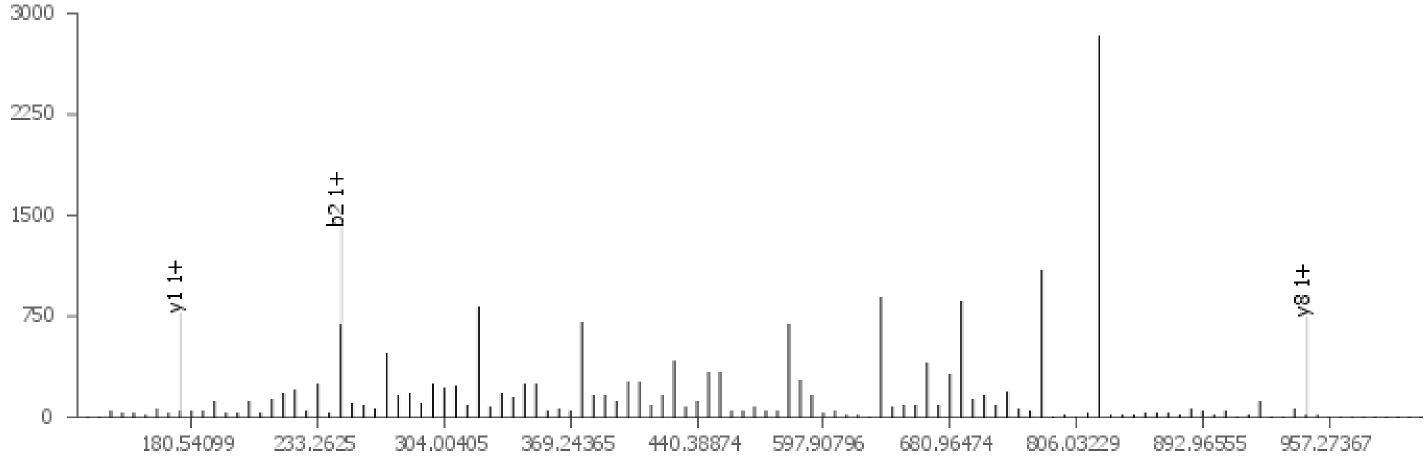

AT1G02380.1 - DENRVETS(pS)QSLDL(pS)EK - 1048.923086 - Charge:2

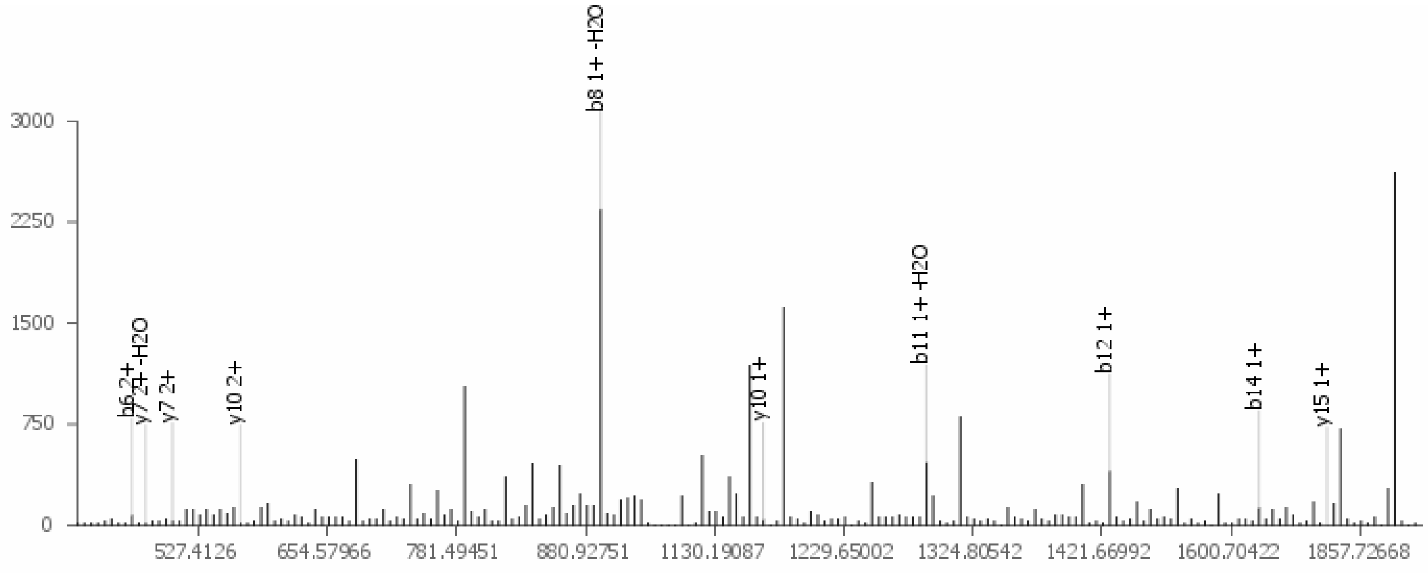

AT4G36550.1 - M(pS)KGGYF(pT)P(oxM)QR - 789.79257 - Charge:2

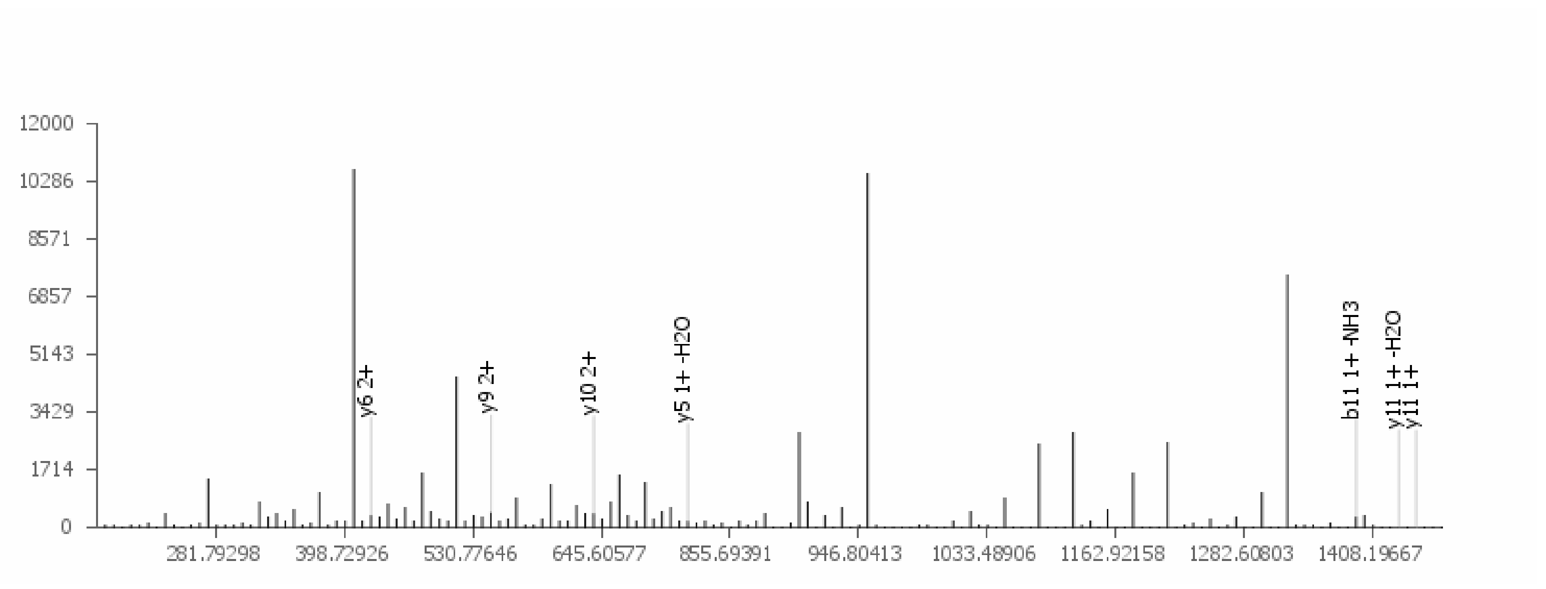

AT1G19410.1 - MEKIN(pT)DDLLVK - 749.863045 - Charge:2

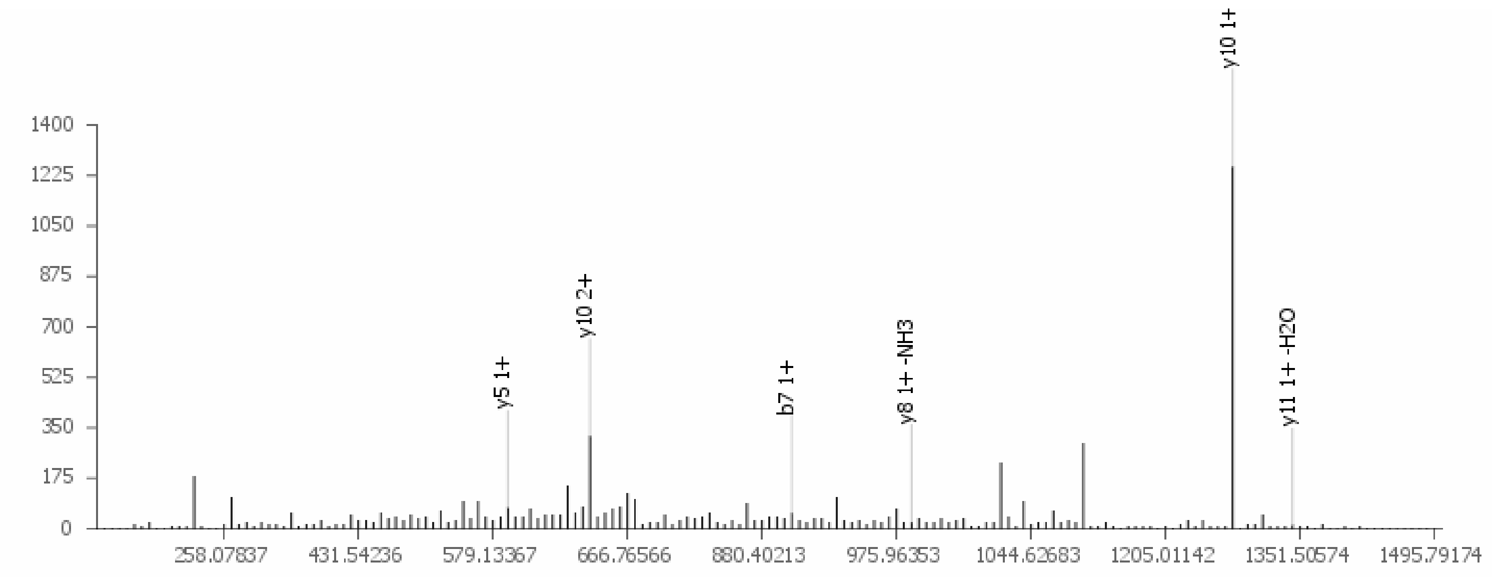

AT3G26350.1 - KTSMFANVHLV(s)(s)QVQLQATQSR - 1320.657026 - Charge:2

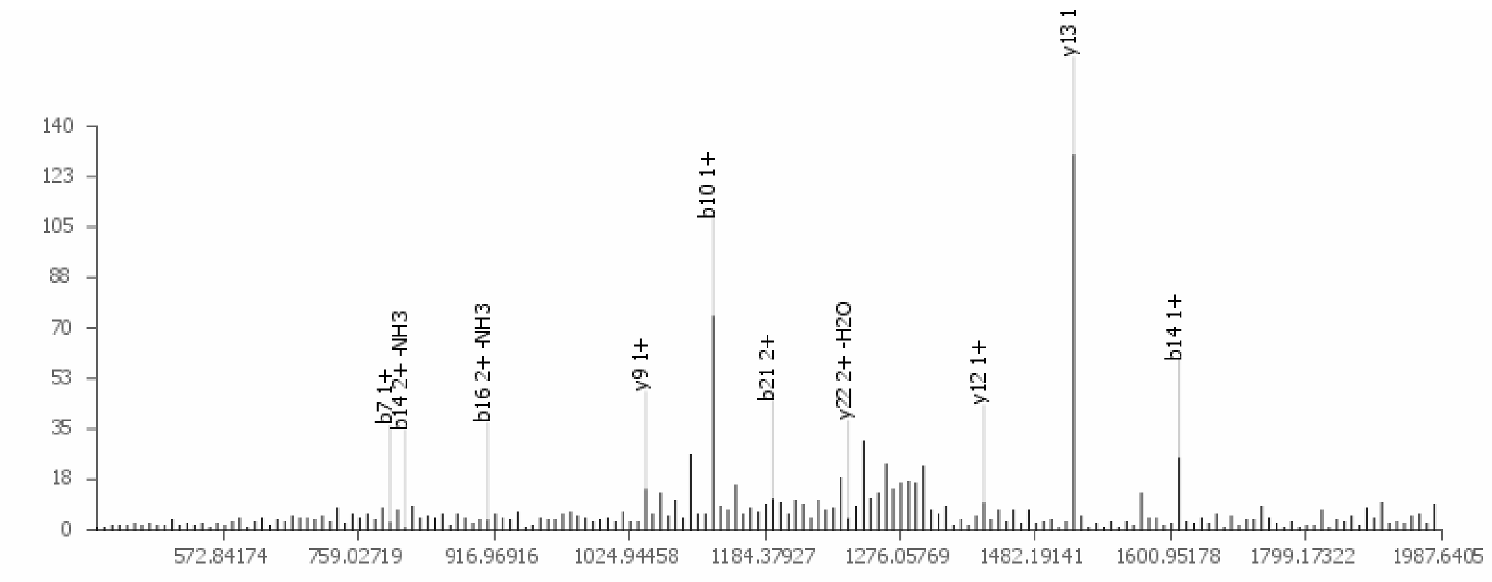

AT2G40820.1 - GLTFLLTFE(pS)EQER - 875.415294 - Charge:2

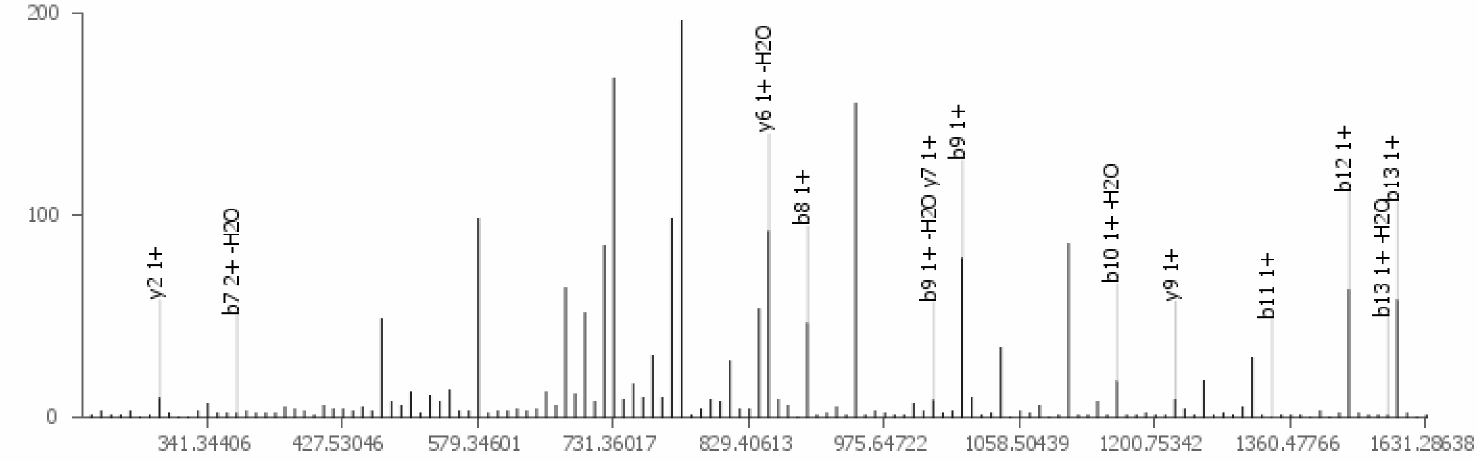

AT4G30960.1 - LLGHG(pT)FAKVYHAR - 825.41522 - Charge:2

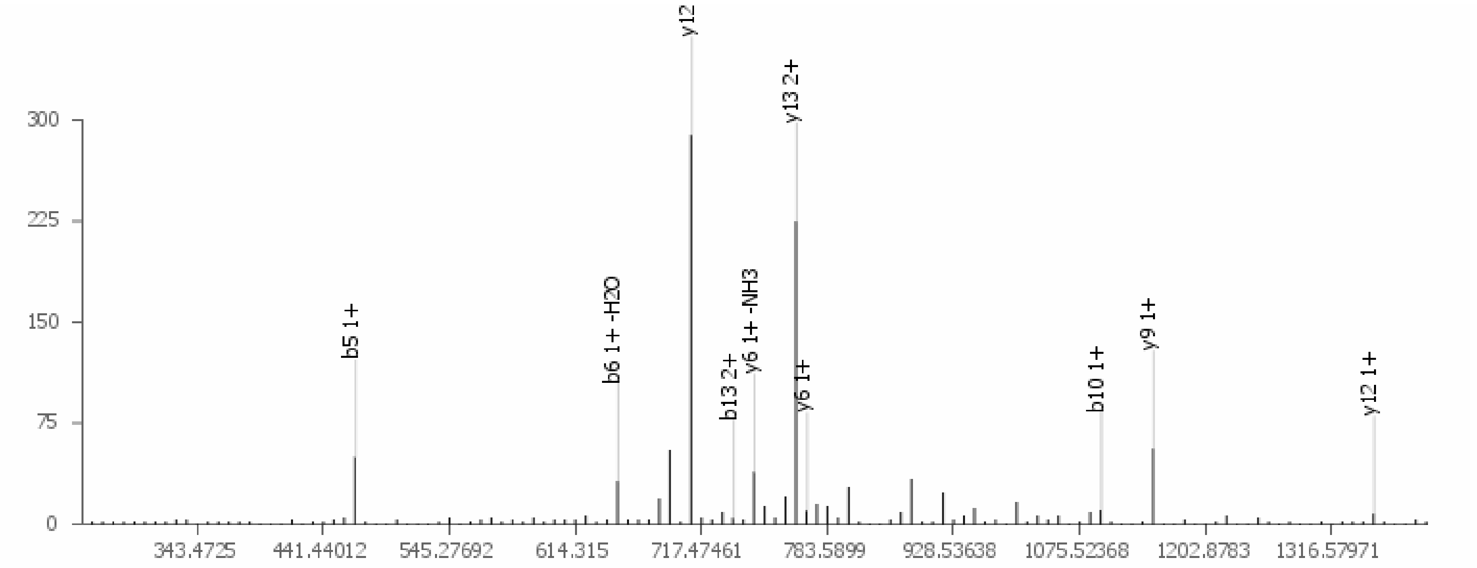

AT5G43630.1 - NSGLK(pS)LWISR - 670.834997 - Charge:2

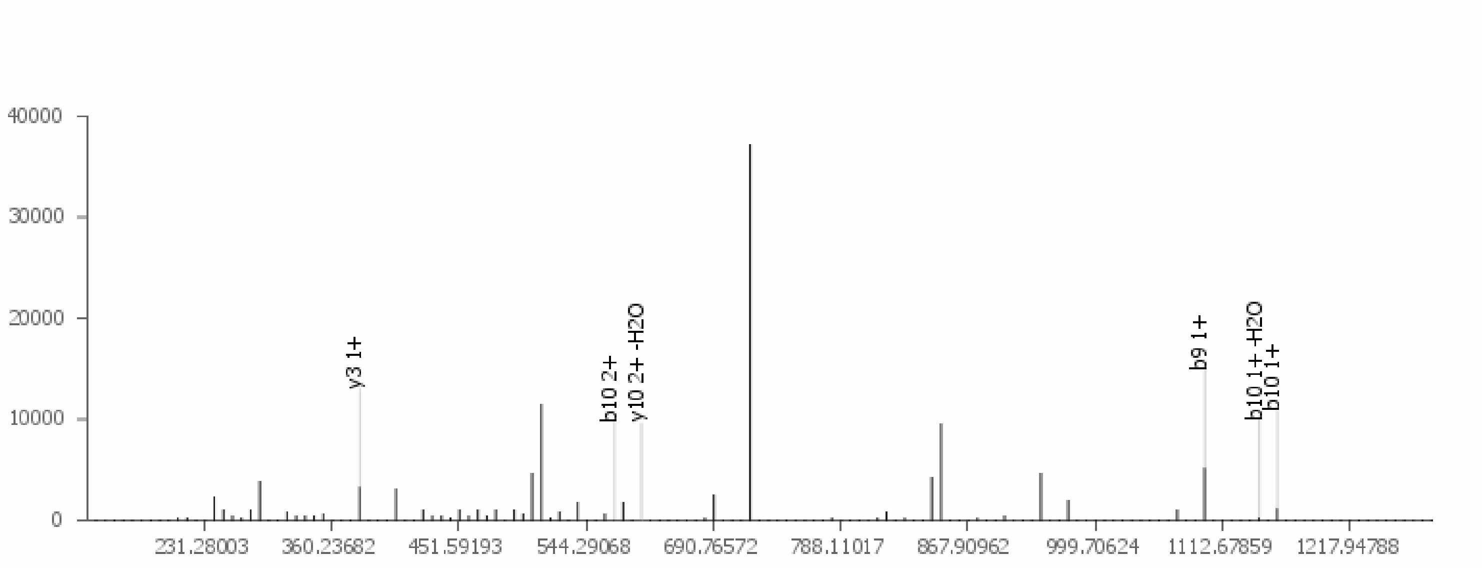

AT4G35080.1 - (t)(s)PFLPR - 897.426933 - Charge:1

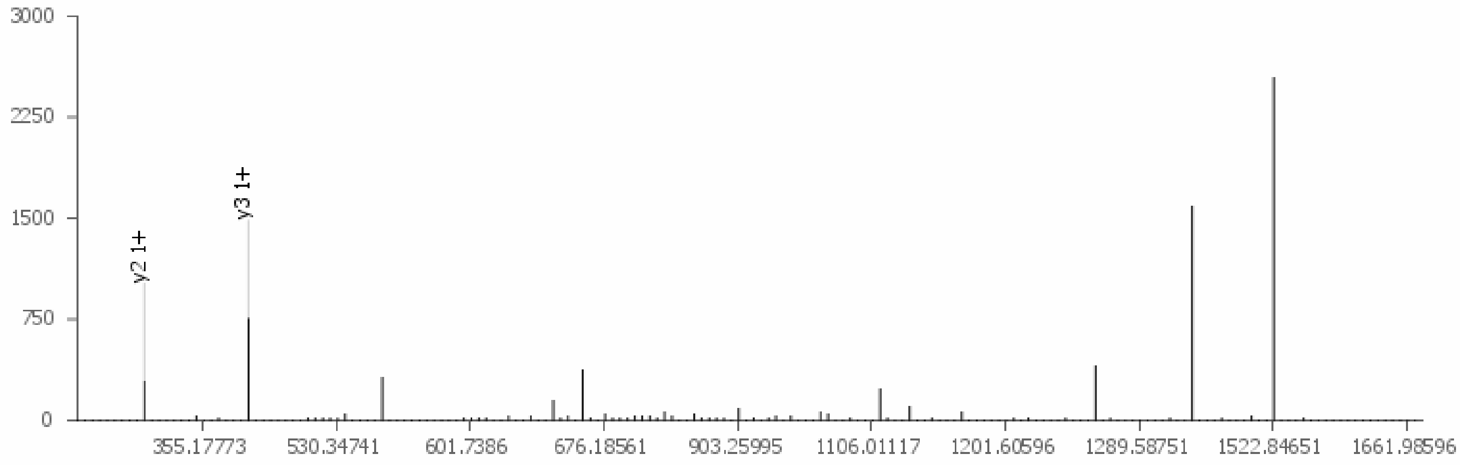

AT5G10610.1 - WIG(pY)KGLEK - 587.283823 - Charge:2

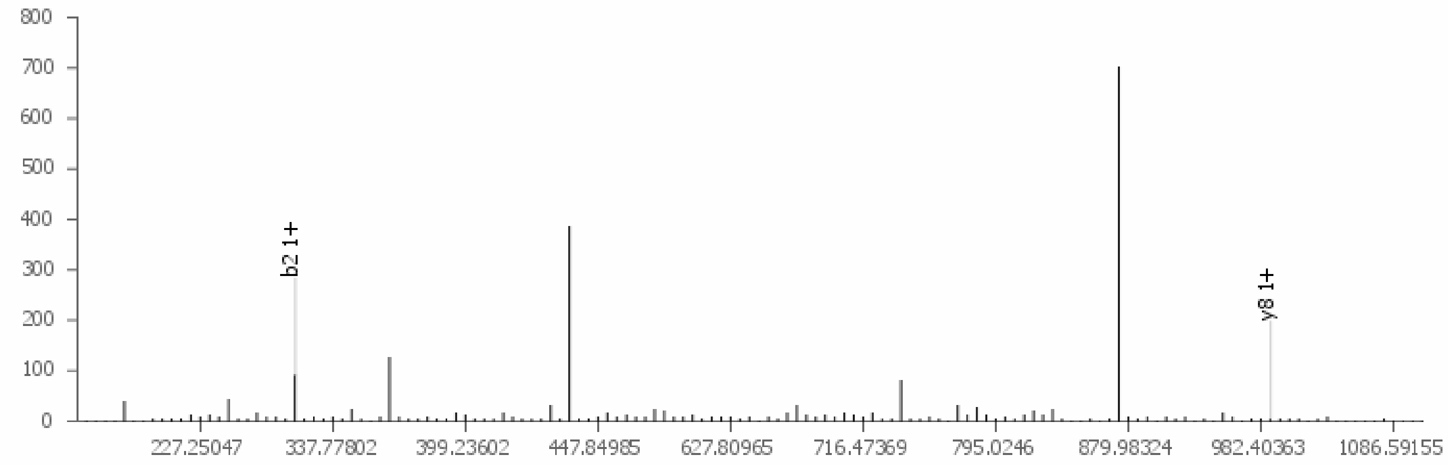

AT4G38350.1 - NLSL(s)F(s)(s)ESSIEEELKR - 1068.007666 - Charge:2

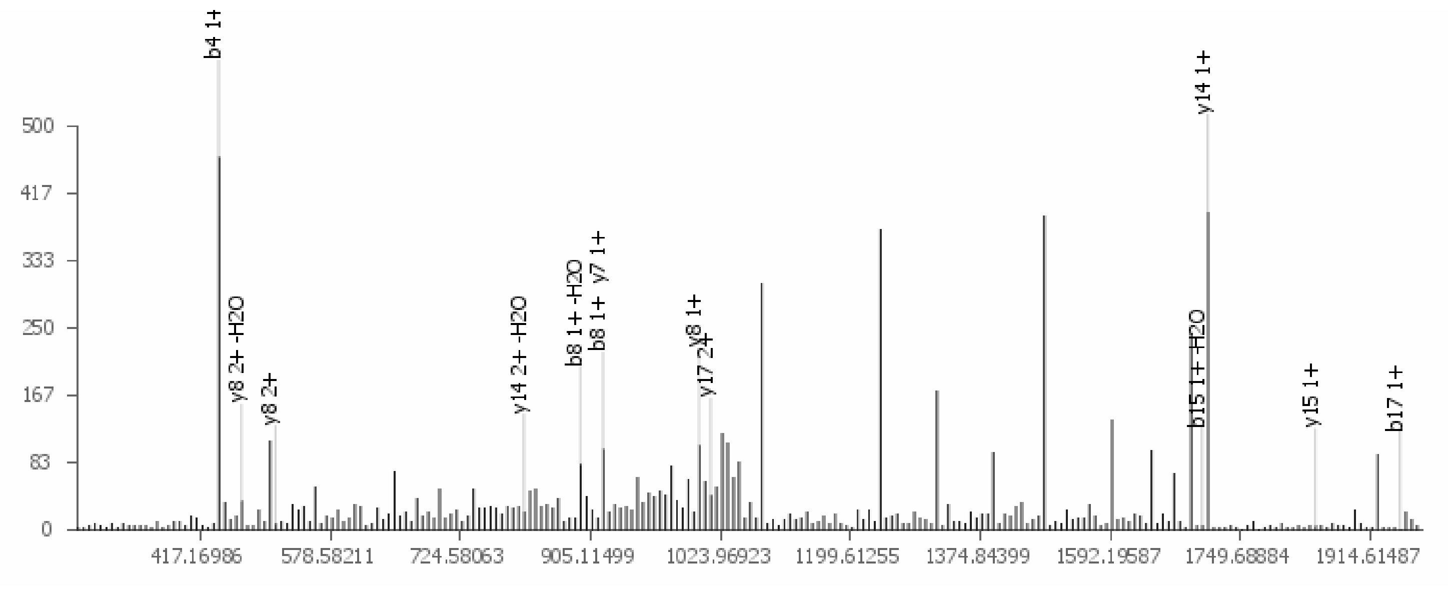

AT1G28580.1 - SII(pS)FGDSIADTGNLLGLSDPK - 1150.561027 - Charge:2

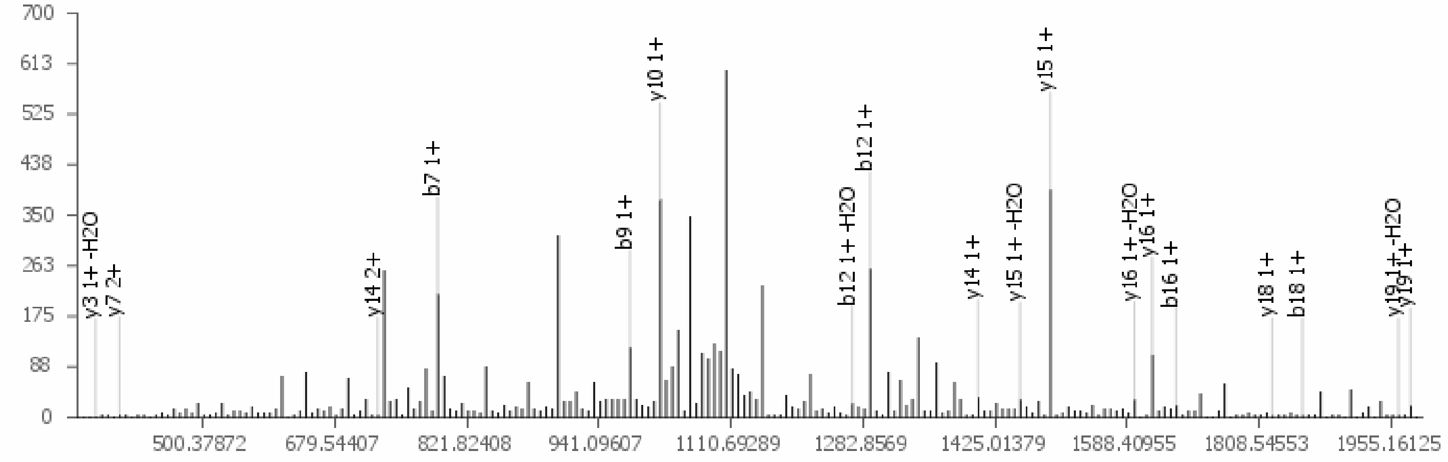

AT3G14550.1 - MATTVHL(pS)SFSLFIQSRGR - 1109.536148 - Charge:2

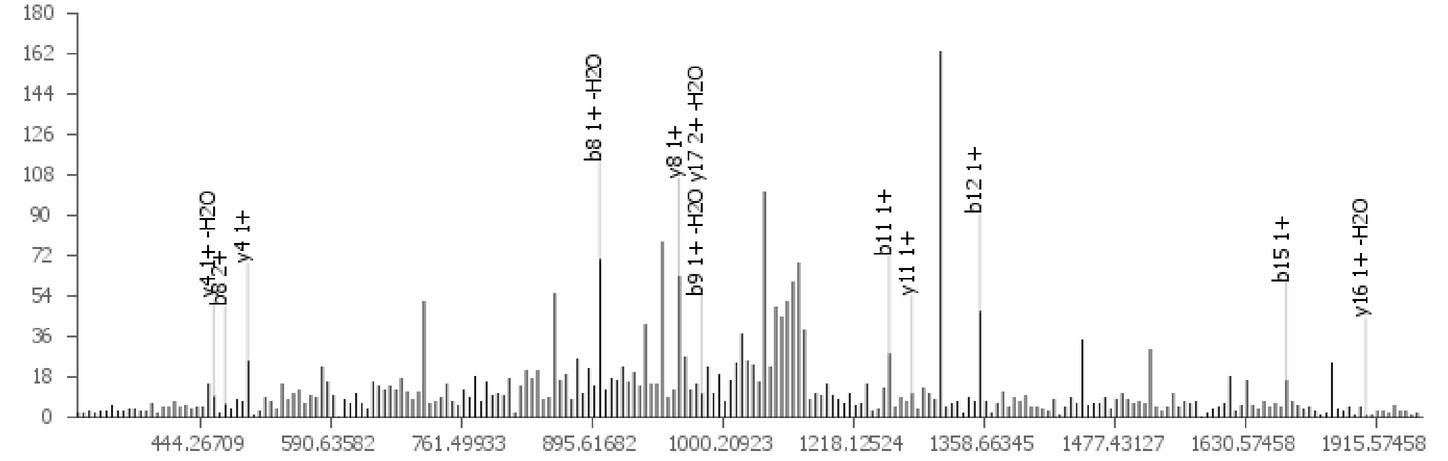

AT5G64010.1 - SDNVKLLLGE(t)(s)V(s)NGVK - 970.485386 - Charge:2

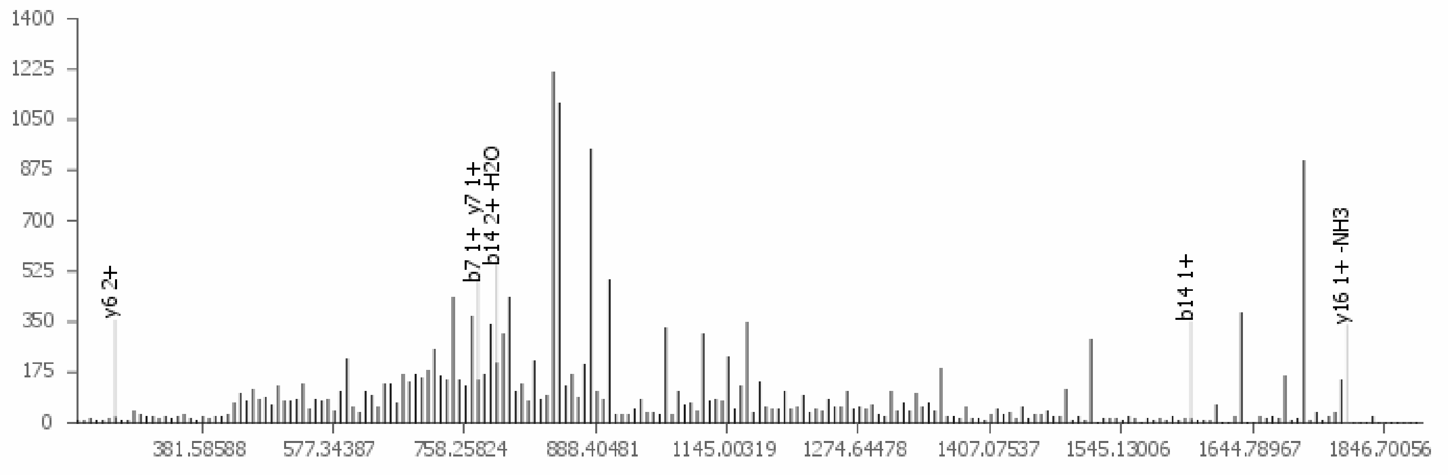

AT1G77210.1 - RG(pS)ILVGSVSFFLGGVINAAAK - 1122.096553 - Charge:2

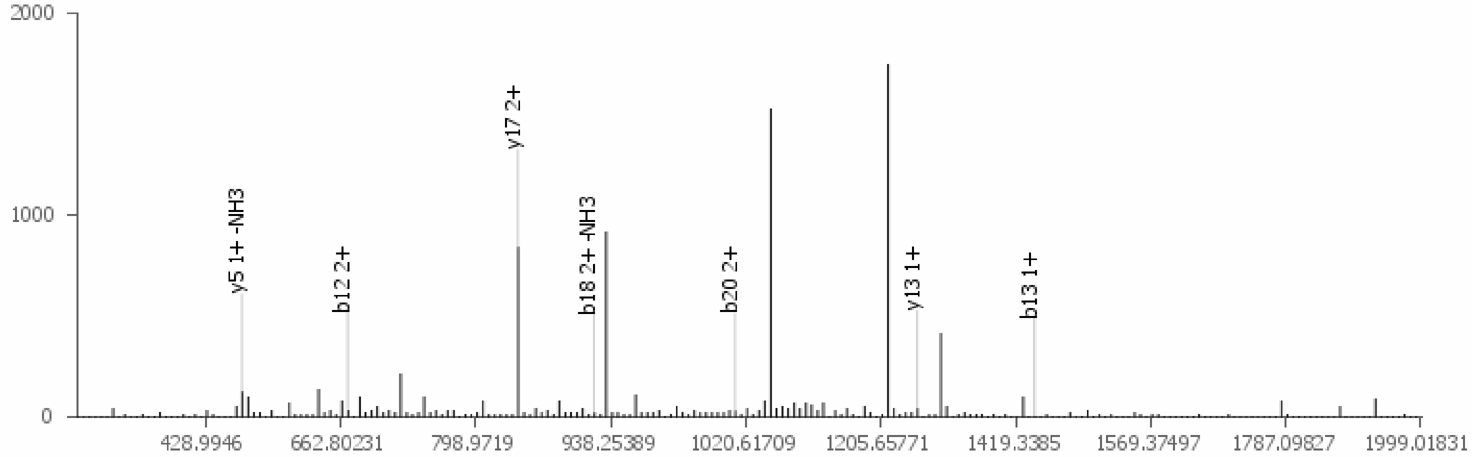

AT2G45330.1 - ANQGHSI(pT)TVESEK - 790.854824 - Charge:2

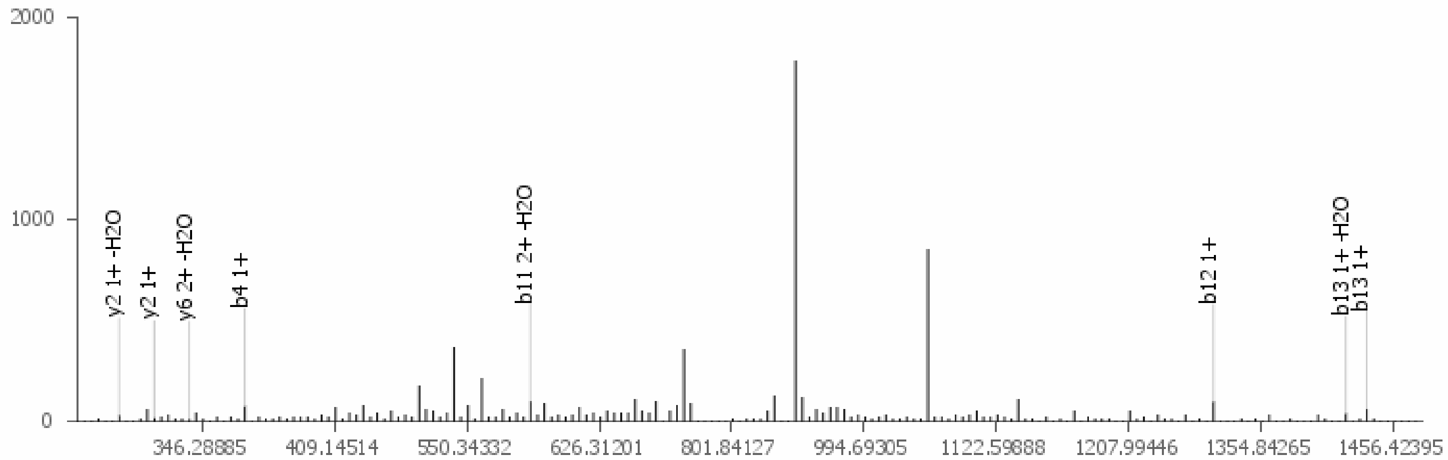

AT3G02700.1 - SGQAA(pS)IVAAA(pS)VLLSSPLR - 1029.505647 - Charge:2

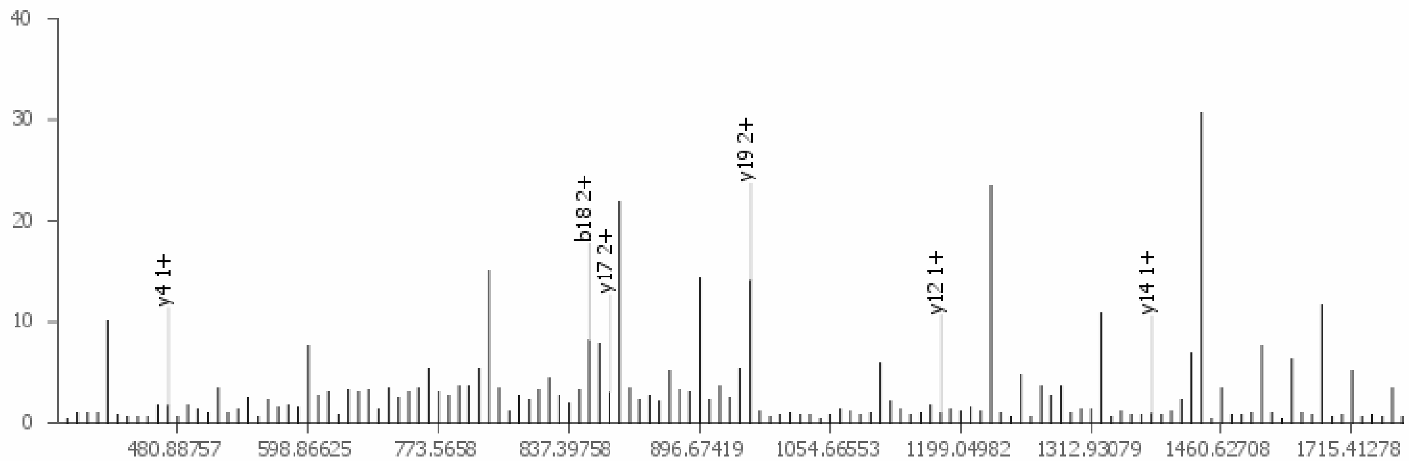

AT3G24760.1 - LILVGG(pS)SR - 491.262872 - Charge:2

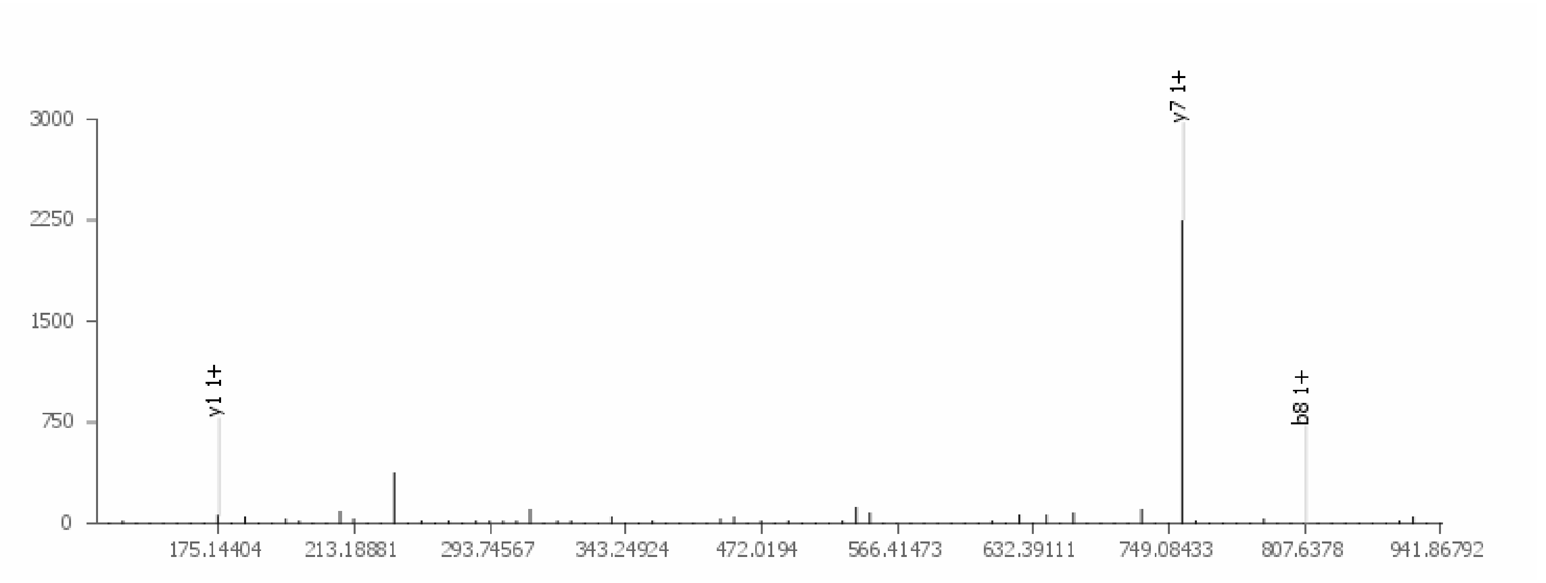

AT1G19220.1 - HLL(t)(t)GWSVFV(s)(t)K - 868.403613 - Charge:2

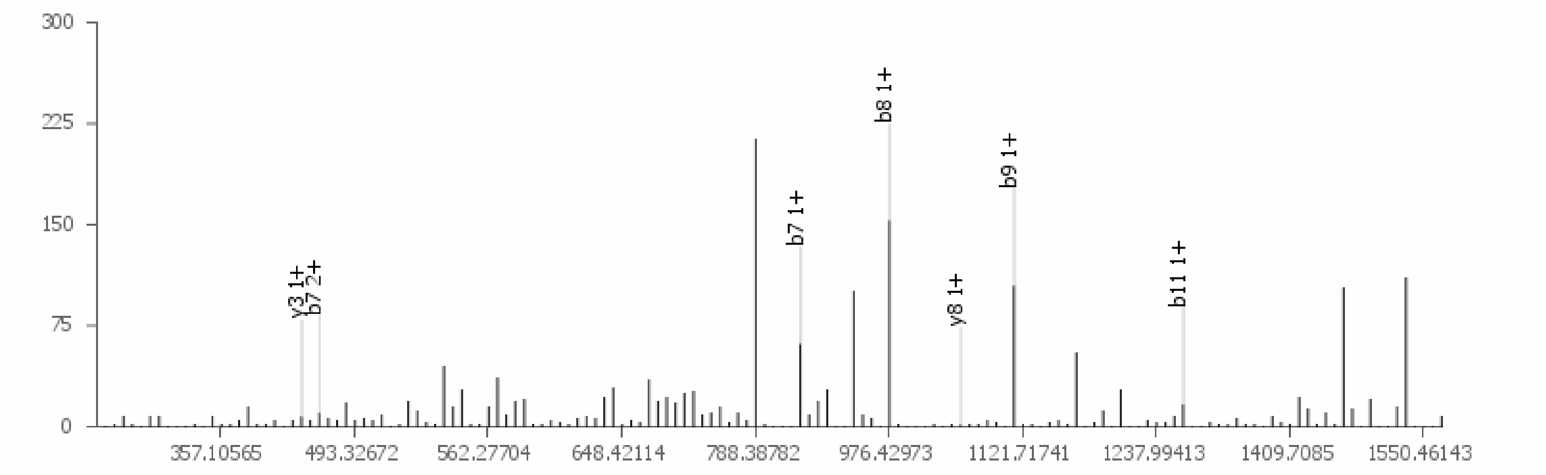

AT4G28320.1 - LMIEQ(pS)CRLGK - 707.834937 - Charge:2

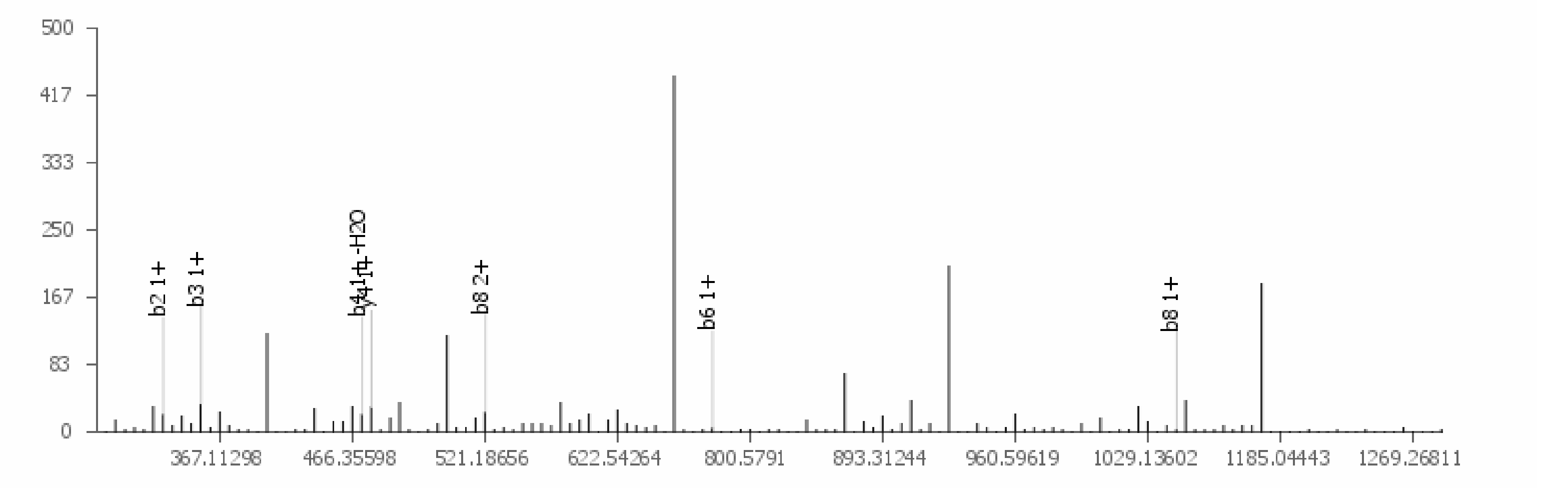

AT1G77460.1 - SMLVLADRIVNA(pS)(pS)LEVR - 1067.012247 - Charge:2

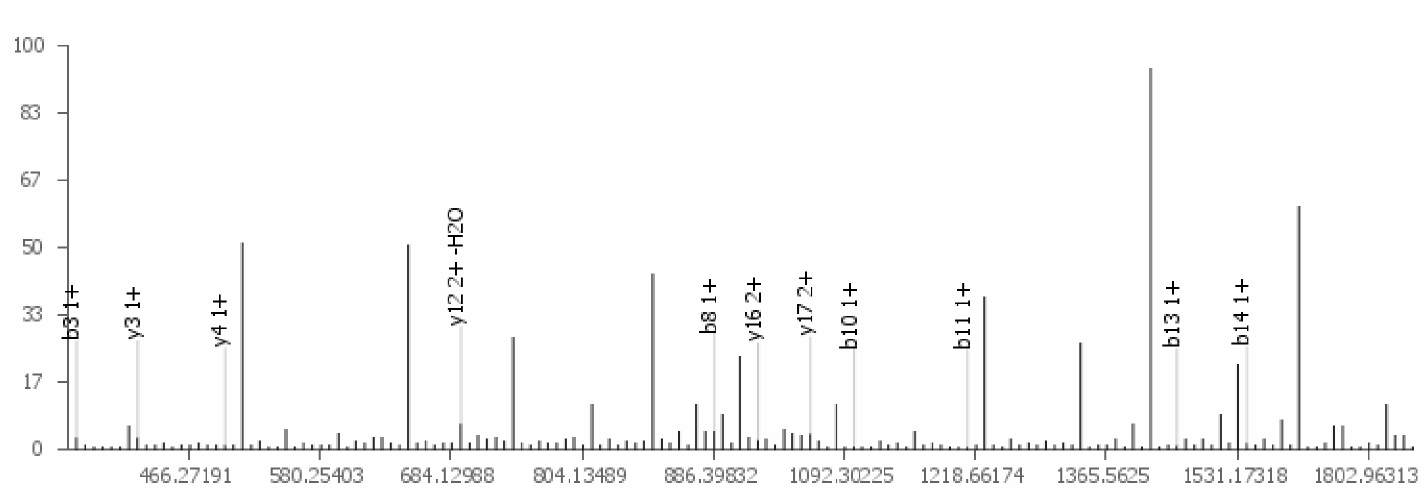

AT1G24570.1 - DSGIATPHKAIDI(pS)K - 816.910607 - Charge:2

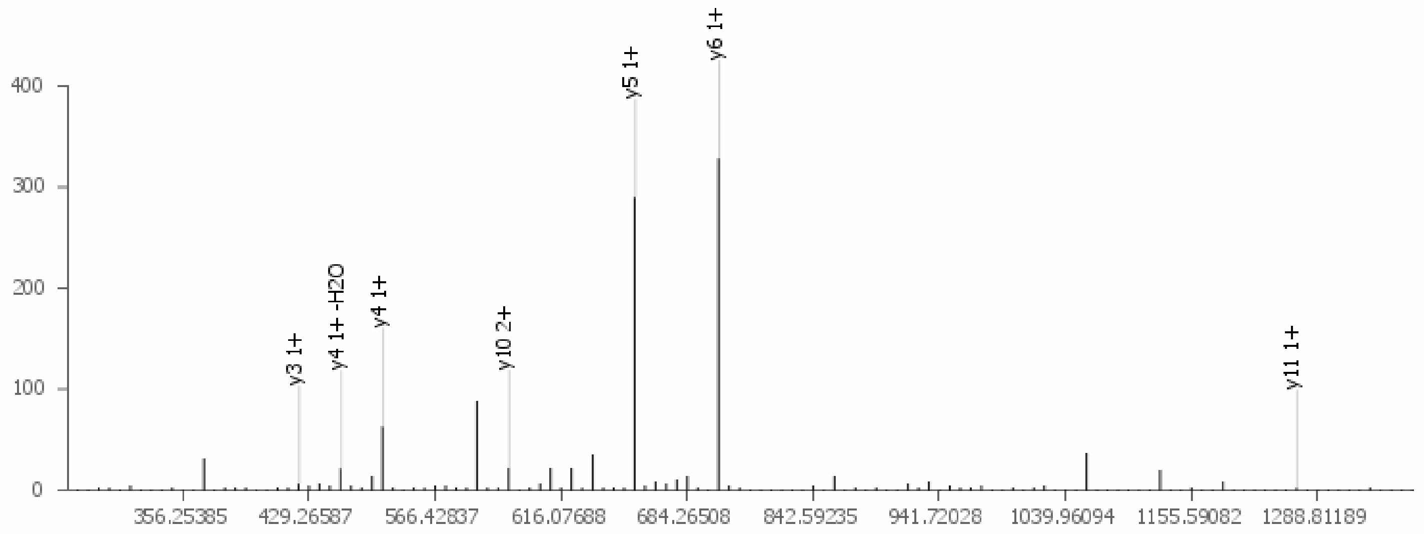

AT5G04290.1 - IDIQALTQKYGGGV(pT)VQK - 1000.01726 - Charge:2

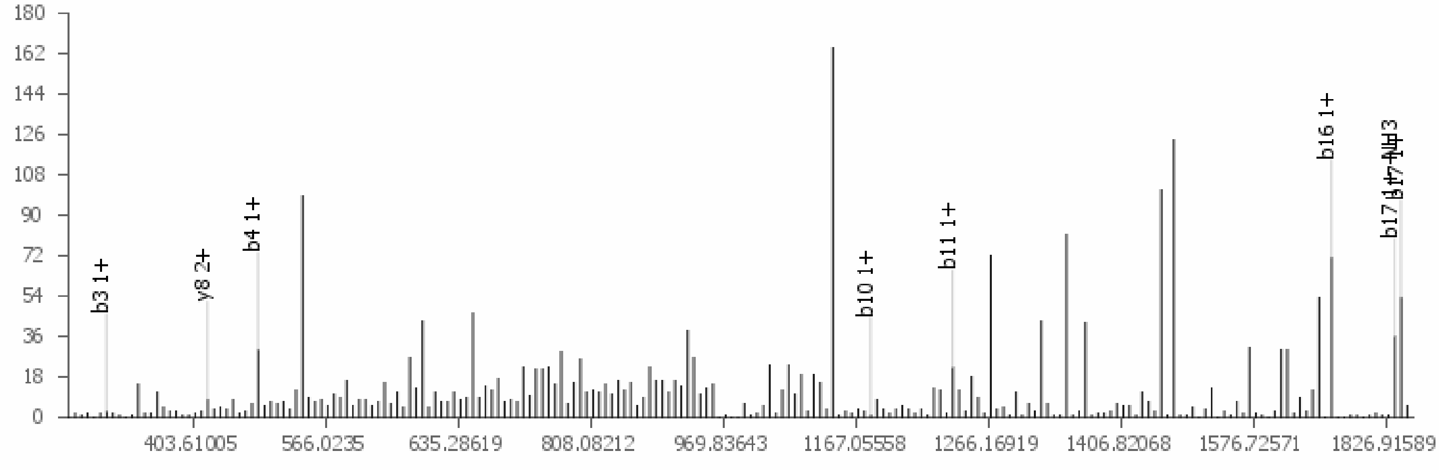

AT1G69660.1 - EDALNVEIEFKVV(s)A(t)K - 657.997083 - Charge:3

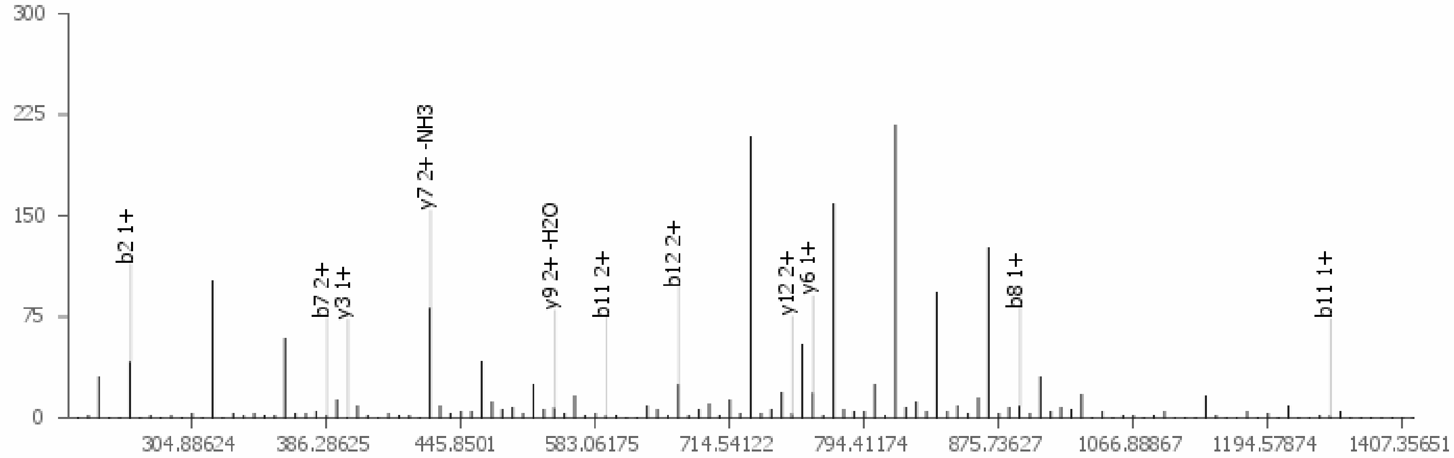

AT3G04740.1 - IEPG(pS)DLQIK - 590.289144 - Charge:2

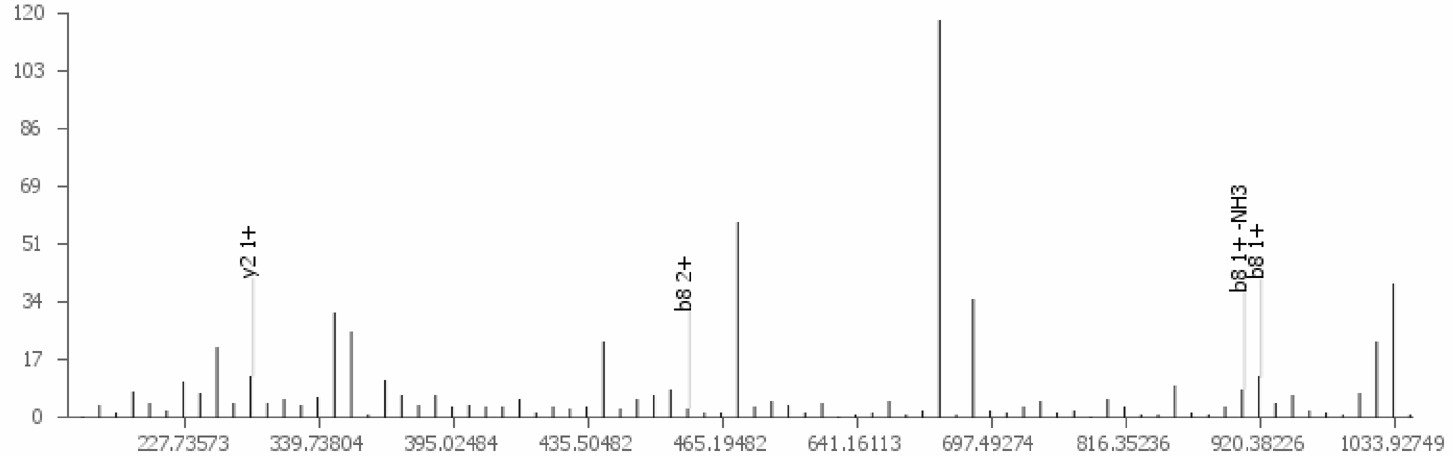

AT1G30240.1 - IEAAIASKIF(pS)AK - 714.876142 - Charge:2

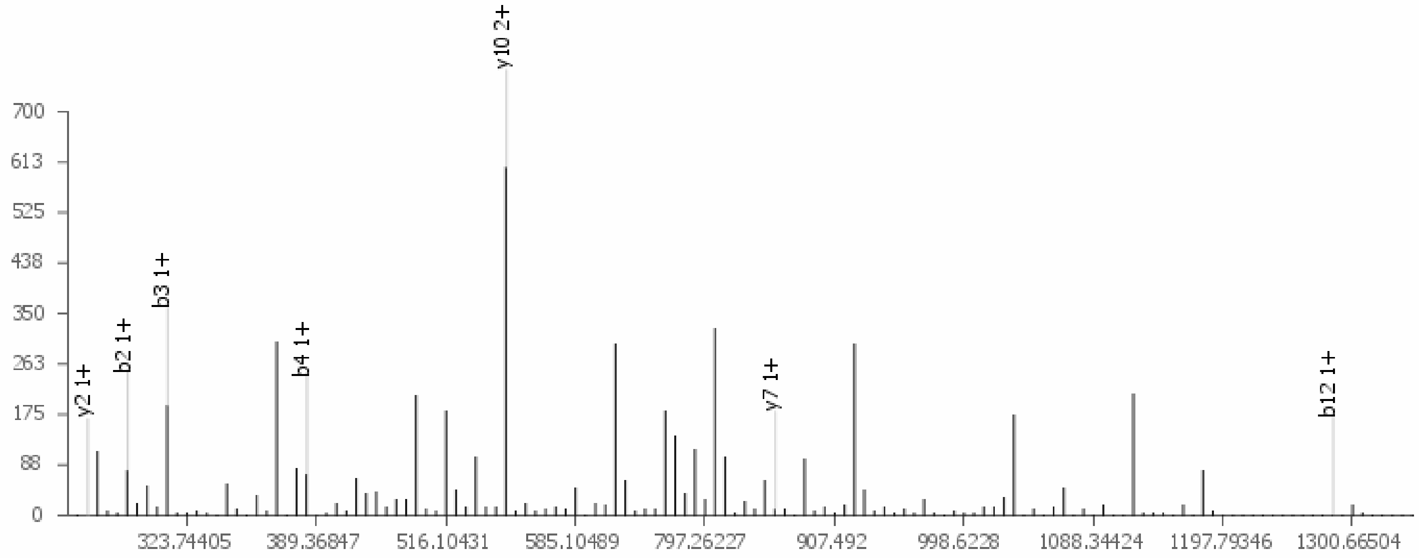

AT1G34440.1 - LHSAT(pS)RP(pT)TSIIKEIK - 1021.514315 - Charge:2

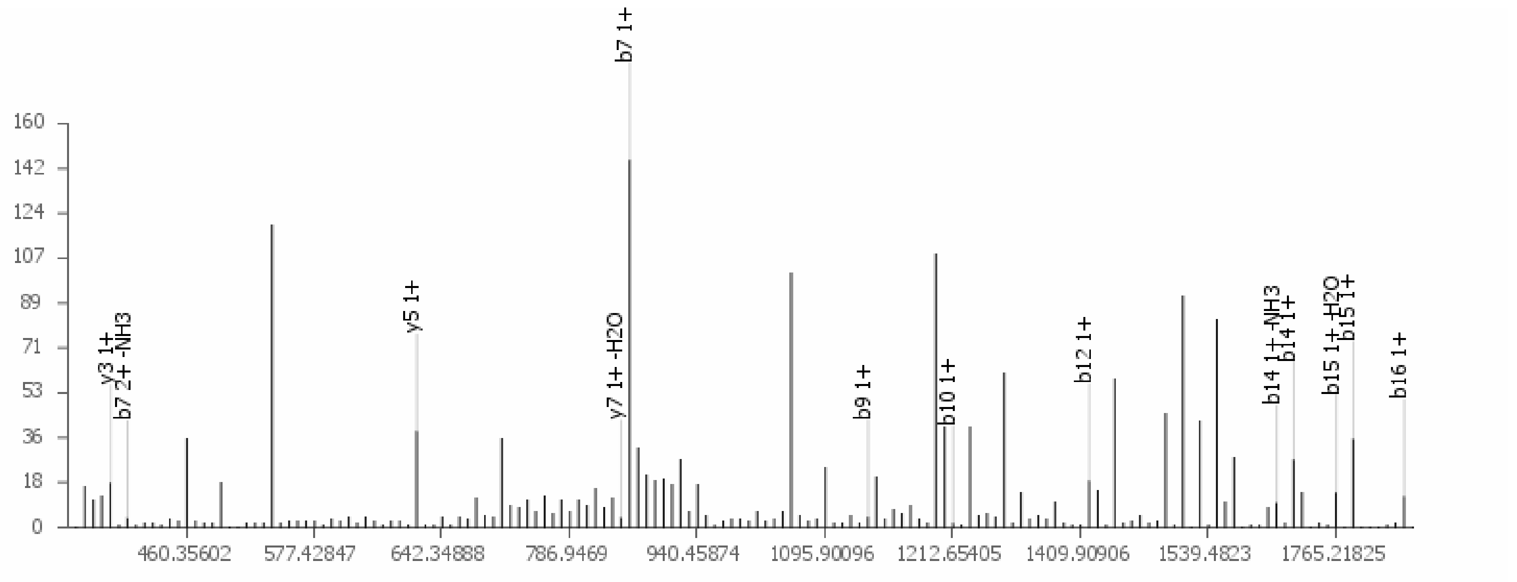

AT2G18510.1 - LLAA(pT)NPTAQK - 604.310739 - Charge:2

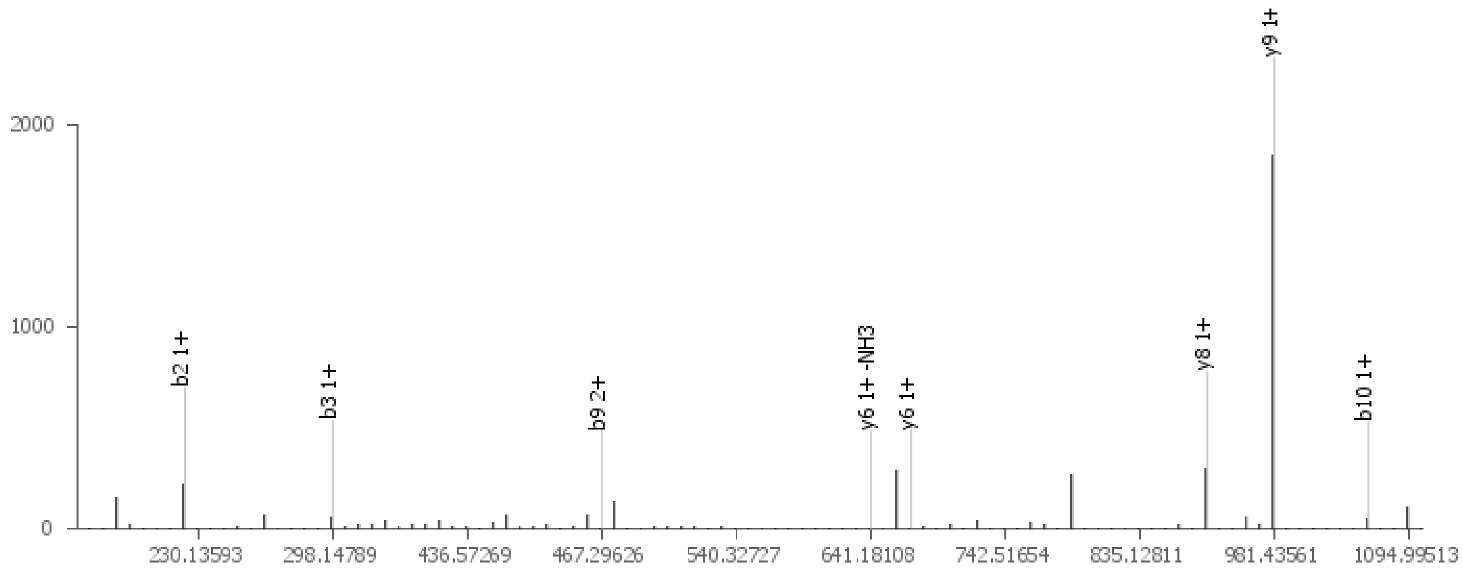

AT1G65250.1 - KPK(pS)DIASER - 605.793069 - Charge:2

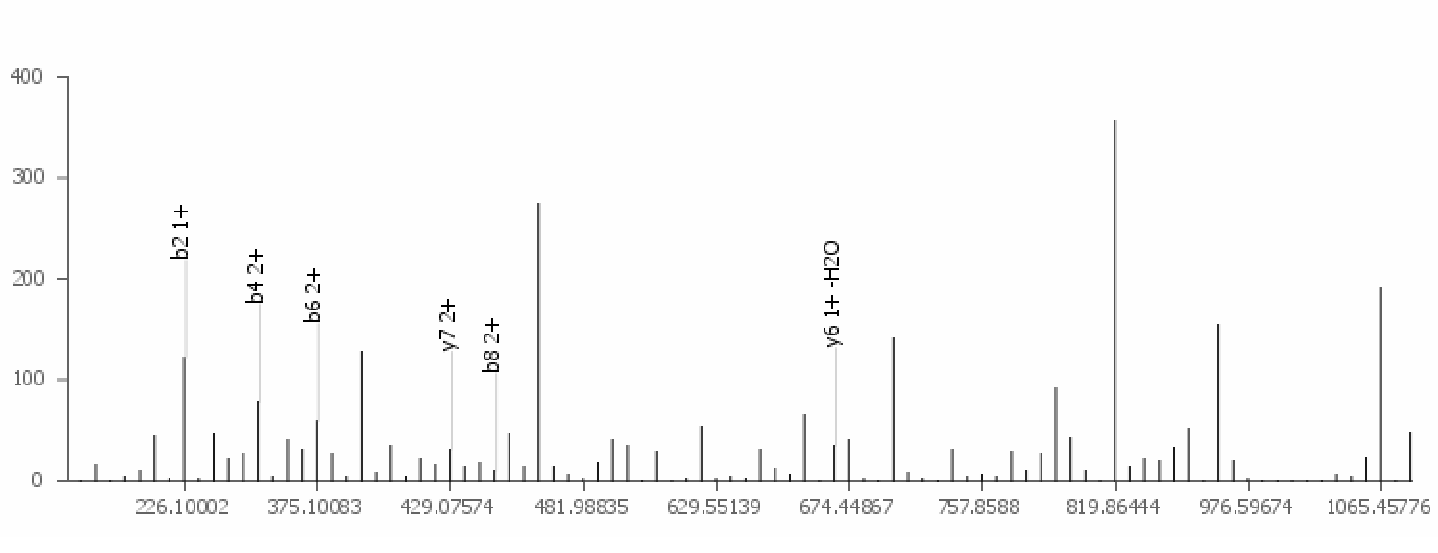

AT4G32260.1 - VEEELKEALASLE(pS)QK - 941.958146 - Charge:2

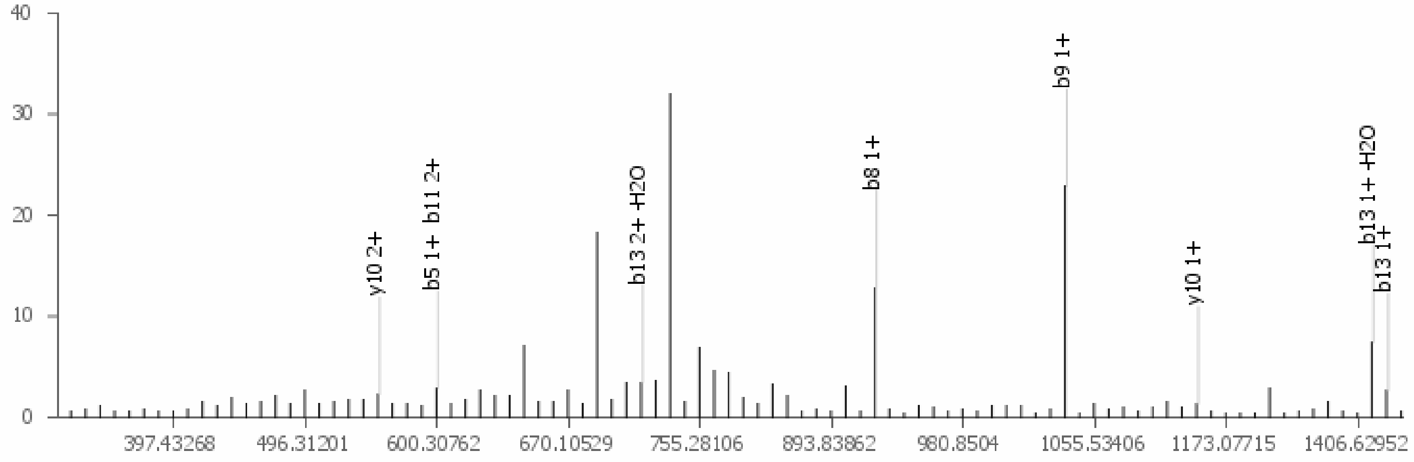

AT1G31410.1 - MSNV(pT)VIVPK - 584.299847 - Charge:2

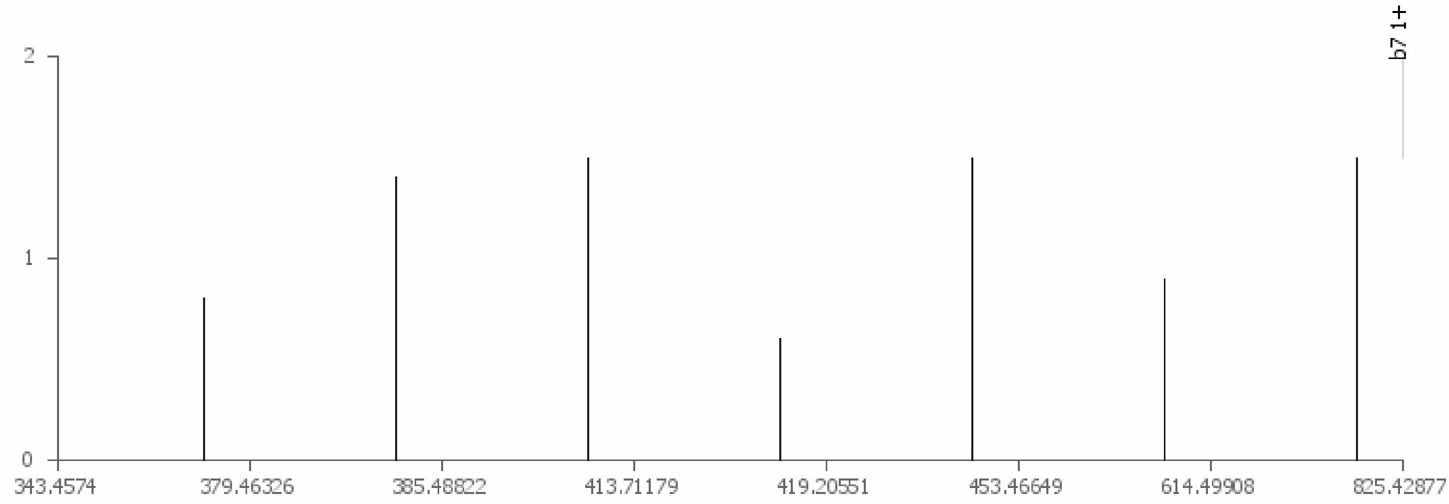

AT5G60610.1 - (pY)LIVTPSLKYFK - 776.408536 - Charge:2

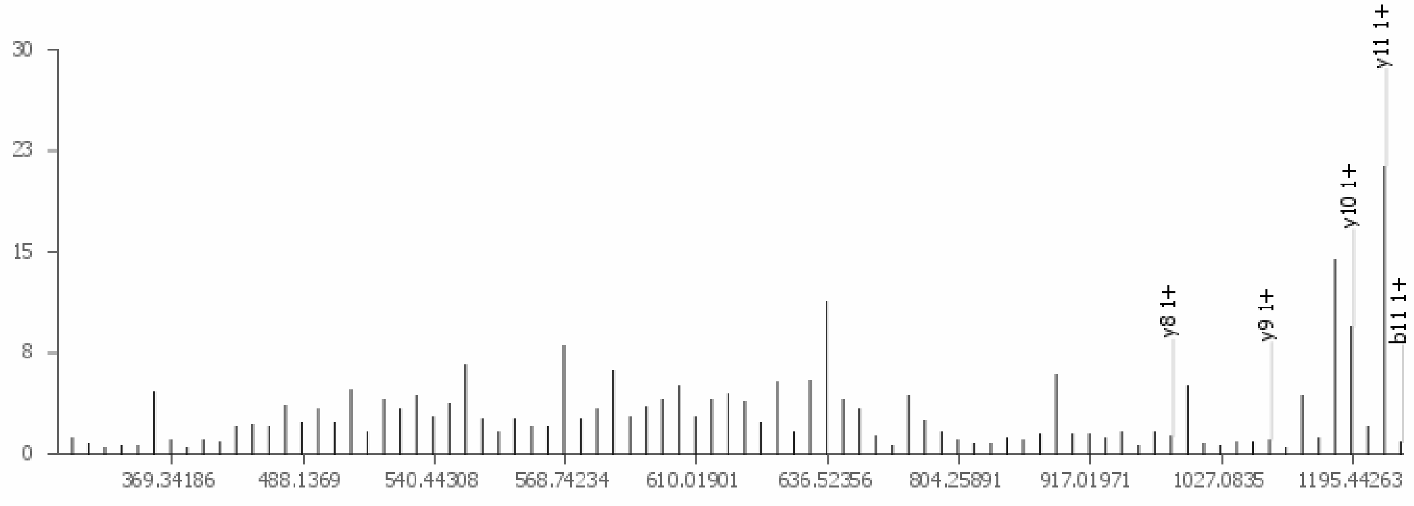

AT1G06590.1 - KDTEA(pT)PVAR - 584.279315 - Charge:2

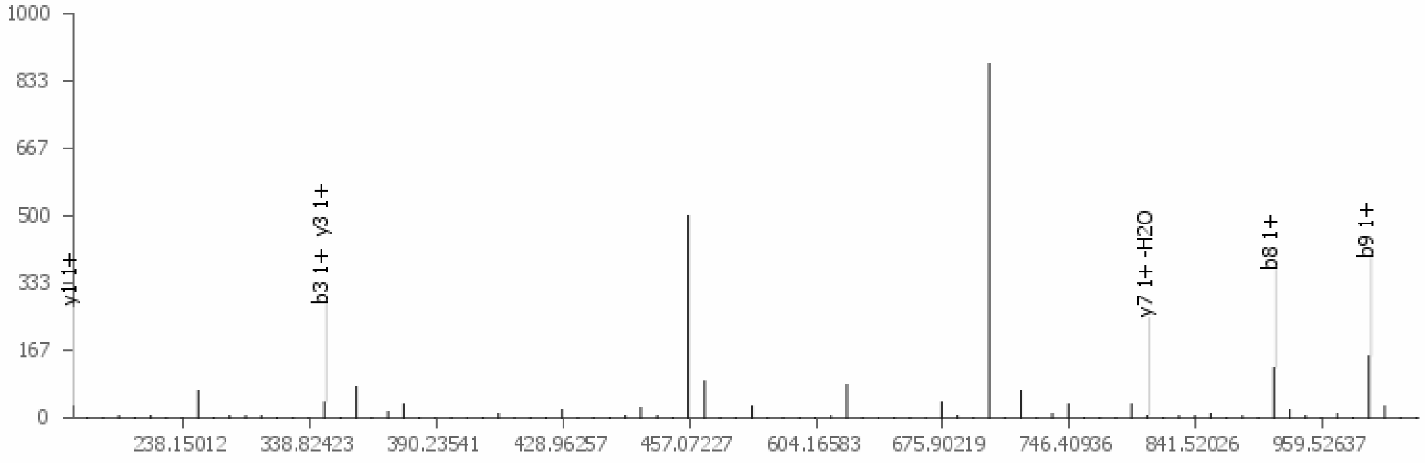

AT4G28200.1 - LGTSE(pT)ANGVYWR - 767.346738 - Charge:2

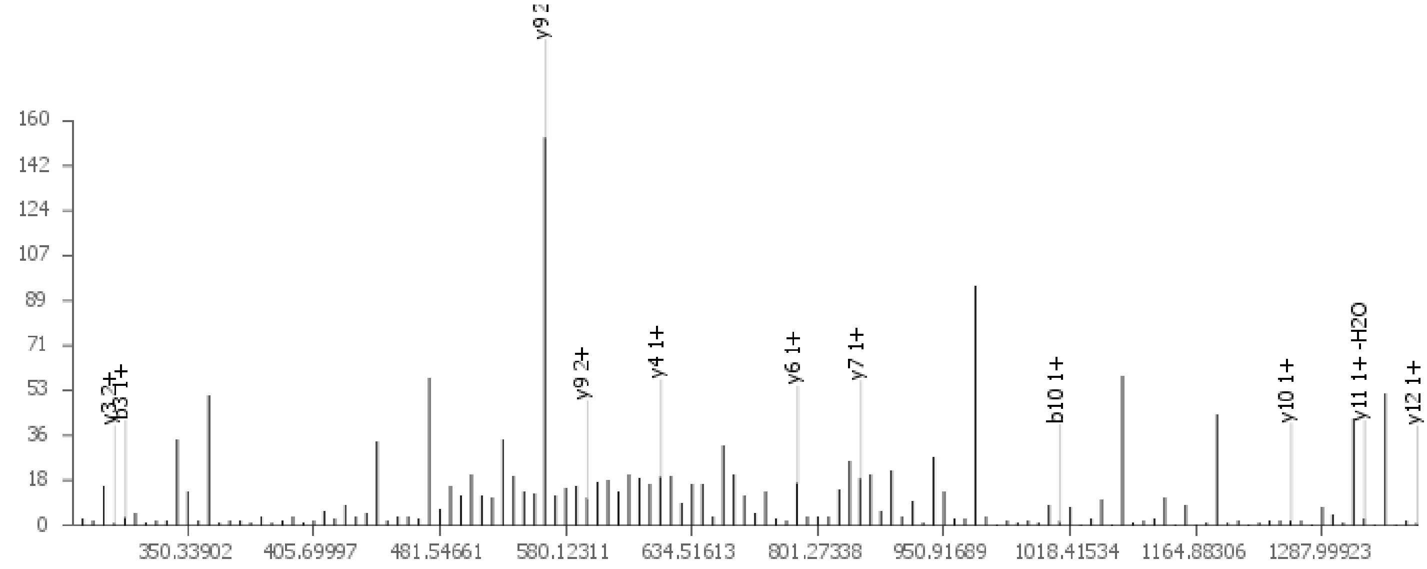

AT5G01820.1 - STATGQ(pS)VAIK - 571.777663 - Charge:2

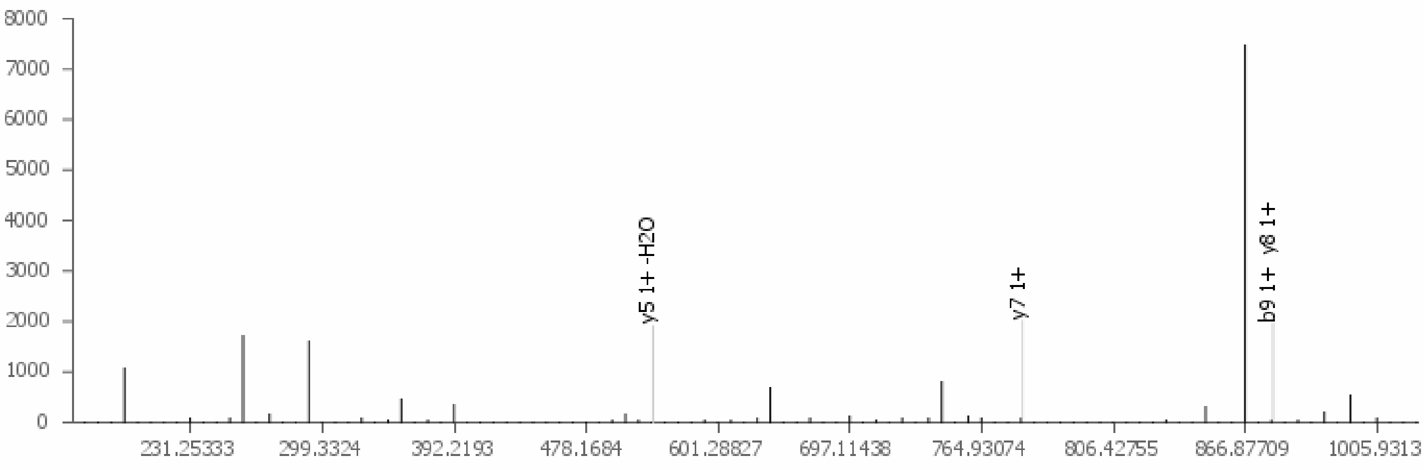

AT5G39940.1 - (pS)VLAA(pS)IKPDGK - 673.317068 - Charge:2

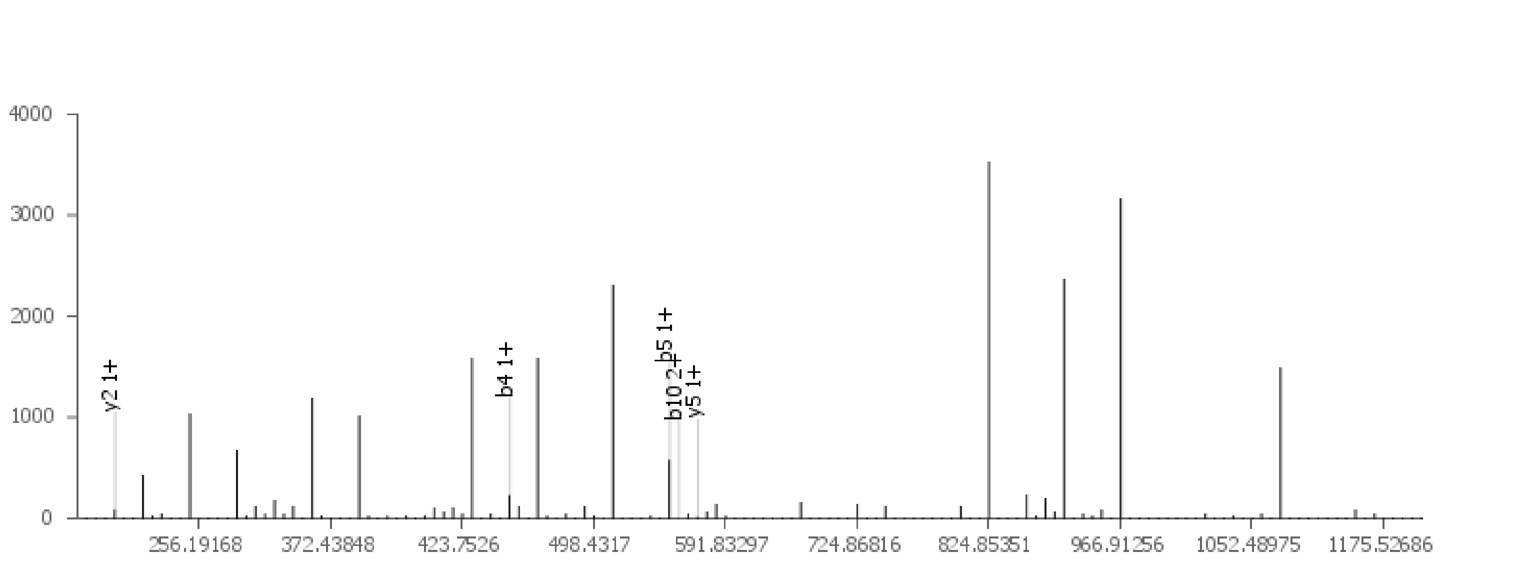

AT3G17240.1 - FPF(oxM)AN(pS)RAK - 632.783631 - Charge:2

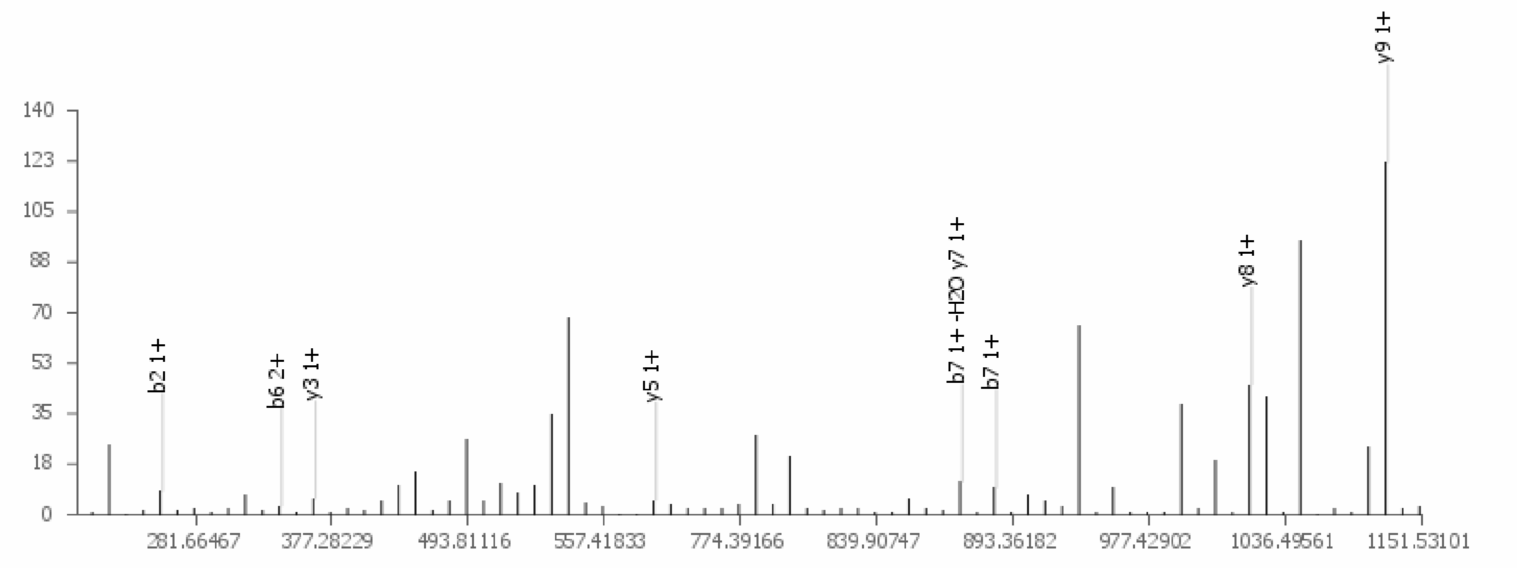

AT1G62000.1 - LGA(pS)LPK - 383.198249 - Charge:2

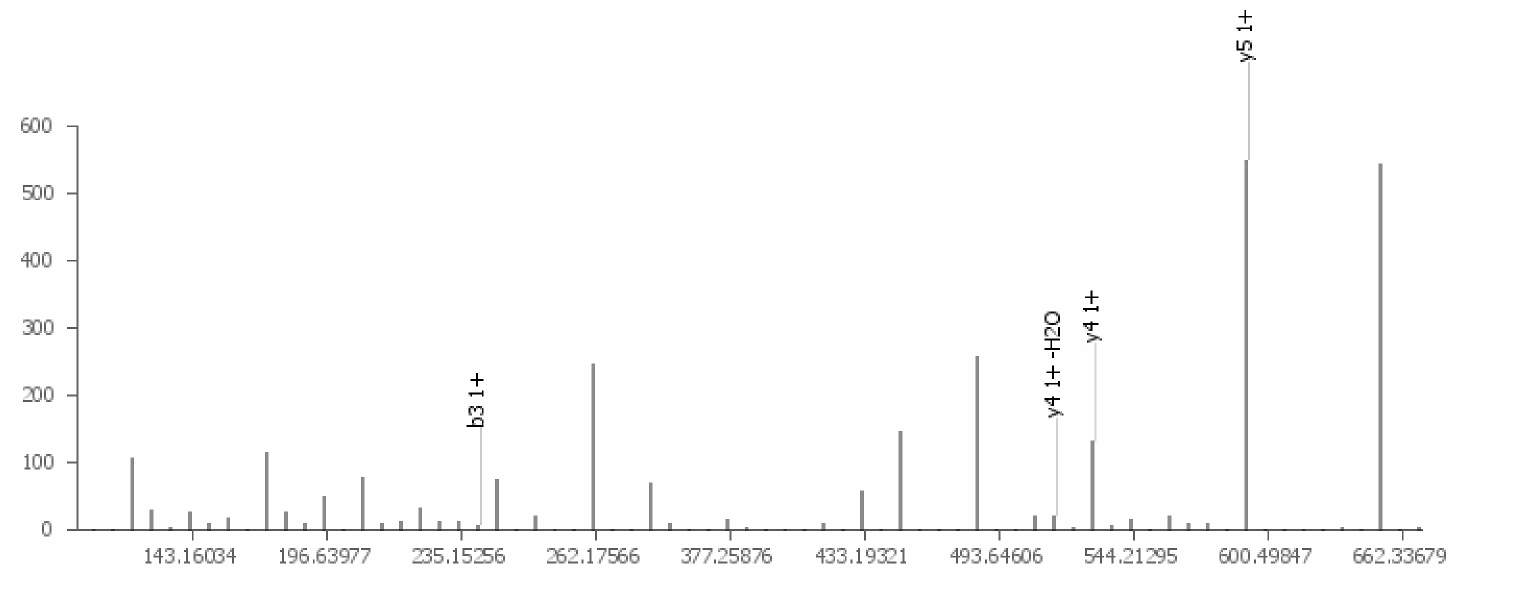

AT1G68050.1 - EVAGG(pT)PPLPR - 587.285111 - Charge:2

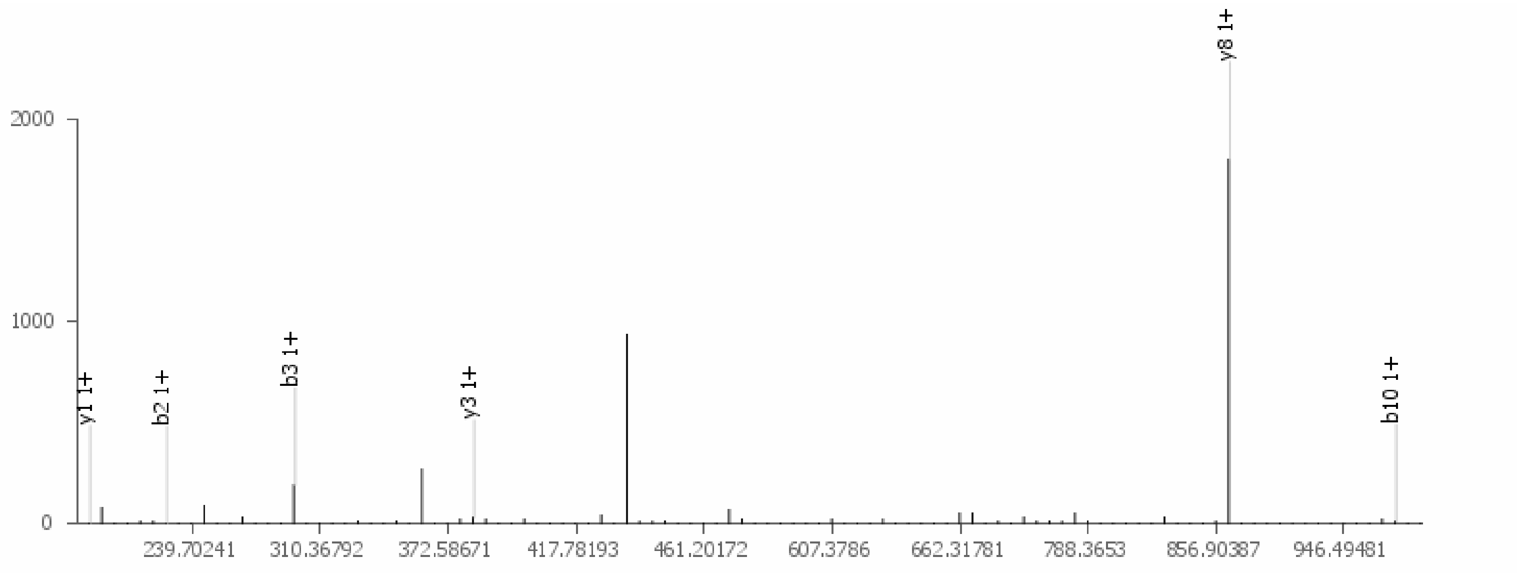

AT2G47500.1 - VLNKVQPGAV(pS)K - 660.363398 - Charge:2

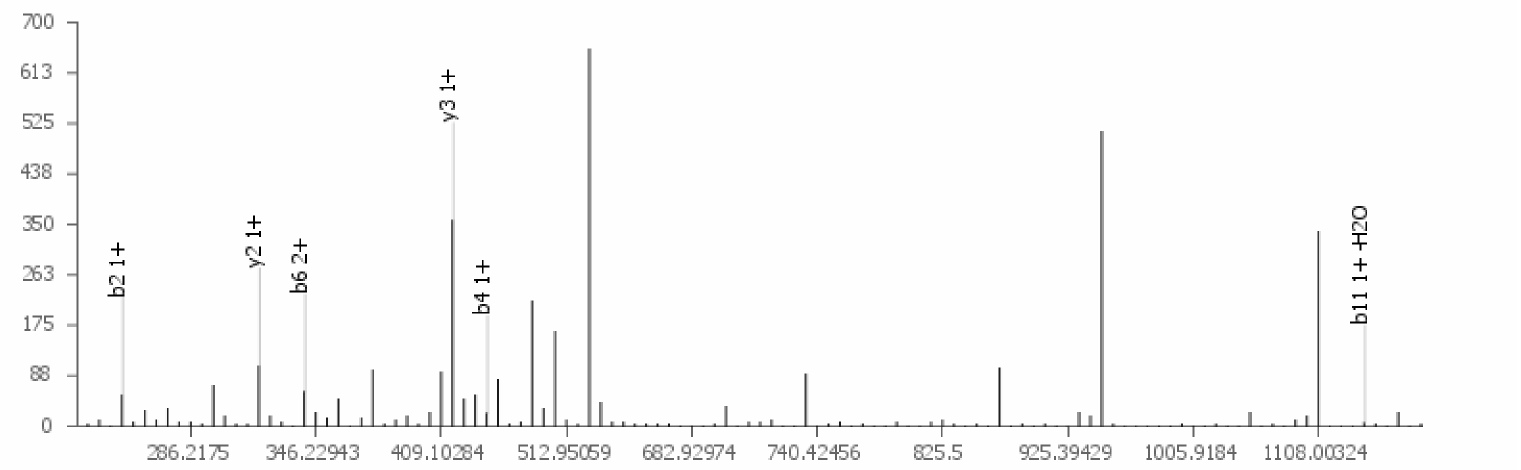

AT1G05790.1 - LQVAAIKVL(pS)QDPK - 795.442917 - Charge:2

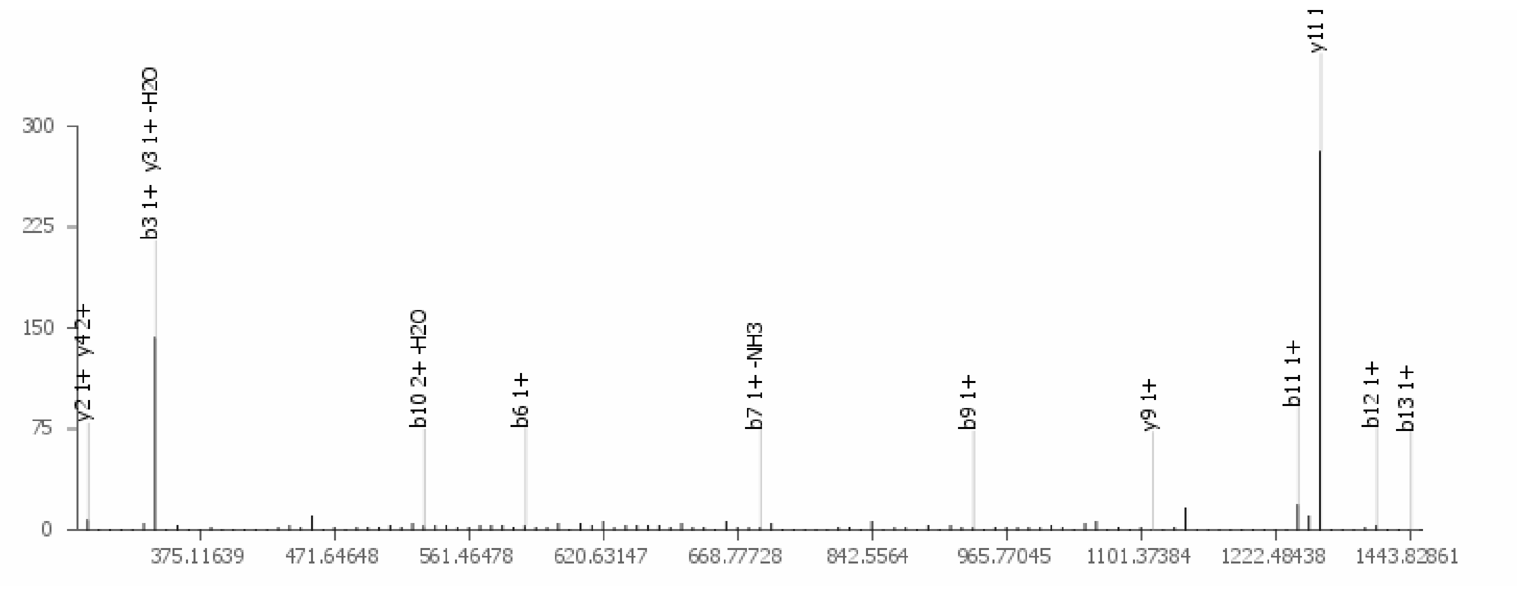

AT3G23280.1 - RVWVVIVP(pT)G(pS)R - 764.879076 - Charge:2

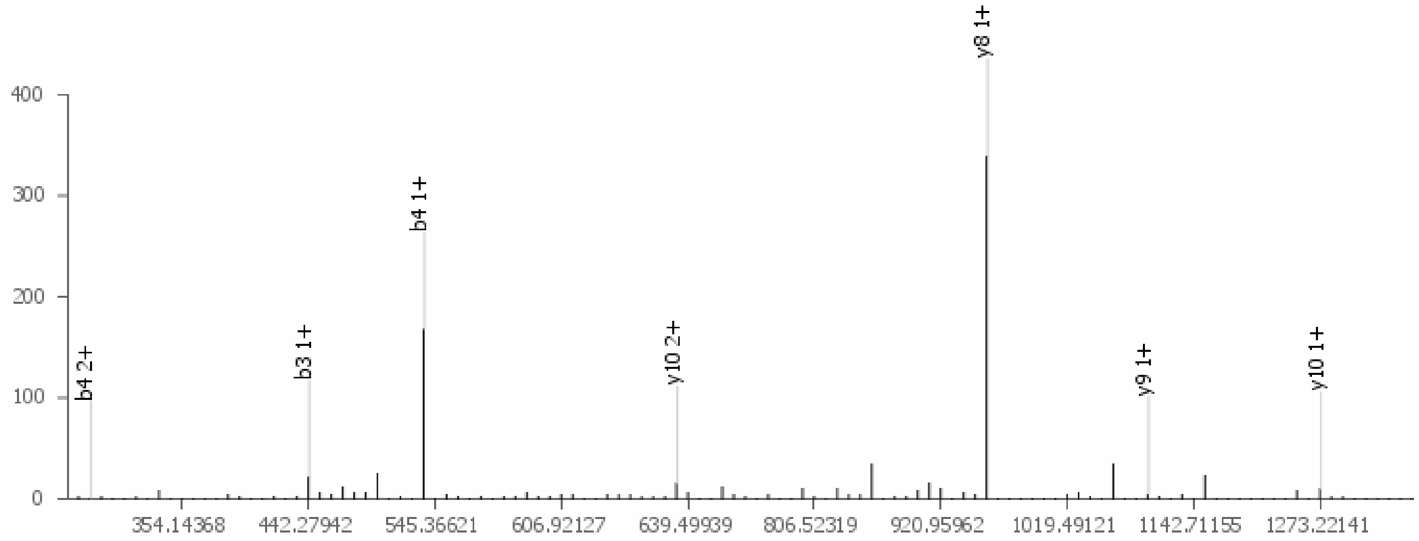

AT5G37410.1 - SLS(pS)LEVLK - 528.276023 - Charge:2

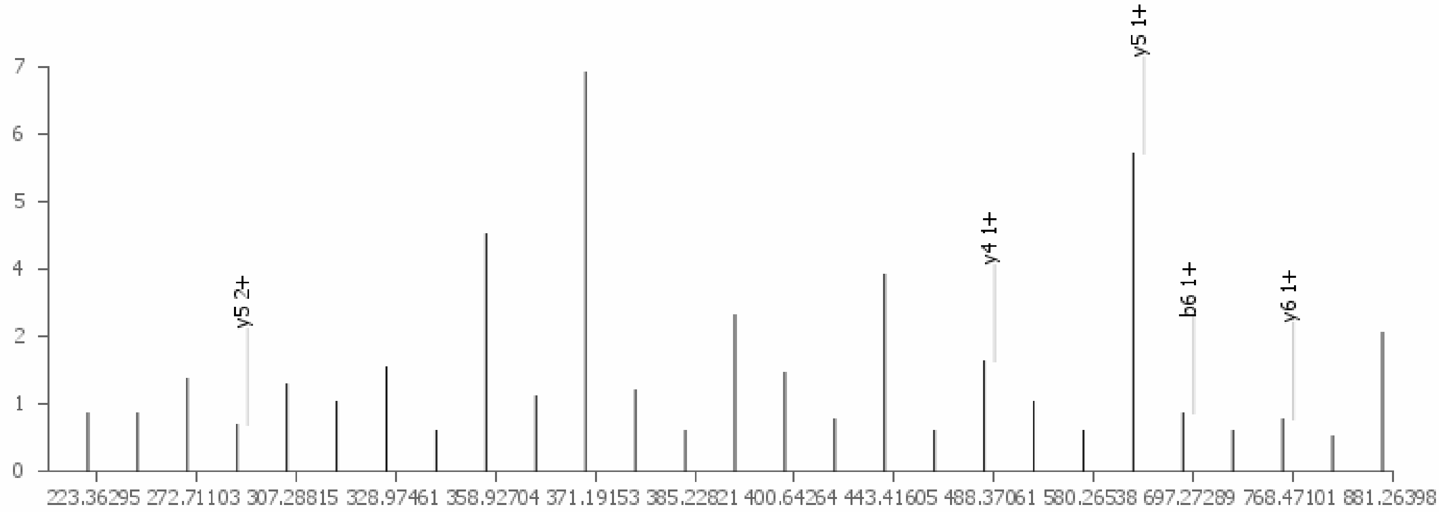

AT3G49055.1 - DQLIAVLK(pS)EVEK - 776.409233 - Charge:2

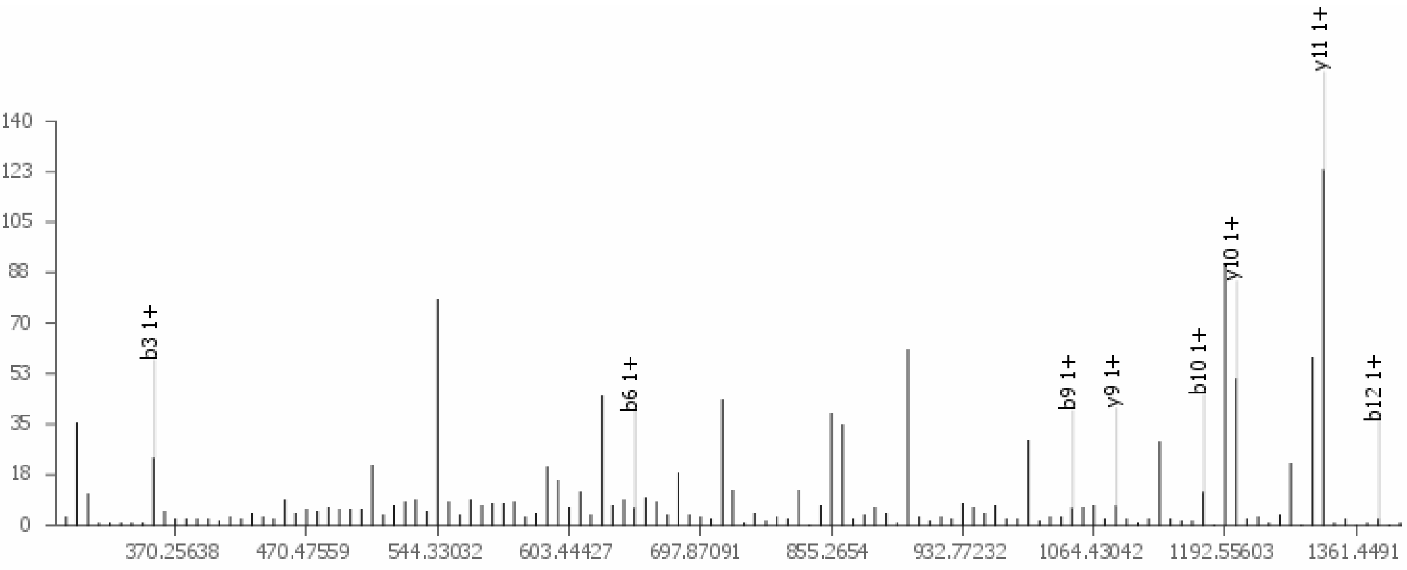

AT5G46400.1 - GISSIVD(pS)PPK - 590.287302 - Charge:2

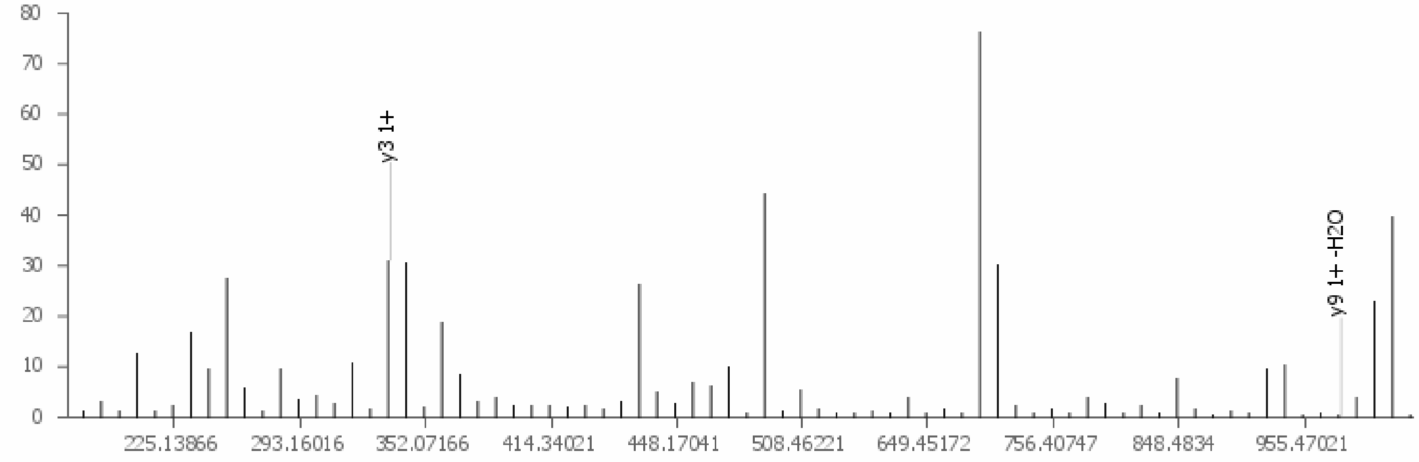

AT4G13730.1 - IESPGAL(pS)R - 505.237954 - Charge:2

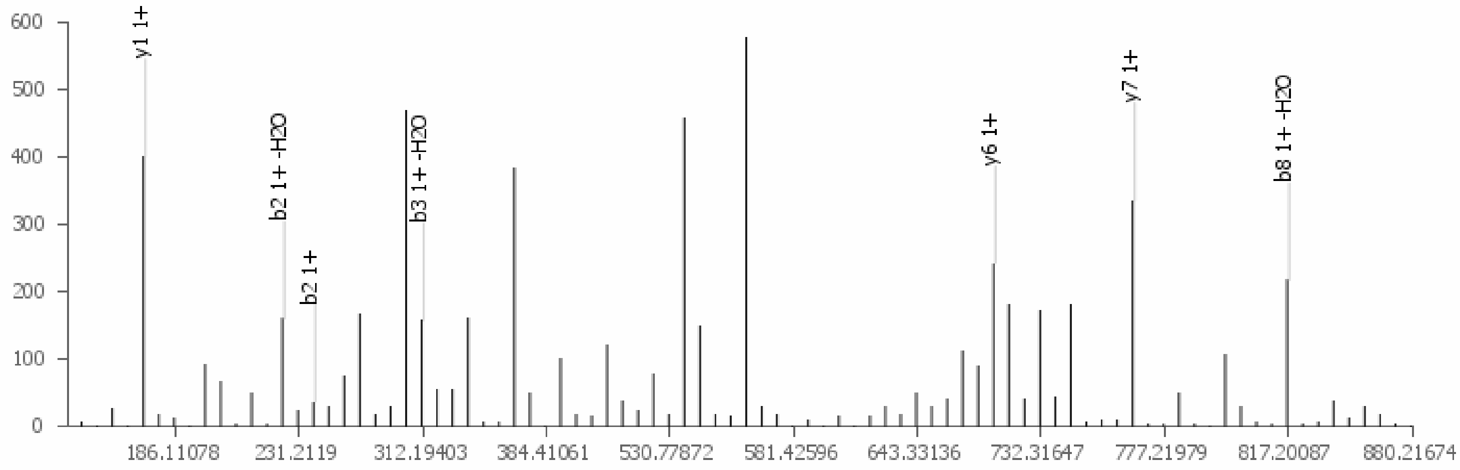

AT3G49220.1 - KPSQAI(pS)K - 469.739236 - Charge:2

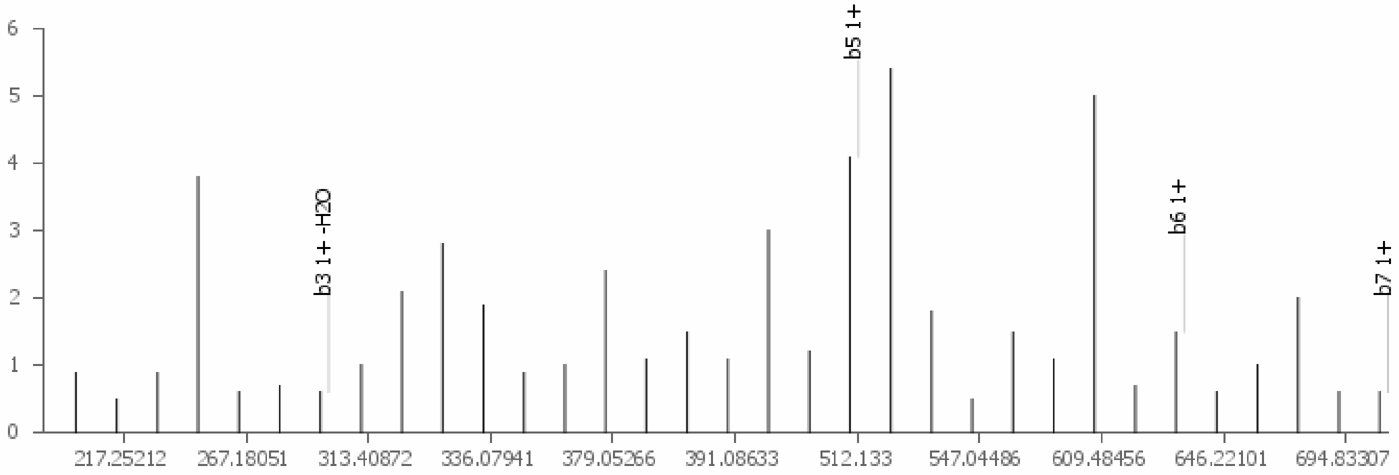

AT1G65260.1 - KSFADNA(pT)ALK - 623.293294 - Charge:2

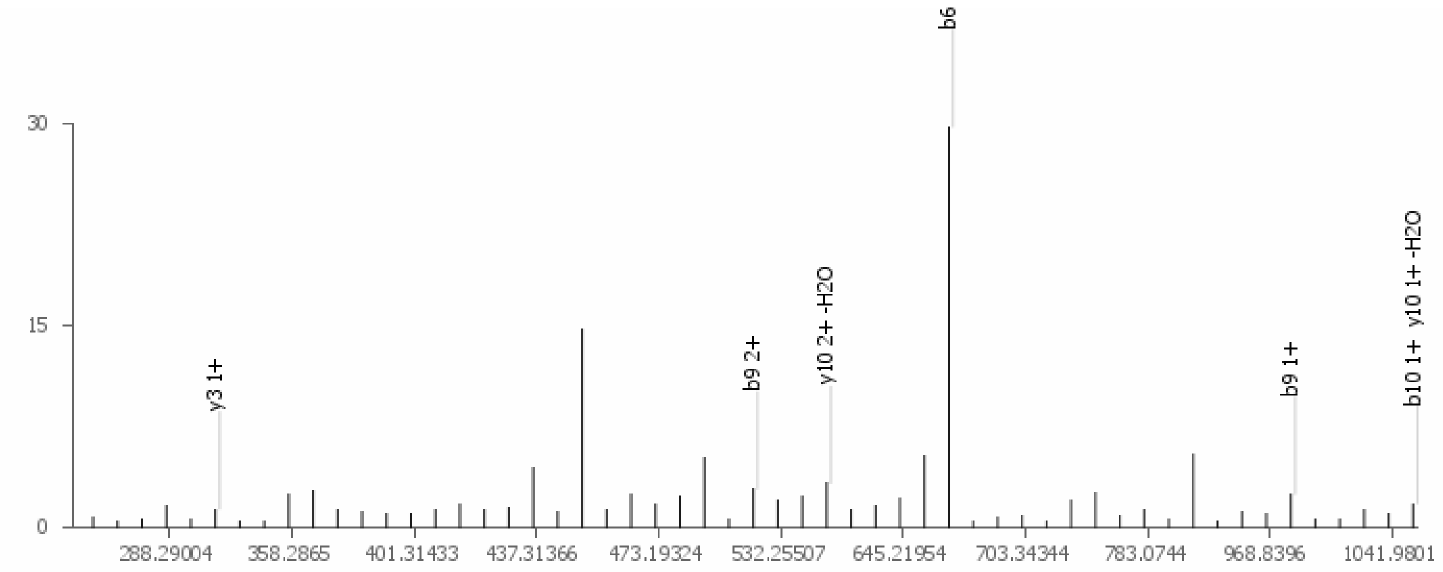

AT2G30010.1 - (pY)IN(pS)PLTR - 562.23625 - Charge:2

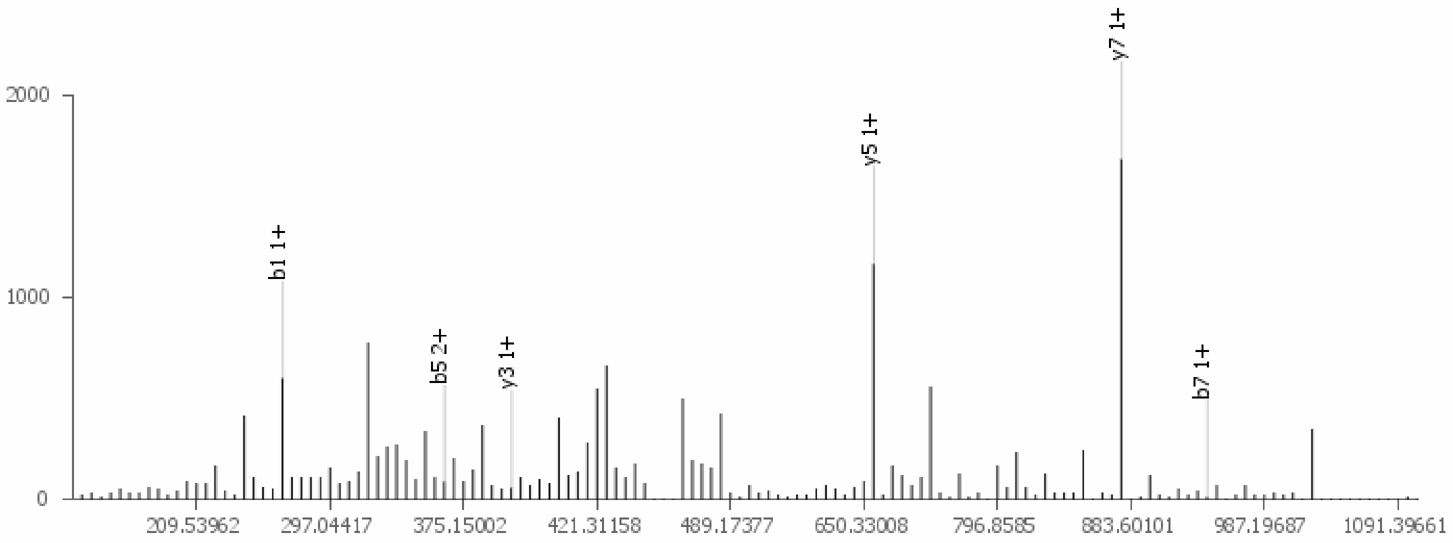

AT5G45510.1 - A(pY)QDGH(pS)IFMELIDRGMLK - 795.347322 - Charge:3

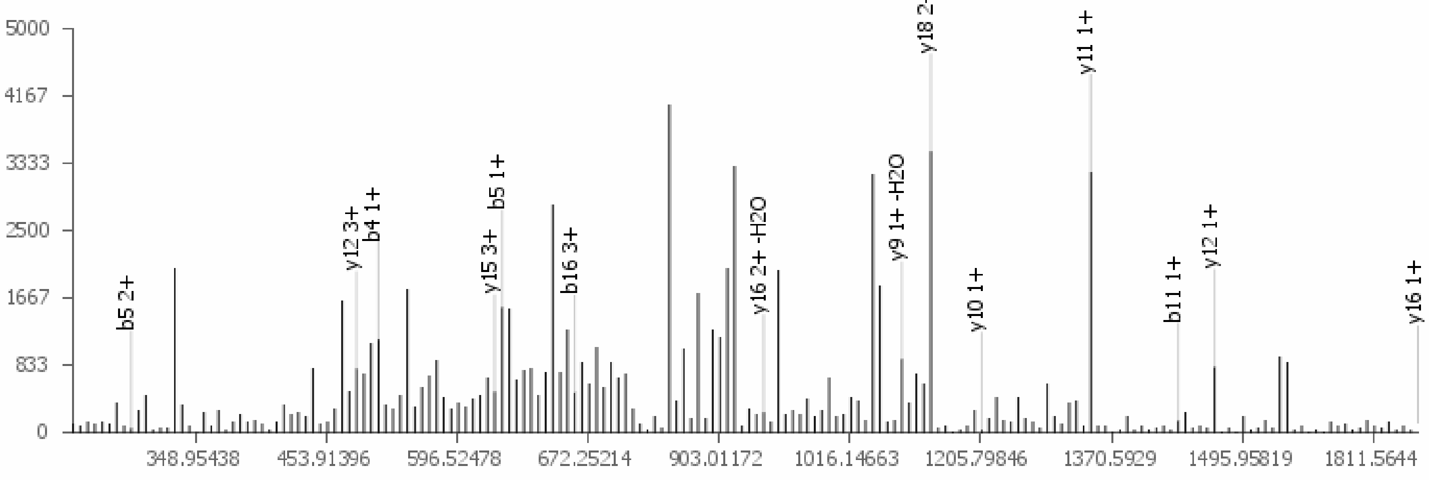

AT1G33120.1 - IDSWFG(pT)R - 531.230419 - Charge:2

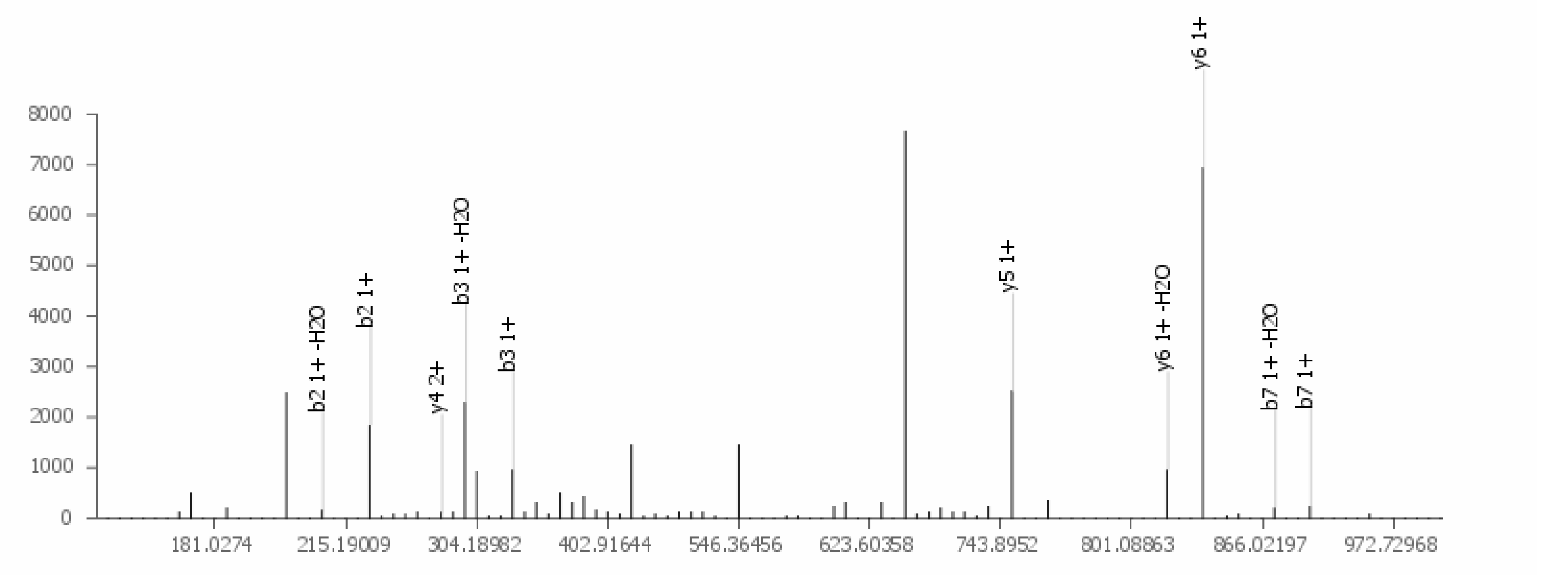

AT3G04250.1 - (pY)DQNMLR - 510.200472 - Charge:2

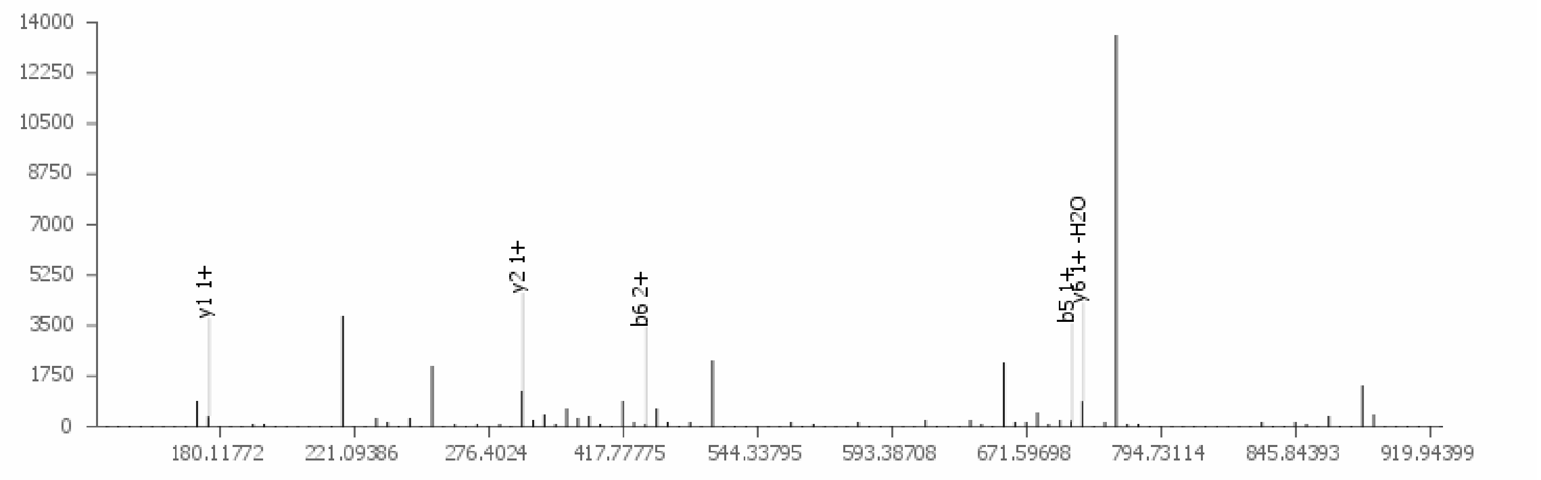

AT4G19515.1 - EIIHMLPA(s)(t)KELVSMA(pS)GSPCNR - 930.086788 - Charge:3

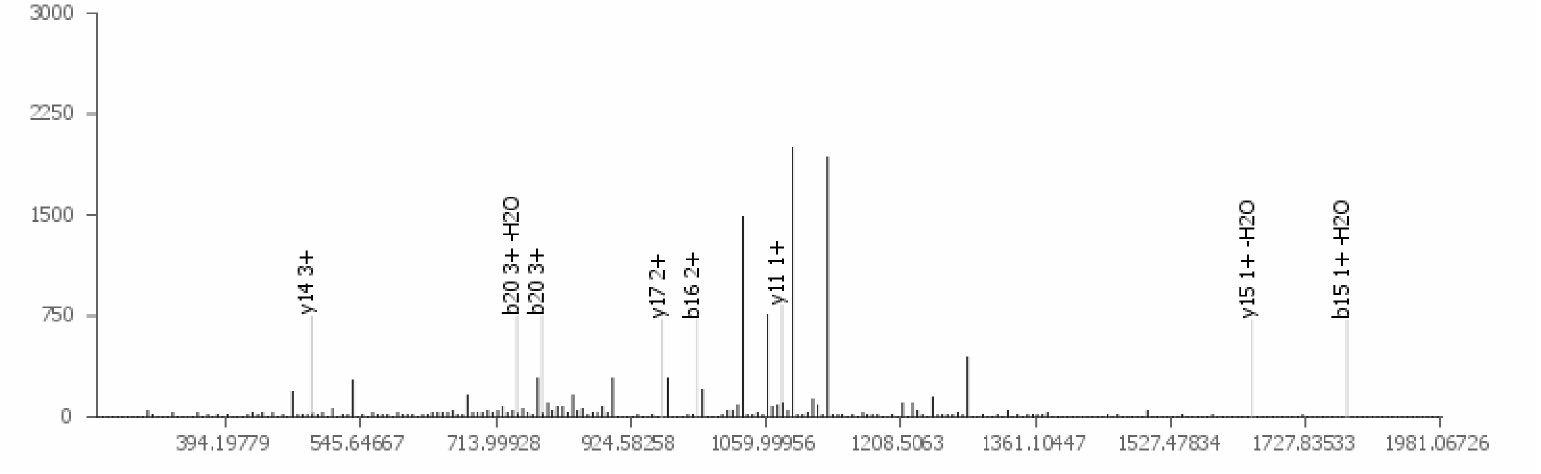

AT1G09540.1 - DKP(pT)(t)(s)NNKR - 660.772062 - Charge:2

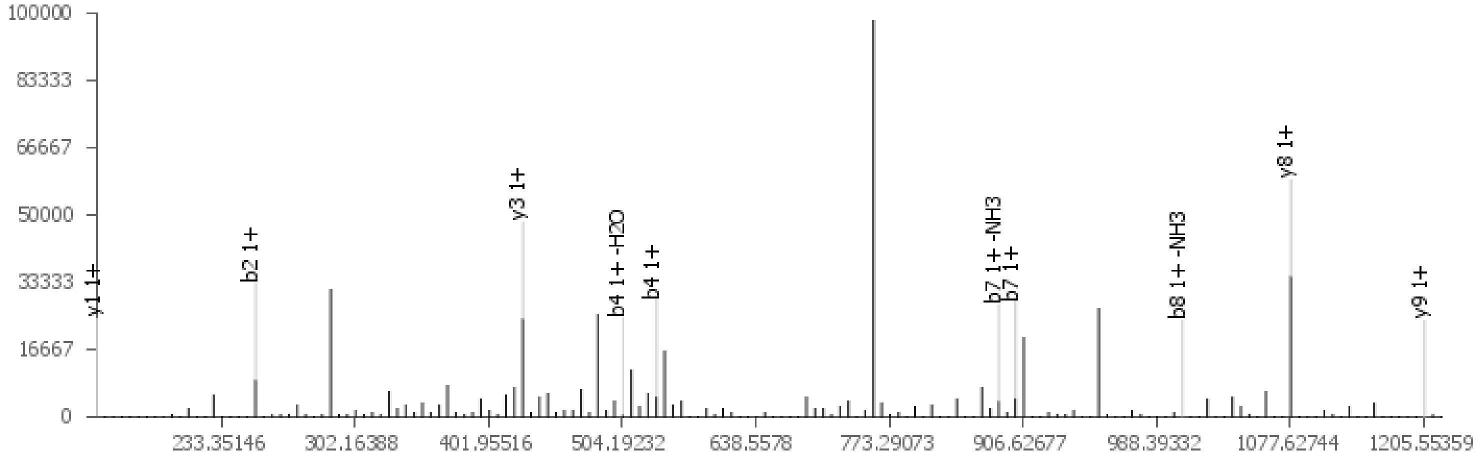

AT2G29170.1 - CRW(pS)LGGM(pT)ALVTGGSK - 970.919443 - Charge:2

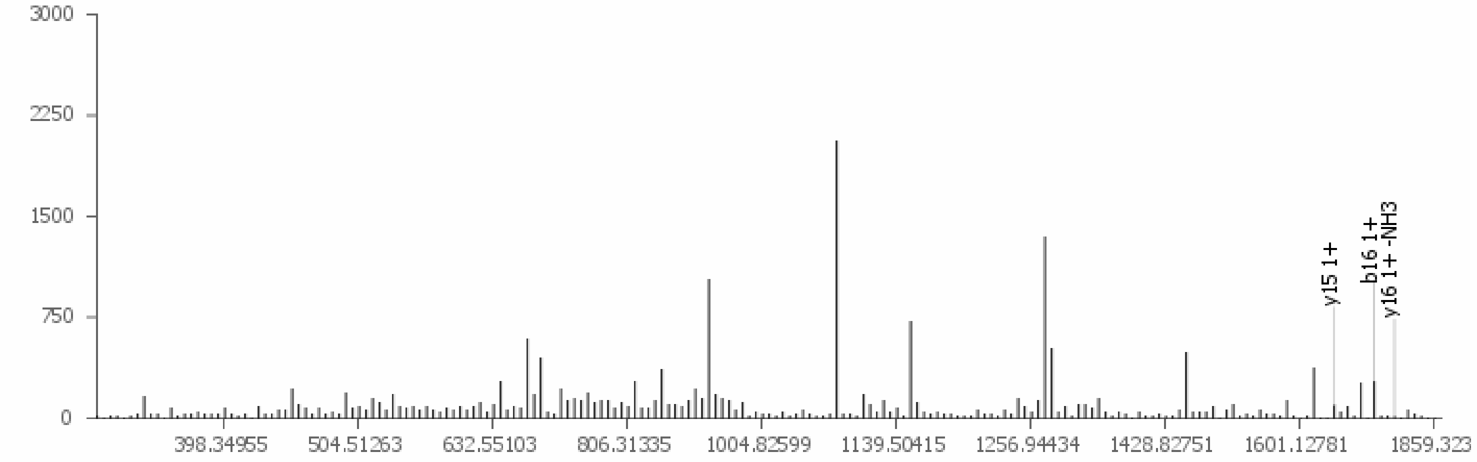

AT2G18130.1 - (pY)FNYTSGYLHHA(pT)IK - 987.903919 - Charge:2

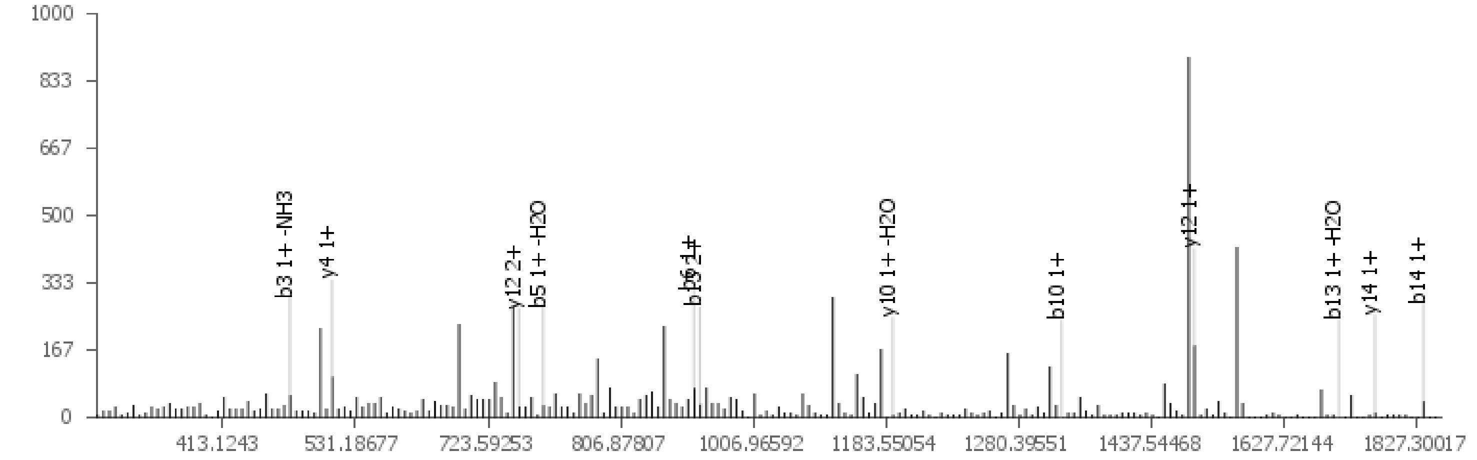

AT4G36250.1 - (pS)FAP(pT)LID(oxM)LKP(pT)IK - 965.921373 - Charge:2

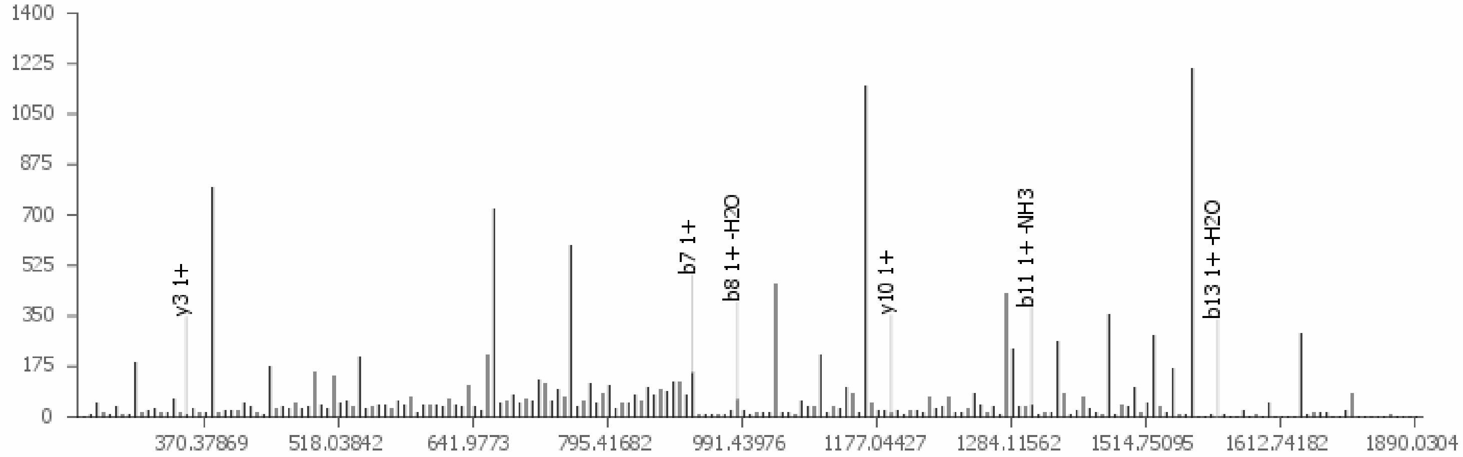

AT3G04800.1 - DGDDG(s)L(t)(t)VIAGLATGVL(pY)R - 1167.486661 - Charge:2

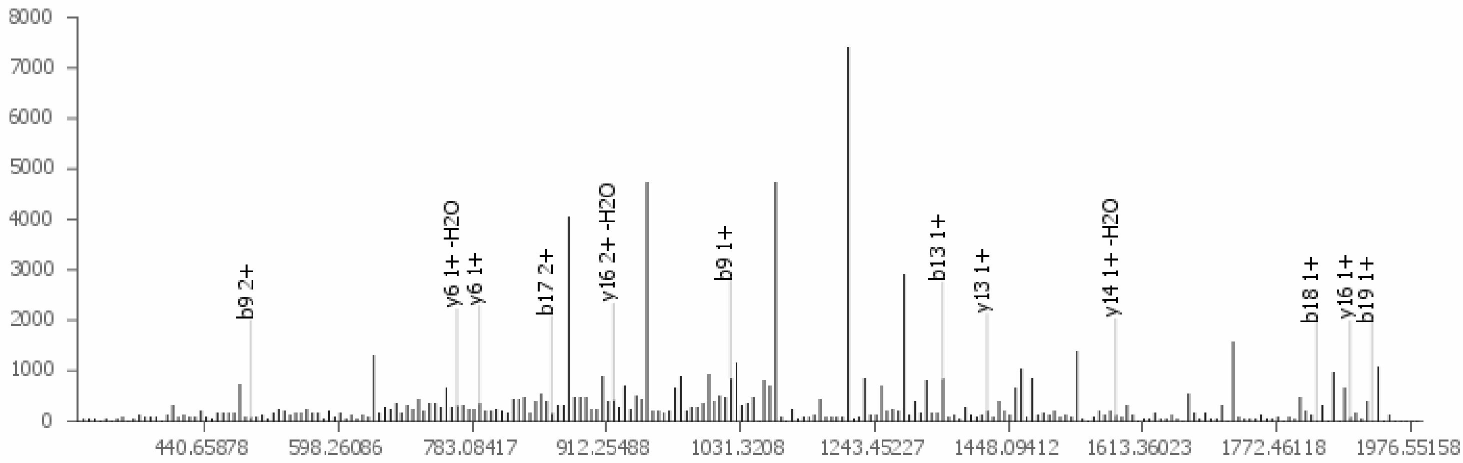

AT3G04950.1 - (pS)I(pT)STAPLNGWMDSIK - 940.895064 - Charge:2

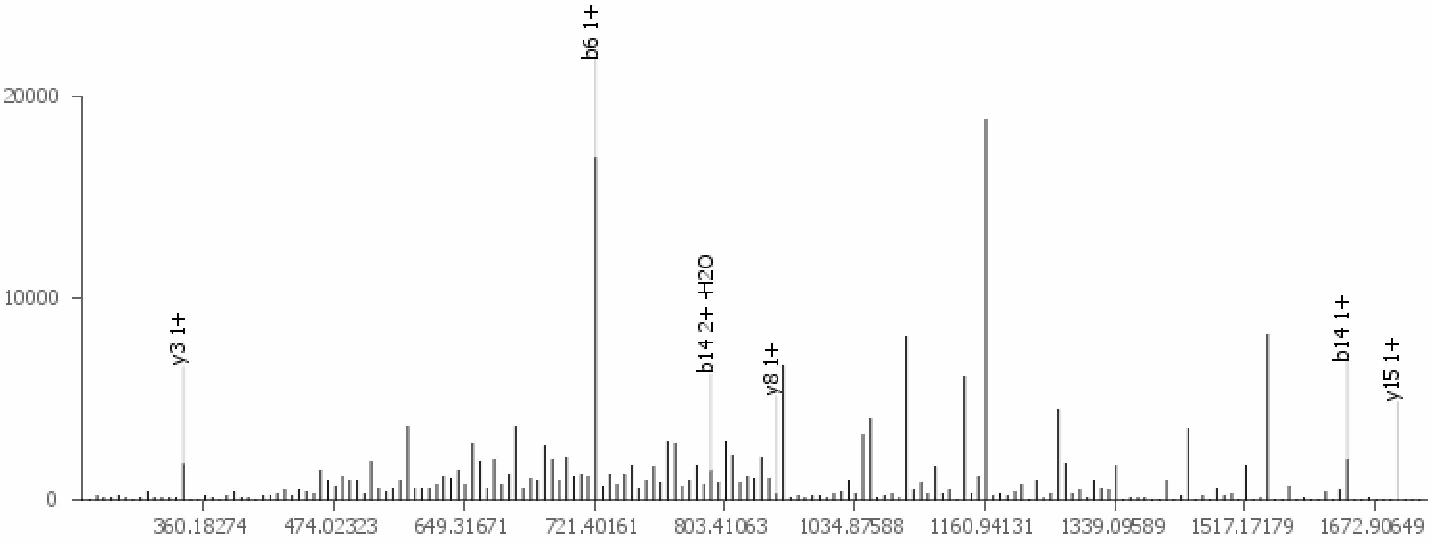

AT5G23720.1 - SYDTGLM(pS)P(oxM)SDR - 778.294834 - Charge:2

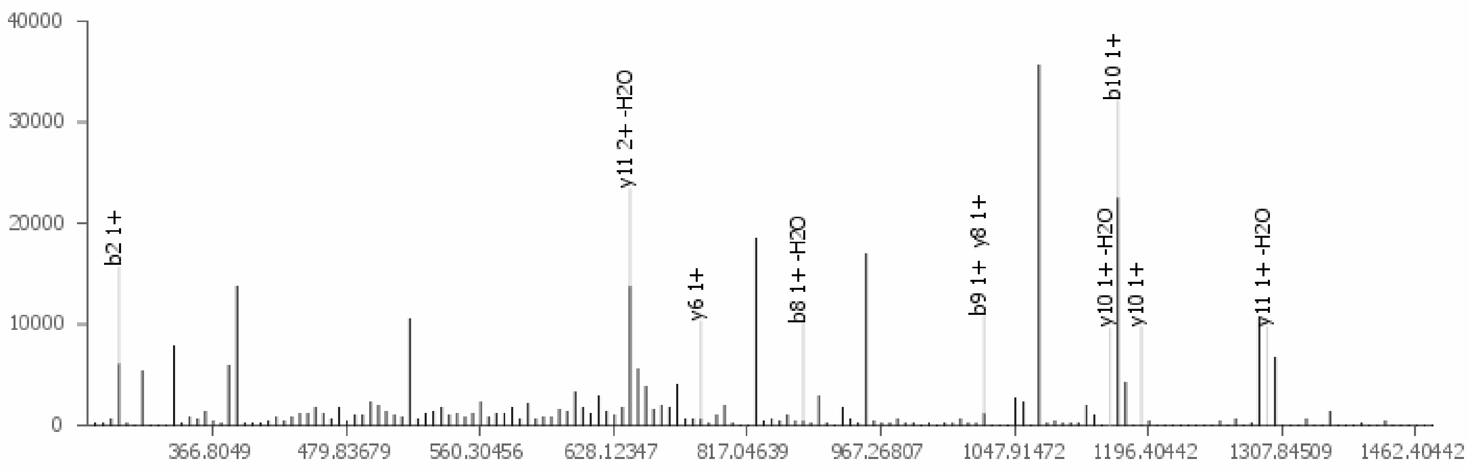

AT1G23190.1 - LSGTG(pS)EGATIR - 614.778351 - Charge:2

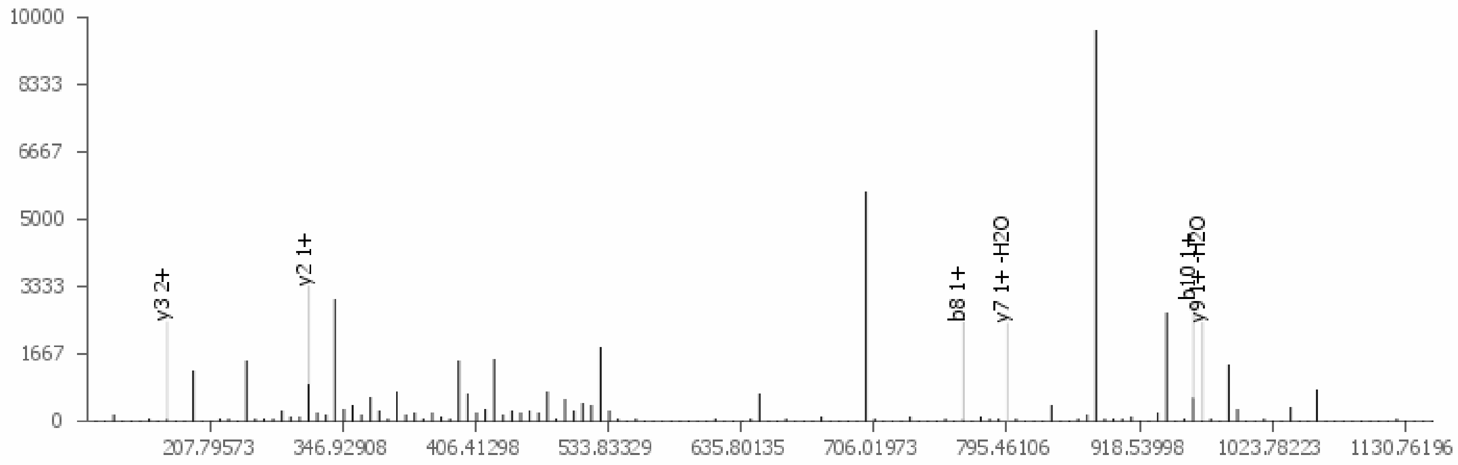

AT4G19515.1 - Q(pT)LKEI(pY)QI(pT)K - 802.843327 - Charge:2

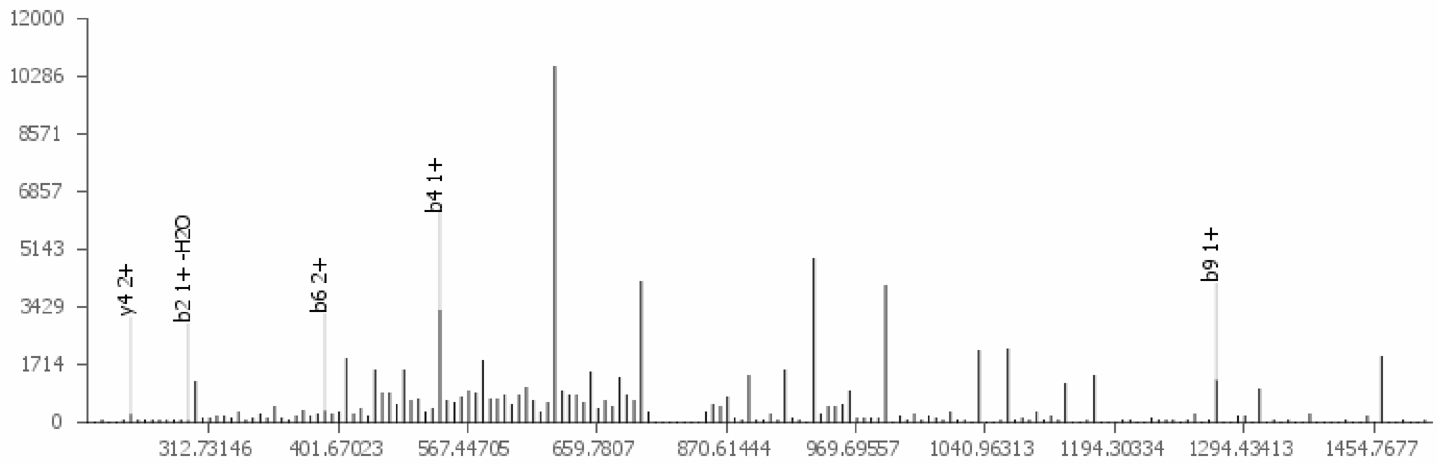

AT3G61480.1 - ADLQL(pS)(pT)VRELSI(oxM)(pT)AK - 1066.457932 - Charge:2

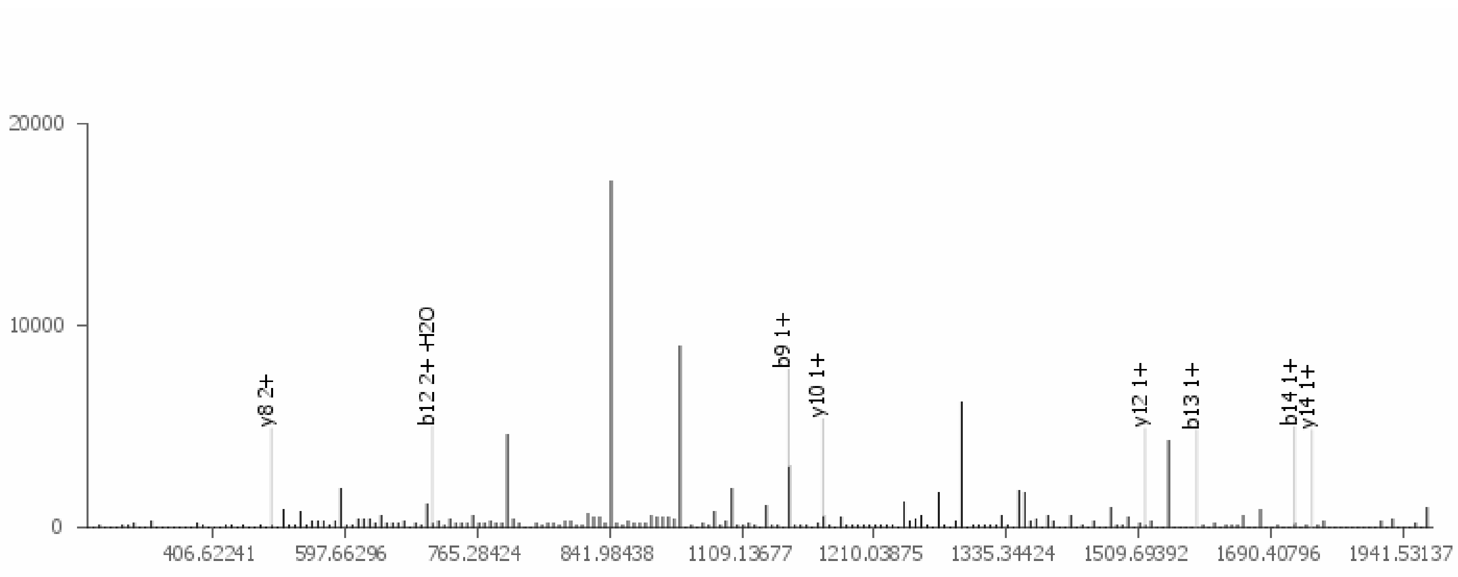

AT5G22740.1 - (pY)QIRENR - 529.743231 - Charge:2

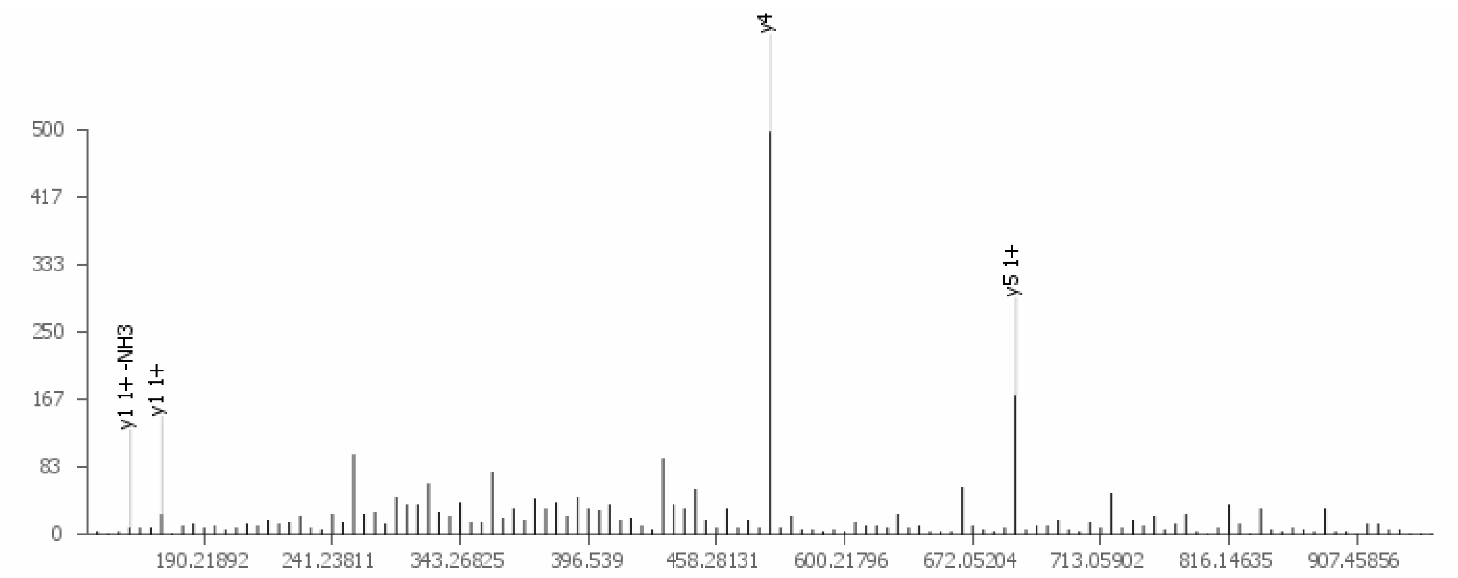

AT5G47950.1 - MD(pT)MKVETIGK - 666.79419 - Charge:2

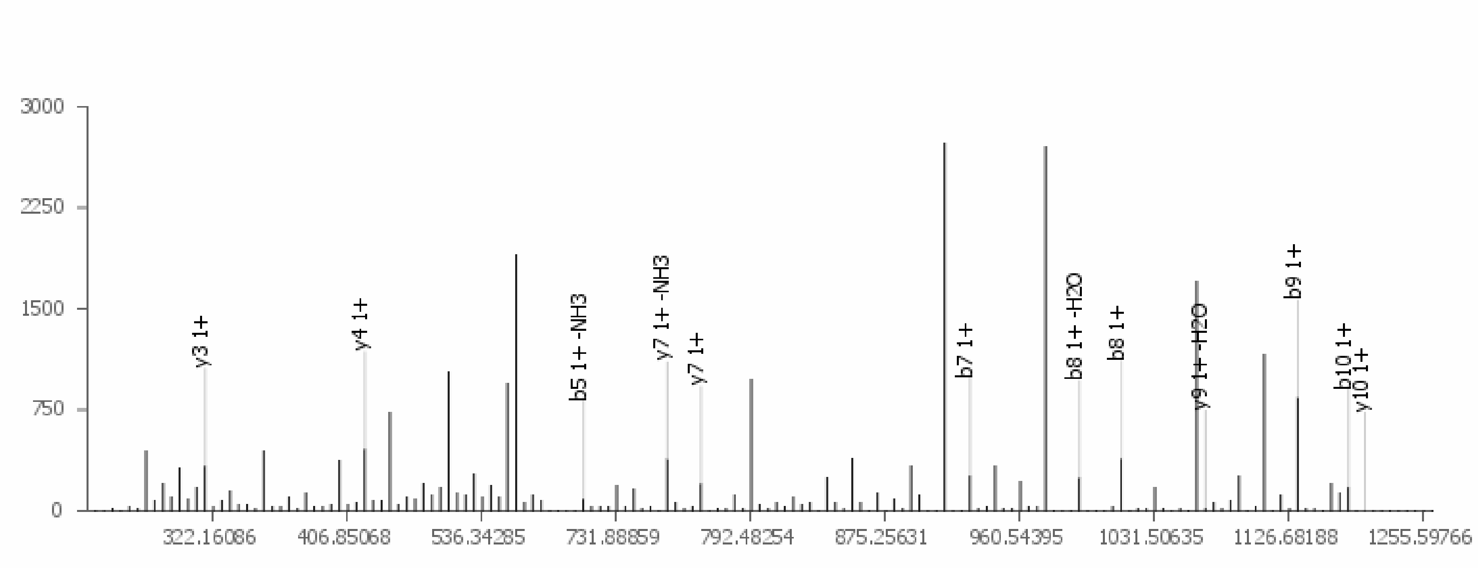

AT3G62900.1 - SPNNLLGESGNAA(s)(s)(s)VPK - 994.915179 - Charge:2

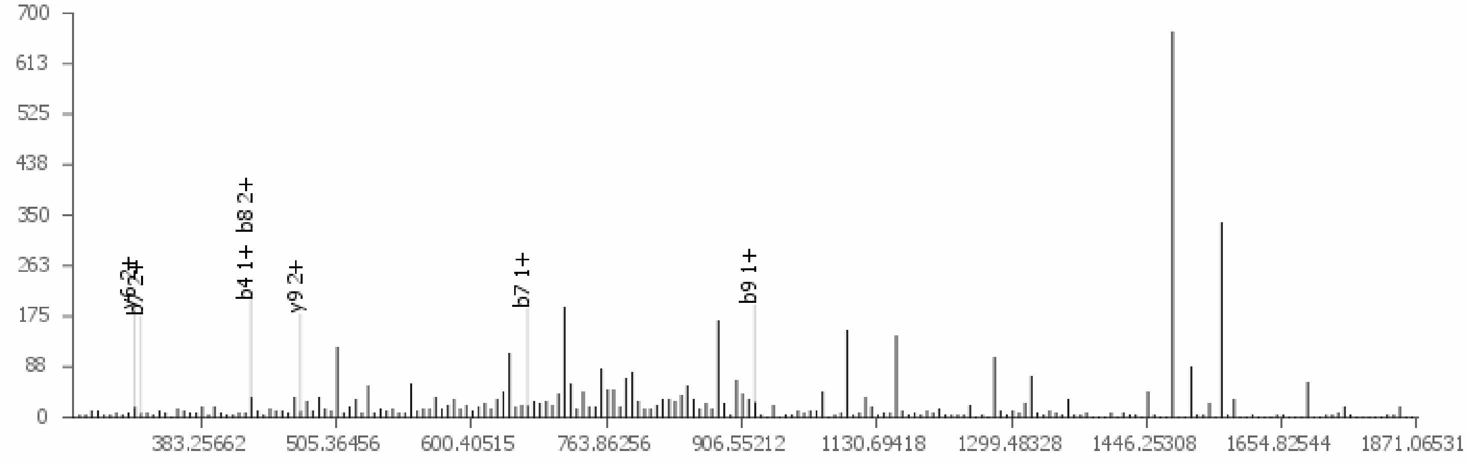

AT5G23720.1 - LHRIDD(oxM)V(pT)(pS)AGER - 888.364632 - Charge:2

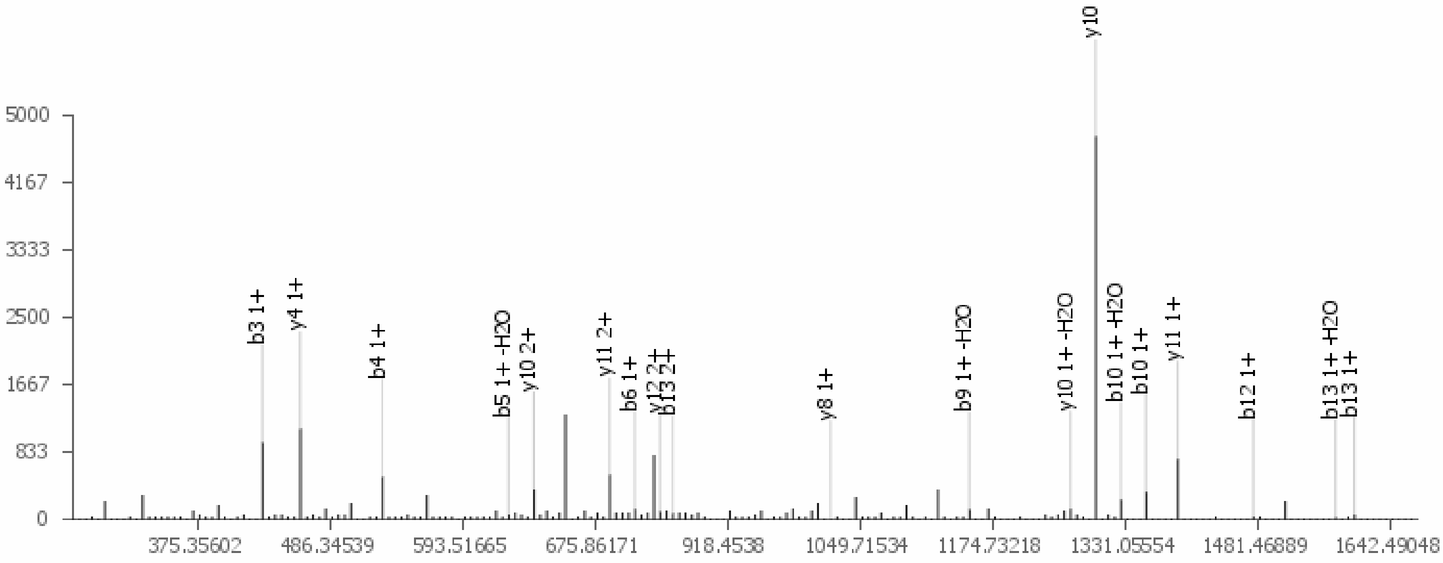

AT1G53380.1 - D(pY)L(pT)ARPK - 562.235948 - Charge:2

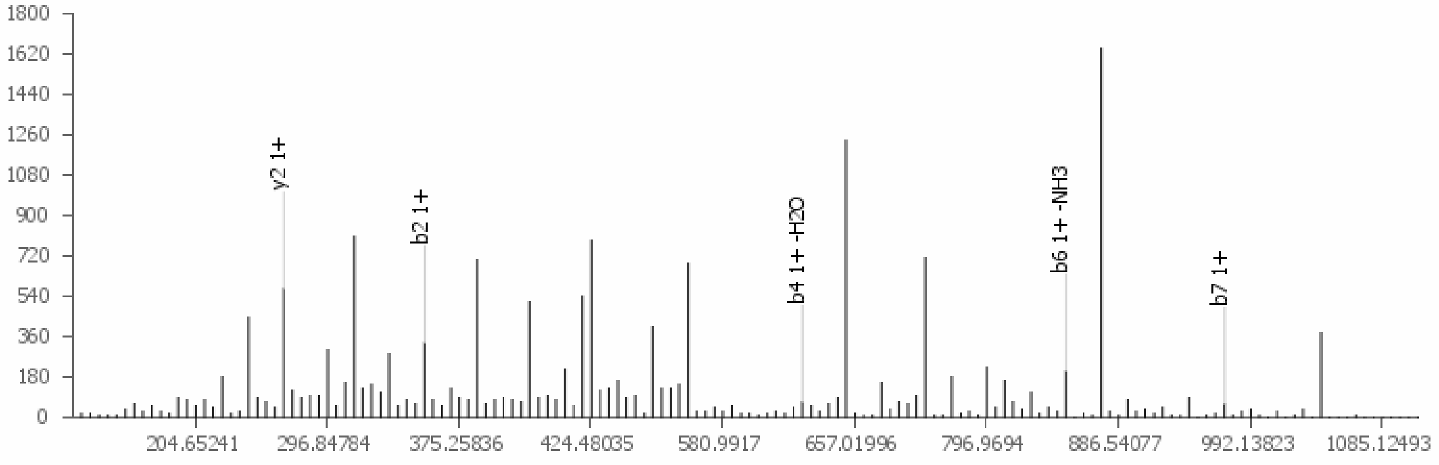

AT5G25070.1 - LAS(pT)(pT)HEELEK - 709.281825 - Charge:2

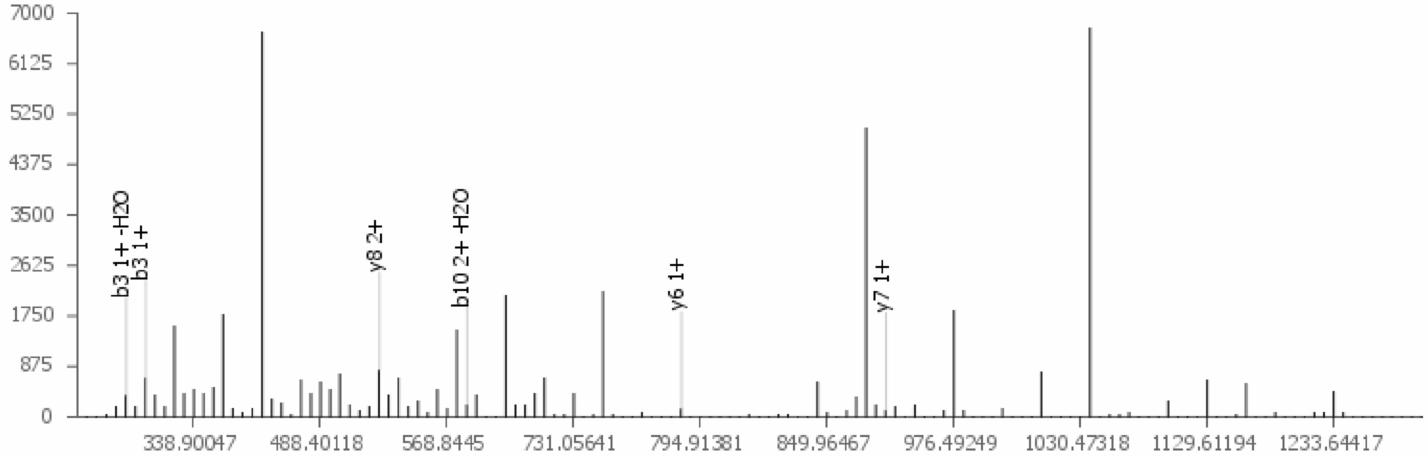

AT1G62290.1 - (oxM)PSPNGE(pS)AVDCSQLSK - 951.88431 - Charge:2

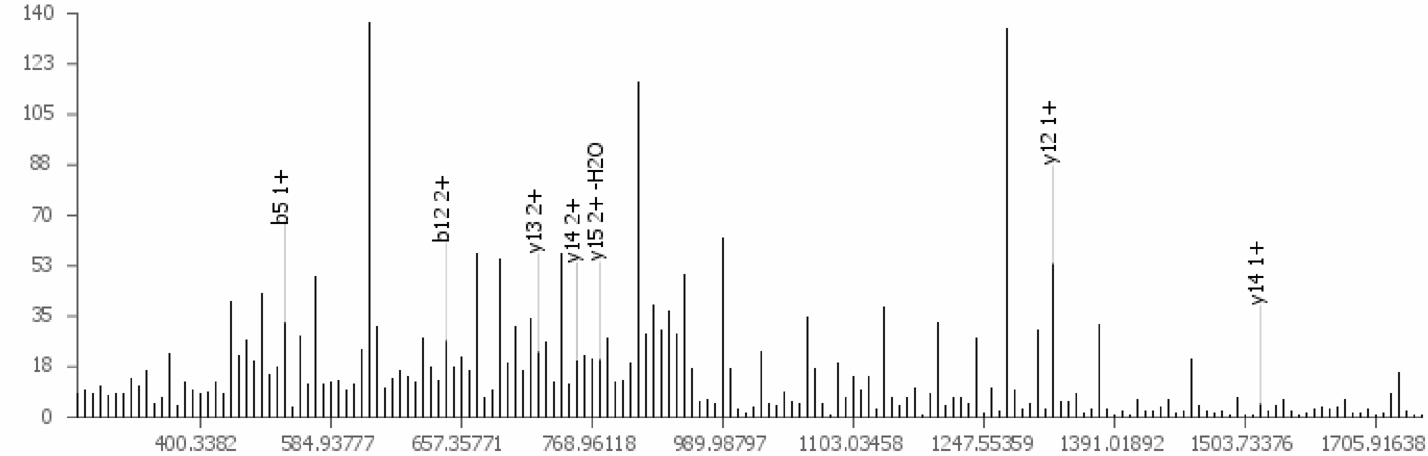

AT1G30780.1 - DPIM(pS)RIL(oxM) - 586.267808 - Charge:2

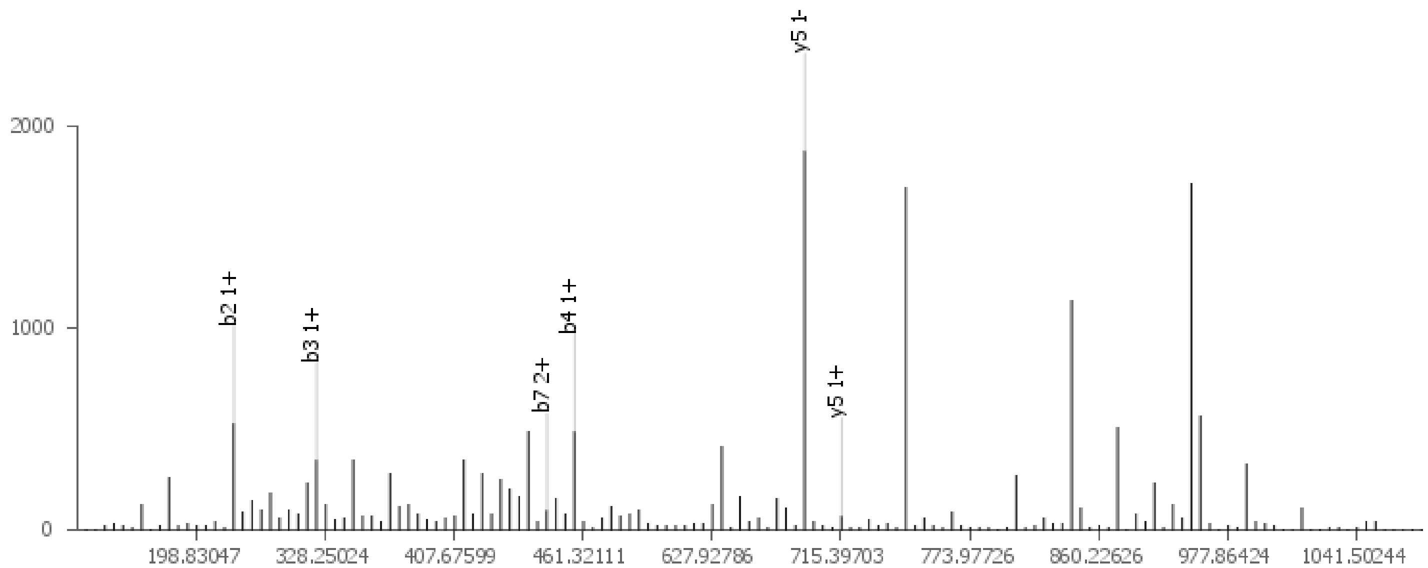

AT2G31650.1 - GP(t)P(s)ACWNK - 599.24064 - Charge:2

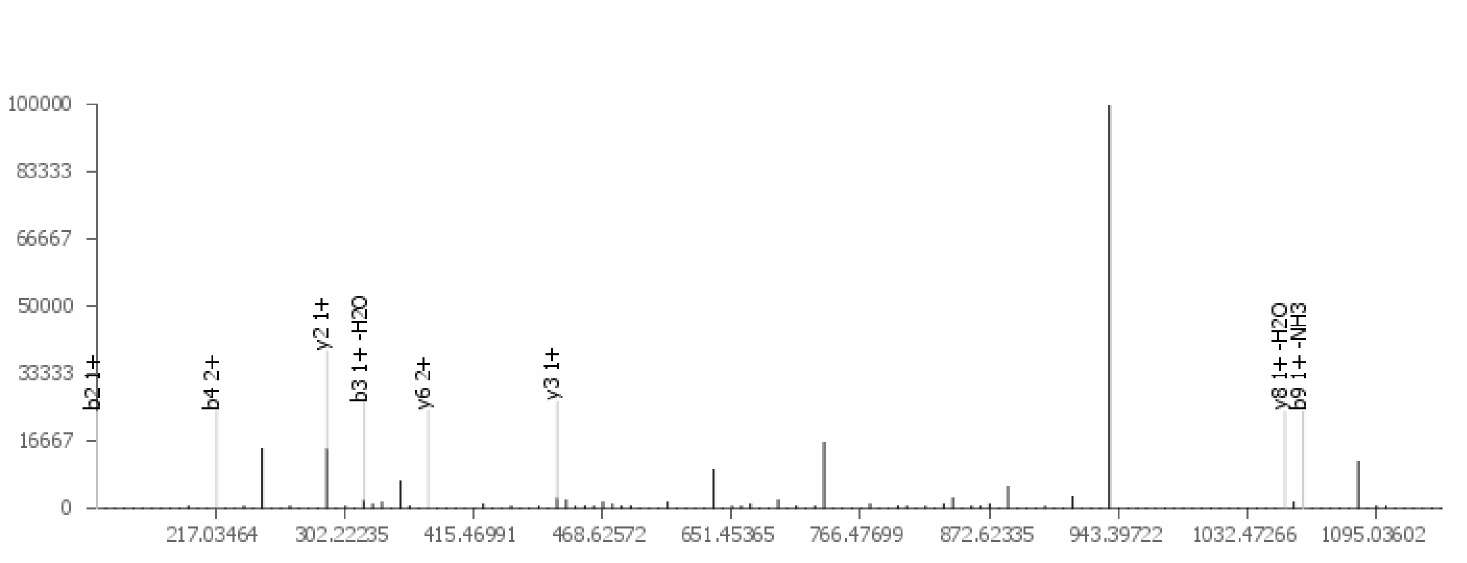

AT5G45610.1 - DMA(pS)LTVDAATR - 665.786895 - Charge:2

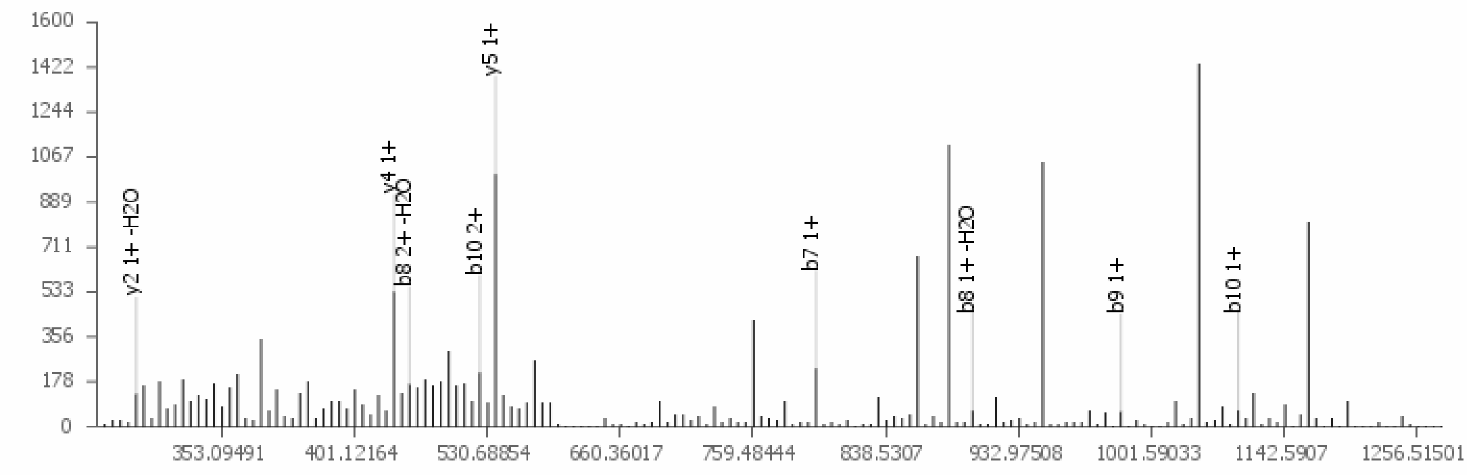

AT3G54510.1 - EYS(oxM)DAFTISNI(t)RG(s)NK - 1065.471585 - Charge:2

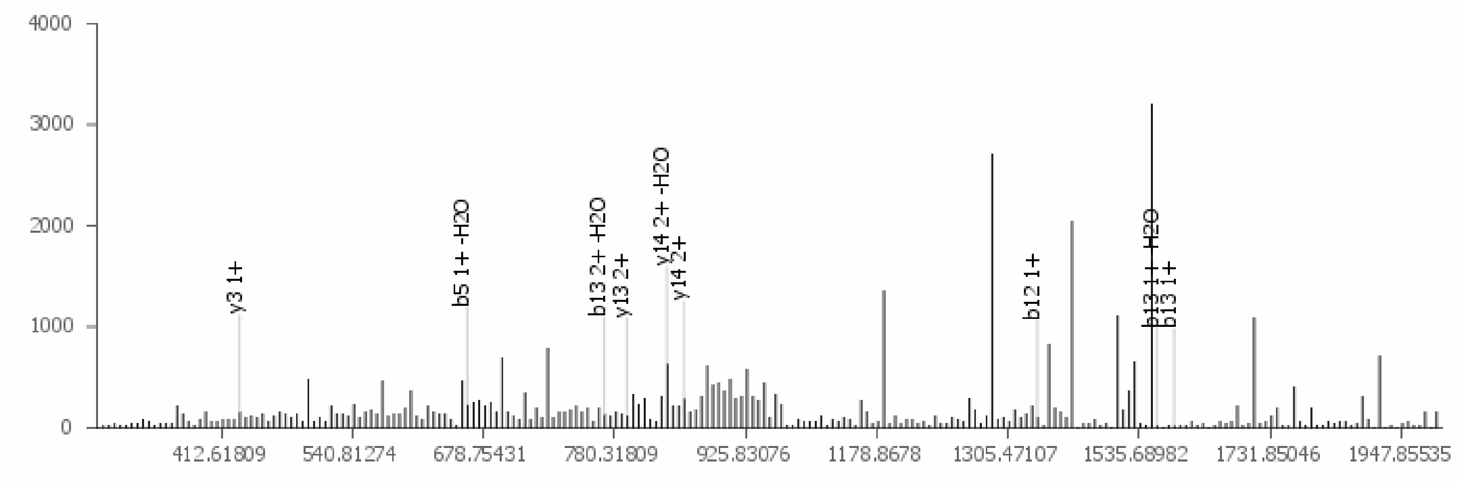

AT5G61700.1 - HDVTQERE(pS)VQK - 768.340474 - Charge:2

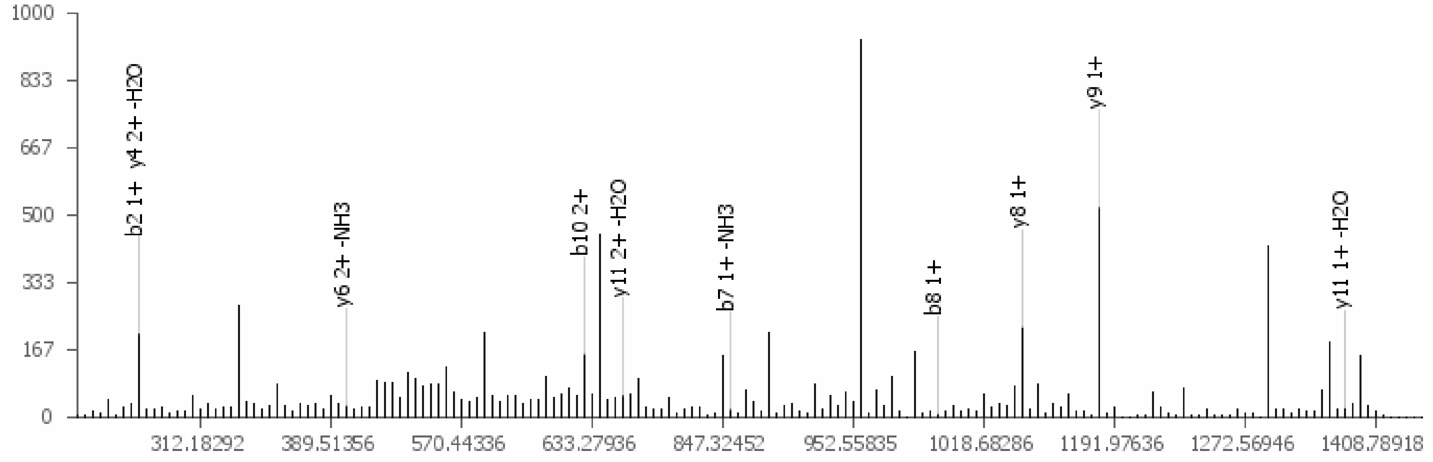

AT1G01580.1 - ILPSDT(oxM)EL(t)F(s)K - 789.357079 - Charge:2

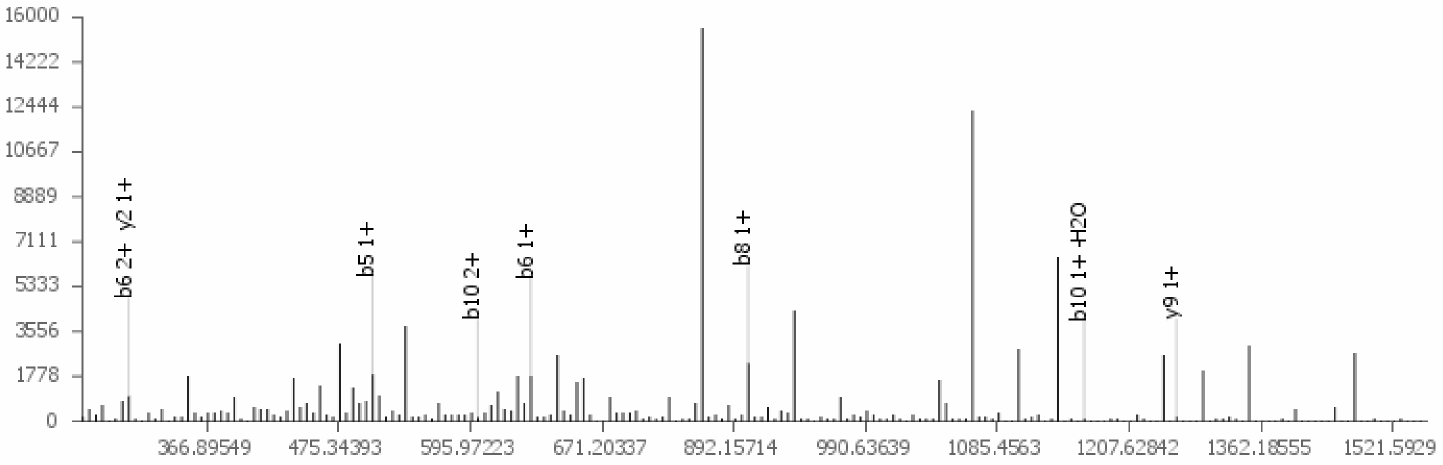

AT3G05360.1 - FGNI(s)(s)(s)(s)R - 517.715325 - Charge:2

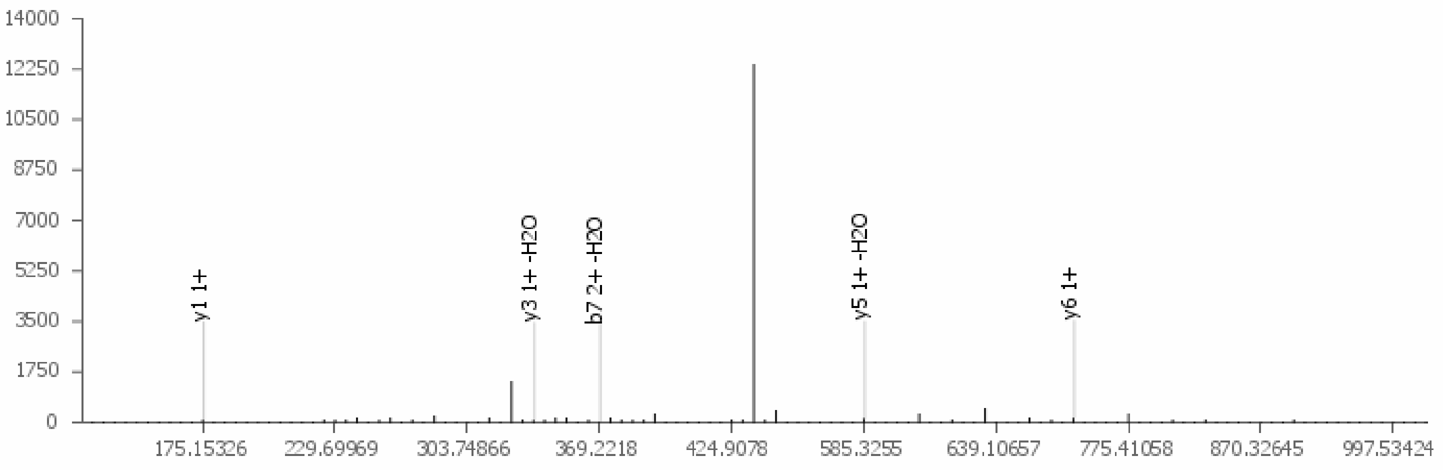

AT2G40840.1 - FNIED(t)(s)(s)FQDLDDH(pS)K - 1079.410905 - Charge:2

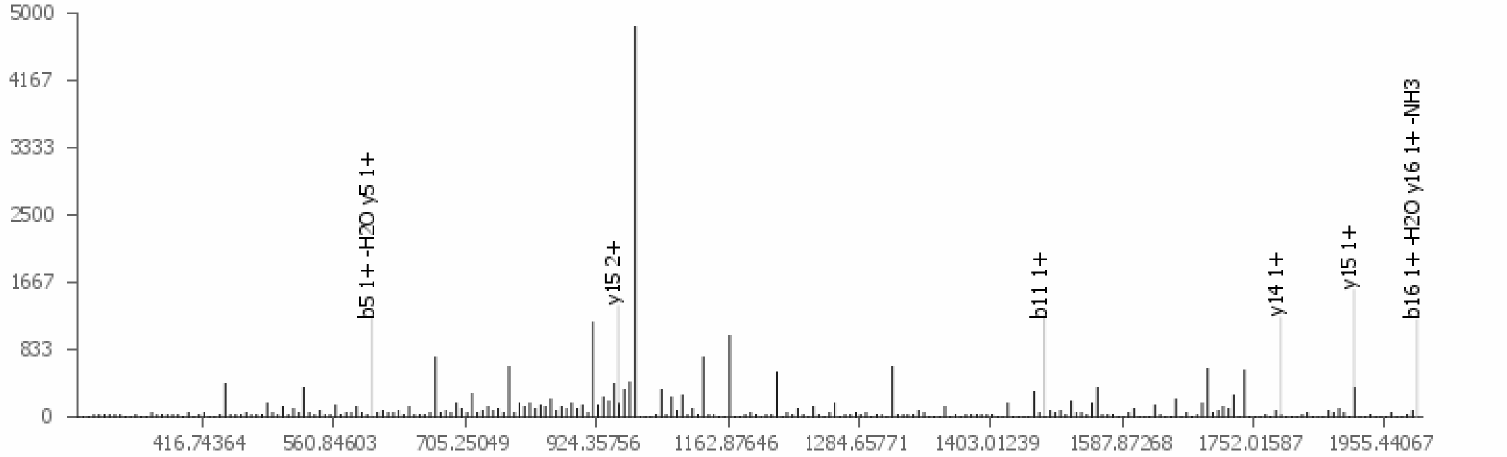

AT5G61460.1 - (oxM)PQDA(pT)SNVVR - 657.286868 - Charge:2

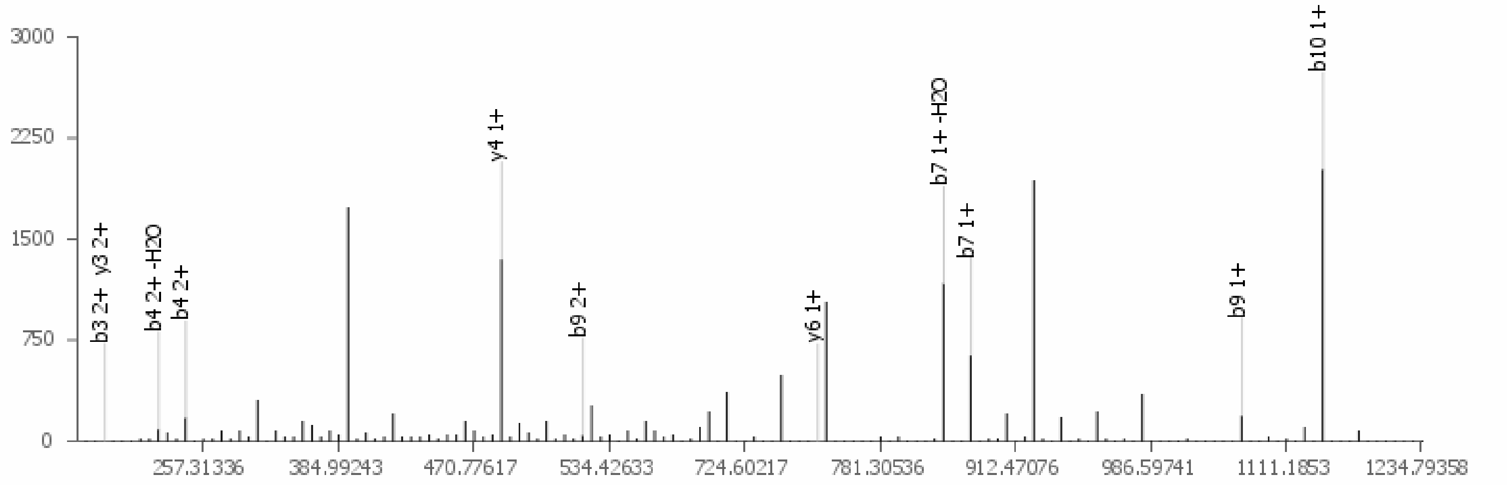

AT3G44630.1 - NLKWMDLSD(pS)R - 722.818583 - Charge:2

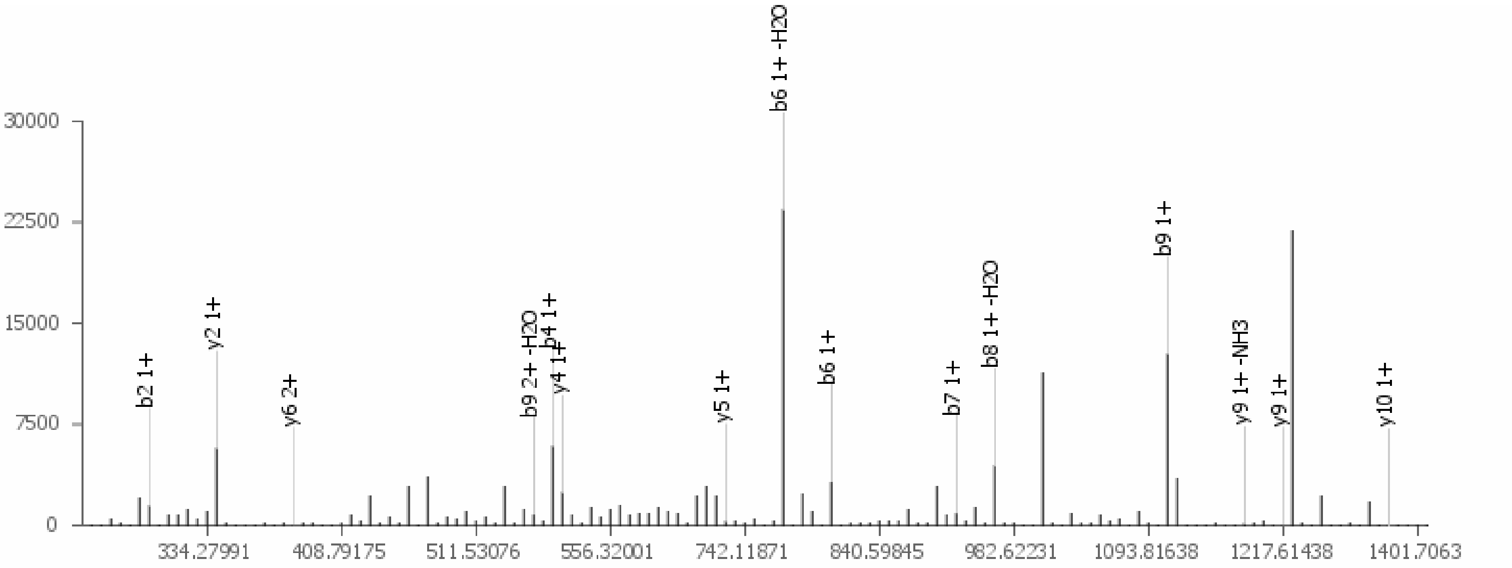

AT3G48970.1 - (oxM)(pS)MTVEIR - 531.719911 - Charge:2

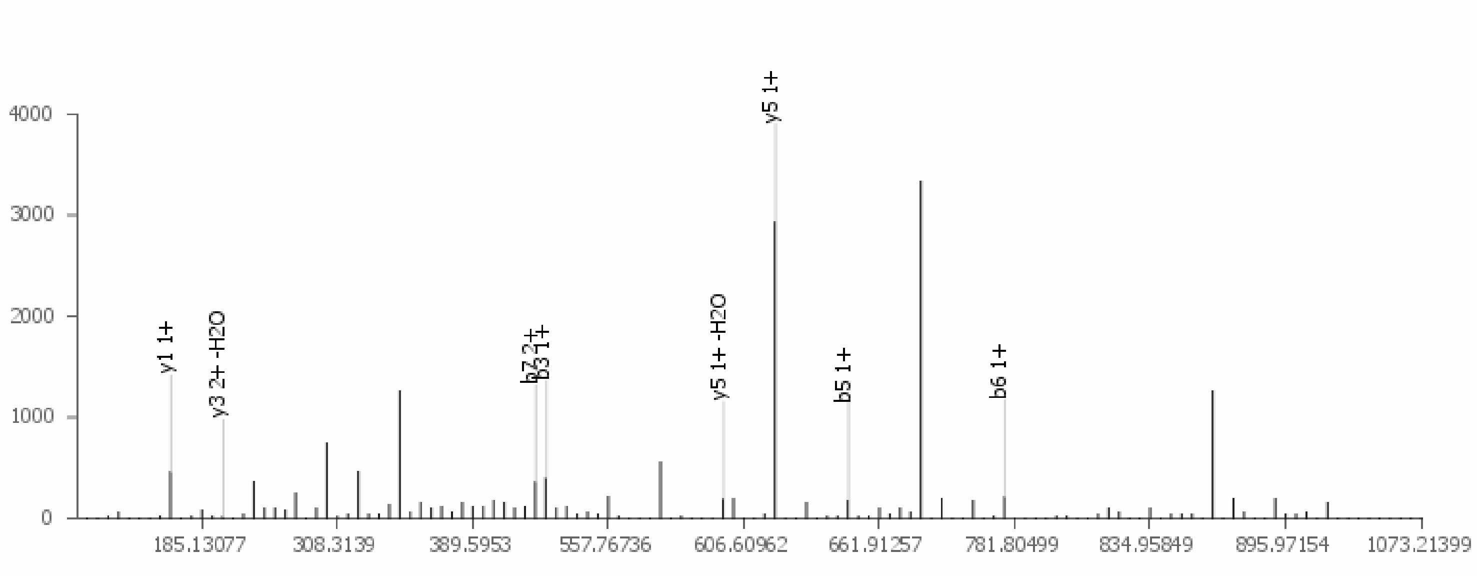

AT4G28410.1 - AVAN(pY)LNR - 500.730077 - Charge:2

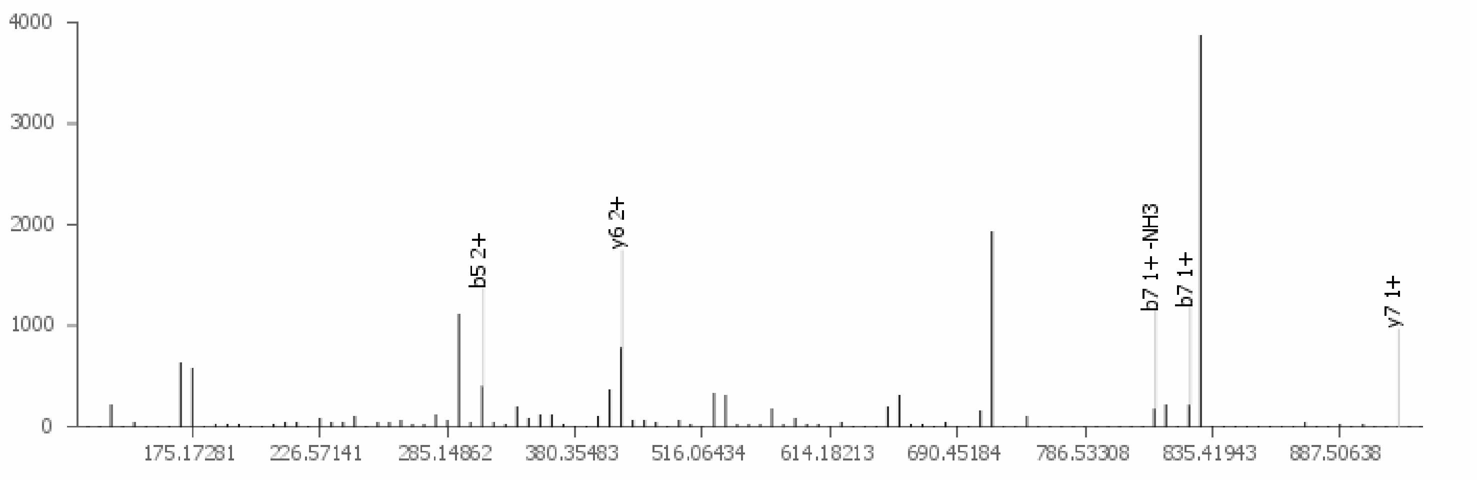

AT1G69020.1 - FVTIN(pS)N(pS)R - 599.233999 - Charge:2

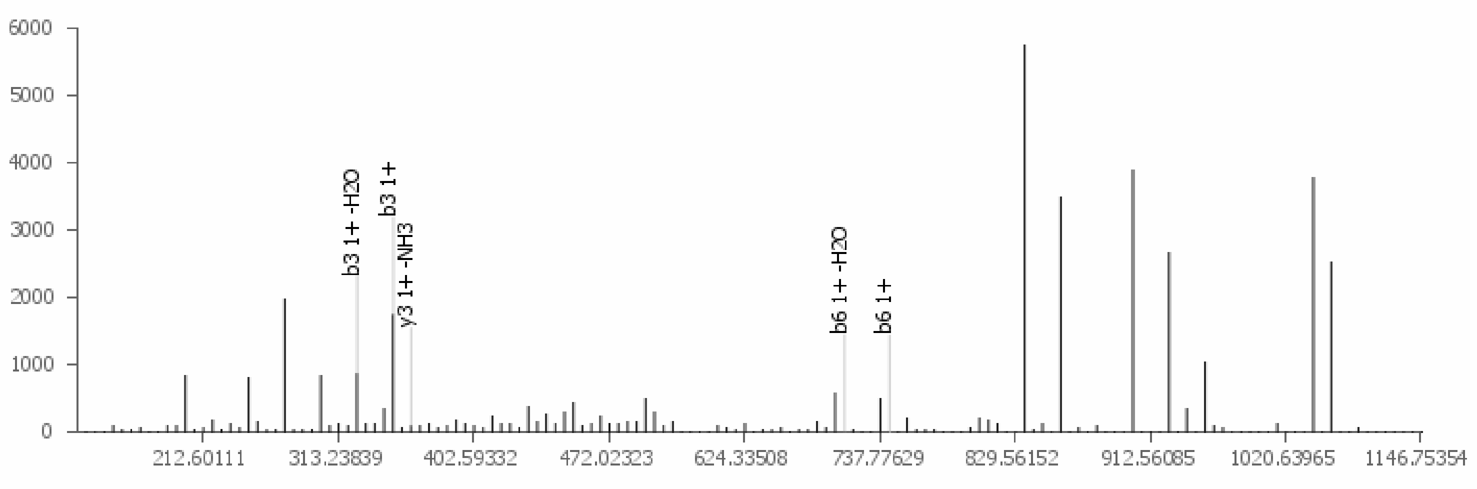

AT3G29180.1 - L(oxM)DHA(pS)FKGLK - 671.81152 - Charge:2

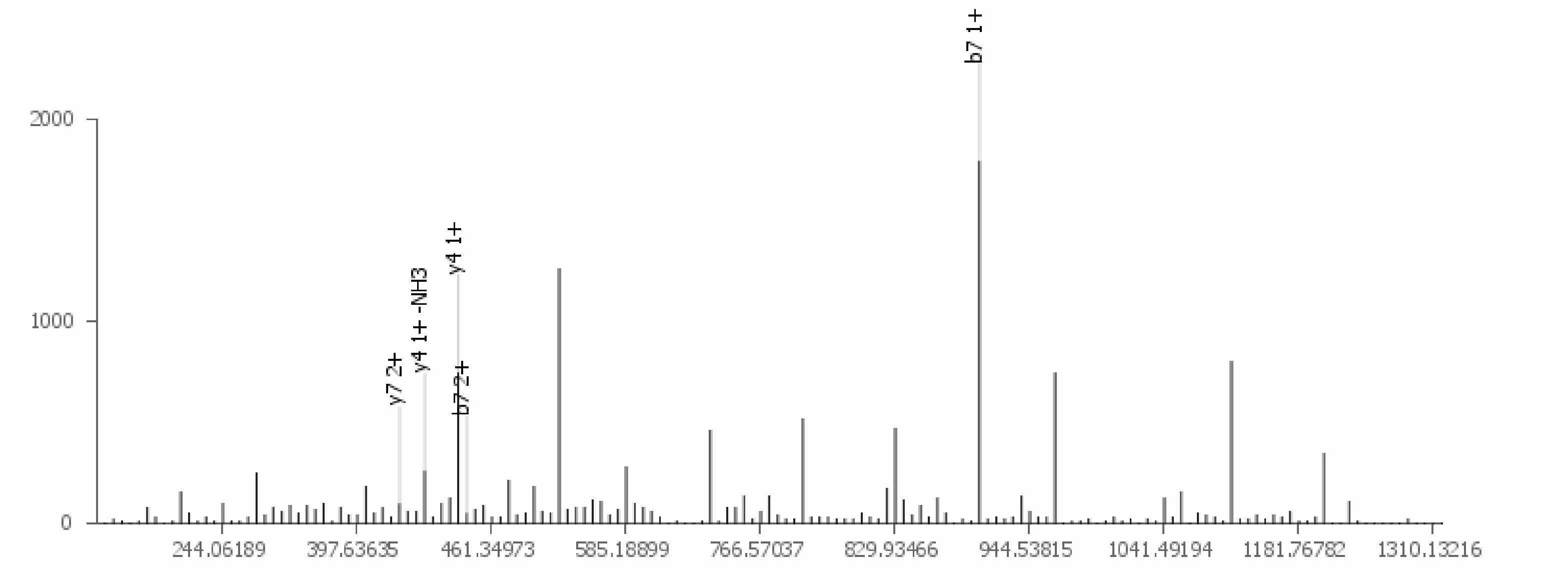

AT3G17340.1 - EATEALDLL(s)(t)K - 685.81935 - Charge:2

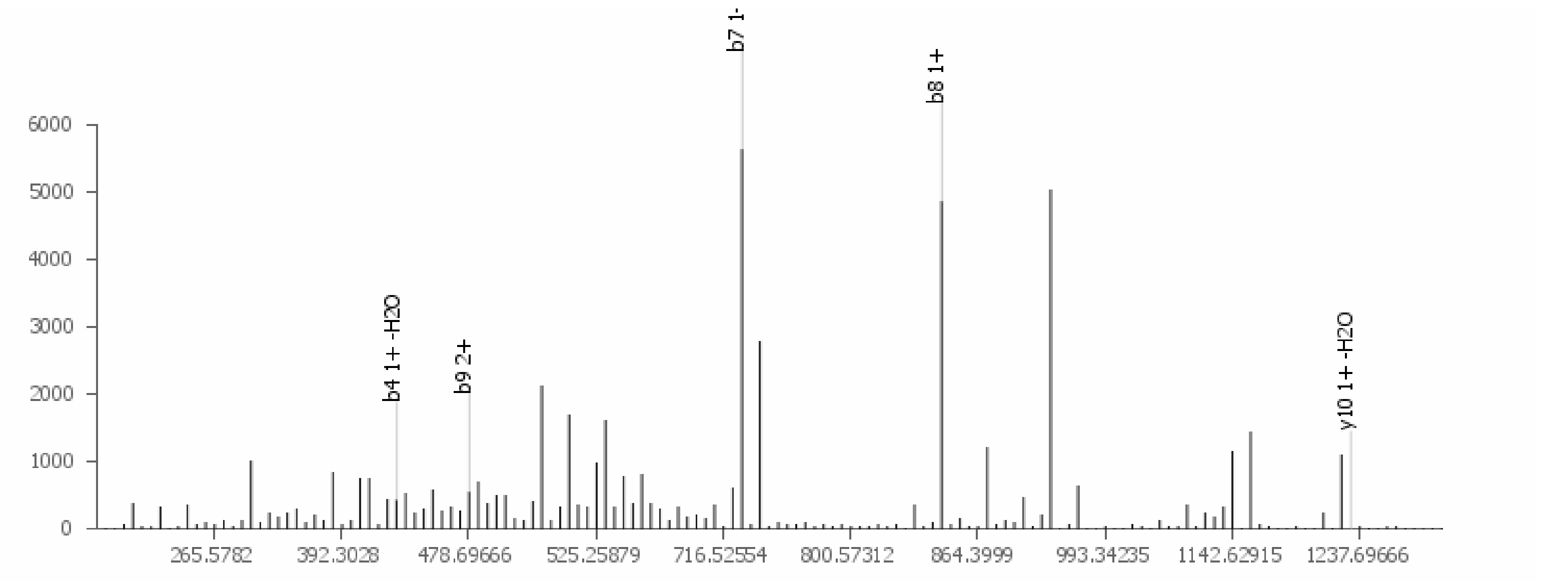

AT3G02710.1 - (oxM)EVSNQ(pT)RK - 594.762307 - Charge:2

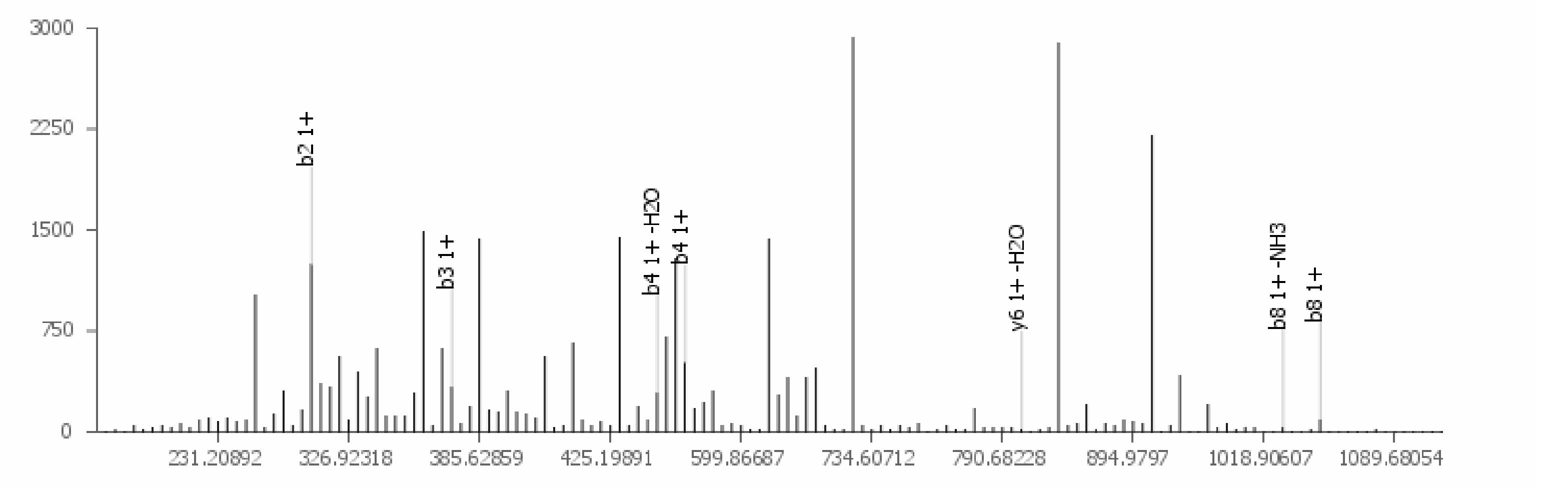

AT3G09310.1 - (pY)VPTCSEY(pS)MEAYKK - 1008.39204 - Charge:2

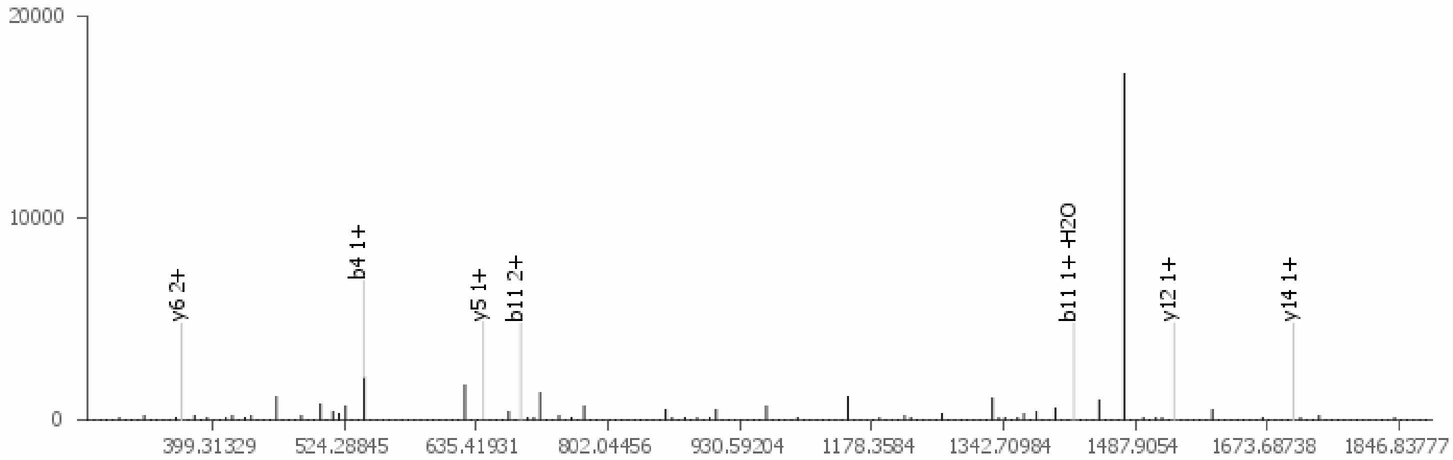

AT1G04710.1 - AA(pS)ATASGKFK - 559.760956 - Charge:2

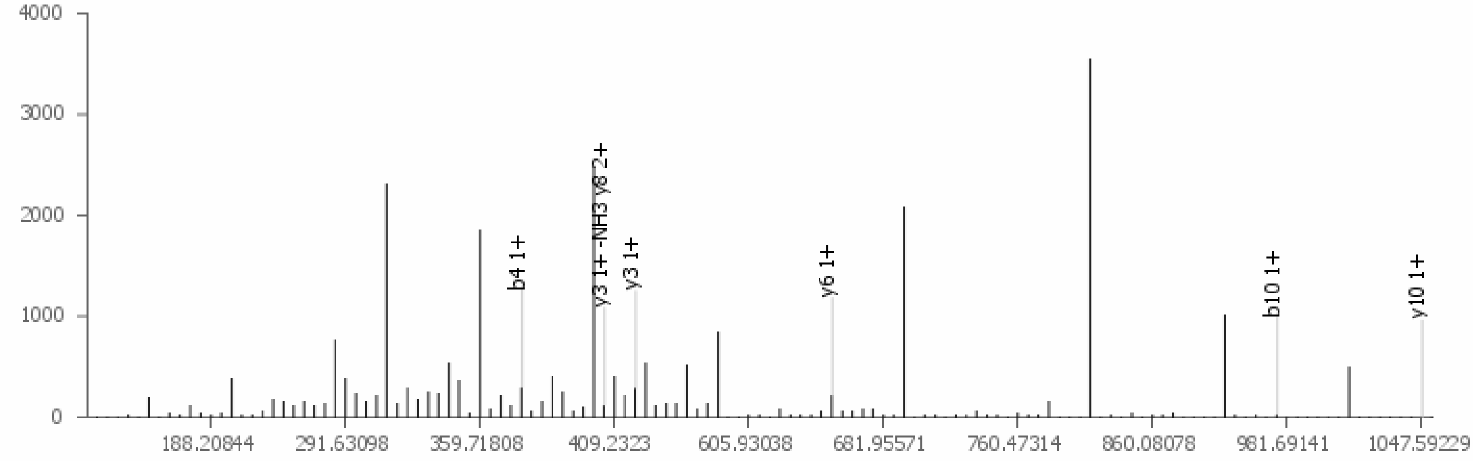

AT5G38670.1 - L(oxM)(s)C(t)ACVLDEK - 761.796126 - Charge:2

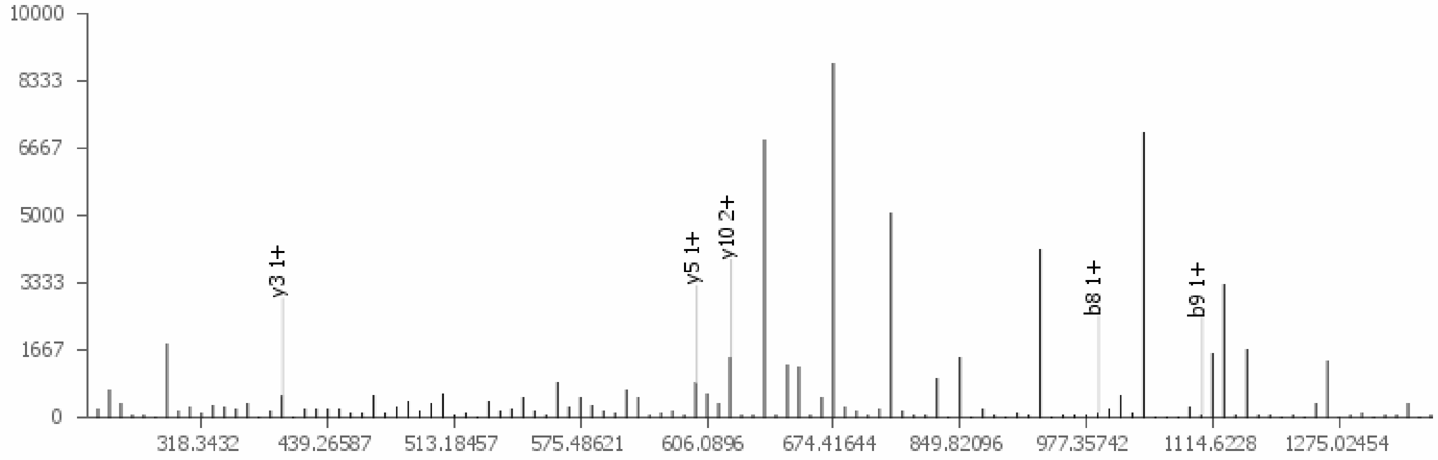

AT4G37980.1 - (m)VVG(pS)(m)VGGIK - 587.275558 - Charge:2

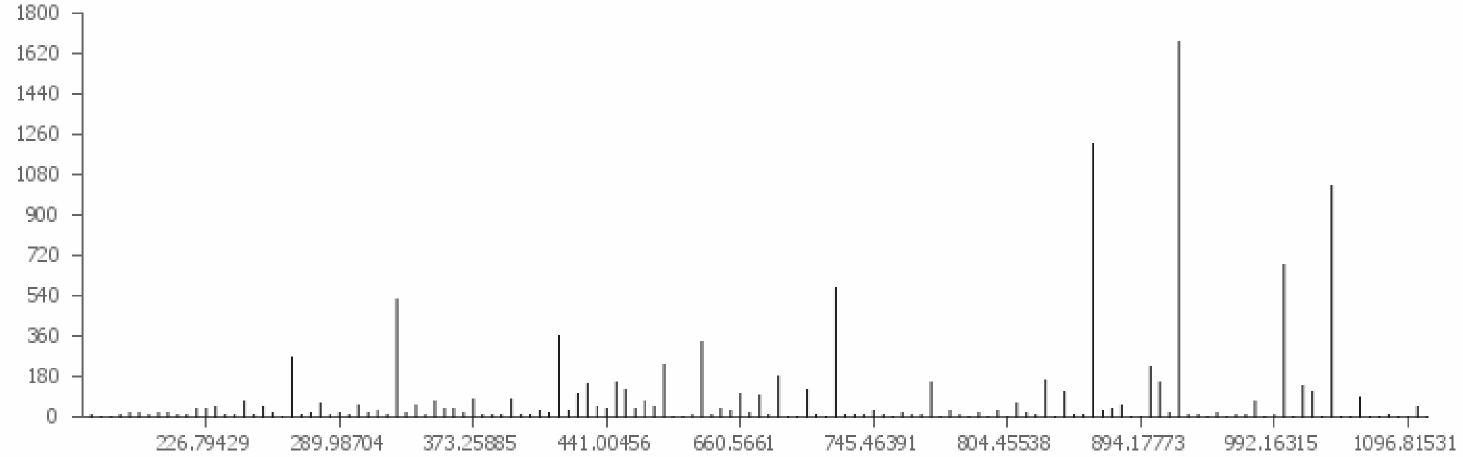

AT5G14530.1 - (pS)(oxM)LL(pT)(pT)TNNNI(pY)VLDA(pY)RGEK - 1416.521141 - Charge:2

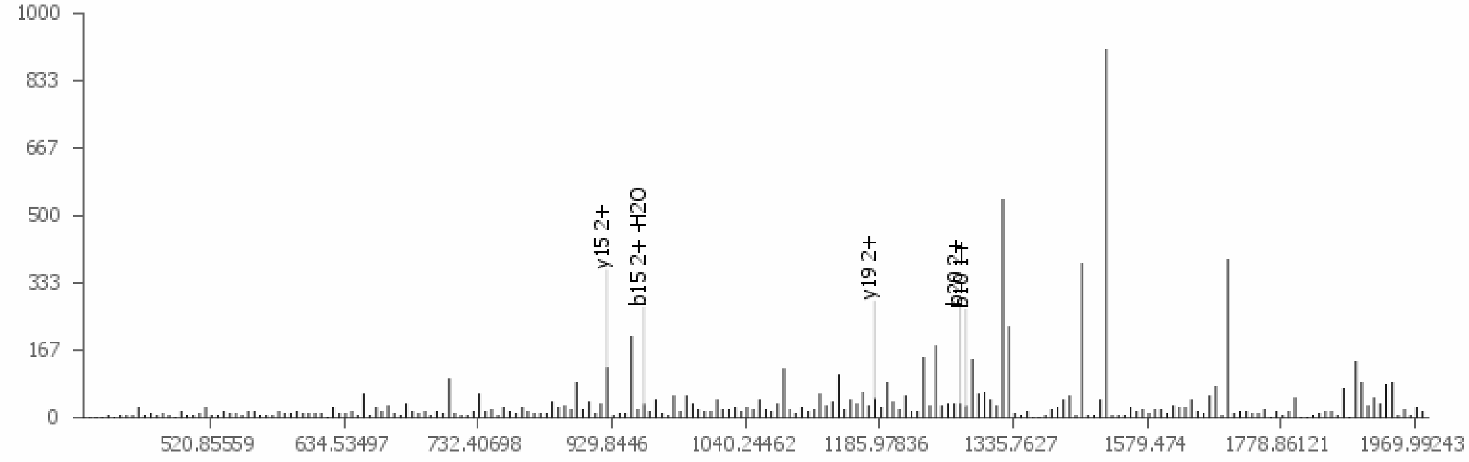

AT5G37850.1 - AGYDP(t)(s)(s)SLEIR - 738.319552 - Charge:2

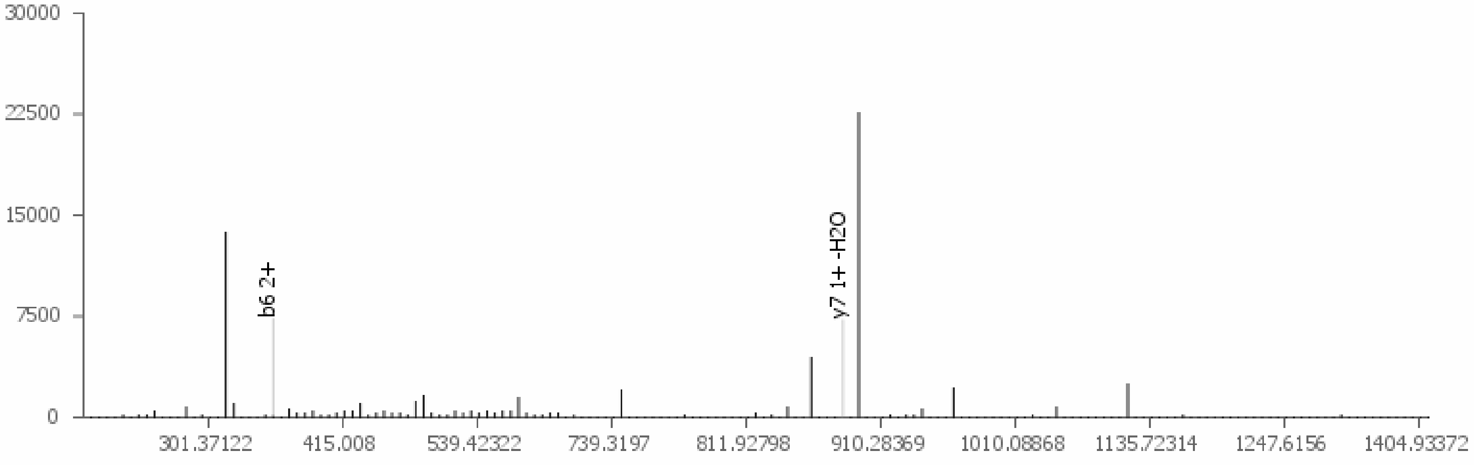

ATCG01110.1 - LSEM(pT)E(pS)IK - 599.234276 - Charge:2

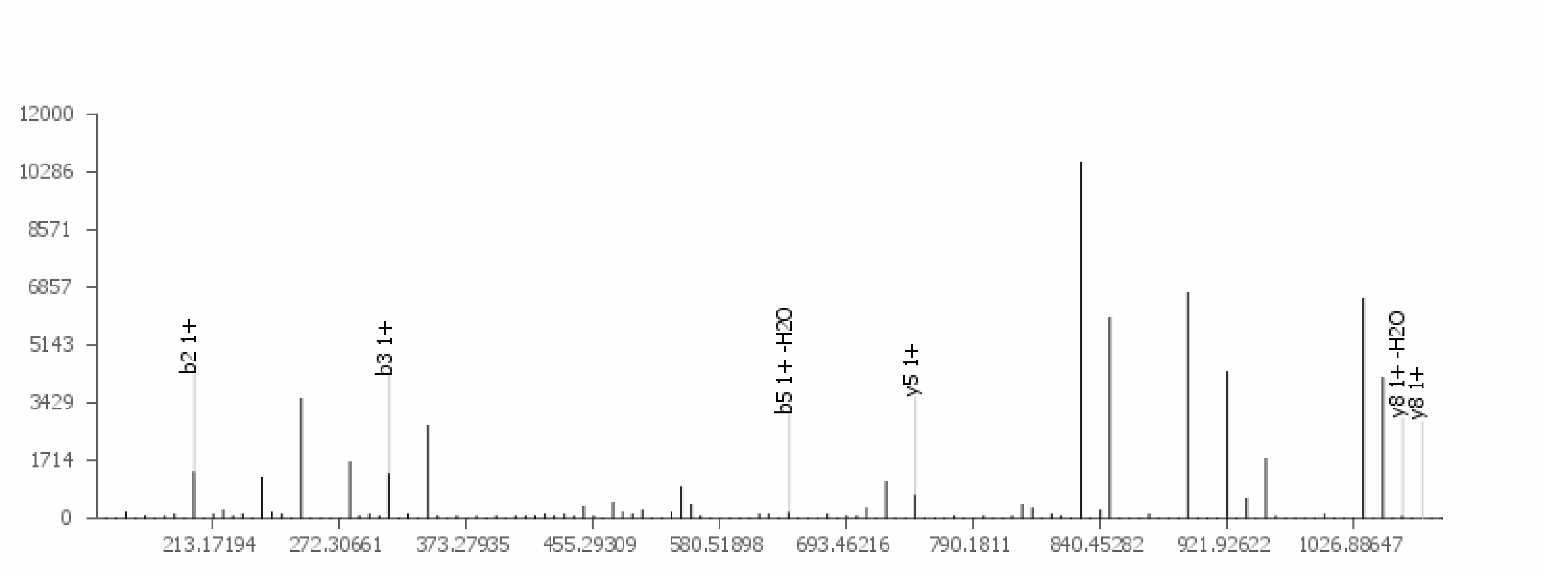

AT4G05190.1 - KEAL(pS)(pS)IPFDK - 697.801548 - Charge:2

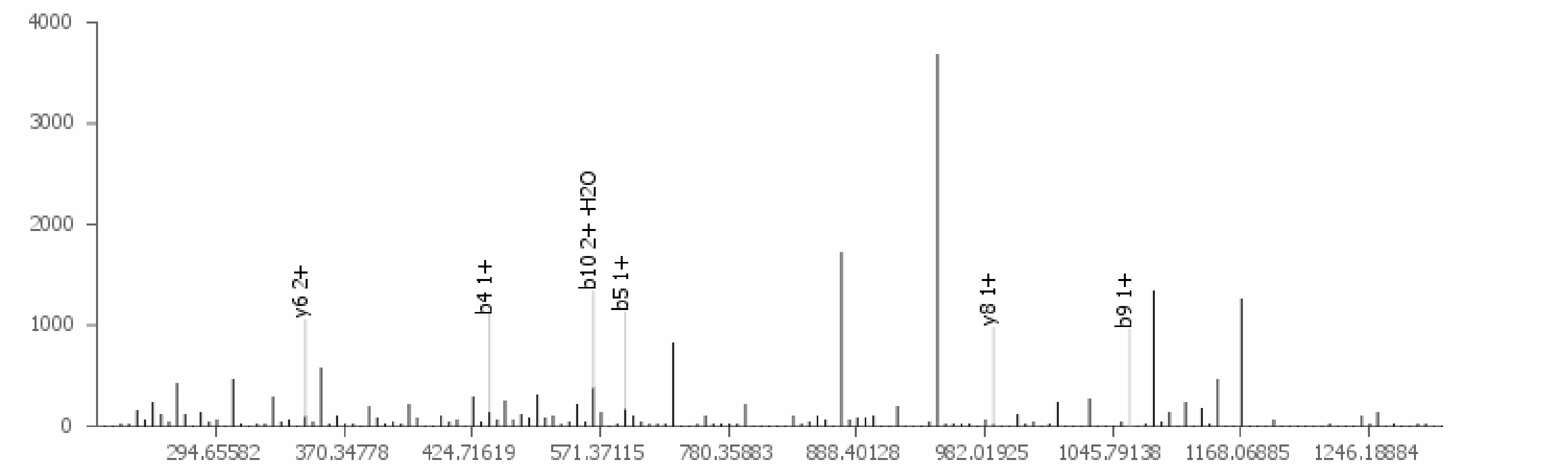

AT1G50030.1 - (pY)QW(pS)LGEER - 664.243582 - Charge:2

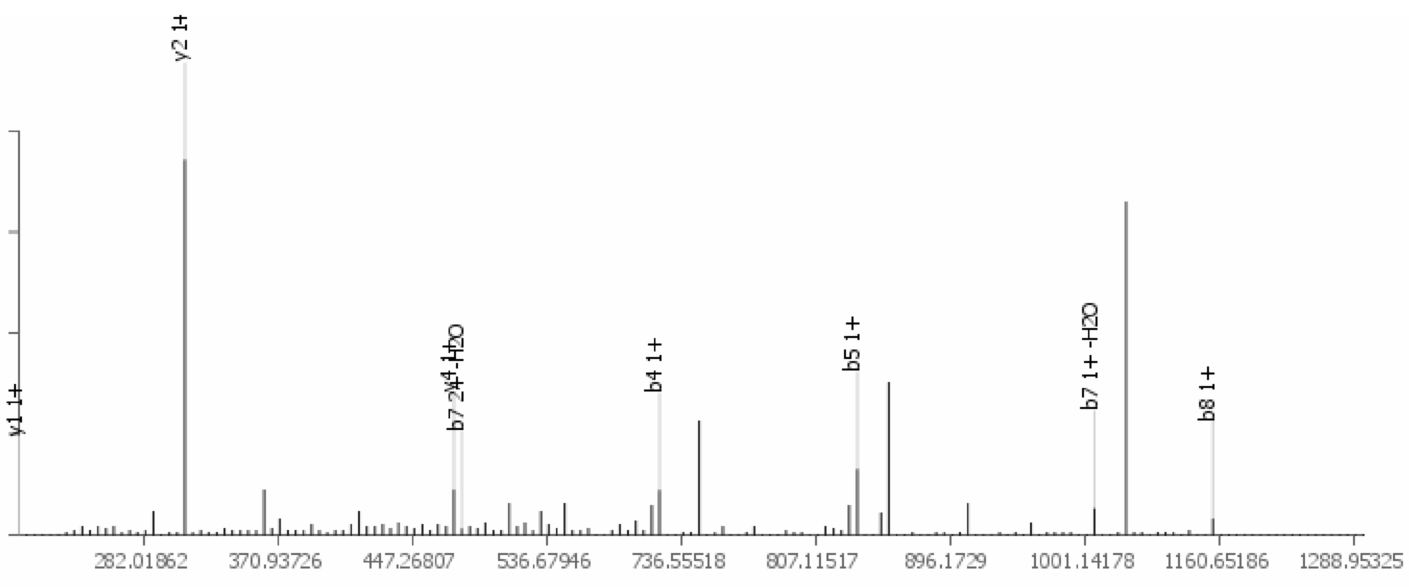

AT1G05870.1 - (pS)FVAGEGSR - 495.212758 - Charge:2

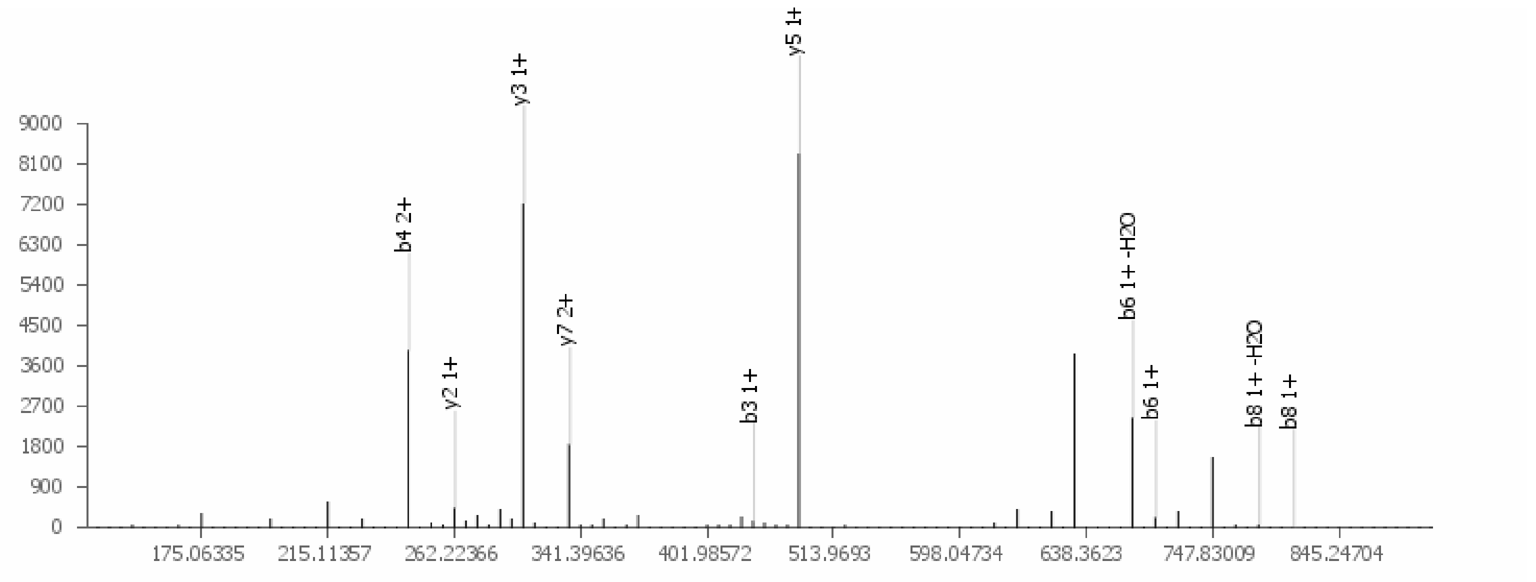

AT3G19180.1 - DKLLFE(pS)E(pY)AGNLK - 893.890831 - Charge:2

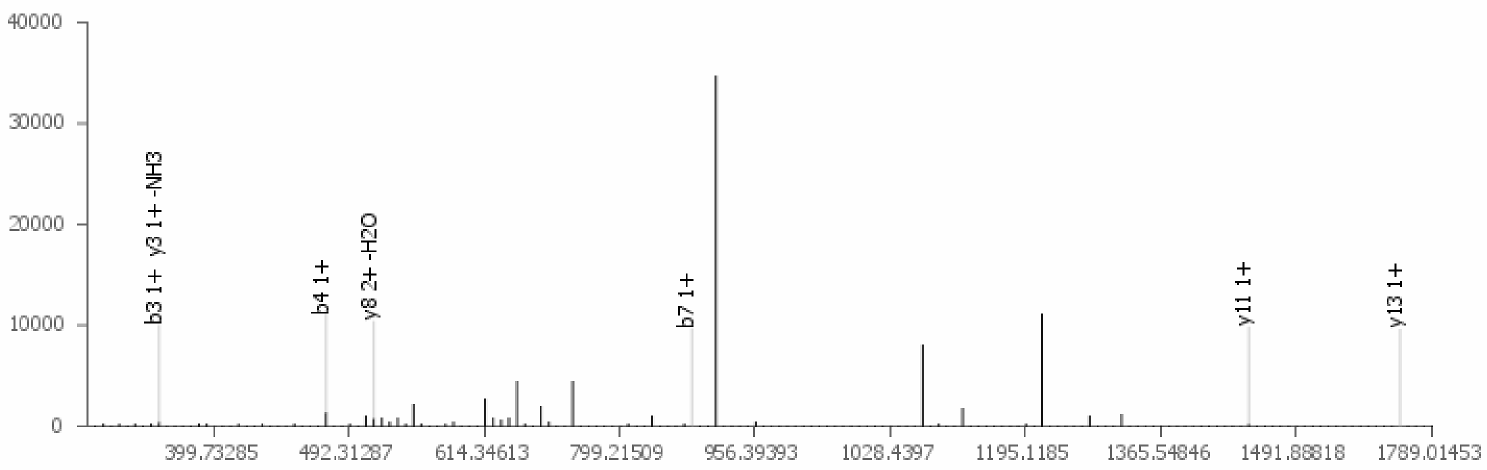

AT2G31170.1 - CF(pT)TL(s)(s)L(t)DGGAPISGGK - 1014.921909 - Charge:2

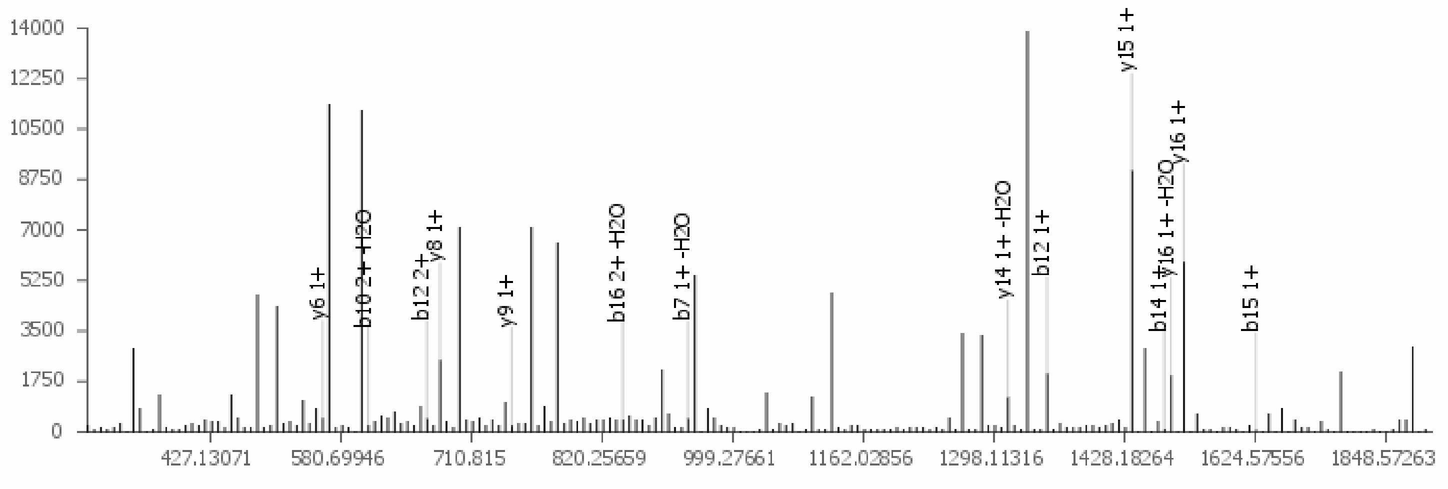

AT1G12970.1 - (pS)VIEAEK - 428.192752 - Charge:2

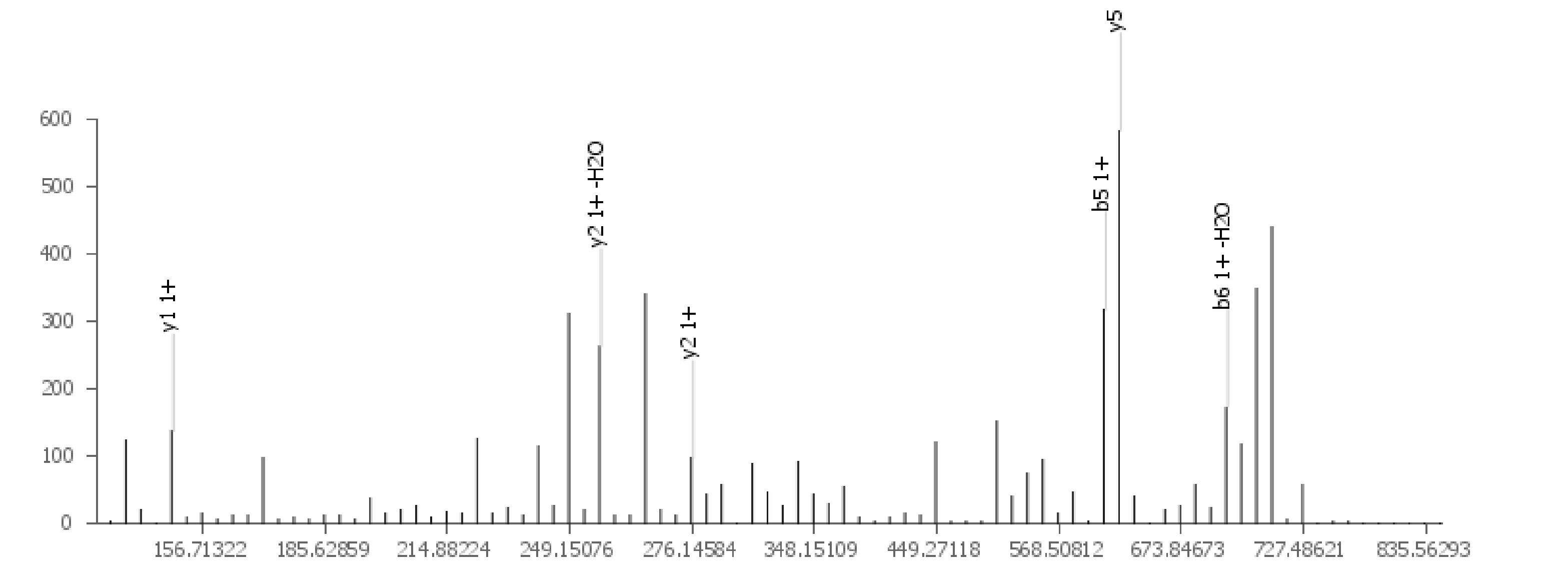

AT4G31400.1 - I(pS)GCLVAEPIK - 633.814336 - Charge:2

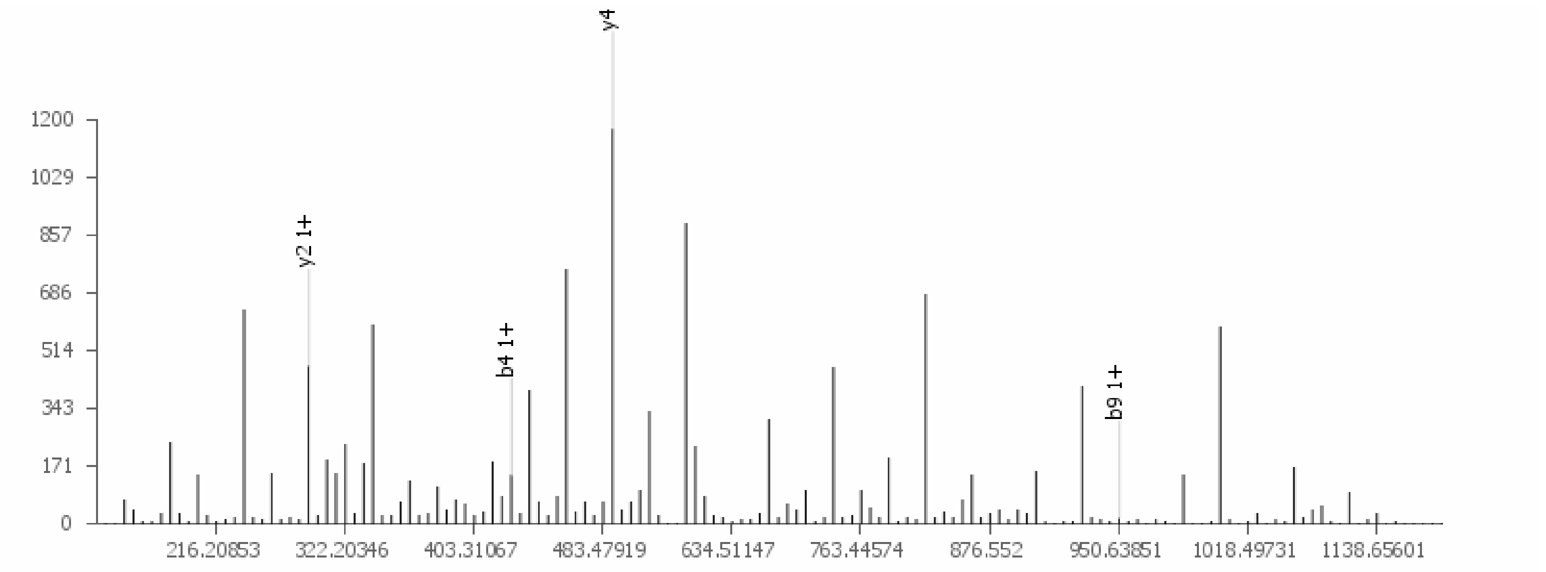

AT4G17190.2 - (oxM)DNSV(pT)RR - 537.720407 - Charge:2

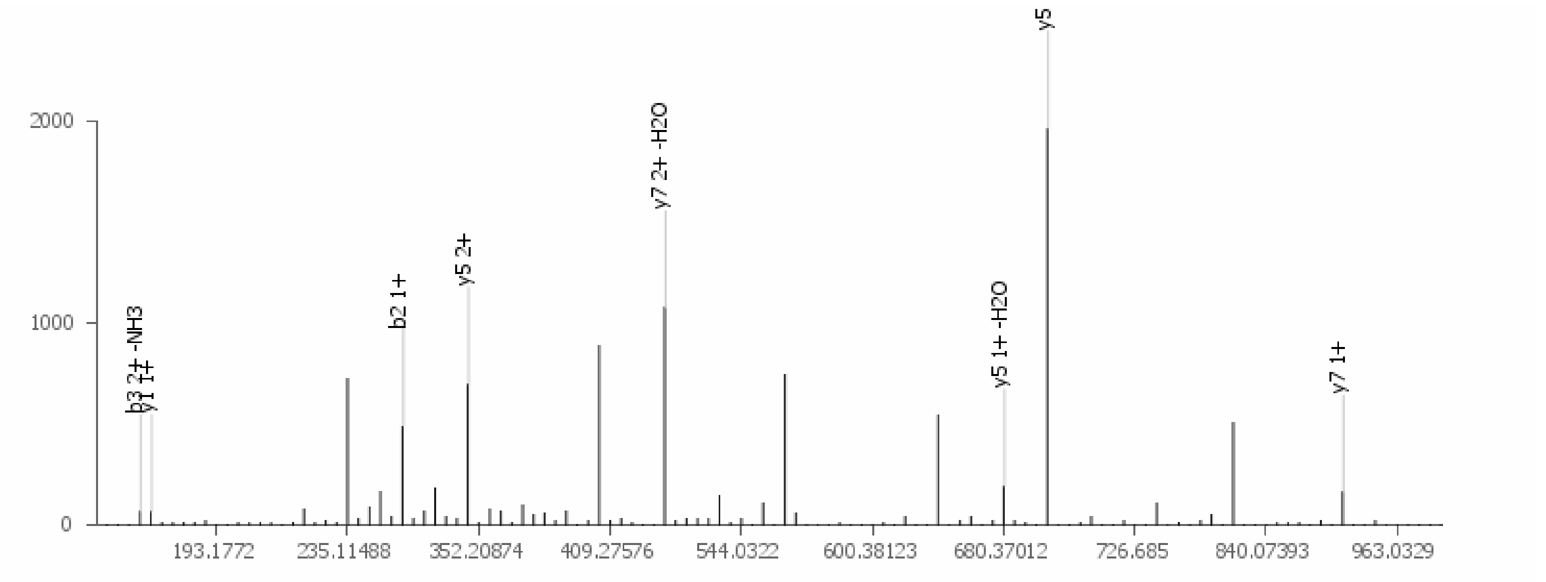

AT2G27120.1 - HRN(pT)QDGWK - 611.257337 - Charge:2

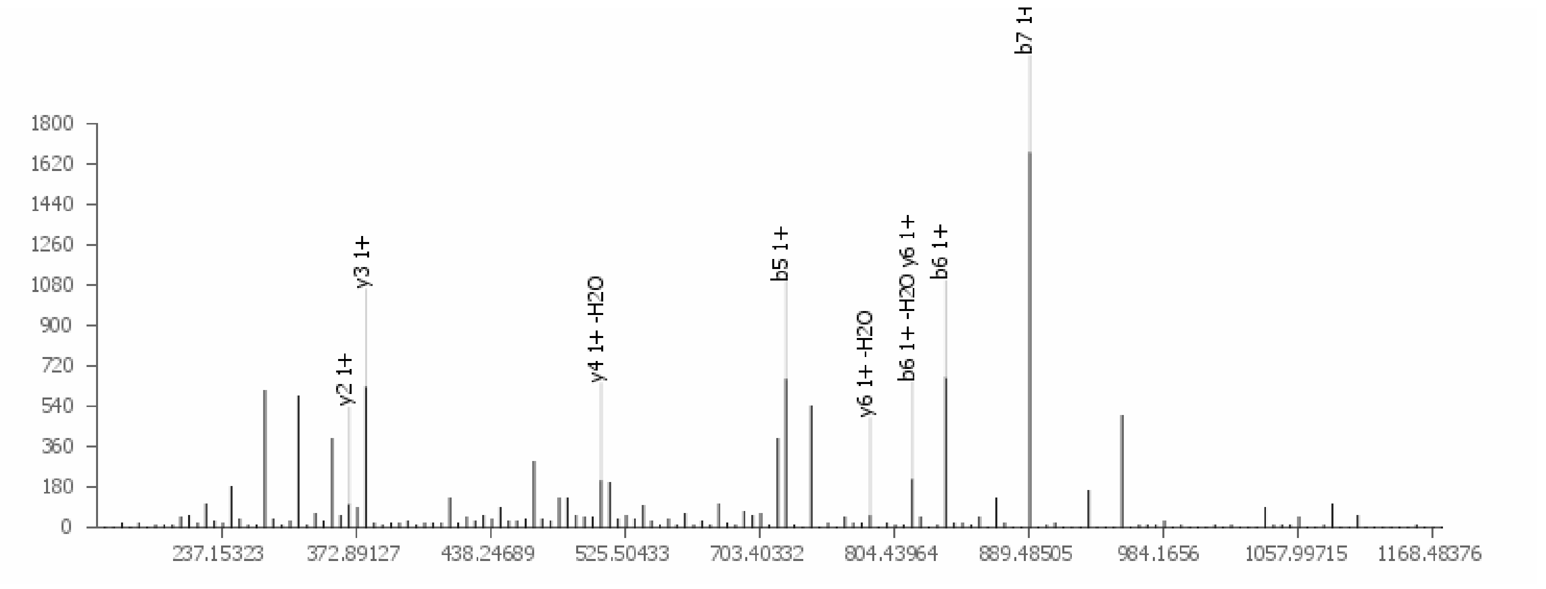

AT3G21300.1 - CC(s)(s)(s)LAPL(pS)PSEFSGGNK - 1072.907627 - Charge:2

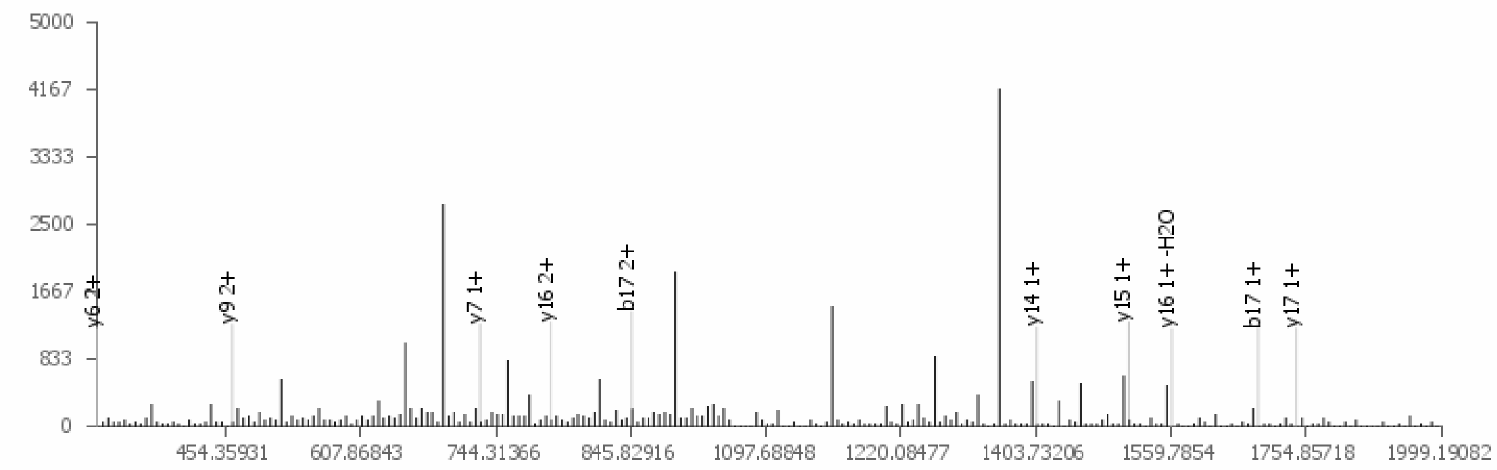

AT2G27120.1 - DIADSELLDYISES(pS)(t)(oxM)(s)K(s)LADYGQQK - 1090.788041 - Charge:3

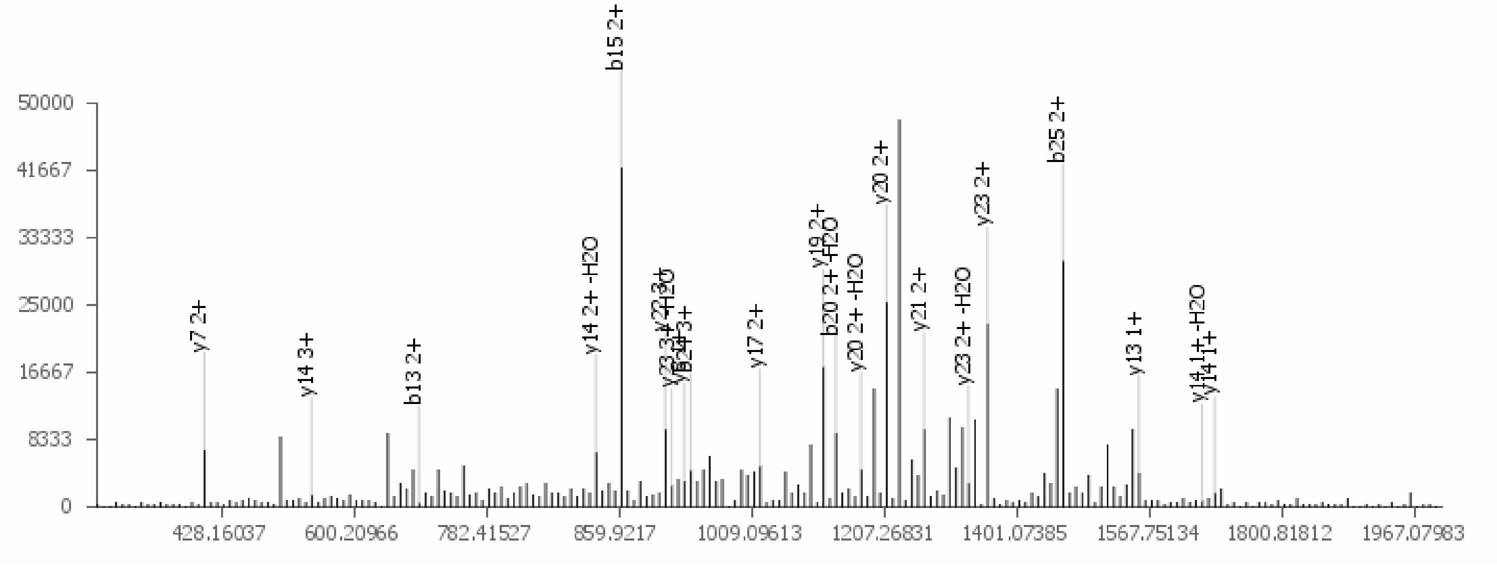

AT1G01450.1 - LFPS(pS)LLDN(pT)K - 697.801014 - Charge:2

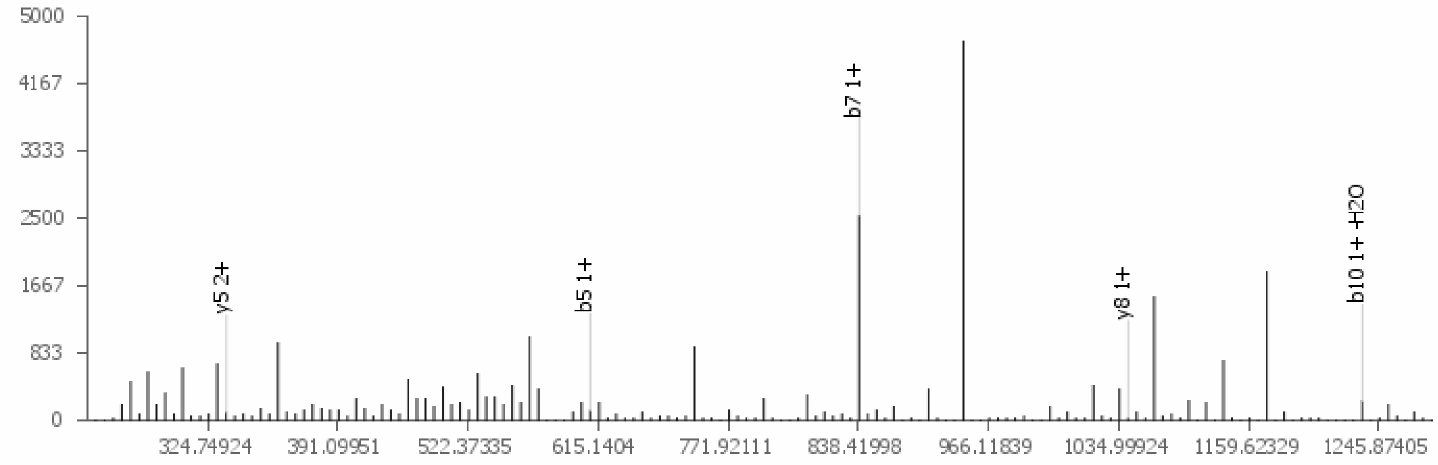

AT5G62720.1 - SSPMLANV(s)(s)R - 614.776532 - Charge:2

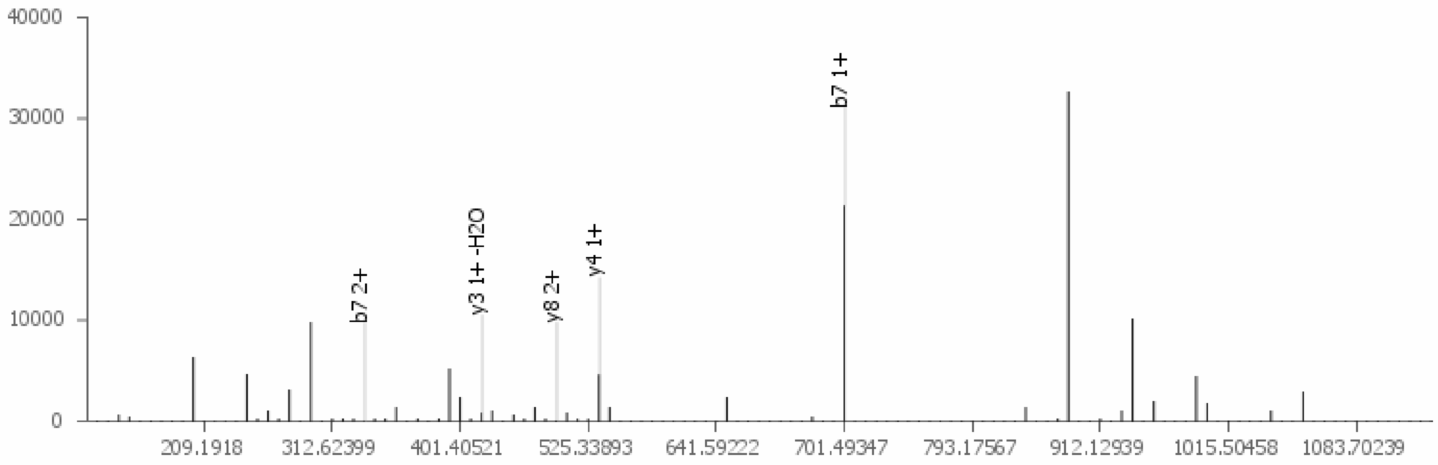

AT1G02300.1 - NQLWGE(pS)K - 521.222976 - Charge:2

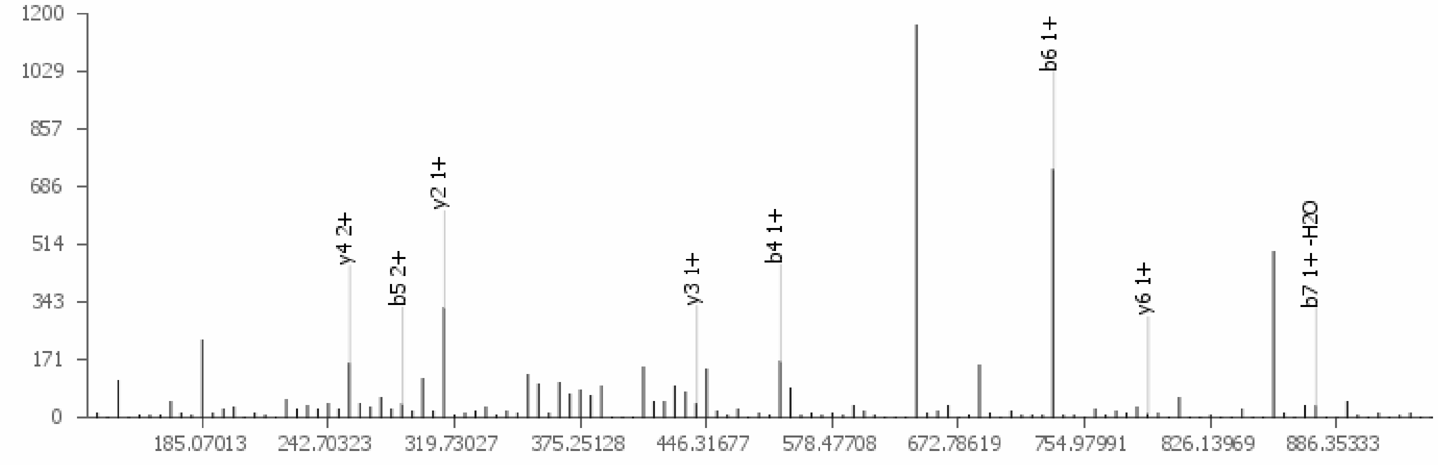

AT1G76590.1 - LM(pS)GL(s)I(s)(t)VK - 648.288482 - Charge:2

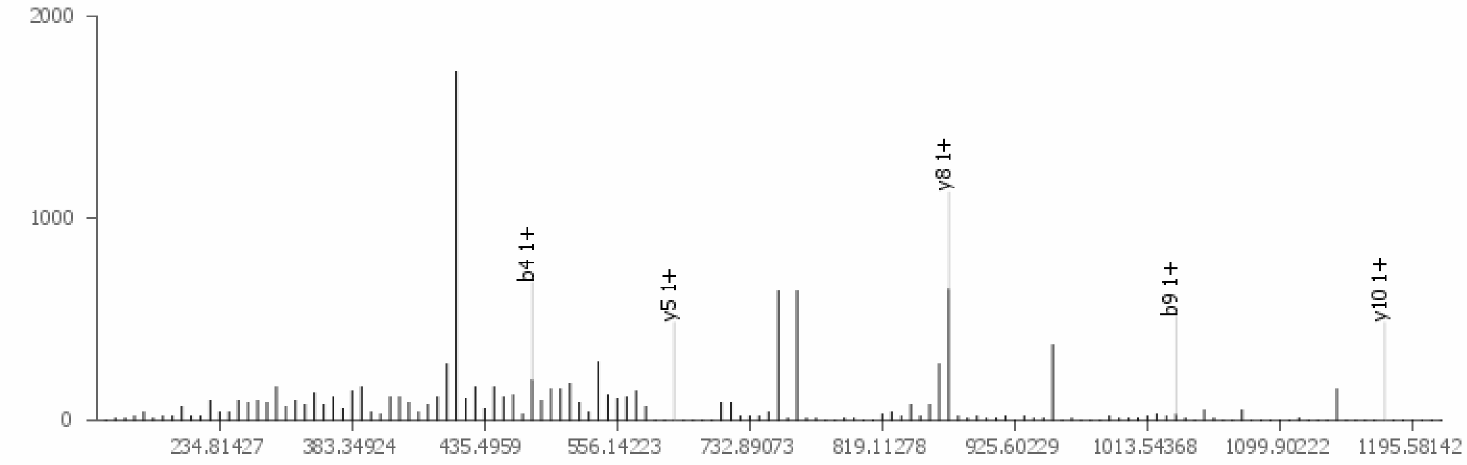

AT5G50320.1 - ELHV(pY)G(pT)AVPVHGR - 847.872803 - Charge:2

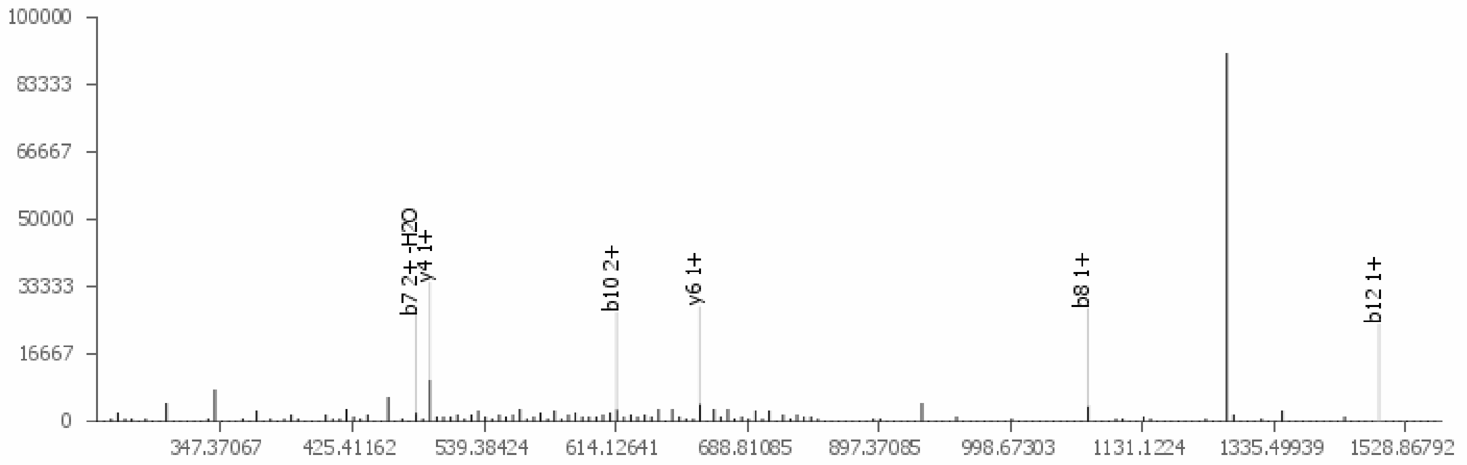

AT1G09040.1 - YLP(pS)DETKR - 594.76462 - Charge:2

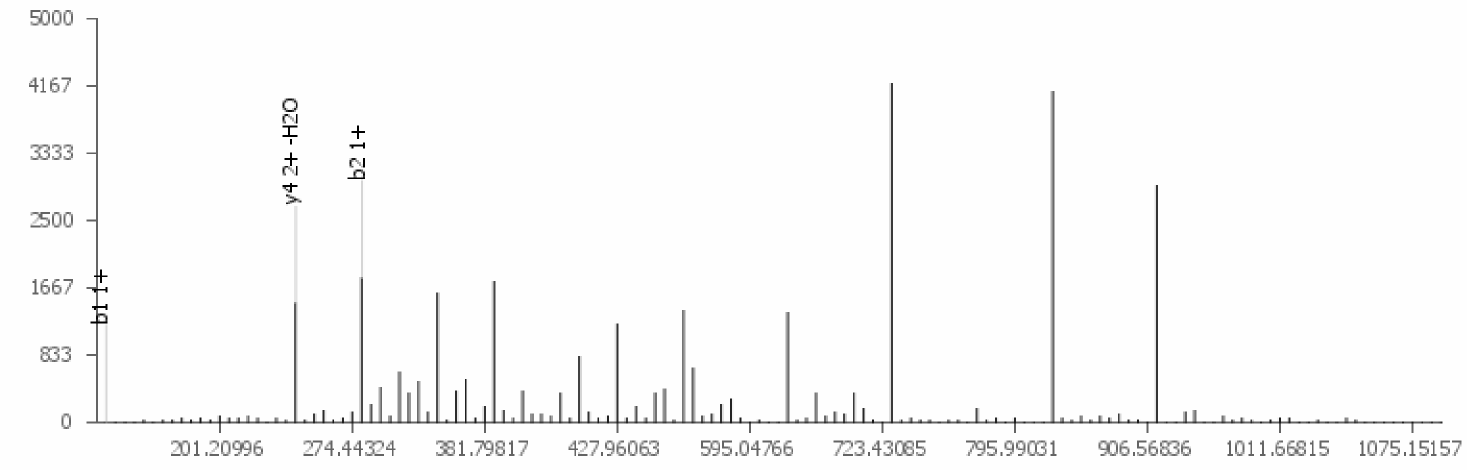

AT4G14750.1 - SSSQLG(pS)NTAK - 580.249274 - Charge:2

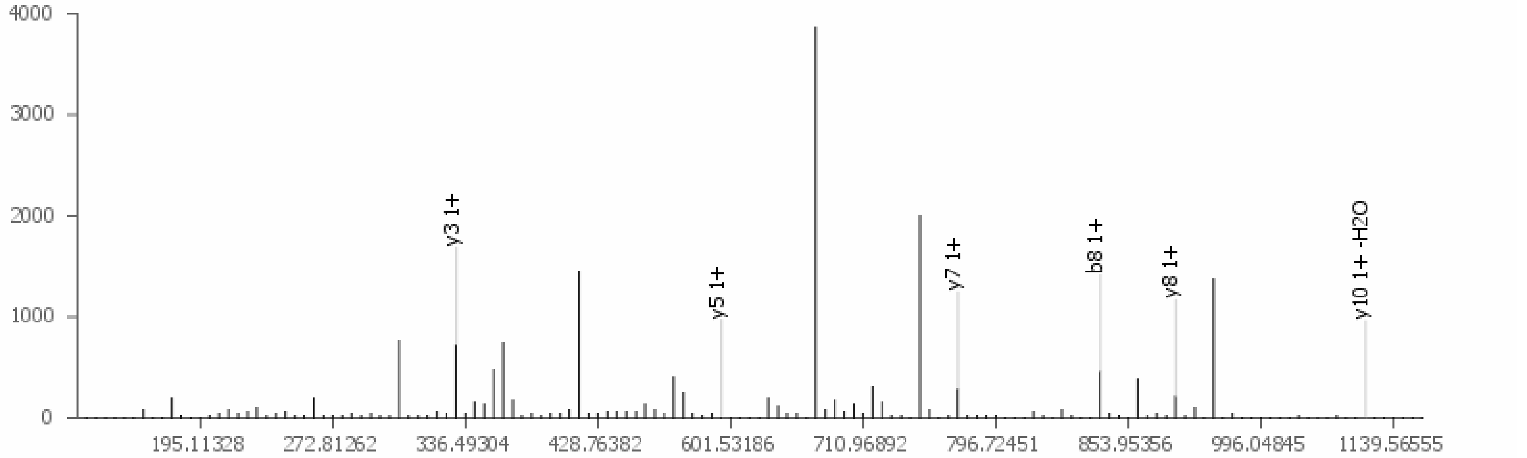

AT3G19680.1 - NLLHSS(s)(m)(m)(s)EISSTR - 938.402716 - Charge:2

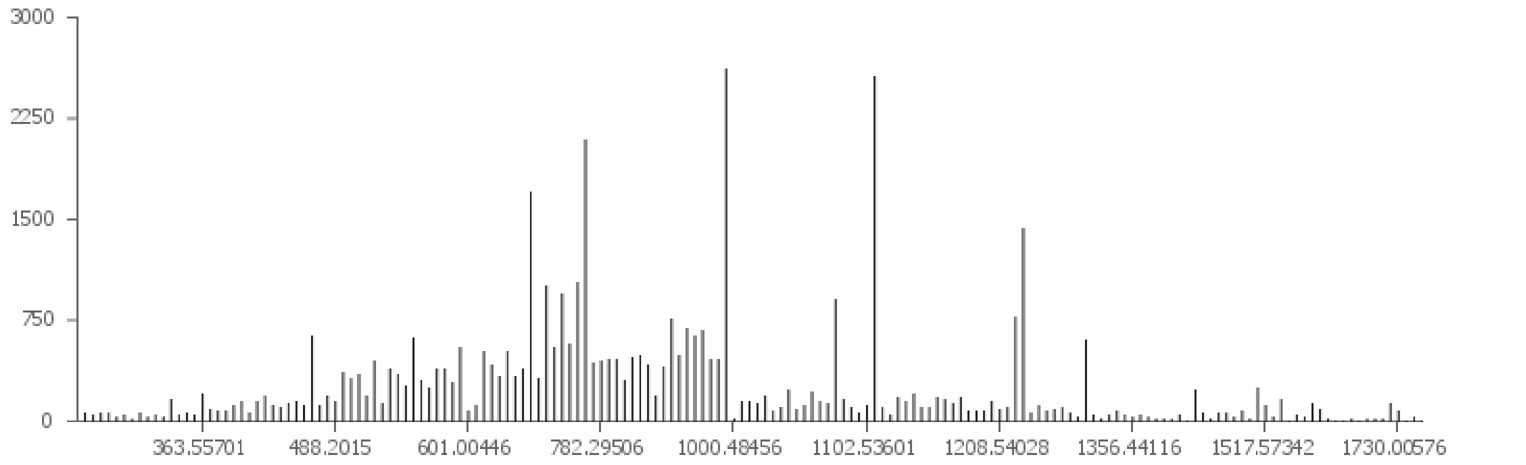

AT1G76420.1 - M(oxM)LAVEDVL(pS)ELAGEER - 994.436661 - Charge:2

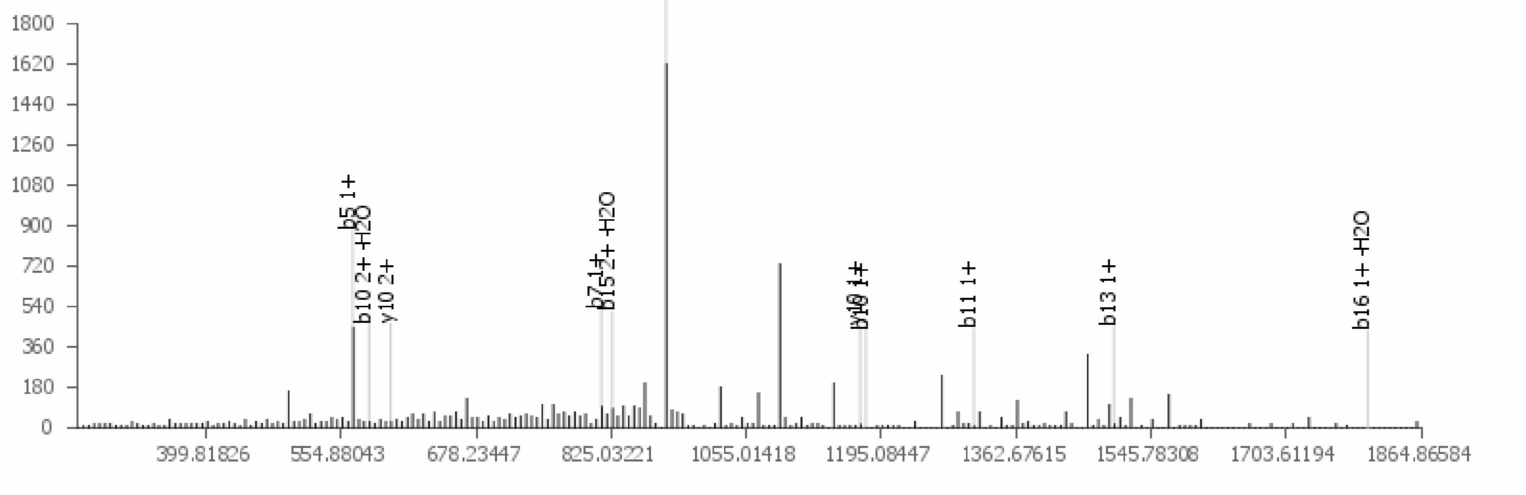

AT3G21600.1 - (pT)MLNGAGVVSGSV(oxM)VP(oxM)MK - 1010.962341 - Charge:2

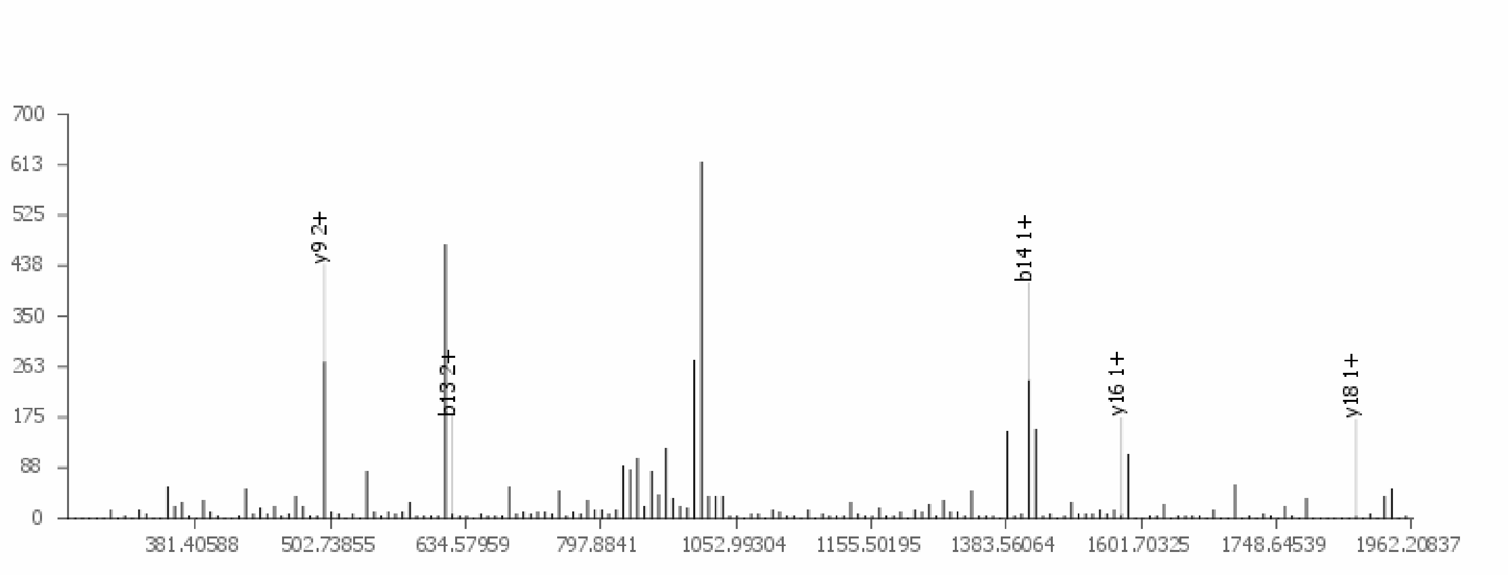

AT5G13460.1 - (pT)KPKDETLNEK - 691.833131 - Charge:2

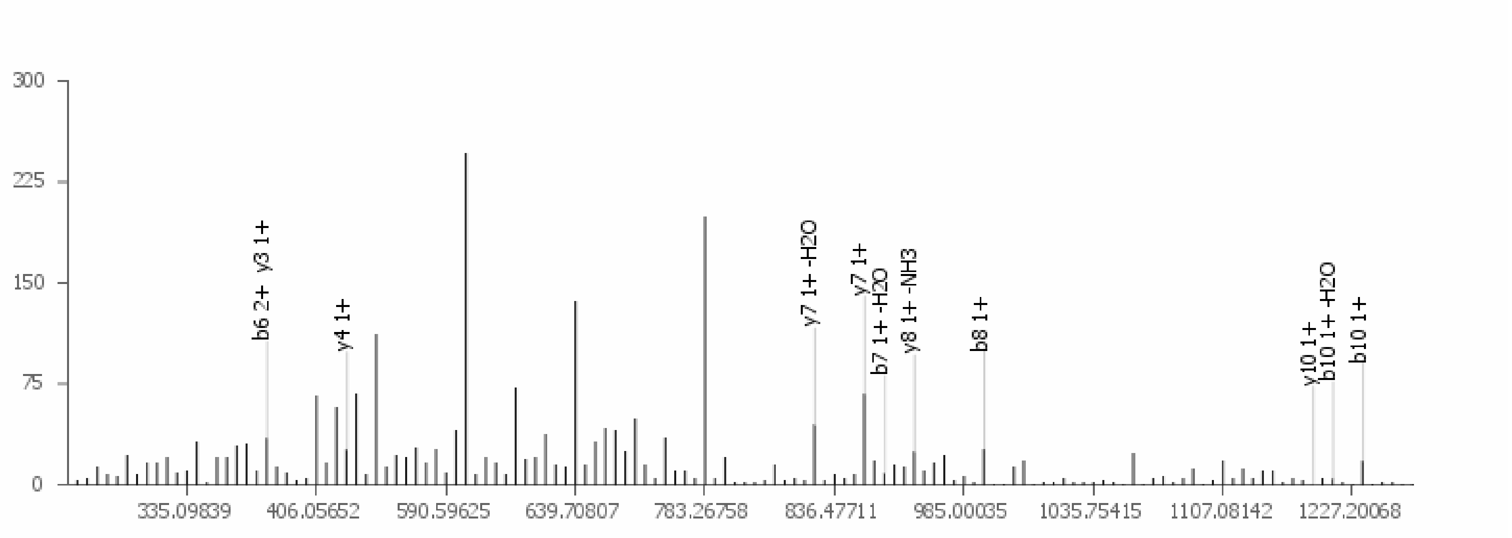

AT3G06545.1 - EF(pT)TKLER - 552.259784 - Charge:2

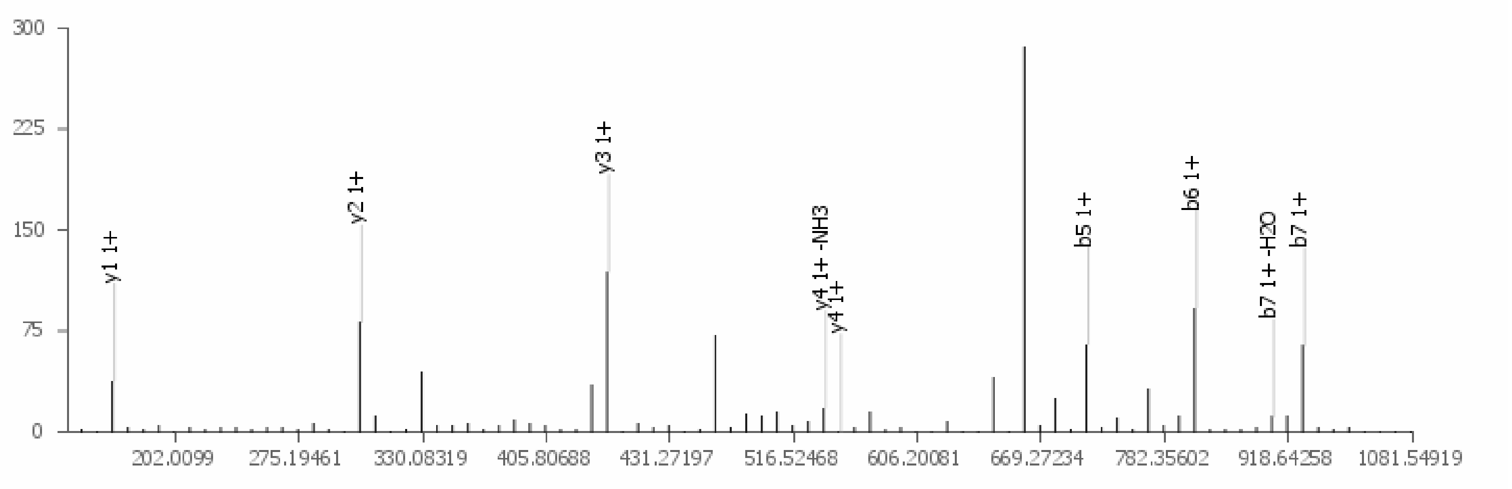

AT4G25650.1 - WL(pS)HFIYK - 587.282443 - Charge:2

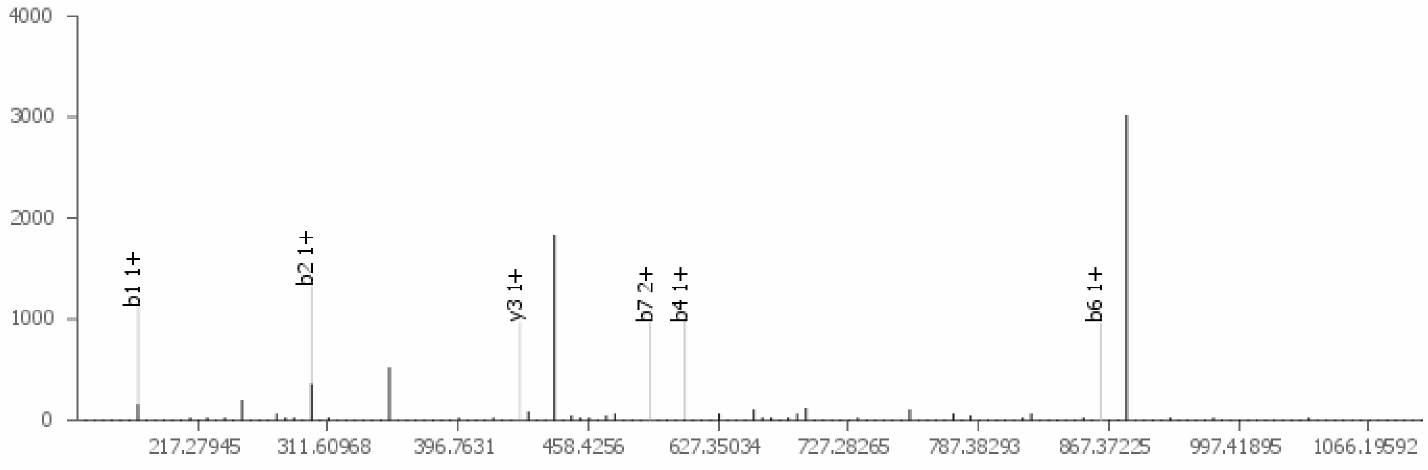

AT2G17510.1 - GT(pS)VYLVER - 552.257958 - Charge:2

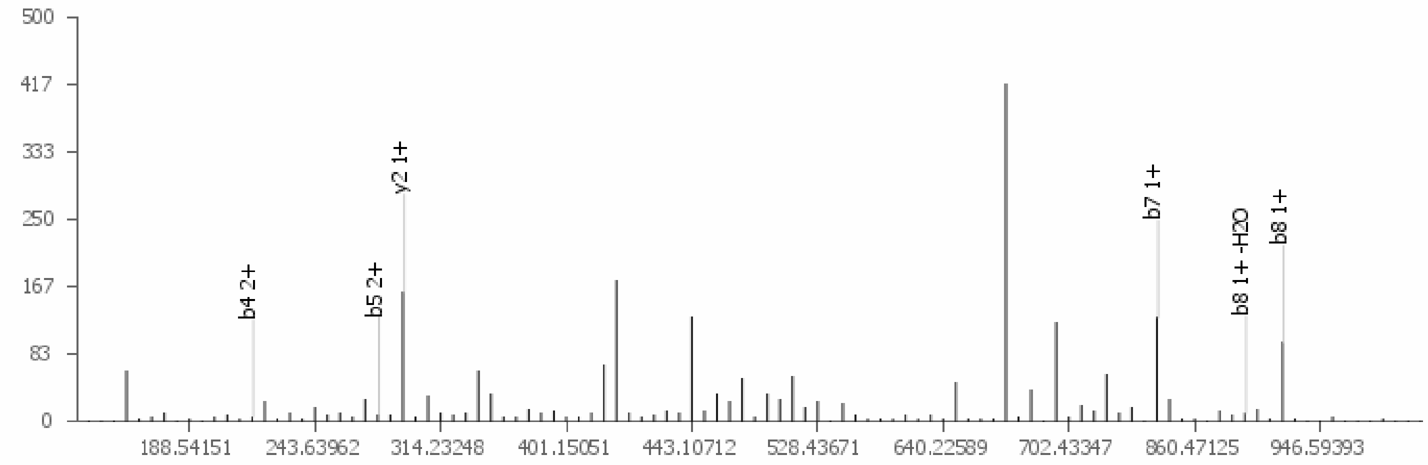

AT3G22790.1 - ATVLH(s)E(S)R - 540.248961 - Charge:2

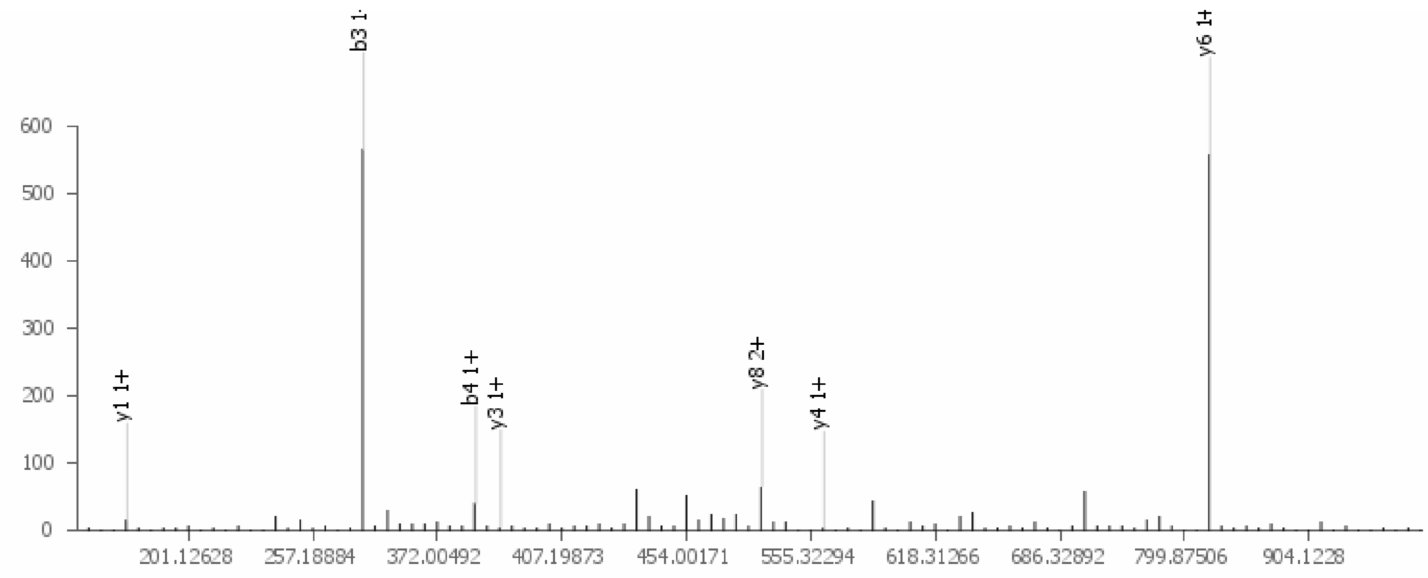

AT1G59610.1 - AESE(pS)LK(s)IL(t)GAPQSK - 953.431896 - Charge:2

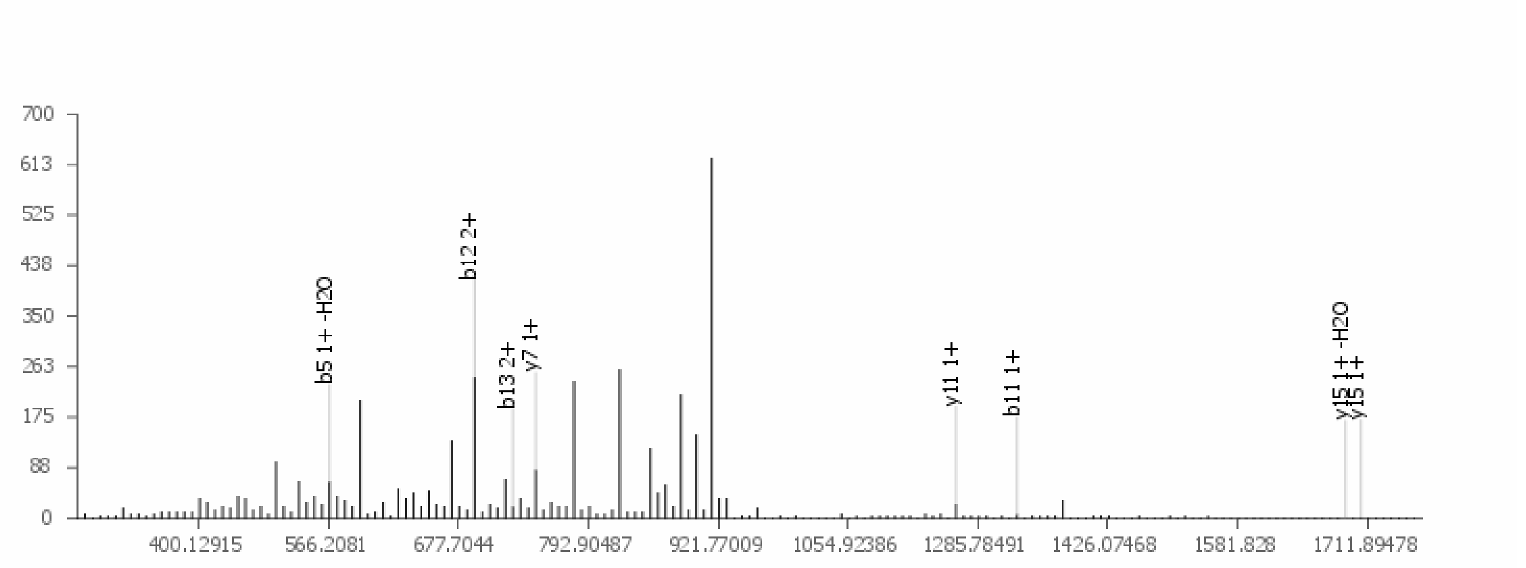

AT5G63510.1 - (pY)VTVGAYSLLR - 661.326205 - Charge:2

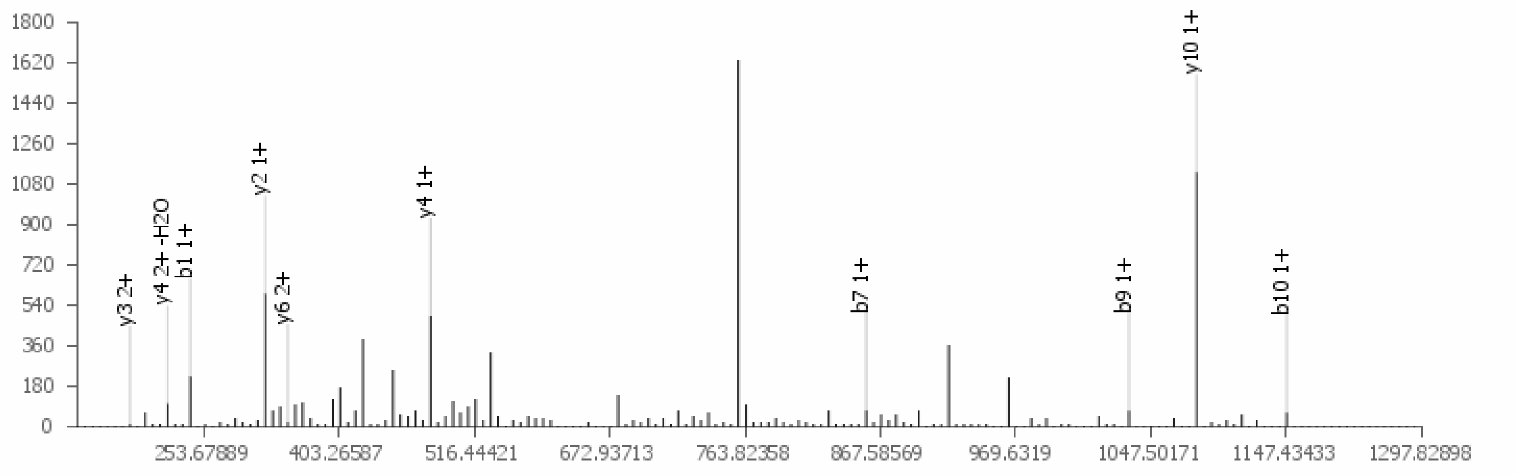

AT4G34190.1 - GGNRAA(s)V(s)IR - 584.281087 - Charge:2

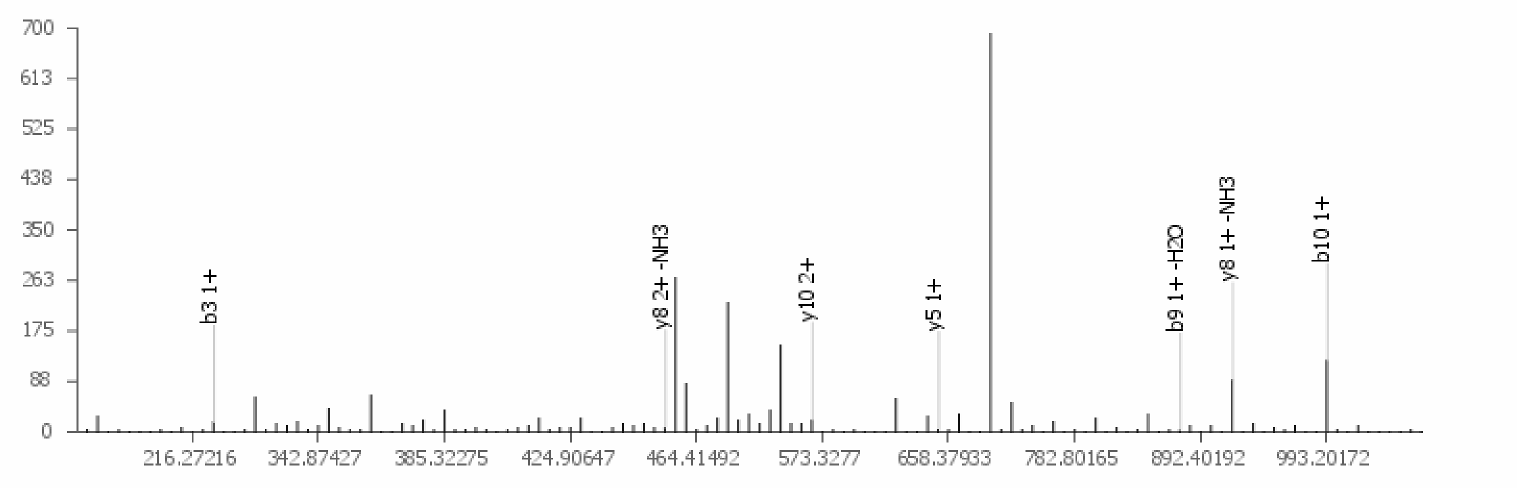

AT1G58440.1 - LLIPFP(pS)PK - 546.303026 - Charge:2

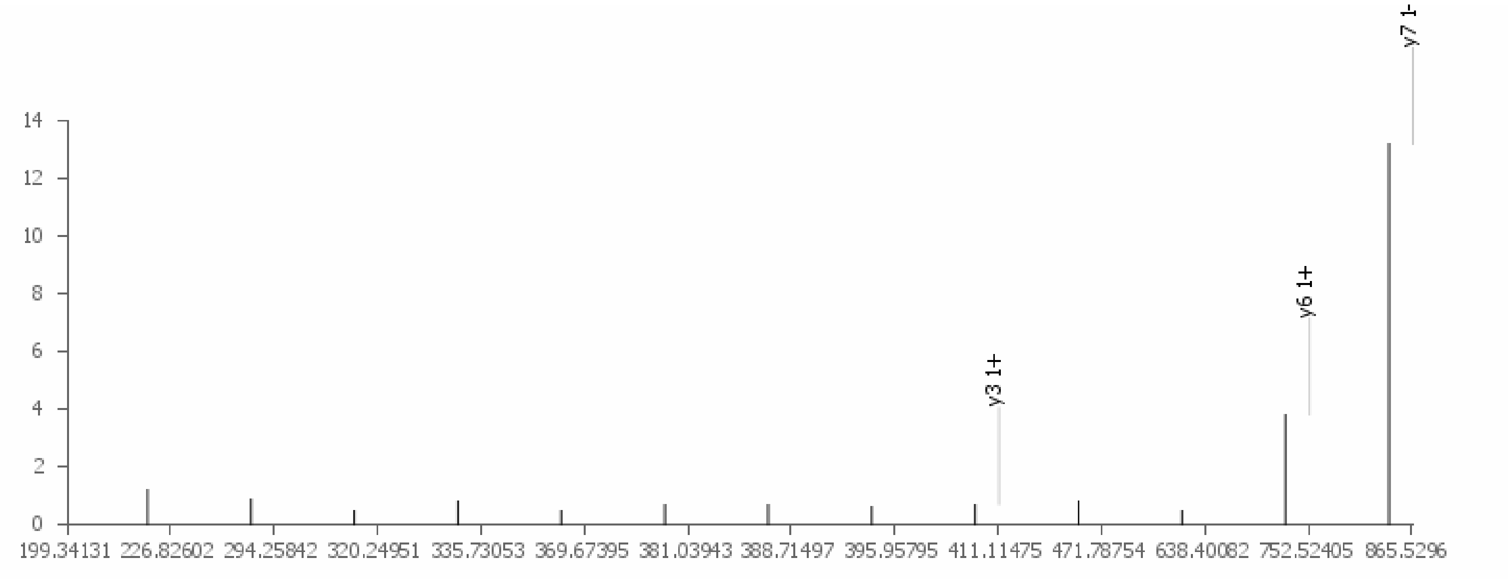

AT4G15110.1 - ETRQE(pT)DVEK - 657.784655 - Charge:2

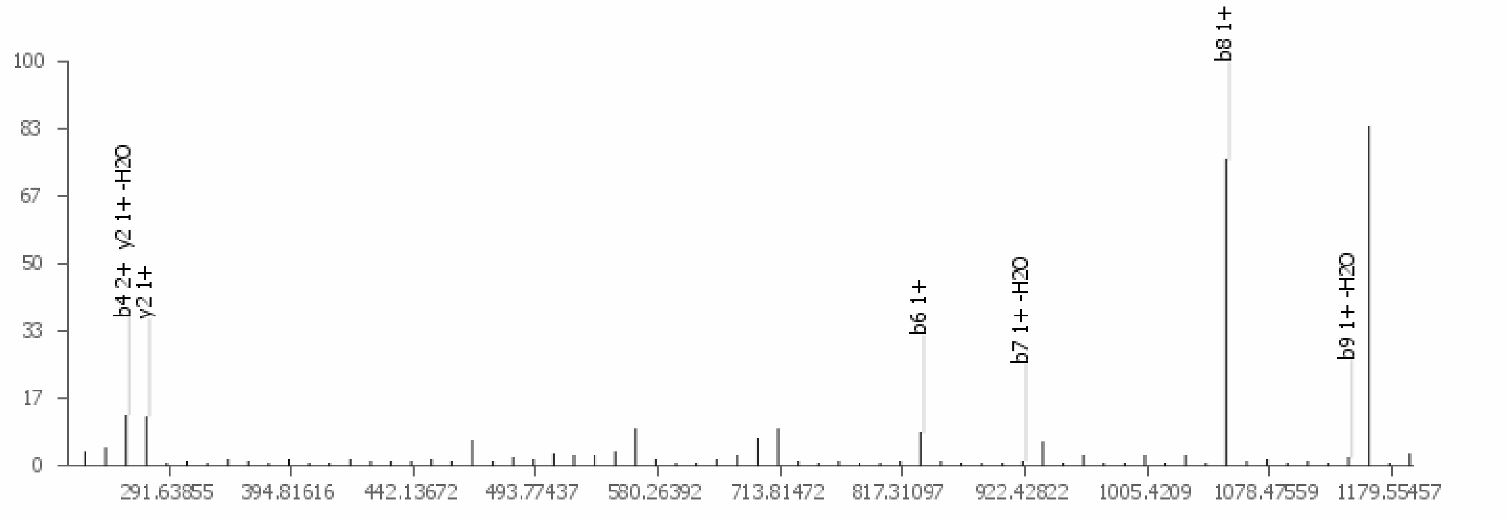

AT4G11876.1 - ILVREELA(pT)DR - 697.861122 - Charge:2

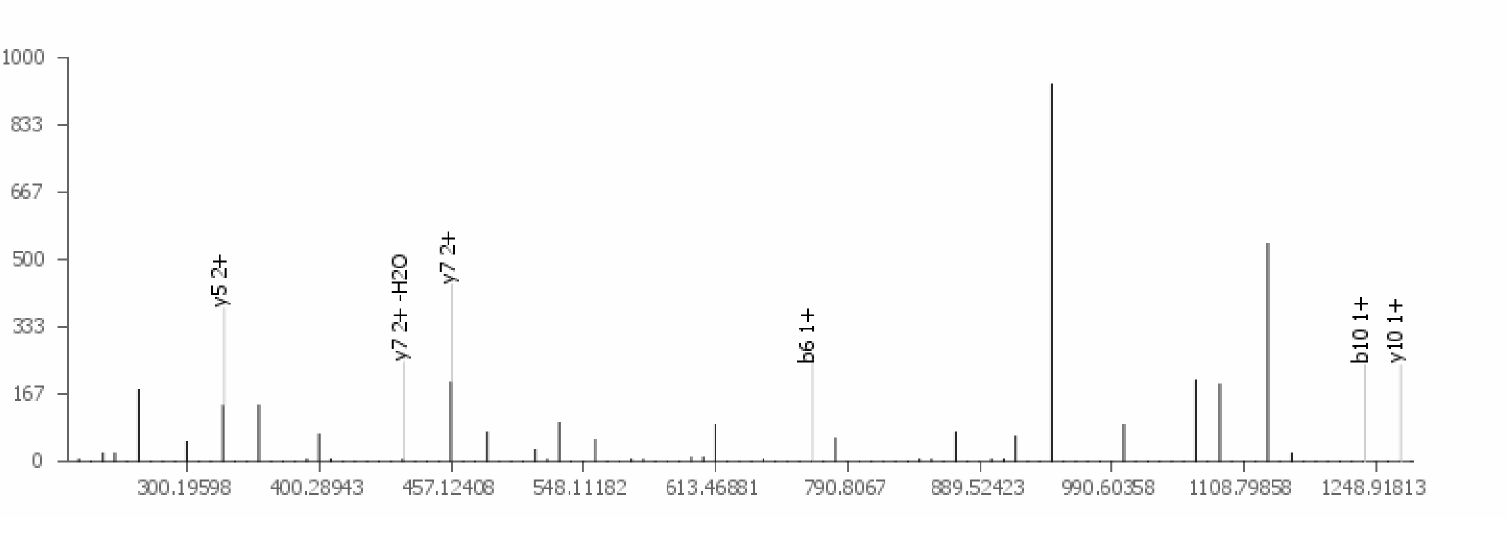

AT5G05680.1 - STPANSVEGRSILLD(pY)VK - 1015.013315 - Charge:2

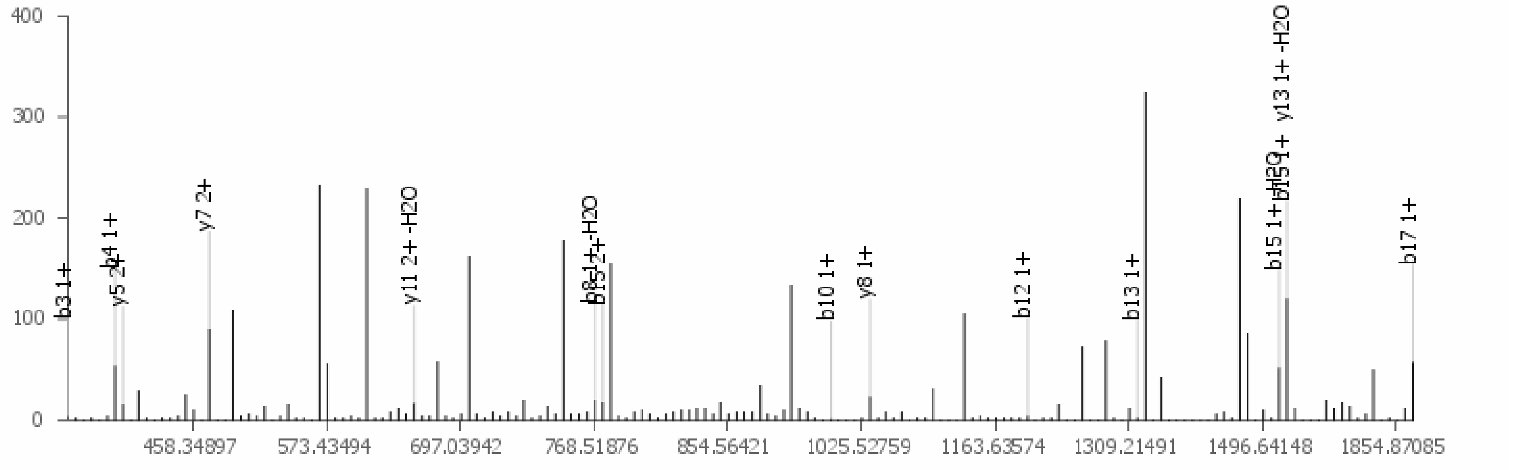

AT5G49870.1 - IYIG(pT)GEVGIVSIK - 764.908139 - Charge:2

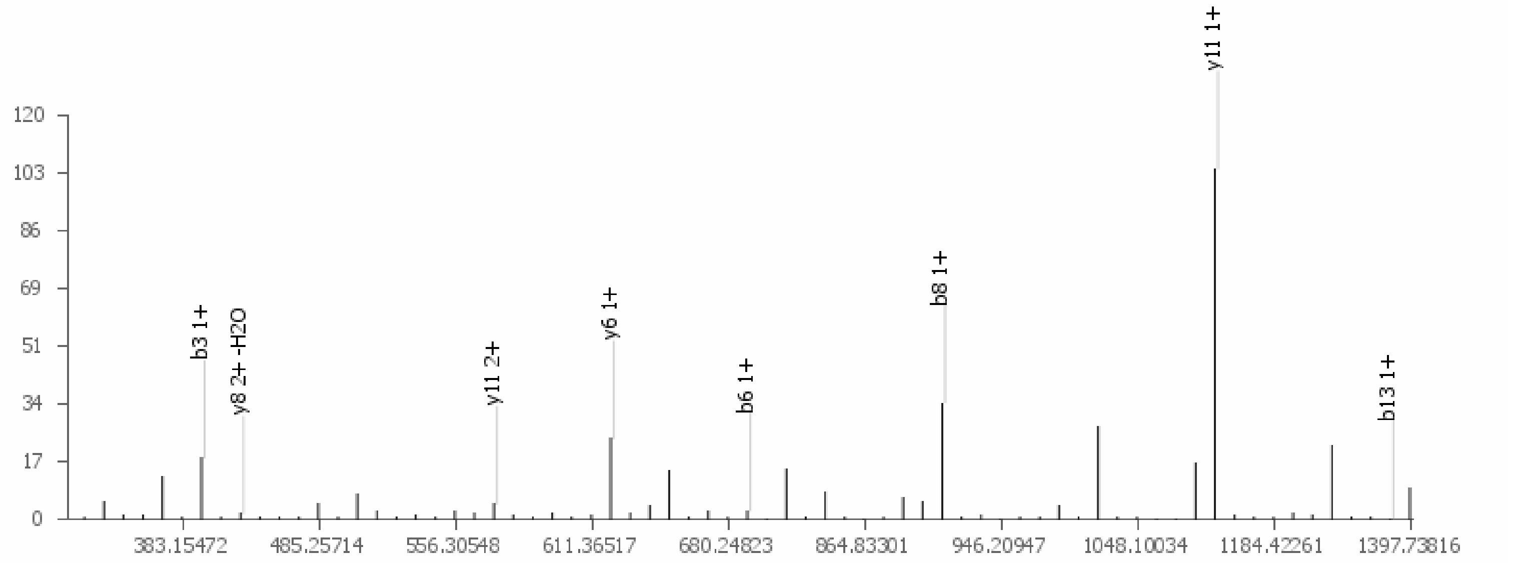

AT1G52430.1 - DVLENVLA(pS)ARK - 697.860889 - Charge:2

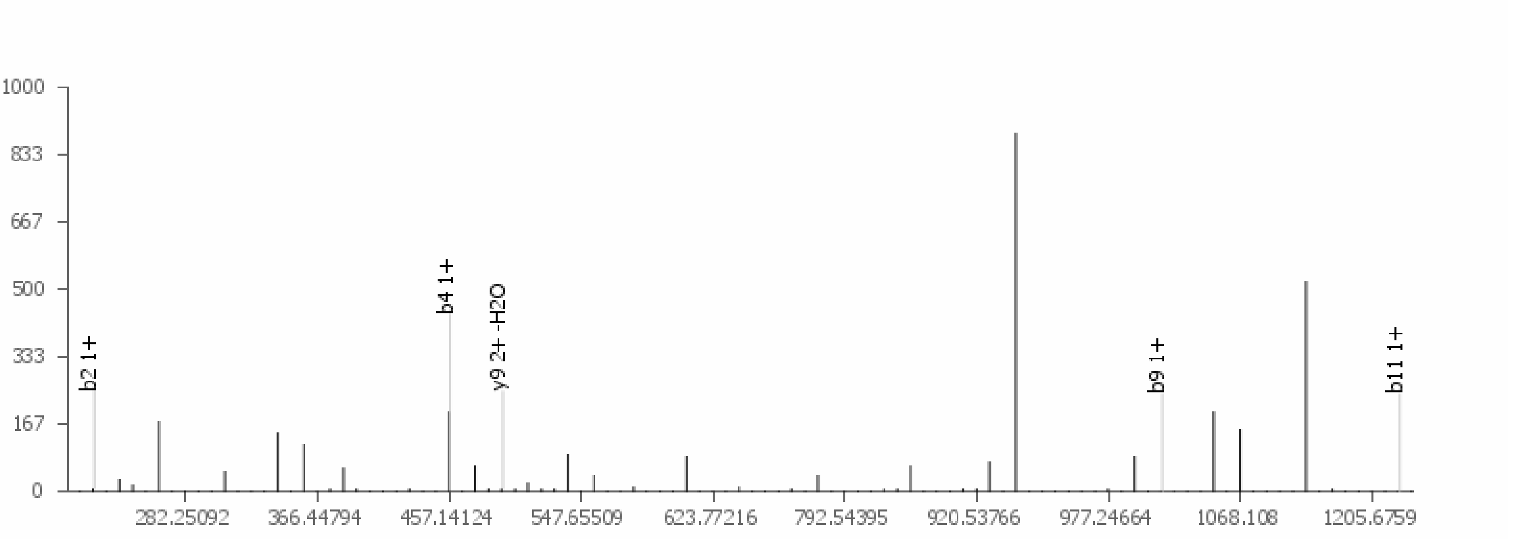

AT5G37600.1 - SLV(pS)DLINLNLSDSTDK - 957.46079 - Charge:2

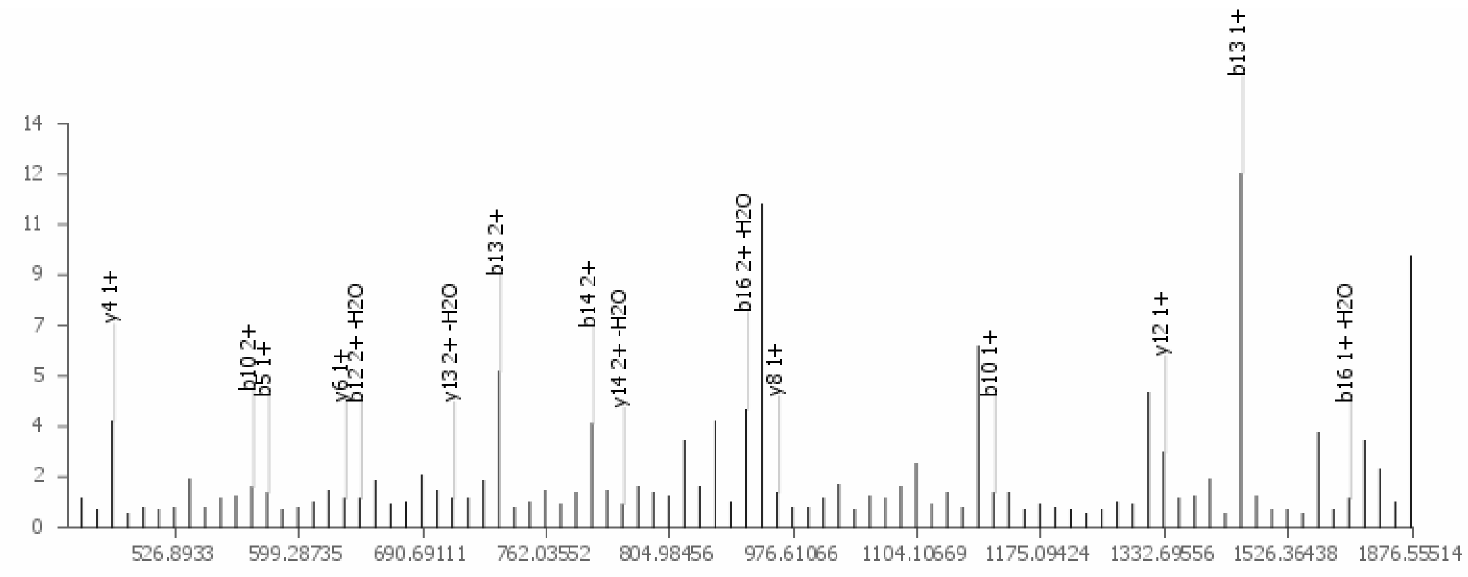

AT2G26410.1 - L(s)(s)(s)(t)SLGKTK - 594.798513 - Charge:2

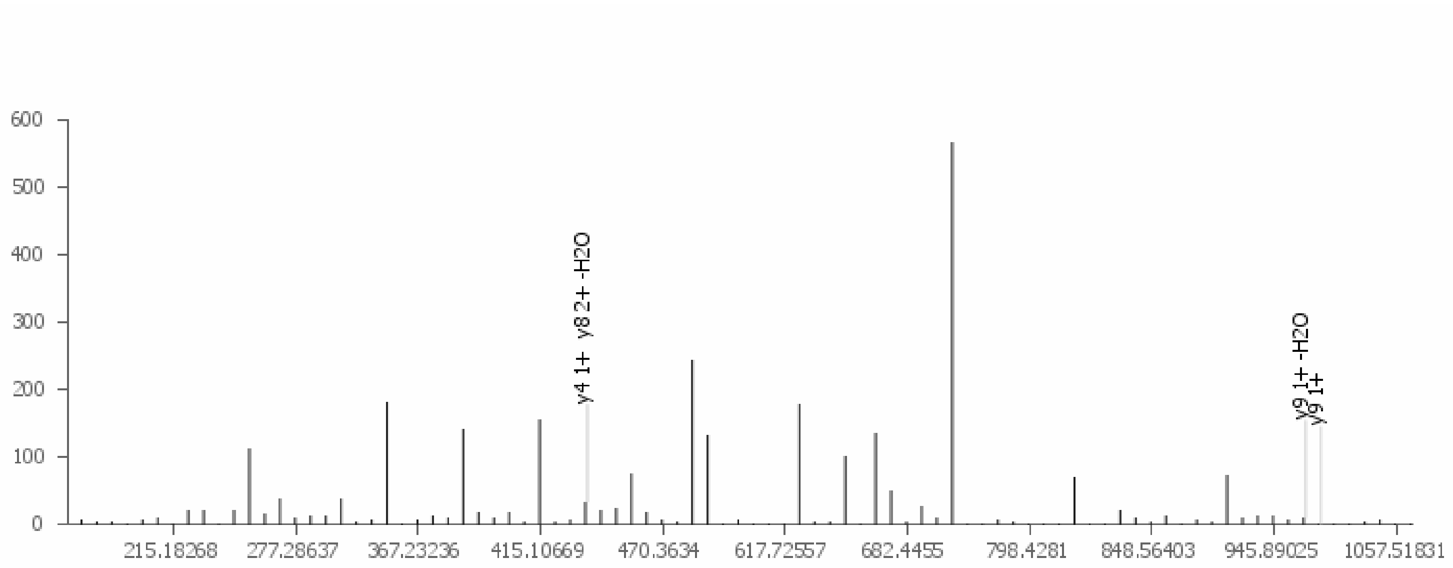

AT4G25450.1 - IIELG(t)H(s)ELVAQKGSYASLVGTQR - 913.127106 - Charge:3

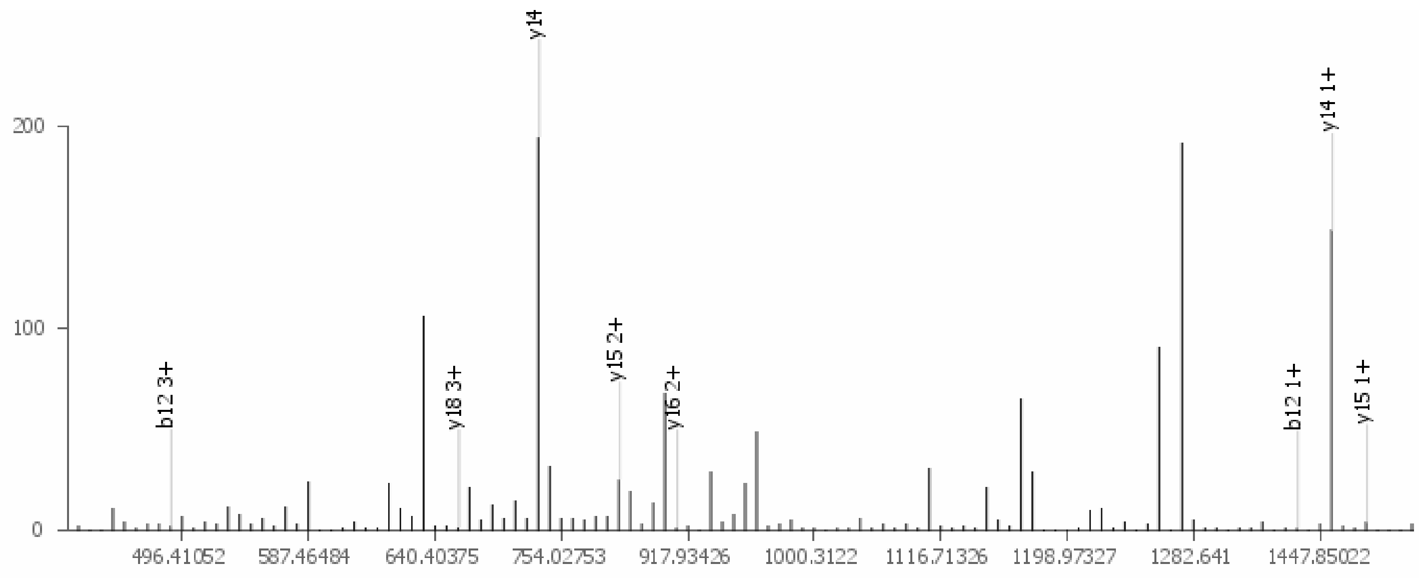

AT1G31410.1 - LPFTREVIPGASPSALDGPLV(pT)EPEK - 963.156809 - Charge:3

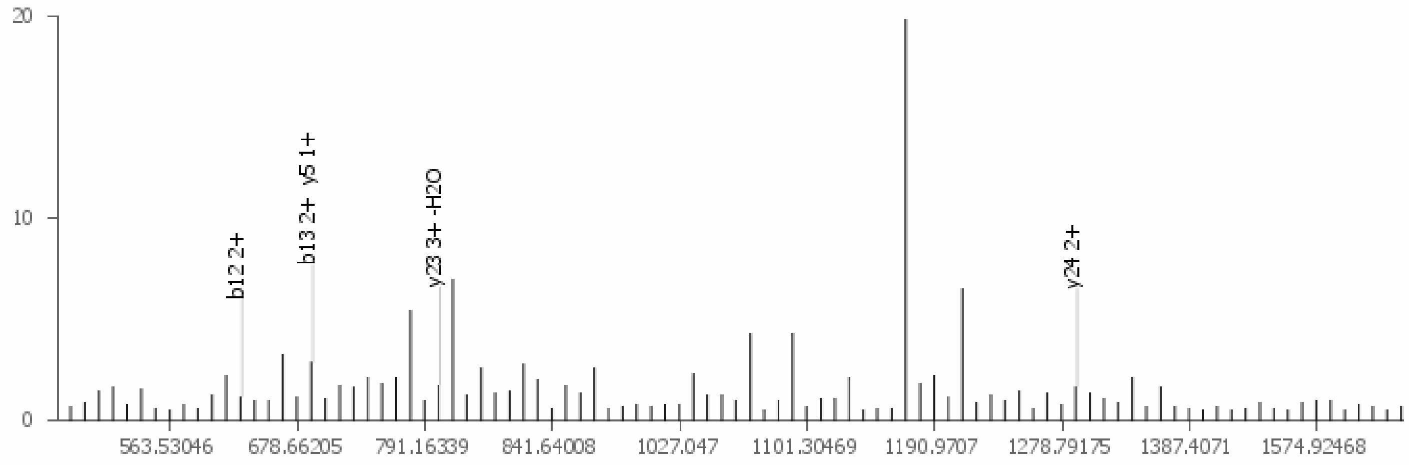

AT1G67140.1 - LLAAHA(pS)LK - 502.269374 - Charge:2

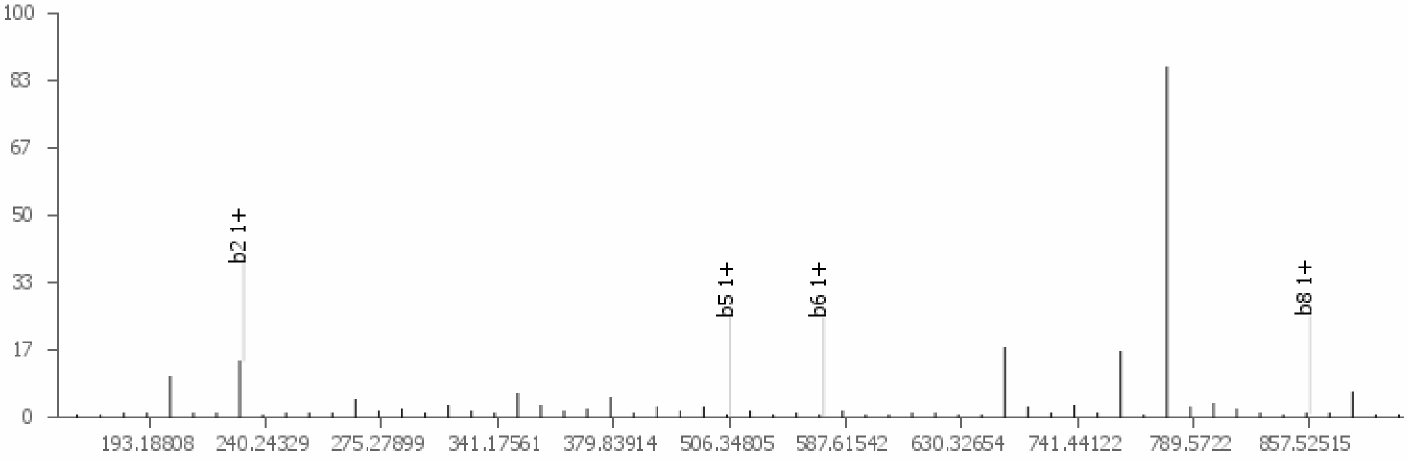

AT1G32660.1 - LELD(pS)LPLDLK - 668.342796 - Charge:2

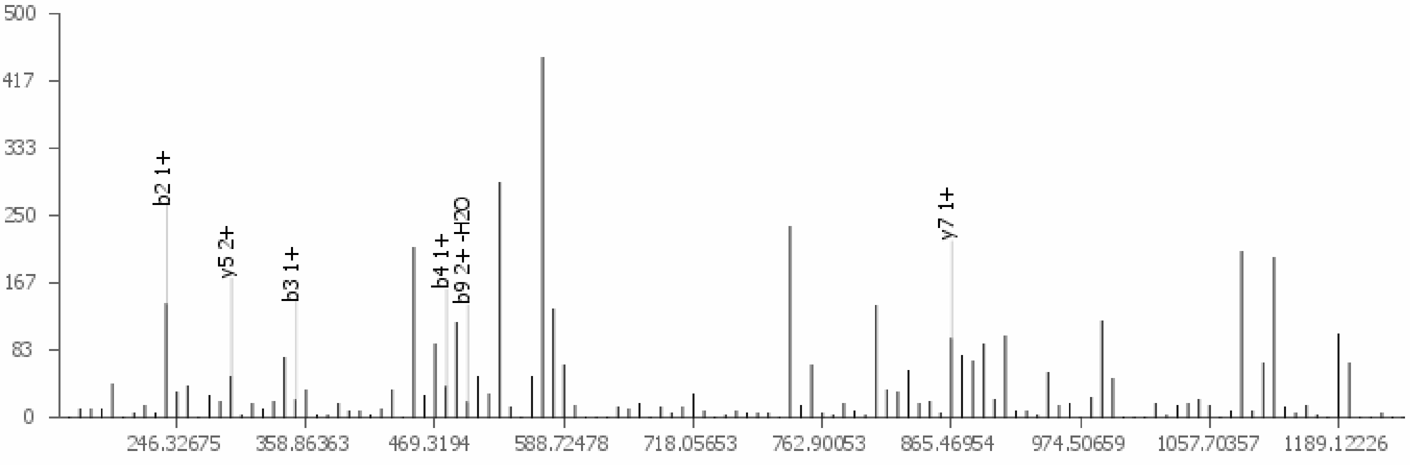

AT3G25180.1 - LVVASDPK(pT)VK - 618.831843 - Charge:2

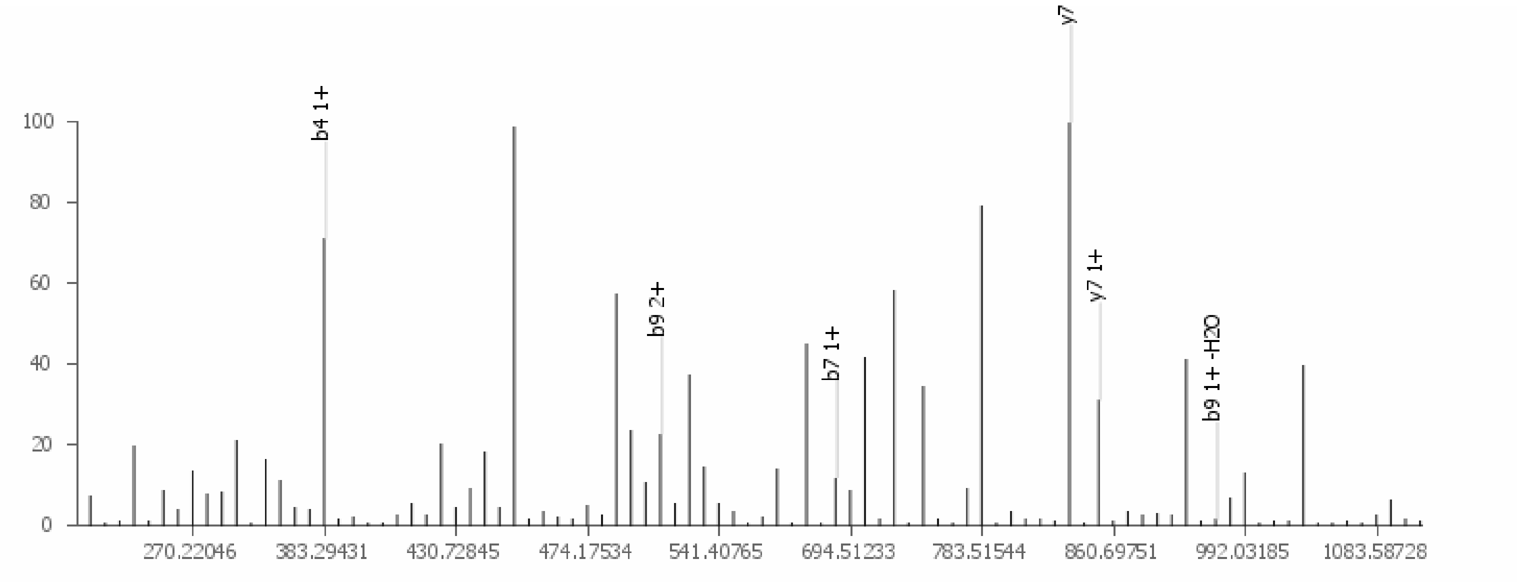

AT3G04740.1 - STATGMPVVPA(t)A(s)SR - 842.398719 - Charge:2

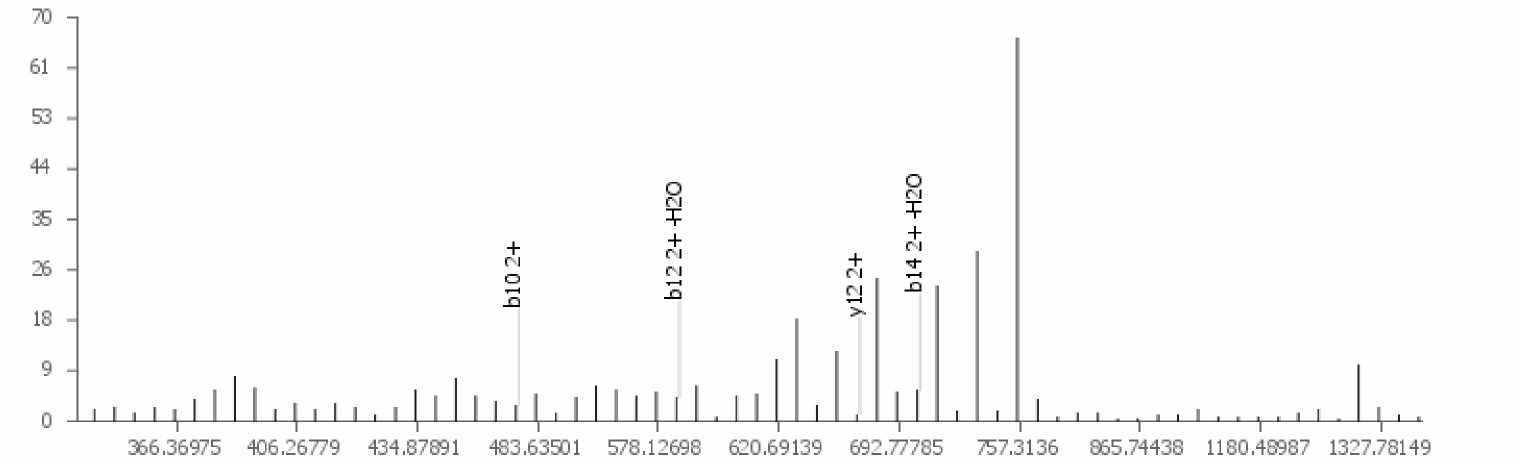

AT3G48390.1 - (s)(t)GSETIRSAR - 622.786842 - Charge:2

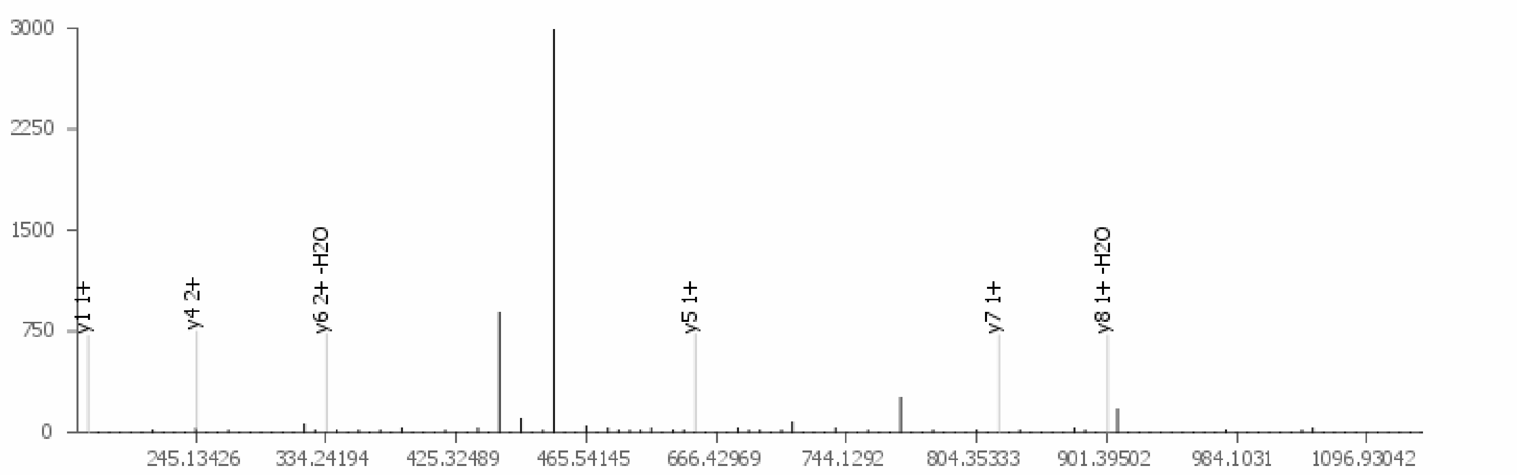

AT1G67470.1 - KPK(pS)EIASER - 612.798525 - Charge:2

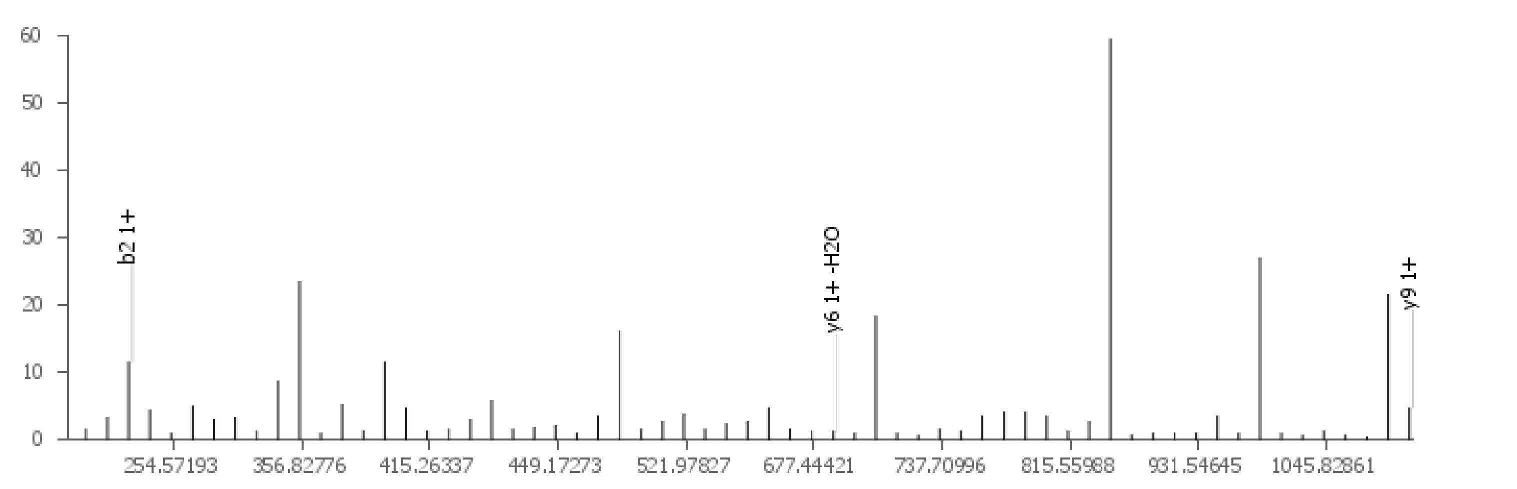

AT3G22910.1 - IIQ(pS)(oxM)AAKSLR - 657.342374 - Charge:2

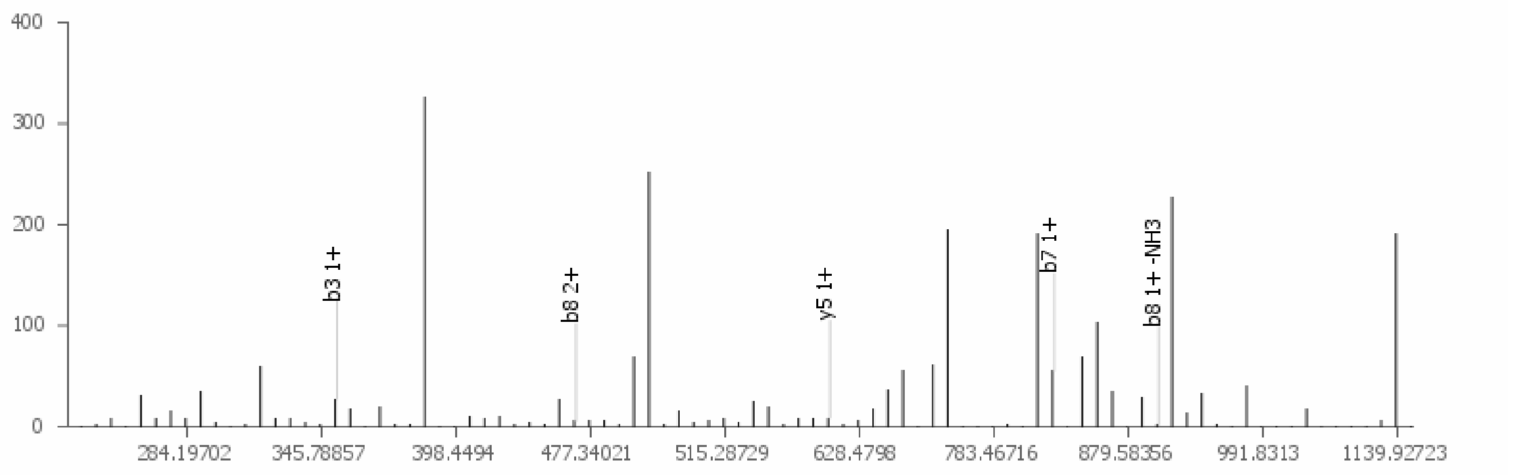

AT5G37970.1 - (pY)VNYFIVLKR - 697.865962 - Charge:2

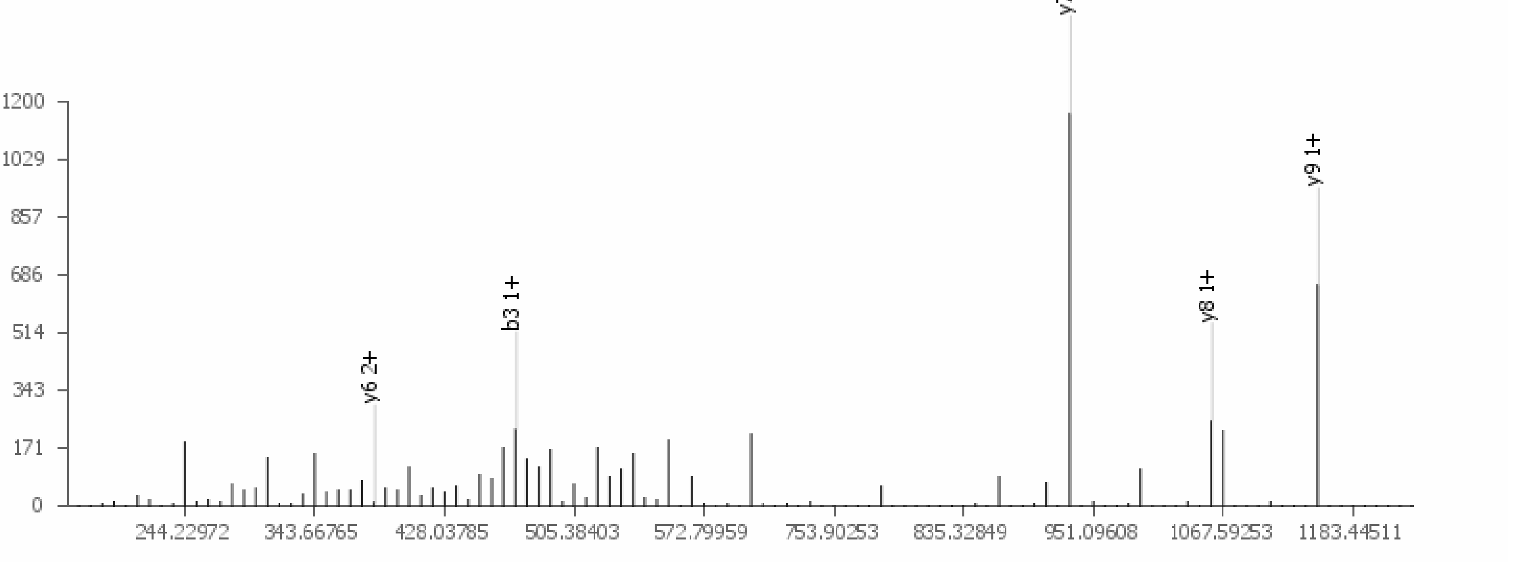

AT2G07750.1 - ALSASGIVKM(pT)R - 657.342421 - Charge:2

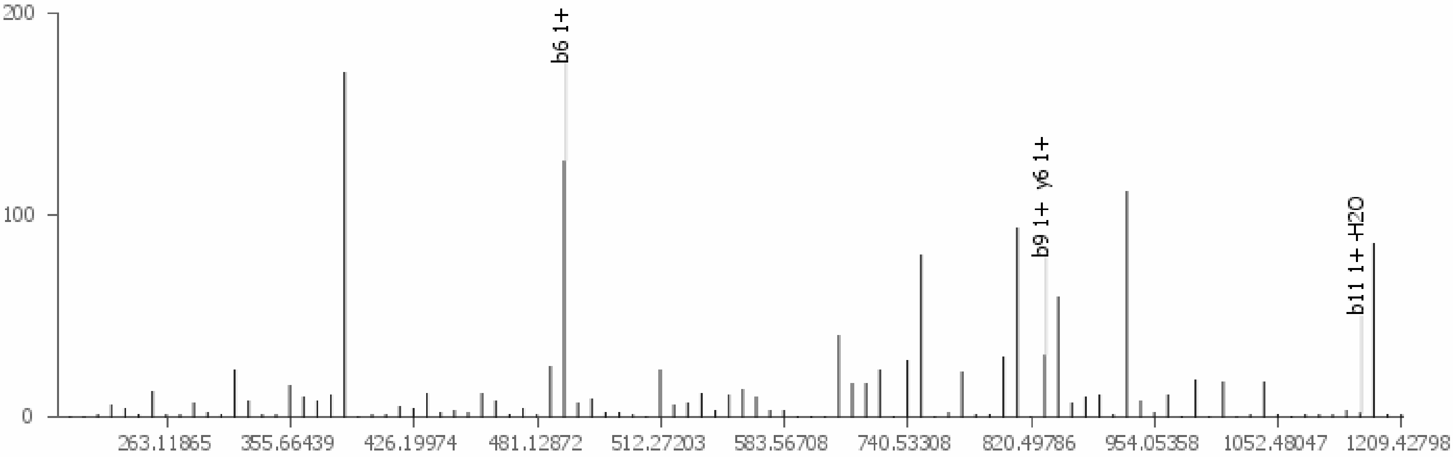

AT2G30600.1 - FSLLP(pY)ELLKR - 729.890779 - Charge:2

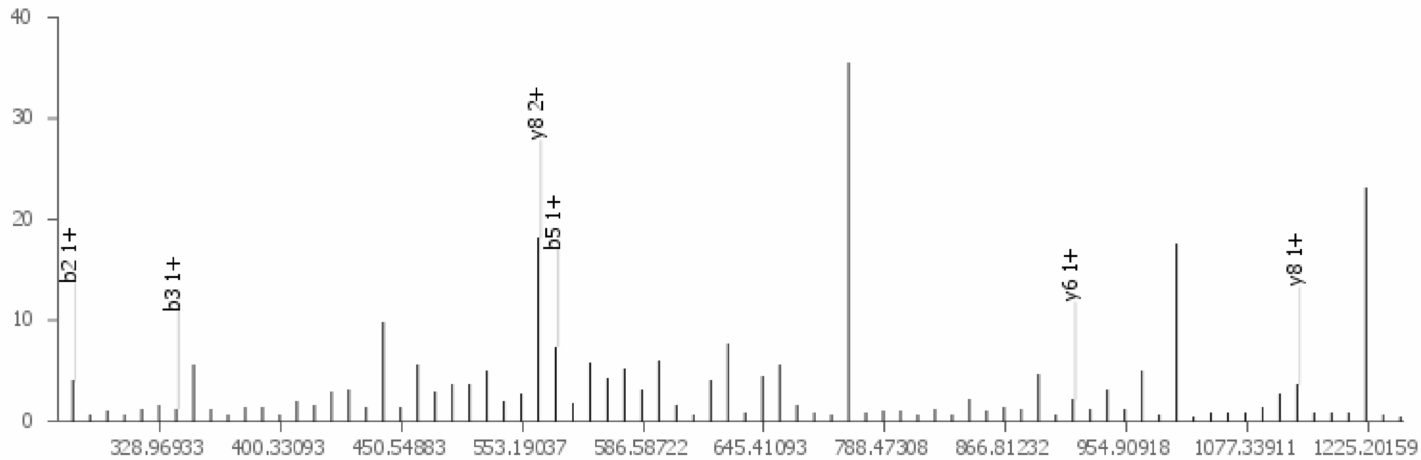

AT4G20360.1 - V(pT)KIMNDK - 514.747116 - Charge:2

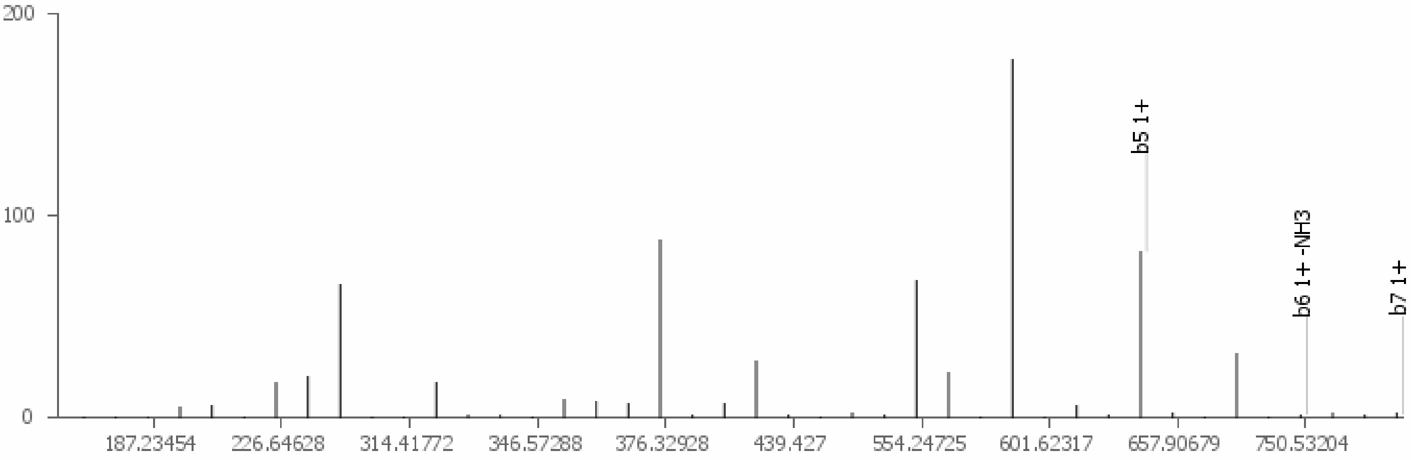

AT4G28560.1 - MGLEGNIPASMG(s)(s)LK - 836.375821 - Charge:2

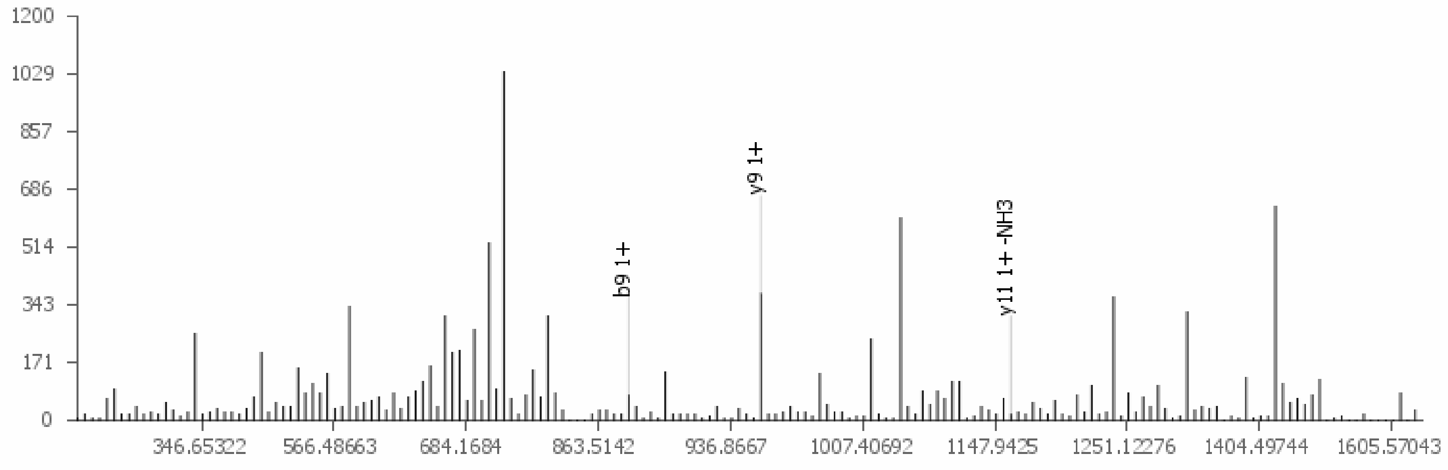

AT3G28650.1 - RNFDLLC(s)(s)ITMPFK - 954.948412 - Charge:2

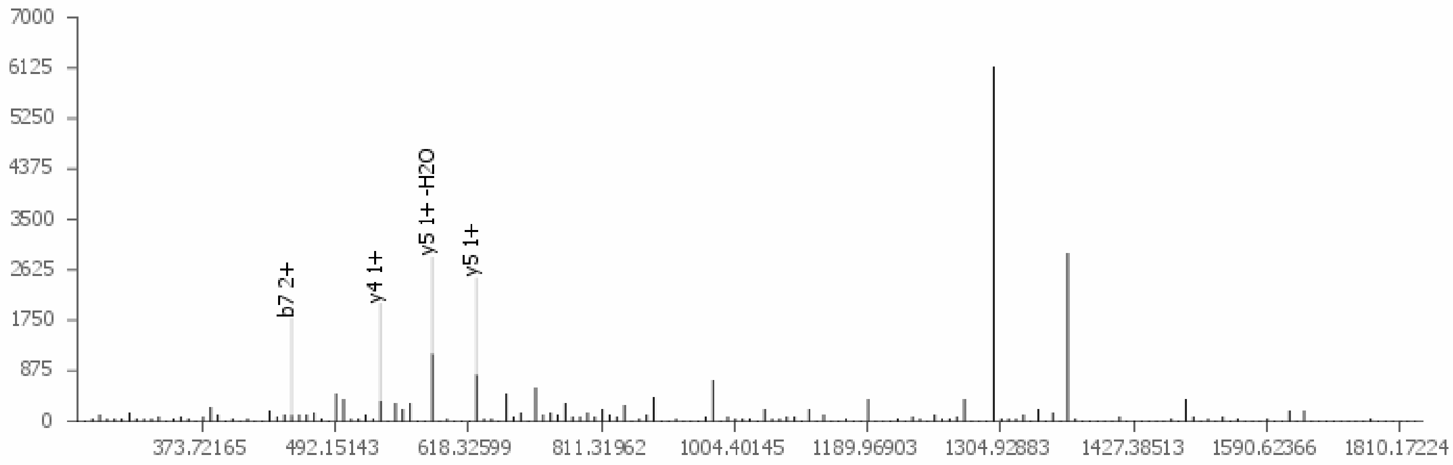

AT1G76630.1 - IFALVESANIFLMLGS(pY)RK - 1126.579599 - Charge:2

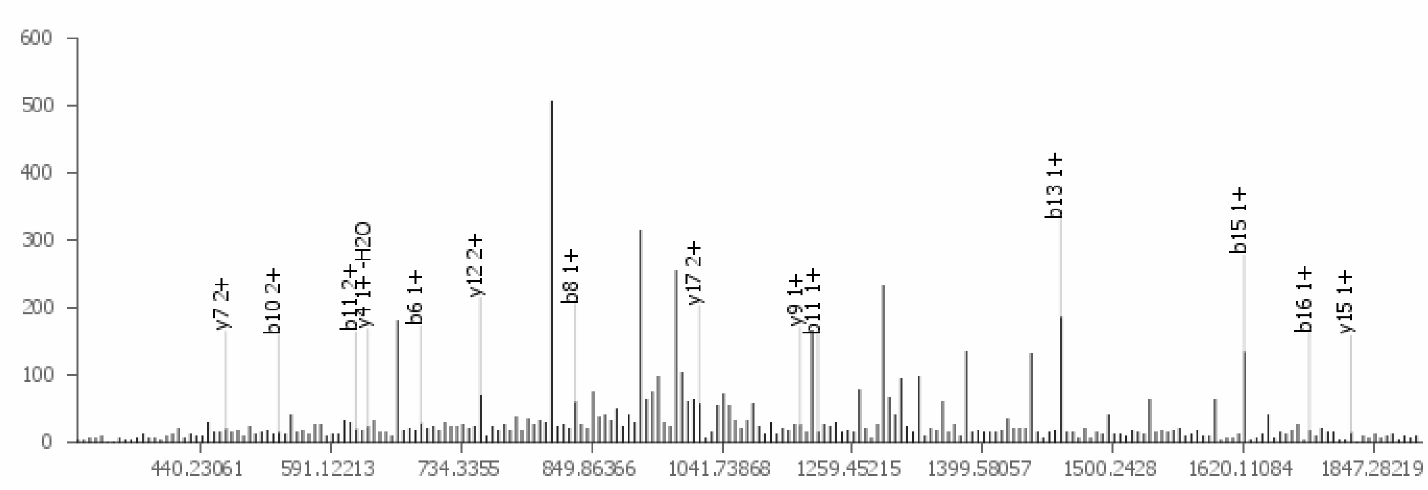

AT3G54280.1 - GTLEEKVM(pS)LQK - 721.854202 - Charge:2

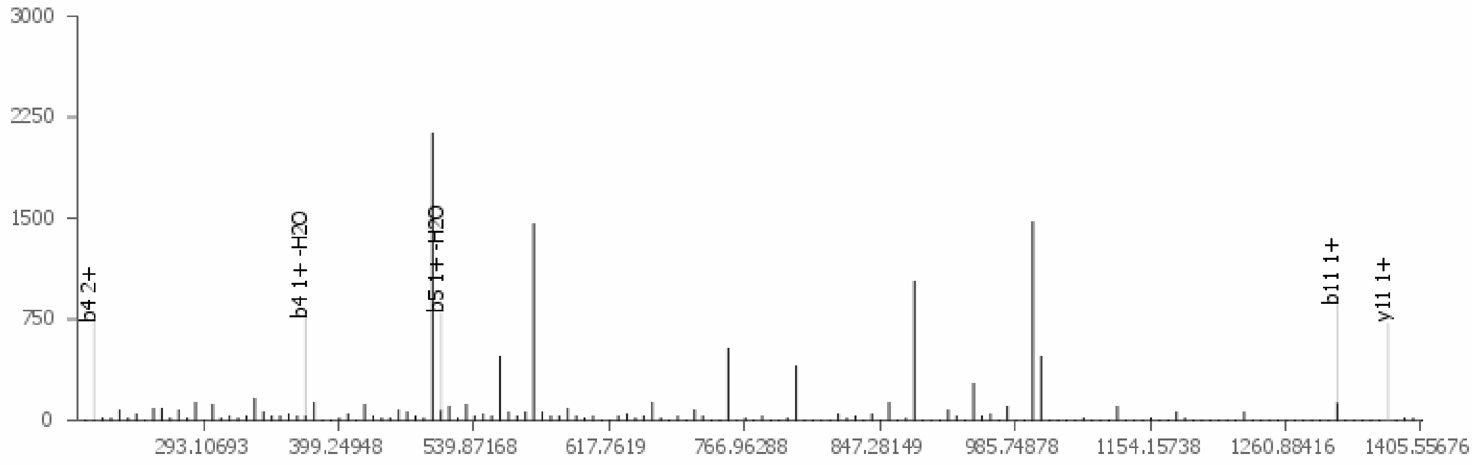

AT4G12270.1 - SIL(pS)SHALFASR - 684.838699 - Charge:2

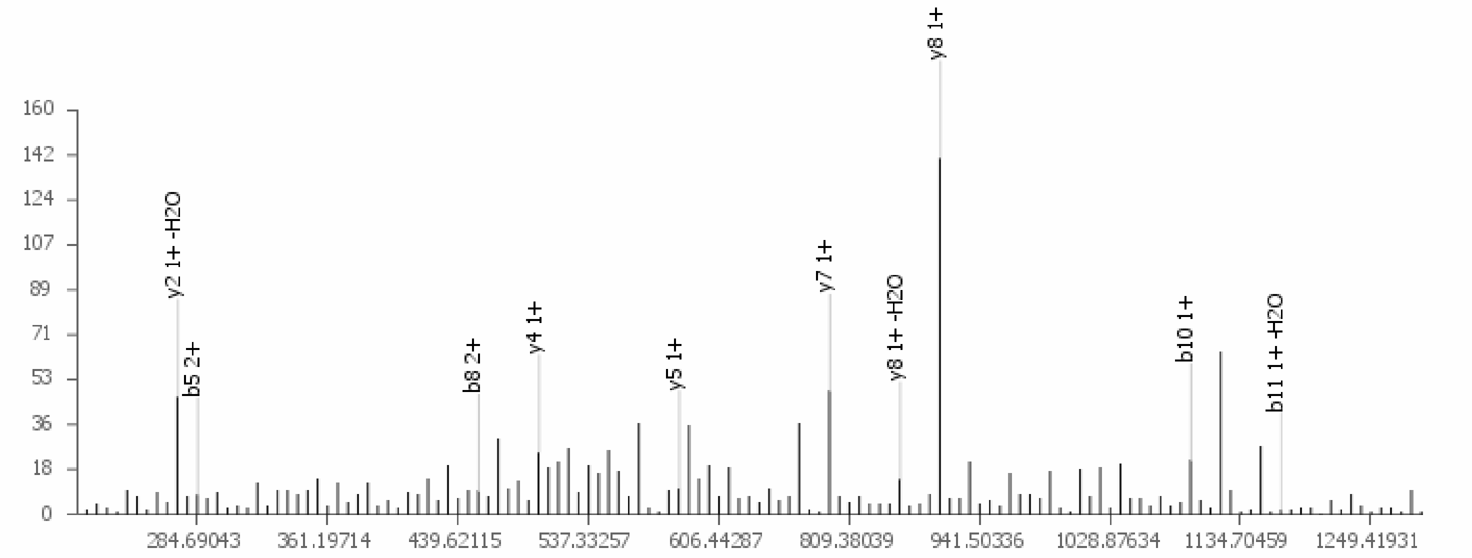

AT1G72410.1 - RL(pS)LGGGSADFSK - 687.821132 - Charge:2

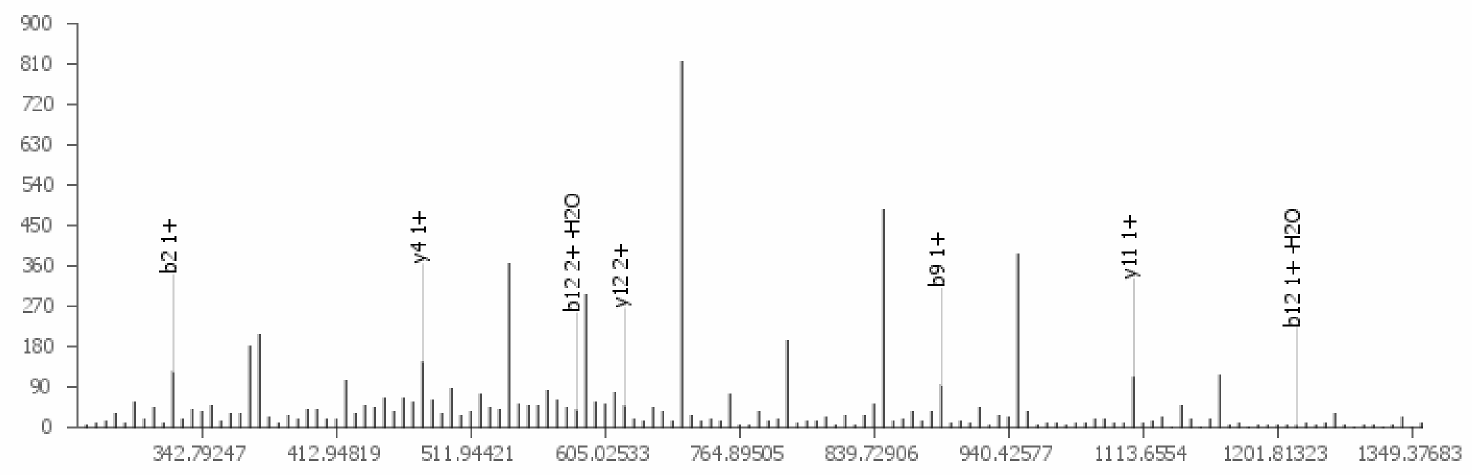

AT3G44730.1 - LTQVLQD(pS)LGGQAK - 769.388695 - Charge:2

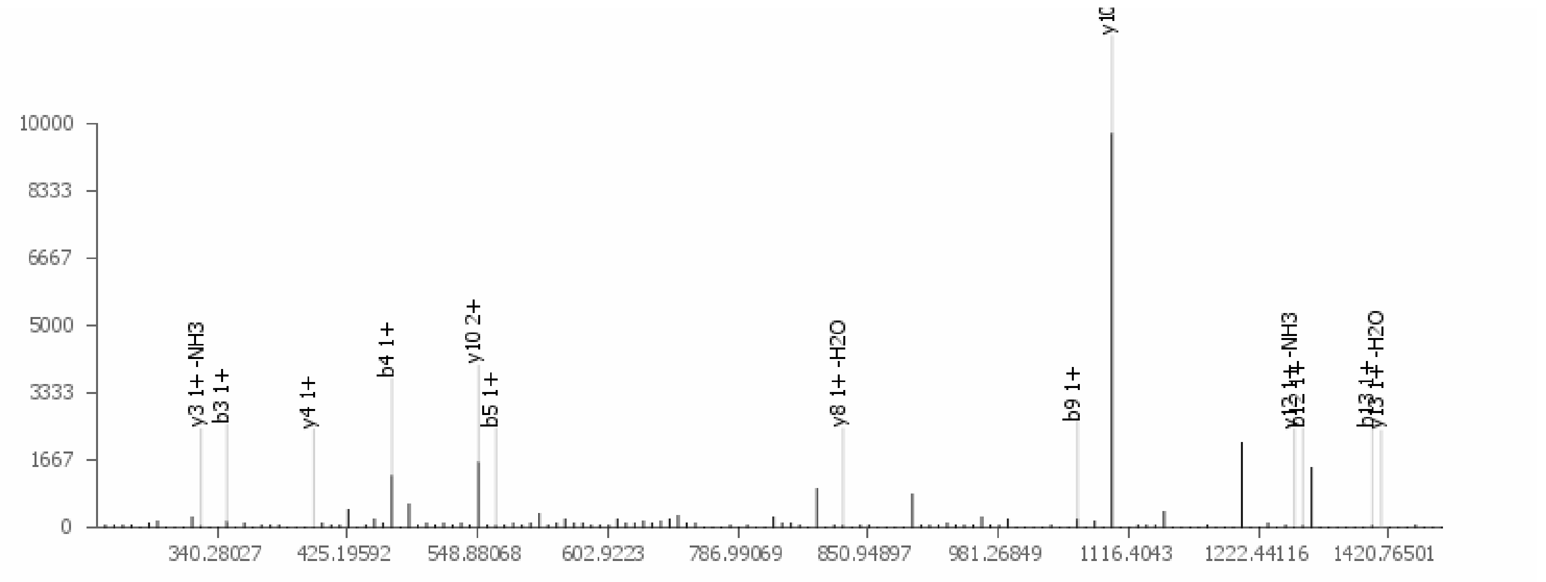

AT3G13220.1 - GITG(s)(t)GPGEILAL(oxM)GP(s)G(s)GKTTLLK - 1360.143204 - Charge:2

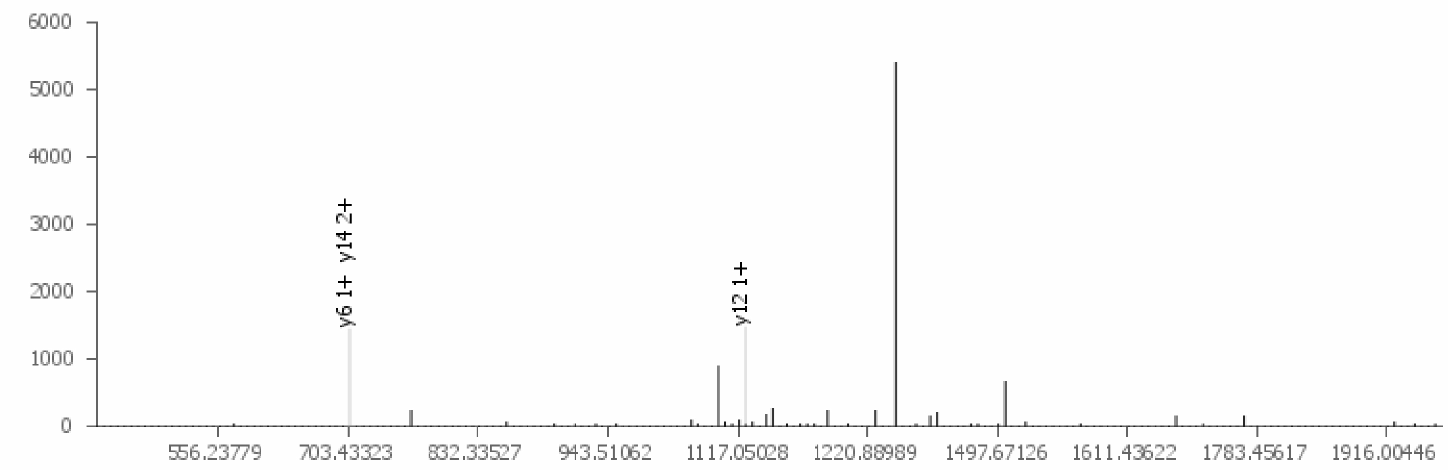

AT1G09060.1 - SCLR(pS)DNTIK - 637.282823 - Charge:2

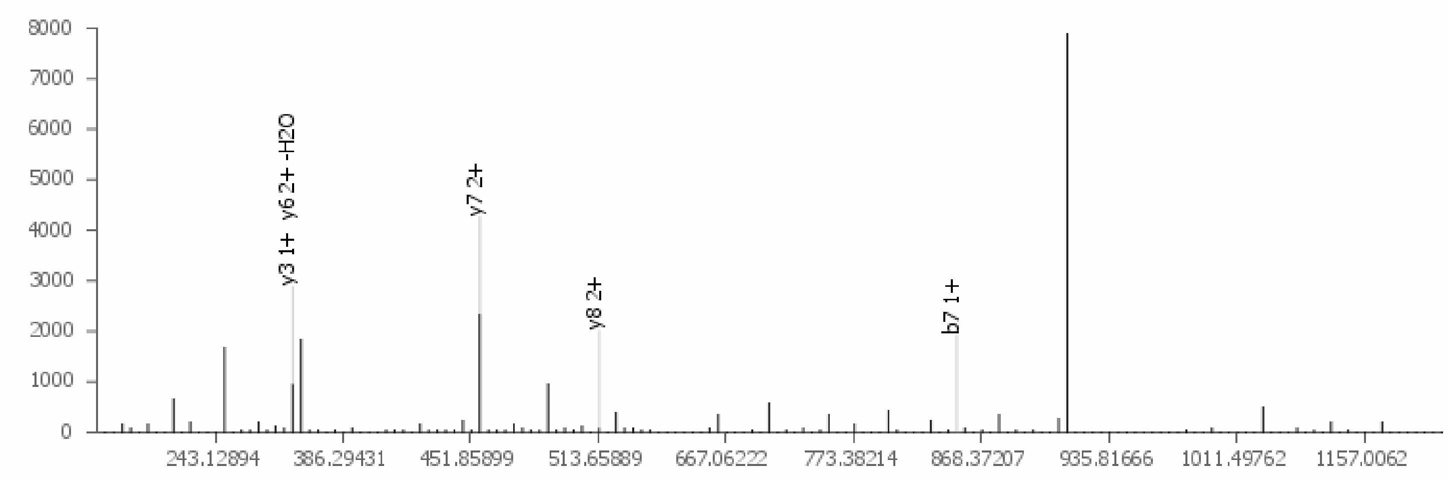

AT5G20320.1 - DEVDLSLTIP(pS)K - 698.829829 - Charge:2

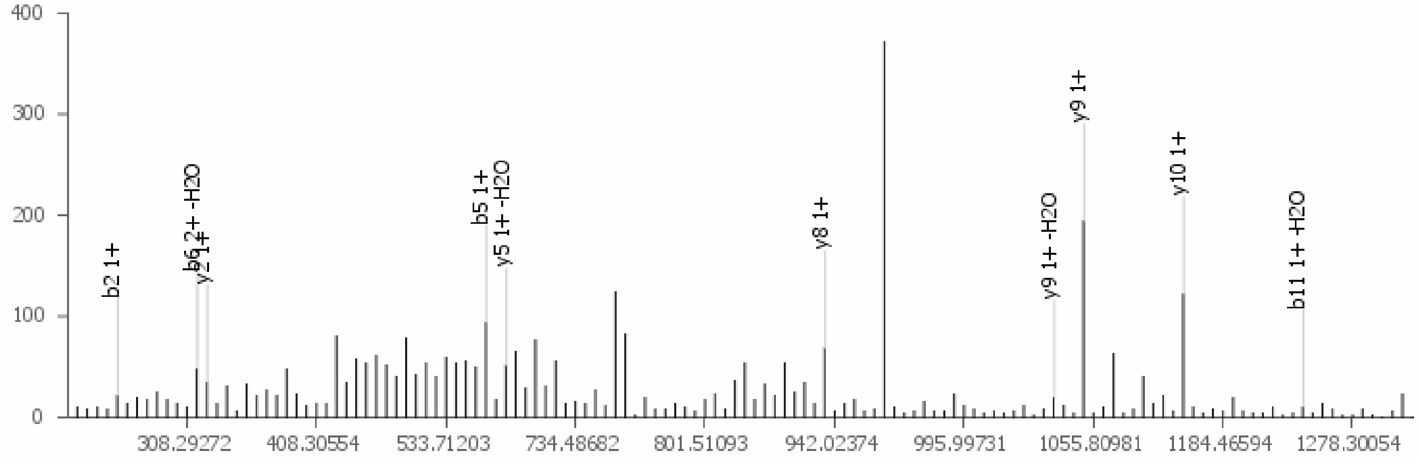

AT4G01023.1 - VAA(oxM)A(pT)KAGETAT(oxM)AAD(oxM)VK - 1048.469066 - Charge:2

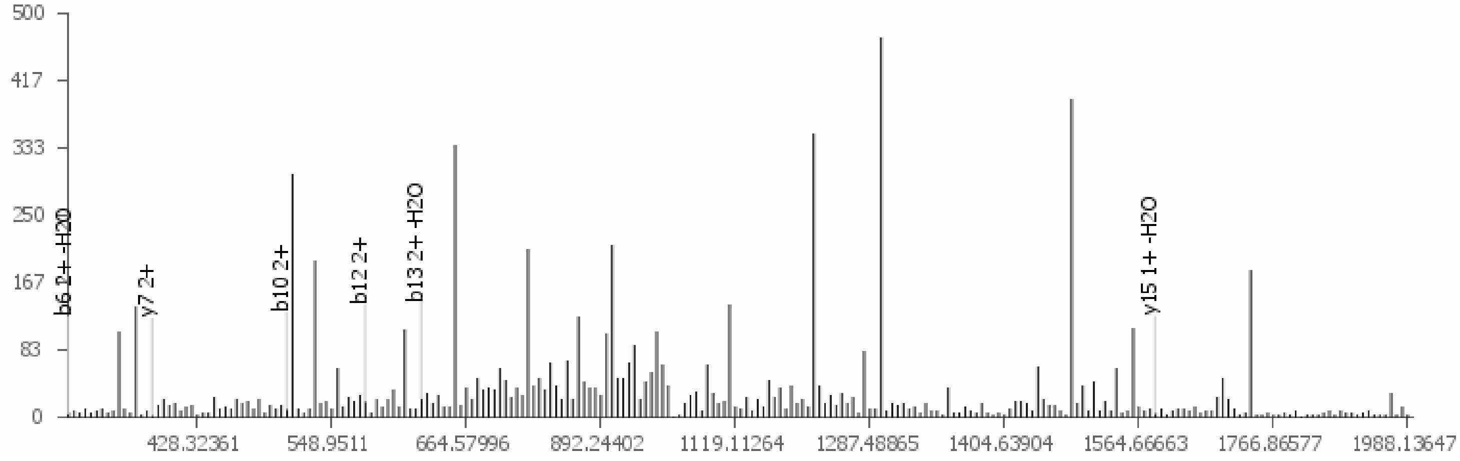

AT3G24513.1 - SLLIFISVI(pT)SNIG(pS)EARELTGAGK - 912.801002 - Charge:3

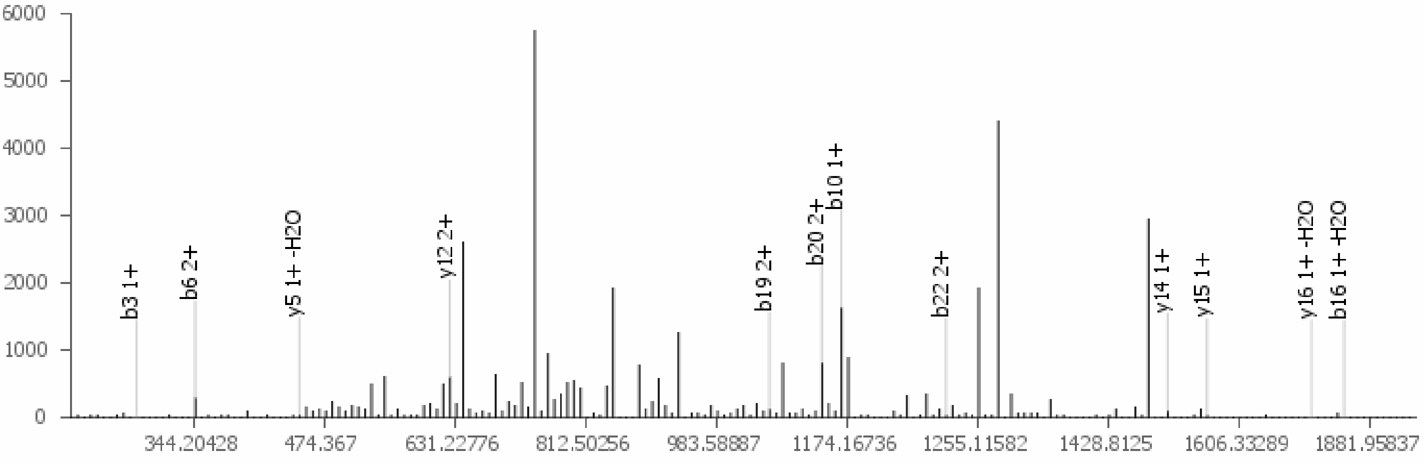

AT1G66410.1 - ADQL(pT)DEQISEFK - 802.349827 - Charge:2

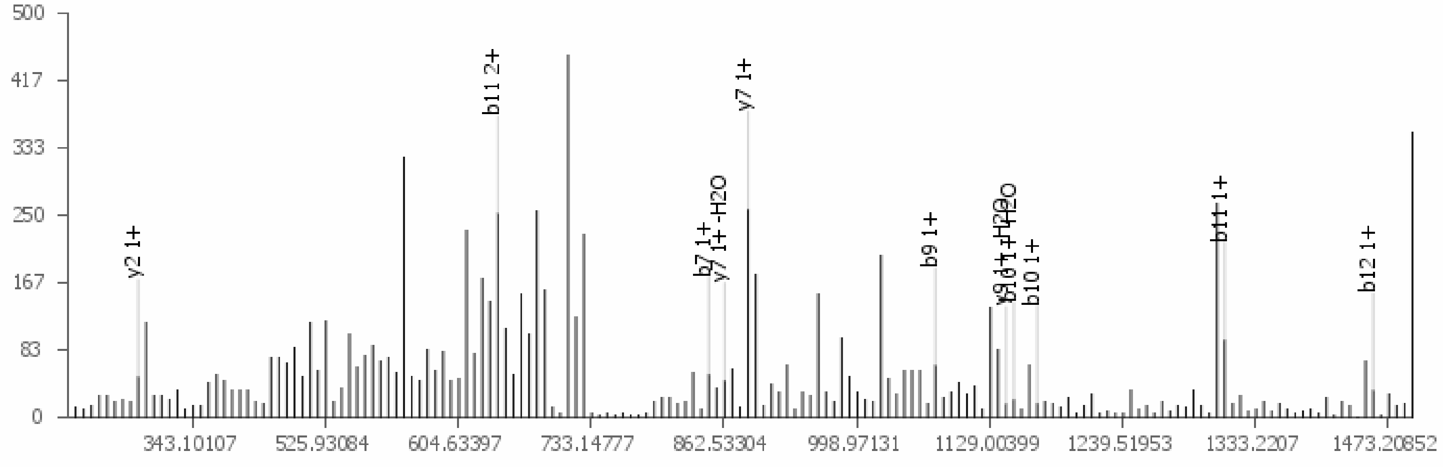

AT3G02250.1 - (pS)R(oxM)SLW(oxM)IR - 646.293366 - Charge:2

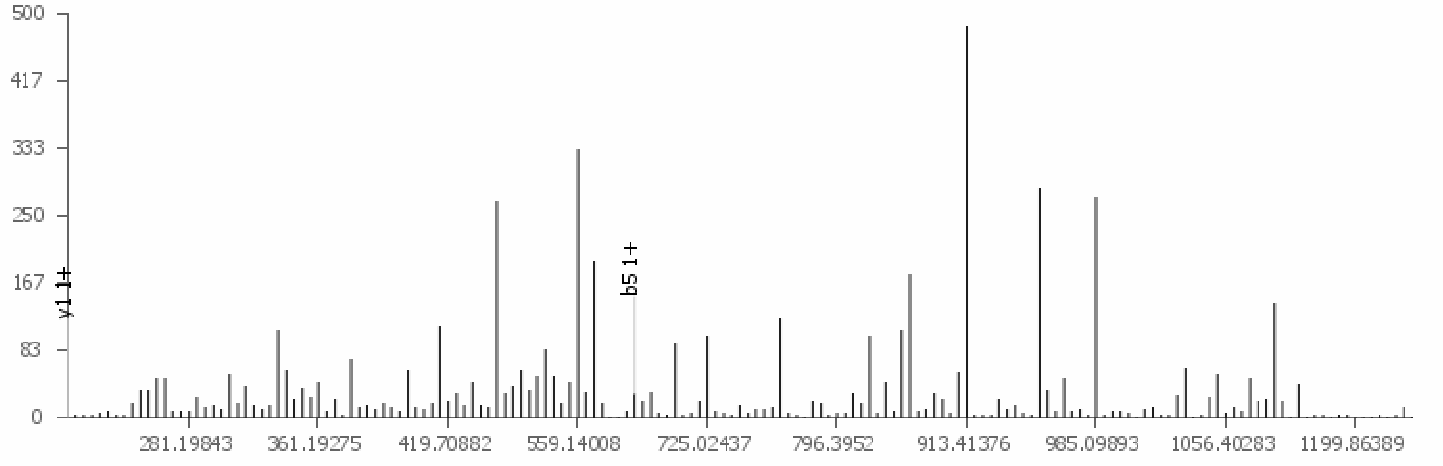

AT4G05420.1 - GDFIVVGDL(oxM)KSISLLL(pY)K - 1104.070664 - Charge:2

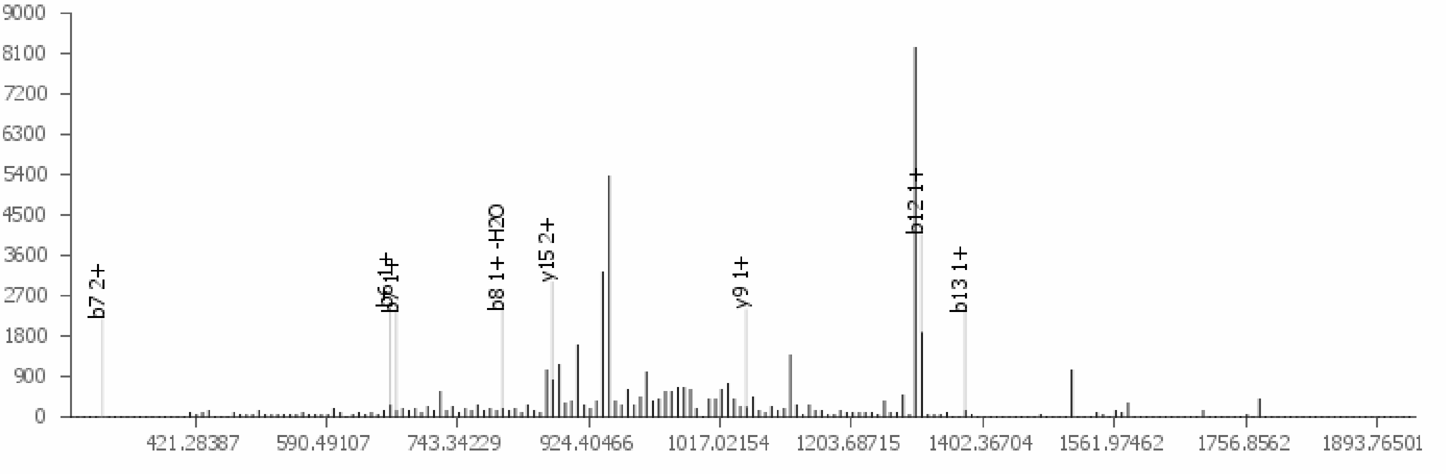

AT5G19920.1 - (oxM)R(pS)LALSPSNR - 664.306419 - Charge:2

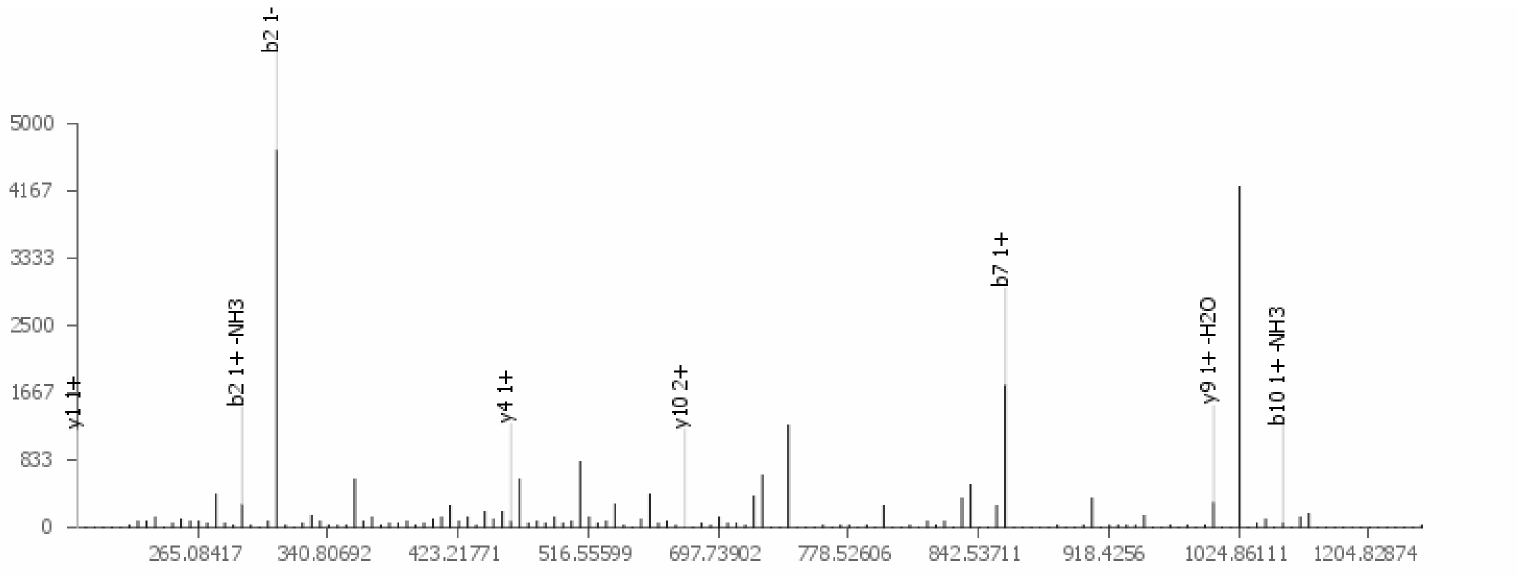

AT4G21490.1 - G(oxM)HGI(pT)DLLK - 590.780665 - Charge:2

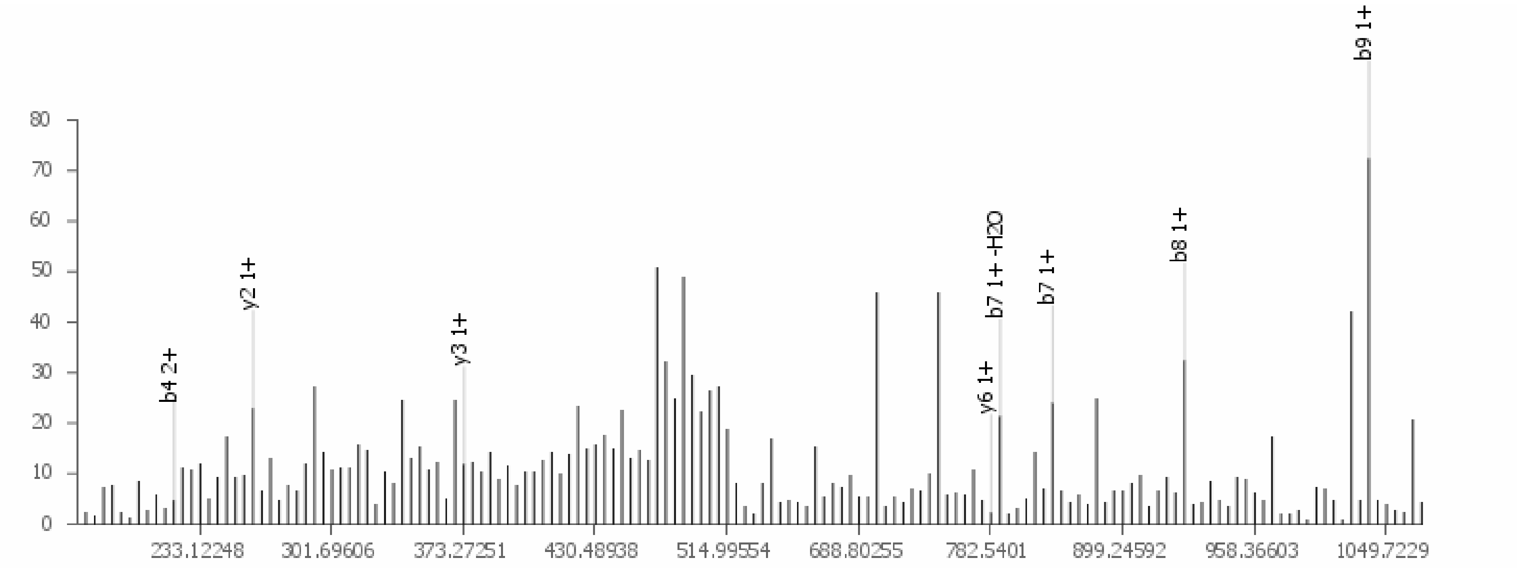

AT2G27030.1 - ADQL(pT)DDQISEFK - 795.342866 - Charge:2

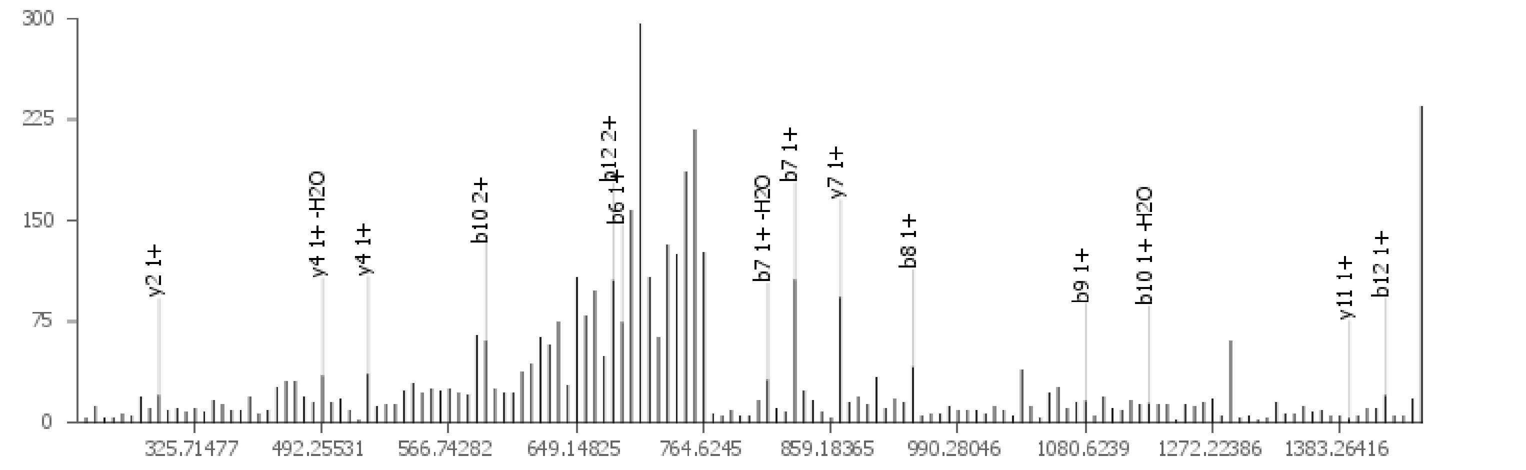

AT5G05680.1 - KNLIQDTQ(oxM)SQLQS(pT)LAK - 1072.030015 - Charge:2

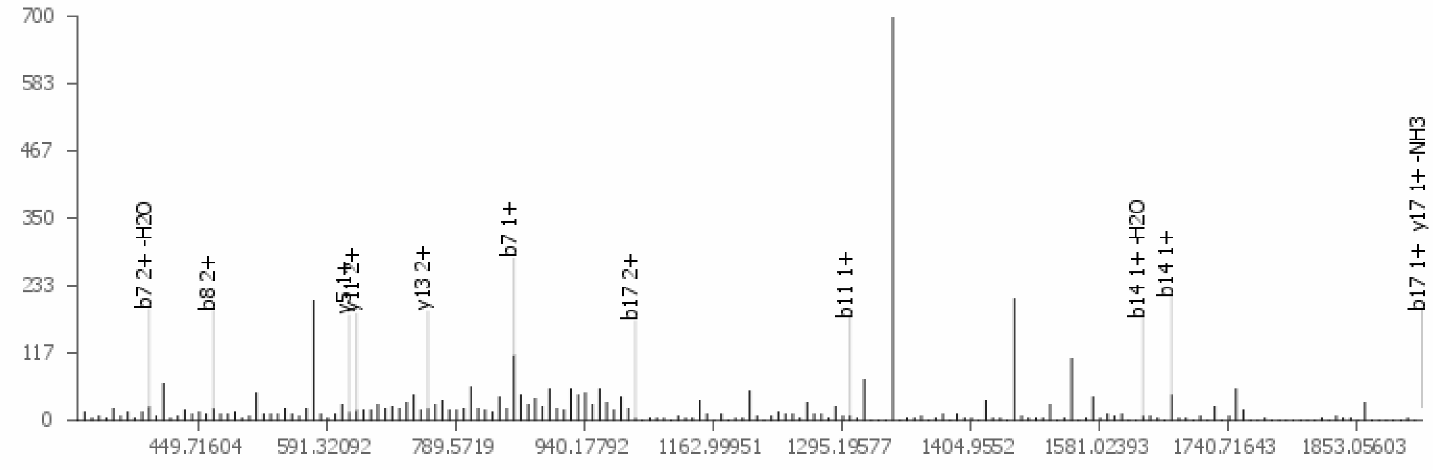

AT2G35250.1 - GAEADI(pS)IHDLK - 674.813222 - Charge:2

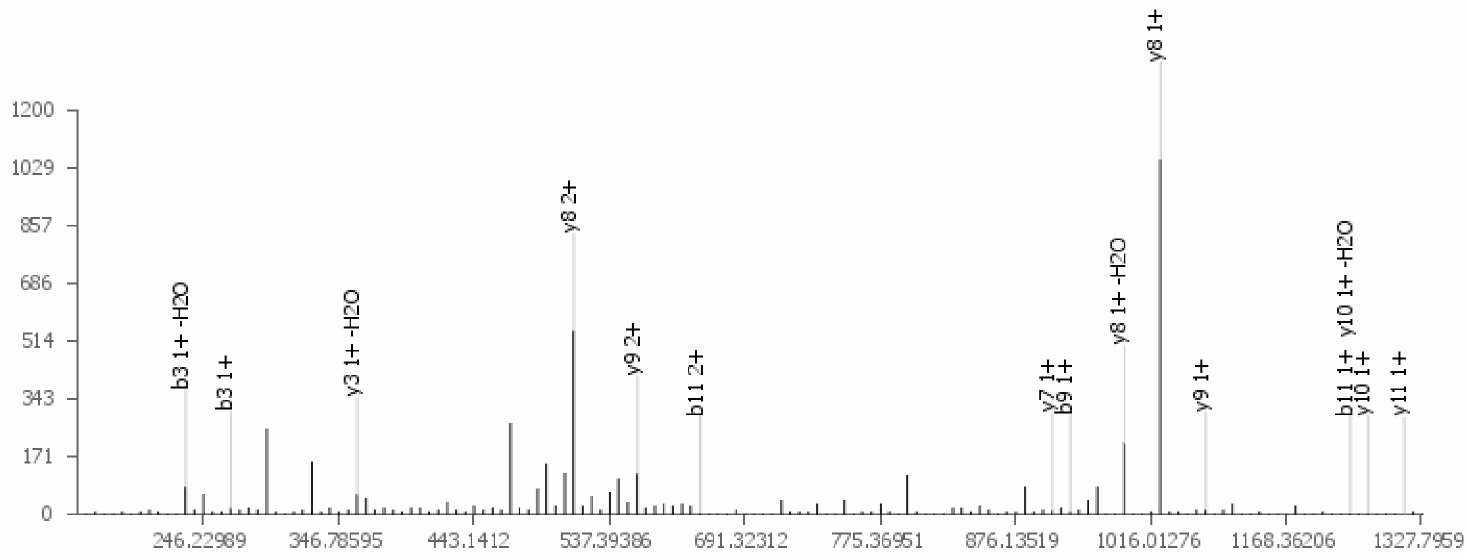

AT1G74920.1 - (pS)PLIVFDDVDLDK - 778.371566 - Charge:2

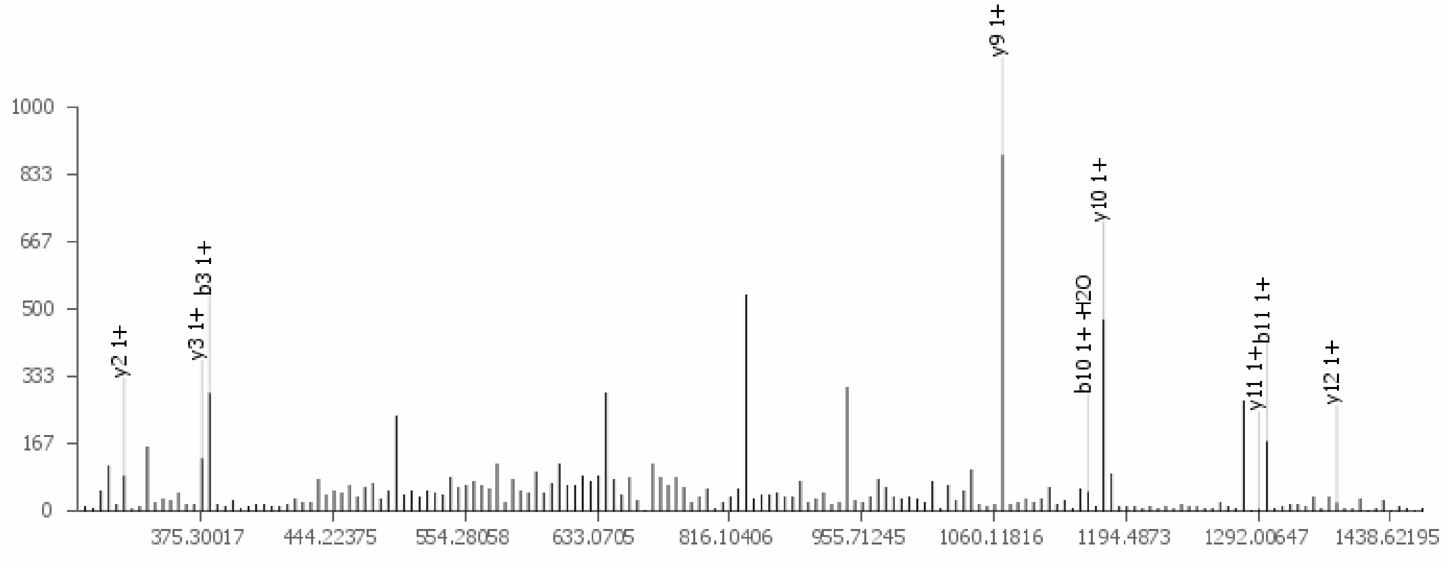

AT1G62250.1 - SSPNIAI(pS)LLR - 625.824157 - Charge:2

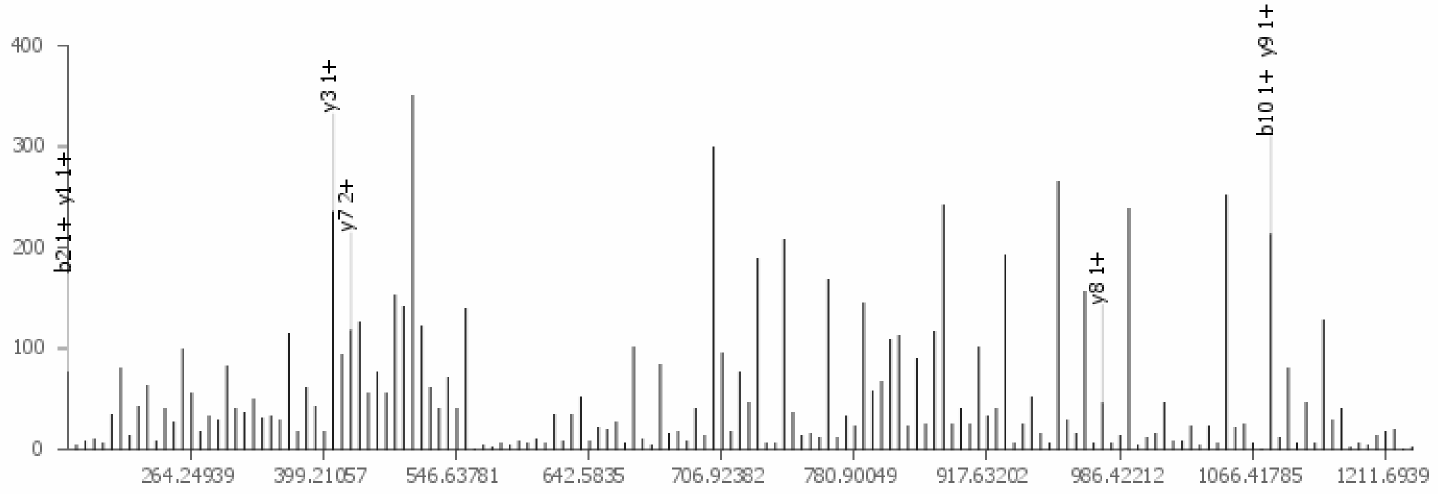

AT4G28815.1 - IGQ(t)QIP(s)(s)DPLPILRGSGSGGR - 1187.092144 - Charge:2

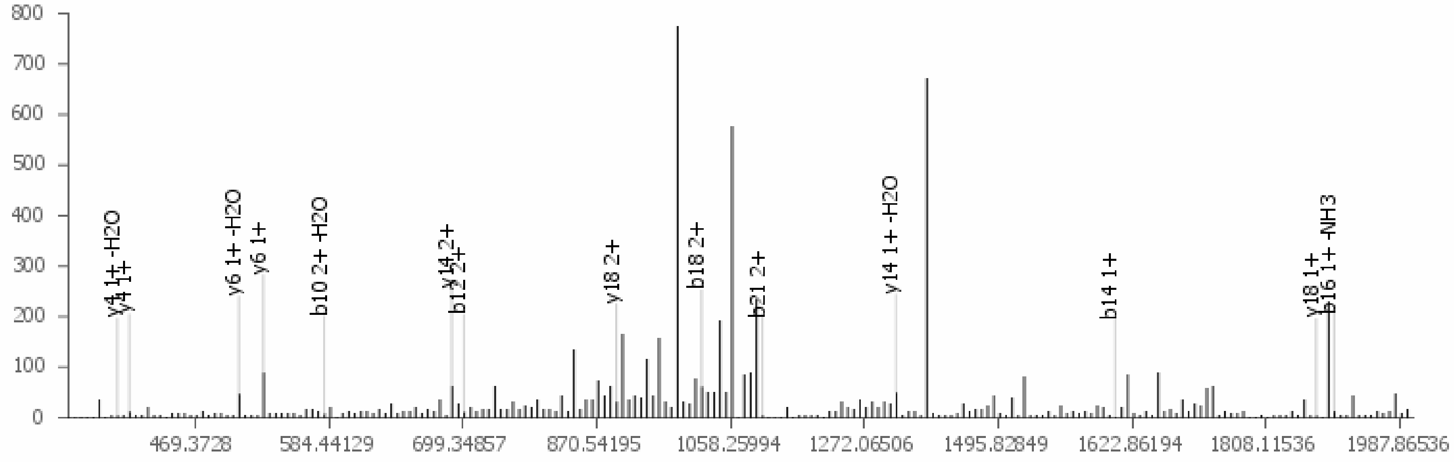

AT2G19570.1 - LLLE(t)I(s)PK - 547.299179 - Charge:2

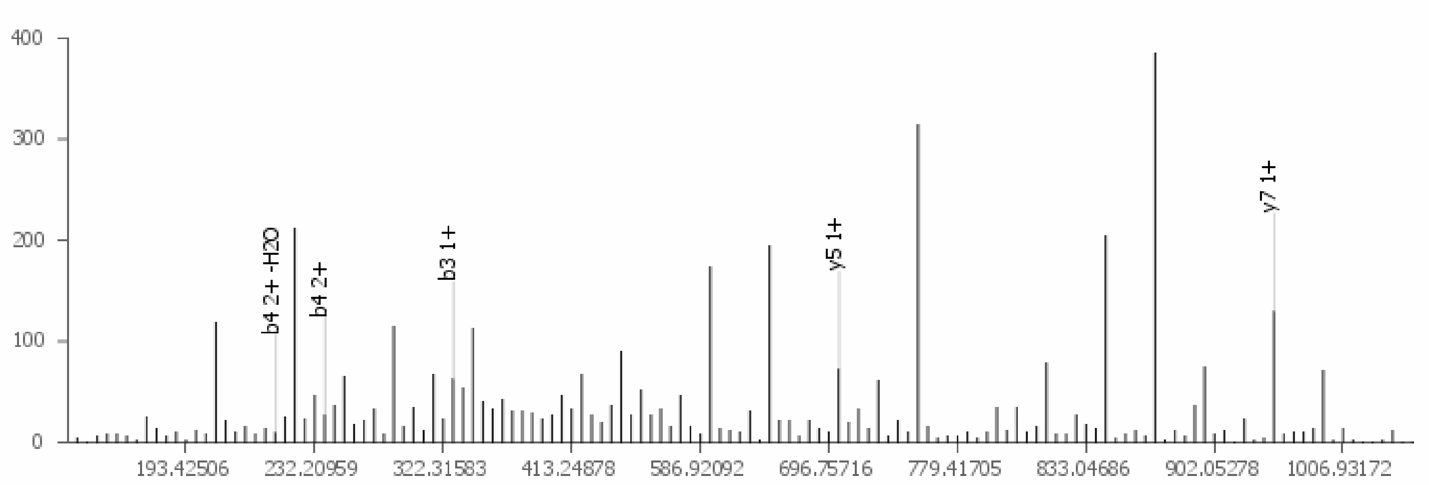

AT5G60610.1 - IISFVP(pT)KVAV(pS)T(pS)IL(pS)K - 1105.491395 - Charge:2

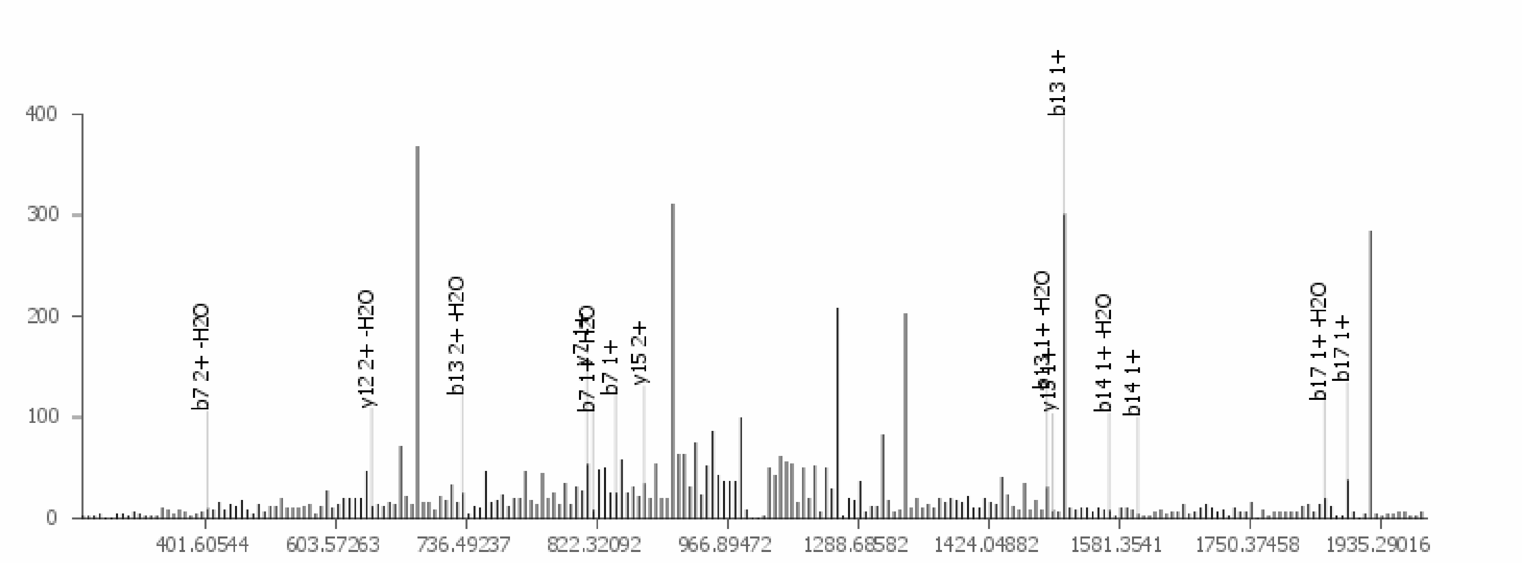

AT3G08550.1 - EA(pT)RNNPNYFLTYGNGK - 1019.960269 - Charge:2

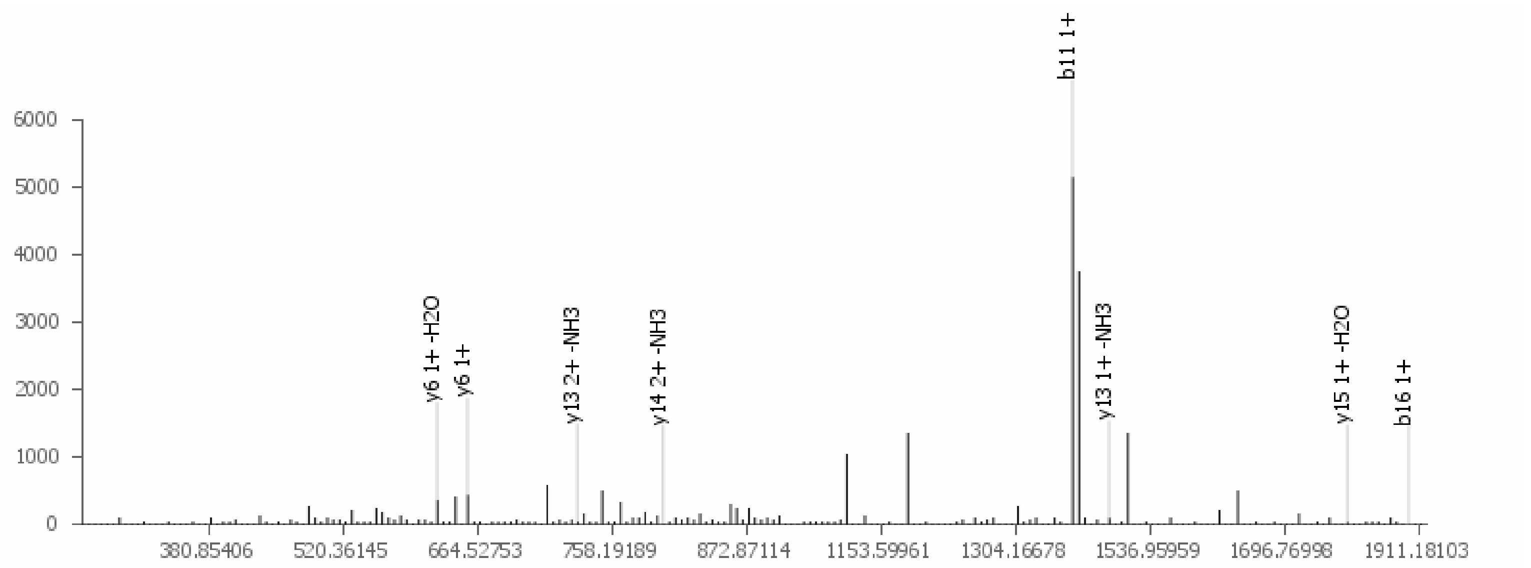

AT2G34310.1 - QLNSCPENFTQ(pT)SR - 881.362686 - Charge:2

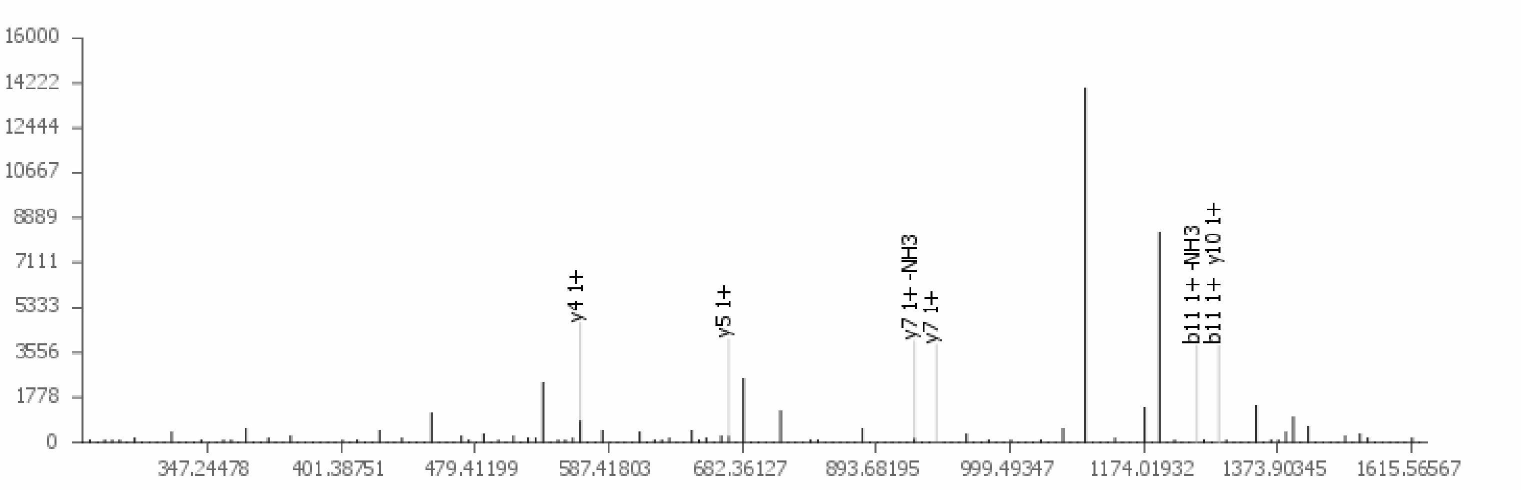

AT2G28910.1 - NFL(pS)(pT)KEDK - 621.240711 - Charge:2

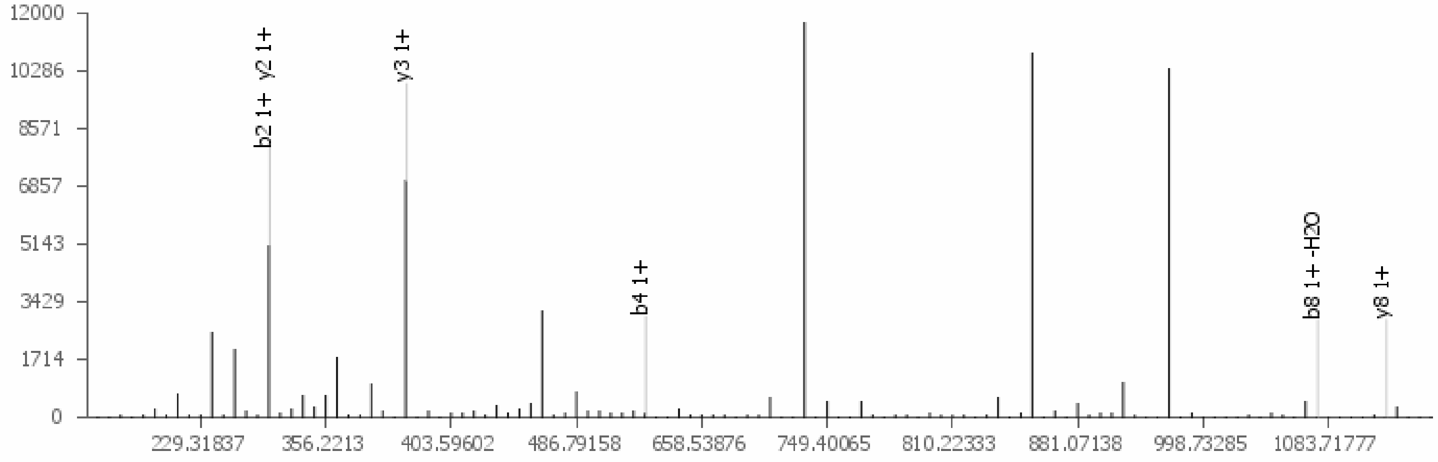

AT5G54250.1 - CA(pS)LGEDKLR - 614.779143 - Charge:2

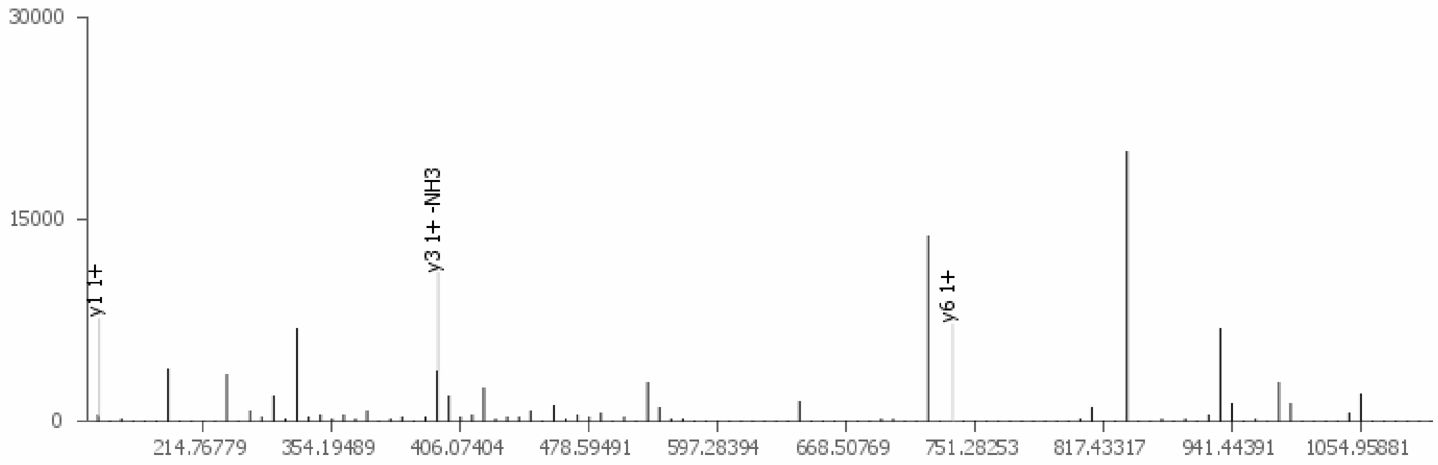

AT1G60930.1 - (pY)DISSGSEER - 611.738041 - Charge:2

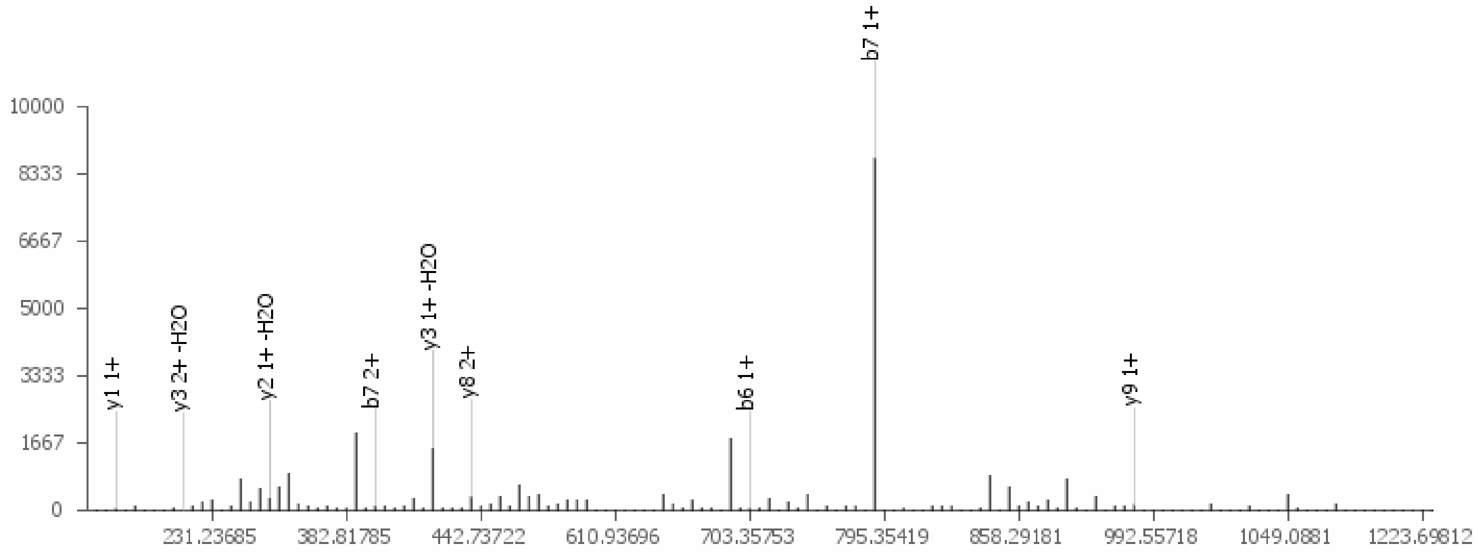

AT3G44730.1 - DLLVSDG(pS)SRR - 642.803519 - Charge:2

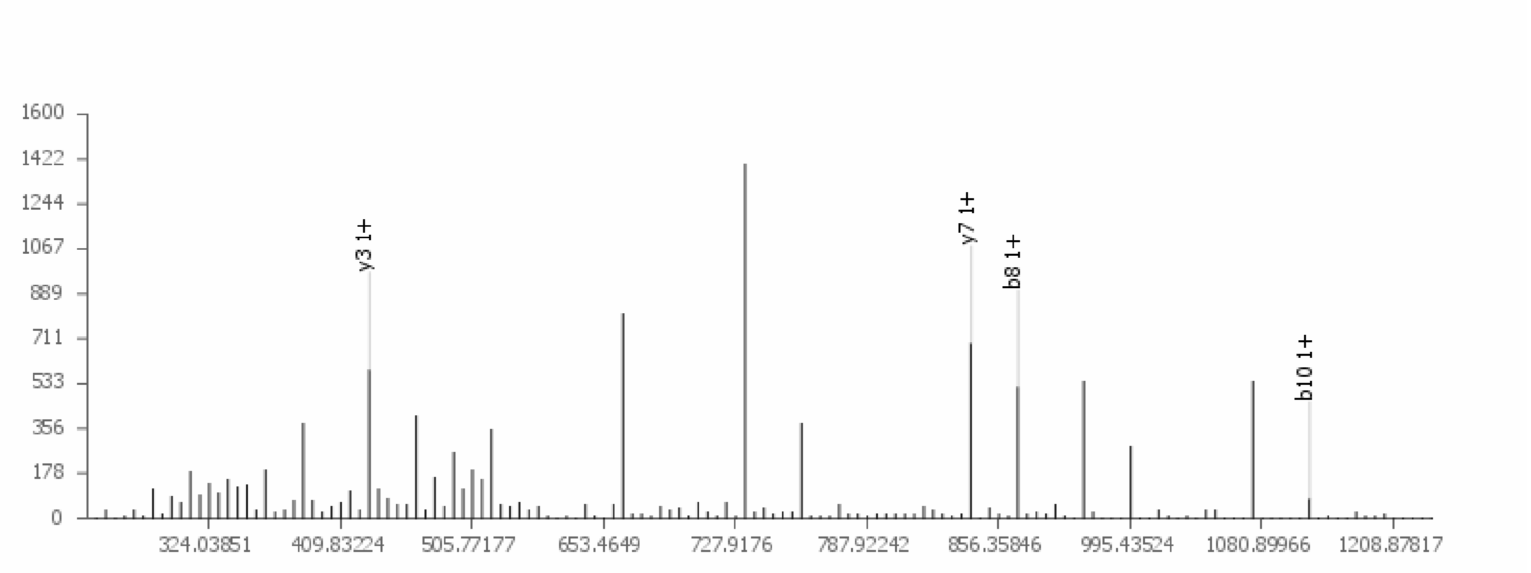

AT4G35650.1 - GGLA(pT)PVGGGV(pS)SLN(oxM)QLR - 995.449024 - Charge:2

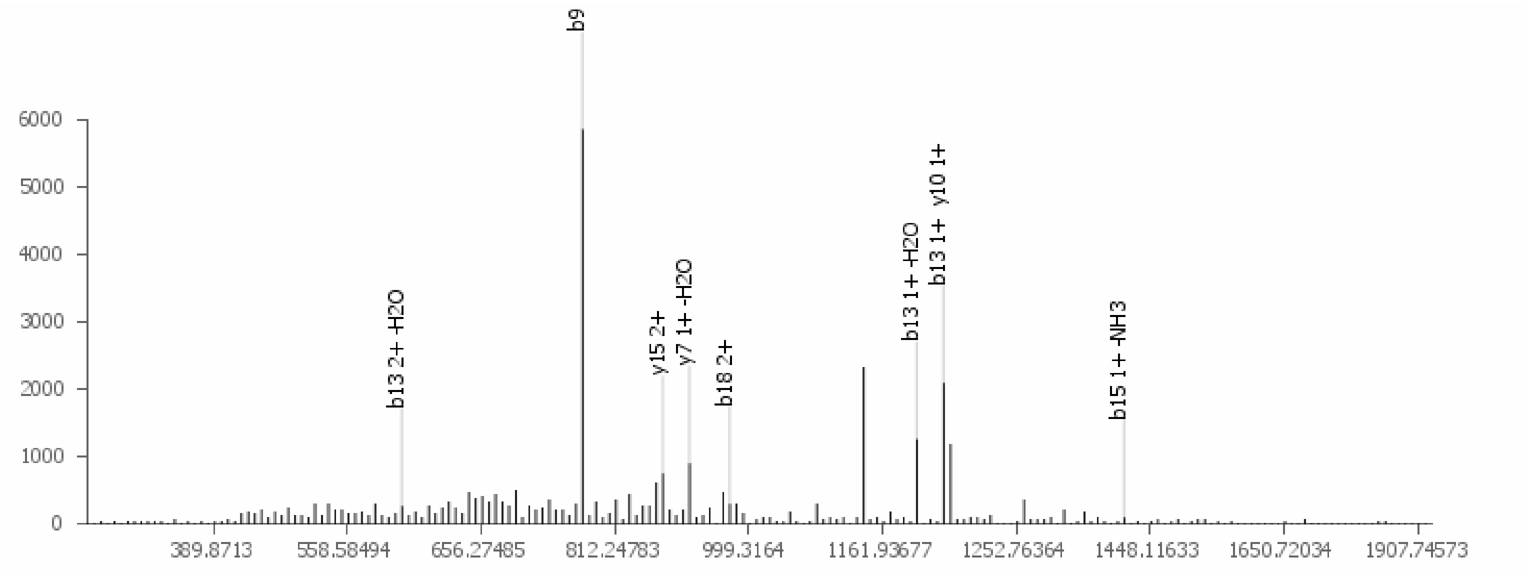

AT4G08810.1 - LDECDV(pS)WR - 630.242698 - Charge:2

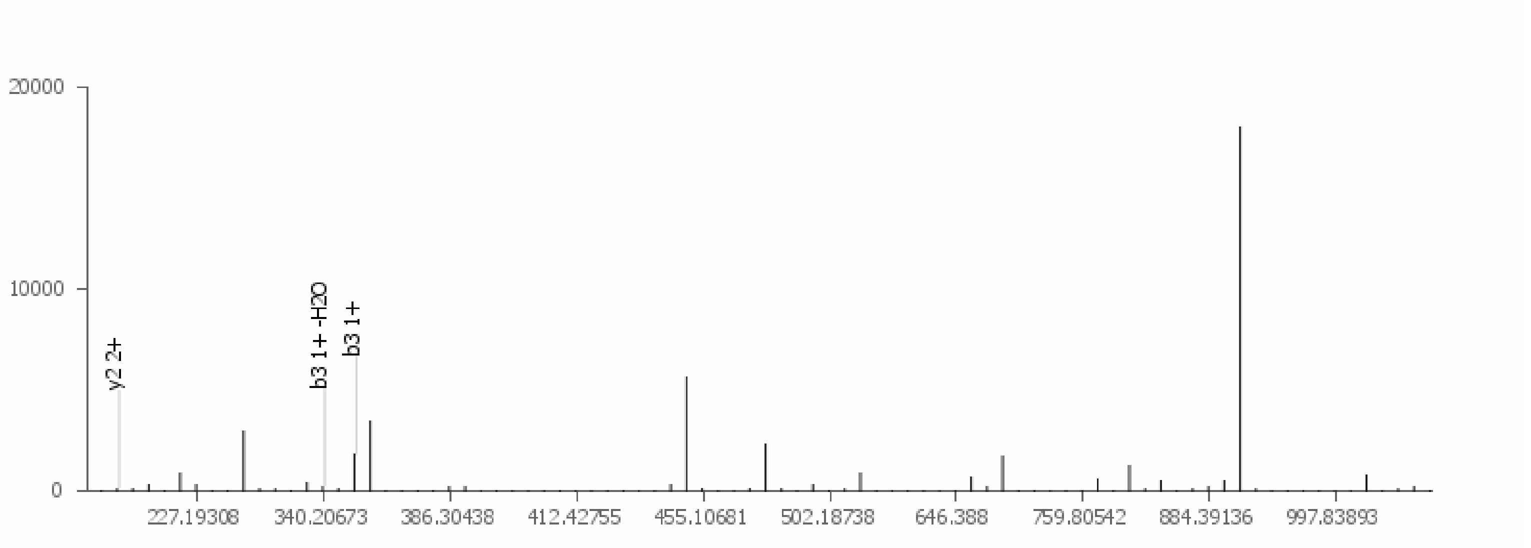

AT5G16260.1 - LL(oxM)(s)V(s)R - 451.211746 - Charge:2

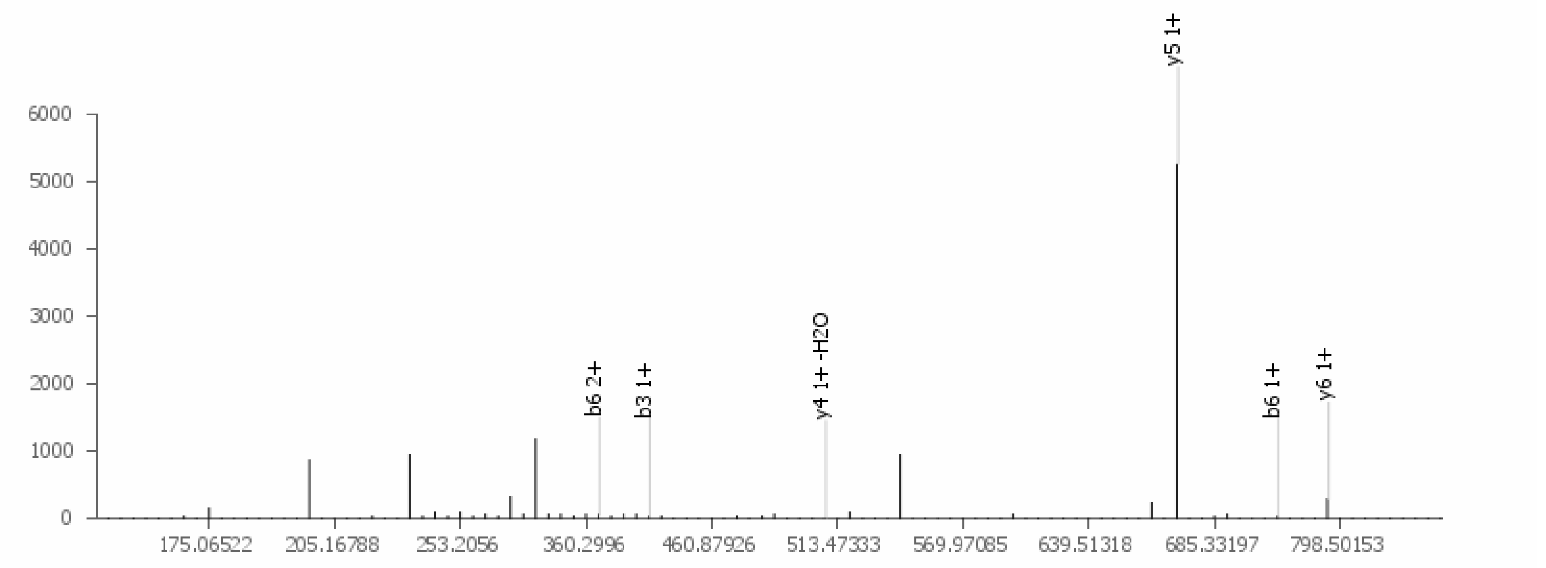

AT1G62975.1 - (pS)LCDITYGANEANKNDDDR - 1125.963095 - Charge:2

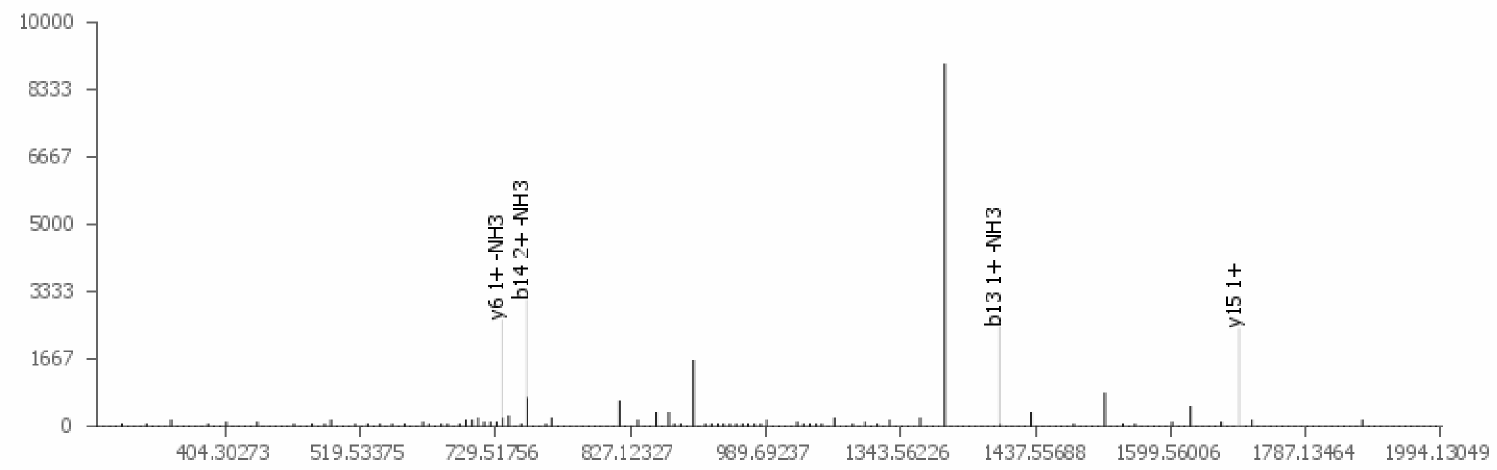

AT1G50750.1 - DKDANE(pS)AETLK - 700.800697 - Charge:2

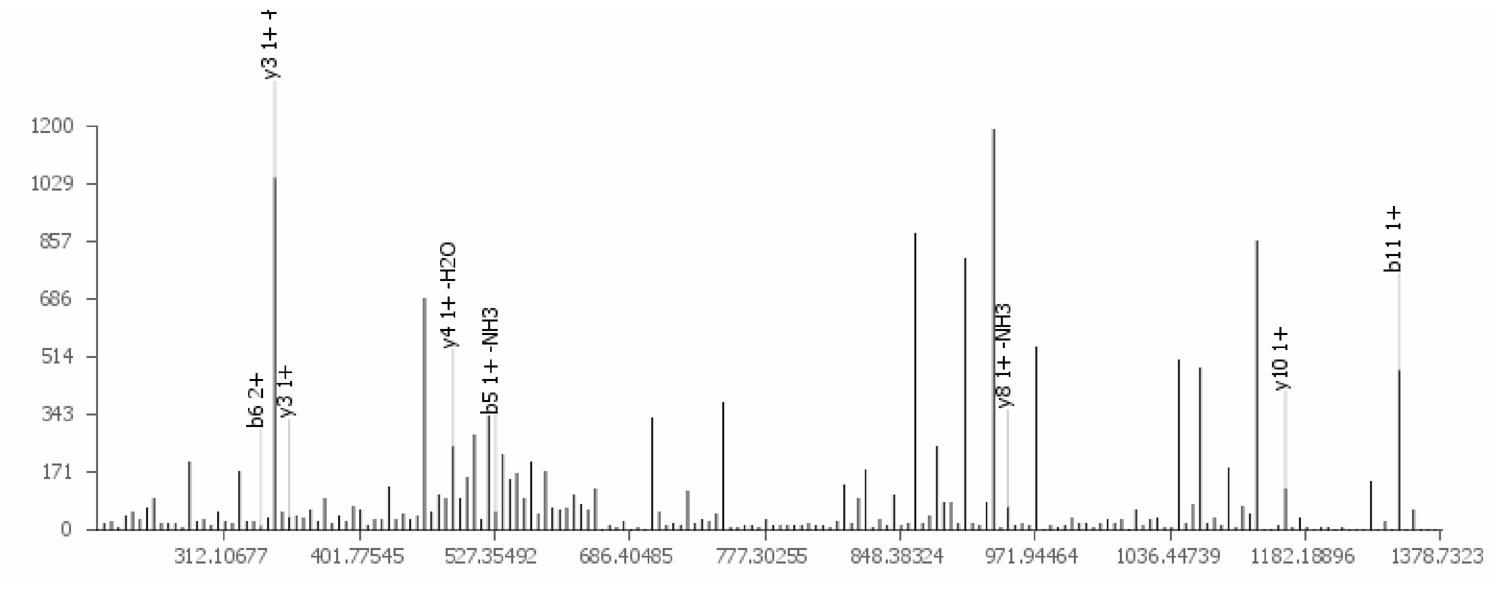

AT1G23440.1 - E(pT)(s)C(s)(t)E(s)IFQLLKK - 965.925209 - Charge:2

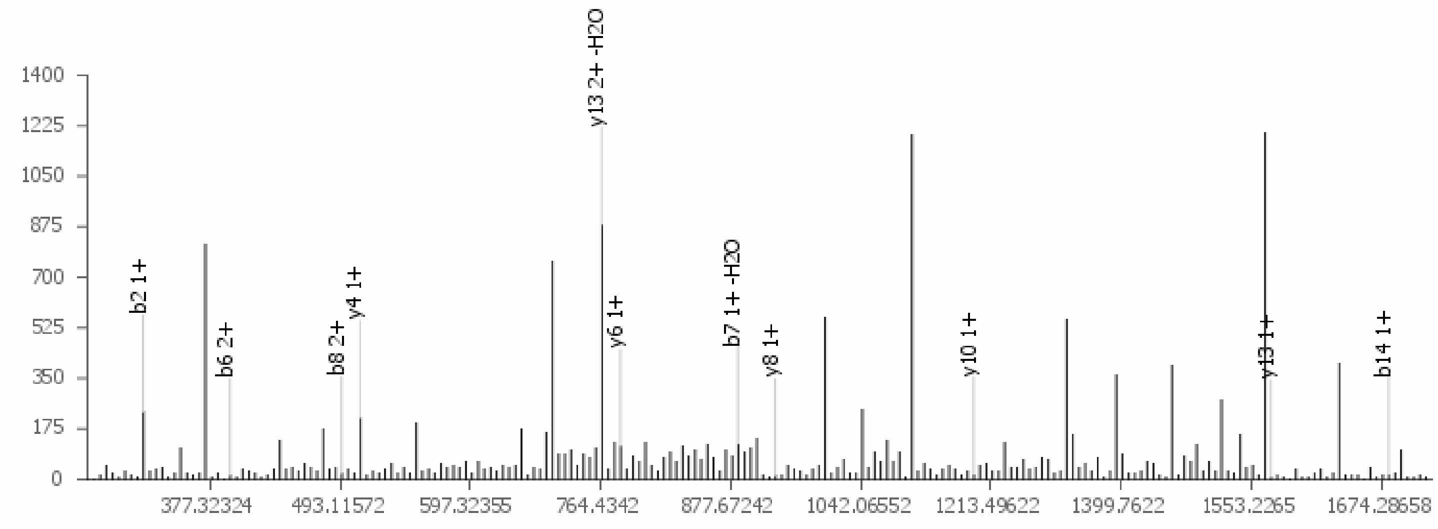

AT1G80170.1 - VIAPATSPNTDGIHISV(pS)R - 1007.992119 - Charge:2

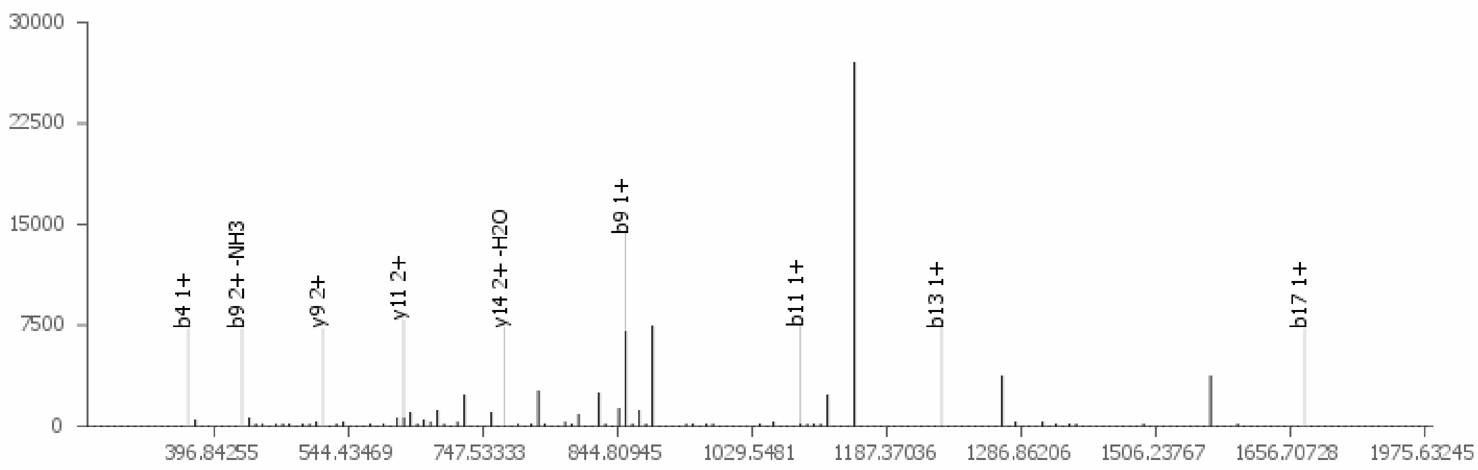

AT2G28290.1 - MD(pT)MQNN(pT)(pS)IDIGITSGK - 1083.407821 - Charge:2

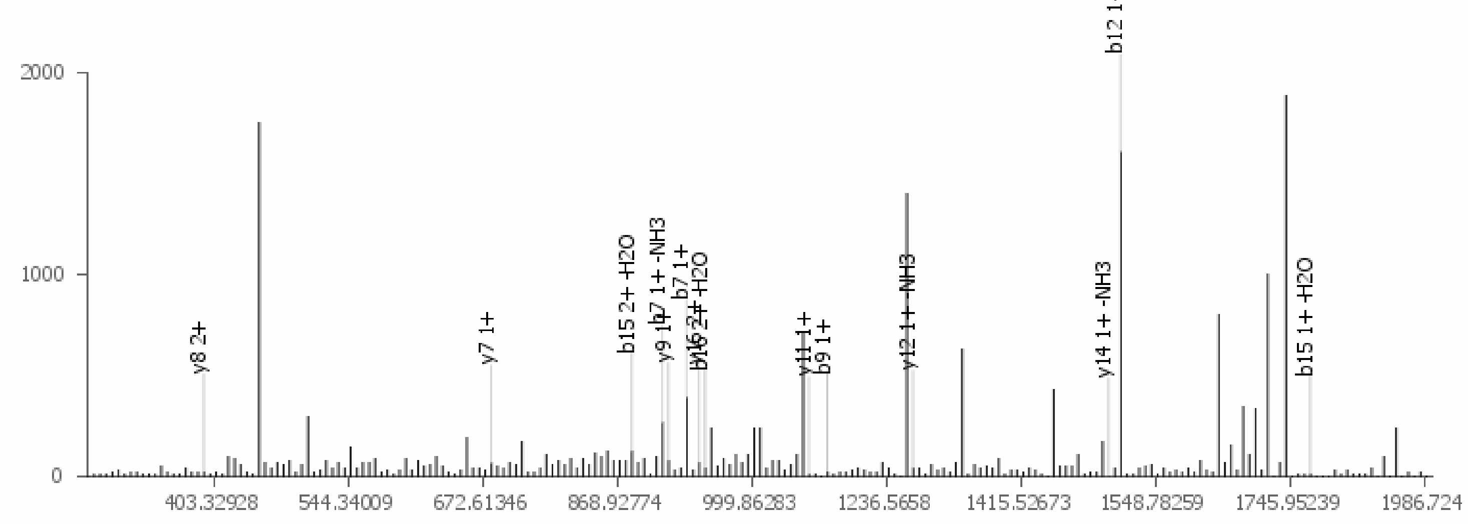

AT5G01490.1 - R(pT)V(pS)ASSLIR - 625.286558 - Charge:2

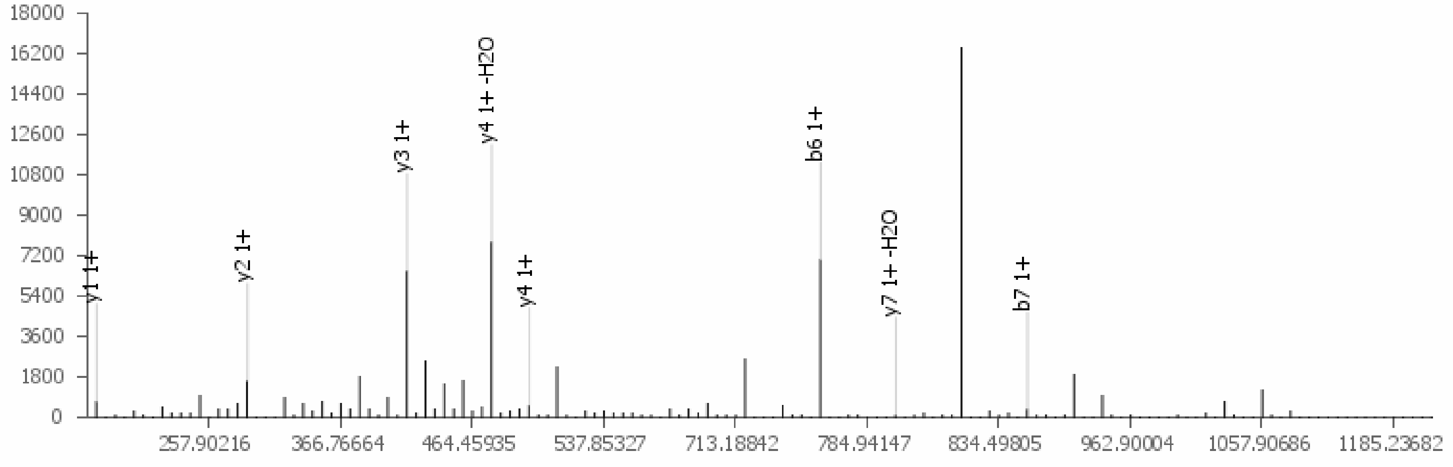

AT3G50370.1 - KSN(pY)VL(s)(s)SR - 650.769903 - Charge:2

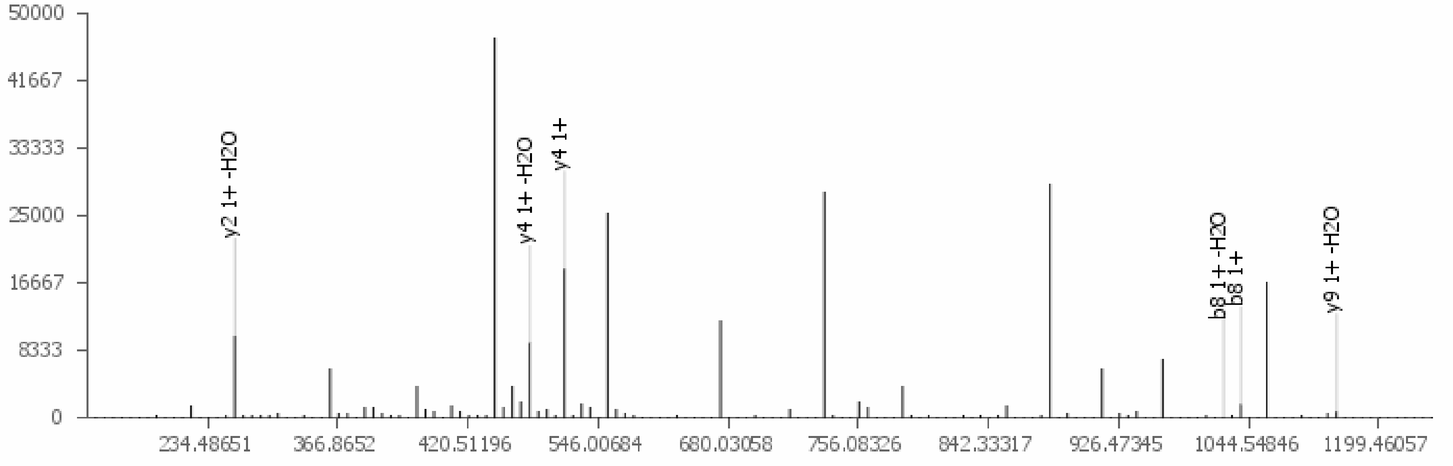

AT3G28510.1 - LLLD(t)(t)(s)K - 525.730482 - Charge:2

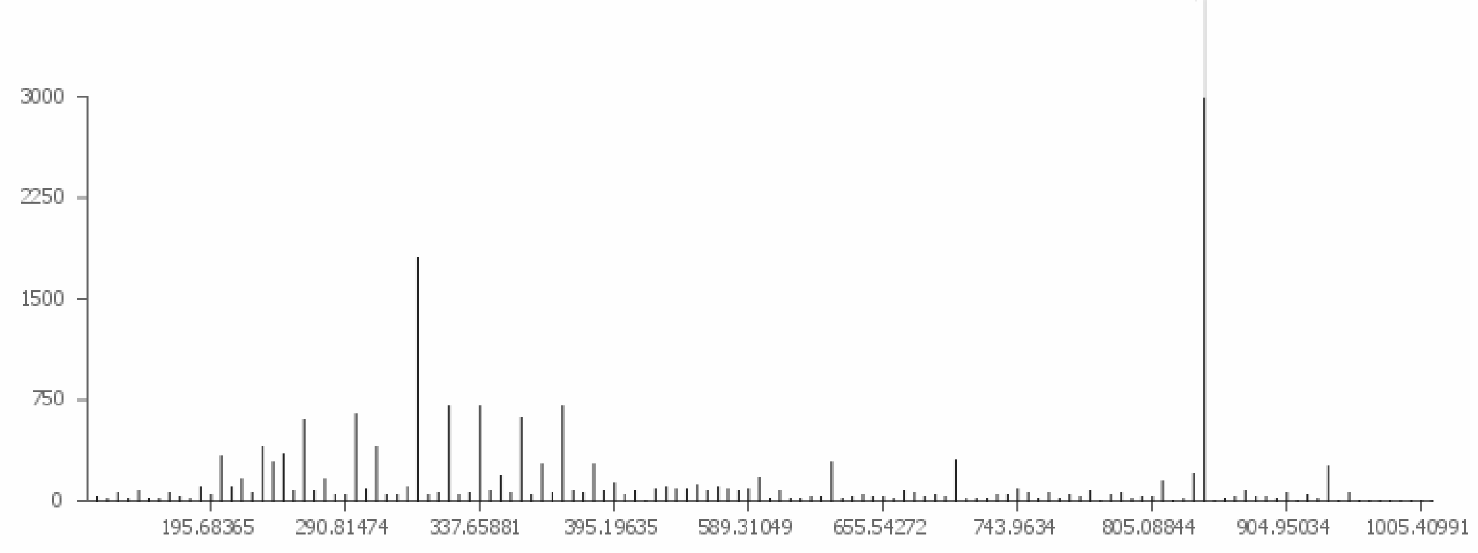

AT1G68560.1 - DFN(oxM)(pS)WK - 512.187854 - Charge:2

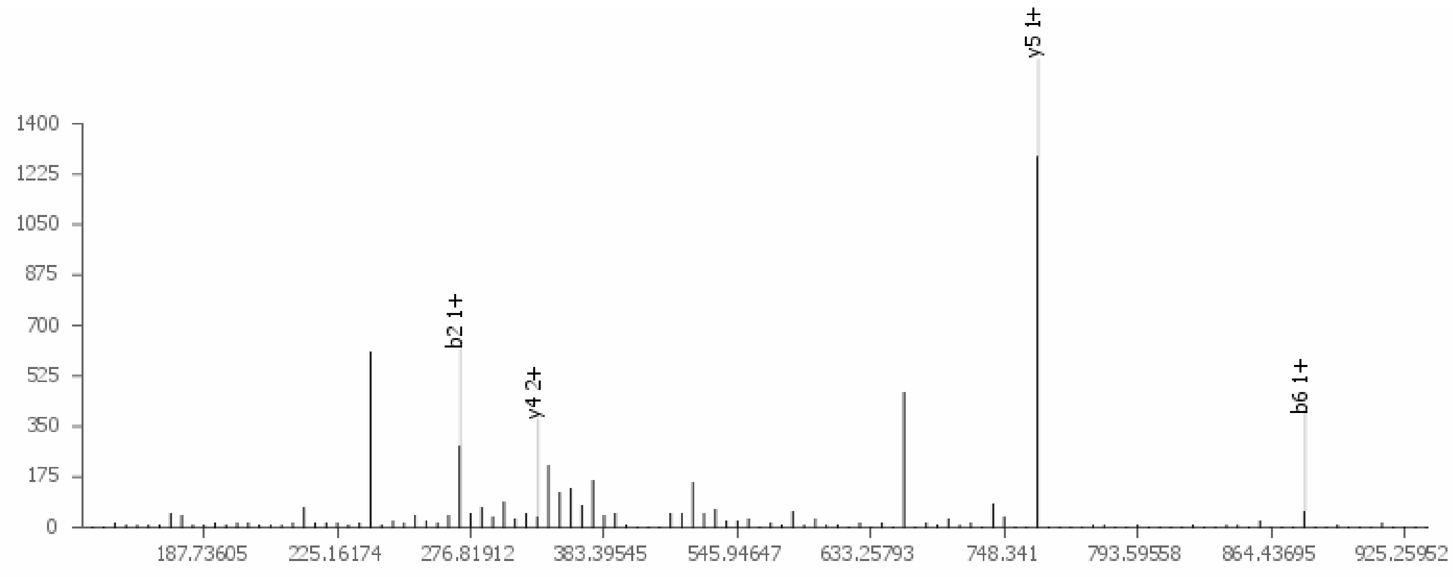

AT5G44800.1 - FLDSLS(pS)LP(pS)KSSR - 561.913496 - Charge:3

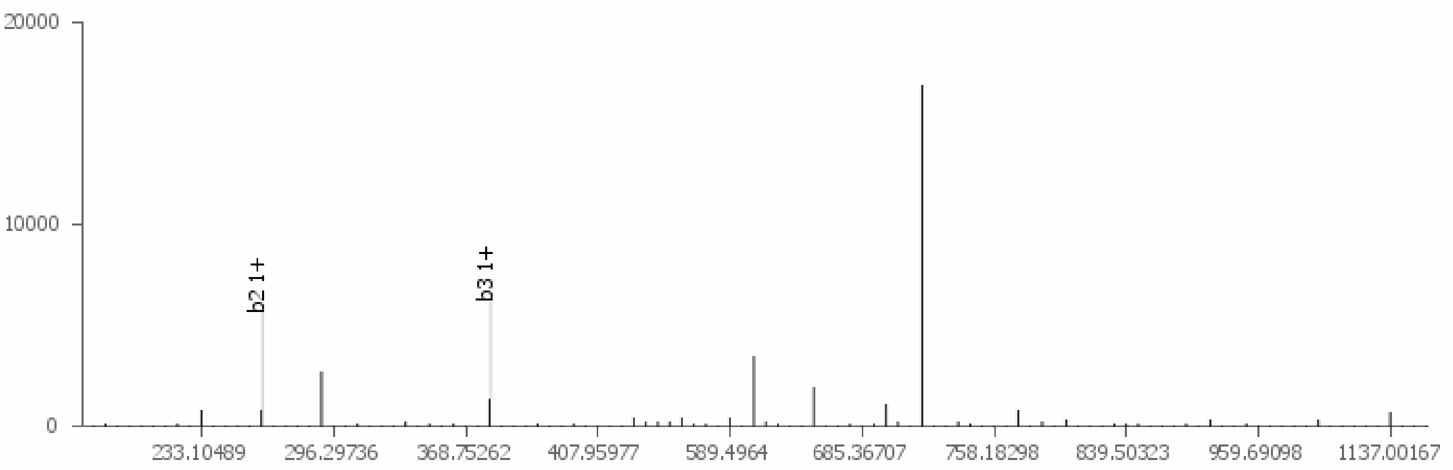

AT1G74720.1 - VKI(pY)G(pS)QFSR - 672.786348 - Charge:2

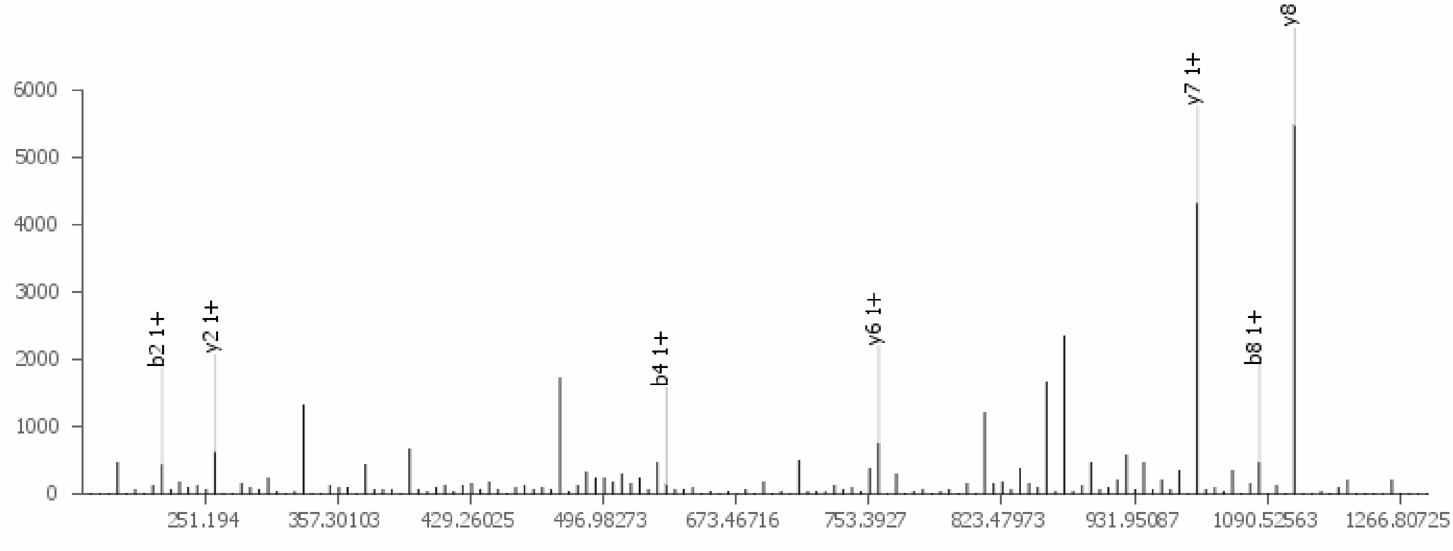

AT5G09630.1 - (pY)CDILREGK - 617.269357 - Charge:2

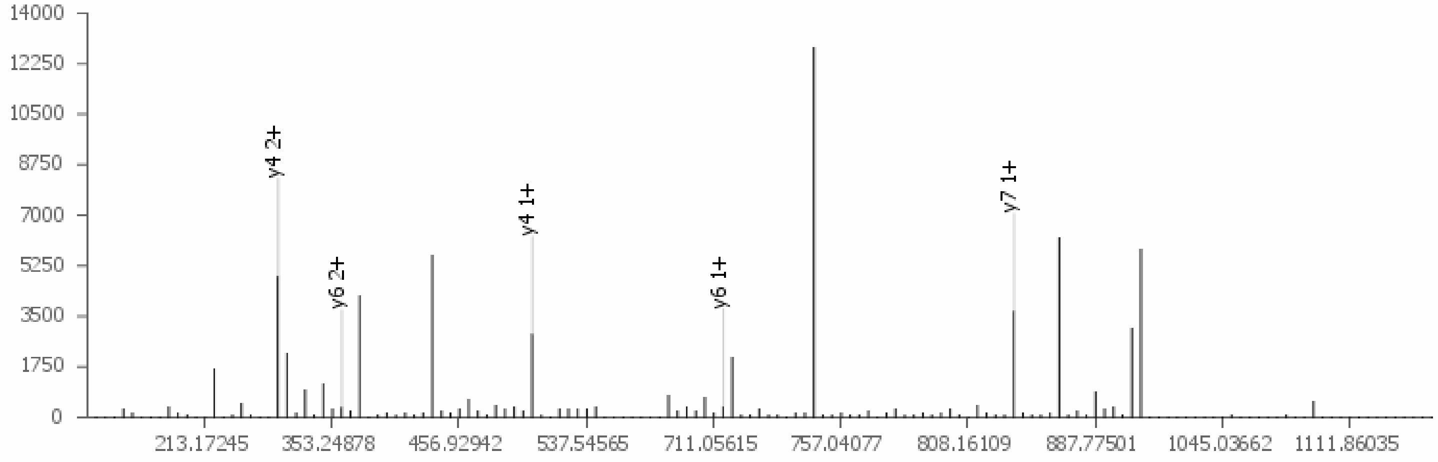

AT2G40840.1 - EL(s)LH(s)IG(pS)K - 615.760401 - Charge:2

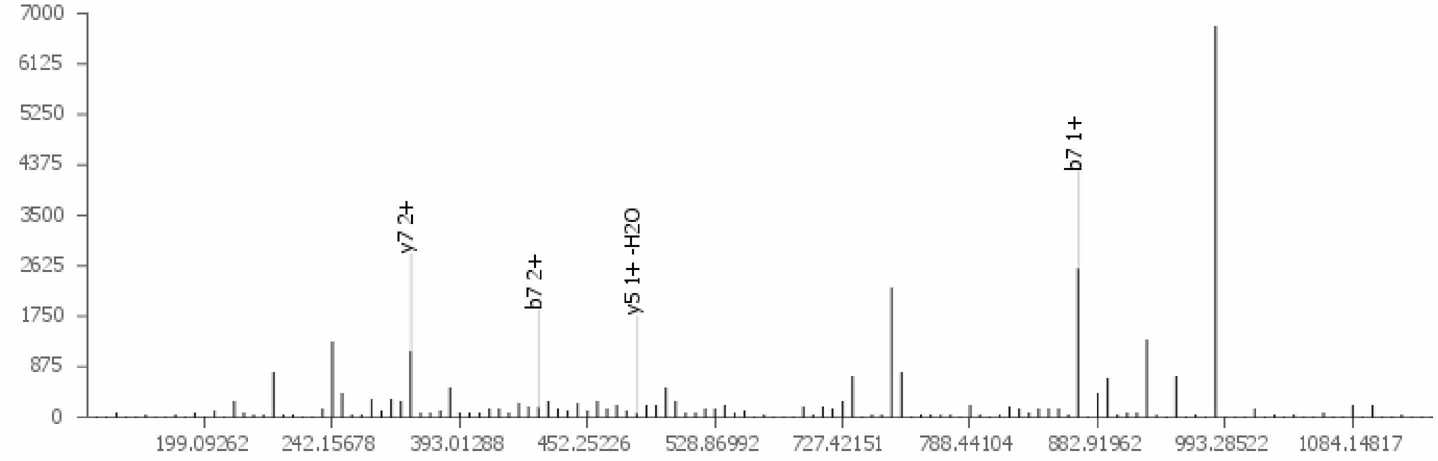

AT1G09000.1 - GPLGG(s)P(s)RATDAT(s)C(s)K - 954.888449 - Charge:2

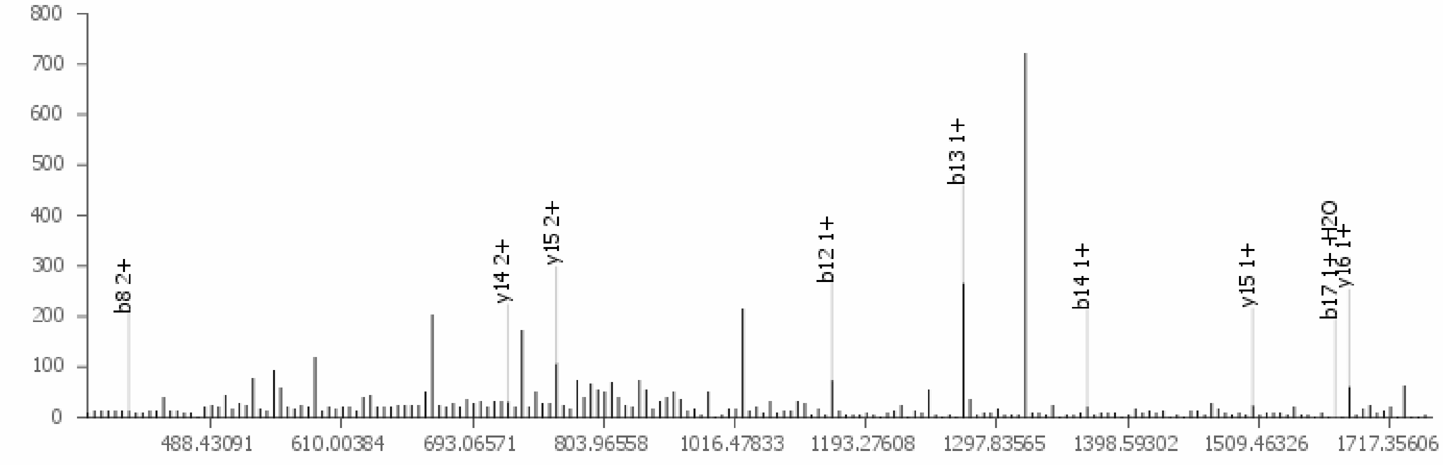

AT1G70610.1 - TDI(s)(s)NIK - 479.222599 - Charge:2

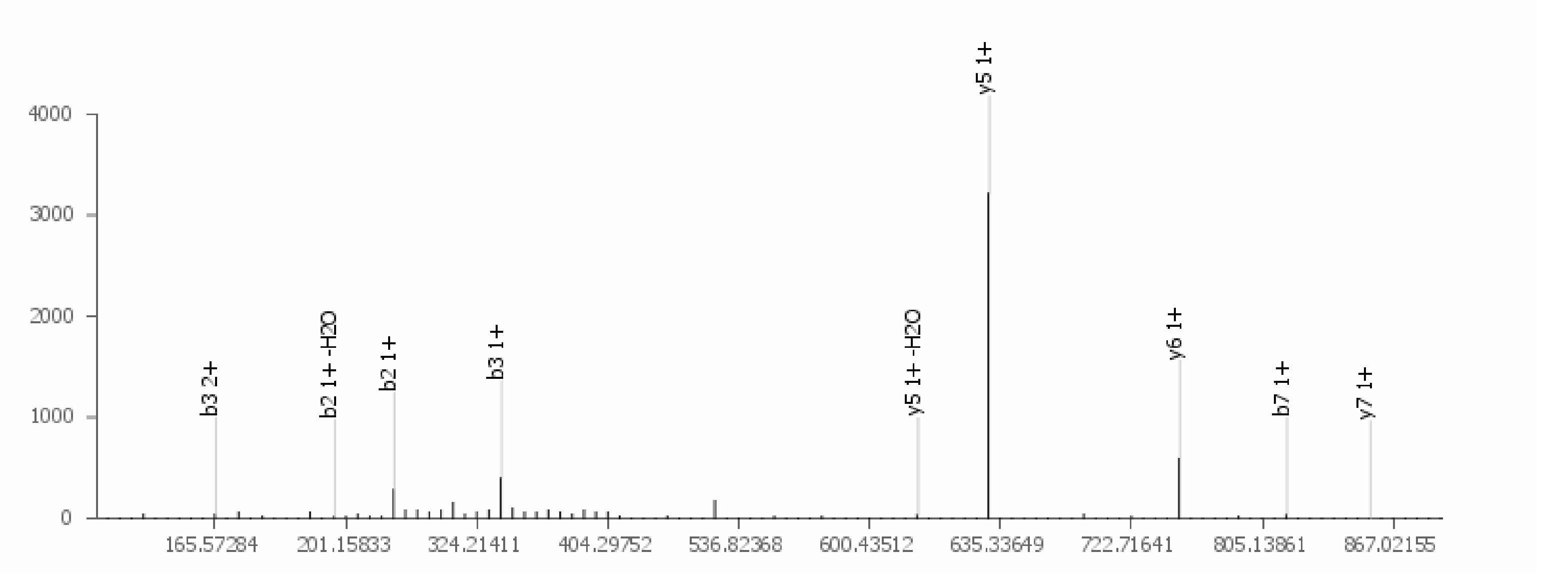

AT4G11310.1 - (pT)(pS)ADDVLPK - 553.212674 - Charge:2

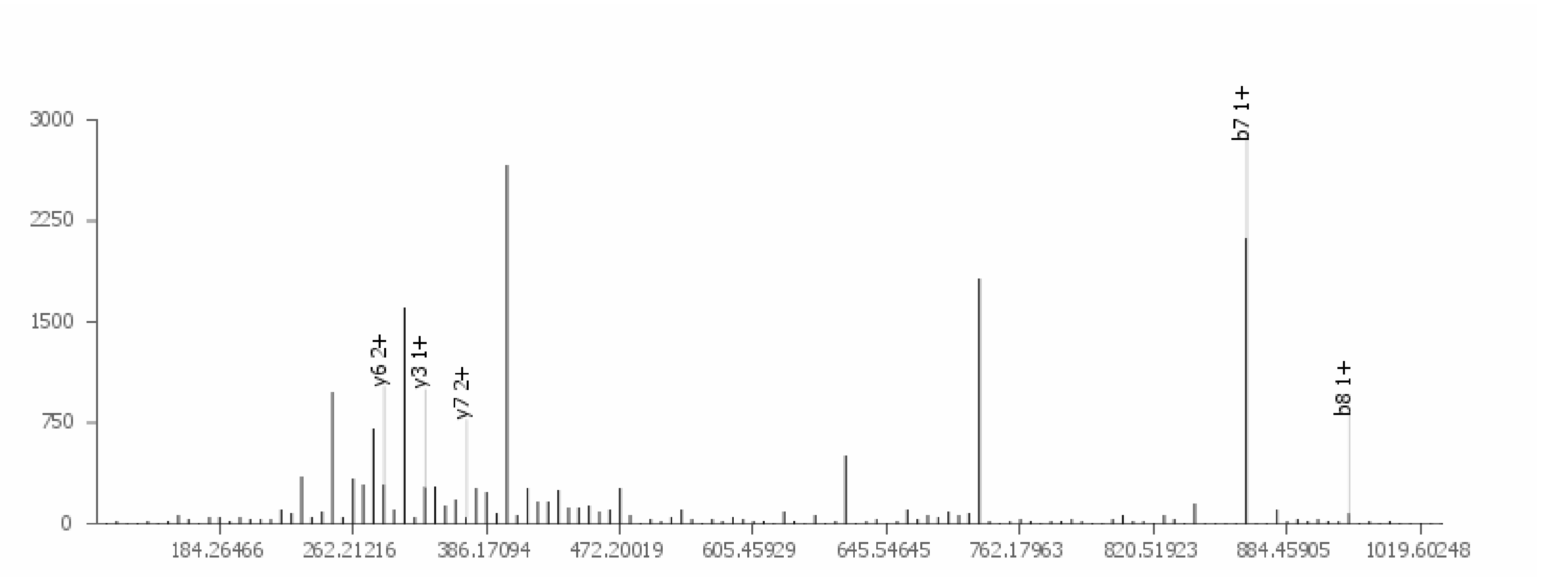

AT3G29680.1 - D(pS)FF(s)(s)ILPK - 650.769614 - Charge:2

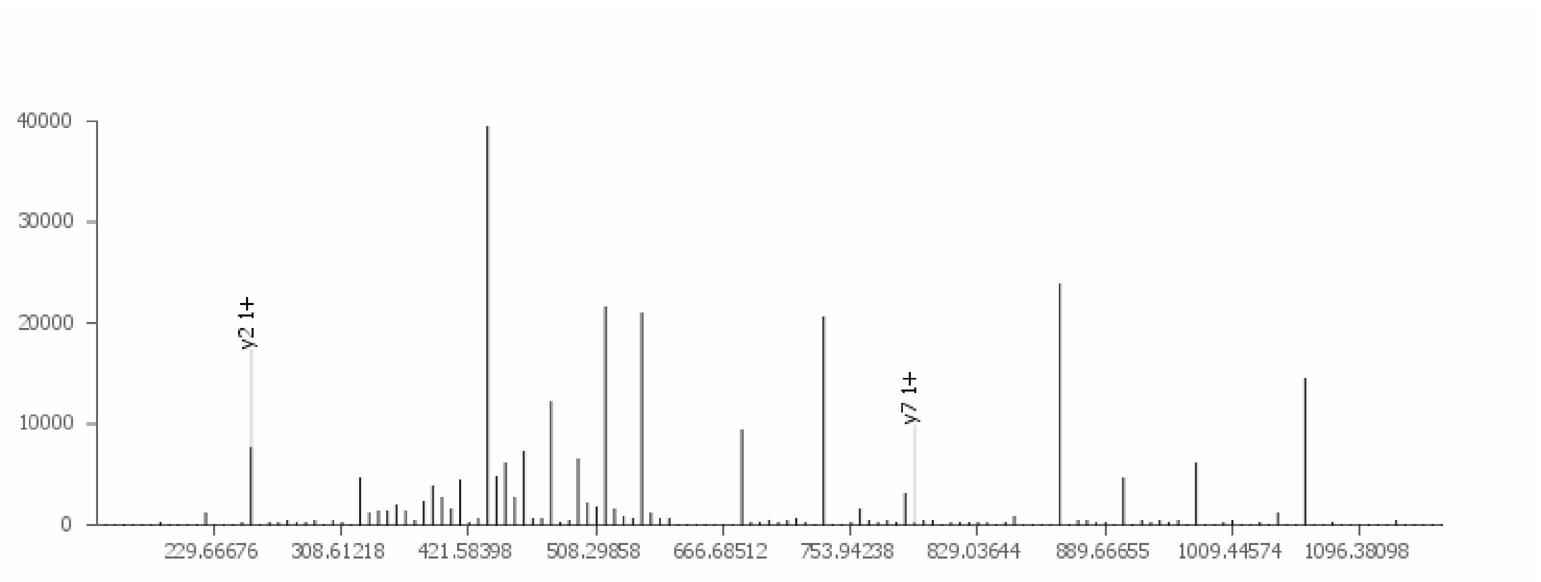

AT1G14970.1 - (pS)T(pY)IFLAAGPI(pY)(pS)ANR - 1032.382788 - Charge:2

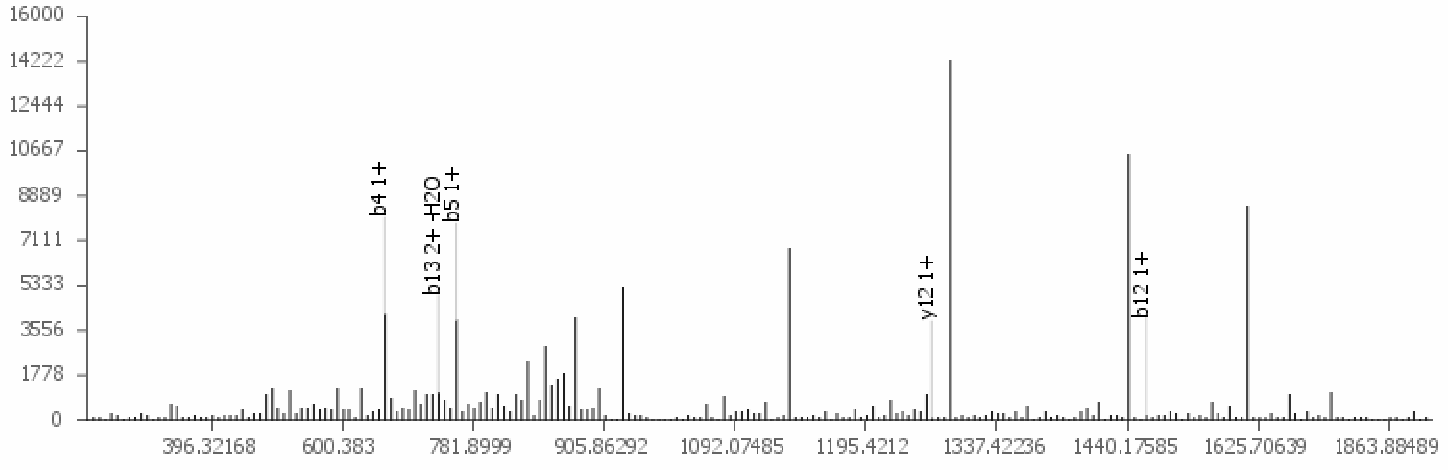

AT2G48120.1 - IEEDA(t)(t)G(s)K - 565.739069 - Charge:2

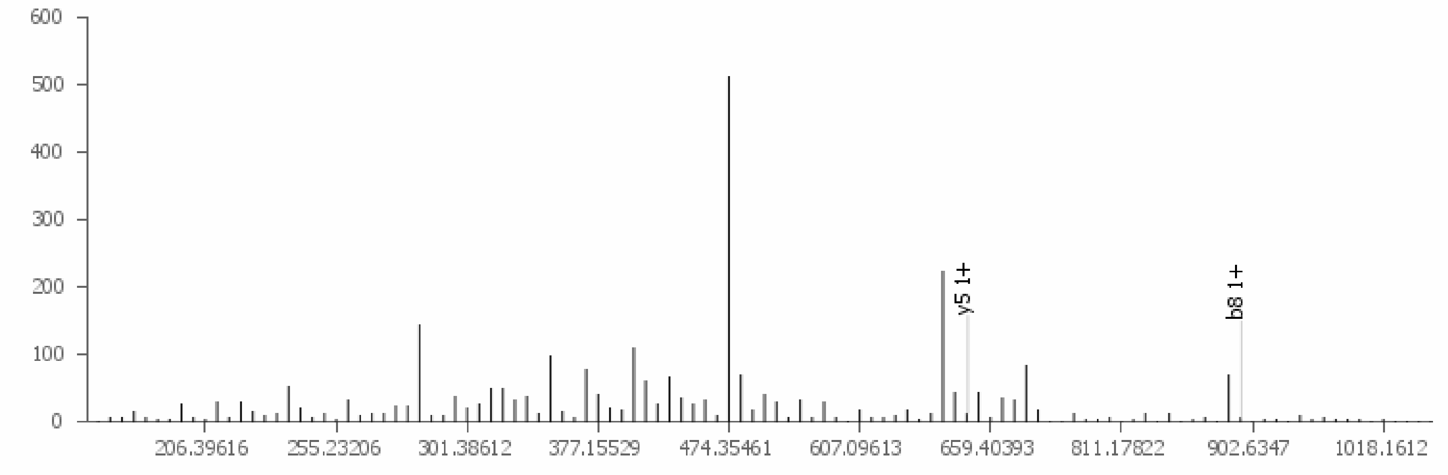

AT4G05091.1 - MI(pS)(pT)(pS)IP(pT)VATTELSSLGKR - 1206.514071 - Charge:2

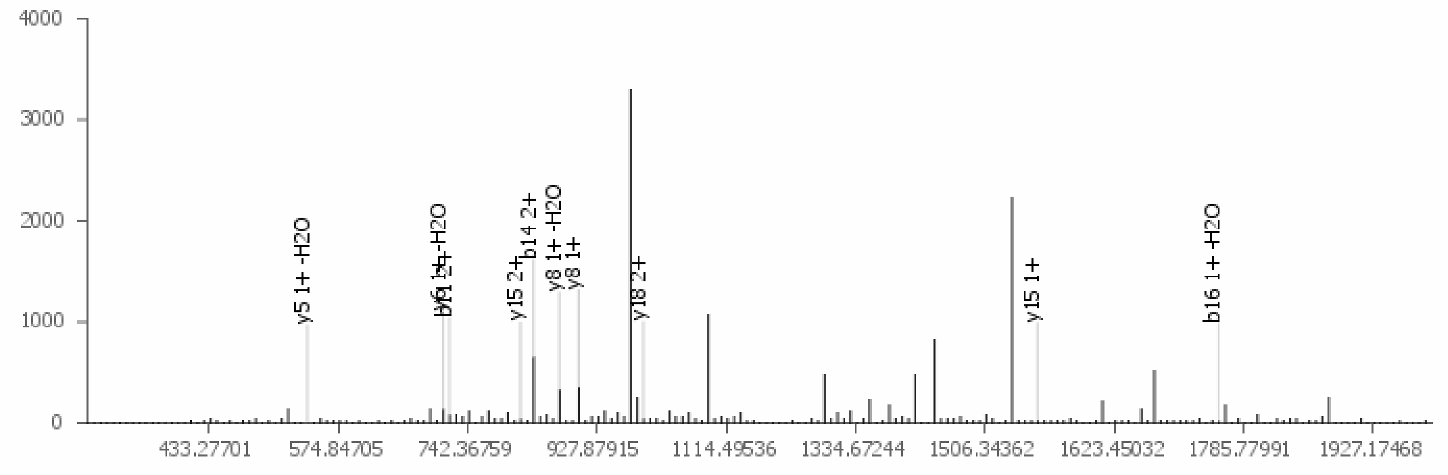

AT3G06930.1 - HVYAVEA(pS)E(oxM)AEYARK - 975.42231 - Charge:2

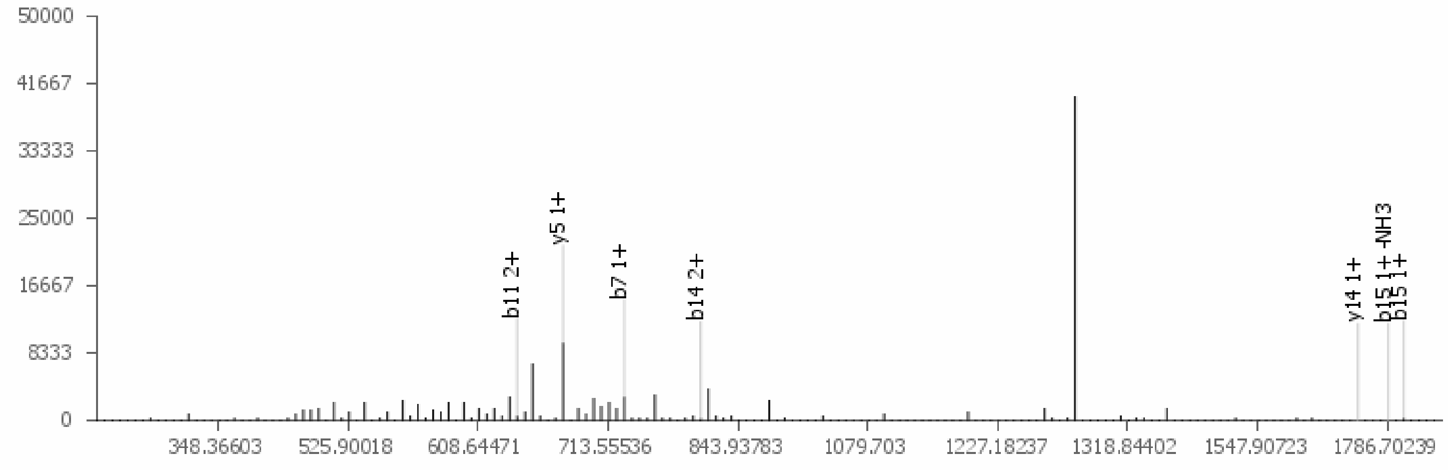

AT2G43040.1 - VP(pT)SDFER - 515.716017 - Charge:2

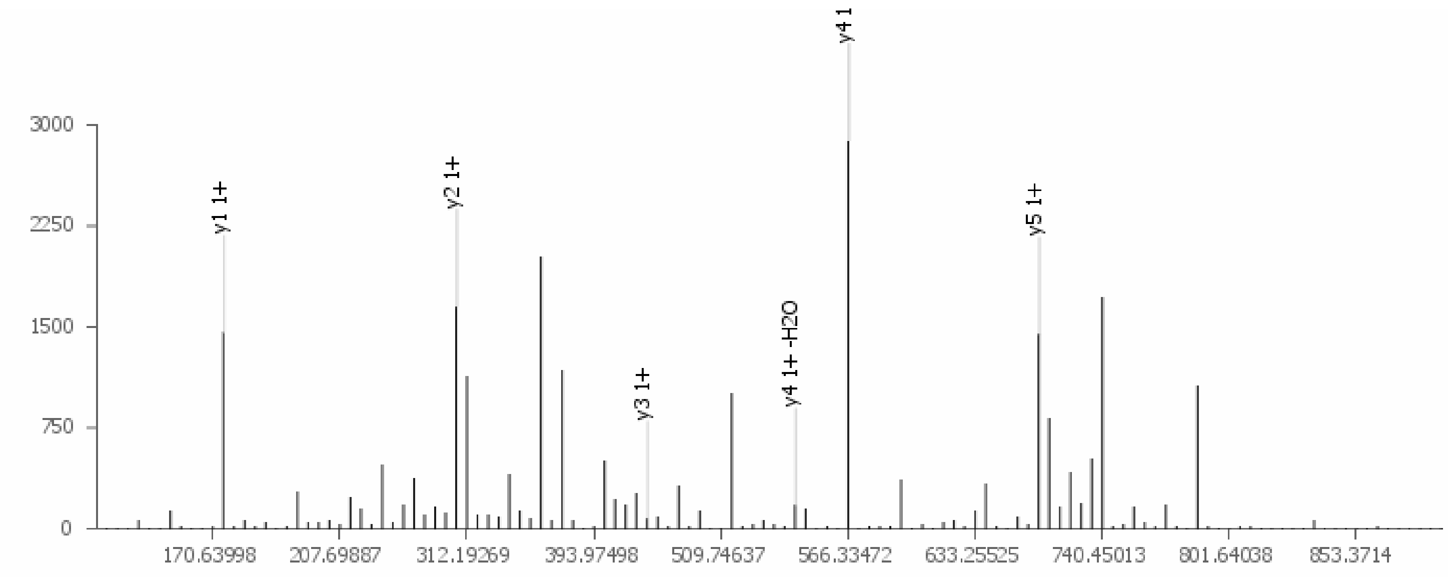

AT5G38710.1 - RV(pS)SVYE(pS)(pT)GLK - 783.300743 - Charge:2

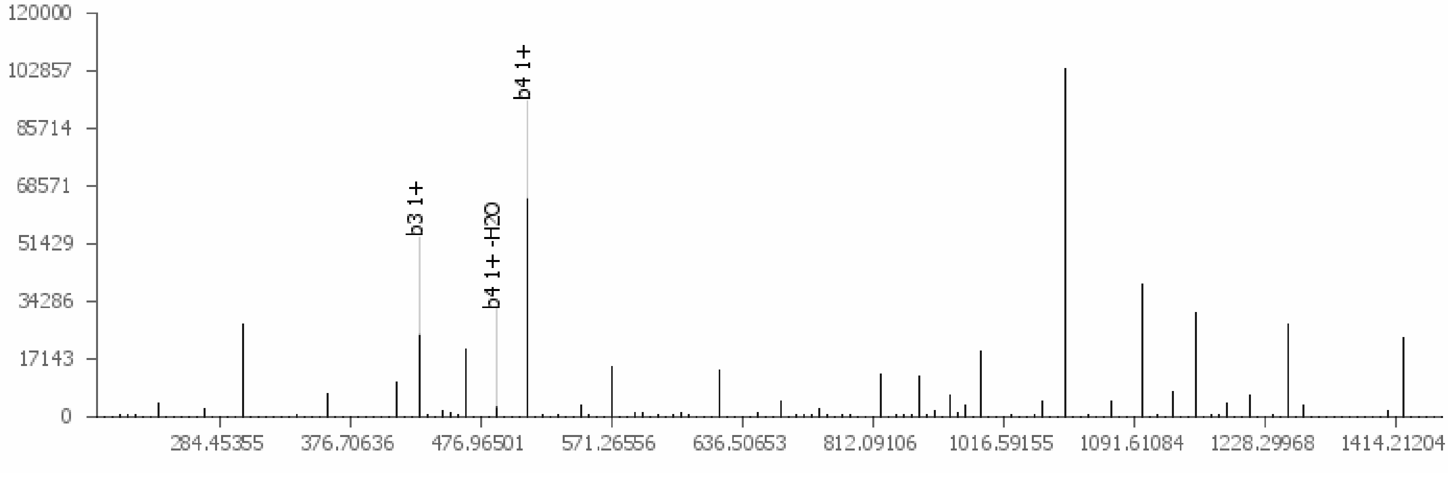

AT1G06690.1 - AVGVSNY(pS)EK - 567.245757 - Charge:2

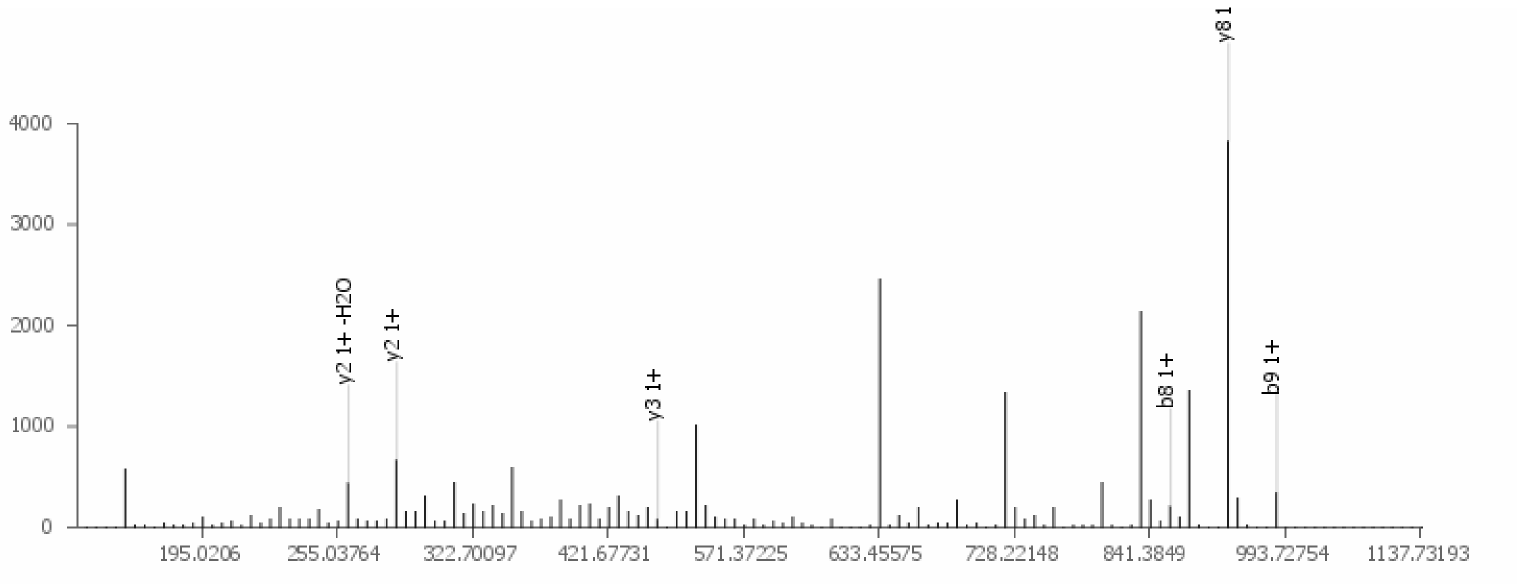

AT2G26410.1 - SKVNVG(pT)(t)(s)(oxM)PK - 712.805233 - Charge:2

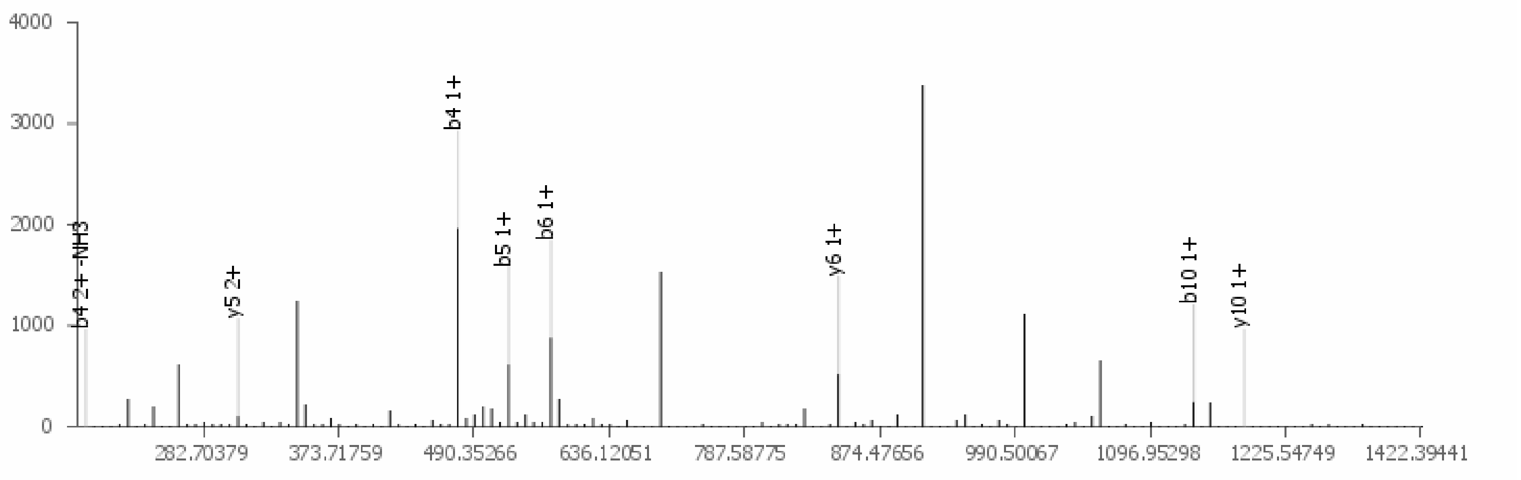

AT1G66330.1 - SSN(pS)SQLLVEYVSNDAK - 640.958401 - Charge:3

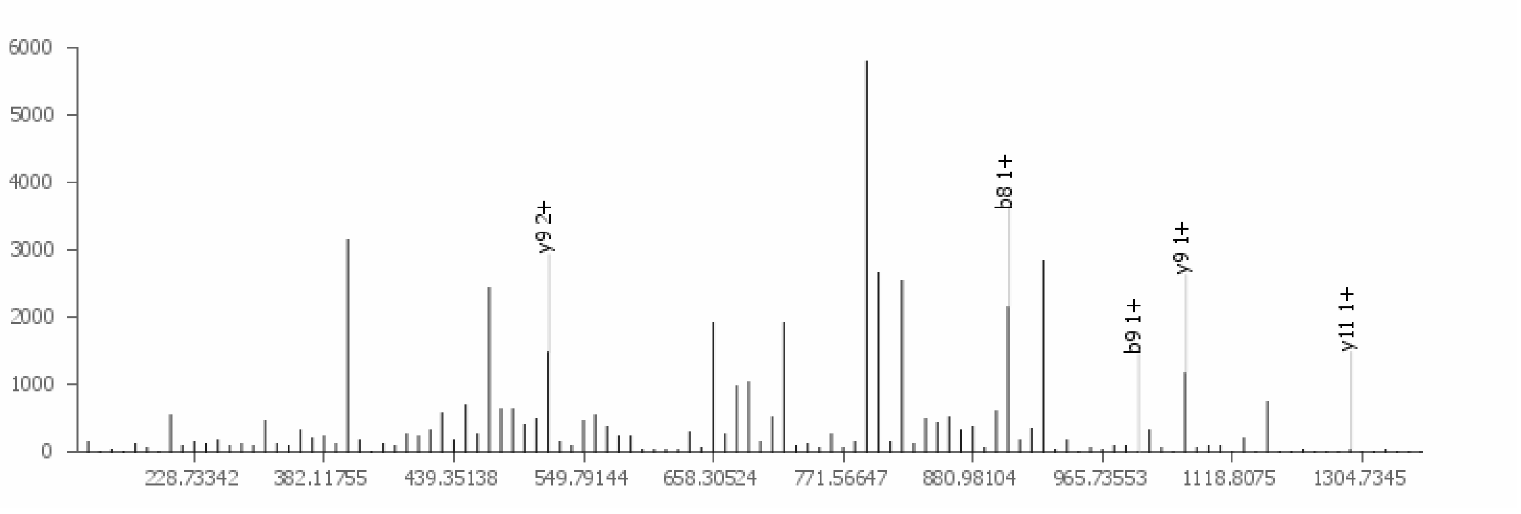

AT1G02630.1 - SA(pT)WACIVR - 572.252421 - Charge:2

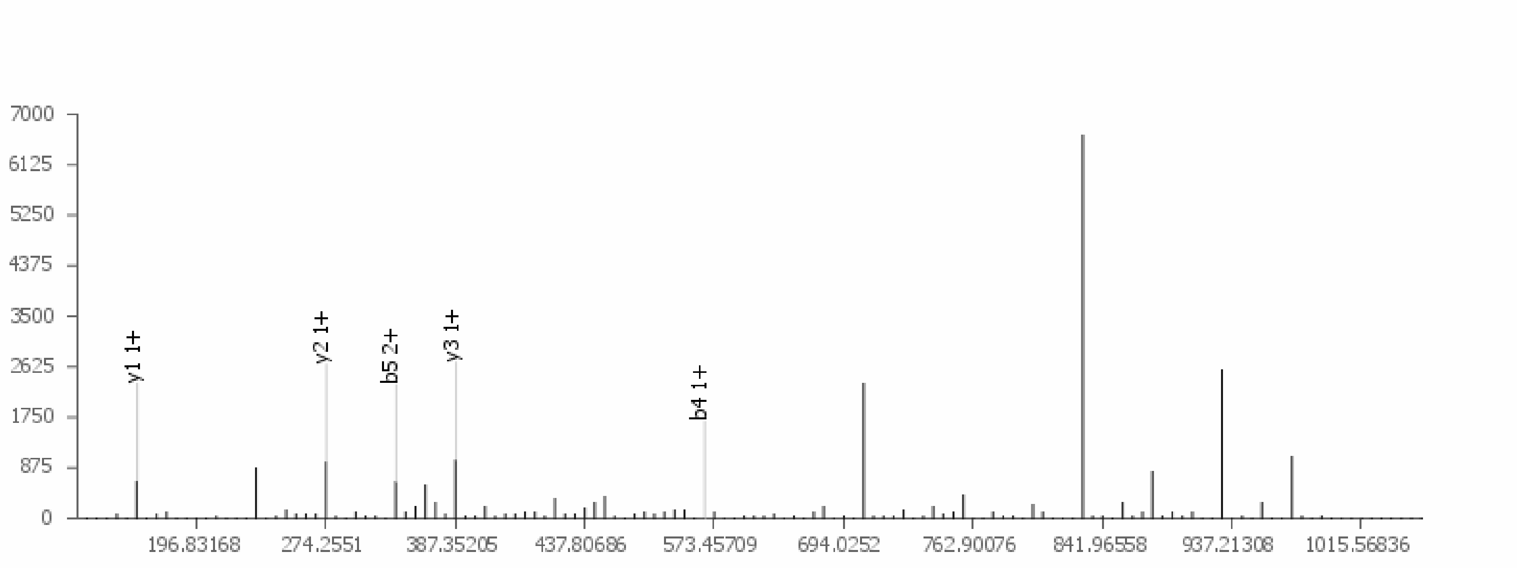

AT1G11720.1 - MEATDDE(pS)(pS)HVK(pT)TAK - 995.356244 - Charge:2

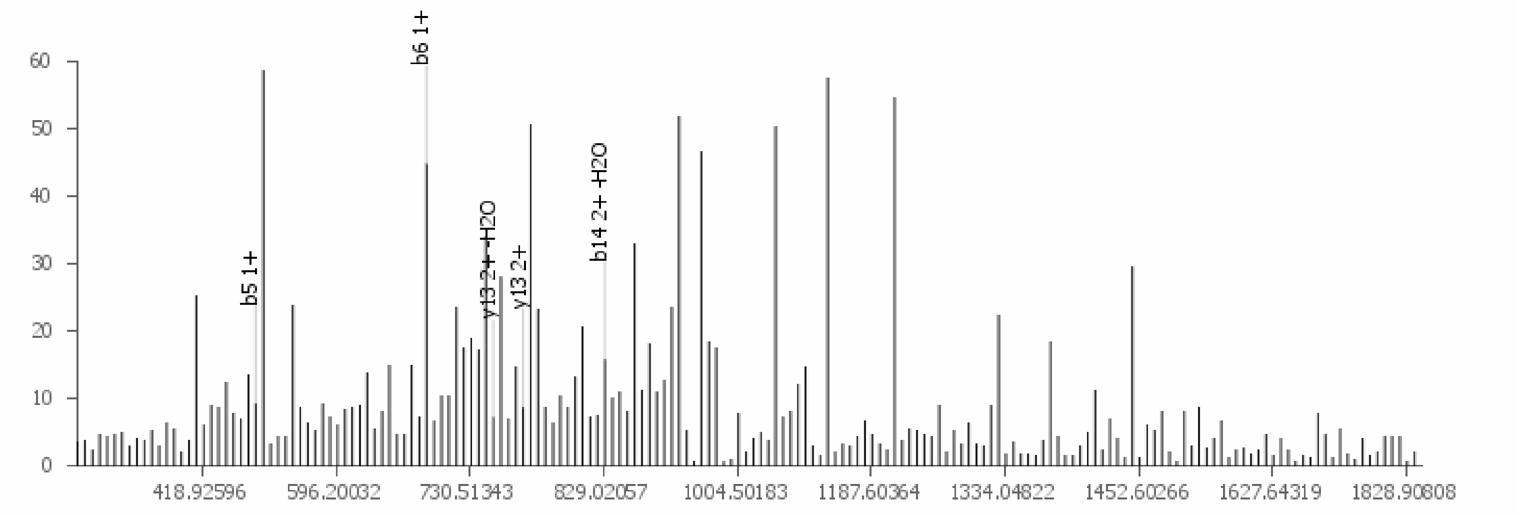

AT5G13260.1 - (pS)SSVTN(oxM)SNVK - 625.25687 - Charge:2

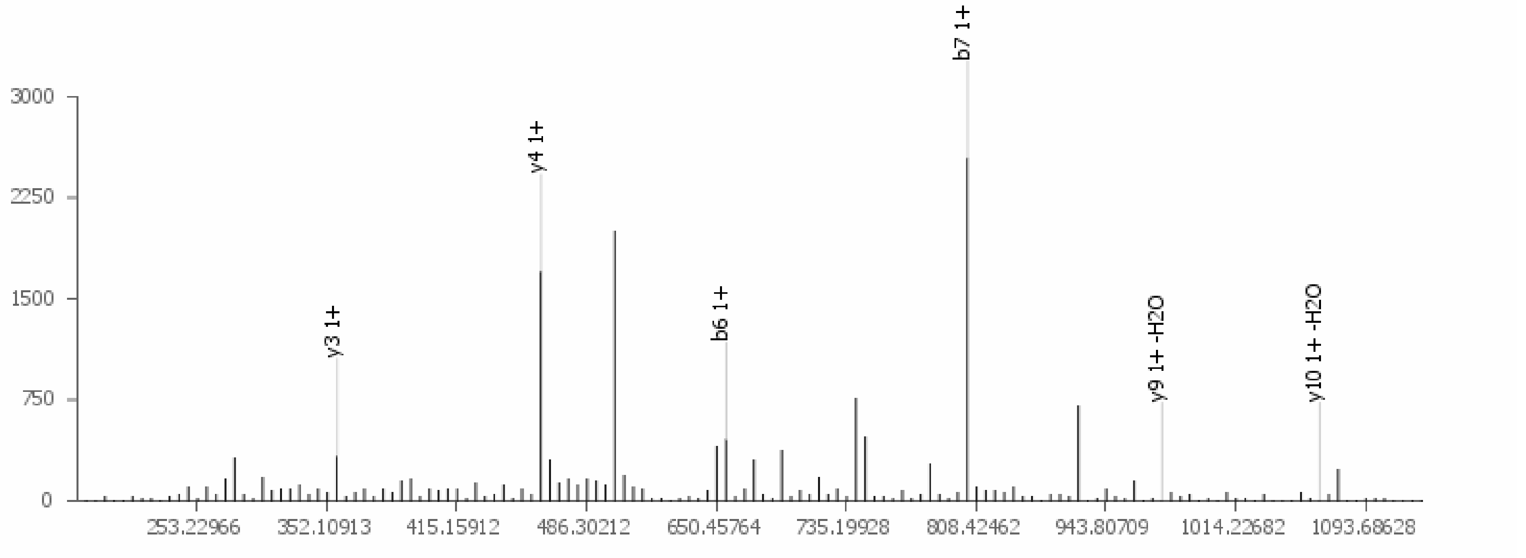

AT2G01280.1 - NIIS(pS)(oxM)K - 444.69869 - Charge:2

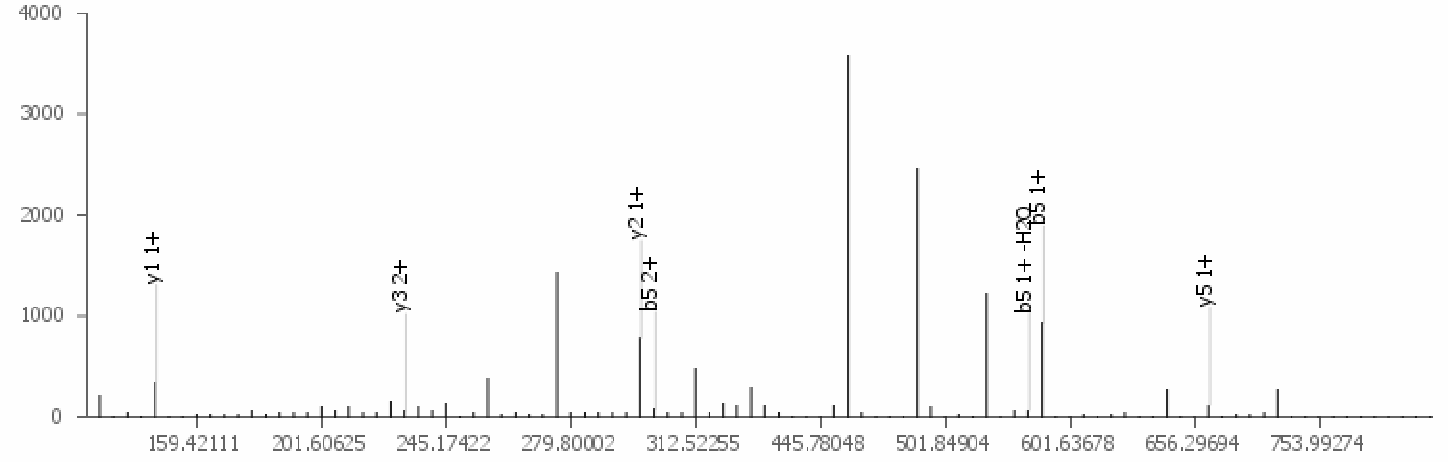

AT1G79850.1 - I(pT)(pS)(pS)LTSSLQALK - 794.832586 - Charge:2

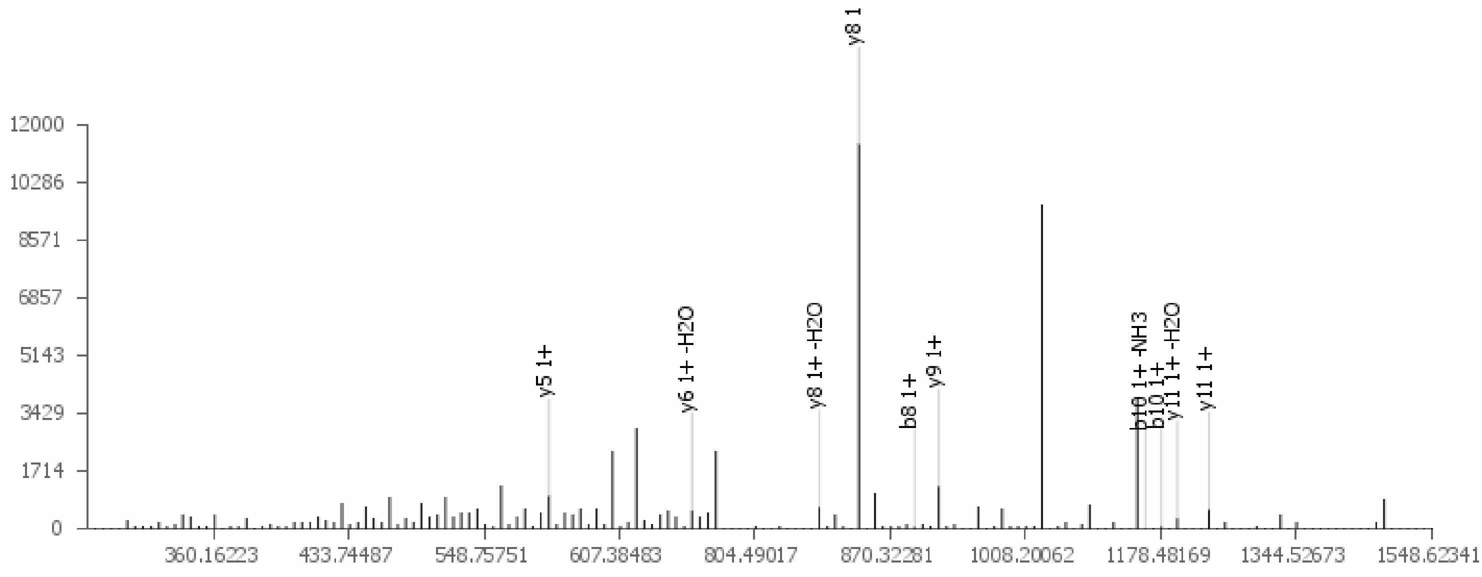

AT1G58370.1 - (pT)CVFKEK - 496.219833 - Charge:2

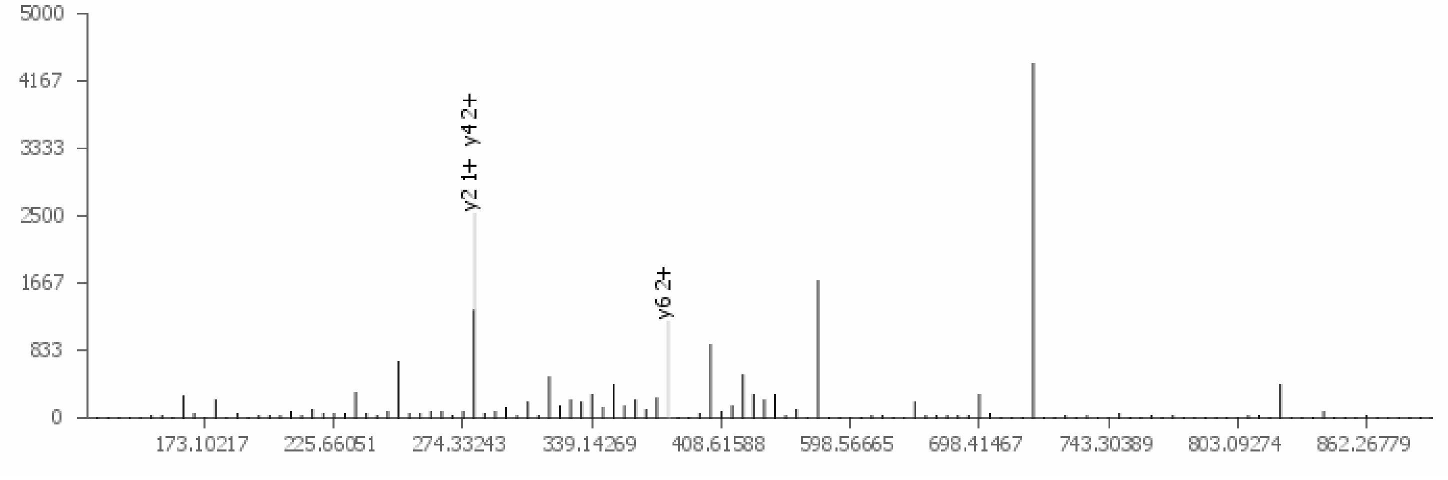

AT2G38760.1 - N(pY)GVTIDK - 495.218202 - Charge:2

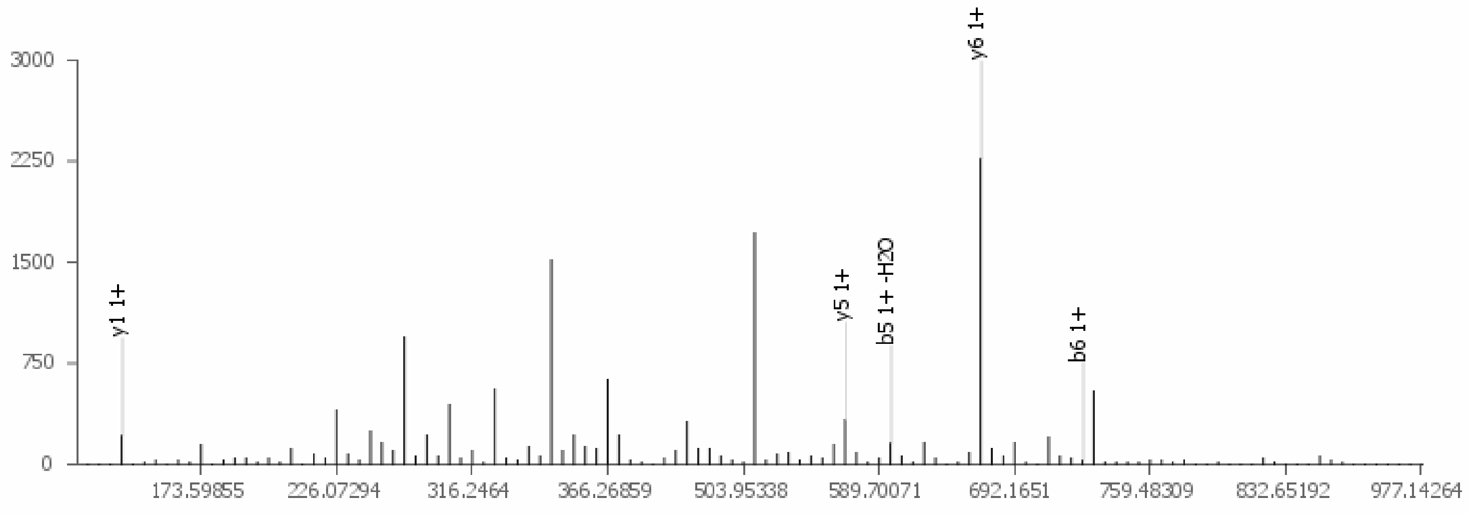

AT5G02530.1 - (oxM)(pS)GGLD(oxM)(pS)LDDIIK - 843.817104 - Charge:2

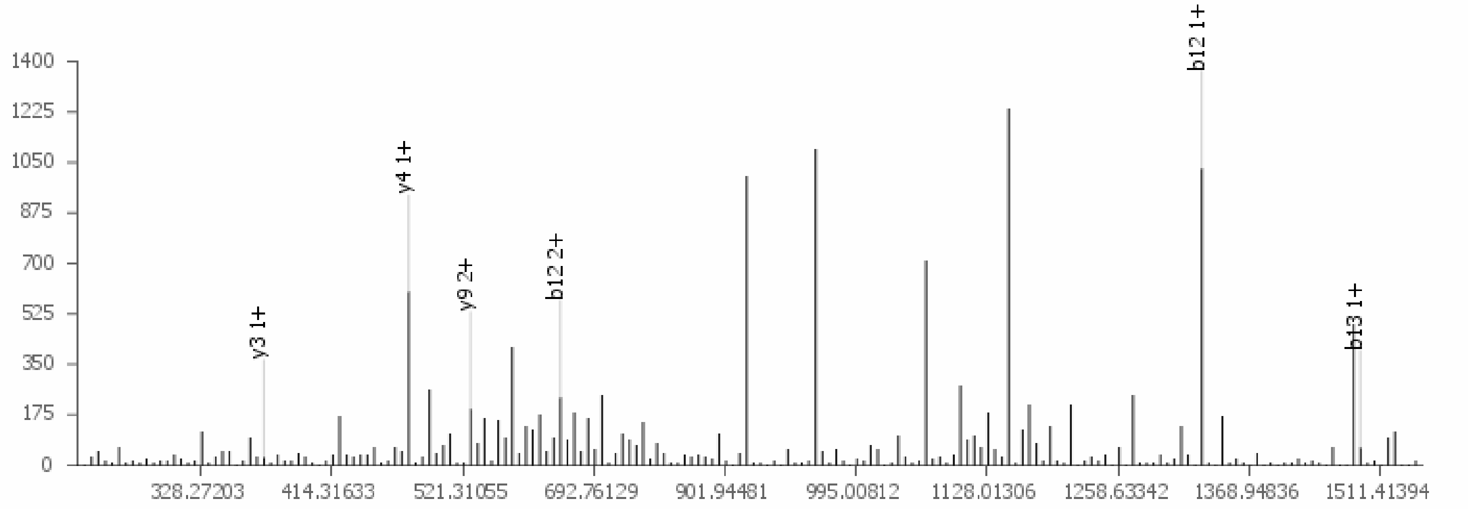

AT3G50370.1 - MVDRI(t)(t)SSTLDL(pS)VP(oxM)R - 1099.485004 - Charge:2

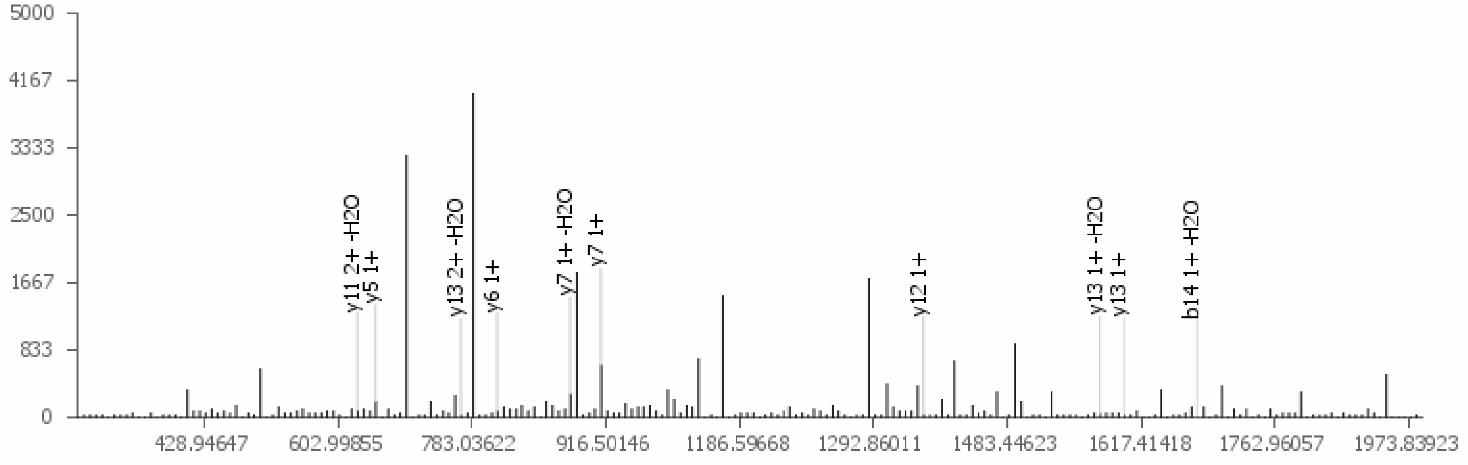

AT5G48960.1 - LDDGFI(pS)ADLGTLDYKGLYK - 762.029733 - Charge:3

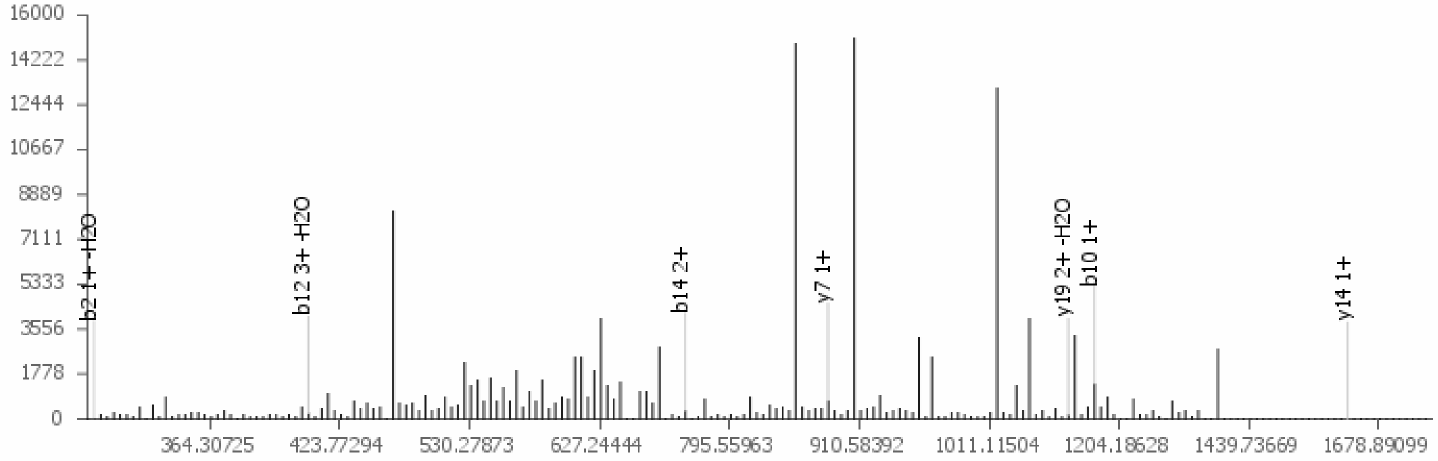

AT1G14700.1 - MT(pY)IYRD(pT)K - 675.757439 - Charge:2

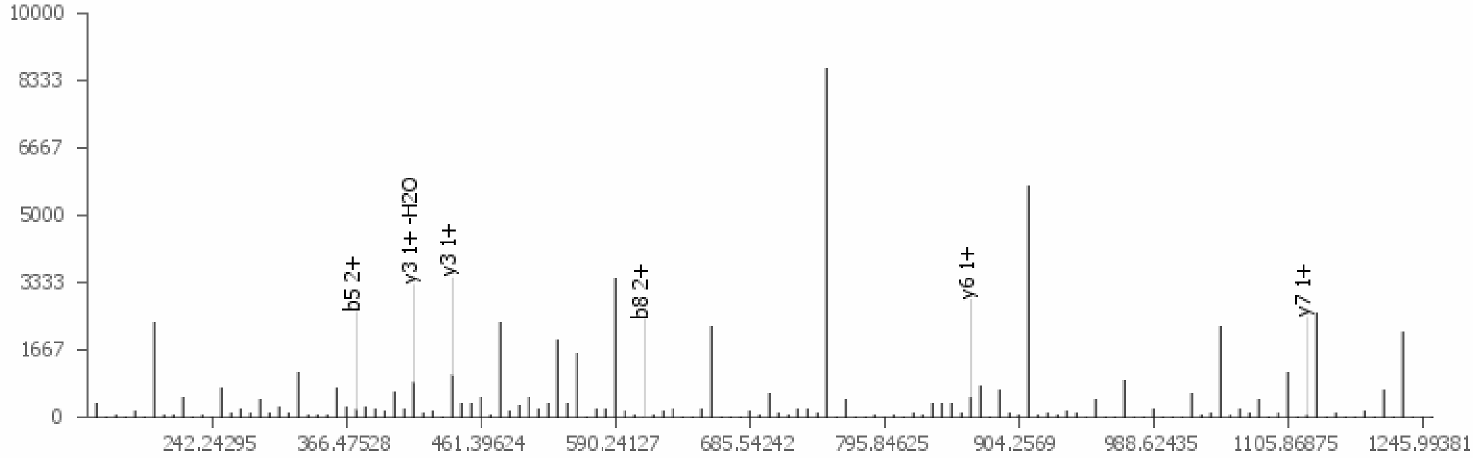

AT3G56410.2 - LA(pS)(pS)KDLK - 511.217137 - Charge:2

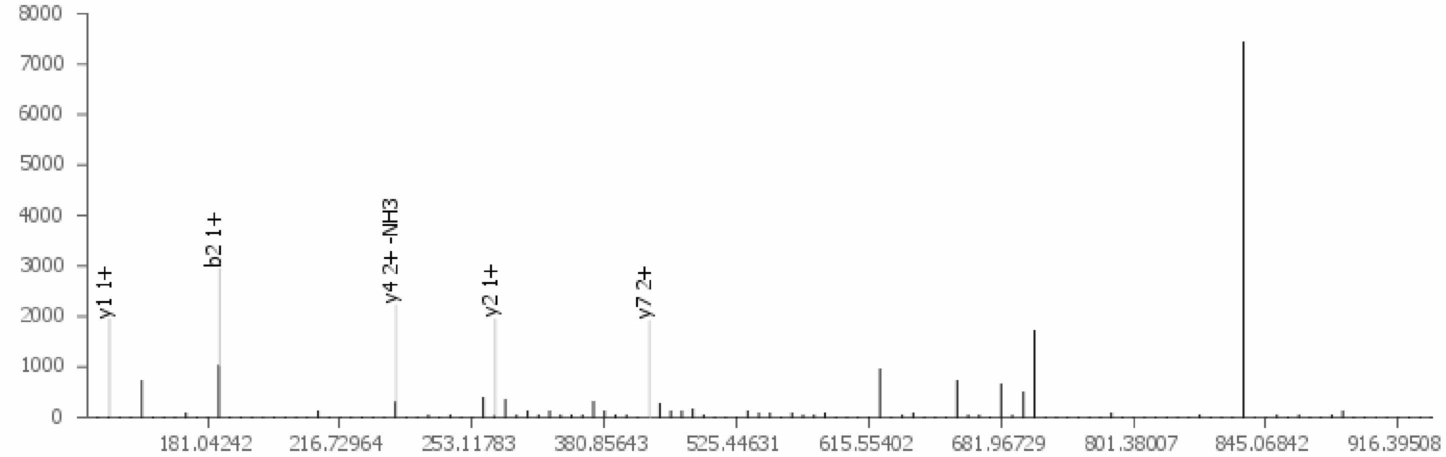

AT5G22550.1 - IEY(pT)NLK - 480.722828 - Charge:2

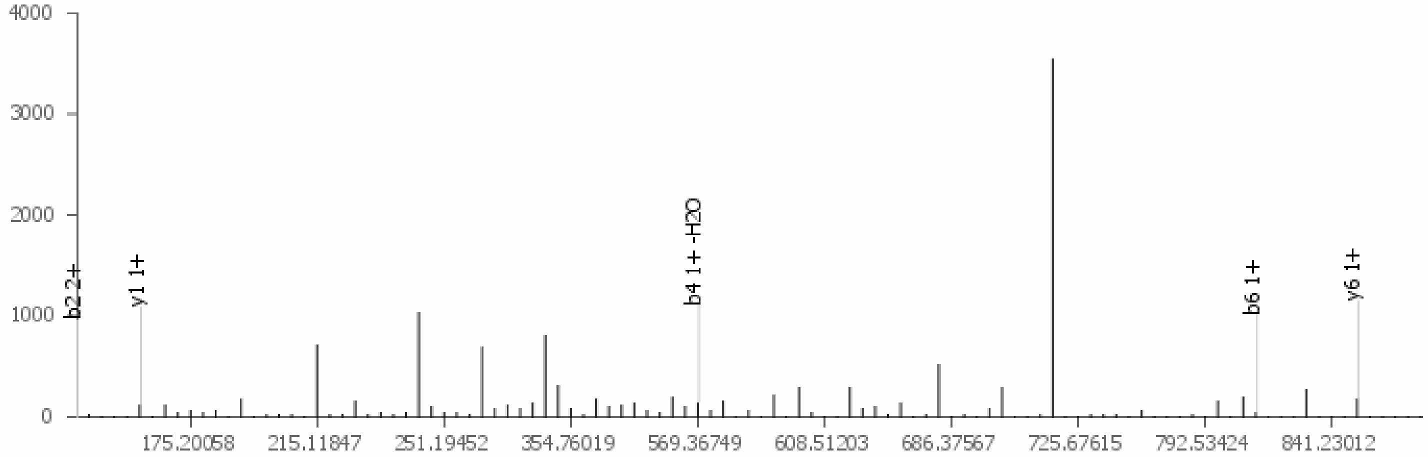

AT5G18910.1 - GKQLTP(pS)(pT)R - 574.253522 - Charge:2

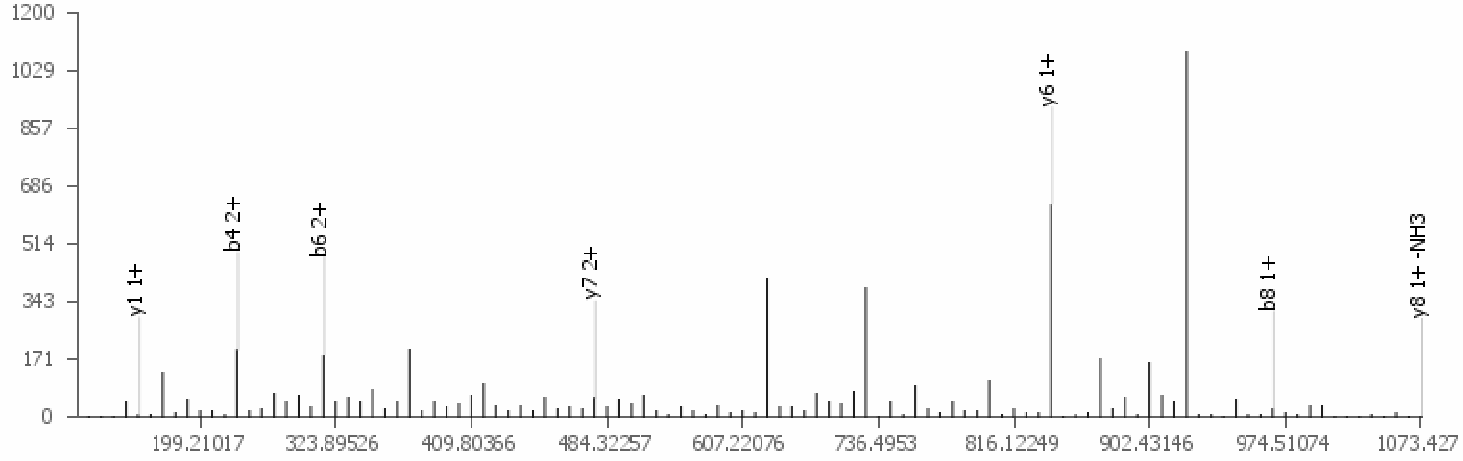

AT5G07660.1 - RI(pS)D(pS)(pT)SLTVLK - 780.332556 - Charge:2

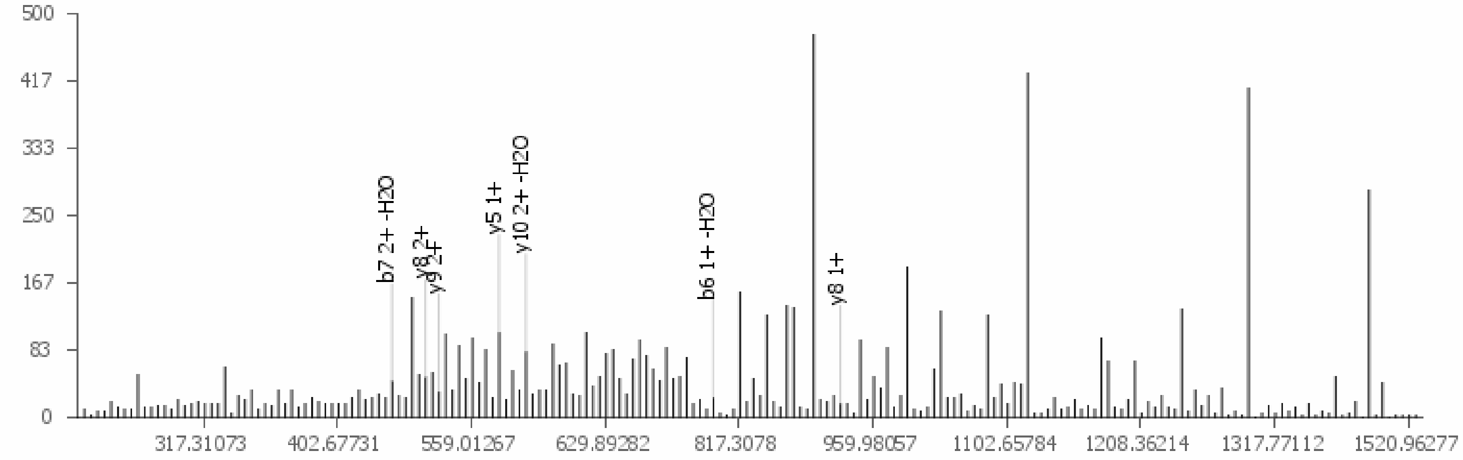

AT4G31210.1 - AEL(pS)(pT)AASPASNGNQATTVKSK - 1147.004235 - Charge:2

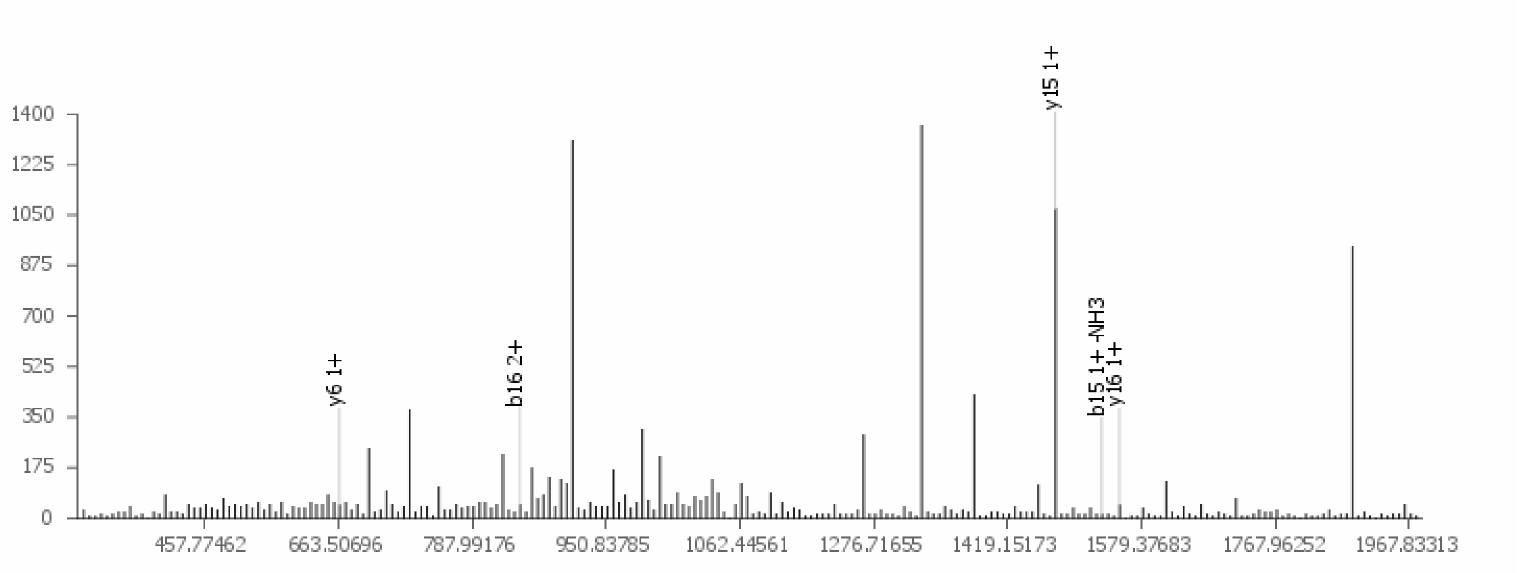

AT3G20410.1 - LESNENL(pY)K - 595.265053 - Charge:2

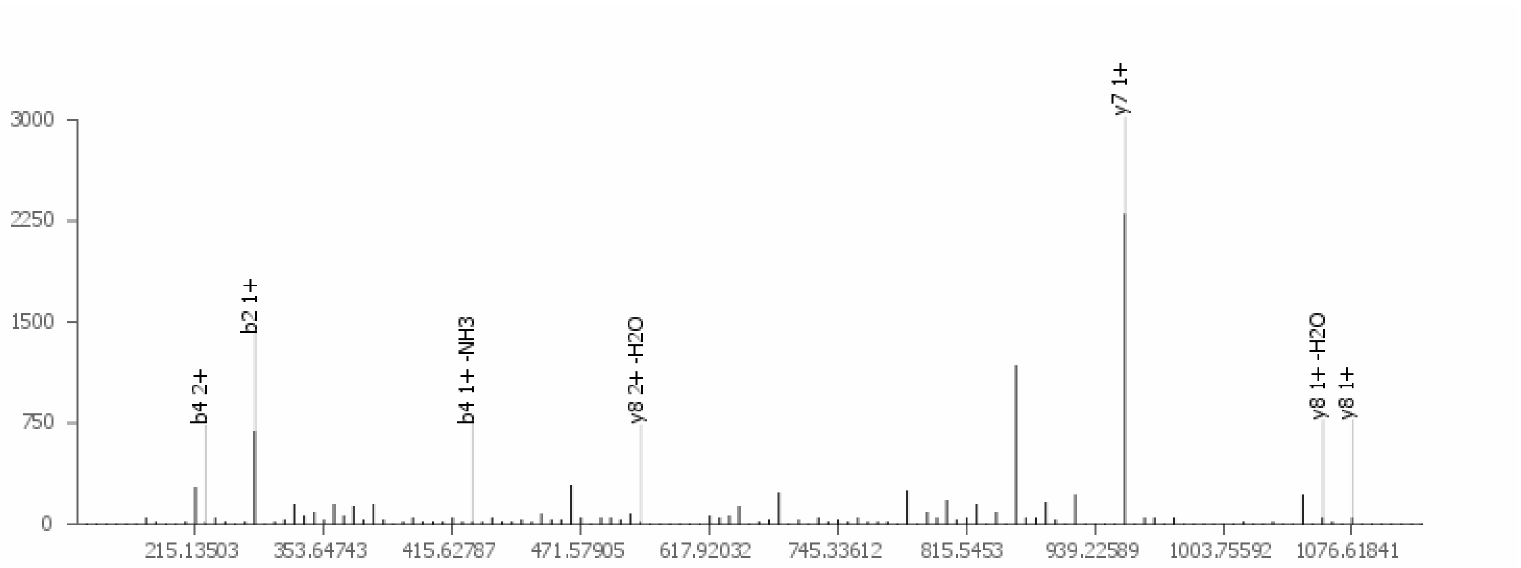

AT1G28090.1 - FNKL(pS)(pS)MAYGSFGVSSK - 657.273706 - Charge:3

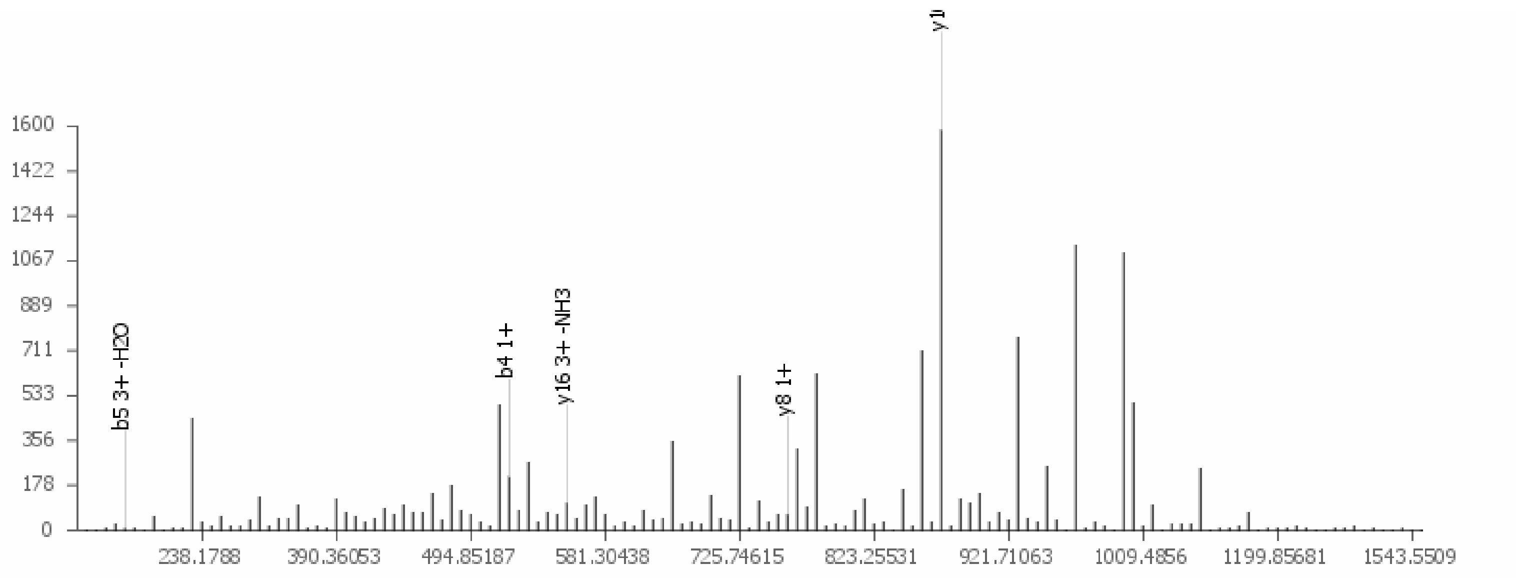

AT5G06120.1 - LDIRA(pY)IVN(pY)LA(pT)R - 960.933823 - Charge:2

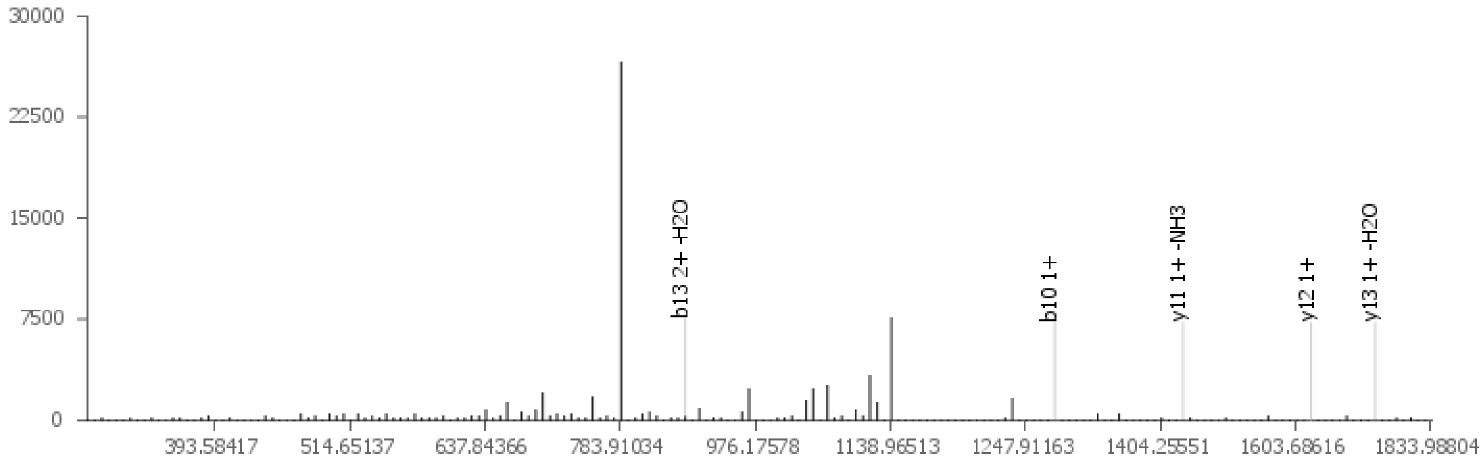

AT3G46450.1 - (s)RN(s)(oxM)MATVSSGK - 726.304318 - Charge:2

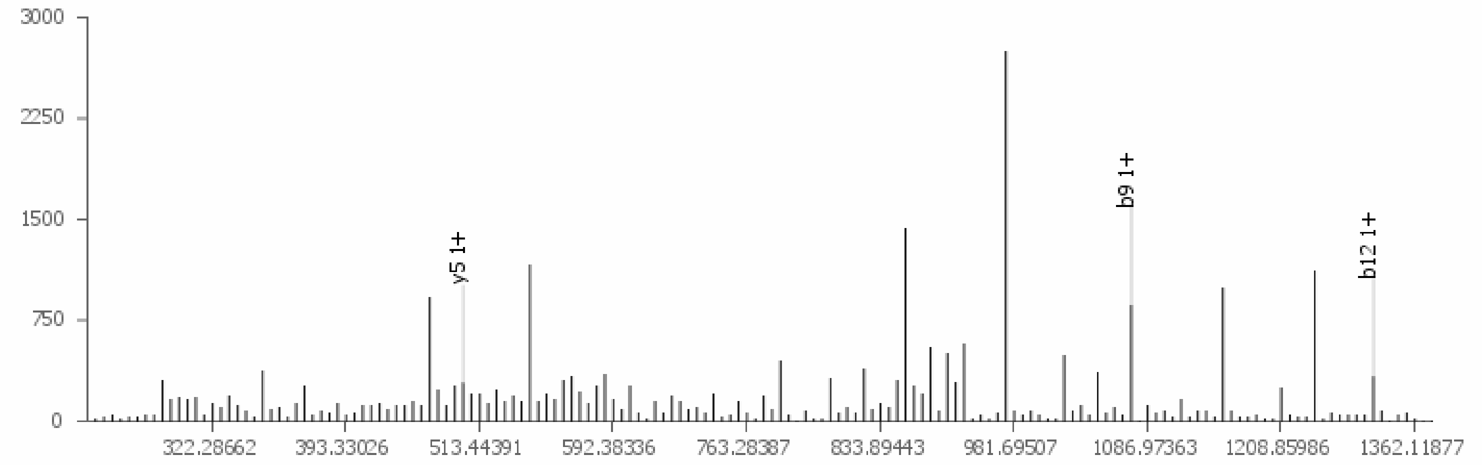

AT1G50250.1 - (oxM)ASN(pS)LLR - 494.216213 - Charge:2

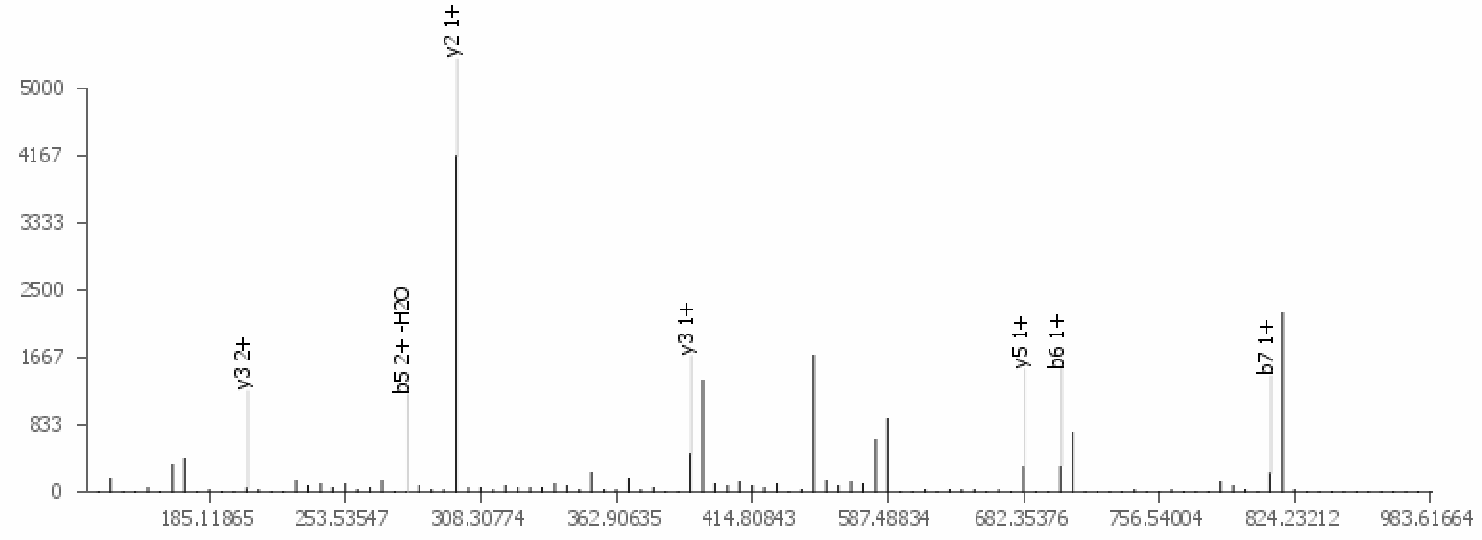

AT1G61180.1 - (oxM)GSCF(pS)LQVSDQTLNR - 969.907733 - Charge:2

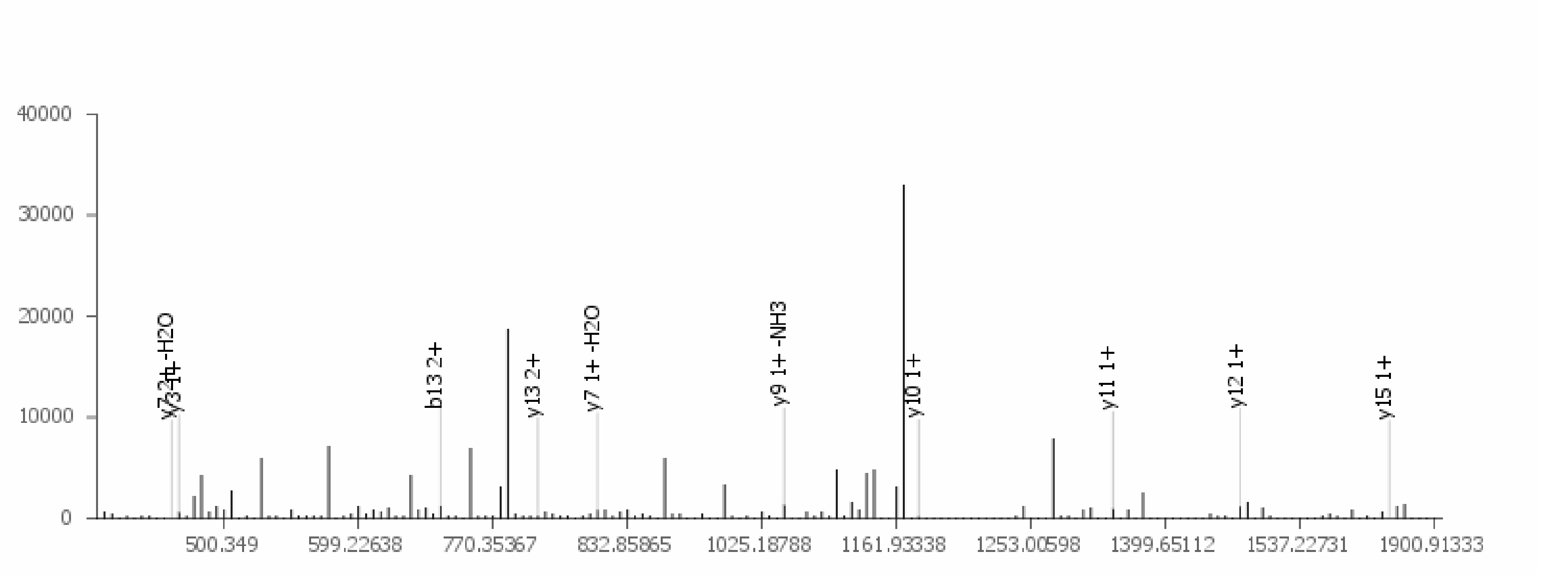

AT4G05190.1 - (oxM)QVSMLEI(pY)NE(pS)IRDLL(s)(t)SR - 914.3841 - Charge:3

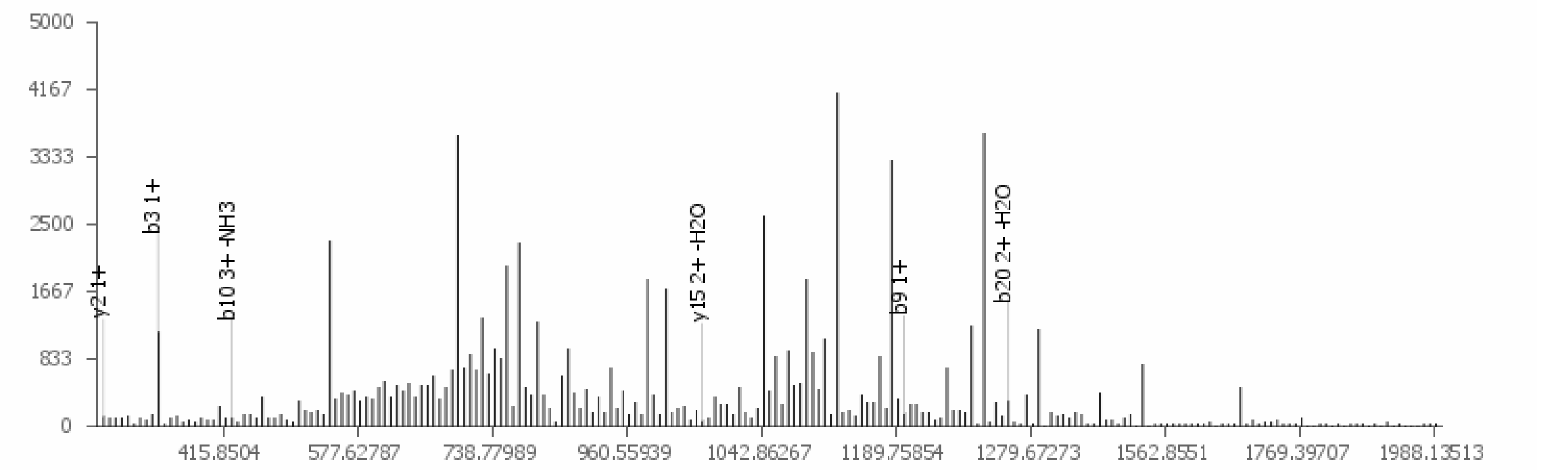

AT3G16010.1 - ADSVSP(pS)EFTYSILIDGYCK - 1166.499874 - Charge:2

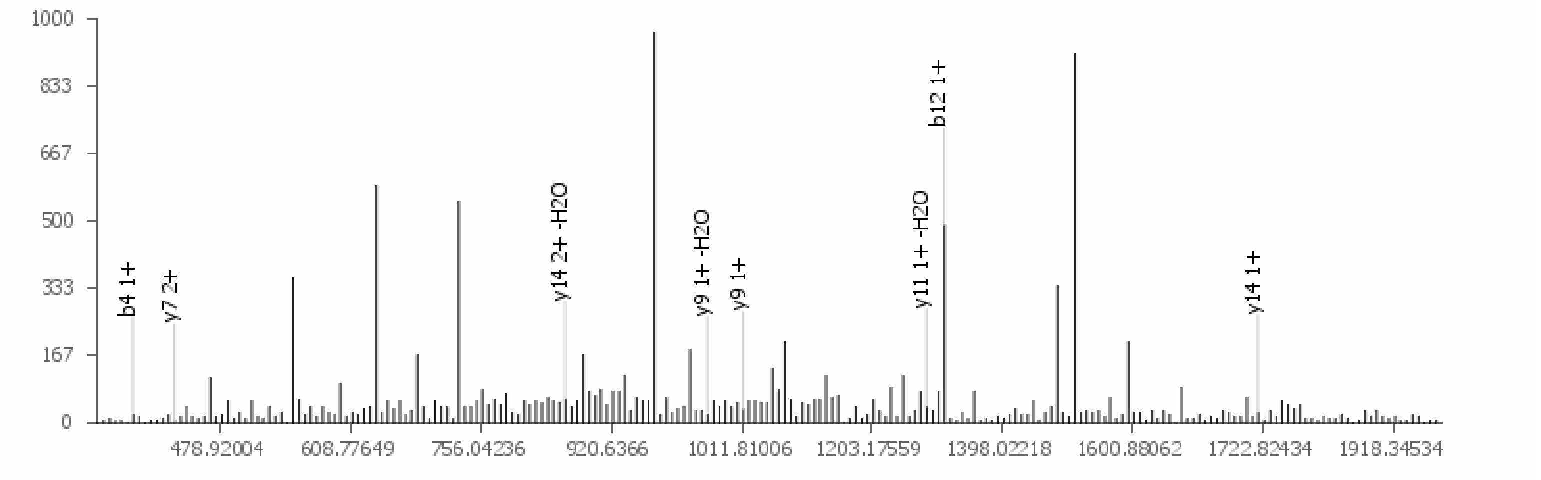

AT5G45940.1 - RS(t)(t)L(s)(s)HPGEVALPGGK - 977.451223 - Charge:2

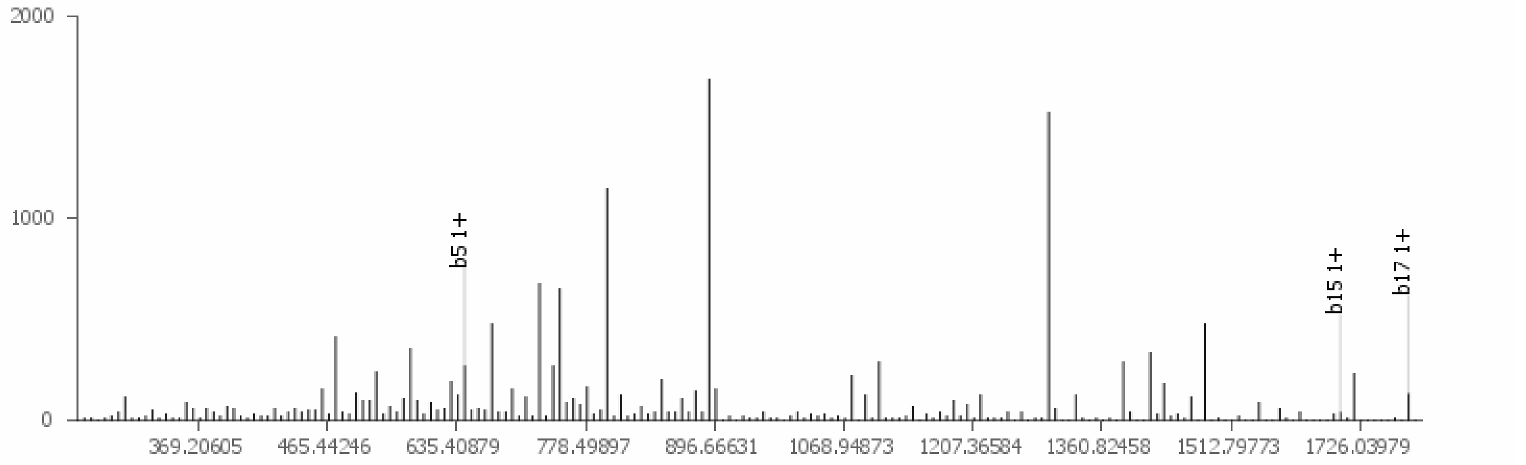

AT1G06900.1 - EY(pY)HGGLMK - 589.239839 - Charge:2

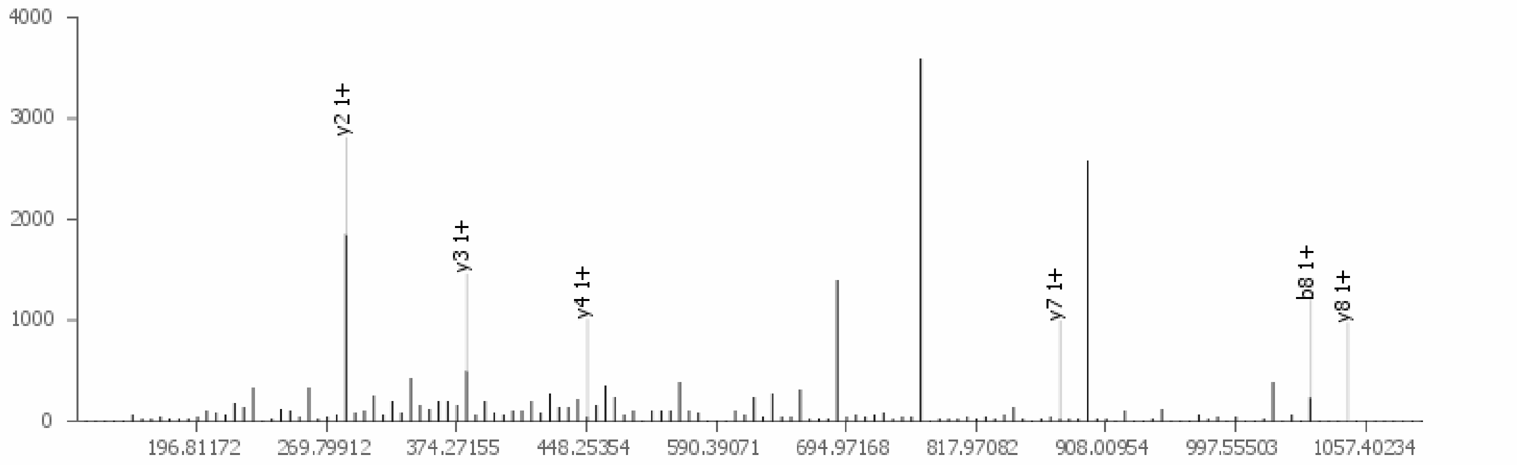

AT2G34060.1 - NFLGRL(pY)D(pY)K - 724.806289 - Charge:2

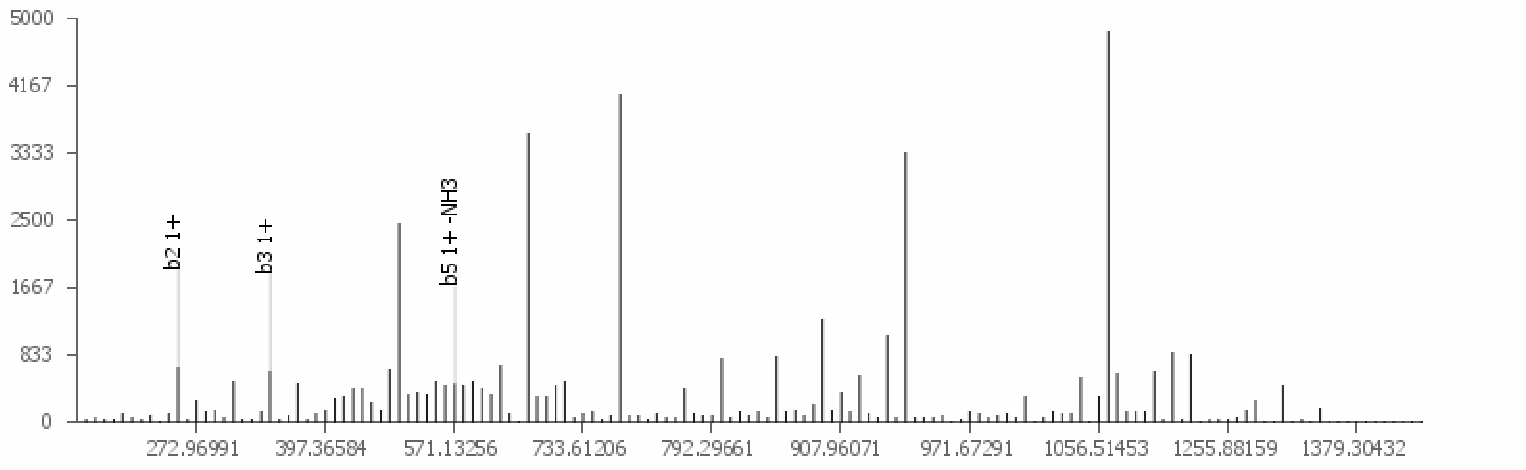

AT5G20200.1 - LIEMI(s)(s)R - 514.745677 - Charge:2

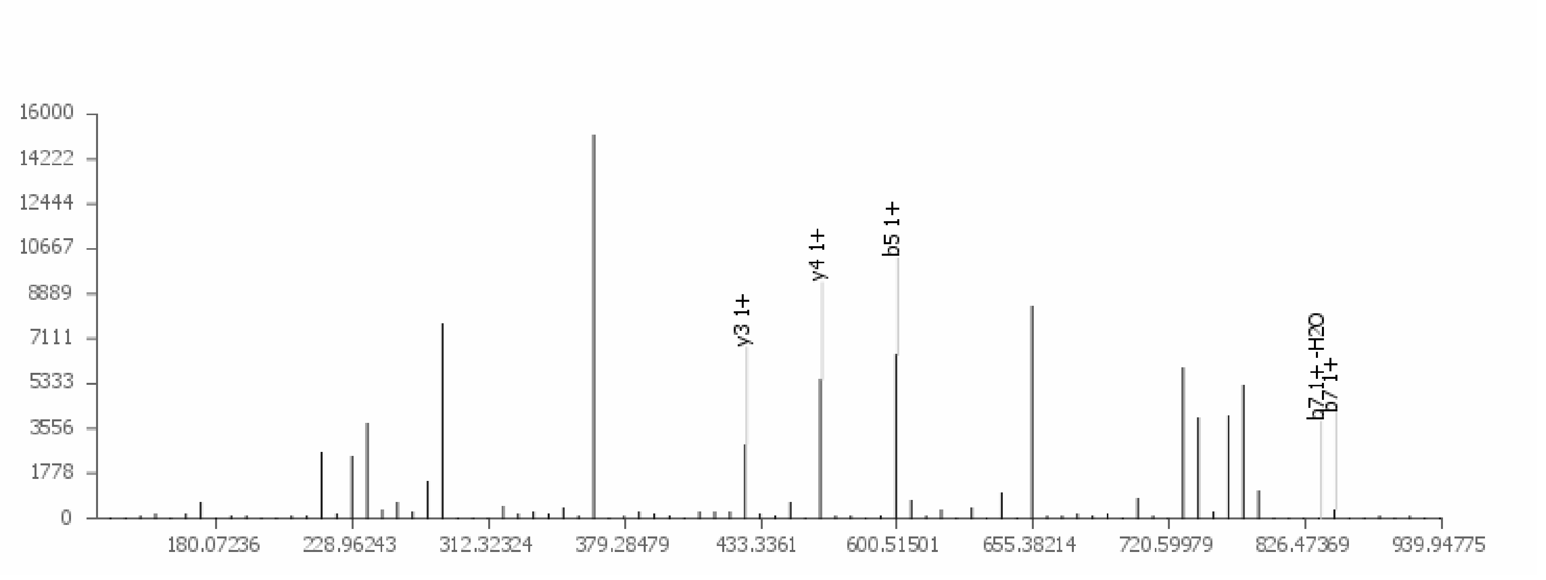

AT3G48430.1 - NEELD(pS)(pY)MEGPSTRLR - 686.279099 - Charge:3

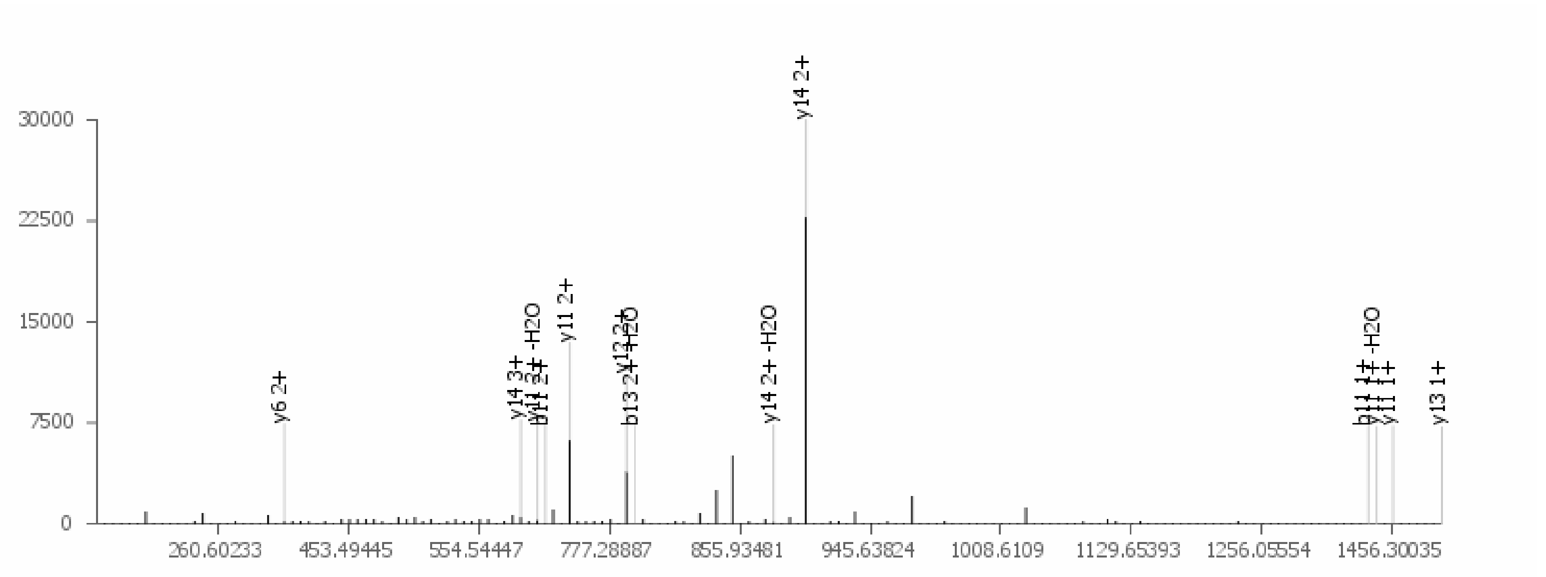

AT3G20740.1 - (oxM)(pS)KITLGNESIVG(pS)L(t)P(s)NK - 778.000509 - Charge:3

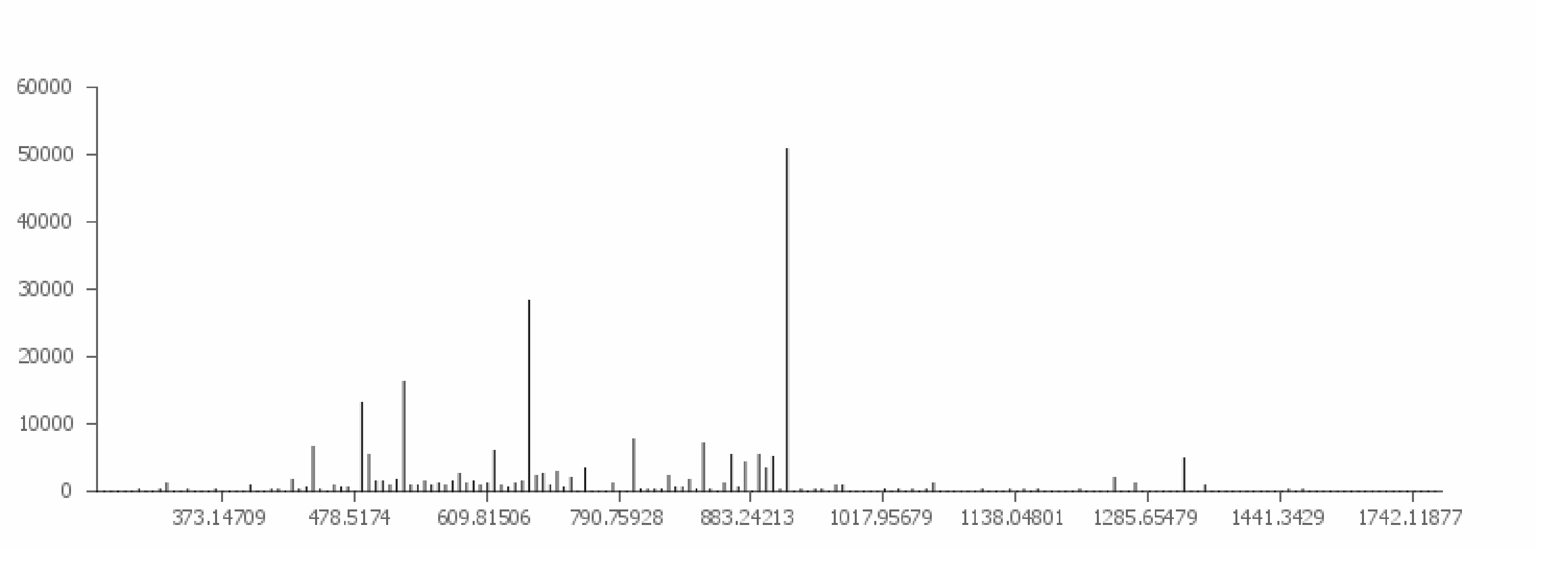

AT1G77510.1 - NGGK(pS)VQD(pY)NGPR - 776.296974 - Charge:2

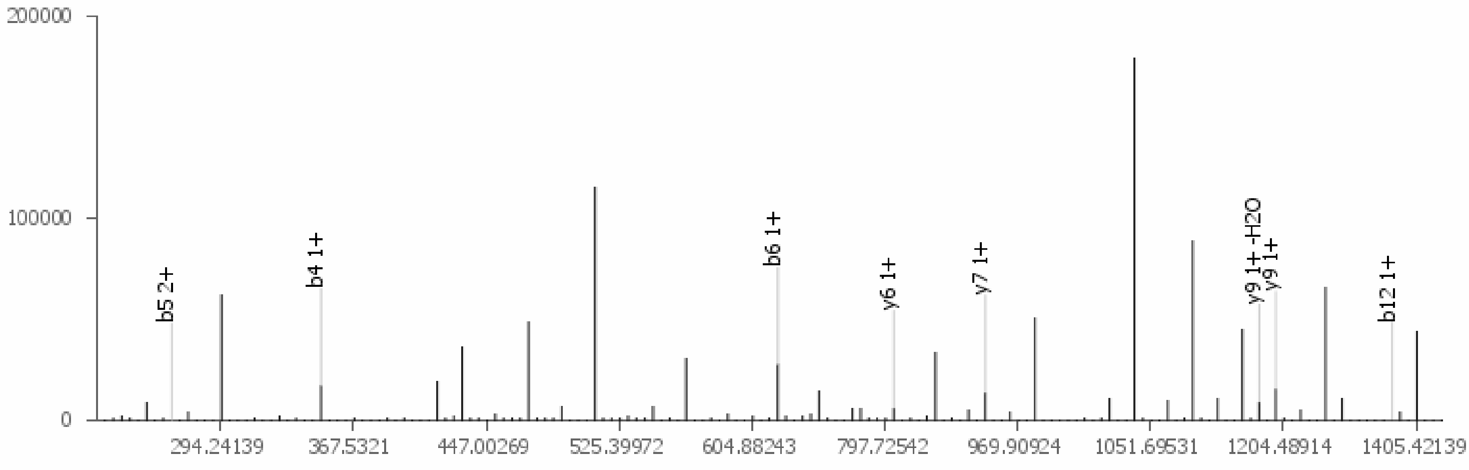

AT4G30620.1 - M(pS)DLAQSLG(oxM)PPGLDGLK - 963.441279 - Charge:2

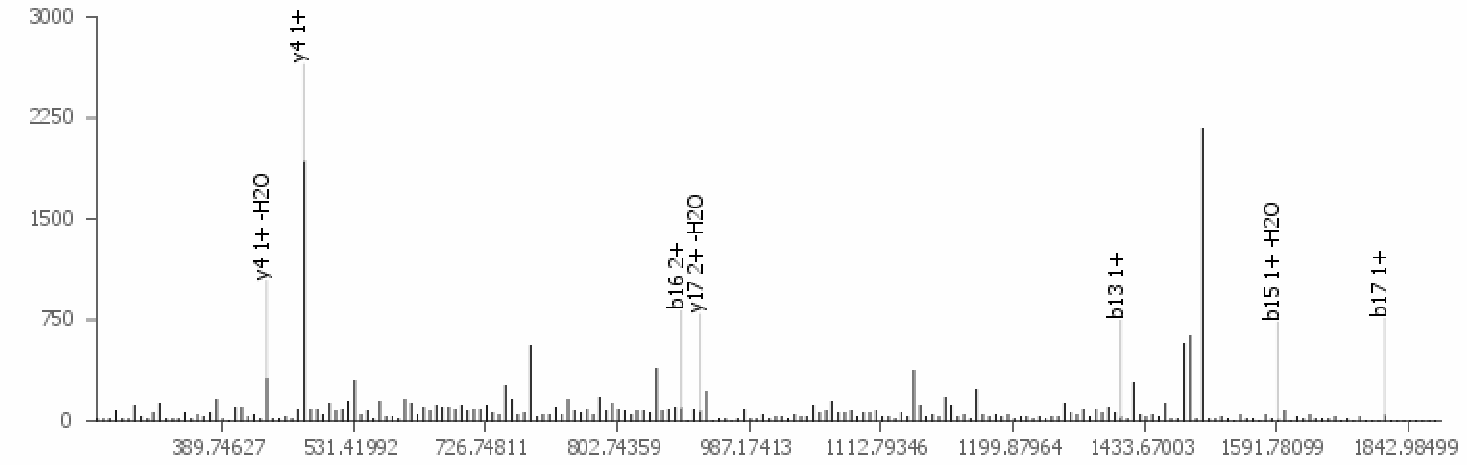

AT2G17050.1 - ALAYIVNIVGE(pS)(pS)Q(pY)WVDK - 1198.000222 - Charge:2

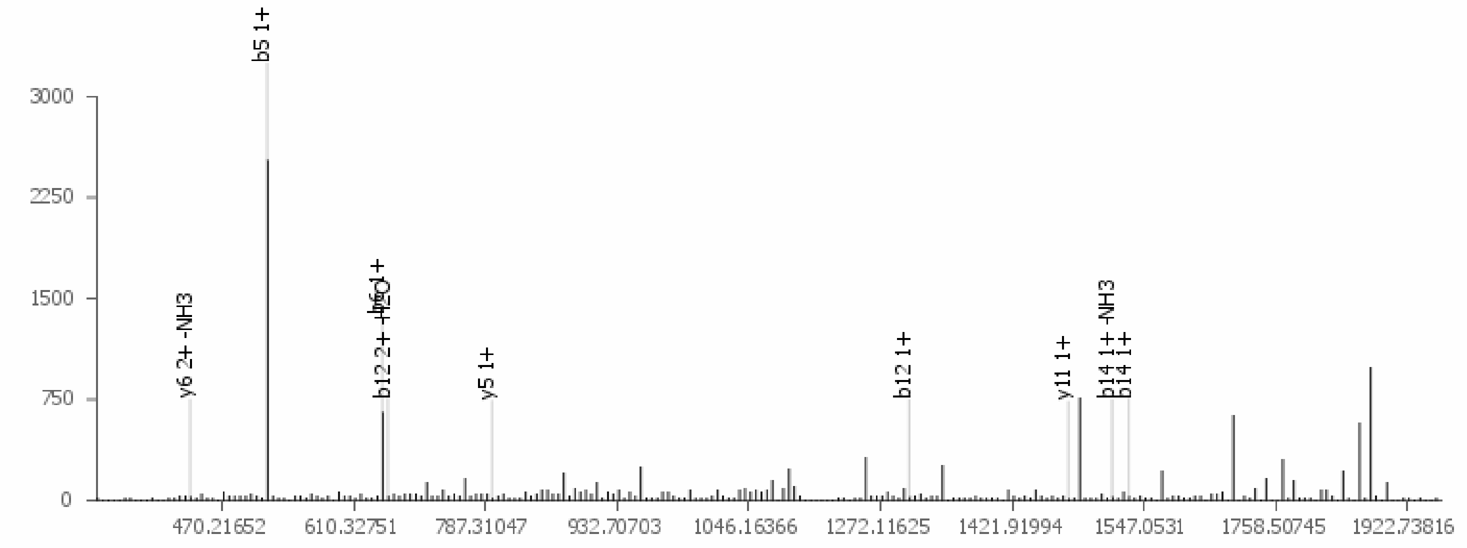

AT3G07770.1 - AQS(t)GD(t)ISLD(pY)MK - 845.320031 - Charge:2

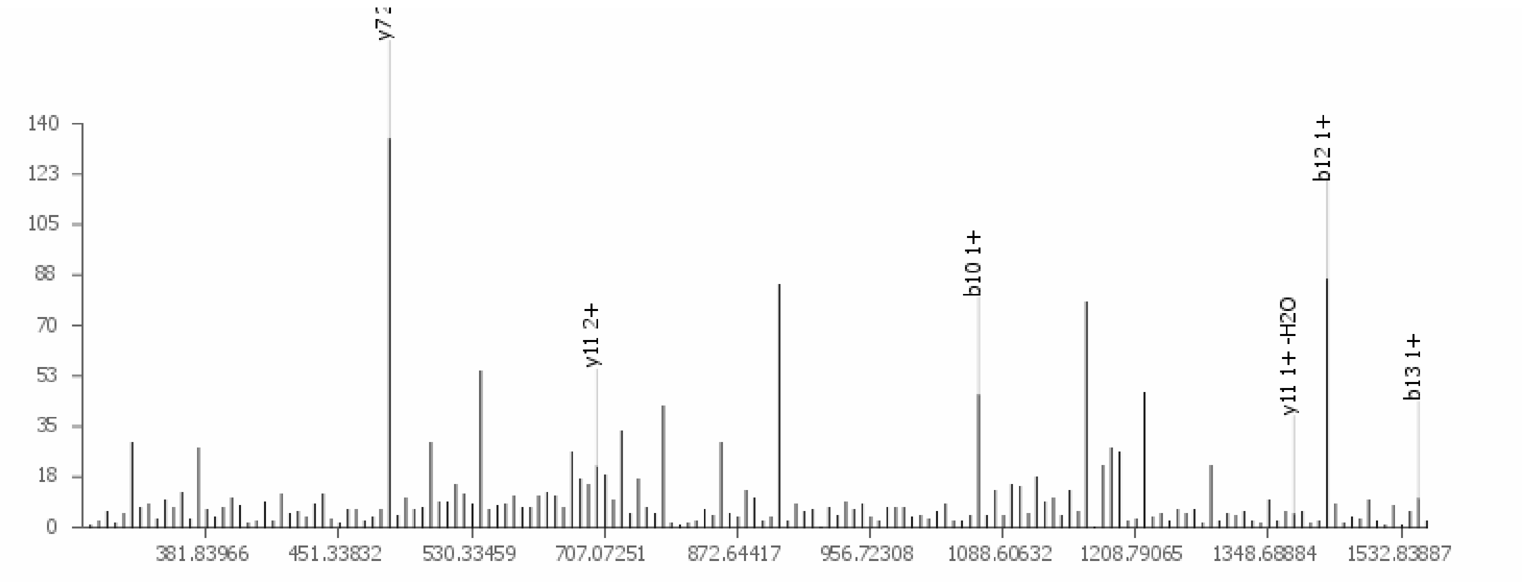

AT1G48970.1 - EA(pT)RVFLGASSIYSNG(pT)L(pY)AR - 1258.544149 - Charge:2

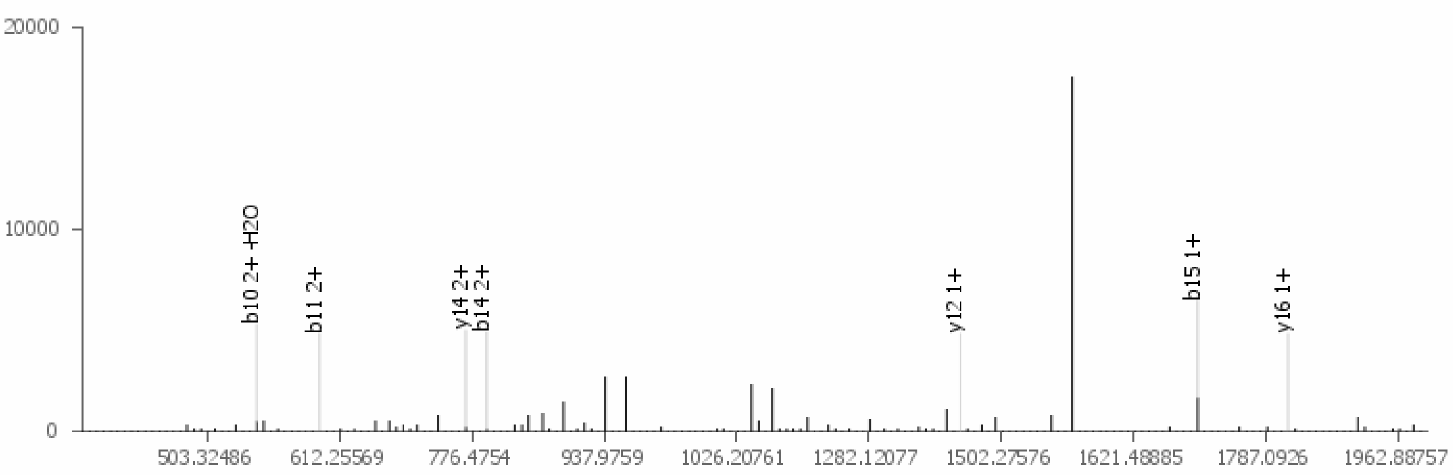

AT2G41520.1 - (pT)PDGA(pS)DALVPIANAL(pS)ISSCSDK - 877.022478 - Charge:3

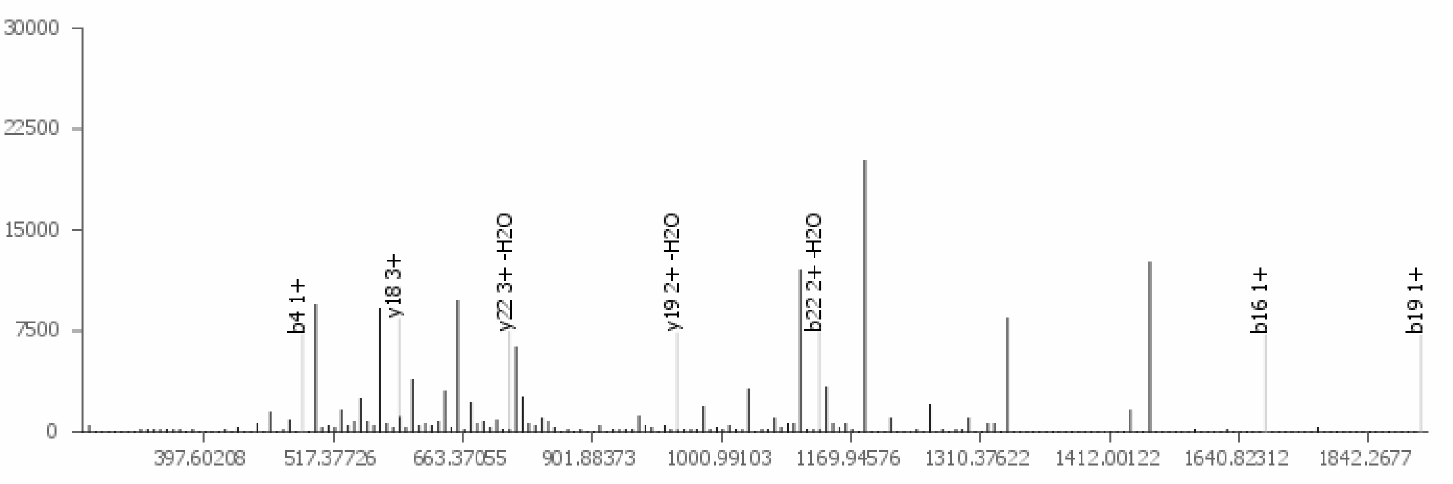

AT3G25905.1 - IFPE(pT)PA(pS)GK - 603.750062 - Charge:2

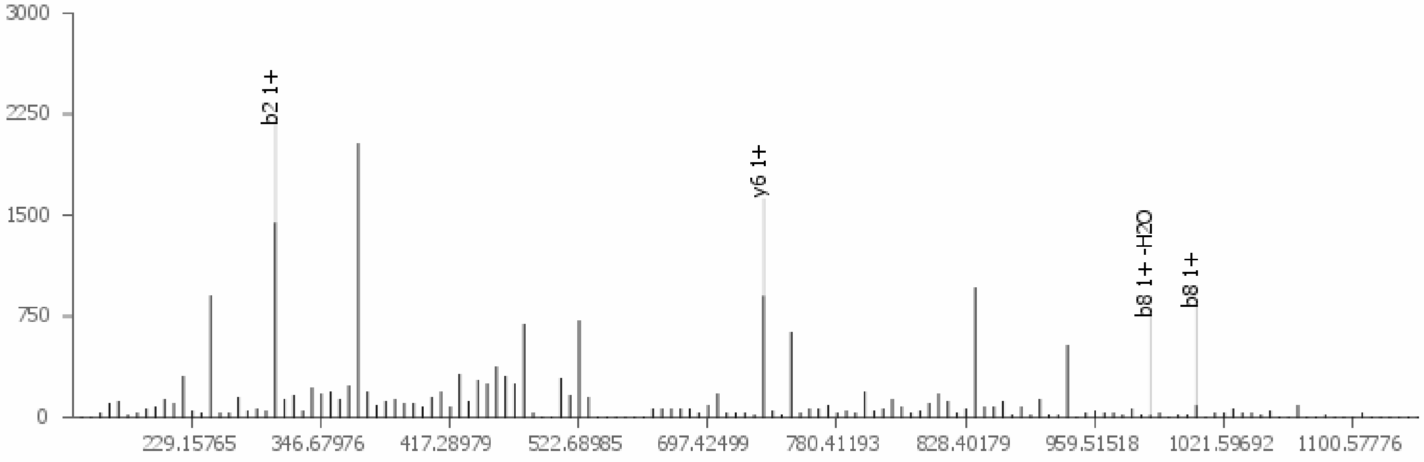

AT1G24180.1 - ESPIPDA(s)ELF(t)NMYVK - 1010.94895 - Charge:2

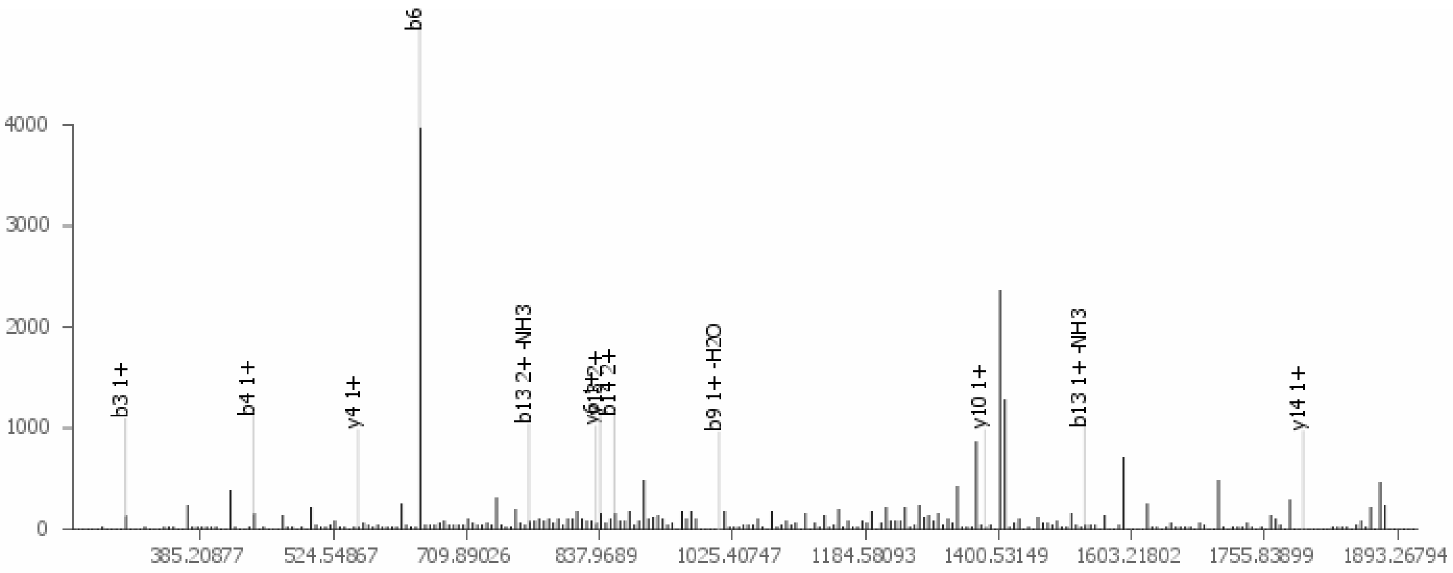

AT5G07070.1 - HPNVVEL(pY)EV(oxM)ATKSR - 984.972816 - Charge:2

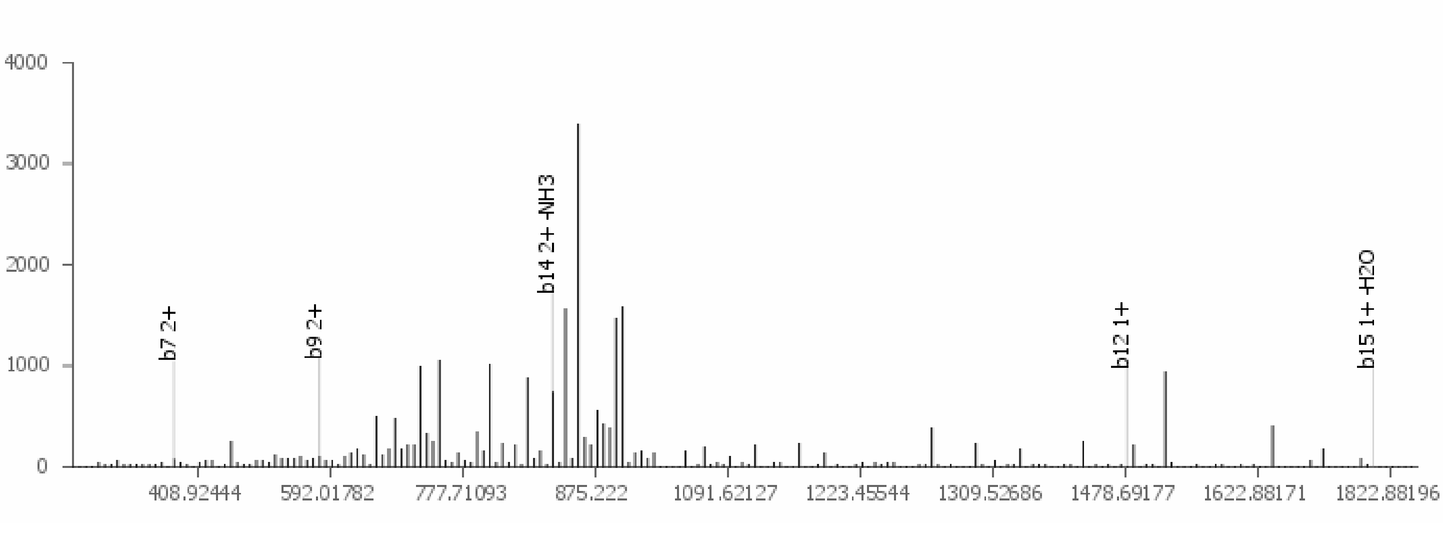

AT4G16900.1 - NI(pS)PNIFRLTILK - 804.93948 - Charge:2

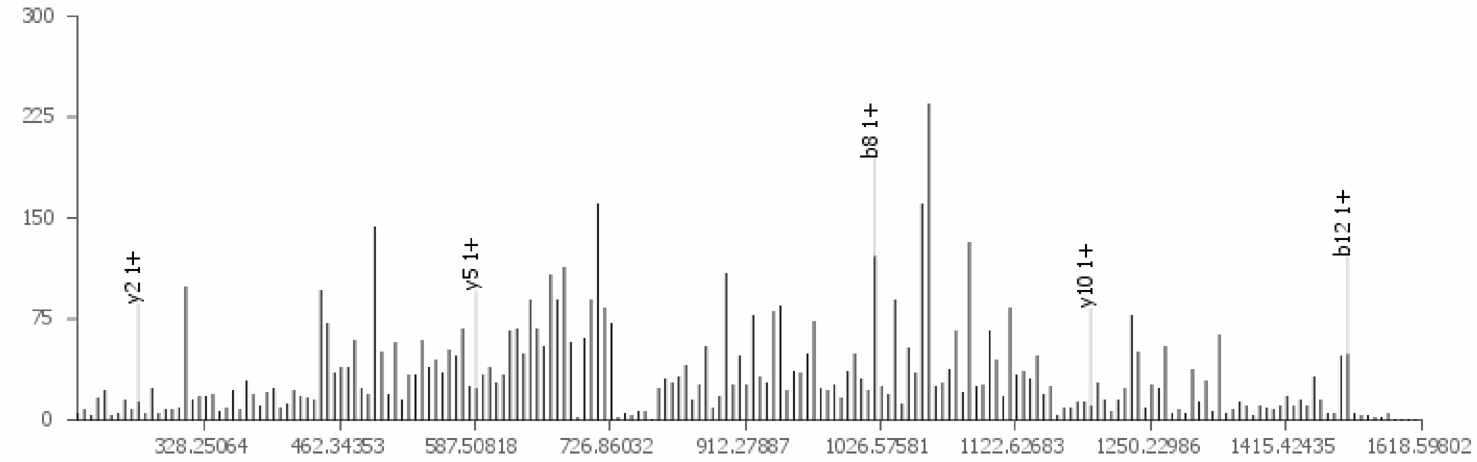

AT5G35200.1 - YLGAIKDTT(pT)VSLAK - 830.934366 - Charge:2

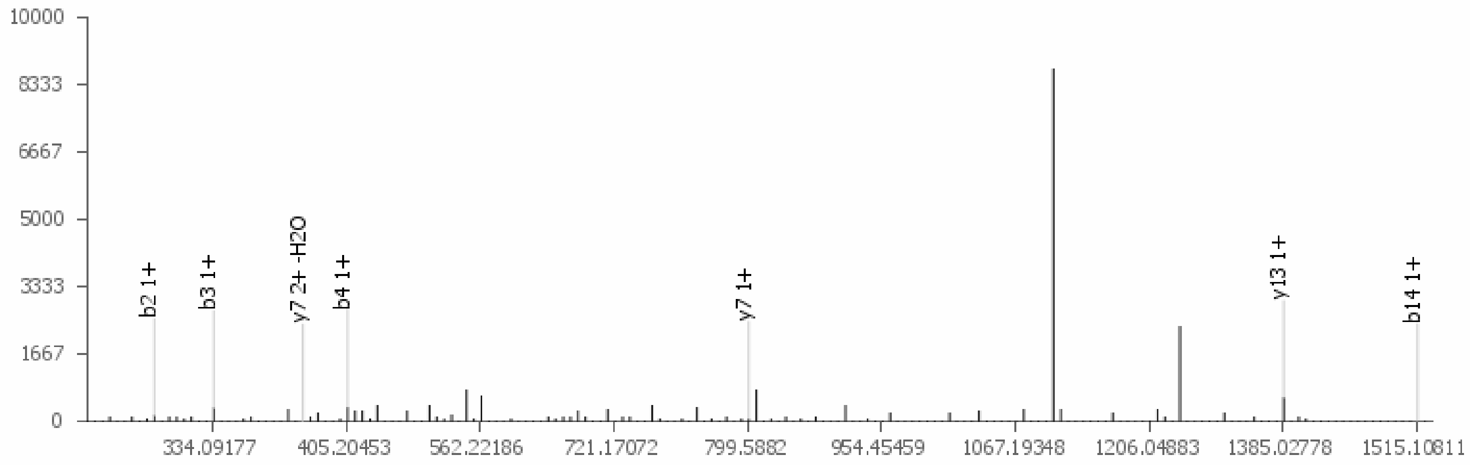

AT5G46310.1 - TSKYPLLDL(pS)FK - 746.377401 - Charge:2

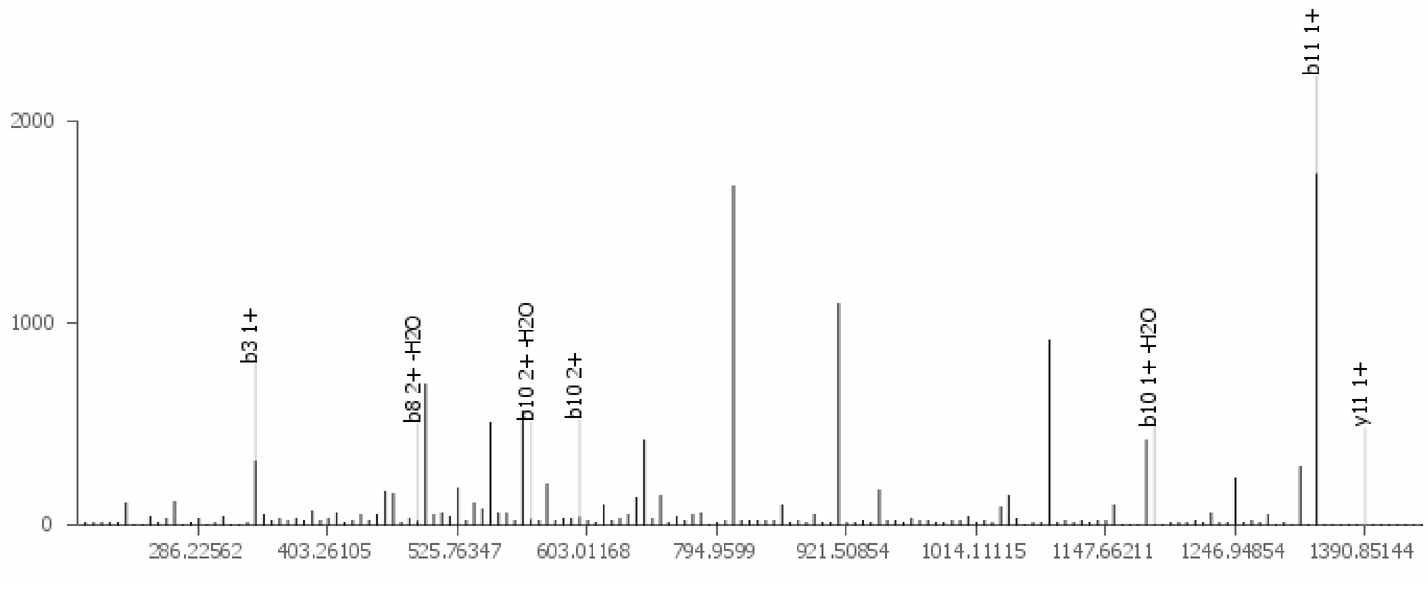

AT2G28000.1 - AIELPNA(oxM)ENAGAALIREVA(pS)K - 1182.5882 - Charge:2

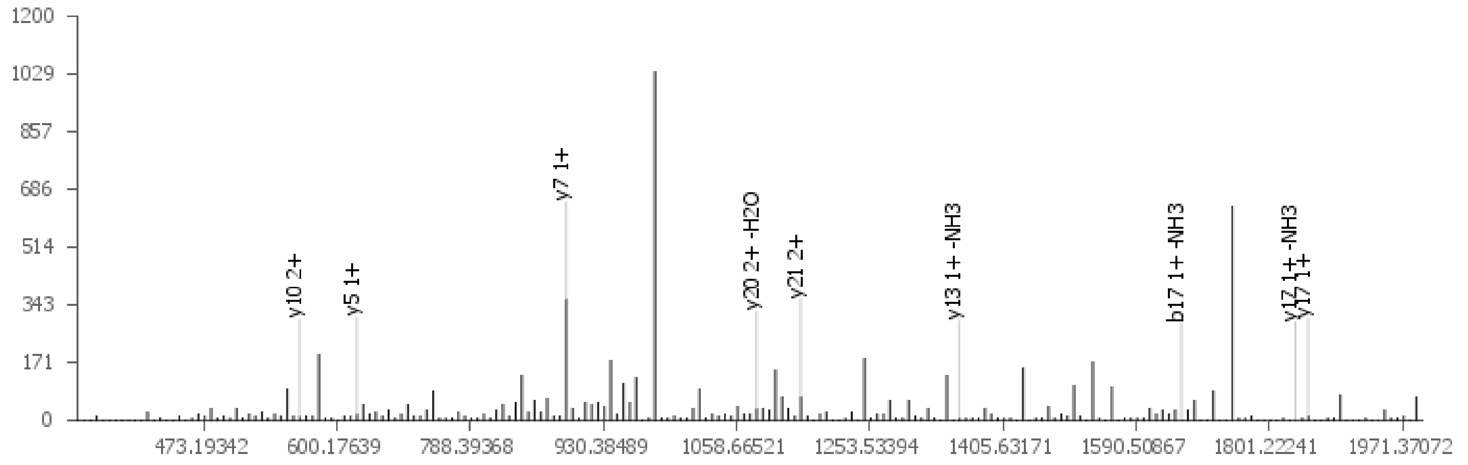

AT3G23110.1 - SS(pS)LFKLR - 1104.539202 - Charge:1

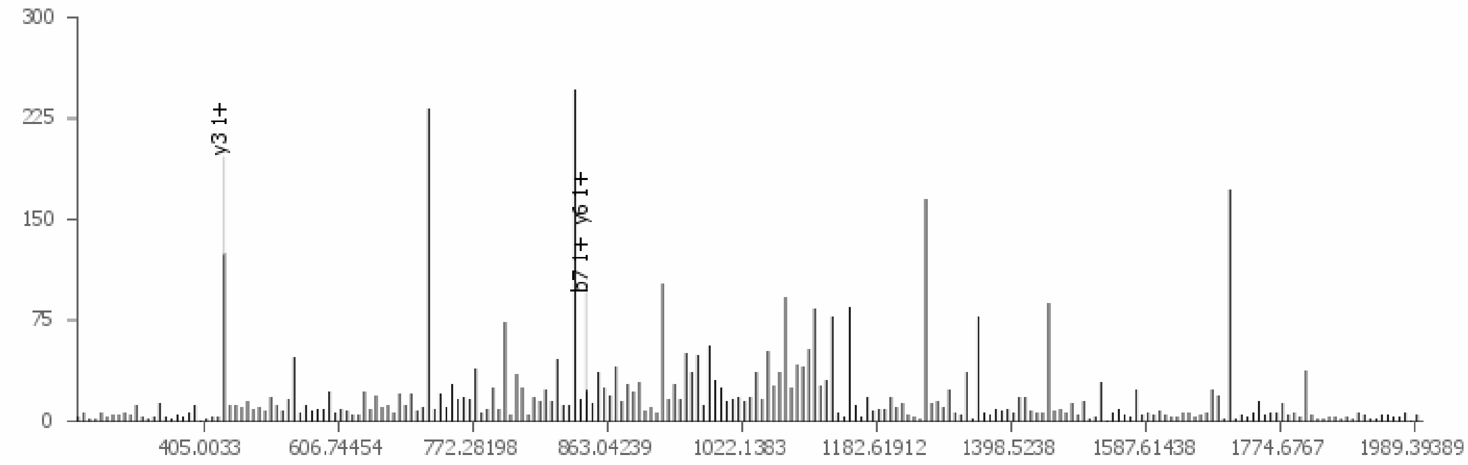

AT3G63300.1 - SHDDIV(t)L(t)AAAATALRGAATLK - 783.076312 - Charge:3

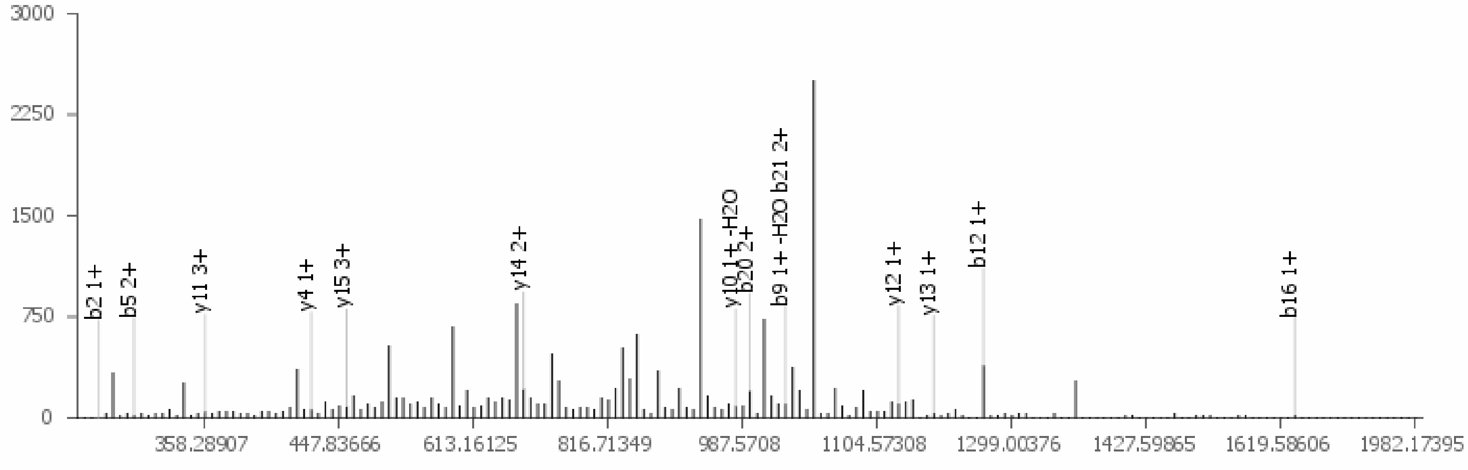

AT3G49030.1 - EVAT(pY)ILENARLLK - 571.640731 - Charge:3

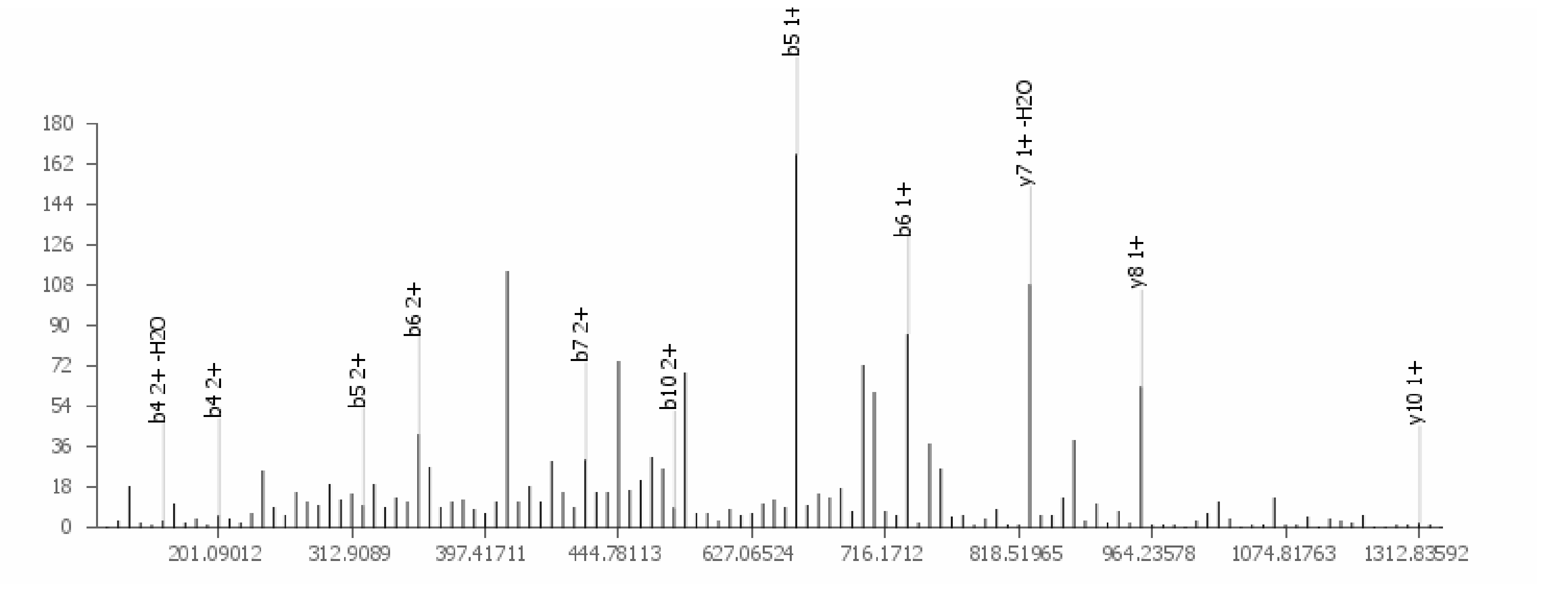

AT3G23110.1 - NNL(pS)GEIPR - 540.248825 - Charge:2

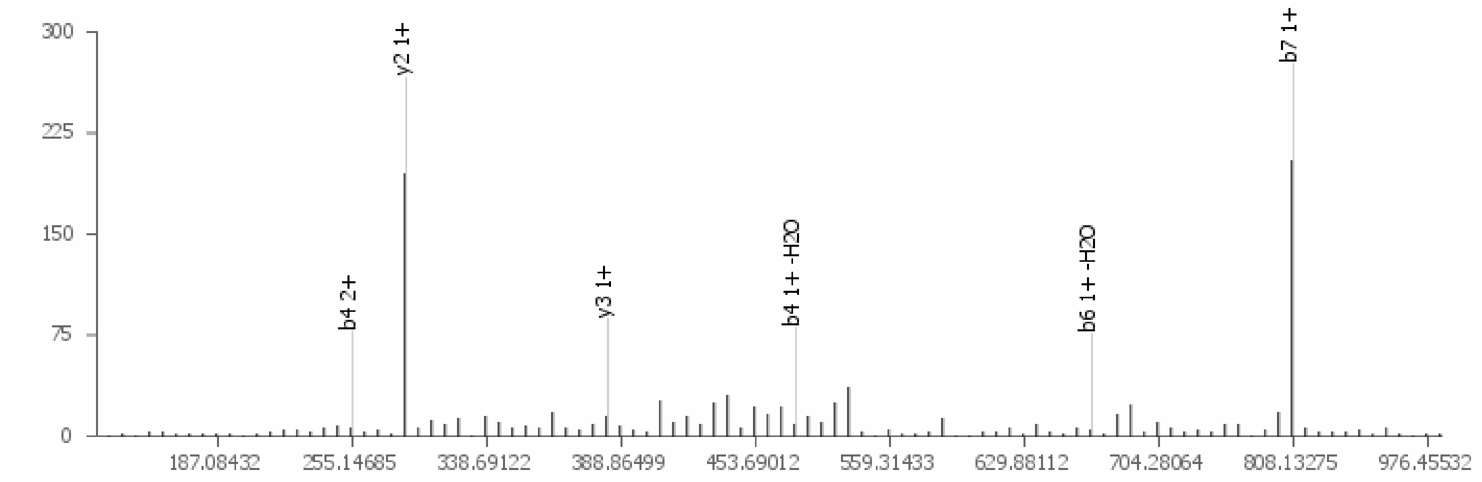

AT4G00280.1 - NCYNVQPPQGKGFE(pY)LIITR - 1239.084705 - Charge:2

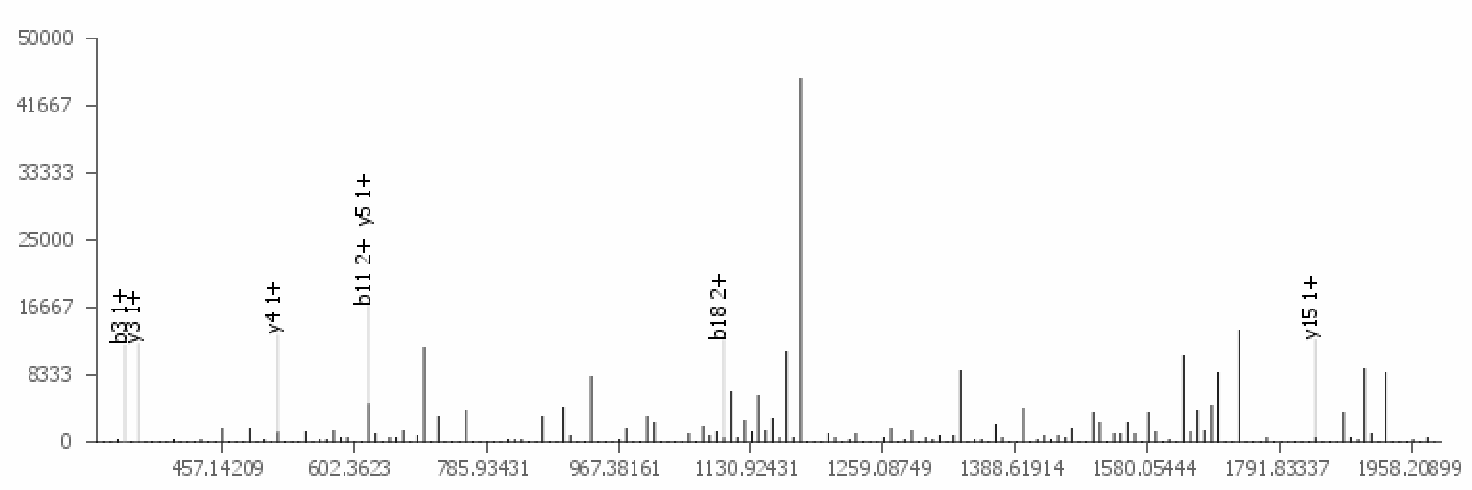

AT3G13410.1 - MENLLL(pS)GLK - 599.299031 - Charge:2

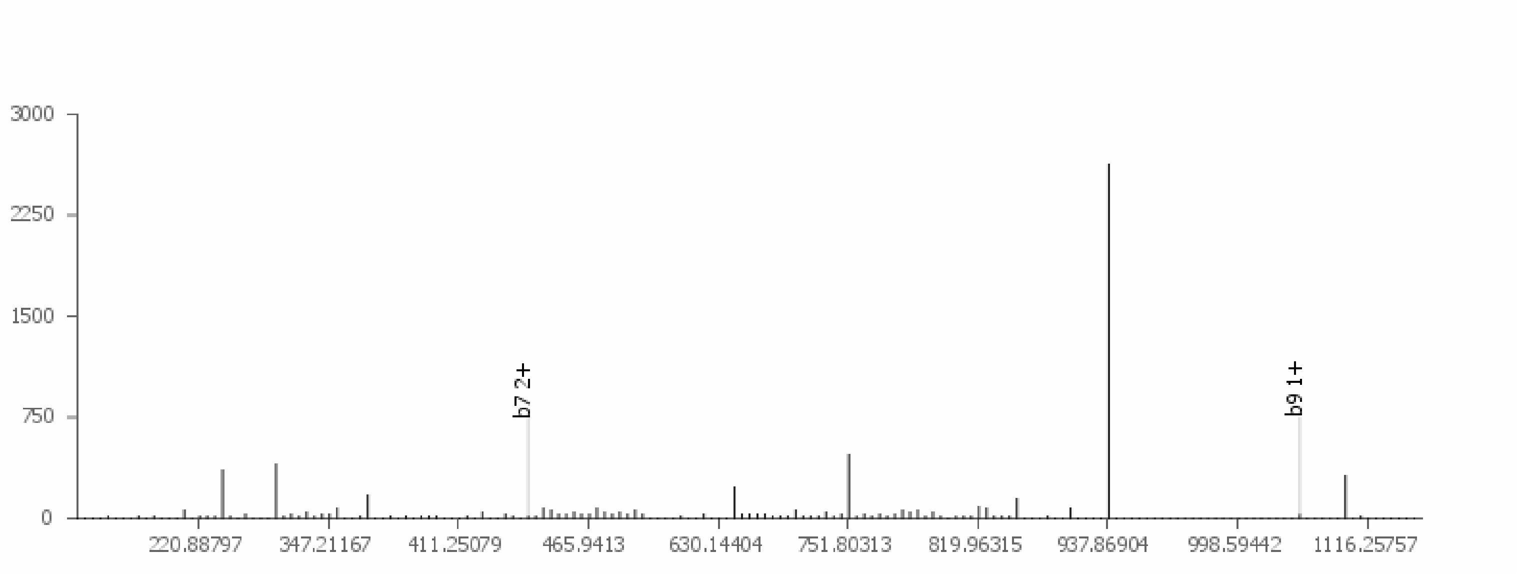

AT2G28830.1 - IVY(s)(s)GAVPGIVHVLQK - 616.331504 - Charge:3

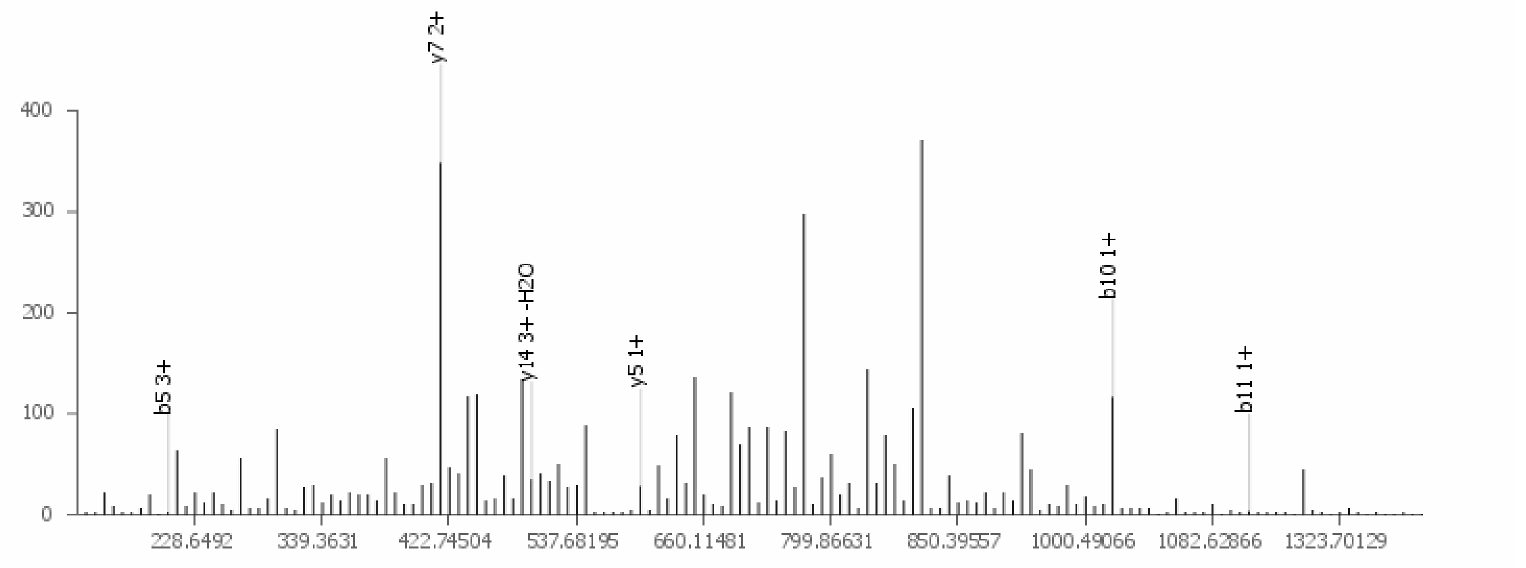

AT5G16440.1 - (pT)QLSVR - 783.37838 - Charge:1

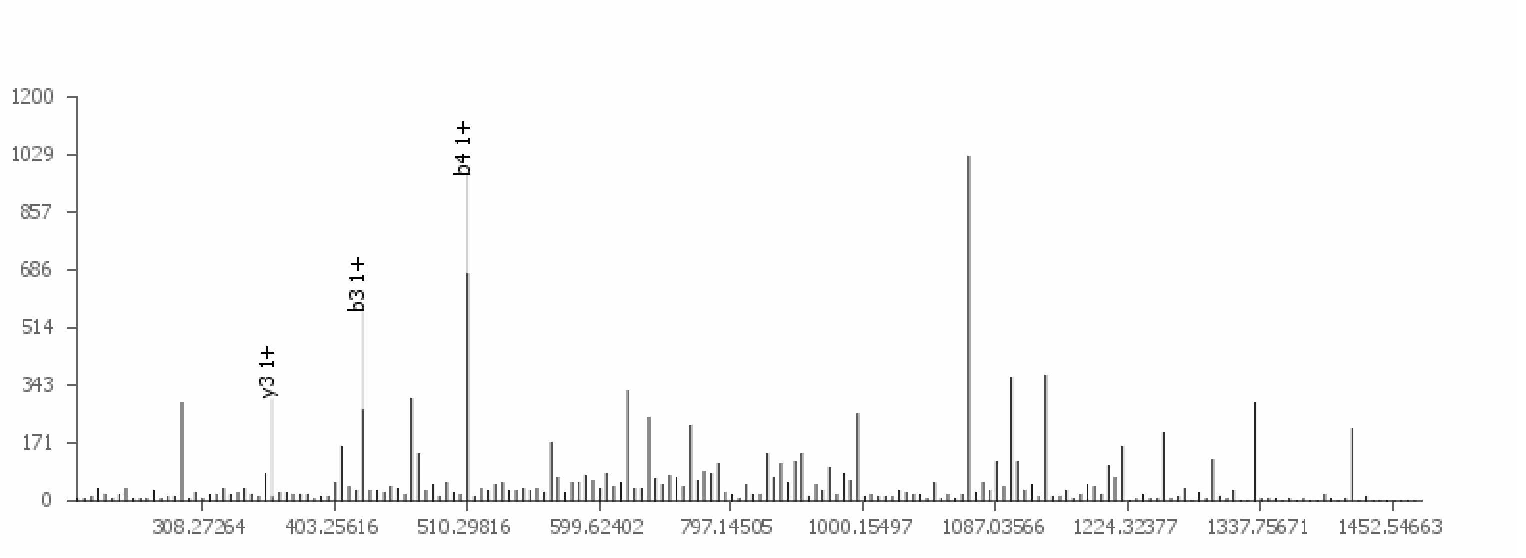

AT1G63640.1 - FEV(pS)DIEQGDMVPVLQSLK - 1107.527081 - Charge:2

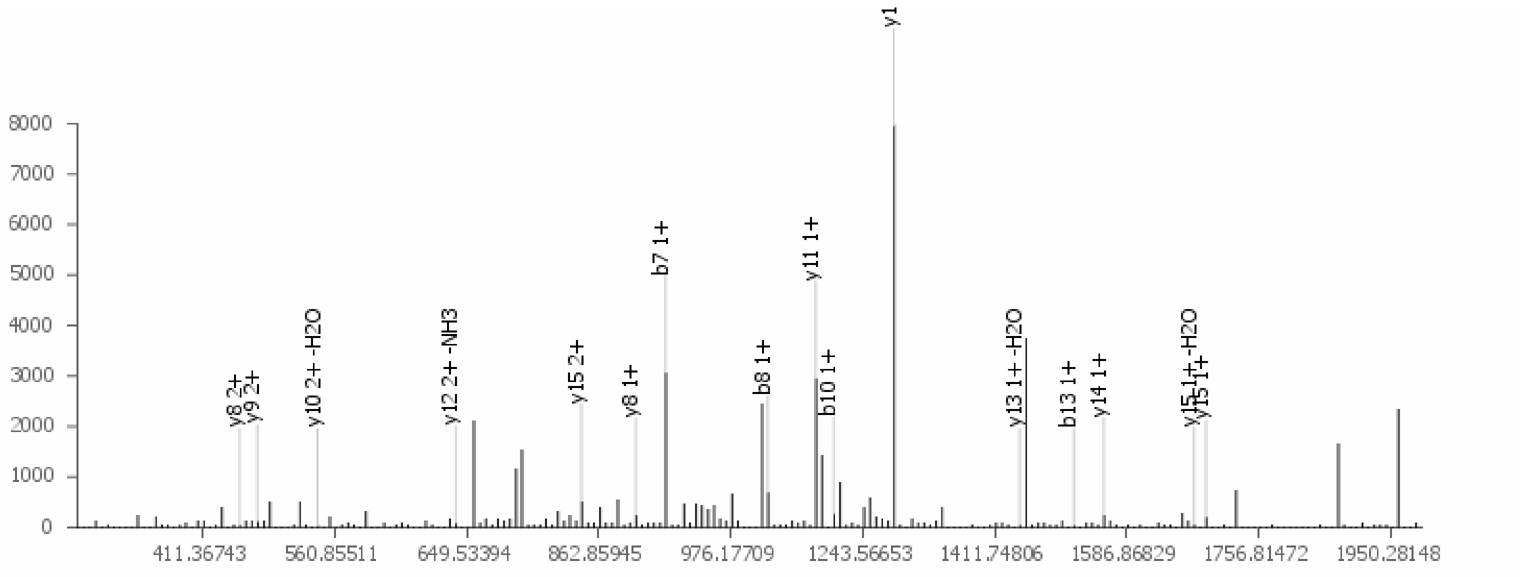

AT3G56760.1 - TAILKSS(pT)EATK - 665.342088 - Charge:2

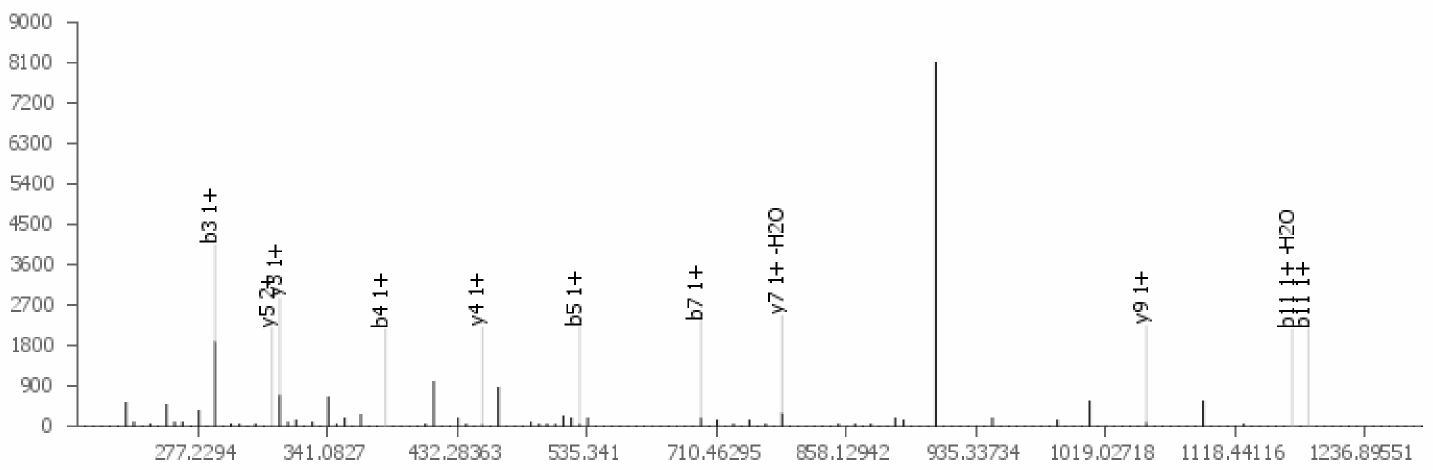

AT1G76430.1 - FVLGLGIGGDYPL(s)A(t)IMSEFANKR - 684.846789 - Charge:4

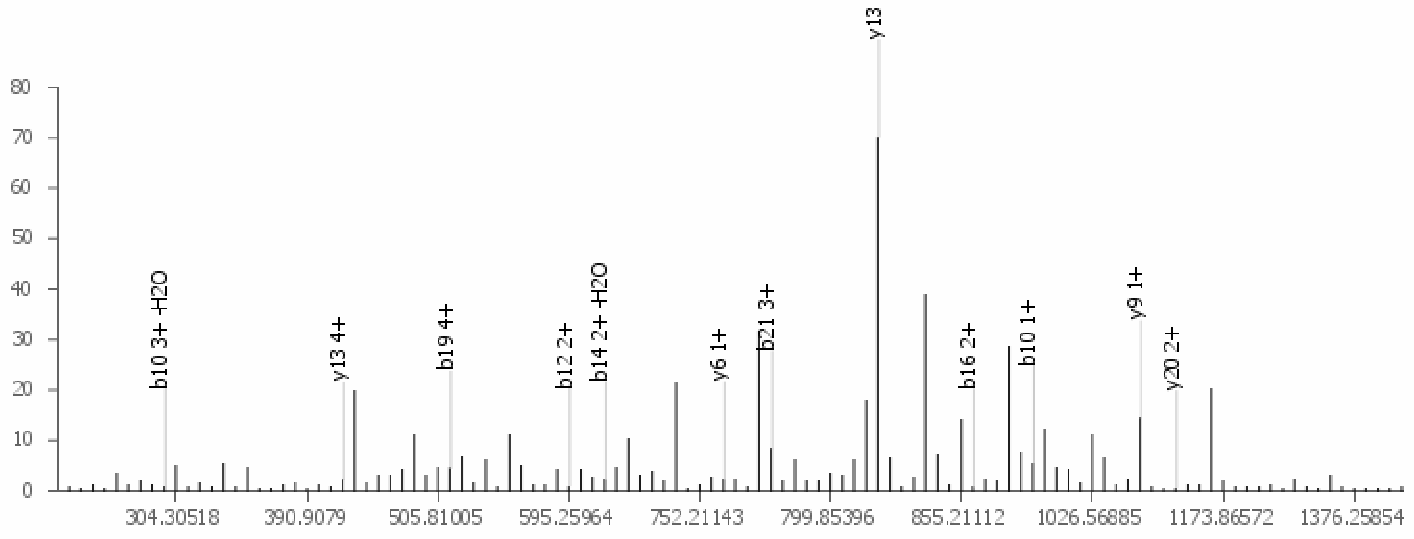

AT3G14380.1 - TDQ(pT)AIDESALVLNRTEK - 1042.508192 - Charge:2

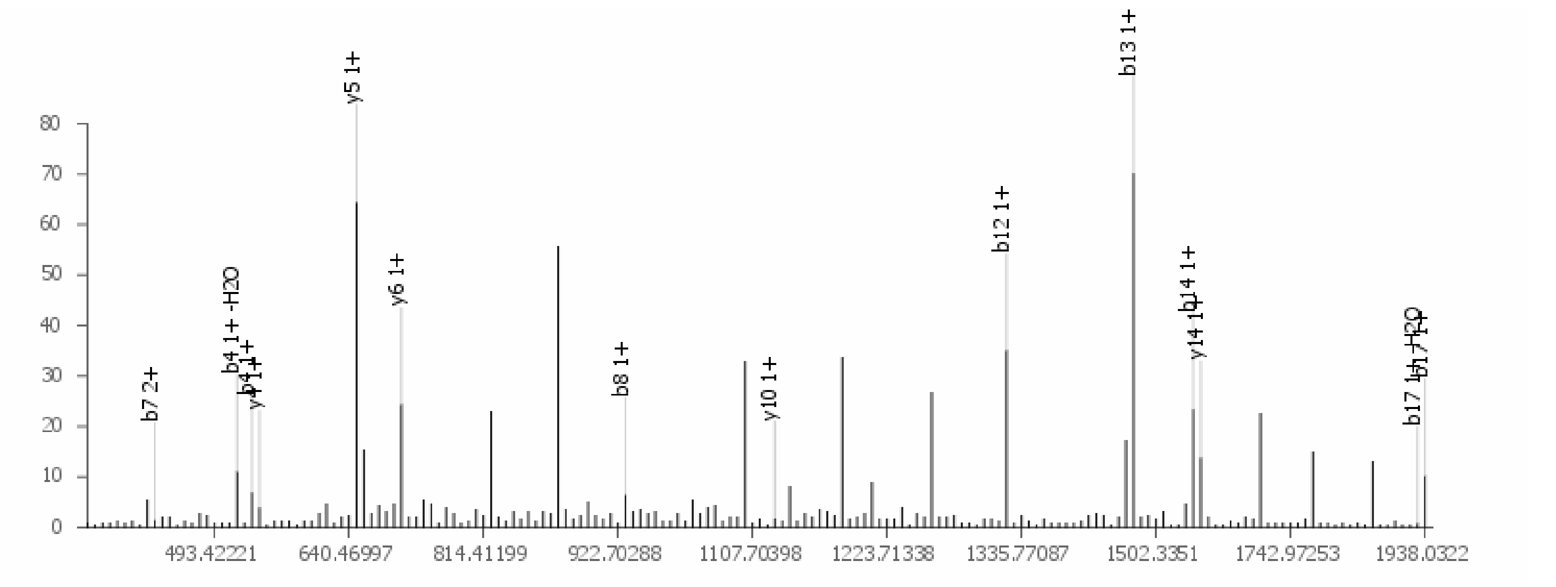

AT1G64290.1 - (pS)IGVDLITWTPSNDVVLFR - 1106.552536 - Charge:2

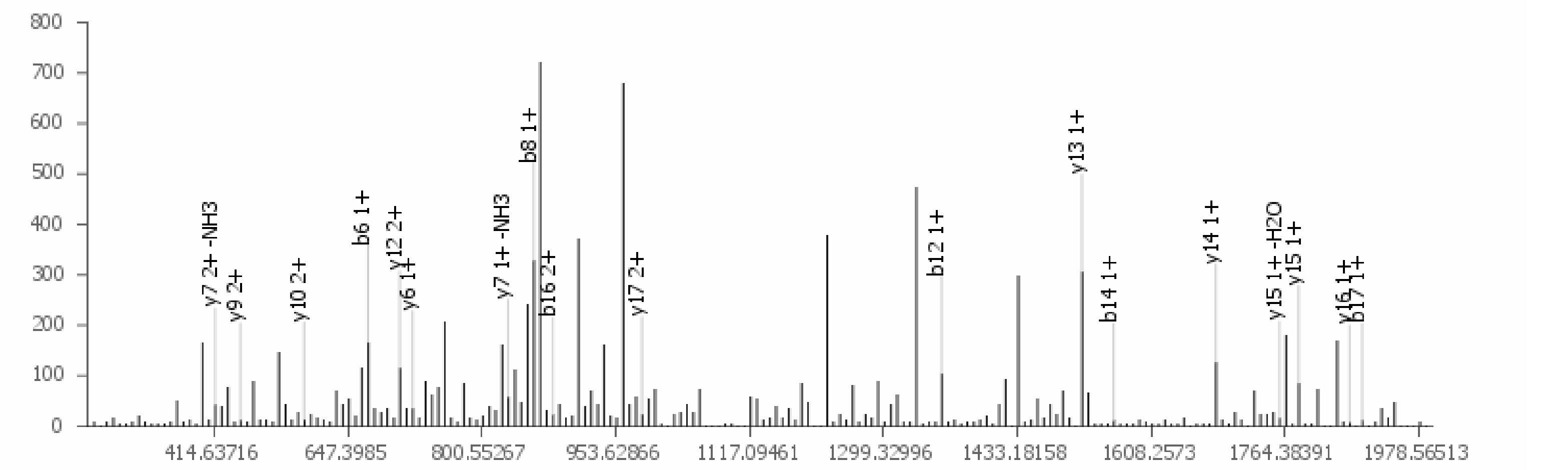

AT2G20260.1 - AMTSAATGFILTANVPAAIGGG(s)(s)K - 1187.081151 - Charge:2

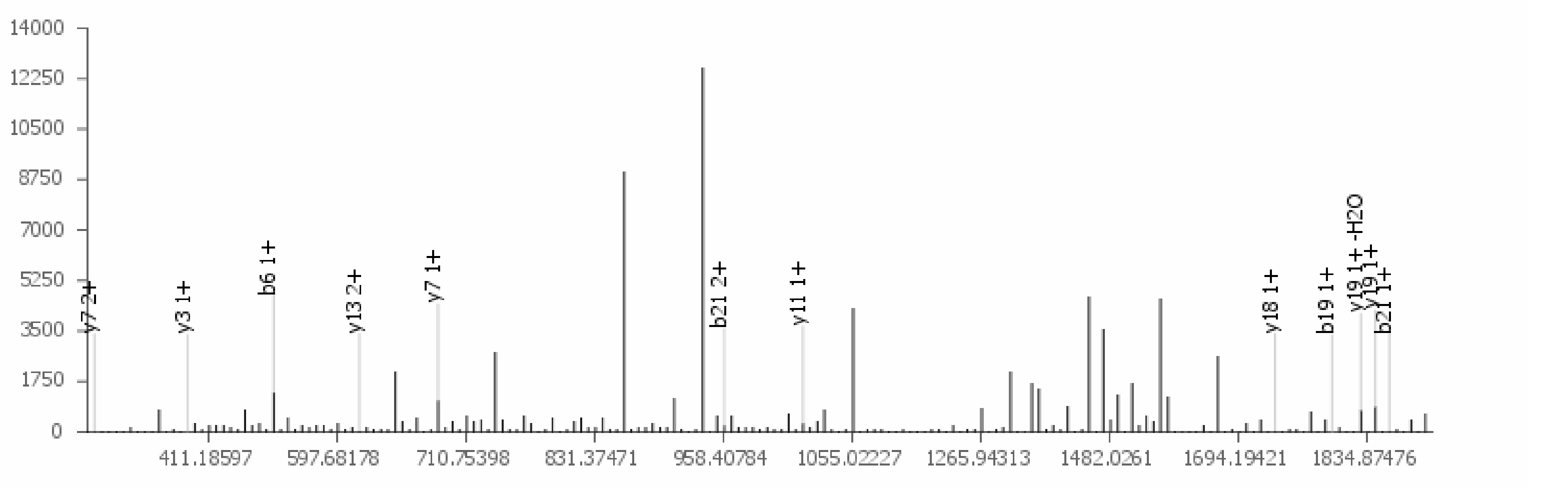

AT1G68760.1 - AVLVDP(pS)QEPK - 631.810519 - Charge:2

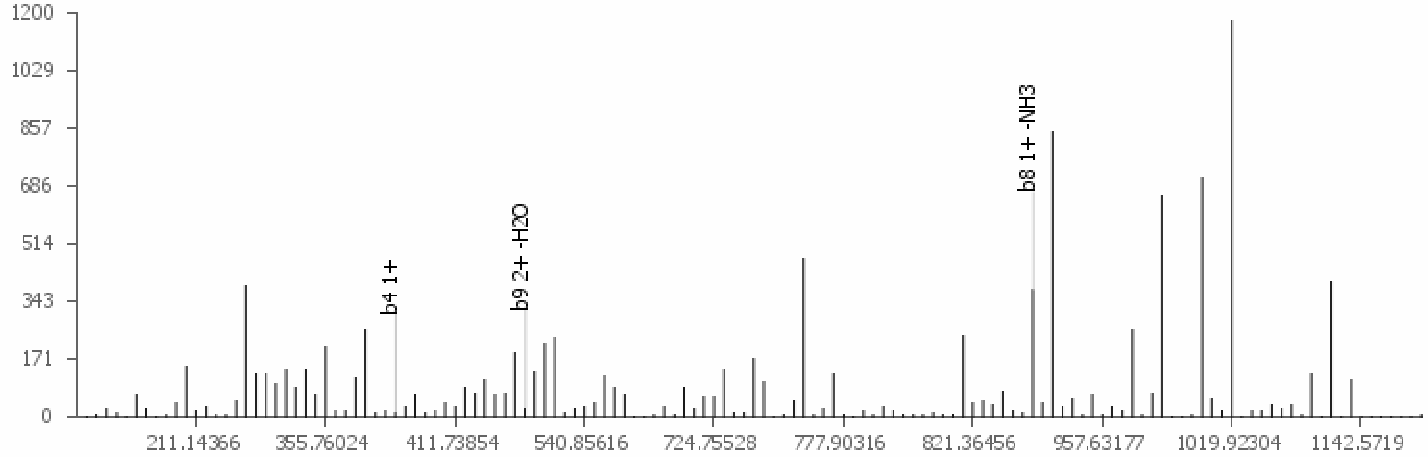

AT2G18960.1 - (pT)LHGLQPK - 487.248006 - Charge:2

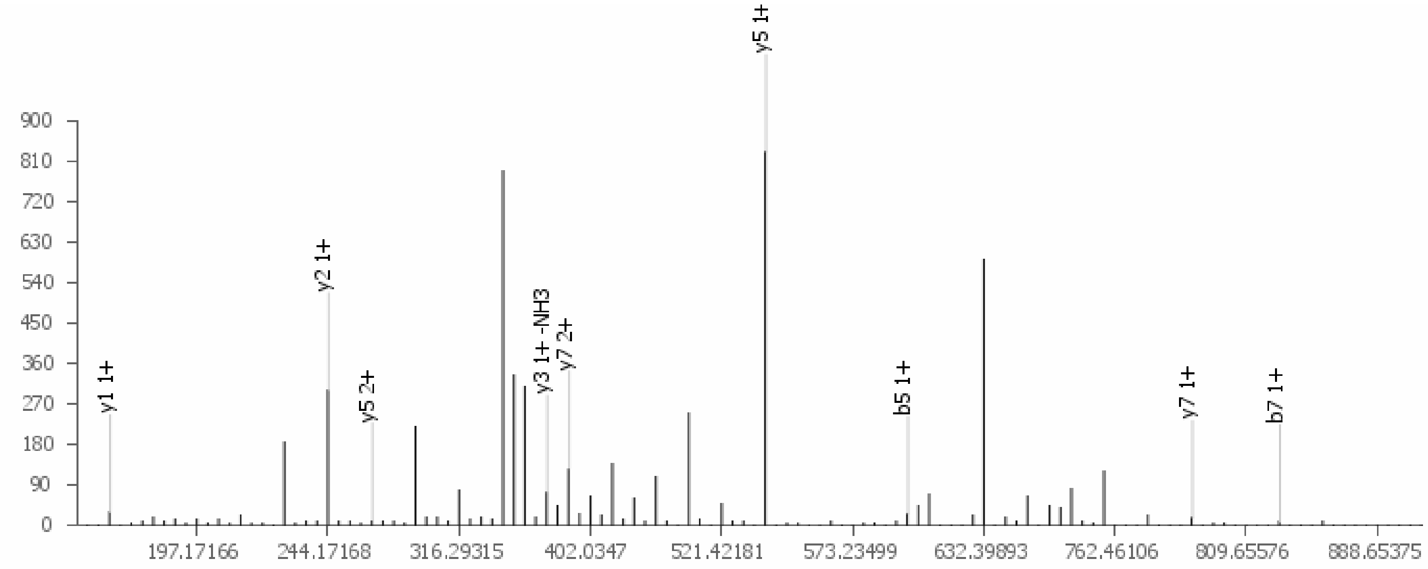

AT1G29870.1 - EAVVK(pT)LDR - 555.785841 - Charge:2

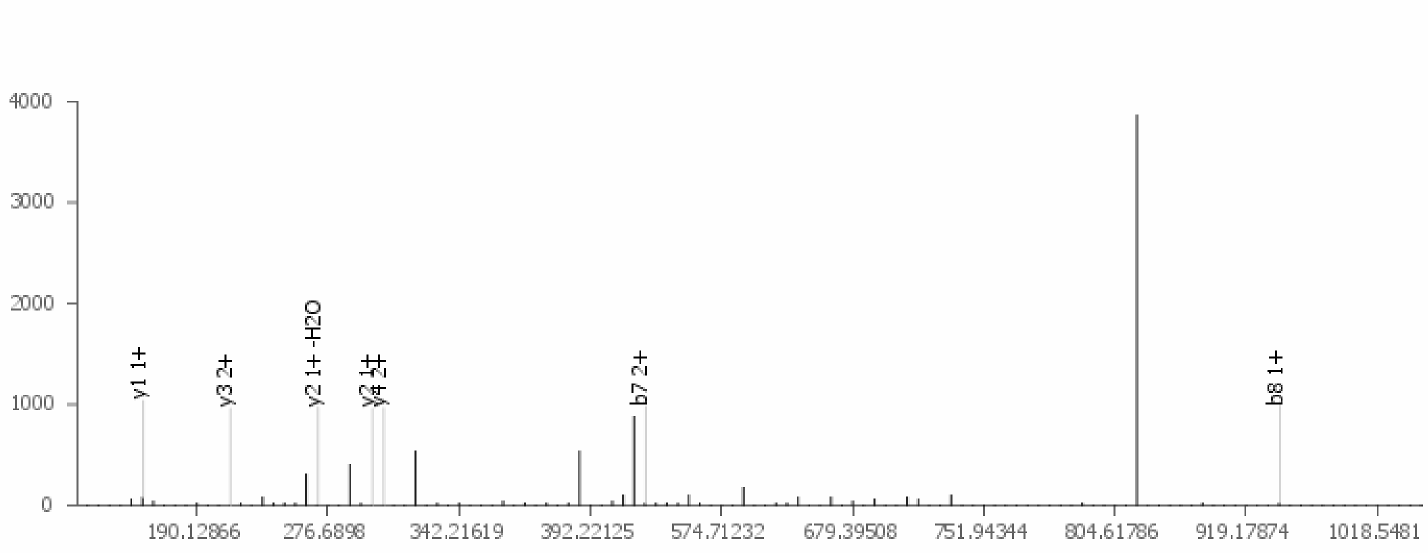

AT1G58250.1 - LSSLE(s)LI(s)R - 592.802034 - Charge:2

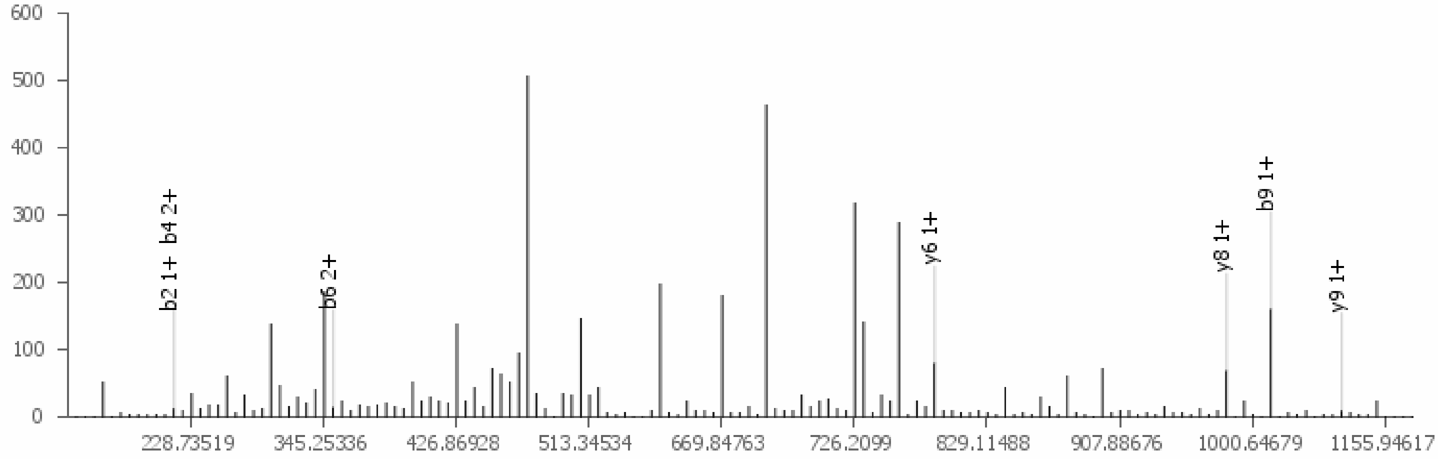

AT2G39010.1 - (pS)QLHELHA - 507.724943 - Charge:2

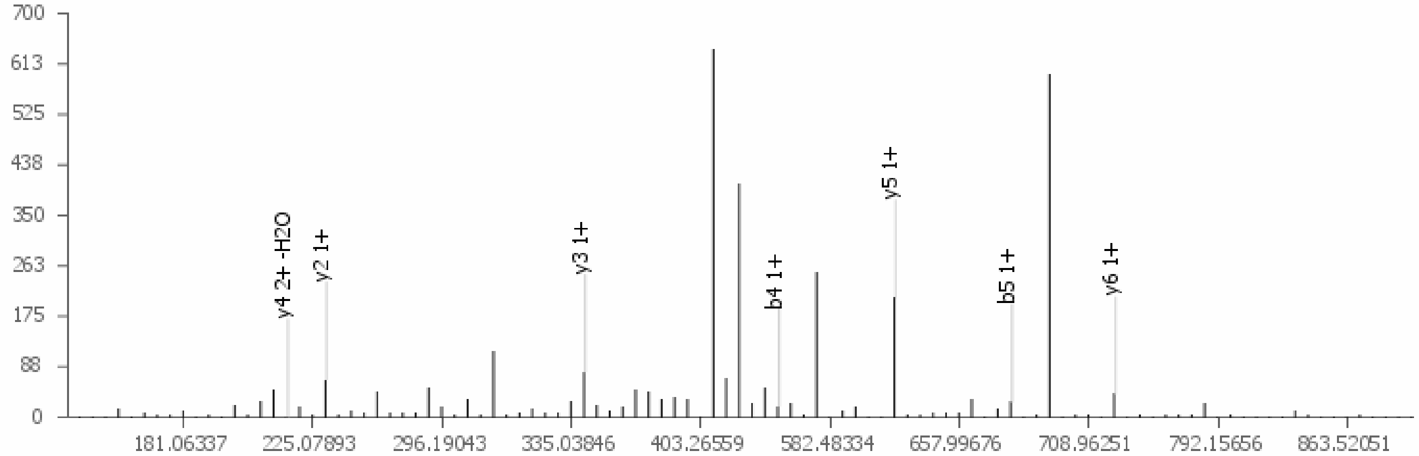

AT3G23750.1 - GGFGVVYAGELHDG(pT)KTAVK - 1043.505094 - Charge:2

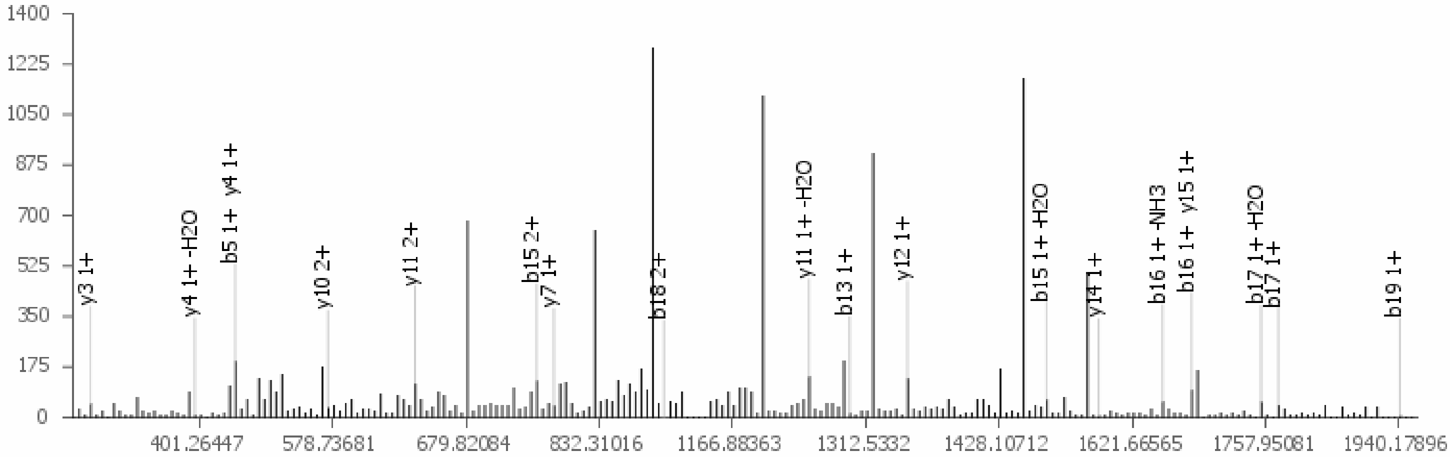

AT4G03520.1 - DTIIGAVPKTTLTS(pS)LDK - 970.50589 - Charge:2

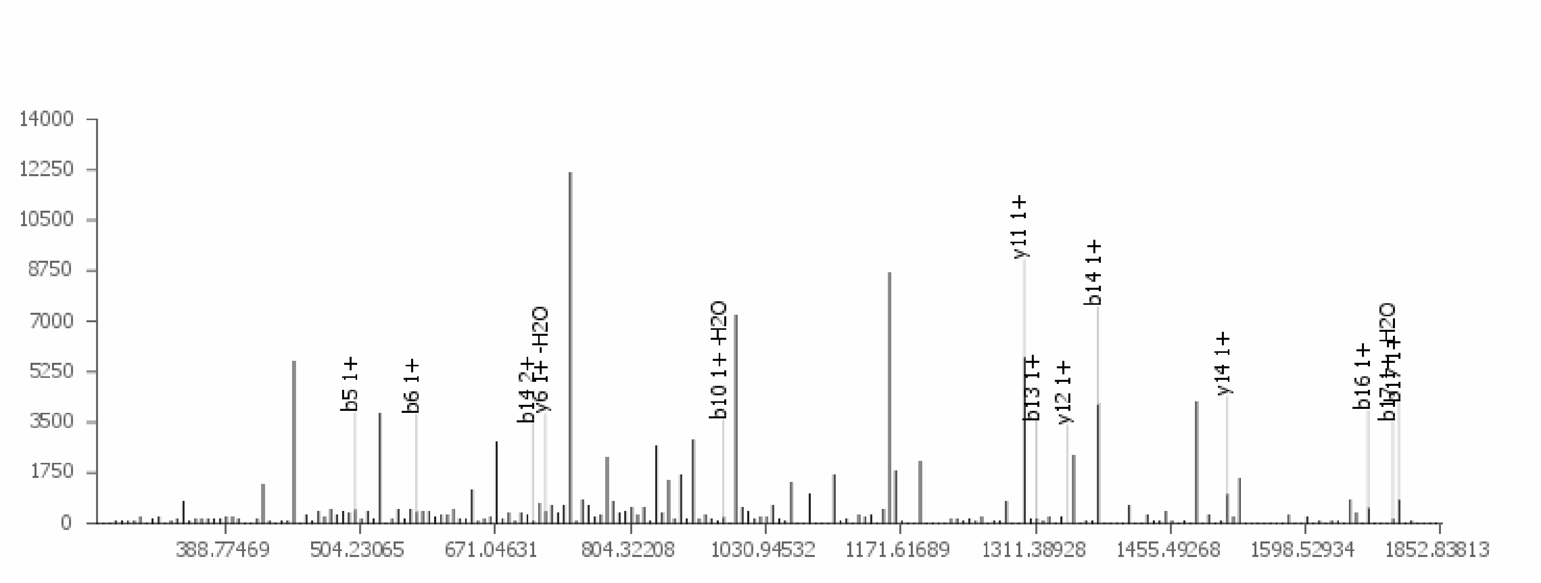

AT4G10640.1 - (pS)SRNQSAGDDR - 636.756503 - Charge:2

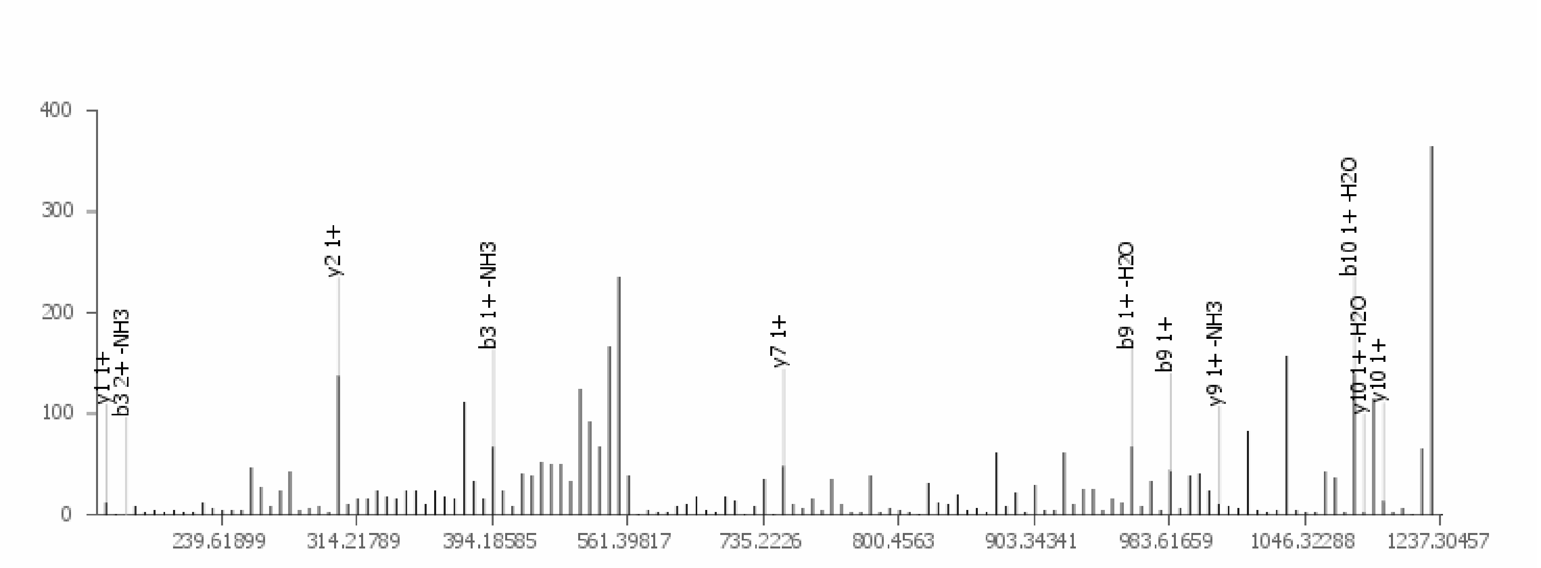

AT2G43490.1 - G(pS)SSESVDLVSGR - 453.86169 - Charge:3

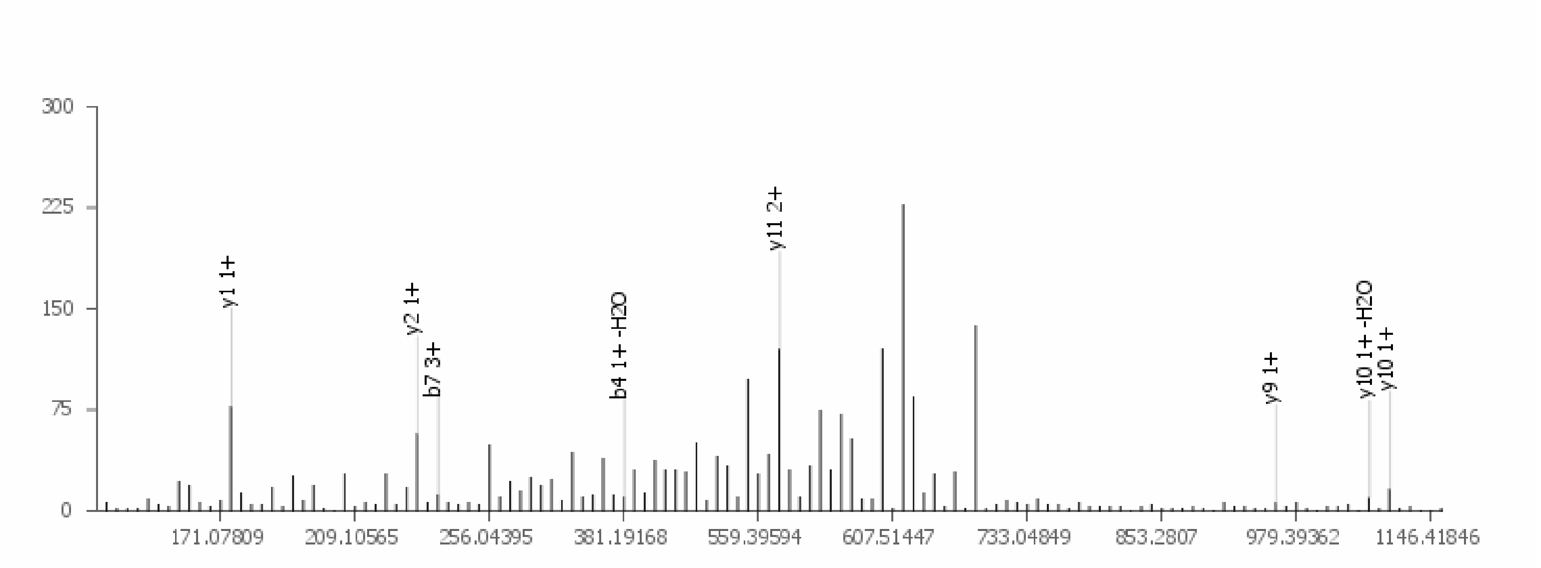

AT5G09400.1 - EKLEN(pS)LILK - 633.843088 - Charge:2

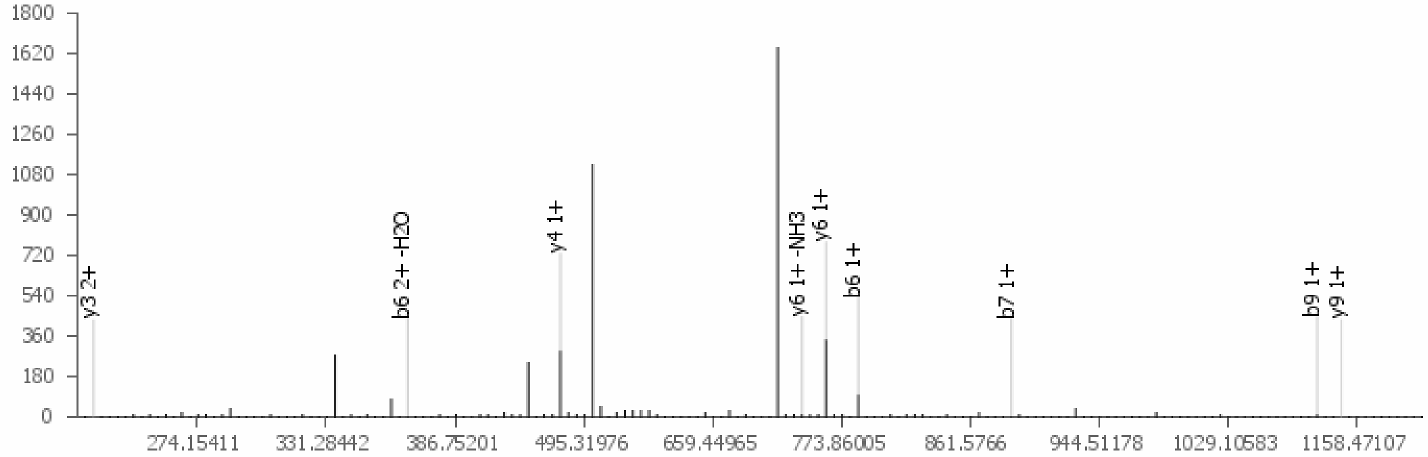

AT1G26761.1 - RFL(pS)DNEER - 623.266014 - Charge:2

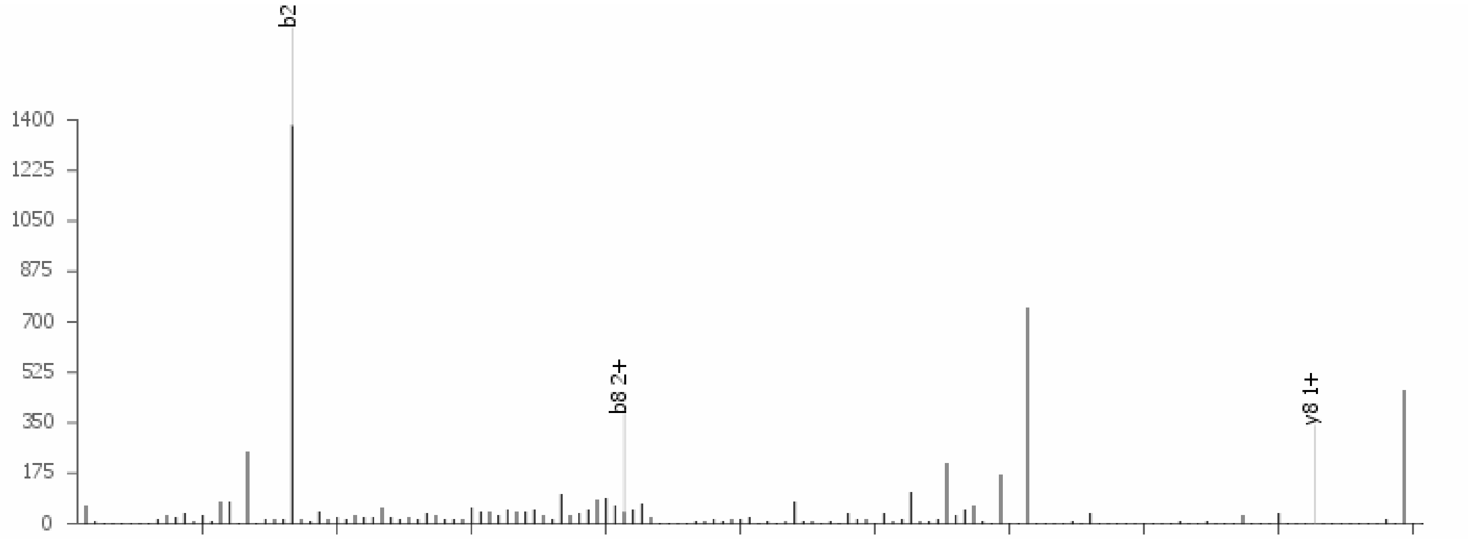

AT4G40020.1 - V(pS)ELLEEMK - 579.263 - Charge:2

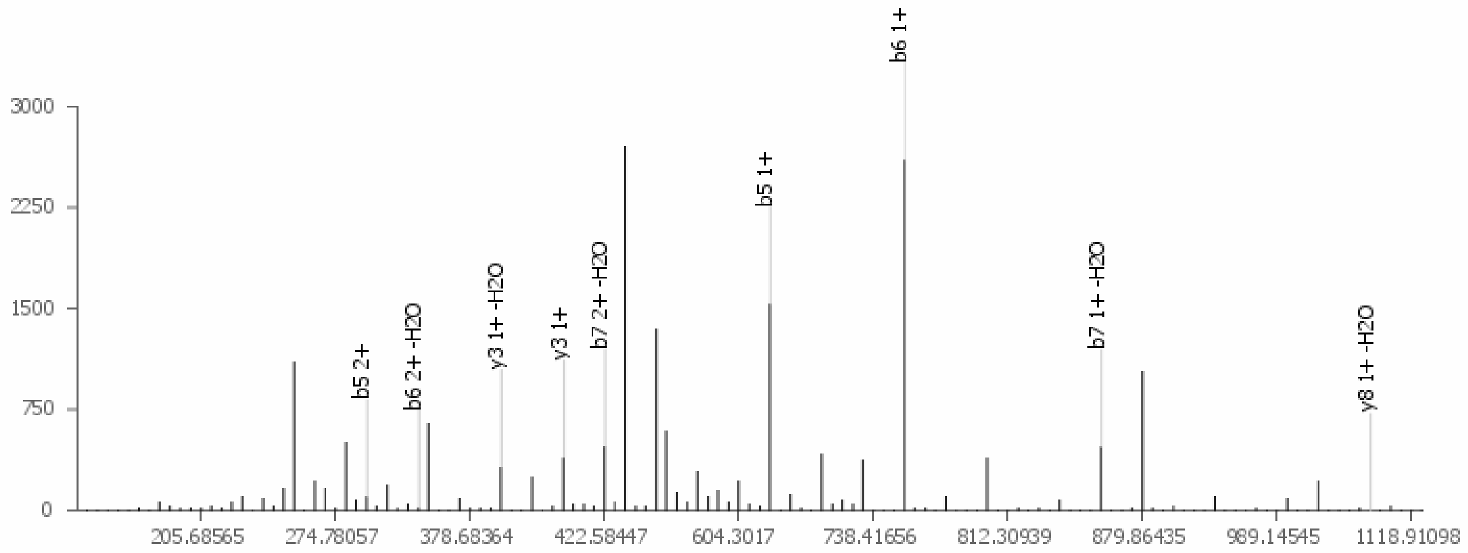

AT4G32230.1 - IRT(pT)ISPLMGL(pS)HELVR - 1042.017749 - Charge:2

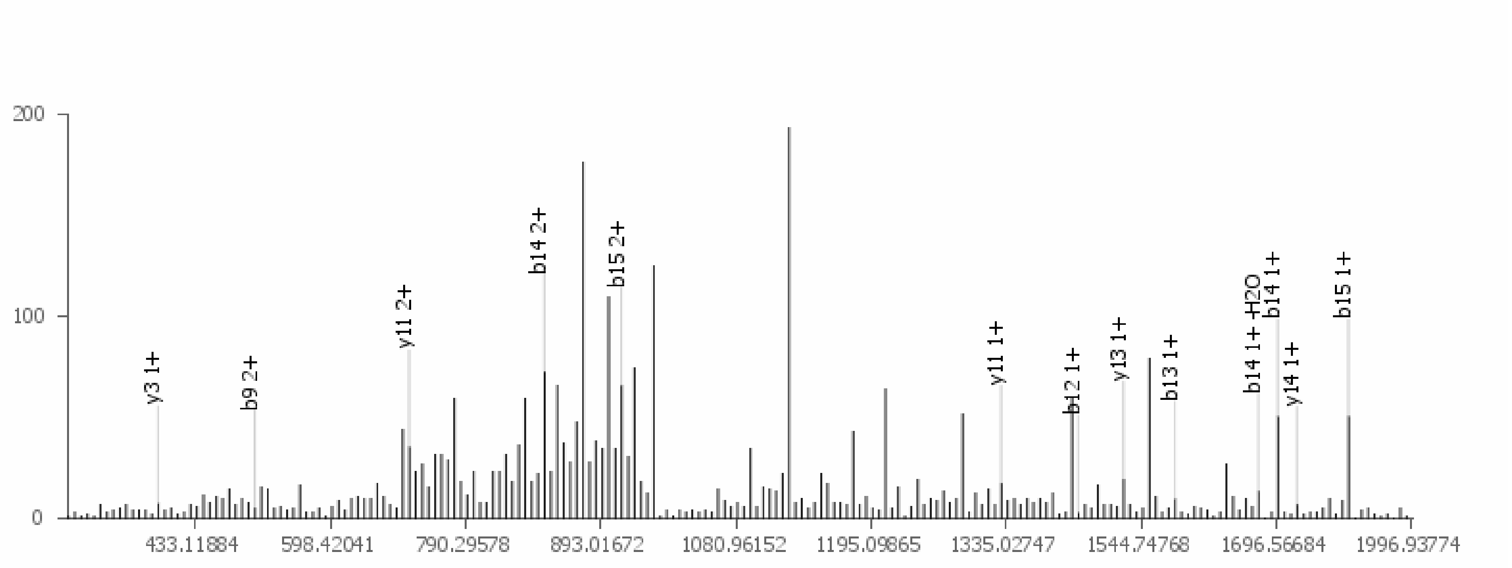

AT1G09795.1 - WSQLL(pS)NLGL - 605.792779 - Charge:2

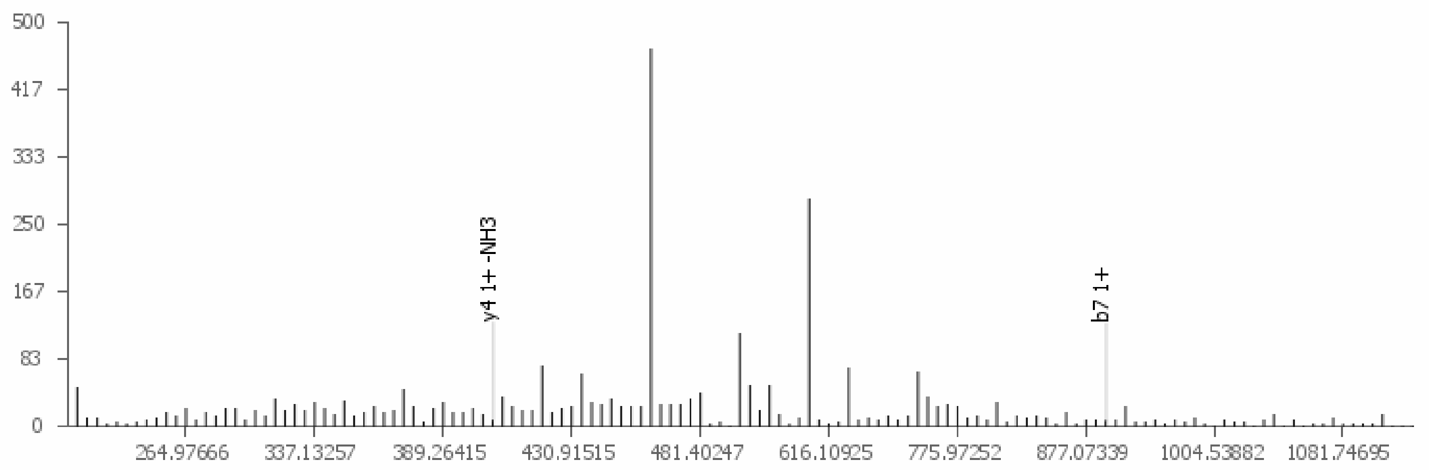

AT1G57990.1 - QTTAEGSANPEPDQIL(pS)PR - 1045.97967 - Charge:2

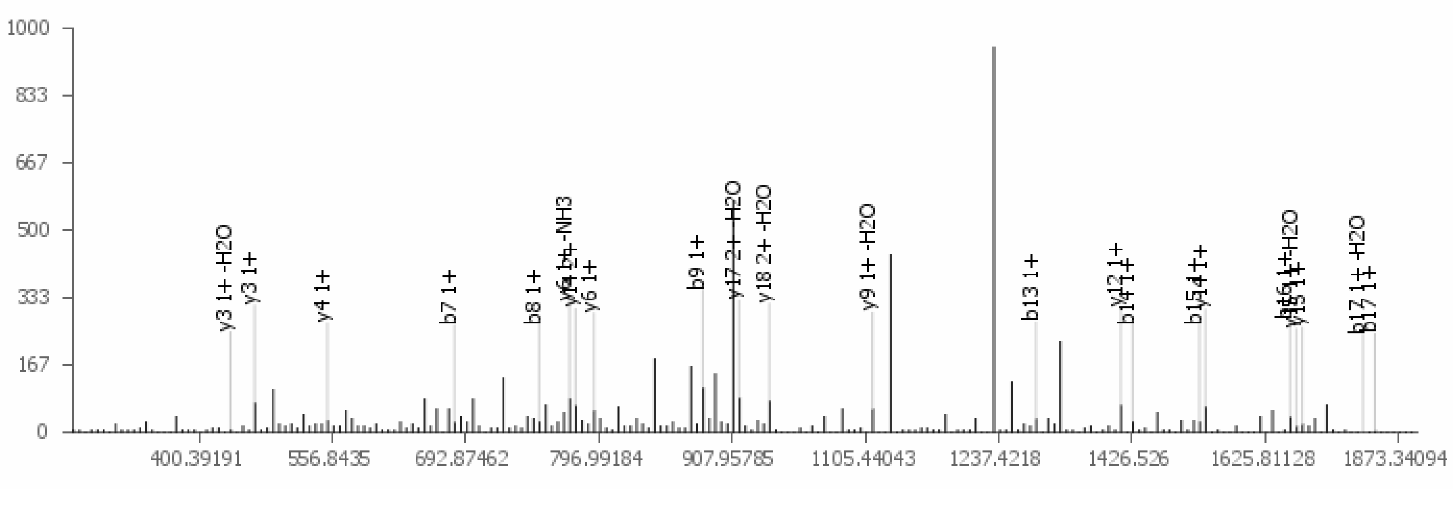

AT1G48830.1 - VEAF(s)AV(y)KK - 611.304541 - Charge:2

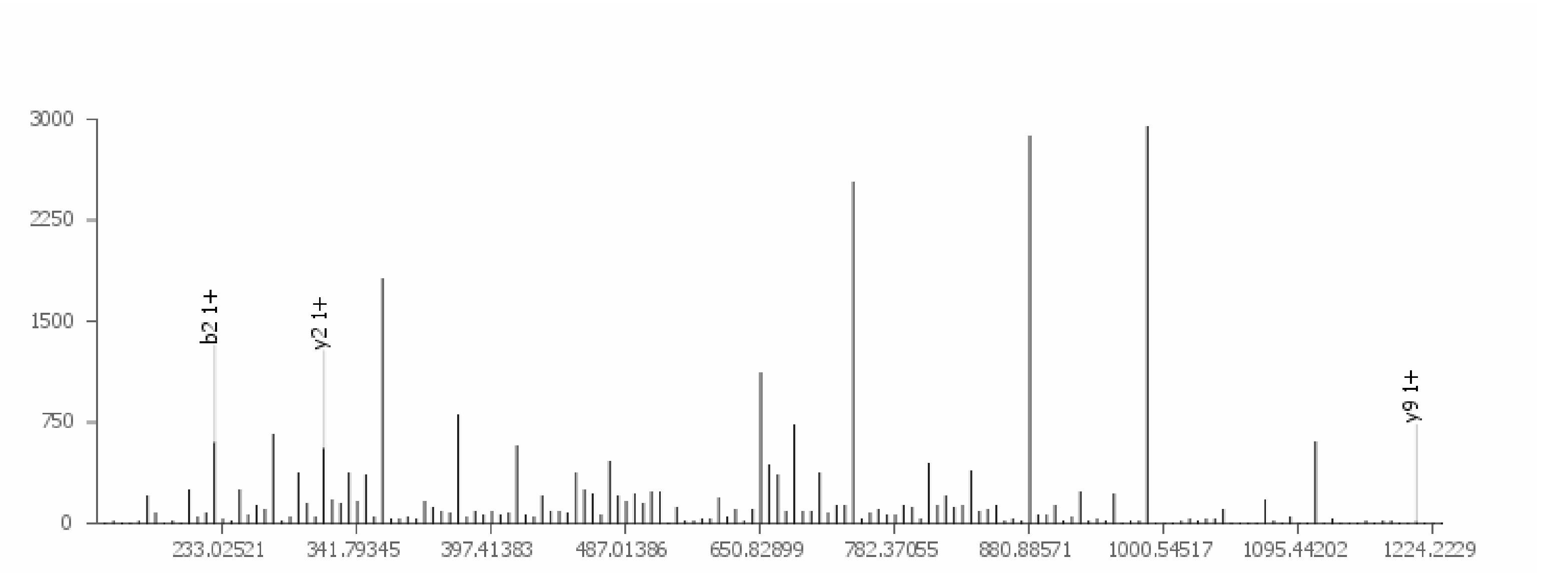

AT1G72350.1 - AAELSVLCGAQIGIITF(pS)R - 1015.019546 - Charge:2

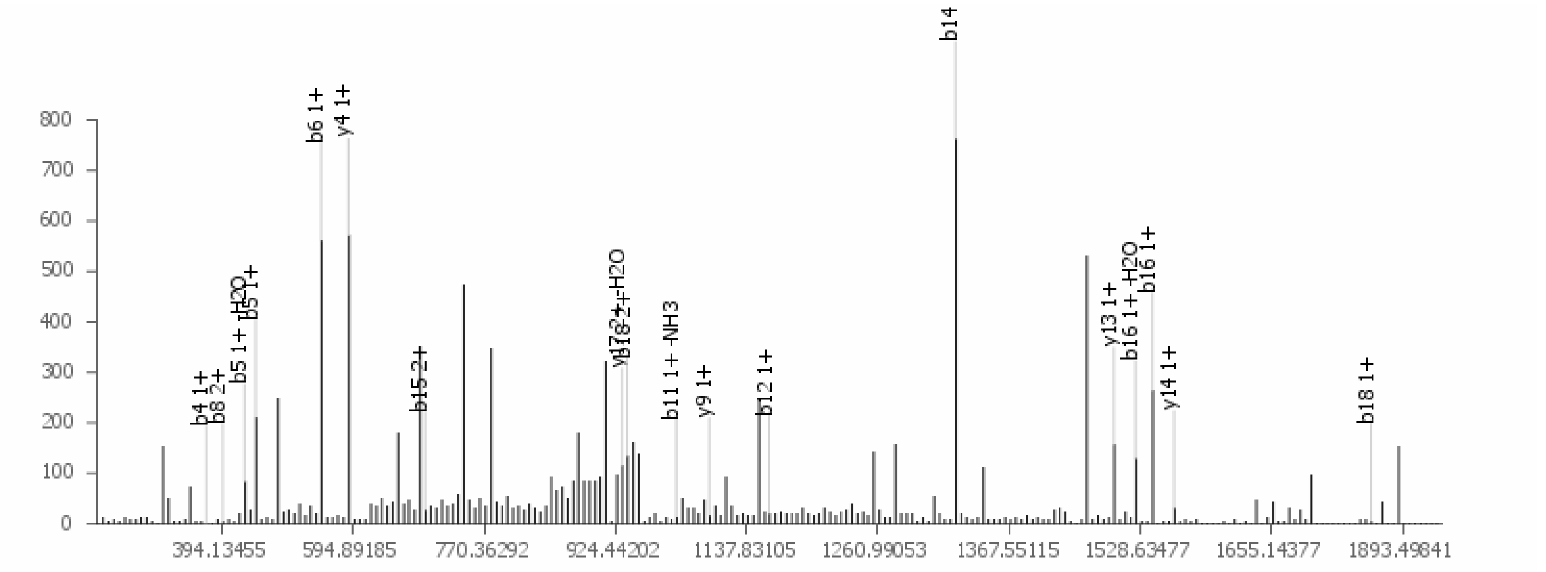

AT1G09180.1 - (pY)YLGLTNFTTGK - 769.325738 - Charge:2

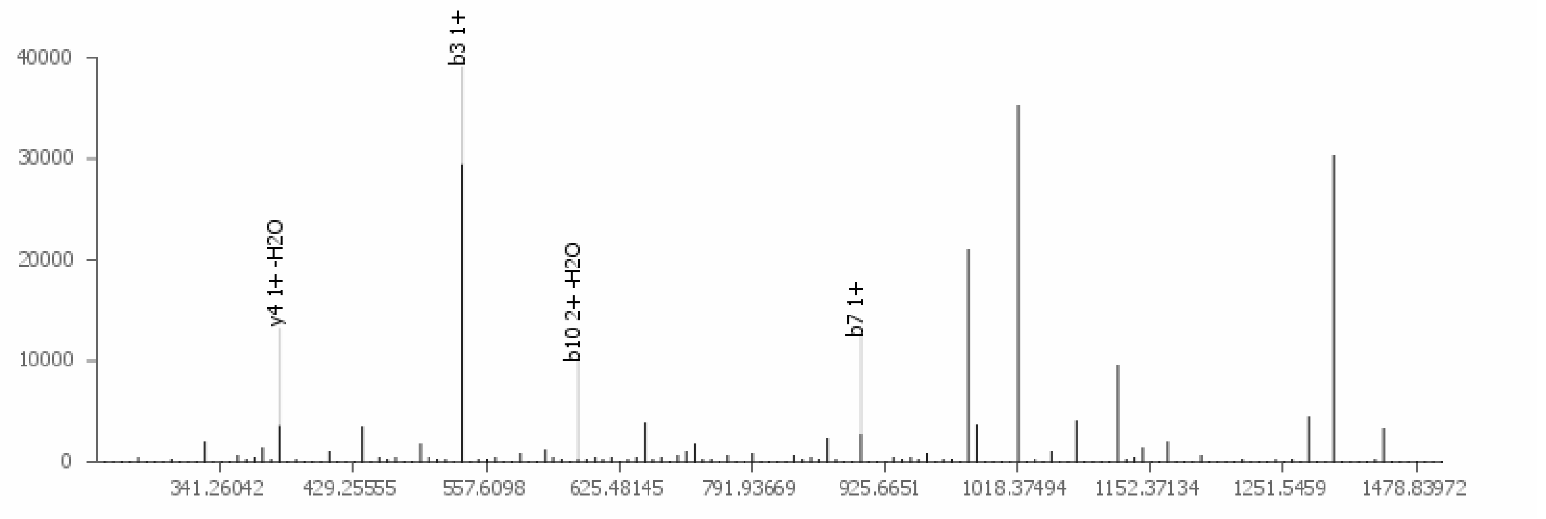

AT5G43280.1 - DLGLV(pS)KVFGSK - 665.344736 - Charge:2

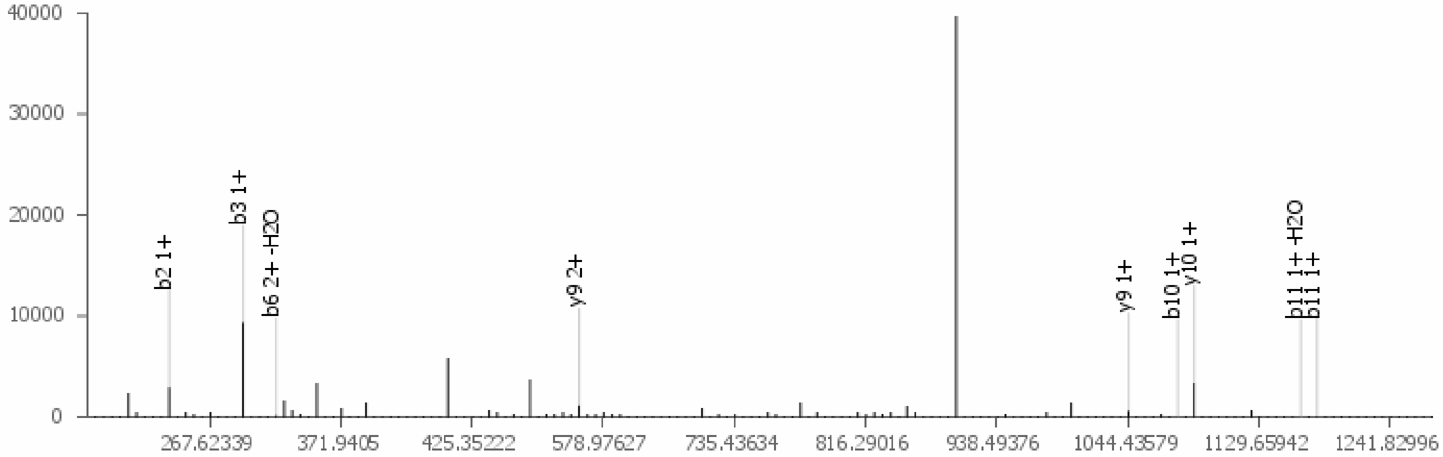

AT1G26270.1 - L(pS)M(s)LK(s)TLLGEK - 783.873817 - Charge:2

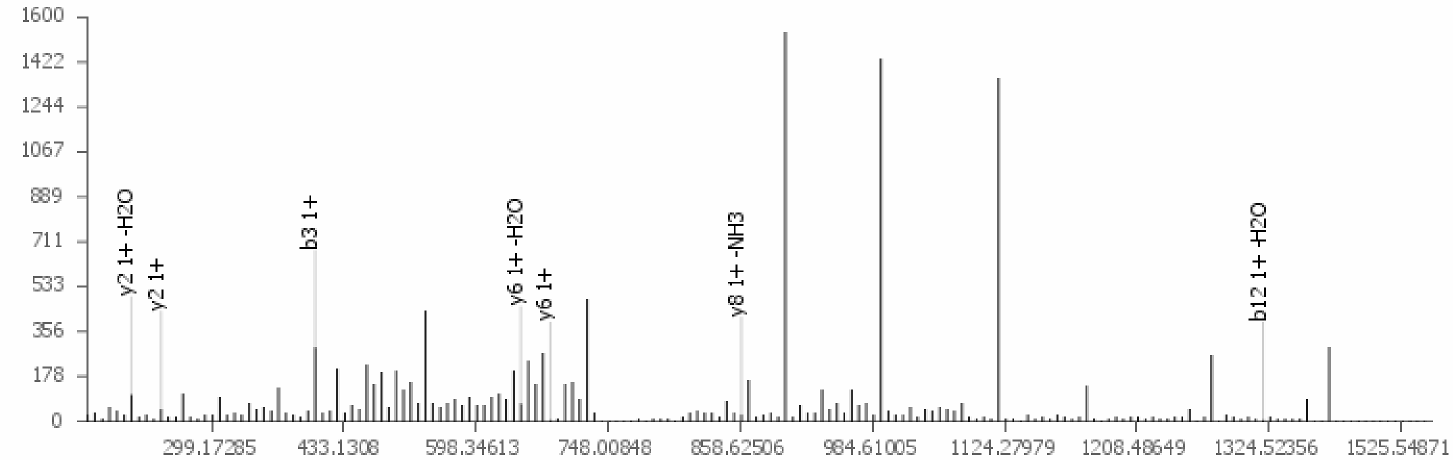

AT1G07000.1 - (oxM)AEAGDENL(pY)AAARDIAR - 1016.943215 - Charge:2

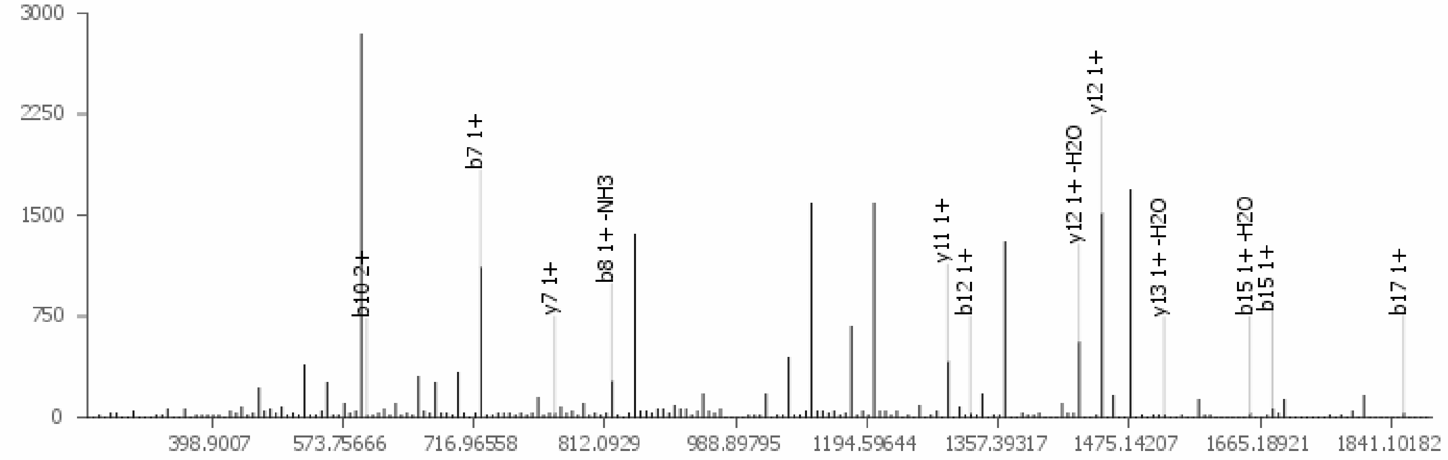

AT2G01190.1 - (pT)LPDQRNLGIEEQFAR - 983.978637 - Charge:2

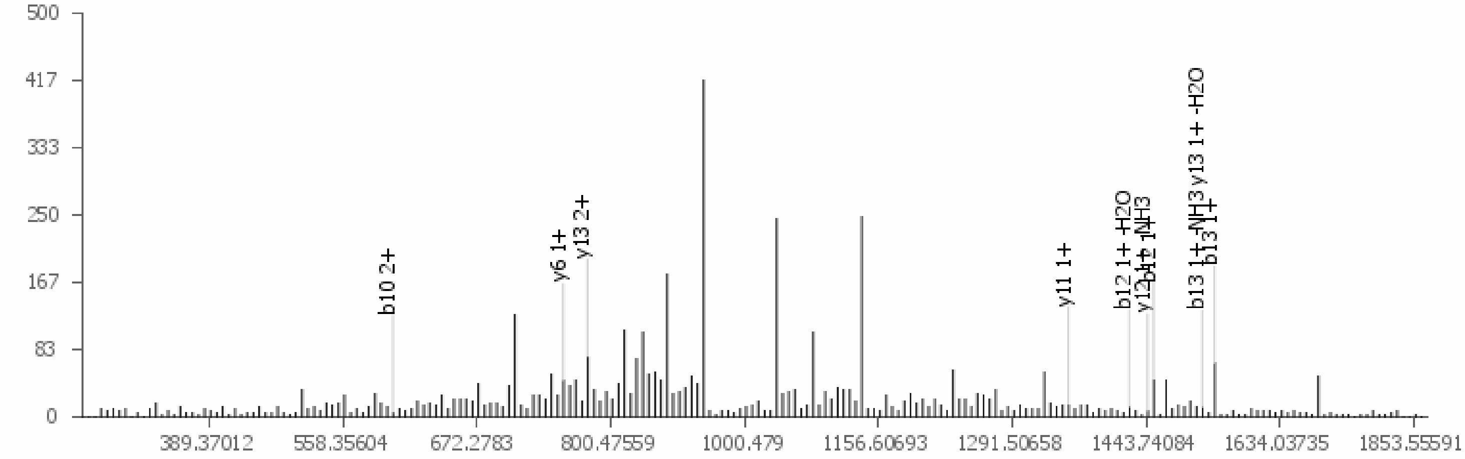

AT5G45190.1 - SRNVDVGDALI(pS)Q(pS)PK - 923.412112 - Charge:2

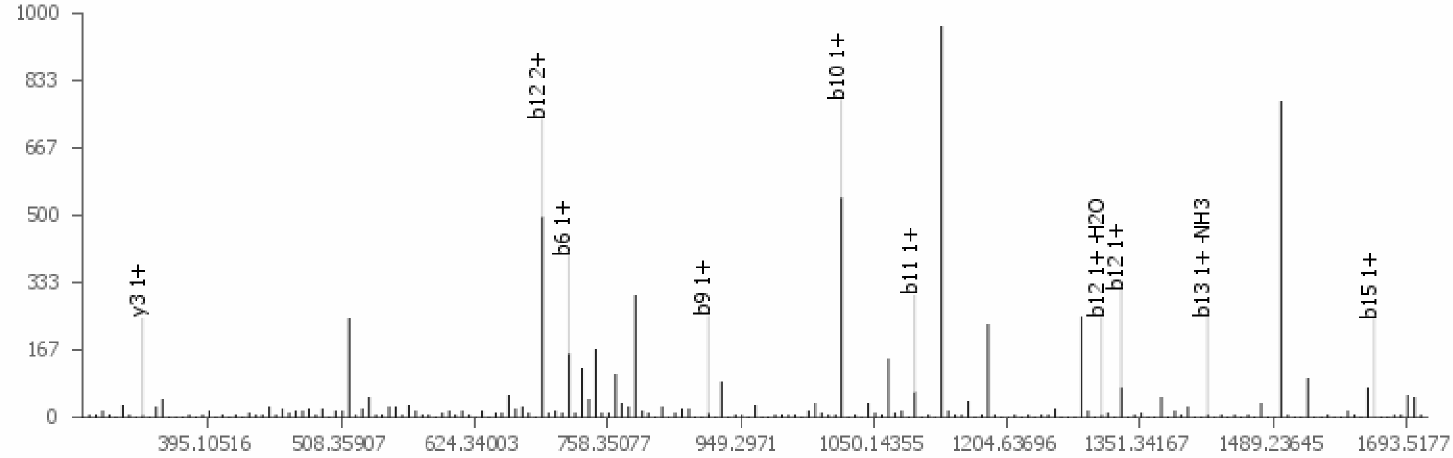

AT3G26240.1 - Y(pS)KPNMVDILNLFK - 881.440005 - Charge:2

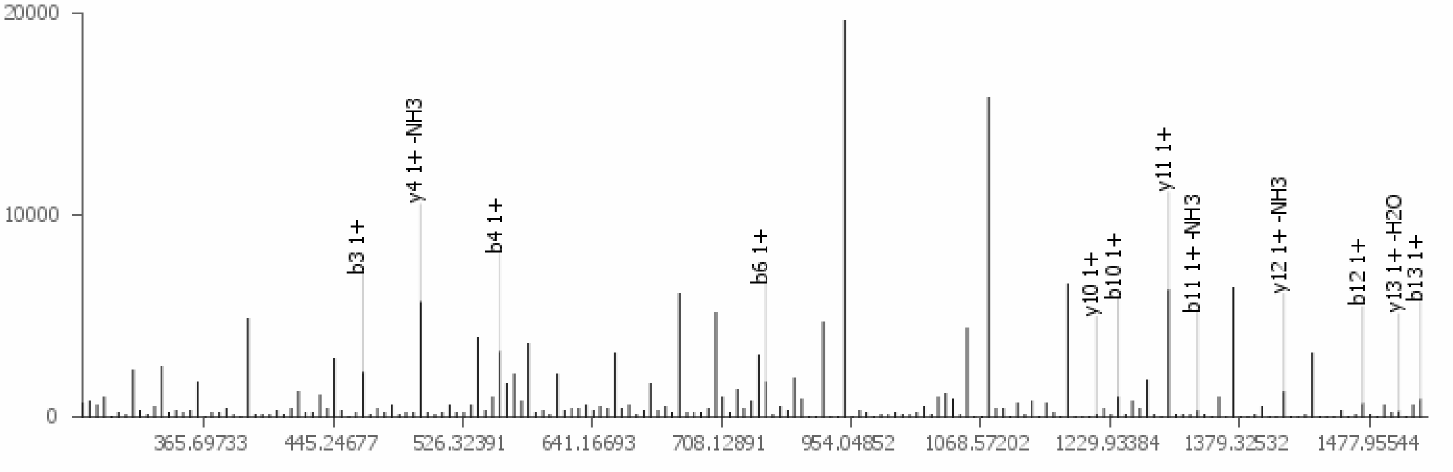

AT3G55650.1 - (pT)LG(oxM)V(pS)LPL(pS)PIESLAASVV(s)(t)AQ(s)VFASAIVVLTK - 981.47722

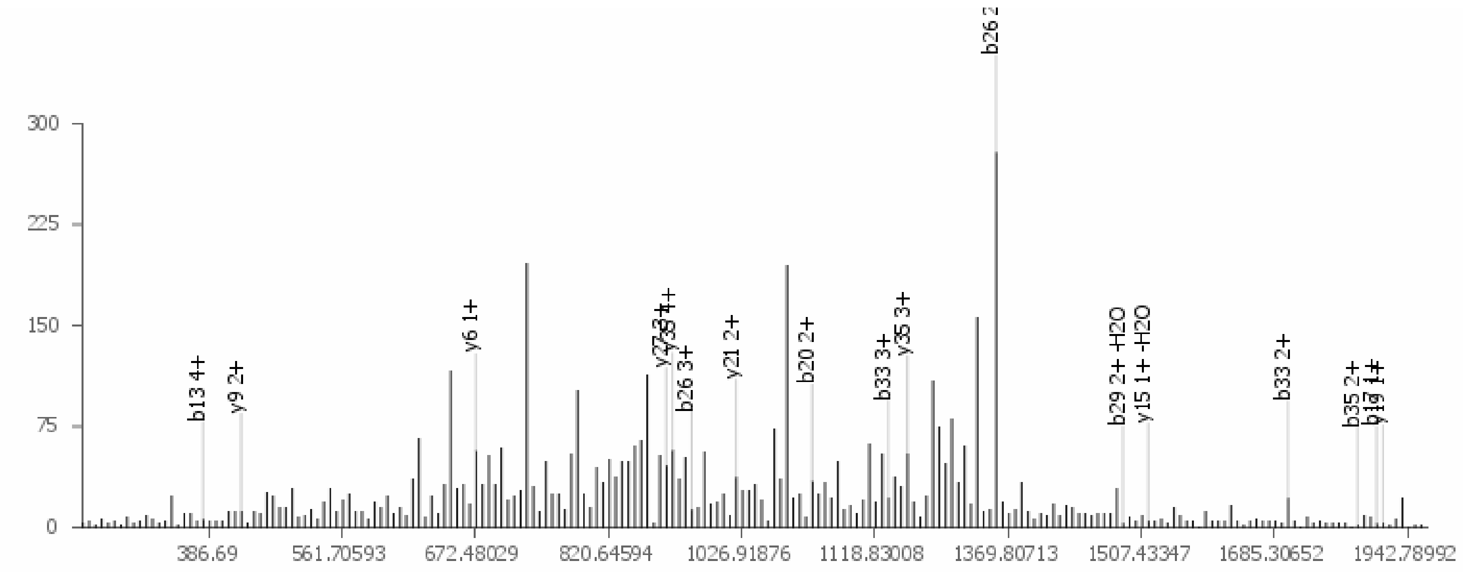

AT3G59170.1 - (pT)(oxM)PRLEQLVFYYDVK - 999.482798 - Charge:2

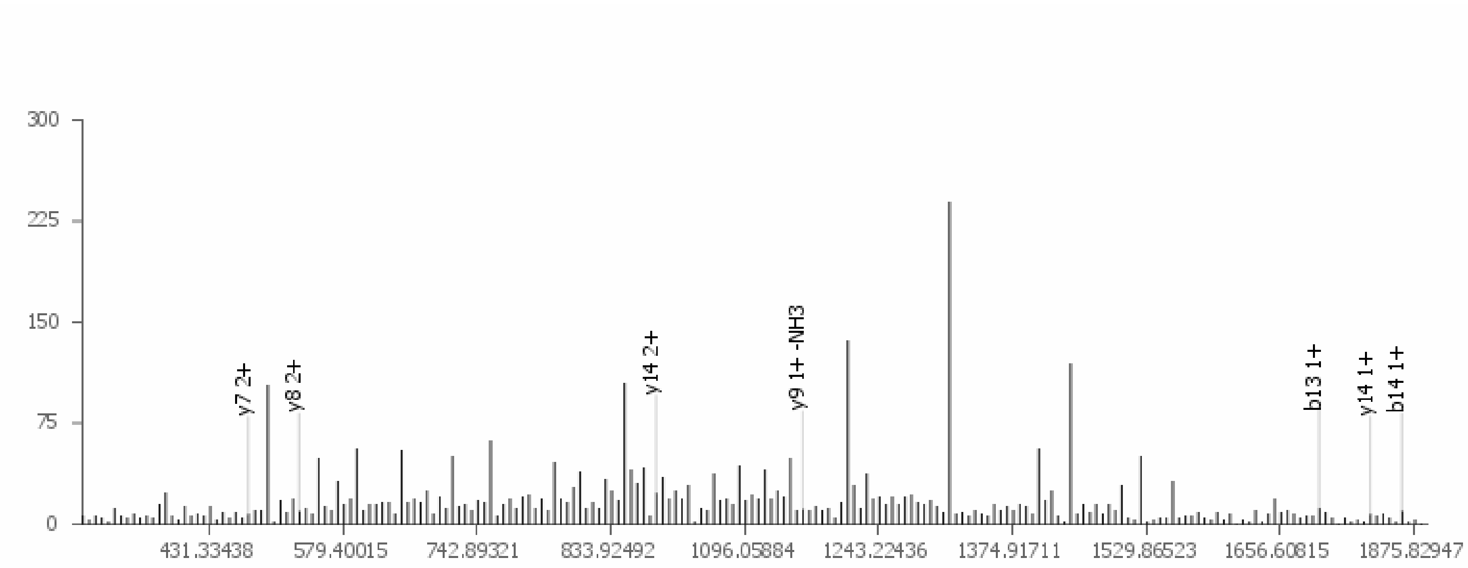

AT5G42030.1 - QTLQF(s)E(t)LK - 637.8111 - Charge:2

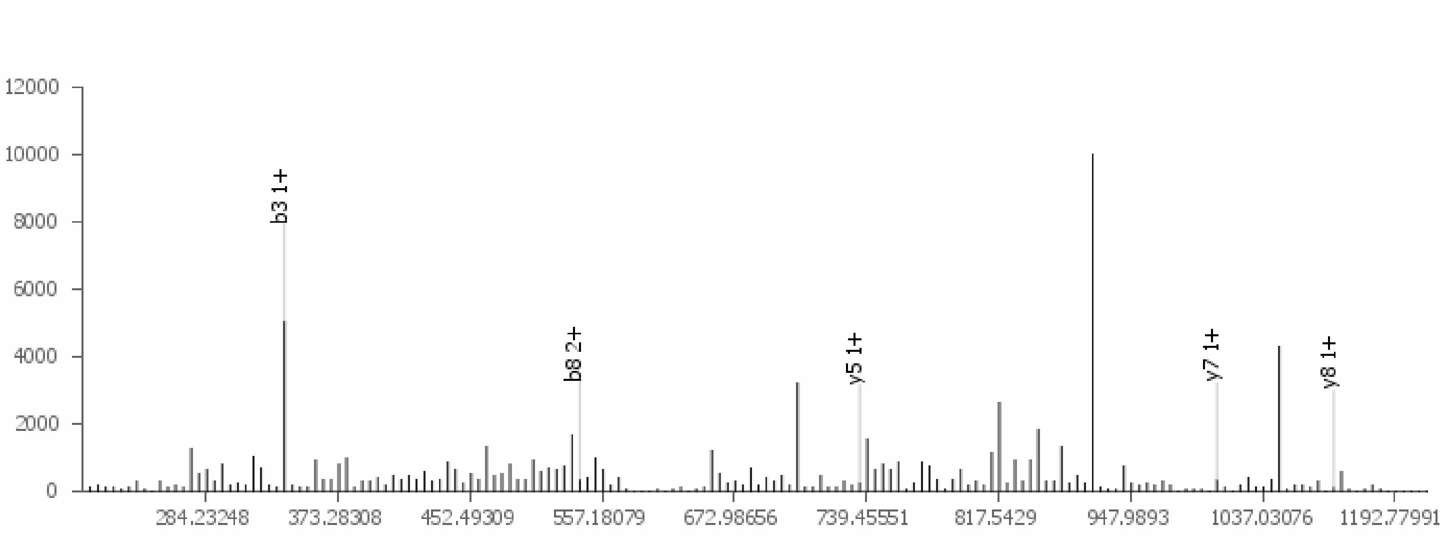

AT3G16710.1 - RSIATGFA(pS)IVK - 665.343512 - Charge:2

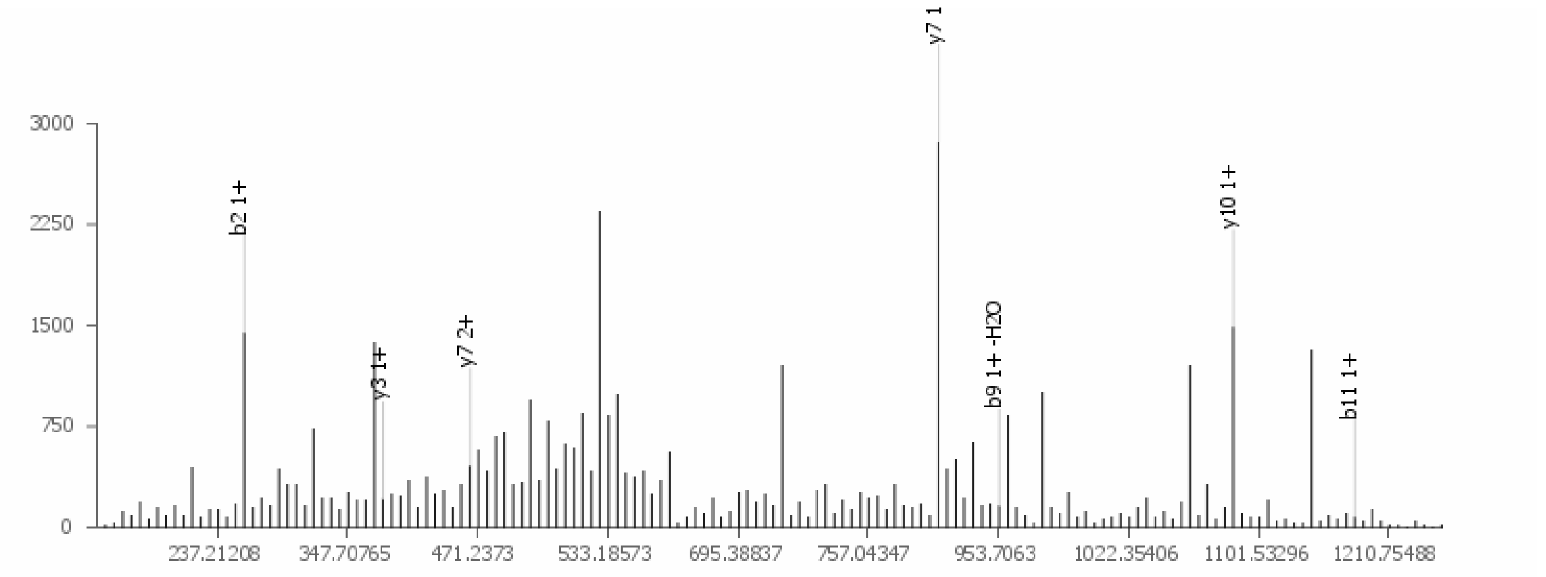

AT3G60190.1 - LDLMDKG(pT)NALEVLEGR - 977.471753 - Charge:2

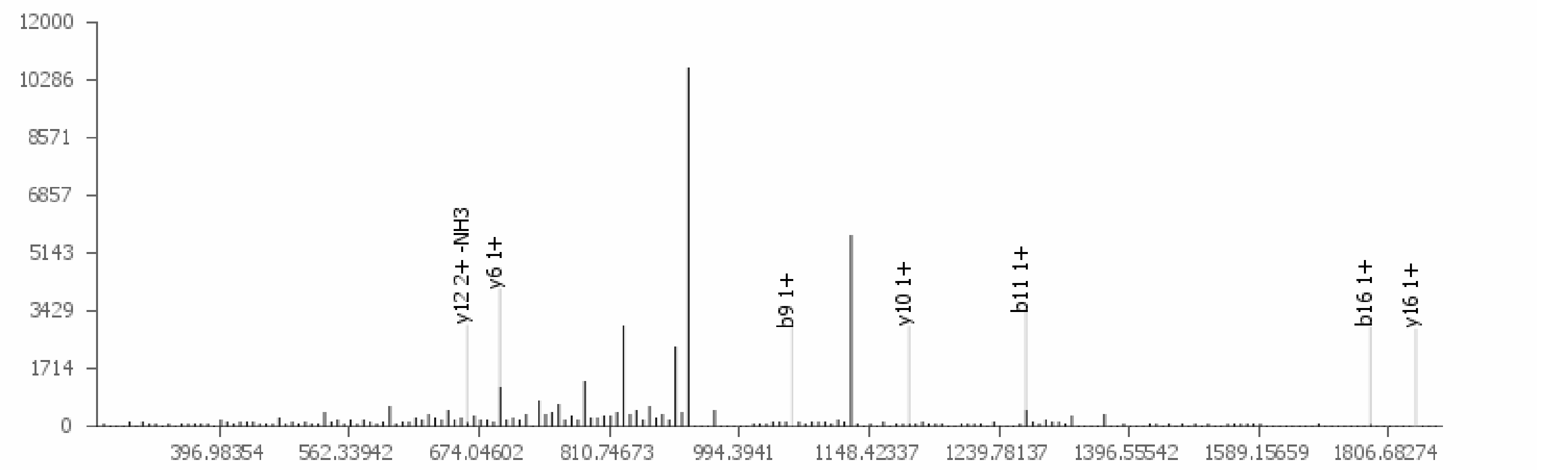

AT1G27880.1 - MKDLLILME(pS)PPYK - 879.441299 - Charge:2

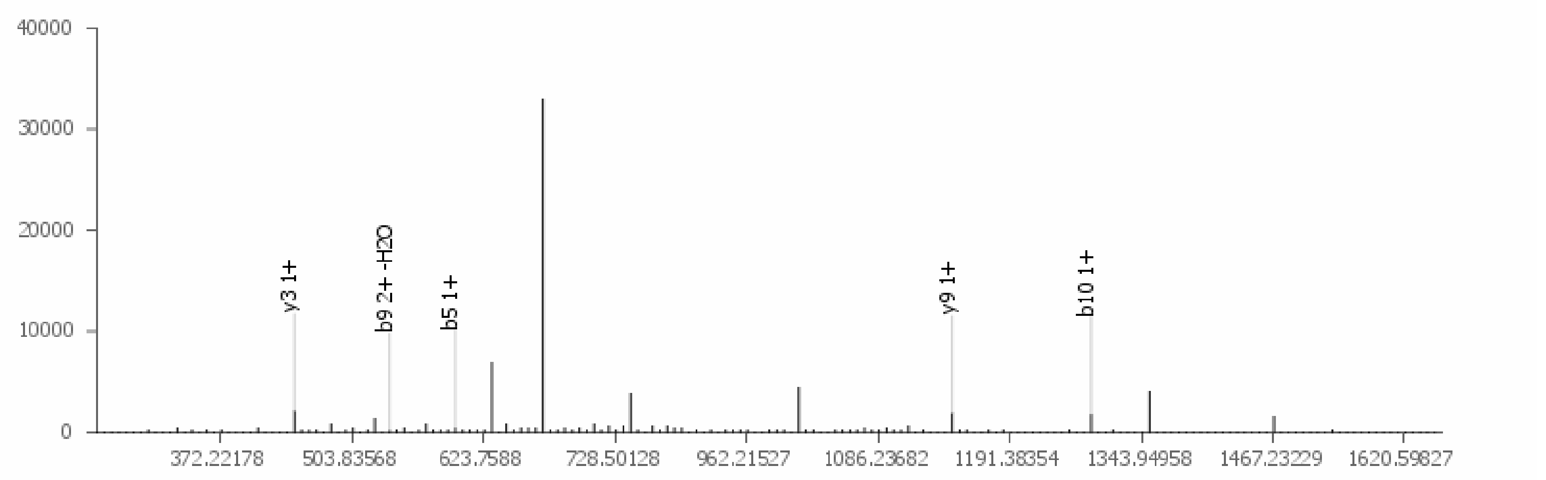

AT5G61910.1 - YL(pT)IL(pS)NIRR - 704.840642 - Charge:2

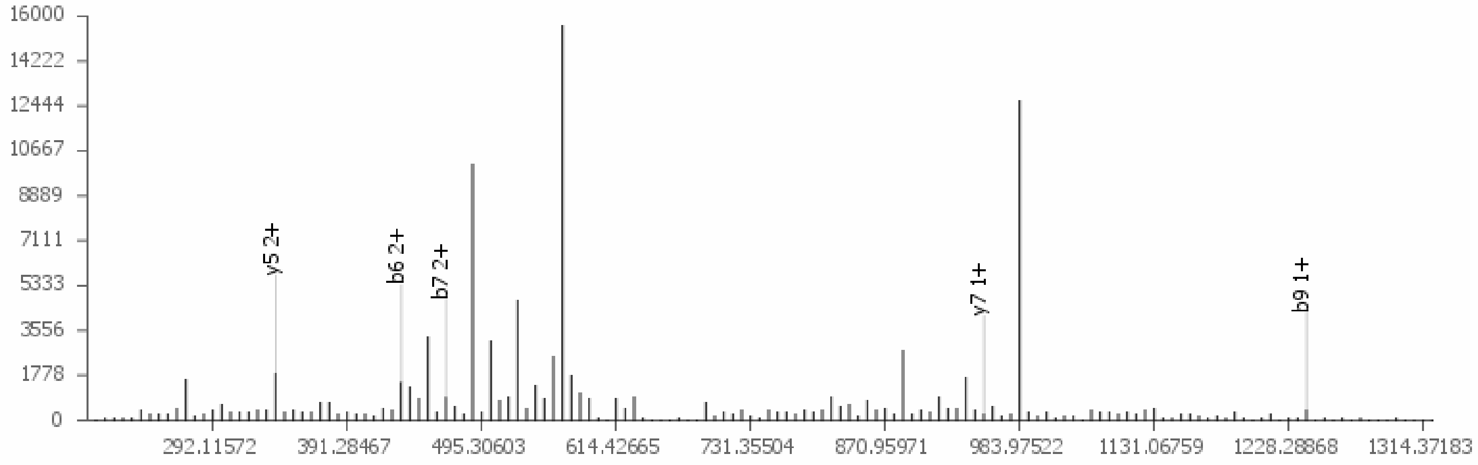

AT1G74070.1 - FSSIVSGKAGI(pT)YR - 783.384727 - Charge:2

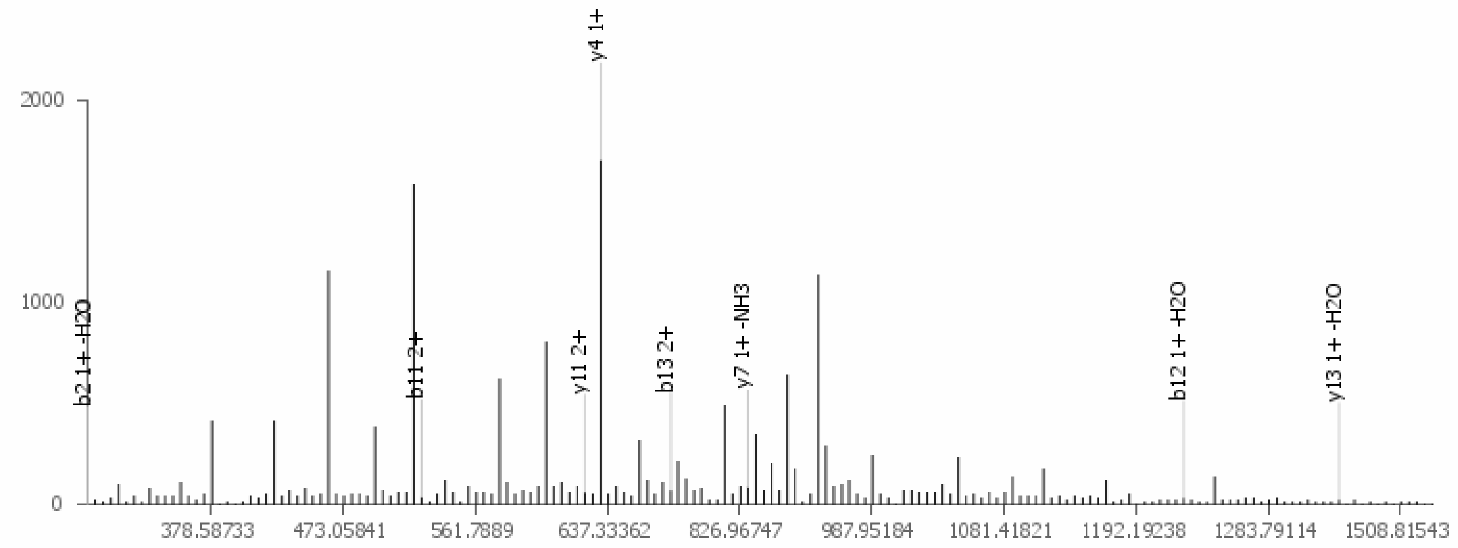

AT2G38940.1 - VYG(oxM)(t)L(m)VMVLC(s)IASGLSFGHEPK - 928.10249 - Charge:3

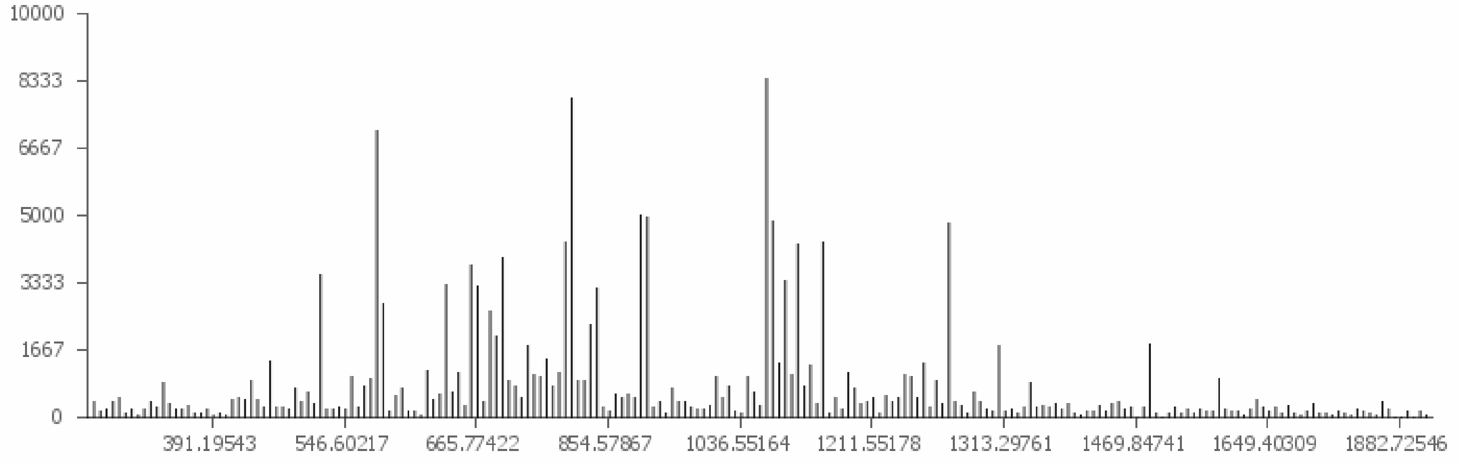

AT1G72860.1 - RAT(pT)LLDDGVLQGCLGLK - 977.004358 - Charge:2

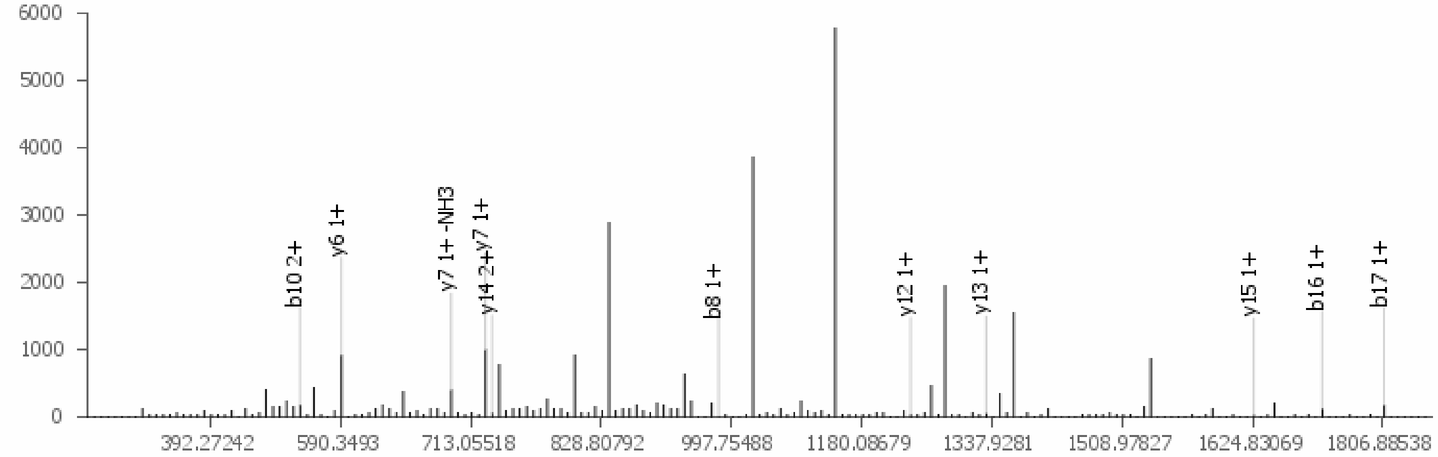

AT2G32540.1 - KYLAEE(s)L(t)R - 645.314222 - Charge:2

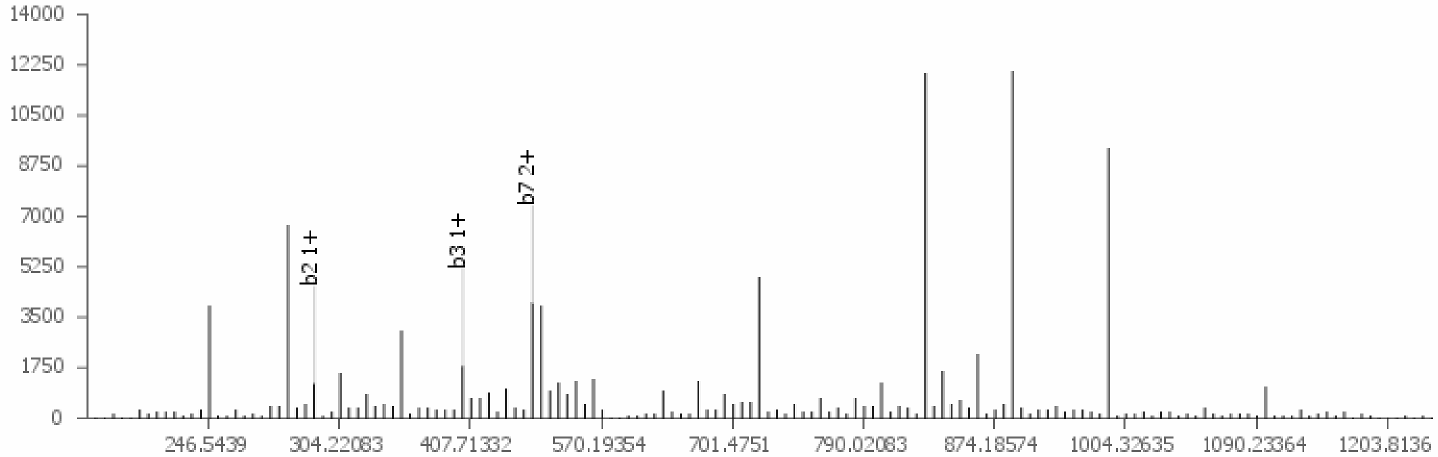

AT5G24230.1 - HVIAFRG(pT)ILKPH(pS)R - 946.469086 - Charge:2

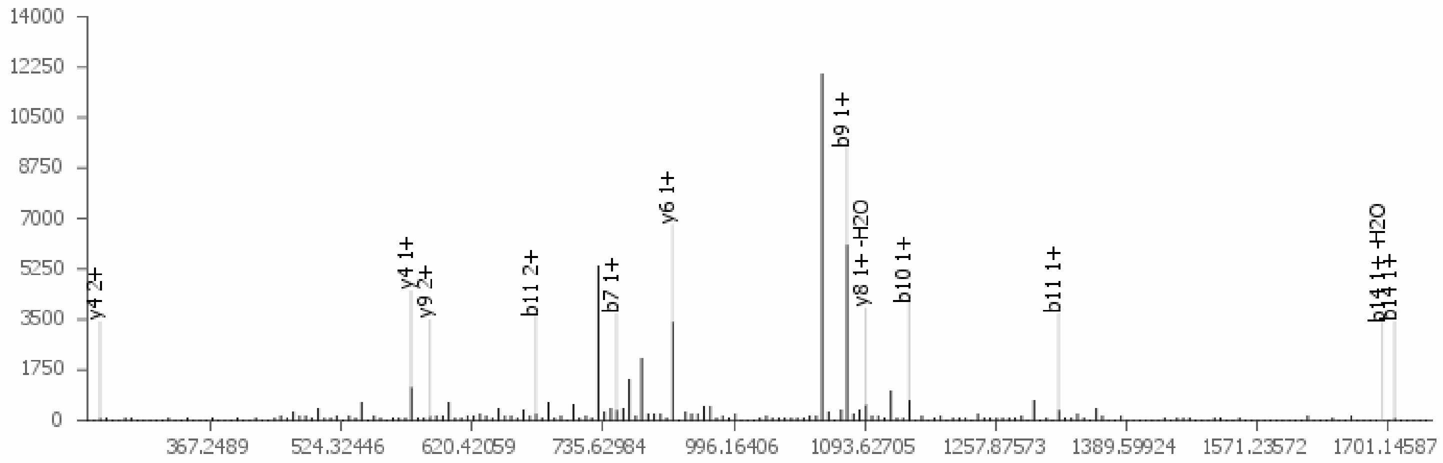

AT3G13340.1 - TYG(pS)LLEFGR - 611.783389 - Charge:2

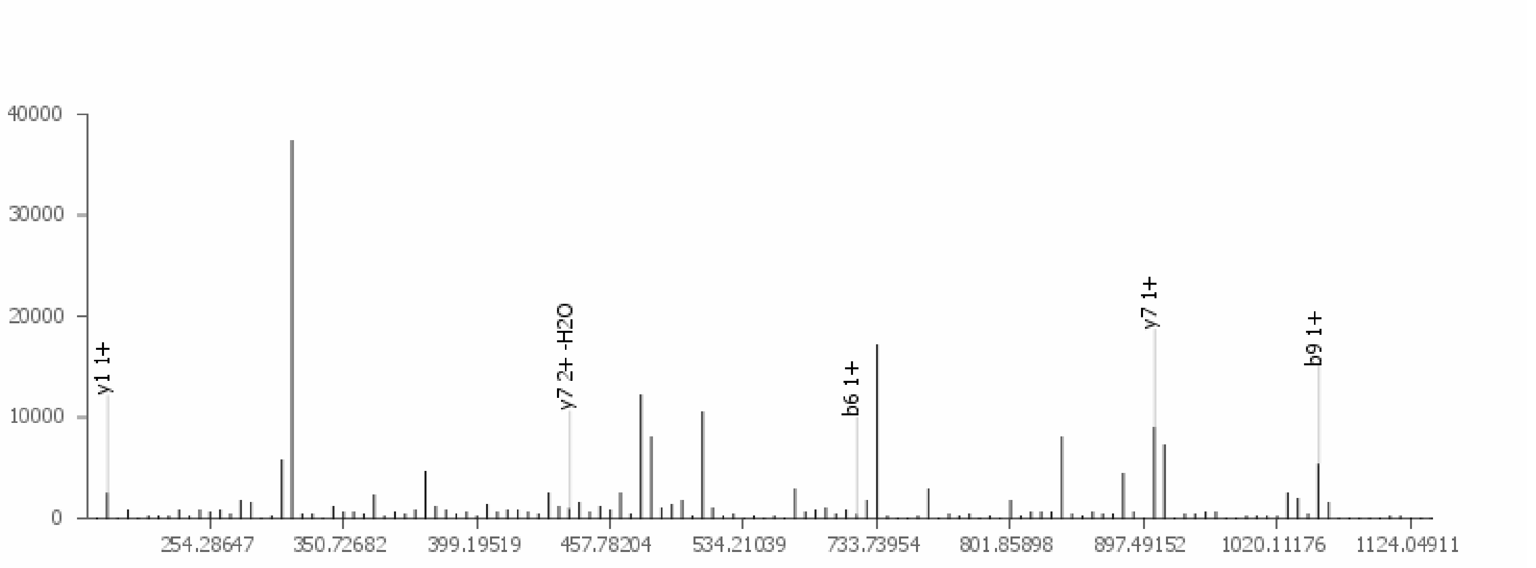

AT2G23770.1 - AKIG(pS)LGSAR - 1039.529214 - Charge:1

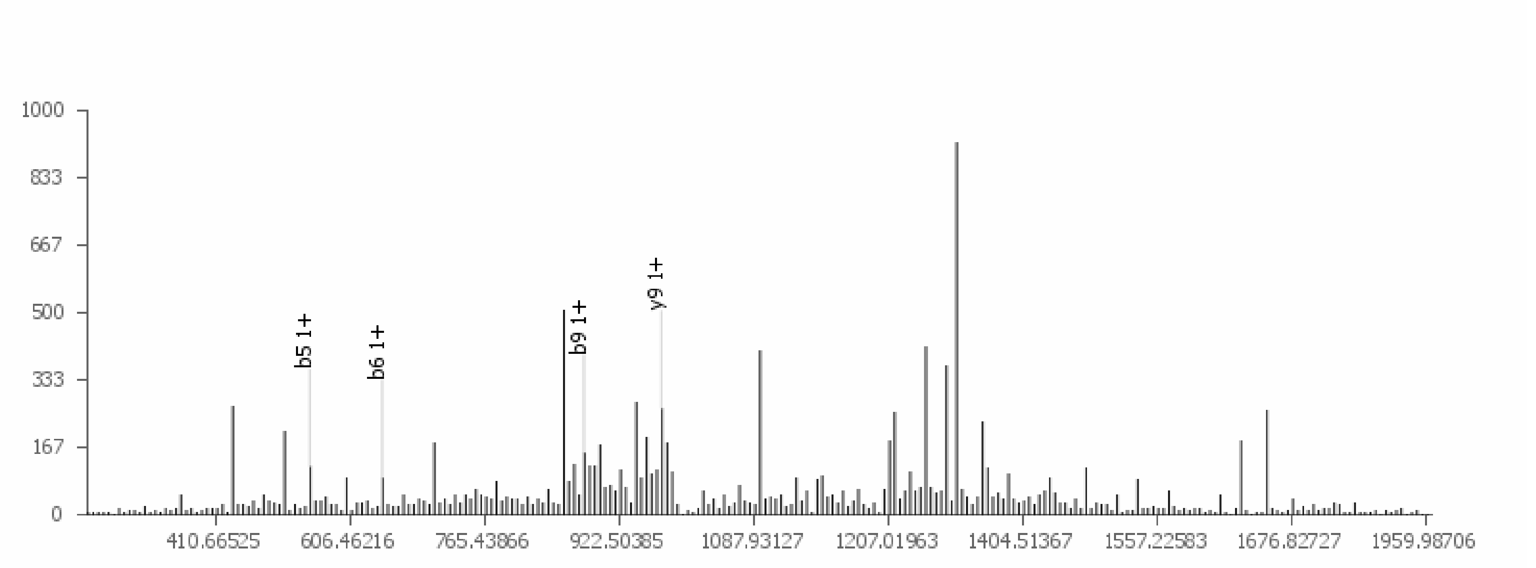

AT2G01410.1 - VFL(s)PLP(s)LPGDDEDIAR - 1010.994069 - Charge:2

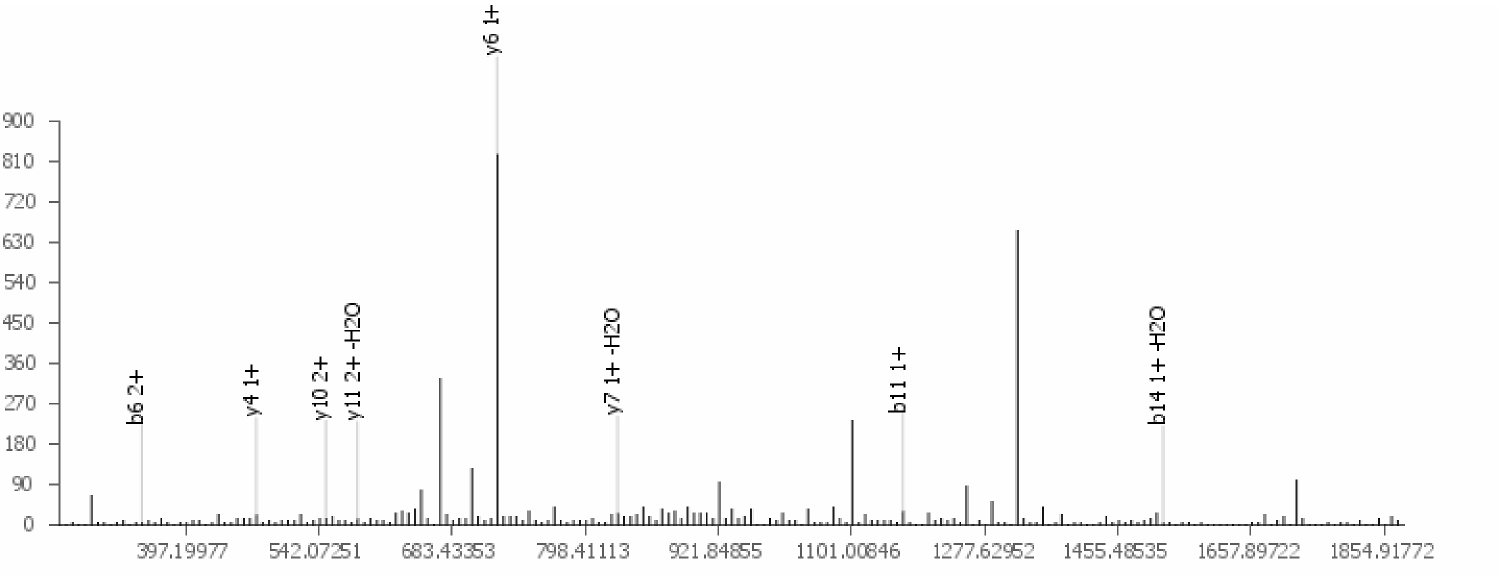

AT2G32480.1 - I(pT)KVRPR - 475.272245 - Charge:2

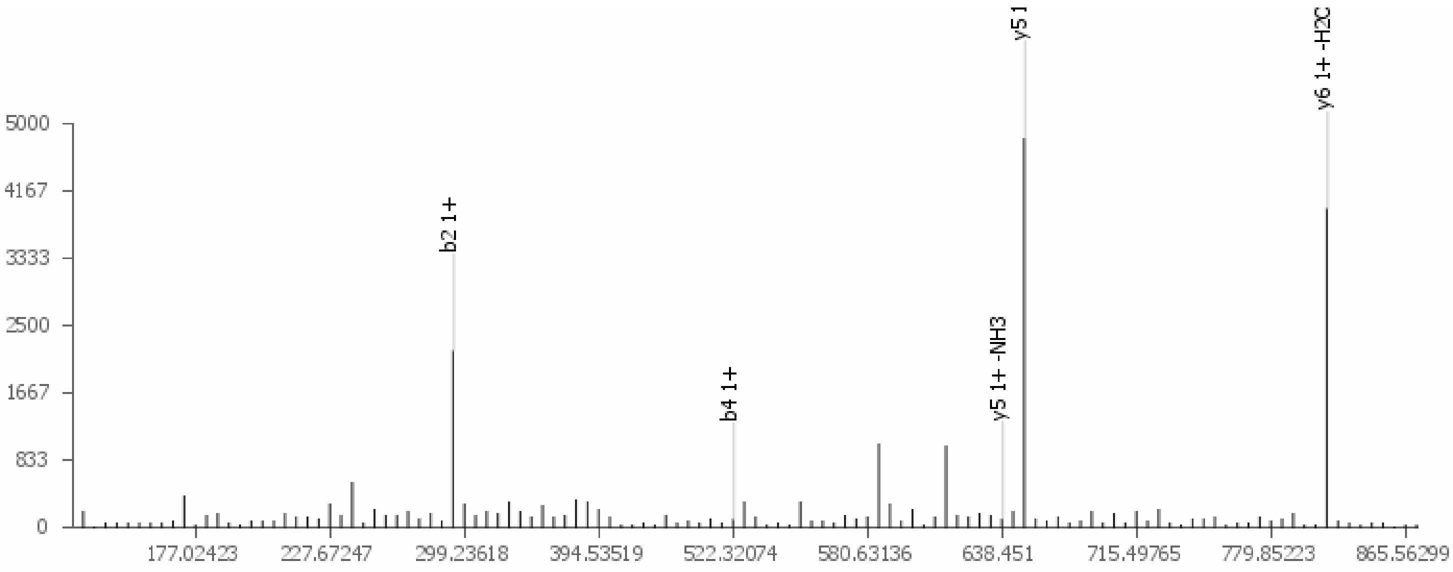

AT1G61010.1 - LLLTD(pY)VK - 522.769686 - Charge:2

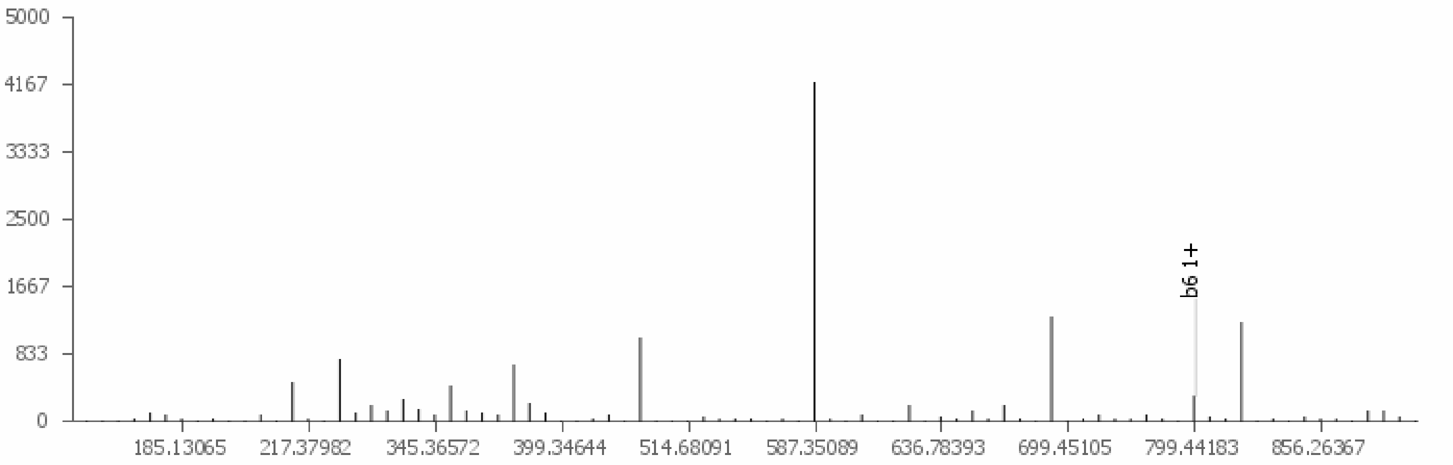

AT3G46660.1 - RSVVLVPFPAQGHI(pS)PMMQLAK - 829.426586 - Charge:3

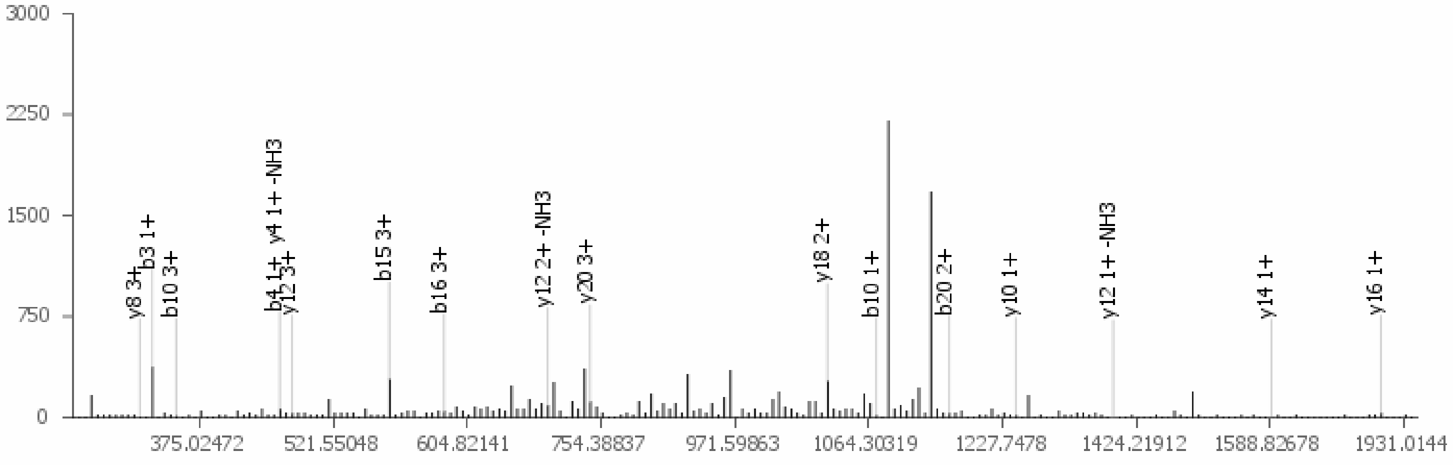

AT1G72880.1 - TSKNNGL(pS)AALVSNLQDVLSK - 747.055913 - Charge:3

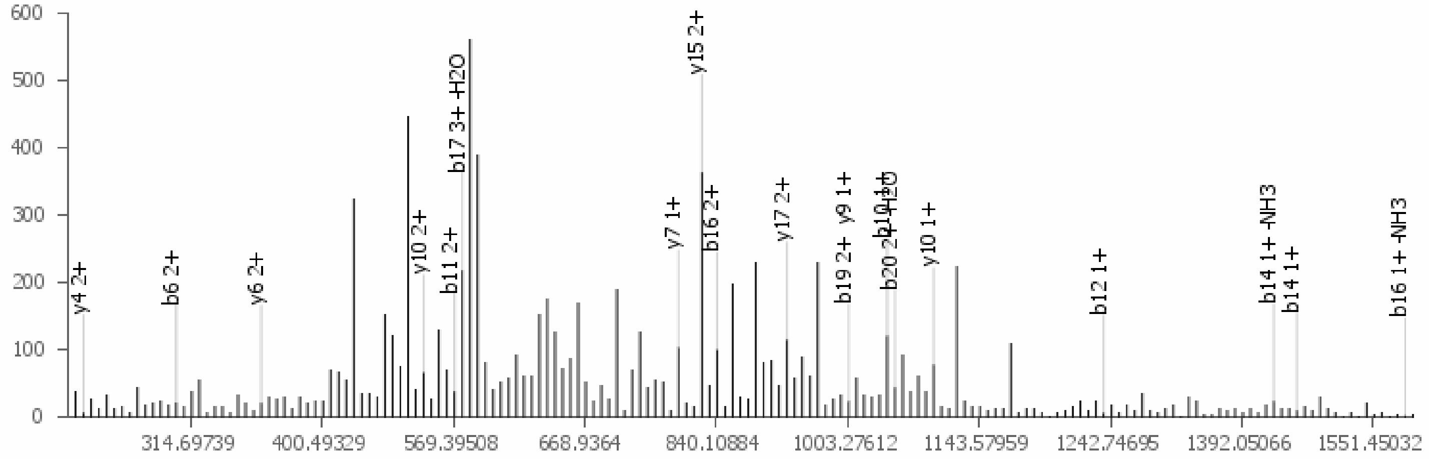

AT4G14180.1 - ELMQAELLN(pS)QQVTKLDR - 732.702265 - Charge:3

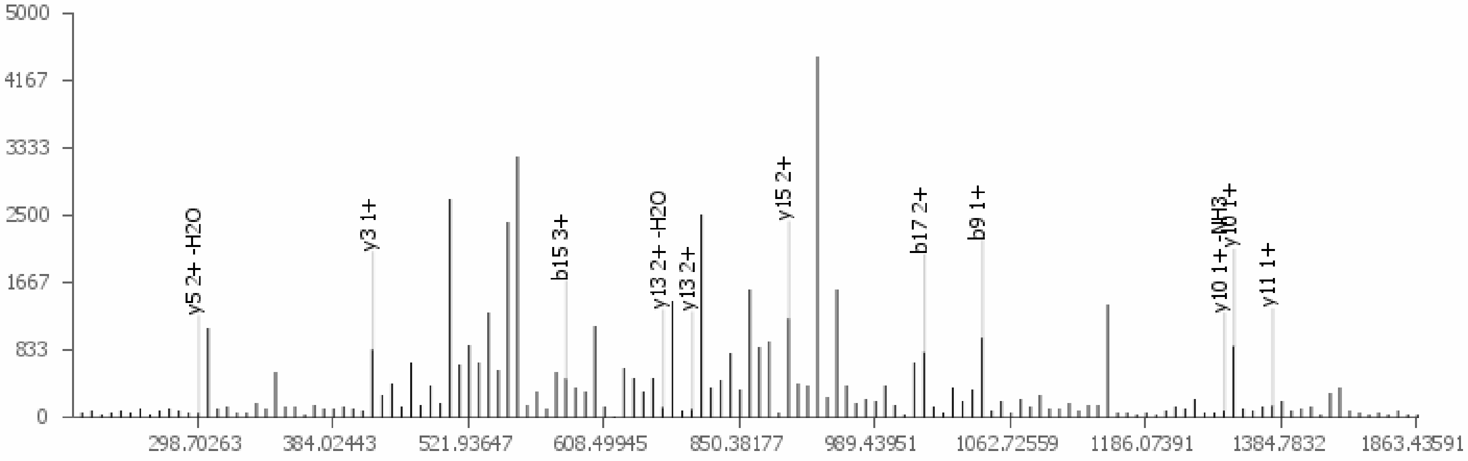

AT1G73850.1 - M(pS)IEEEMEILMGLIDLK - 1037.472435 - Charge:2

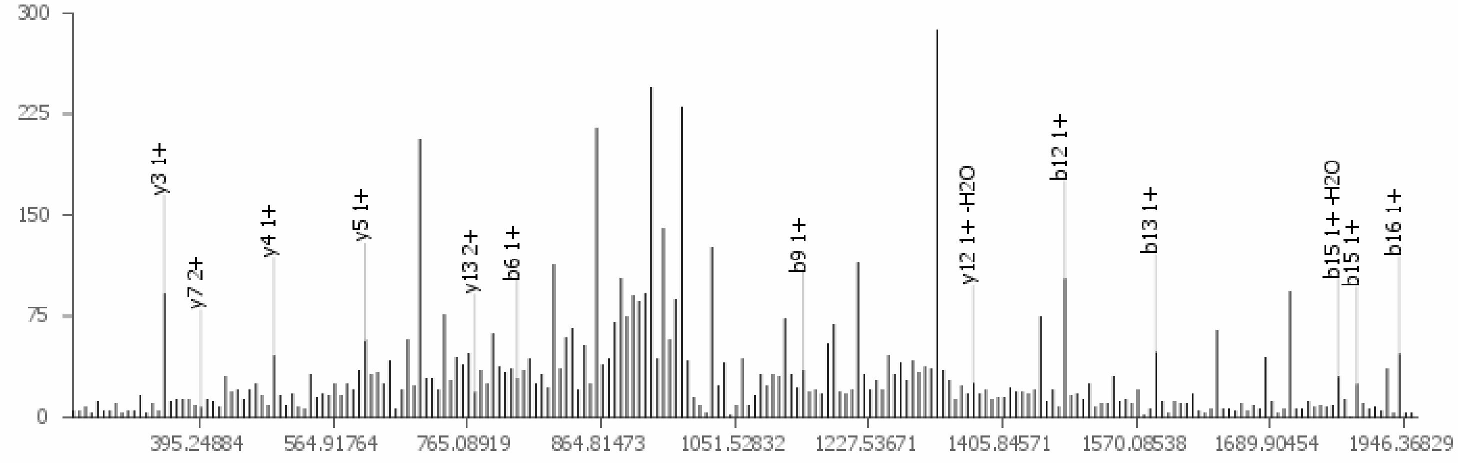

AT1G56230.1 - ITEVRVGSWSYVEG(pS)IGK - 1023.991662 - Charge:2

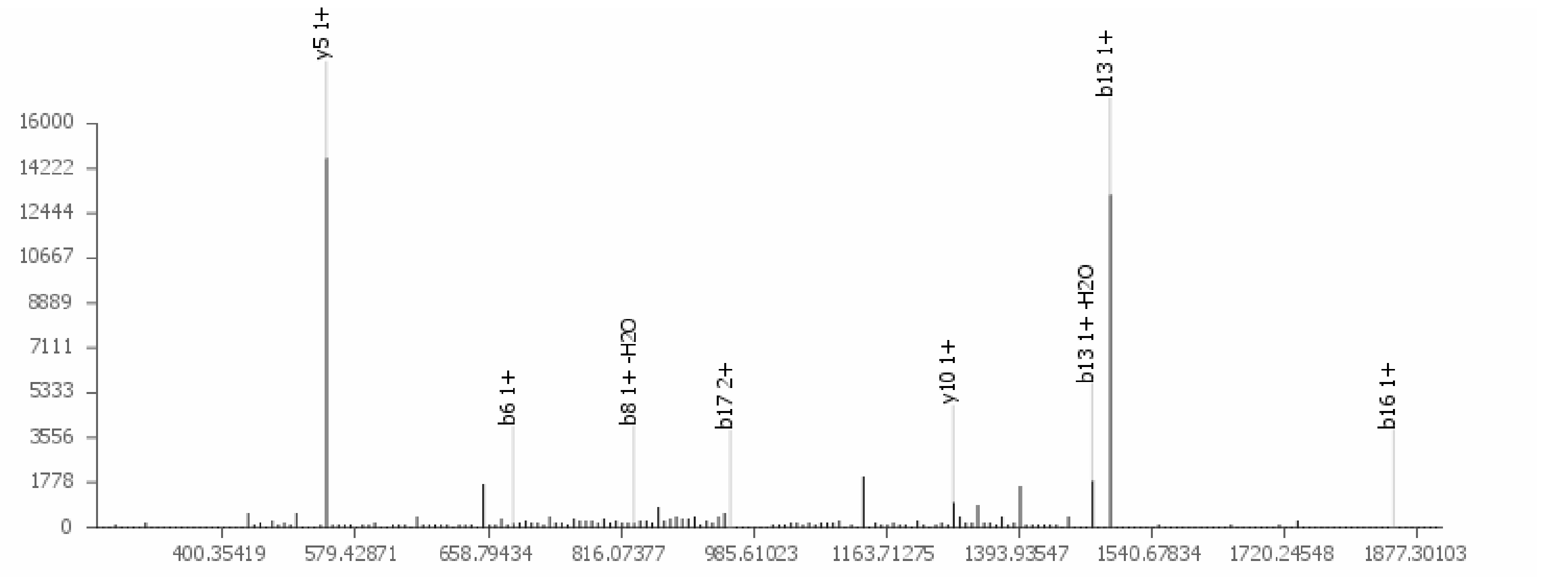

AT3G19508.1 - (pY)SIDESAATKLK - 703.327215 - Charge:2

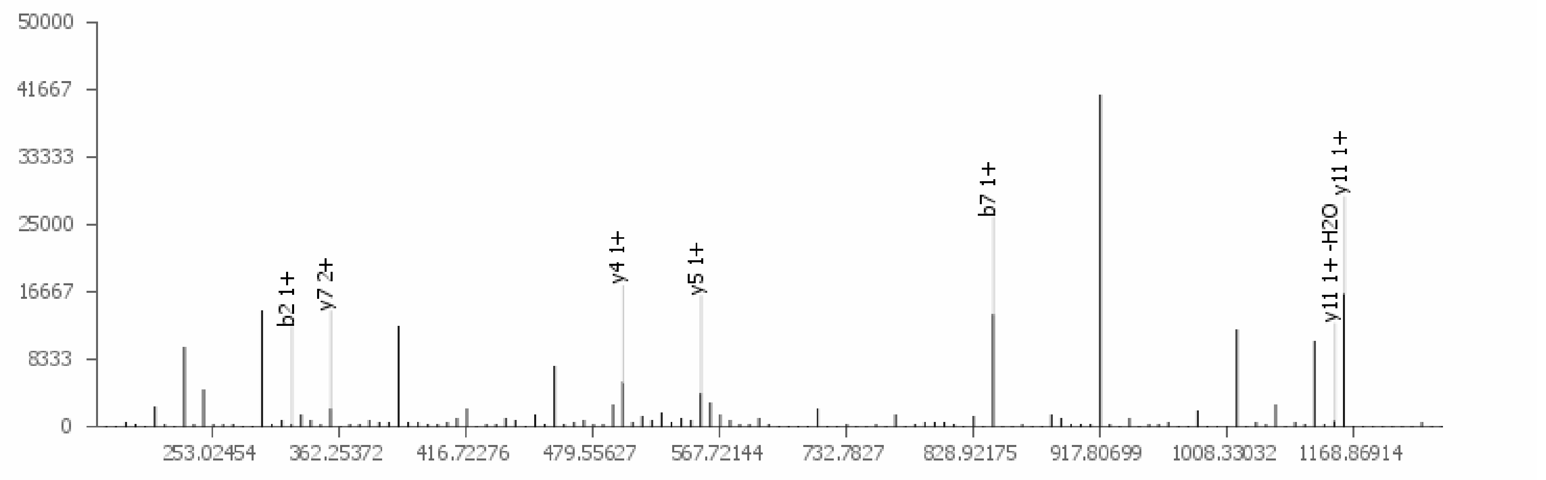

AT5G53700.1 - LKP(t)G(s)GHPTIGVER - 814.907801 - Charge:2

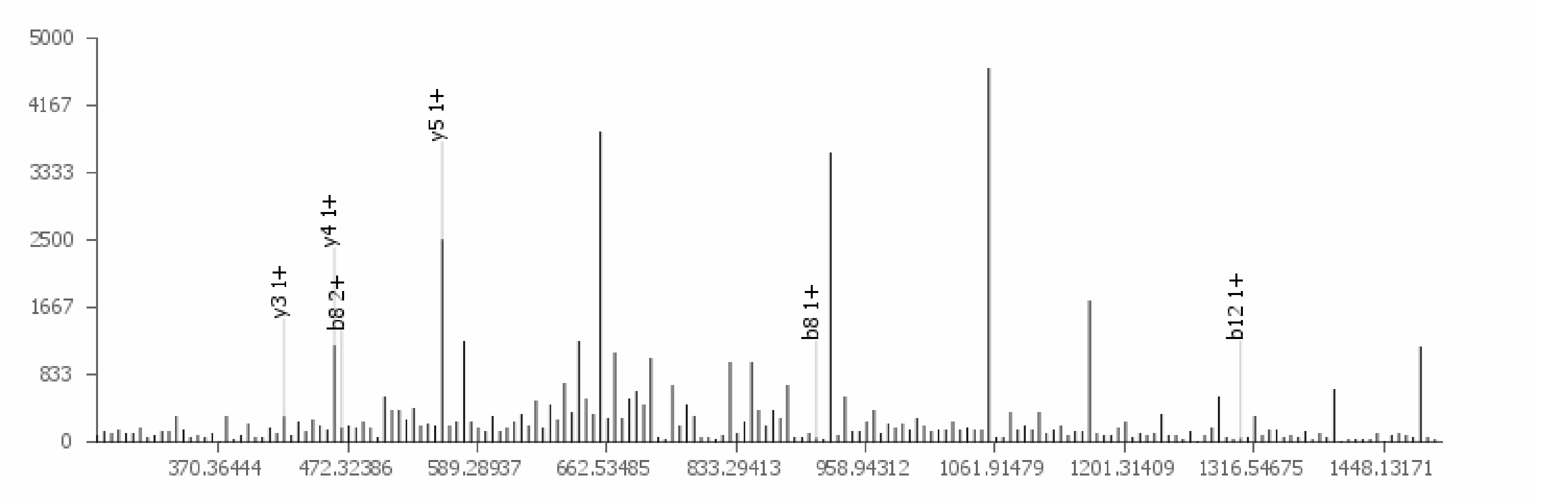

AT1G29900.1 - (oxM)AAKLSVGY(pT)LDQIPNDITR - 1151.550329 - Charge:2

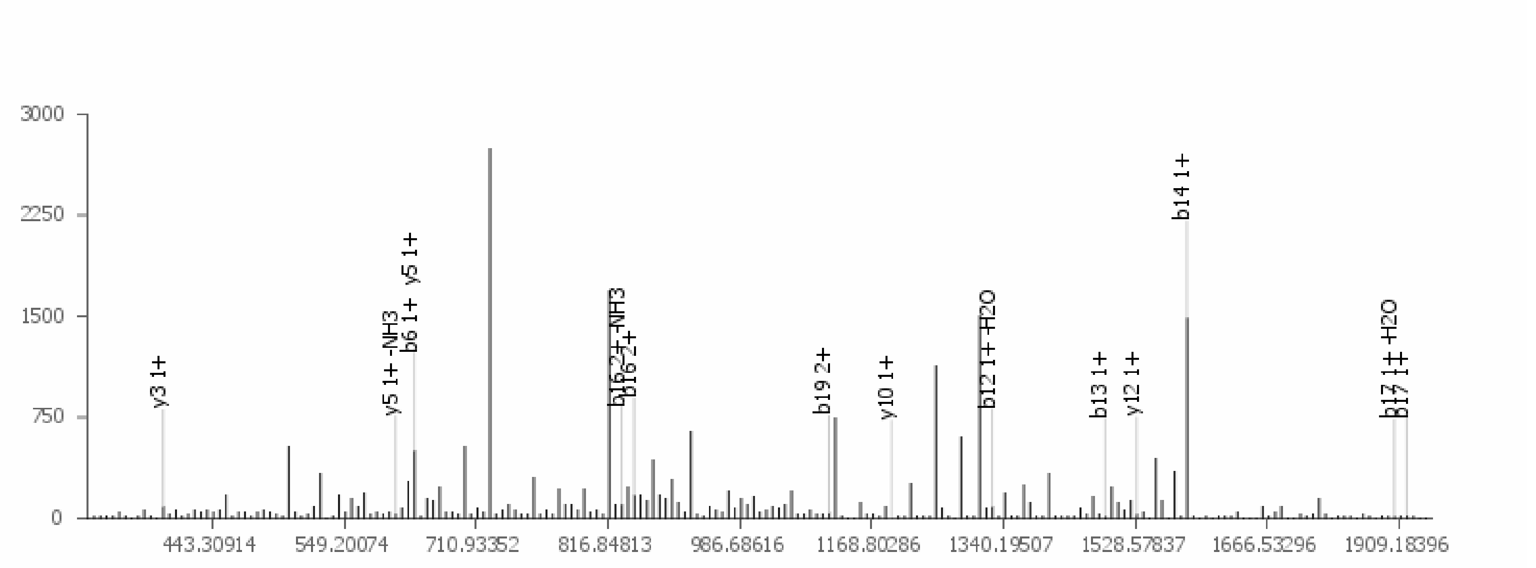

AT2G38940.1 - SLEE(oxM)SGENEDNEN(pS)NNDSR - 1183.433134 - Charge:2

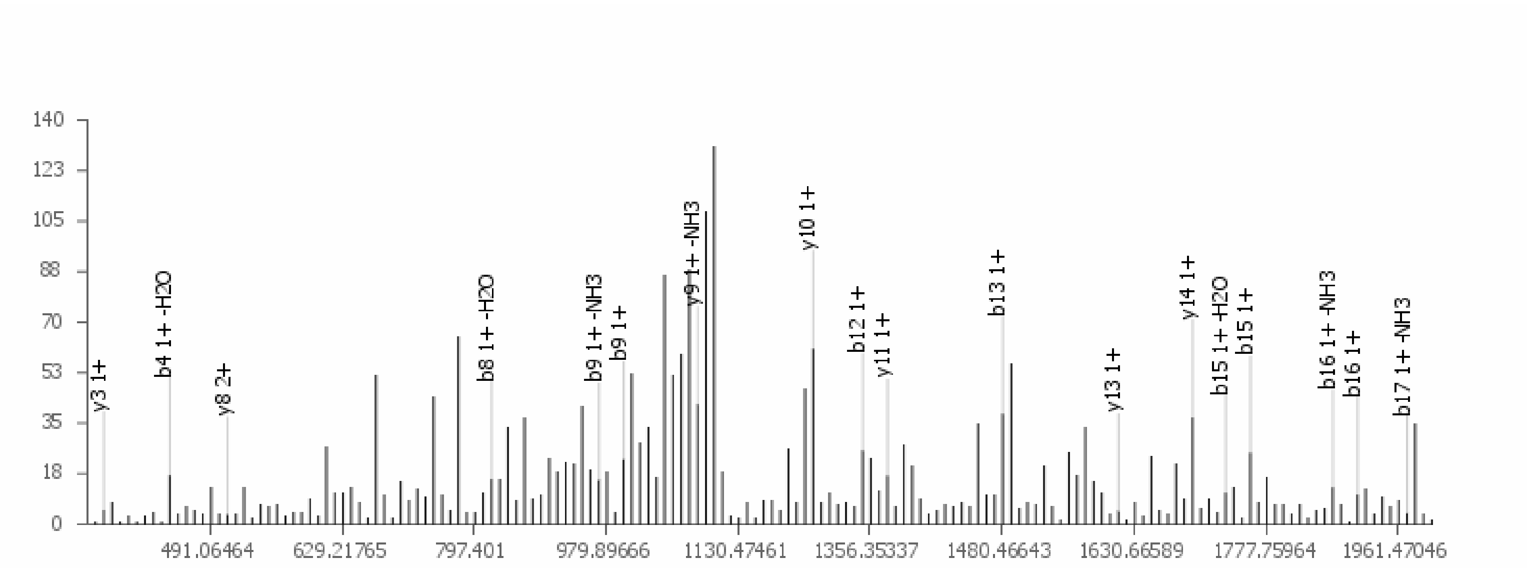

AT1G18620.1 - ELPRLSLD(pS)R - 633.314042 - Charge:2

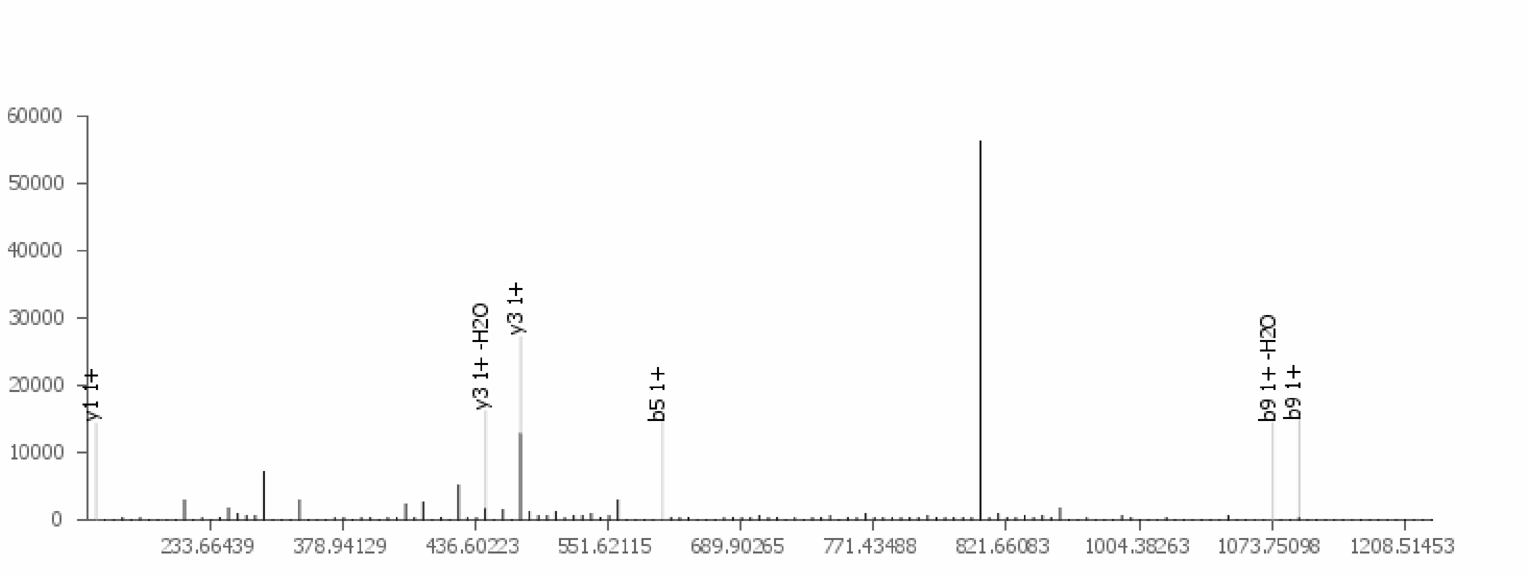

AT3G50920.1 - WVVSVLFG(pS)IILLR - 841.468789 - Charge:2

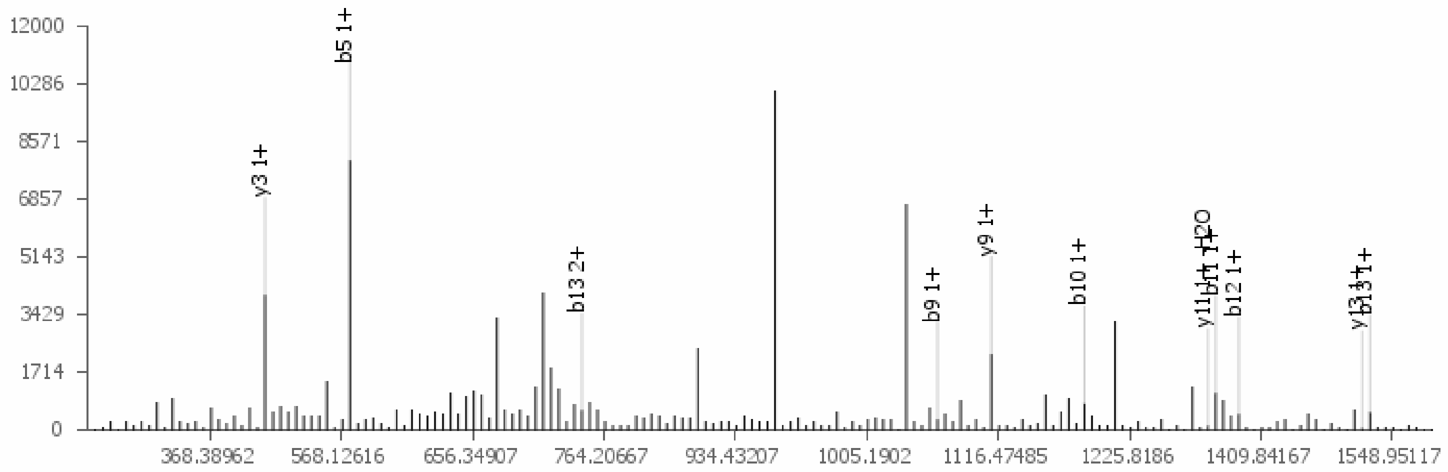

AT3G04200.1 - LF(pY)KVLK - 495.772396 - Charge:2

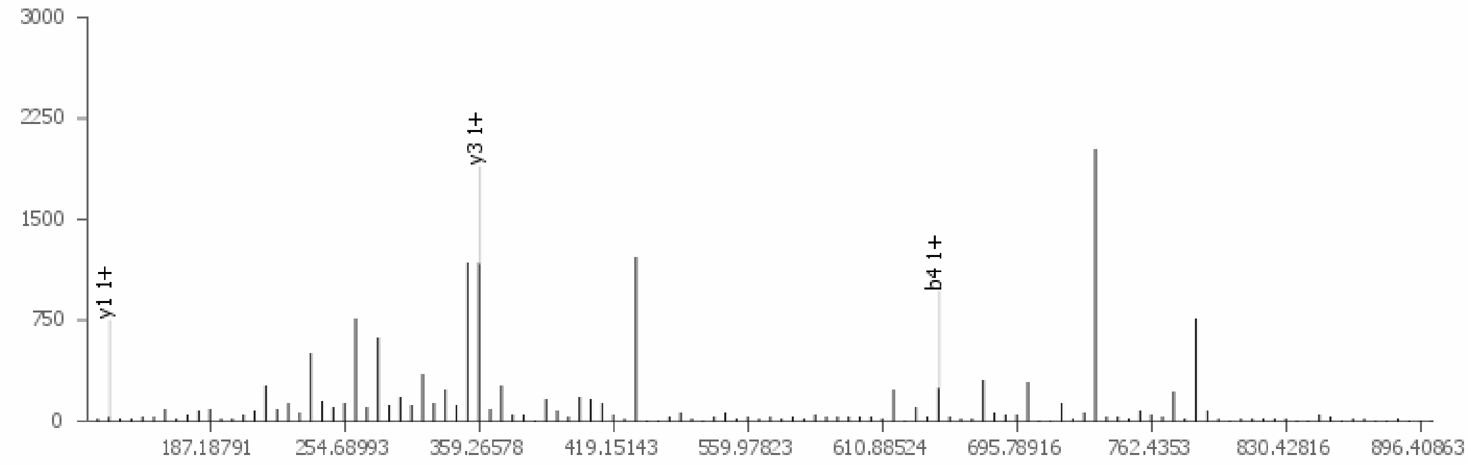

AT1G34320.1 - VSEVSSLLGRAG(pT)(oxM)GLGK - 929.461257 - Charge:2

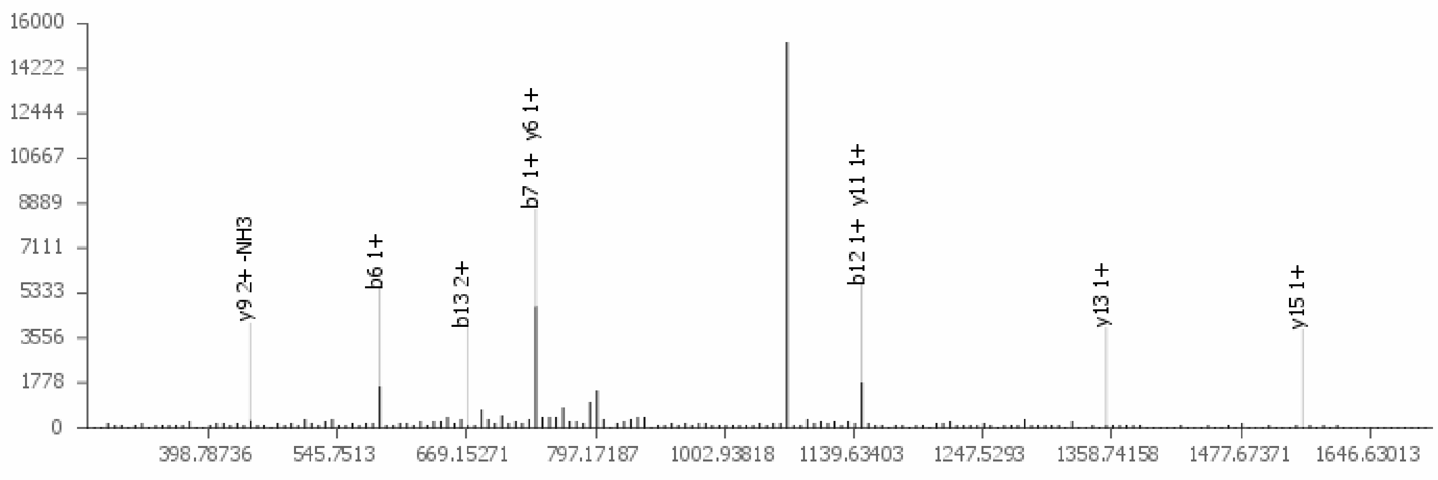

AT5G08530.1 - IFTNL(pY)GLHDPFLK - 879.441619 - Charge:2

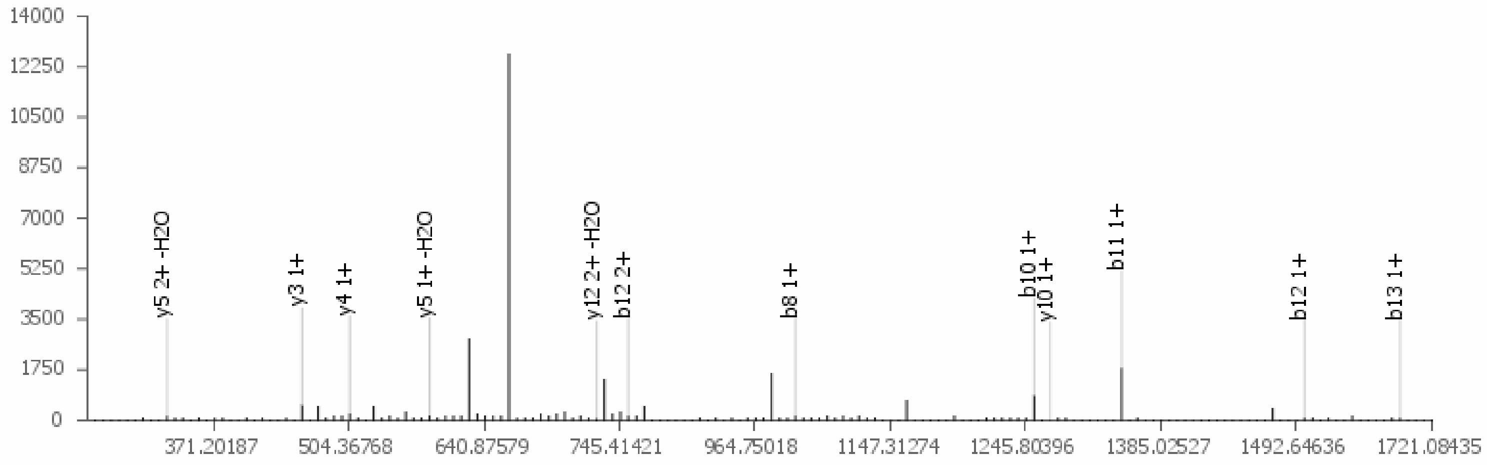

AT1G72410.1 - RSSIVSD(oxM)STDLA(pS)EK - 911.407621 - Charge:2

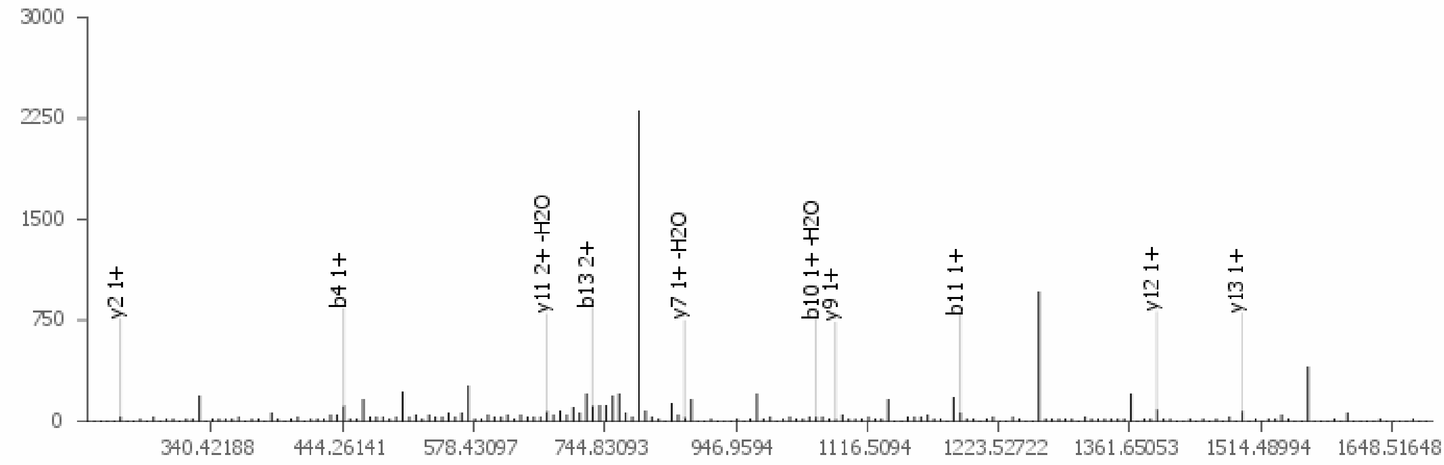

AT5G45450.1 - (pS)MAKLGVPR - 519.764632 - Charge:2

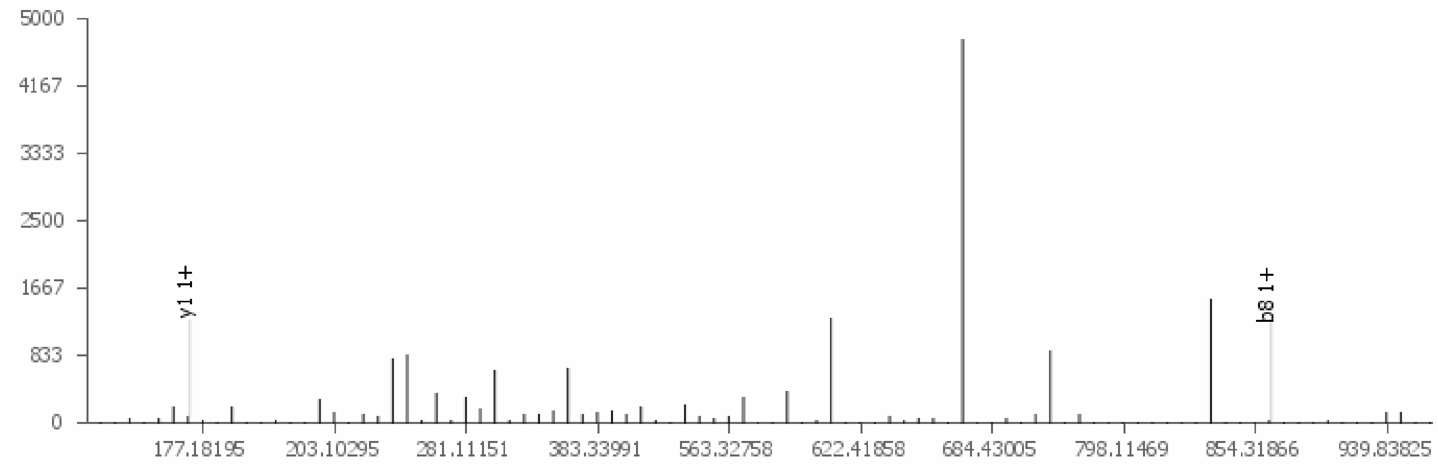

AT3G22980.1 - SSSISLK(pY)K - 546.770085 - Charge:2

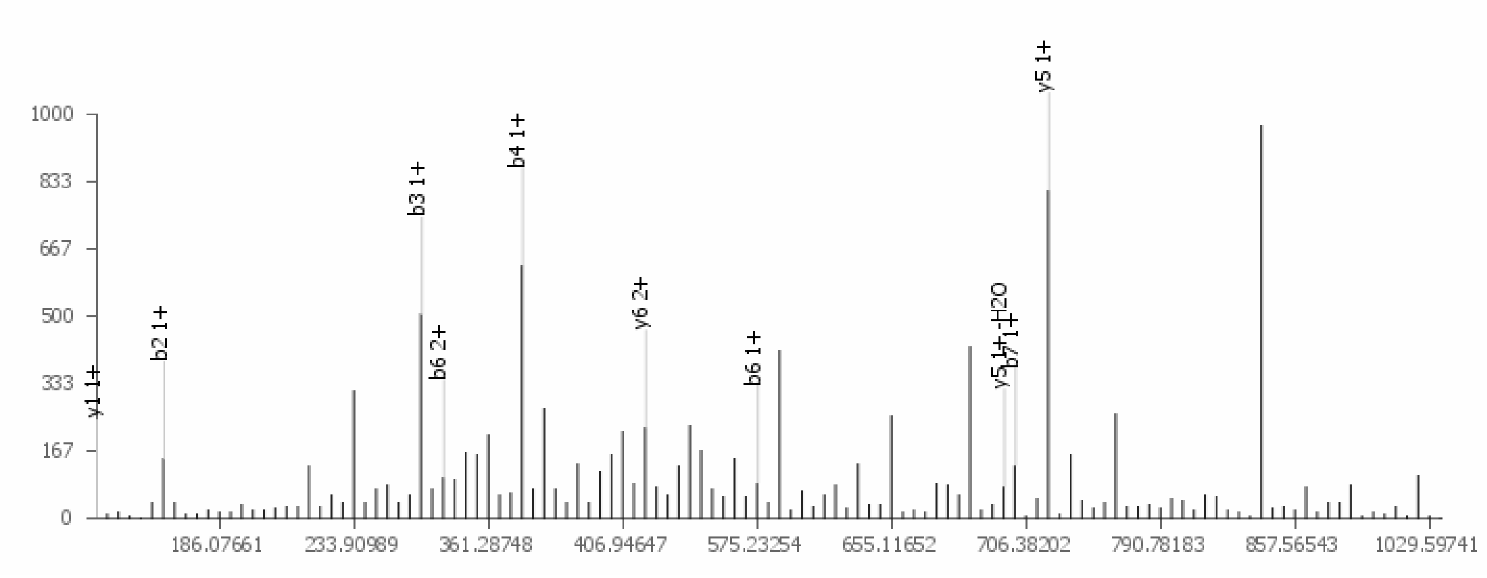

AT1G68585.1 - (oxM)IETY(pS)ILV(pS)VLEKK - 964.961075 - Charge:2

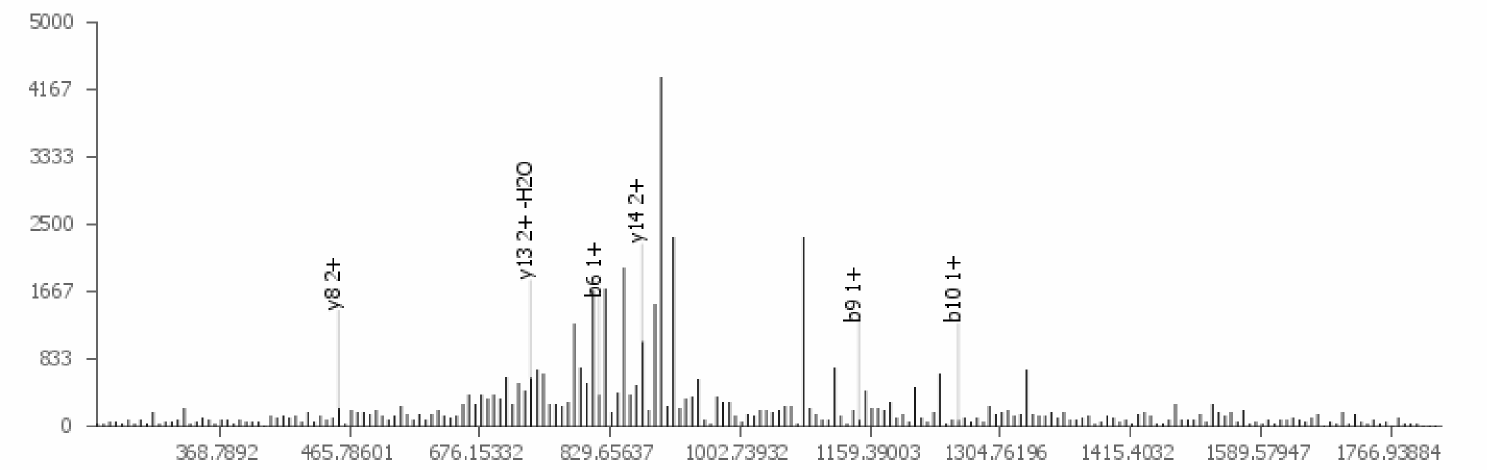

AT1G33870.1 - R(oxM)(pT)QLLER - 571.775466 - Charge:2

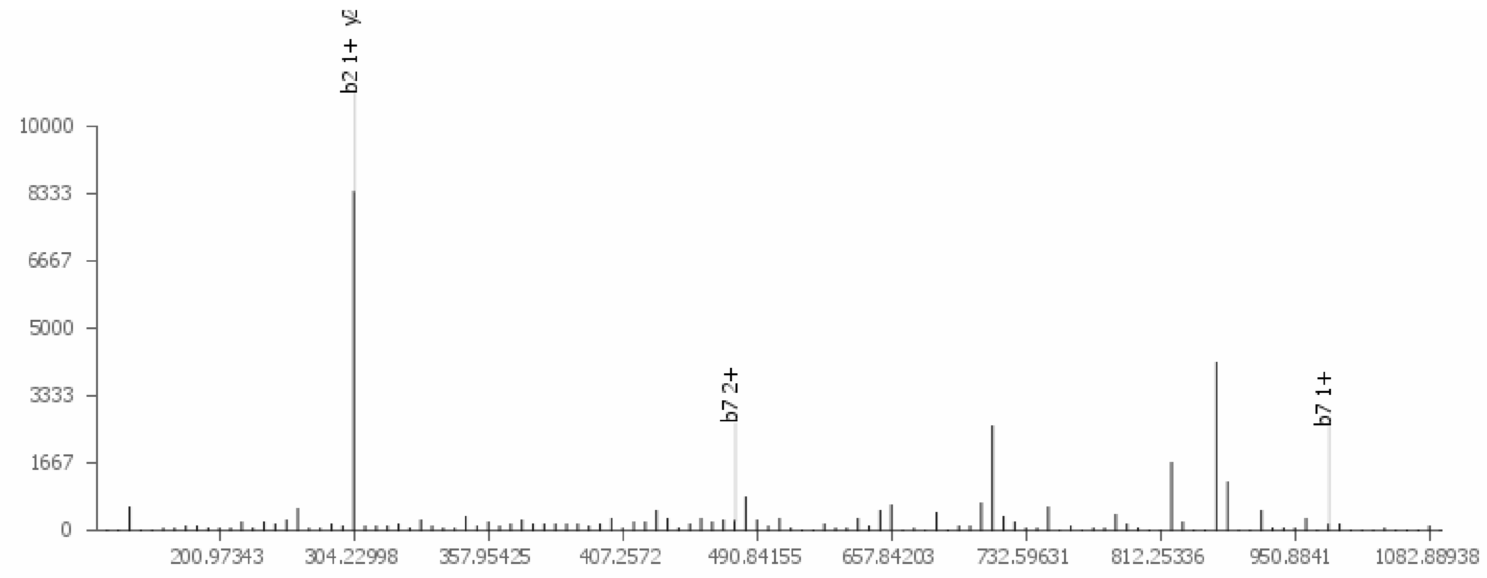

AT4G15053.1 - DFLQAGLTVNPVL(pY)K - 879.449468 - Charge:2

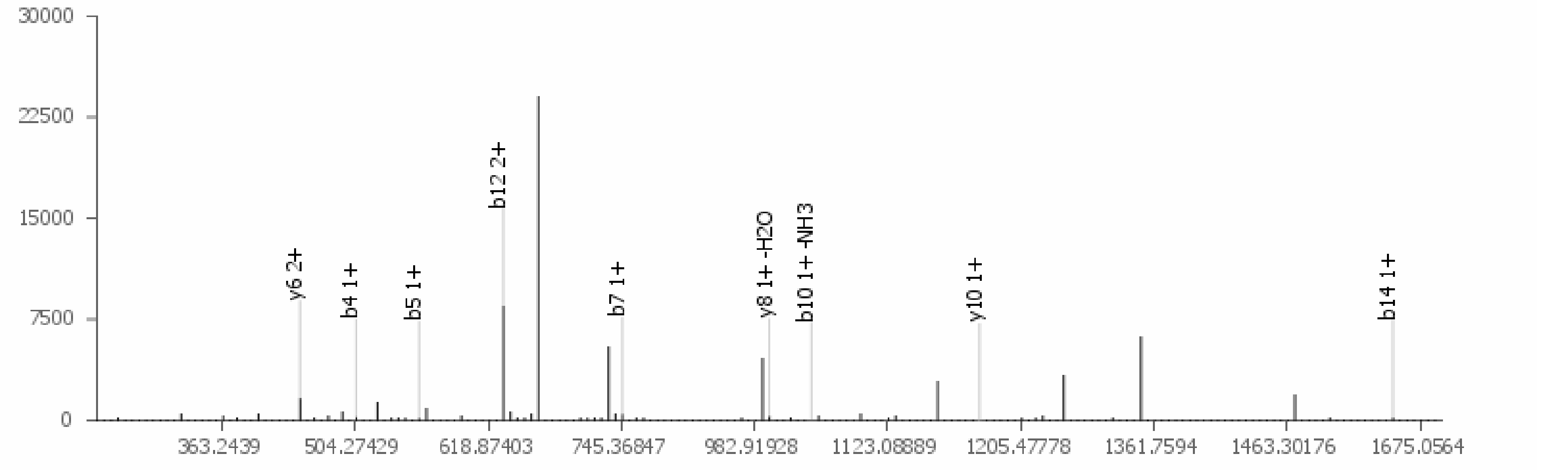

AT1G10040.1 - ISFVGH(pS)LGGLIAR(pY)AIGR - 1074.04248 - Charge:2

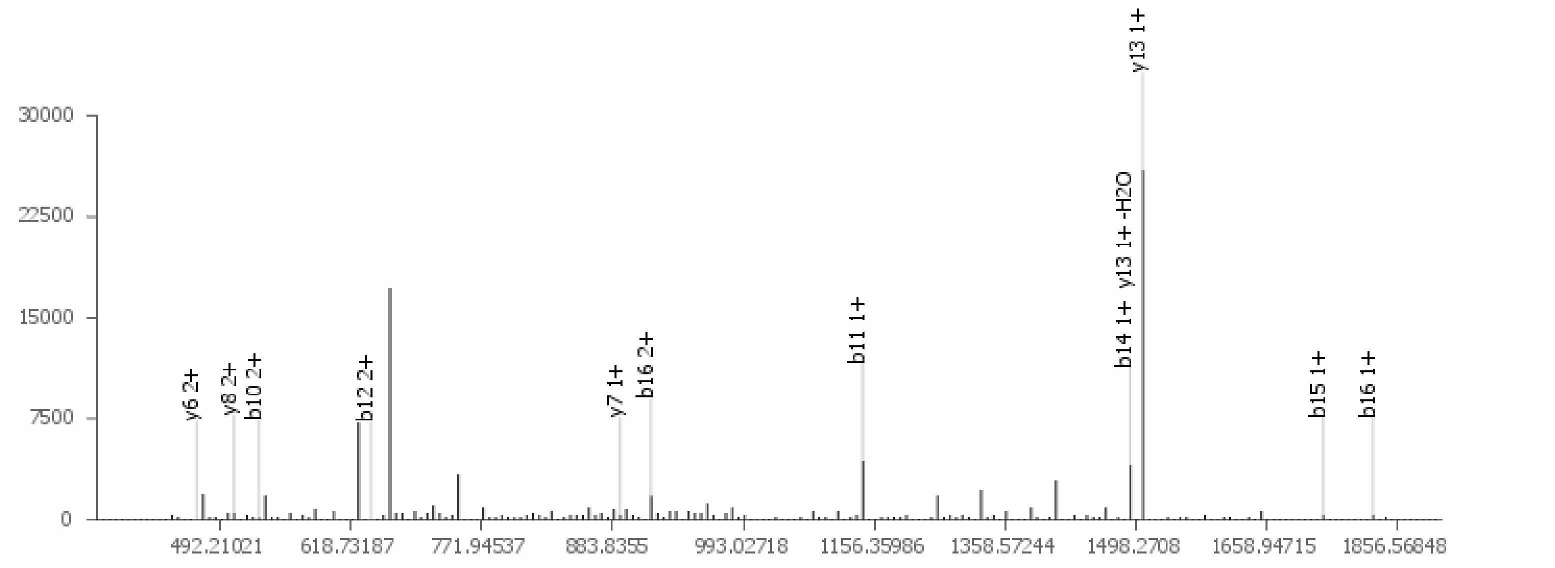

AT3G59410.1 - GALRADRP(pT)R - 596.802793 - Charge:2

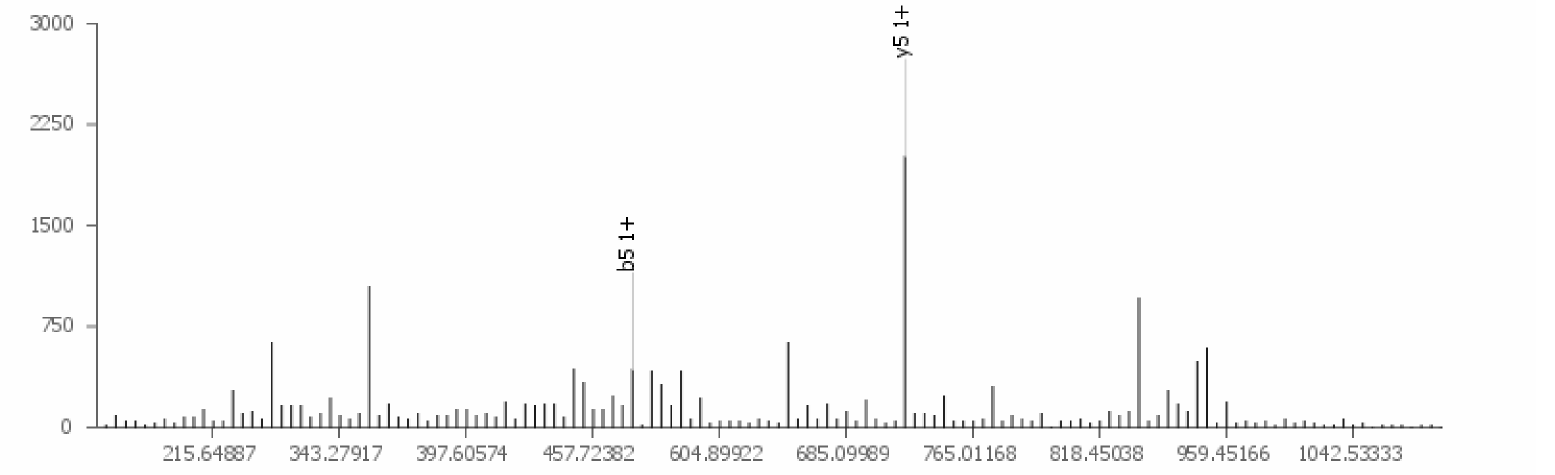

AT3G48050.1 - NAGAGGV(pS)EVLAAVKDEK - 897.932511 - Charge:2

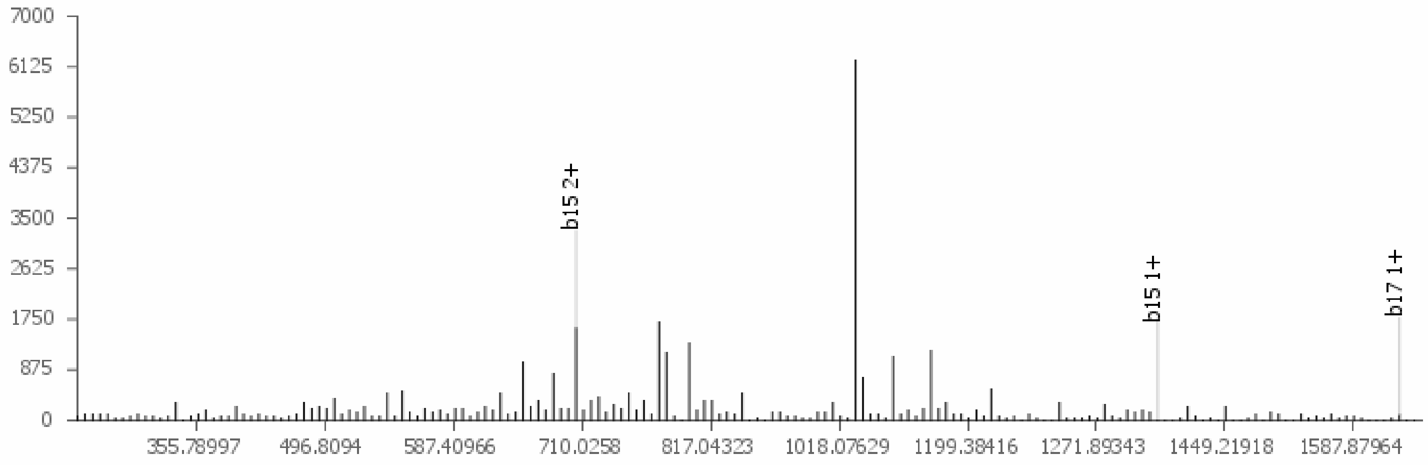

AT2G36350.1 - (pS)PRVGPSDSISLK - 711.852976 - Charge:2

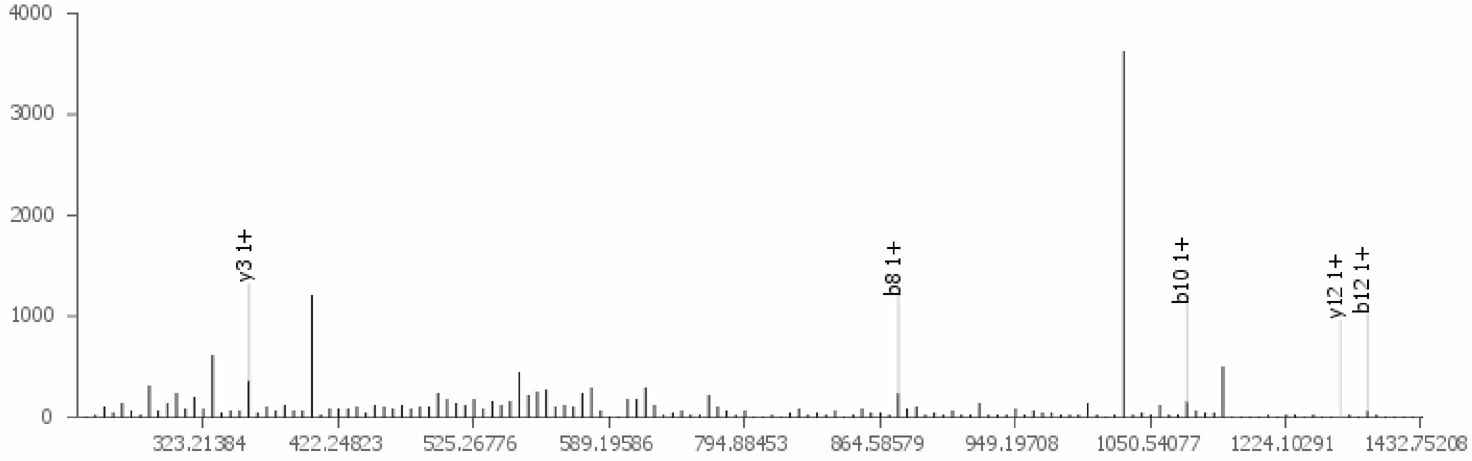

AT3G47570.1 - V(pT)HLELGR - 502.754505 - Charge:2

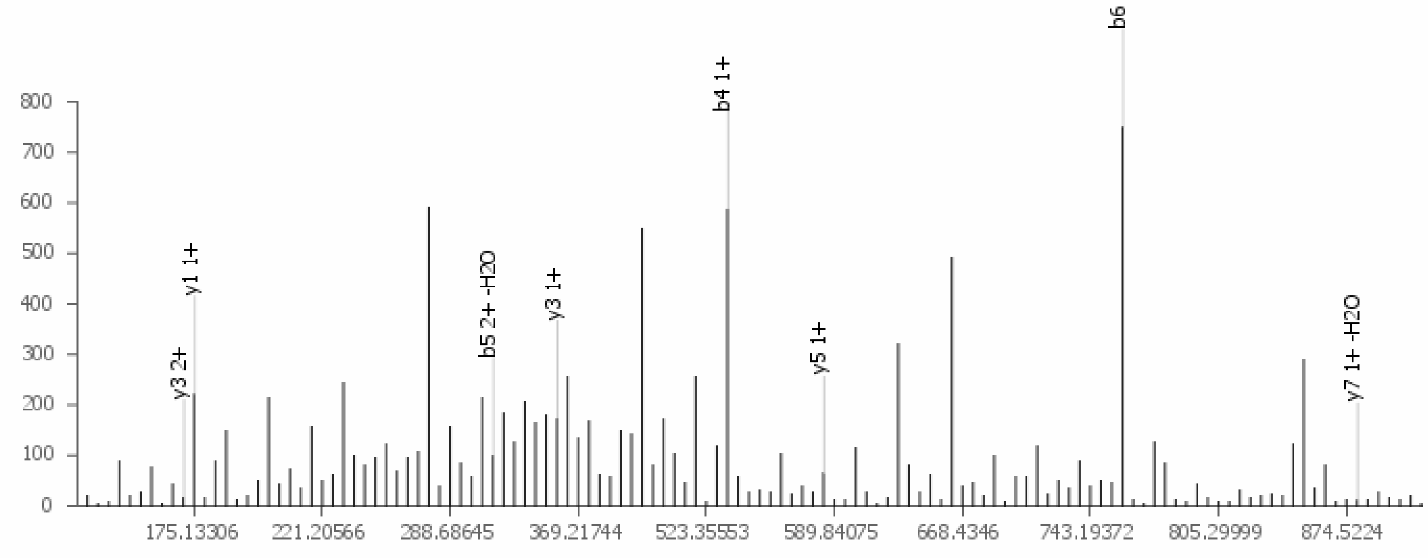

AT3G26160.1 - FLVGELFC(pS)K - 611.787812 - Charge:2

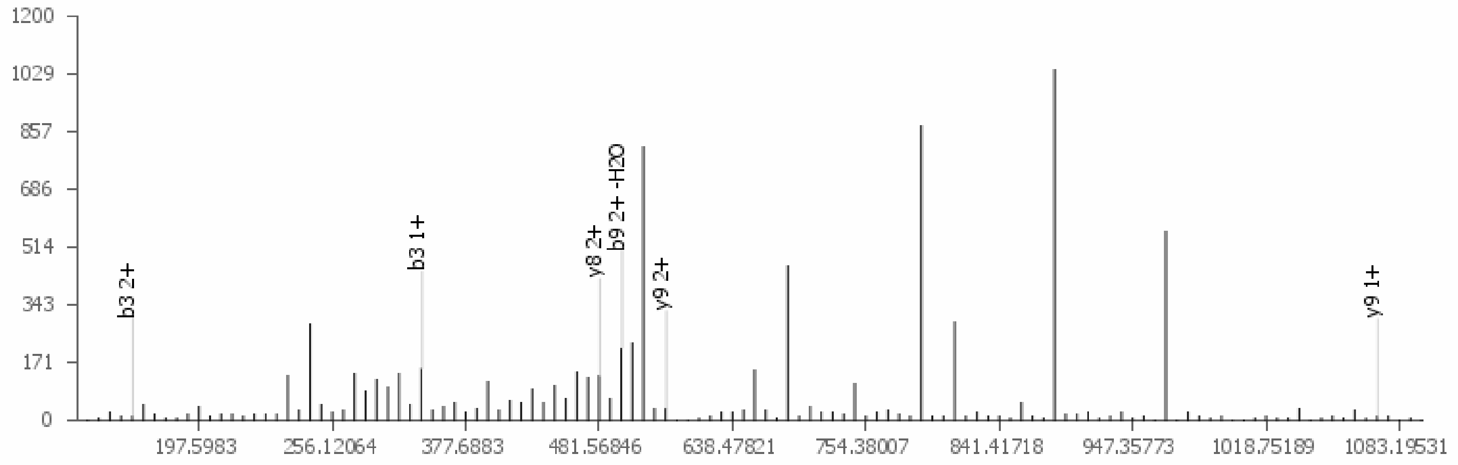

AT2G38995.1 - KT(pS)DPDALPTTAATR - 812.886051 - Charge:2

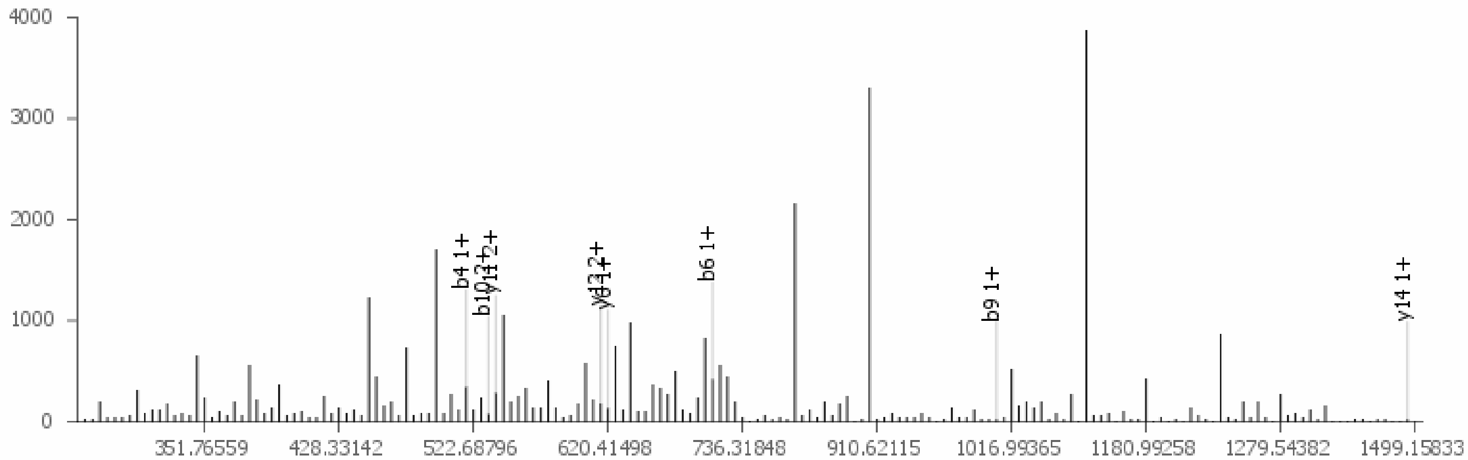

AT1G50730.1 - LV(oxM)MVRGLADPLTSL(pY)CR - 1067.523817 - Charge:2

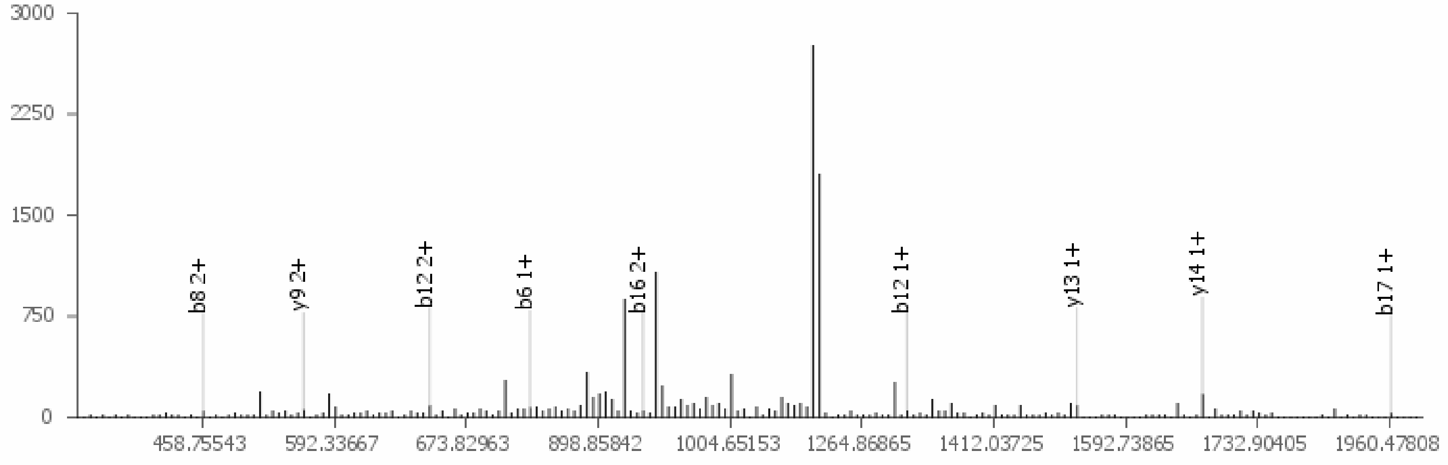

AT2G26680.1 - (pS)NKEIAVQVK - 598.310732 - Charge:2

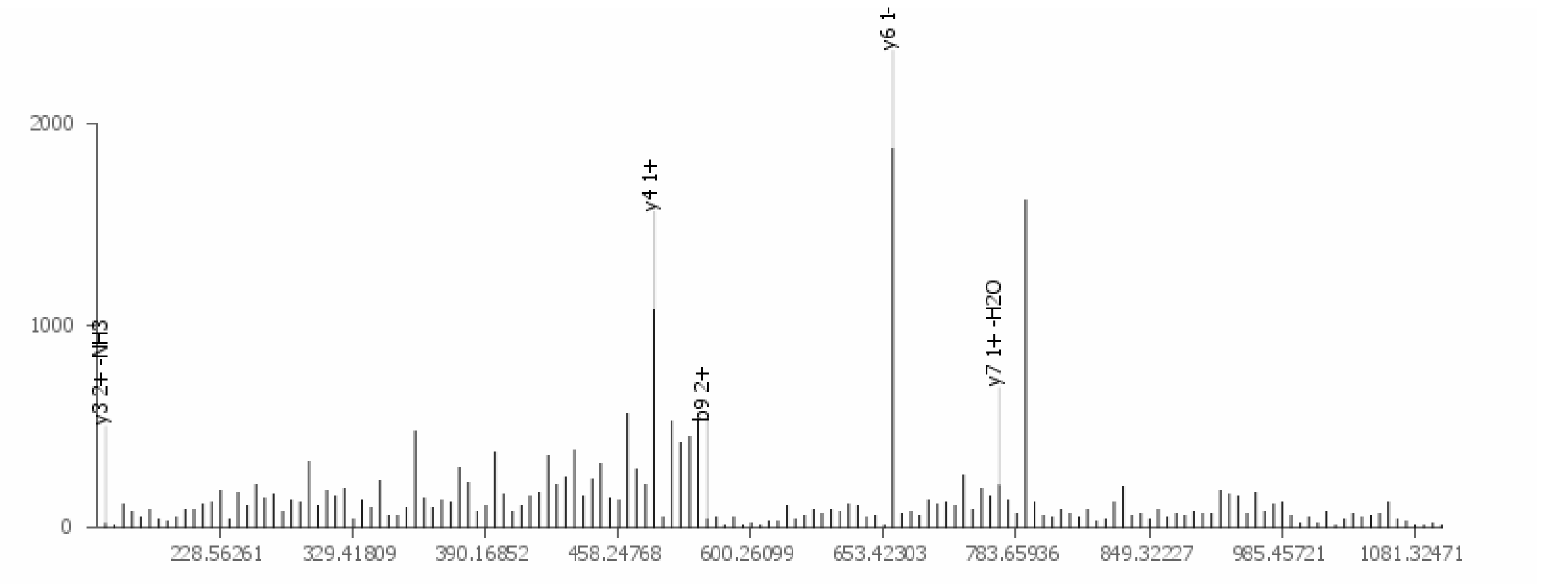

AT3G12020.1 - EGSYINK(pS)LL(pT)LGTVISK - 1042.014347 - Charge:2

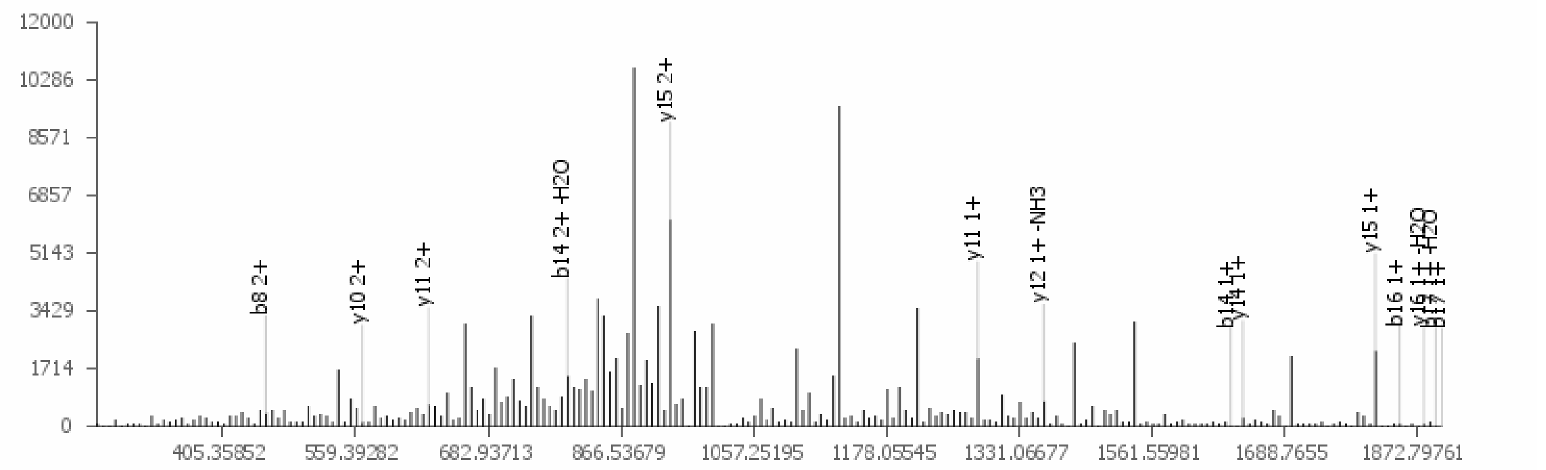

AT4G18750.1 - DIISWNTIIGGY(pS)K - 823.893407 - Charge:2

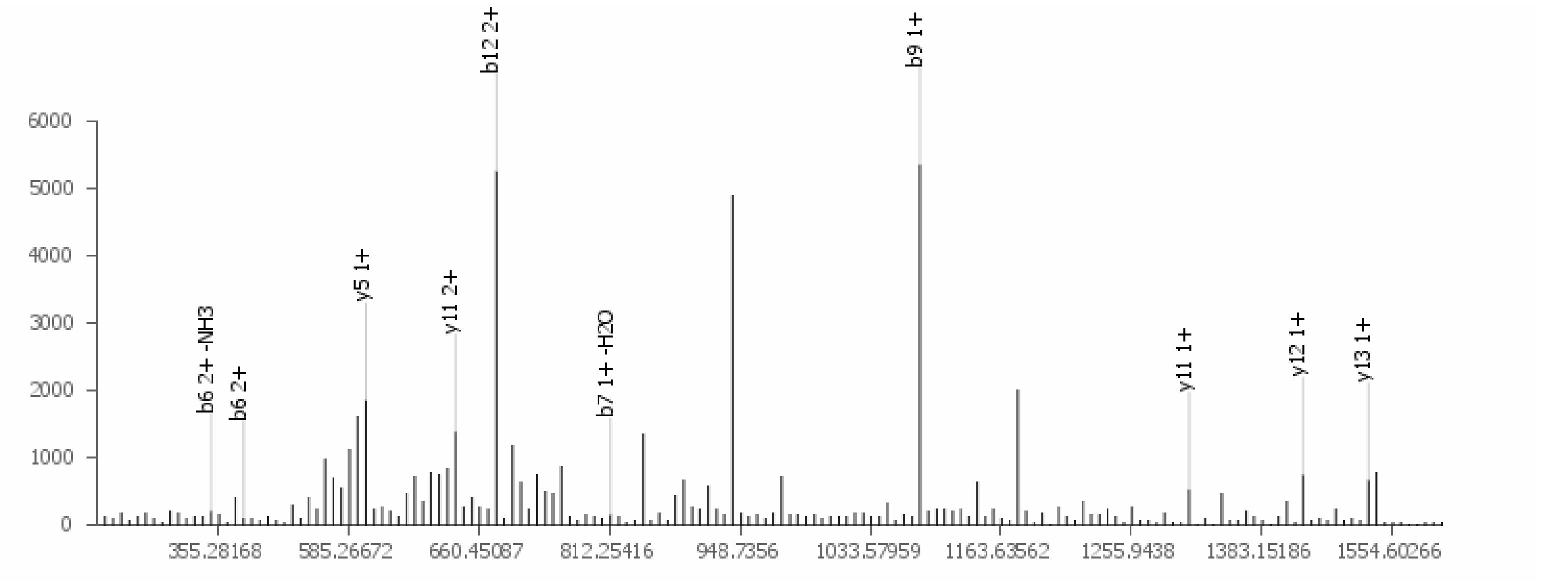

AT1G20620.1 - GFFEVTHDISNL(pT)CADFLR - 1133.005702 - Charge:2

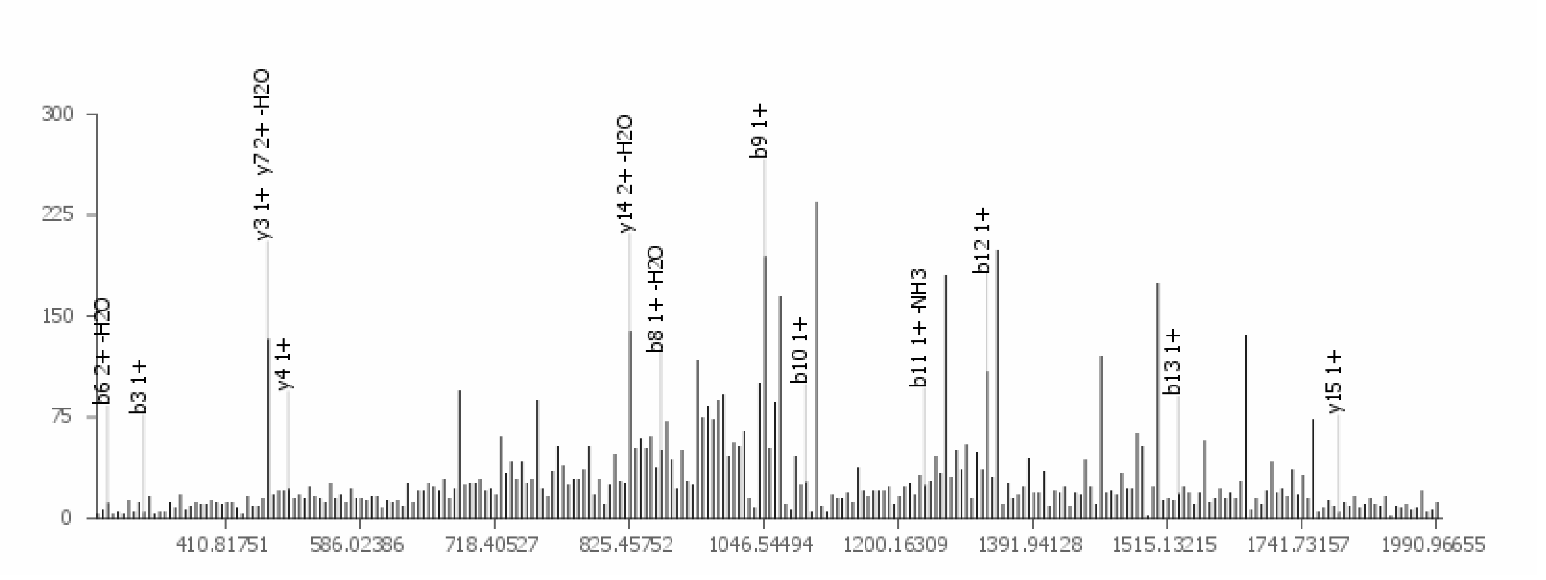

AT1G33490.1 - ITGGPHFPLT(pS)DALKK - 881.447969 - Charge:2

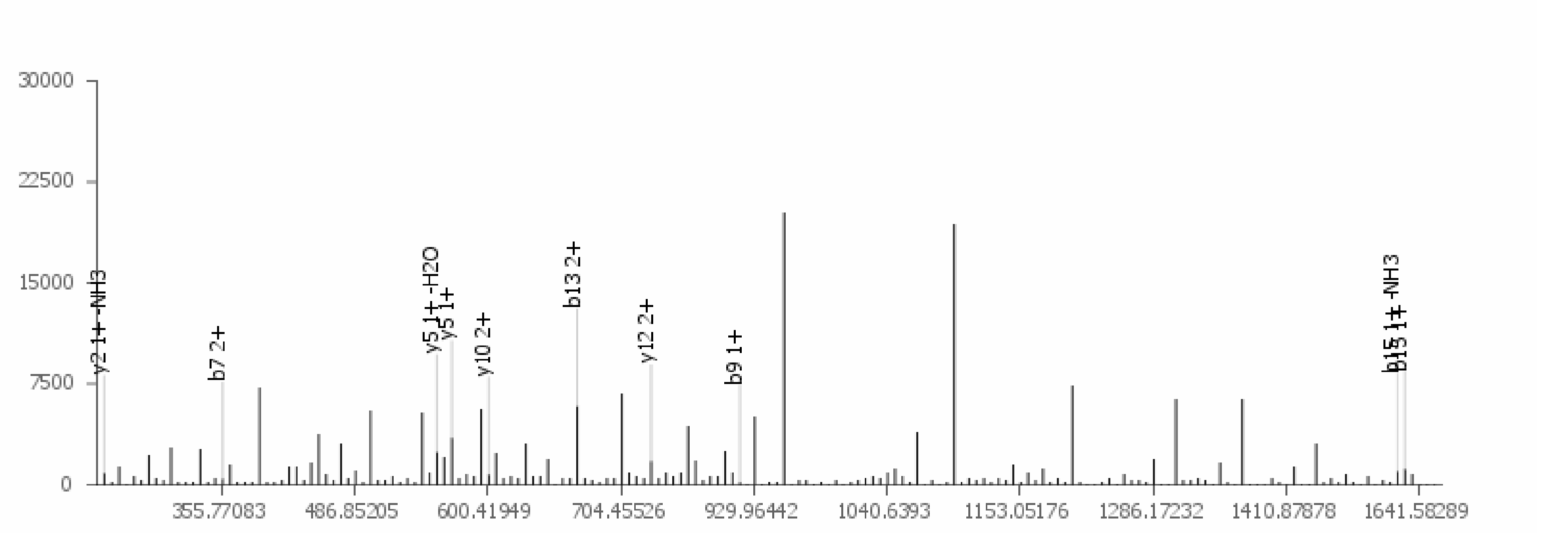

AT4G23610.1 - (pS)ETIPAKR - 981.471038 - Charge:1

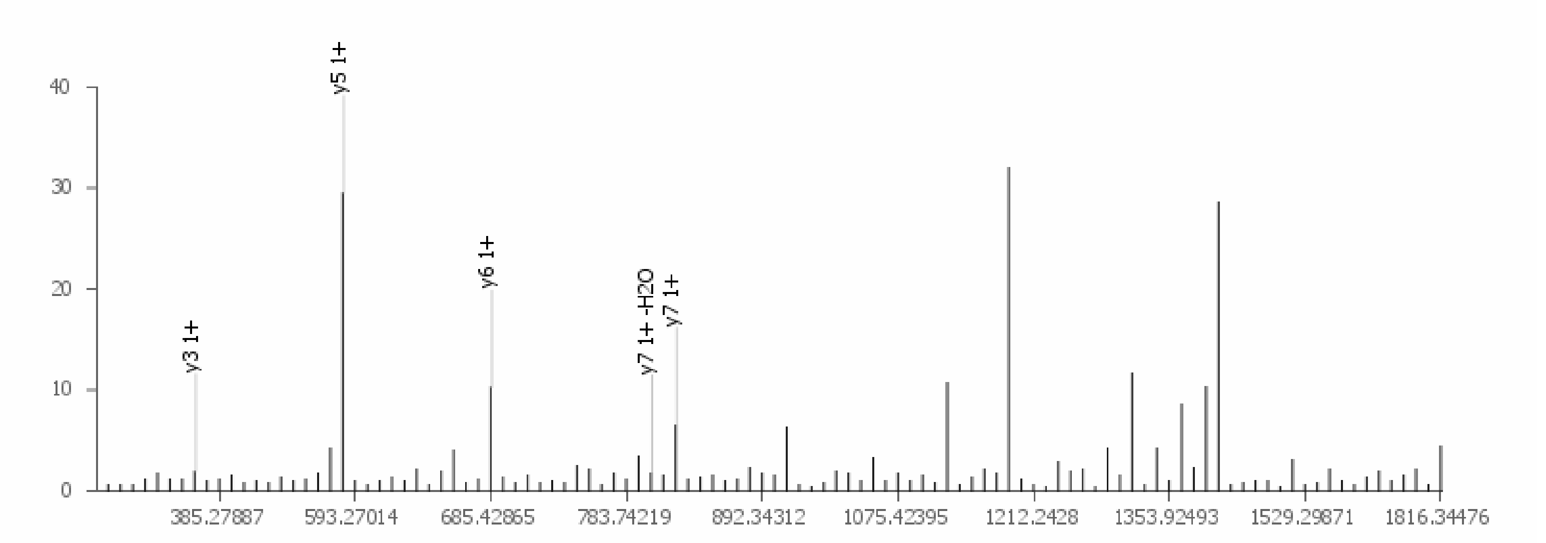

AT3G48470.1 - K(oxM)AS(pS)IAFMFSK - 722.33039 - Charge:2

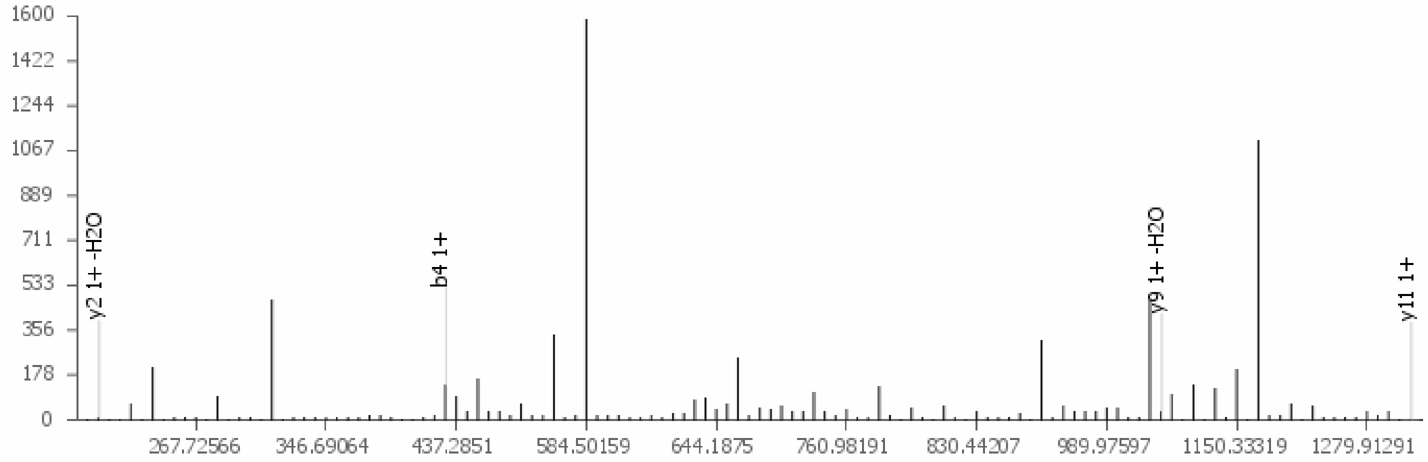

AT5G48820.1 - LEKP(pS)SLIEPK - 660.842113 - Charge:2

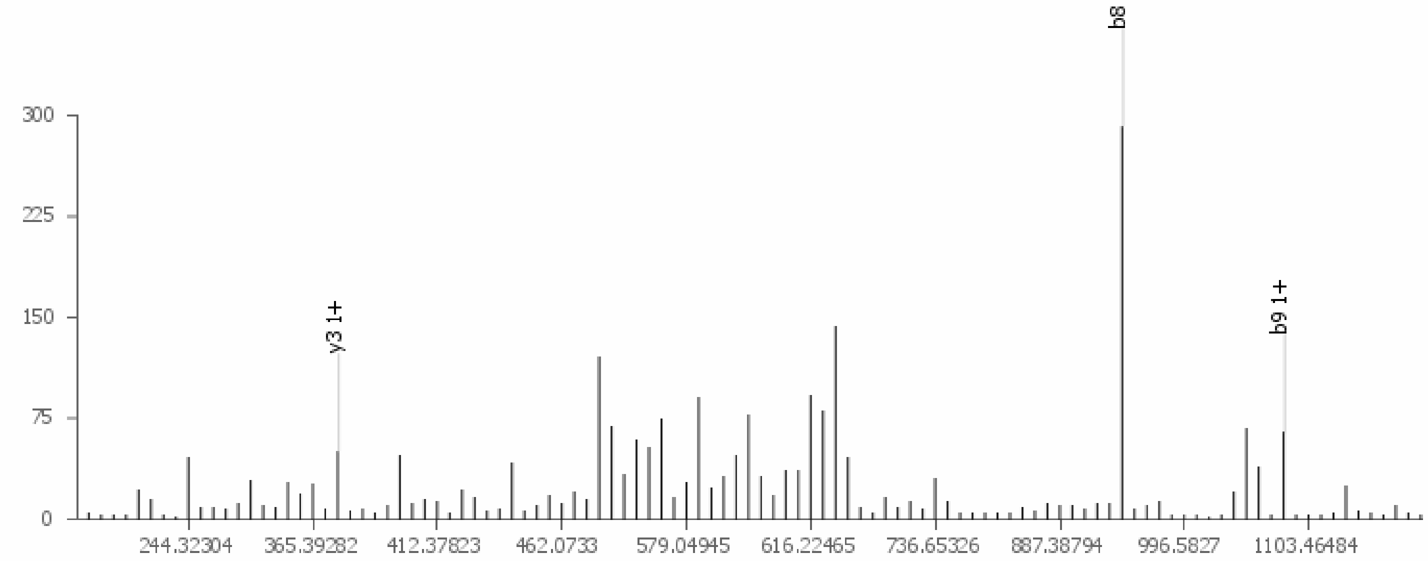

AT3G54400.1 - SSL(y)(y)VNLVGIR - 732.363773 - Charge:2

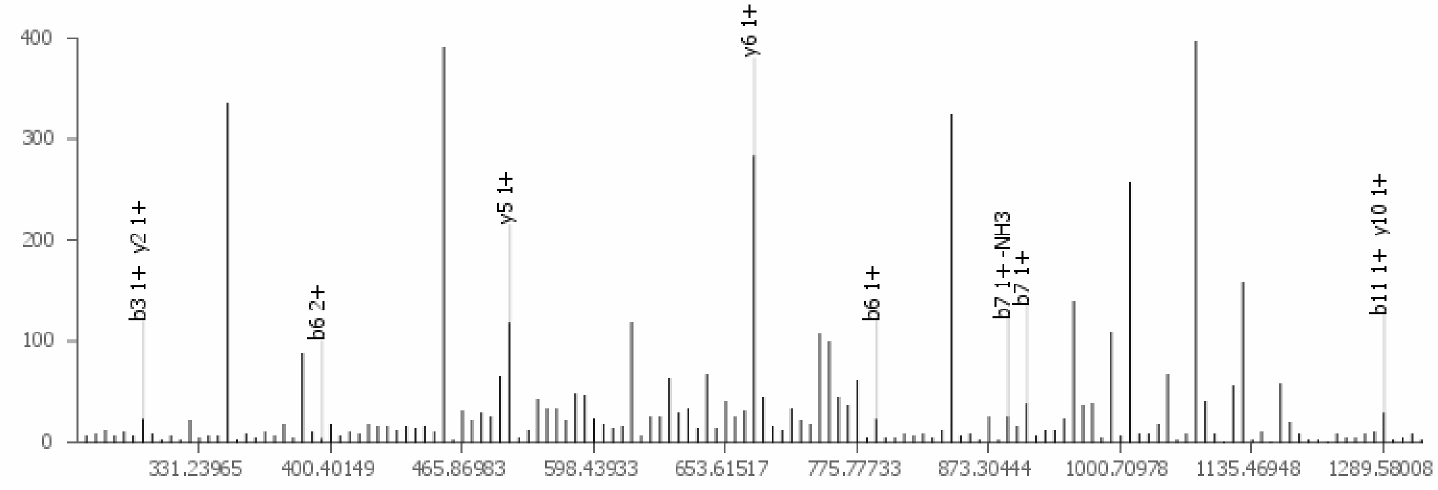

AT1G04445.1 - FLLPKMPP(pT)TMPETPK - 636.649501 - Charge:3

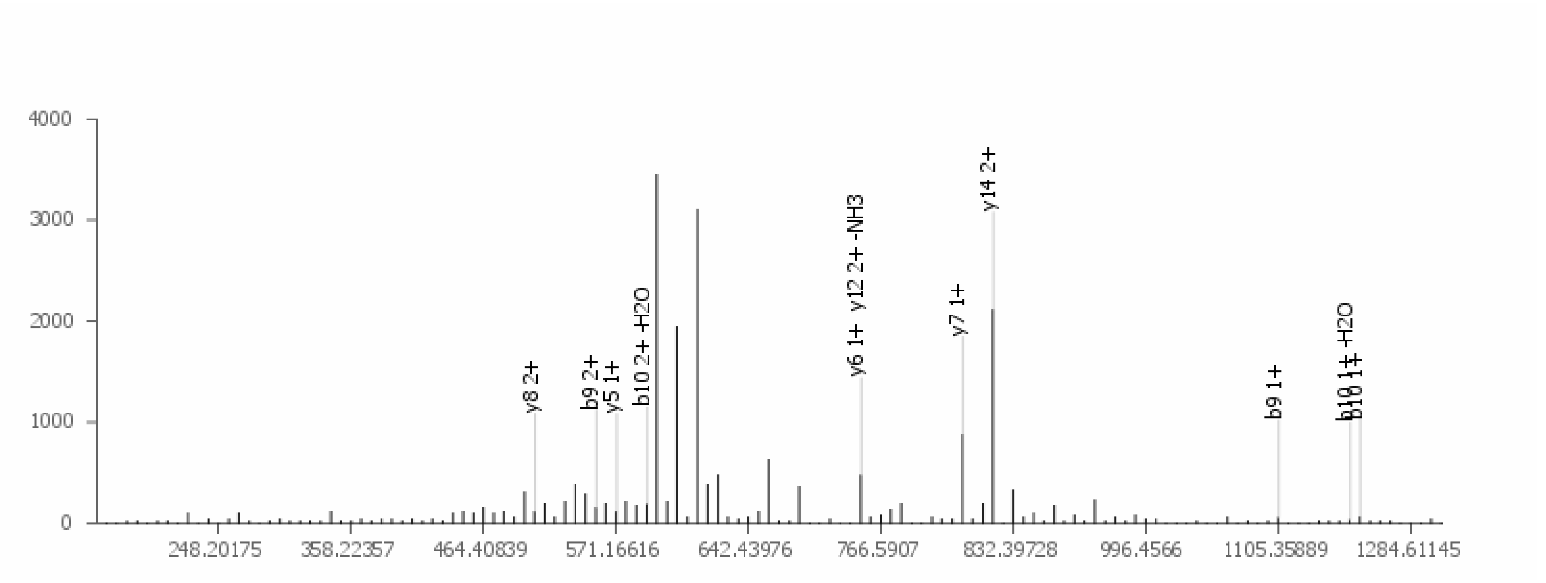

AT4G14730.1 - SDIE(pT)GGGNELYPGMK - 874.358445 - Charge:2

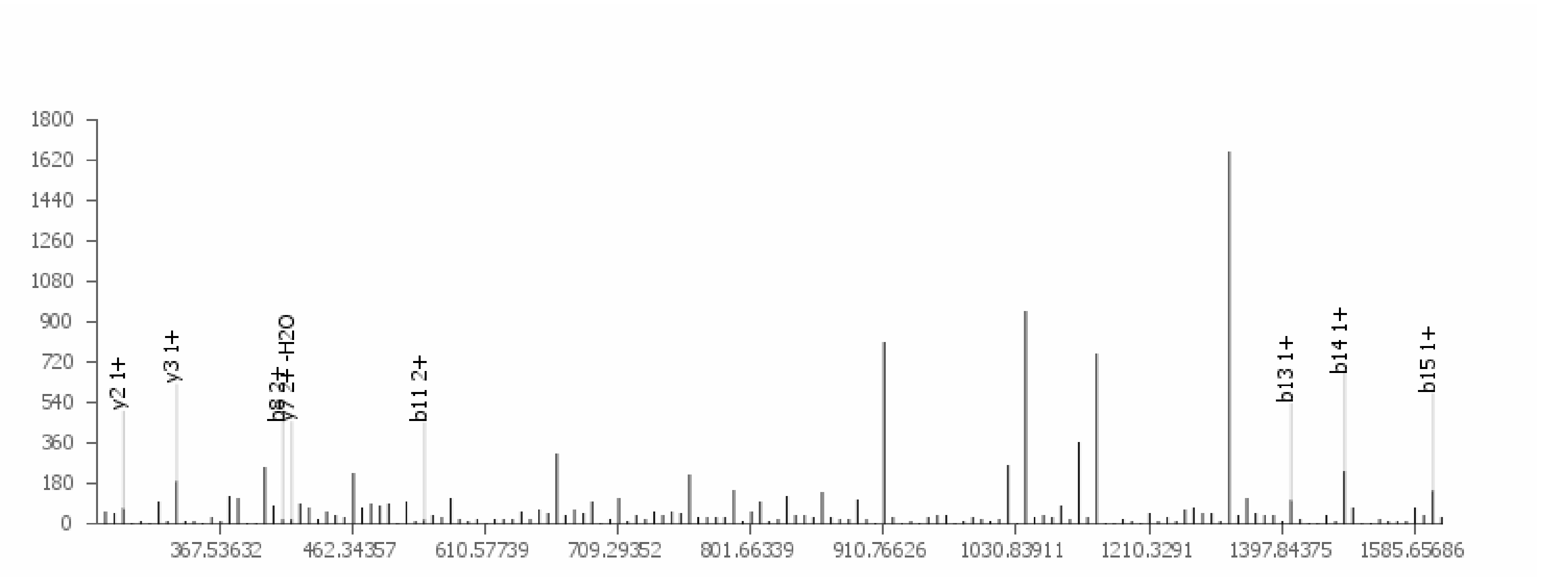

AT3G02810.1 - LSSK(s)(s)QK - 472.725563 - Charge:2

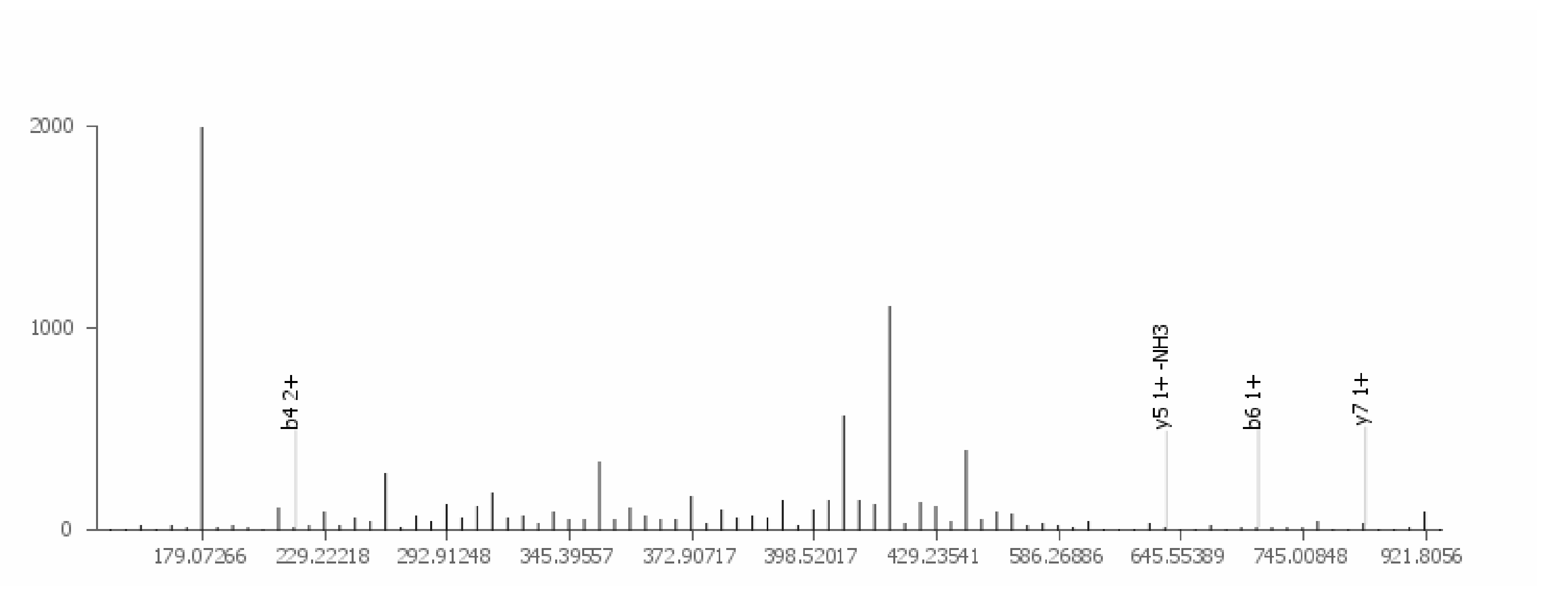

AT1G26170.1 - TETT(pT)LVTPVLK - 691.863906 - Charge:2

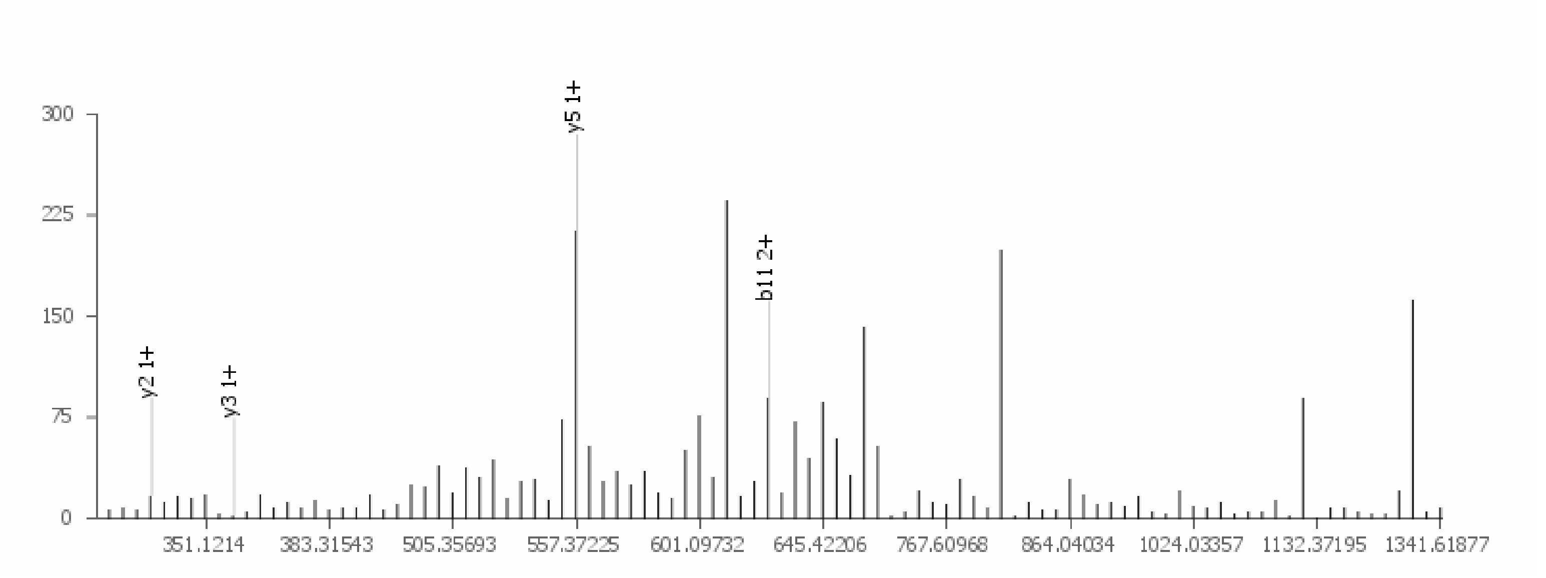

AT5G53310.1 - SASDYELEVLL(pS)NR - 838.377971 - Charge:2

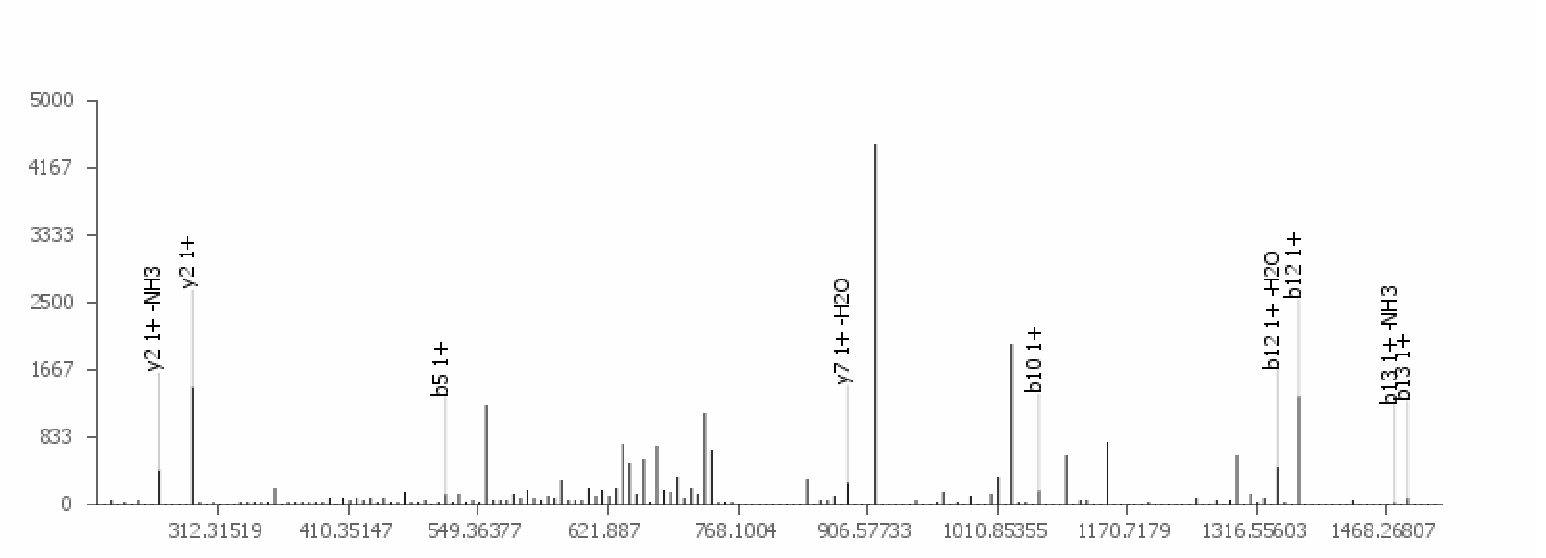

AT5G48110.1 - LYSVE(pY)GIDEWKR - 579.934503 - Charge:3

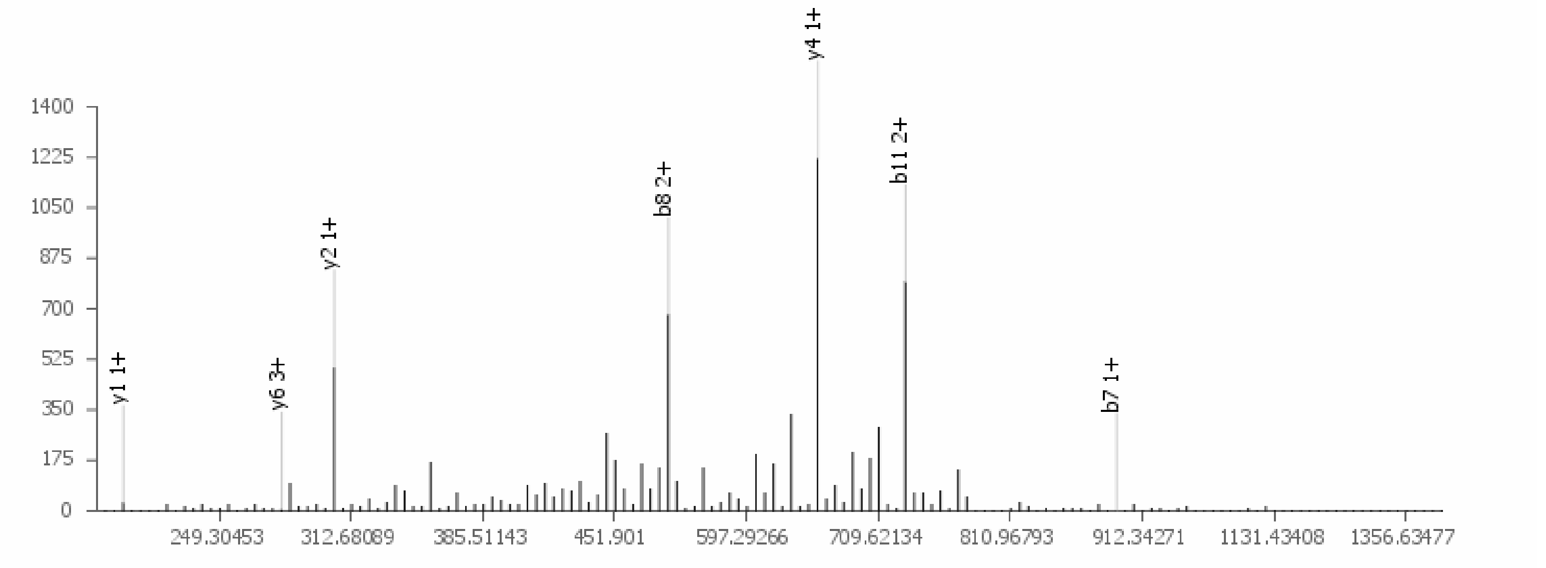

AT5G04930.1 - VRVDPVLLQL(pT)K - 730.910978 - Charge:2

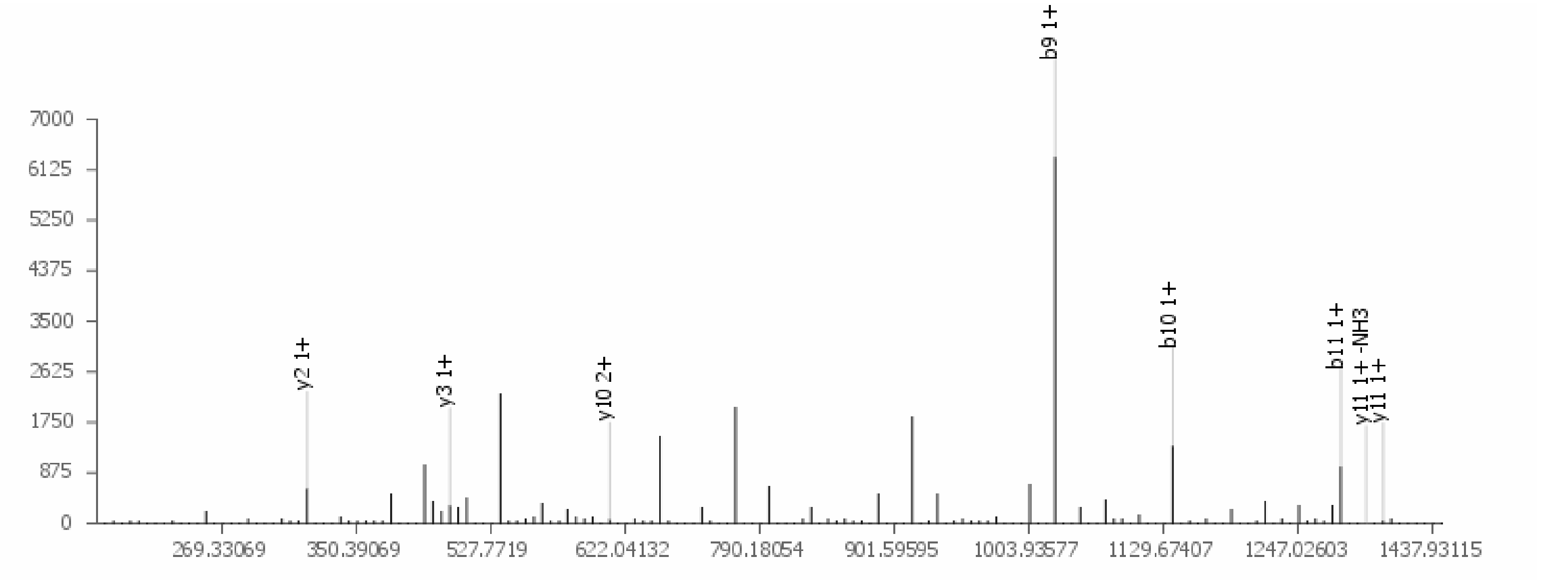

AT4G39450.1 - HE(pT)A(pT)LLEAR - 650.77509 - Charge:2

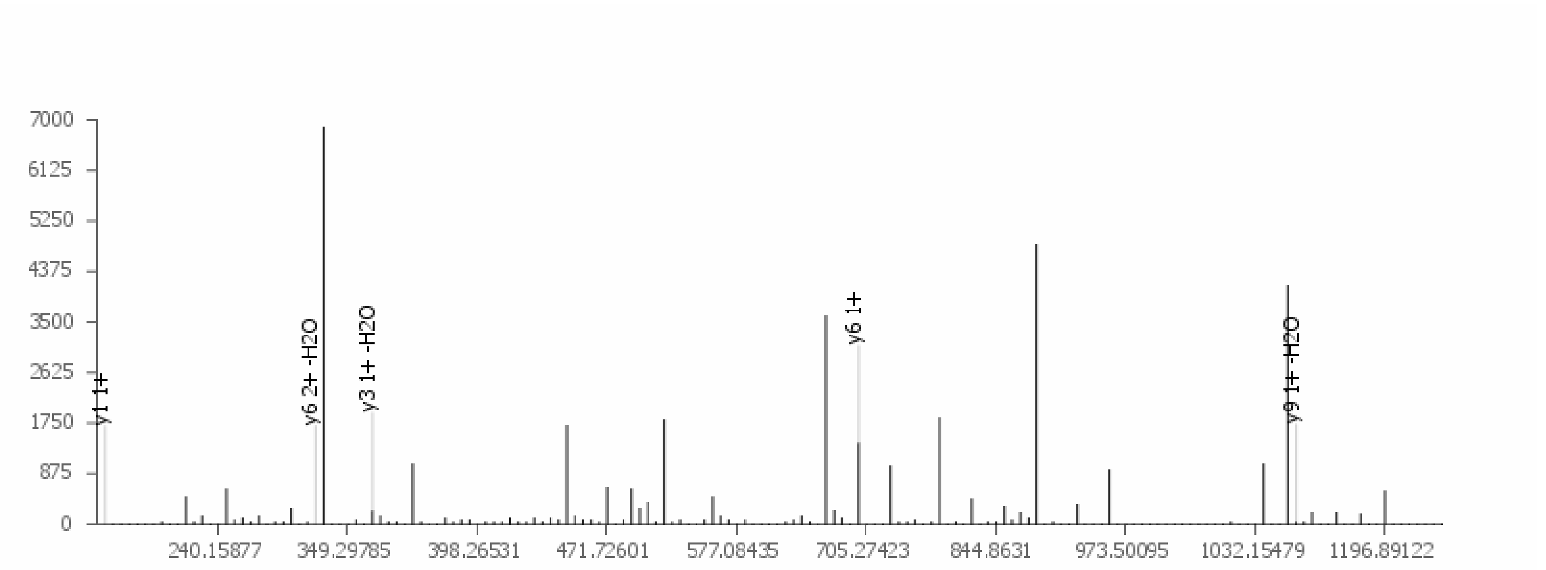

AT1G11720.1 - AED(pT)VKLYYNK - 712.335959 - Charge:2

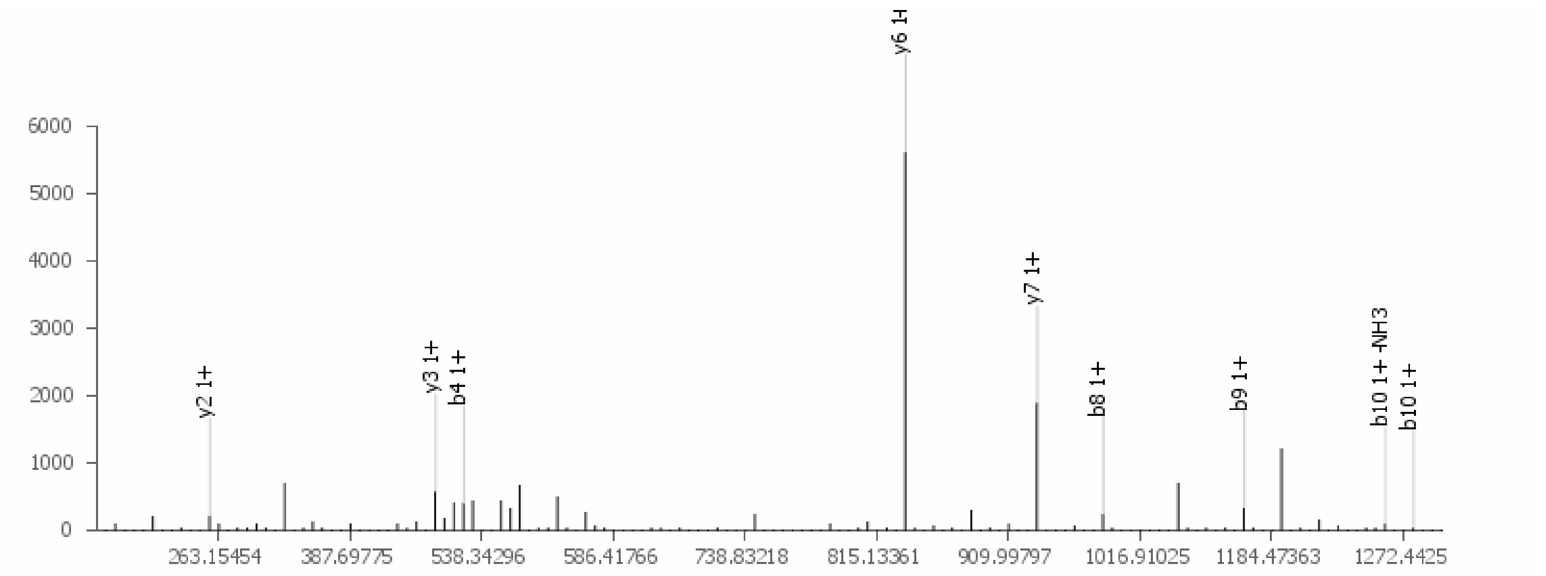

AT4G35390.1 - NKPKPP(t)II(t)R - 672.870584 - Charge:2

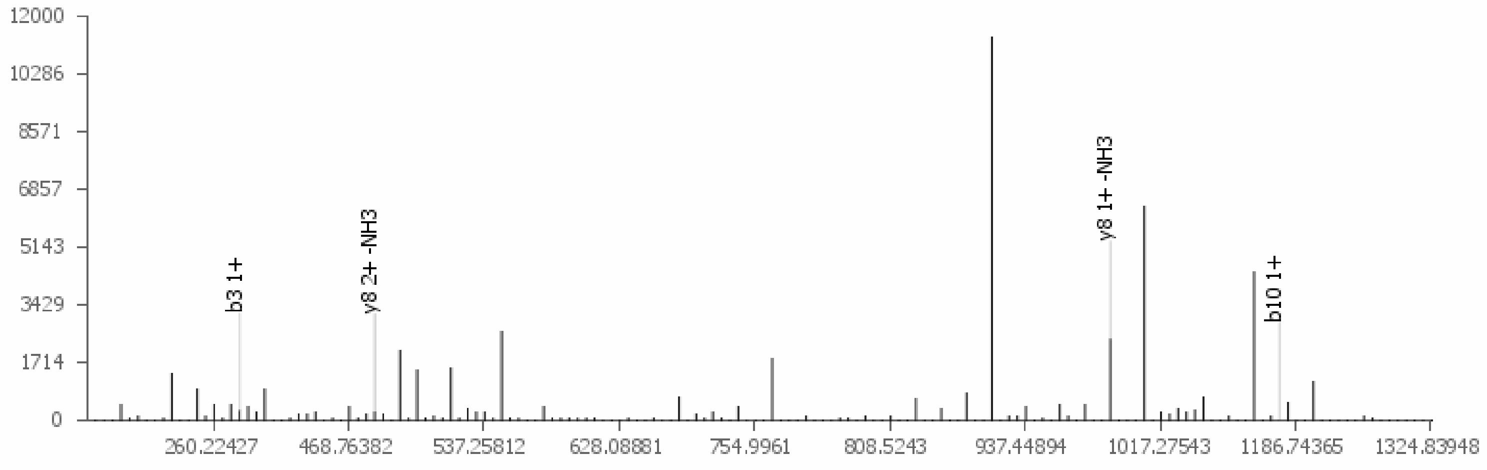

AT1G05690.1 - KP(s)(s)L(oxM)RLVPK - 676.361012 - Charge:2

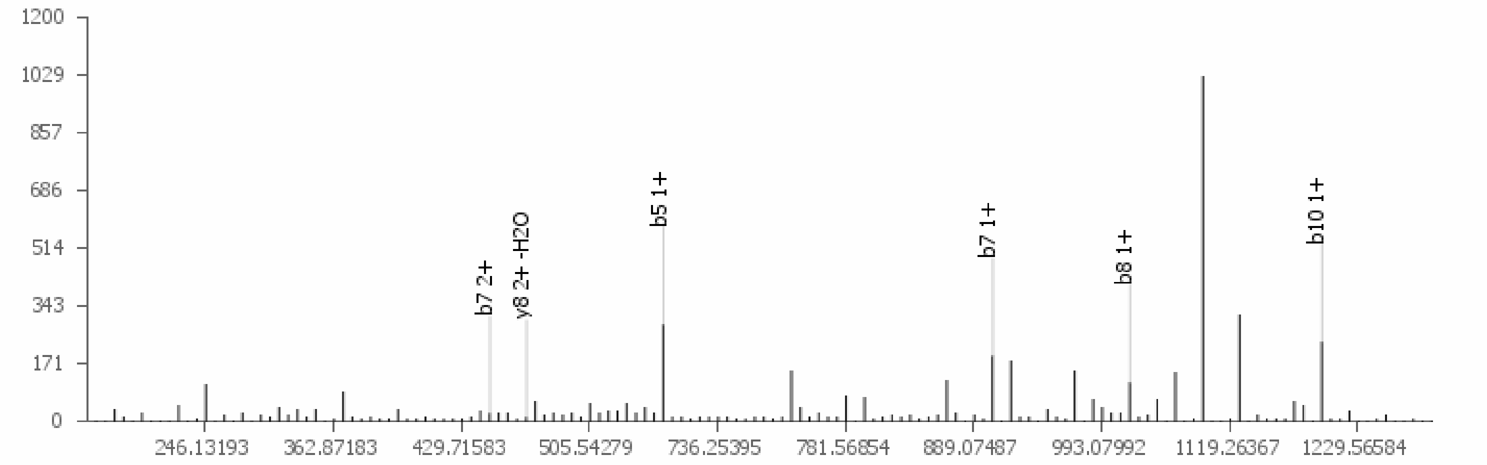

AT2G36370.1 - NFMLVK(pS)DK - 581.267567 - Charge:2

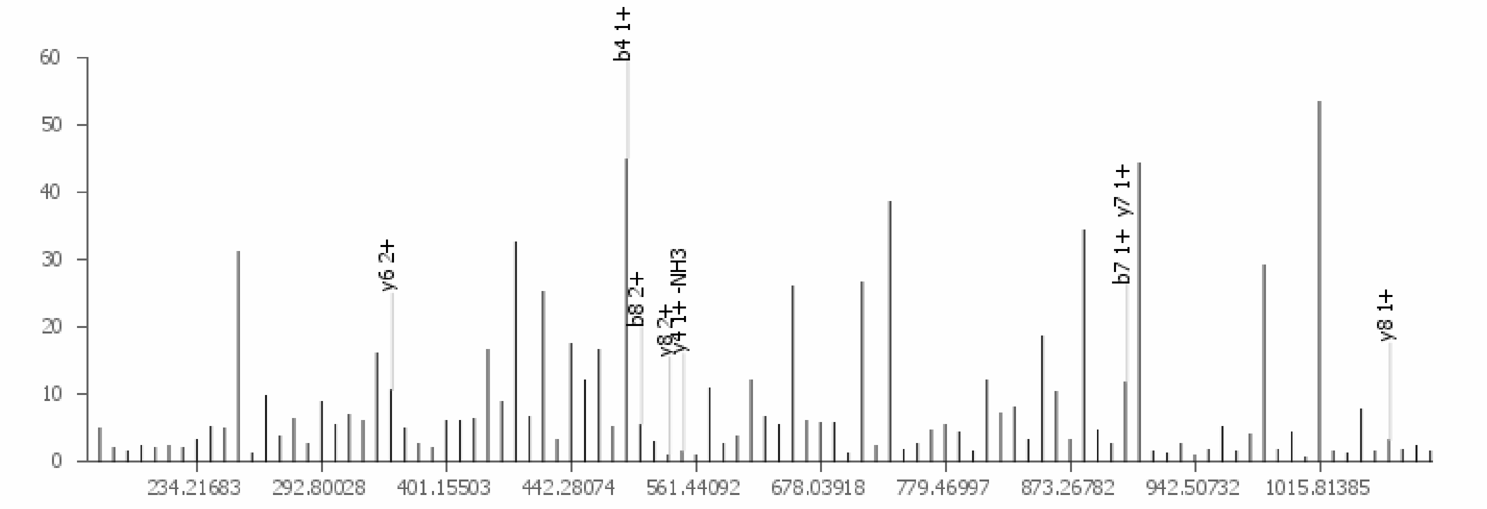

AT1G33770.1 - (pT)VIVERPSR - 568.796358 - Charge:2

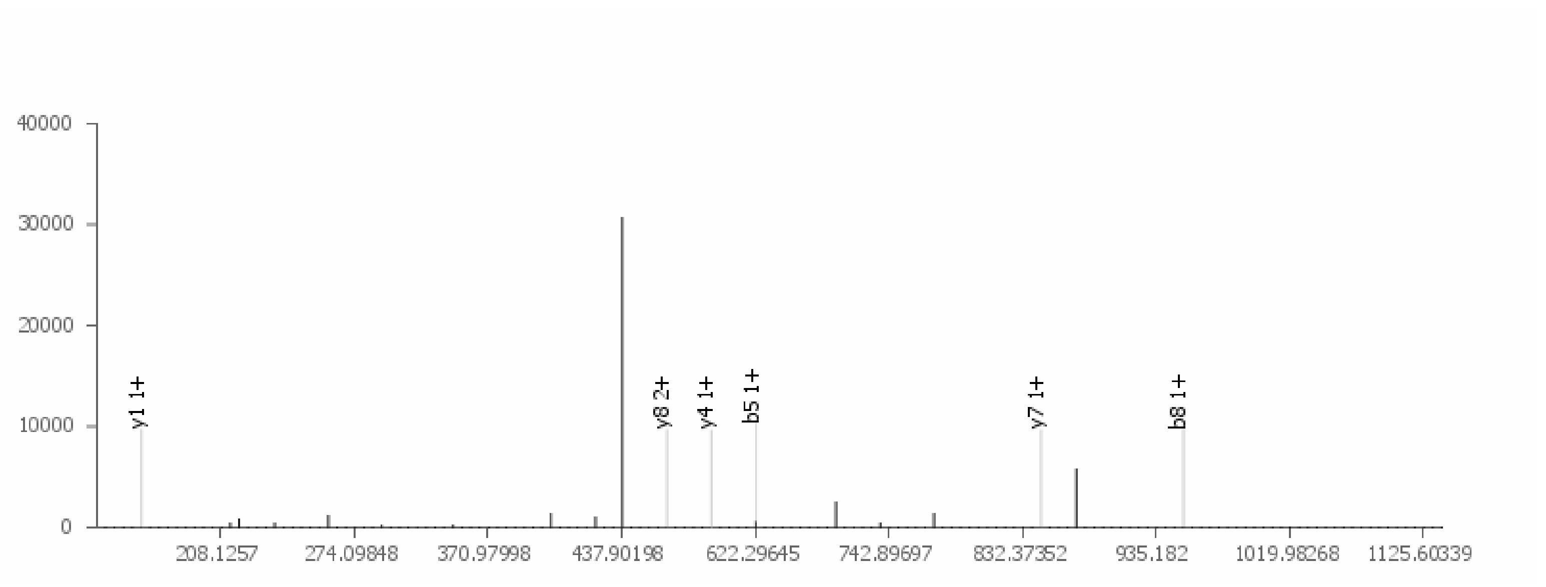

AT2G21940.1 - FRGL(pS)LAR - 500.264332 - Charge:2

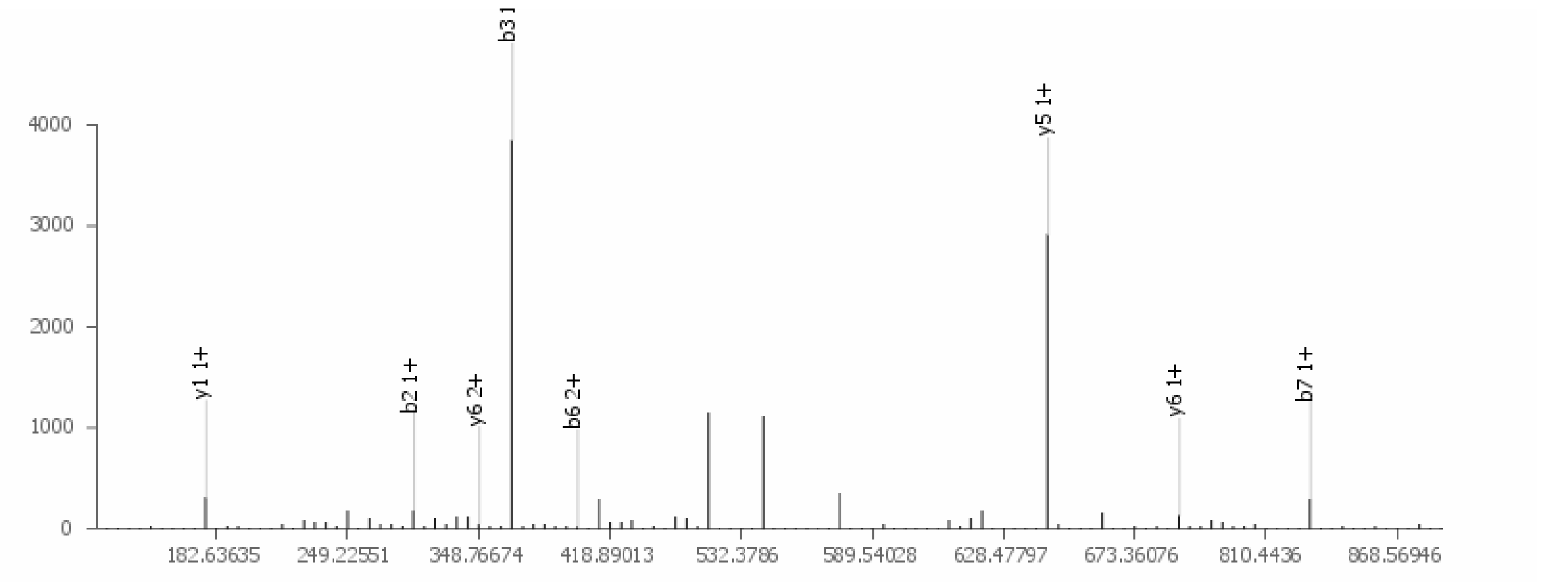

AT2G36840.1 - ELLG(pT)VKR - 498.263069 - Charge:2

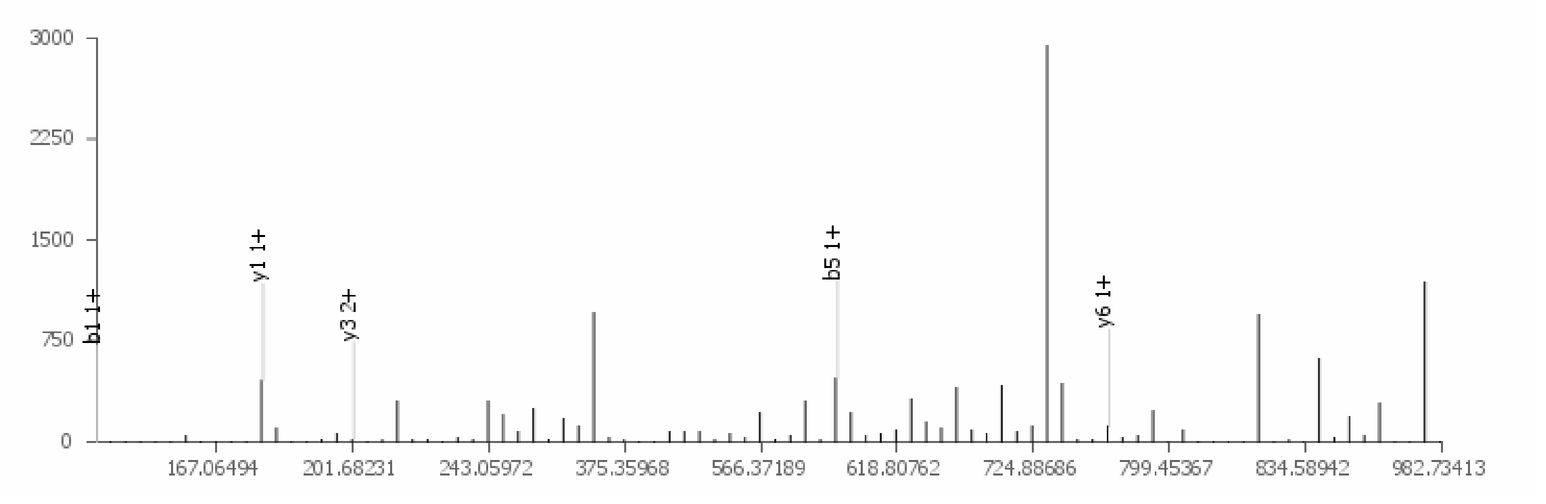

AT1G20110.1 - (pT)PVDIEAKR - 554.768717 - Charge:2

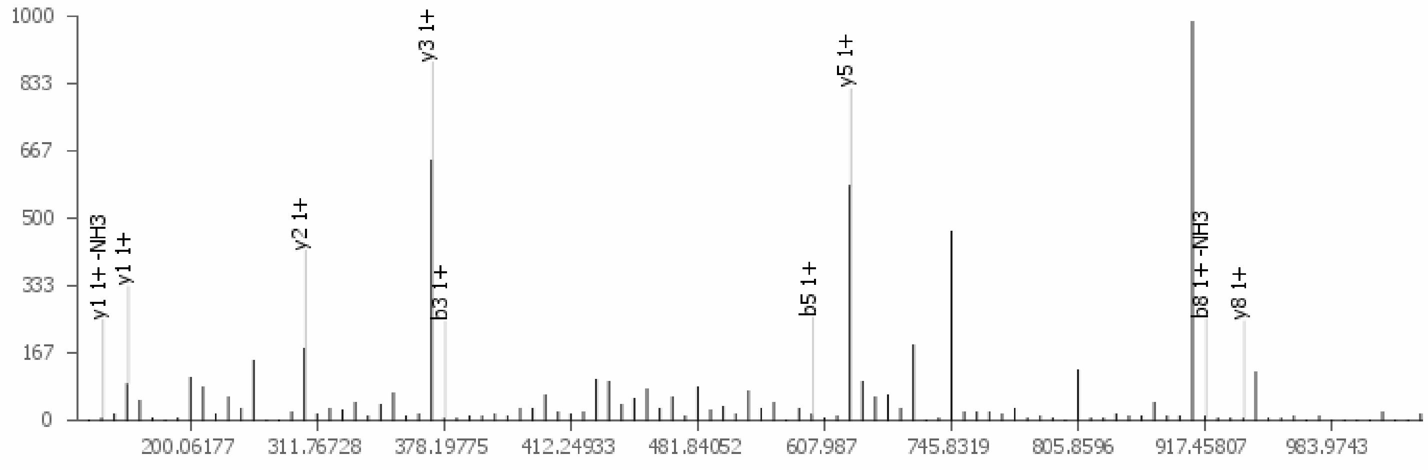

AT1G68580.2 - RTISLPVA(t)(t)K - 633.839535 - Charge:2

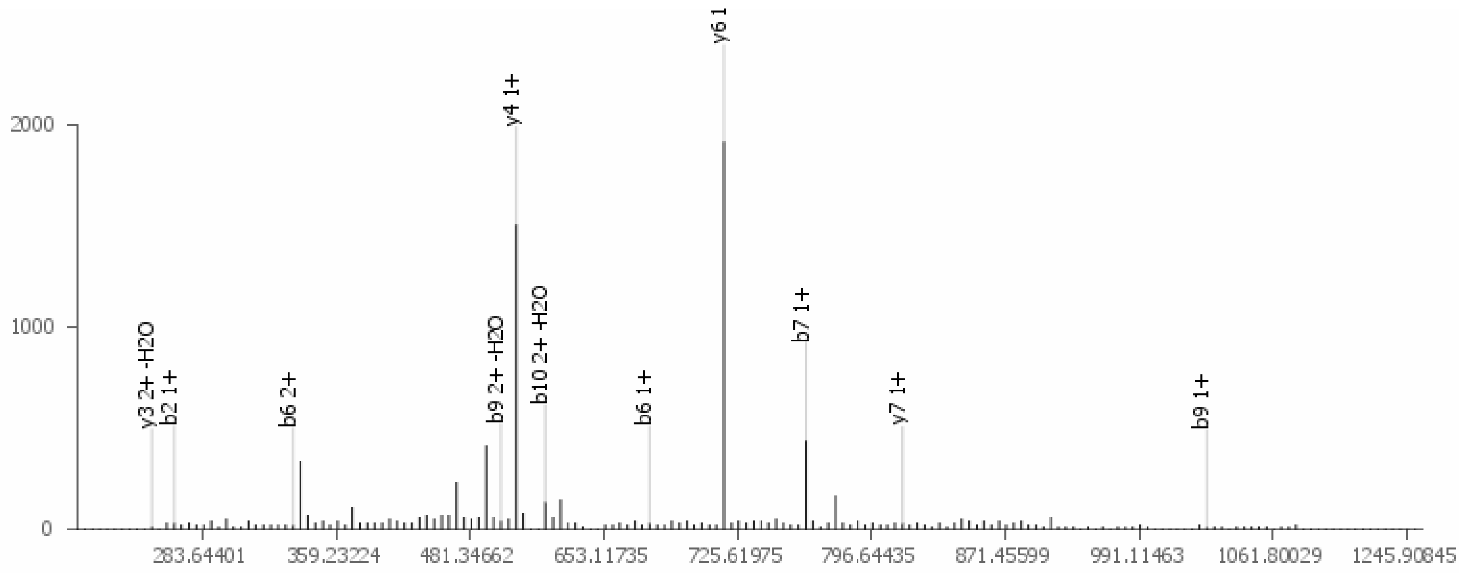

AT1G20160.1 - SAKSADASEG(pS)AR - 658.774055 - Charge:2

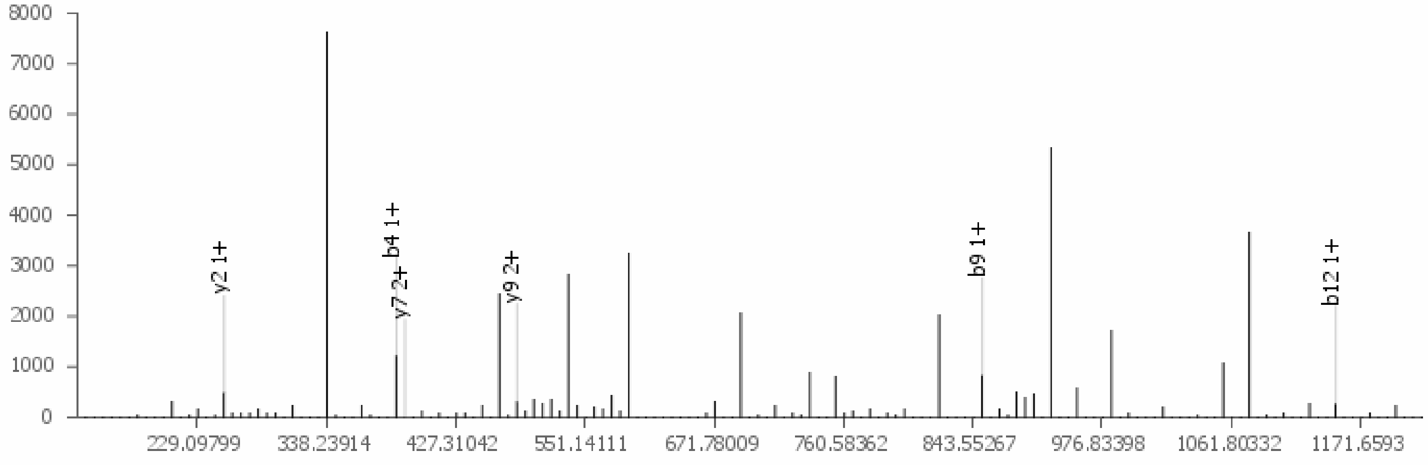

AT2G44530.1 - ASIVQP(s)P(t)FPALNLR - 895.965283 - Charge:2

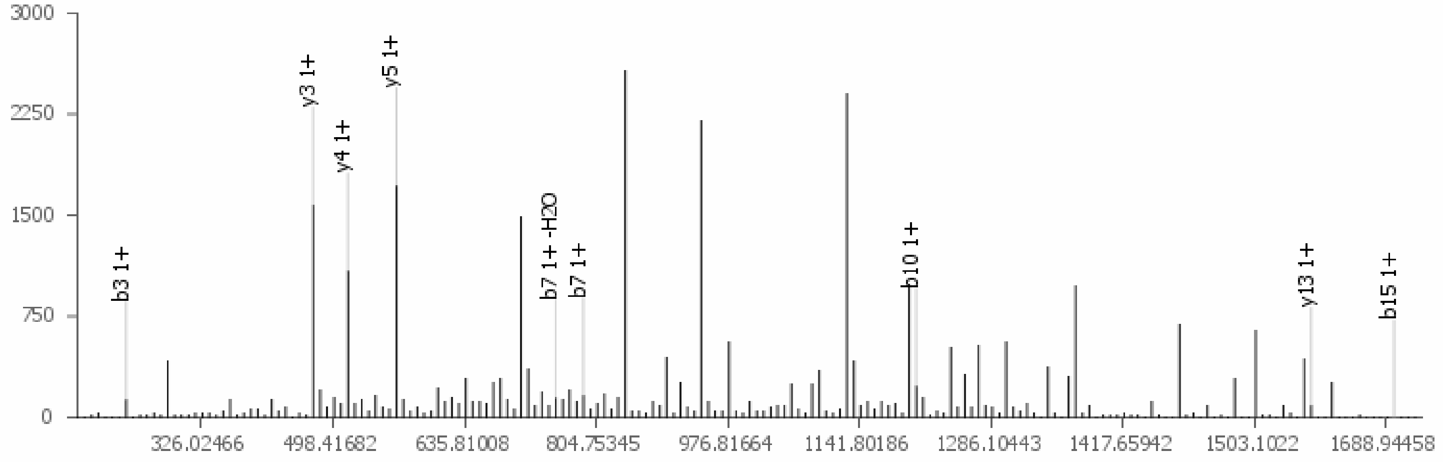

AT5G17020.1 - FLVMVIRDLLNLCEI(pT)K - 1050.543455 - Charge:2

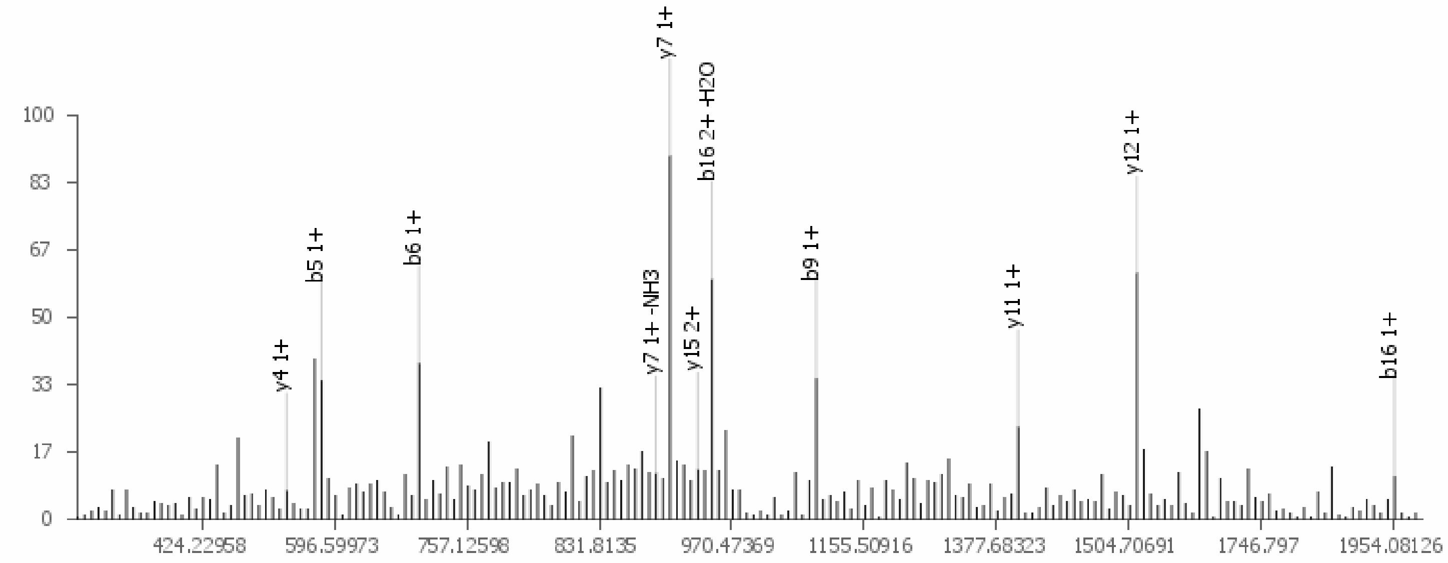

AT5G02010.1 - (pT)NSKPATNTK - 571.269313 - Charge:2

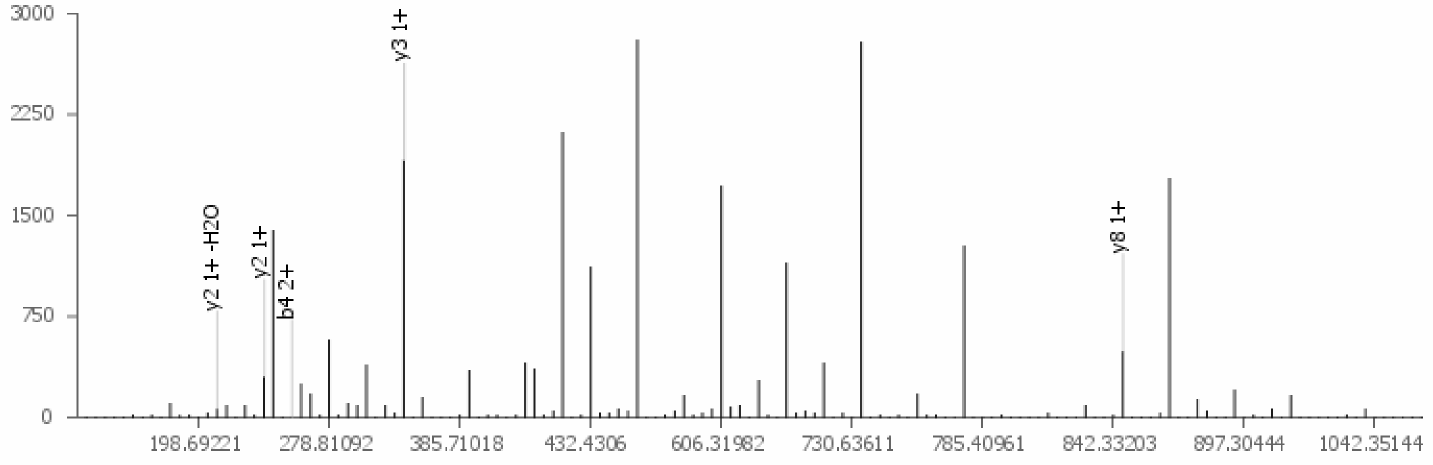

AT2G31660.1 - LICKIFW(s)(s)IYLELPR - 1031.042943 - Charge:2

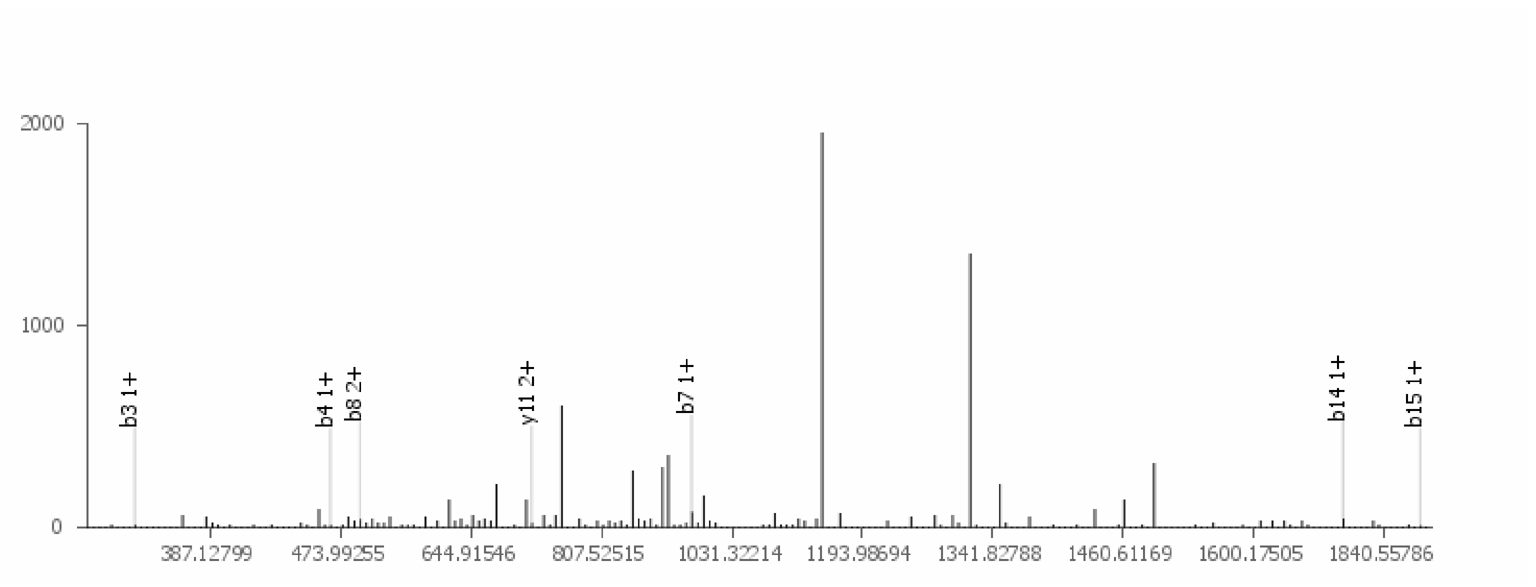

AT3G54180.1 - LLGTP(pT)EQQWPGVSTLRDWHVYPK - 963.480591 - Charge:3

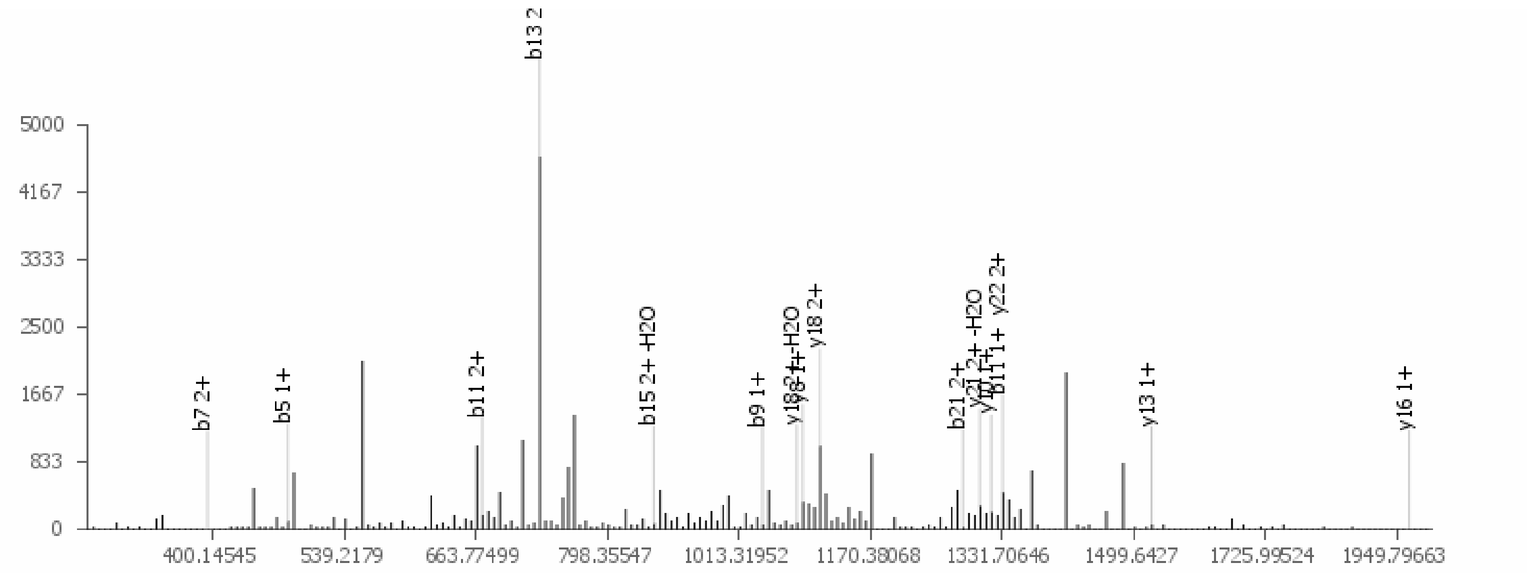

AT2G26510.1 - INEL(oxM)P(pT)RFL - 665.317789 - Charge:2

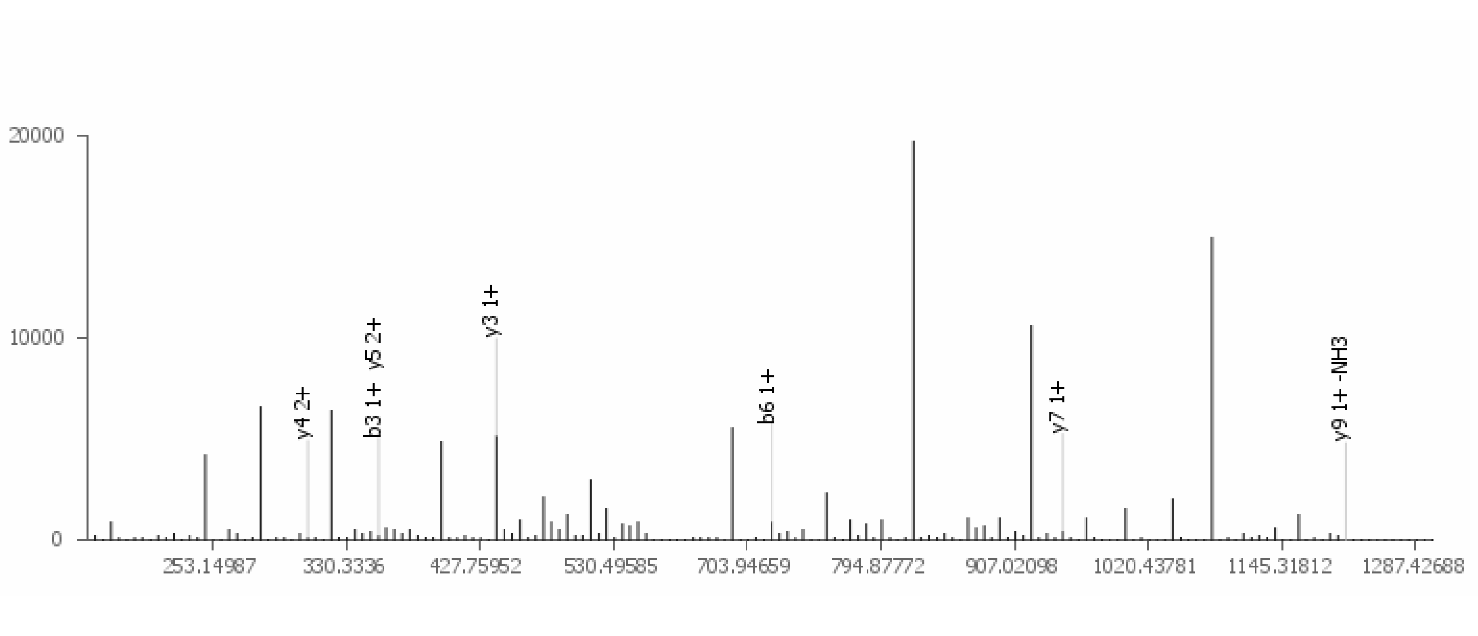

AT4G13250.1 - VENLE(oxM)VF(s)(s)VAVQIAR - 994.491449 - Charge:2

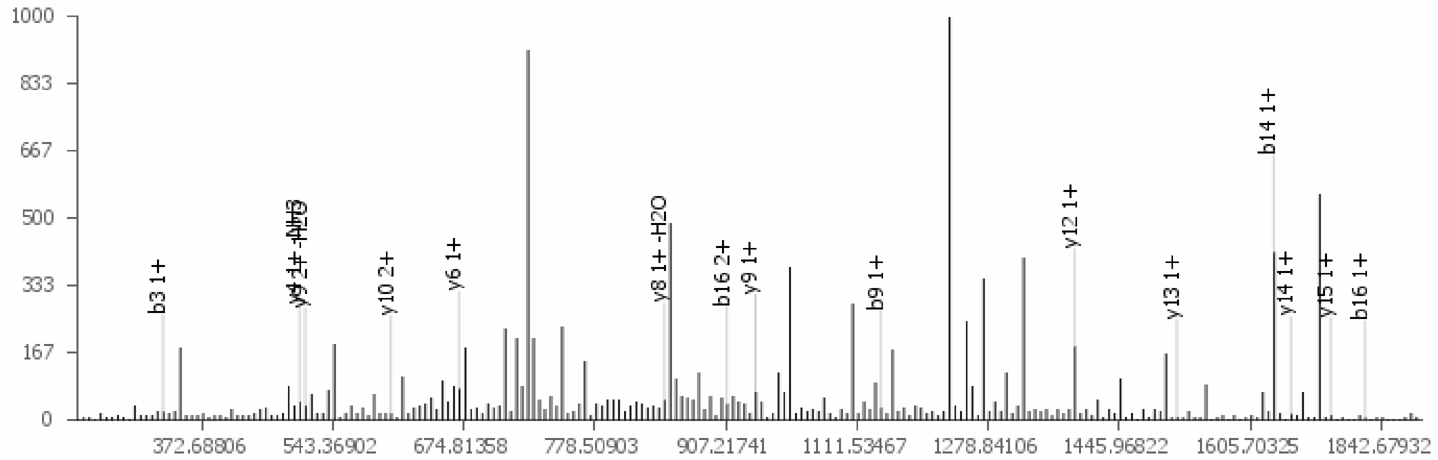

AT1G31950.1 - IITKA(pY)QEIAK - 679.358913 - Charge:2

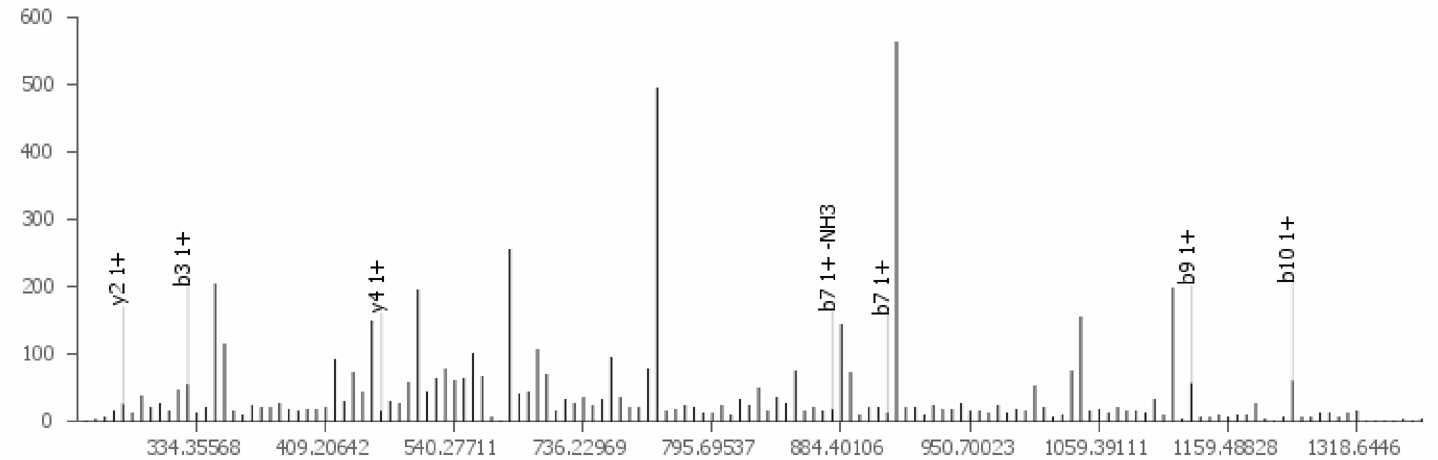

AT3G53280.1 - KV(pS)LELVPTLHR - 491.273917 - Charge:3

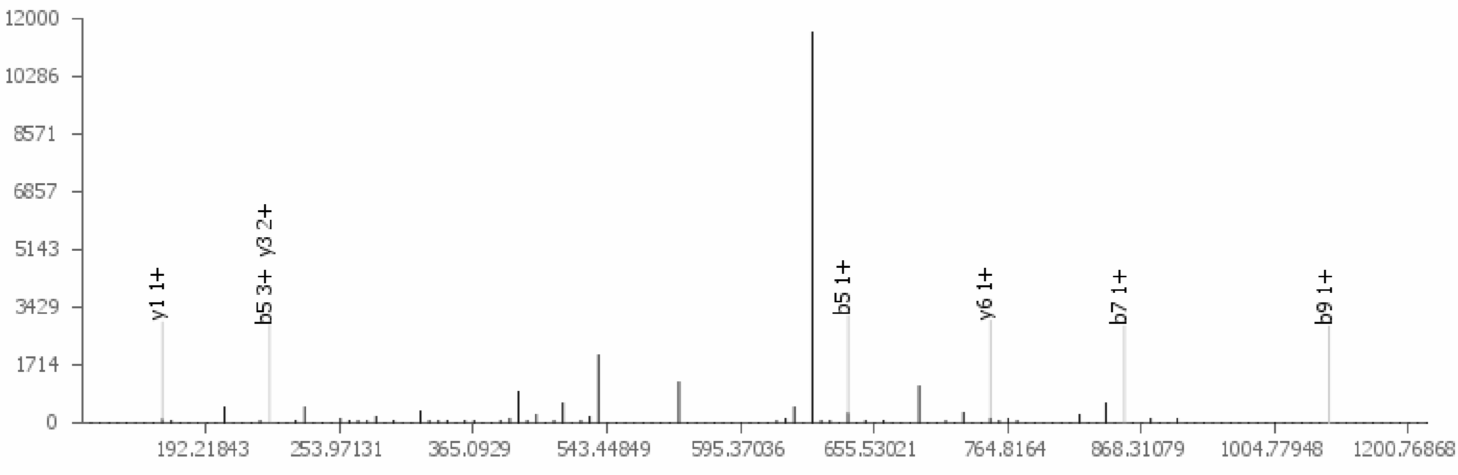

AT1G33490.1 - ITGGPHFPL(pT)SDALKK - 881.446285 - Charge:2

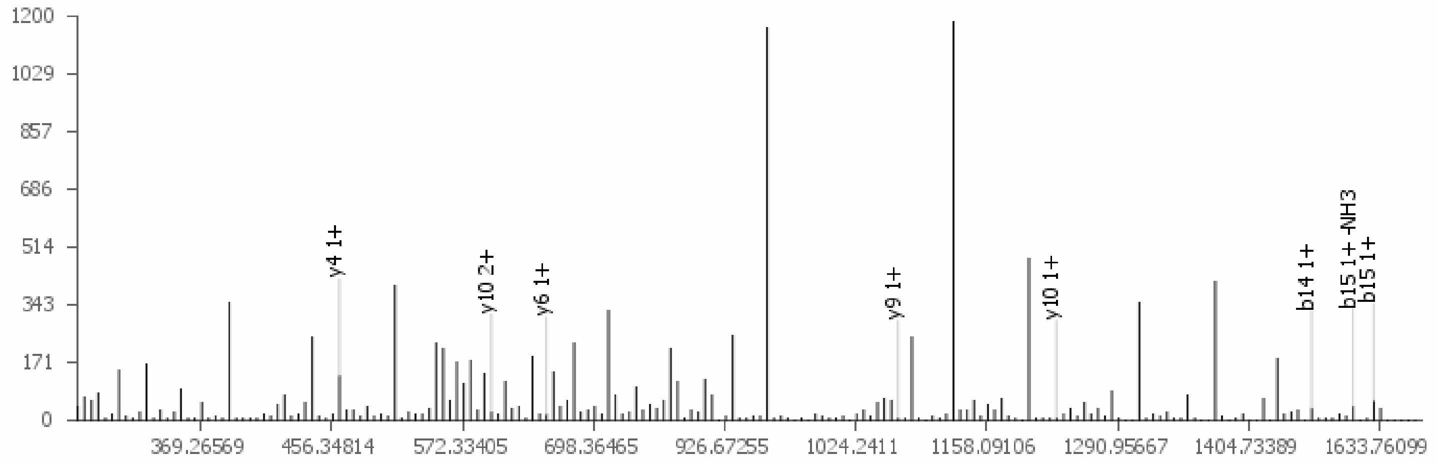

AT5G16850.1 - FI(t)I(s)VR - 458.241563 - Charge:2

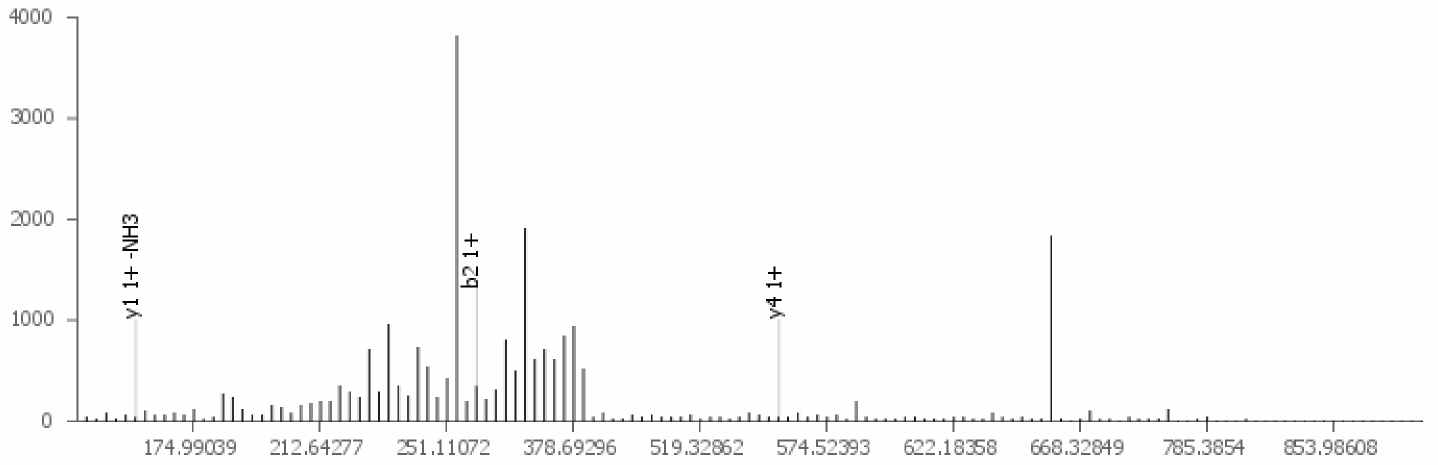

AT1G61105.1 - QRLLTPC(pS)K - 591.794483 - Charge:2

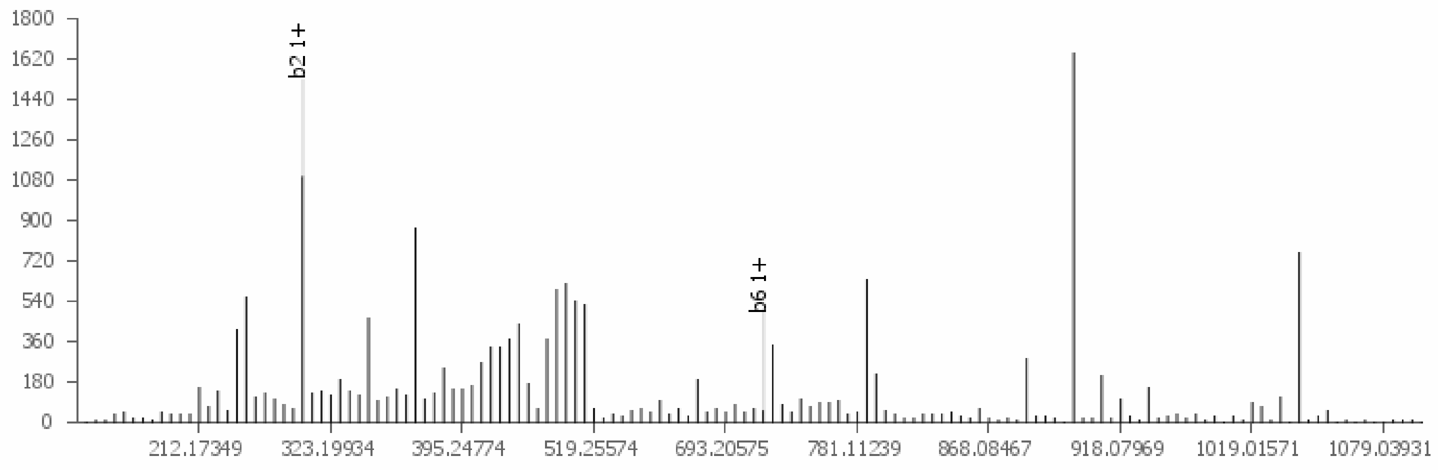

AT1G62610.1 - VVLVTGAS(pS)GIGR - 648.340523 - Charge:2

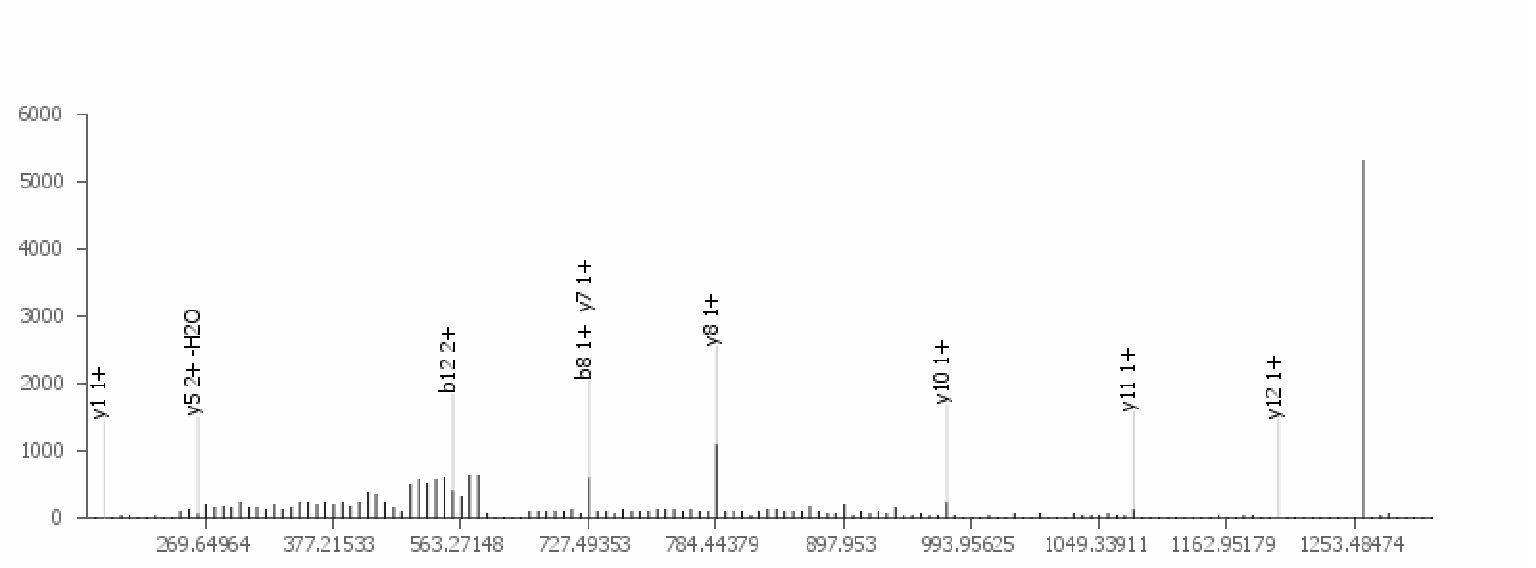

AT3G22950.1 - DAM(pT)PAEITDALNLHSIK - 1010.48397 - Charge:2

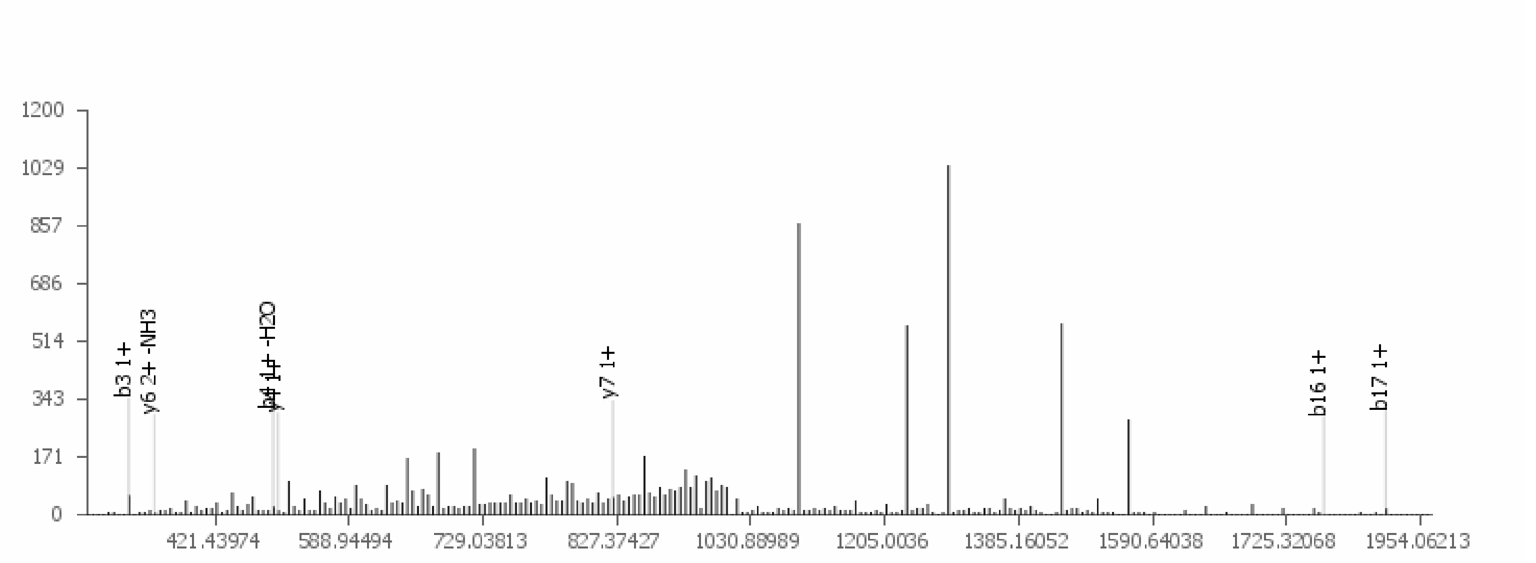

AT1G16800.1 - EQWF(pT)DAFTLLISLPK - 663.667888 - Charge:3

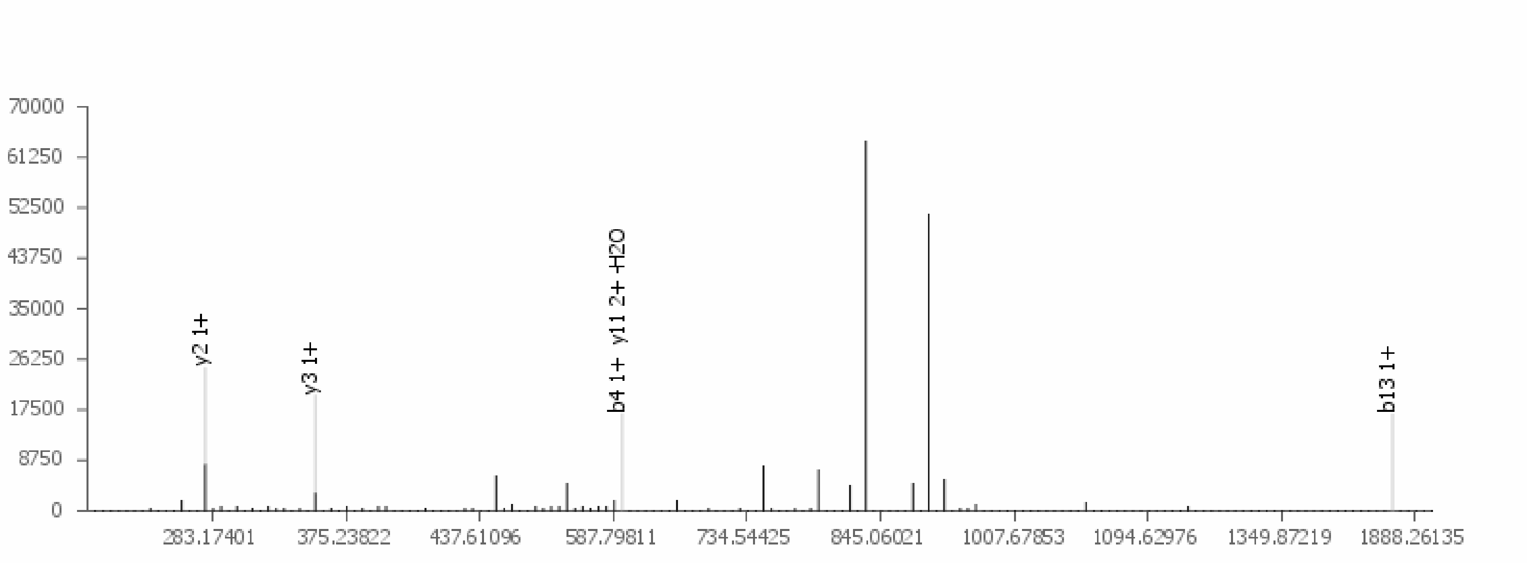

AT3G22790.1 - VE(pT)LEEILK - 577.289312 - Charge:2

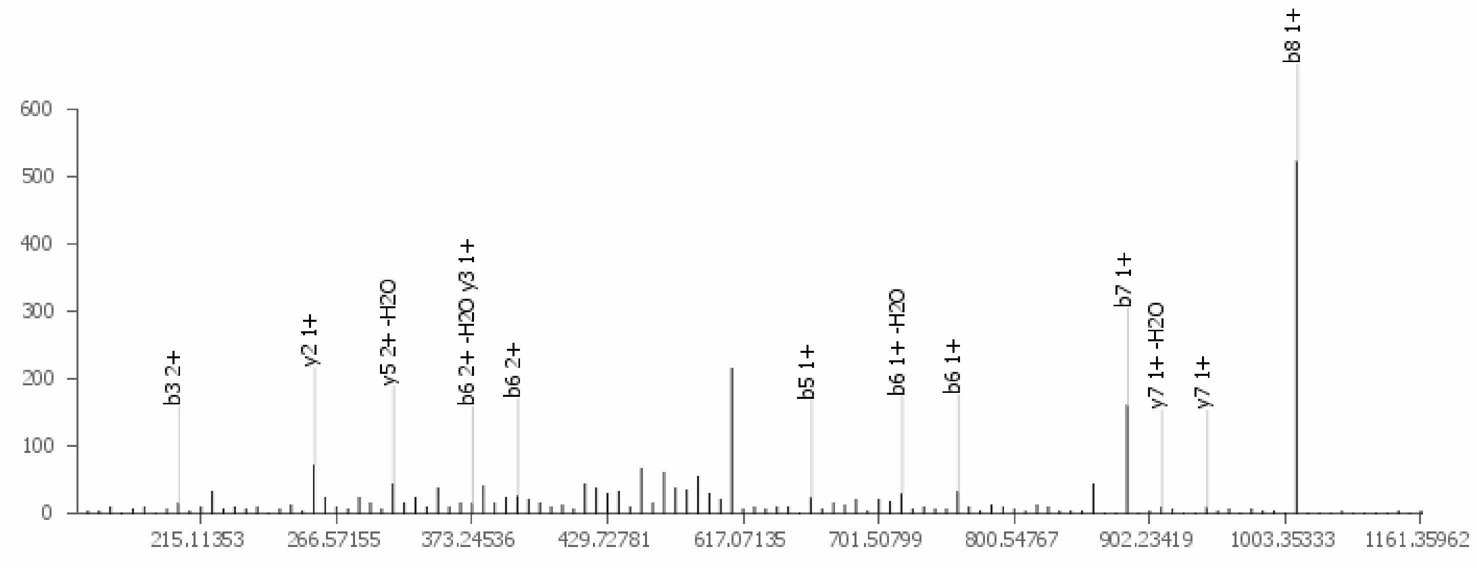

AT5G15350.1 - (pT)DYEGCIADHPIR - 785.318047 - Charge:2

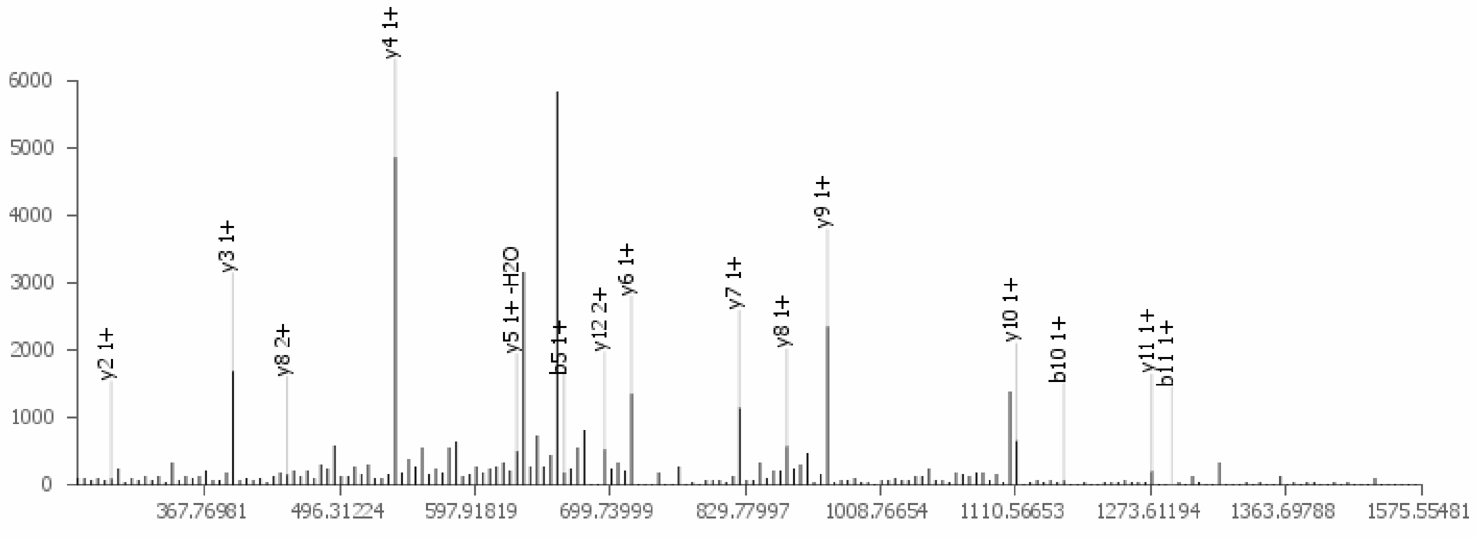

AT1G06970.1 - RSVLMSY(pT)WR - 689.817077 - Charge:2

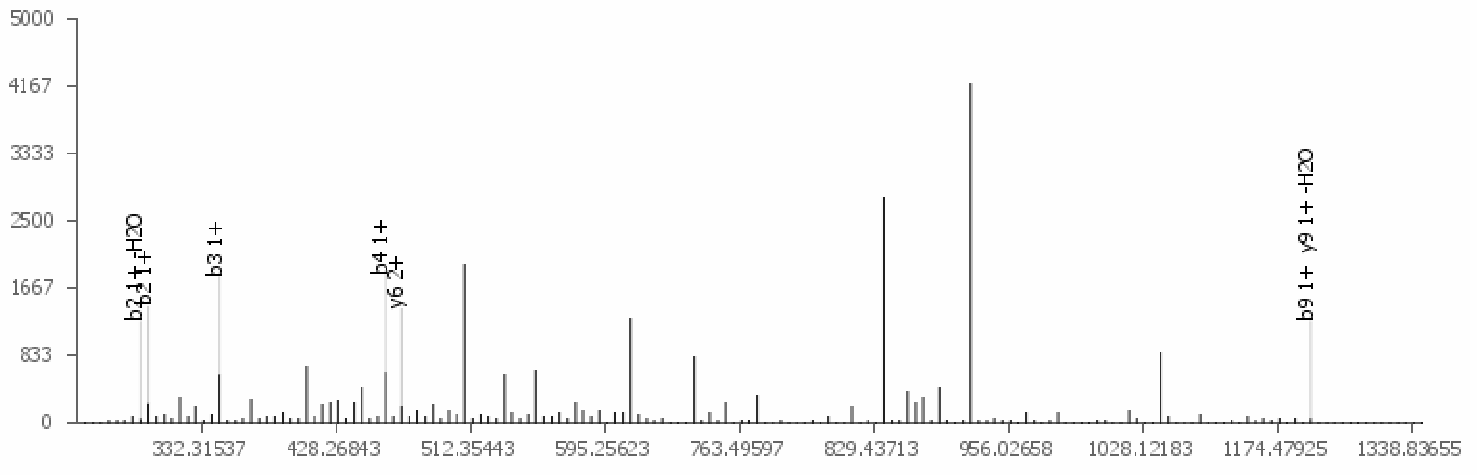

AT5G59250.1 - LVDDAYL(pS)VK - 601.789682 - Charge:2

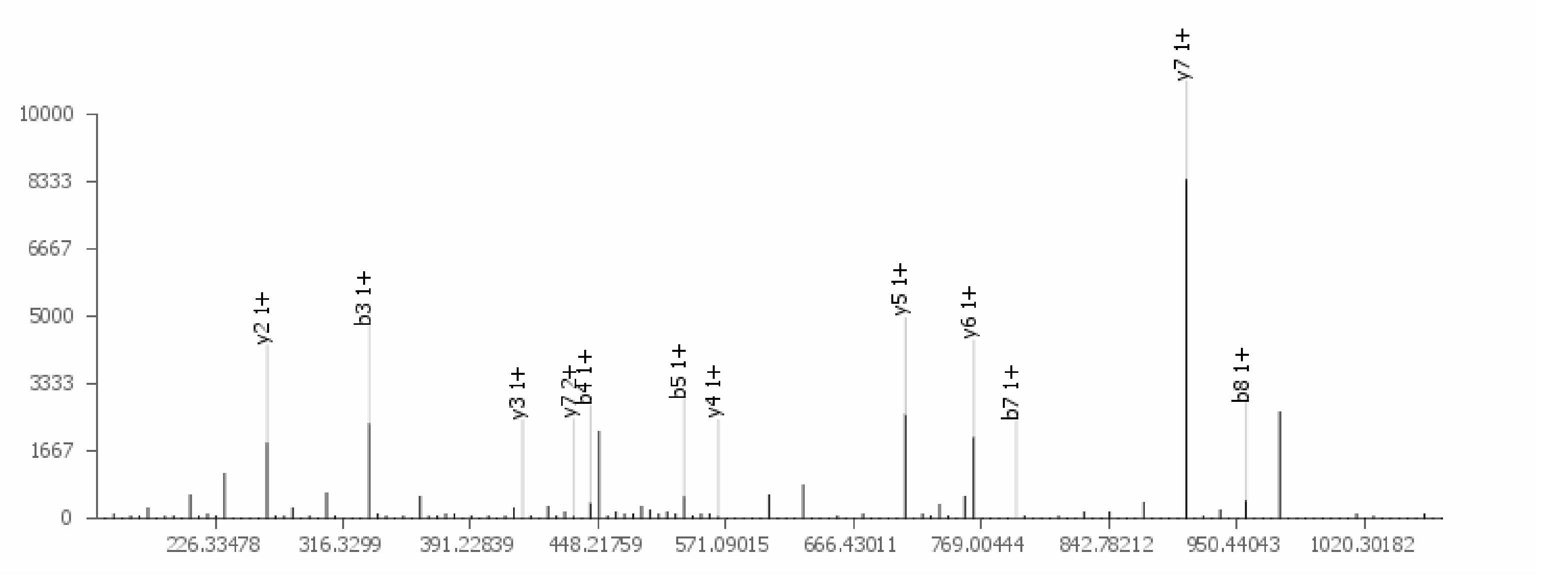

AT1G09600.1 - IFKLCGSPSEE(pY)WK - 883.895043 - Charge:2

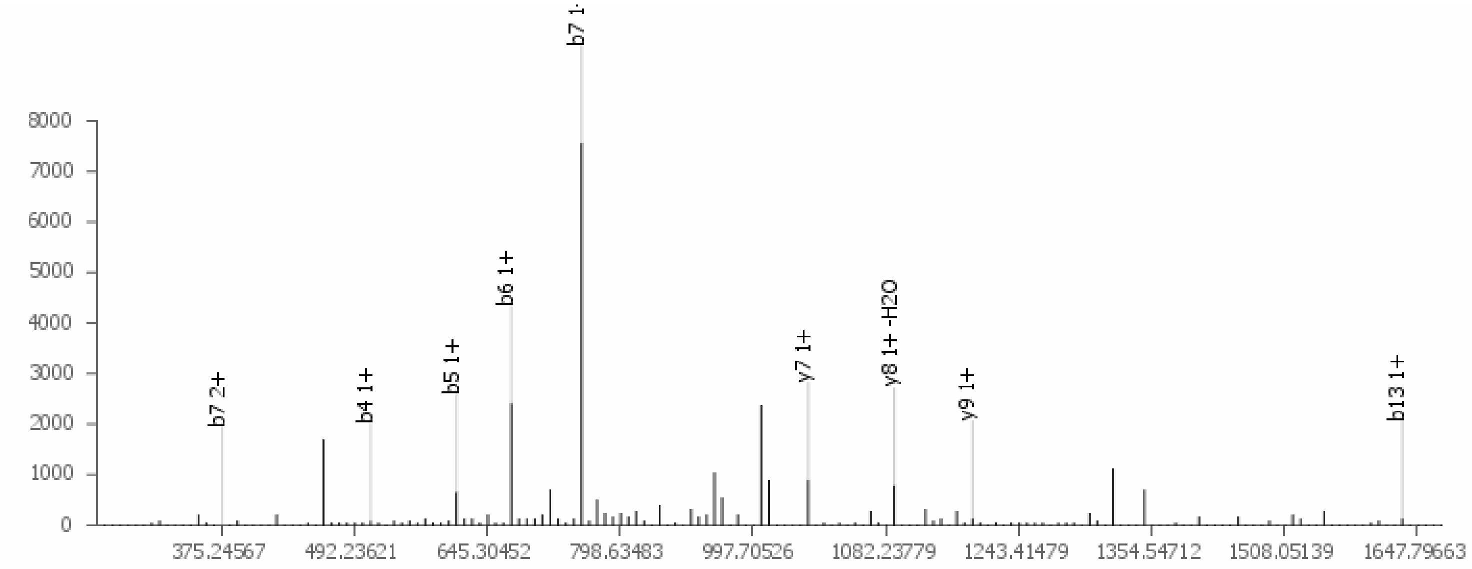

AT1G55890.1 - SSL(pS)RVLR - 499.258534 - Charge:2

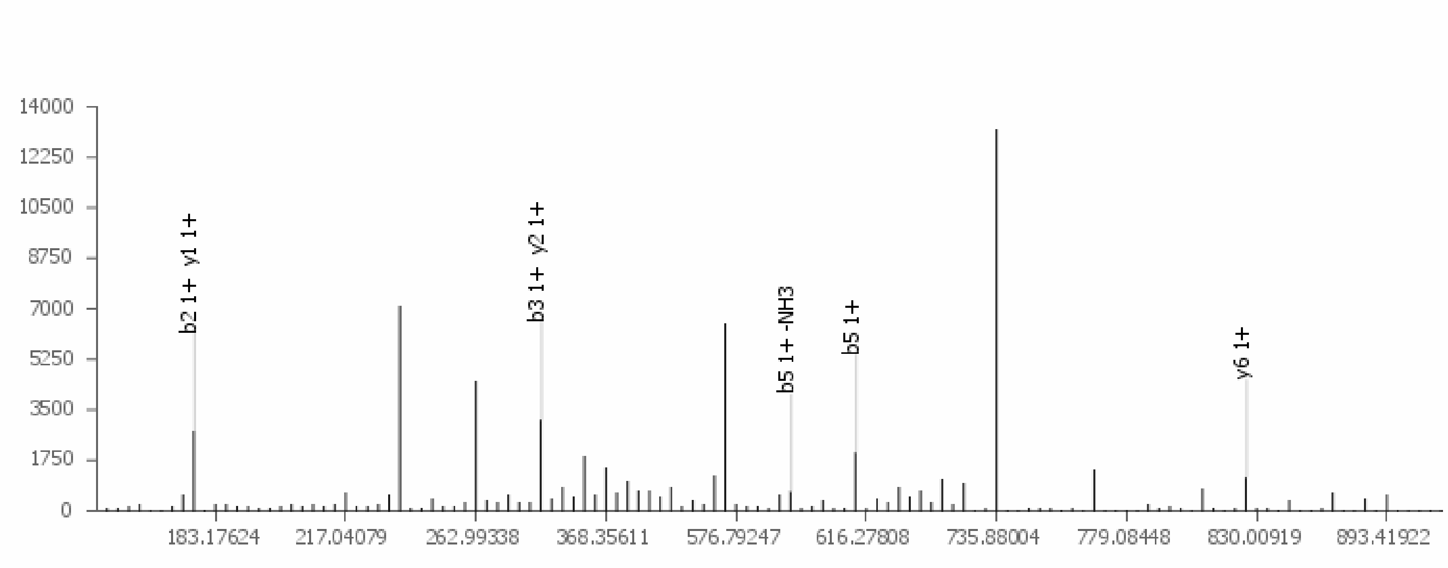

AT2G26780.1 - VLEIL(pS)HVNKR - 694.37885 - Charge:2

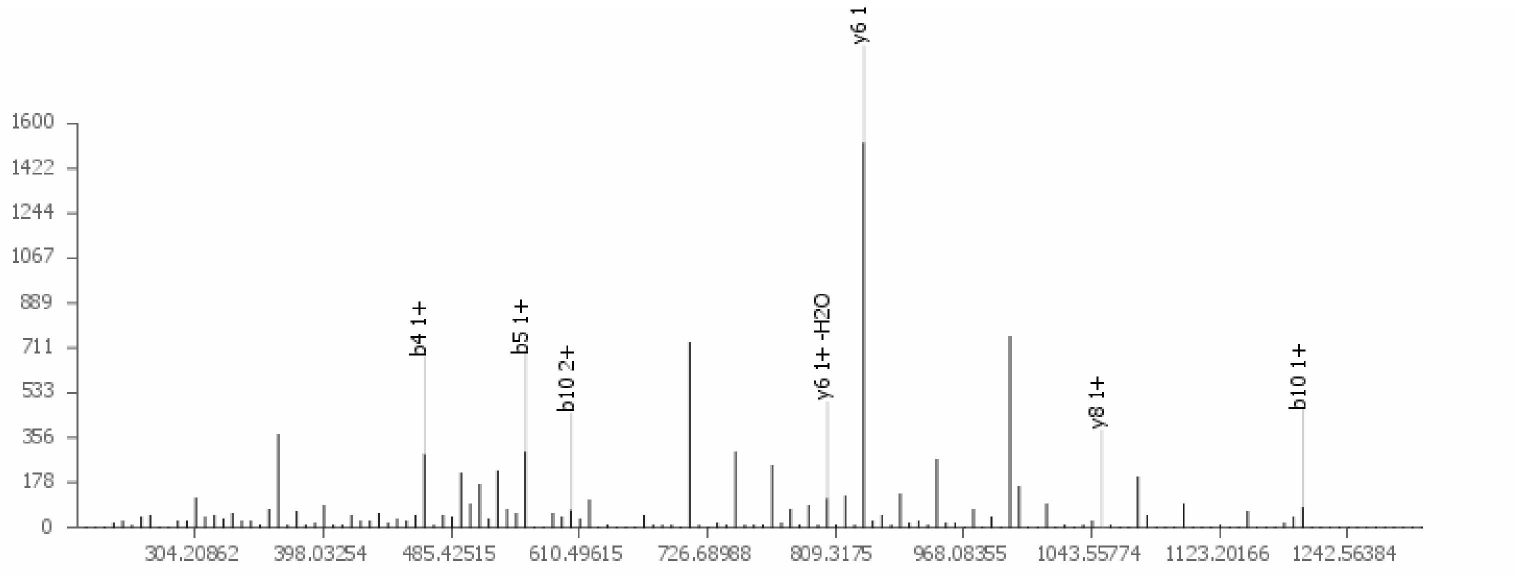

AT5G24450.1 - DRPDNLVLRLPVTS(pS)PK - 994.019841 - Charge:2

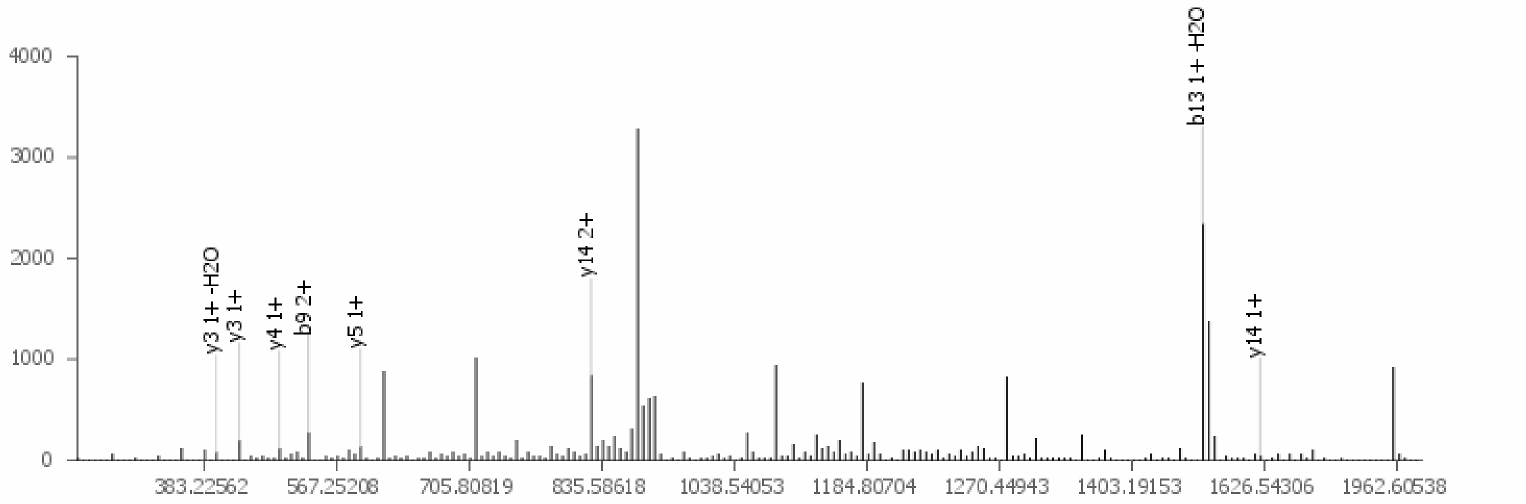

AT3G26910.1 - NV(pY)E(oxM)SLVK - 589.767179 - Charge:2

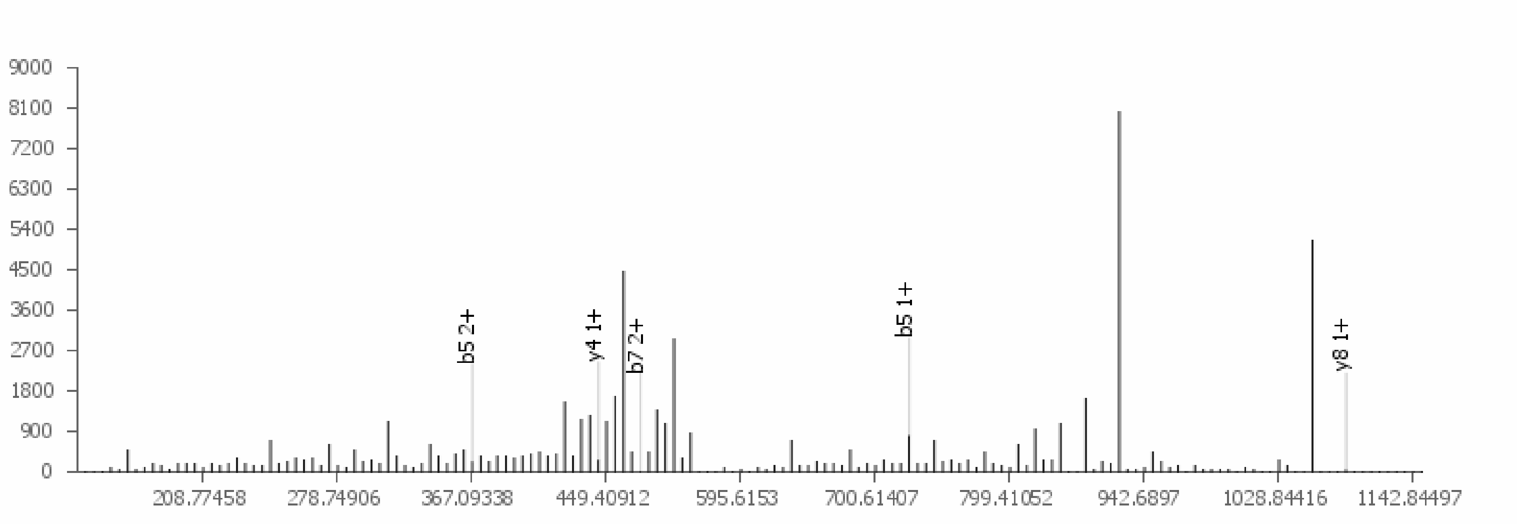

AT1G59870.1 - ISGYCEQ(pT)DIHSPQVTVR - 1056.968617 - Charge:2

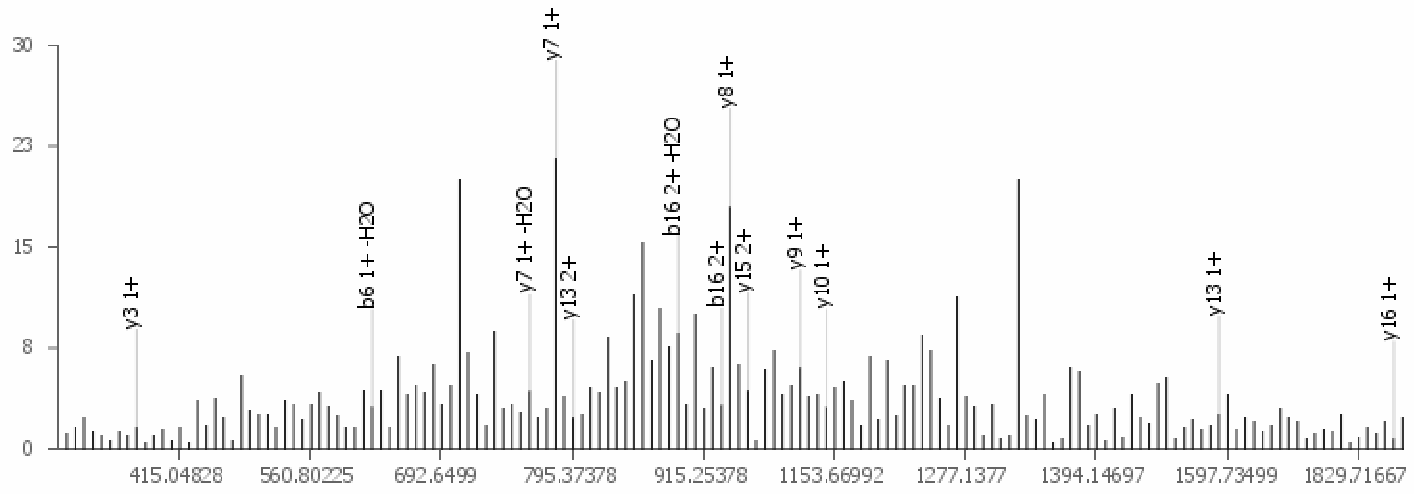

AT2G24700.1 - FVTL(pT)LTH(pS)SK - 697.312191 - Charge:2

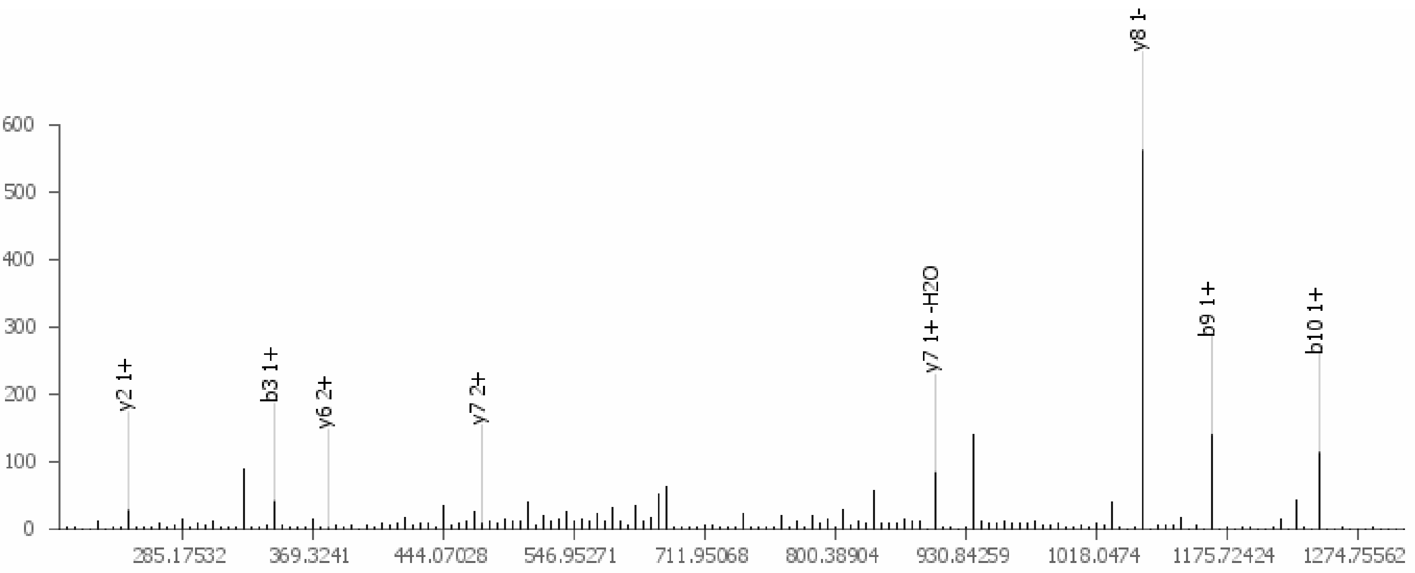

AT1G02090.1 - VLPYD(pT)LMVELDV(pS)NVR - 1061.983443 - Charge:2

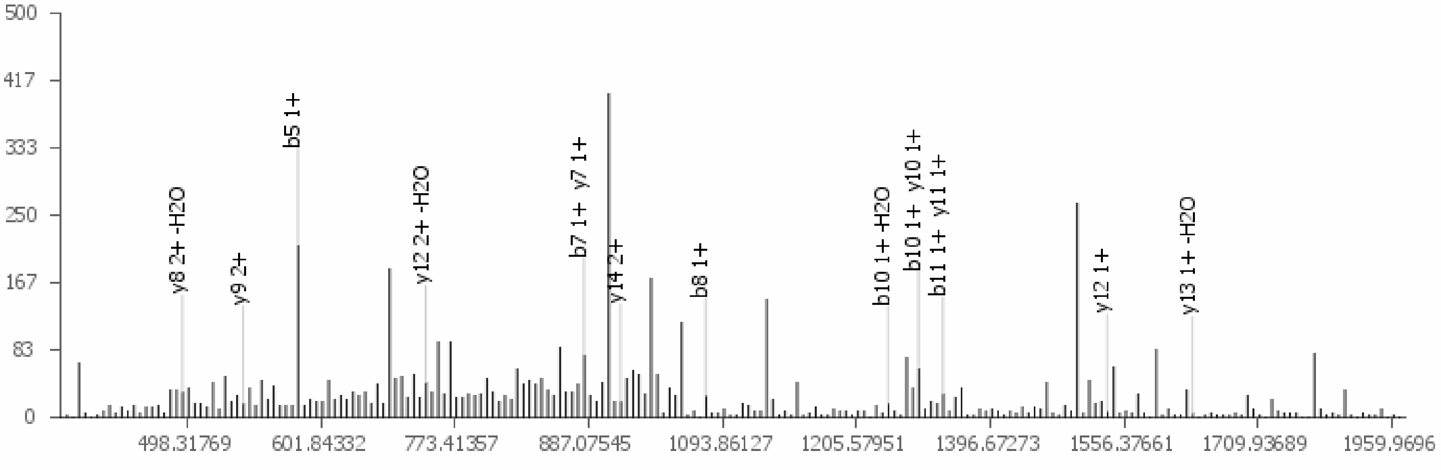

AT3G09530.1 - LLDEMRI(pS)IGR - 461.570655 - Charge:3

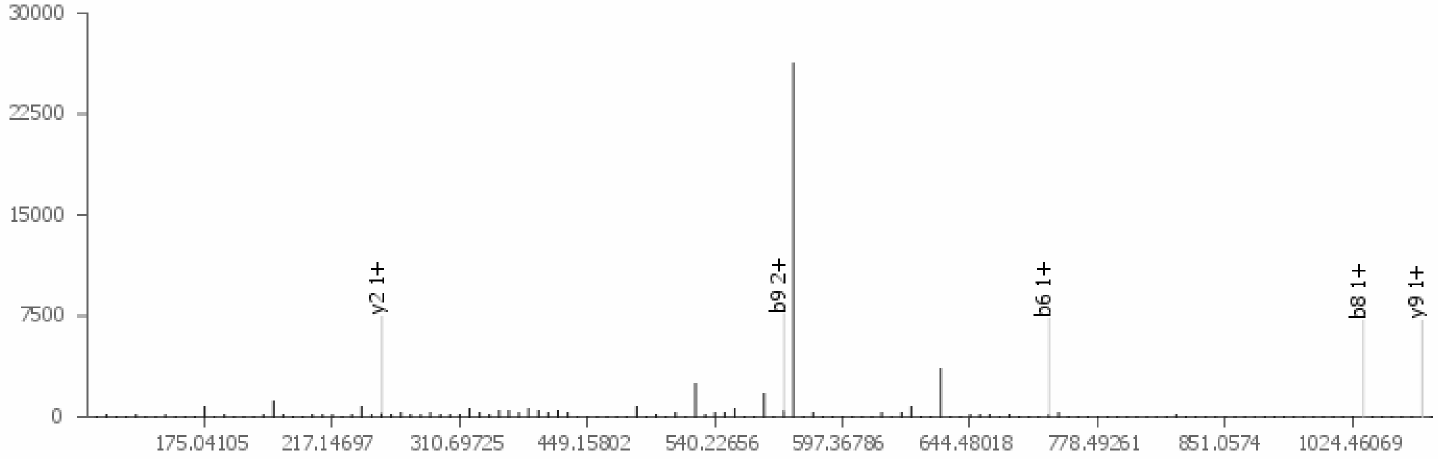

AT1G52780.1 - LEDISR(pT)PR - 583.787134 - Charge:2

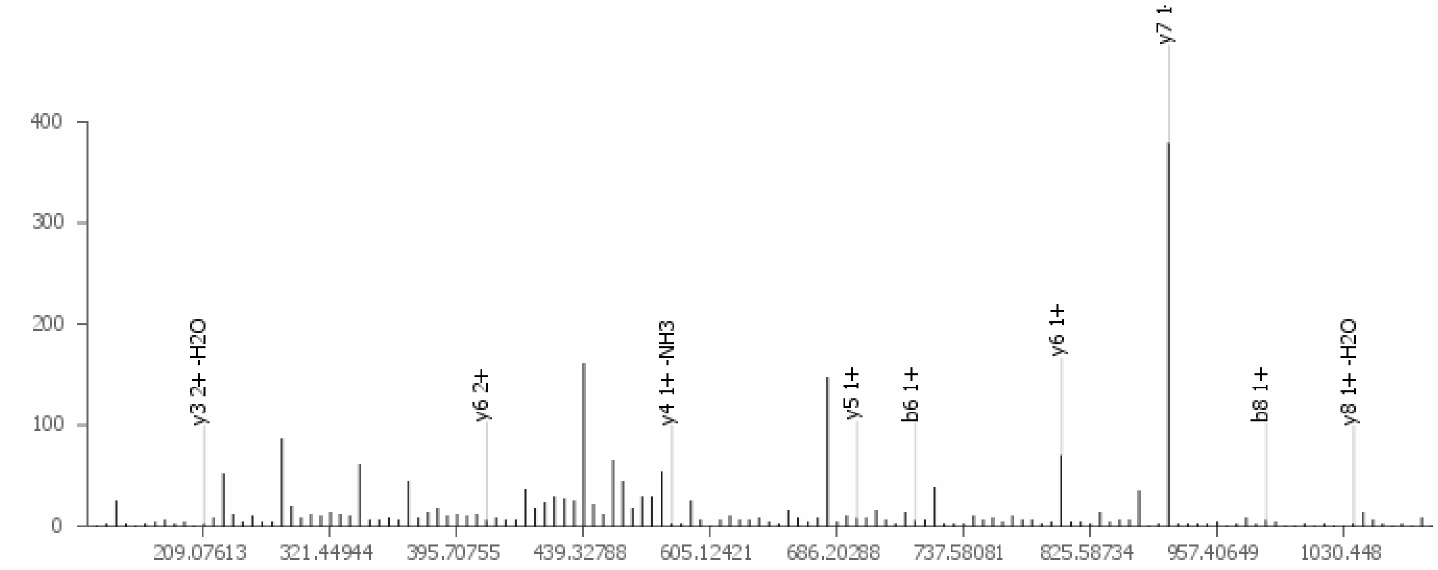

AT5G19390.1 - (oxM)EA(pS)LAALERPRGSASNTVFK - 777.705147 - Charge:3

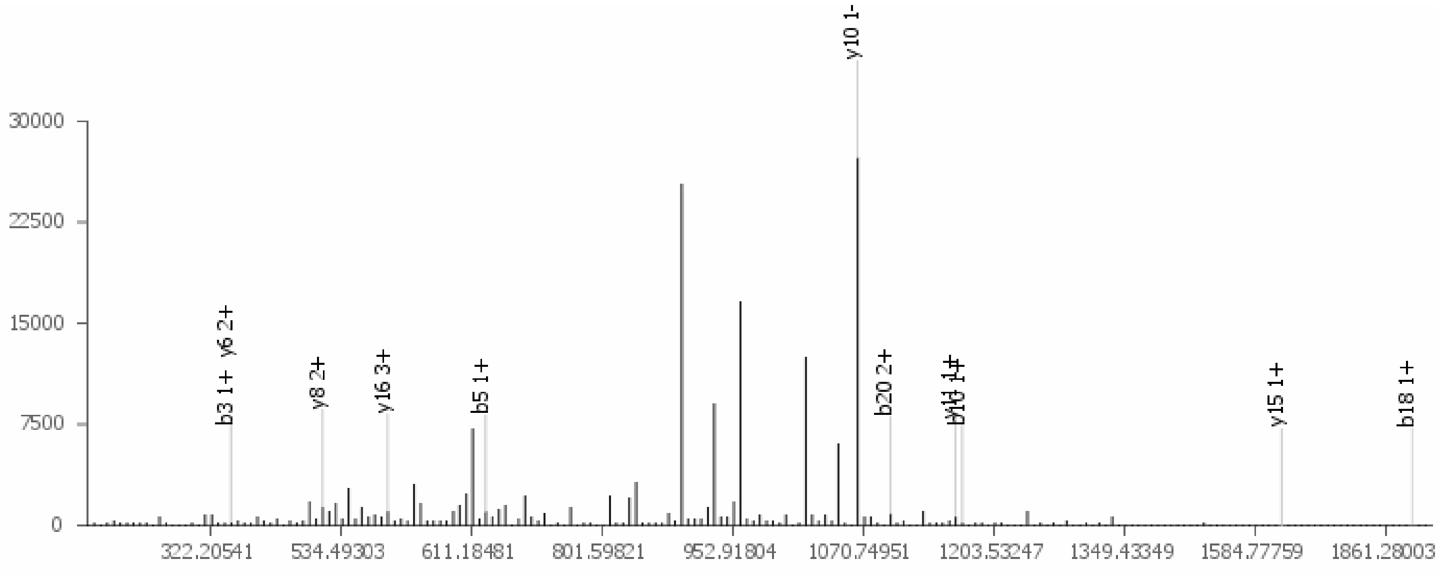

AT3G25480.1 - (pY)KPISAMNAFR - 689.325053 - Charge:2

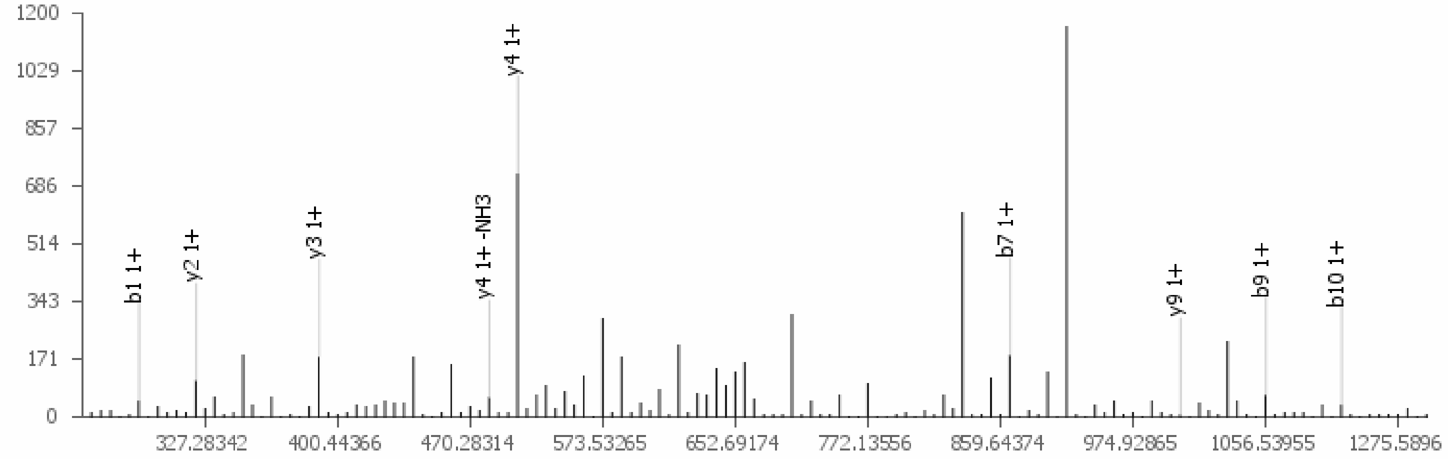

AT3G07750.1 - IGGTDVIA(pS)VK - 570.283406 - Charge:2

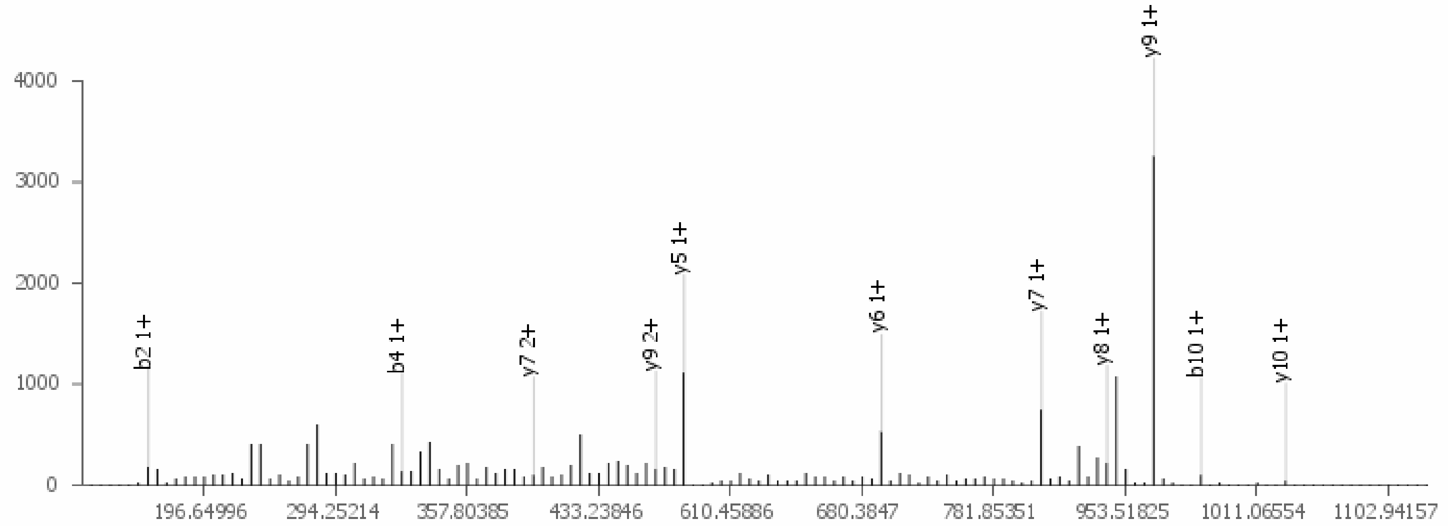

AT3G51140.1 - LNNRN(pS)AWPVLK - 746.372611 - Charge:2

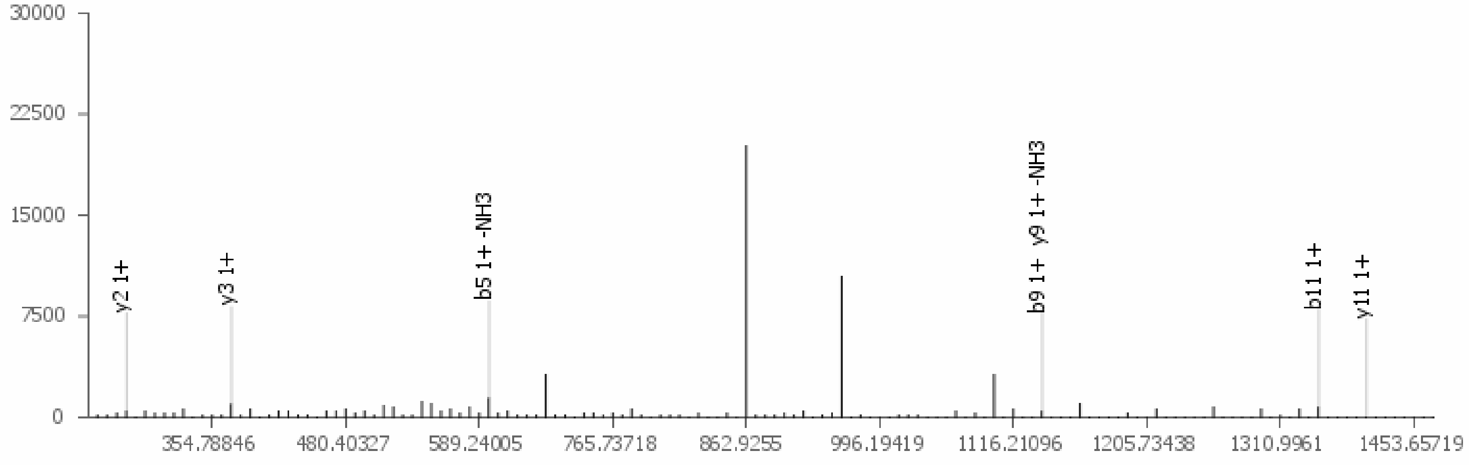

AT1G26170.1 - TE(t)(t)TLVTPVLK - 691.861251 - Charge:2

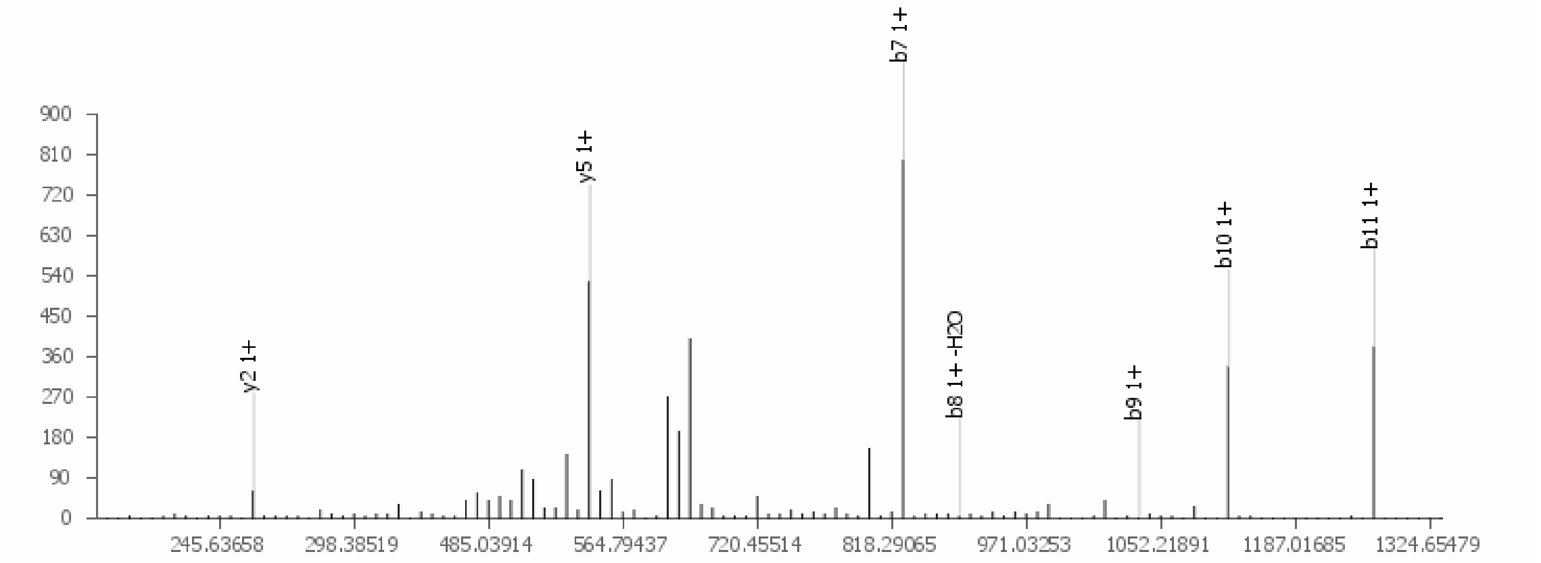

AT5G52410.2 - VEV(oxM)V(t)TV(t)K - 601.789476 - Charge:2

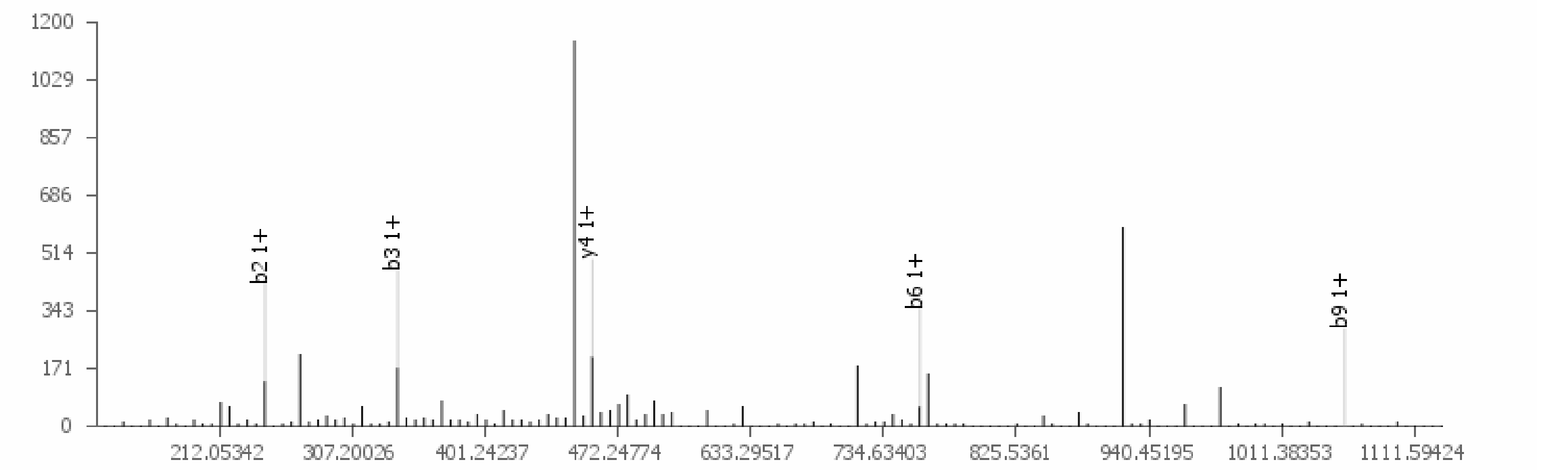

AT4G17130.1 - GVSV(pT)INR(pS)LR - 681.325662 - Charge:2

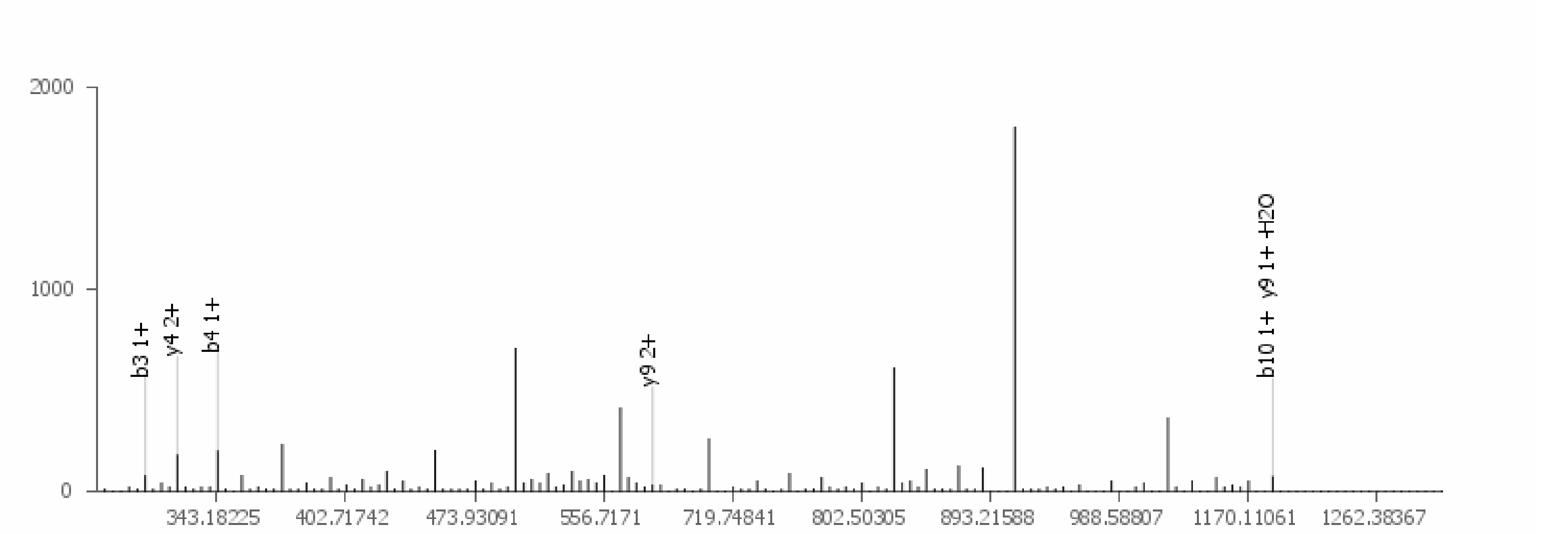

AT1G62560.1 - VPDPFENEVVVVIGNFA(pS)GADISRDIAK - 1013.498086 - Charge:3

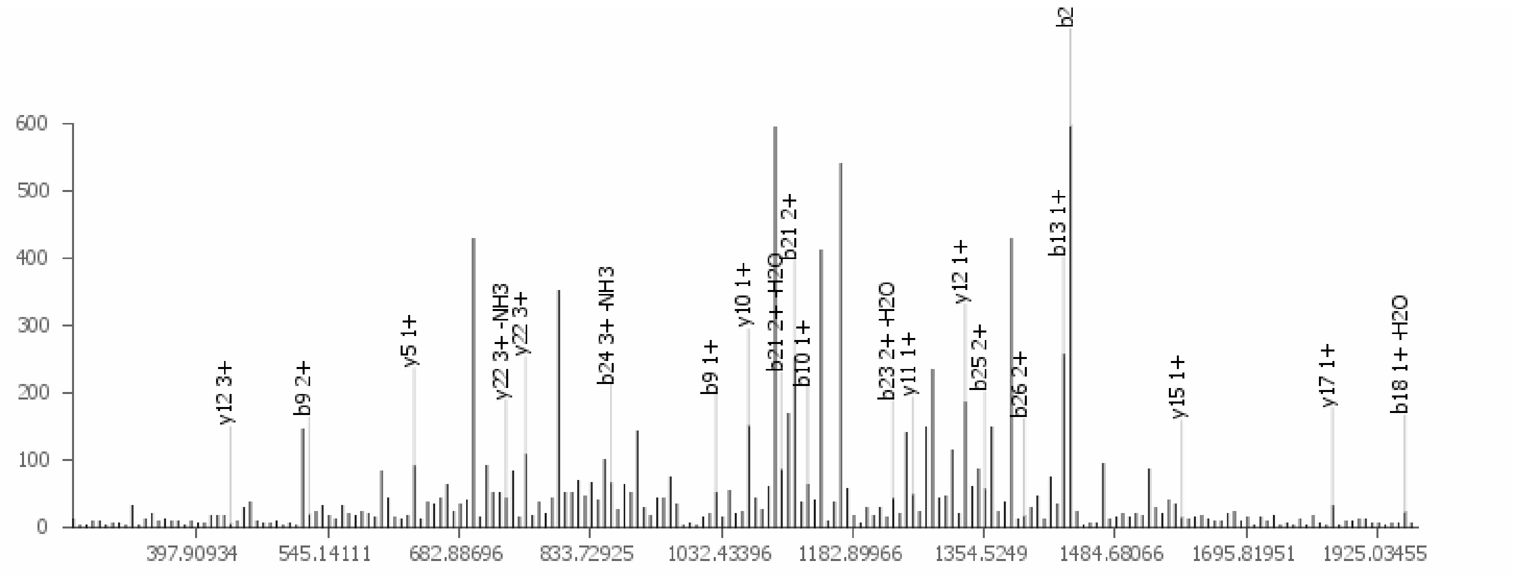

AT2G23300.1 - ELEVE(pT)LLK - 577.289676 - Charge:2

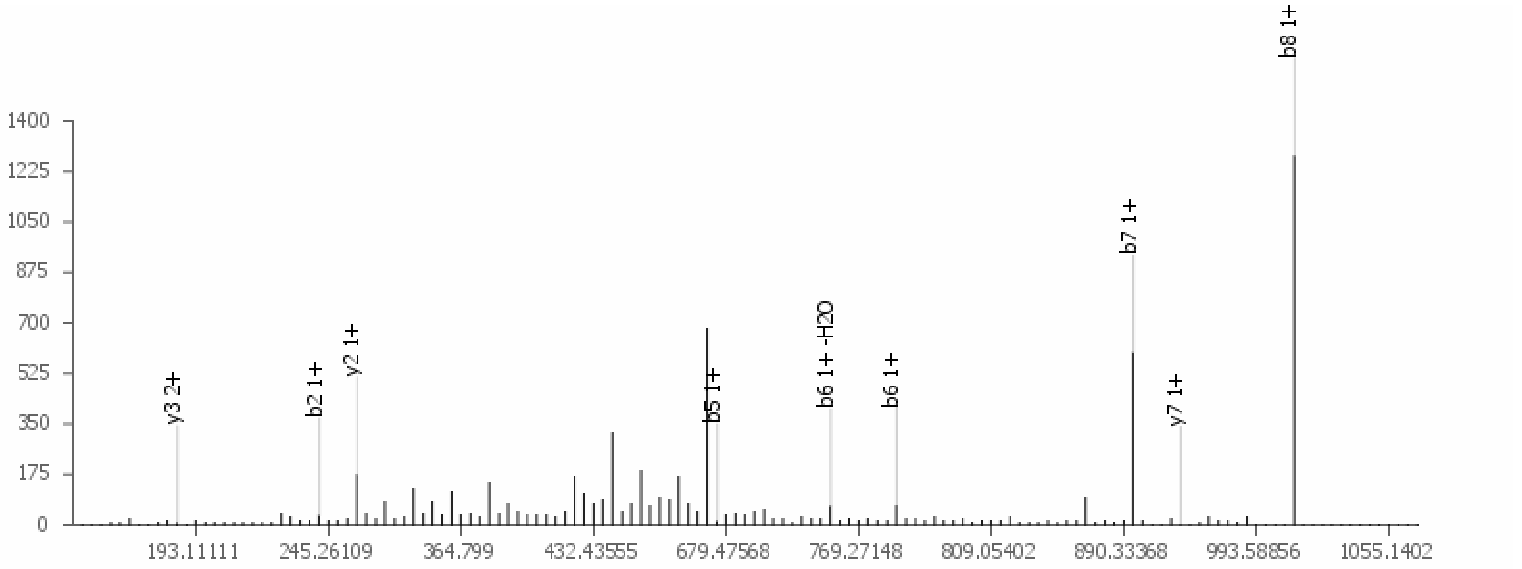

AT1G51580.1 - GNQVD(pY)L(oxM)SKGGK - 746.829487 - Charge:2

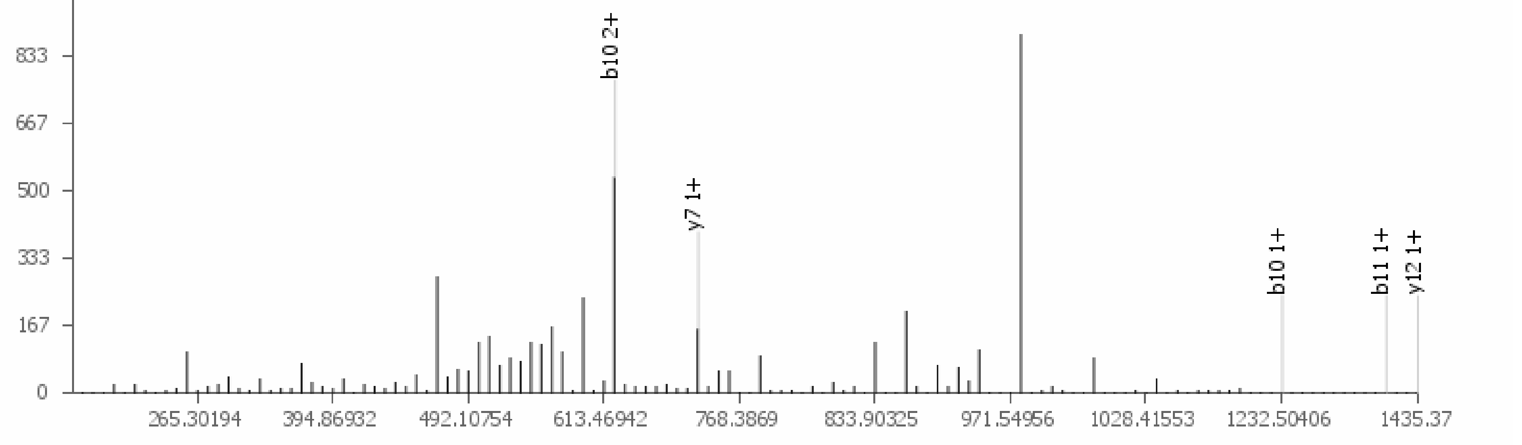

AT2G17850.1 - N(oxM)DGG(pY)IAWVNK - 732.299265 - Charge:2

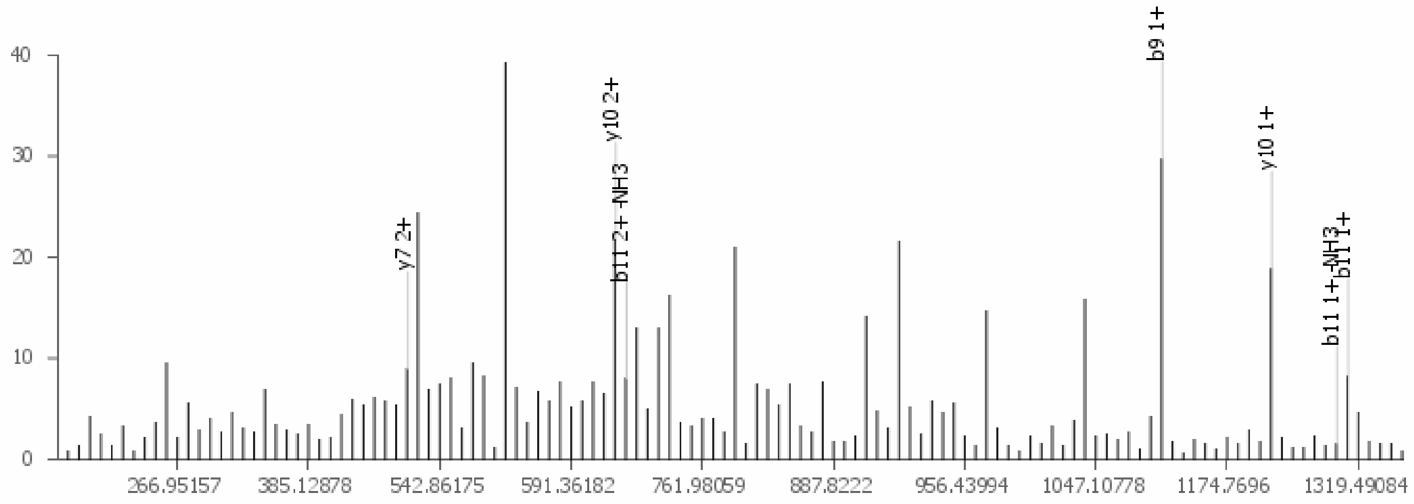

AT5G35550.1 - RGNI(pS)SDEEELIIR - 855.905853 - Charge:2

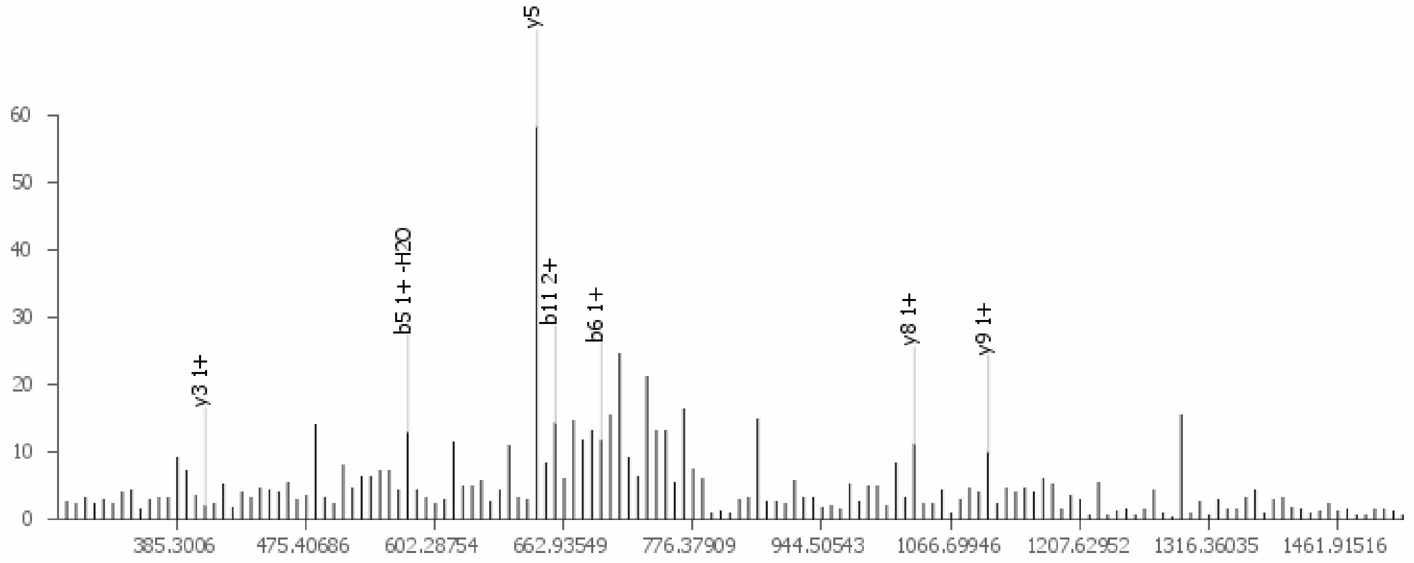

AT5G16930.1 - LLNL(pY)LEKYI(pS)K - 828.911988 - Charge:2

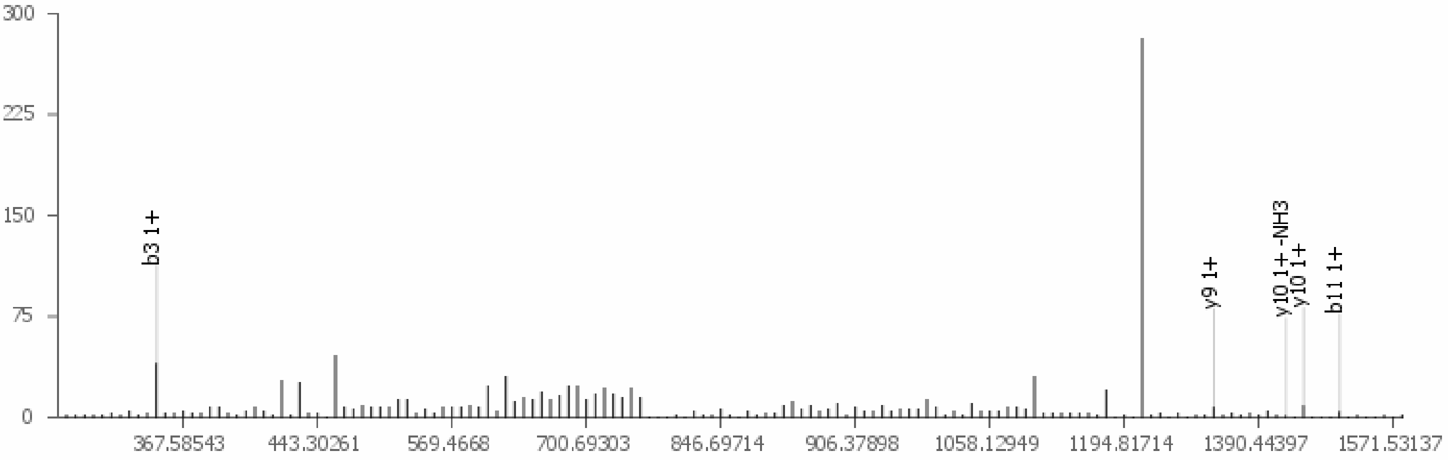

AT3G25210.1 - LILL(t)S(t)NR - 555.795682 - Charge:2

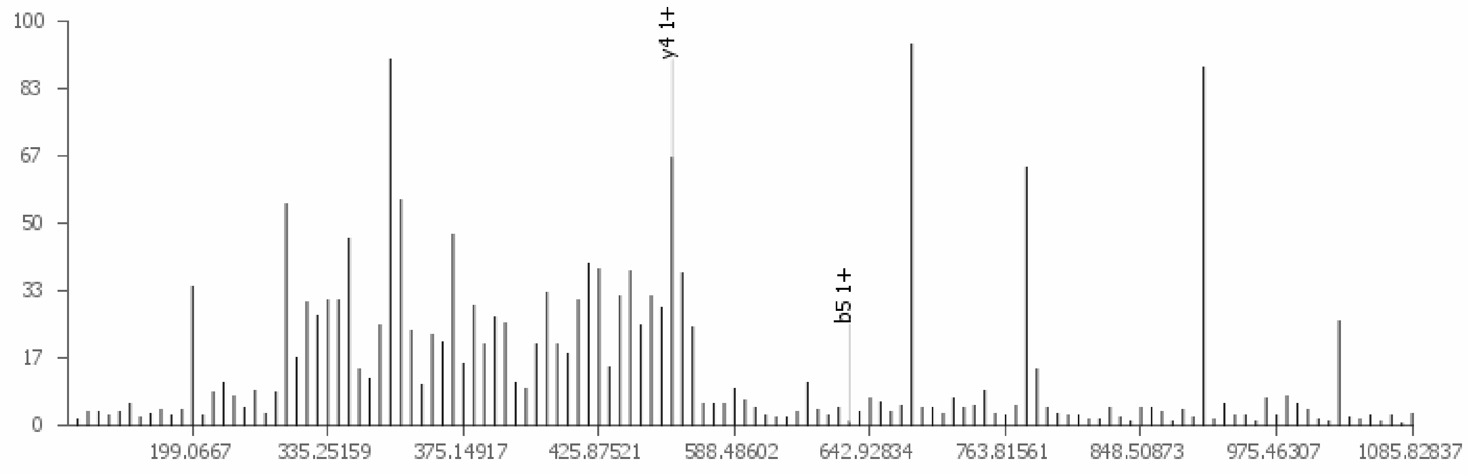

AT1G03060.1 - SGMVTGI(pS)GHQYK - 722.816397 - Charge:2

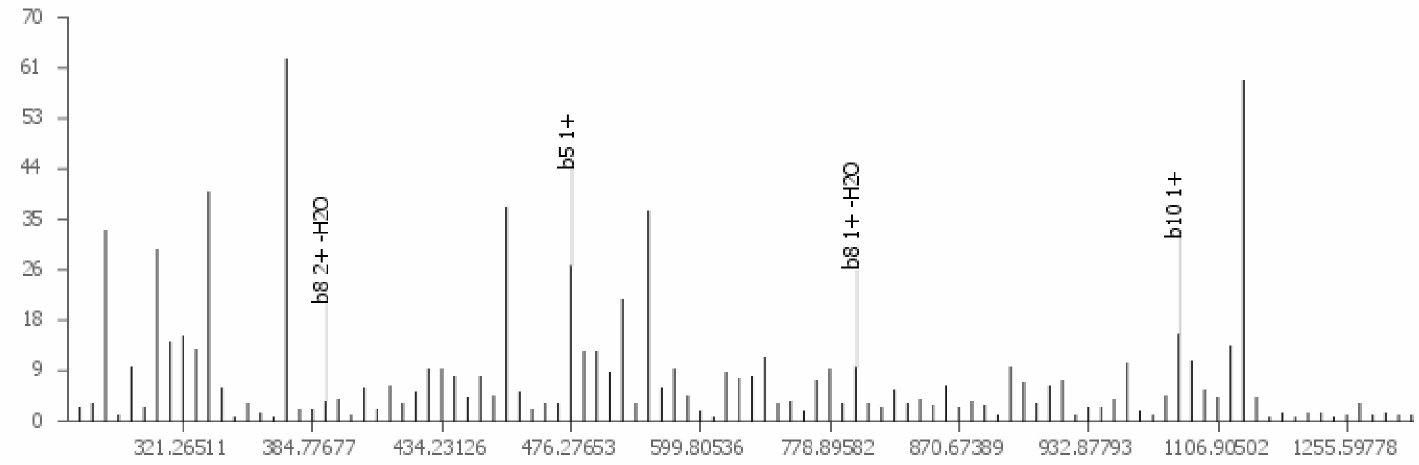

AT4G39610.1 - ITGTLFG(pY)R - 554.26475 - Charge:2

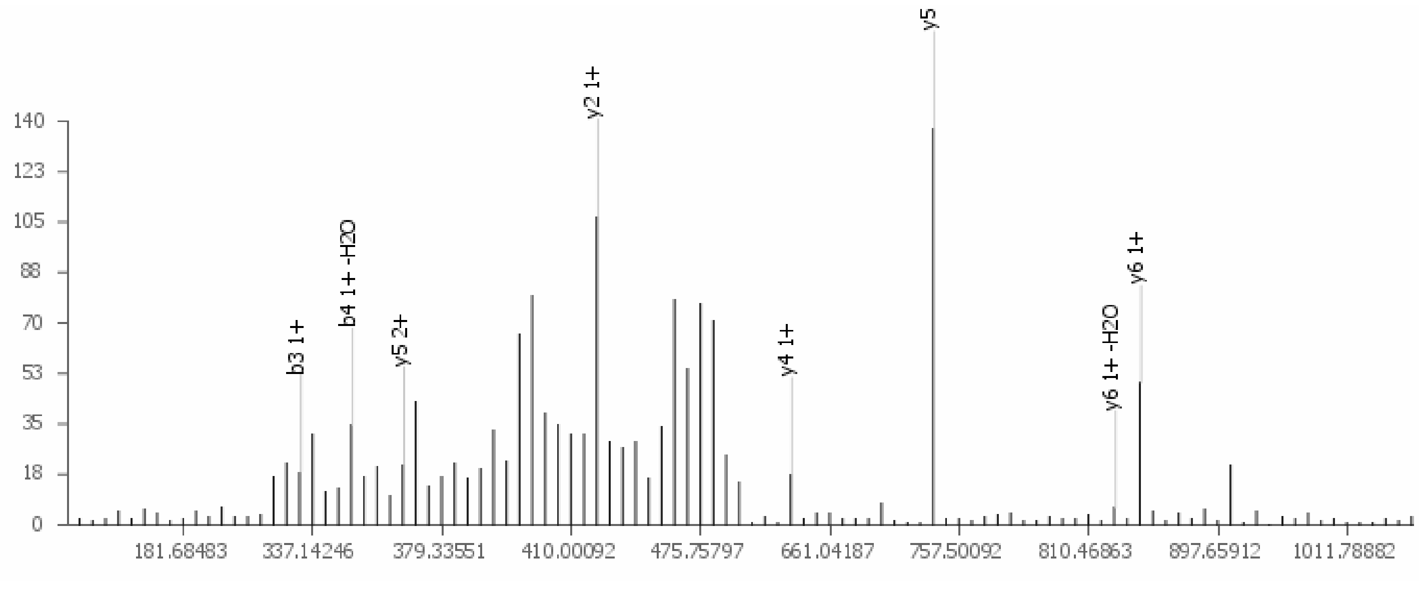

AT3G60280.1 - AG(pY)DNCDSSGATQNFADGDTK - 1108.911965 - Charge:2

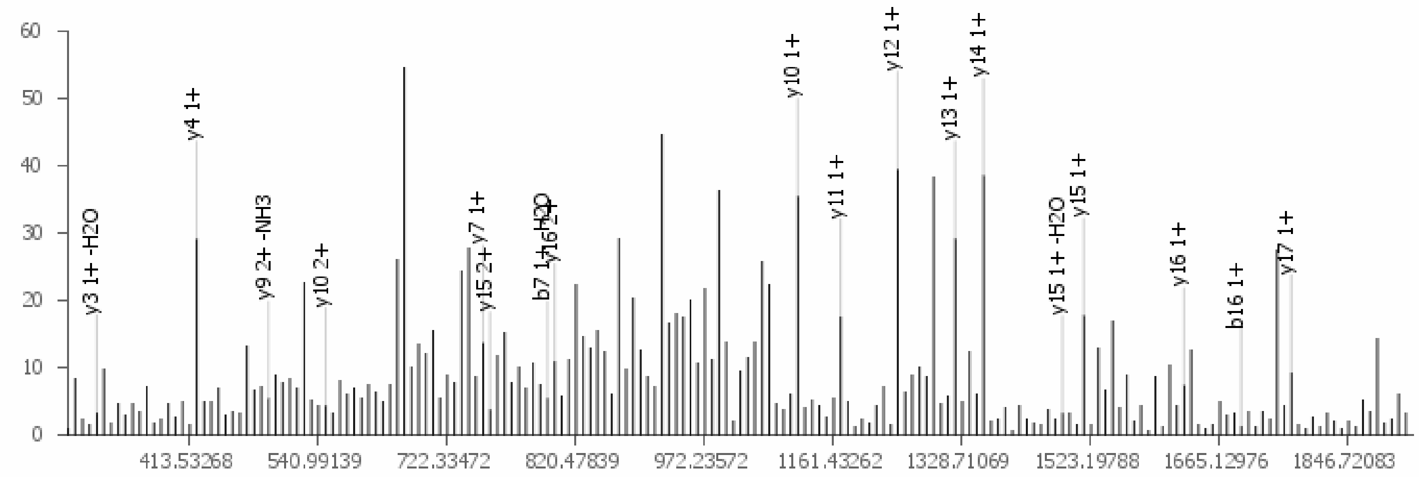

AT1G17840.1 - SKWINL(pS)VILS(oxM)III(pY)R - 742.38537 - Charge:3

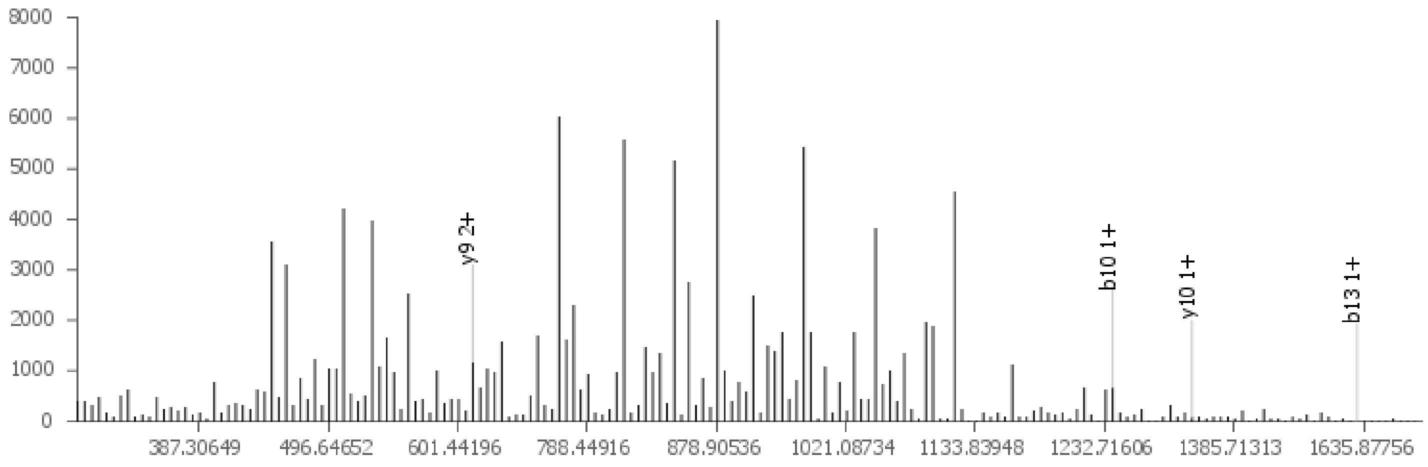

AT3G01290.1 - LQQLDVQCE(pT)K - 692.809179 - Charge:2

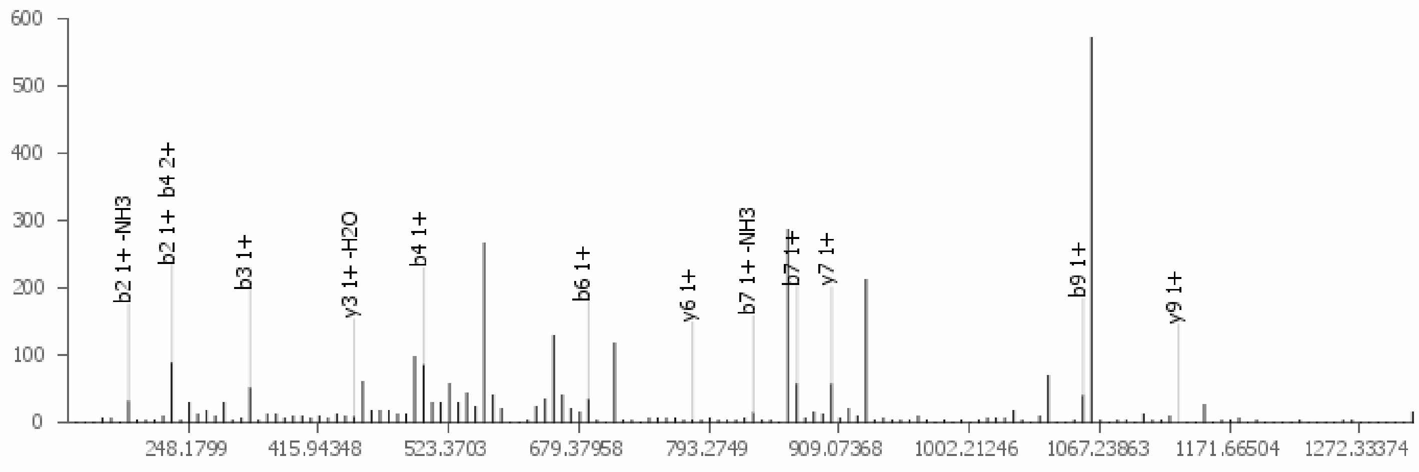

AT1G15690.1 - QFN(pT)IPGLMEGTAKPDYATCVK - 1232.567488 - Charge:2

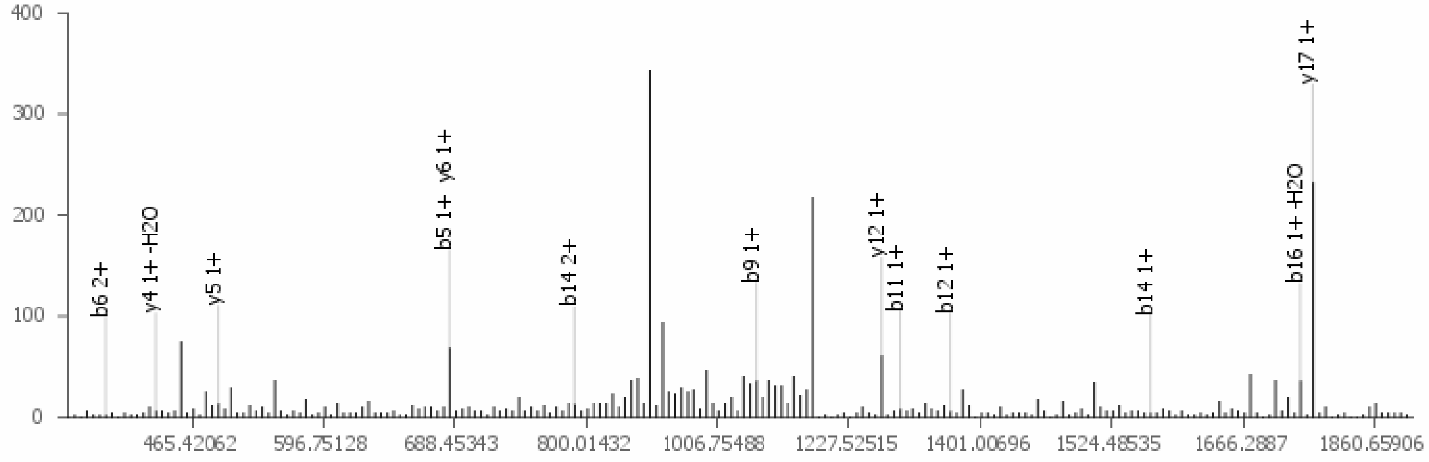

AT5G57110.1 - AIALECGIL(pS)SDADLSEPTLIEGK - 1263.118148 - Charge:2

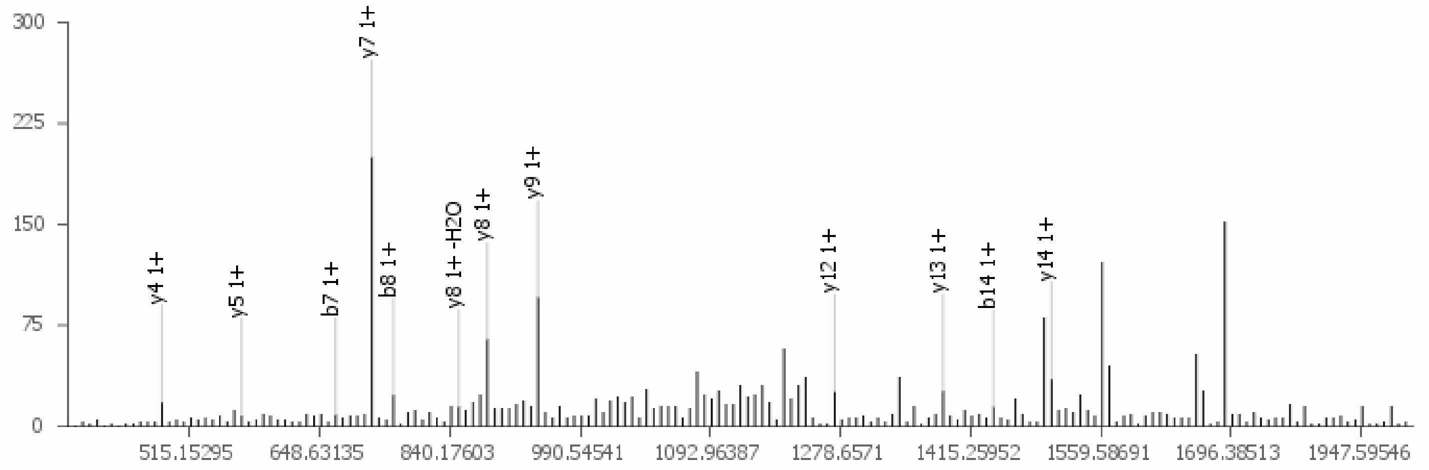

AT2G18960.1 - (pS)GLEDIKNETVDLEK - 885.417948 - Charge:2

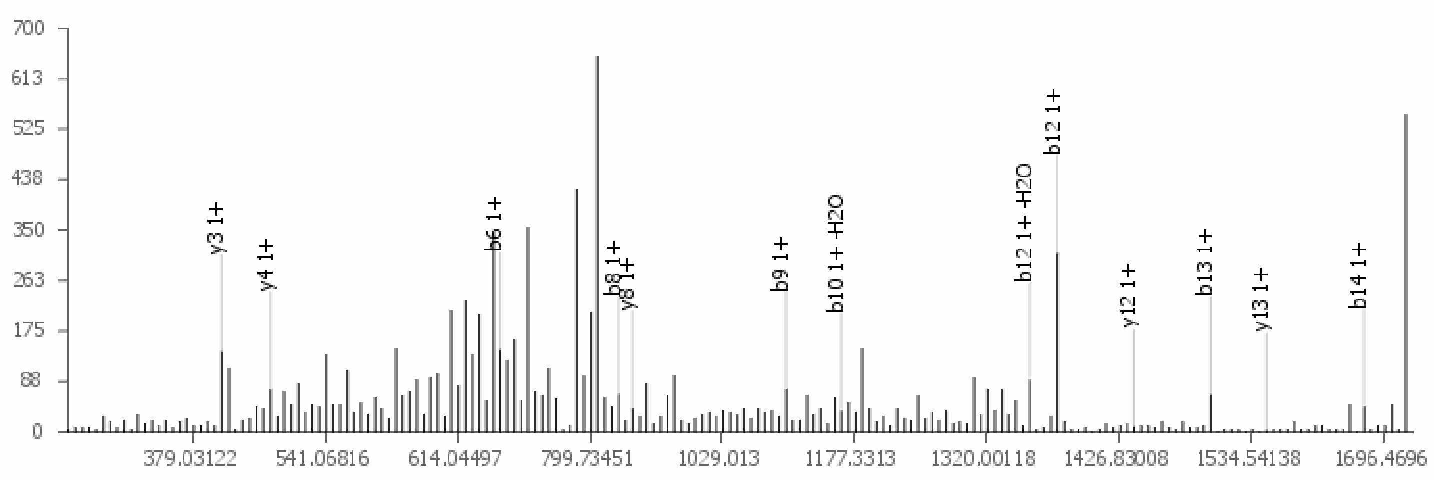

AT1G59820.1 - DVSA,(pS,)LSKR - 528.234018 - Charge:2

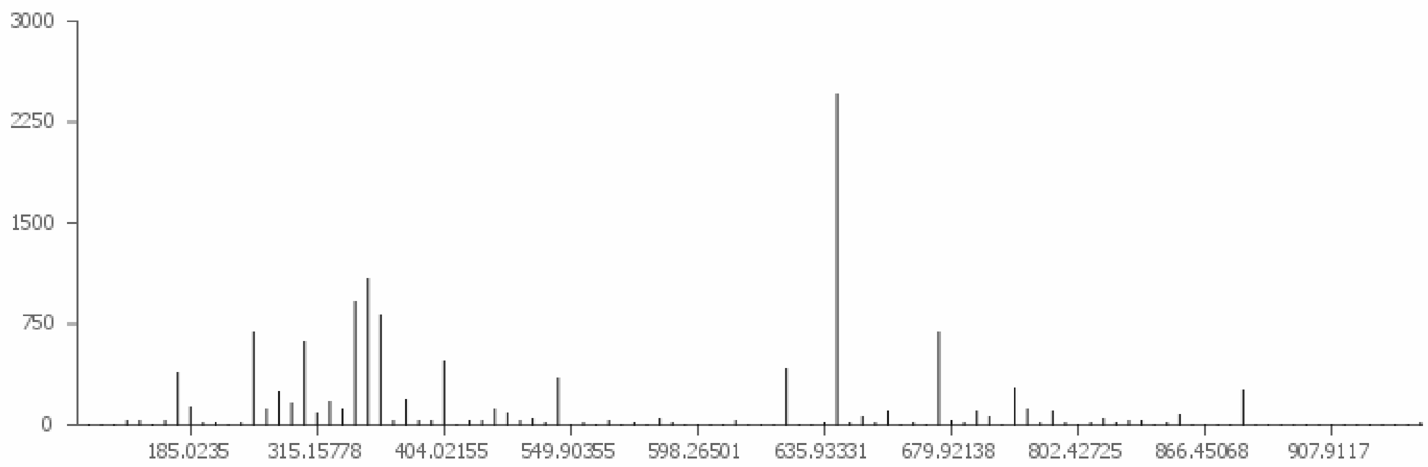

AT3G63490.2 - AG(pT)VTANIPQSSRR - 769.376801 - Charge:2

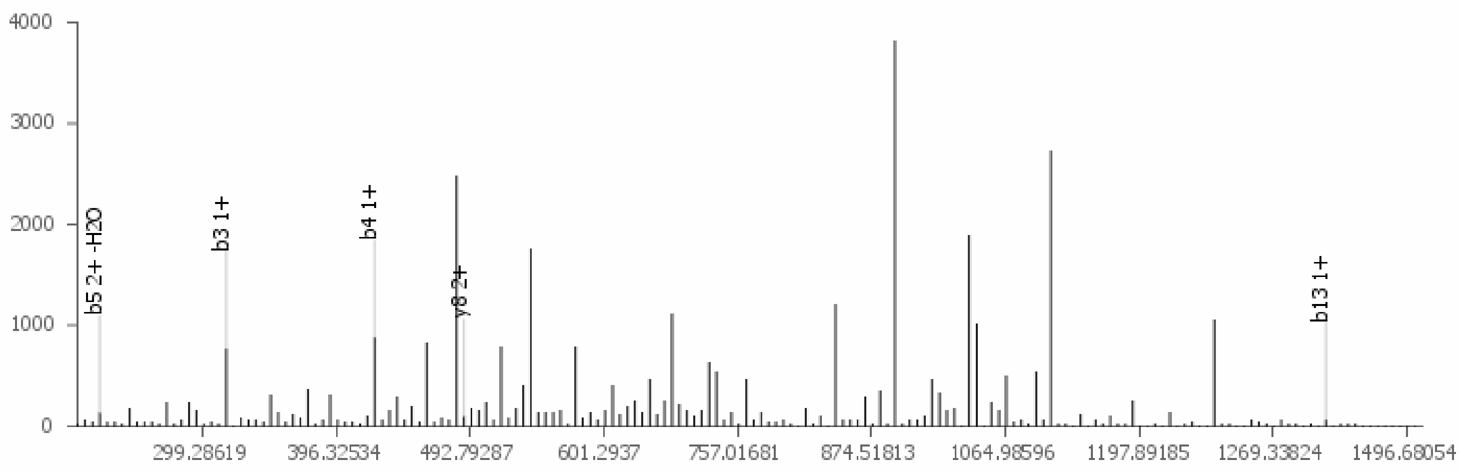

AT4G08850.1 - VTEIAI(pY)DNLL(pT)GPIPSSFGNLTK - 917.413393 - Charge:3

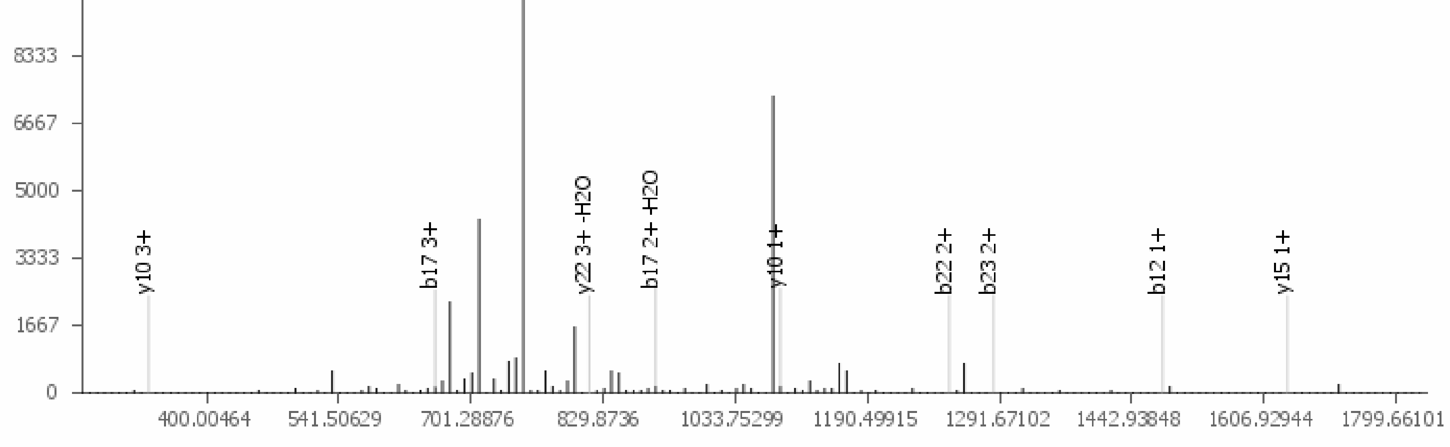

AT3G05030.1 - GFVPFVPG(pS)PTER - 735.35208 - Charge:2

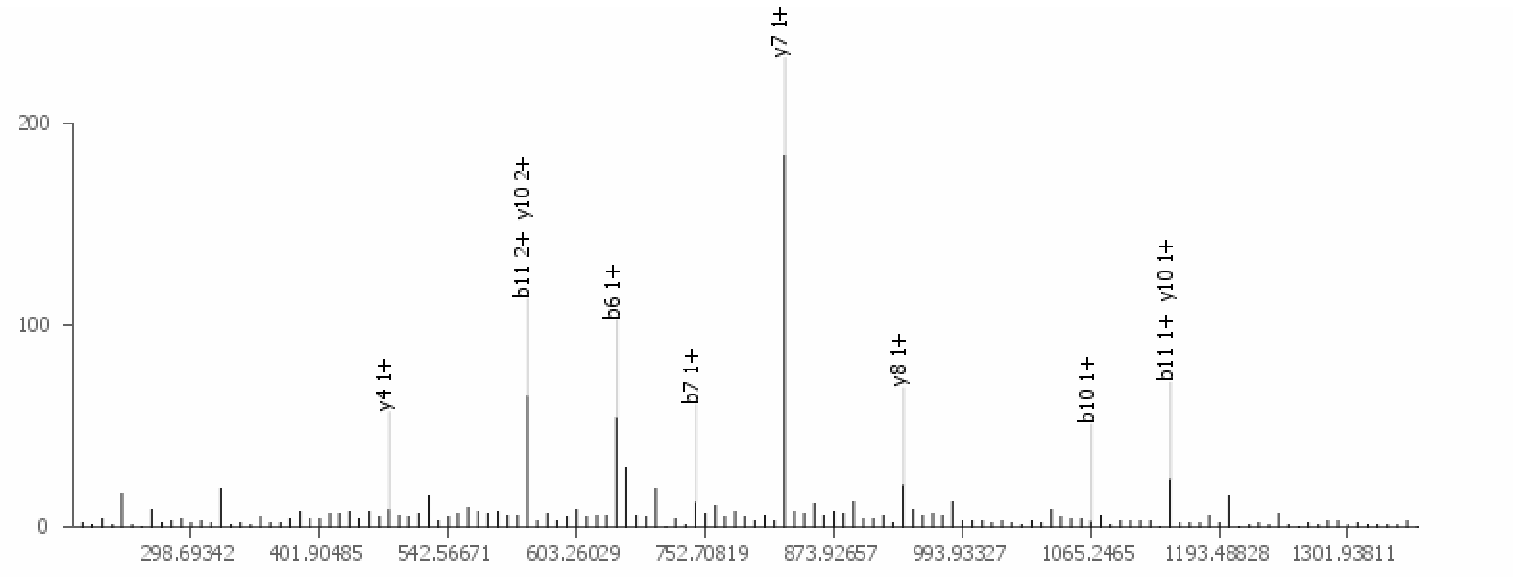

AT5G37280.1 - ANY(pT)LYQRPFLM(pT)VKVR - 1130.545032 - Charge:2

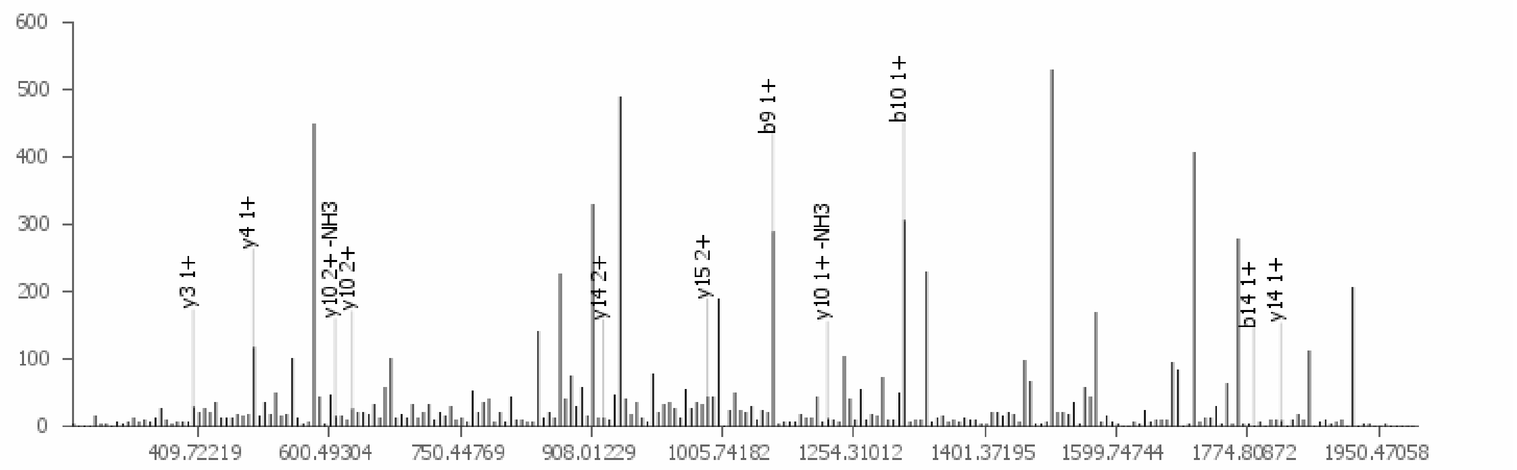

AT4G35100.1 - ALG(pS)FR - 365.669947 - Charge:2

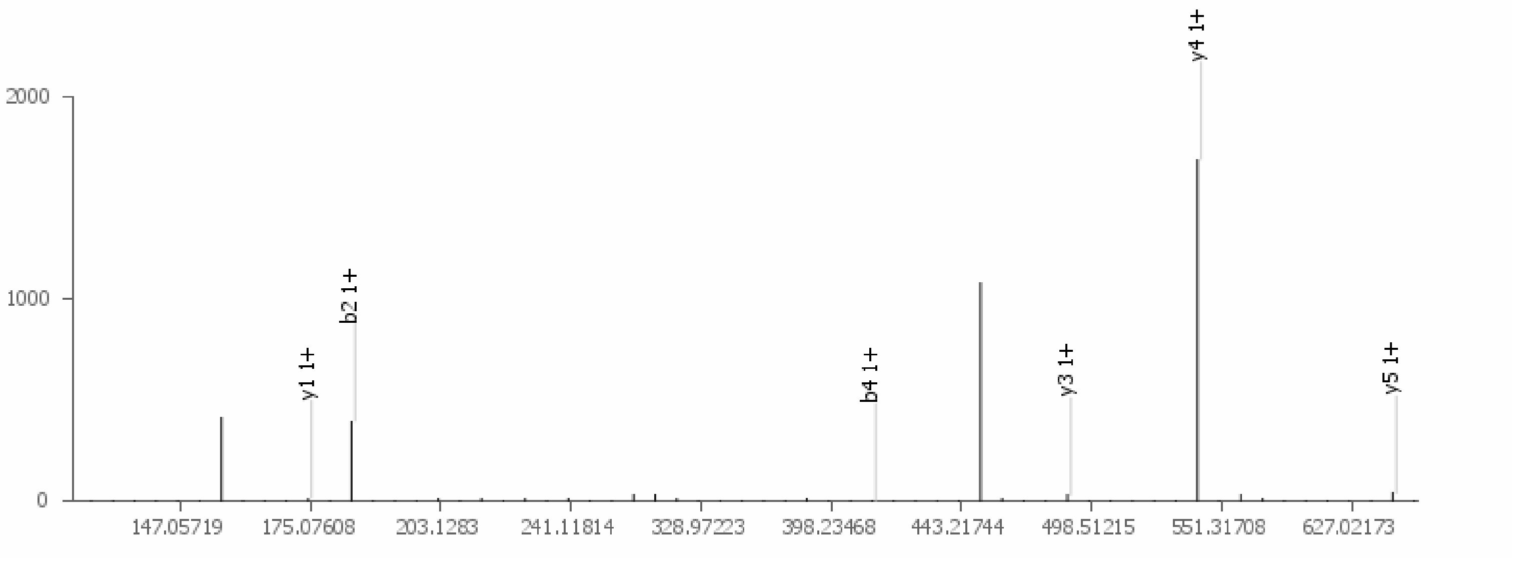

AT4G34280.1 - S,(pS,)SLSEIVK - 515.244277 - Charge:2

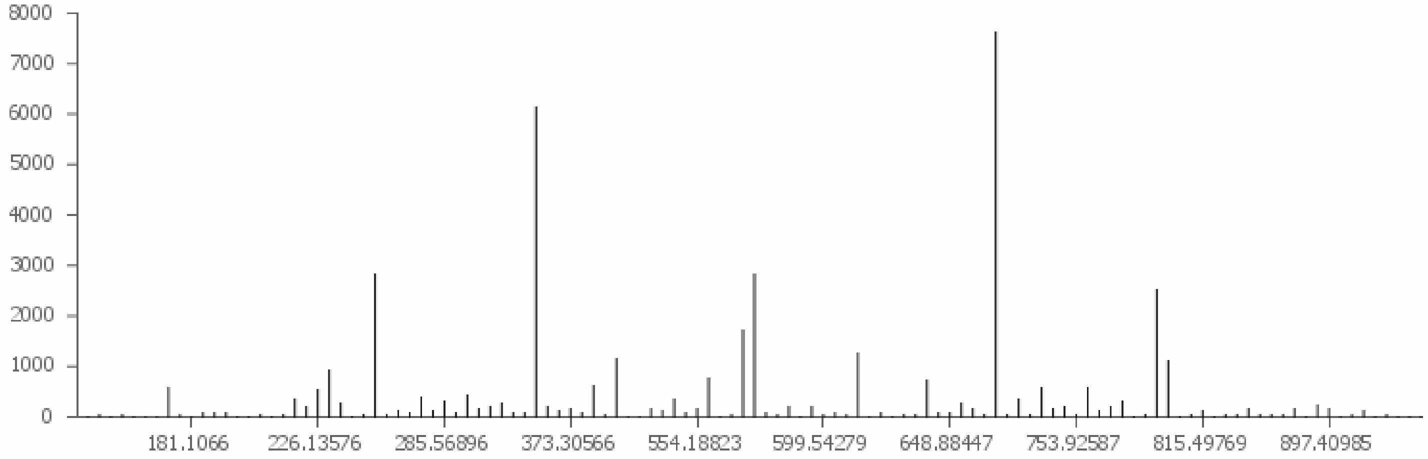

AT4G35100.1 - ALG(pS)FR(pS)NATN - 649.258002 - Charge:2

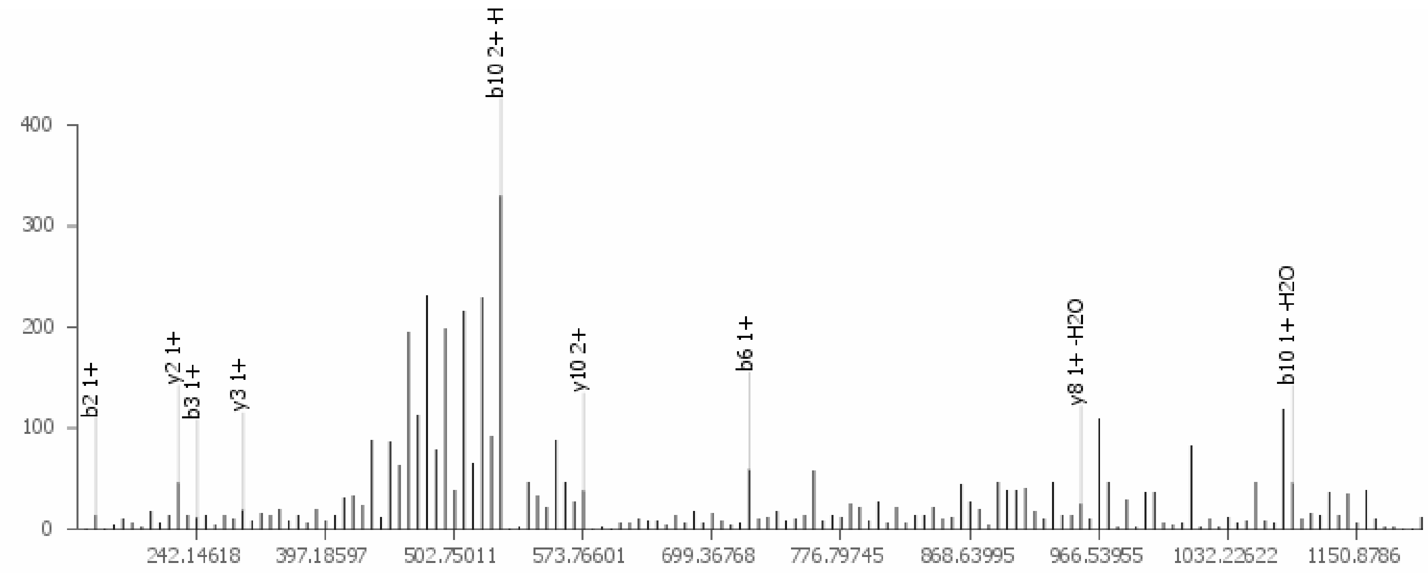

AT1G29430.1 - ISFQR(s)(s)I(t)(t)SSQTAVEK - 1065.485469 - Charge:2

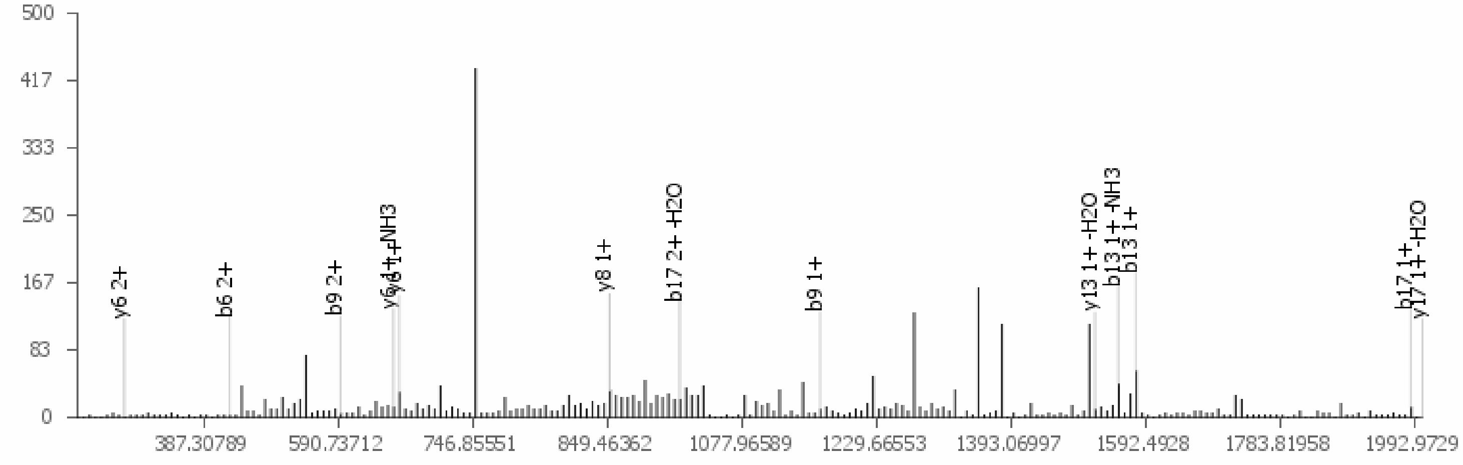

AT1G52200.1 - VTTPSEEDSNNGLPVQQPG(pT)PNQR - 1323.100939 - Charge:2

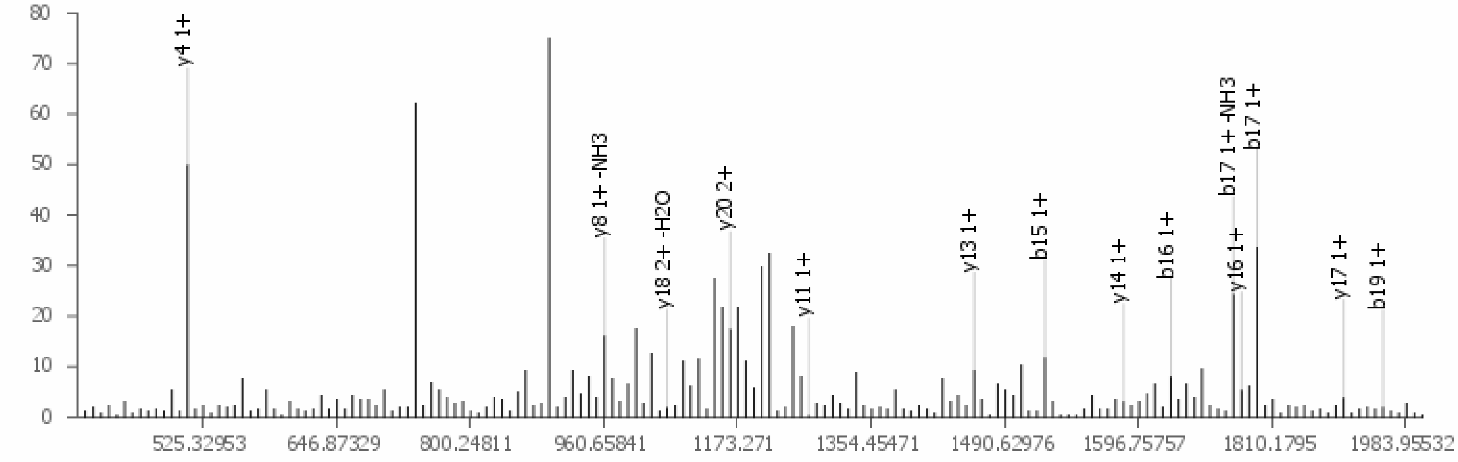

AT4G39850.1 - IASLNG(t)(t)VKYVLEQDK - 980.00029 - Charge:2

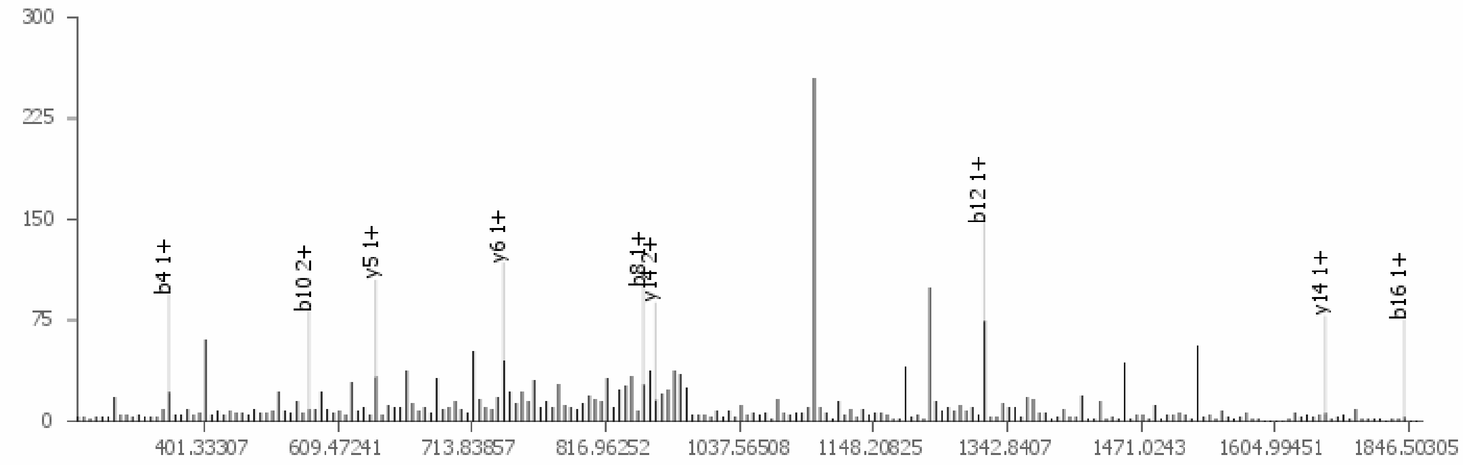

AT3G57350.1 - DFLHSL(pS)KTSMLPK - 842.413135 - Charge:2

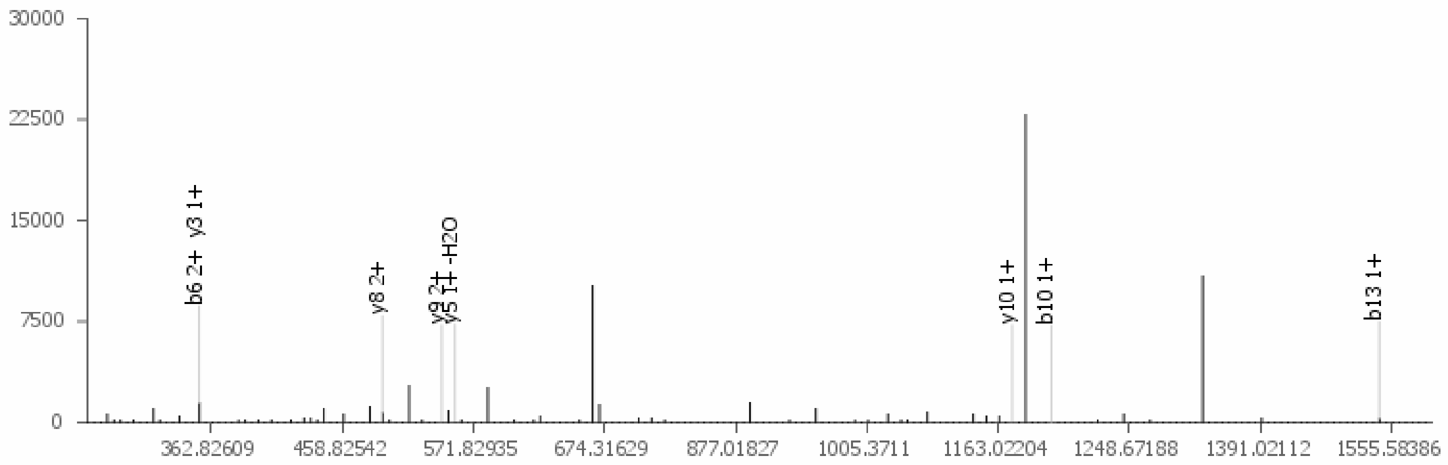

AT5G24740.1 - SWKD(pS)TAMILSVI(pS)GR - 955.934033 - Charge:2

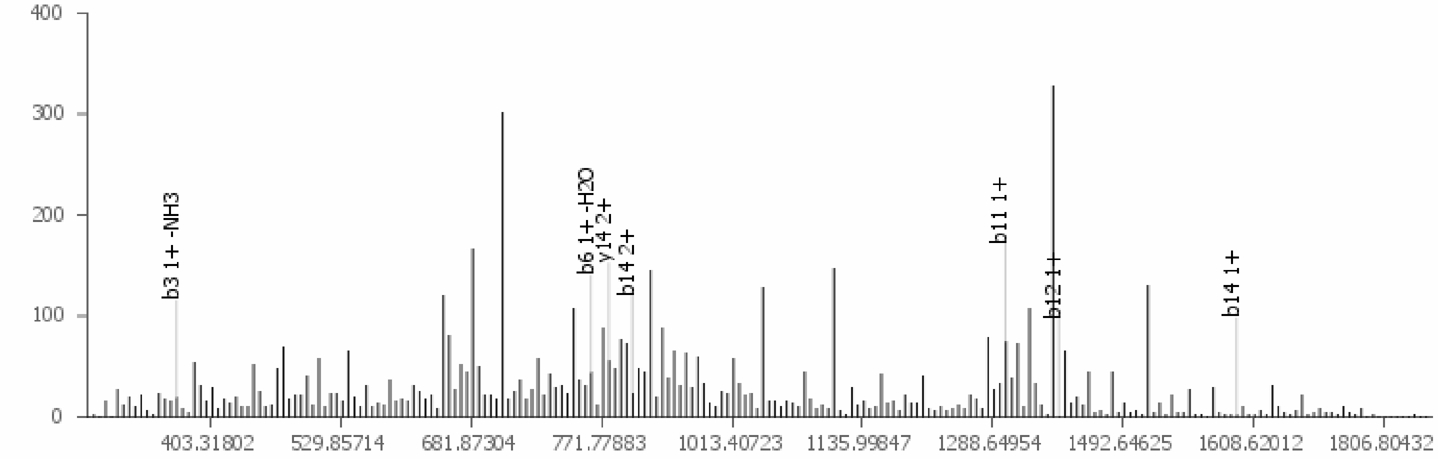

AT1G22530.1 - EF(pT)APPPPPAPVK - 714.348275 - Charge:2

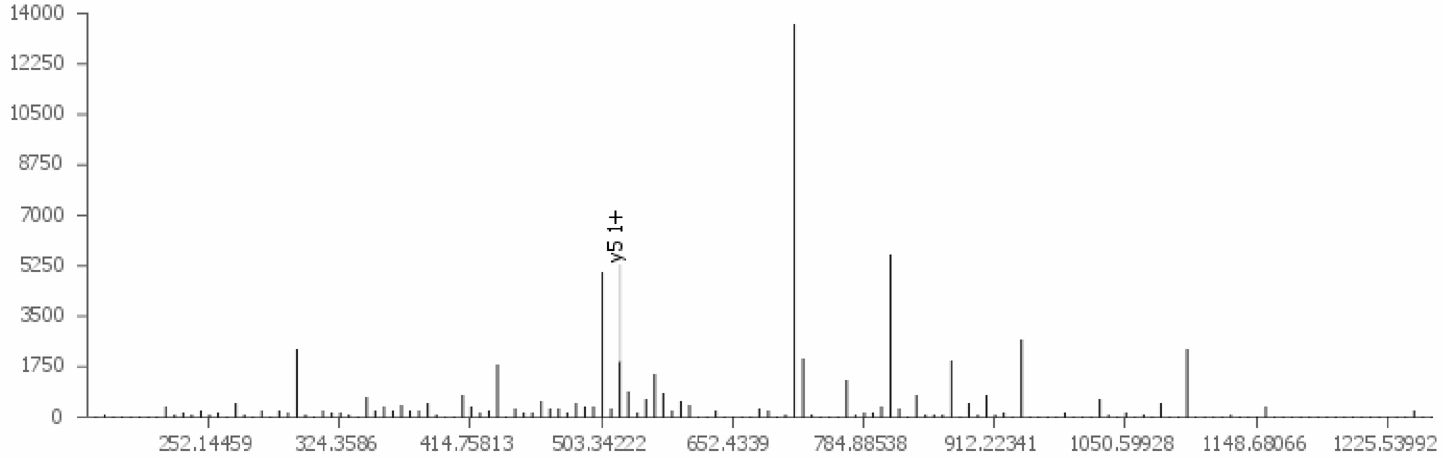

AT4G16195.1 - Q(pY)LSLFILIIFI(pT)(pT)K - 1026.993979 - Charge:2

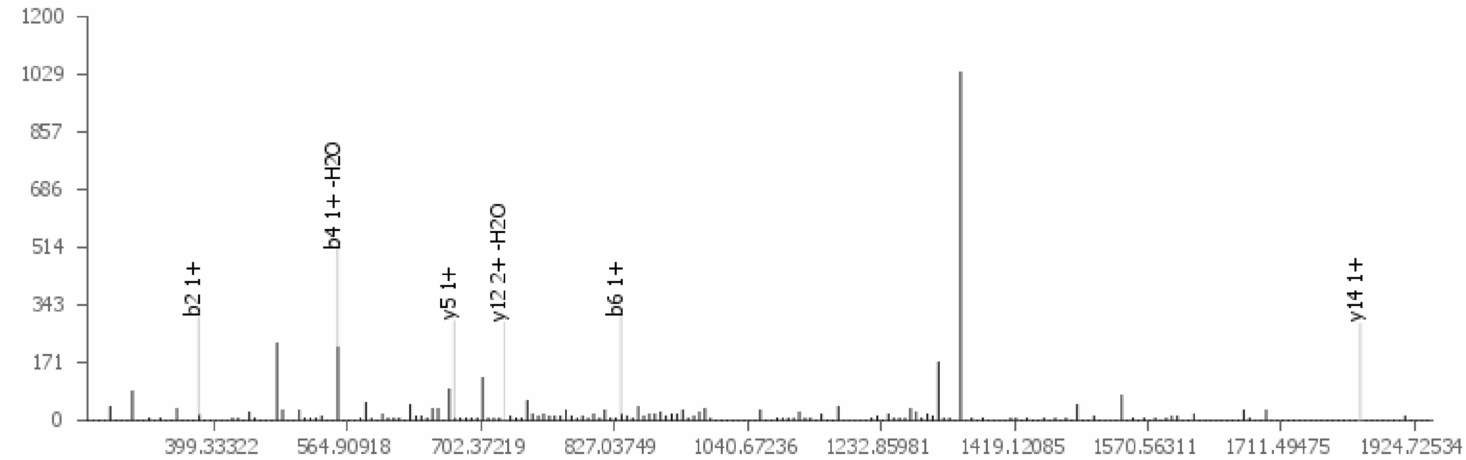

AT2G28100.1 - NCLFLLNVPPN(s)(s)GLI(pS)EQDIKVLEEFSEMK - 1218.570097 - Charge:3

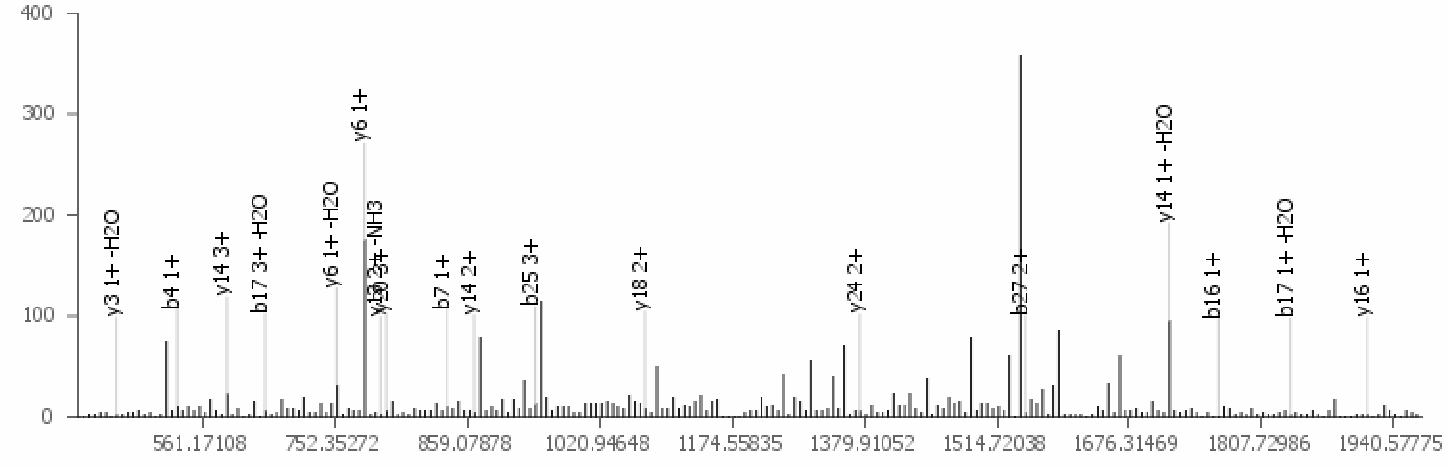

AT1G59870.1 - SL(pS)TADGNR - 500.710553 - Charge:2

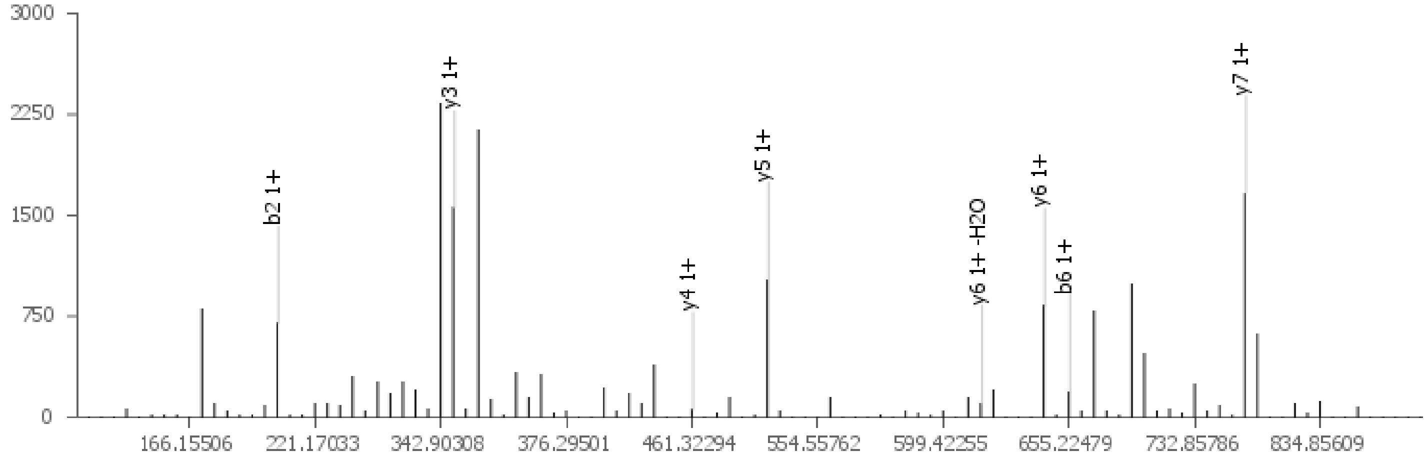

AT4G26380.1 - VVSGPESQLV,(pS,)LV,(pT,)R - 875.380335 - Charge:2

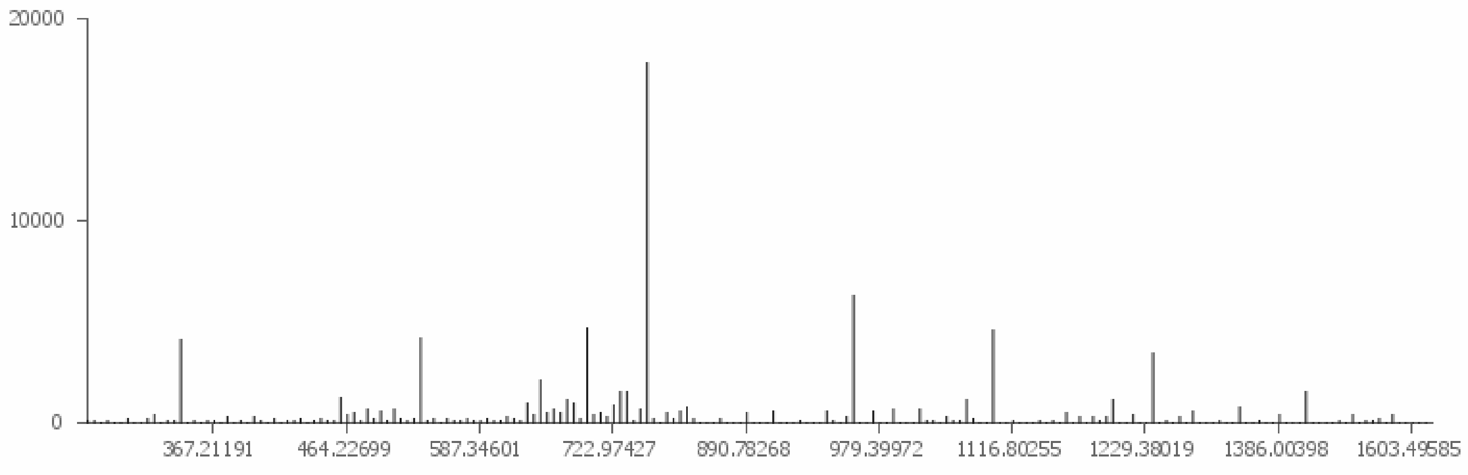

AT3G53420.1 - SLG(pS)FR - 373.666744 - Charge:2

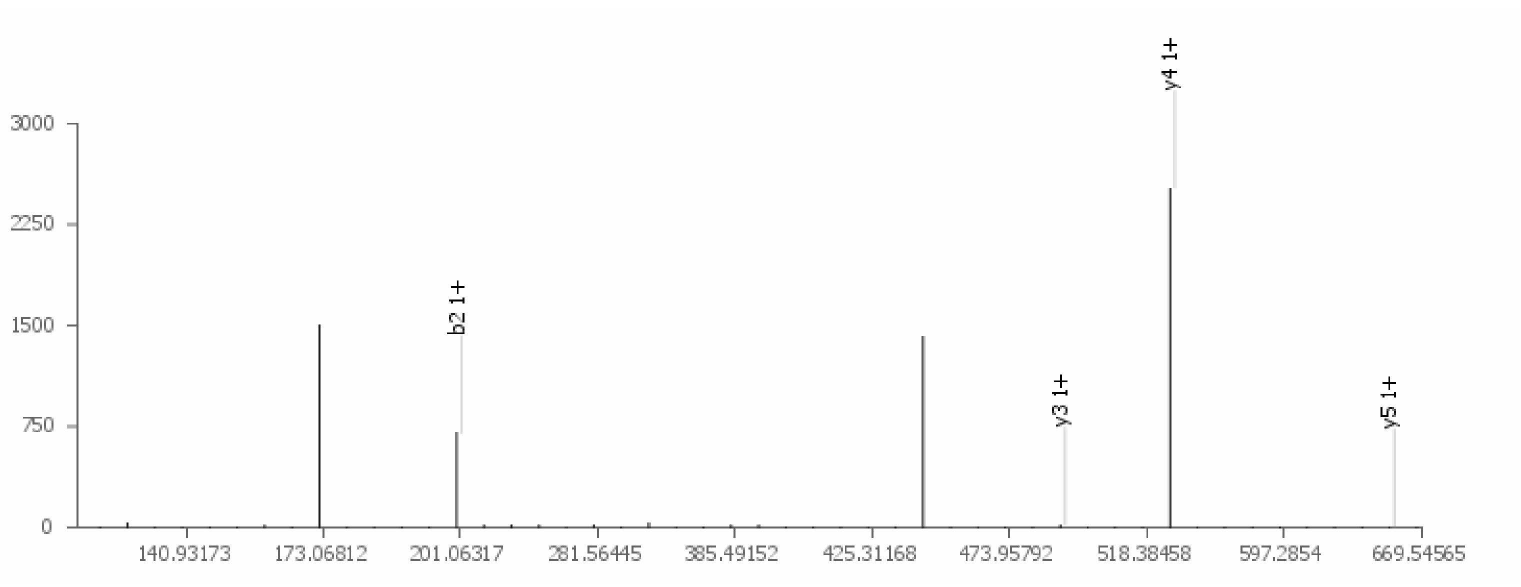

AT5G37450.1 - ELENI(pY)GLIPK - 684.84002 - Charge:2

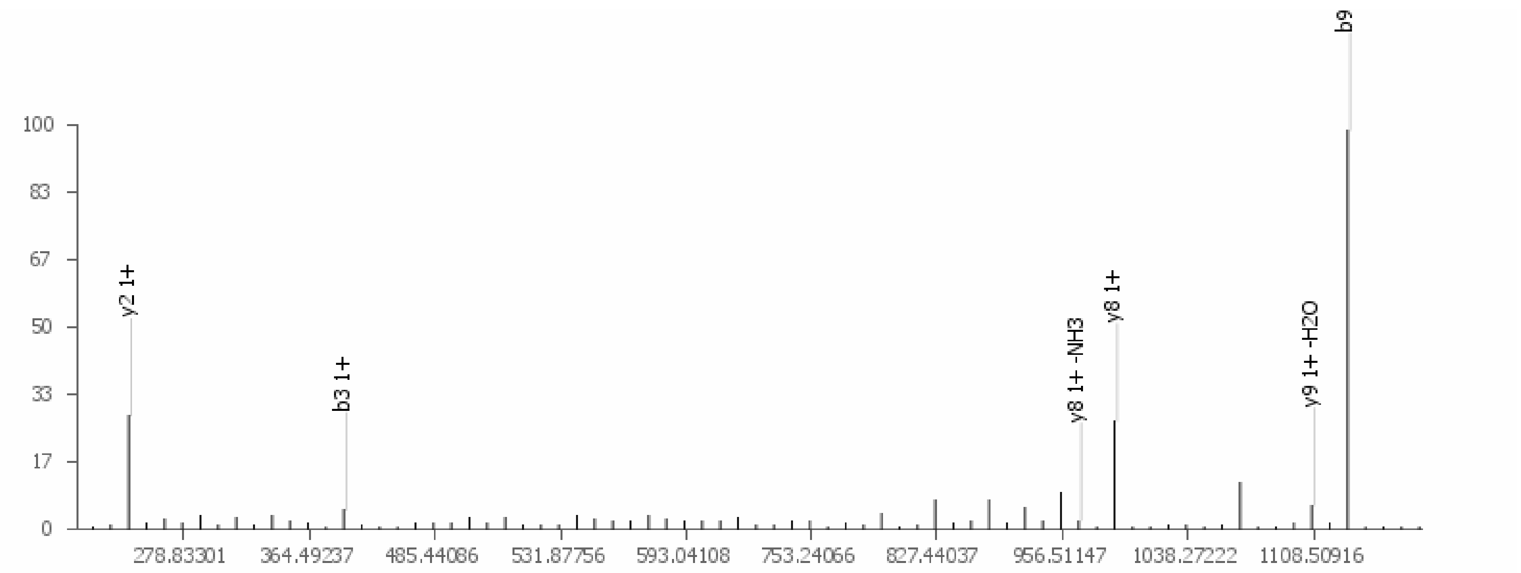

AT1G78800.1 - QNL(pS)DV(pT)LTVAGGYDER - 500.21466 - Charge:4

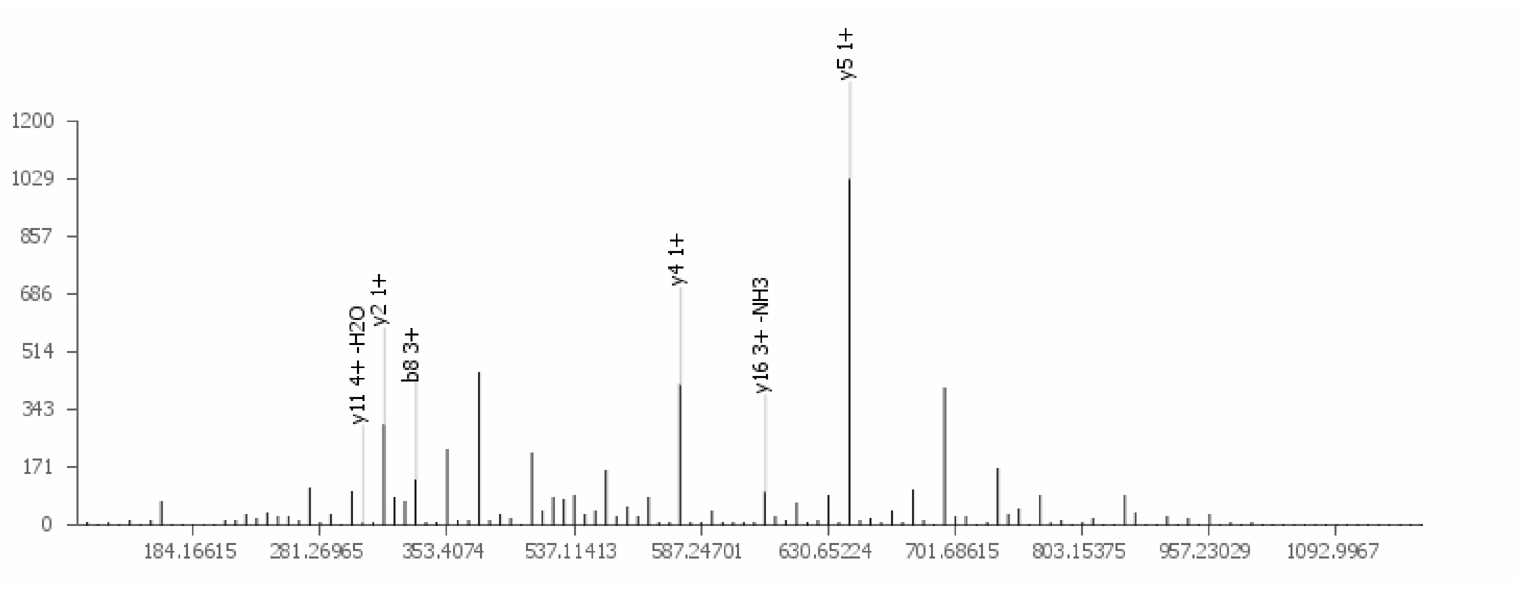

AT2G44530.1 - ASIVQP(pS)PTFPALNLR - 895.958891 - Charge:2

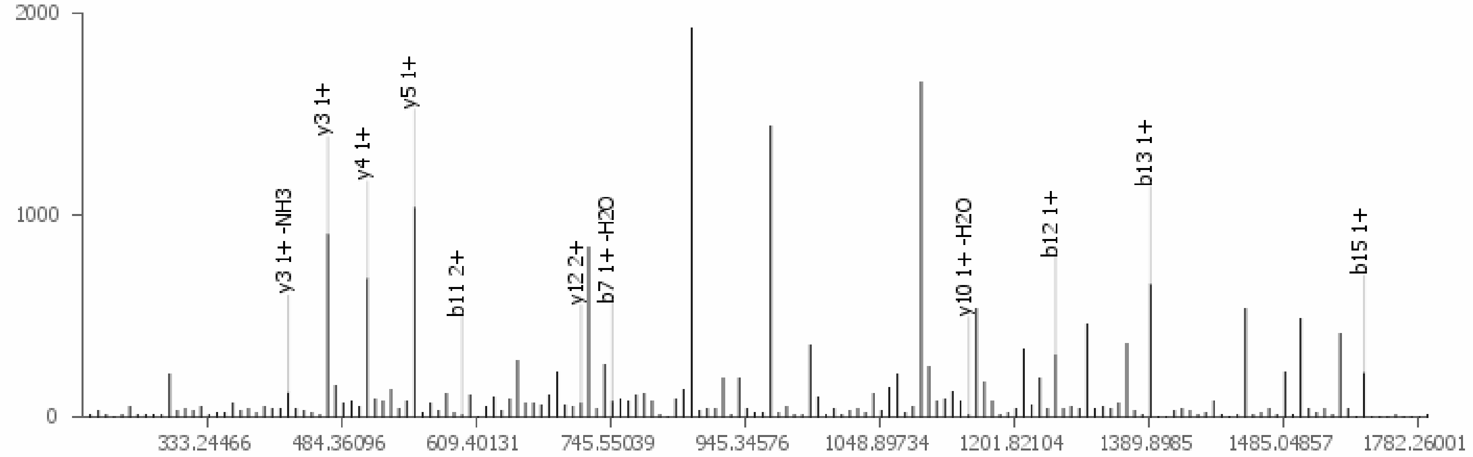

AT1G69380.1 - A(pY)FLSTSIDLK - 669.324072 - Charge:2

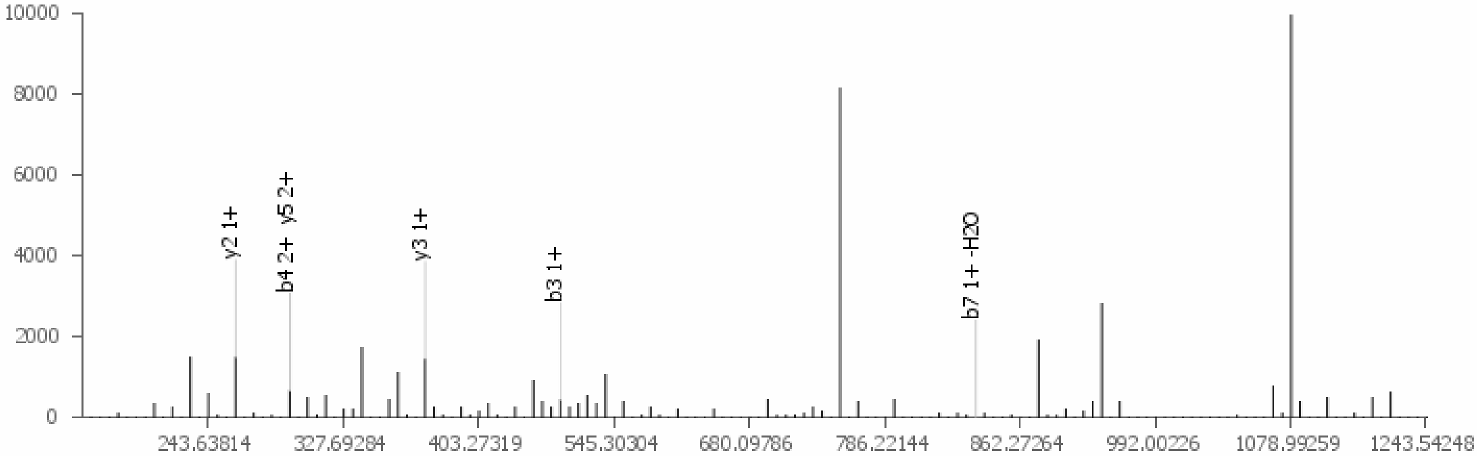

AT3G26240.1 - (y)(s)KPNMVDILNLFK - 881.438739 - Charge:2

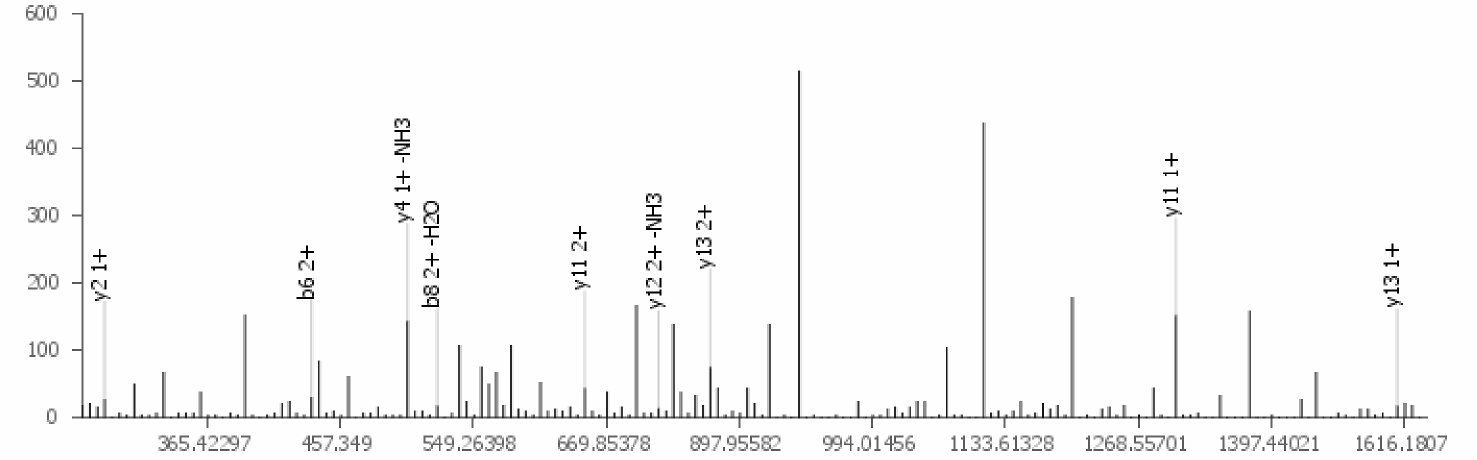

AT1G42740.1 - QTAK(pS)S(pT)APLLLNPPPK - 961.964978 - Charge:2

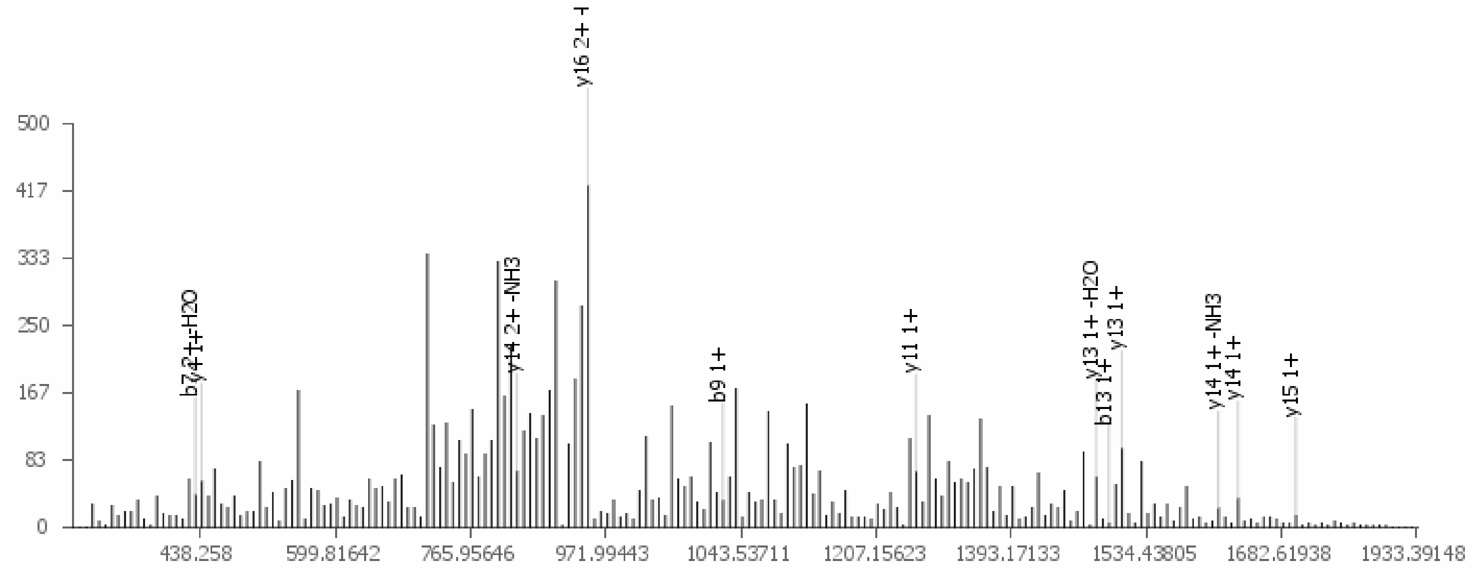

AT1G57690.1 - L(pT)FSGSYLQNLSVAEKR - 996.989952 - Charge:2

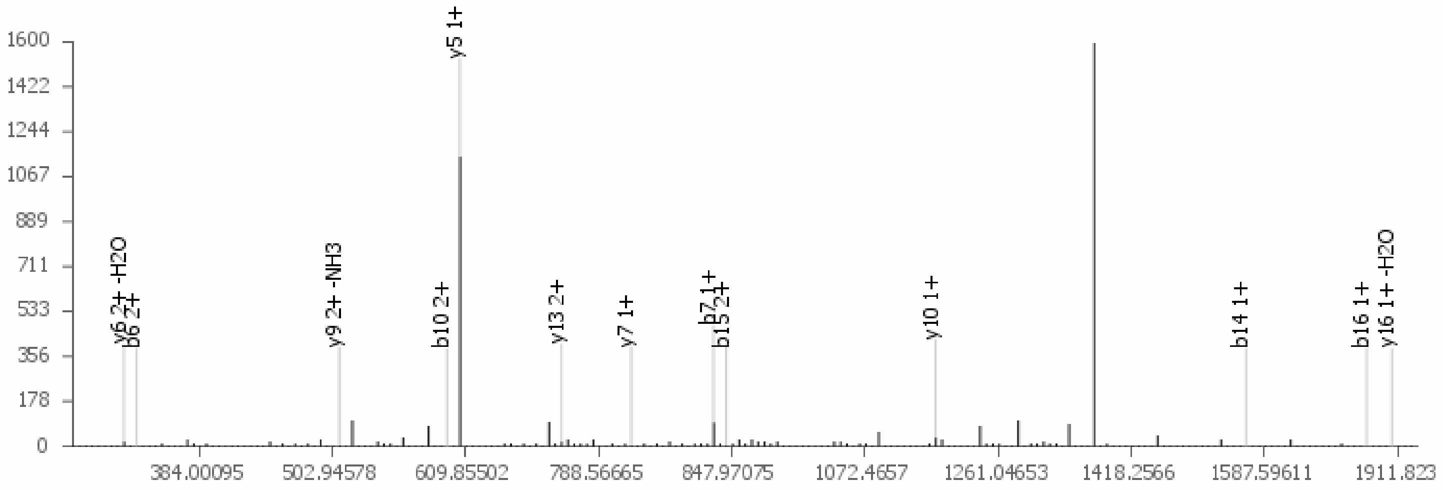

ATCG00040.1 - IPI(pS)(pS)IIGSLAK - 679.846764 - Charge:2

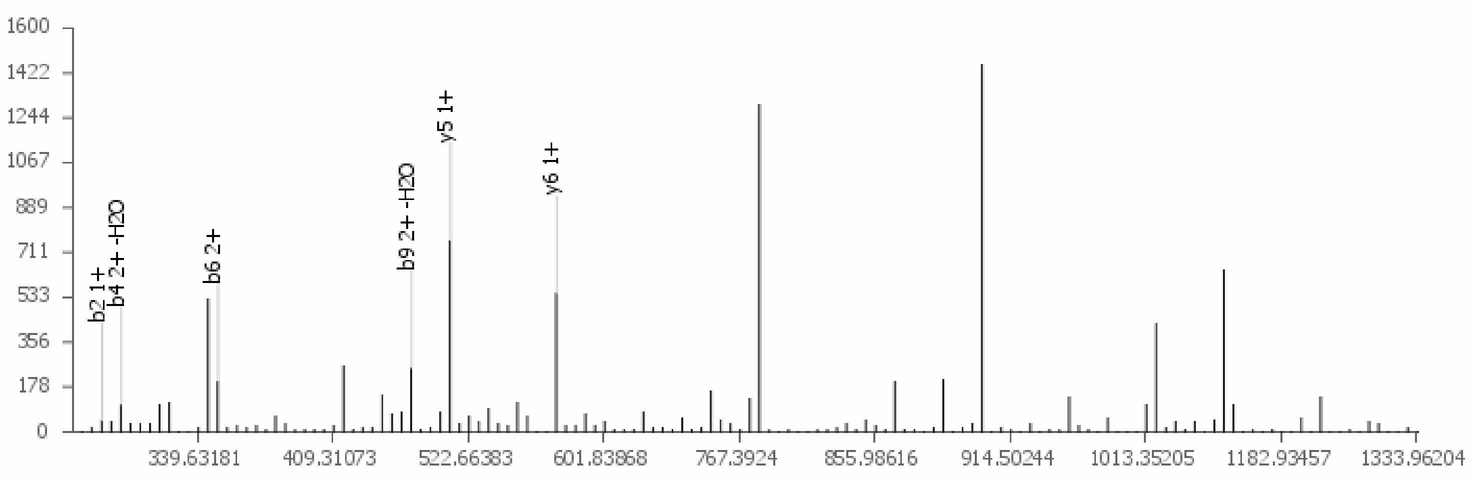

AT4G35300.1 - GG(pS)TMSVLSR - 537.736553 - Charge:2

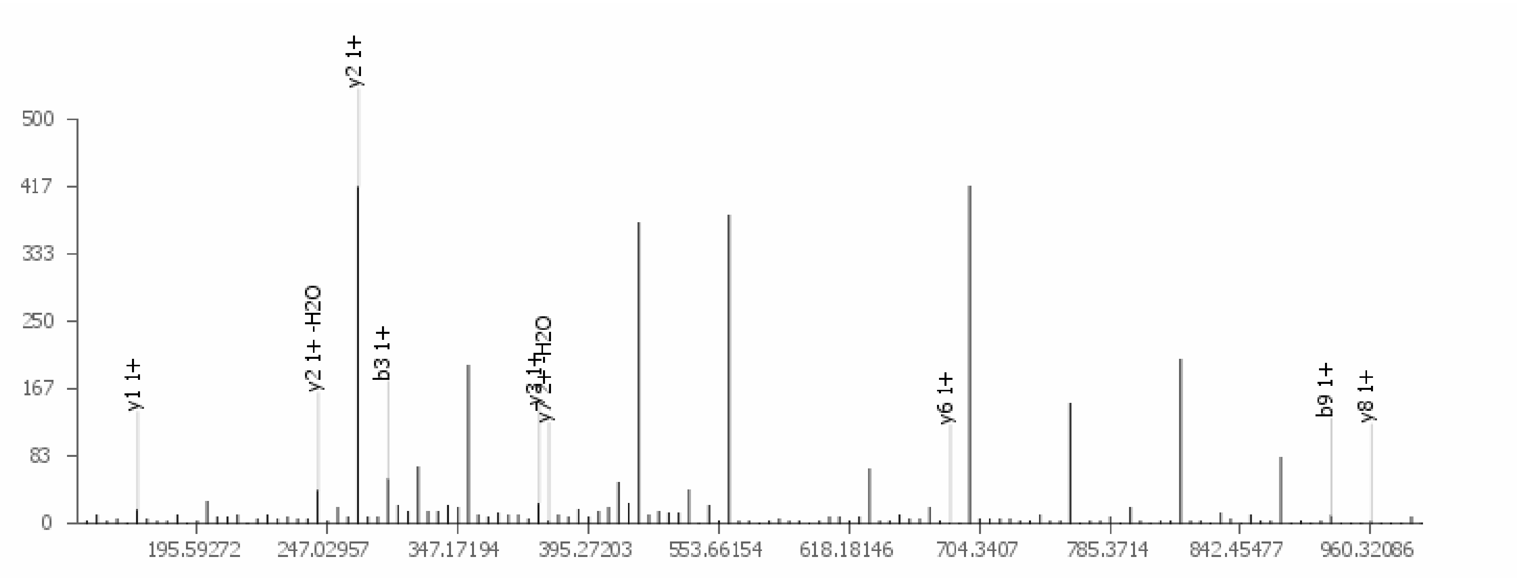

AT2G34780.1 - LSAHLI(pS)K(pS)GR - 664.804136 - Charge:2

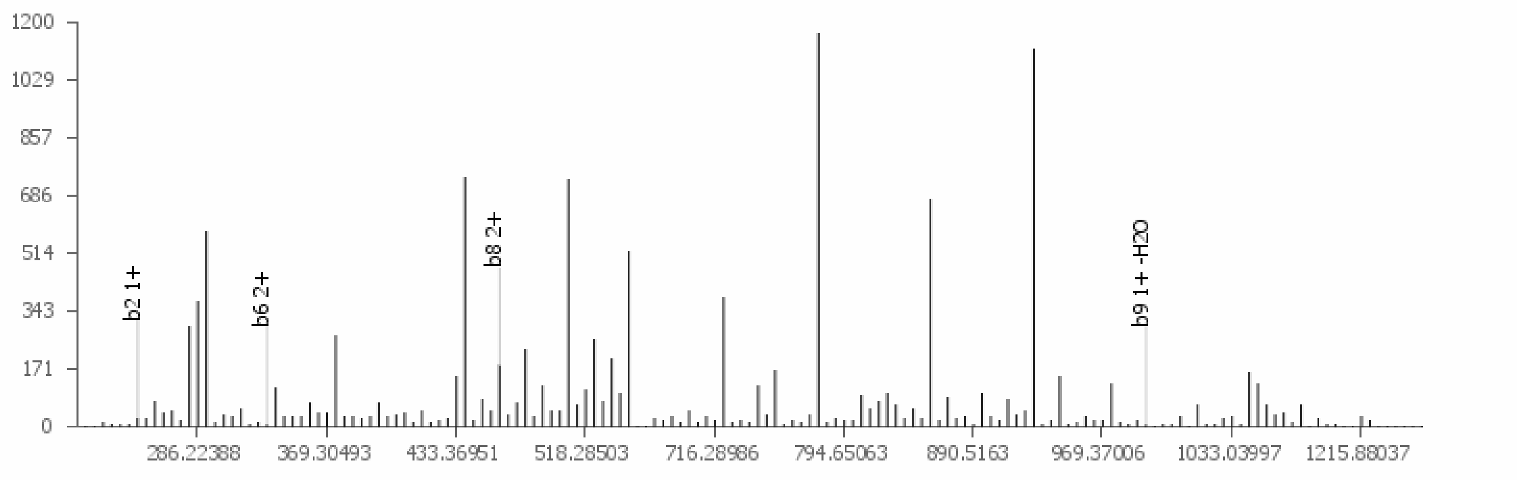

AT2G42150.1 - KG(t)(s)EPDK - 471.200235 - Charge:2

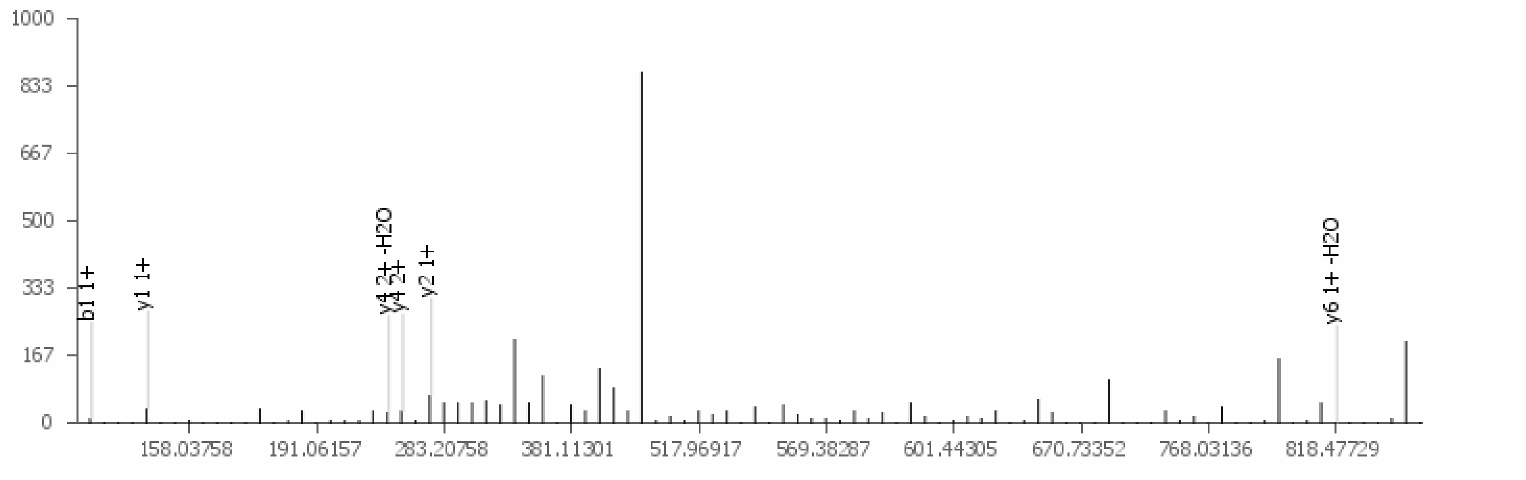

AT2G13790.1 - LE(pS)LVDAELEGK - 691.823523 - Charge:2

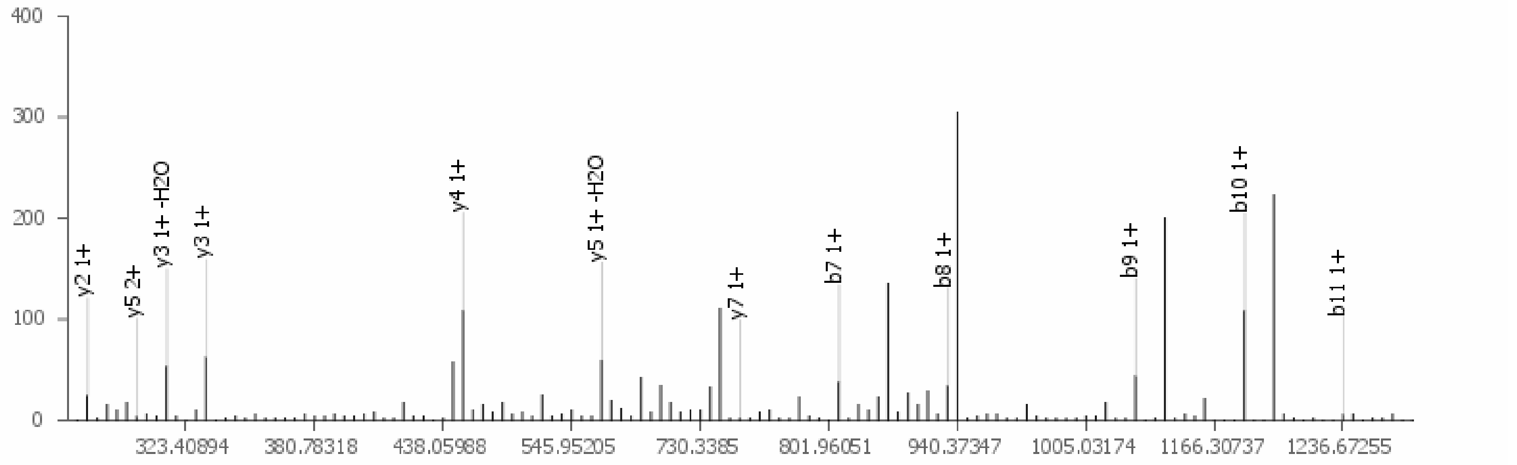

AT5G61350.1 - INIGGDLI(pS)PK - 603.806782 - Charge:2

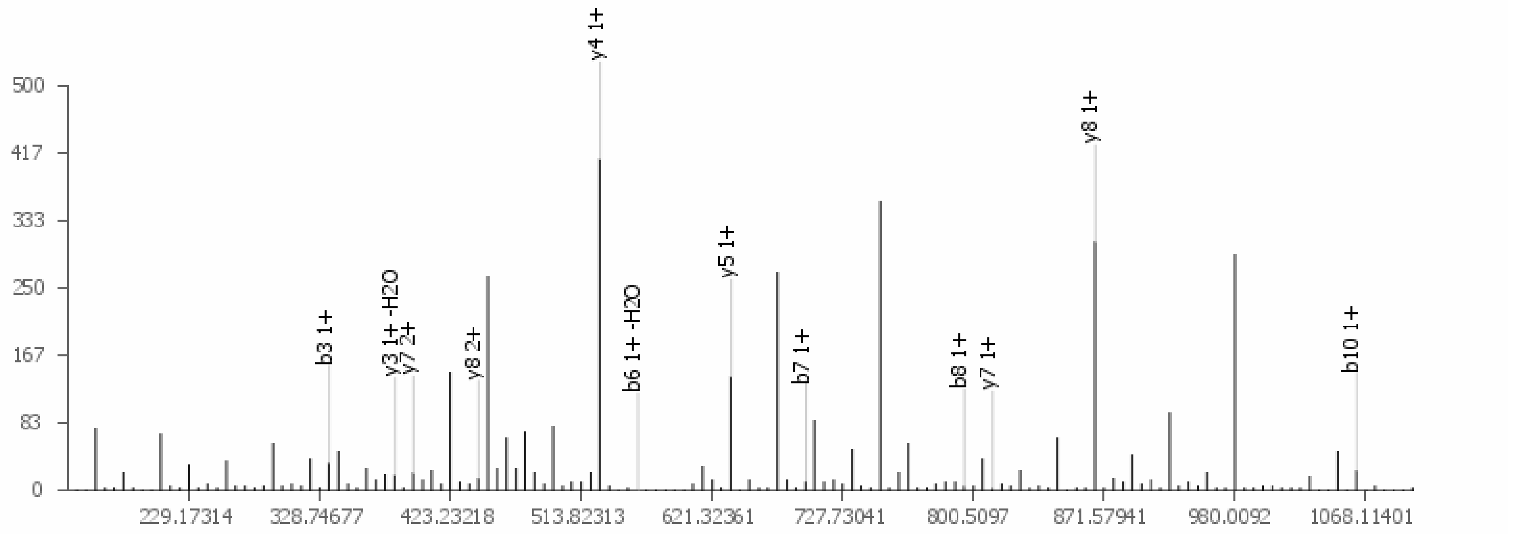

AT5G46750.1 - ES(pS)LKQEAAVVSSPK - 820.407585 - Charge:2

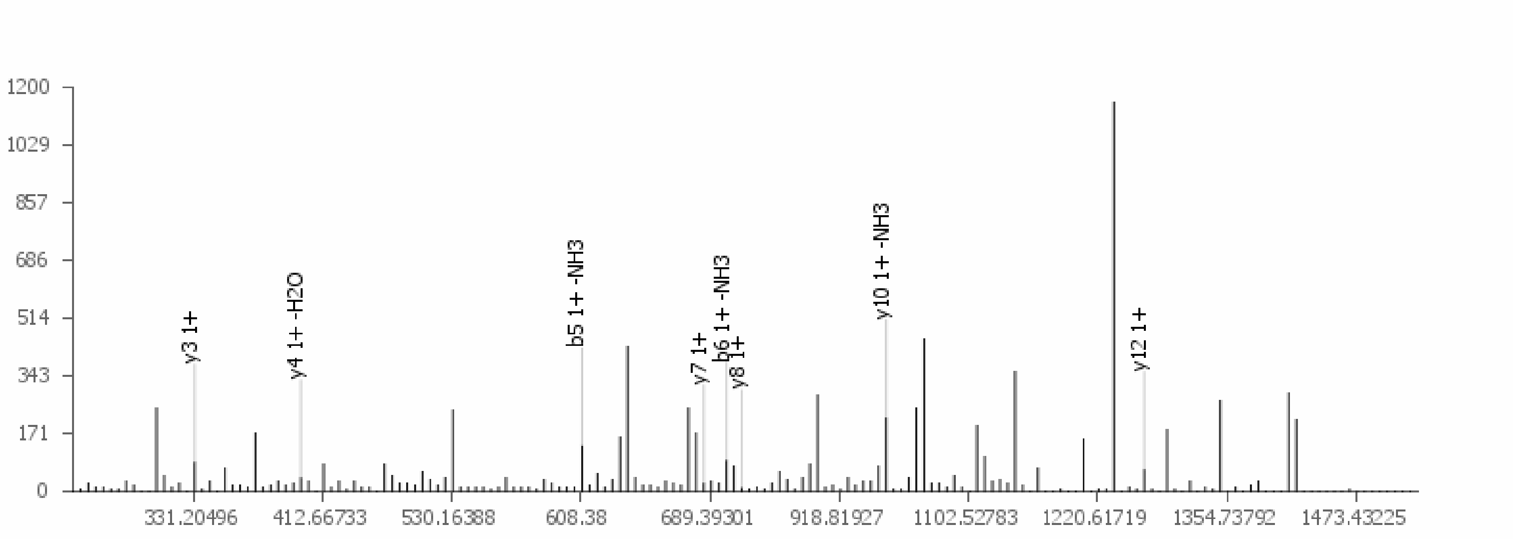

AT4G22860.1 - (pS)(pT)PKLPEFQEFHLK - 930.913111 - Charge:2

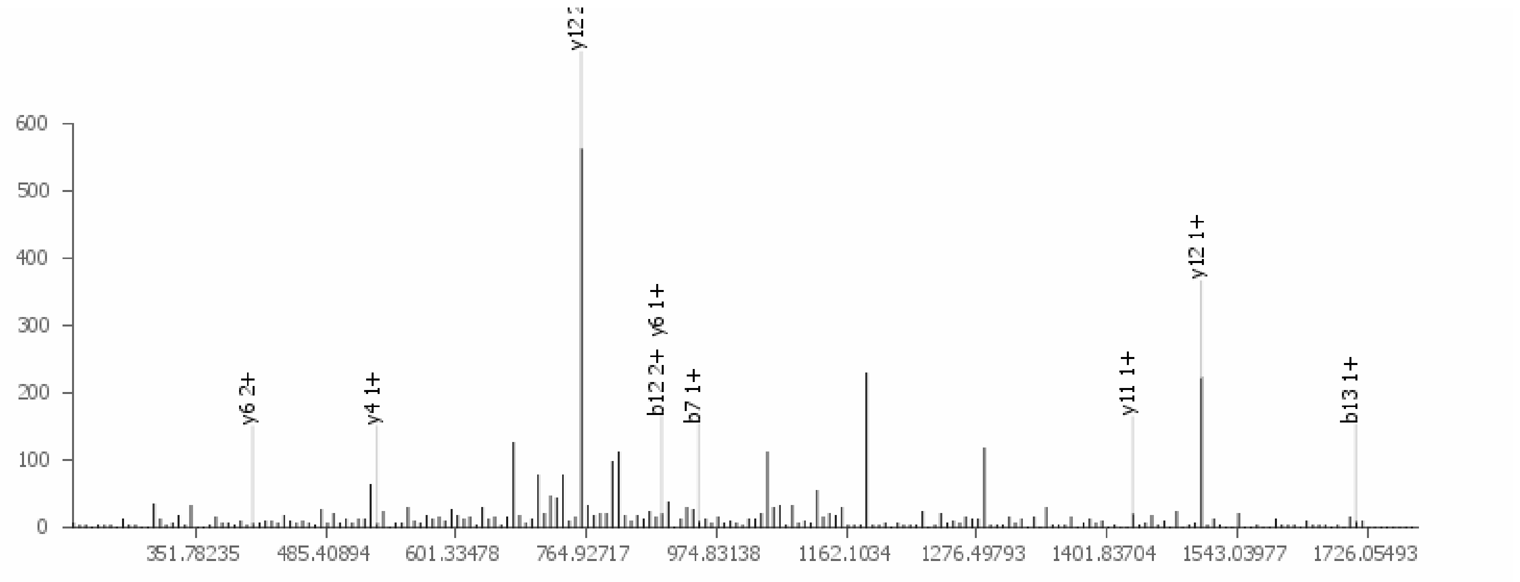

AT5G12280.1 - SPLF(pS)TSRPGGSR - 714.838942 - Charge:2

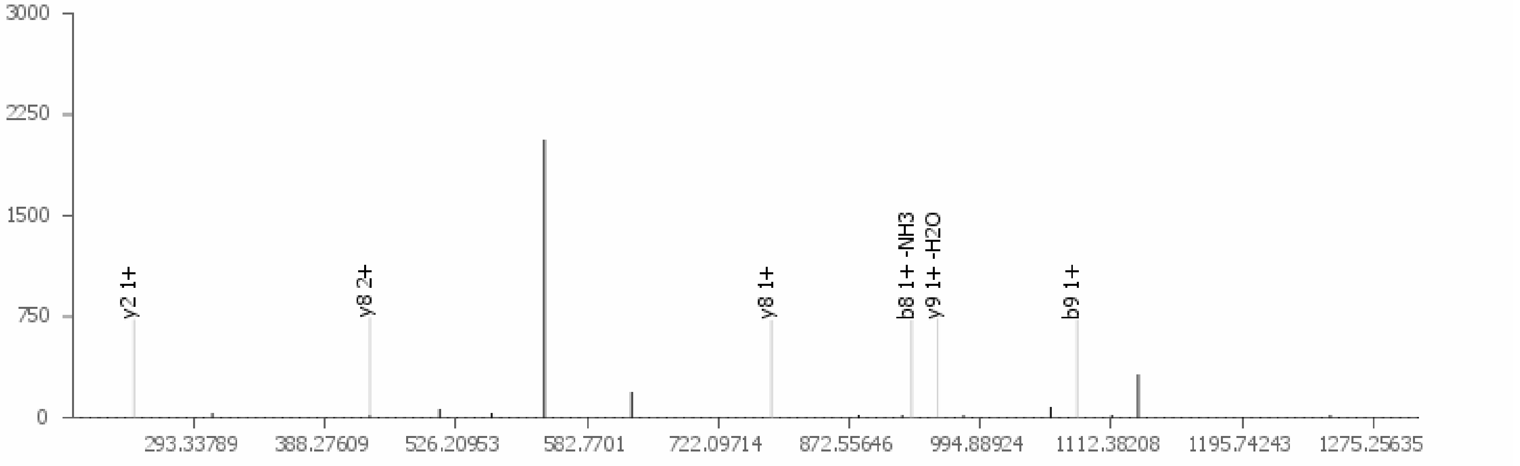

AT2G28100.1 - (pY)GEIKEVWLDGAK - 794.380521 - Charge:2

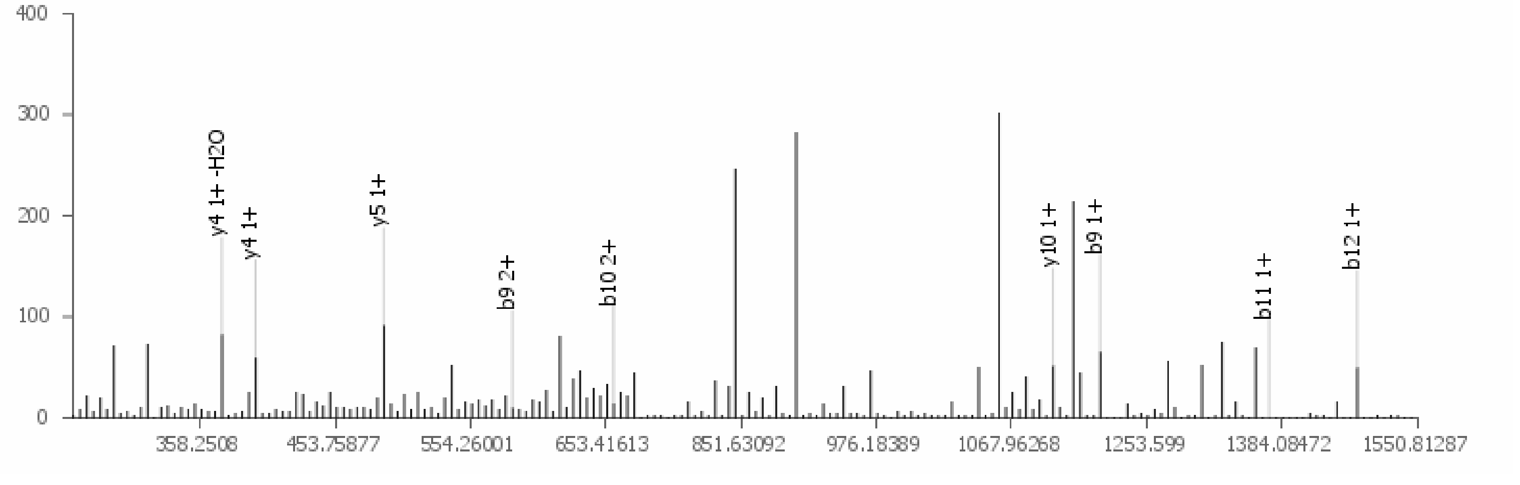

AT1G75180.1 - SEVATFI(s)(t)DNIR - 766.860058 - Charge:2

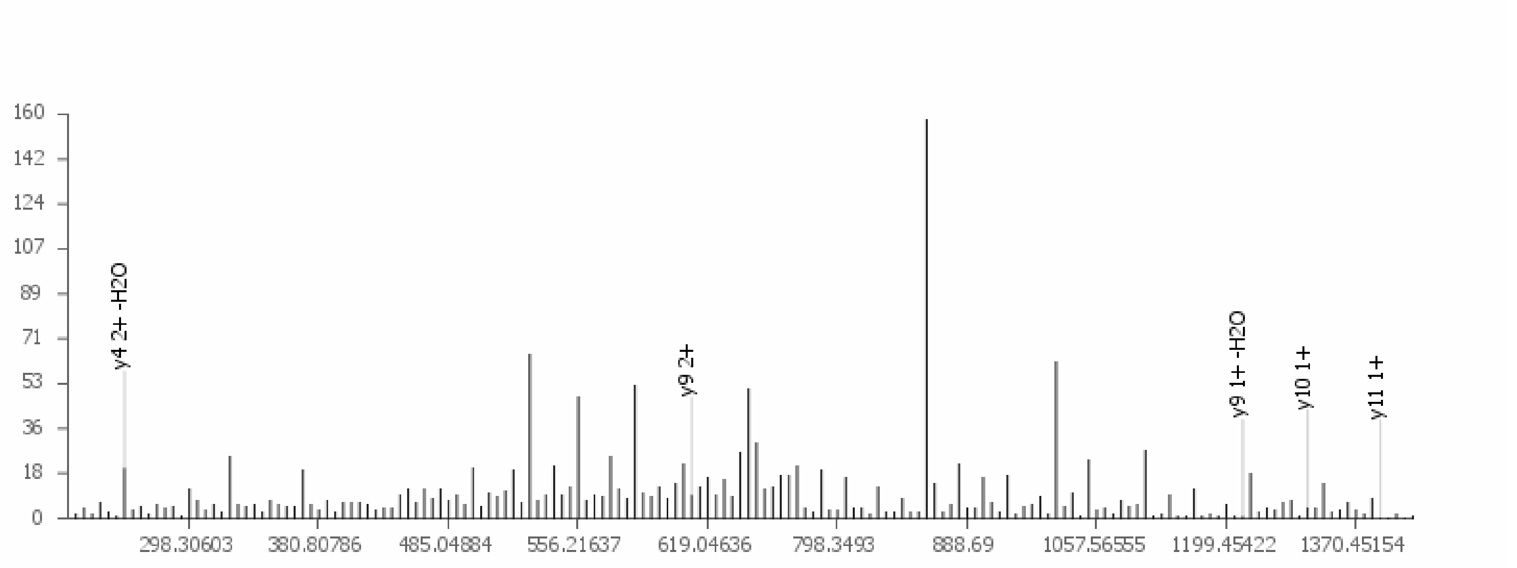

AT4G28370.1 - QEAEVGPF(s)AWNI(t)G(t)YR - 1053.478899 - Charge:2

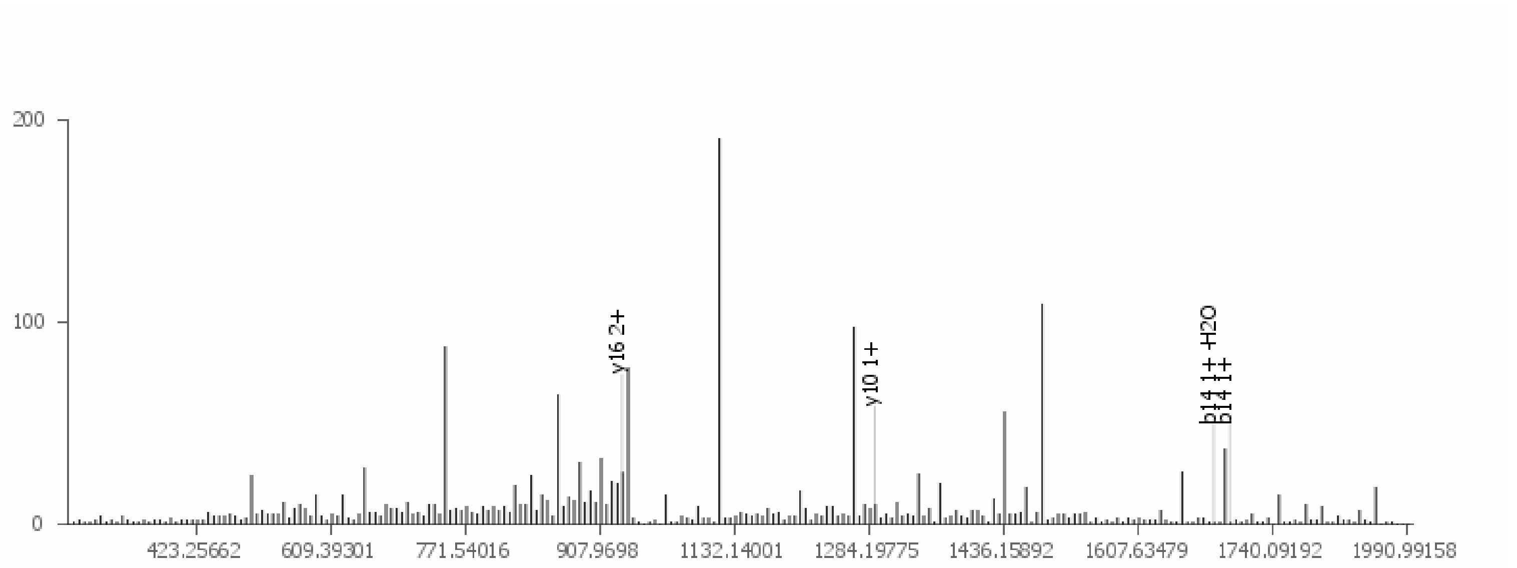

AT2G42690.1 - EFRDEVM(pS)HK - 679.284619 - Charge:2

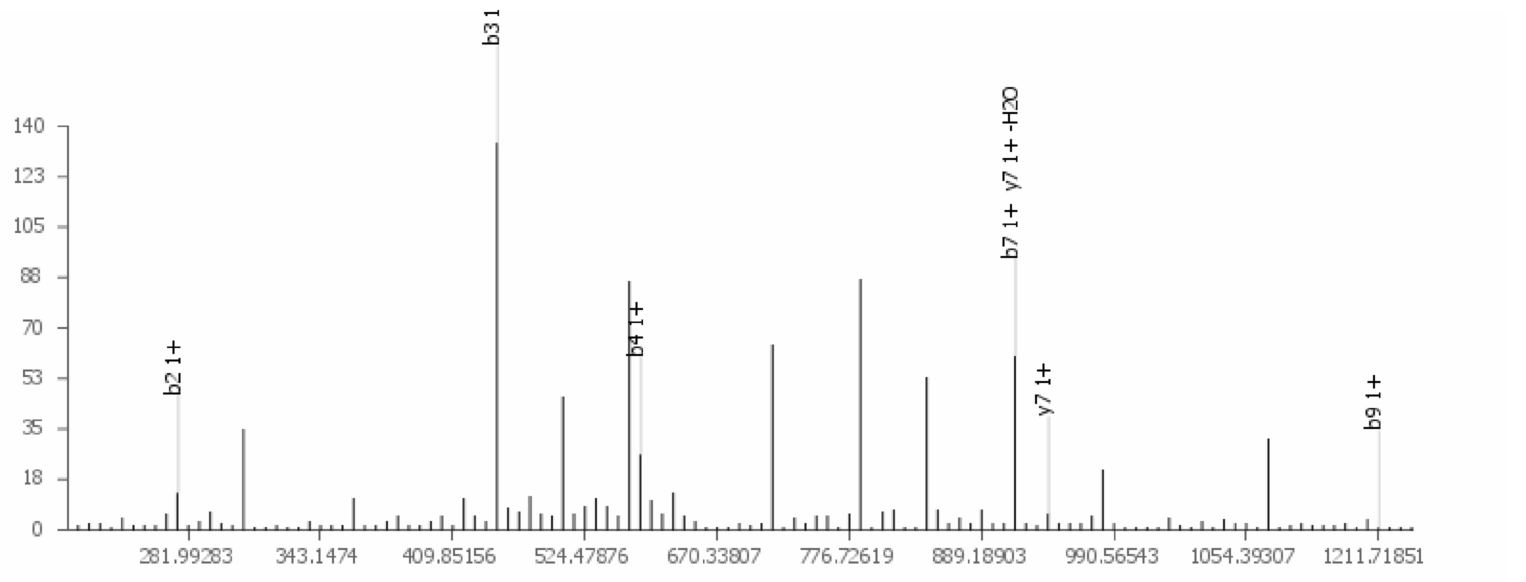

AT1G62120.1 - (pT)EFLVTEMNWPLK - 844.387201 - Charge:2

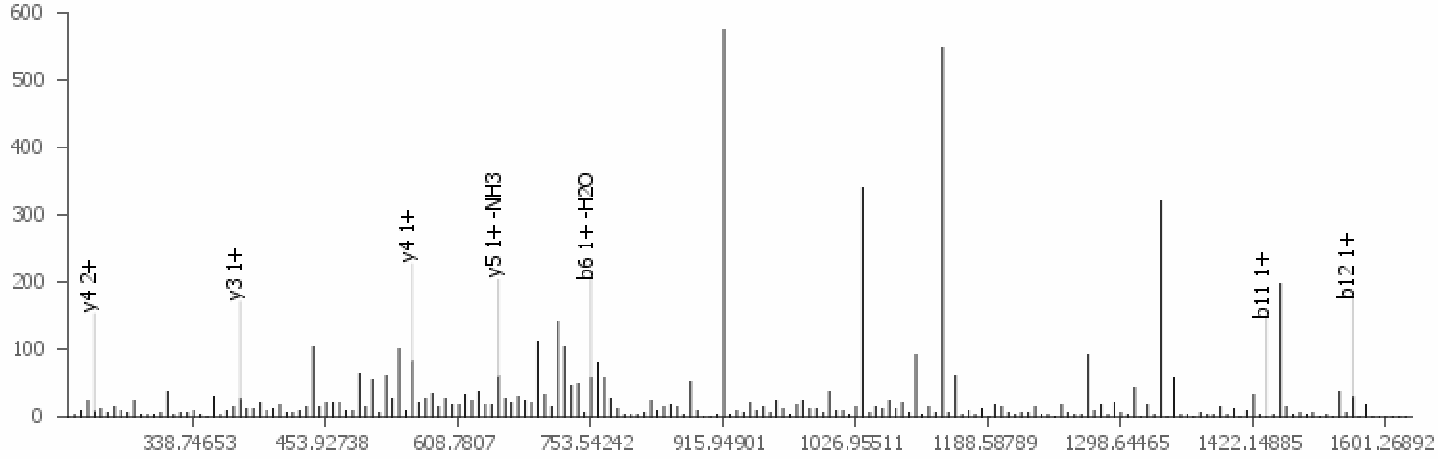

AT1G76010.1 - TV,(pT,)IVELIK - 553.288992 - Charge:2

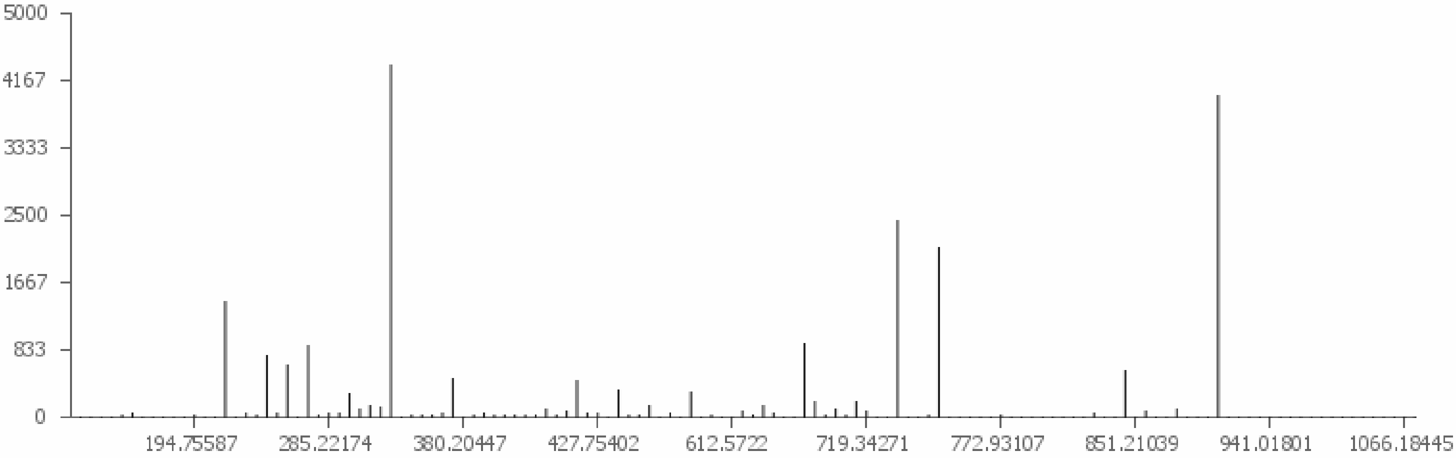

AT1G51300.1 - VTAEFH(pS)KK - 563.768084 - Charge:2

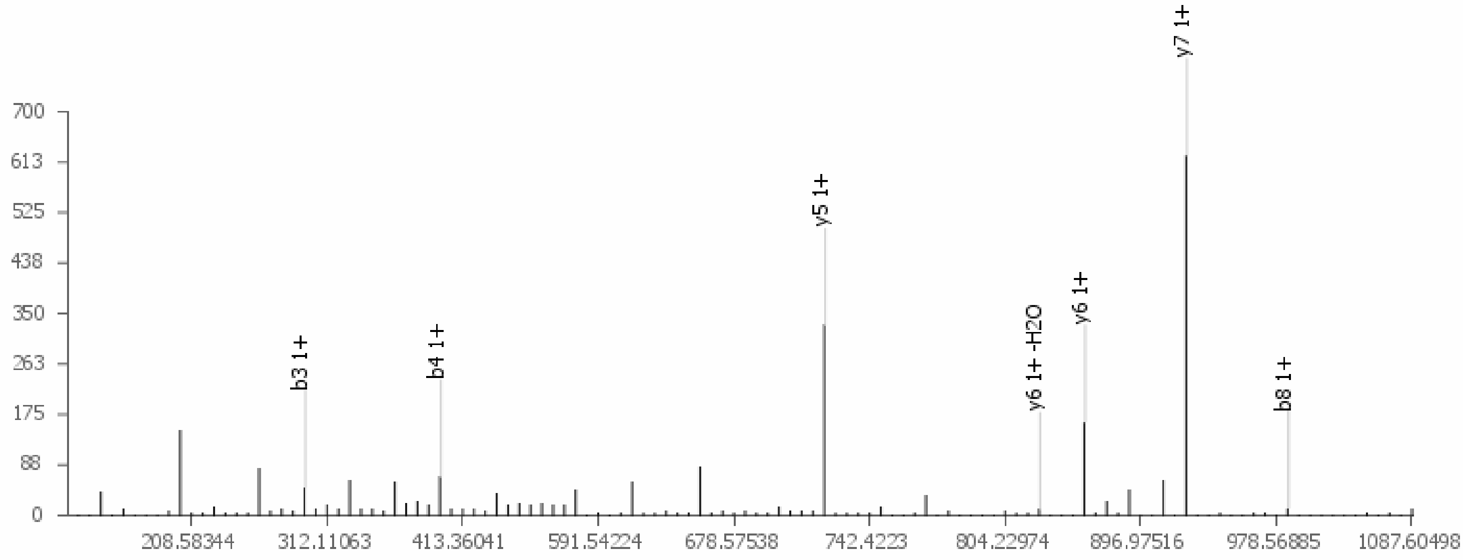

AT5G29560.1 - (pY)ARTVPDK - 515.240765 - Charge:2

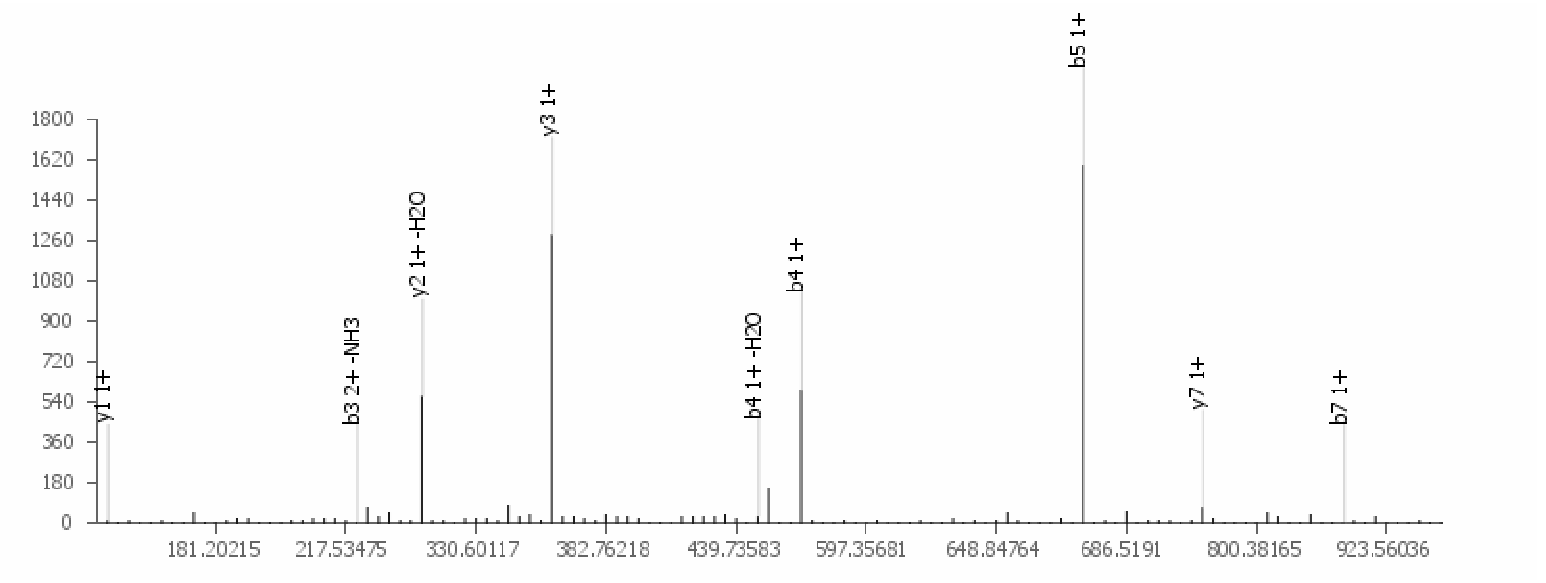

AT4G03115.1 - AAAL(t)A(s)QLA(t)YDEAK - 852.406452 - Charge:2

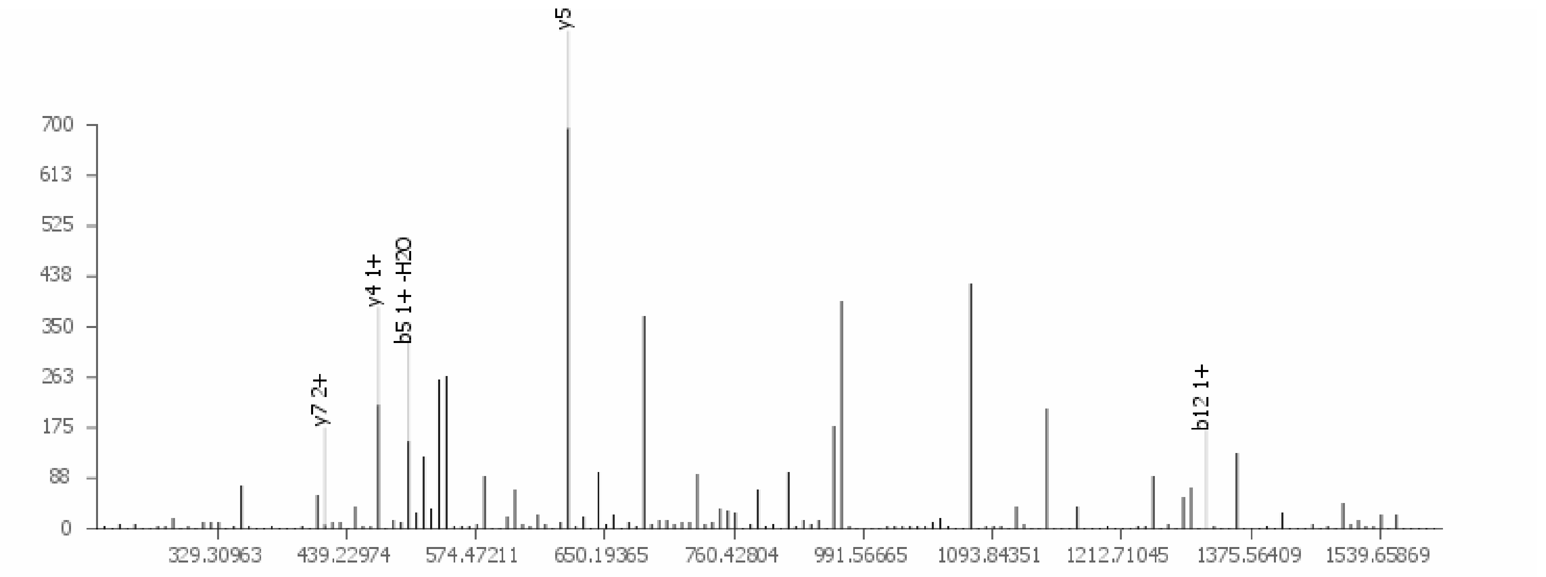

AT3G18020.1 - KEFVP(pS)LTNYNR - 774.370289 - Charge:2

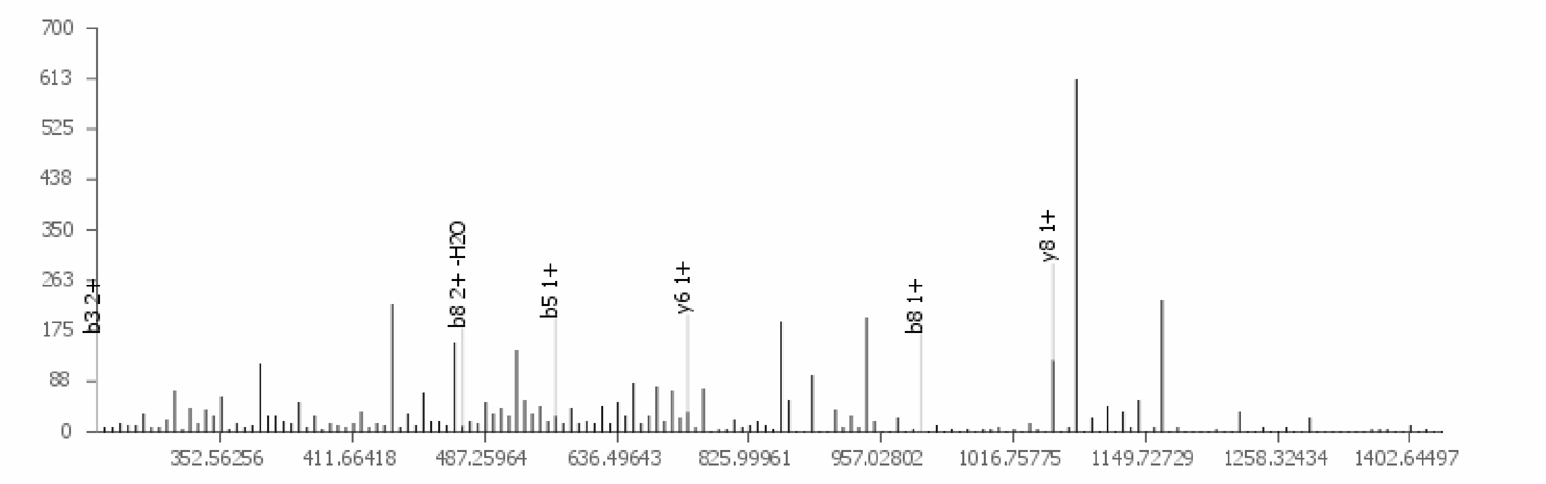

AT3G10820.1 - DSVLLE(pS)LR - 556.275914 - Charge:2

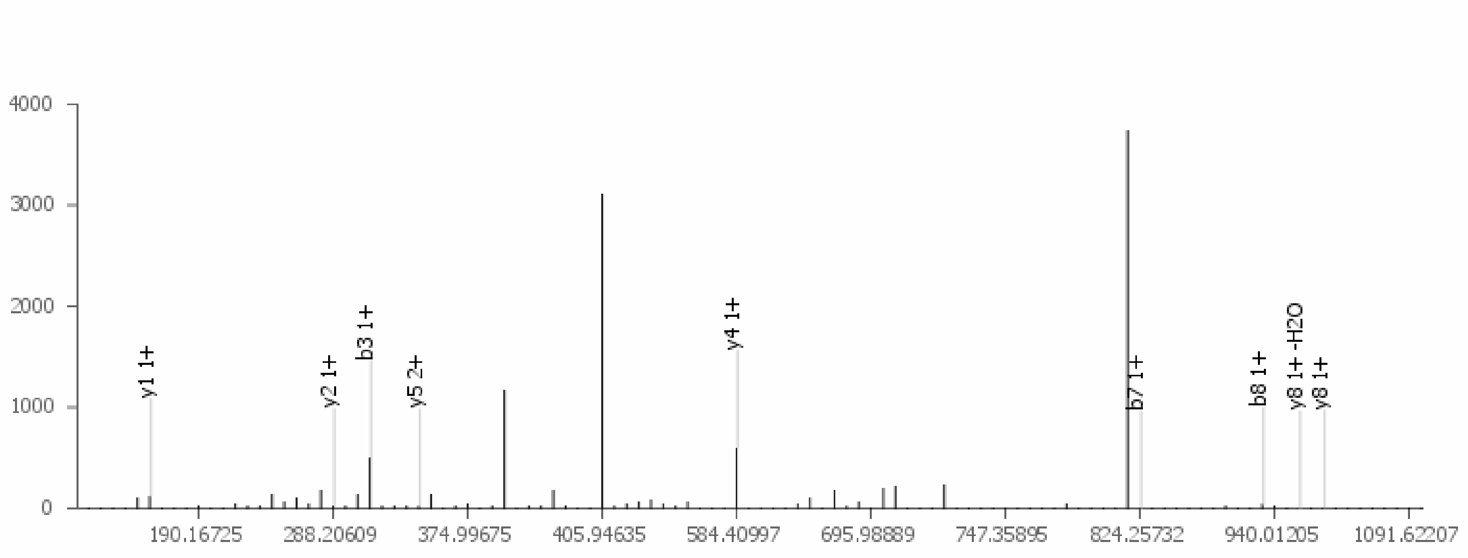

AT1G52360.1 - IKIF(pS)K - 408.22291 - Charge:2

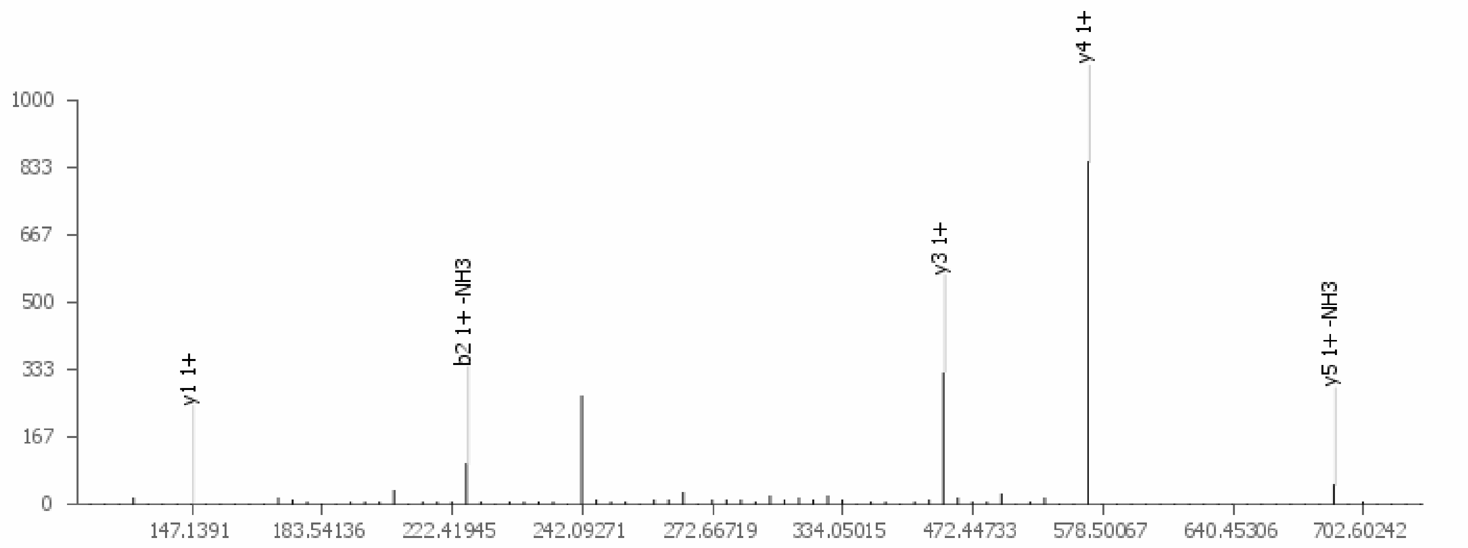

AT2G31820.1 - QL(pS)FIGERR - 593.293326 - Charge:2

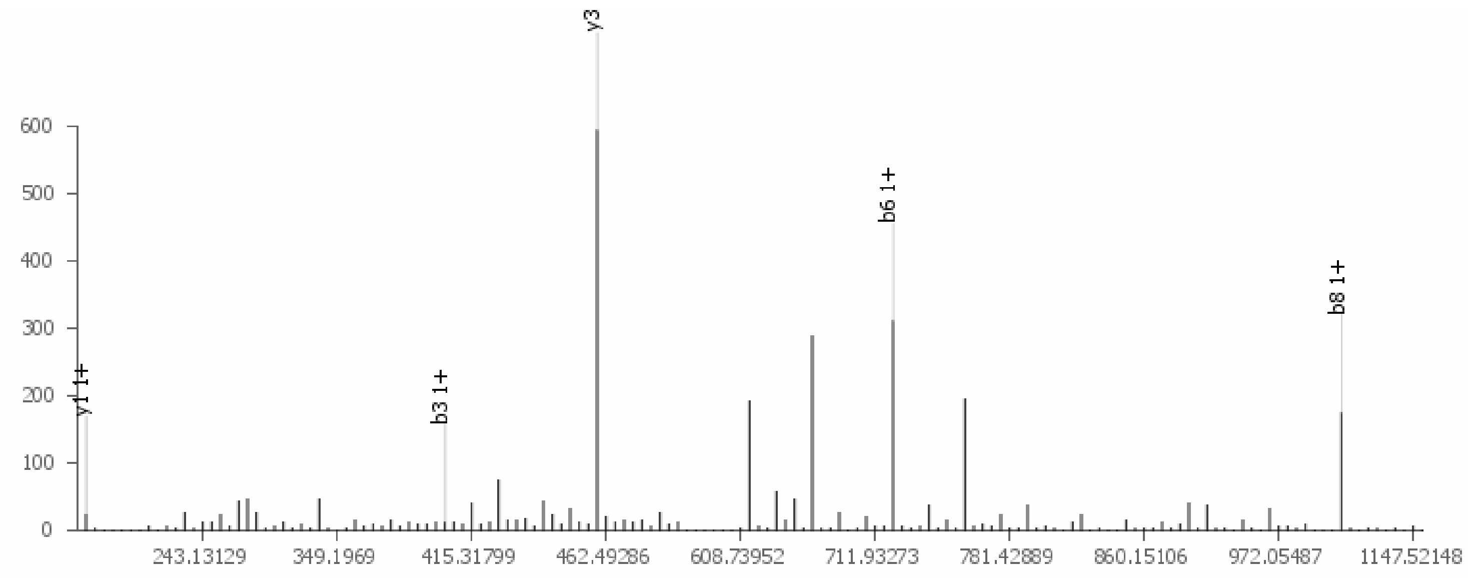

AT2G41370.1 - (pS)GLPPEILAK - 552.783748 - Charge:2

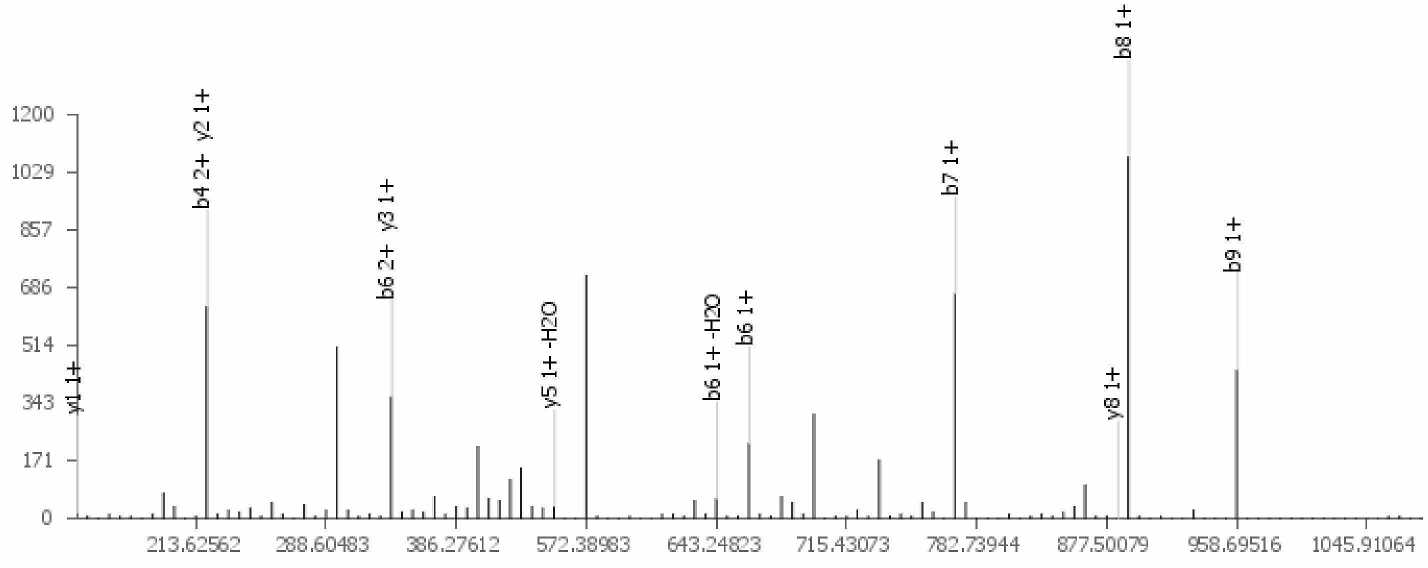

AT3G03500.1 - KFFE(t)(t)PTAAVGEIGLDK - 1002.482127 - Charge:2

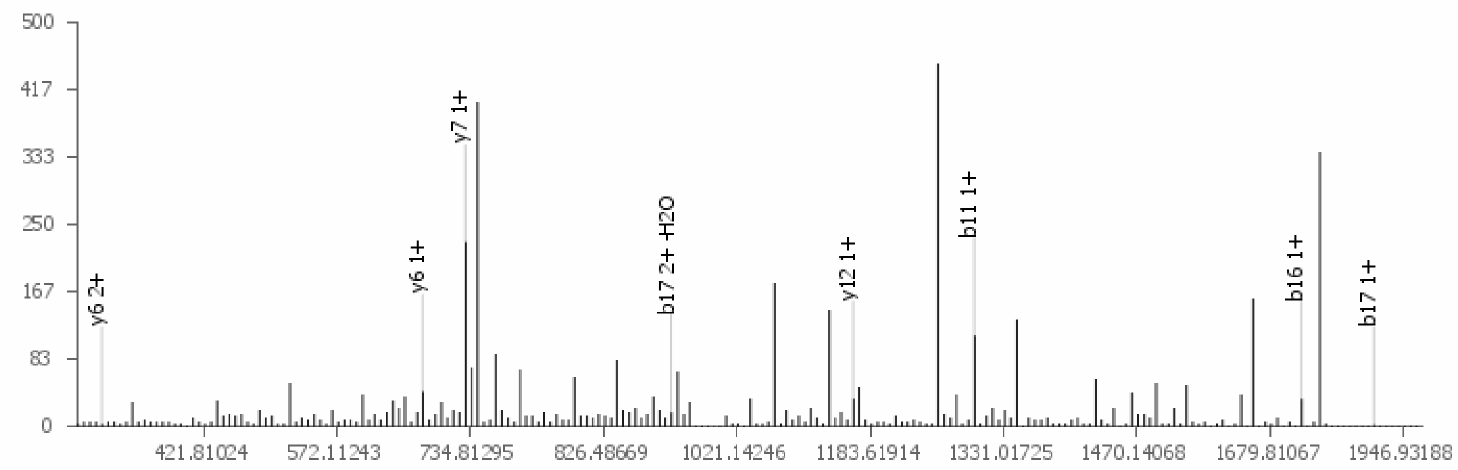

AT5G42140.1 - (pY)NIRGTDR - 537.736337 - Charge:2

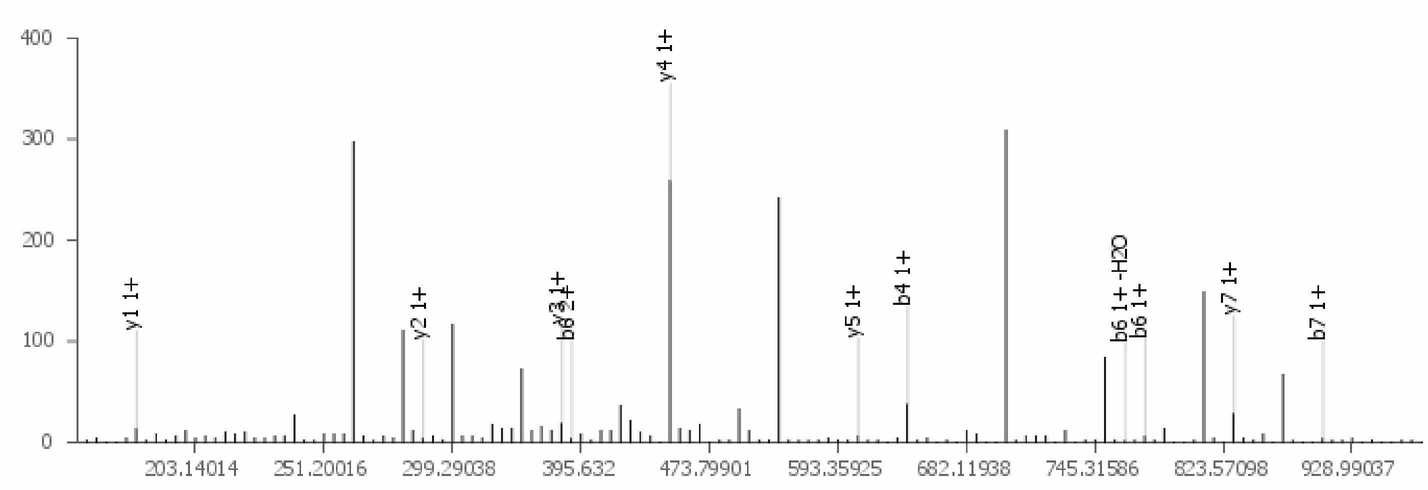

AT3G21790.1 - LLEDY(s)(s)KPDSPK - 779.860358 - Charge:2

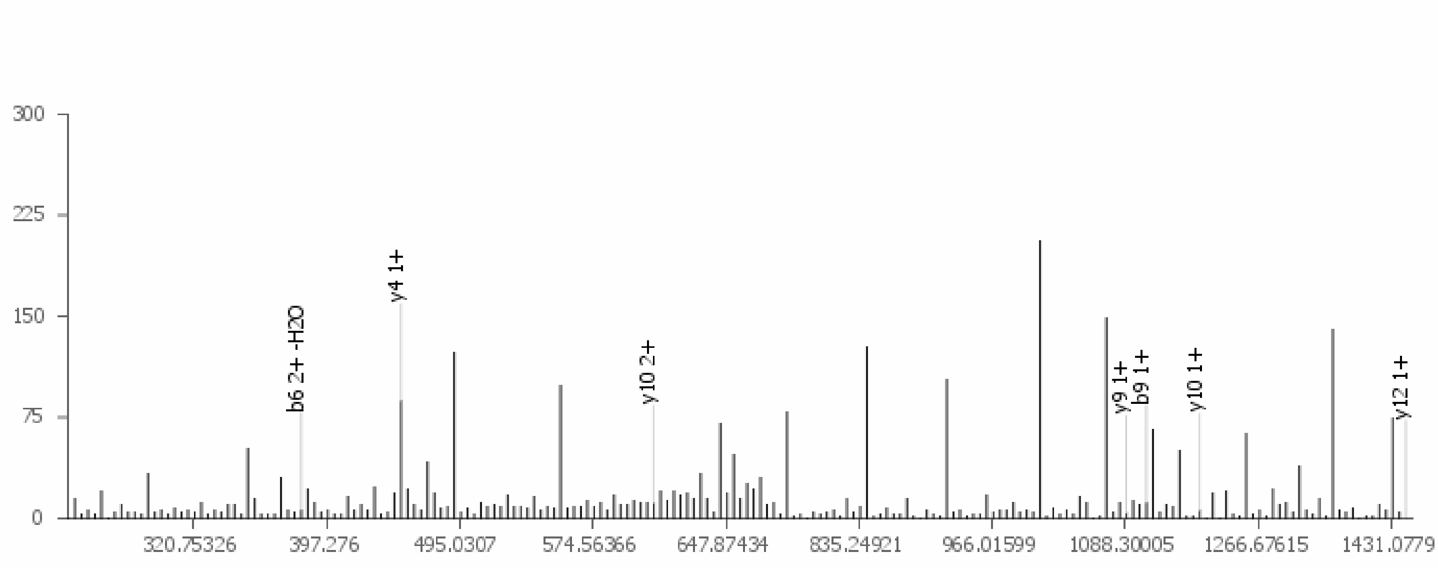

AT5G66680.1 - FGGSLDSKSIADFVD(pS)GR - 969.436493 - Charge:2

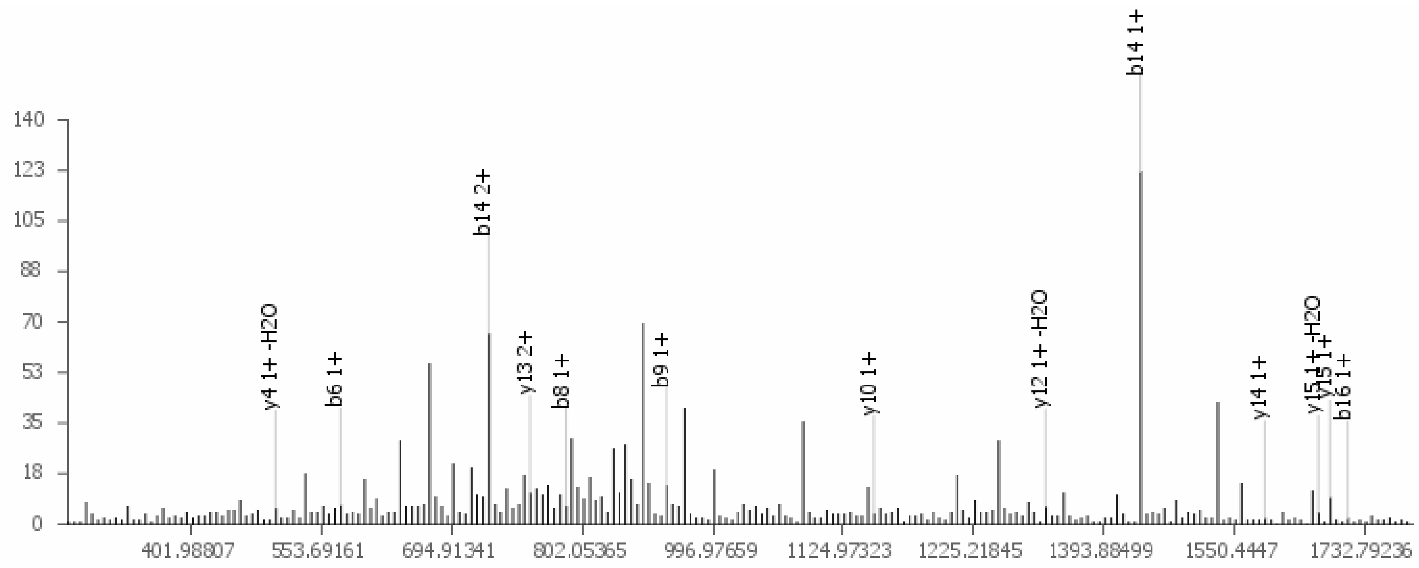

AT5G02330.1 - EAFPT(pY)VCLR - 639.78045 - Charge:2

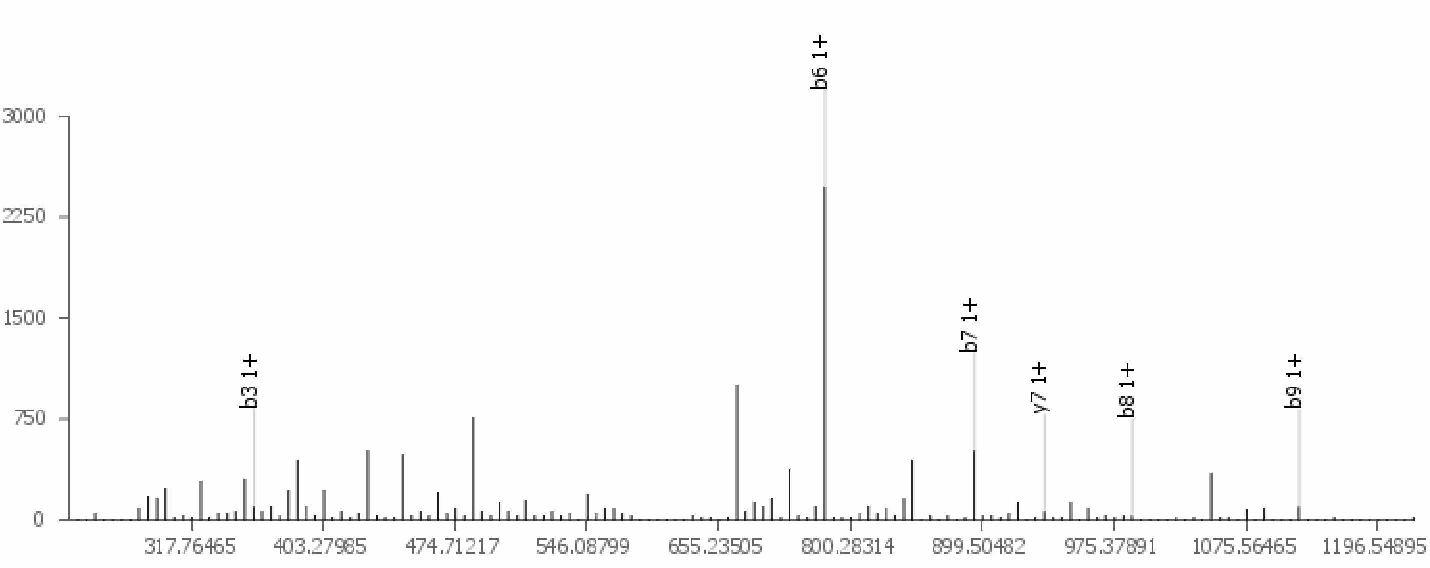

AT1G19690.1 - DLGV(s)(t)QILRLGGIYGPGR - 1039.50398 - Charge:2

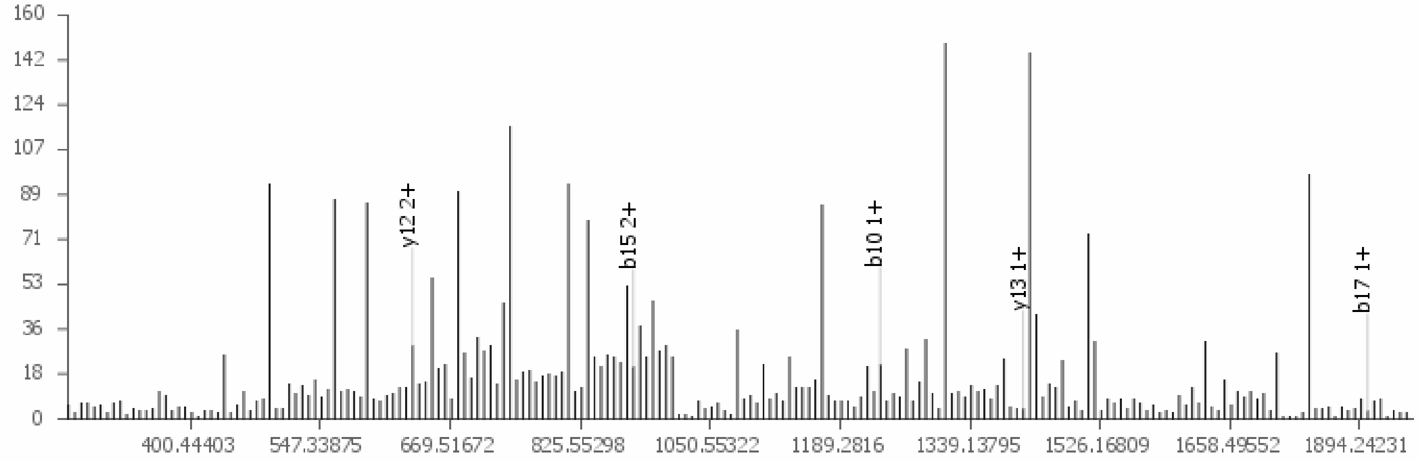

AT1G50900.1 - ANSEEIRDLILG(pY)(s)(t)QK - 1048.978318 - Charge:2

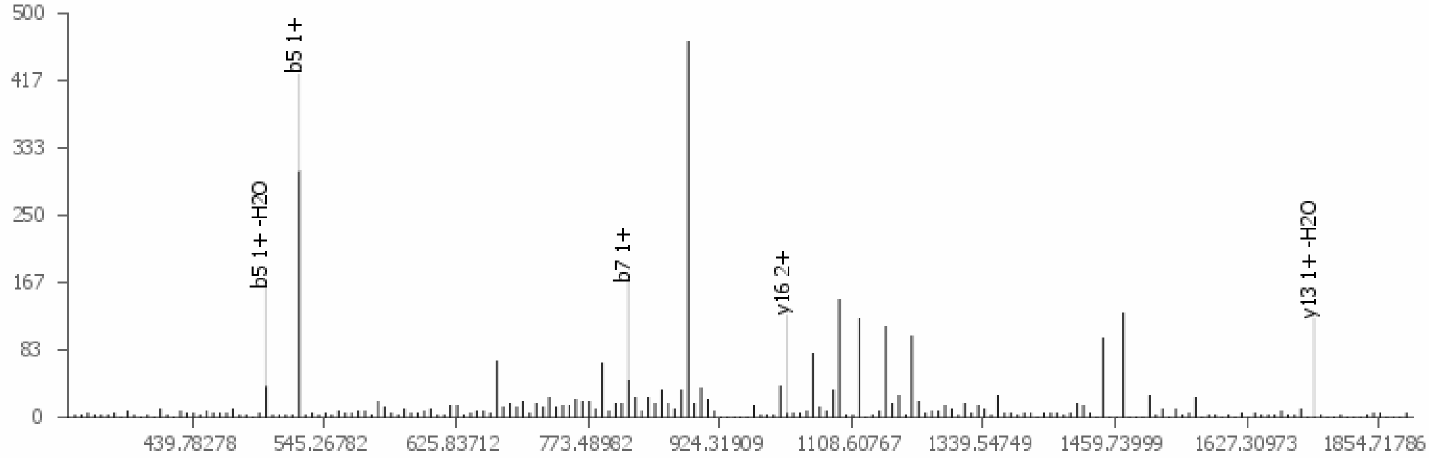

AT1G54300.1 - NLEKLVTD(s)FW(s)K - 823.890034 - Charge:2

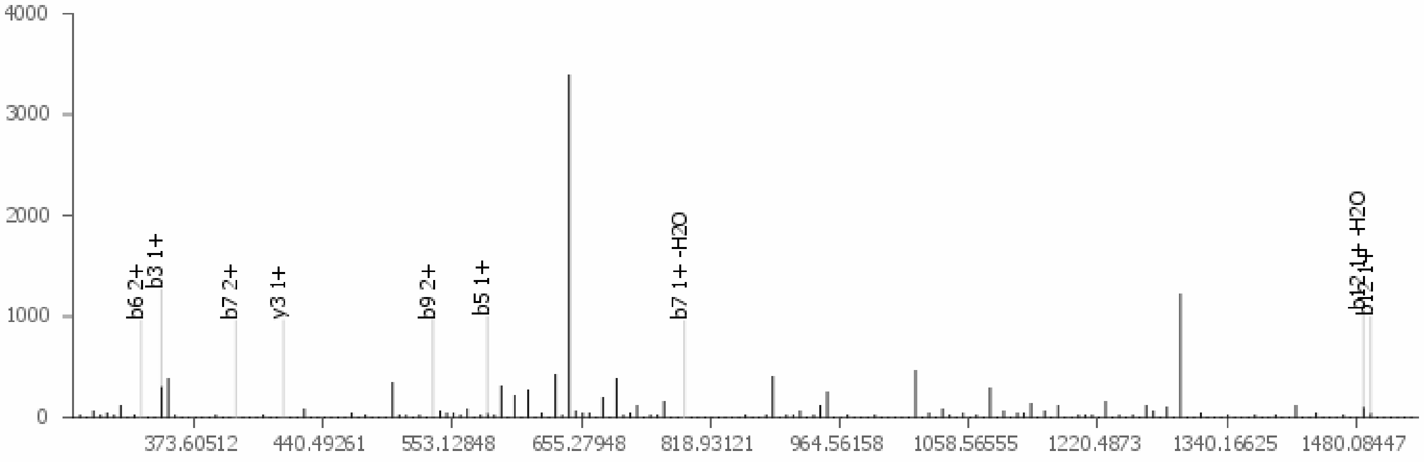

AT1G27750.1 - DLSCGR(s)(s)VPR - 628.7729 - Charge:2

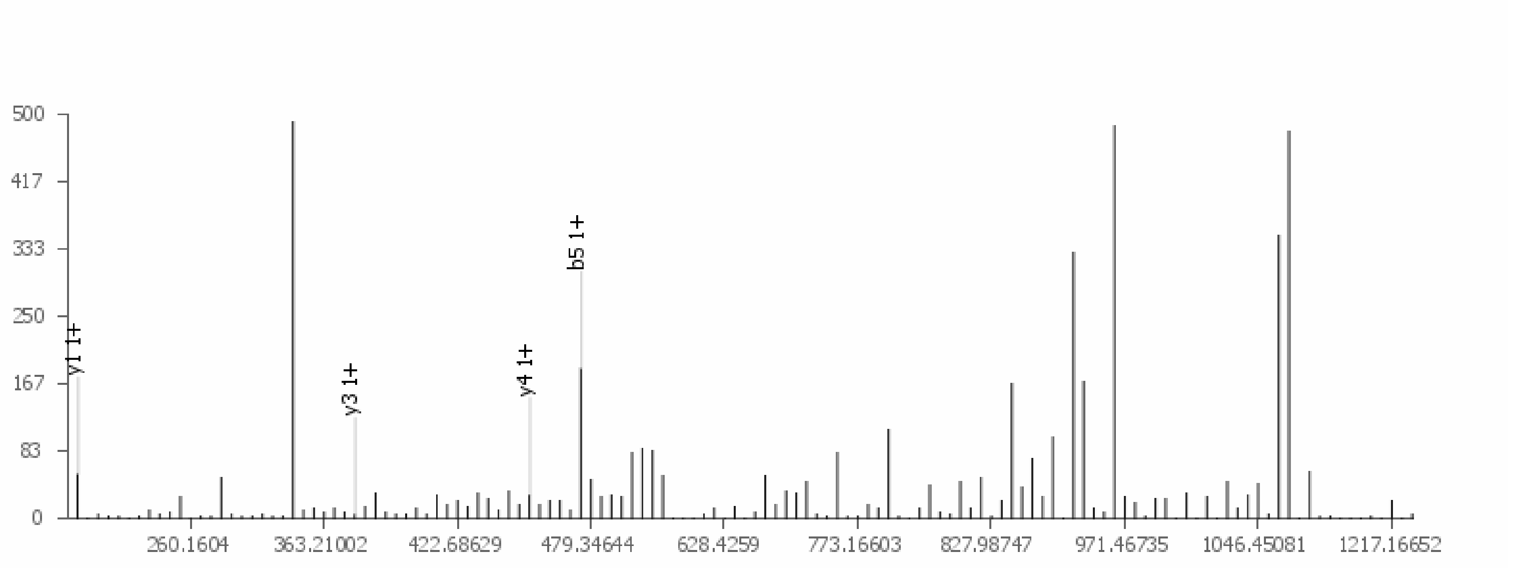

AT5G44600.1 - YLLLDSTKNVH,(pS,)VINK - 973.458295 - Charge:2

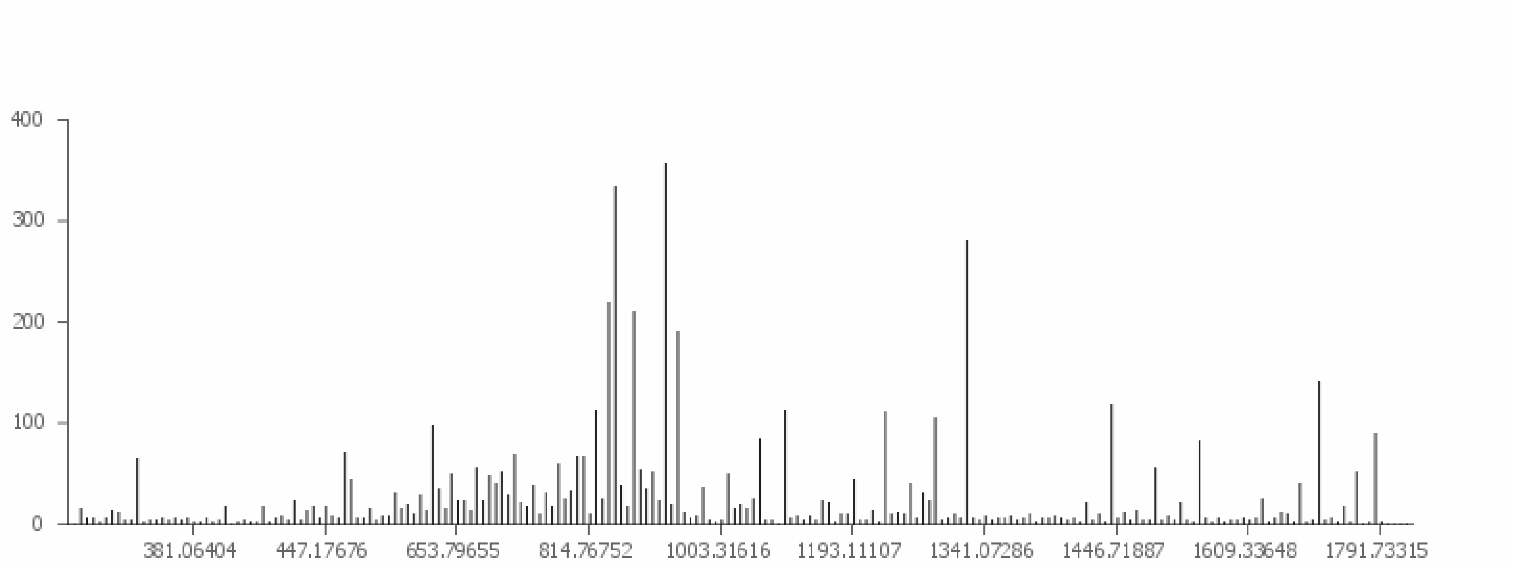

AT5G55320.1 - RWNLIV(pS)ASLR - 697.865331 - Charge:2

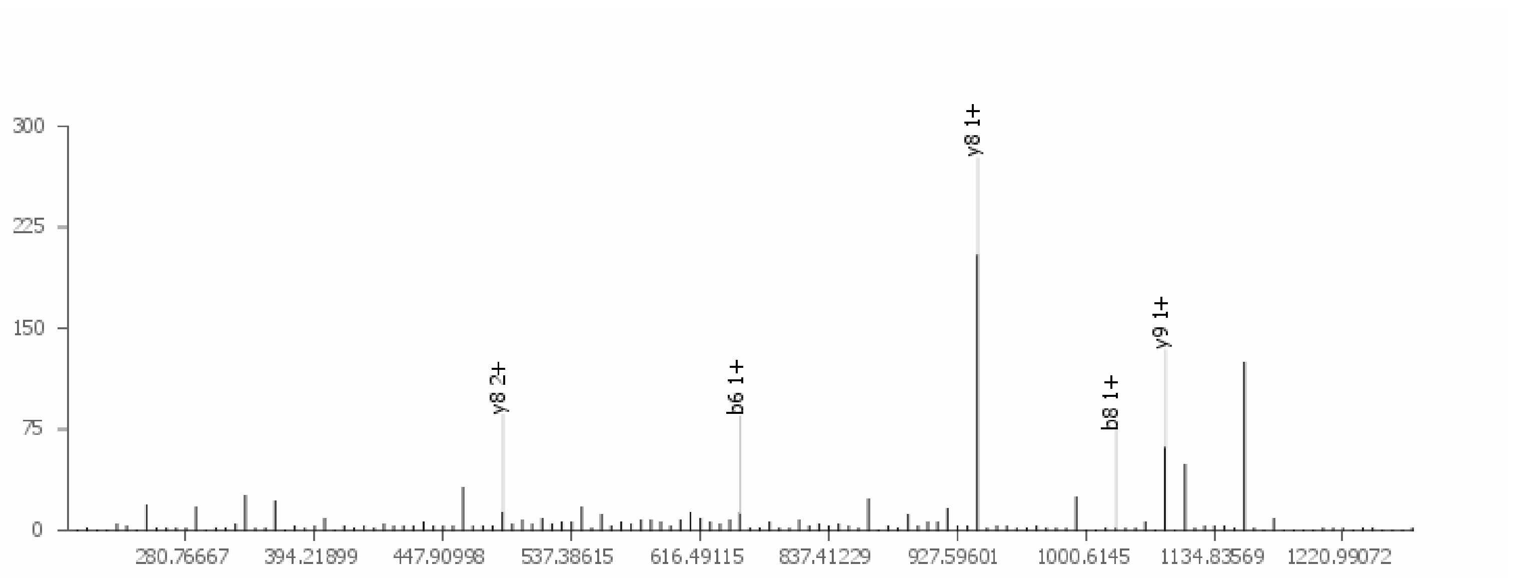

AT5G23450.1 - KQ(pS)I(pT)PIFEDK - 733.321536 - Charge:2

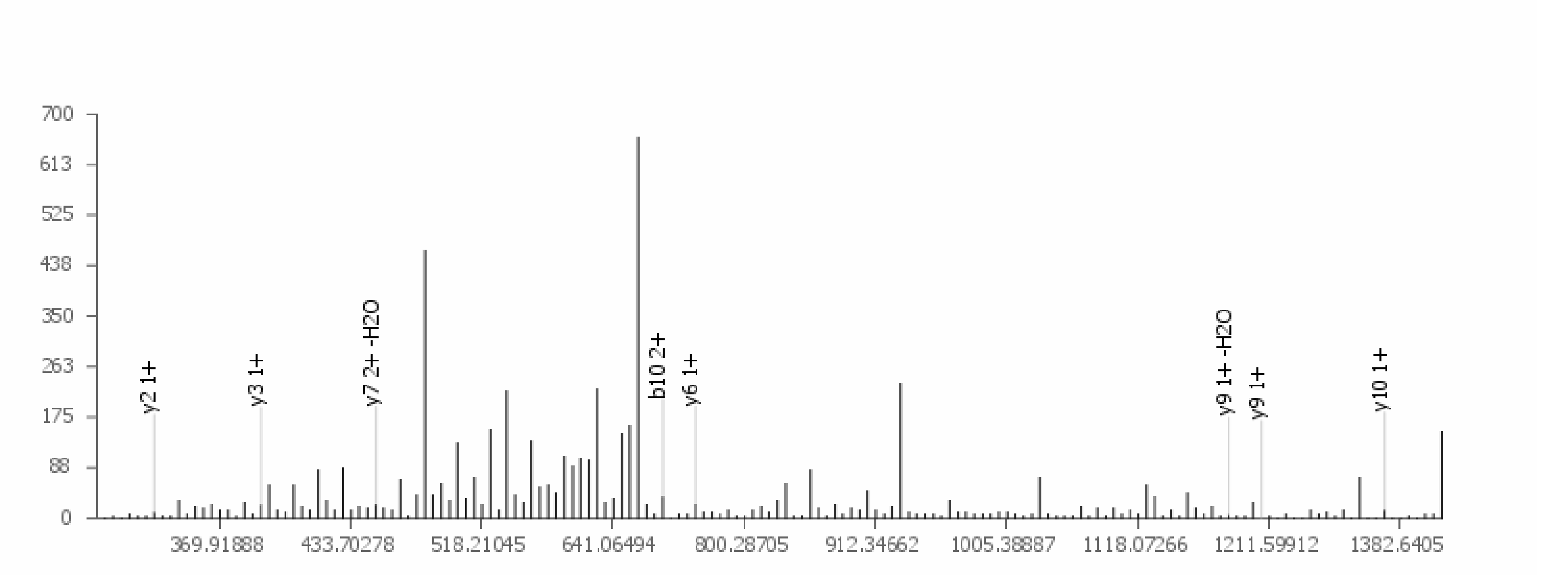

AT5G65080.1 - QLSILCGSSVALFIVSSTGKL(pY)NSSSGD(pS)MAK - 1137.526952 - Charge:3

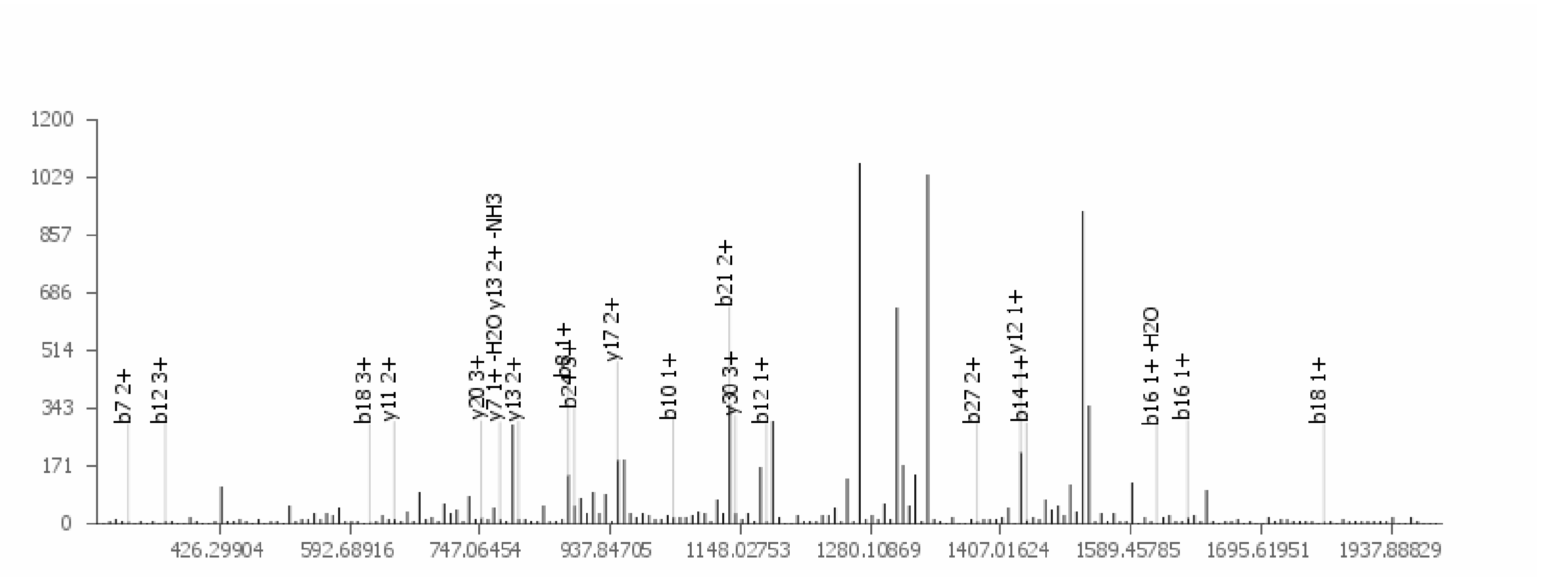

AT5G38396.1 - LFKLD(pS)DR - 610.791412 - Charge:2

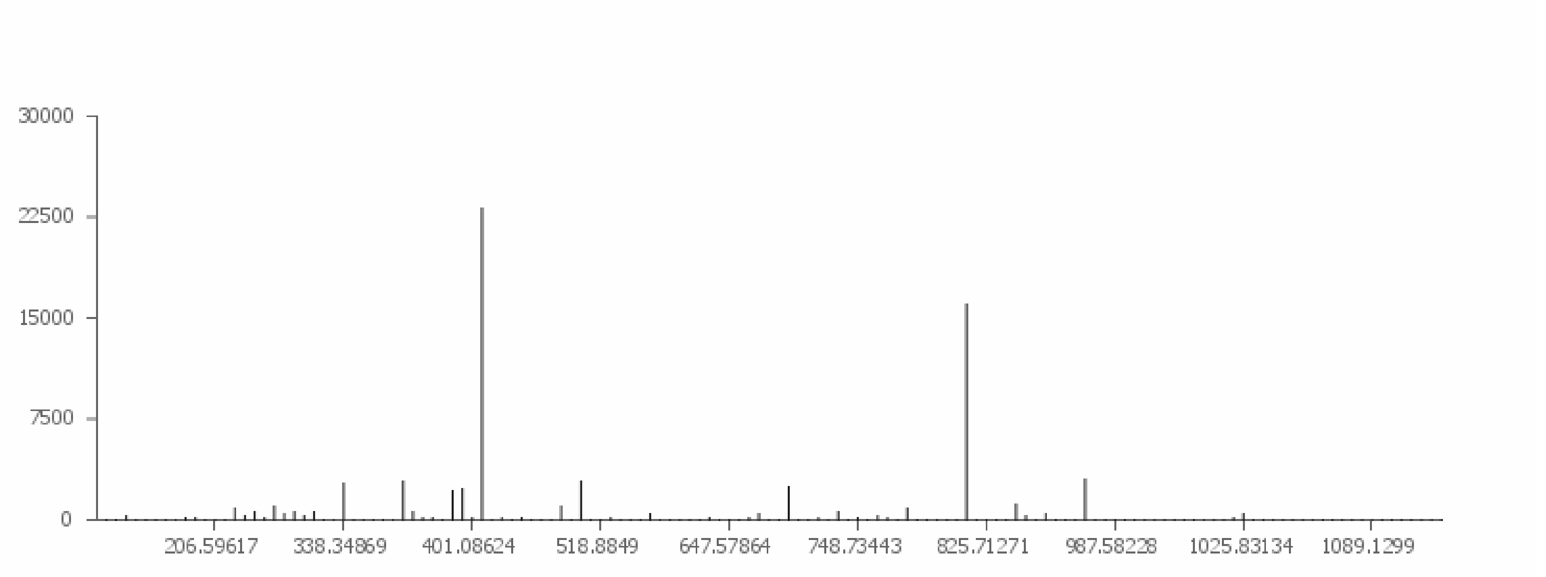

AT1G77140.1 - AV(pY)FIRPTSDNIQK - 866.435108 - Charge:2

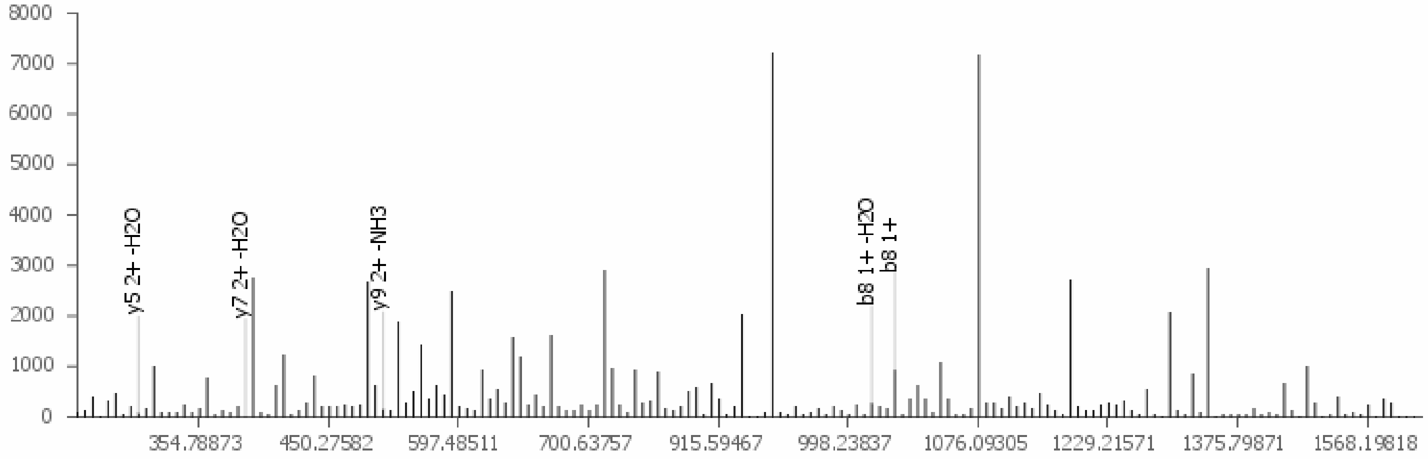

ATCG00860.1 - KEQDVSFVP(pS)R - 686.32261 - Charge:2

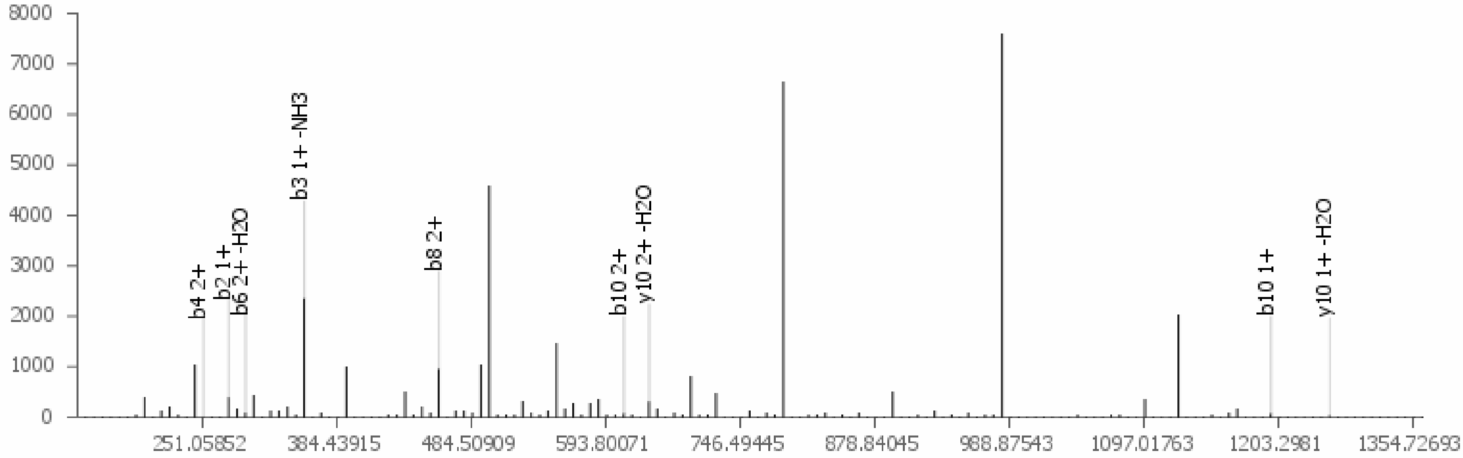

AT5G61020.1 - DYNKIDFPET(pY)TEAK - 957.404534 - Charge:2

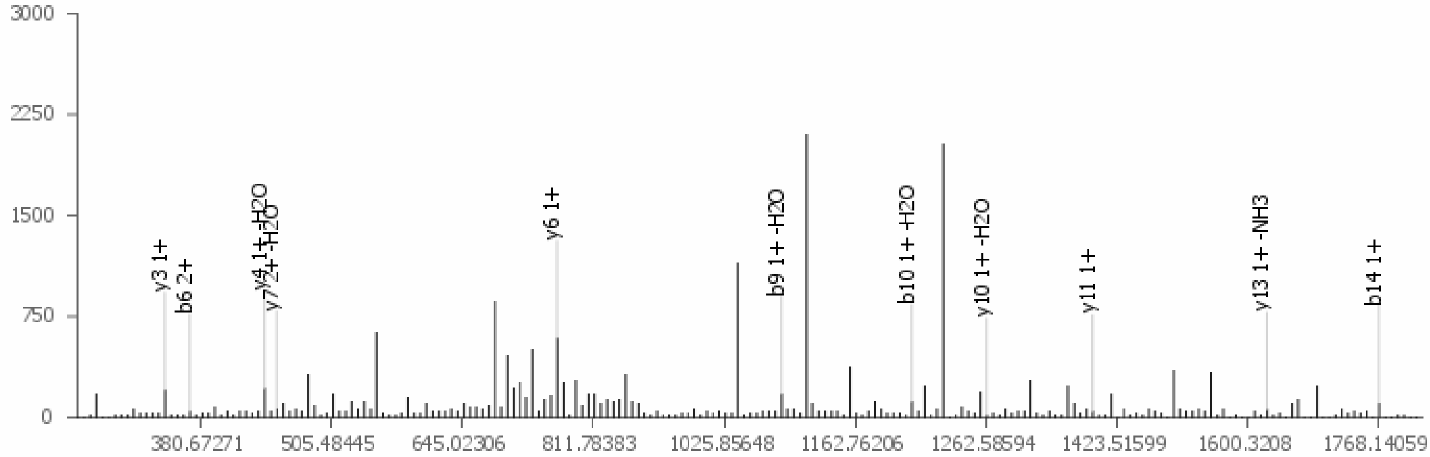

AT1G42740.1 - QTAK(s)(s)(t)APLLLNPPPK - 961.967216 - Charge:2

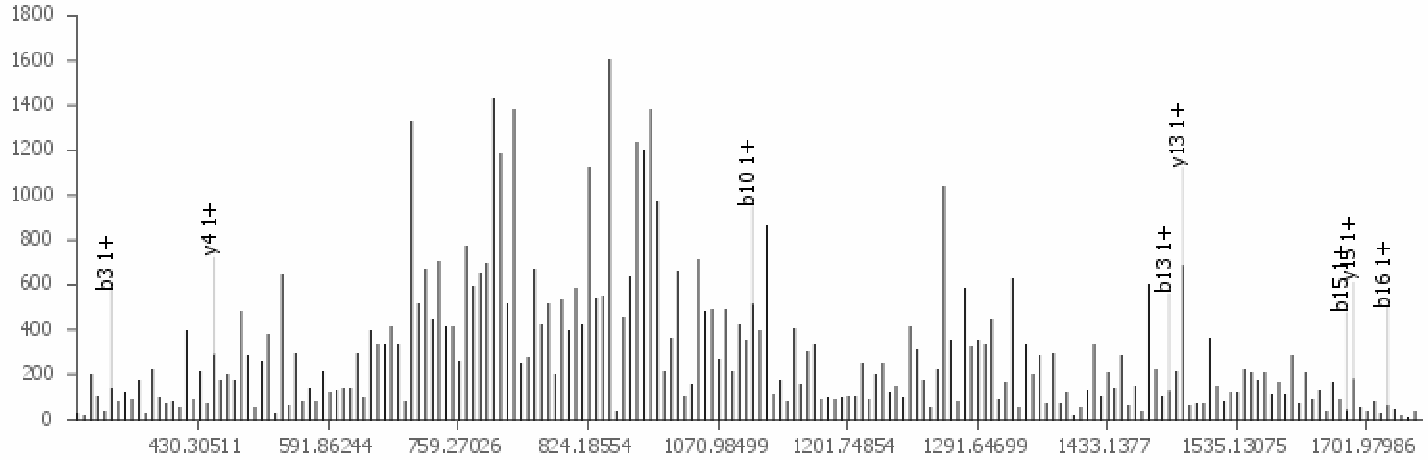

AT3G42800.1 - LT(pS)FLNSIFQSNAK - 825.408258 - Charge:2

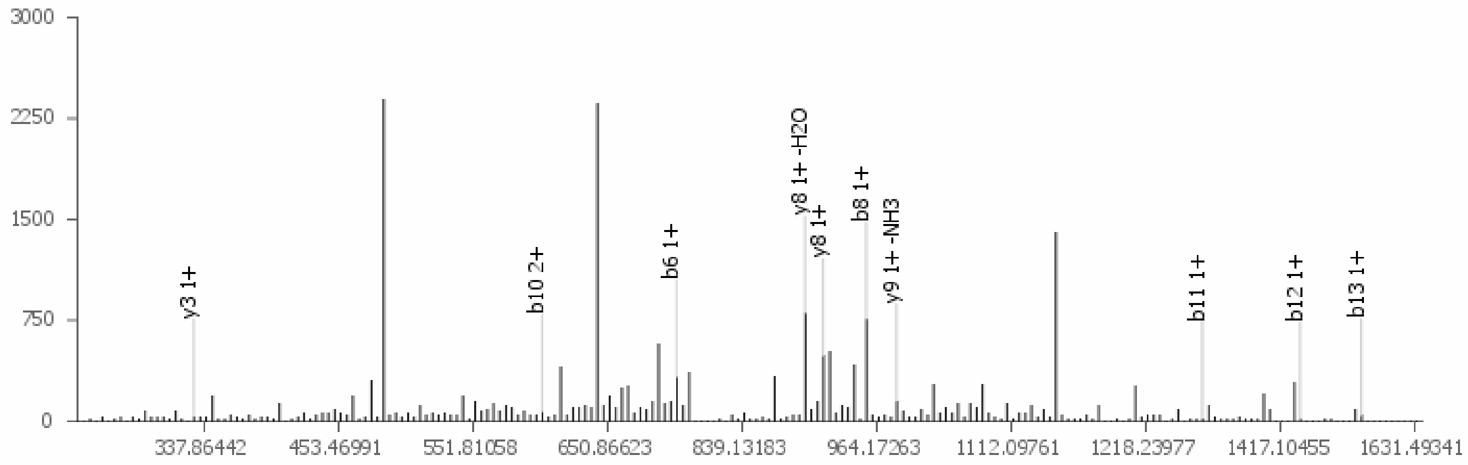

AT2G18080.1 - FQAGVVSLEHR(pY)(pY)GK - 957.416045 - Charge:2

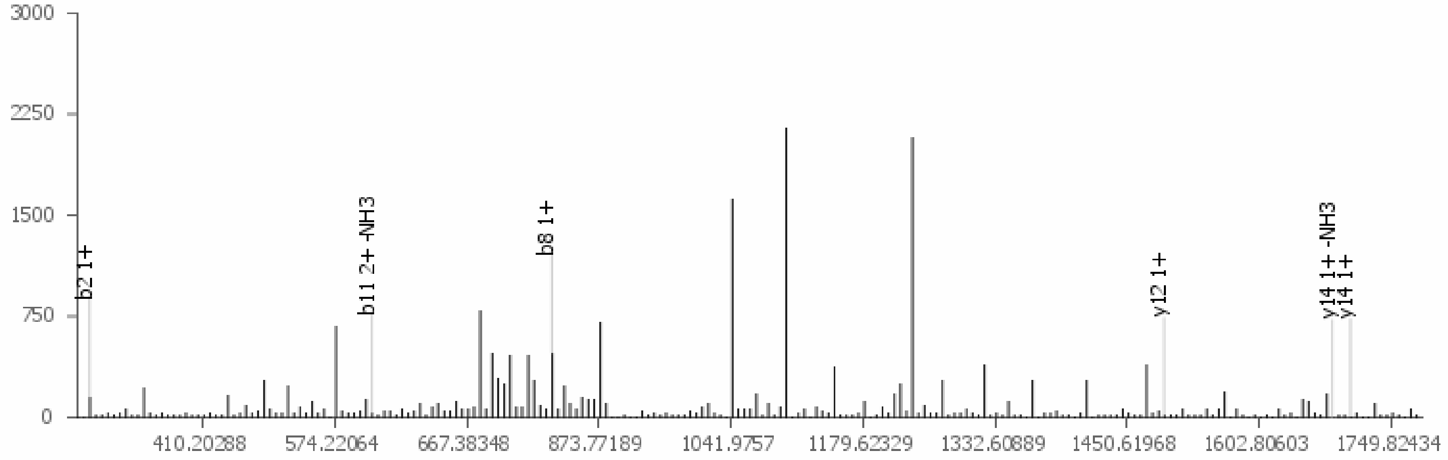

AT5G10370.1 - F(pY)FKGSVLK - 390.20033 - Charge:3

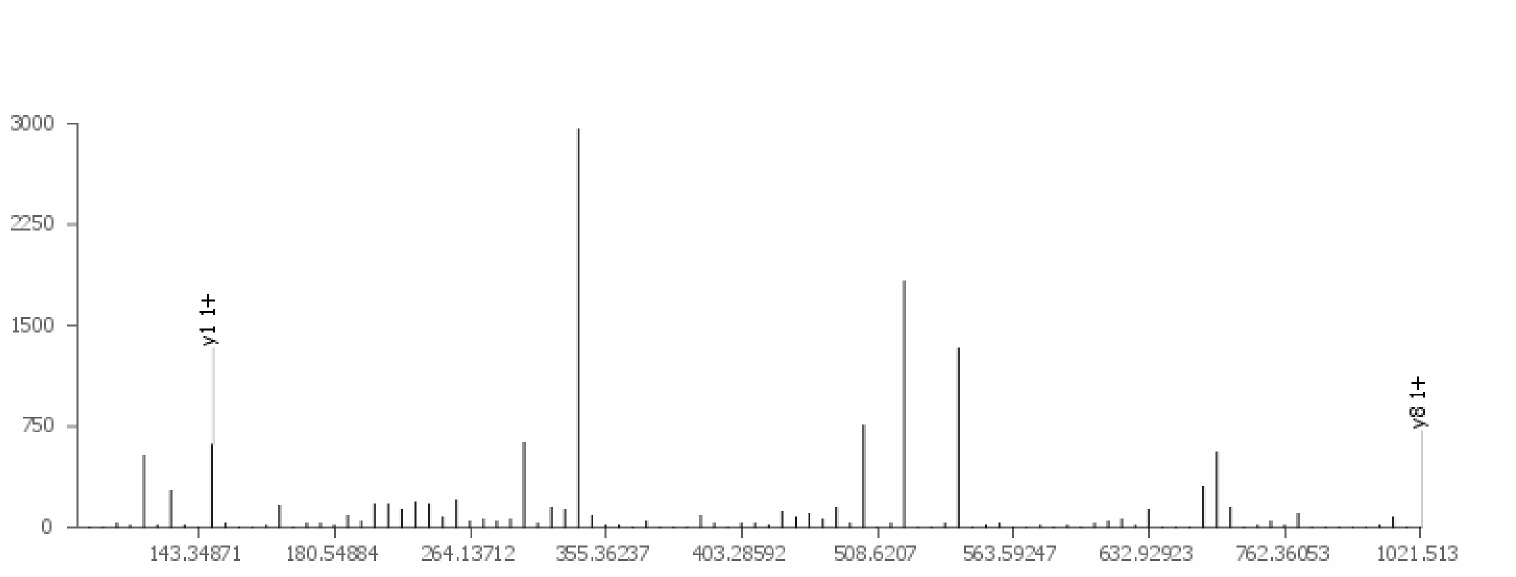

AT5G51660.1 - LLSRAEFHVGAHV(pS)K - 865.932031 - Charge:2

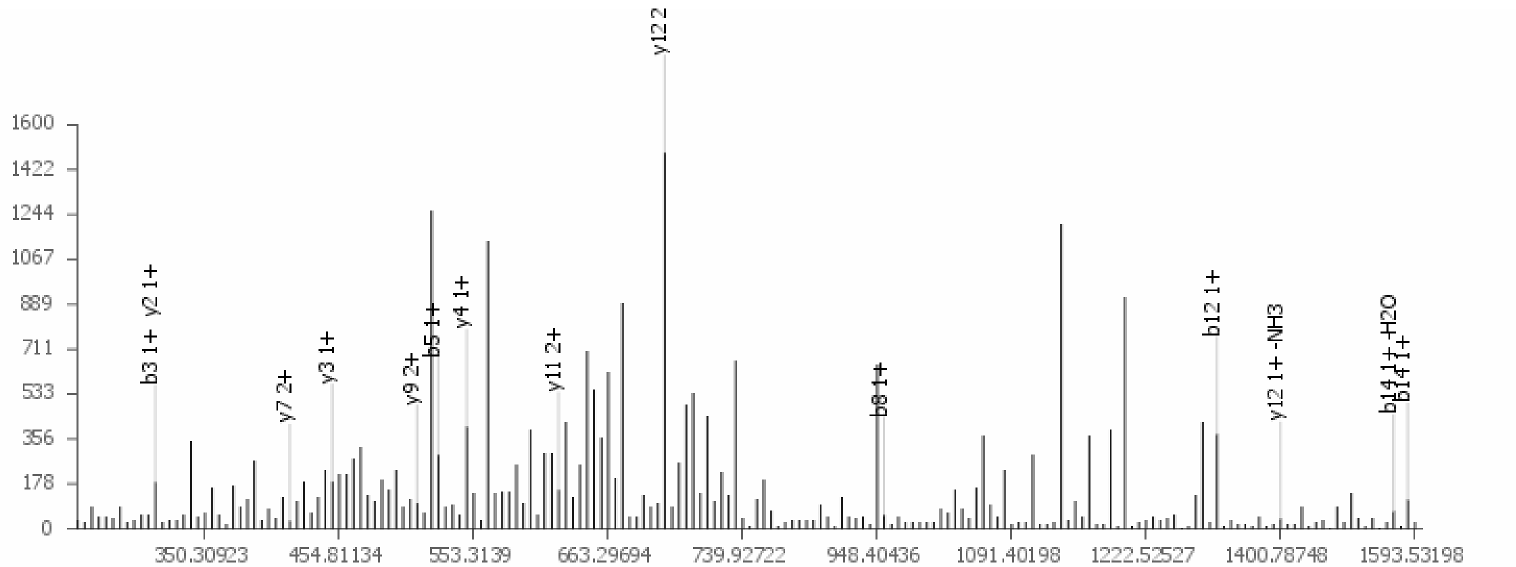

AT1G69240.1 - TMEDD(pY)AVPVSLQDAMIK - 1053.457813 - Charge:2

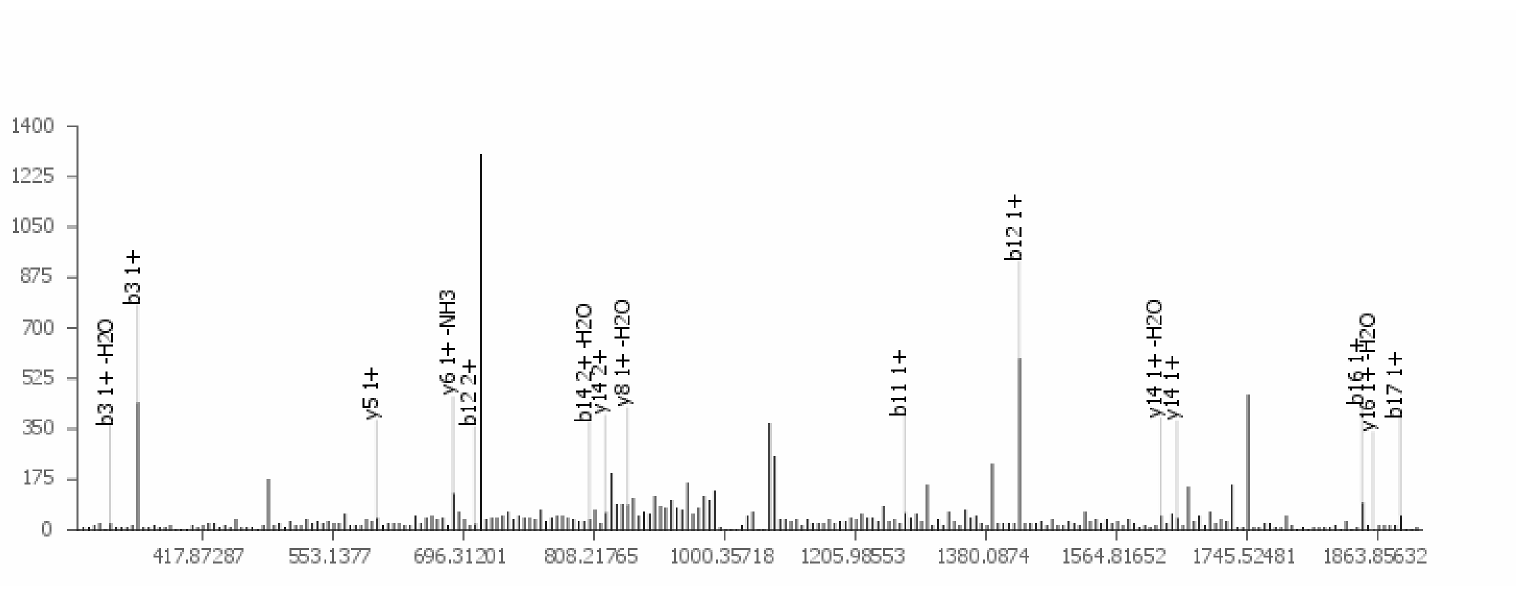

AT3G59220.1 - YKELS(pS)LDIPR - 700.849405 - Charge:2

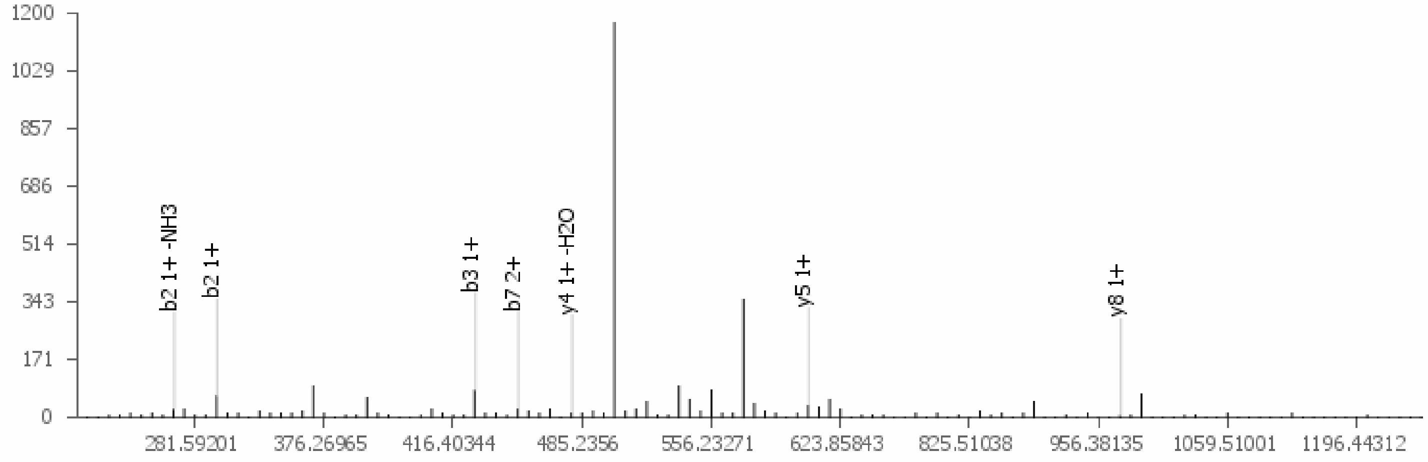

AT3G10240.1 - SG(pS)SSIPLDLV(pS)EILLR - 973.460523 - Charge:2

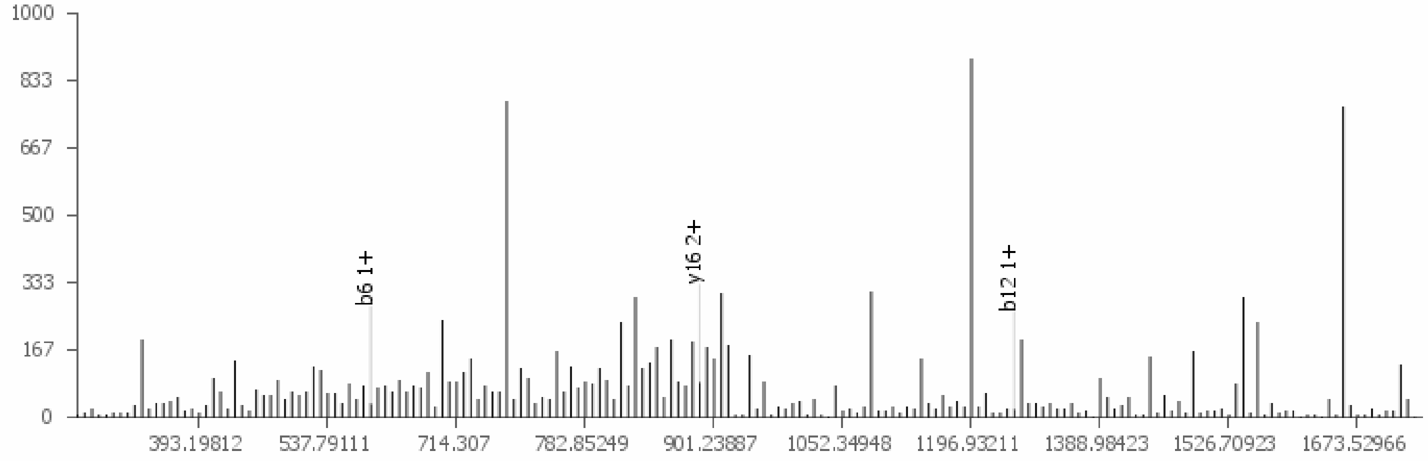

AT4G37590.1 - GNV(pS)ADVIGEALHAYAIK - 954.469542 - Charge:2

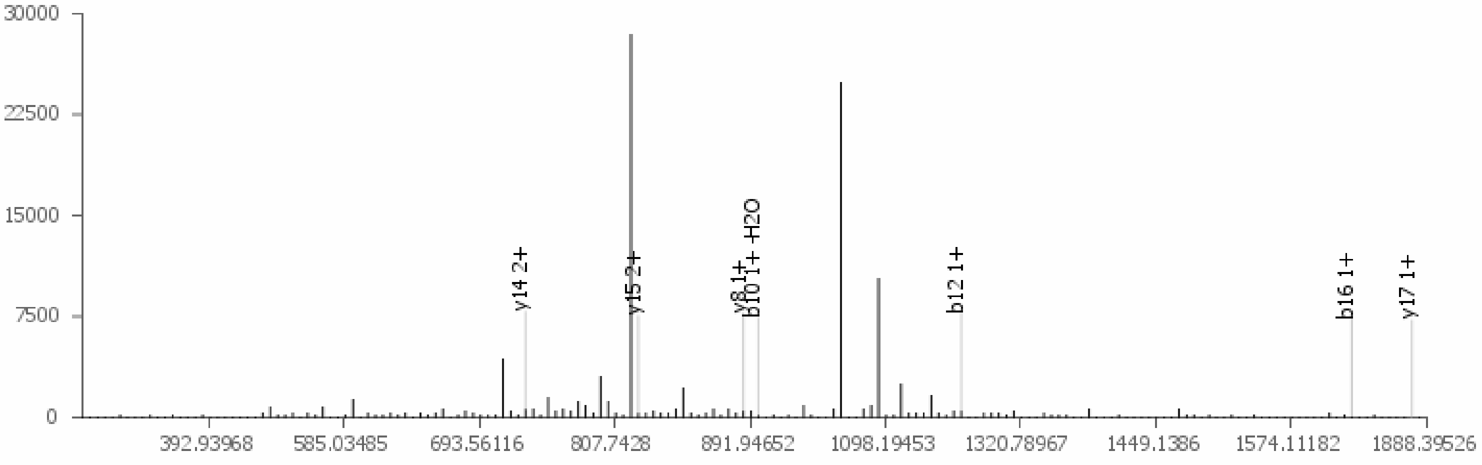

AT4G29420.1 - NAWL(pS)VVGLTEMPNLR - 940.453809 - Charge:2

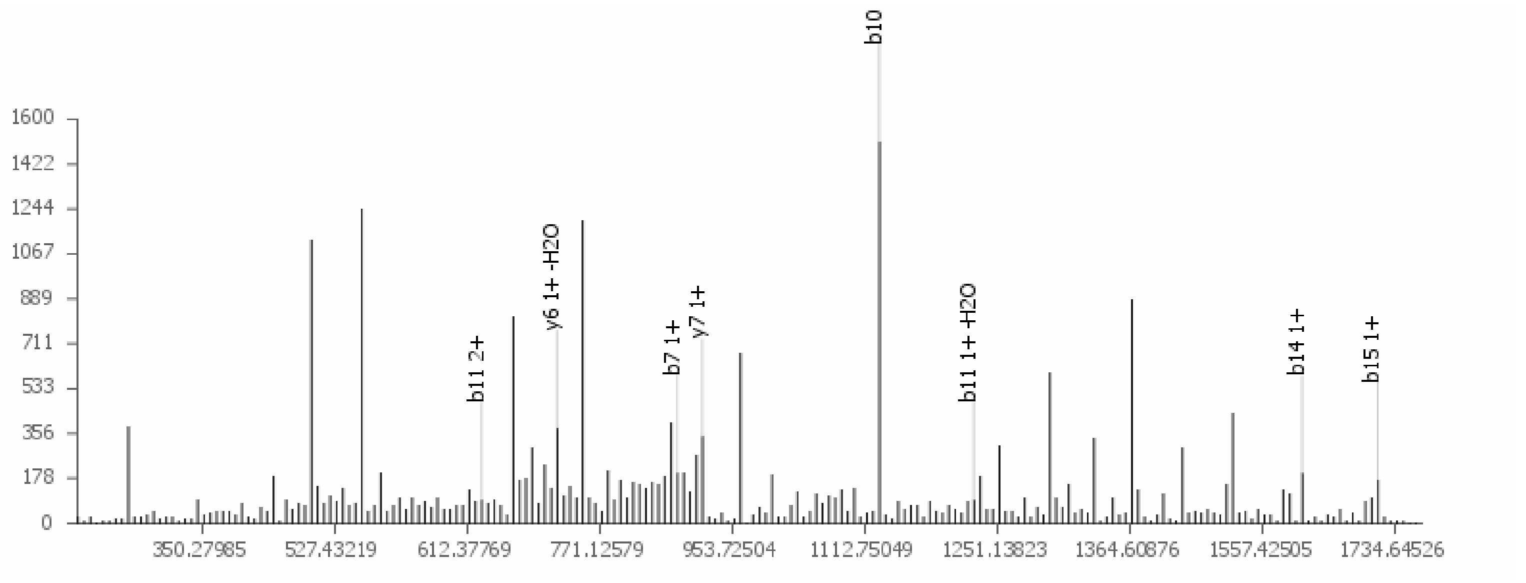

AT4G39050.1 - MLAGEIAF(s)(t)(s)TLK - 774.865982 - Charge:2

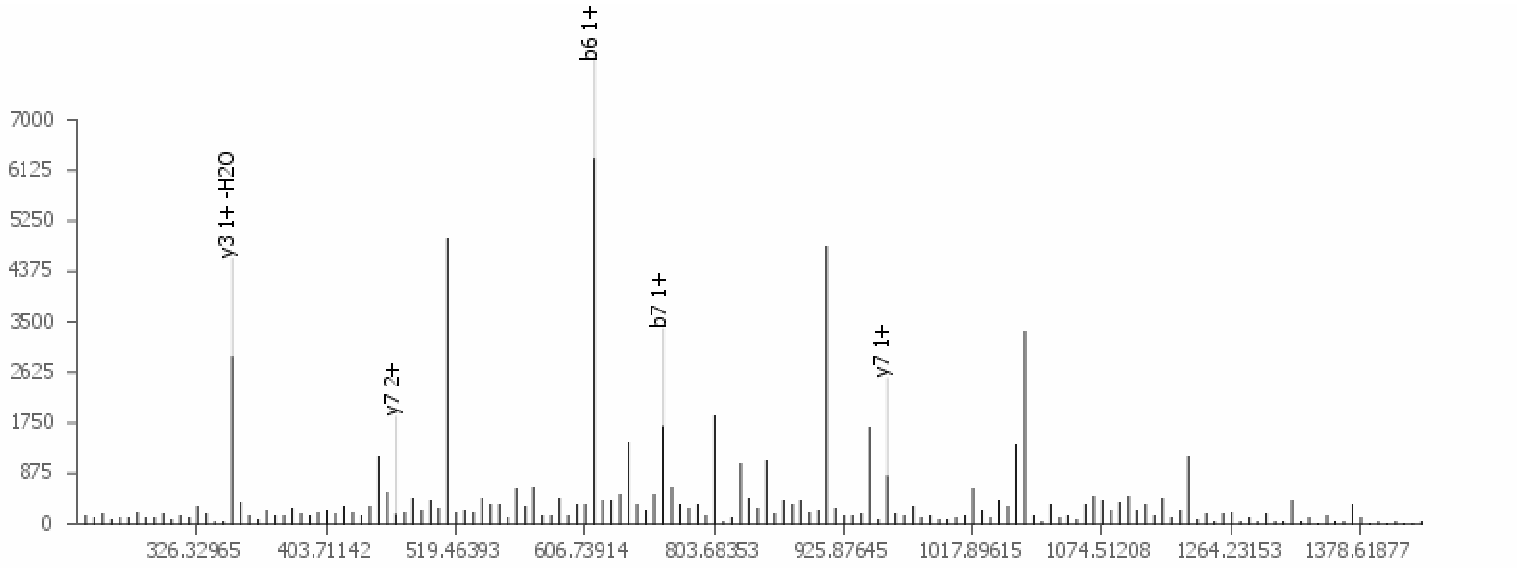

AT5G67610.1 - GD(pY)RLLR - 486.740256 - Charge:2

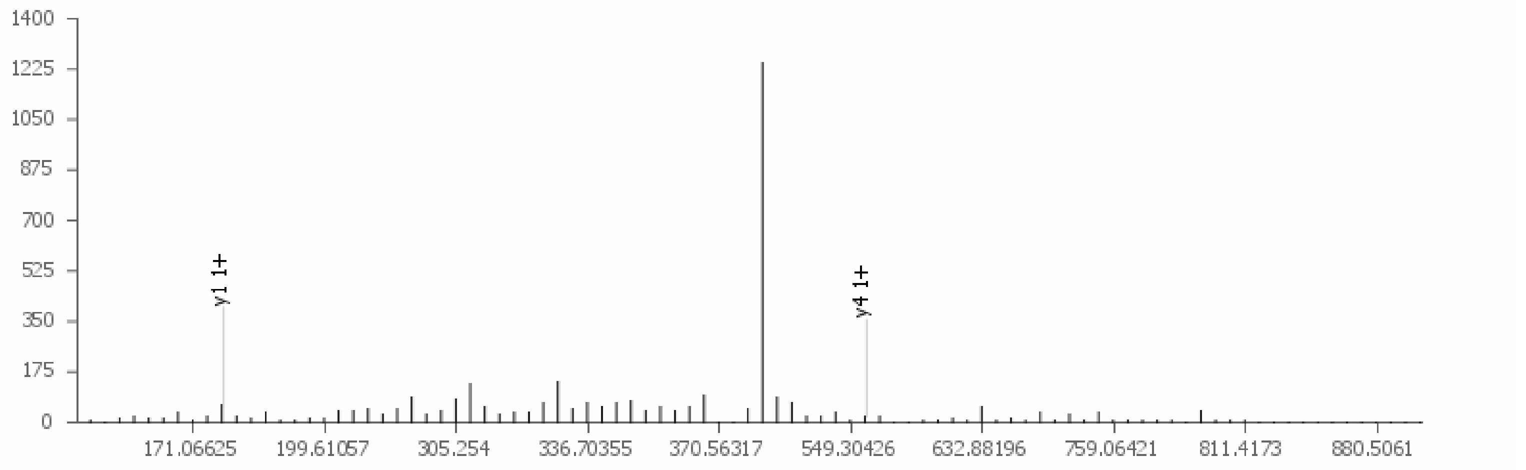

AT2G46020.1 - NMLSS(pS)NLIVQPGGPGGERK - 1061.01958 - Charge:2

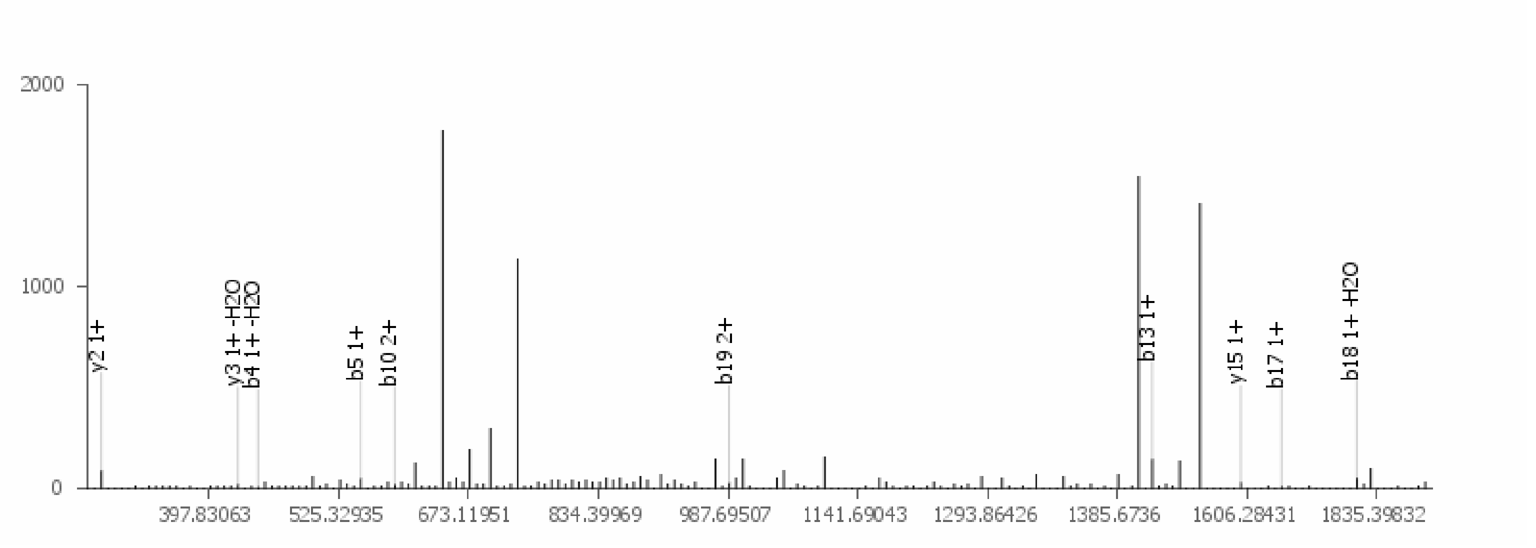

AT1G72920.1 - TASKL(pY)VLTR - 616.327145 - Charge:2

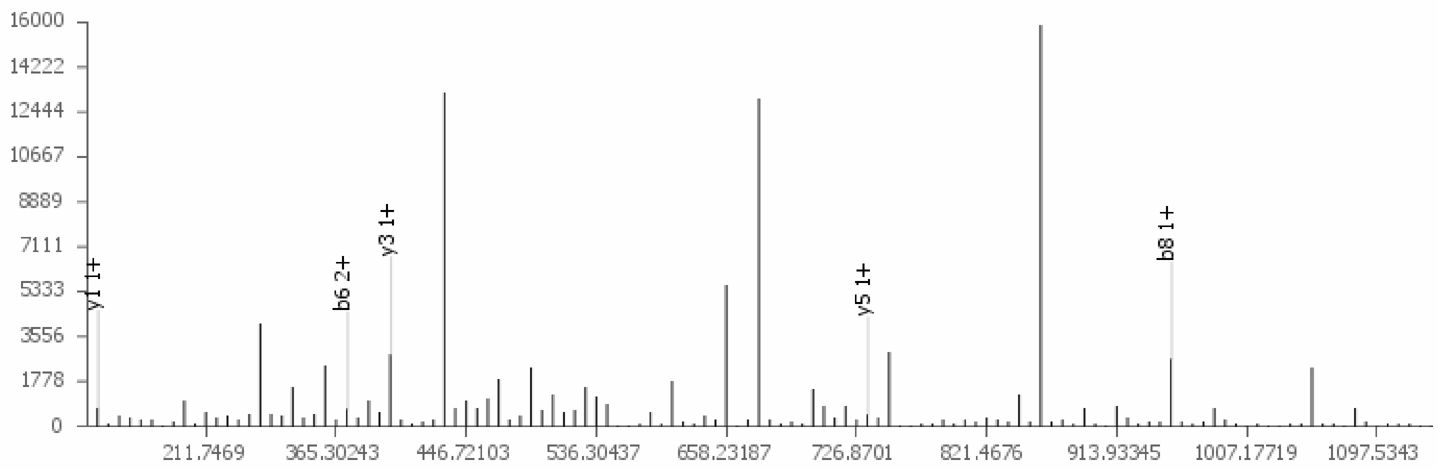

AT4G24030.1 - (pT)V(oxM)GFVVVVVVVVMR - 865.469002 - Charge:2

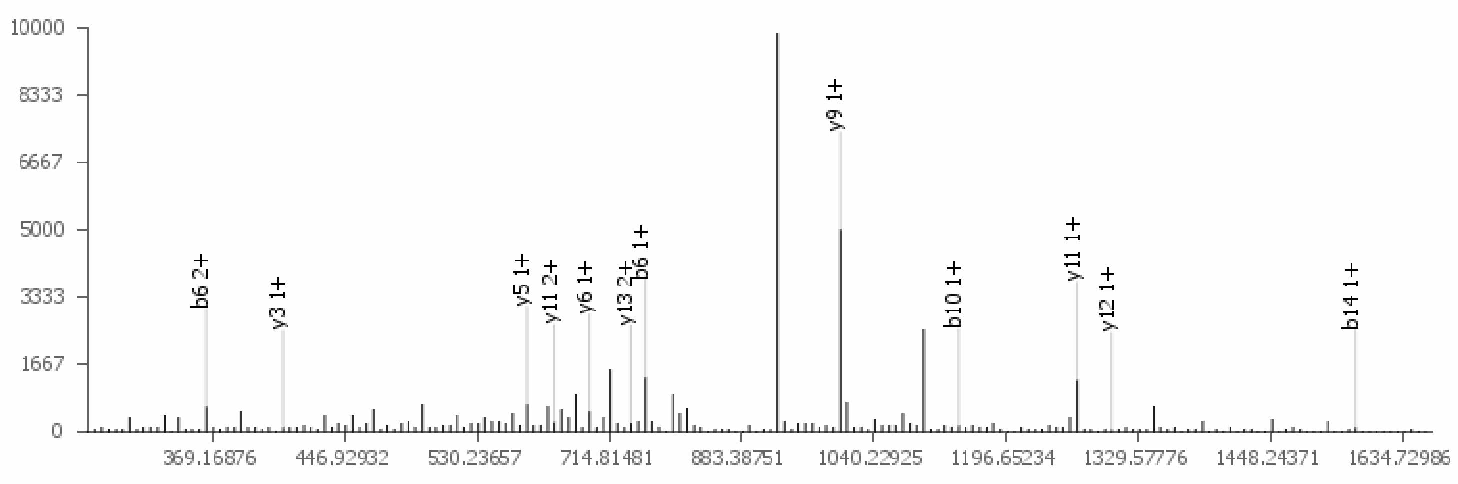

AT5G63840.1 - IILLGHS(pS)GPK - 601.321653 - Charge:2

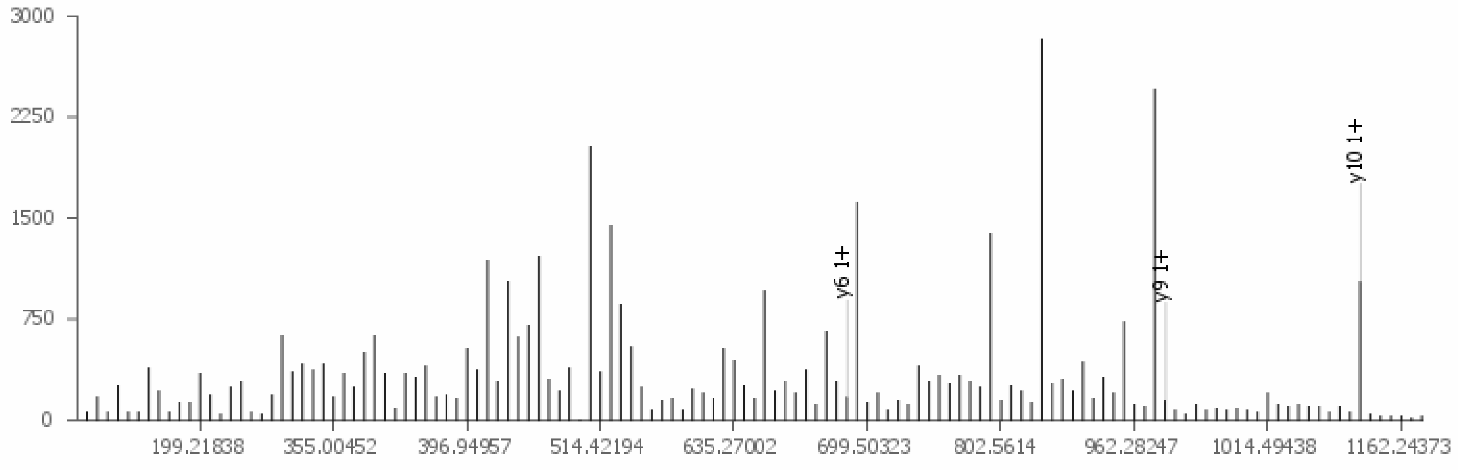

AT4G13510.1 - ISSEDEMAG(m)D(m)(pT)R - 826.807377 - Charge:2

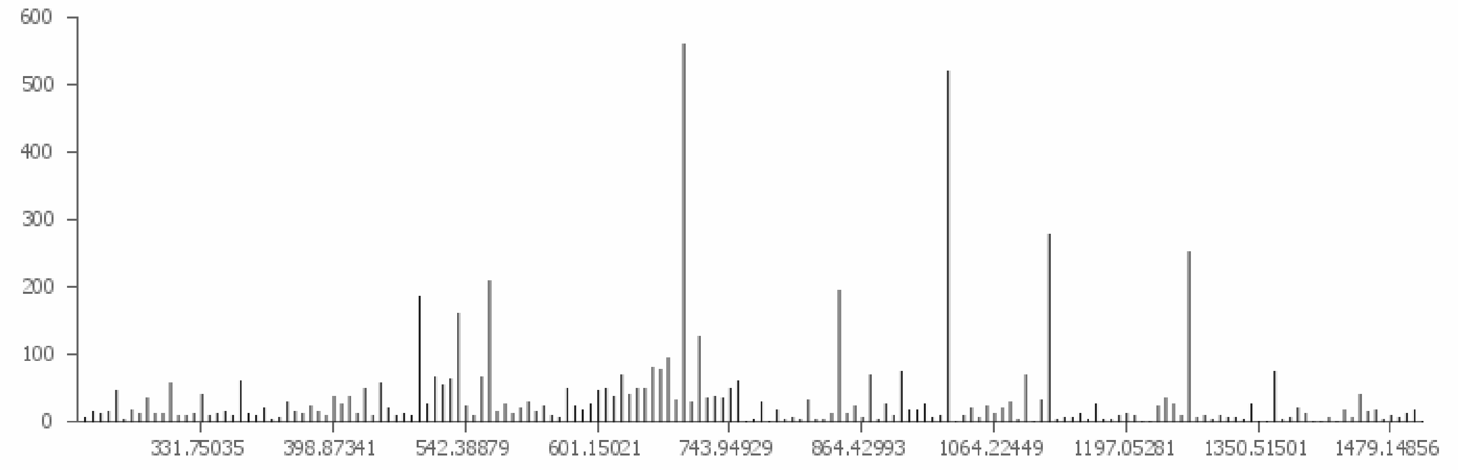

AT5G45060.1 - DVNL(t)(s)LK - 485.239651 - Charge:2

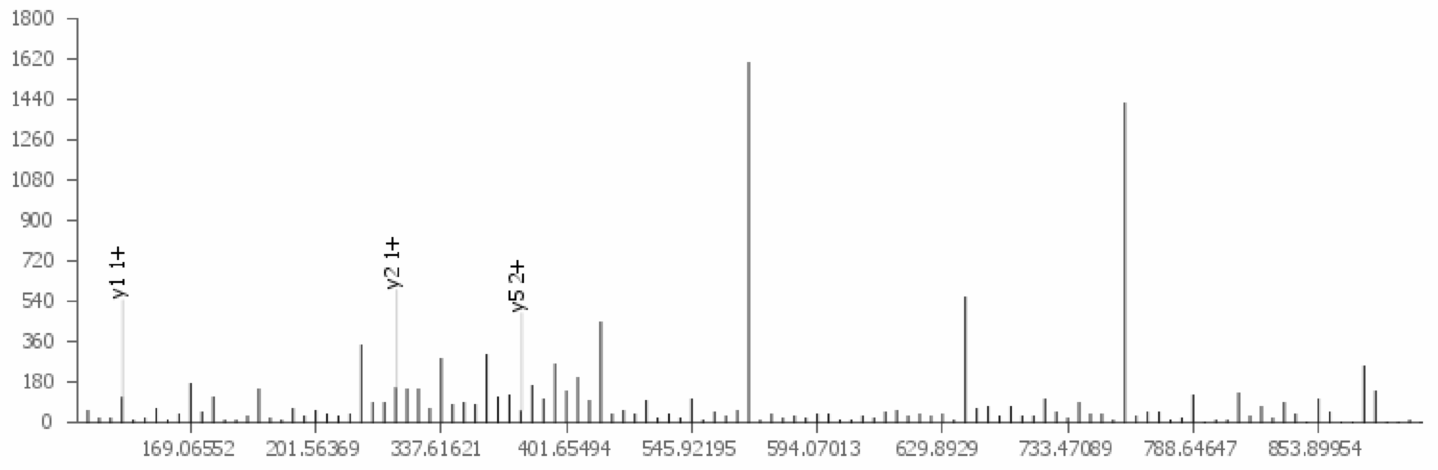

AT1G16020.1 - G(pT)VQ(oxM)LTLAR - 593.290903 - Charge:2

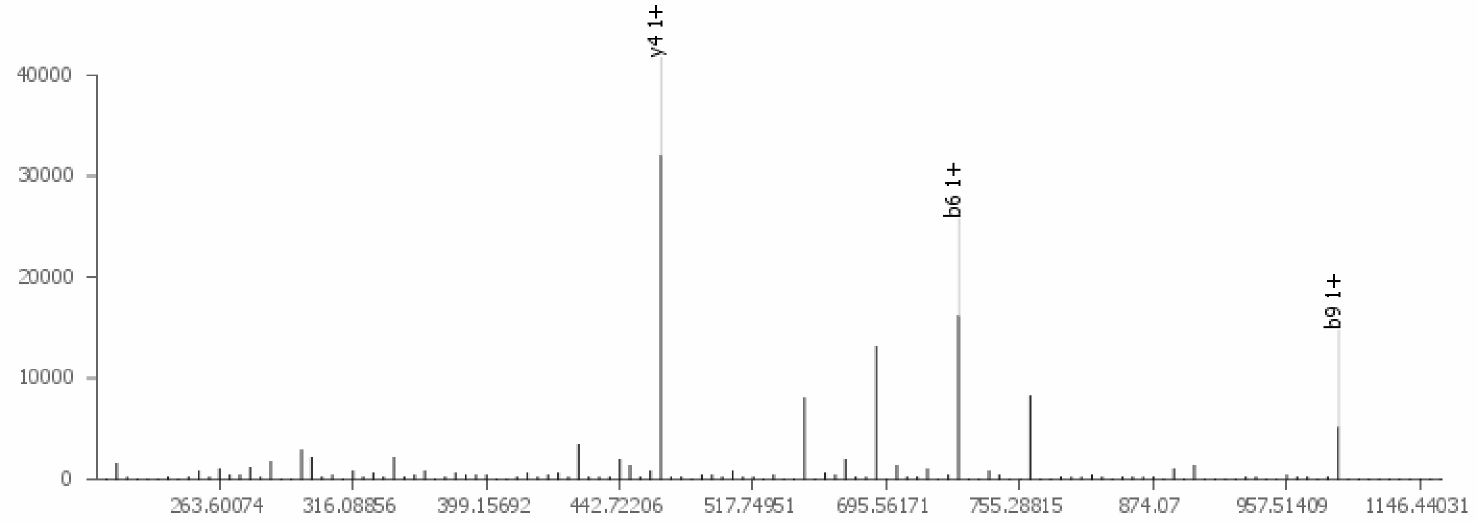

AT1G49250.1 - LGMGKGS(pY)IIK - 623.820909 - Charge:2

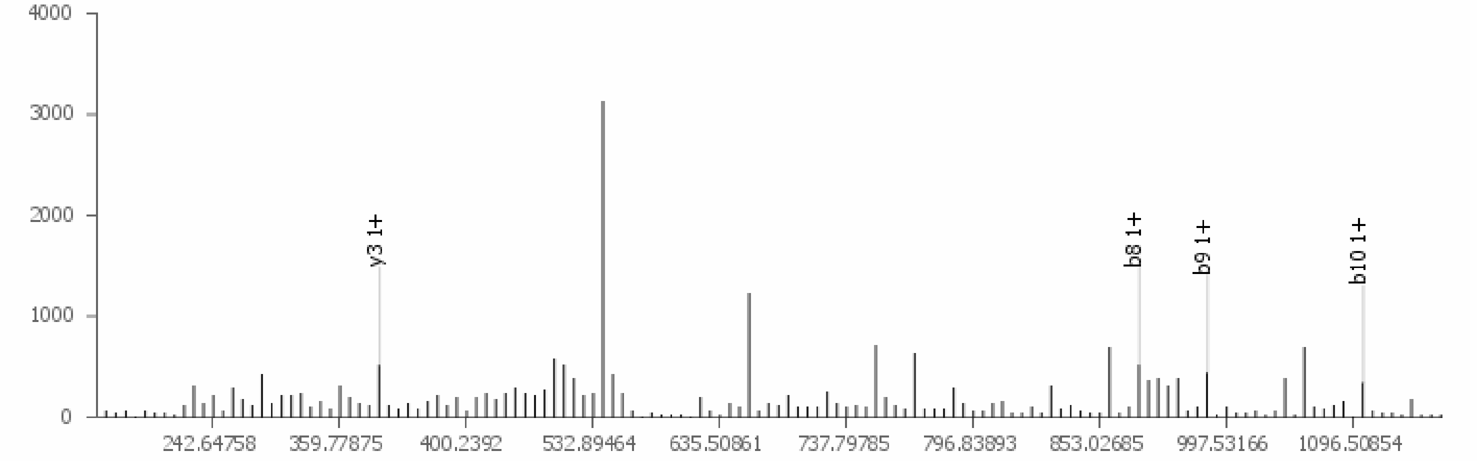

AT3G28770.1 - L(pS)DAMAVLQAKK - 677.847712 - Charge:2

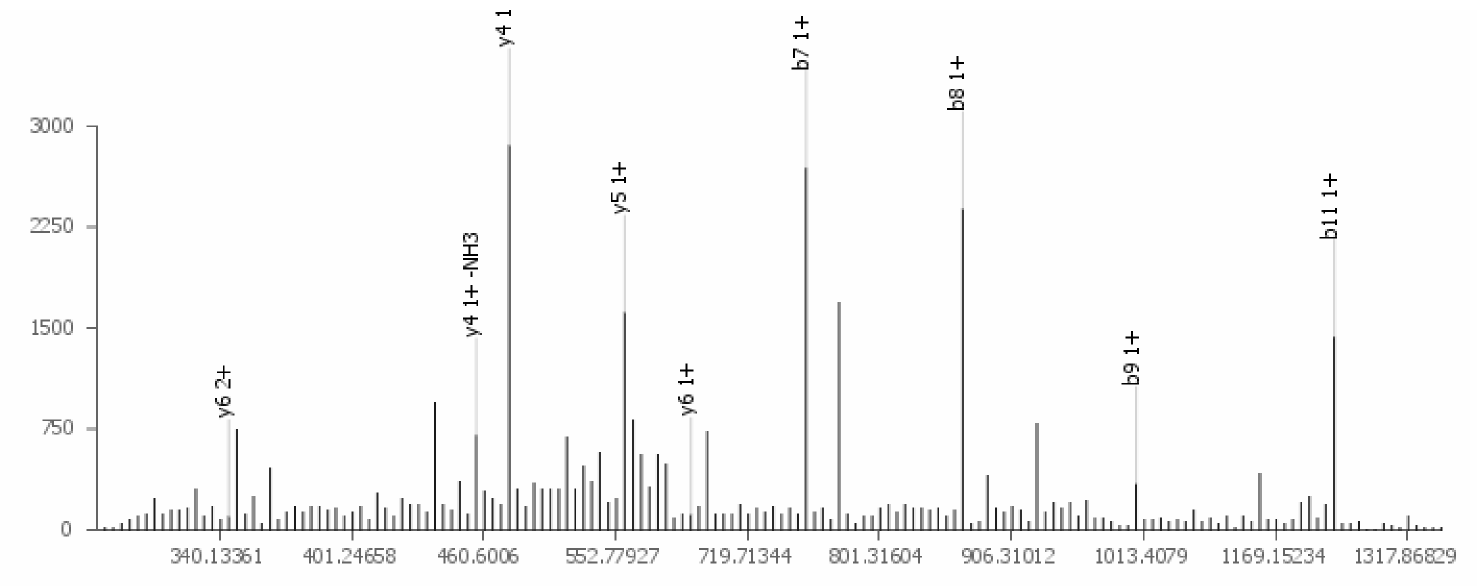

AT1G32400.1 - AANTPAEYD(pS)DDEYLAPR - 1039.41406 - Charge:2

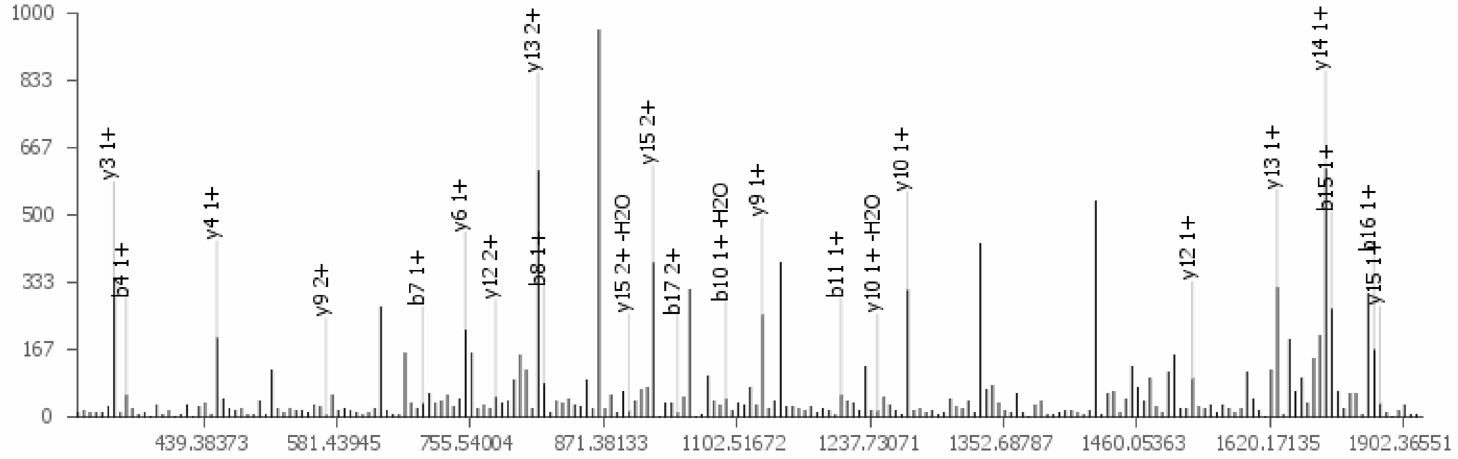

AT1G53070.1 - SR(pS)GAIAL(pT)R - 596.269012 - Charge:2

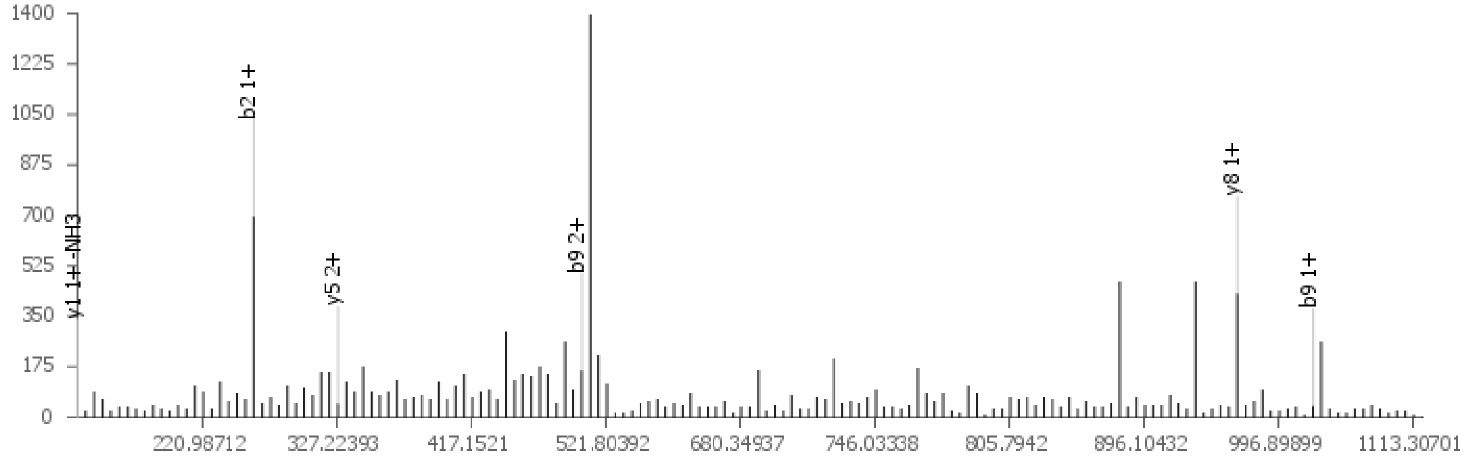

AT2G43200.1 - VKAI(pT)NQ(oxM)R - 578.778109 - Charge:2

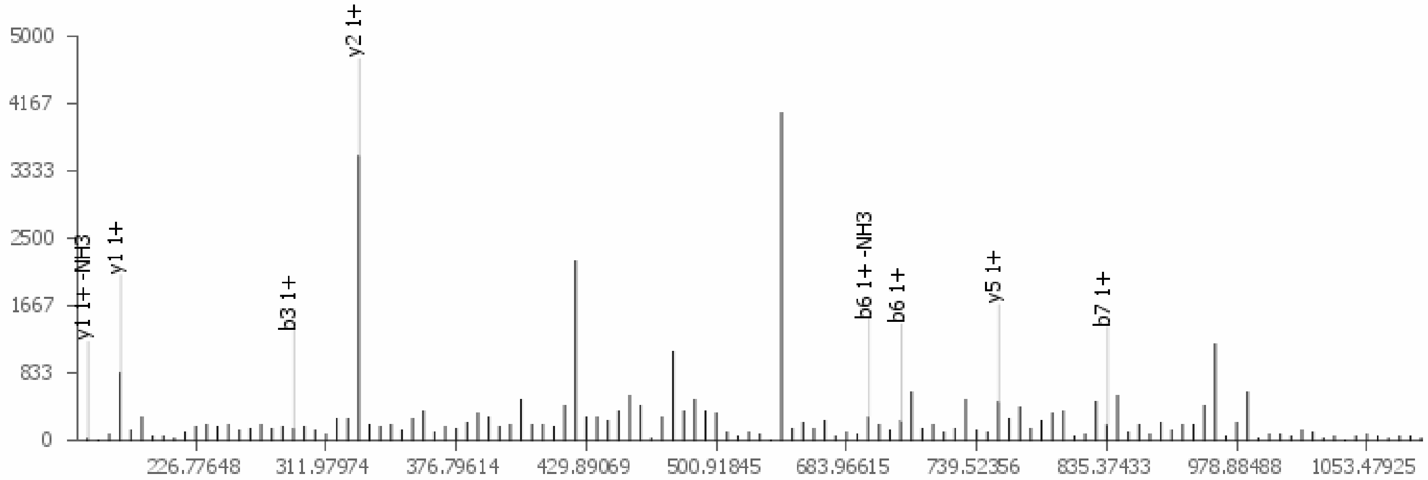

AT4G17920.1 - IINSFPTFP(pY)SSVKDLR - 1032.521833 - Charge:2

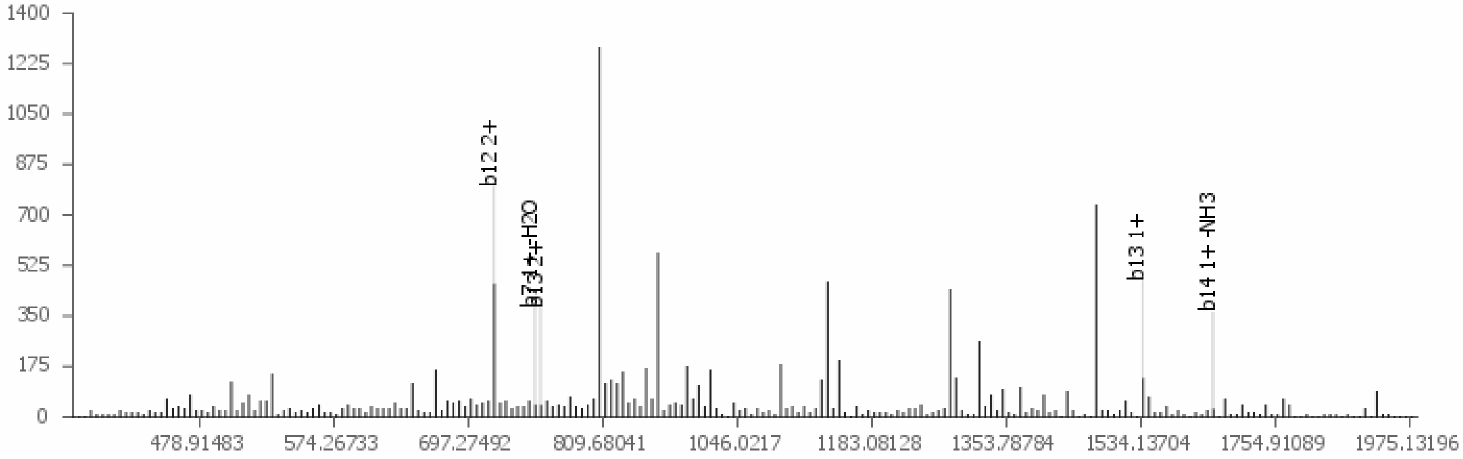

AT1G77990.1 - LGFLVDFL(pS)HAALVGF(oxM)AGAAIVIGLQQLK - 1065.91636 - Charge:3

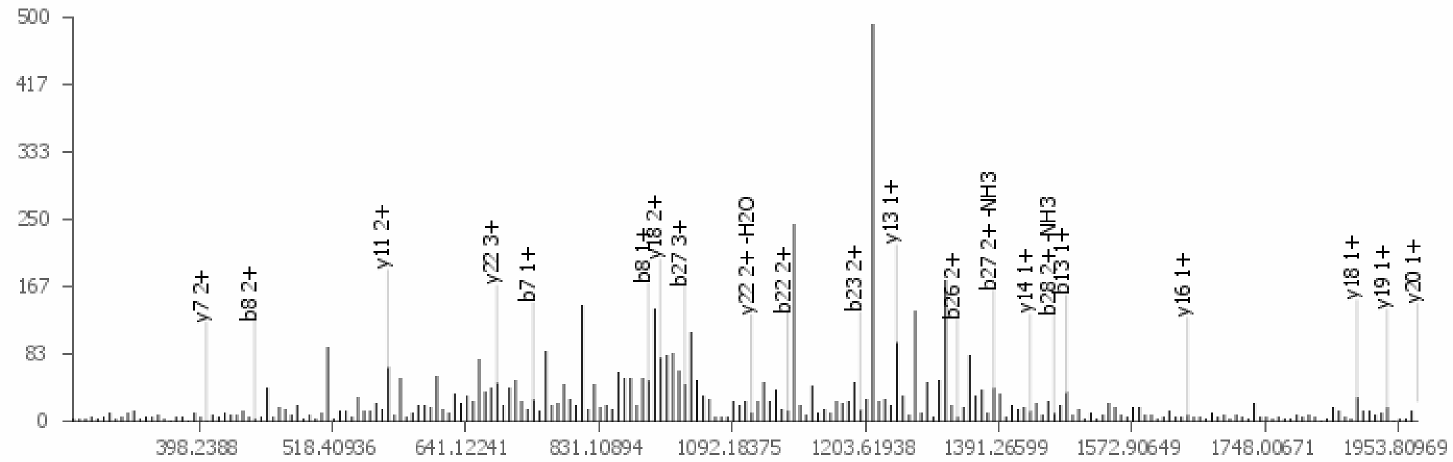

AT3G06350.1 - VVIANR(pT)YER - 650.830584 - Charge:2

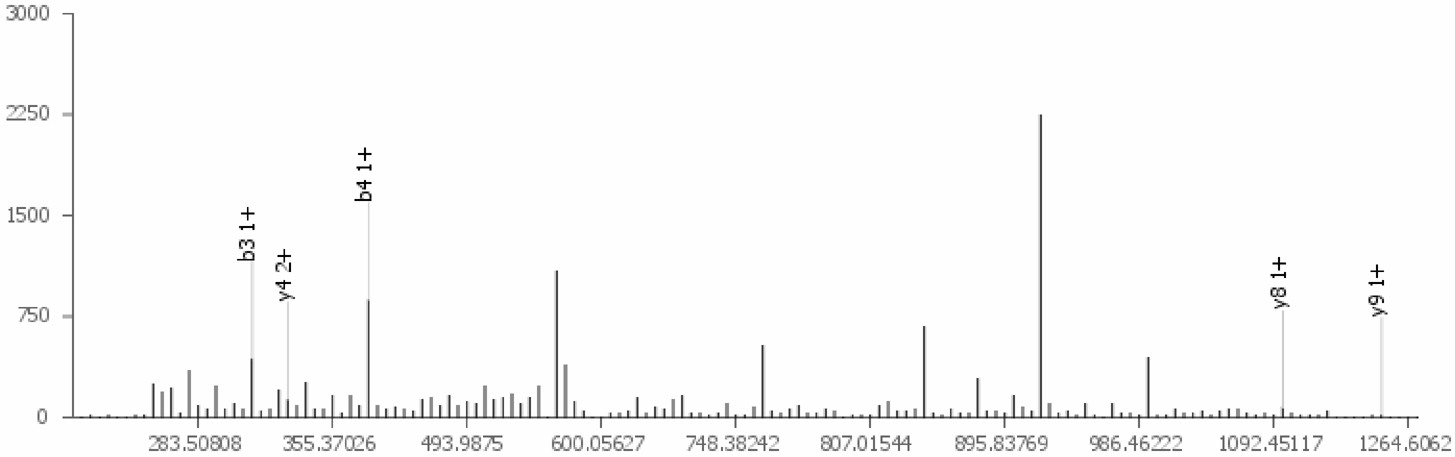

AT3G04220.1 - K(pY)DVFPSFR - 619.782894 - Charge:2

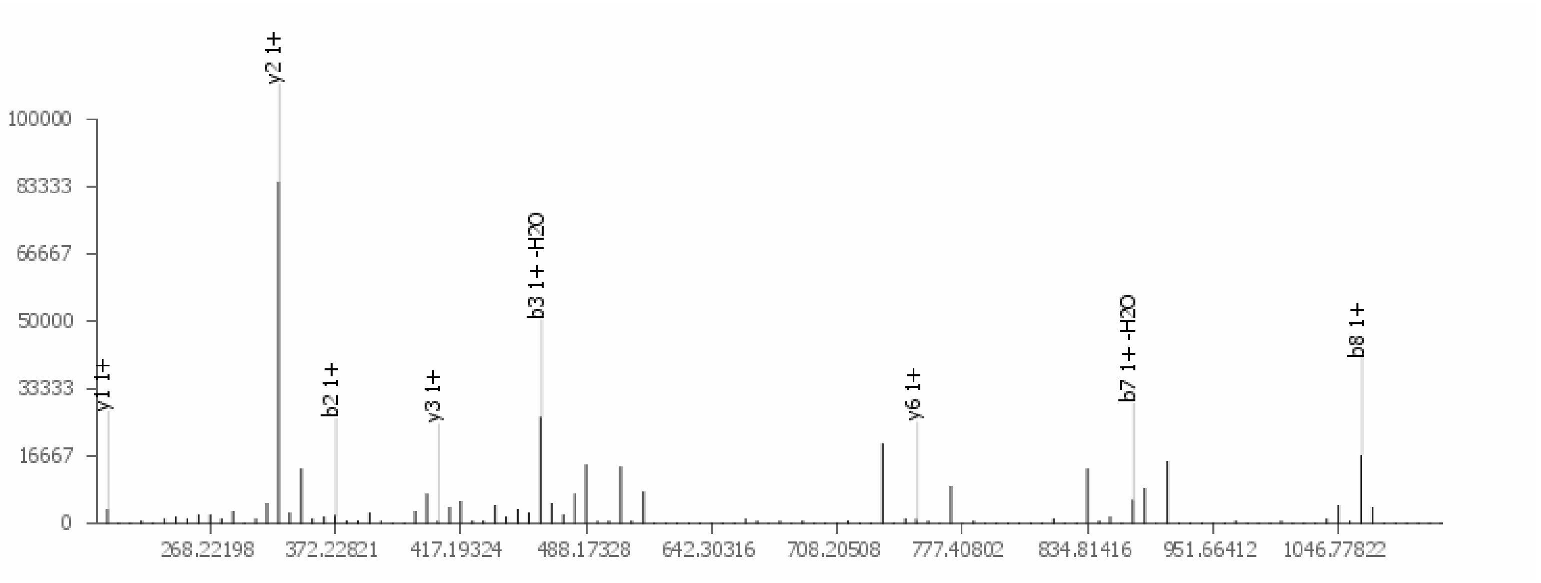

AT4G36610.1 - FLASIH(pS)EDKIK - 734.371657 - Charge:2

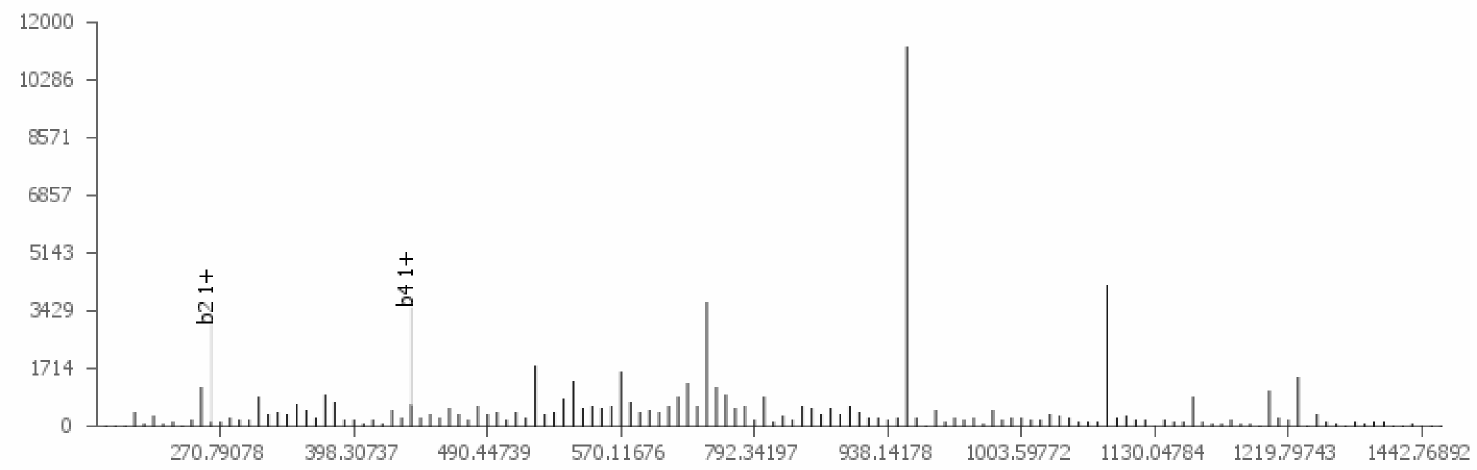

AT3G20980.1 - DITL(oxM)AKGIGDIIIM(pT)K - 964.999938 - Charge:2

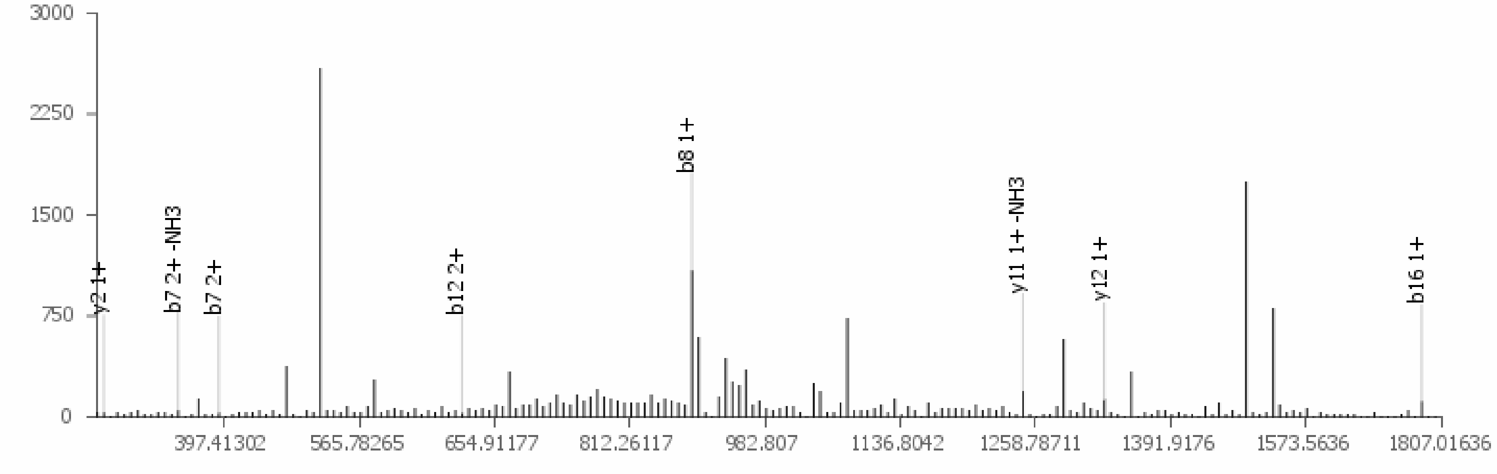

AT4G17720.1 - YVL(t)GA(t)WVTGAFNK - 854.418559 - Charge:2

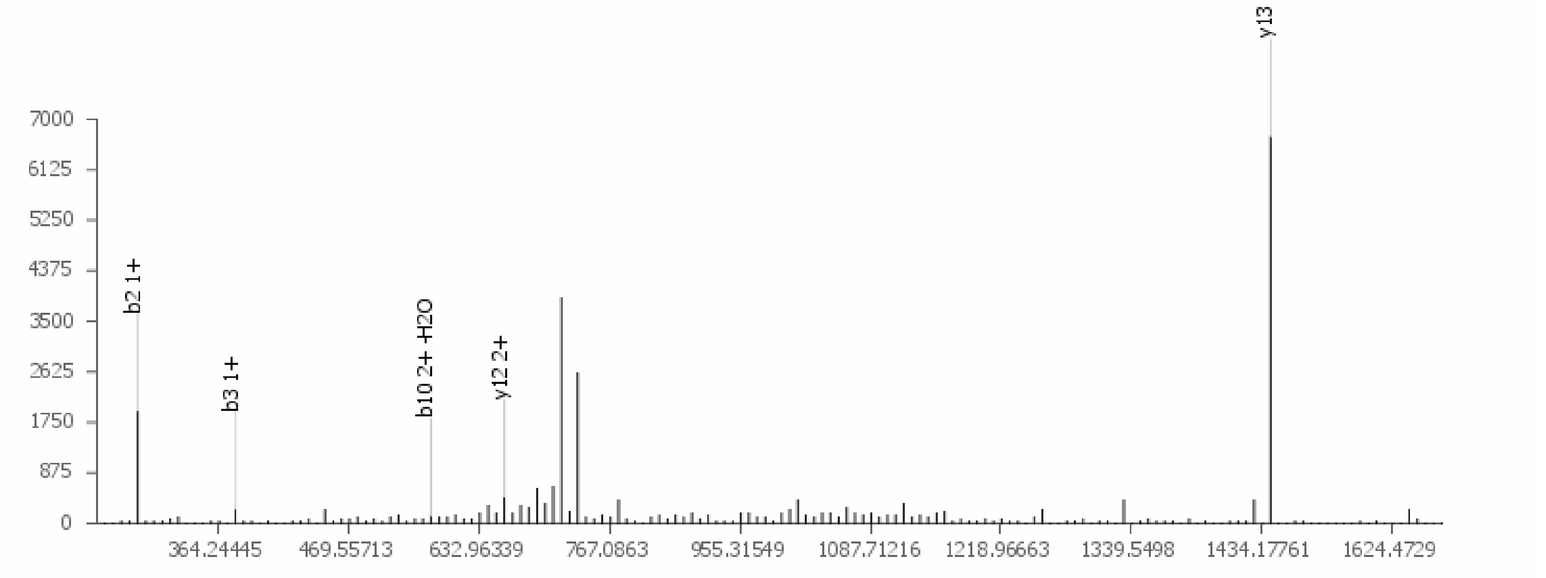

AT4G29810.1 - AIPD(pS)YLSAIFR - 716.853218 - Charge:2

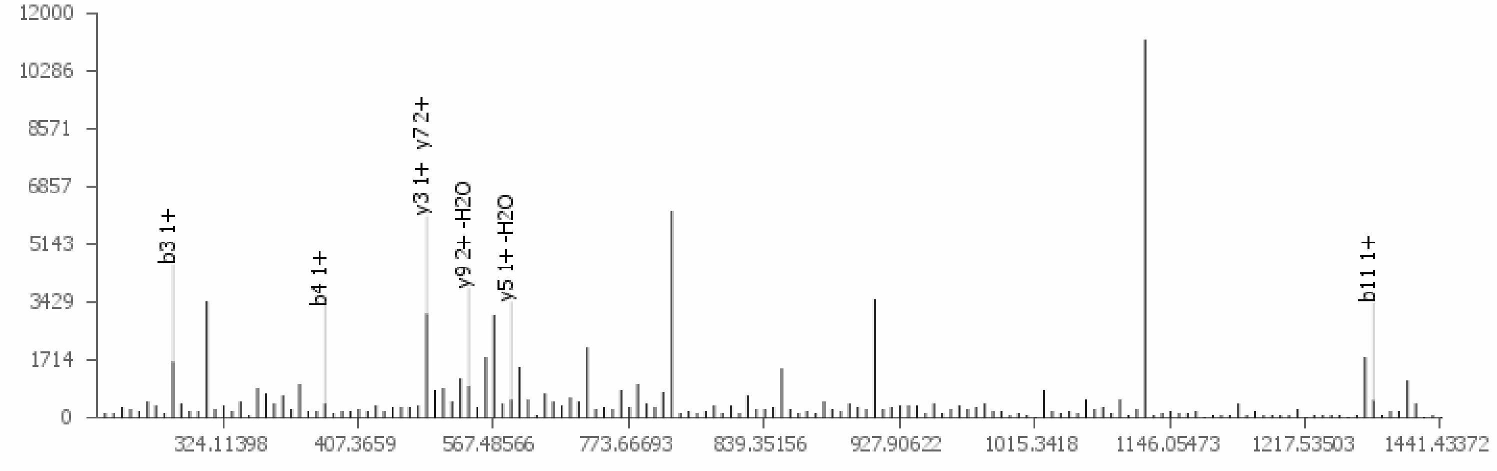

AT4G11540.1 - STIDYYDI(pS)YEVVVNI - 986.941783 - Charge:2

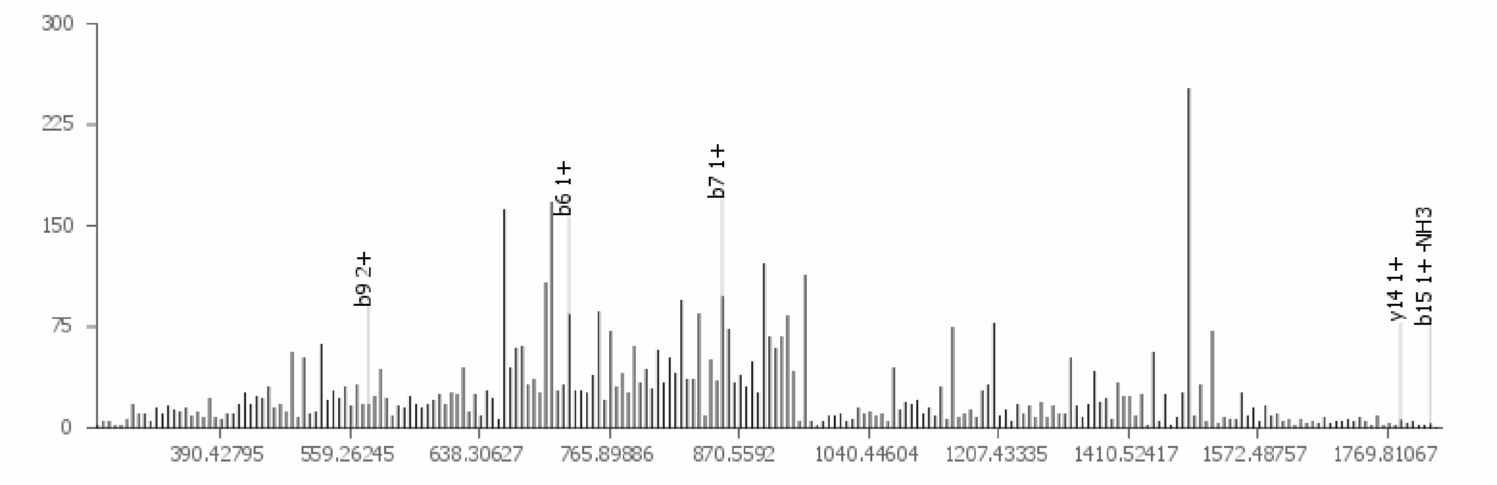

AT1G74070.1 - DP(pS)KPPPKTK - 587.798947 - Charge:2

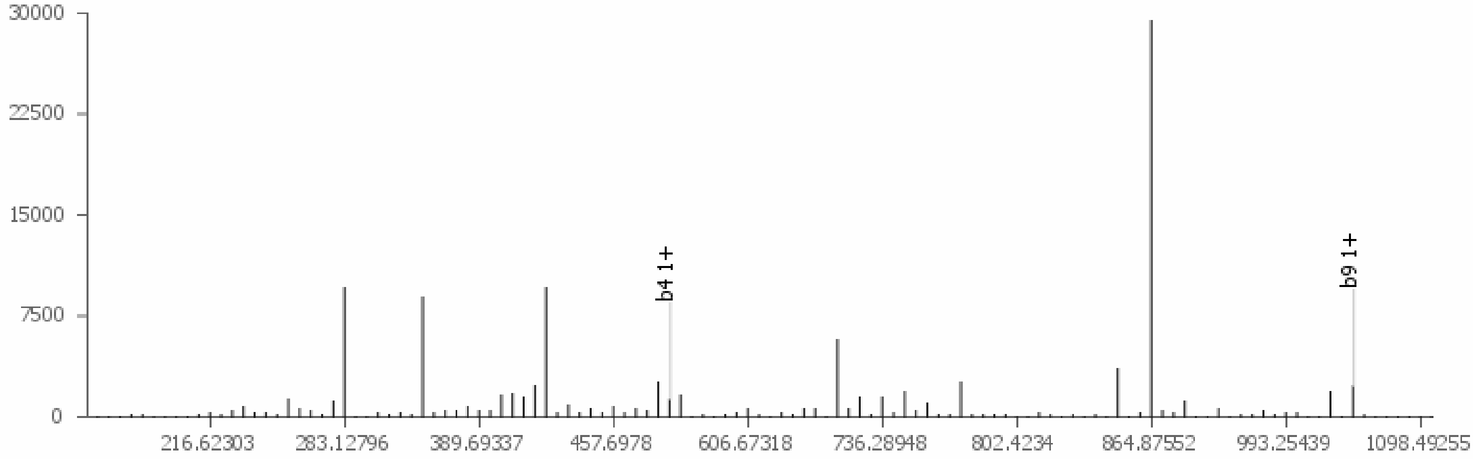

AT1G63840.1 - IFFYLL(pS)FLGLIR - 841.463017 - Charge:2

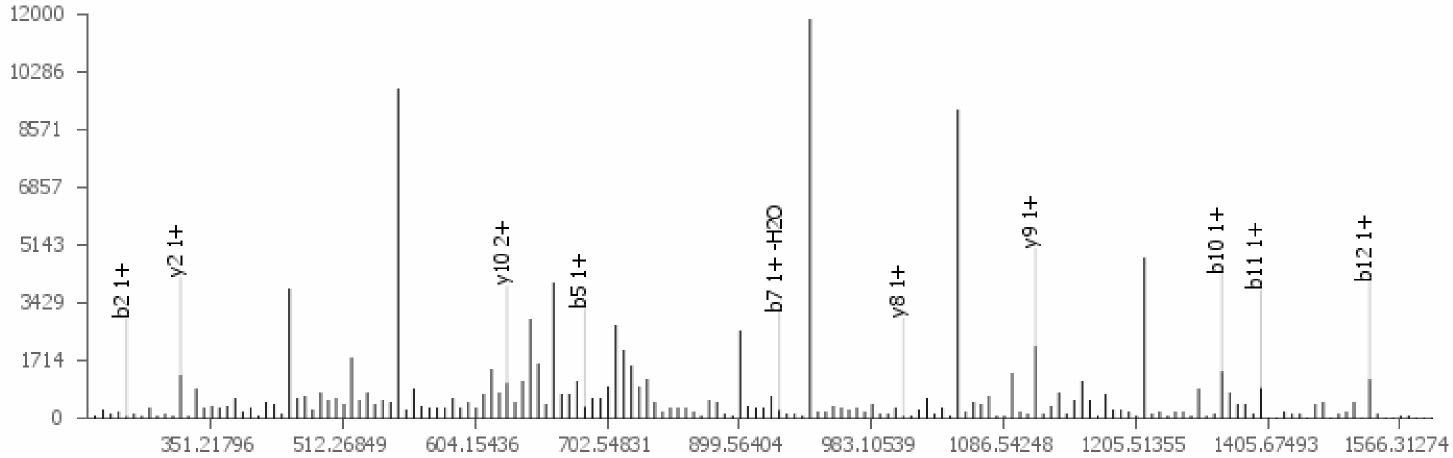

AT1G63870.1 - SLLLG(pT)(pT)LLPGR - 700.854753 - Charge:2

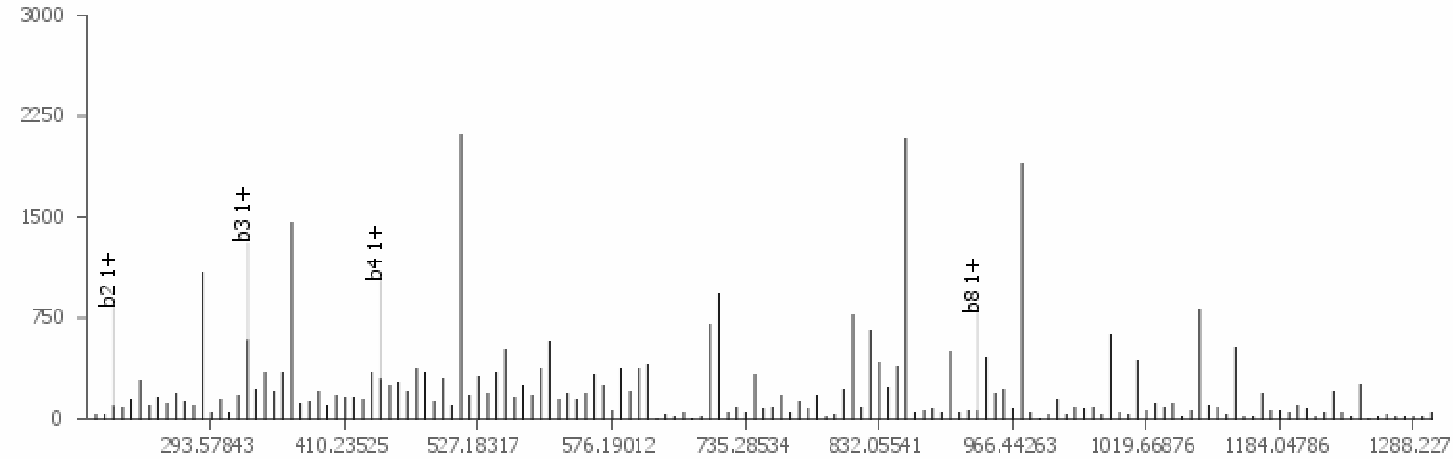

AT5G45580.1 - (pY)LLTLLEKAQK - 700.38449 - Charge:2

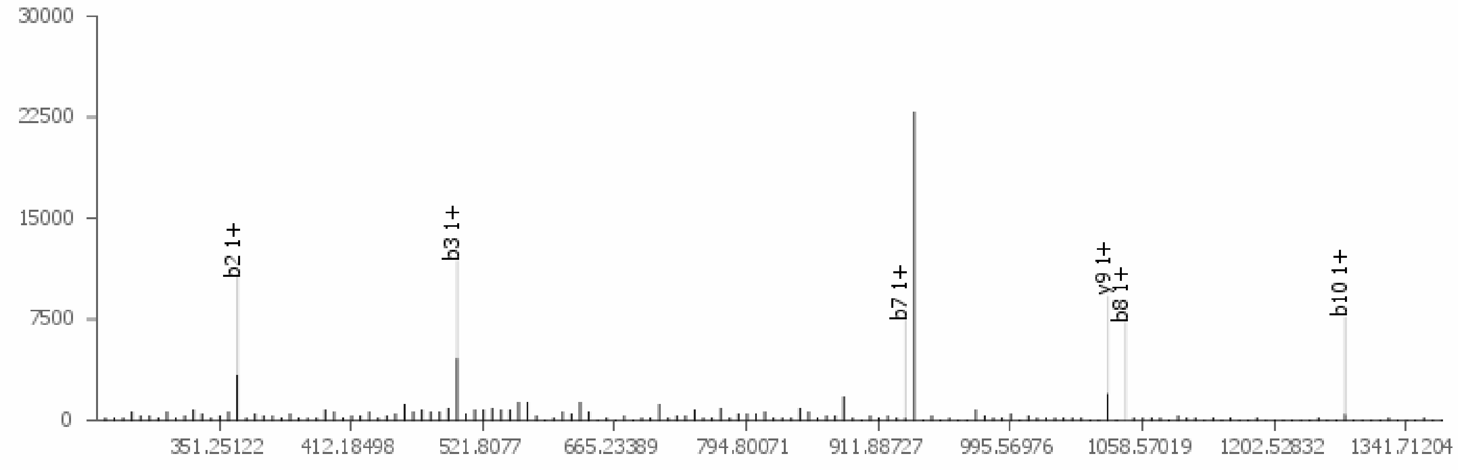

AT3G48990.1 - LLL(t)(s)KEGNAPAQEAASK - 954.468076 - Charge:2

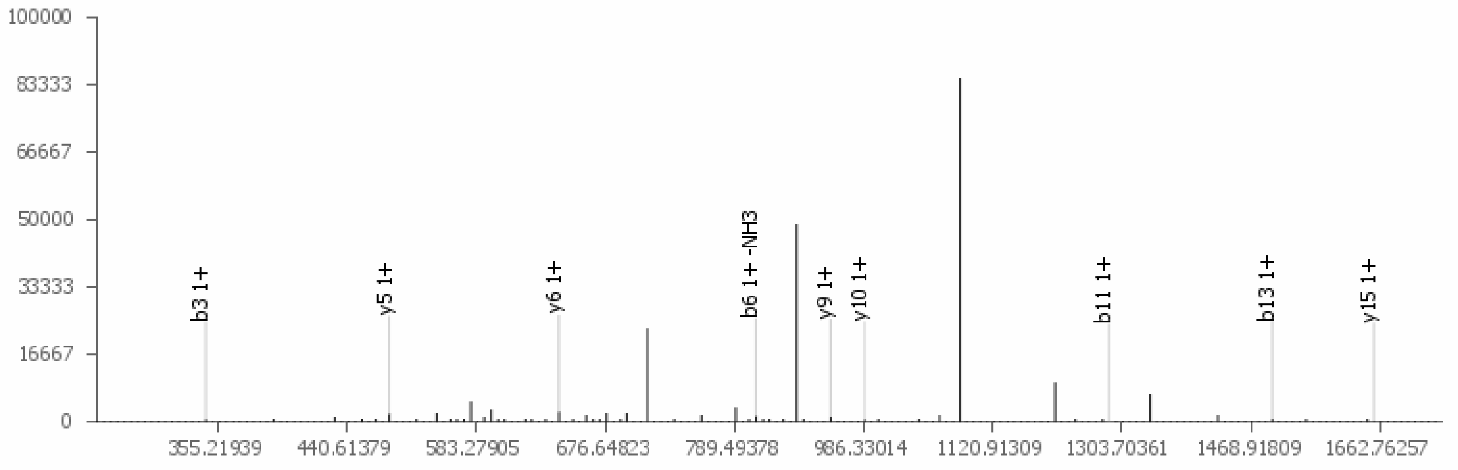

AT3G16720.1 - (oxM)L(pS)FTR(oxM)LSR - 677.308806 - Charge:2

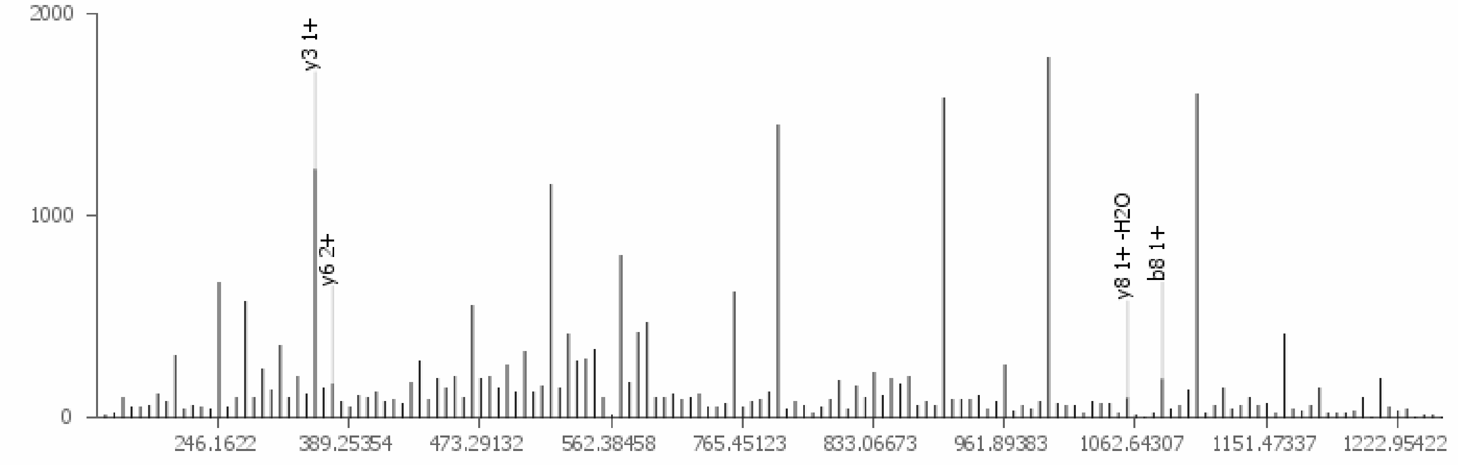

AT1G34420.1 - LEV(pS)DN(pS)LSGTIPEGIK - 959.927018 - Charge:2

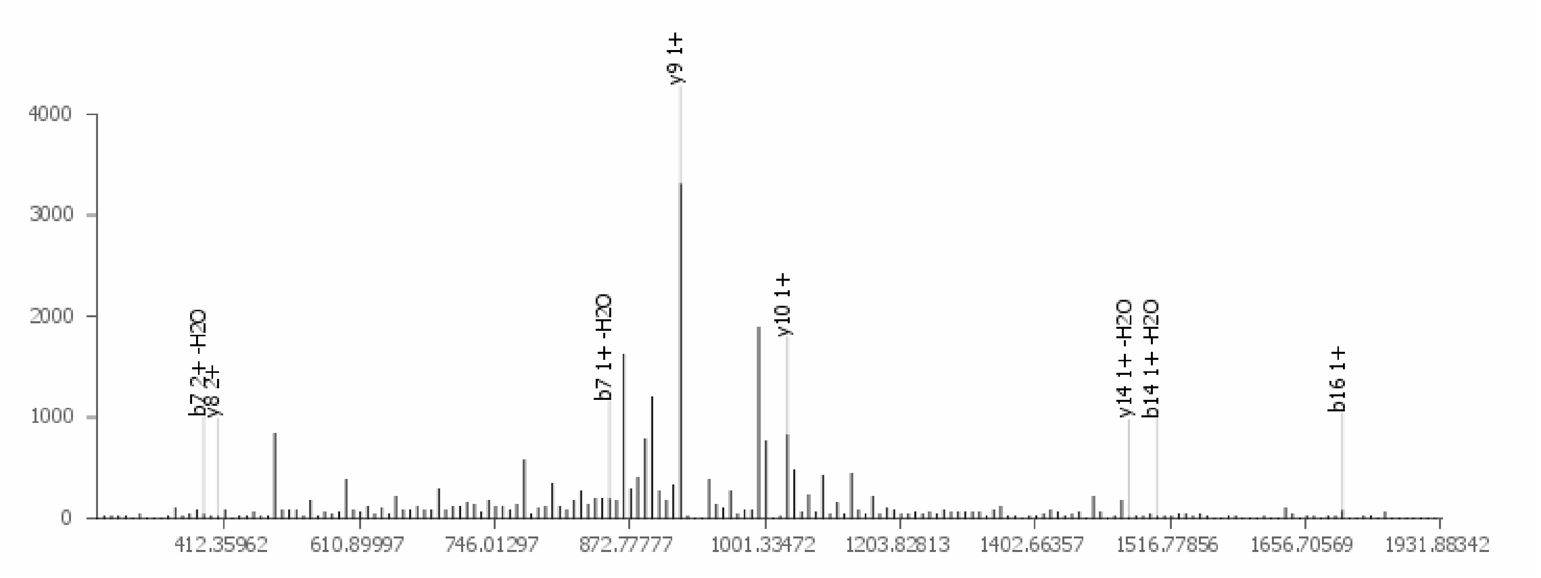

AT1G71060.1 - T(pY)DIILHHLIR(oxM)QR - 952.97674 - Charge:2

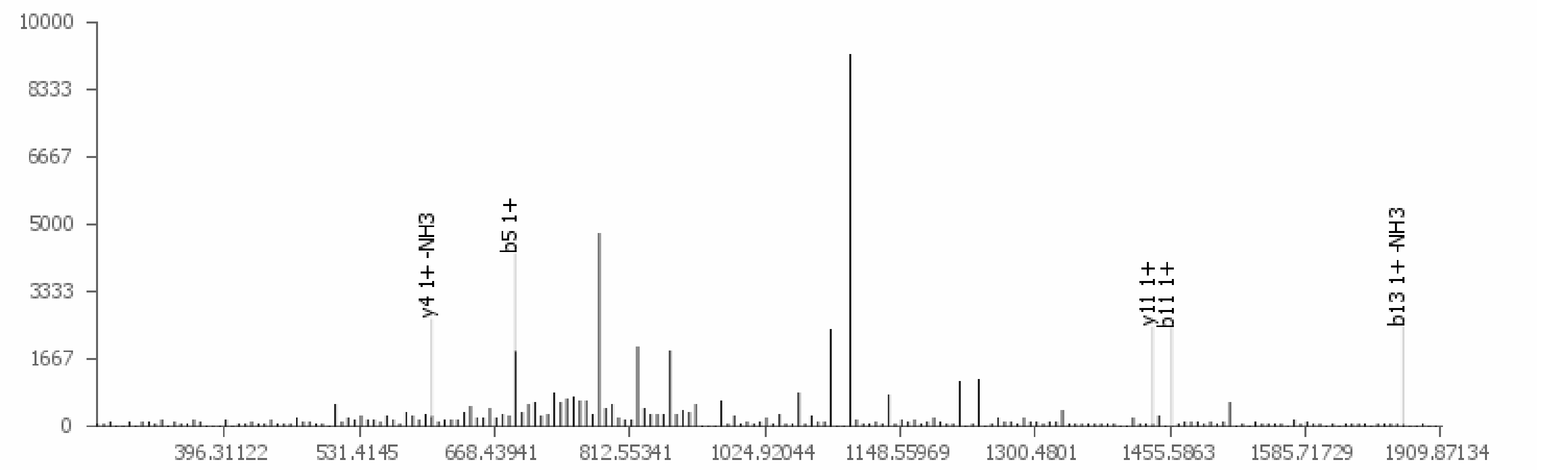

AT4G22770.1 - GK(oxM)KPA(pT)P(pT)PSSFIRPK - 1009.979156 - Charge:2

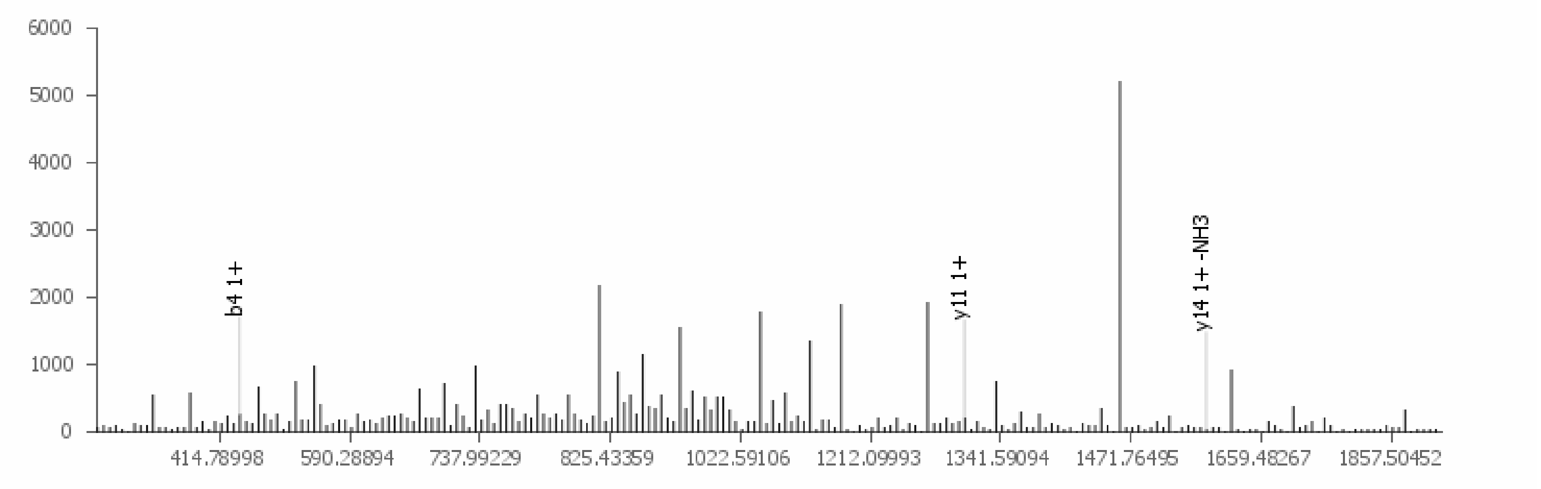

AT3G49790.1 - V(t)FE(t)VRSLLEVLIER - 992.532398 - Charge:2

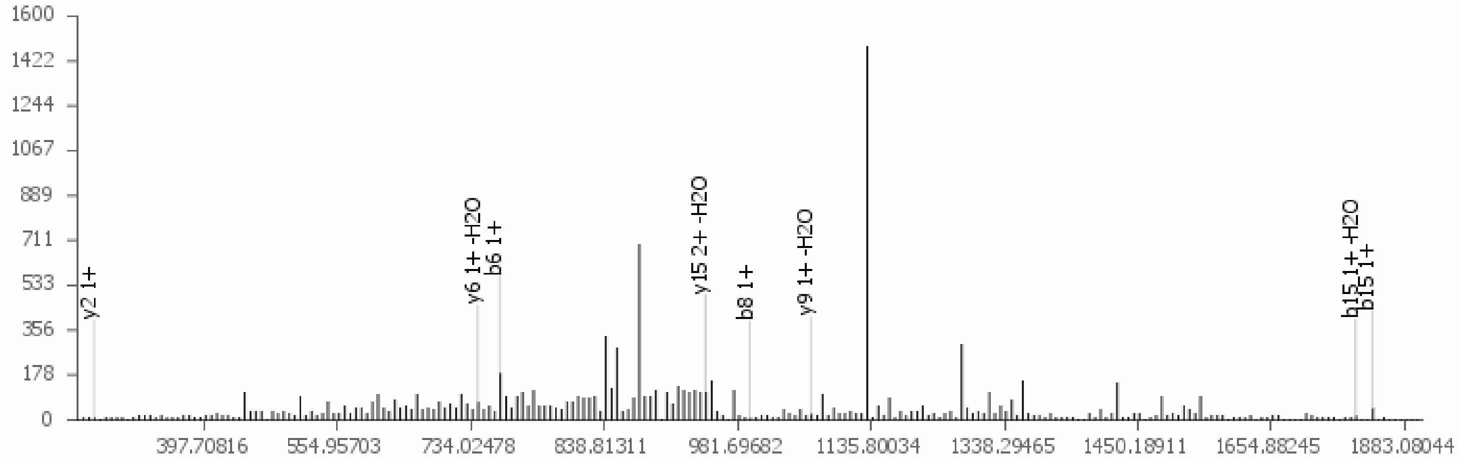

AT1G60760.1 - TKLAI(pT)D(pS)AR - 618.274082 - Charge:2

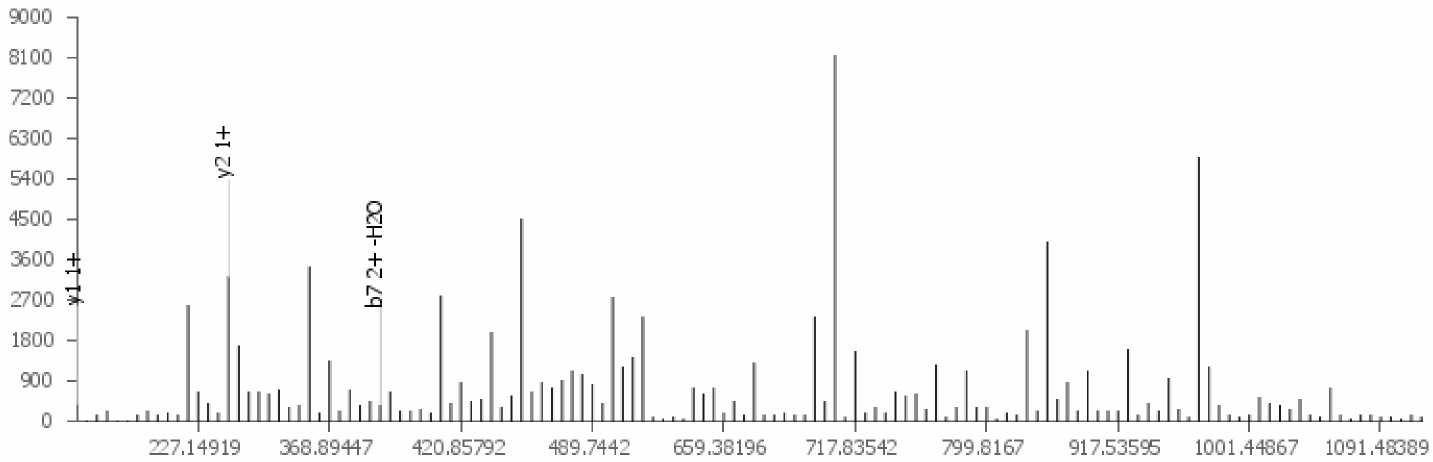

AT1G67690.1 - Q(pY)FPVNLVLSGIFK - 852.936733 - Charge:2

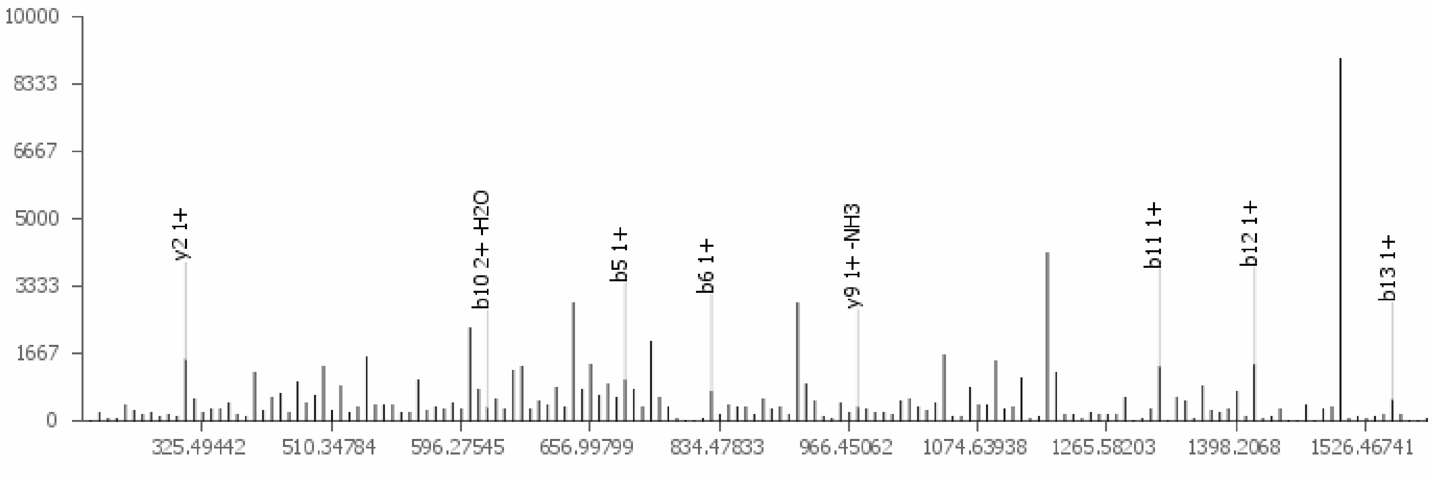

AT1G06990.1 - QN(s)D(s)QEFNQKLK - 823.369135 - Charge:2

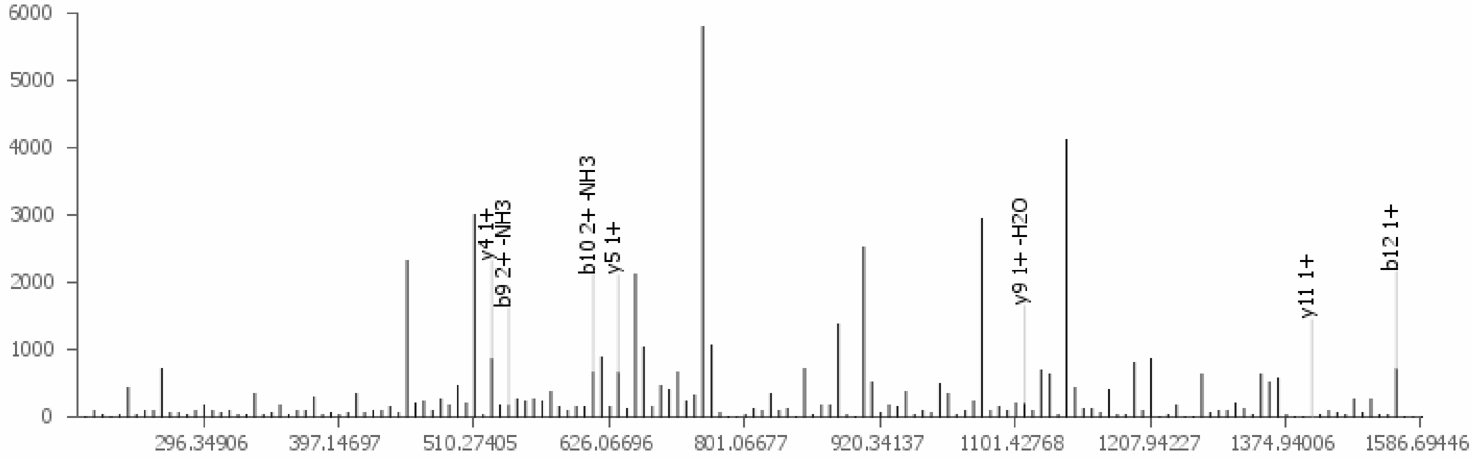

AT3G19515.2 - VSLA(t)(t)(y)MVVGASPKK - 866.433483 - Charge:2

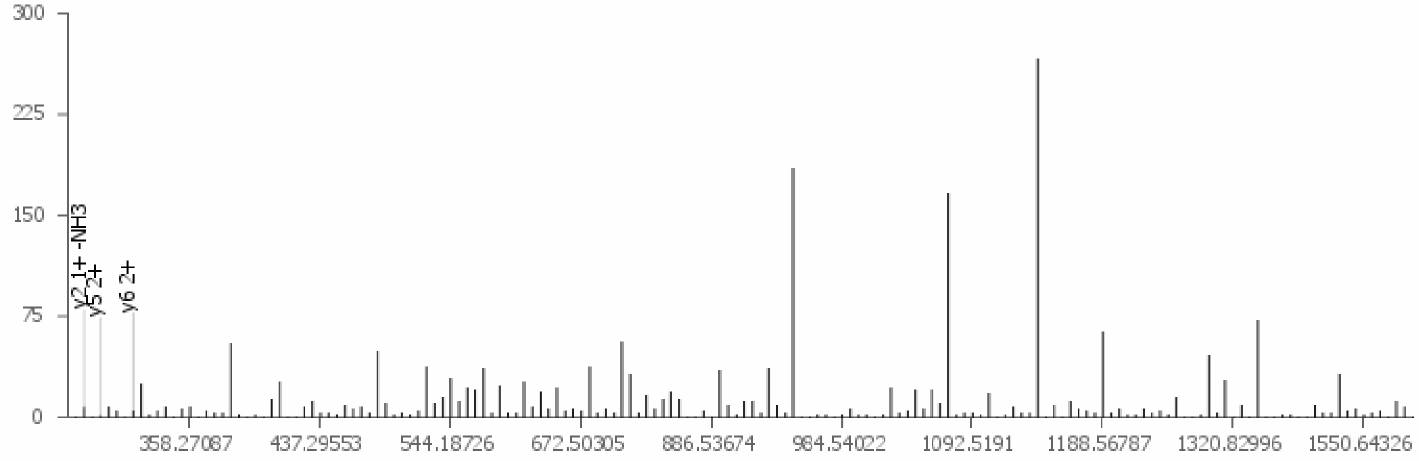

AT1G53830.1 - DITFQN(pT)AGP(pS)KHQAVALR - 738.676048 - Charge:3

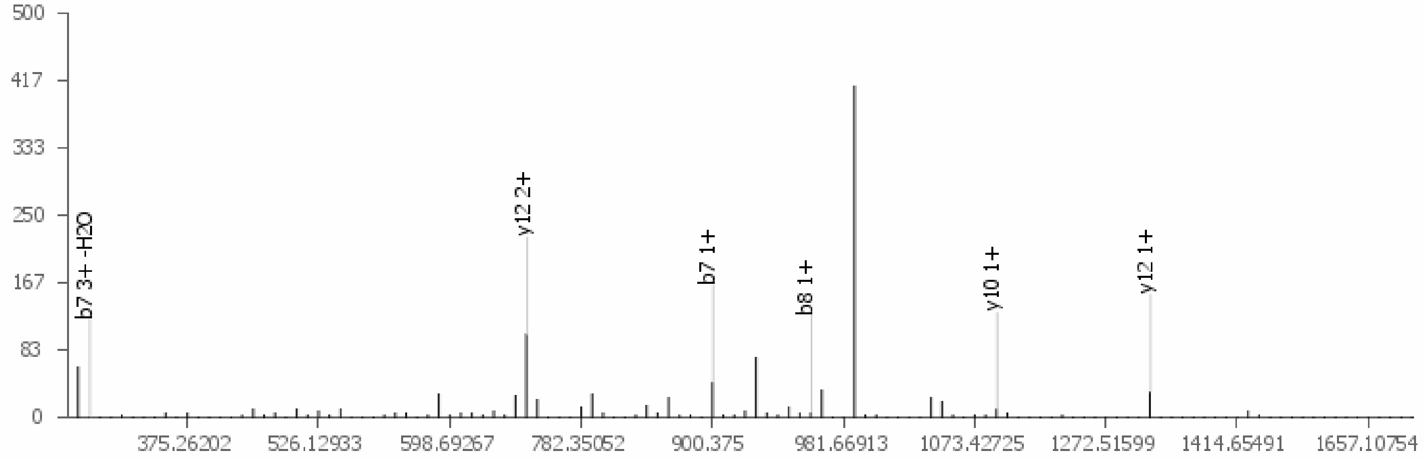

AT2G14250.1 - EELILKDN(t)G(s)TPLK - 869.430154 - Charge:2

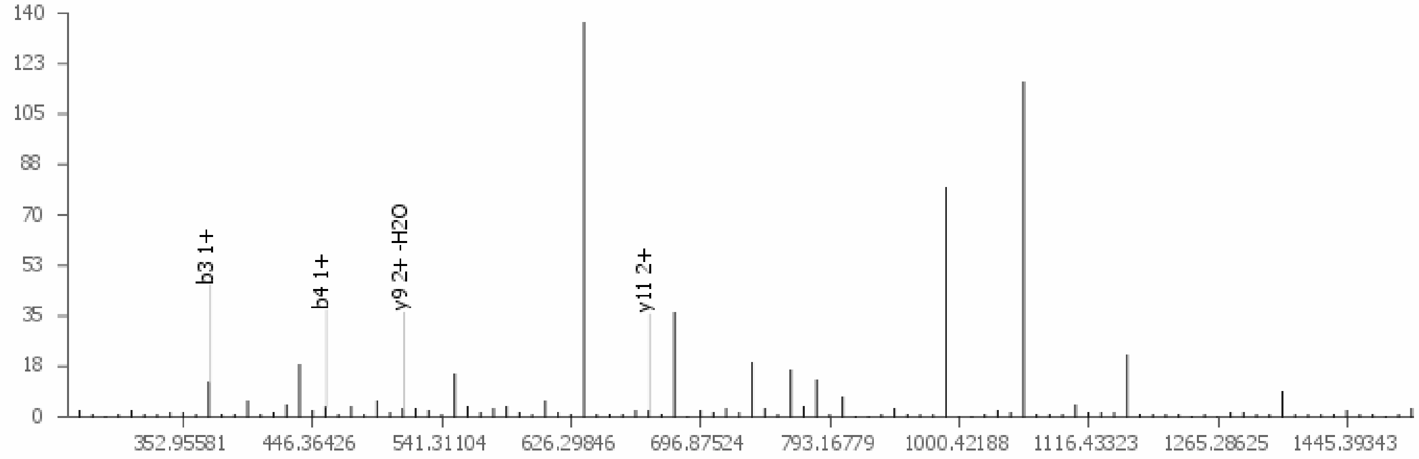

AT3G14350.1 - DGNLLNSGPAPPPPPG(pT)PPISK - 1102.039544 - Charge:2

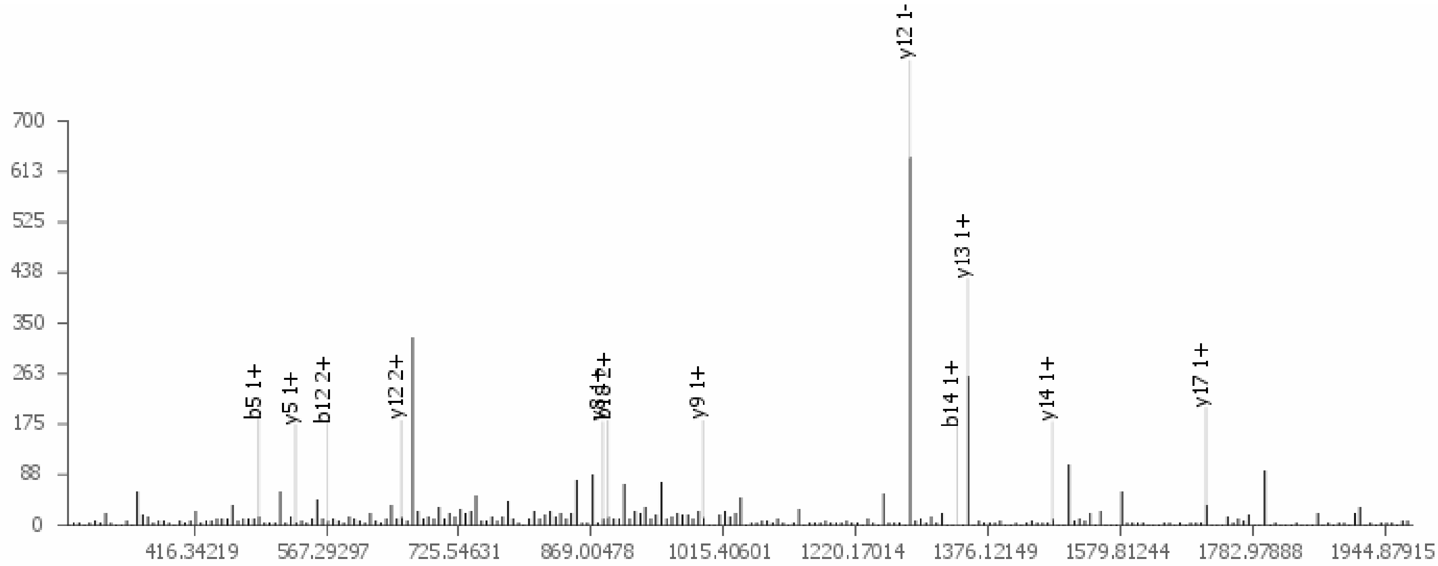

AT4G34220.1 - STAPINPLTEKPNQ(pT)GK(pS)K - 1086.015278 - Charge:2

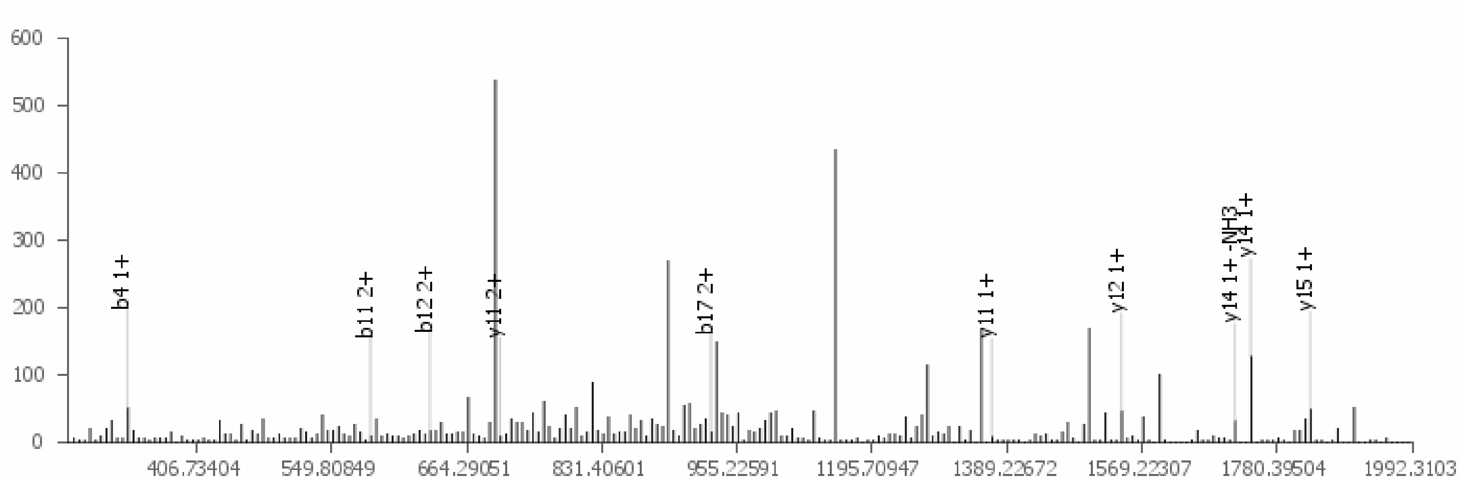

AT3G48860.1 - QVASLGEGV(pS)LEAK - 734.363394 - Charge:2

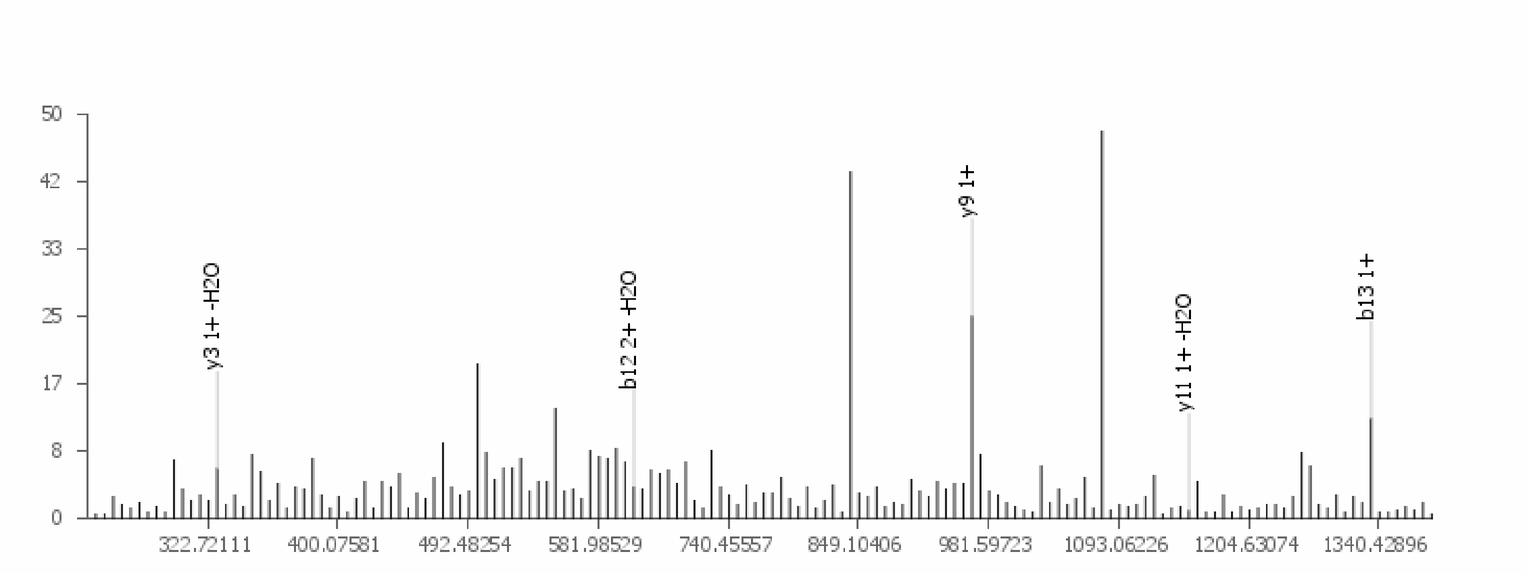

AT3G01370.1 - WL,(pT,)GGTLISRDK - 722.327579 - Charge:2

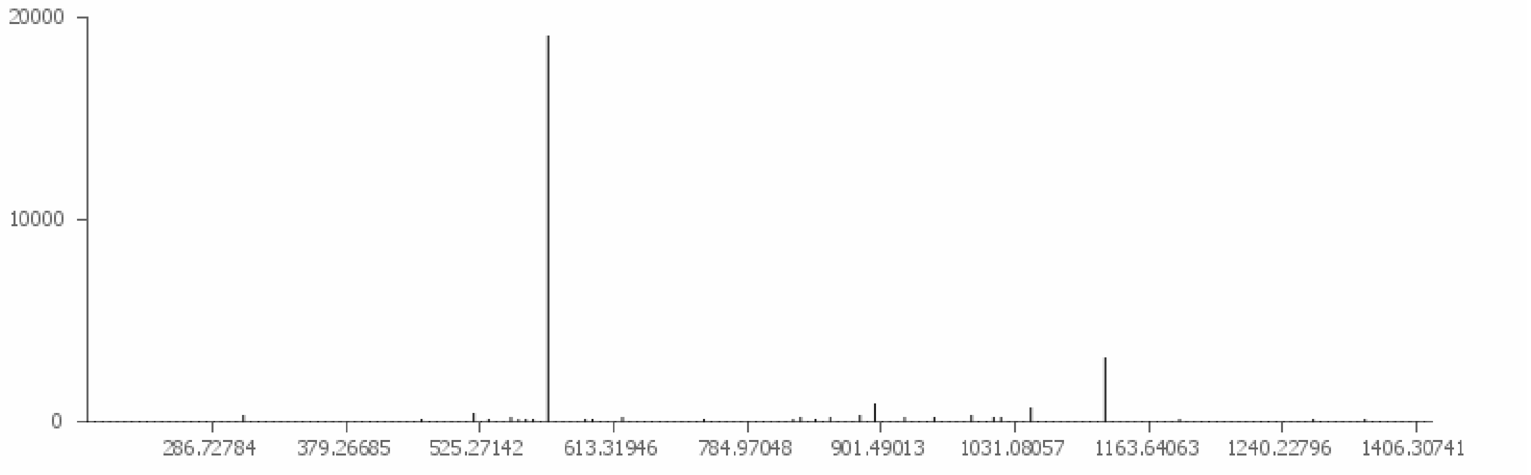

AT3G47430.1 - (pT)GRSLTGFNALR - 686.847001 - Charge:2

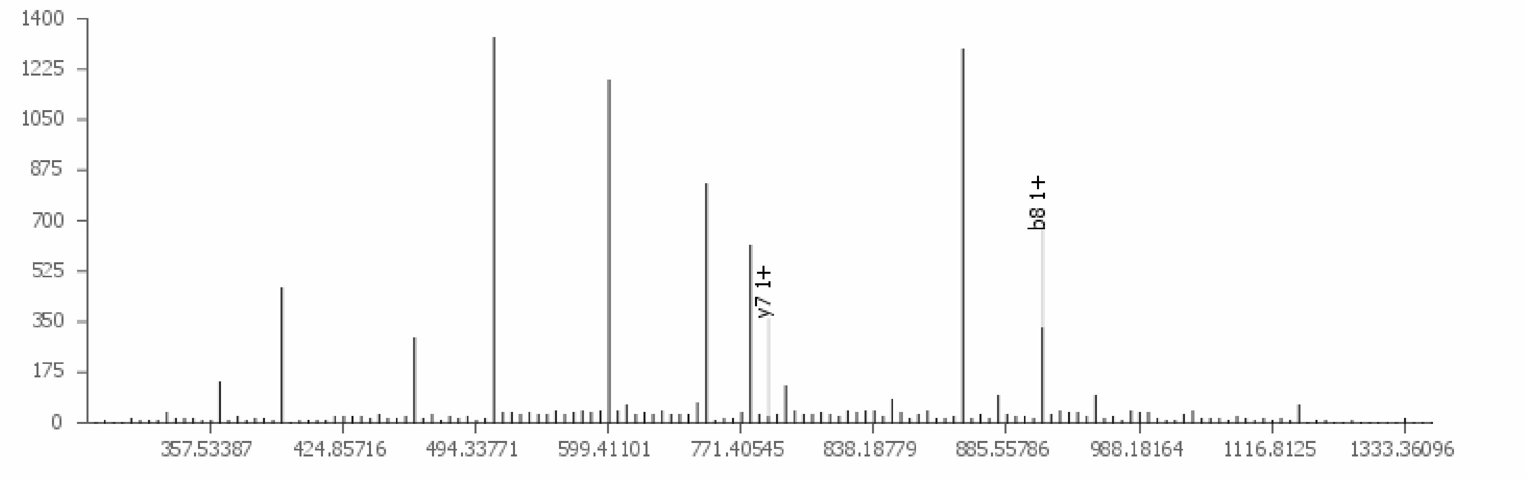

AT5G45470.1 - FLYPI(pY)FEFISR - 837.895694 - Charge:2

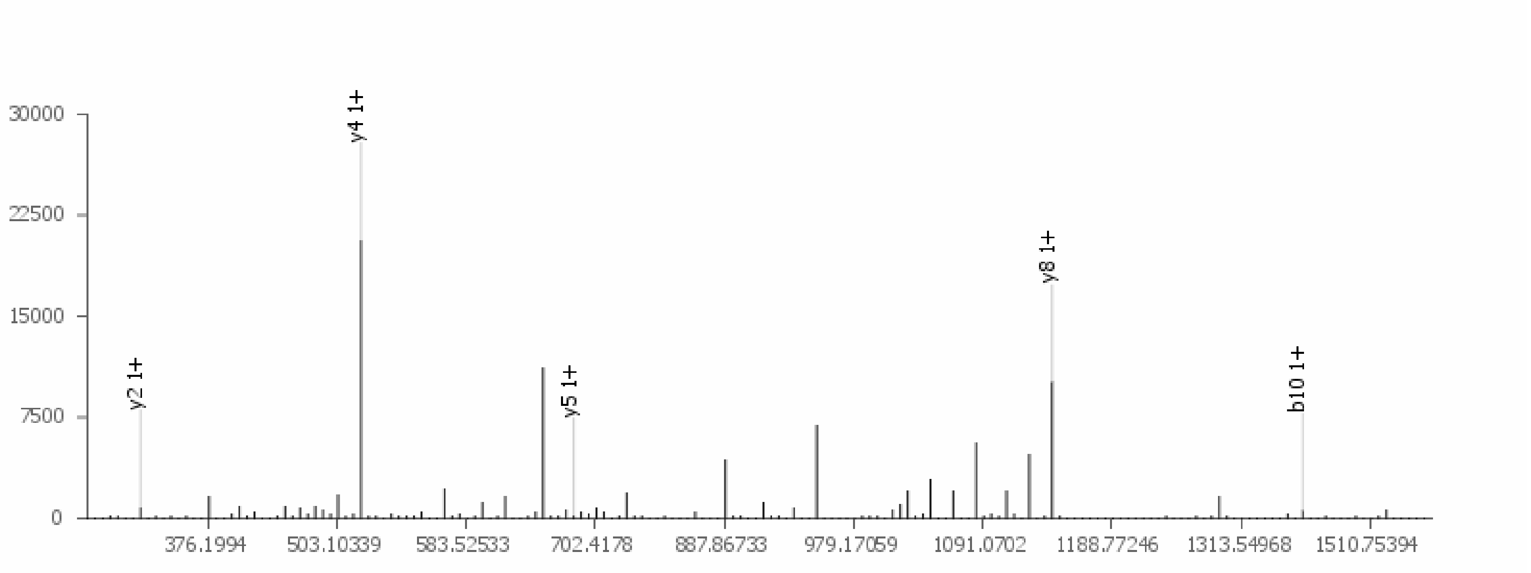

AT2G46220.1 - KATV(pS)AIDAR - 556.273802 - Charge:2

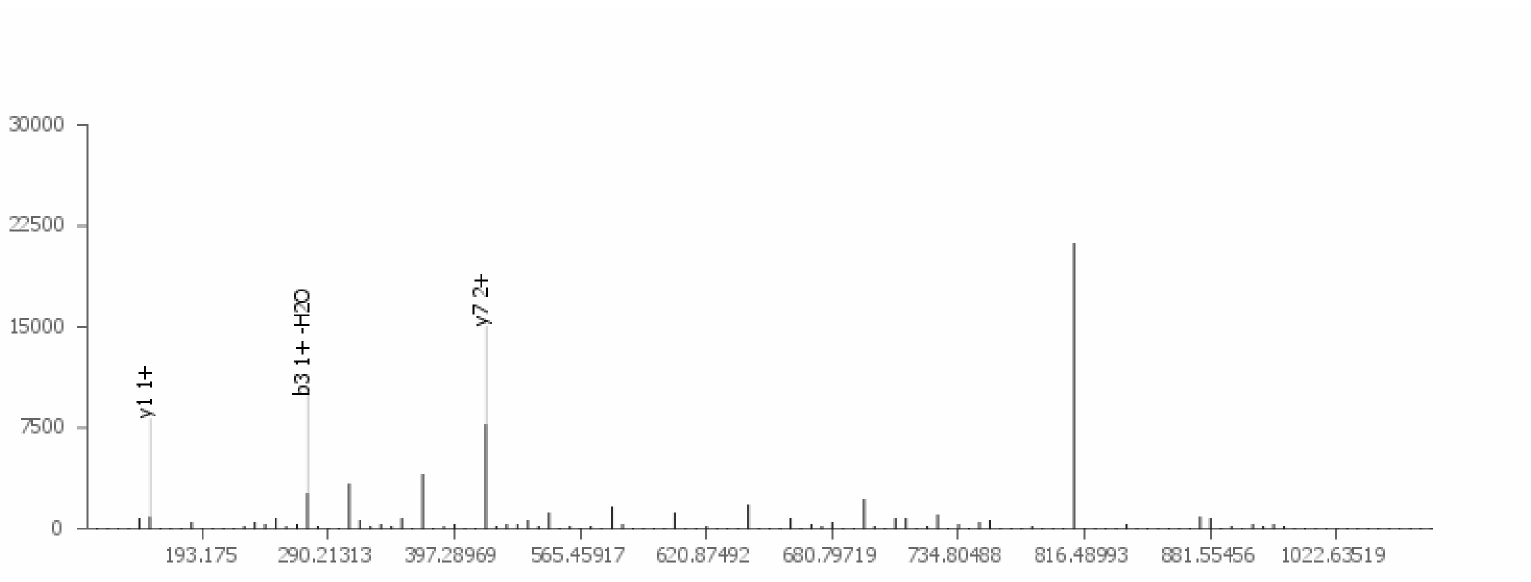

AT2G44600.1 - KSDAG(t)G(t)GAGGR - 616.237806 - Charge:2

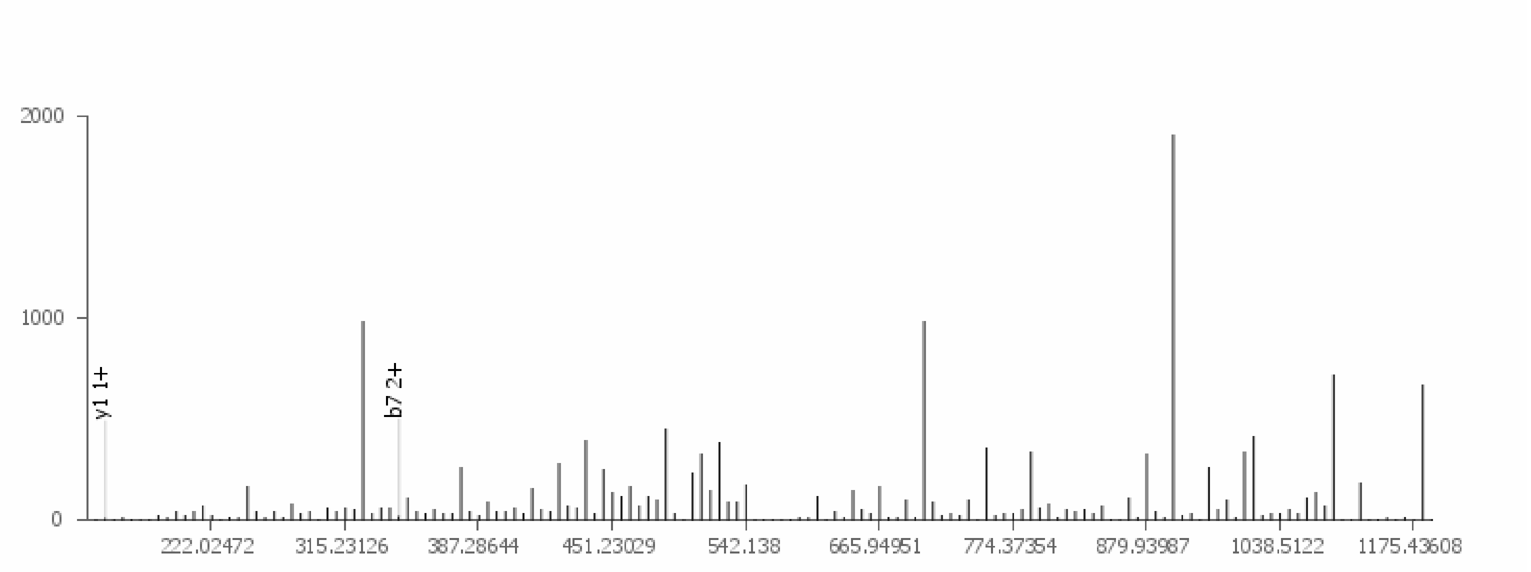

AT2G29210.1 - IGGSHAANHLE(s)P(s)P(s)SLSPPGR - 1168.042605 - Charge:2

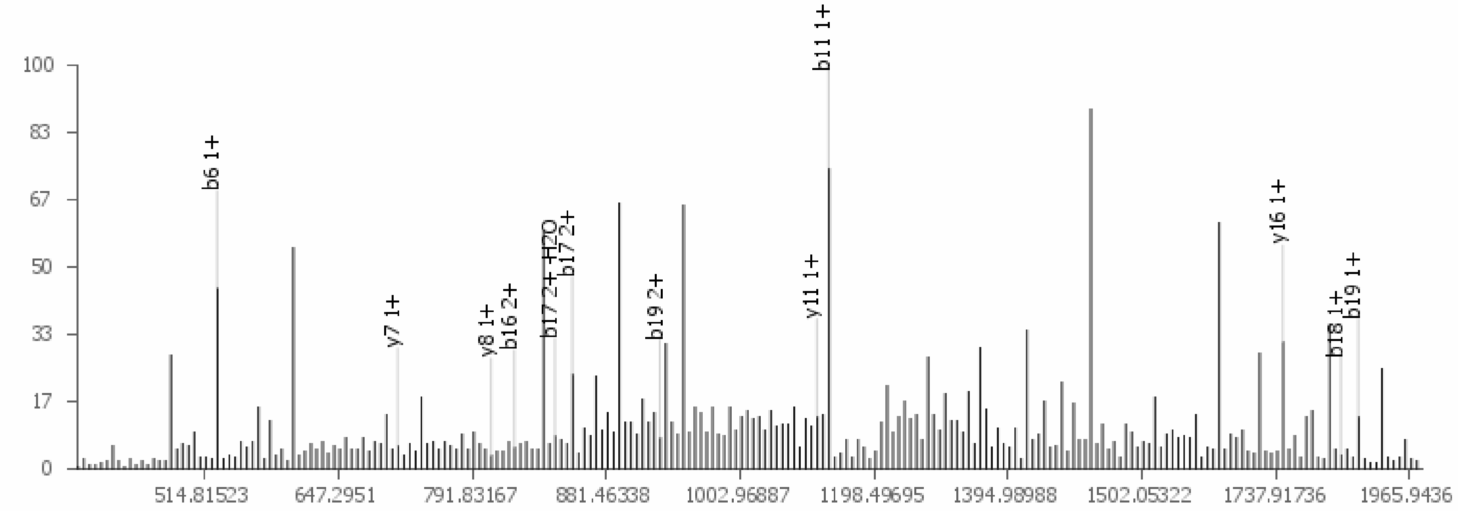

AT5G61030.1 - FGNILKQT,(pT,)NK - 680.321891 - Charge:2

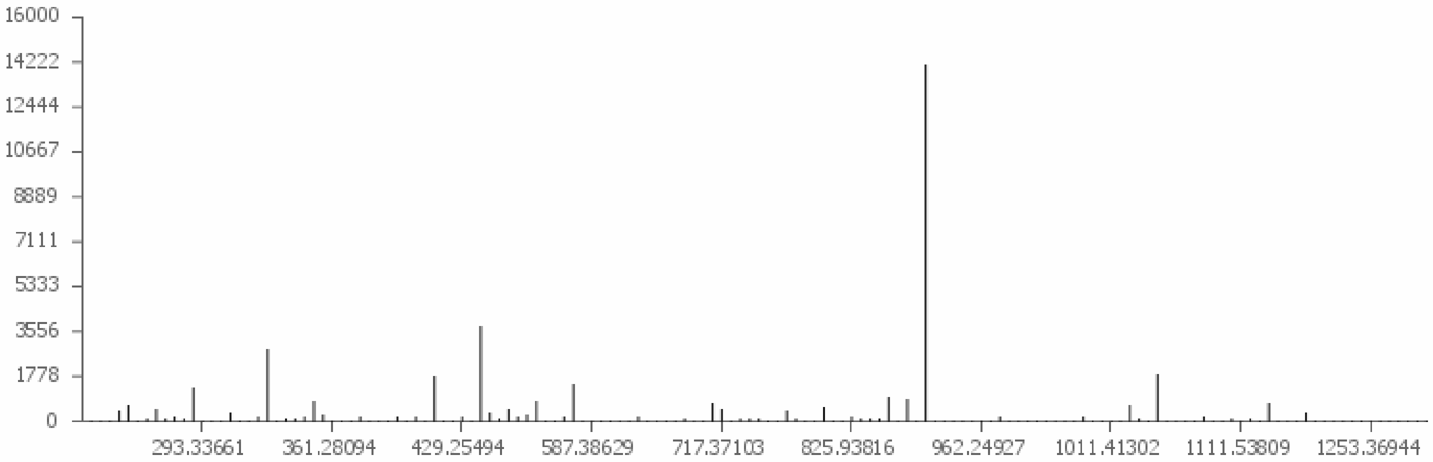

ATCG01000.1 - IP(pS)PIF(pT)K - 1062.476712 - Charge:1

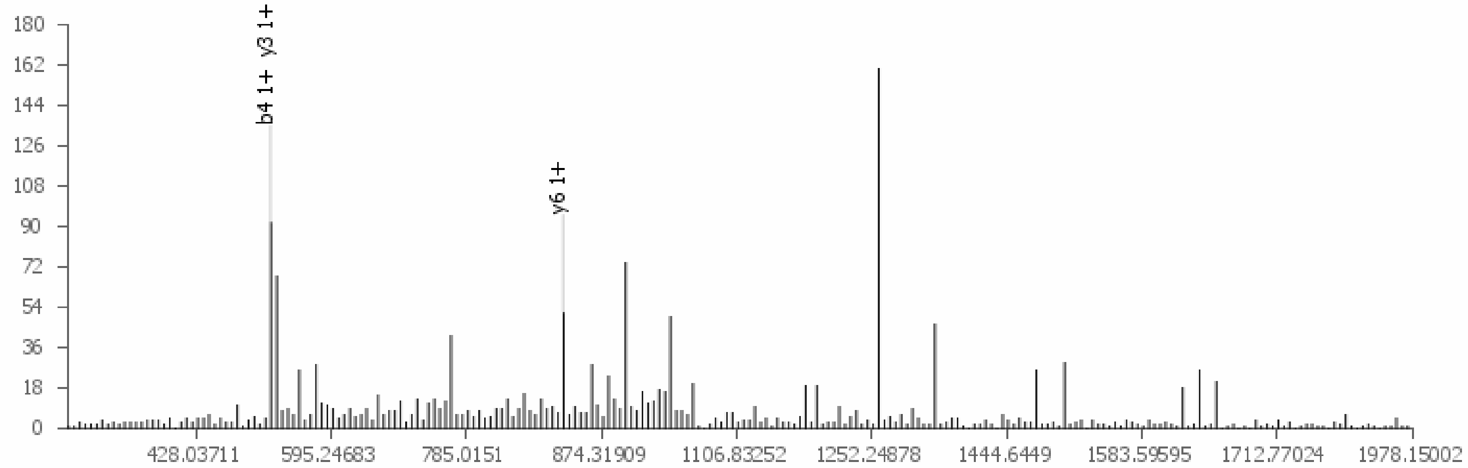

AT4G18195.1 - LK(t)(s)H(t)(s)PVGDPHLLPAEEGHTNIHSV - 985.140592 - Charge:3

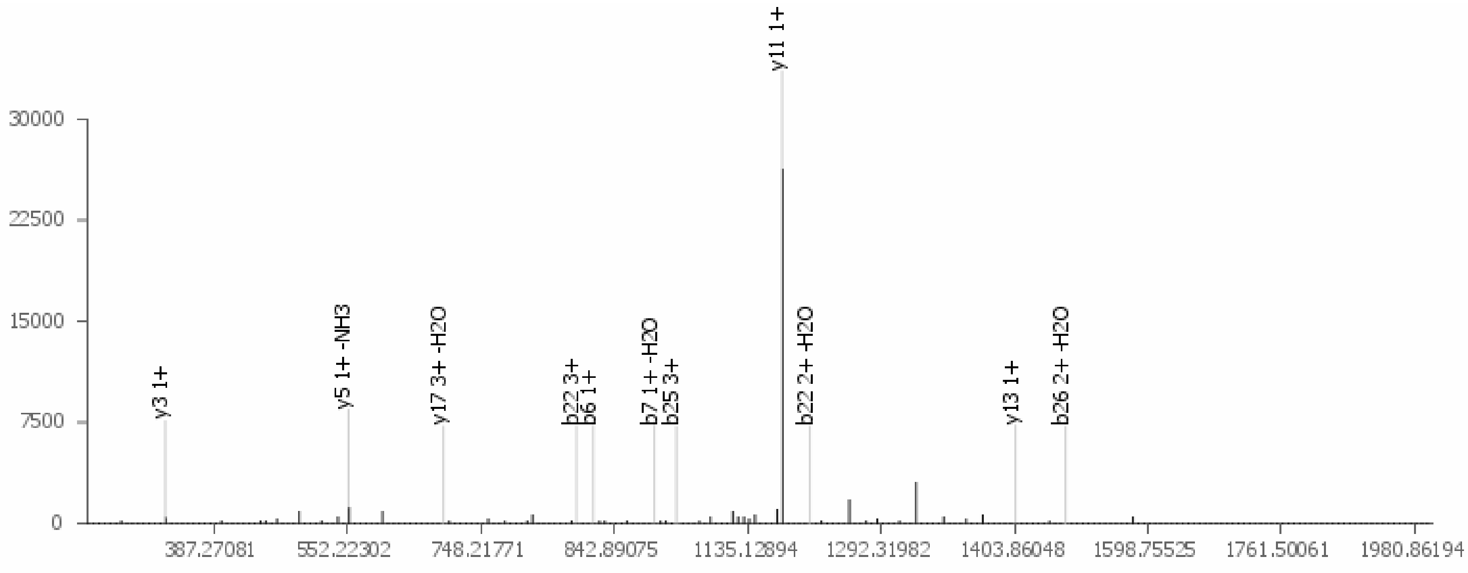

AT4G13510.1 - (pS)PSPSGANTTPTPV - 696.808427 - Charge:2

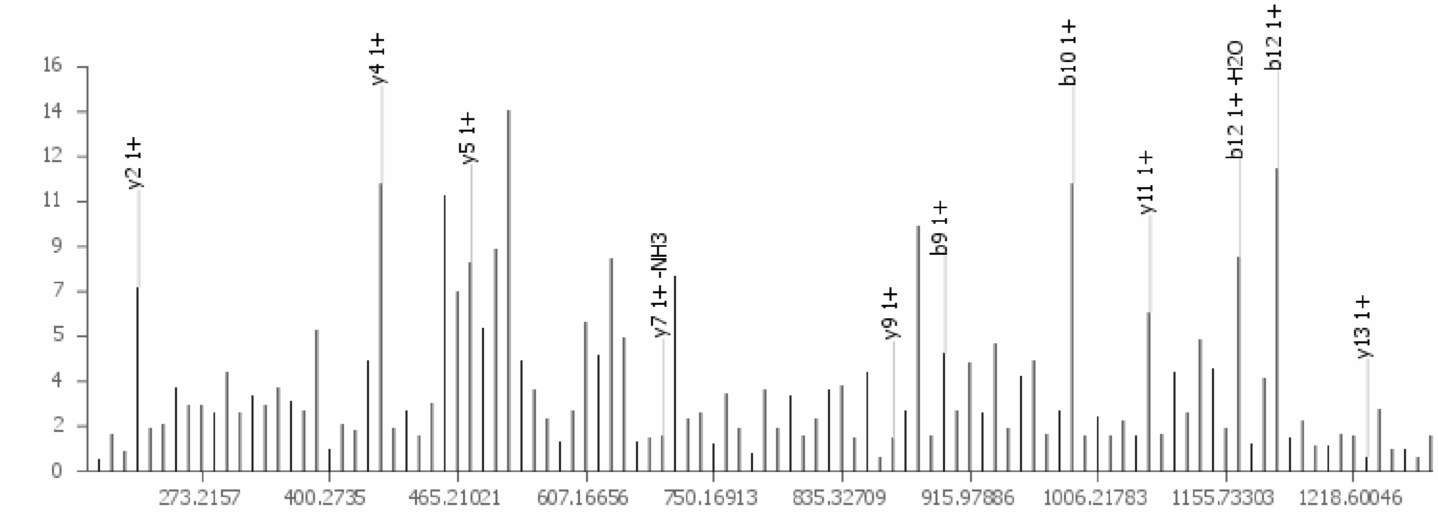

AT4G14940.1 - VRLG(pS)DPVELLIVNPNK - 972.01948 - Charge:2

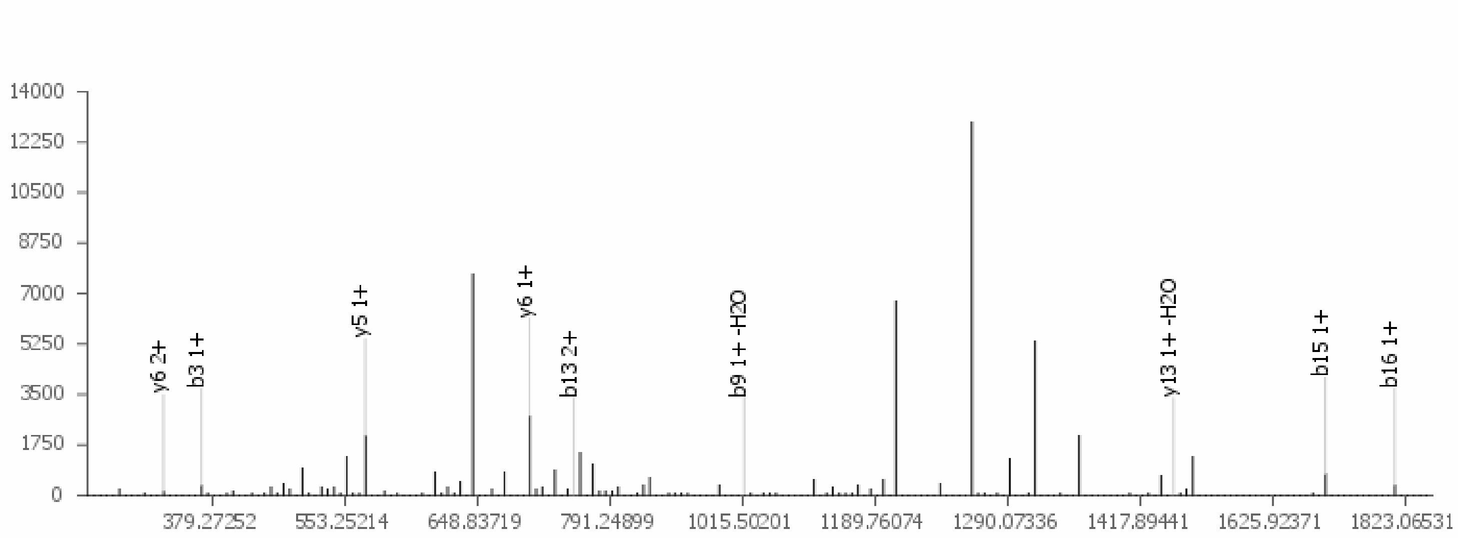

AT1G78400.1 - VNCGPGHGI(pS)VGSLGKFK - 918.944727 - Charge:2

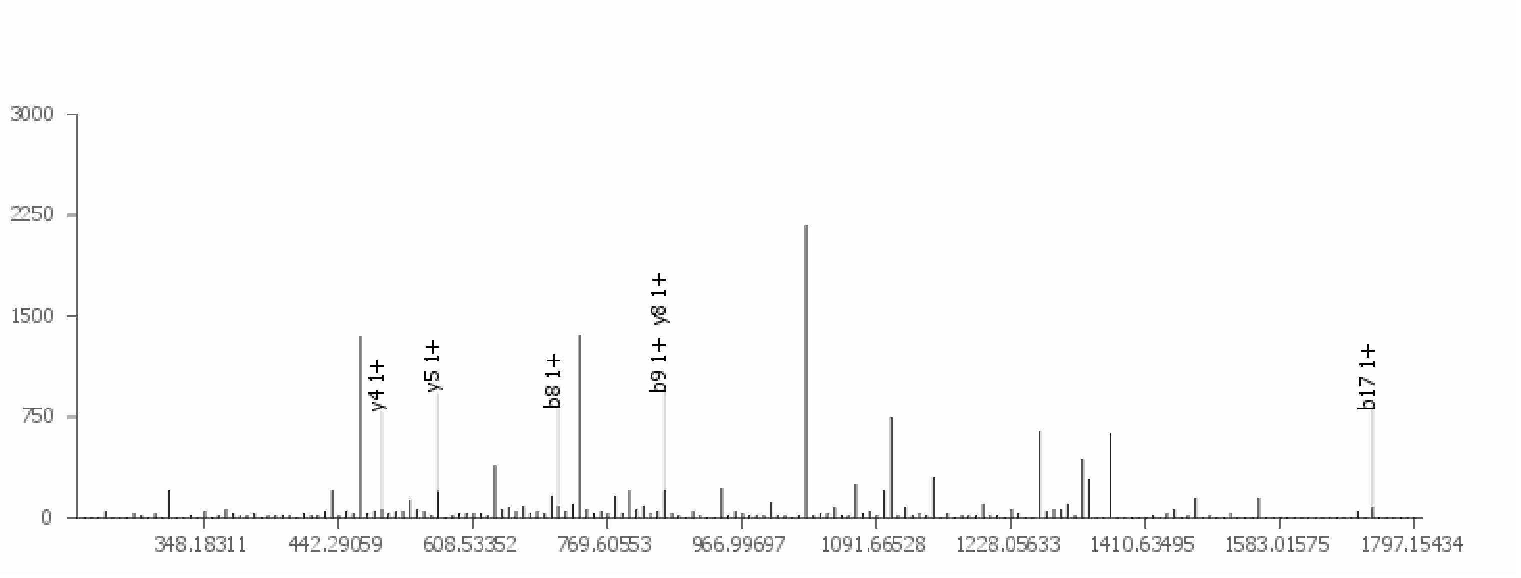

AT1G76290.1 - V(pT)NFGG(pS)PPVLNMIANAR - 1009.45696 - Charge:2

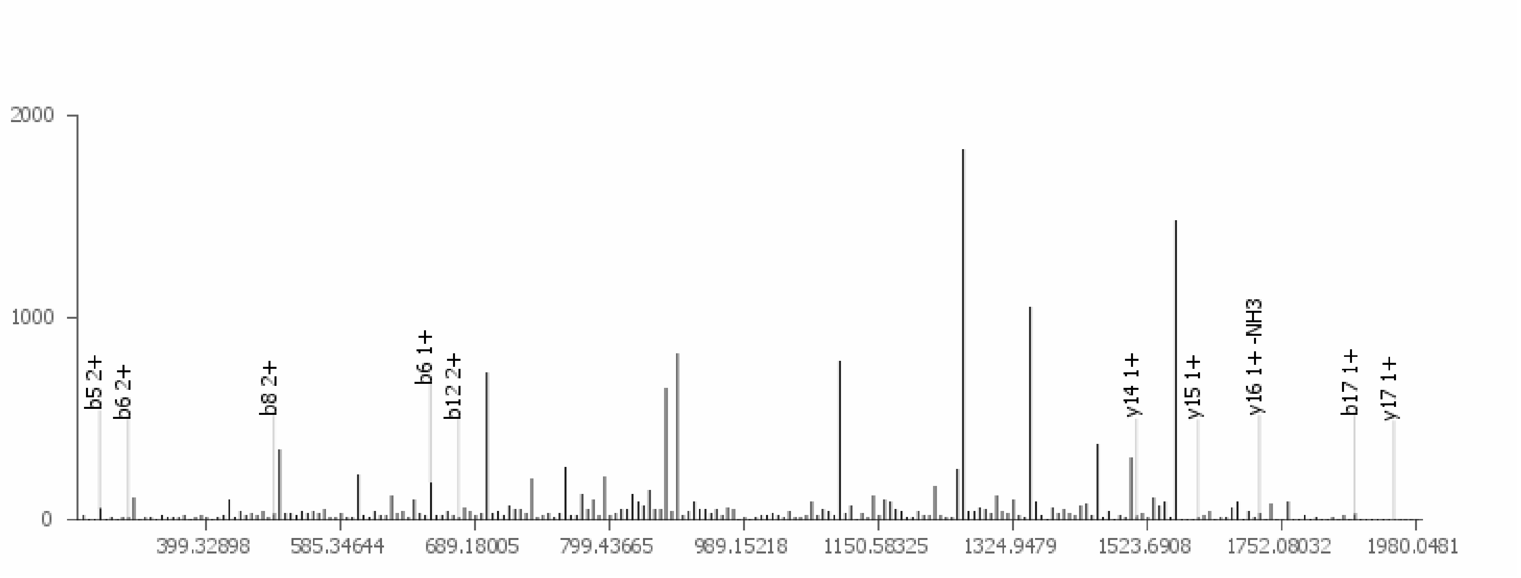

AT1G24160.1 - QNPAKP(pT)IIP(pS)VA(pT)RQK - 1044.97733 - Charge:2

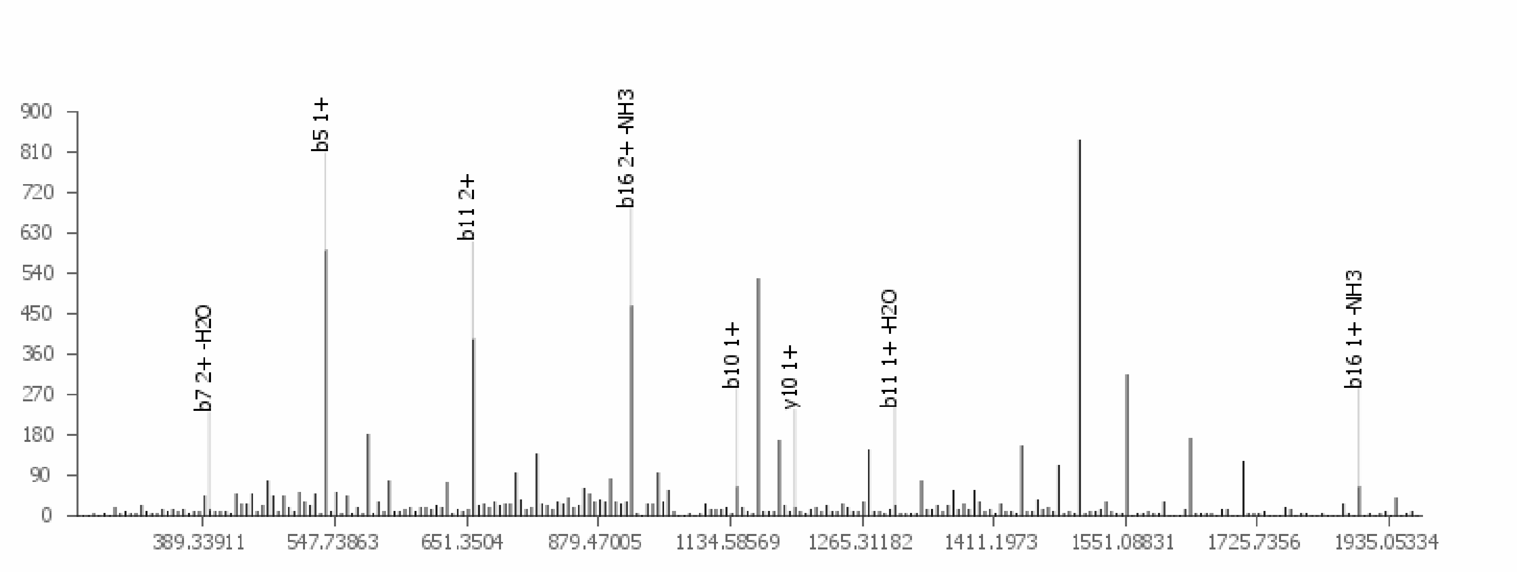

AT2G25670.1 - EPEPQV(pY)WAPTPLKVK - 981.482531 - Charge:2

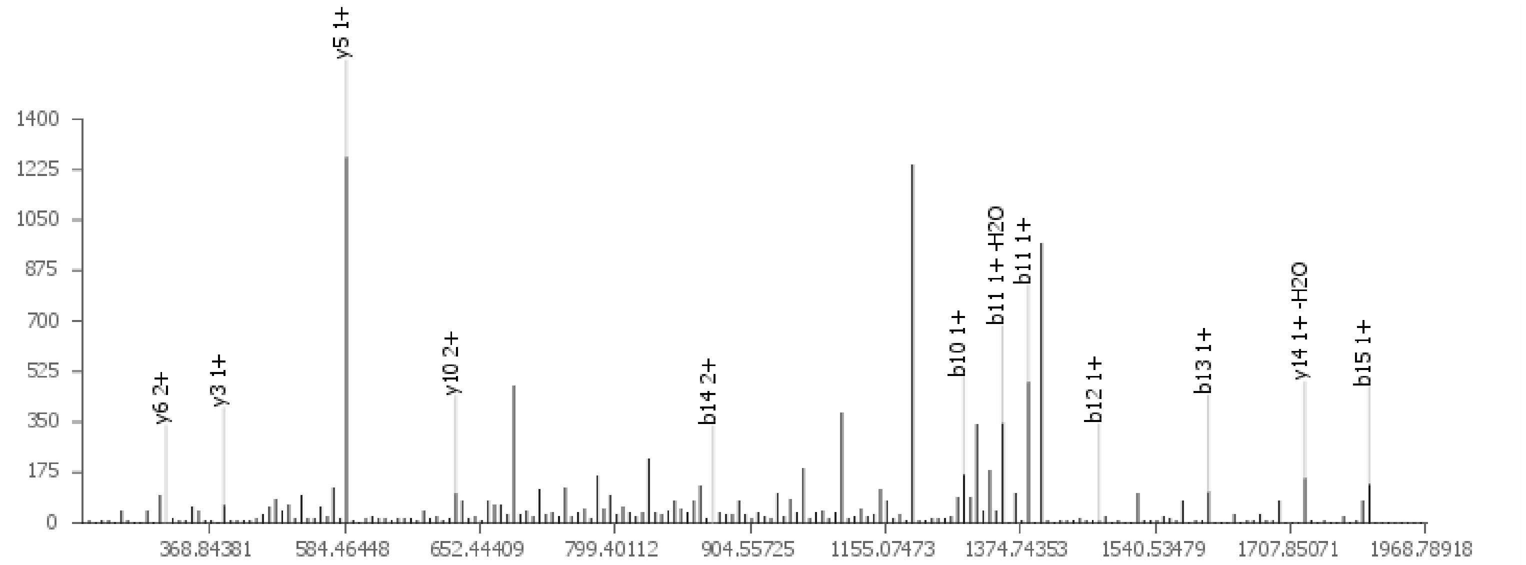

AT4G02580.1 - VKEILSYYPSN(pY)K - 842.407053 - Charge:2

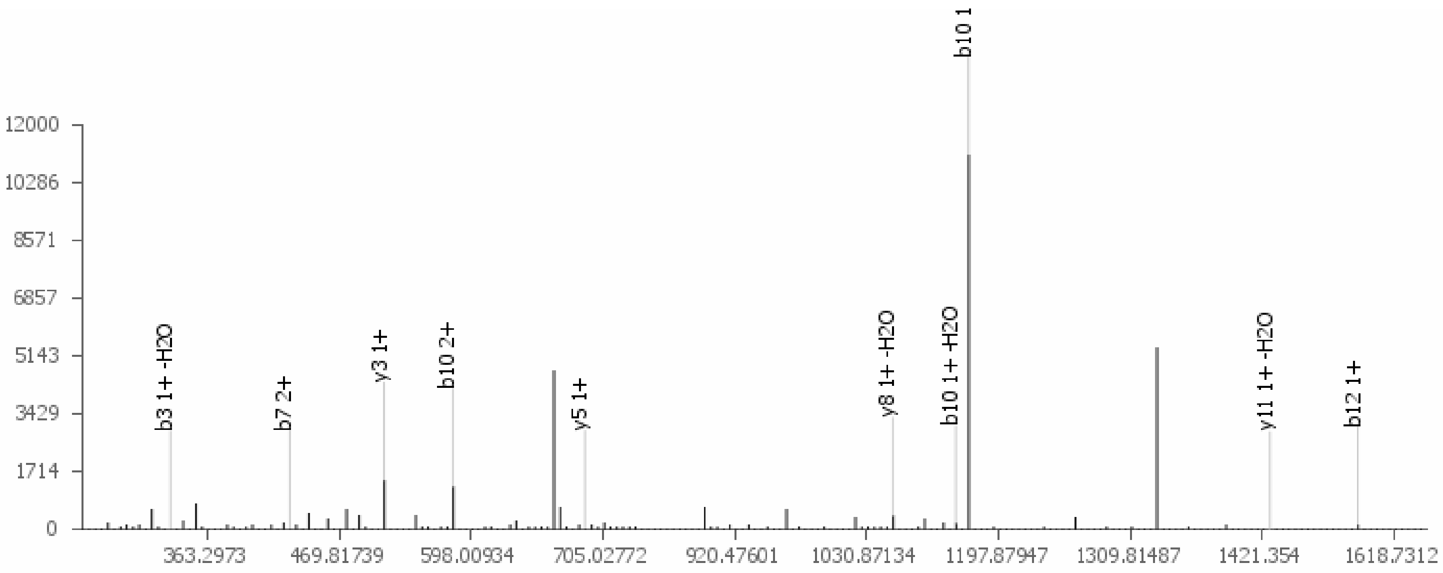

AT5G51630.1 - QEHLV(s)LHM(t)HSK - 813.87467 - Charge:2

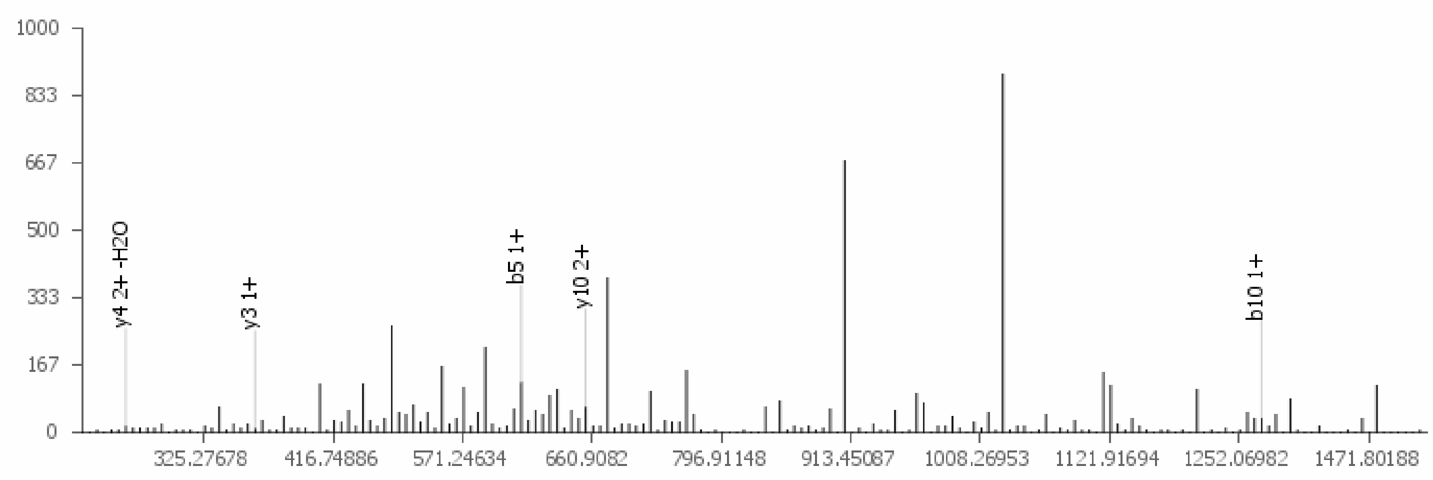

AT3G16510.1 - MDVYAVVWI,(pT,)GDDSR - 913.369751 - Charge:2

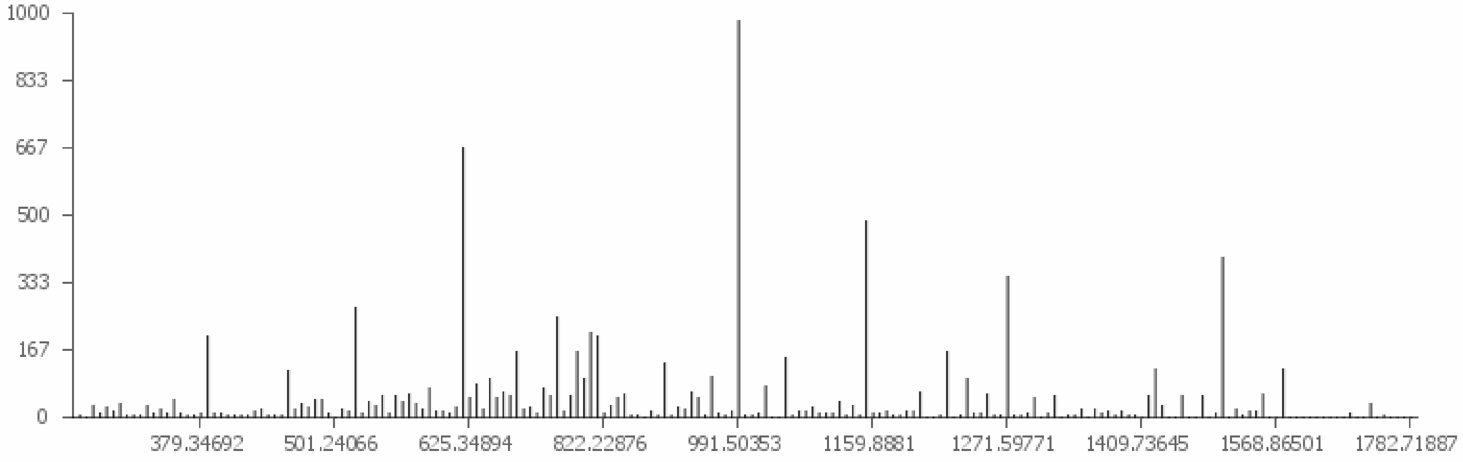

AT5G49840.1 - SNVLLLGP(pT)G(pS)GKTLLAK - 964.993357 - Charge:2

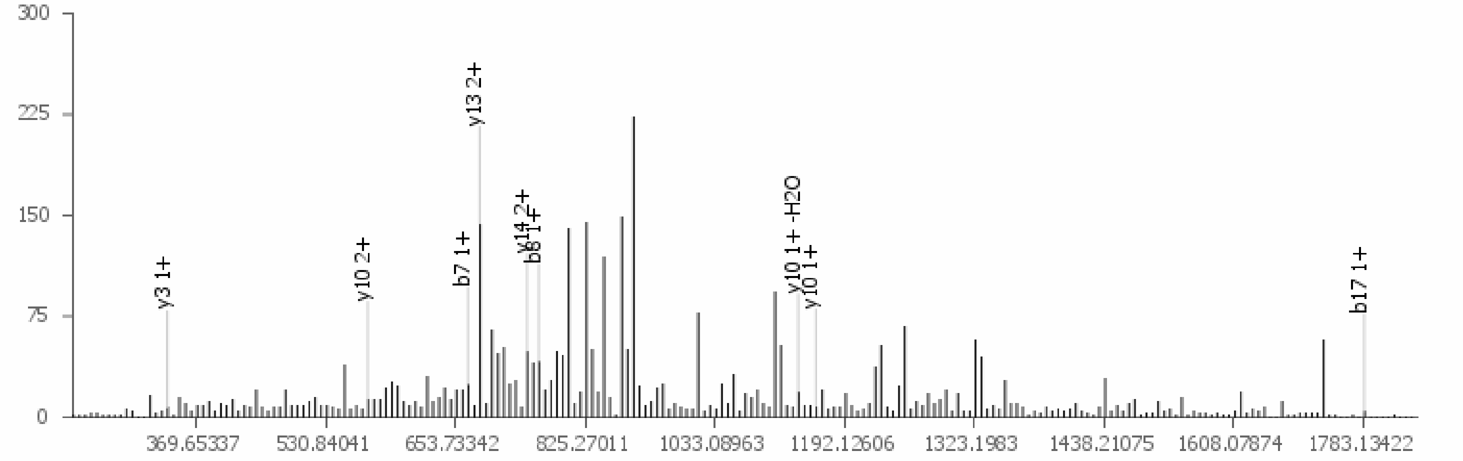

AT4G32860.1 - ,(pY,)N,(pT,)LPKVR - 582.237755 - Charge:2

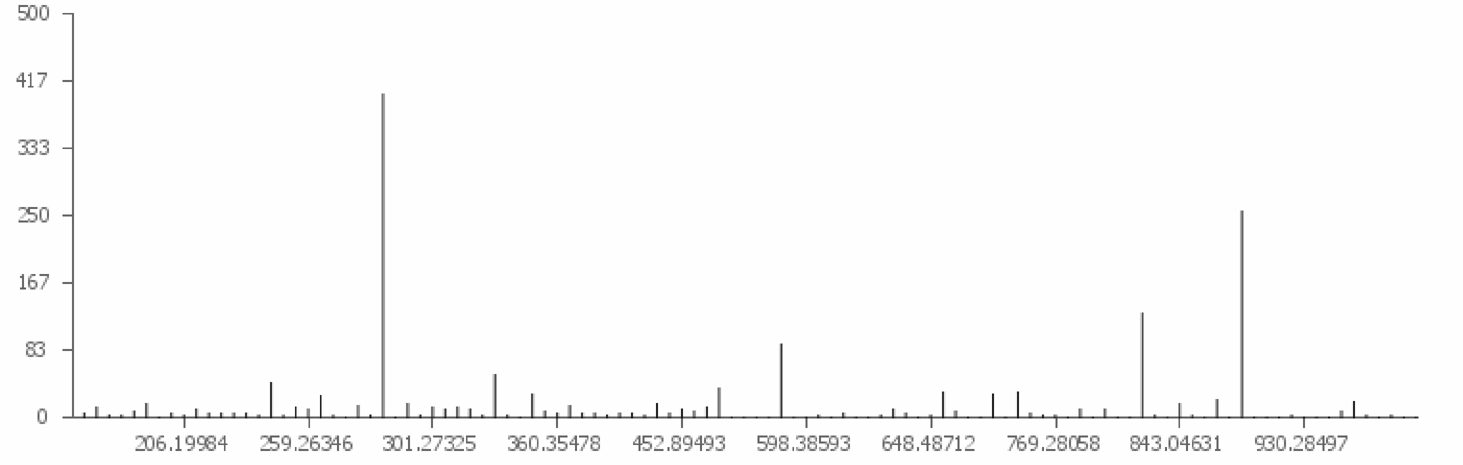

AT2G43260.1 - LF(s)(t)PGSWISKILK - 828.946935 - Charge:2

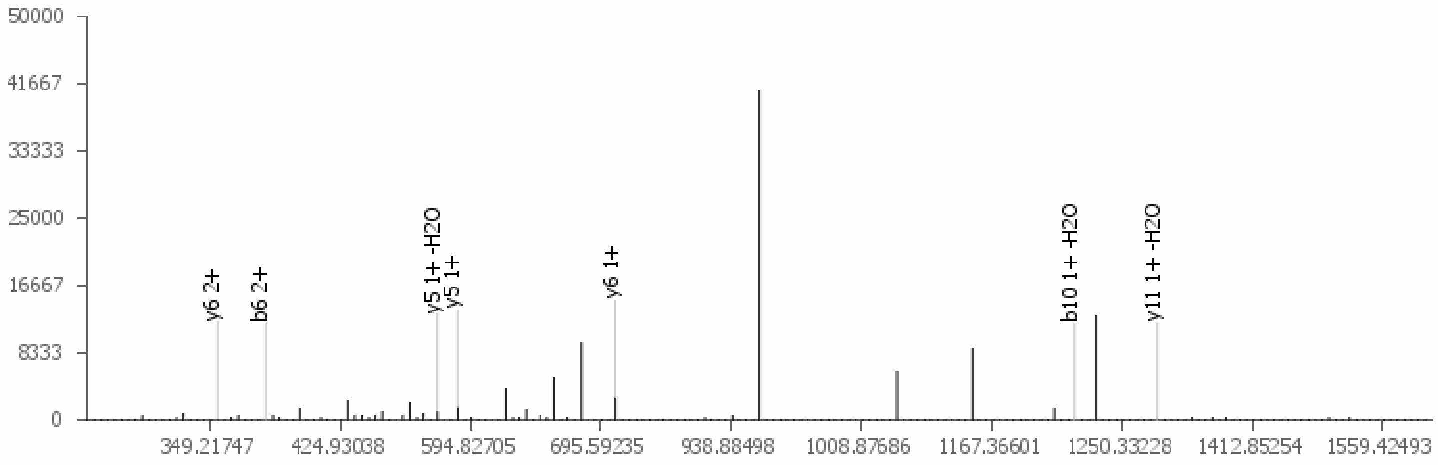

AT4G30690.1 - SATVRLIDDQQNMIGLV(pS)K - 1084.542335 - Charge:2

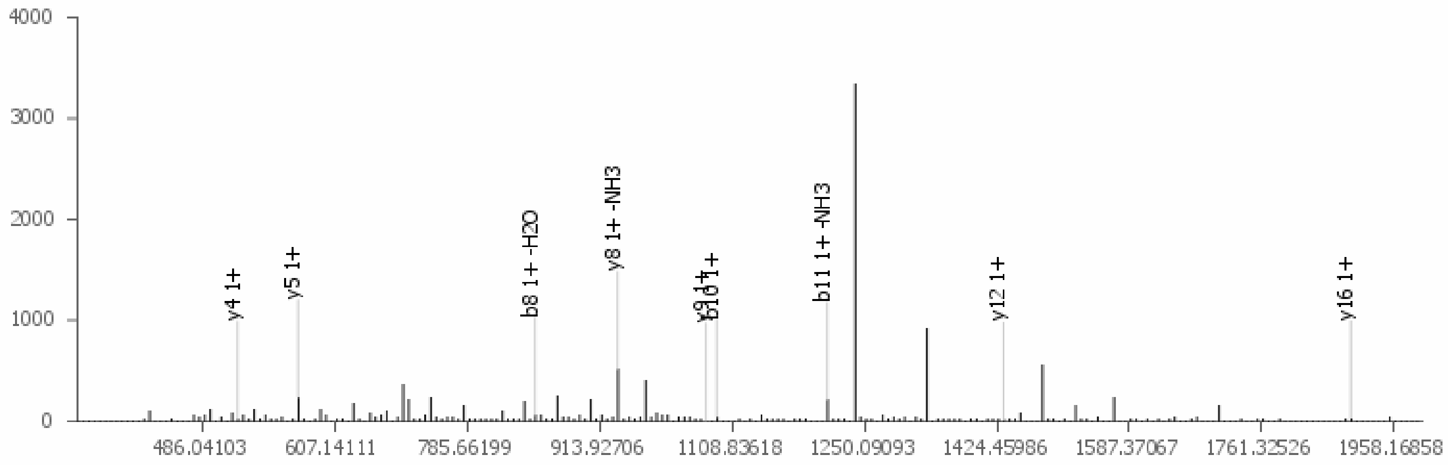

AT1G52050.1 - TSEFIG,(pY,)QK - 582.235933 - Charge:2

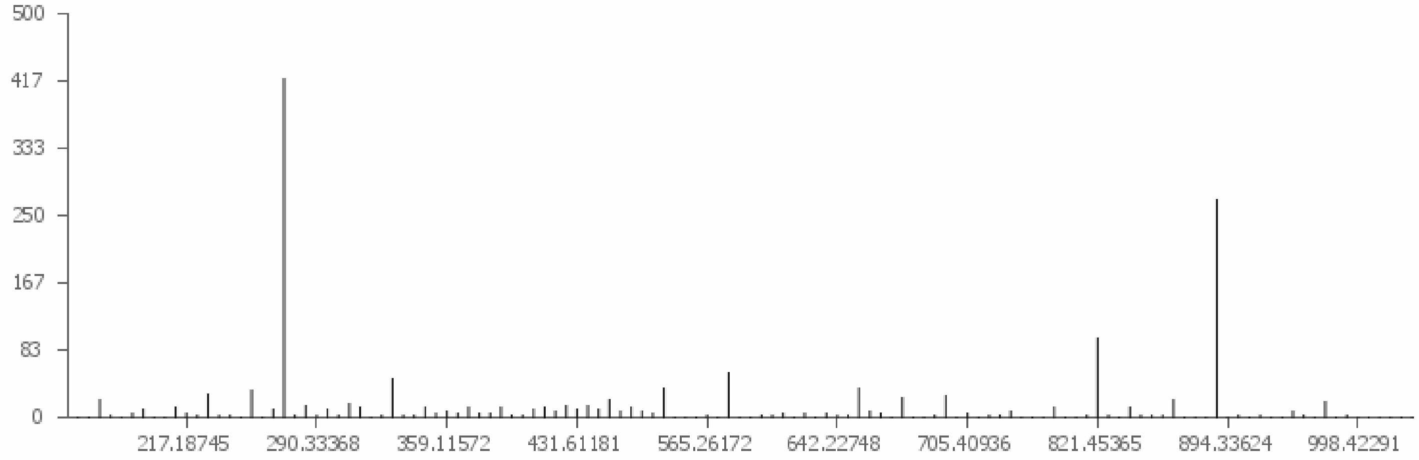

AT5G14060.1 - LF(pS)AYLNK - 518.244429 - Charge:2

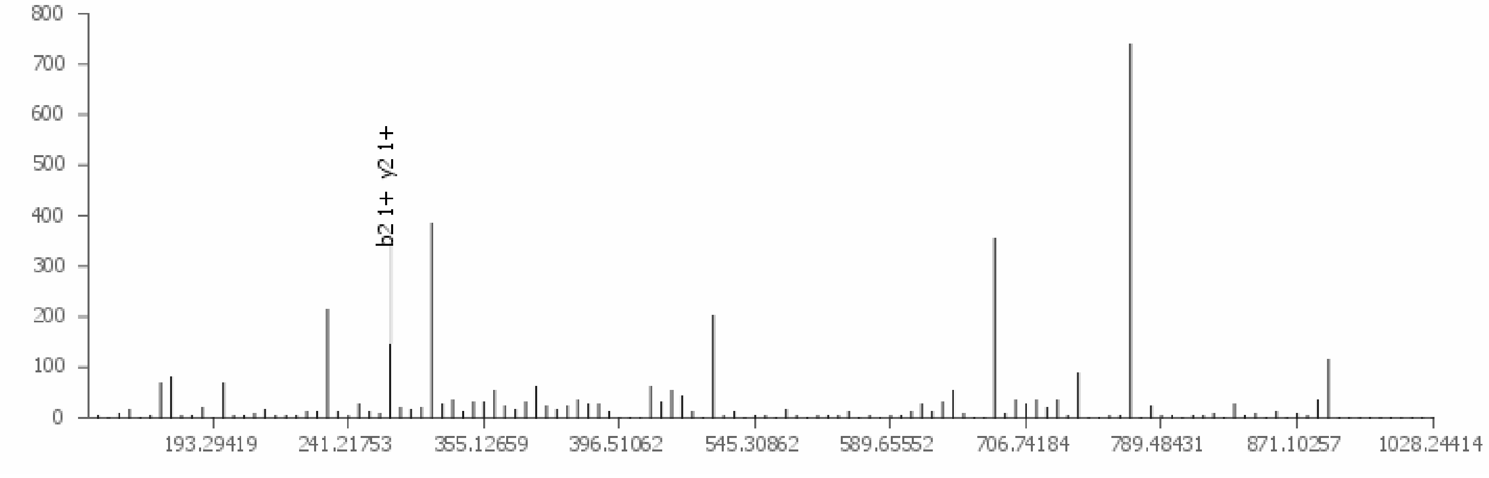

AT1G26790.1 - WL(pS)HLFMSLSK - 714.841905 - Charge:2

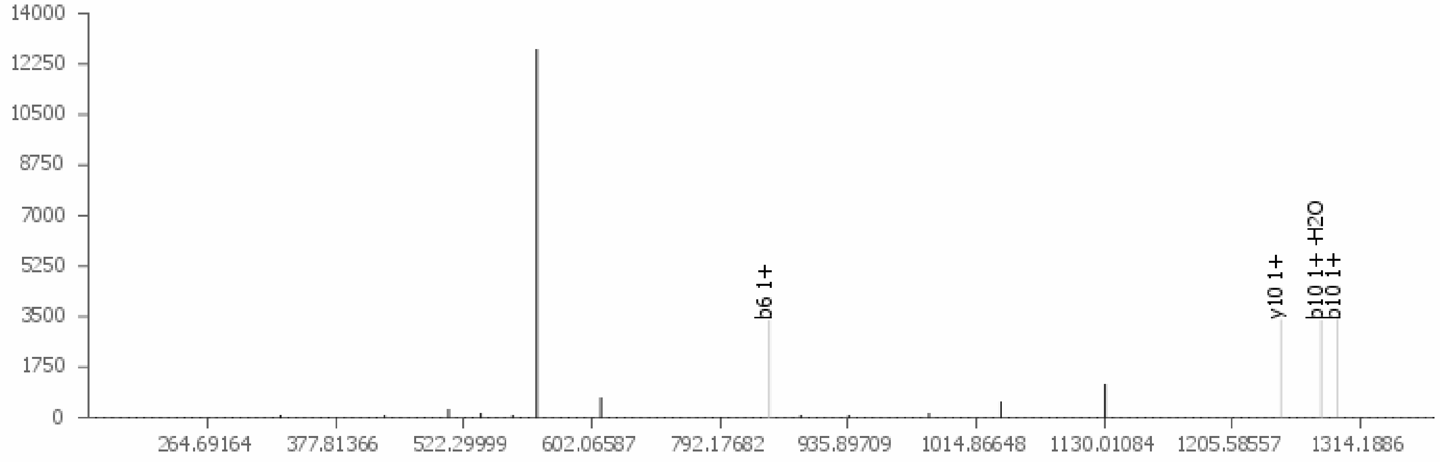

AT4G36990.1 - SVIA(pS)(pT)AGK - 497.206861 - Charge:2

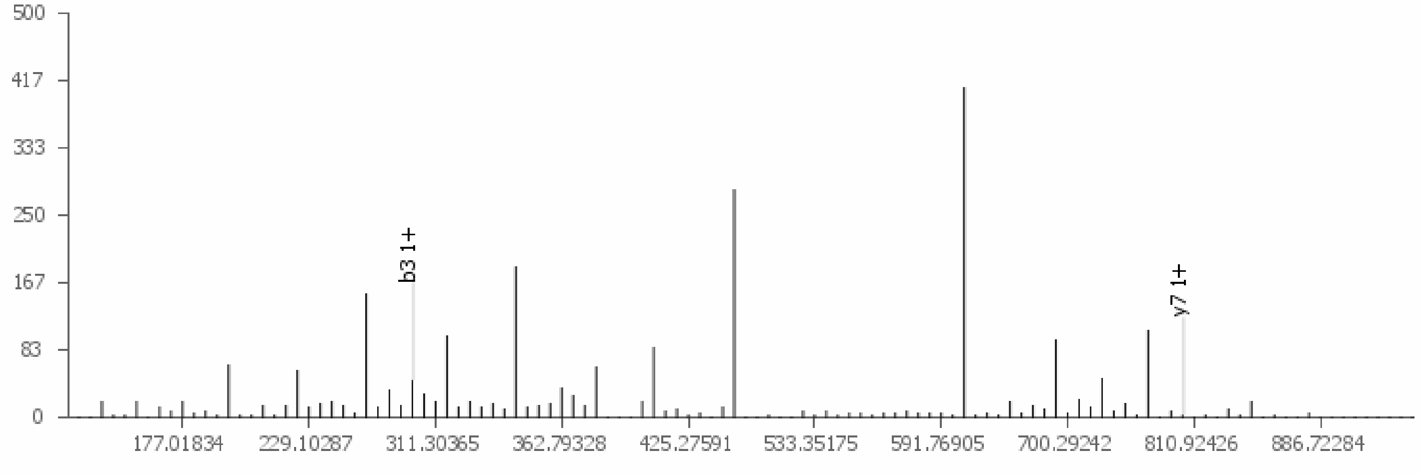

AT5G66950.1 - SVISCLQ(s)Q(s)GKTSSGIVK - 994.986099 - Charge:2

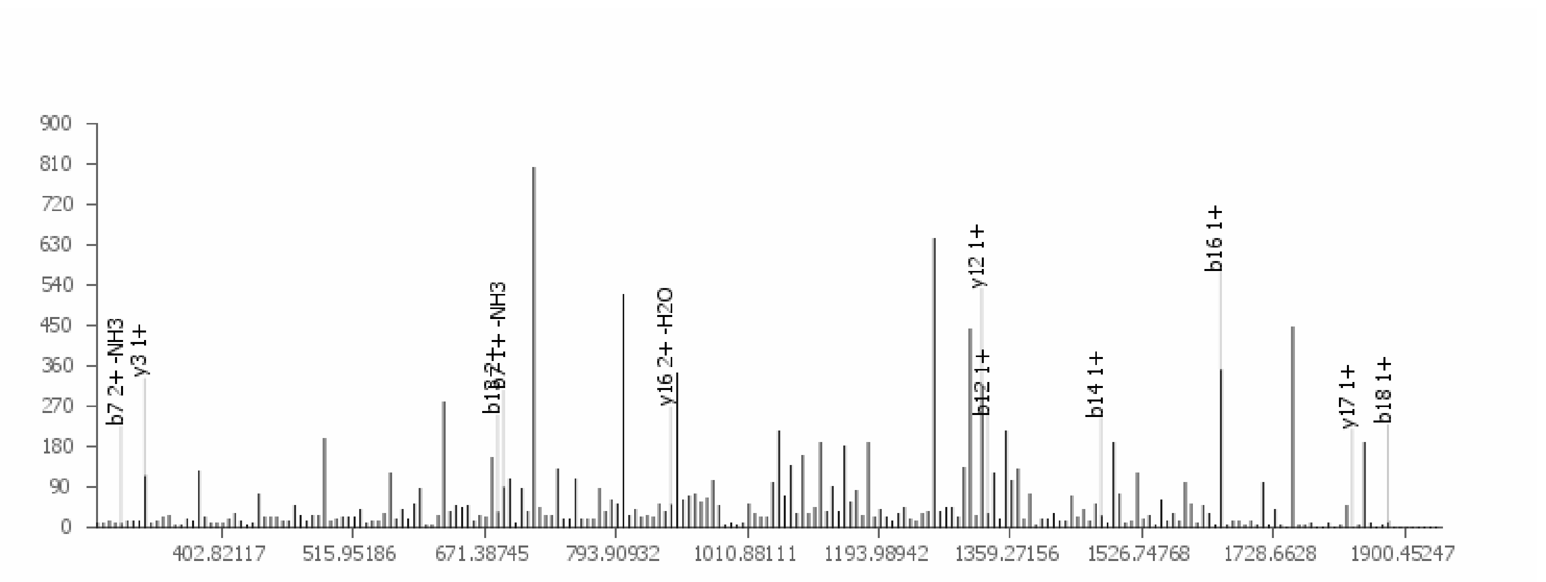

AT1G59620.1 - LR(pS)LLFIEELGG(pY)R - 923.413873 - Charge:2

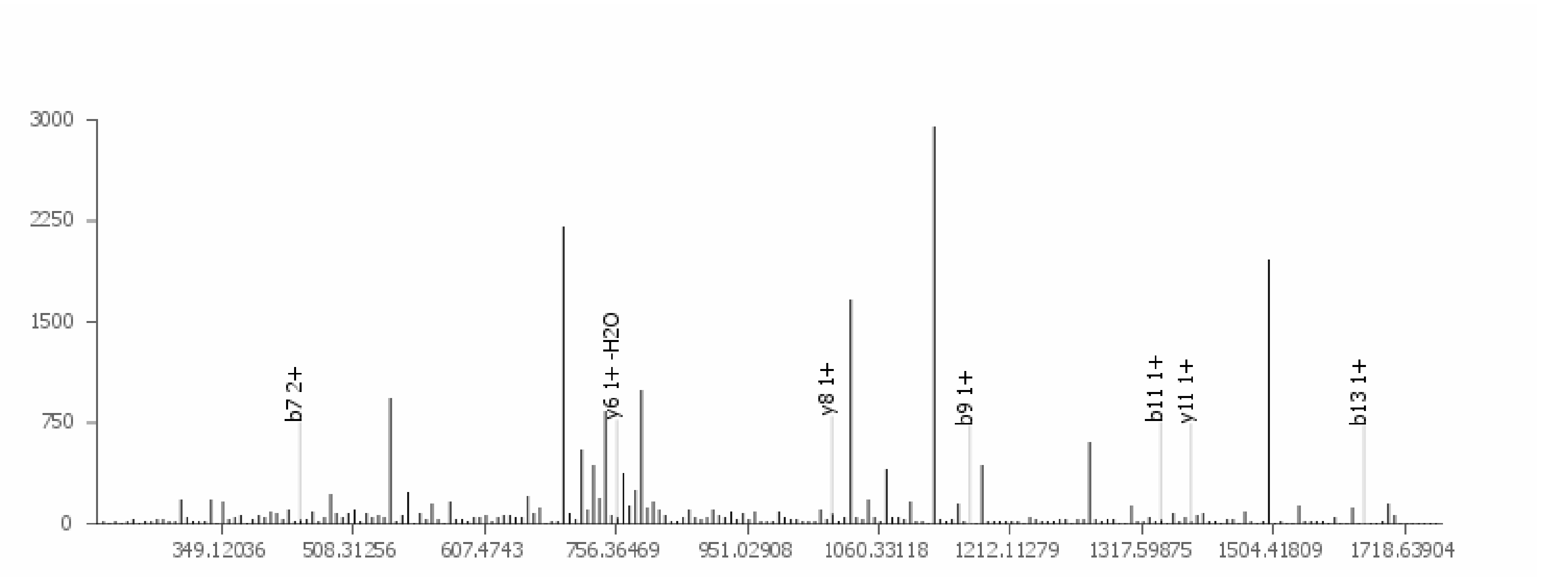

AT2G24190.1 - GIL(pS)DAENLTEVR - 748.85531 - Charge:2

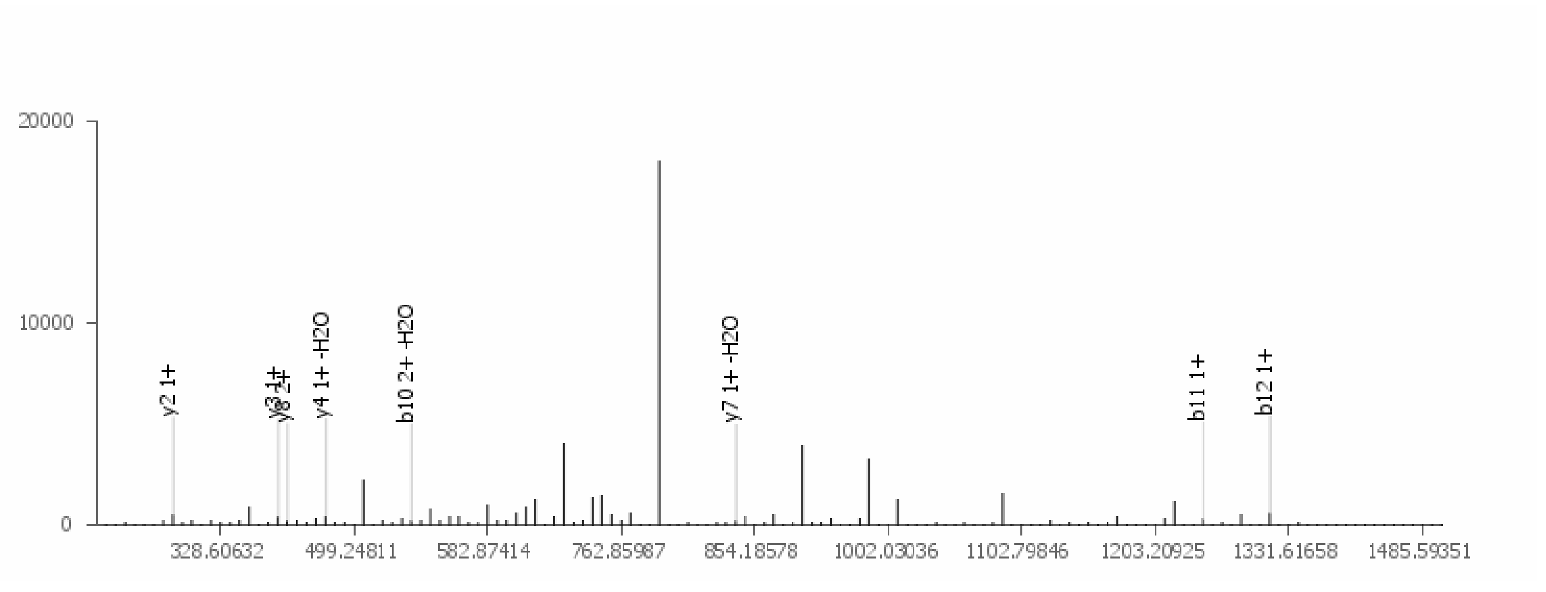

AT1G12700.1 - SGD,(pT,)SLALDLLRK - 743.354862 - Charge:2

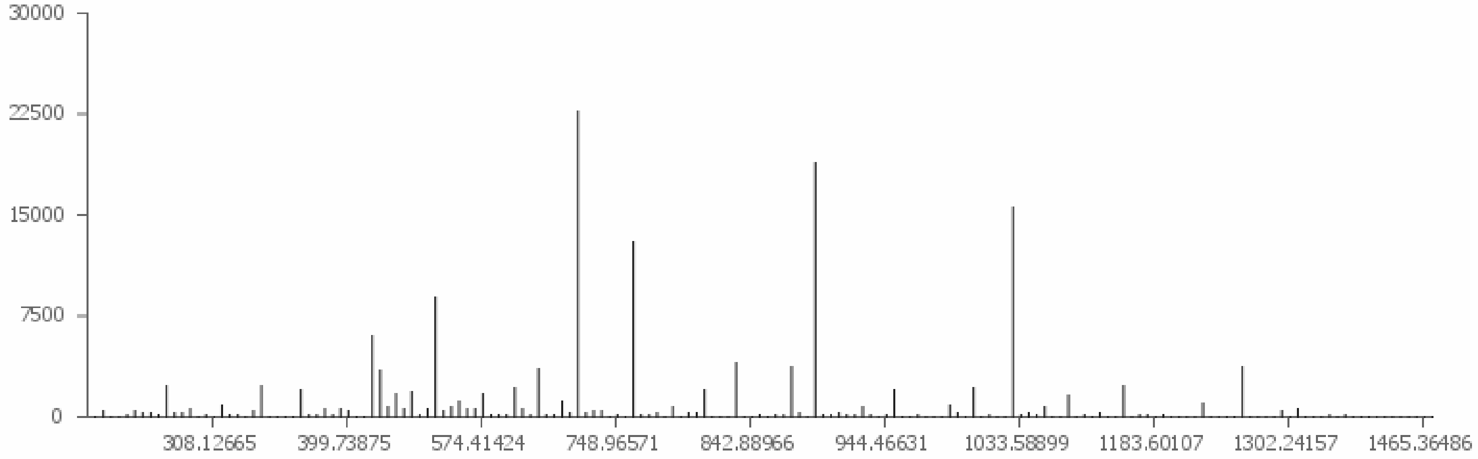

AT2G24340.1 - NLYLSPTMSIYDTLIG(pS)PATTLKR - 912.463192 - Charge:3

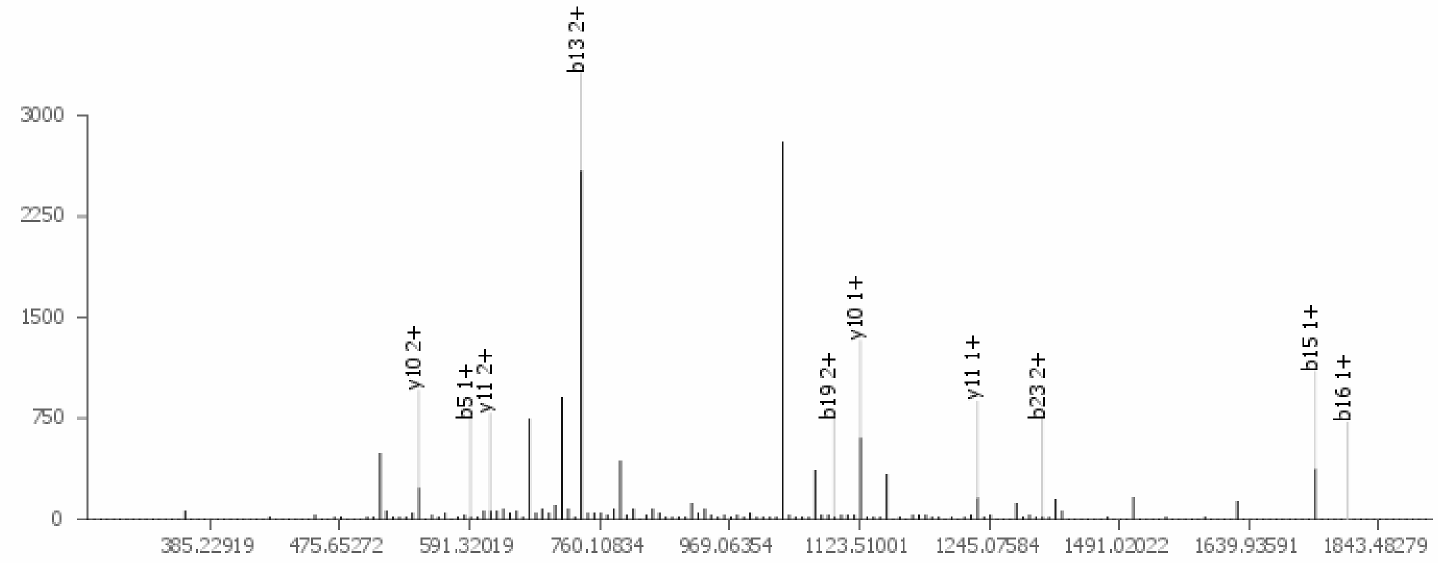

AT3G26480.1 - VLV(pT)AVALSRNGSVMTTAEAR - 1113.570889 - Charge:2

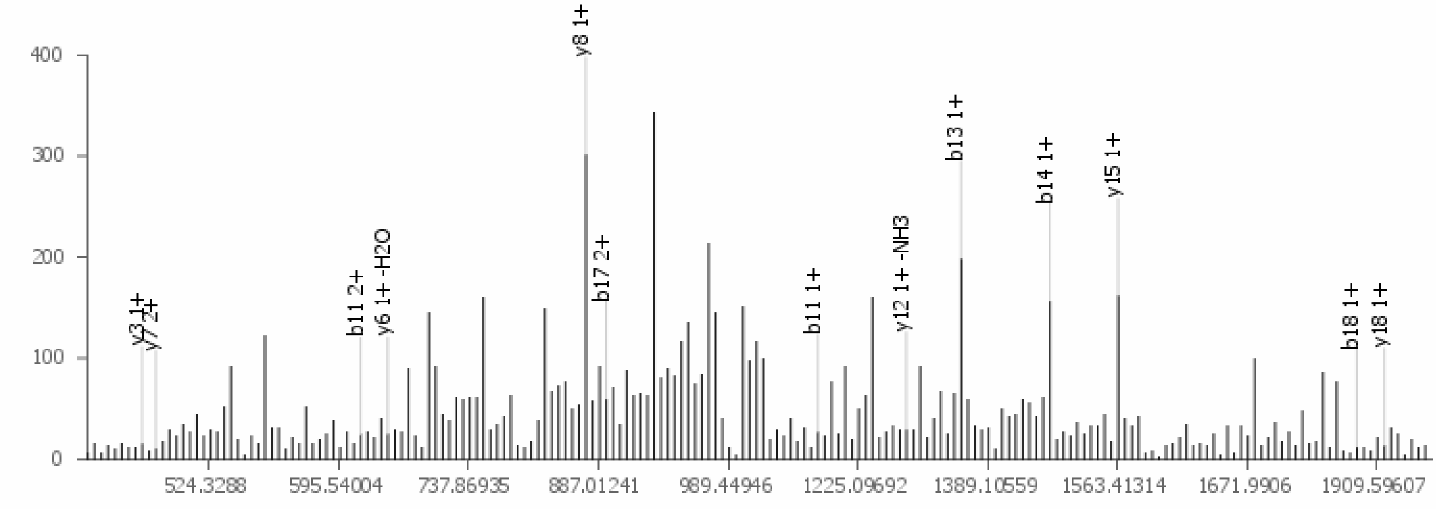

AT4G37510.1 - AA(pS)LQNLIFPYDD(oxM)DKLIR - 1160.069546 - Charge:2

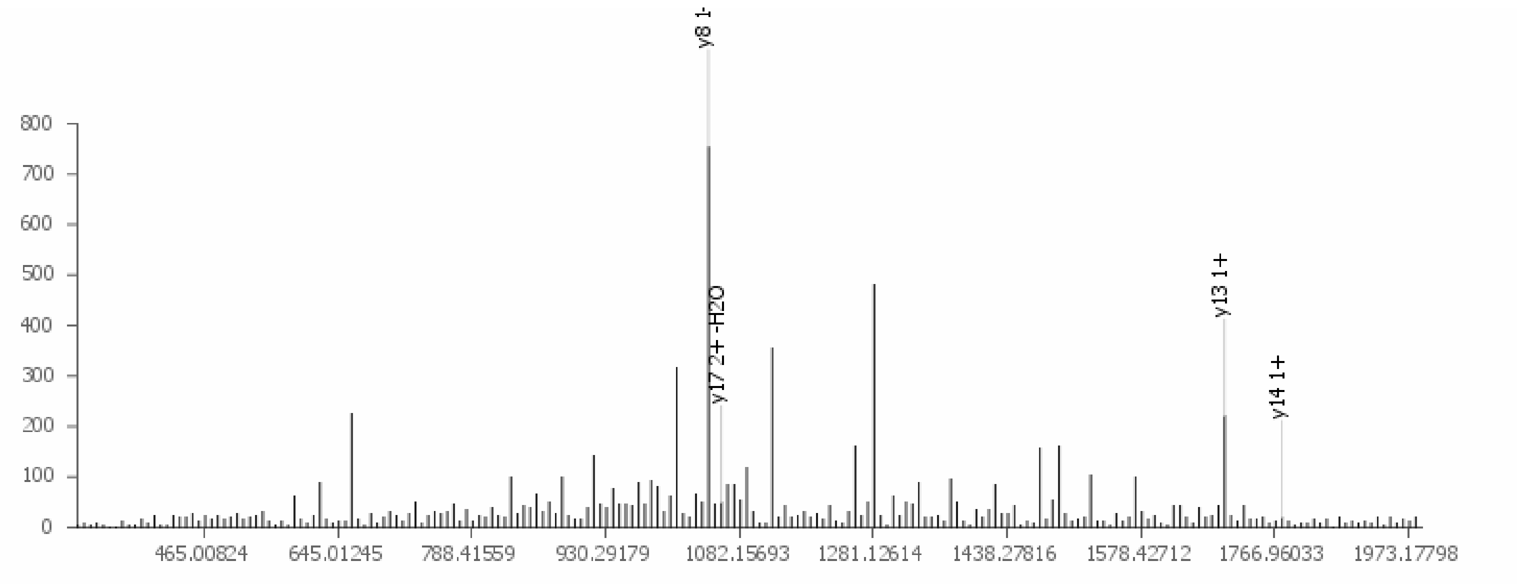

AT5G19100.1 - IFA(s)(t)PLIGNGK - 649.328758 - Charge:2

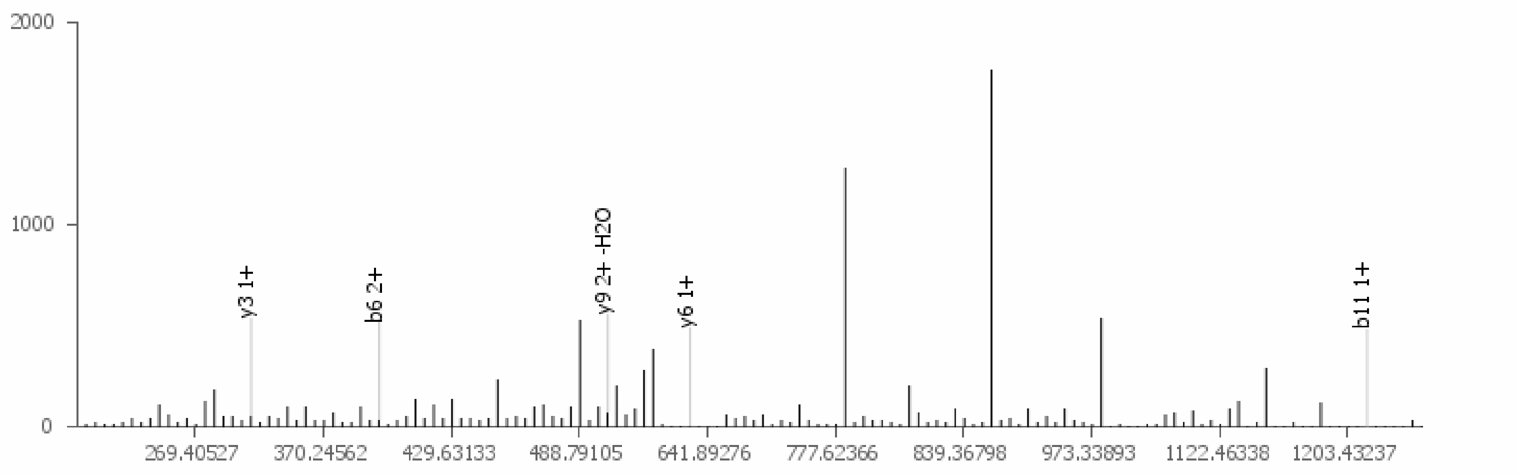

AT3G48425.1 - FGA(pT)IKEGR - 529.757237 - Charge:2

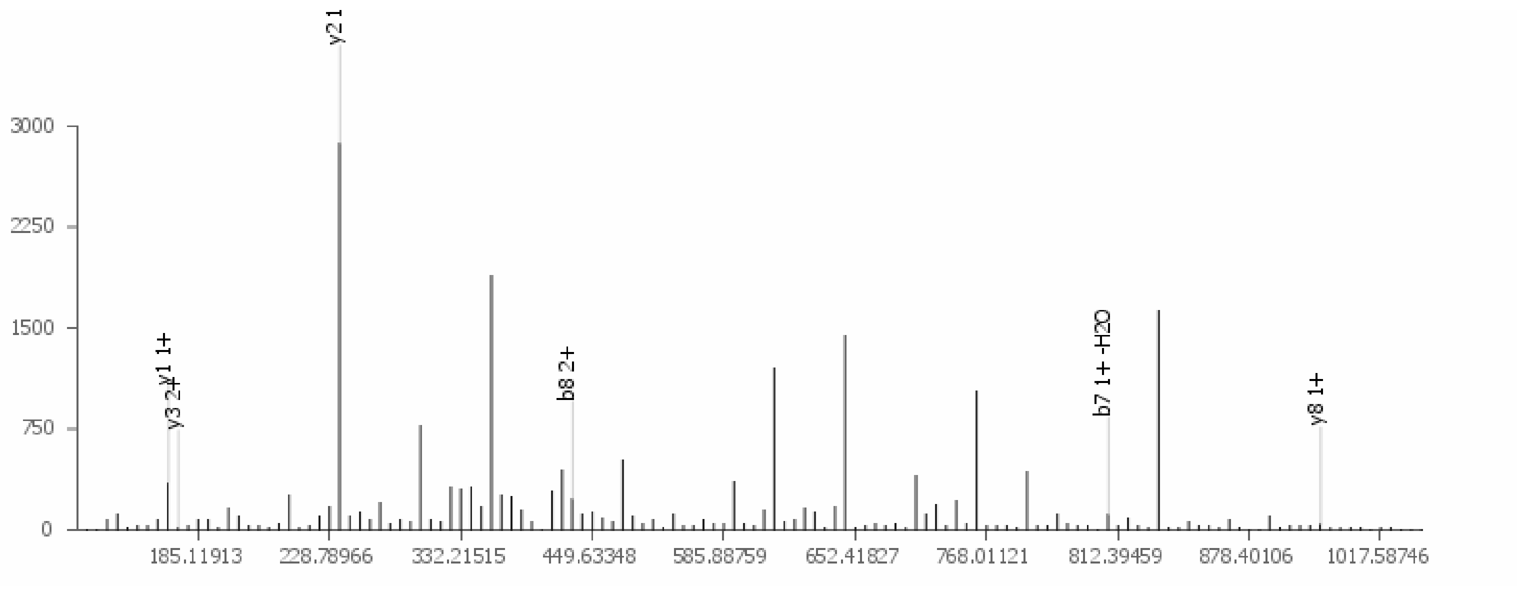

AT1G65930.1 - DKLI(pT)PFVELDIK - 805.930119 - Charge:2

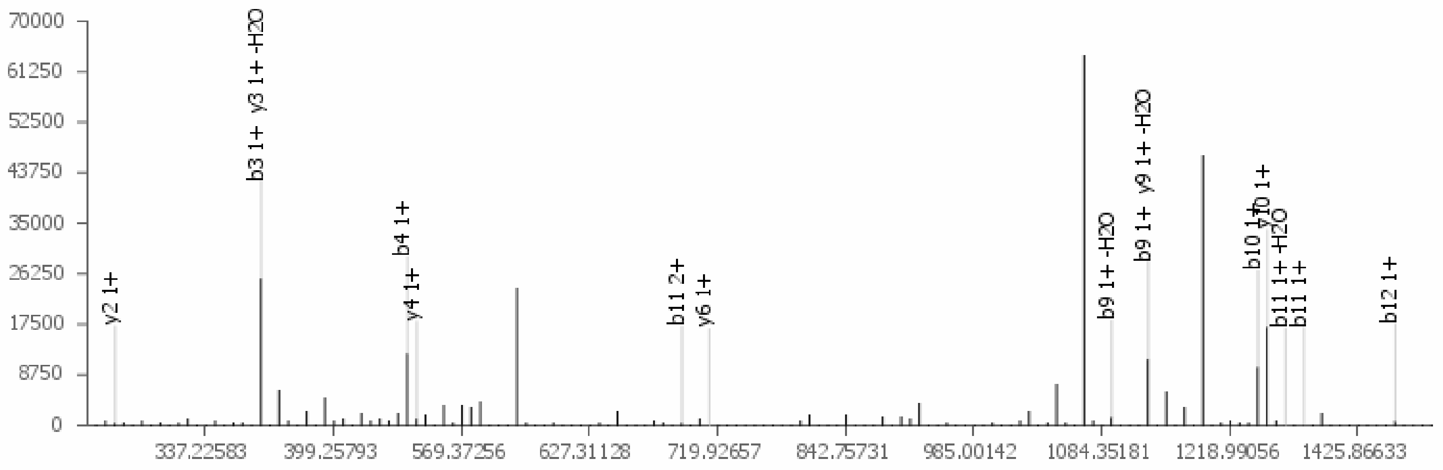

AT5G47040.1 - DGP(pS)AGVTLVTALVSLFSQKR - 1113.574697 - Charge:2

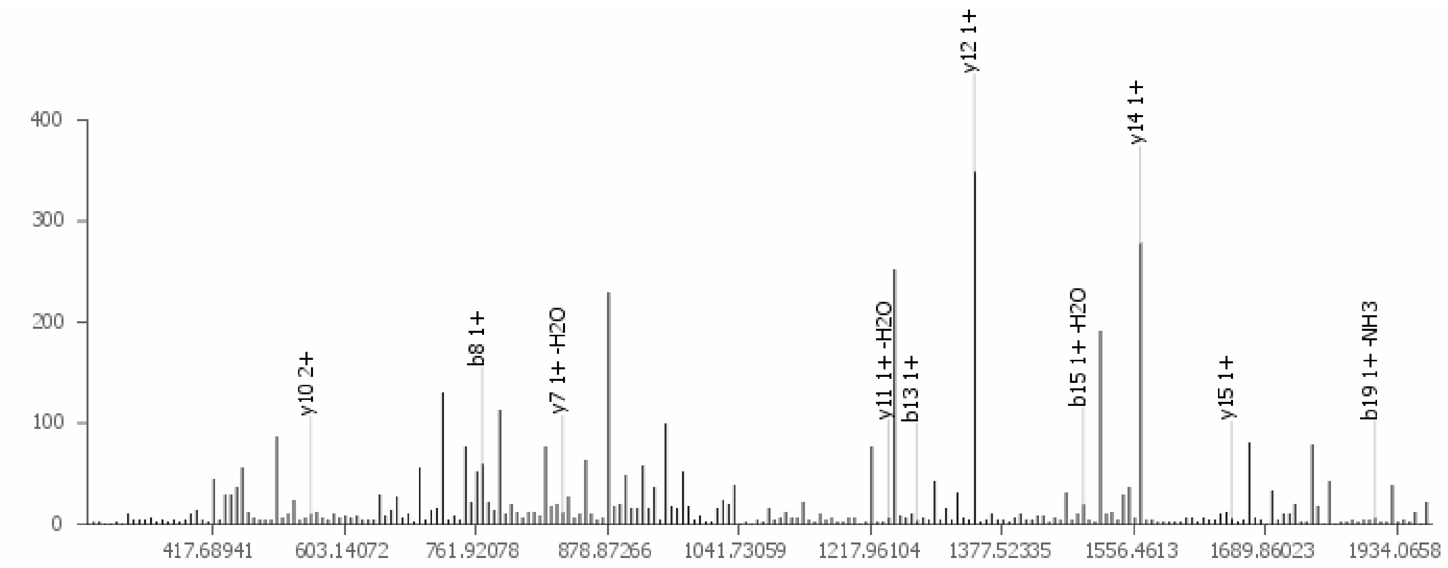

AT1G77460.1 - IVNA(pS)(pS)LEVRVGSTALLLCAAK - 1188.102304 - Charge:2

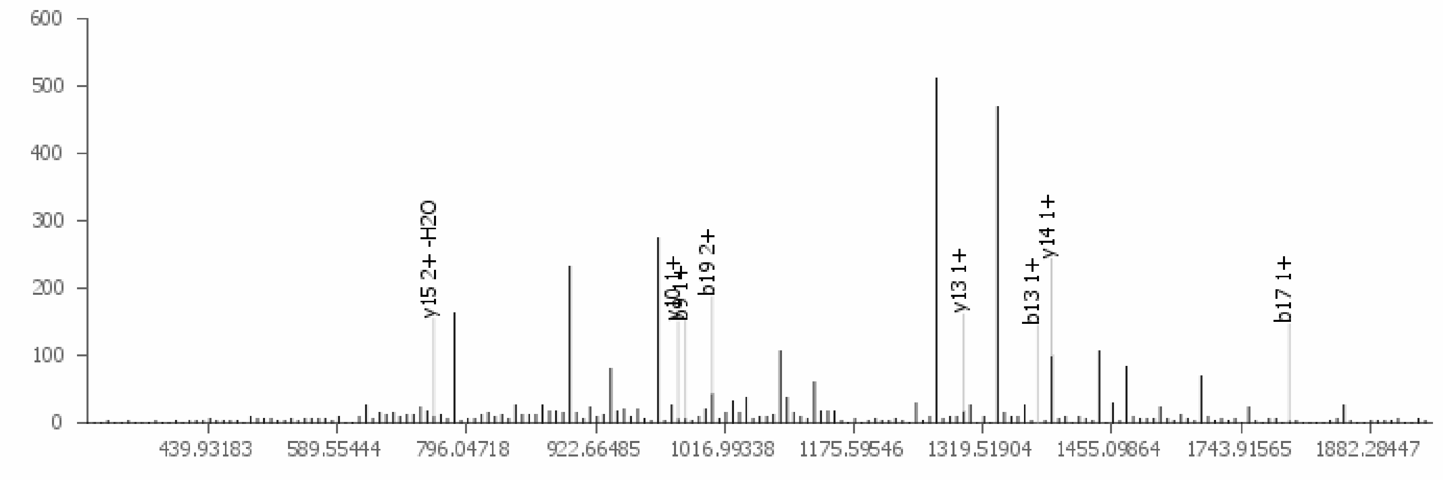

AT5G08650.1 - IPAPLD(pT)AGKPLR - 714.8912 - Charge:2

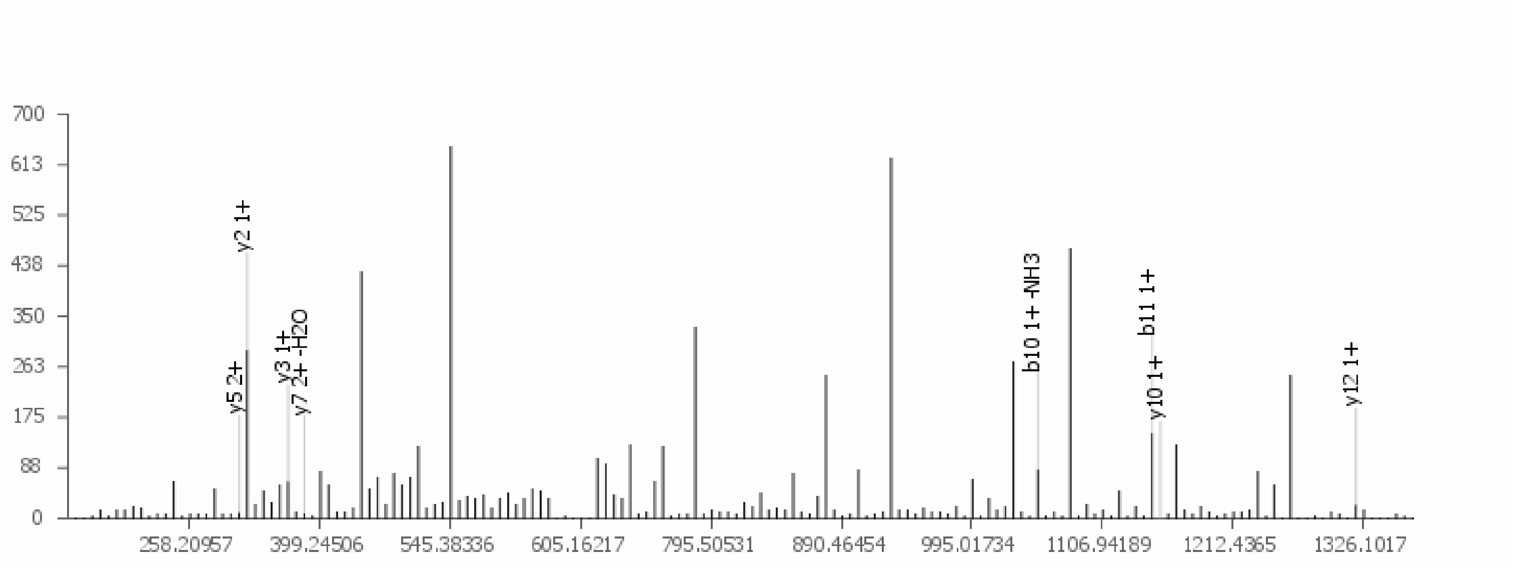

AT1G58370.1 - LKLFL(pT)YQISVWVK - 909.493556 - Charge:2

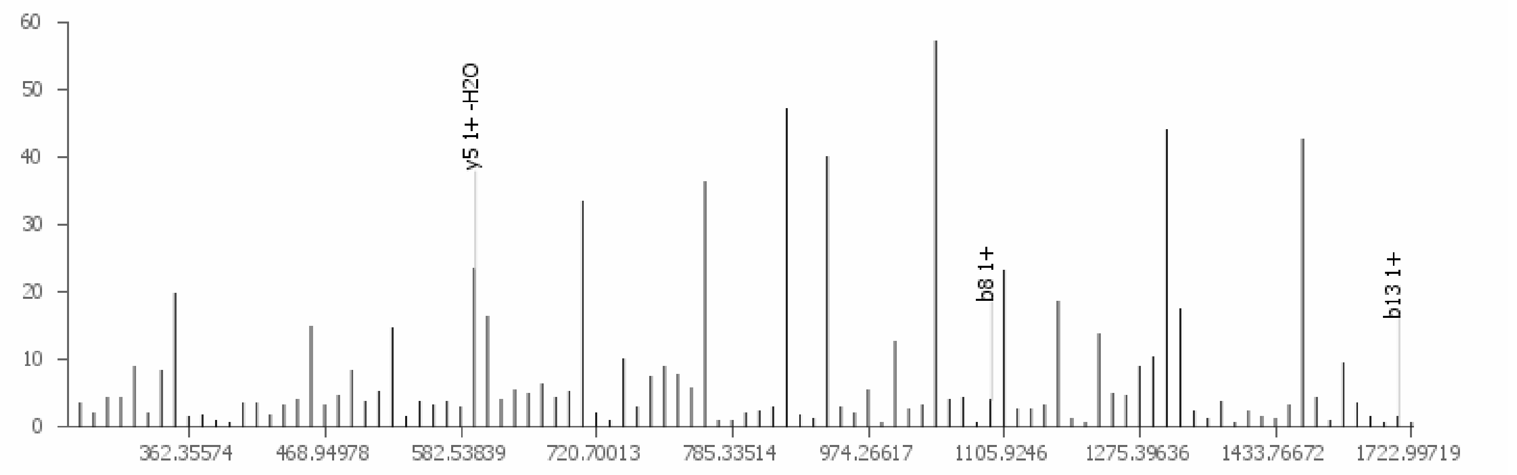

AT2G34350.1 - ICSF(pT)FLLLLLA(pS)PLLVAVR - 1175.121729 - Charge:2

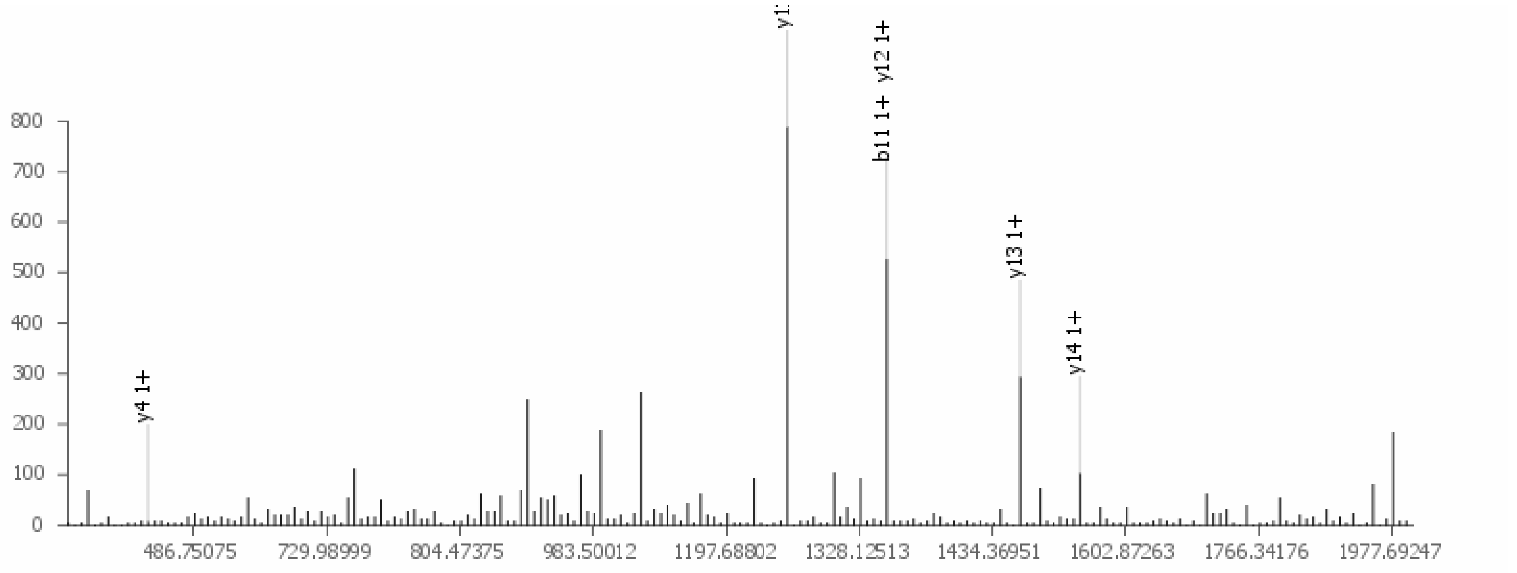

AT3G57410.1 - TS(pS)PSRDR - 493.205856 - Charge:2

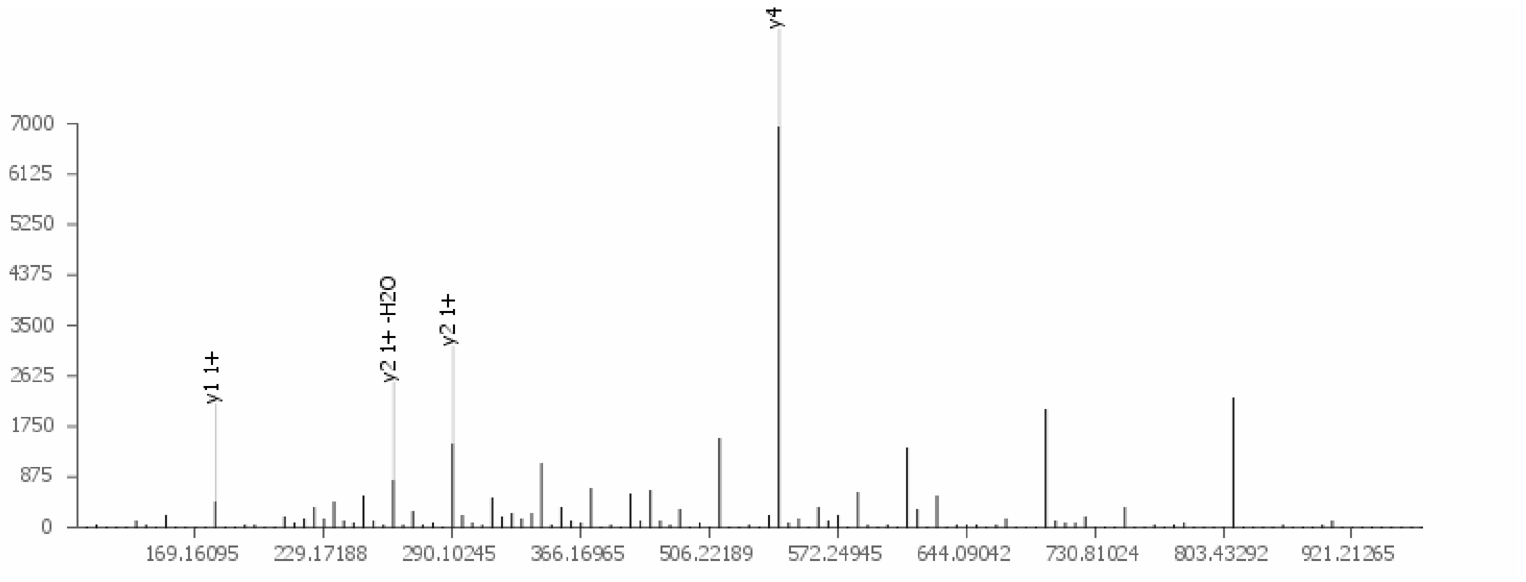

AT1G04840.1 - DMHMATTLFR(pS)(oxM)PER - 959.897138 - Charge:2

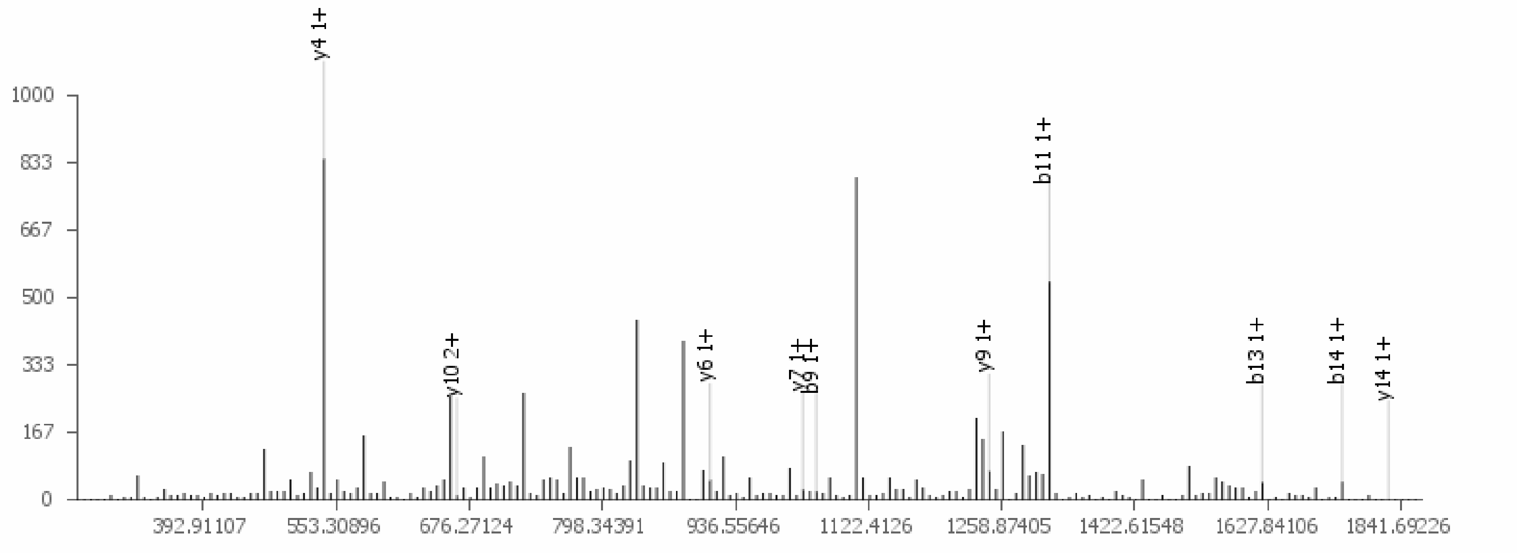

AT5G54600.1 - IF(pT)HNSTIVIK - 676.851643 - Charge:2

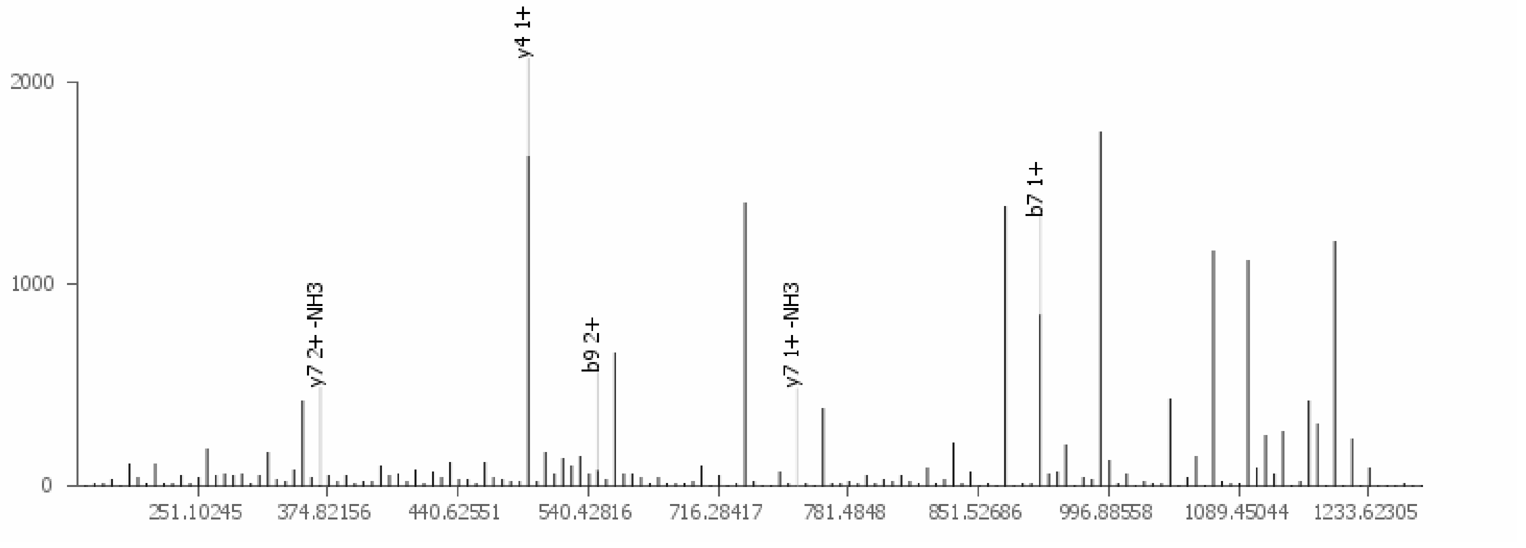

AT2G11520.1 - LIITE(pY)VRNGTLR - 814.438471 - Charge:2

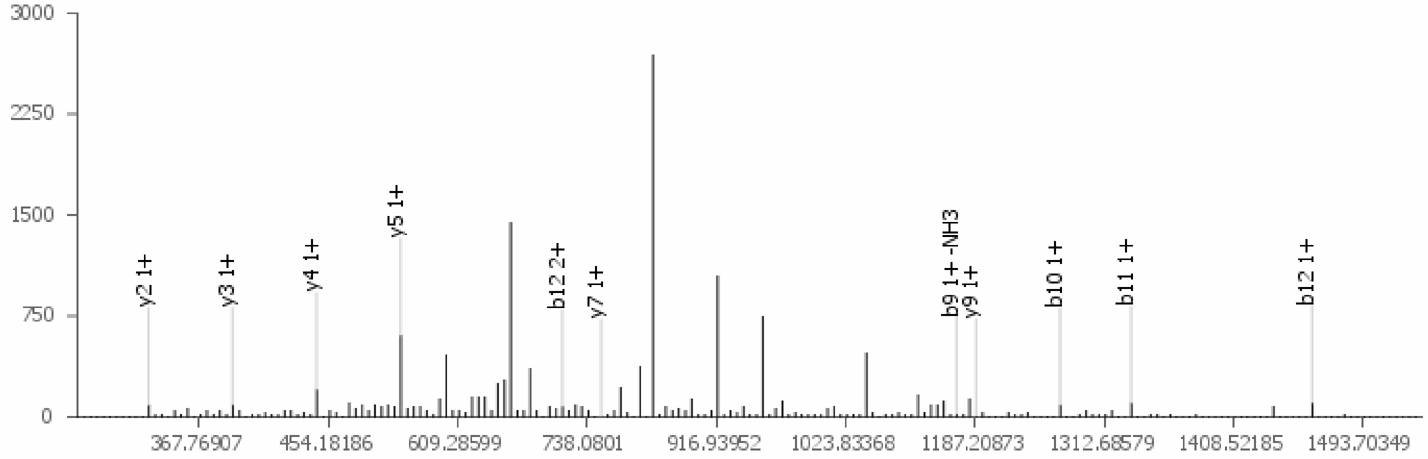

AT2G11626.1 - K(pT)DFHWSLFNPSSPFPLNLK - 819.062045 - Charge:3

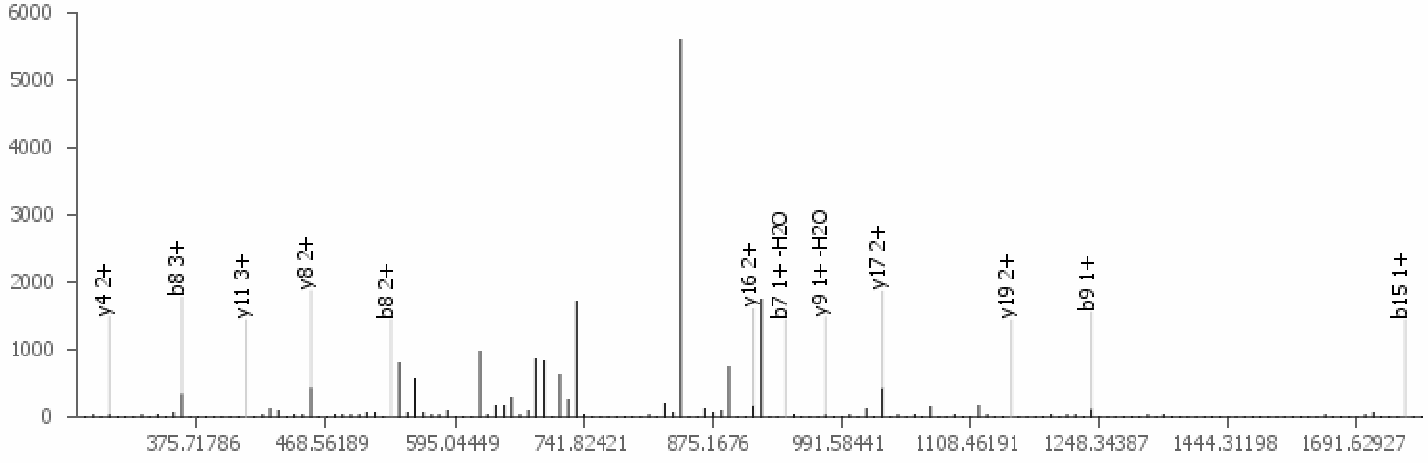

AT5G14060.1 - MA(pS)LQLYGVKTPGLALSSK - 1022.529067 - Charge:2

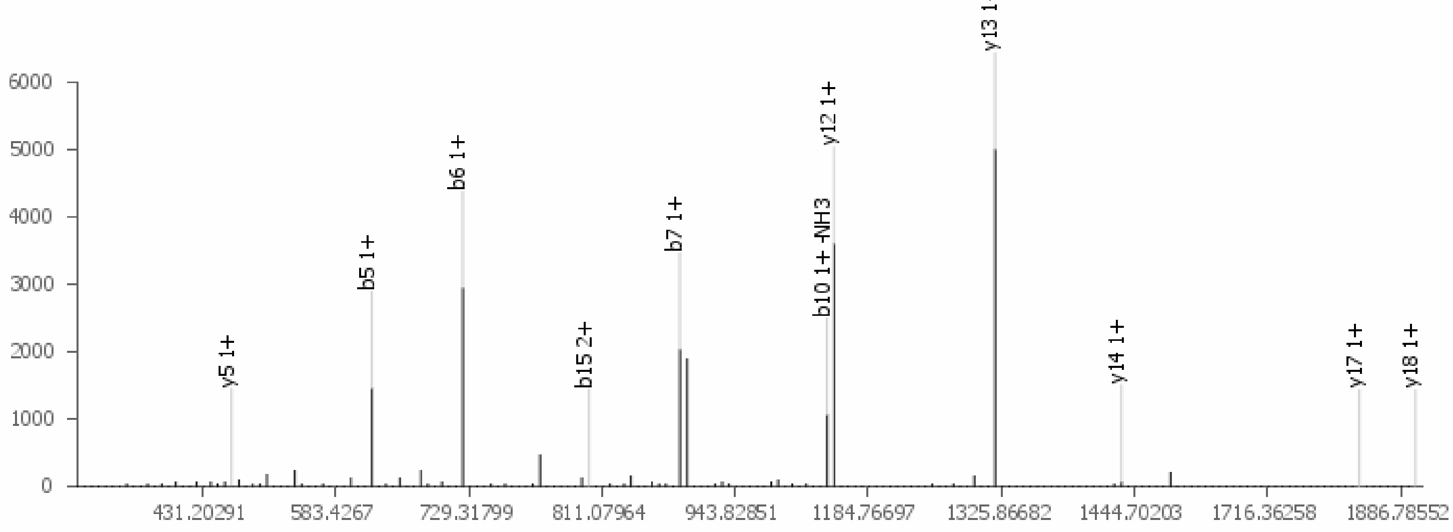

AT5G66610.1 - IVEE(pS)LKEK - 577.793462 - Charge:2

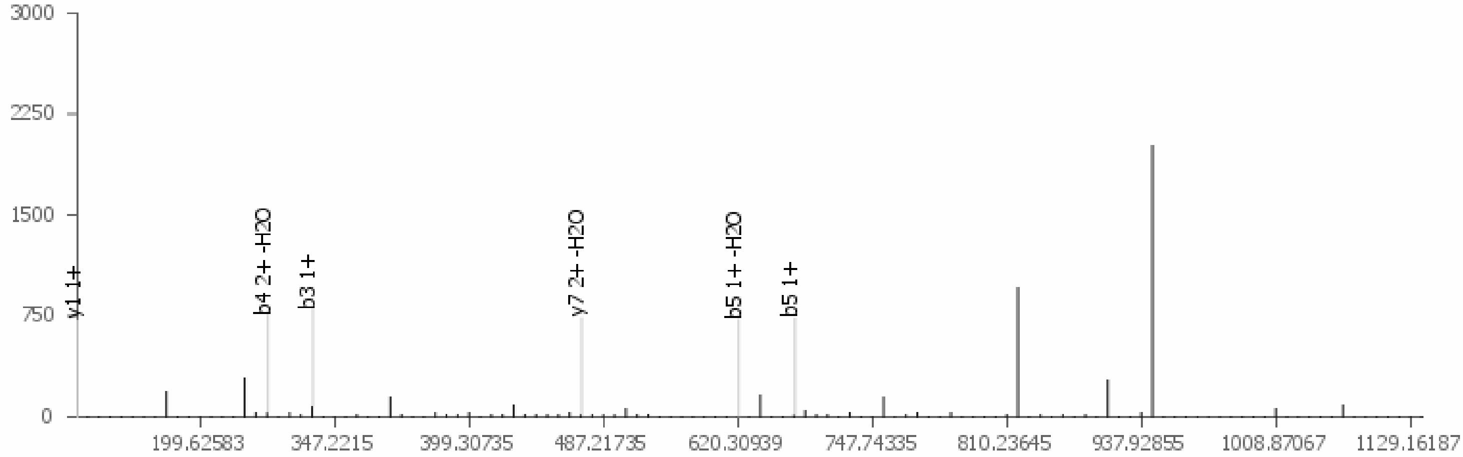

AT1G64610.1 - TATDDDSEDELEPSHTDSNVV(pS)EVVNV(s)(s)GVK - 1174.483567 - Charge:3

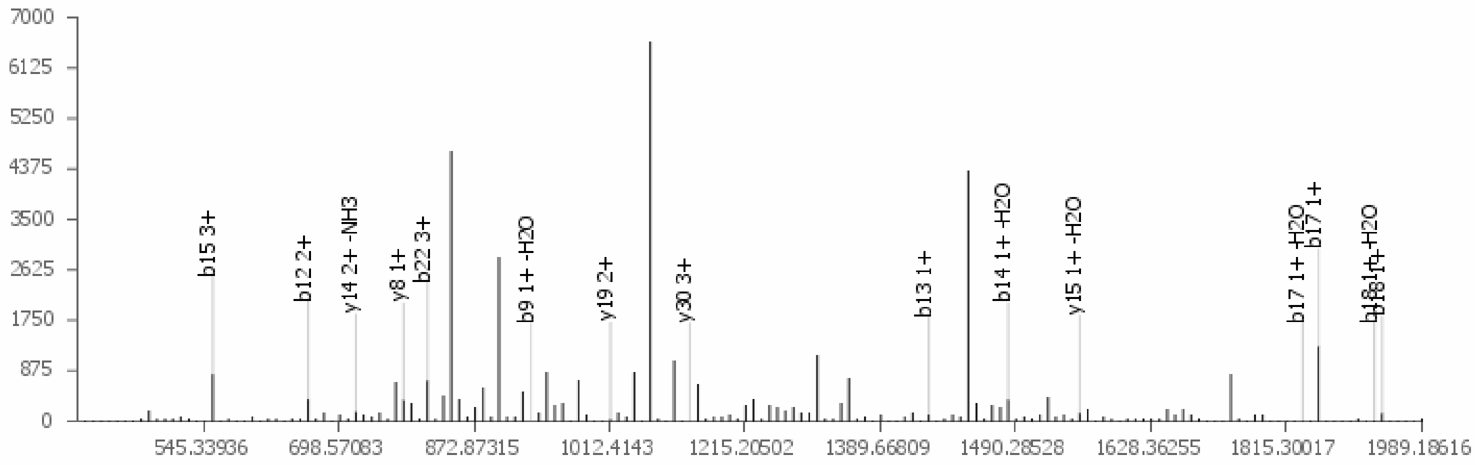

AT1G67290.1 - (pY)GQ(oxM)FDVKIELK - 783.874142 - Charge:2

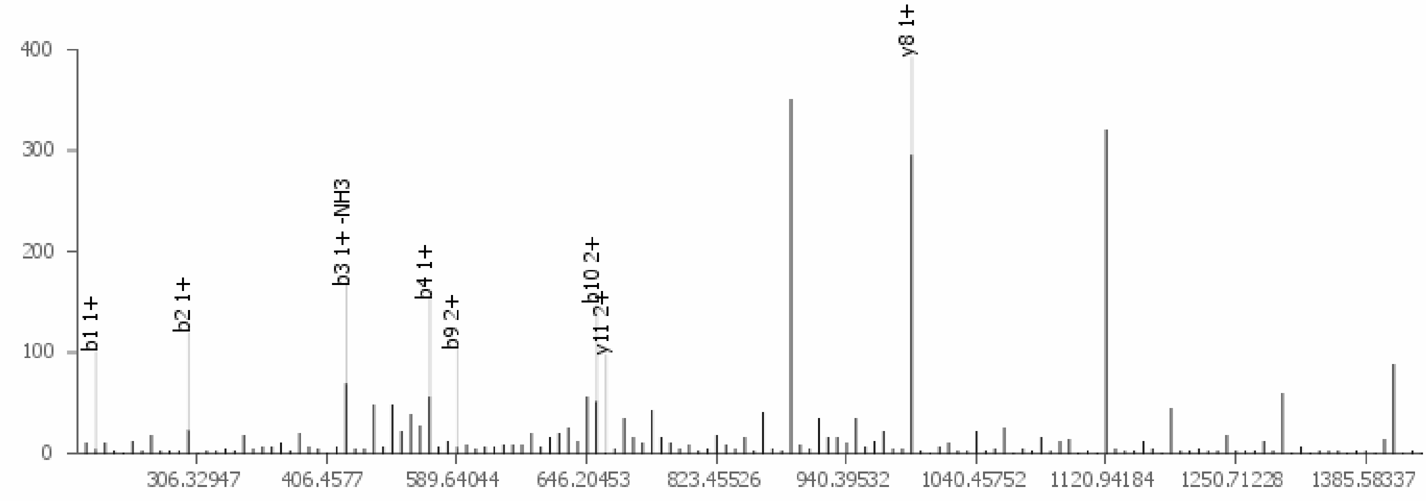

AT2G06140.1 - IDDIKV(pT)LFSK - 679.847881 - Charge:2

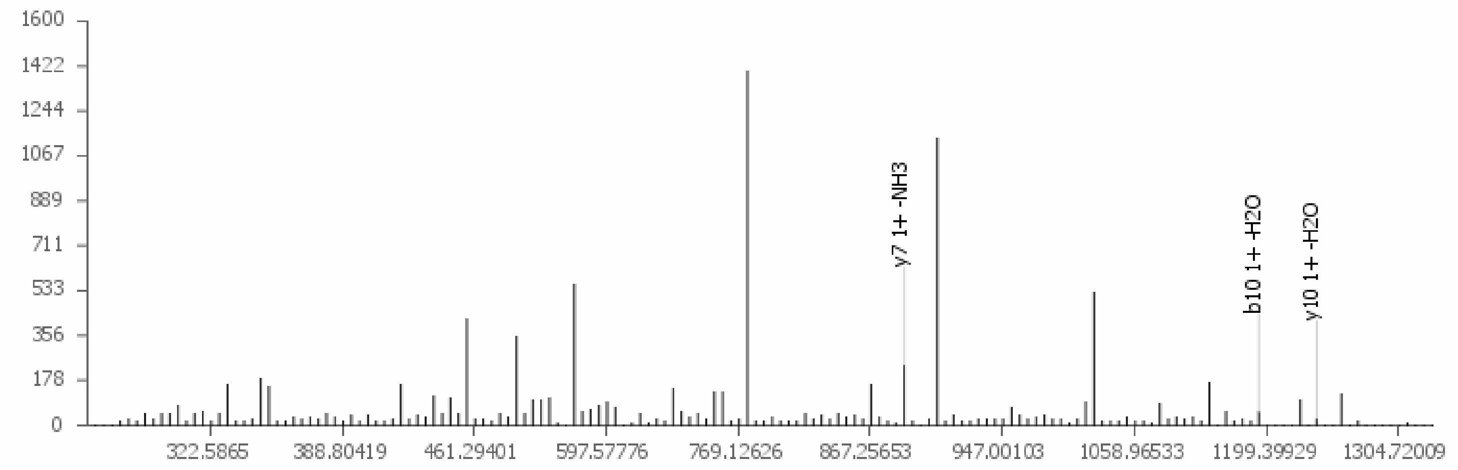

AT5G01150.1 - S(pT)ELVLNK - 492.243155 - Charge:2

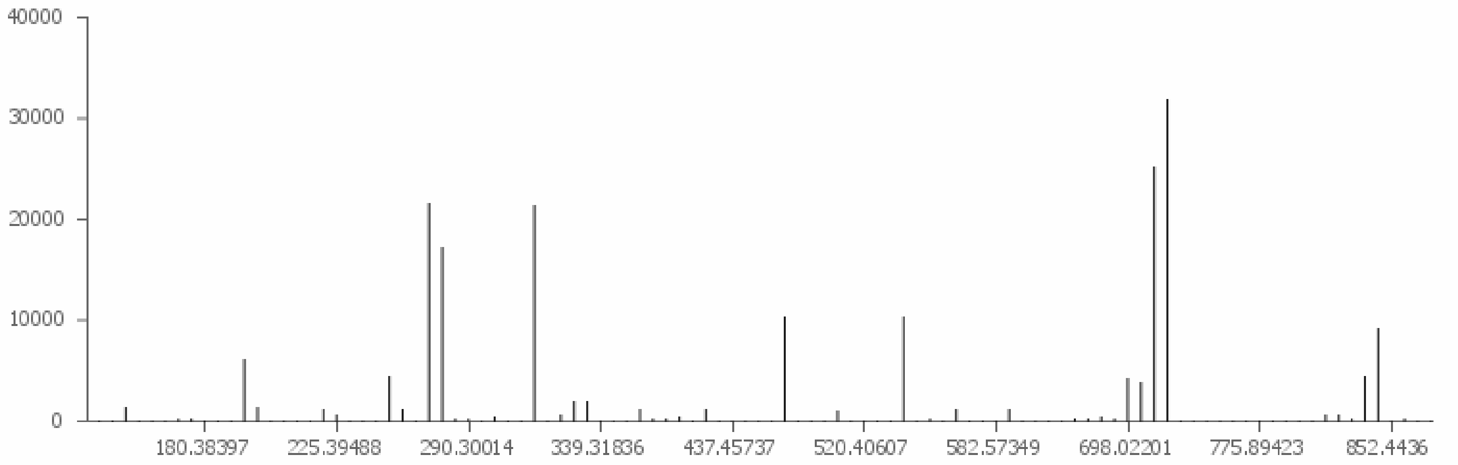

AT3G54230.1 - LASKTSLLA(pS)K - 599.824194 - Charge:2

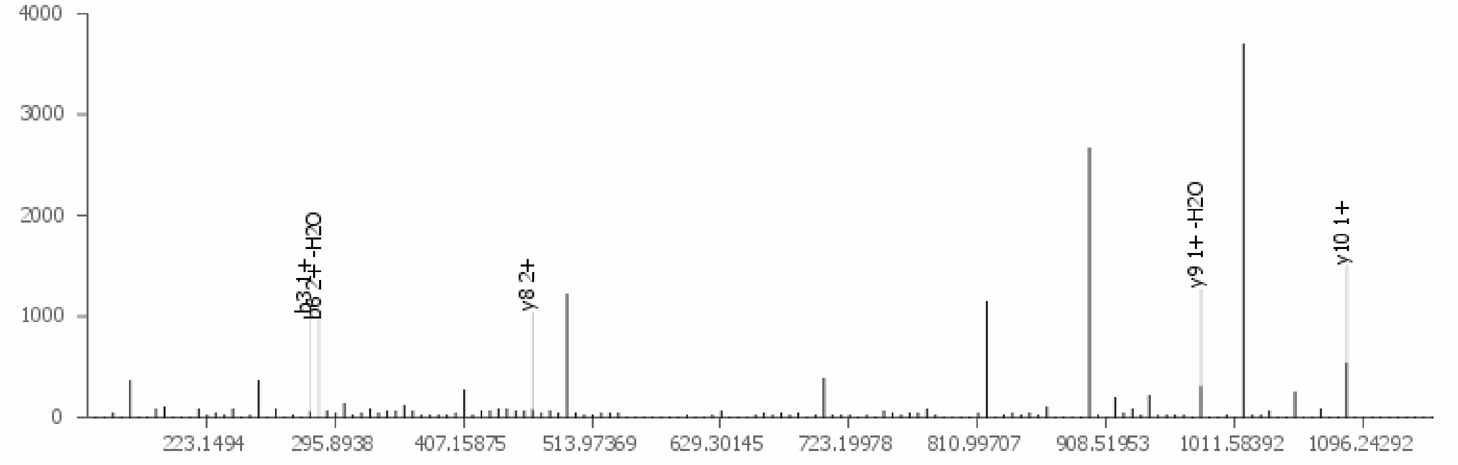

AT4G18205.1 - VQ(pY)VIGFICTIGASAGIGLVL(pS)LIQLLFR - 1075.576337 - Charge:3

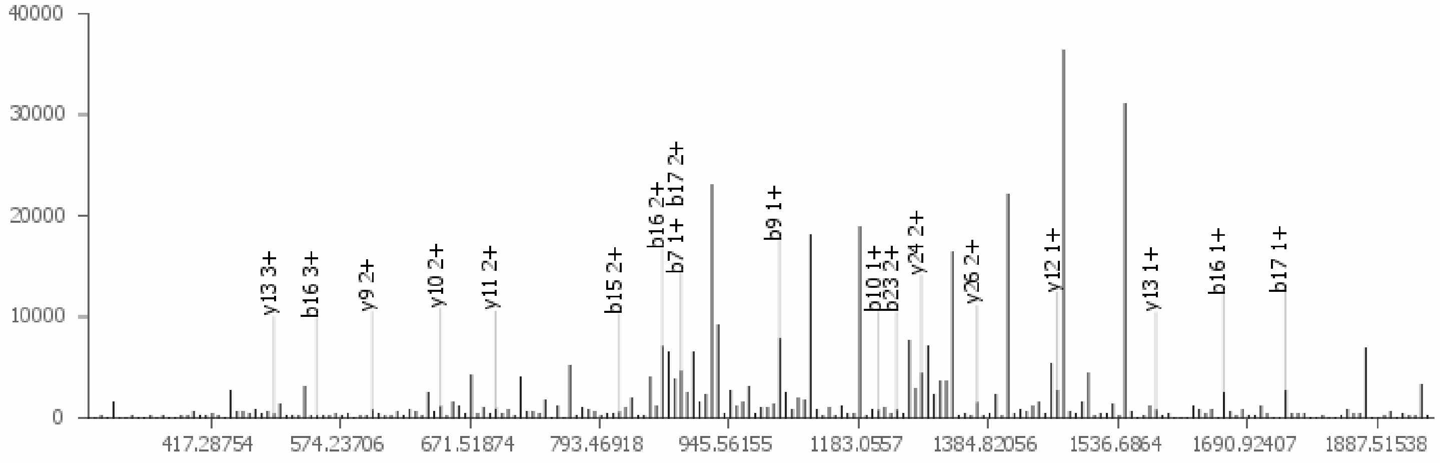

AT3G19710.1 - DN(pS)GLNLKIEDQFR - 864.897103 - Charge:2

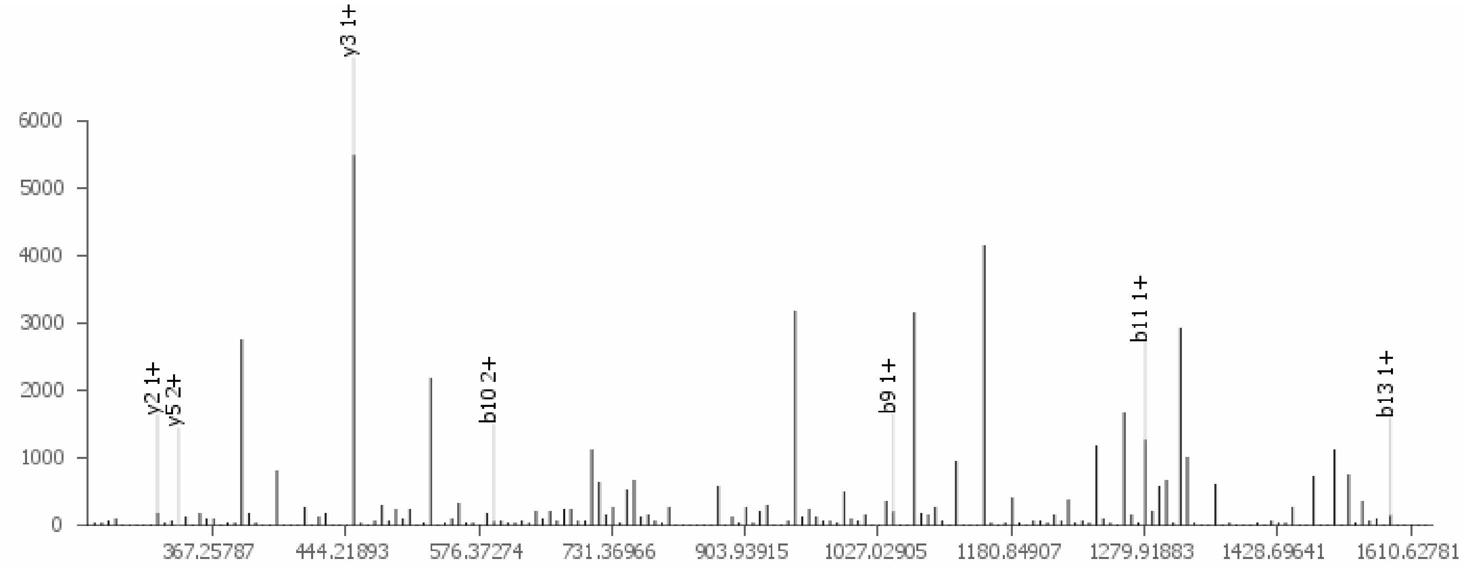

AT5G27970.1 - AEV(pS)KISITTL(oxM)AR - 539.271456 - Charge:3

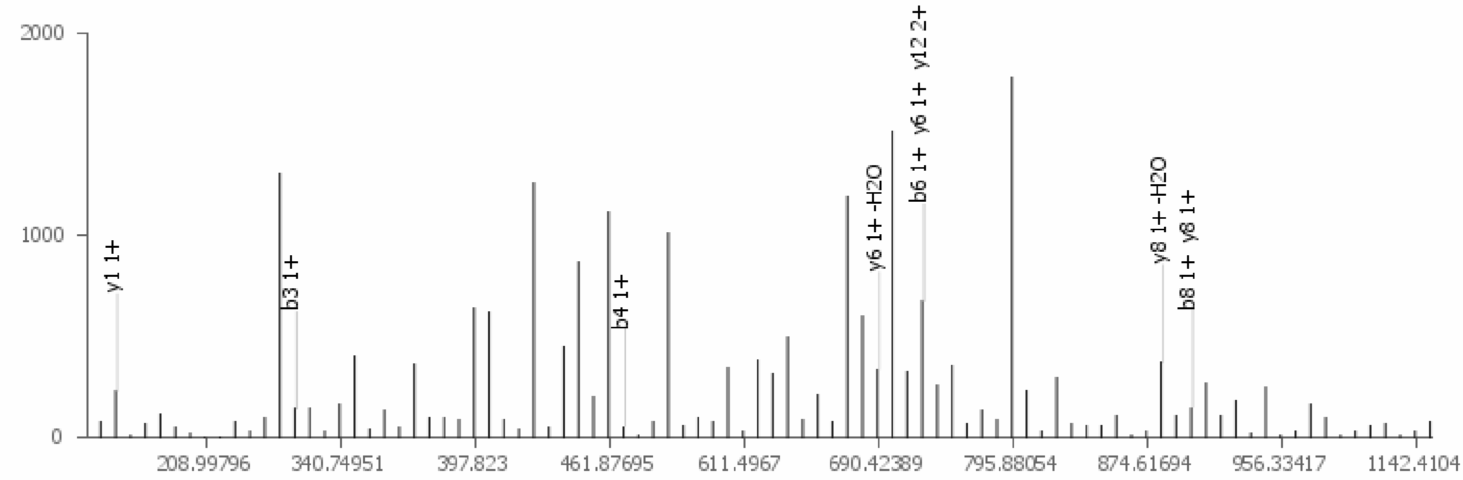

AT1G06980.1 - GSI(pY)FLIPDSSLPEKK - 937.479823 - Charge:2

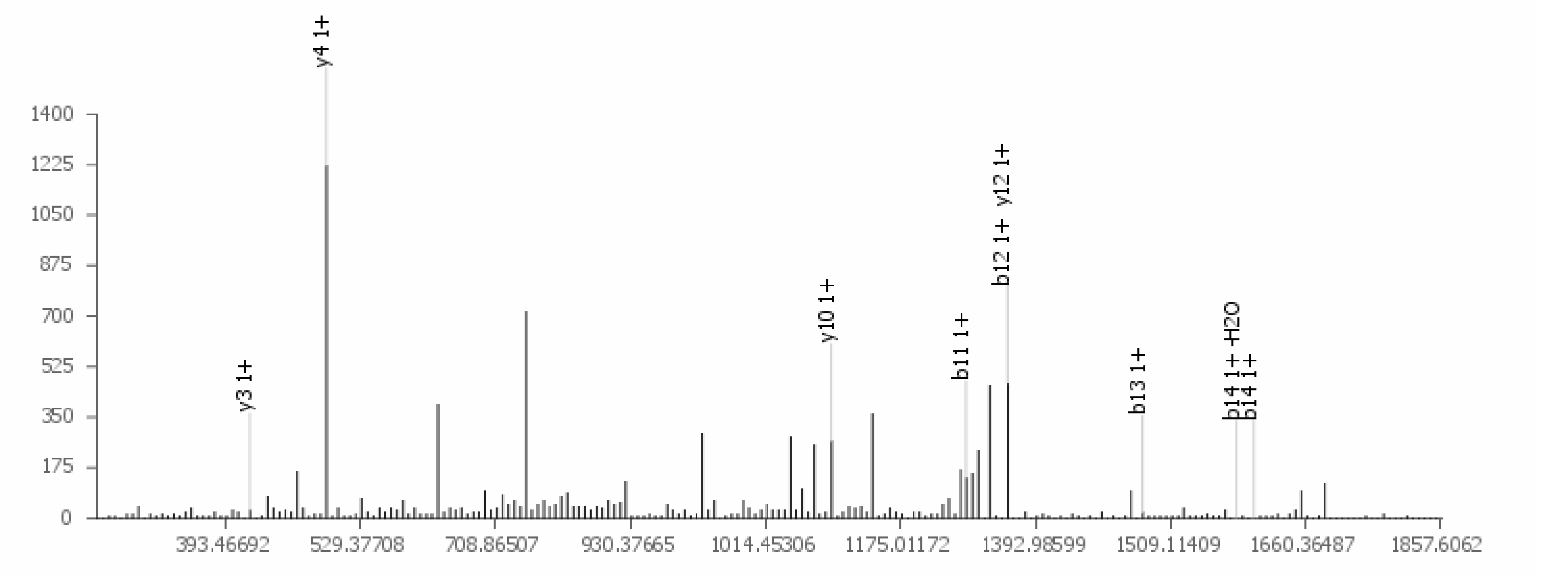

AT1G23740.1 - GPFPF(pS)RVADAFSYLETNHATGK - 1296.606922 - Charge:2

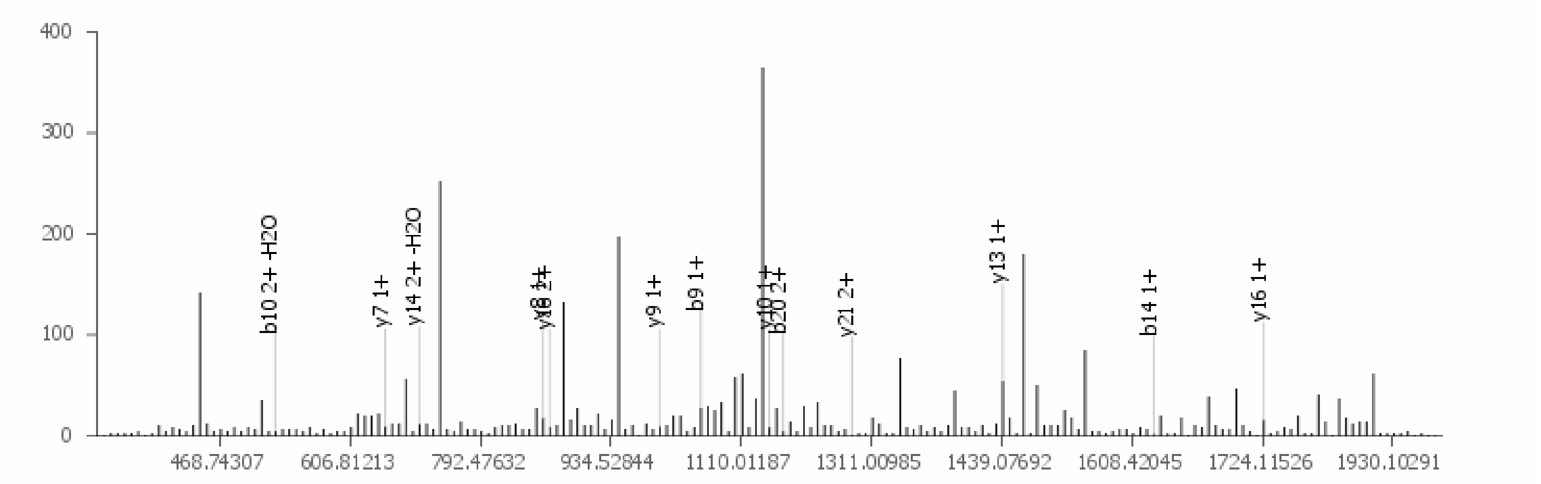

AT1G66440.1 - YKHDNHVLIL(pS)YGK - 883.933111 - Charge:2

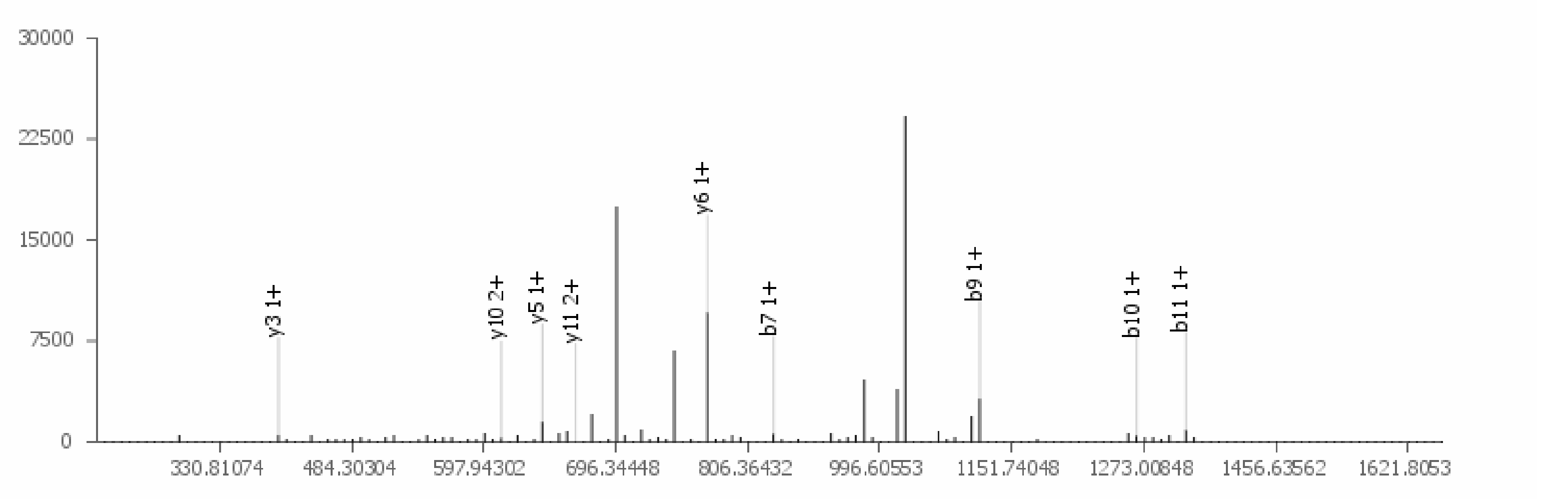

AT1G75370.1 - (oxM)FIINGGSGFKLVWA(pT)VK - 1032.519457 - Charge:2

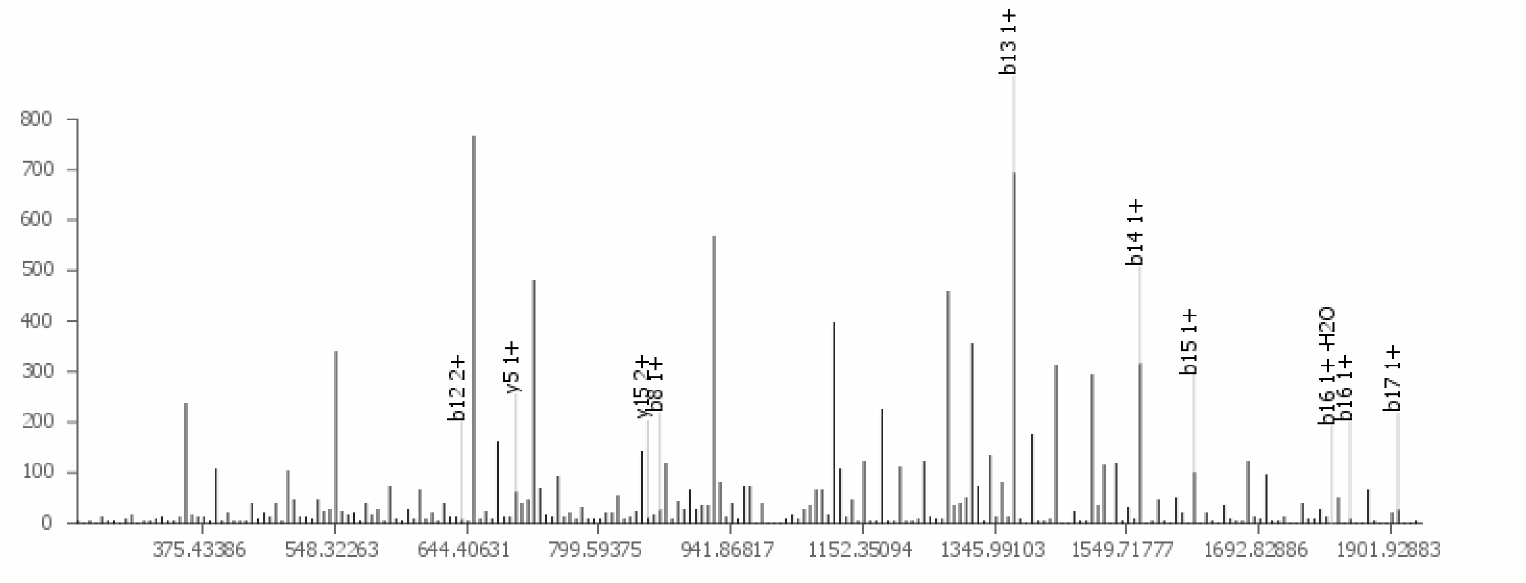

AT1G72500.1 - IPHVAGG(s)IF(s)VNVTWSQK - 1054.025233 - Charge:2

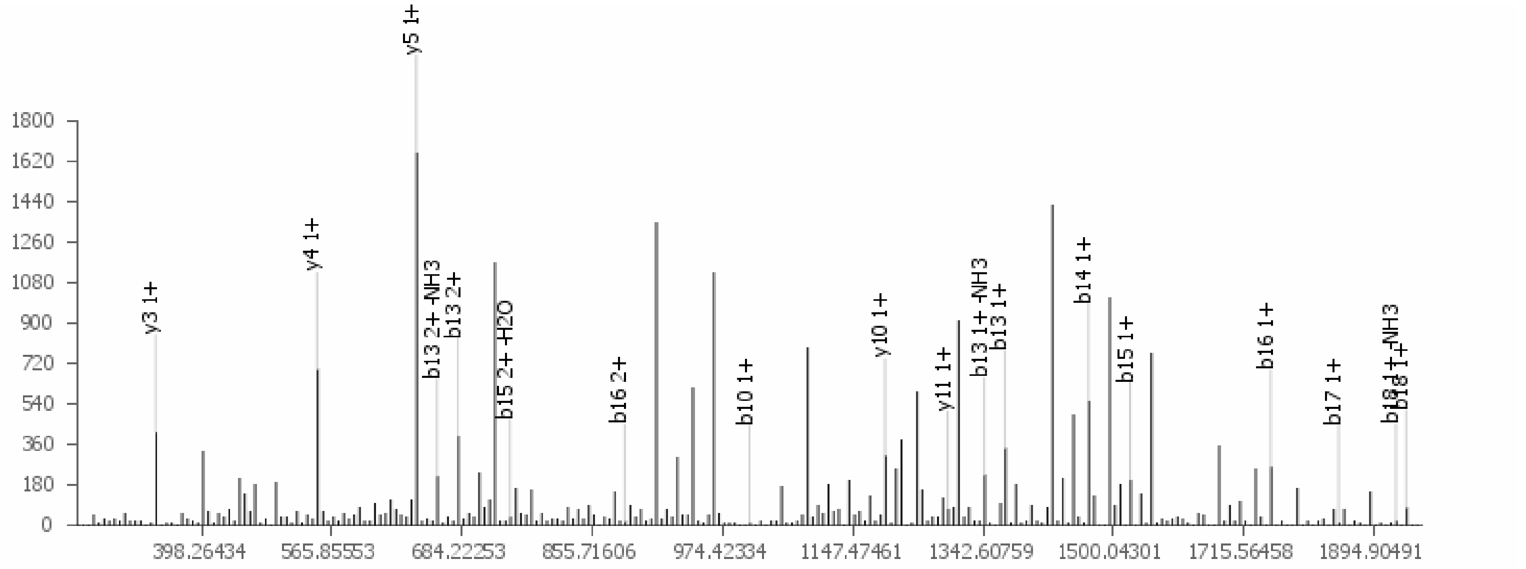

AT3G13670.1 - VQVGG(pS)PLYK - 564.275904 - Charge:2

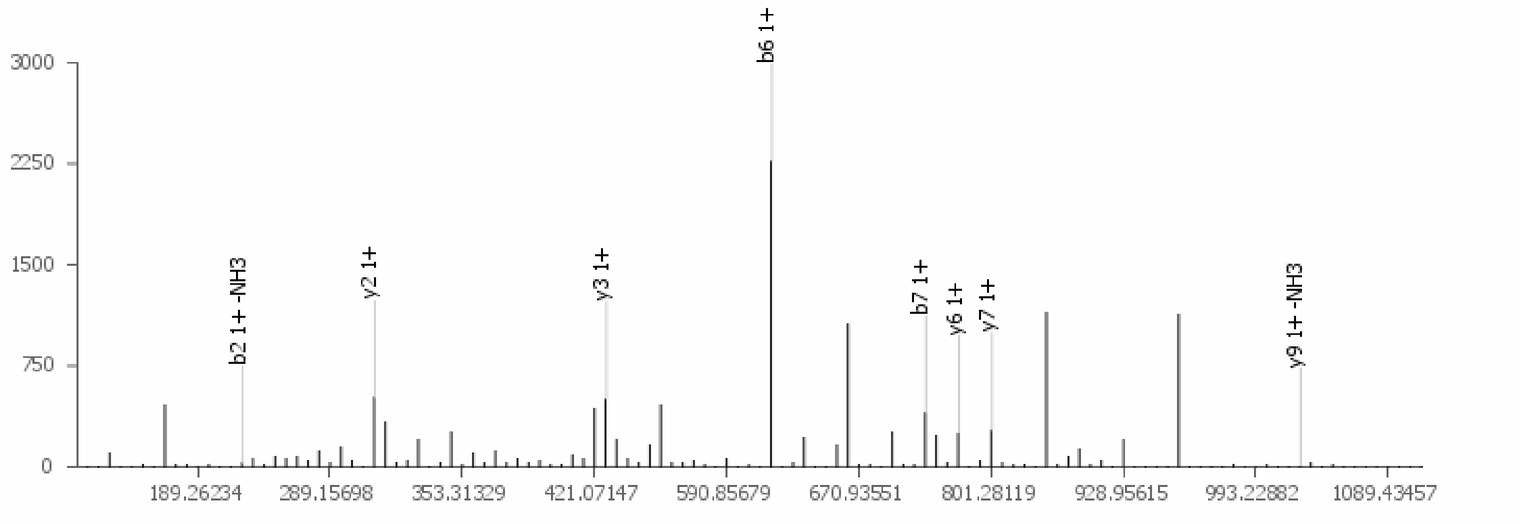

AT2G26780.1 - LESLVSNLLPLTISSL(pS)(pS)Q(pS)PVVR - 695.586582 - Charge:4

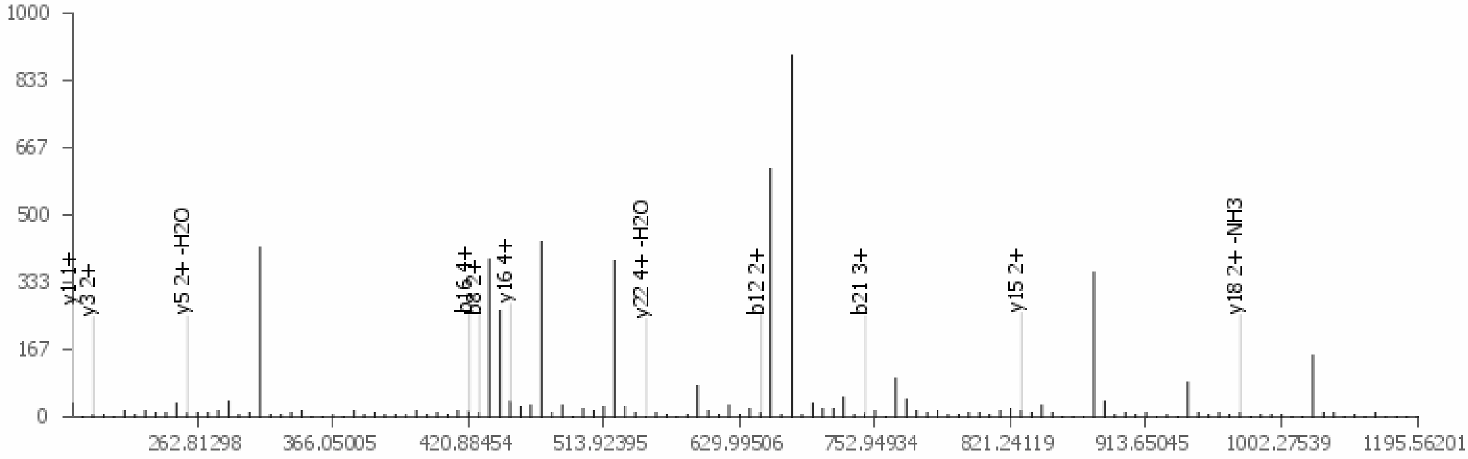

AT1G29900.1 - ELVDVEQYLMSGTLSEI(pT)K - 1118.024733 - Charge:2

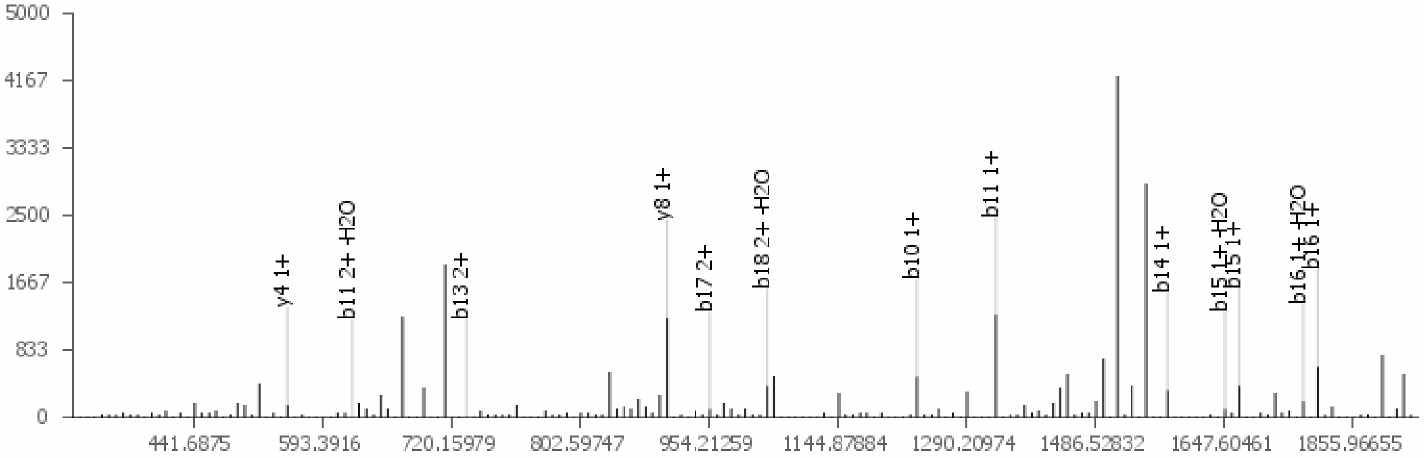

AT5G66800.1 - ISFSNDFVEIRPE(pT)(pT)K - 1021.950201 - Charge:2

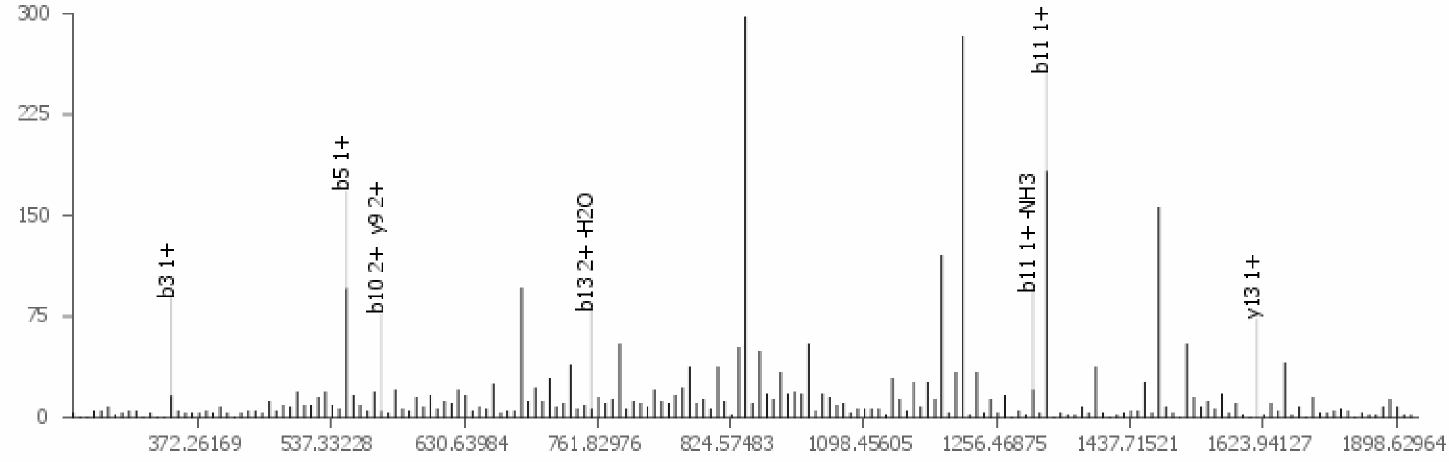

AT1G67500.1 - SSLGQRPLN(pS)K - 633.817236 - Charge:2

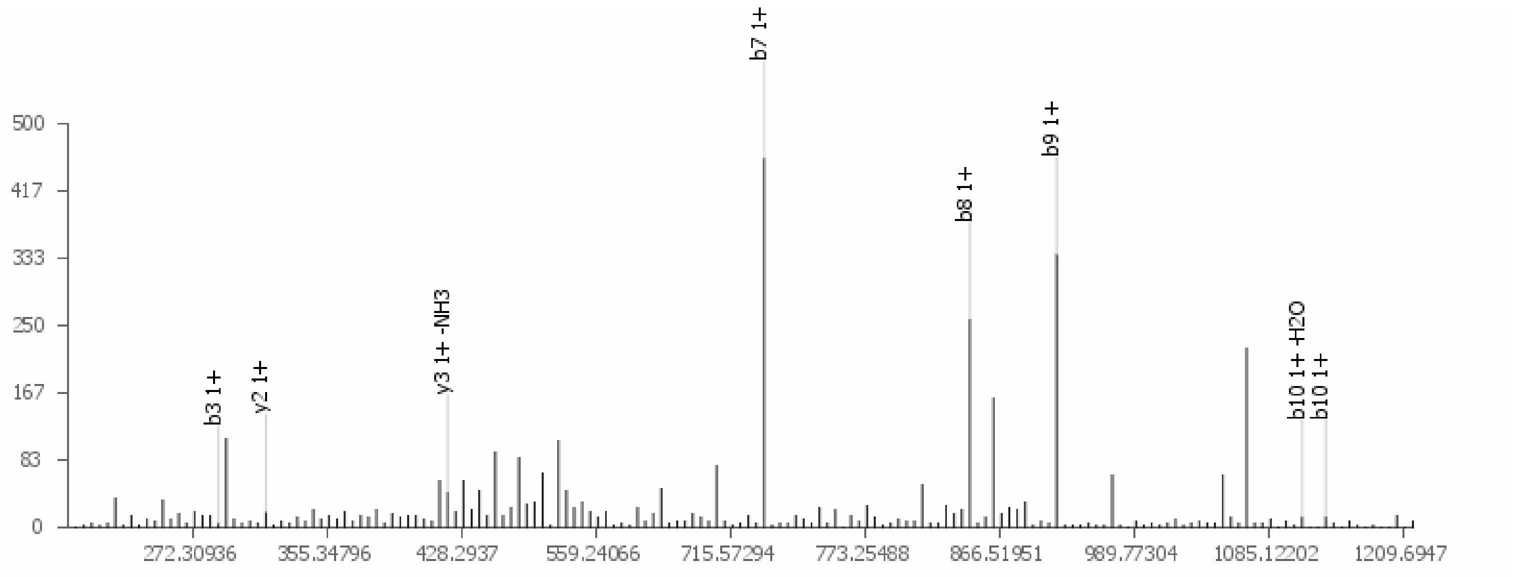

AT1G51260.1 - SLP(pS)PRNVLIPR - 714.891606 - Charge:2

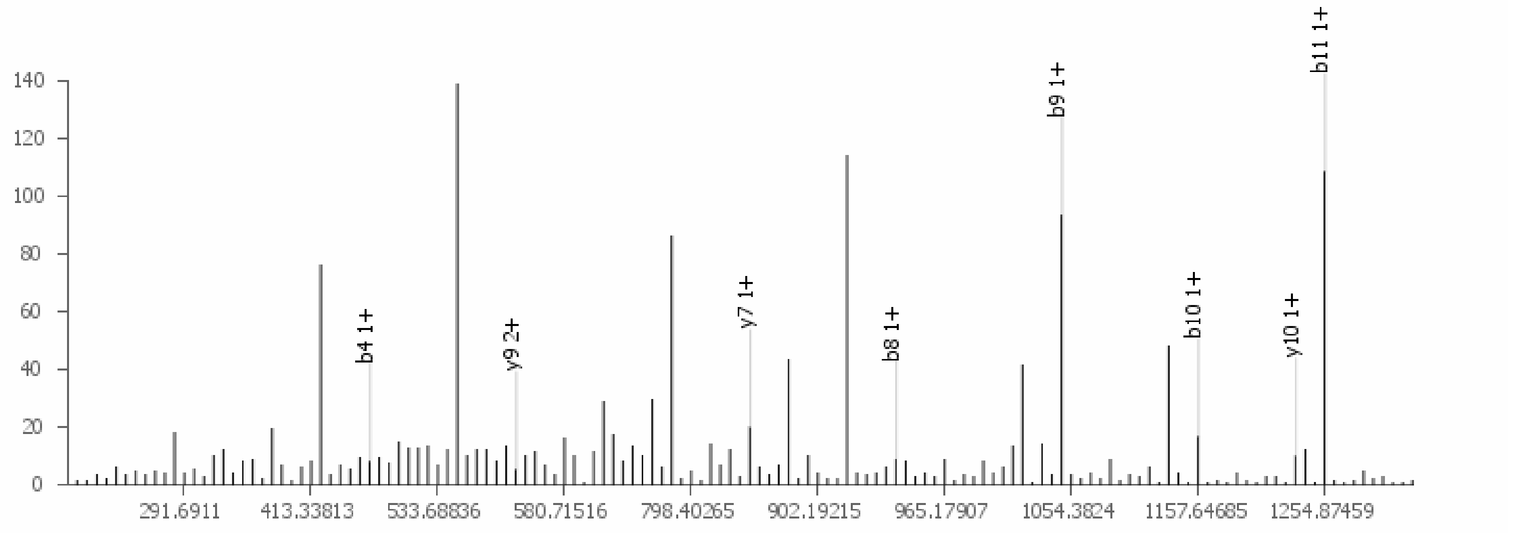

AT5G24390.1 - QRAPPTEDLLL(pY)VVAASVLQR - 1210.148653 - Charge:2

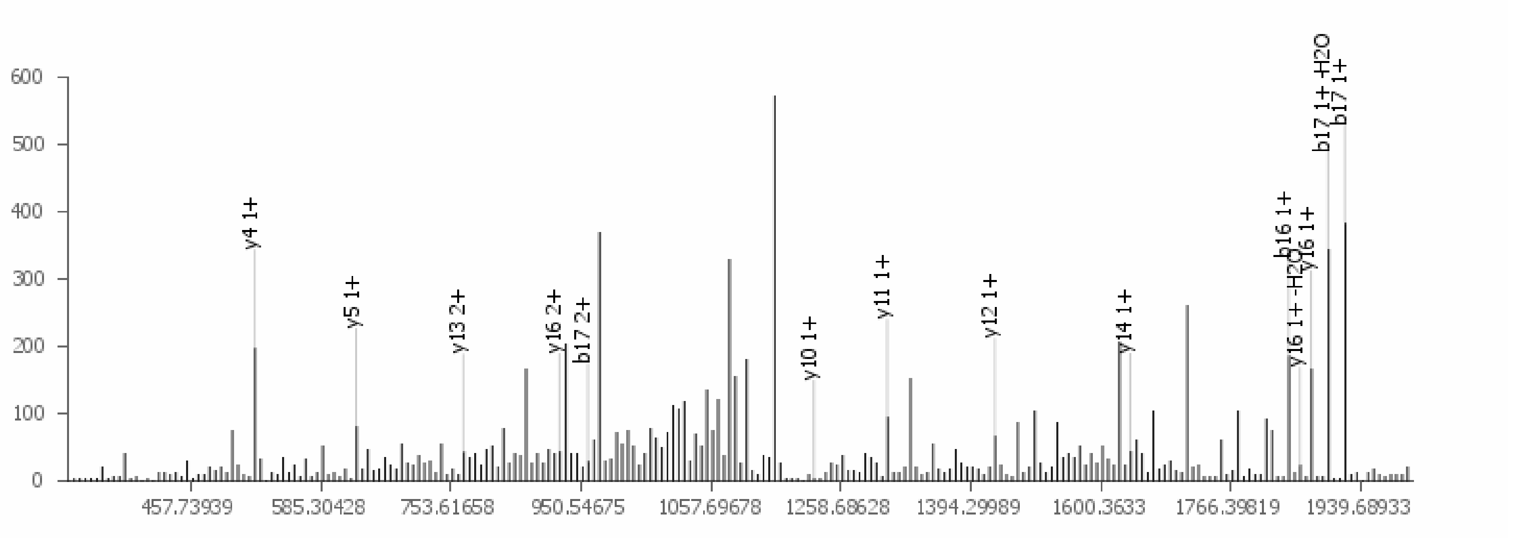

AT2G29070.1 - NISLVKGT(pY)IK - 658.36119 - Charge:2

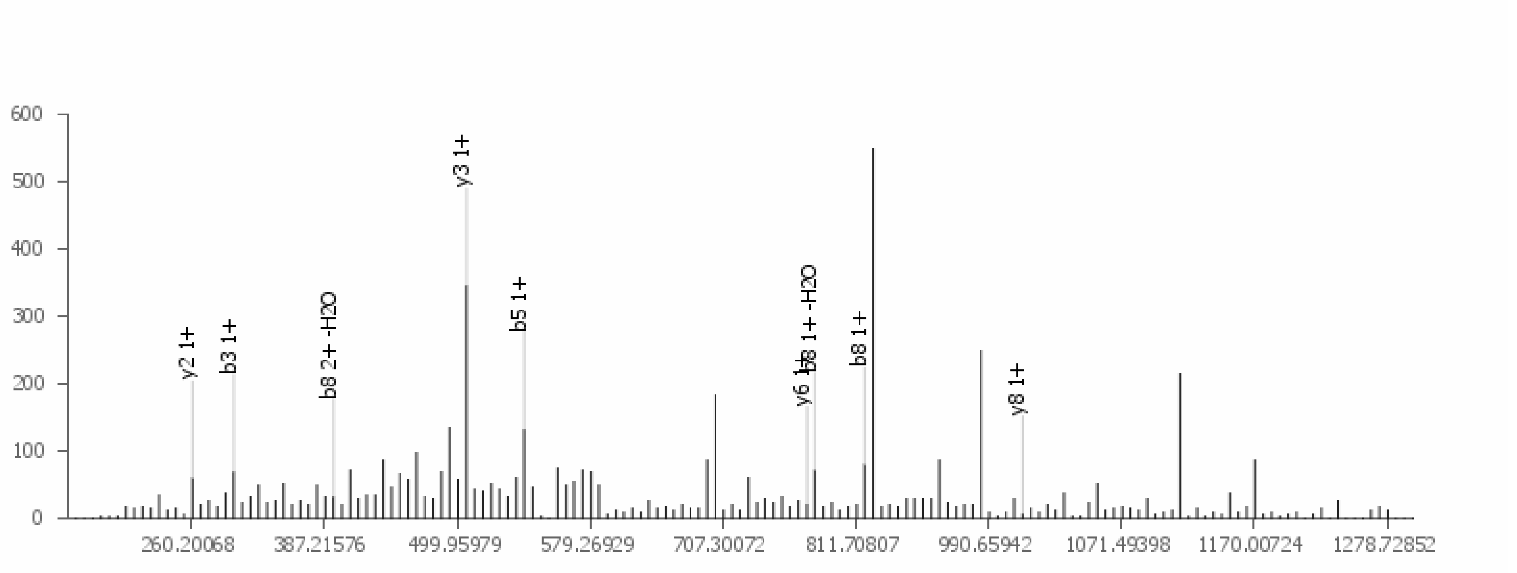

AT2G36695.1 - KGVAP(pS)PSP(oxM)R - 611.785421 - Charge:2

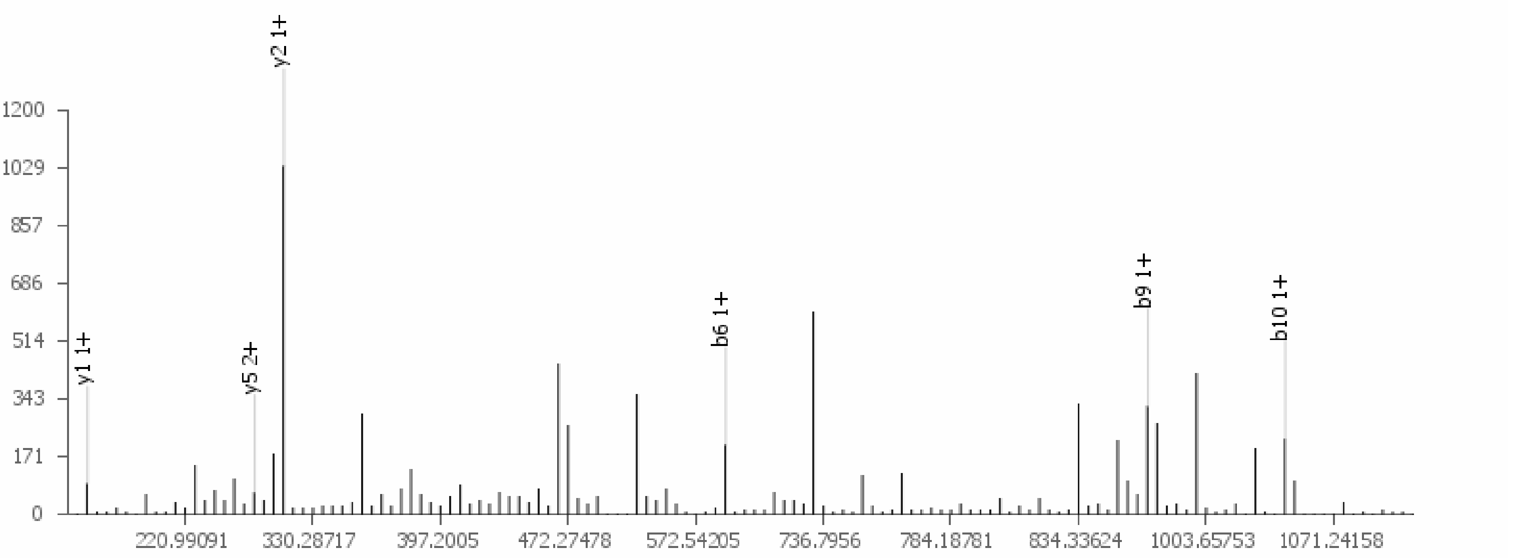

AT1G23360.1 - MAALLGIVSPV(pT)FTGKHPVN(pS)R - 1228.102381 - Charge:2

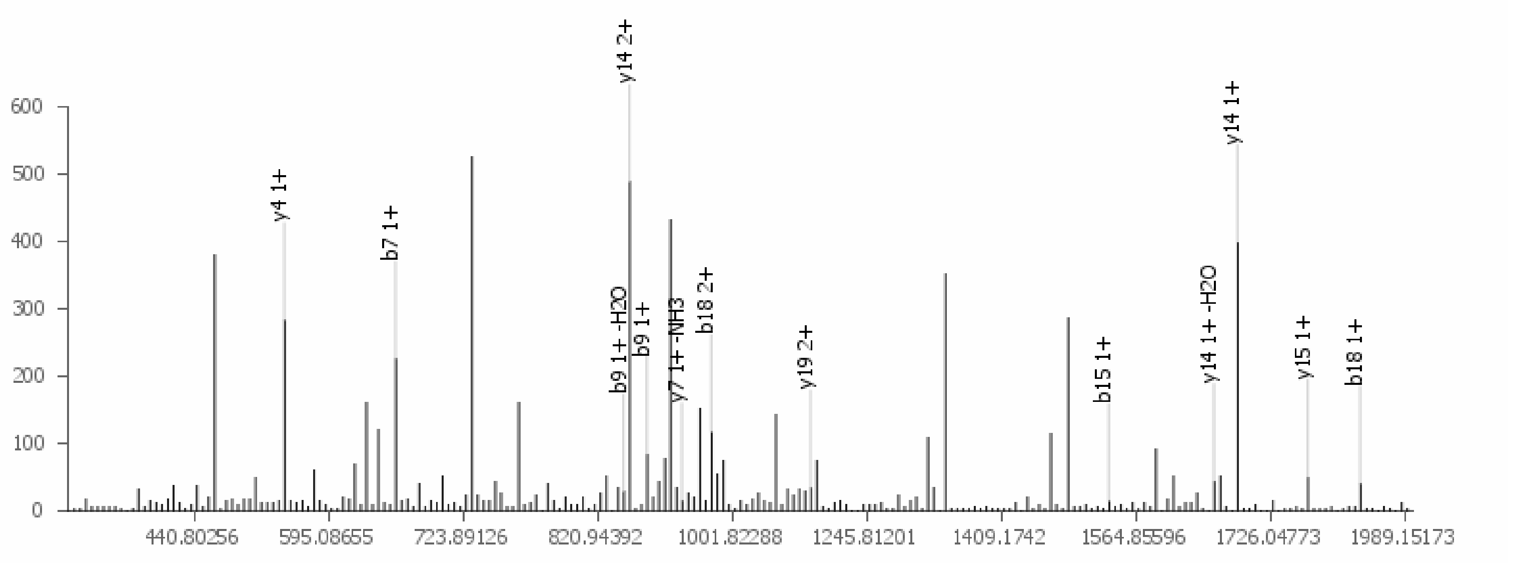

AT1G52430.1 - AVD(pS)FLDR - 501.720152 - Charge:2

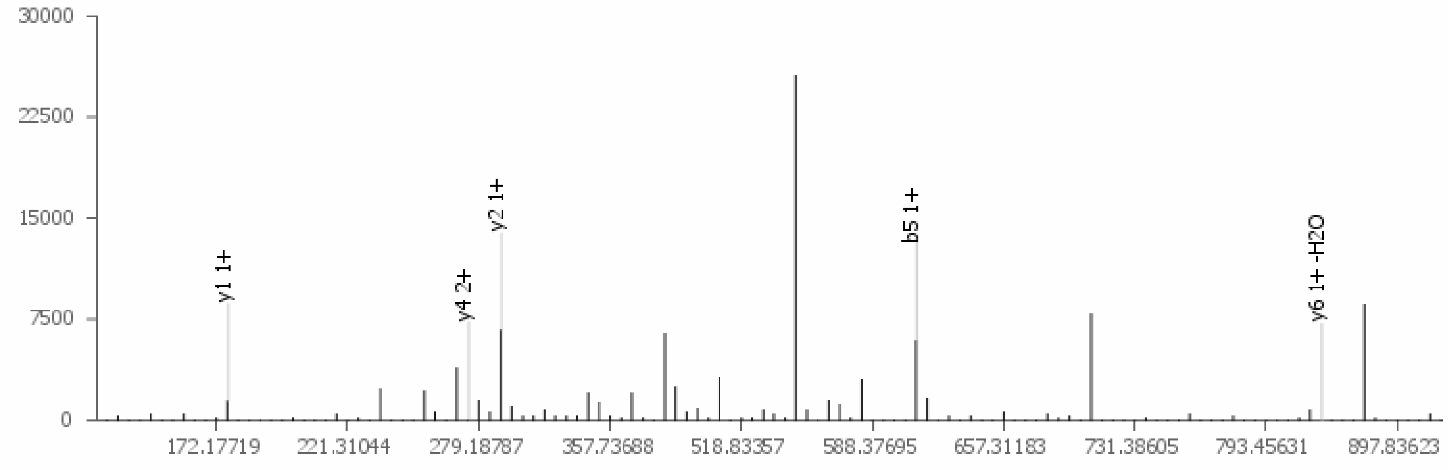

AT2G29000.1 - IISLDL(pS)NR - 555.782504 - Charge:2

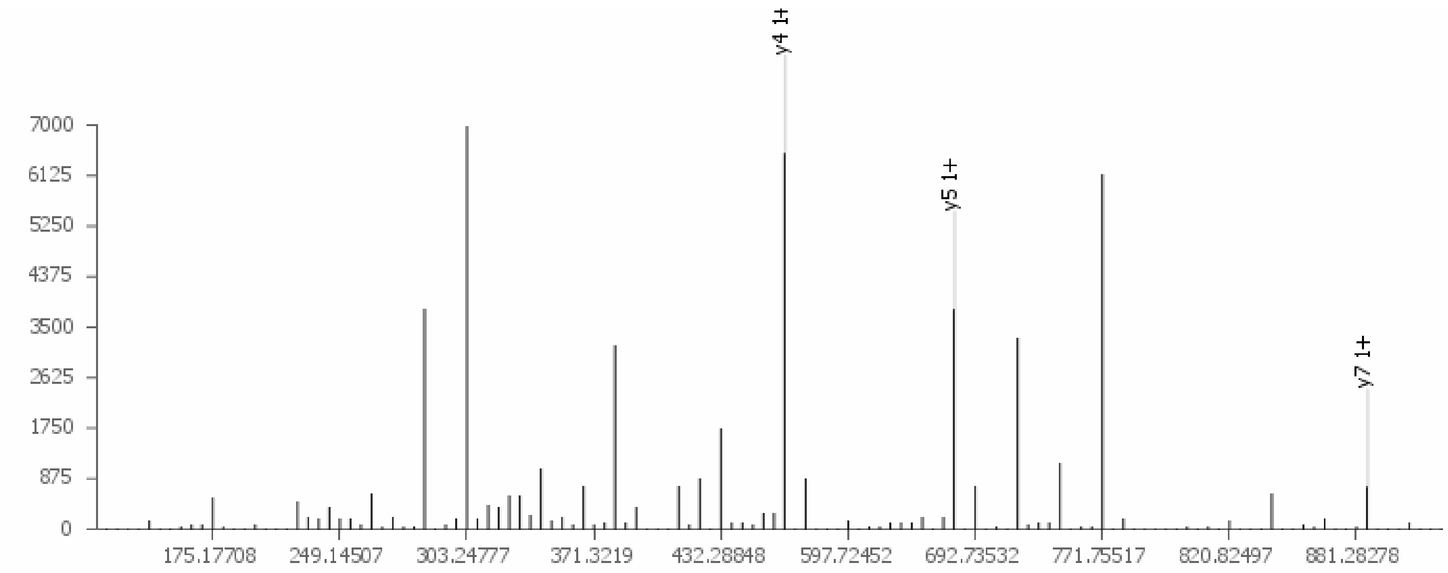

AT4G02710.1 - GQLEEGEEAIEKLF(pT)VNR - 1071.517361 - Charge:2

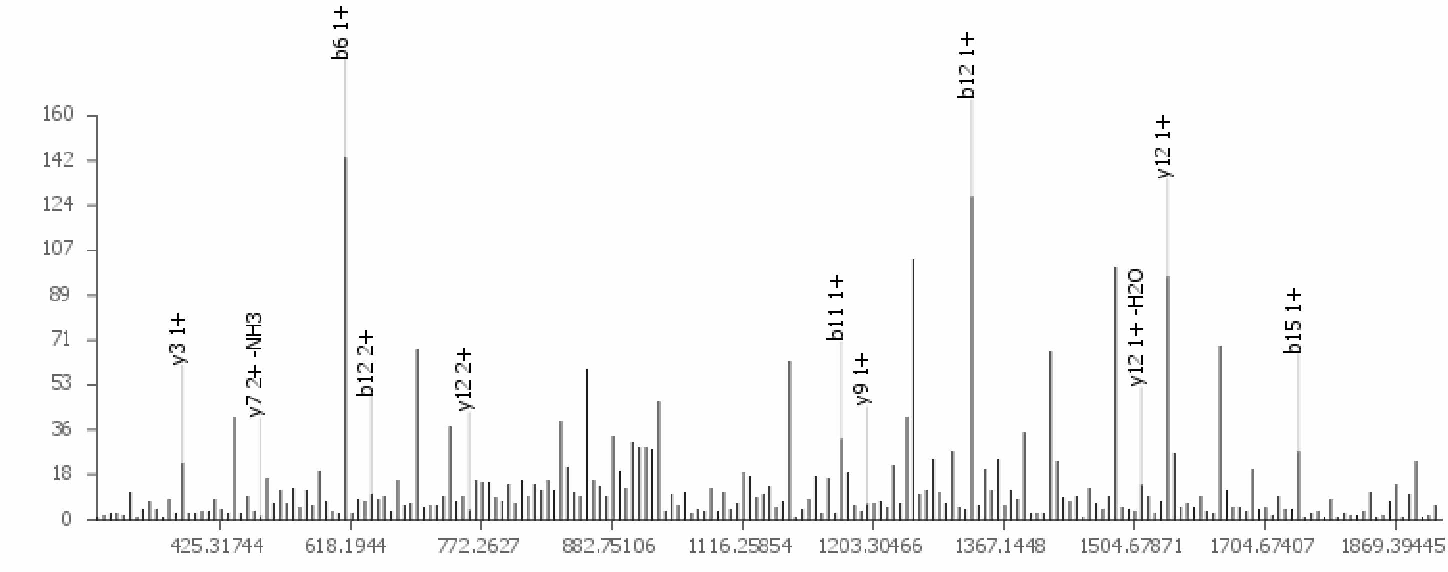

AT1G21610.1 - SEVNEMSGG(pS)IGGELLR - 907.909912 - Charge:2

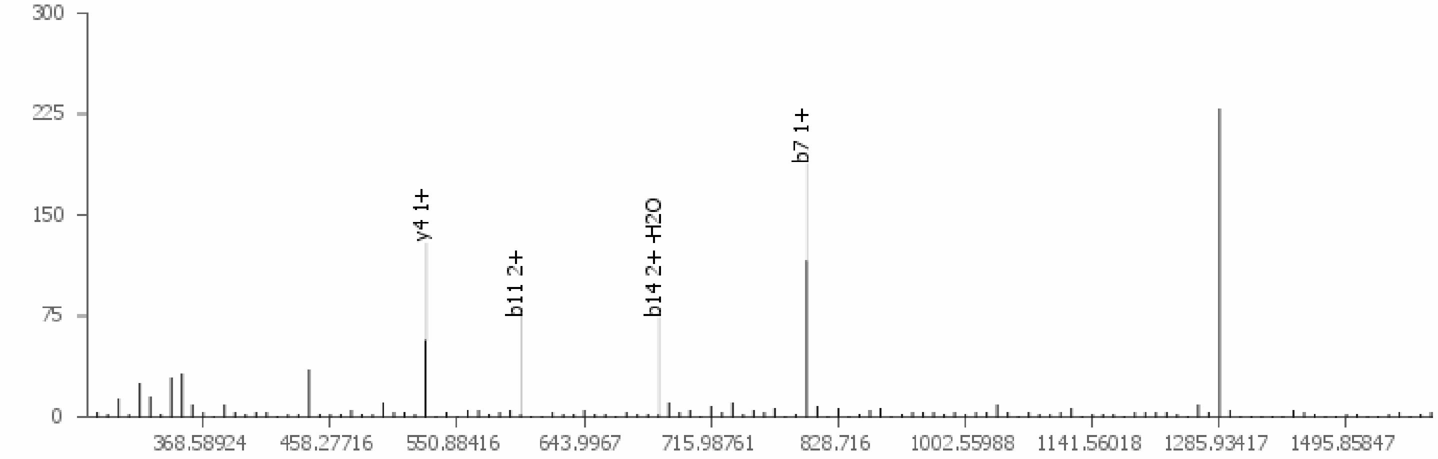

AT4G25510.1 - GEFFITLE(s)PIL(s)DEHI(pS)K - 1161.514406 - Charge:2

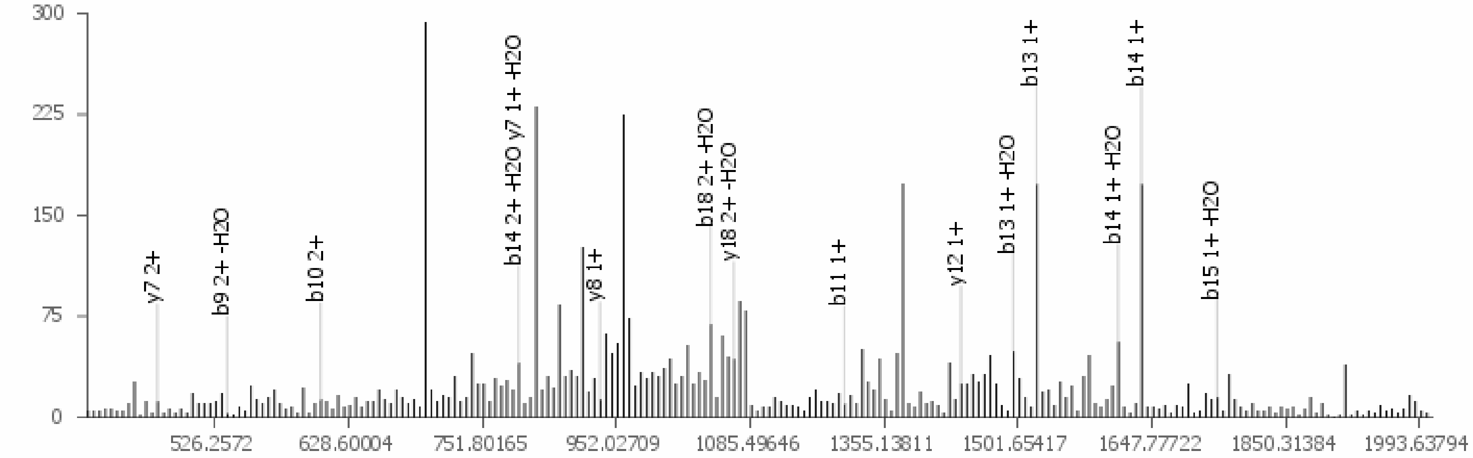

AT2G36800.1 - TQVPVET(pY)VPAGDWK - 885.41438 - Charge:2

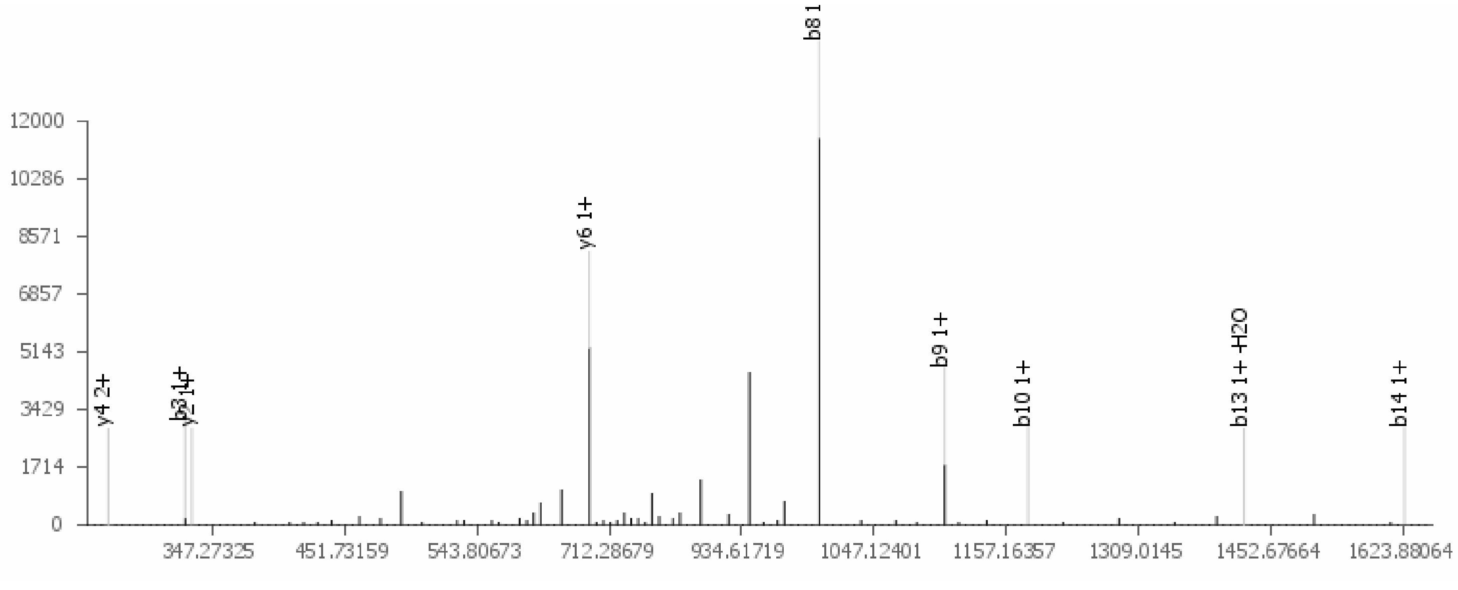

AT5G51150.1 - FLASQISNPSSKYPHS(pS)(pS)YSYLQTLDALK - 1131.521102 - Charge:3

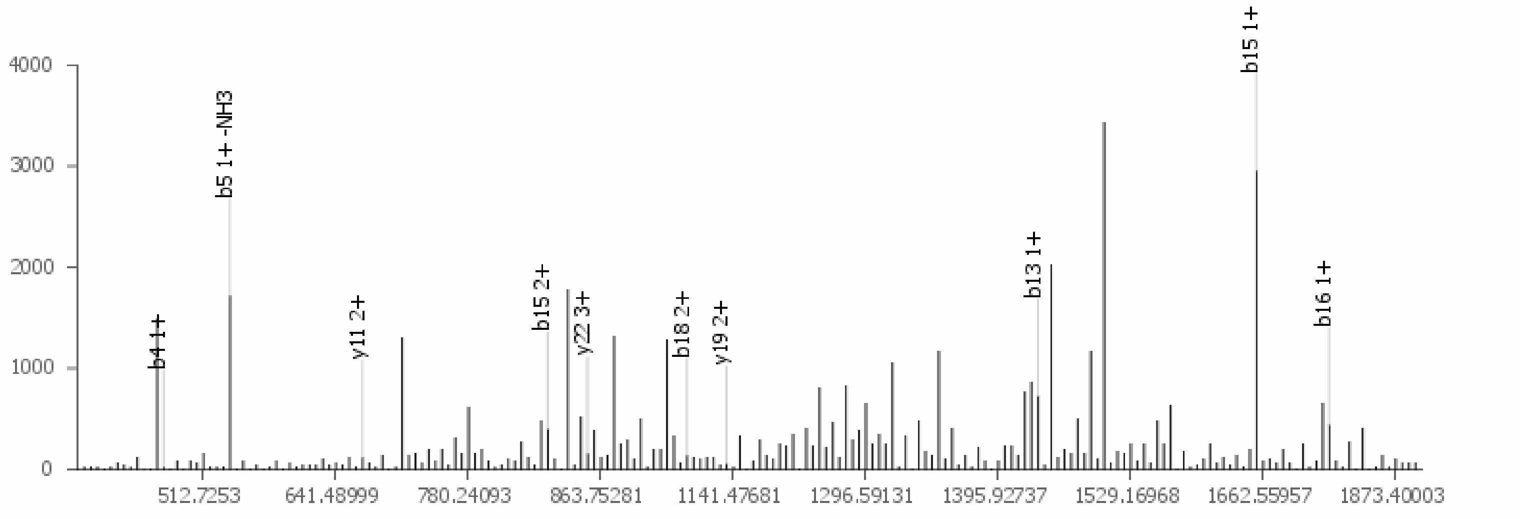

AT1G13670.1 - KL(t)(s)FLN(s)LLR - 686.370411 - Charge:2

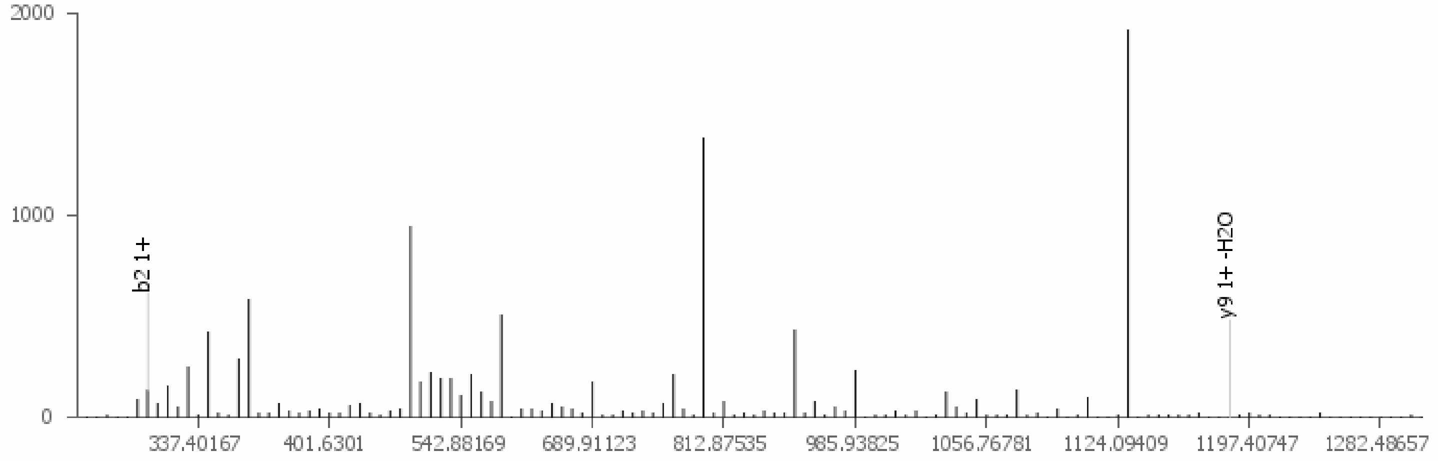

AT1G12800.1 - ST(pS)(pS)LDGLKLVKPVLK - 922.98334 - Charge:2

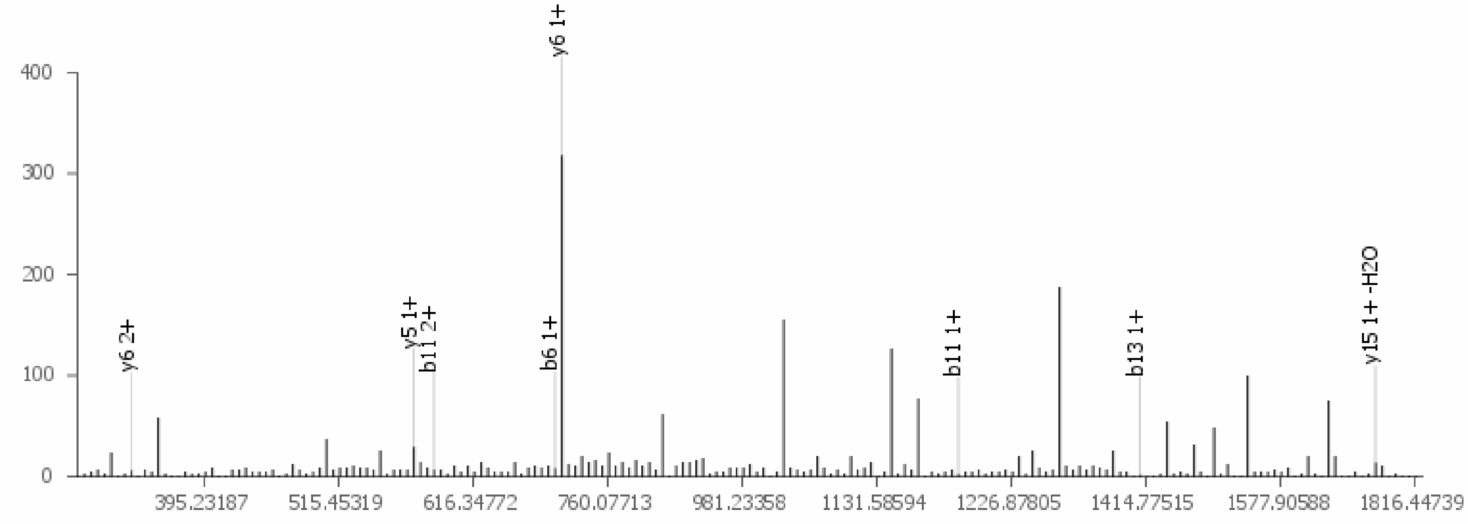

AT5G47690.1 - YL(pS)IKQTQQTADAPEIQK - 1071.517732 - Charge:2

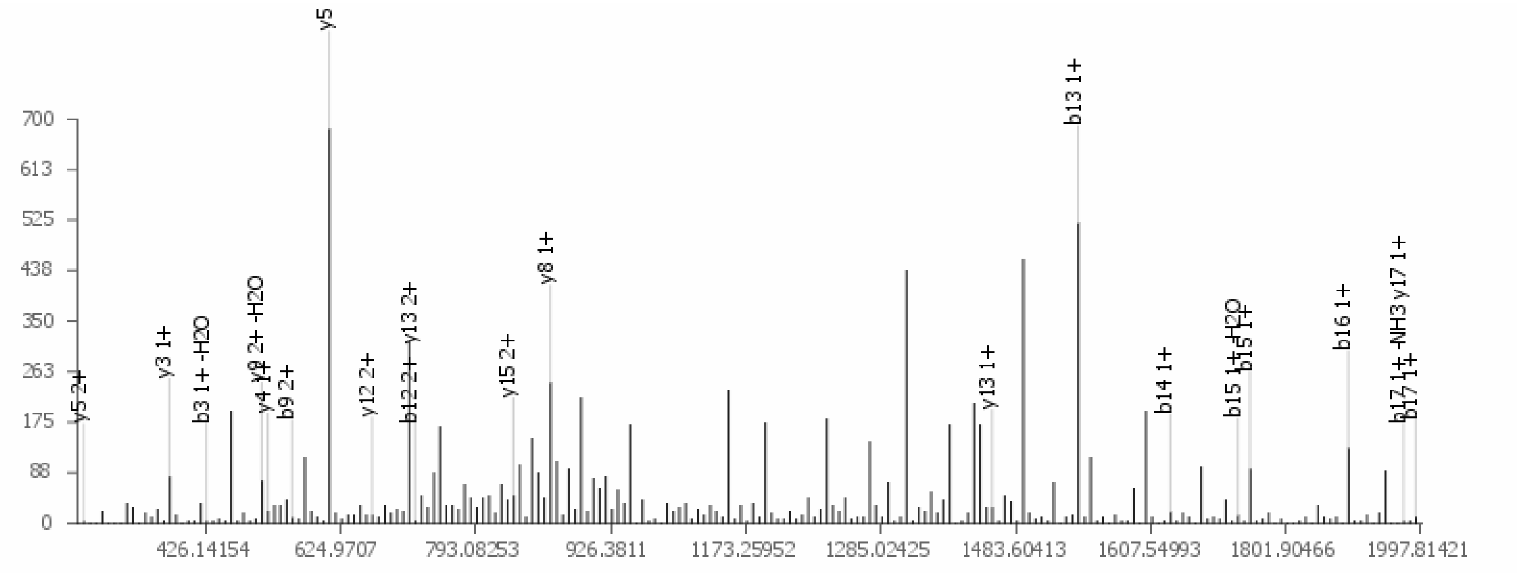

AT2G16280.1 - TGV(pS)L(pS)KDLMAIAGEALK - 982.47144 - Charge:2

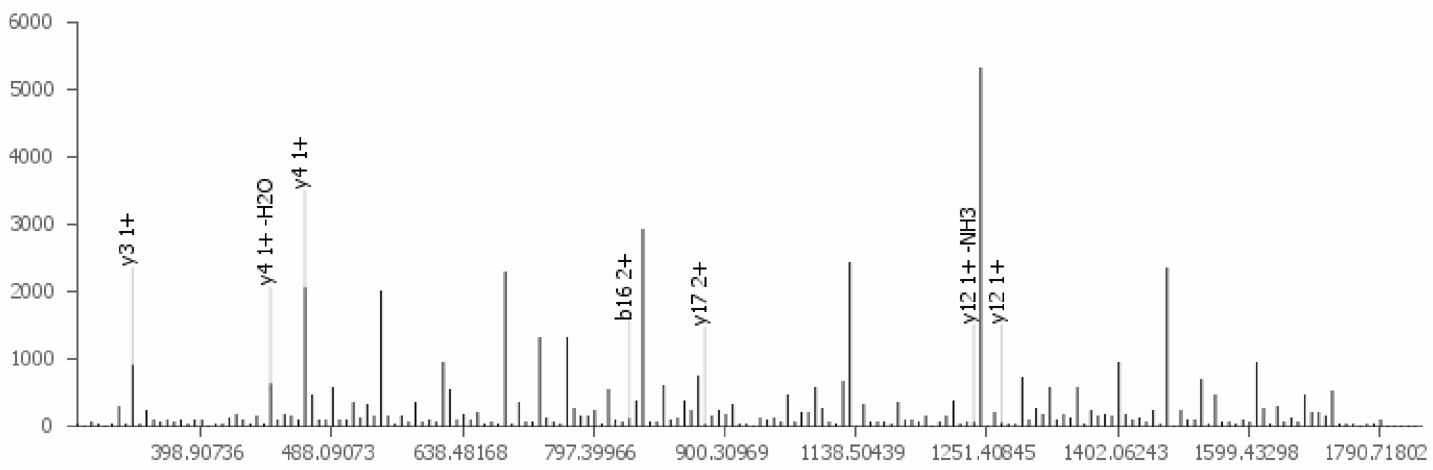

AT3G29750.1 - KVAAVG(s)(t)EVR - 598.80136 - Charge:2

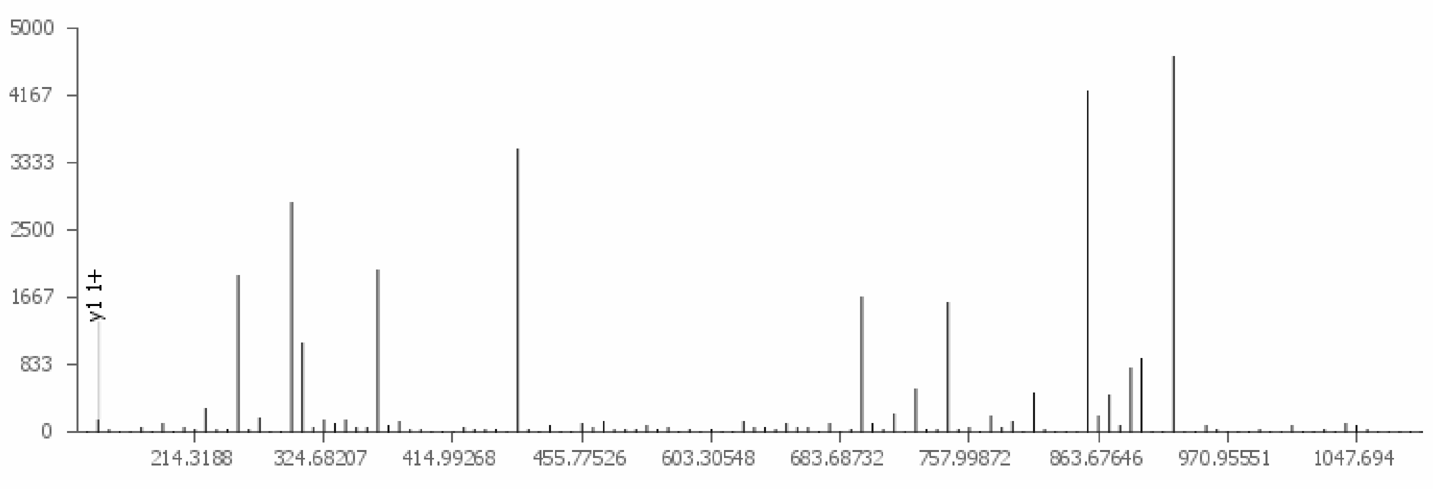

AT1G73840.1 - (pT)LIDQNHQQAREILIR - 1014.522462 - Charge:2

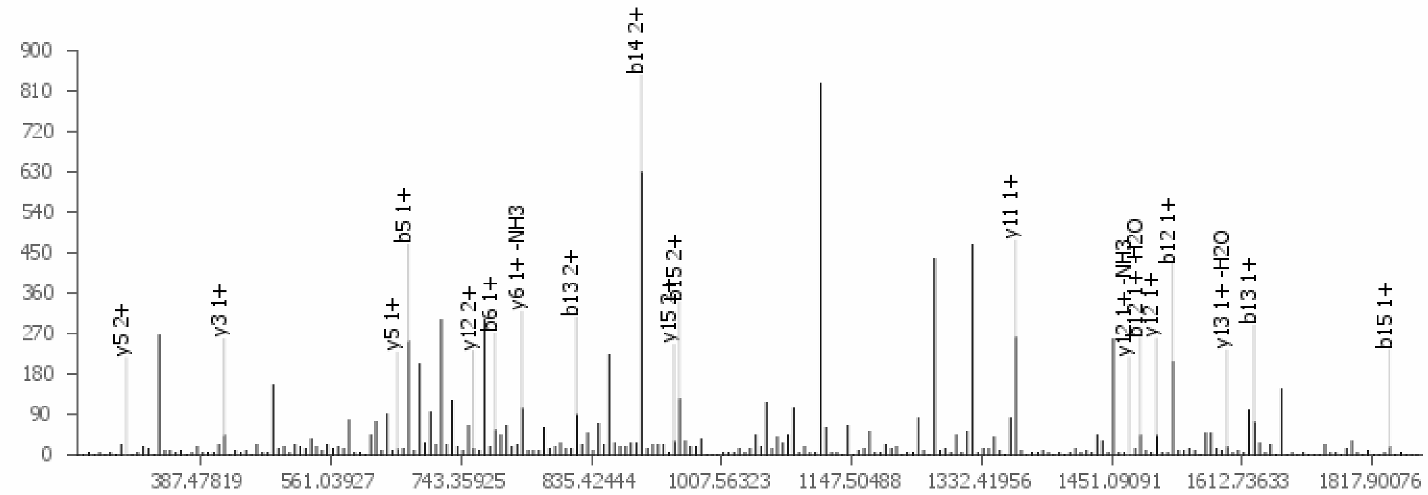

AT5G01600.1 - ADLAIPITSHA(pS)LAR - 539.274814 - Charge:3

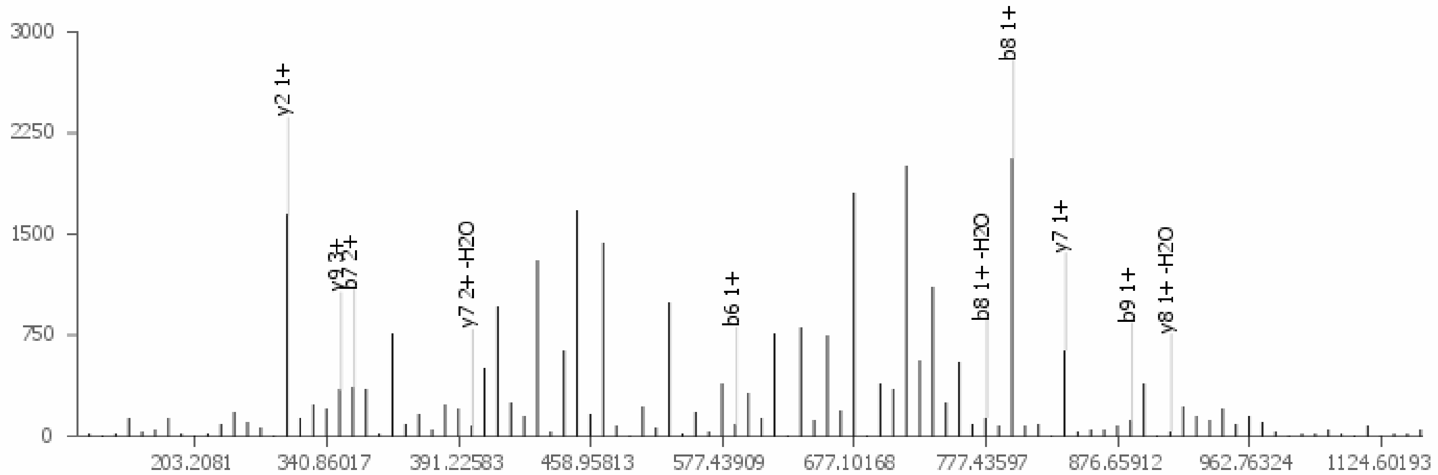

AT3G55100.1 - Y(oxM)INWTR(pT)PELIGTR - 973.95707 - Charge:2

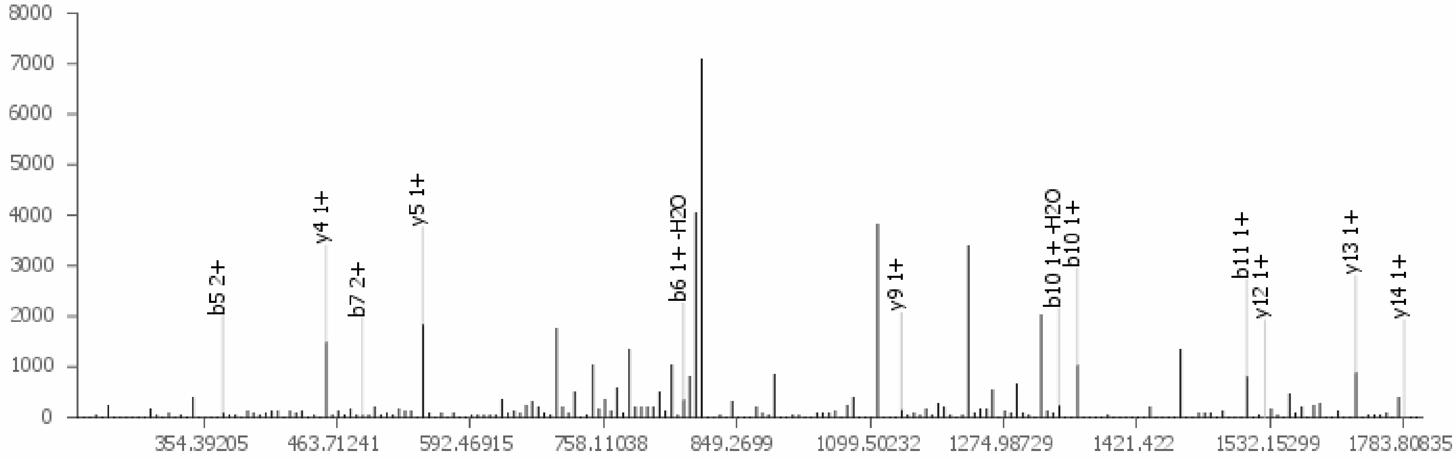

AT1G32730.1 - QLSSNFAQFNNLNA(pS)(pS)R - 1029.433027 - Charge:2

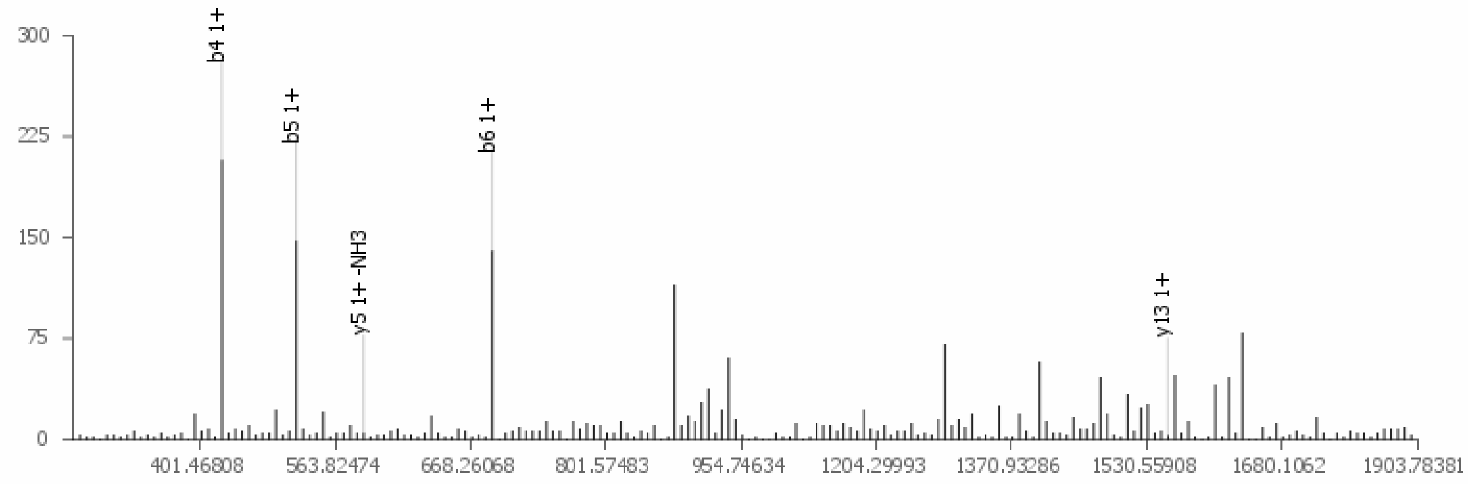

AT4G33210.1 - FCPKISSV(pS)MSK - 697.311995 - Charge:2

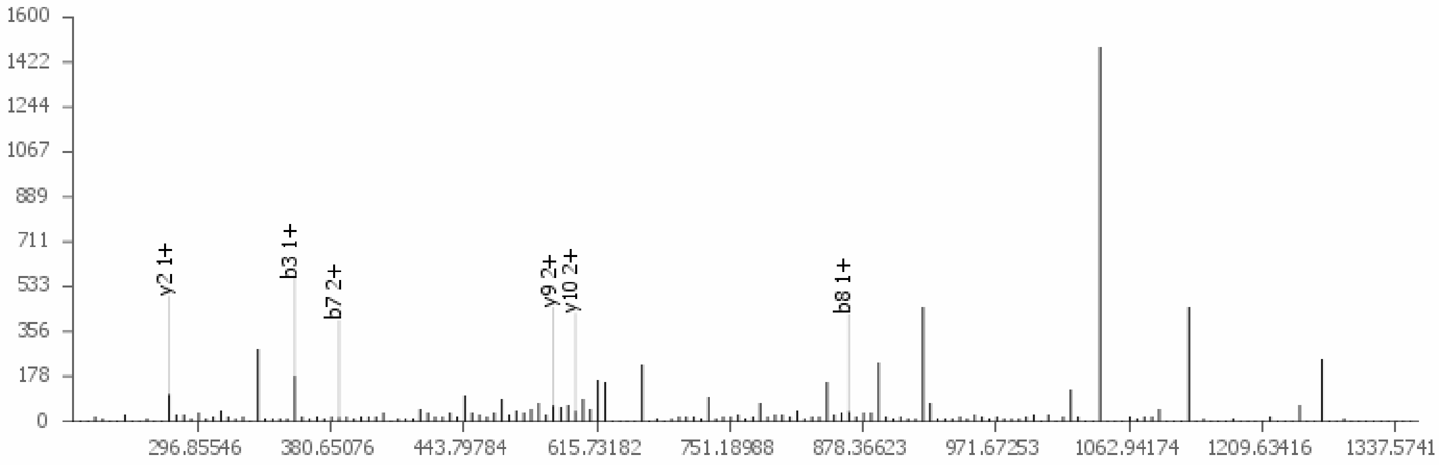

AT5G13630.1 - GSDKGIL(pS)DVELLK - 777.393979 - Charge:2

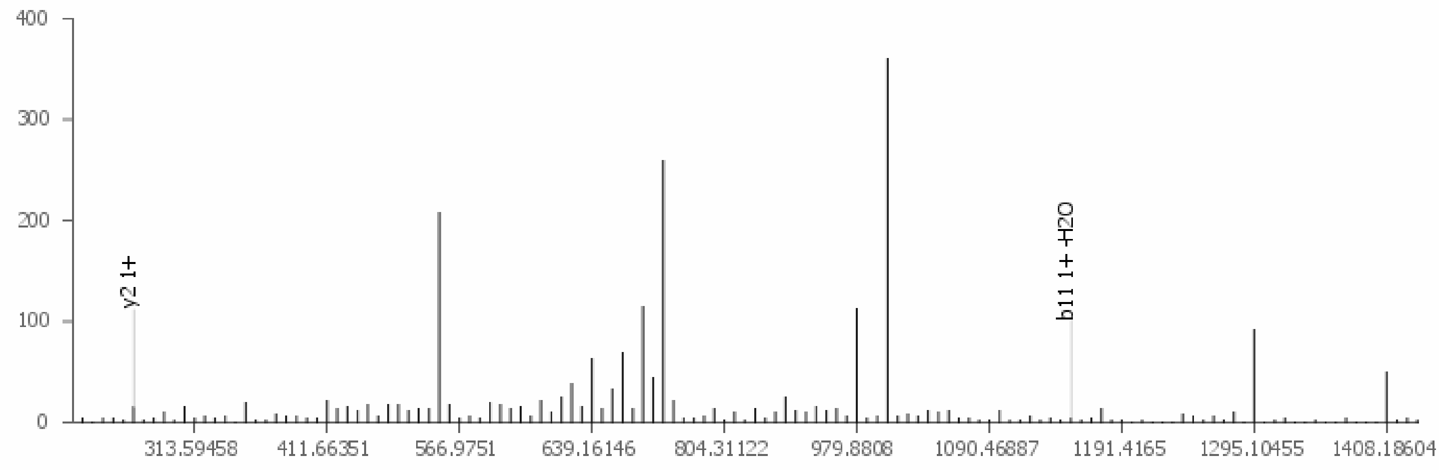

AT1G67940.1 - VADDG(pS)RILK - 577.288959 - Charge:2

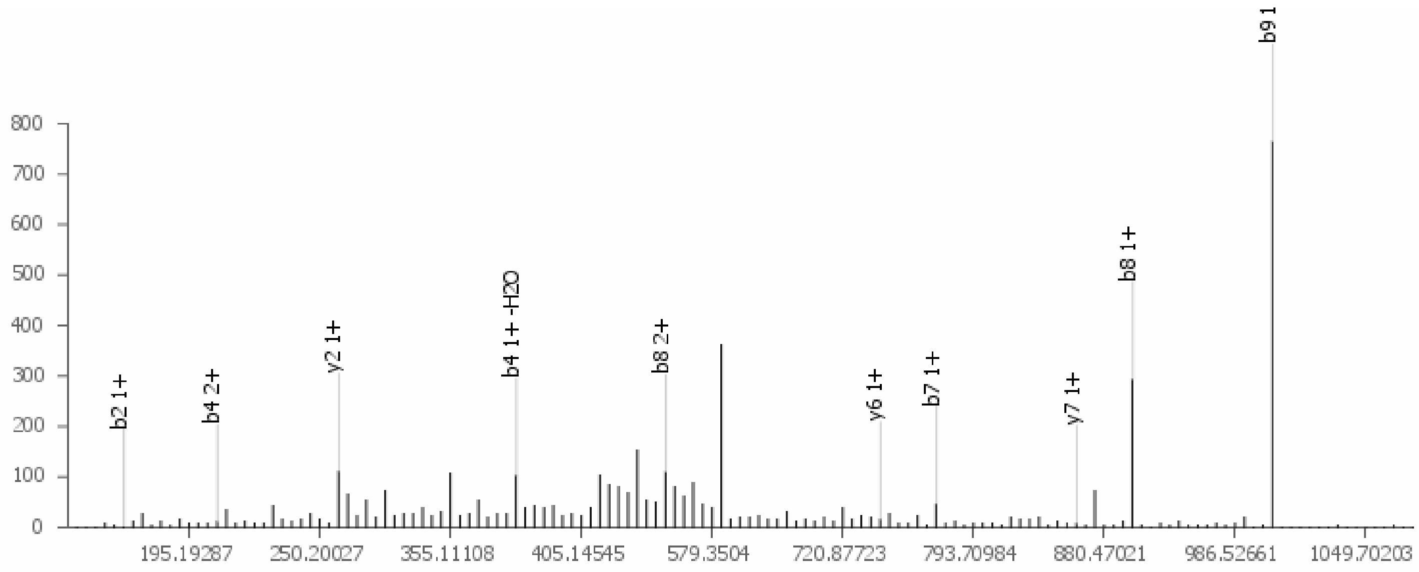

AT2G47830.1 - Q(pT)ILQVEGVK - 597.808634 - Charge:2

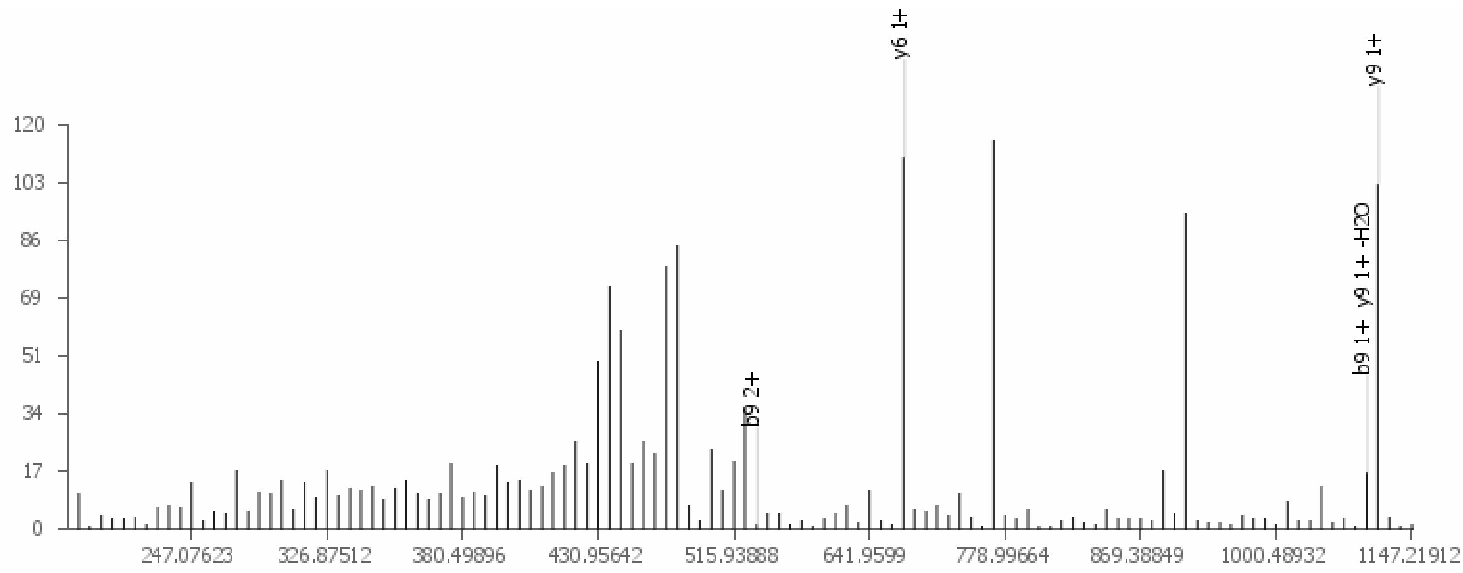

AT5G14610.1 - GGPPS(t)(s)(s)PPR - 560.24718 - Charge:2

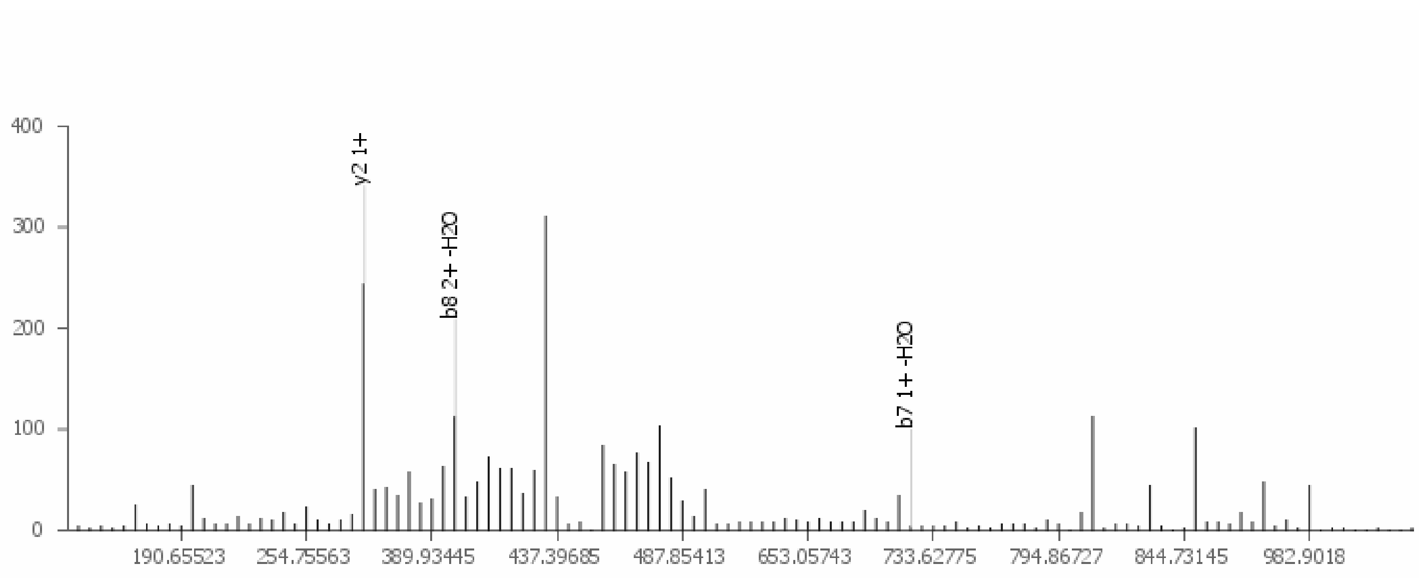

AT4G28070.1 - (pT)ISRLTEMNSK - 680.318882 - Charge:2

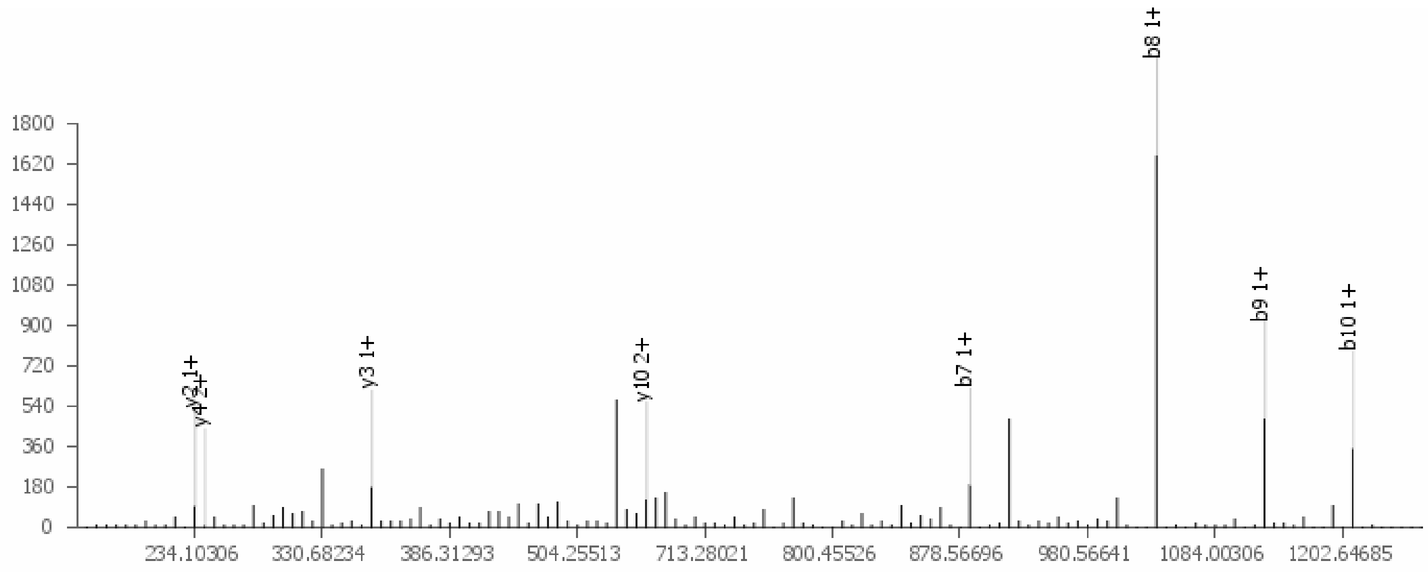

AT5G50900.1 - ISMVES(pS)K - 480.70979 - Charge:2

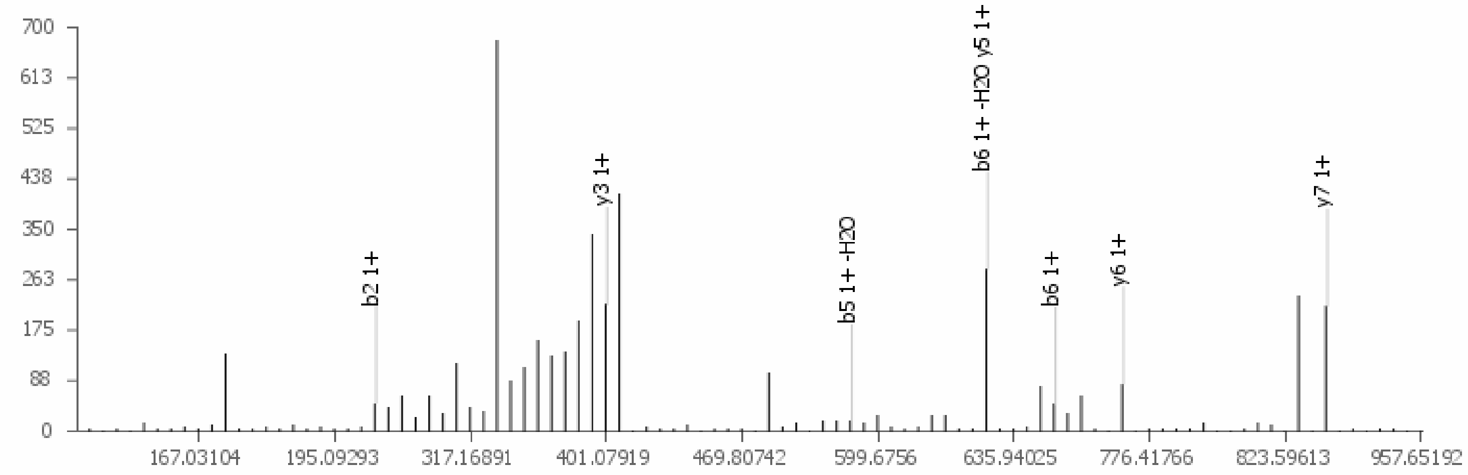

AT5G23820.1 - Y(pS)VSLRR - 480.740249 - Charge:2

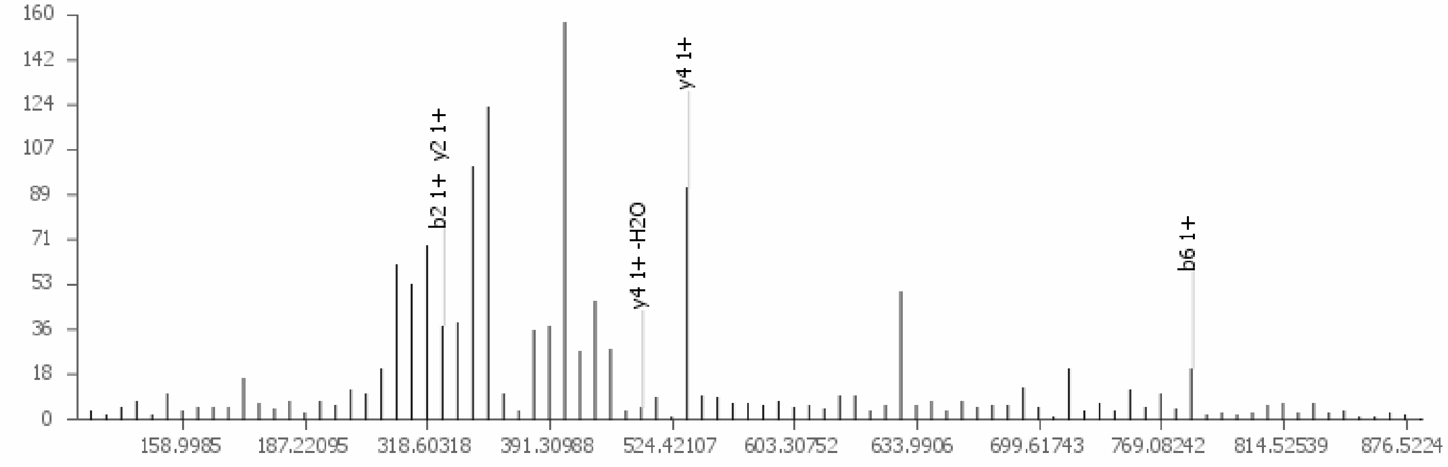

AT1G10180.1 - LVFSNI(pS)VATK - 629.825641 - Charge:2

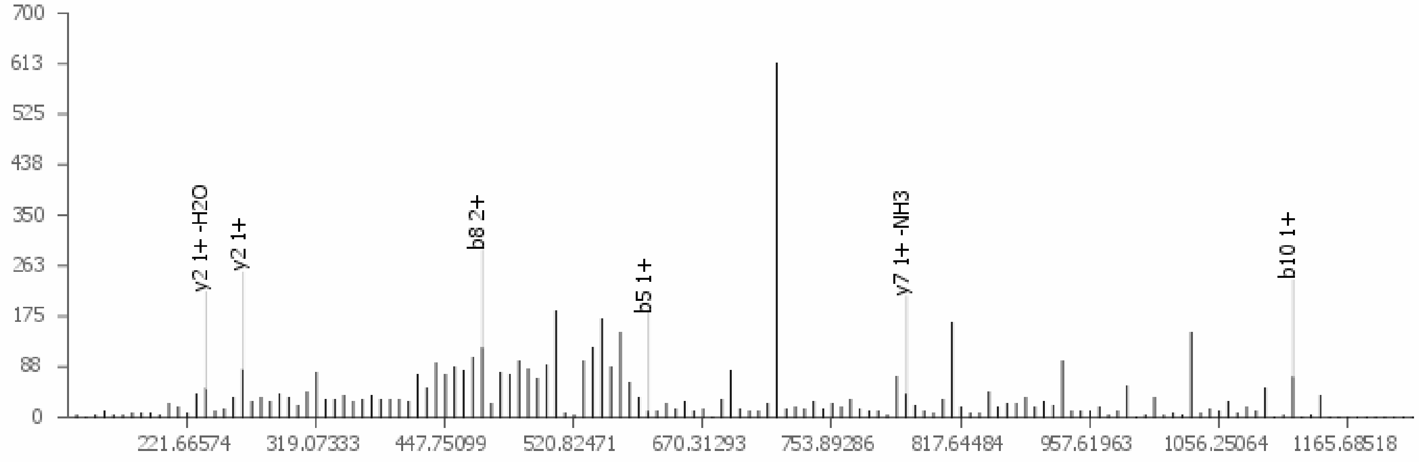

AT1G51500.1 - IFGVQVTH(pS)K - 598.301337 - Charge:2

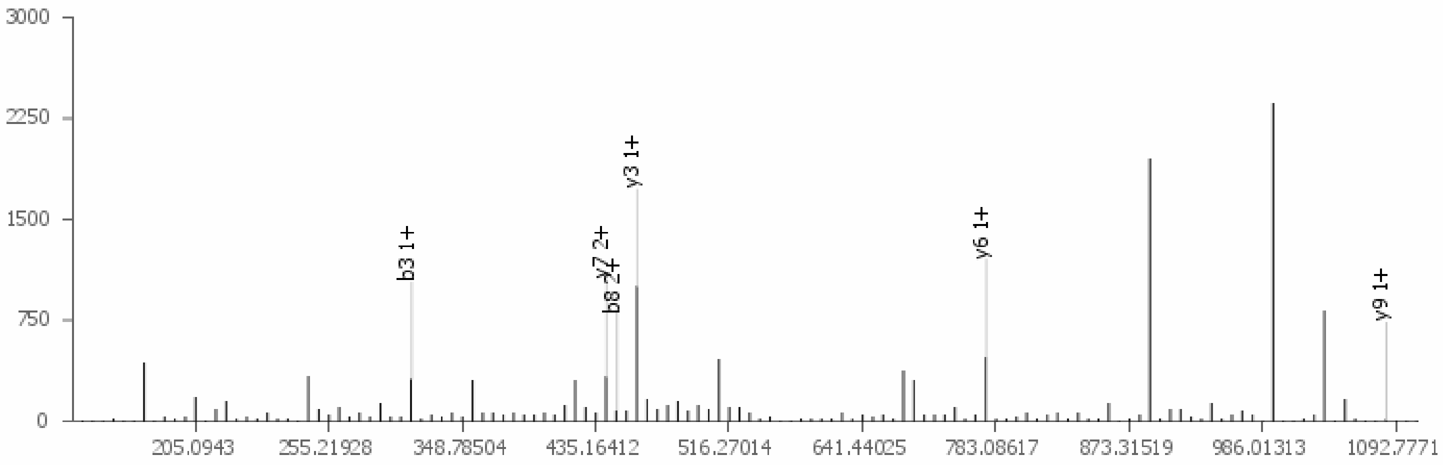

AT3G46330.1 - SLLVINL(pS)GNK - 619.325998 - Charge:2

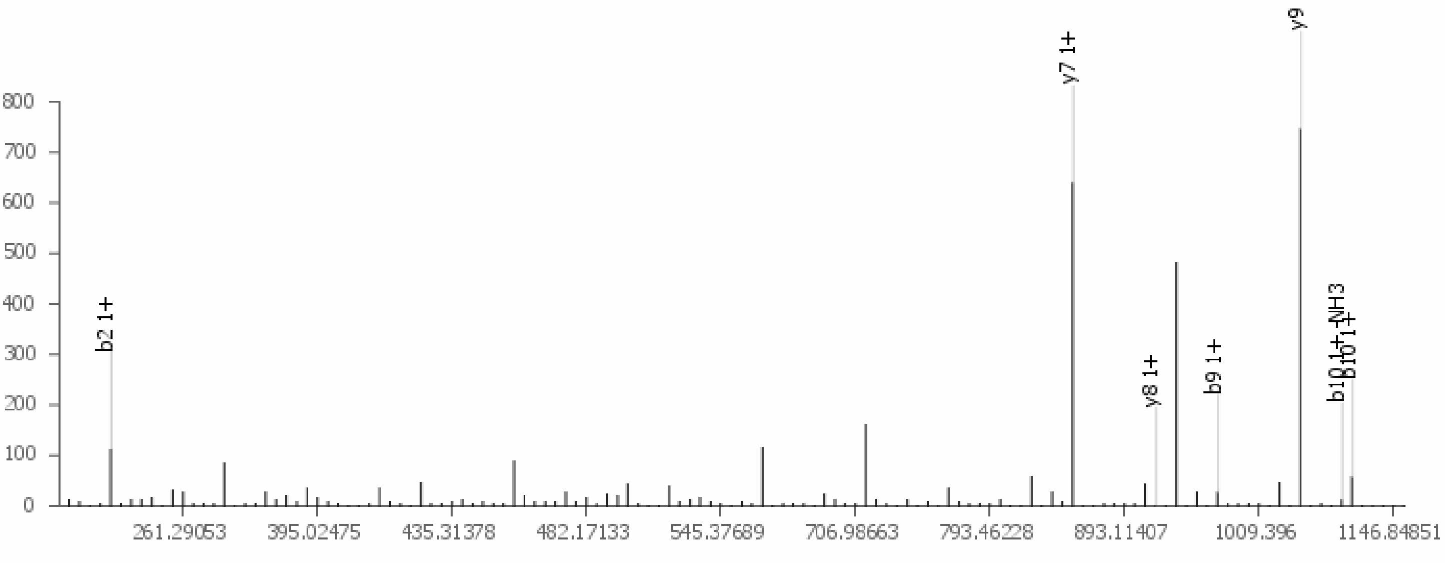

AT1G28190.1 - (pT)PVLSETETLK - 649.31695 - Charge:2

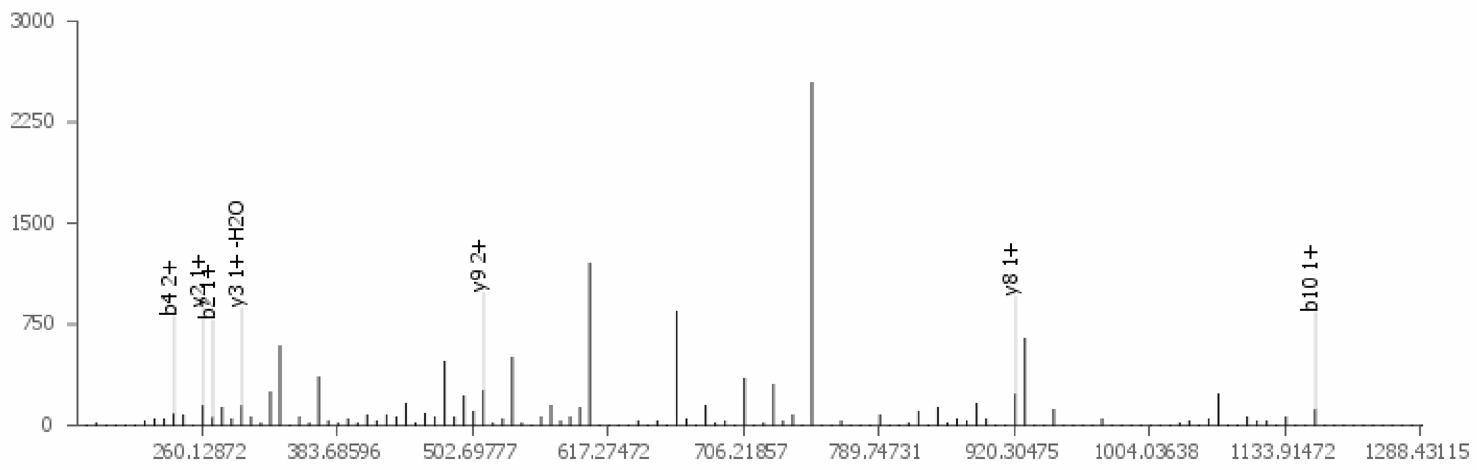

AT4G38710.1 - ER(pS)AEELDR - 592.753253 - Charge:2

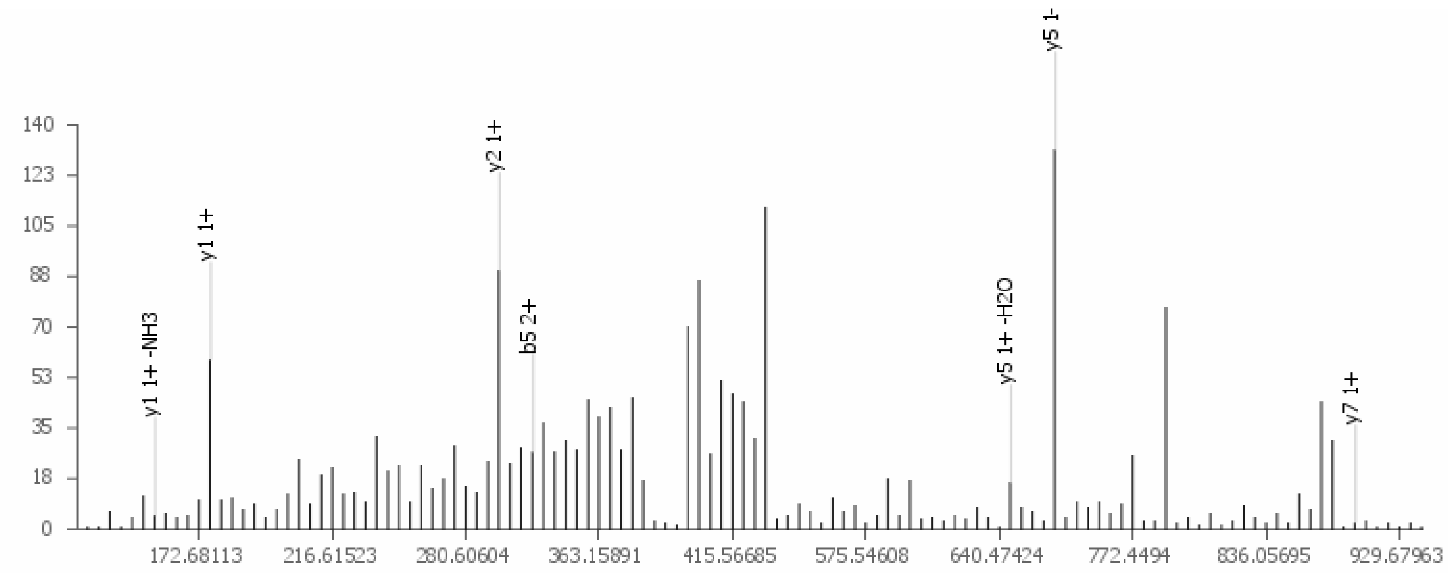

AT4G26180.1 - QLF(pS)GLSINYLK - 731.86815 - Charge:2

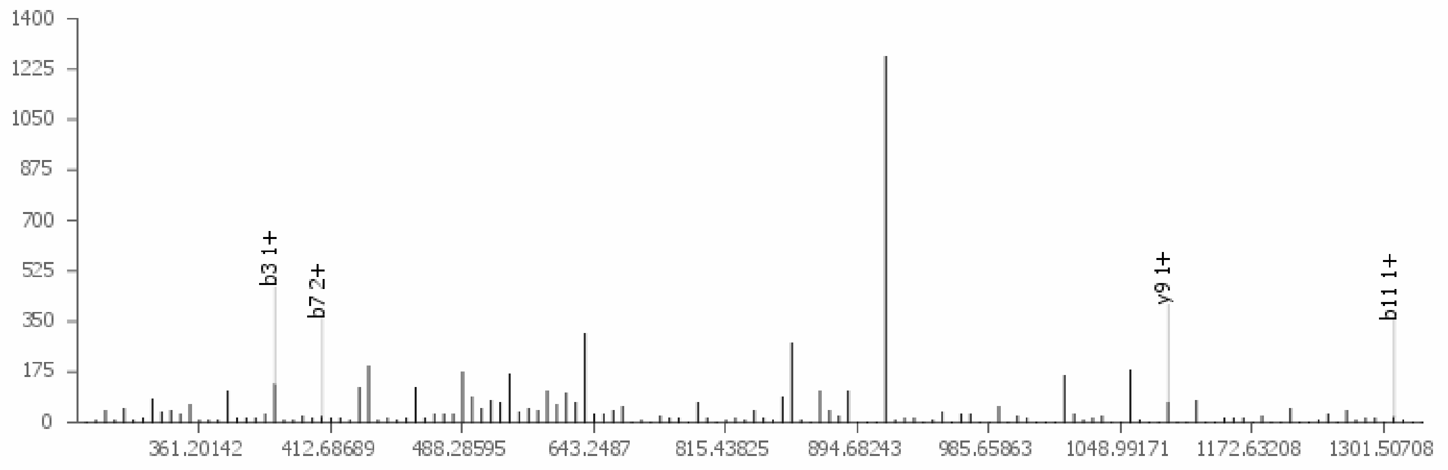

AT2G40070.1 - K(pS)LD(oxM)AIR - 515.243617 - Charge:2

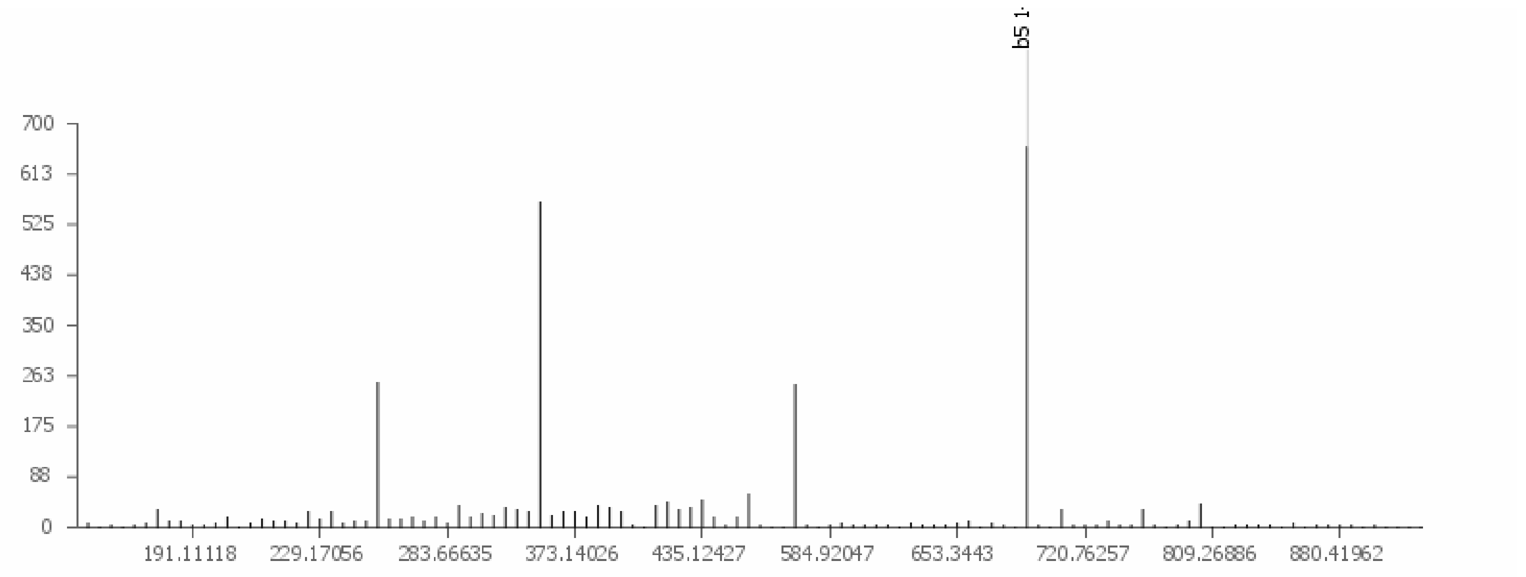

AT5G52840.1 - (pT)EIPAATPSDPQLK - 774.372676 - Charge:2

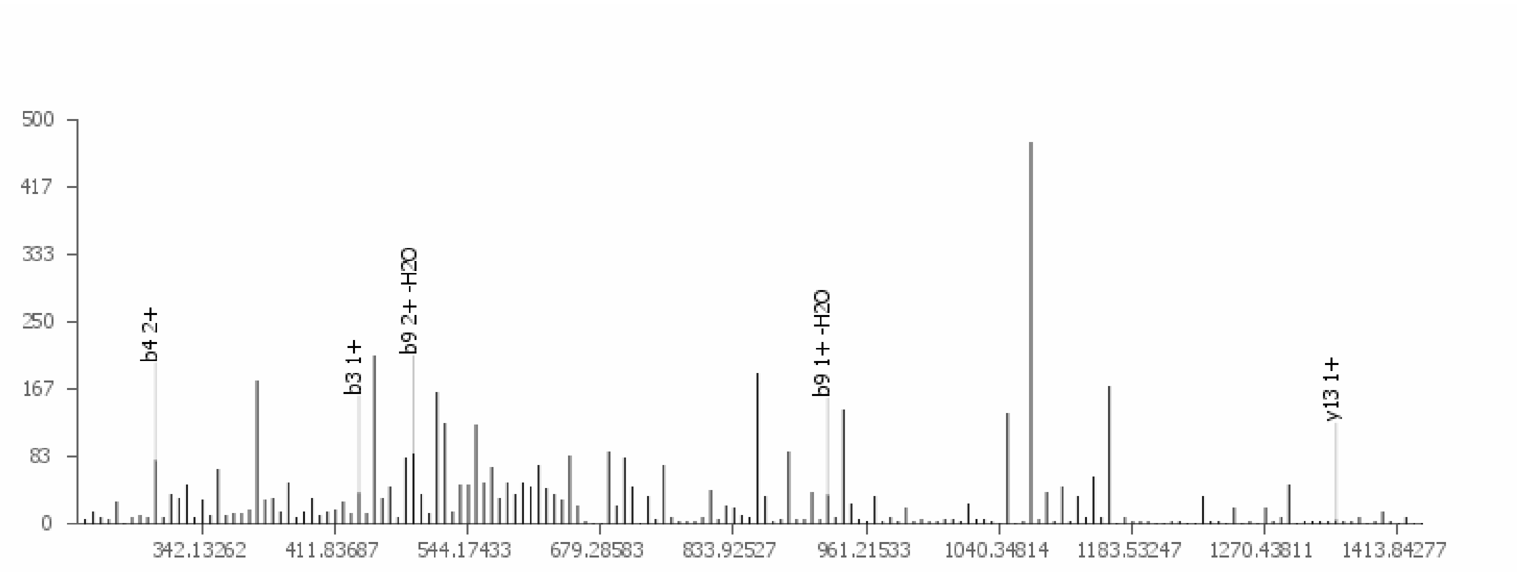

AT3G45310.1 - YKLGAAQNC(pS)ATLK - 774.372577 - Charge:2

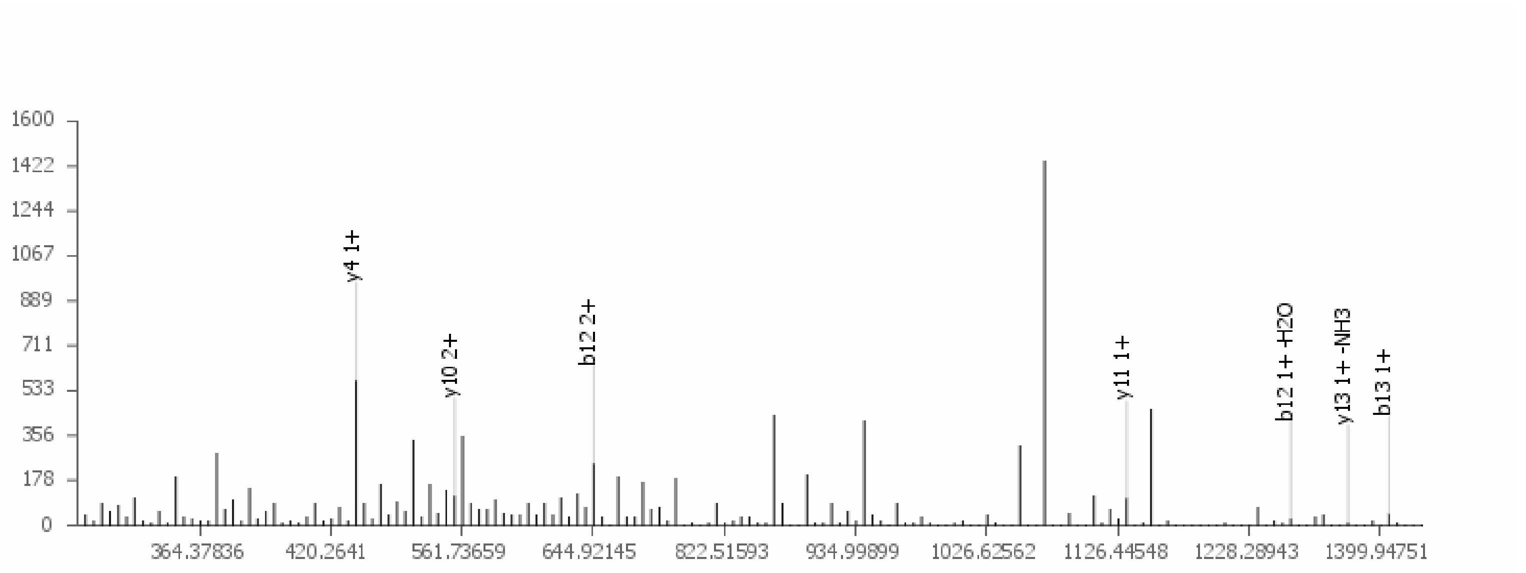

AT5G08610.1 - (pS)(pS)KFPLGVR - 575.758239 - Charge:2

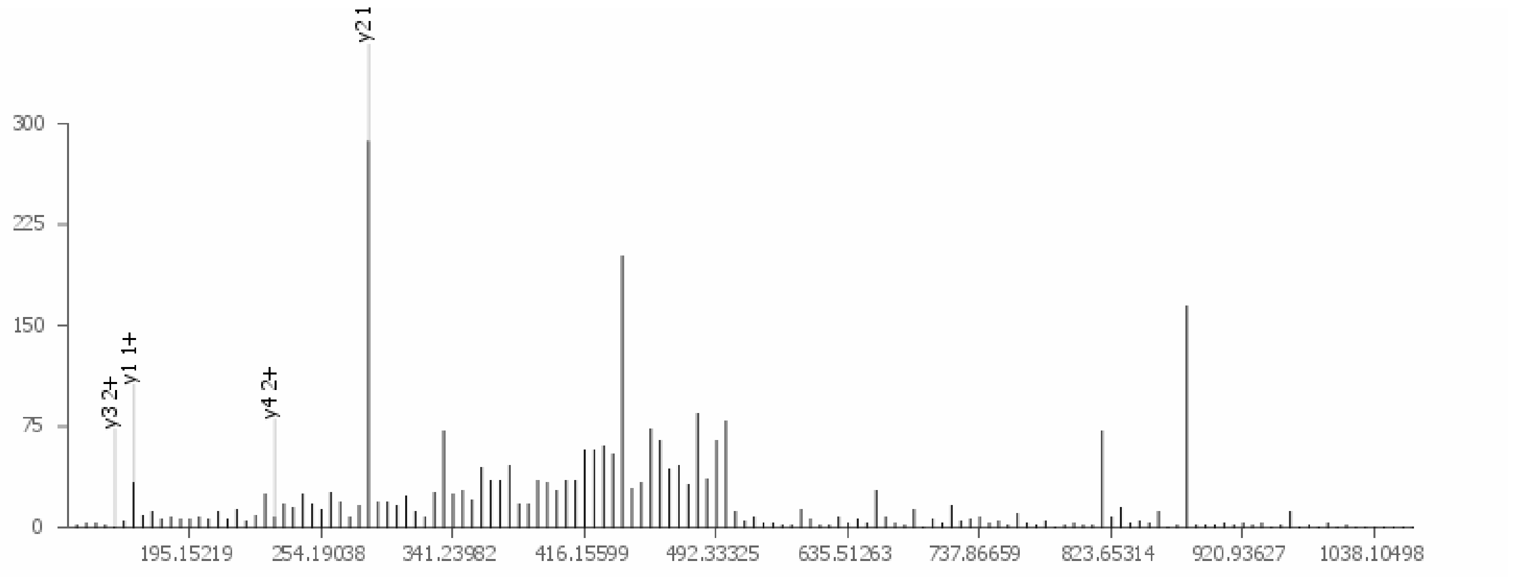

AT4G30190.1 - (oxM)TAIEE(oxM)AG(oxM)DVLC(pS)DK - 986.377525 - Charge:2

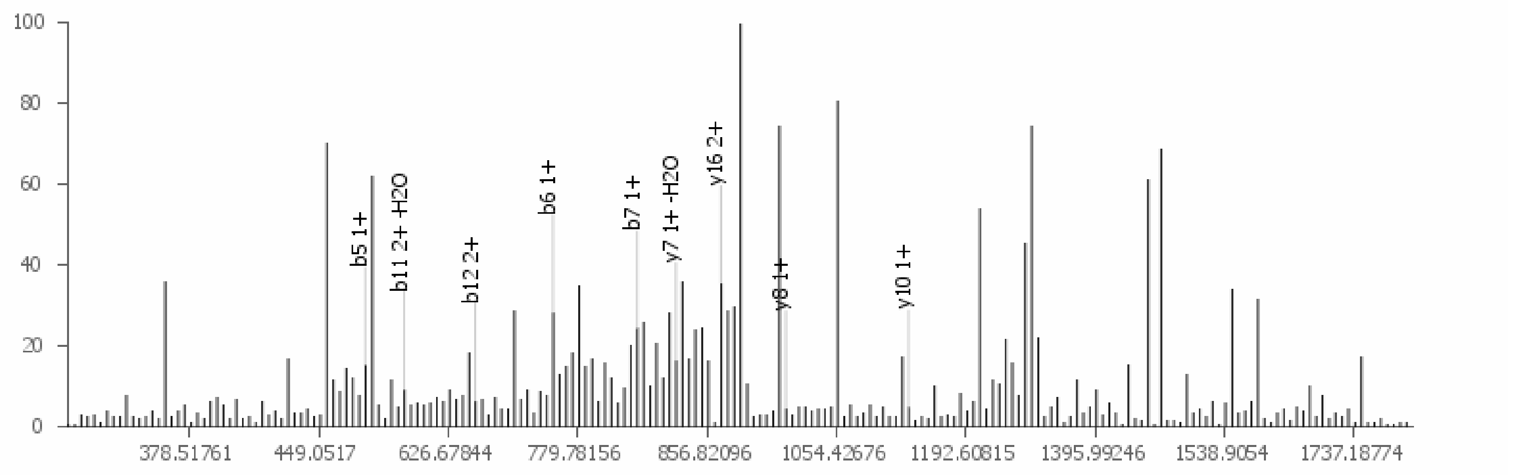

AT1G17730.1 - I(pY)AENAIR - 515.245286 - Charge:2

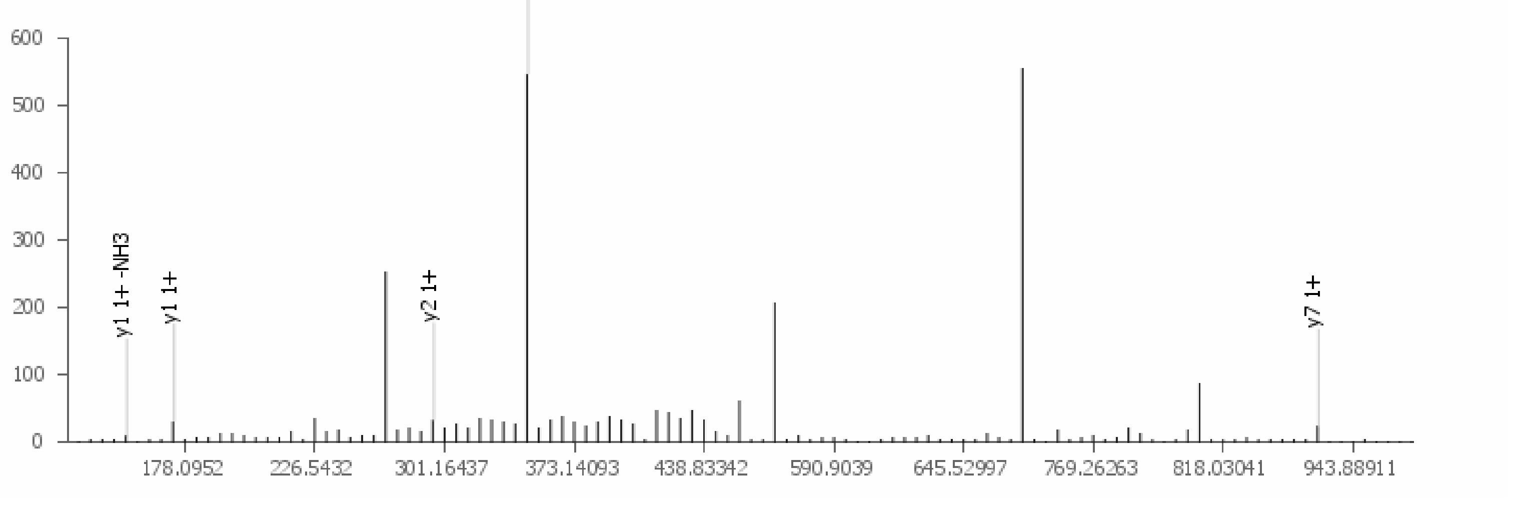

AT1G33475.1 - (pS)GGSQSIER - 500.70403 - Charge:2

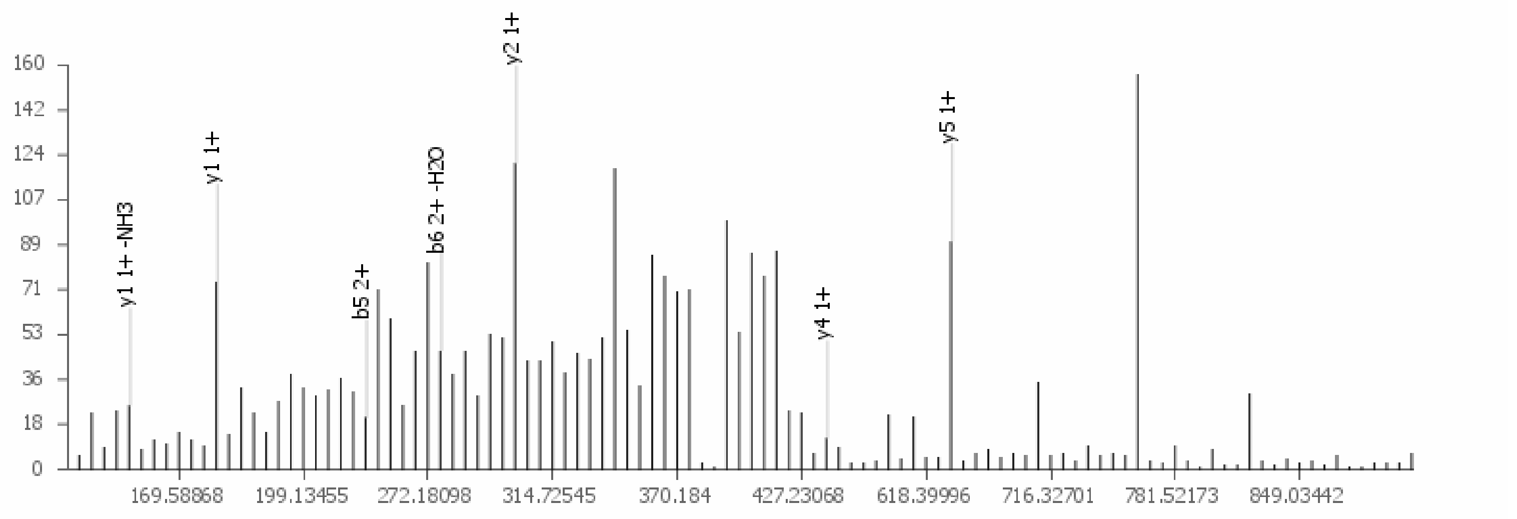

AT5G58760.1 - ML(pY)GMDINSEK - 690.785708 - Charge:2

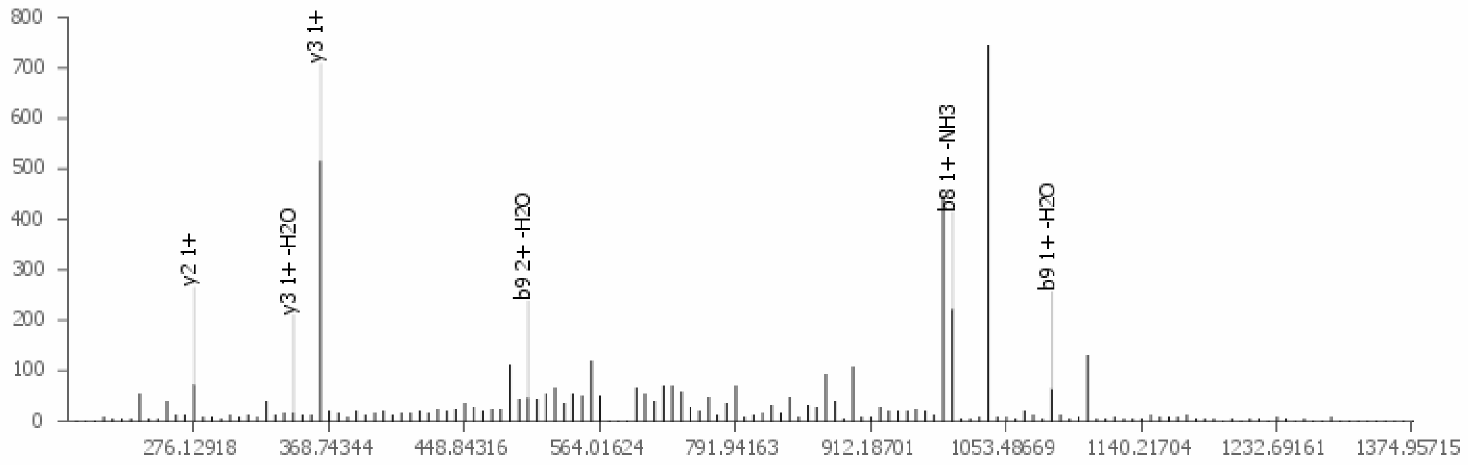

AT4G19910.1 - LVLIPVF(pY)R - 600.331899 - Charge:2

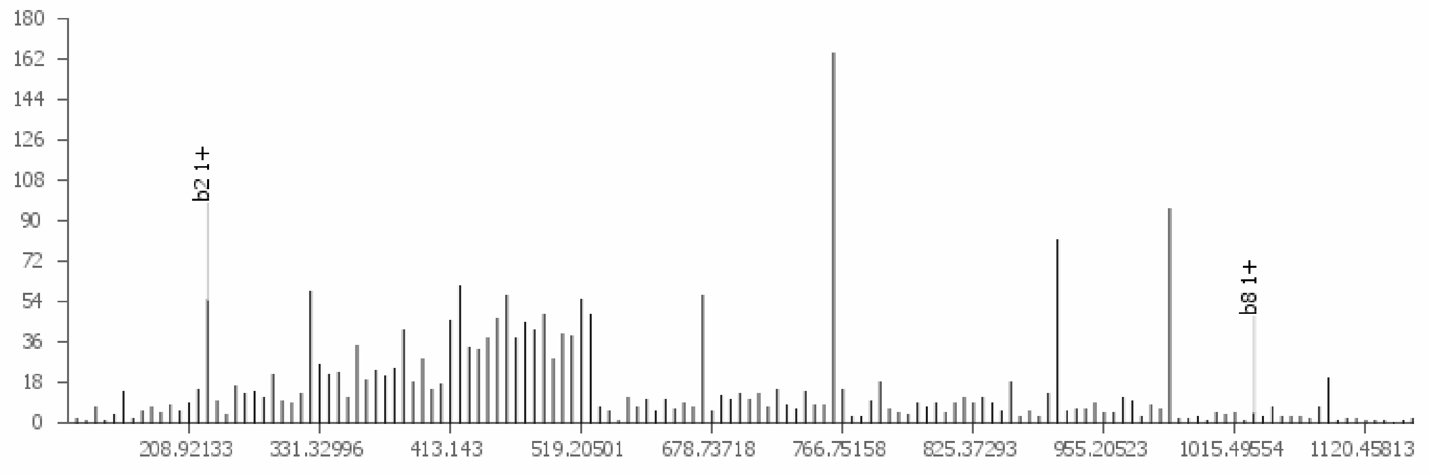

AT4G13510.1 - SP(pS)PSGANTTPTPV - 696.80994 - Charge:2

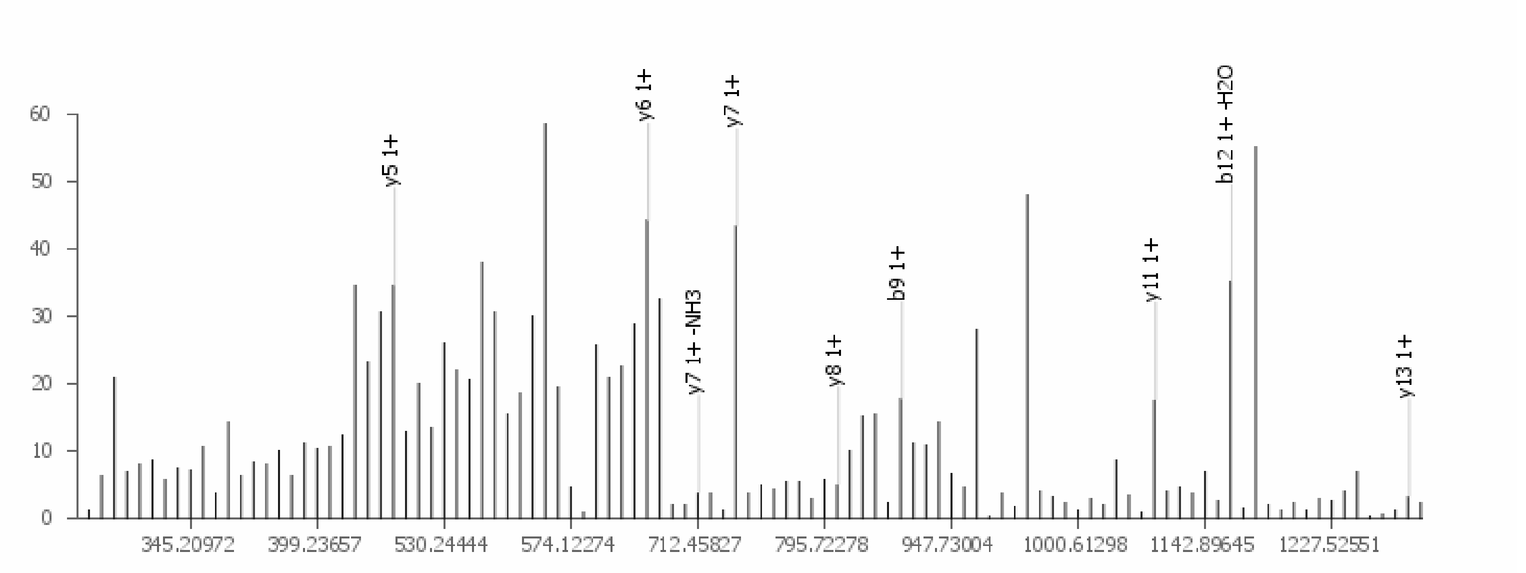

AT5G14900.1 - TNLAN(pT)VLTLKGL(pS)VK - 611.315687 - Charge:3

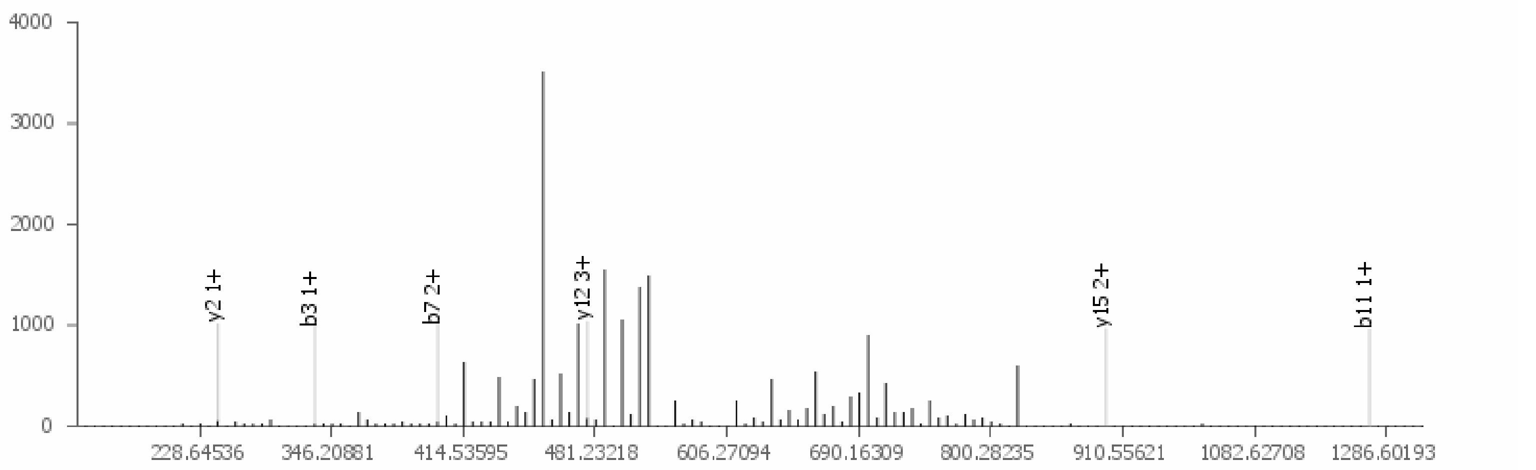

AT5G47800.1 - INNTT(pY)HLHR(pS)CLVPK - 685.97471 - Charge:3

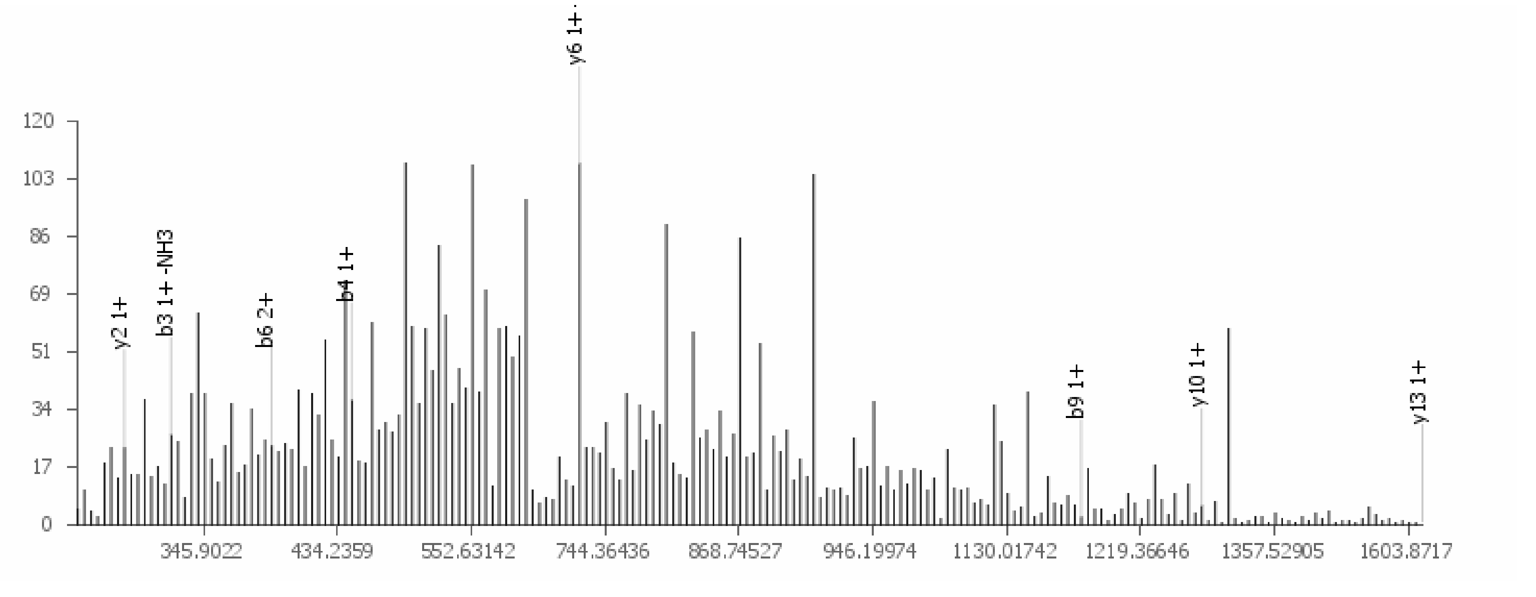

AT5G41790.1 - KSLS(pS)MILEITDELK - 893.946782 - Charge:2

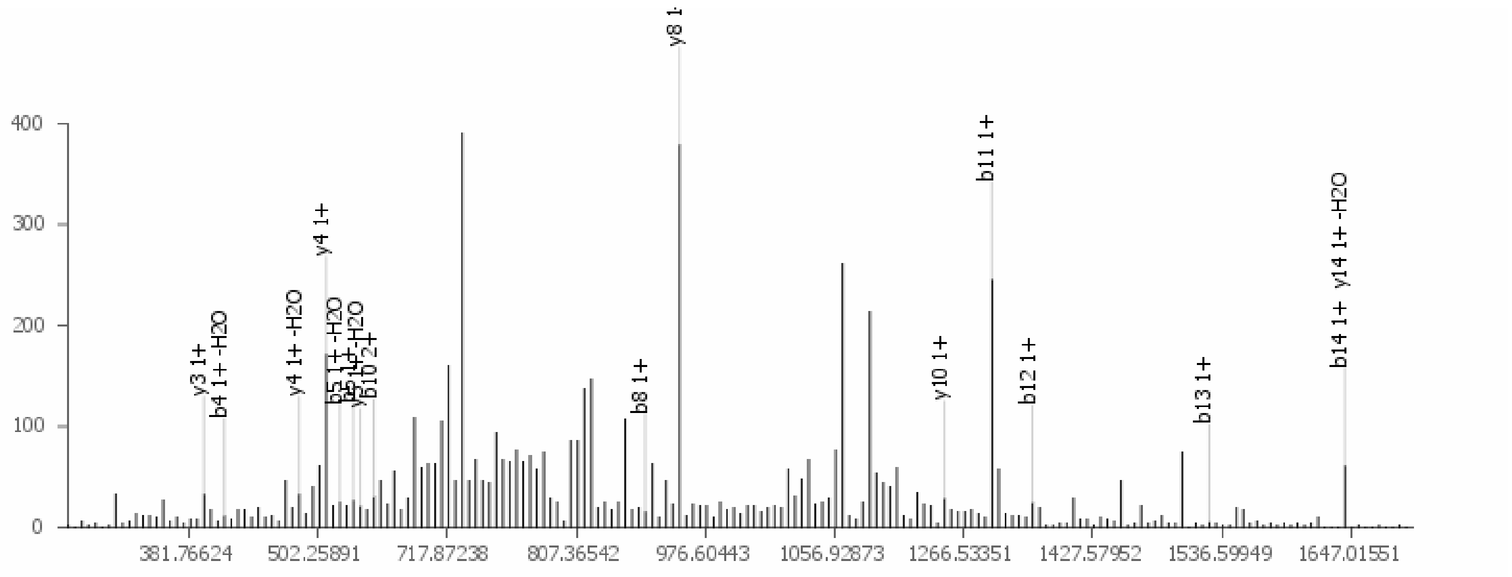

AT3G20810.1 - M(pT)KSLEEASGDFK - 761.833217 - Charge:2

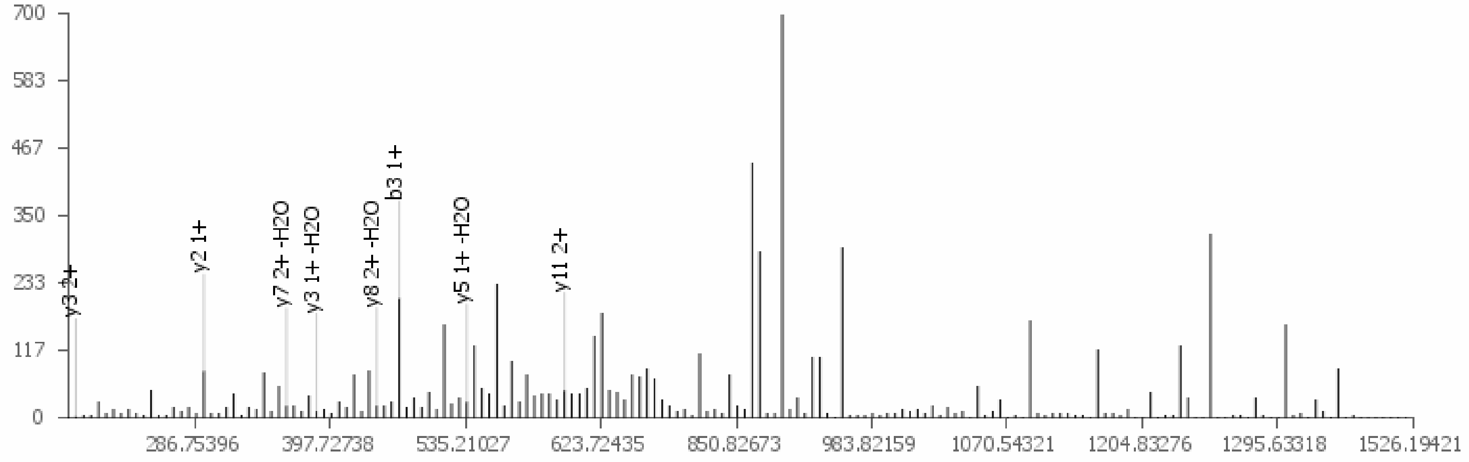

AT5G56960.1 - ASVL(pS)IAR - 448.735021 - Charge:2

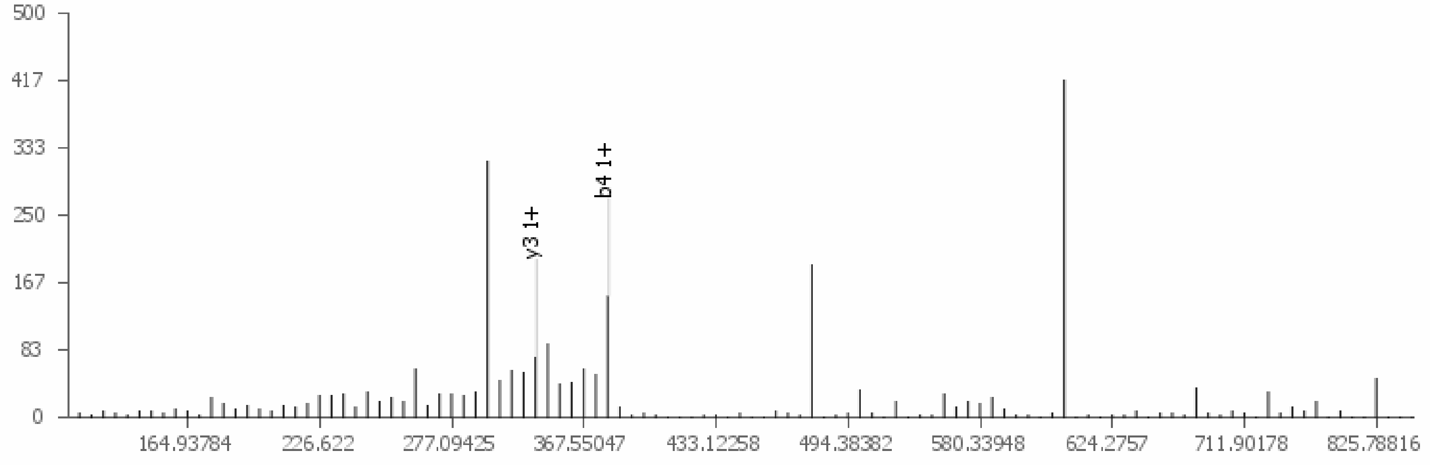

ATMG01290.1 - TTSTSSSI(pY)SKK - 685.320998 - Charge:2

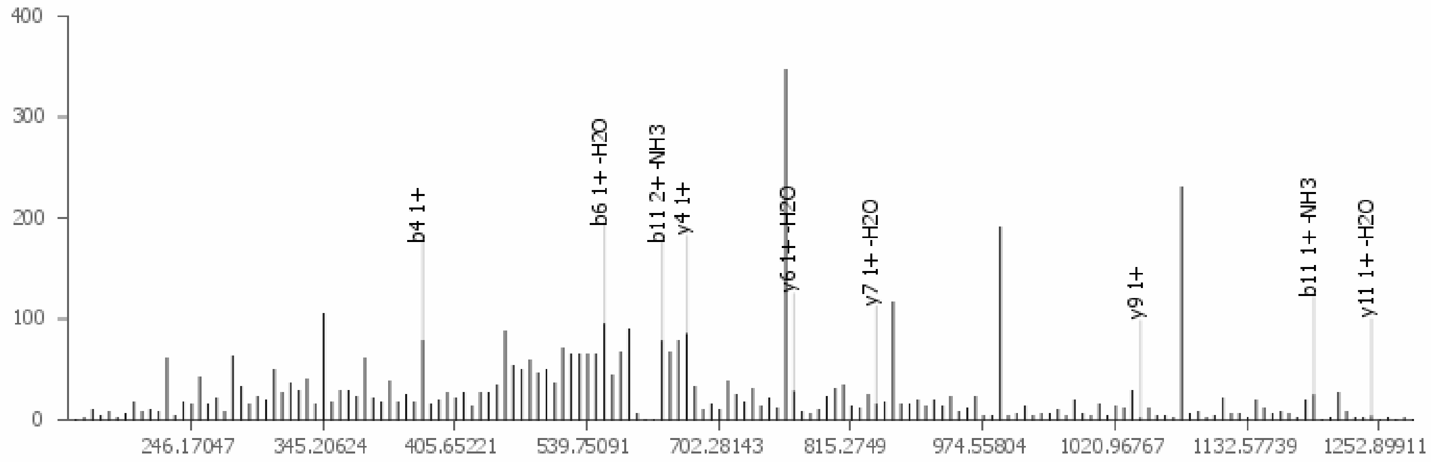

AT2G01820.1 - LLDV(pS)NNDF(pY)GIPPKFR - 1077.985369 - Charge:2

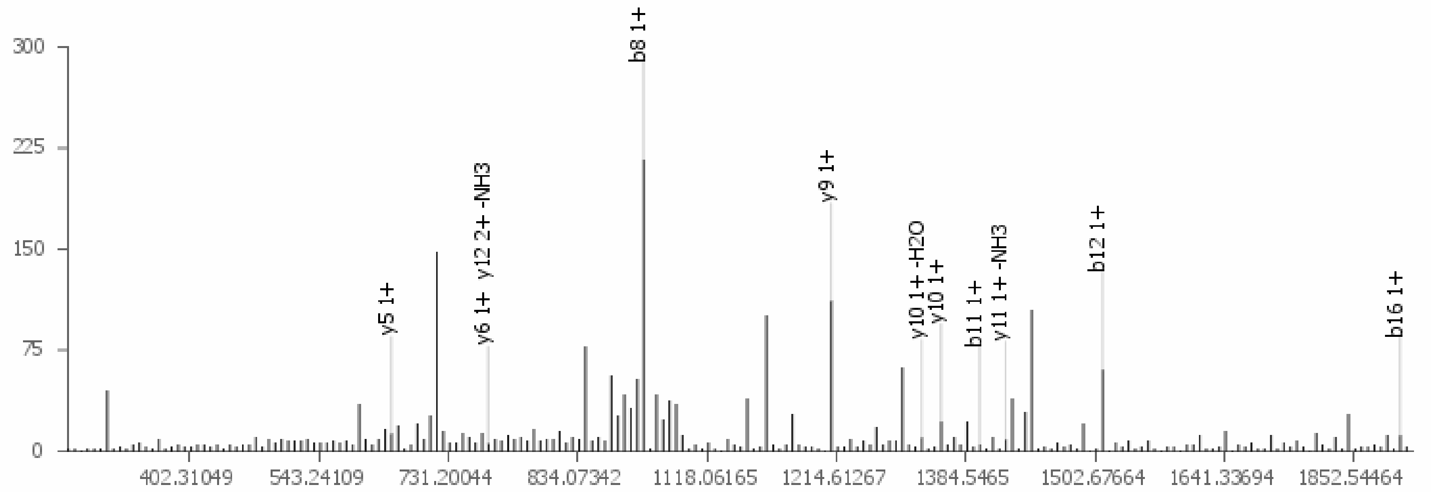

AT1G60730.1 - VELA(pT)KYGIR - 615.316371 - Charge:2

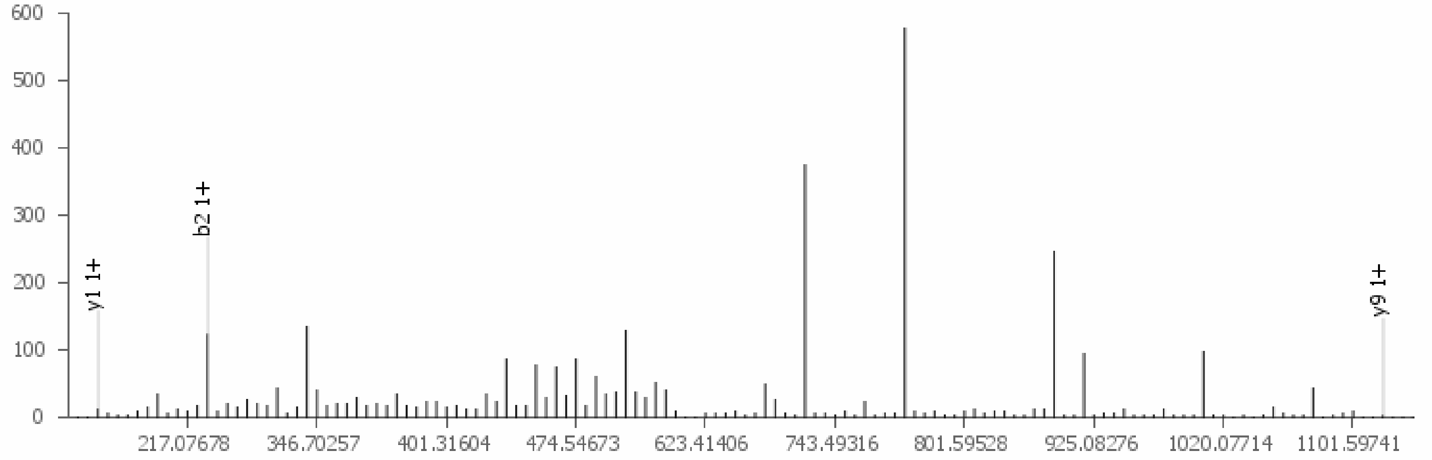

AT5G54140.1 - (pS)L(pT)TNGINWLIK - 760.349063 - Charge:2

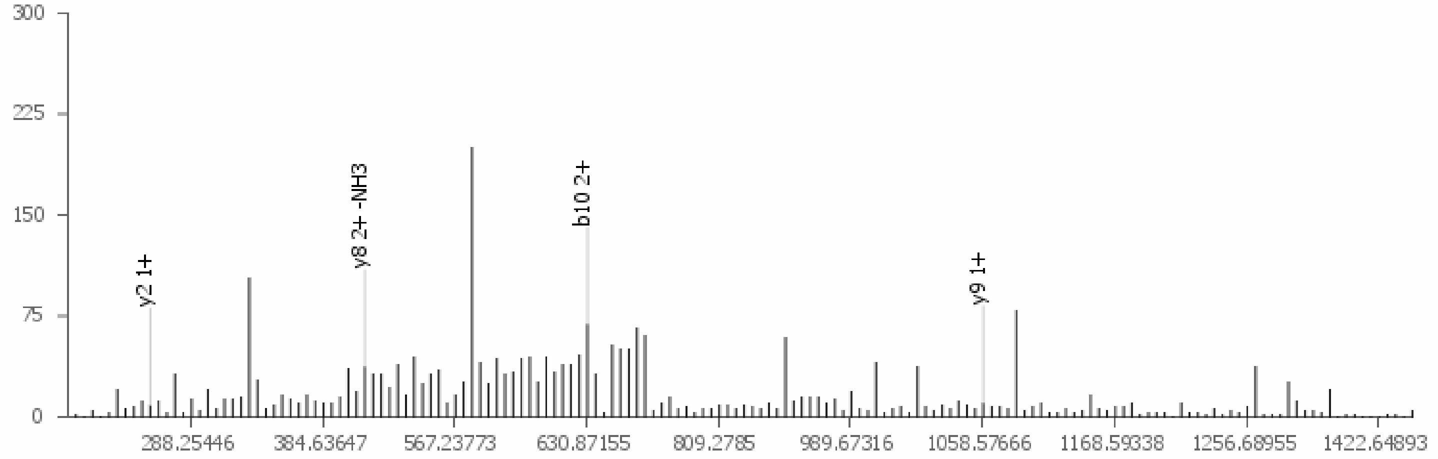

AT4G40020.1 - ENEVV(pS)LKQELLK - 804.909585 - Charge:2

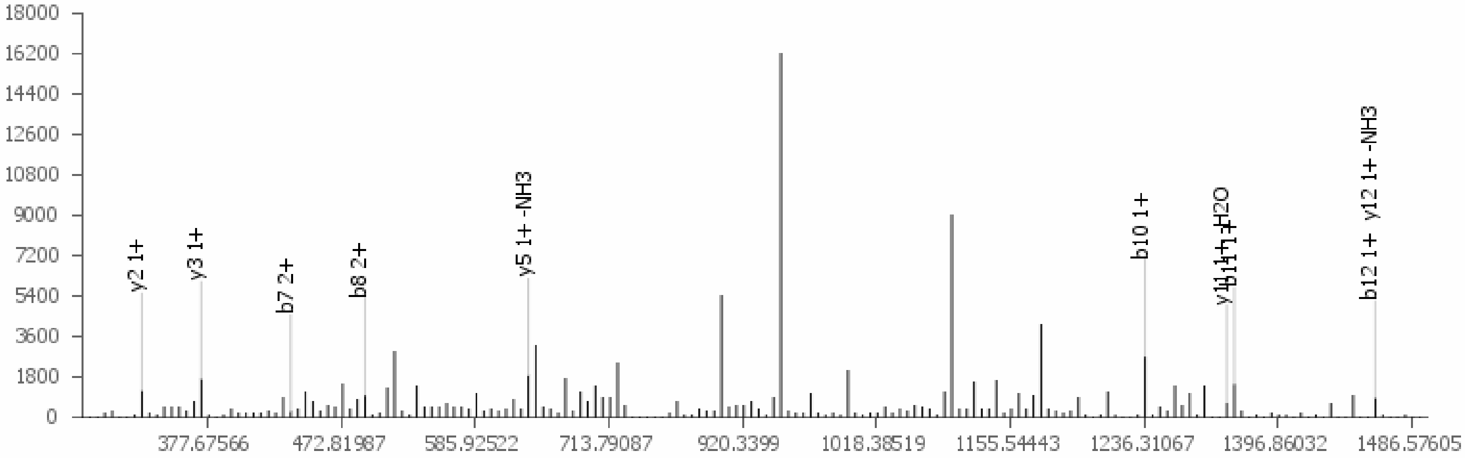

AT2G06140.1 - IDDIKV(t)LF(s)K - 679.849793 - Charge:2

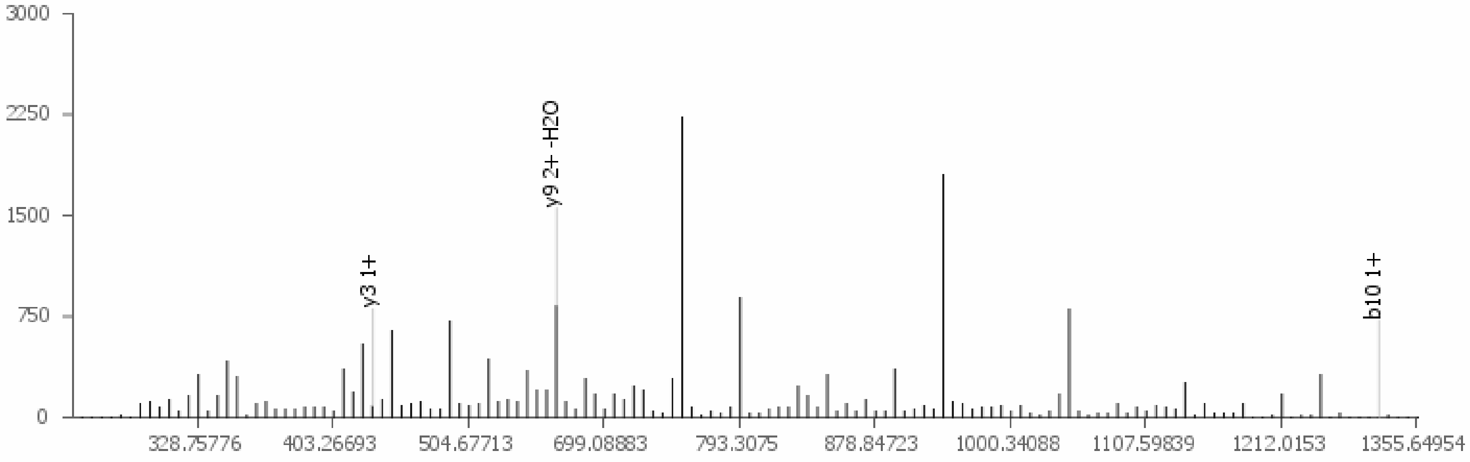

AT2G40130.2 - SVTTDLNLRV(pS)(pS)VTTGSGLK - 1098.031615 - Charge:2

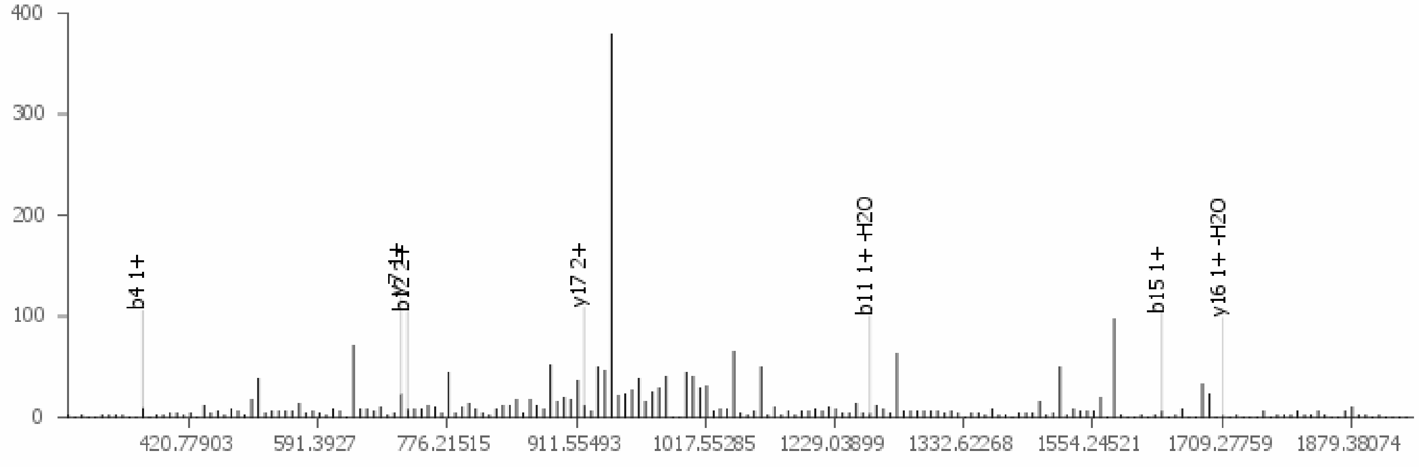

AT1G33490.1 - I(pT)GGPHFPLTSDALKK - 881.44849 - Charge:2

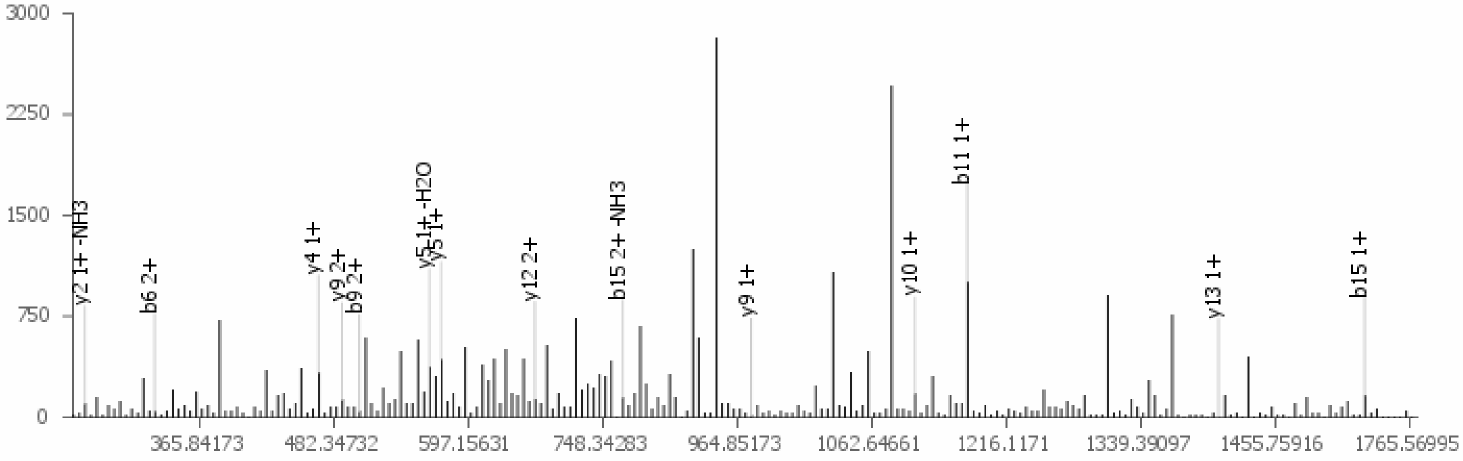

AT3G05910.1 - QLQFTGIL(pS)DK - 665.331718 - Charge:2

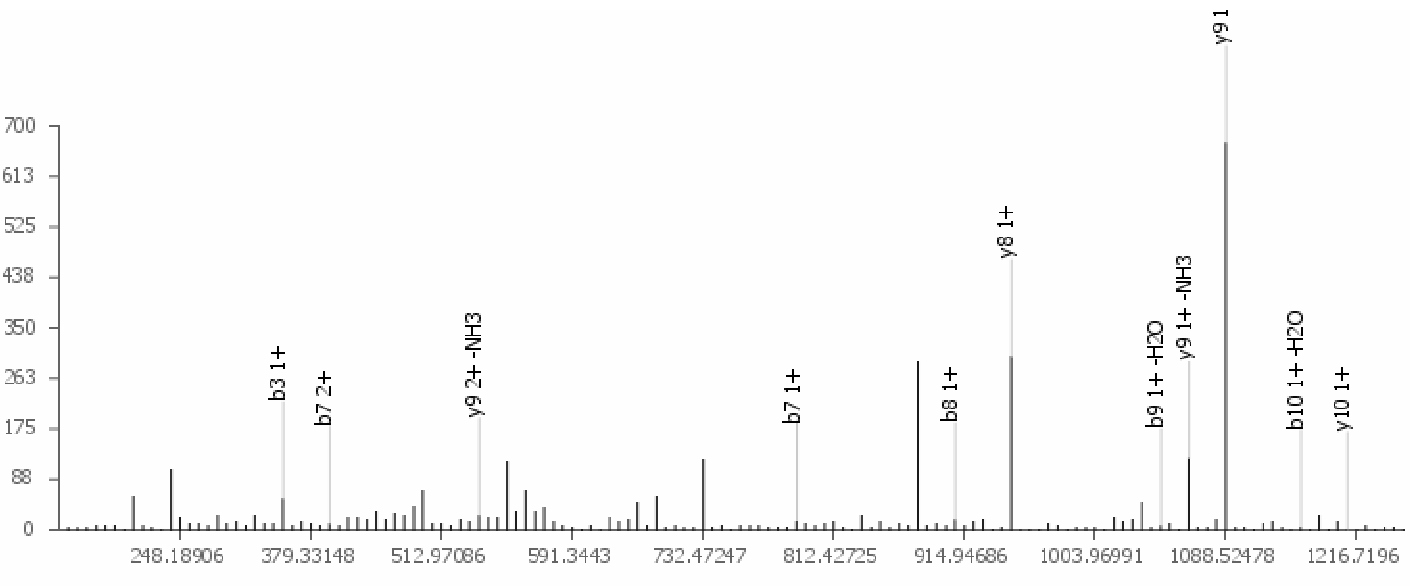

AT5G23790.1 - VK(s)A(y)PLVVATLPDVPEEHR - 767.396392 - Charge:3

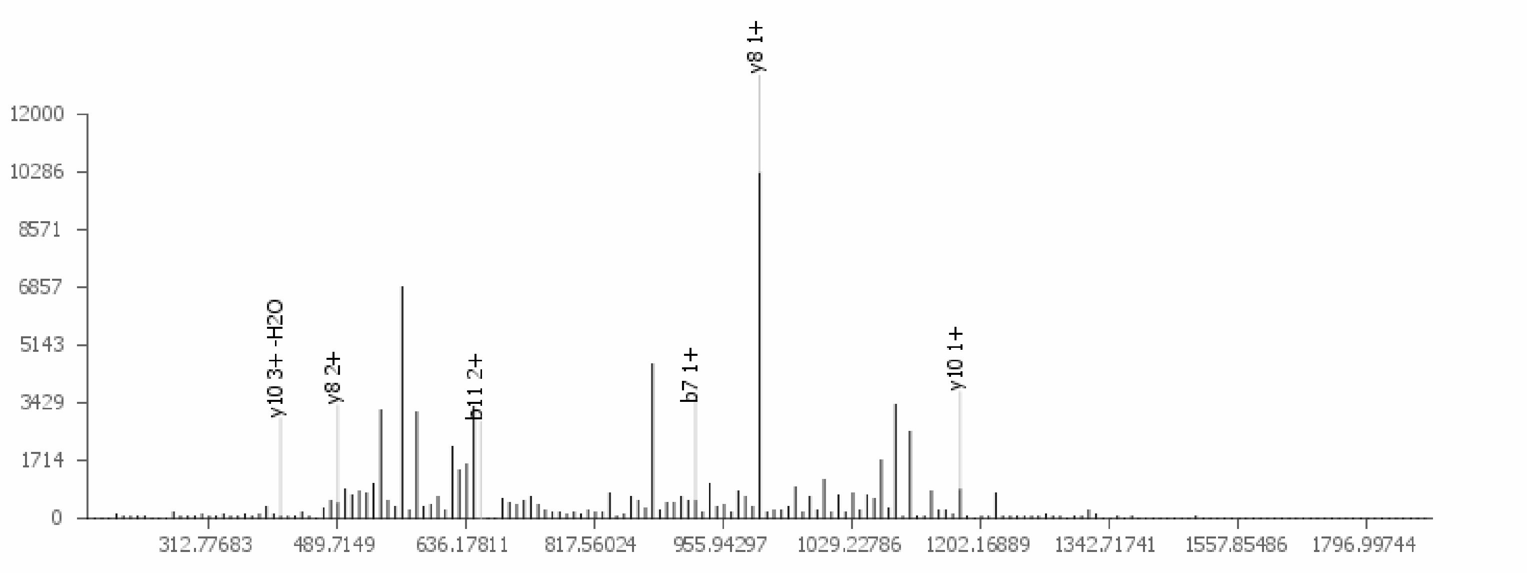

AT3G63400.1 - YH(pT)PSPERSPPR - 752.334509 - Charge:2

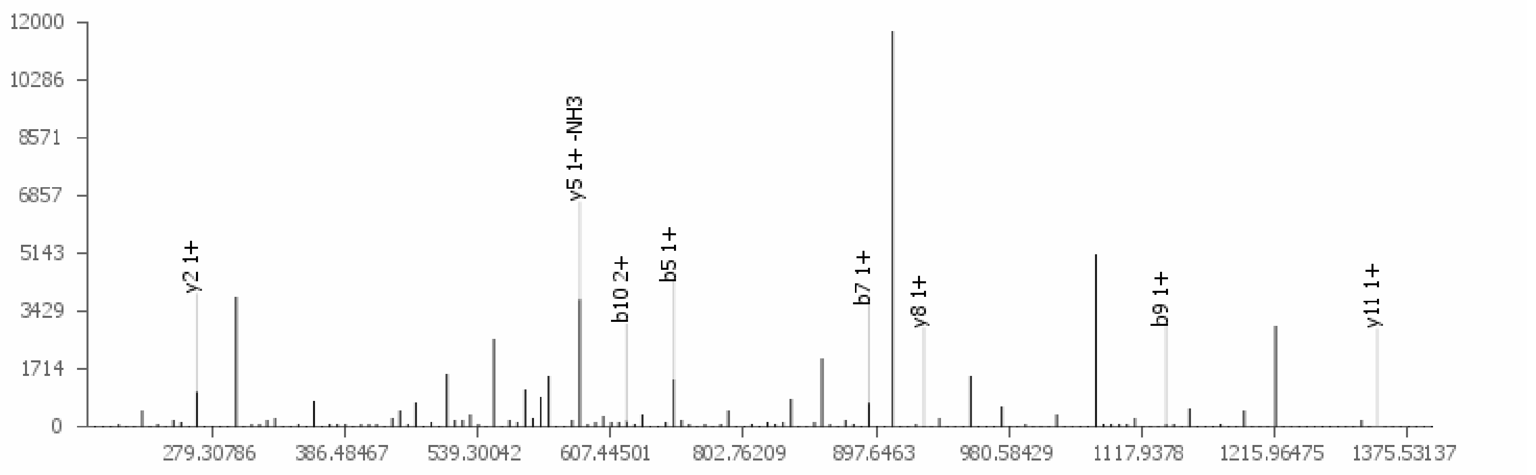

AT4G03100.1 - (pT)LITKTLAER - 613.327804 - Charge:2

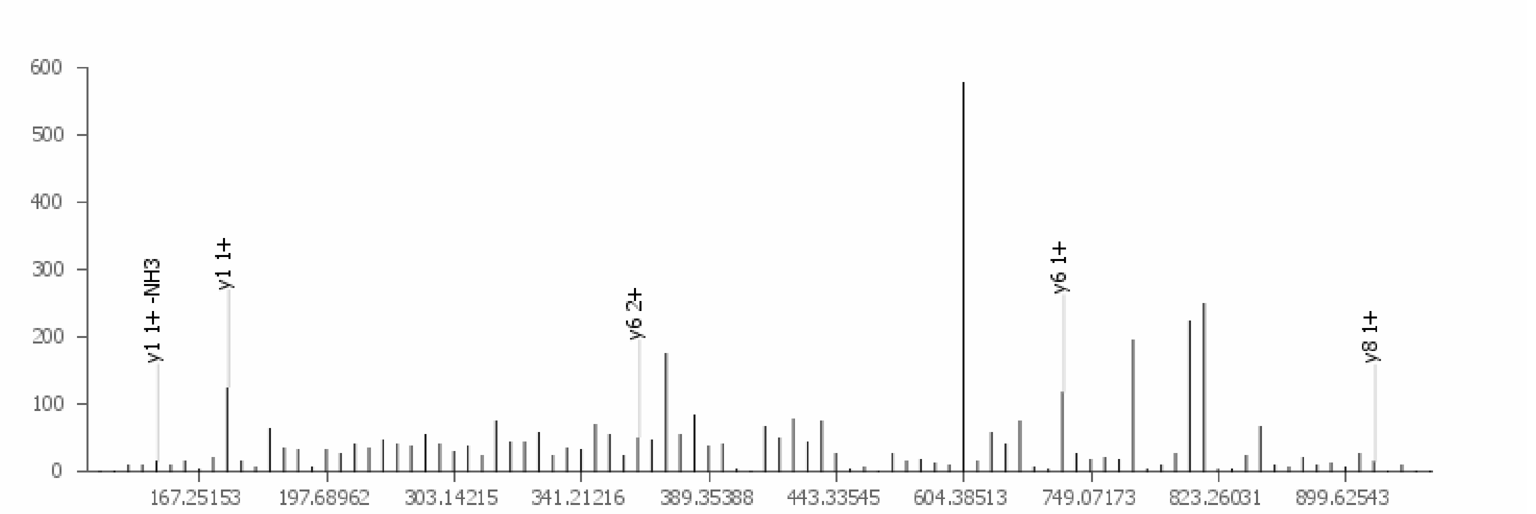

AT1G80150.1 - DPEKL(pY)NLFK - 673.821907 - Charge:2

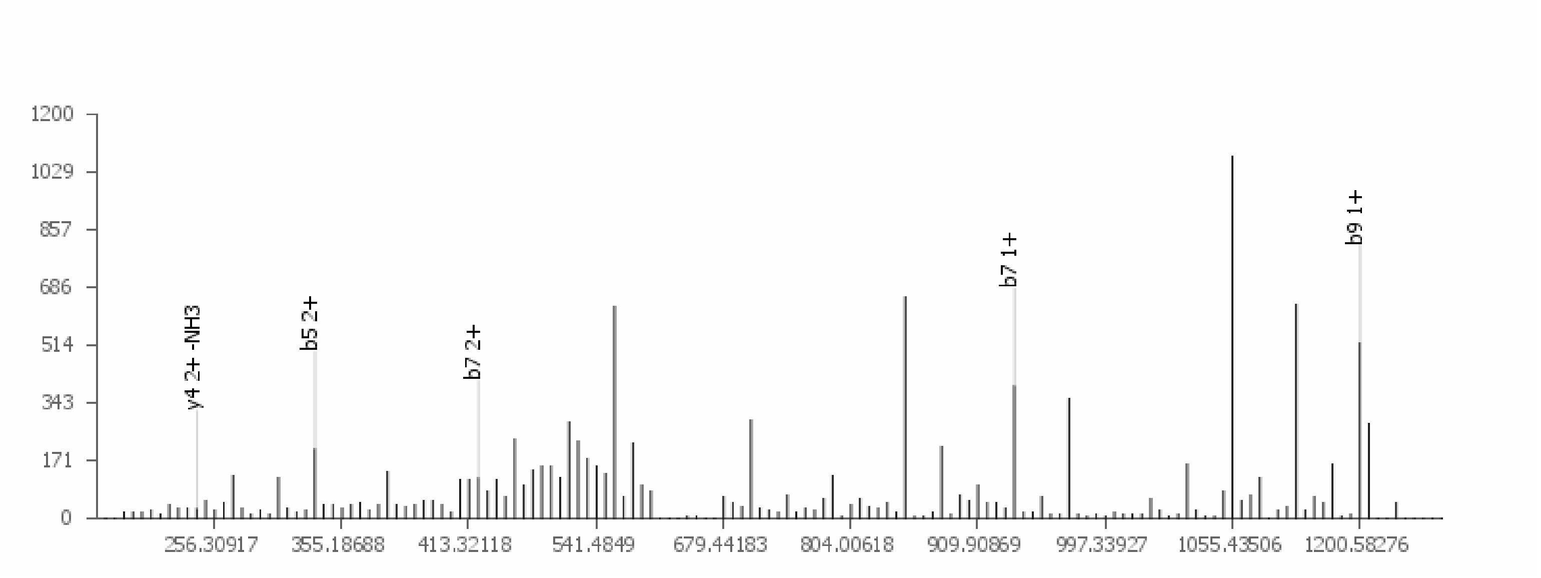

AT2G34590.1 - SLKPFDL(y)(t)IGNSVK - 881.448597 - Charge:2

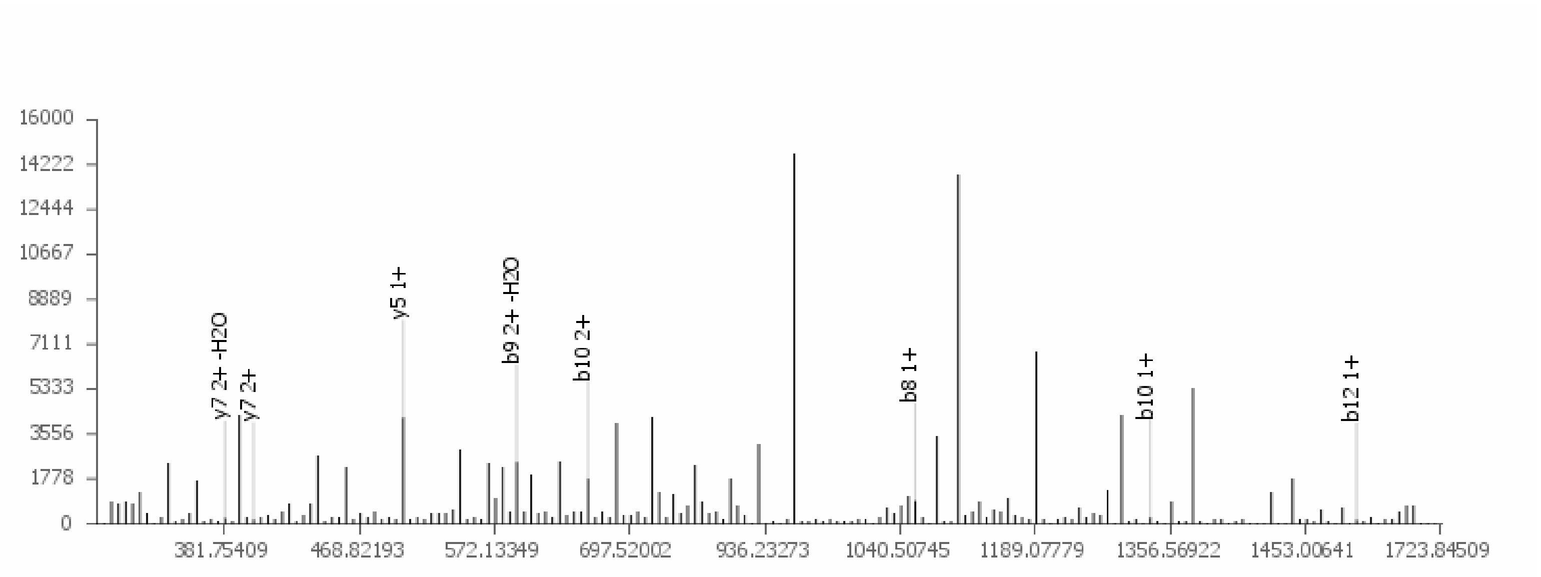

ATCG01130.1 - KP(pS)FFEPISKELK - 815.418047 - Charge:2

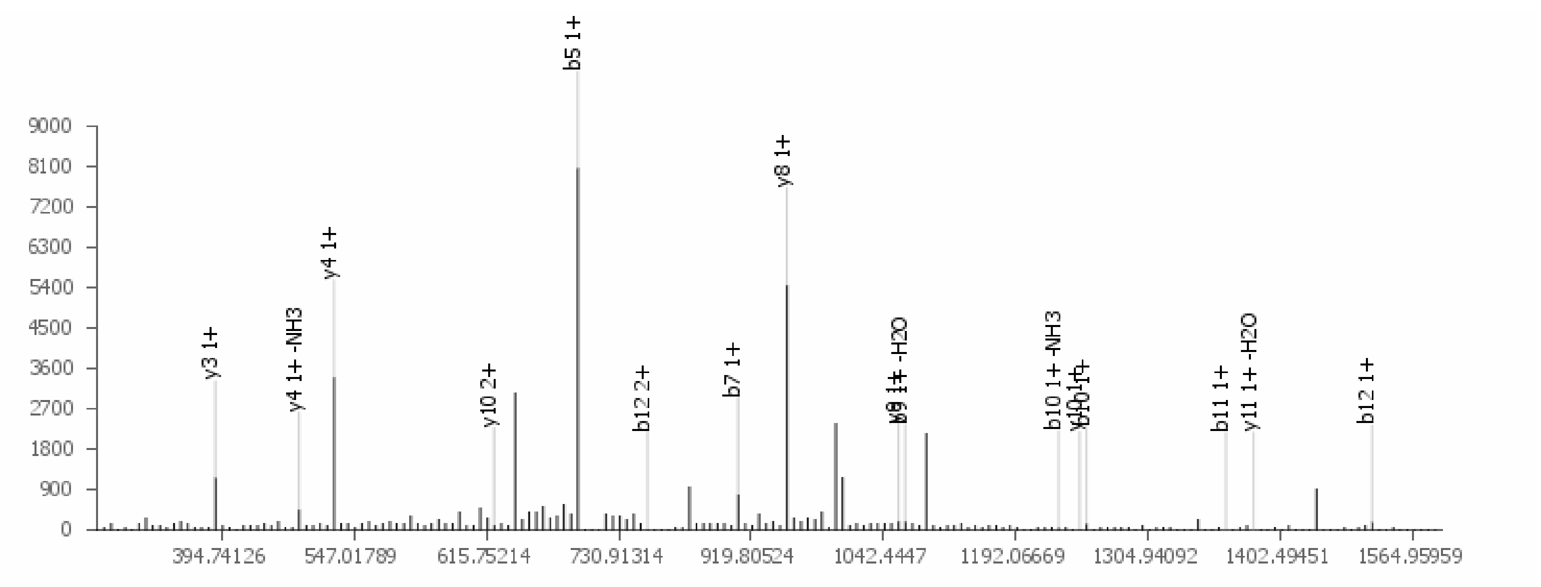

AT3G26010.1 - RLFV(pS)VHR - 547.28278 - Charge:2

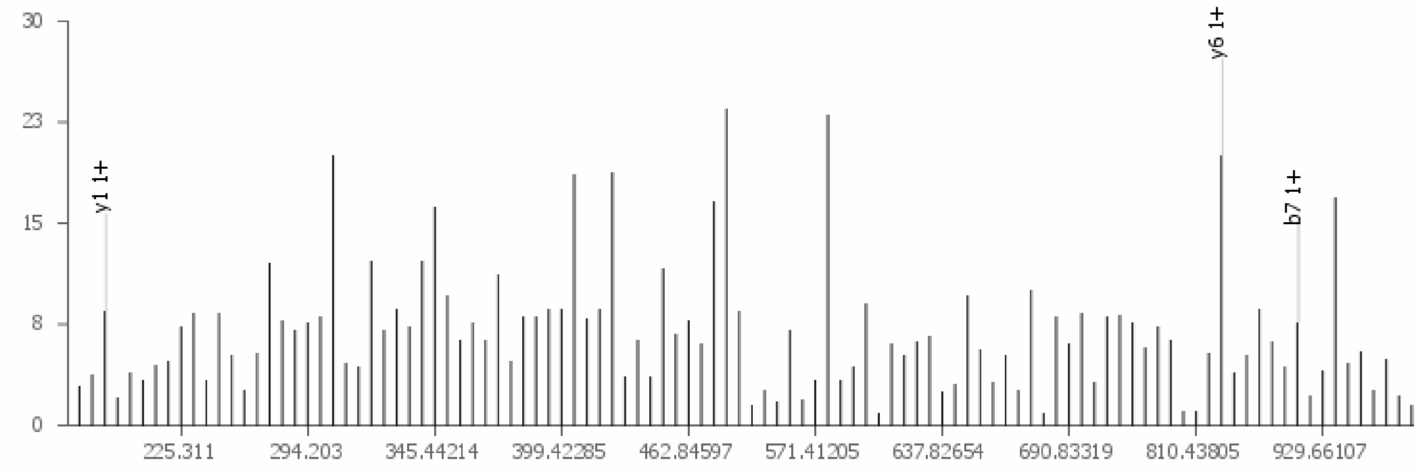

AT1G69920.1 - (t)(t)(oxM)AQNG(s)N(t)TVK - 724.80879 - Charge:2

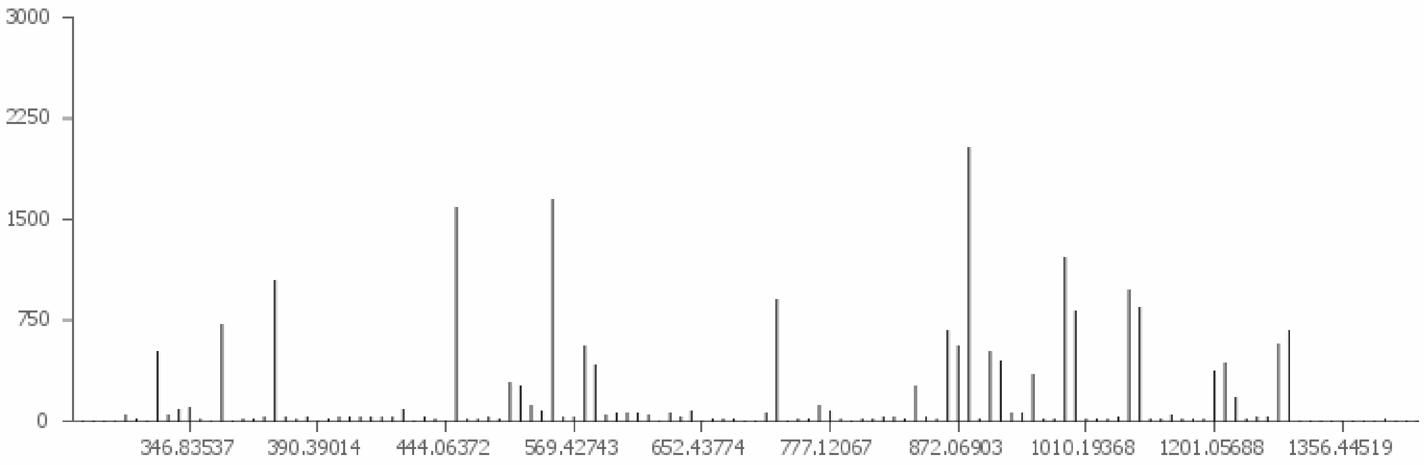

AT5G60590.2 - GESSILEK(s)LNPGIG(t)IGVR - 703.030538 - Charge:3

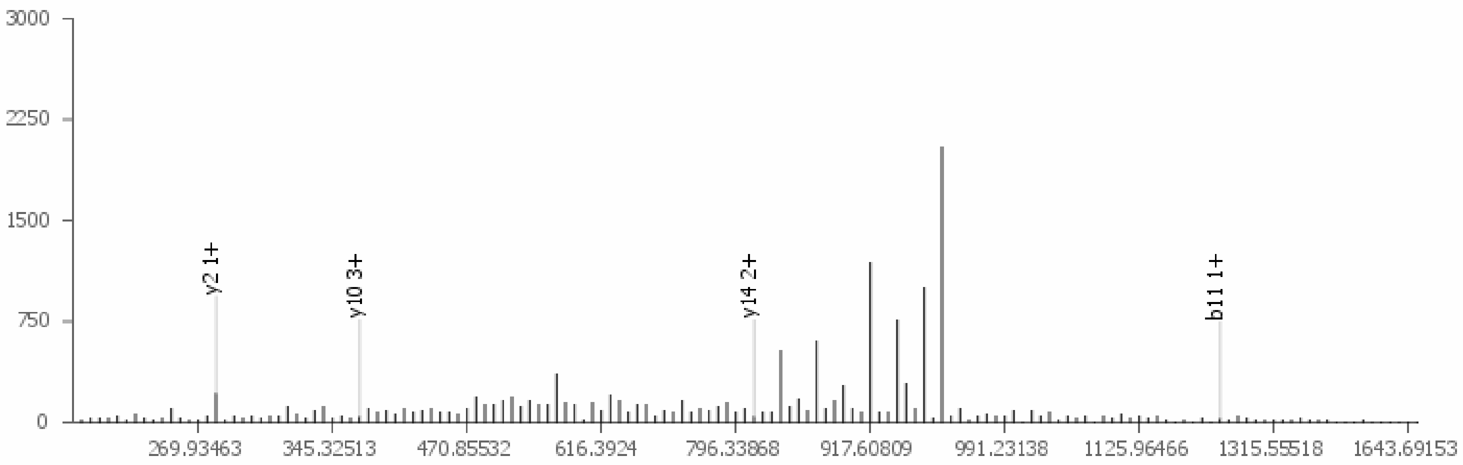

AT2G38940.1 - S(pT)CHGISAASGK - 599.746515 - Charge:2

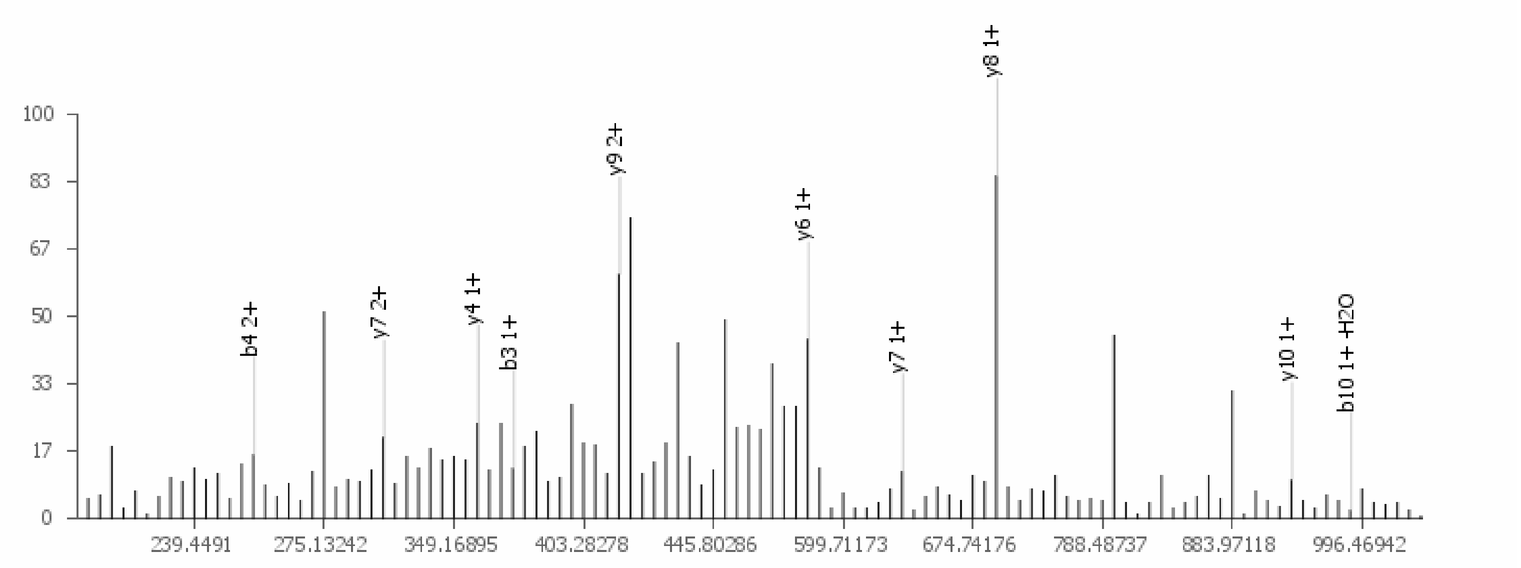

AT3G17340.1 - EILVPLV(pS)VLHRLIDK - 962.547051 - Charge:2

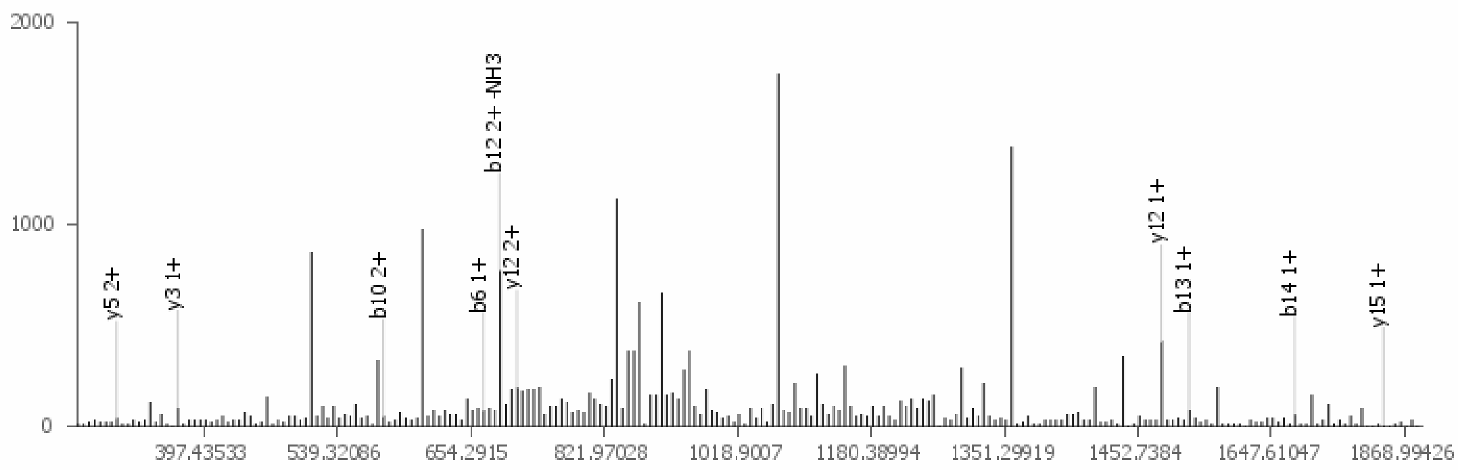

AT2G38770.1 - DVPAR(pY)LLR - 591.810249 - Charge:2

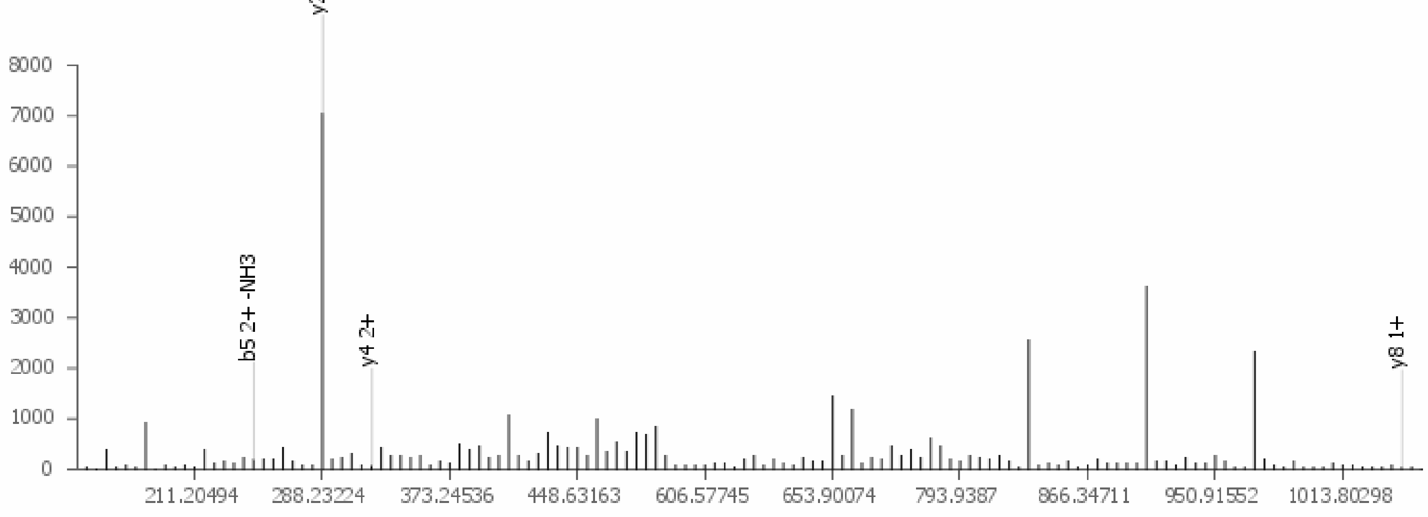

AT1G33530.1 - (oxM)(pT)FSIEKLVDLK - 760.37835 - Charge:2

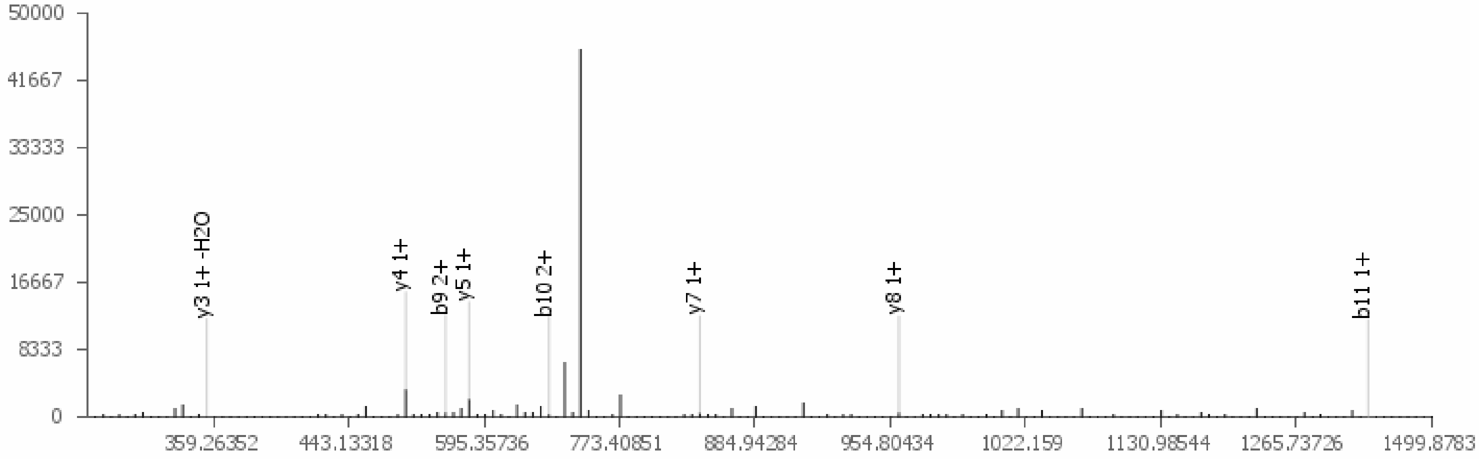

AT3G02270.1 - EVDG(pT)FLR - 508.721206 - Charge:2

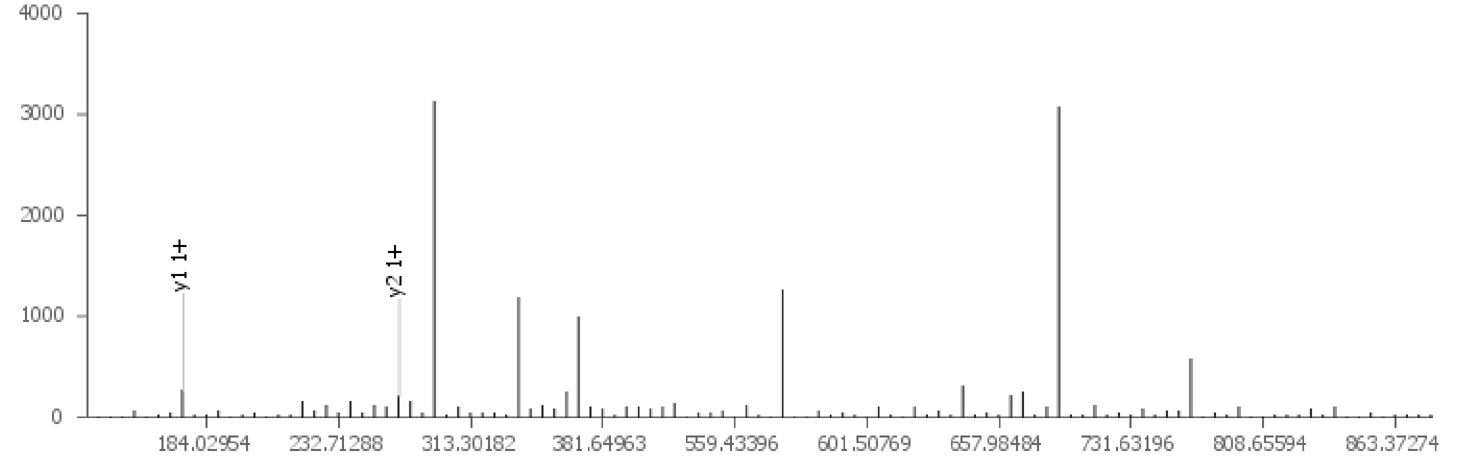

AT1G10560.1 - HCKTNGIVLAGI(pS)R - 774.887401 - Charge:2

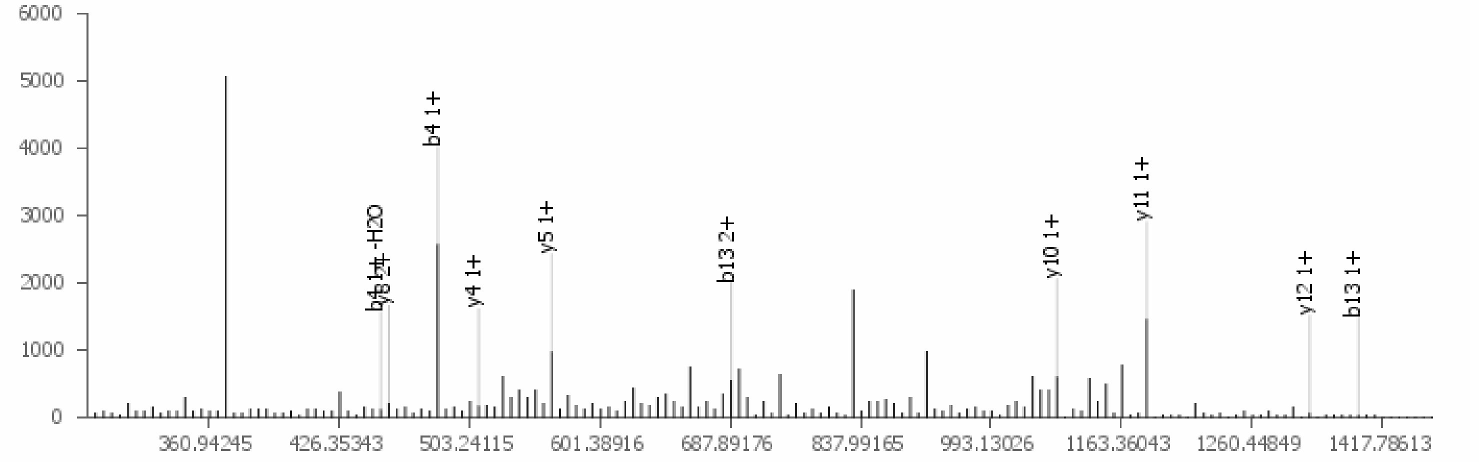

AT5G06270.1 - (pS)PKLELK - 894.476864 - Charge:1

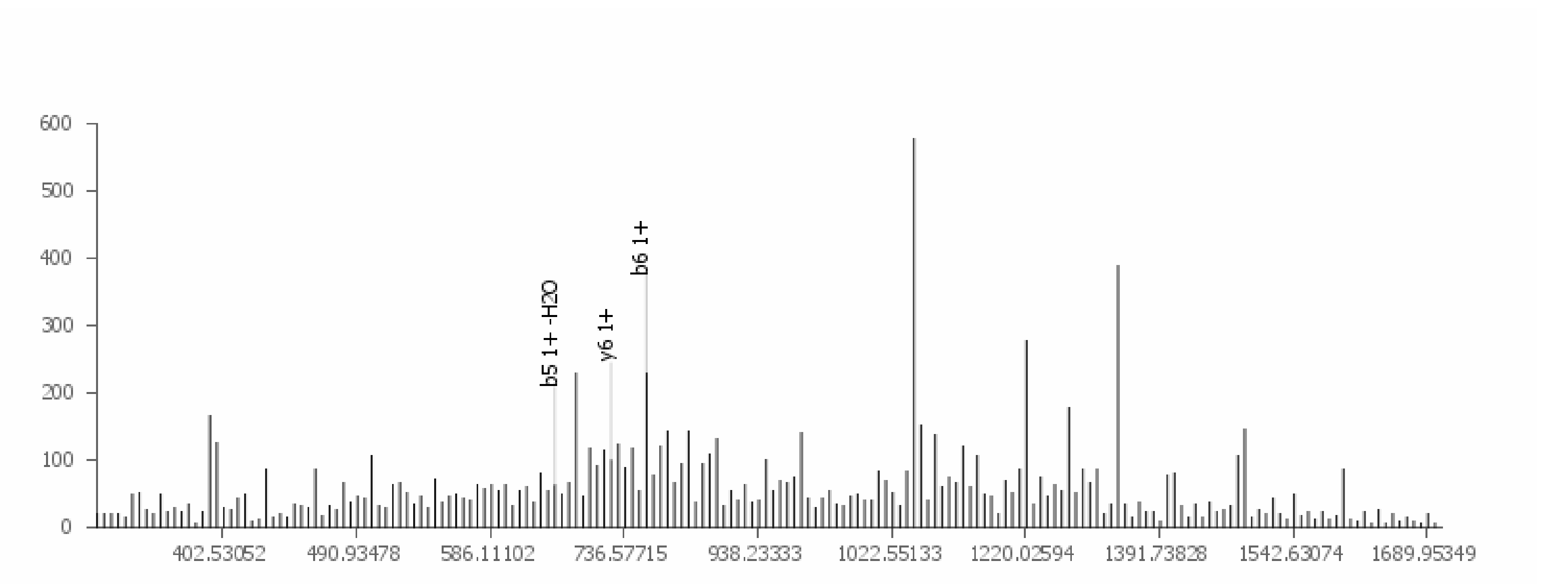

AT1G10420.1 - FKLQLQ(pS)MIGEVR - 814.90784 - Charge:2

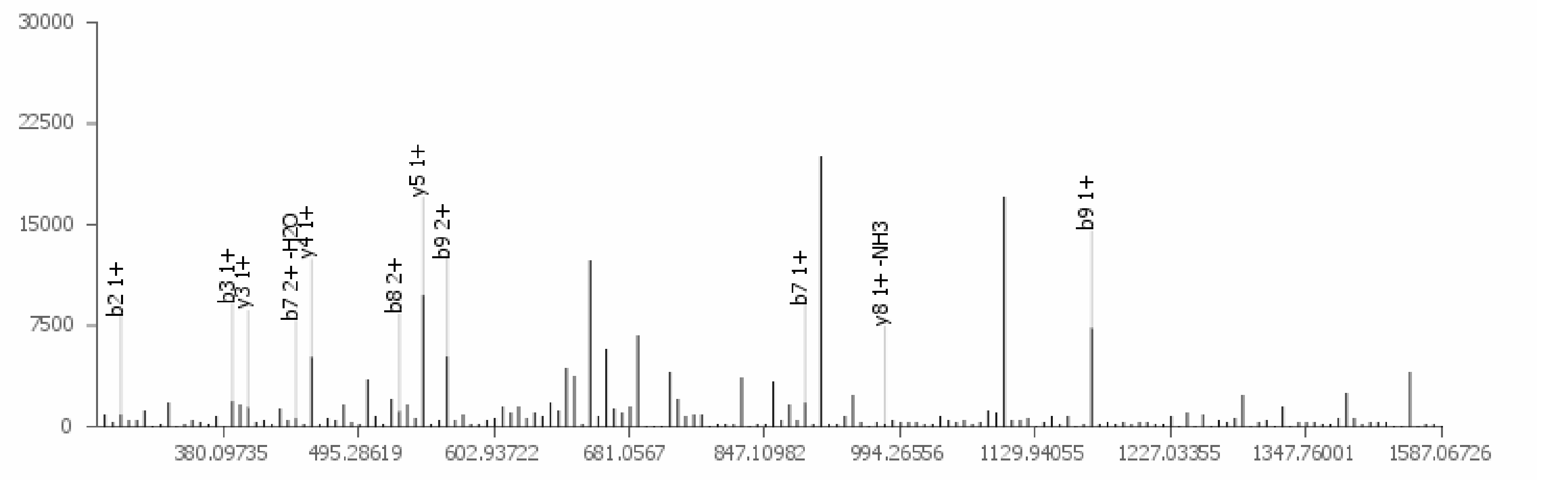

AT2G38940.1 - AVMA(pT)LCFFR - 619.782872 - Charge:2

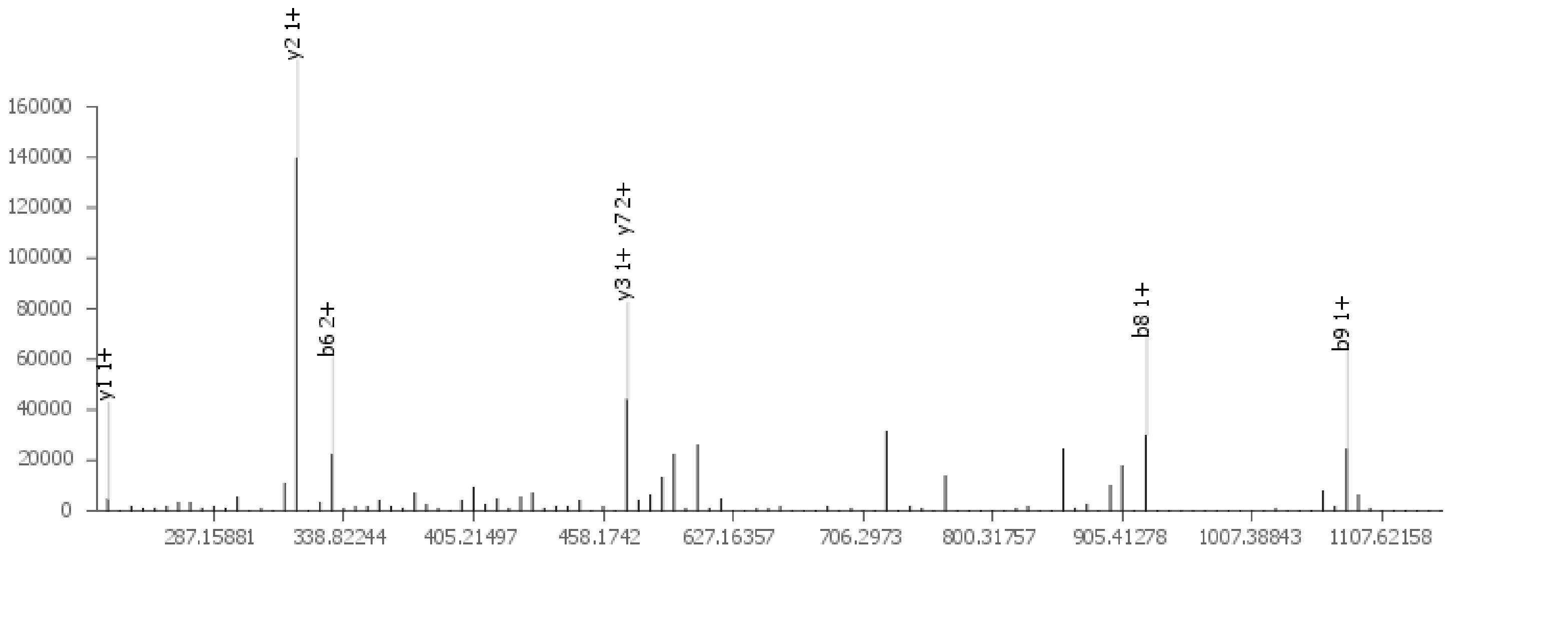

AT1G60160.1 - I(pS)MDFMRELGSTLGTIR - 1003.975561 - Charge:2

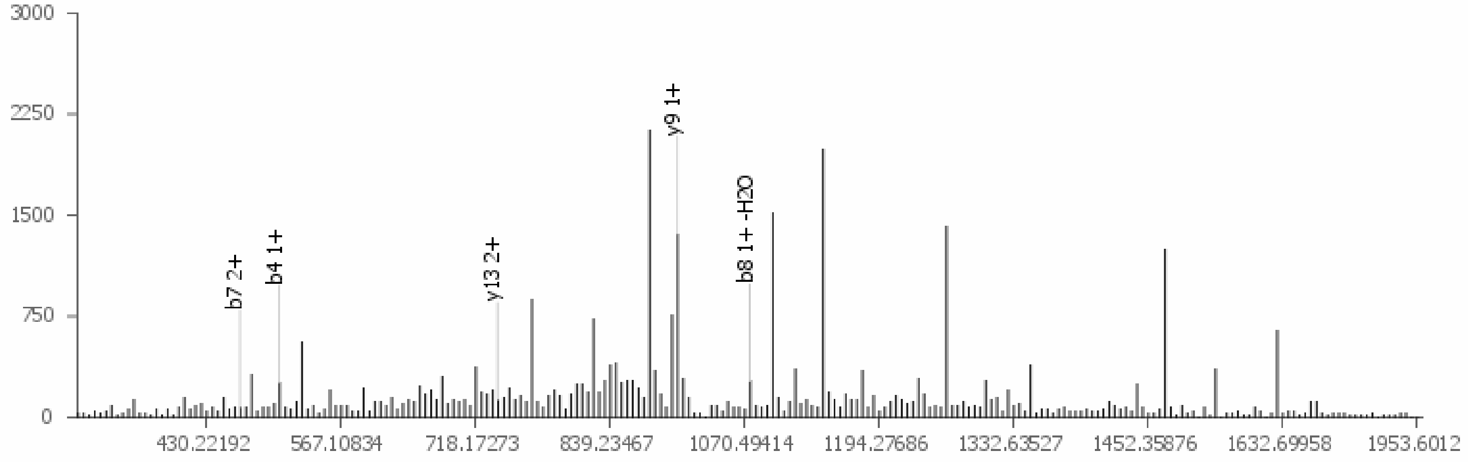

AT5G58150.1 - LSALH(pY)LNLSR - 683.842438 - Charge:2

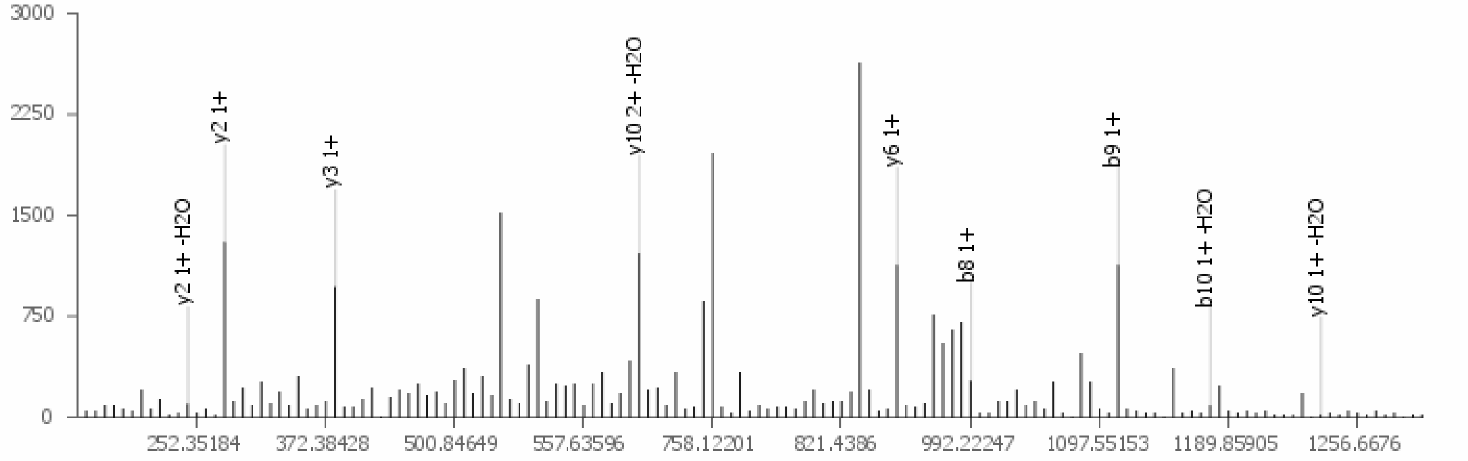

AT2G28050.1 - EAQMLI(pT)IVVK - 662.853759 - Charge:2

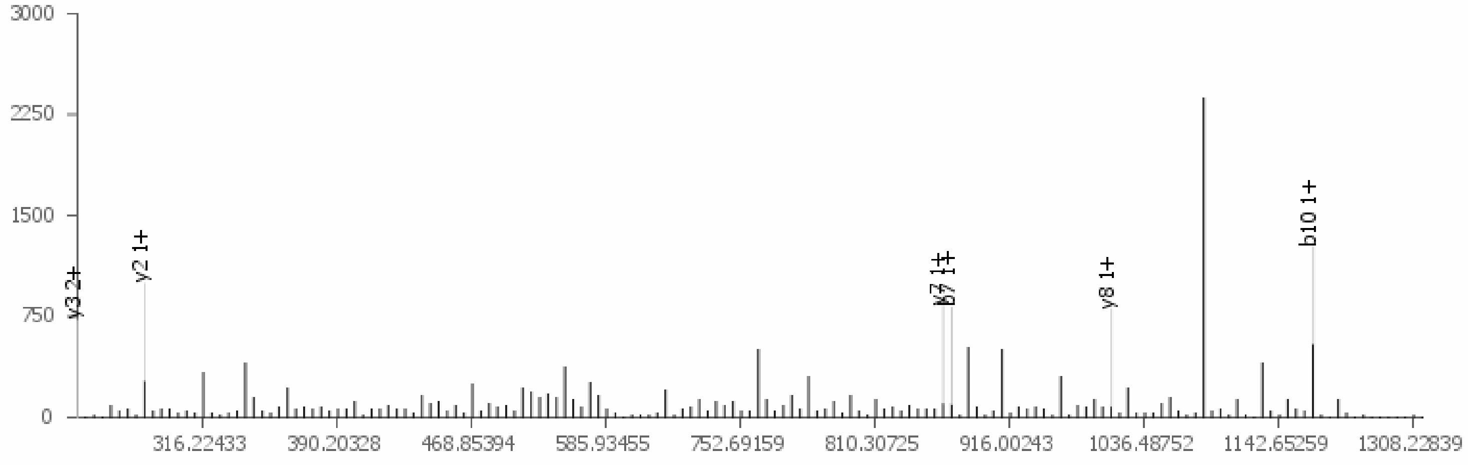

AT5G58150.1 - VLDLSSNNLTGHVPML(pS)VK - 1052.531693 - Charge:2

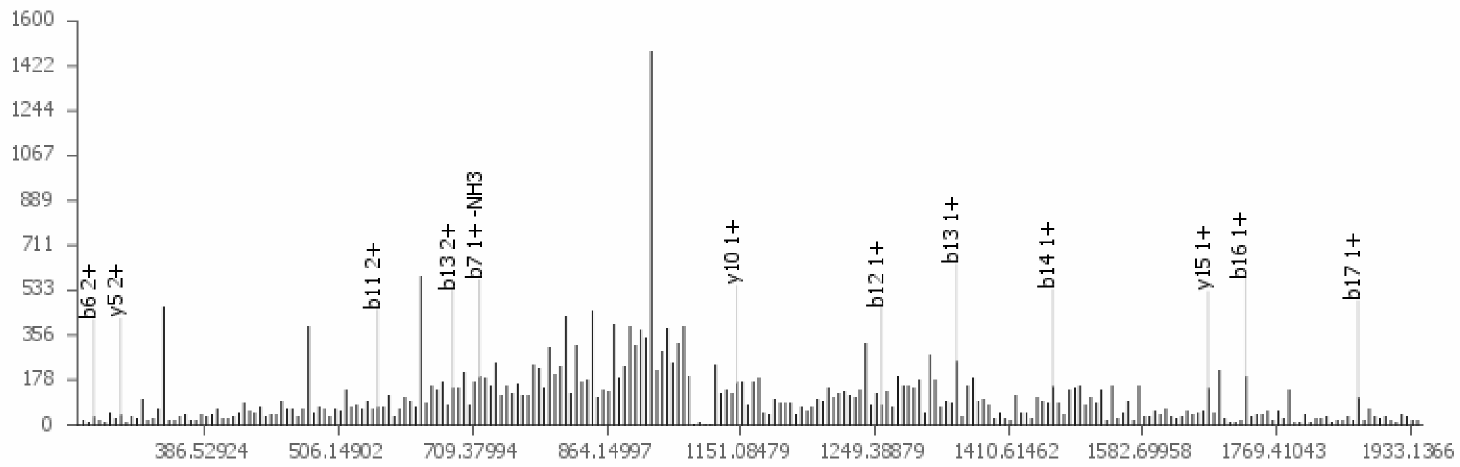

AT1G05790.1 - YV(pS)RDGLPR - 571.775749 - Charge:2

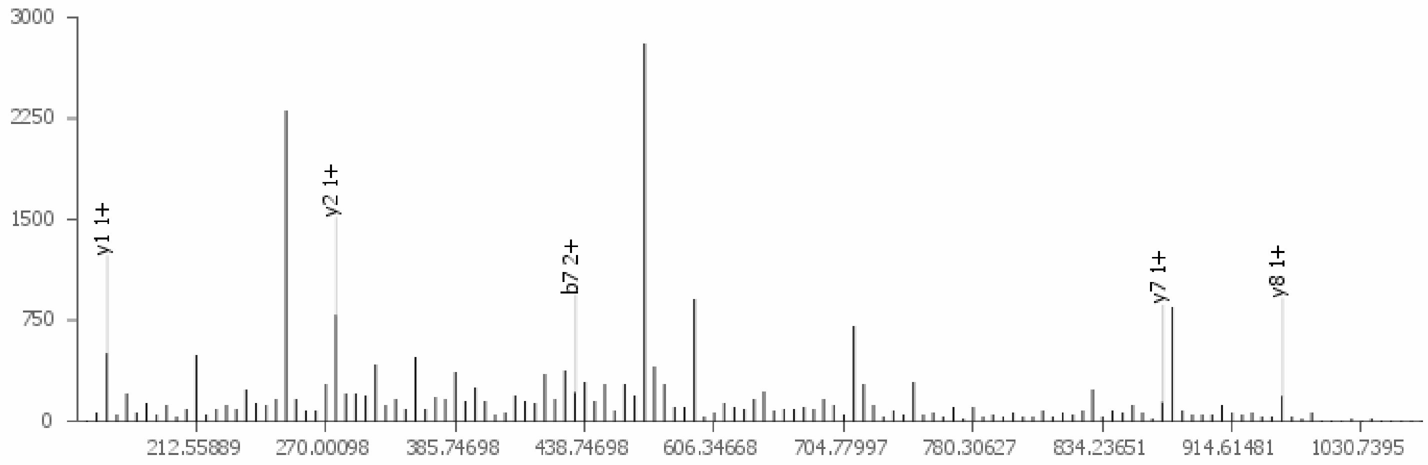

AT5G48460.1 - SVNWSLVTNGV(pT)DEEKK - 993.464507 - Charge:2

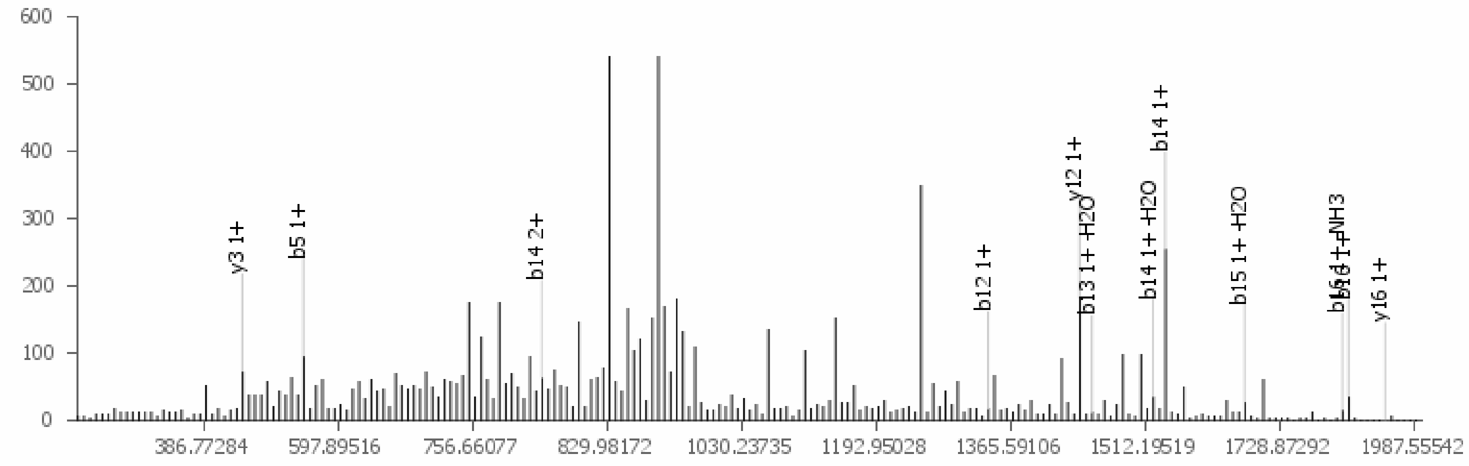

AT2G35790.1 - I(pS)IGPQEK - 476.23228 - Charge:2

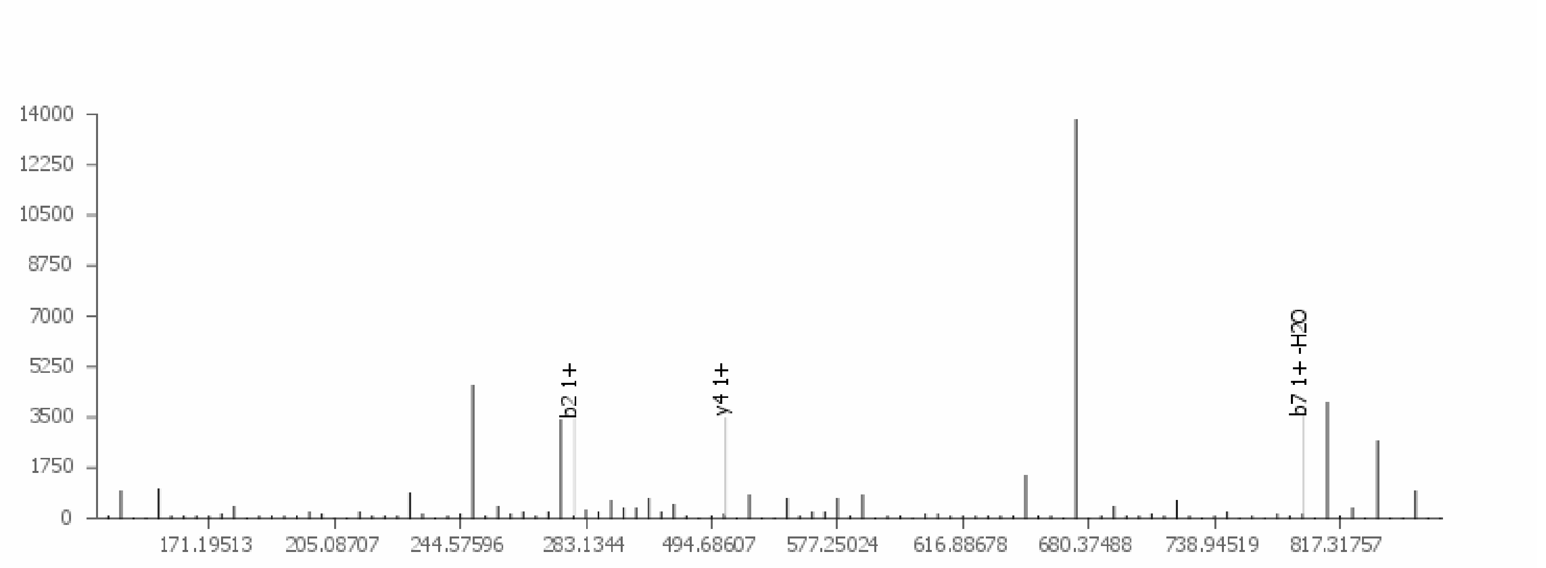

AT1G10520.1 - LGLK(pY)FDDIK - 646.322881 - Charge:2

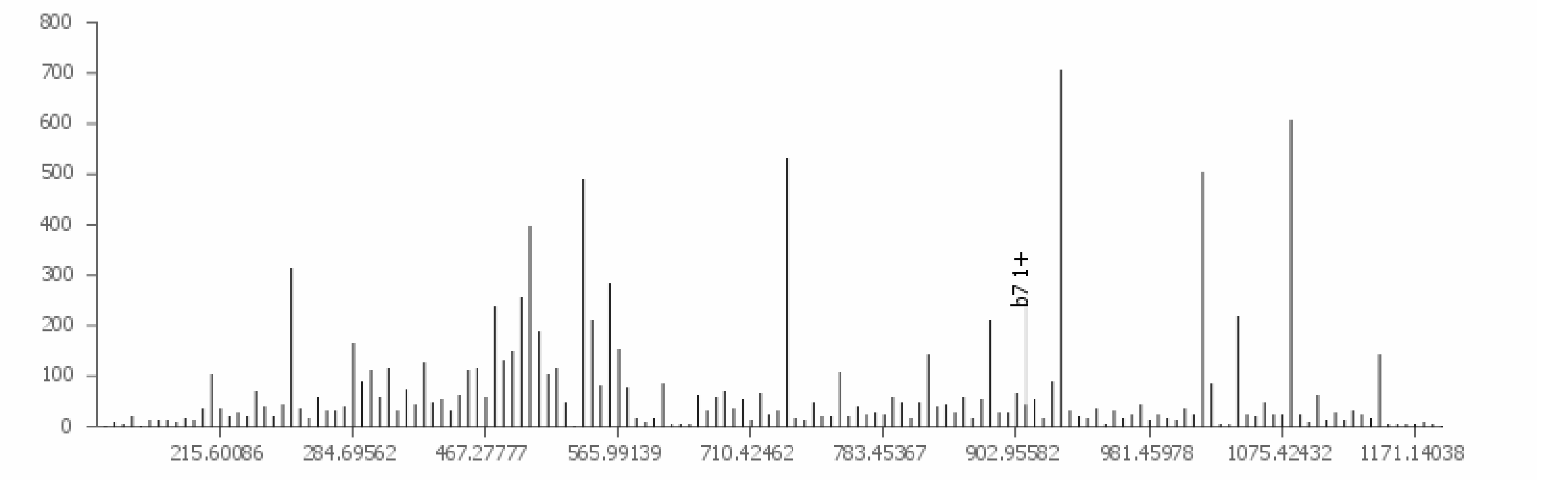

AT4G17140.1 - DIVK(oxM)P(pS)GDVRPVK - 818.907447 - Charge:2

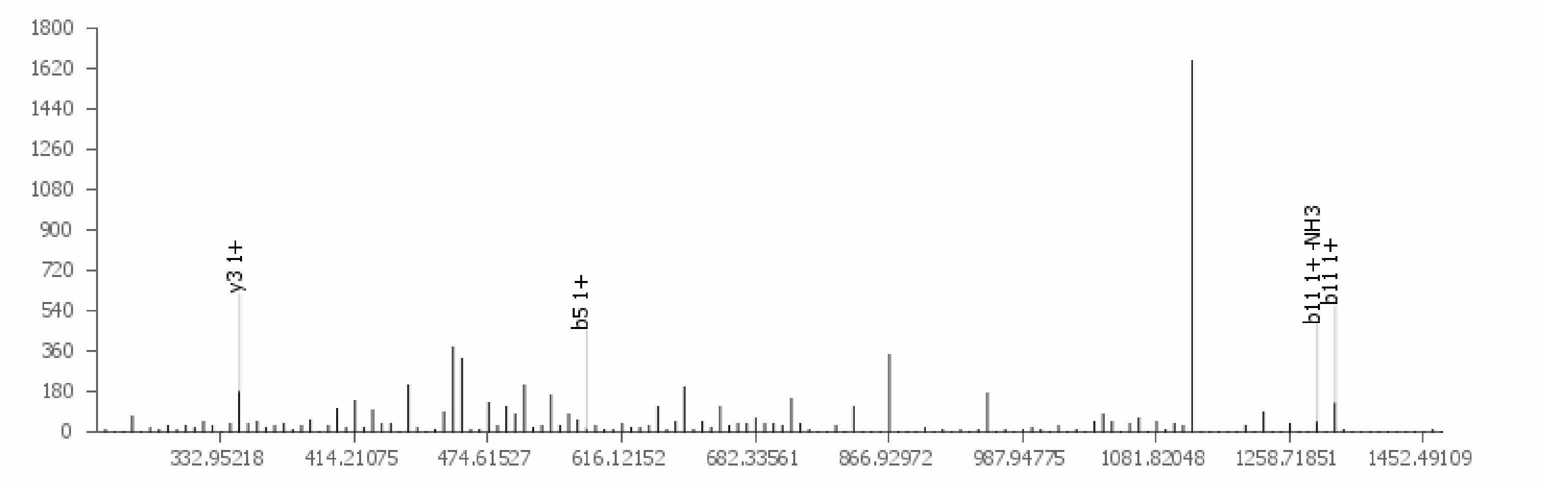

AT4G01400.1 - AFELFK(s)(s)R - 582.777943 - Charge:2

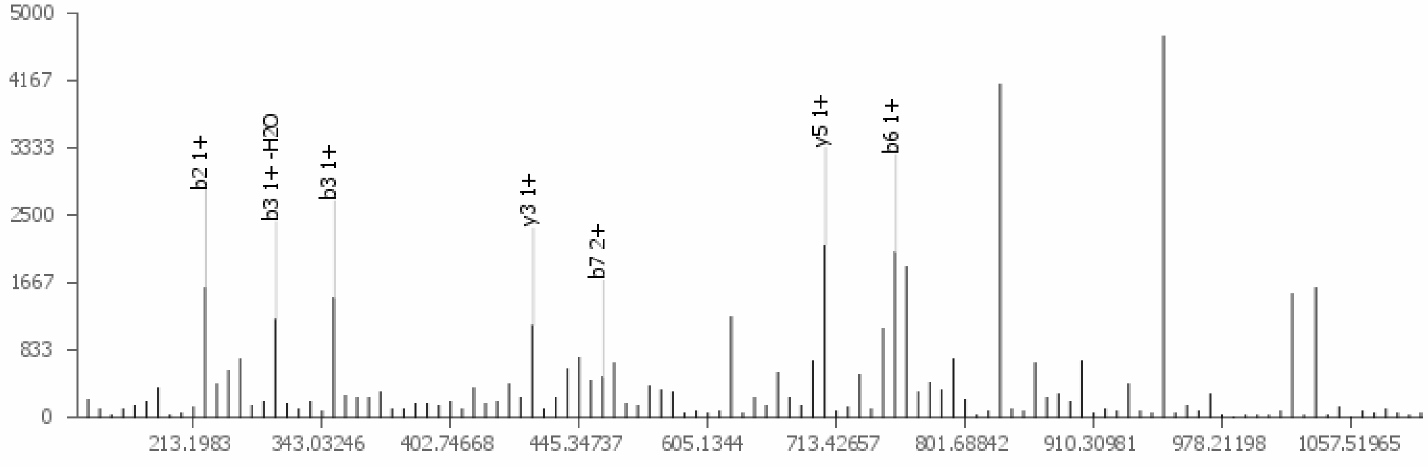

AT5G54870.1 - RLG(pT)EGLDLSDVLVEVEK - 1026.519243 - Charge:2

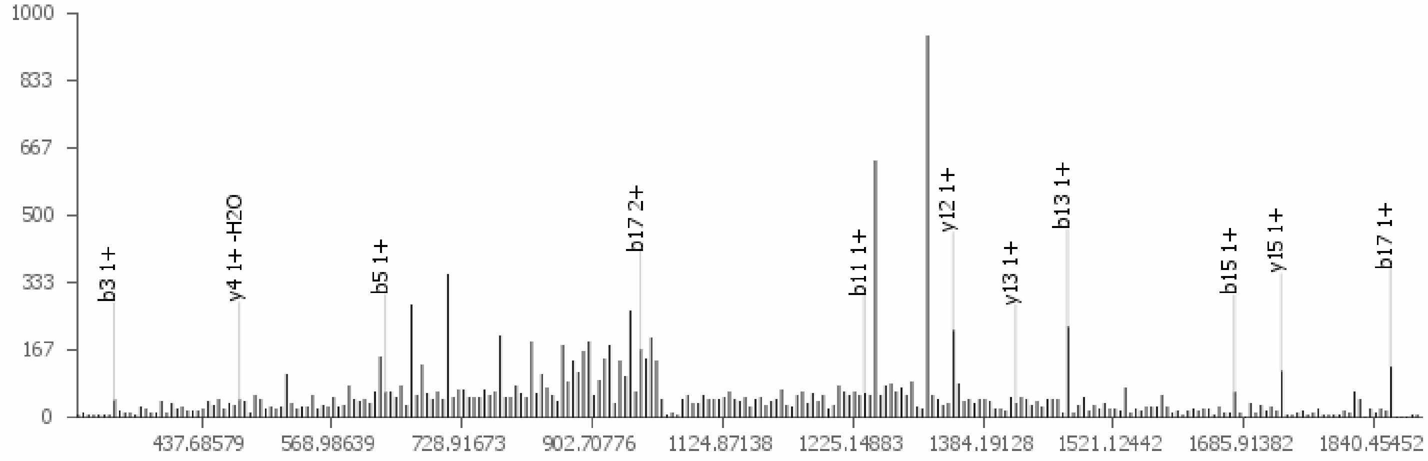

AT5G16510.1 - L(oxM)EKSVPFFD(pS)LK - 818.88511 - Charge:2

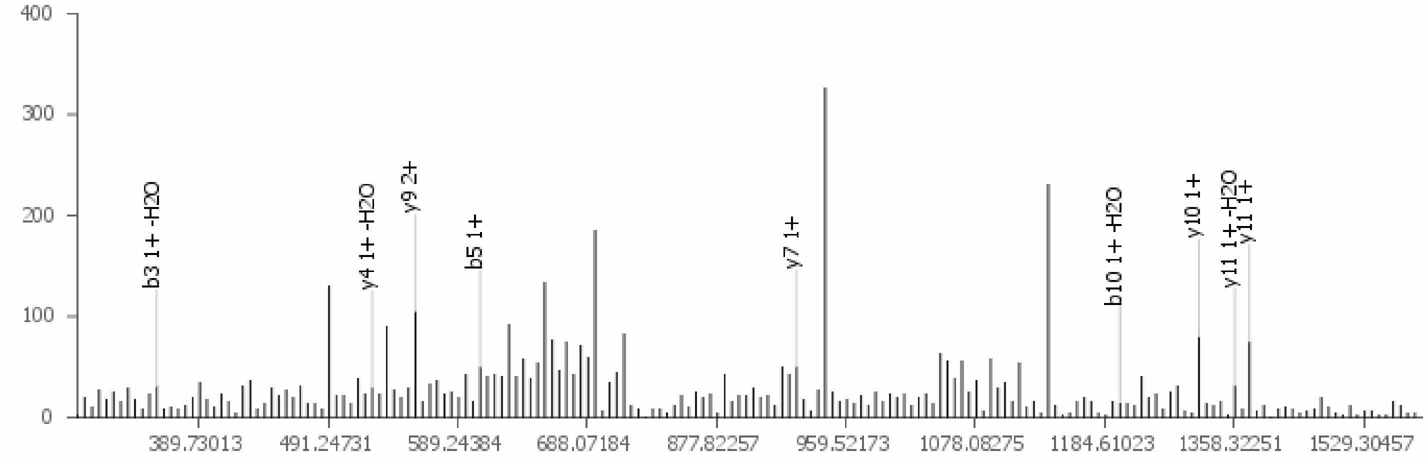

AT4G02400.1 - ML(s)RGL(t)ER - 571.775552 - Charge:2

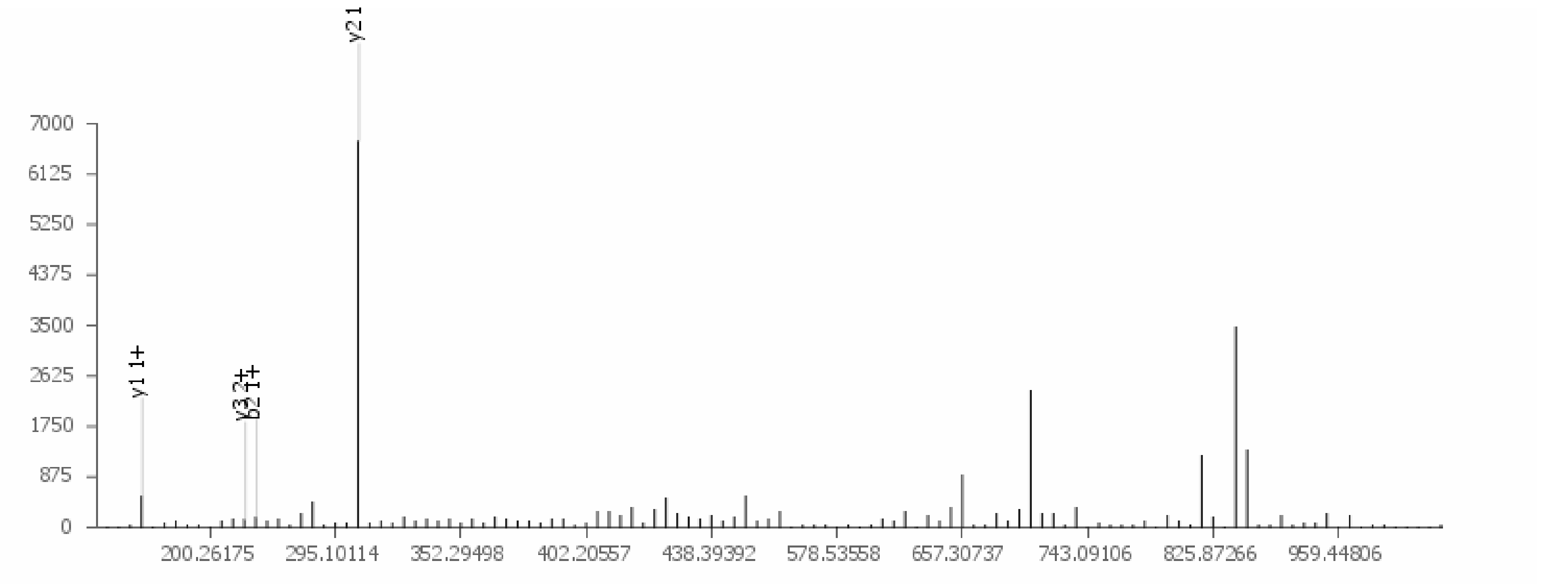

AT3G45130.1 - F(s)(s)KHSSDLLYR - 760.350789 - Charge:2

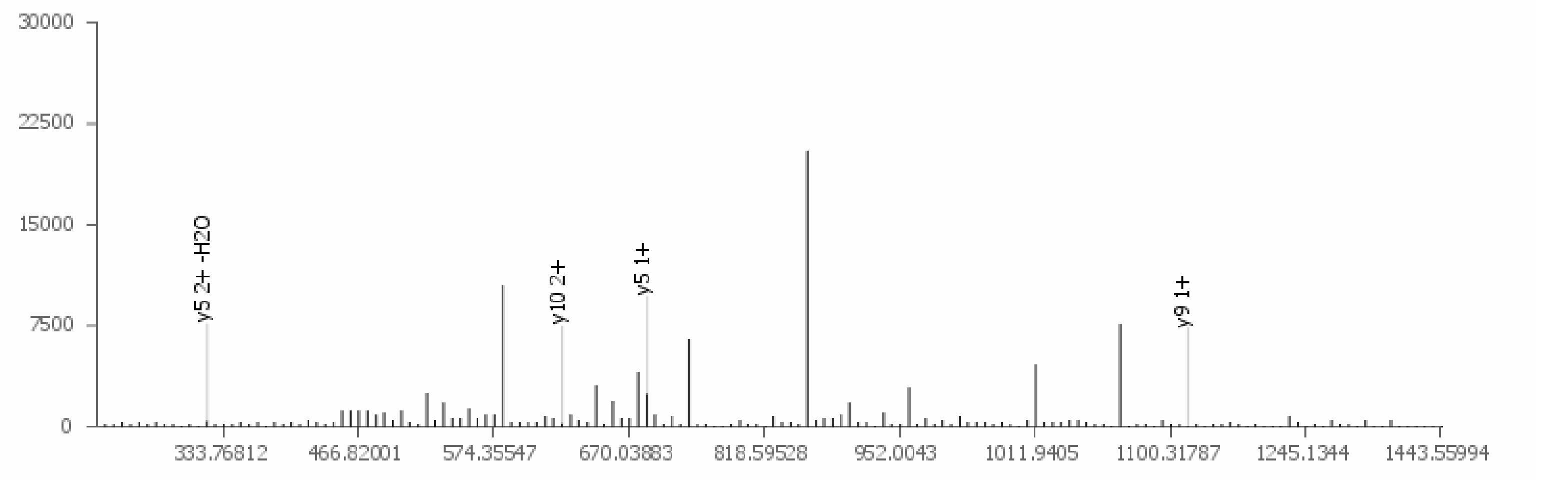

AT5G51760.1 - QSSDNI(pS)VVVIDLK - 798.898493 - Charge:2

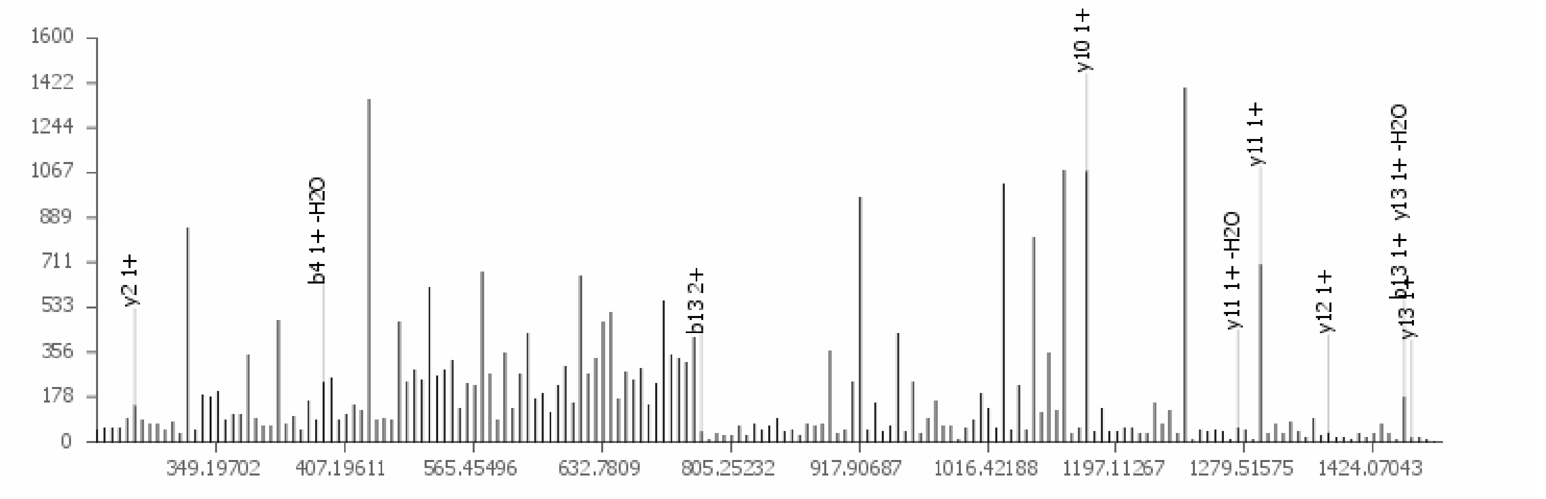

AT4G00320.1 - GVLDIDLQIPS(pS)R - 746.868467 - Charge:2

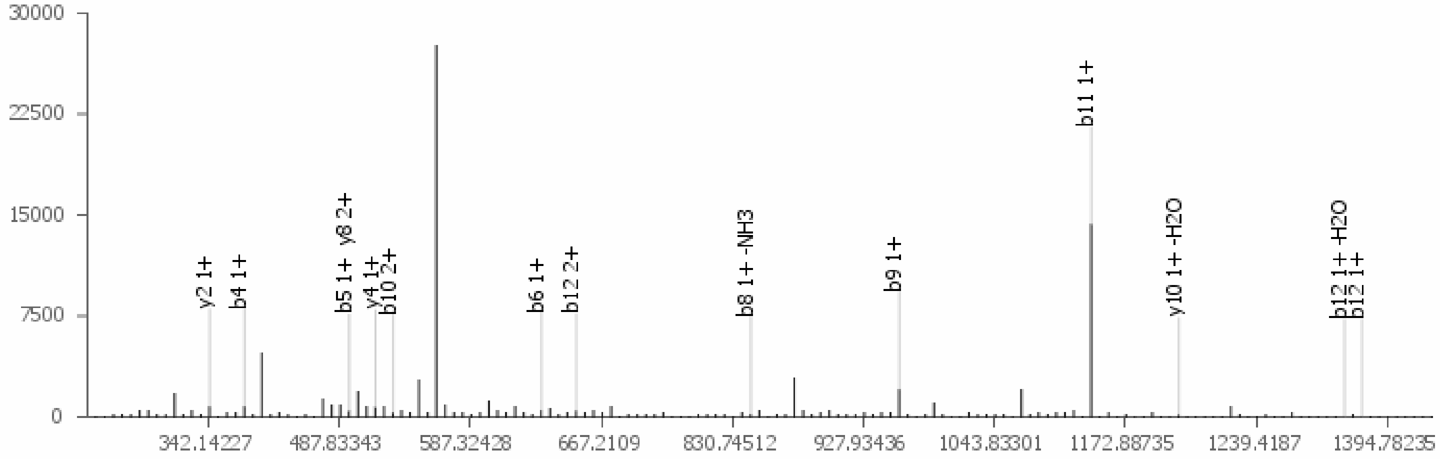

AT3G61490.1 - (pT)AVGRGAFVK - 543.276945 - Charge:2

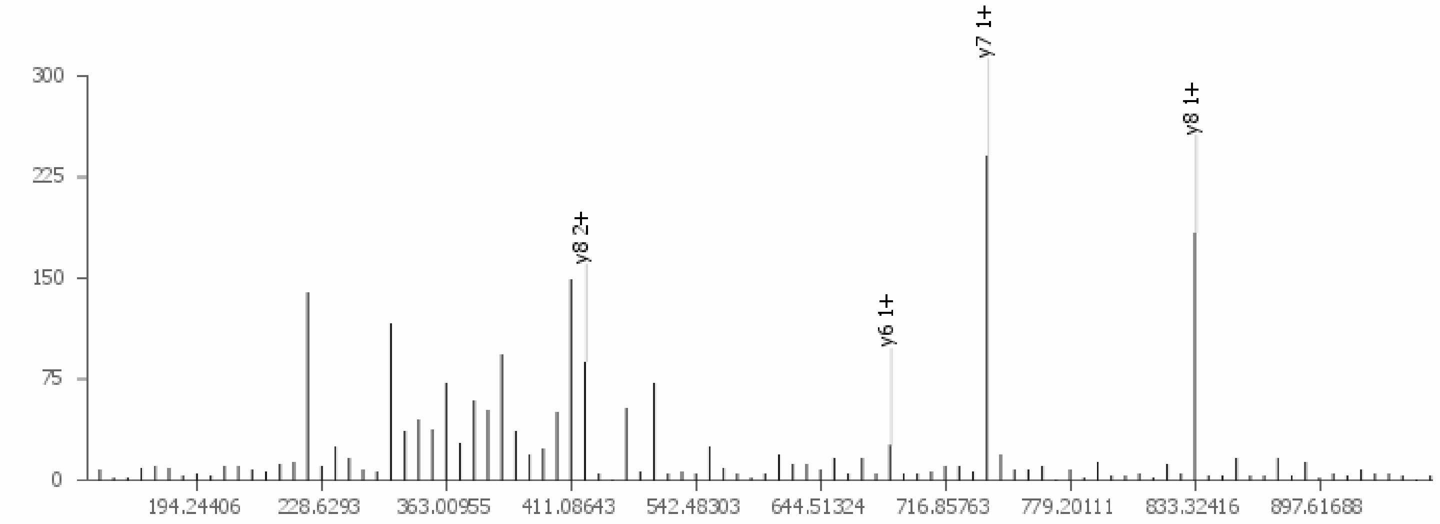

AT1G16760.1 - GQ(pT)LALIHVLPK - 685.388584 - Charge:2

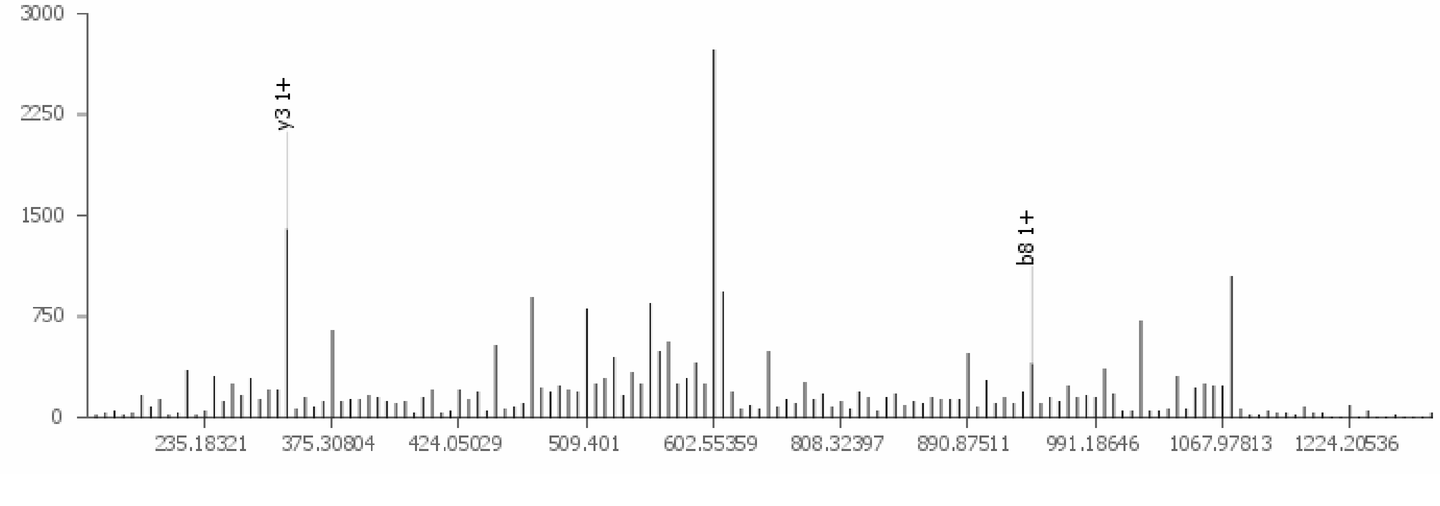

AT1G10060.2 - GNTIVTPATSGTILGGI(pT)RK - 1019.031434 - Charge:2

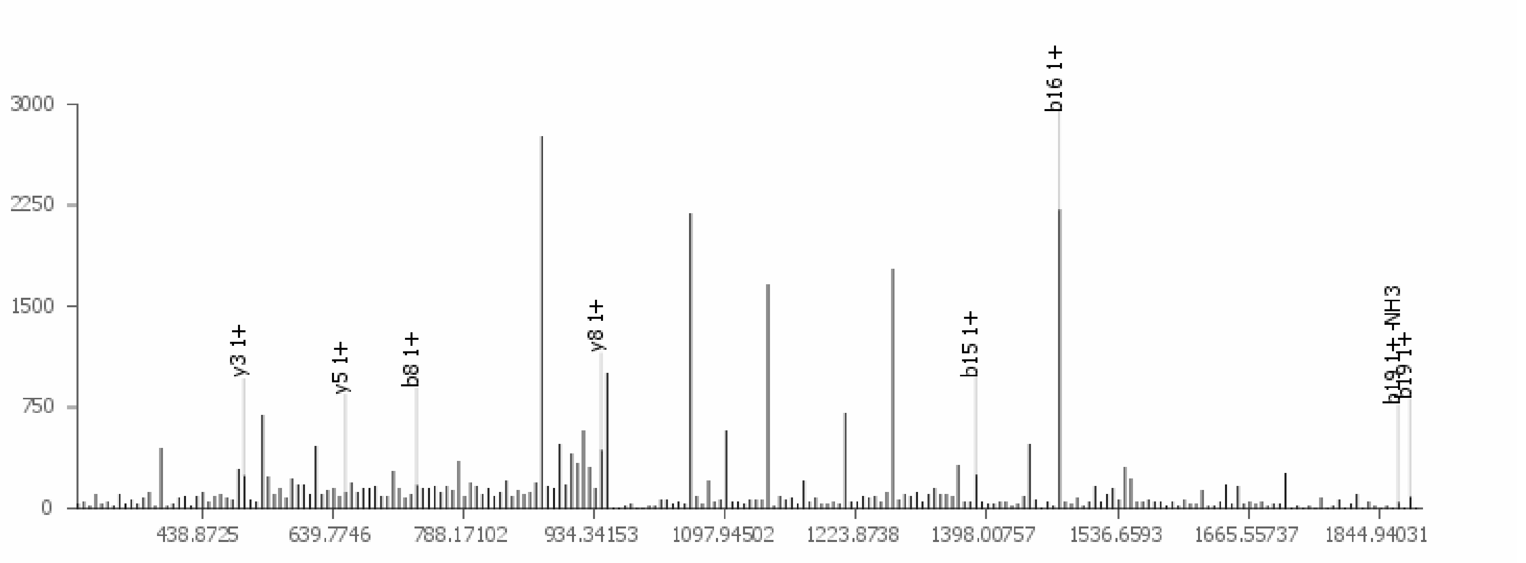

AT4G17740.2 - INPNF(pS)IQISK - 670.840759 - Charge:2

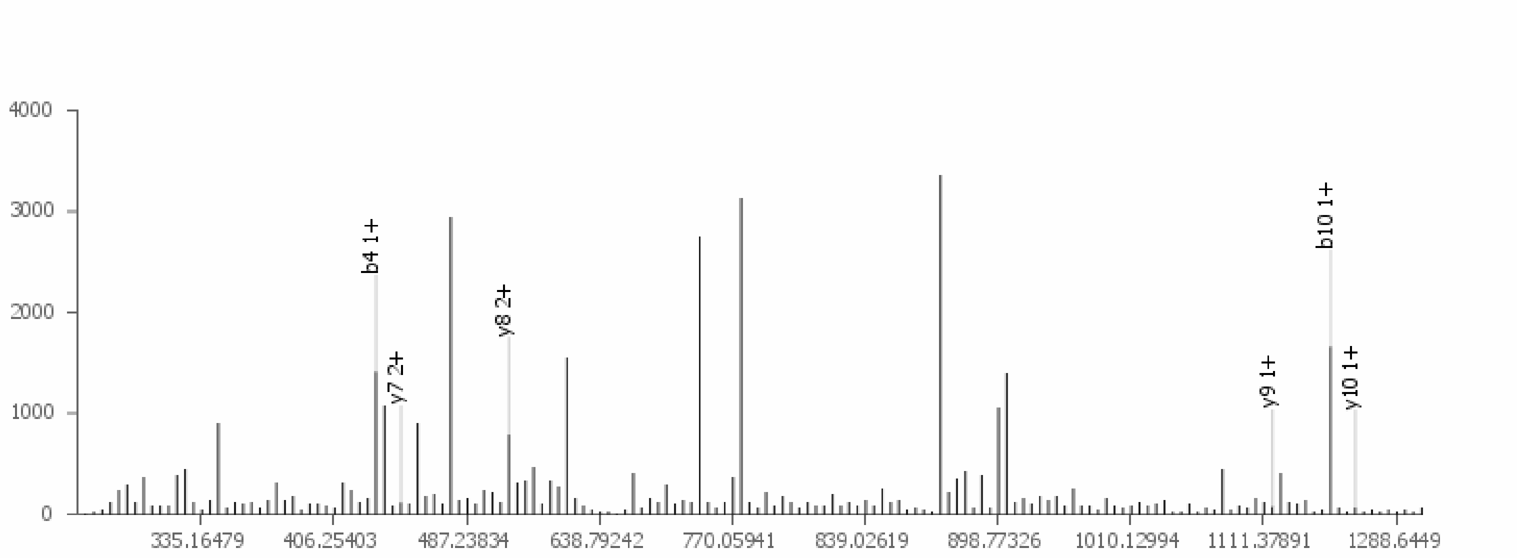

AT5G20250.1 - (oxM)(pT)IKPAVRISDGNLIIK - 983.039589 - Charge:2

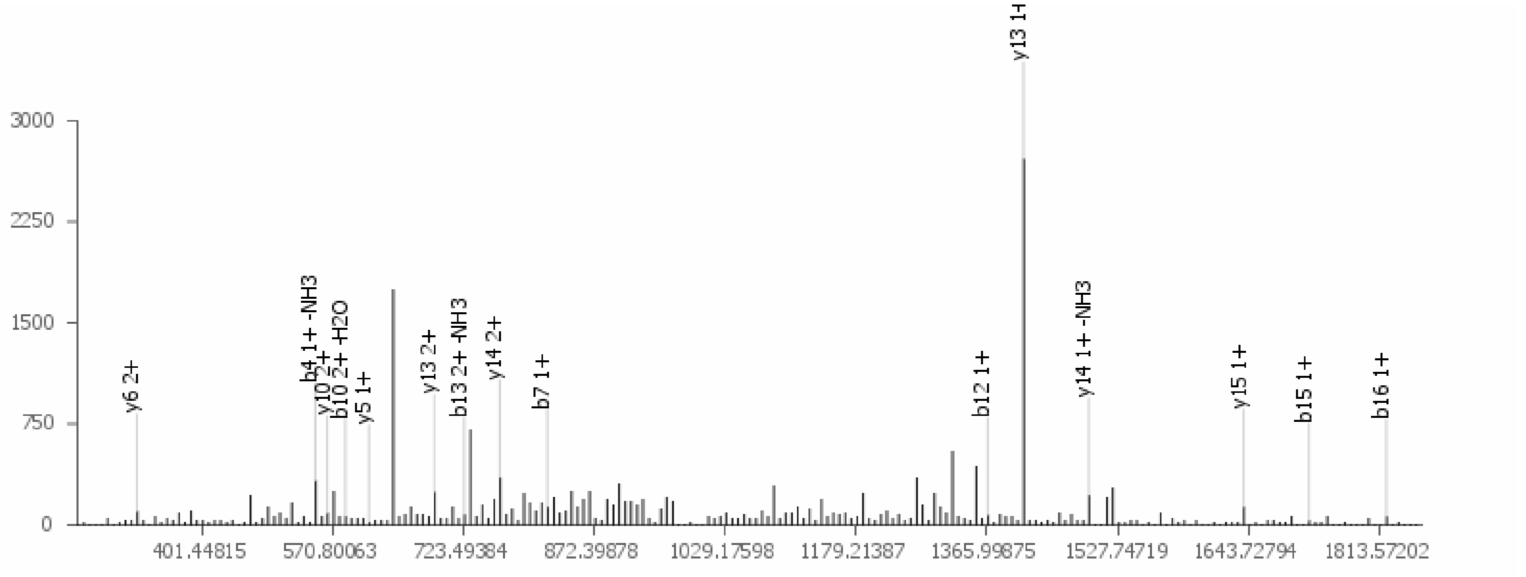

AT1G45130.1 - YVDGI(pS)FRTDNGPFK - 898.409804 - Charge:2

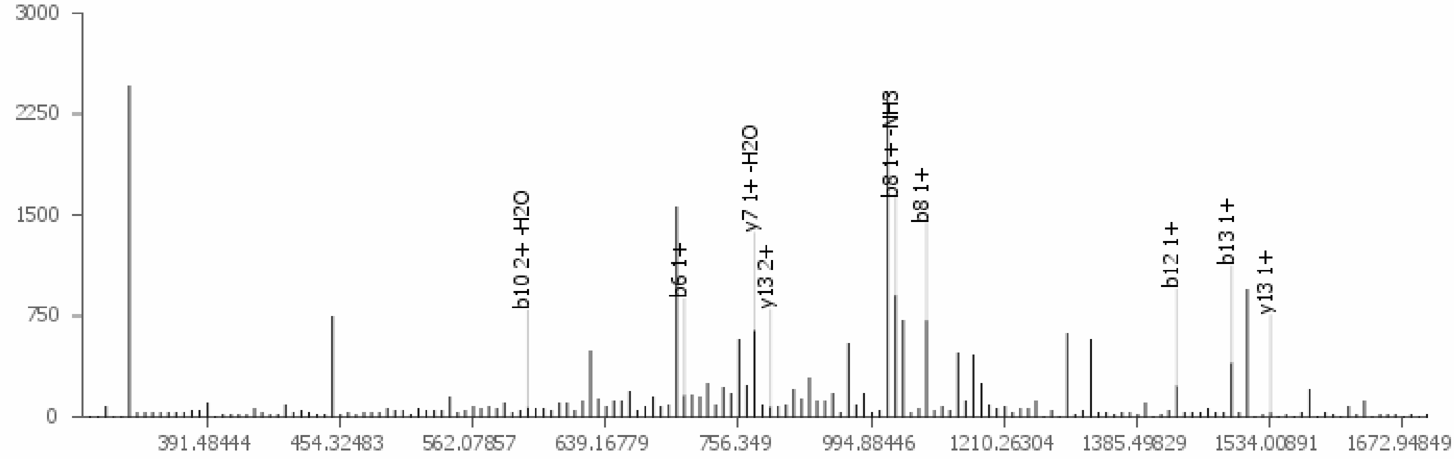

AT1G78900.1 - GV(pS)VPALDKDCLWEFQPNK - 1113.518341 - Charge:2

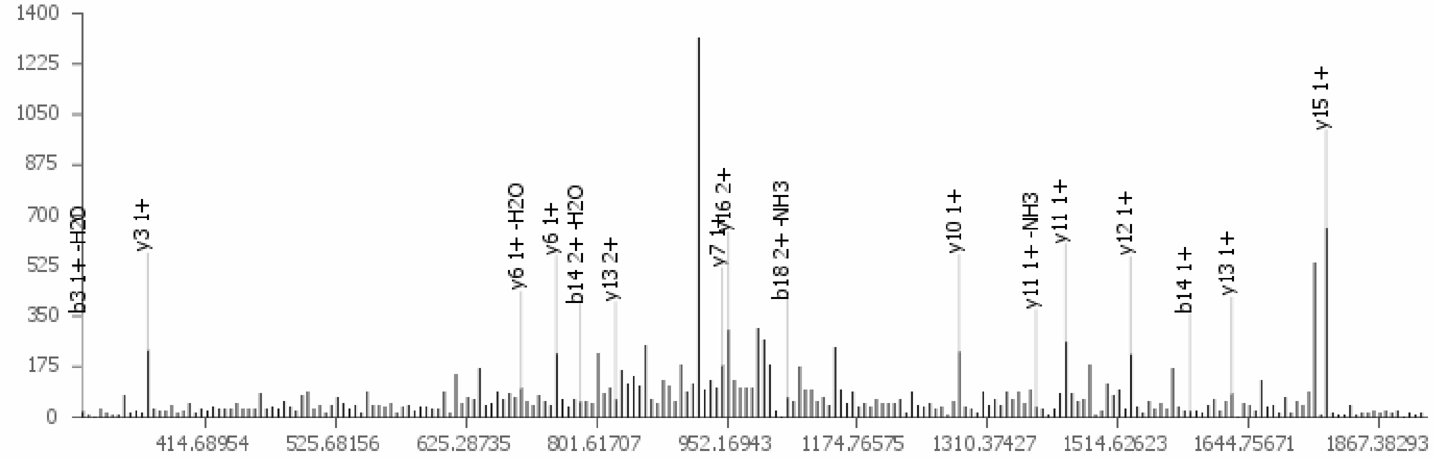

AT1G09180.1 - YYLGL(pT)NFTTGK - 769.327794 - Charge:2

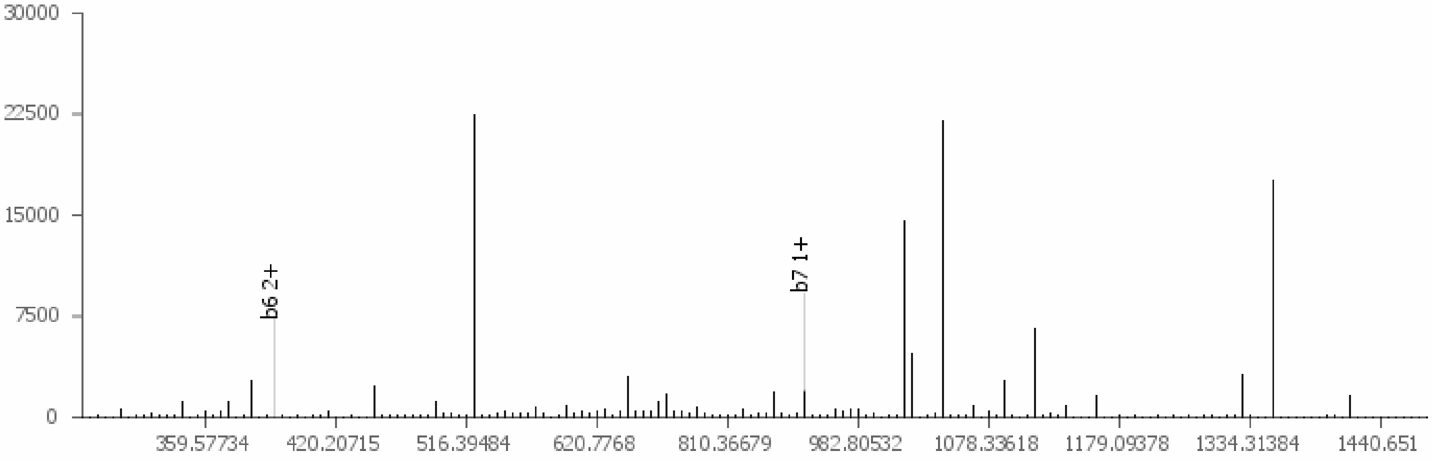

AT2G34040.1 - GAPSSRFLI(pY)LNK - 773.398735 - Charge:2

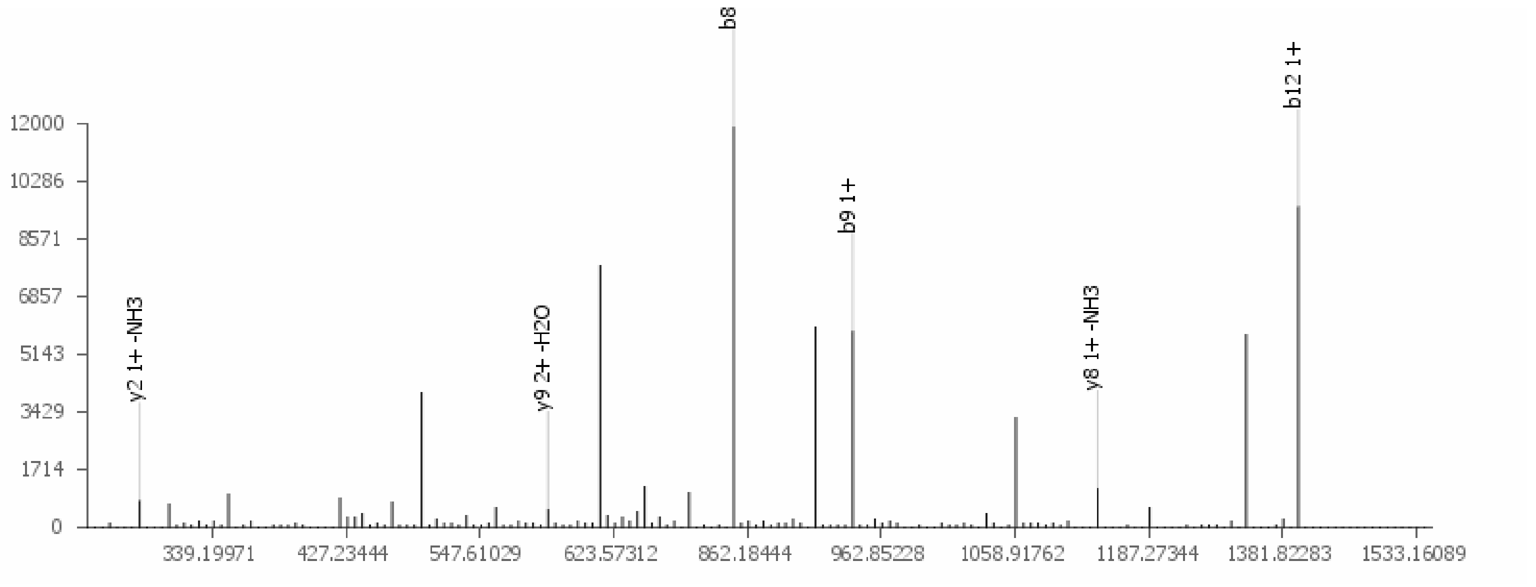

AT1G15690.1 - QFNTIPGLMEGTAKPDYA(pT)CVK - 1232.568371 - Charge:2

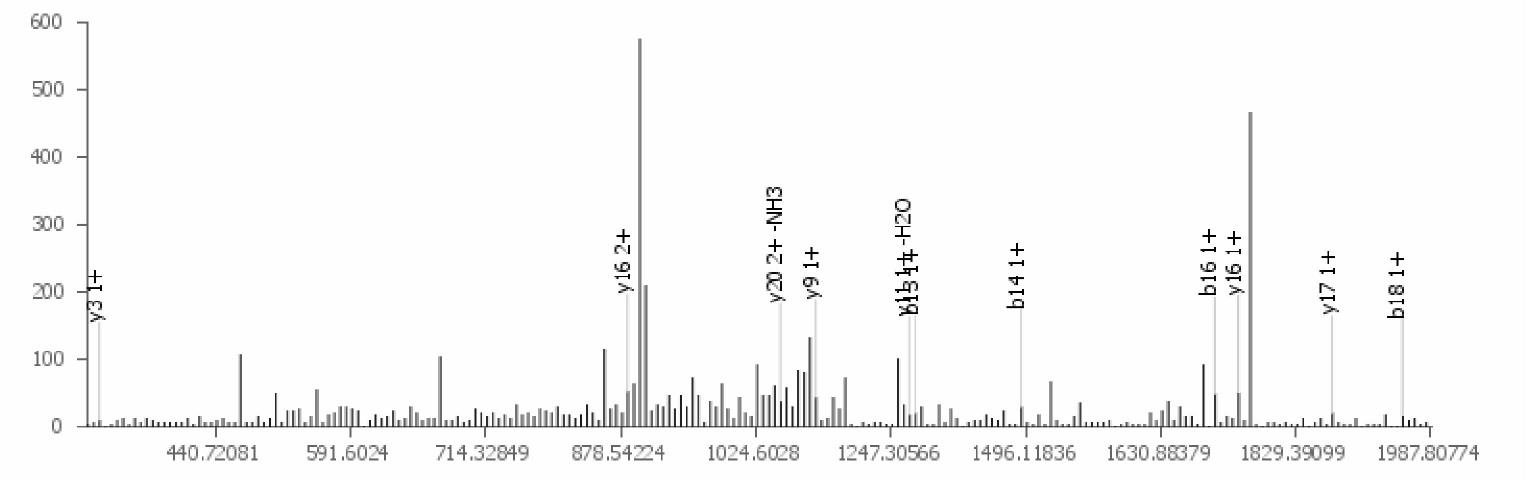

AT5G47690.1 - ILFGFRV(oxM)(pS)R - 661.324469 - Charge:2

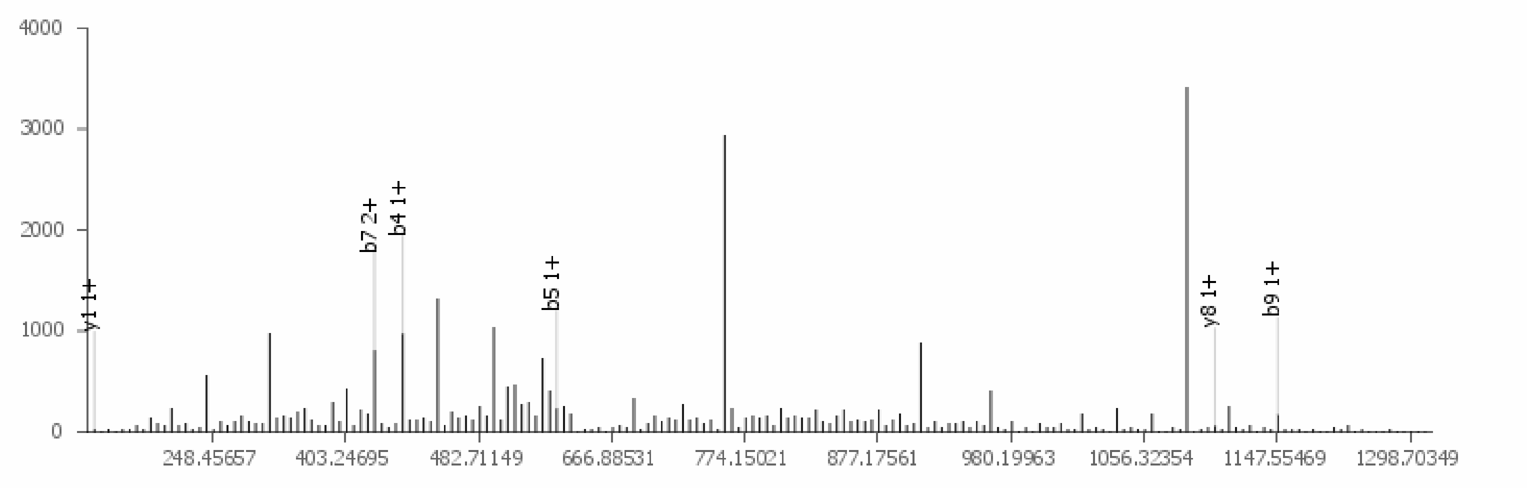

AT1G79190.1 - A(pT)DAITSRQER - 664.302779 - Charge:2

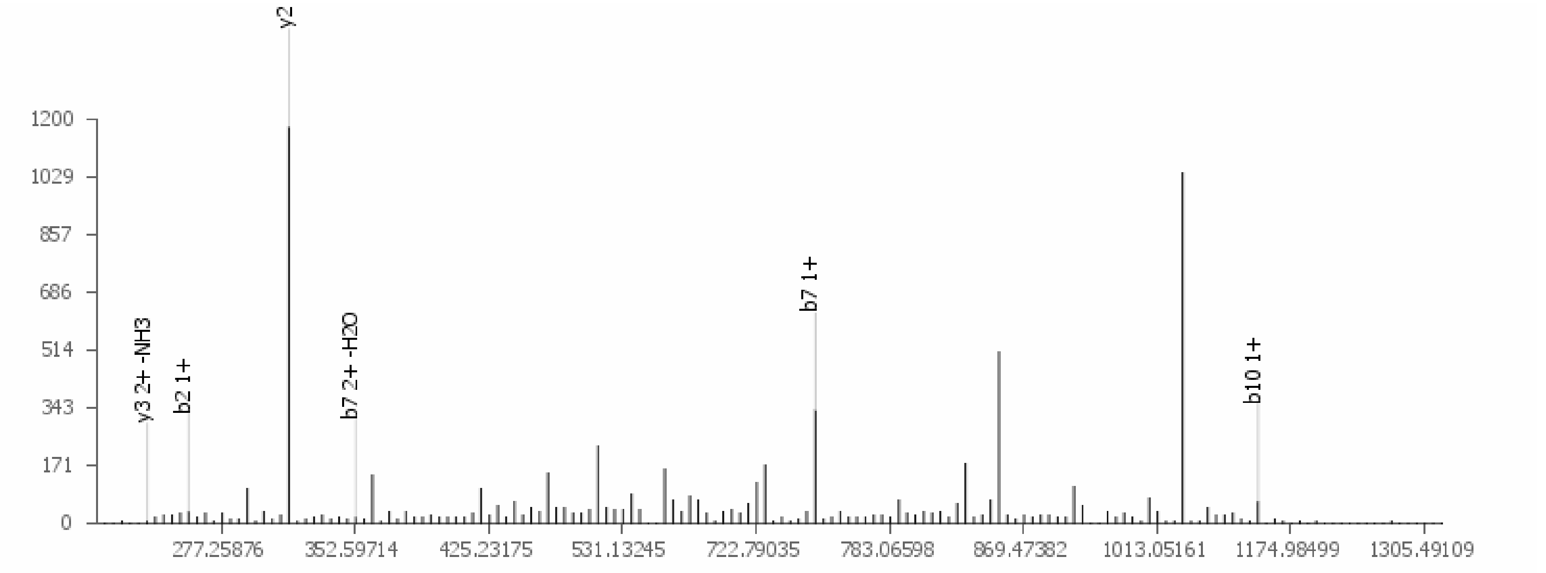

AT5G63330.1 - KIPM(pS)KPPVIPLTSSASLESEIPFEVAP(oxM)R - 837.676636 - Charge:4

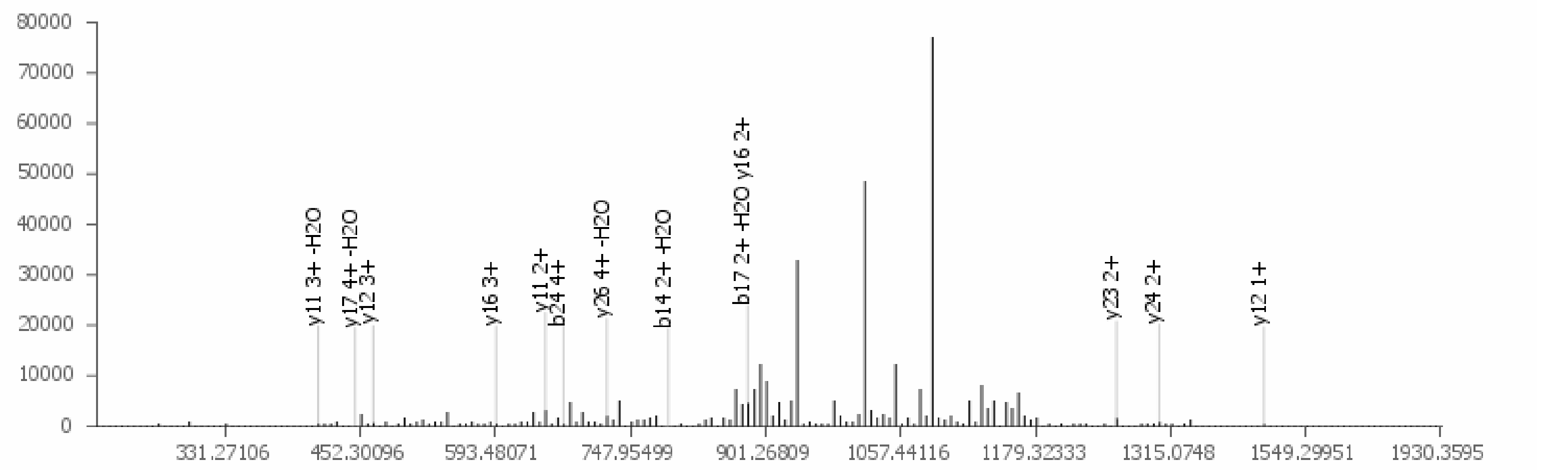

AT3G23750.1 - GGFGVV(pY)AGELHDGTKTAVK - 1043.503341 - Charge:2

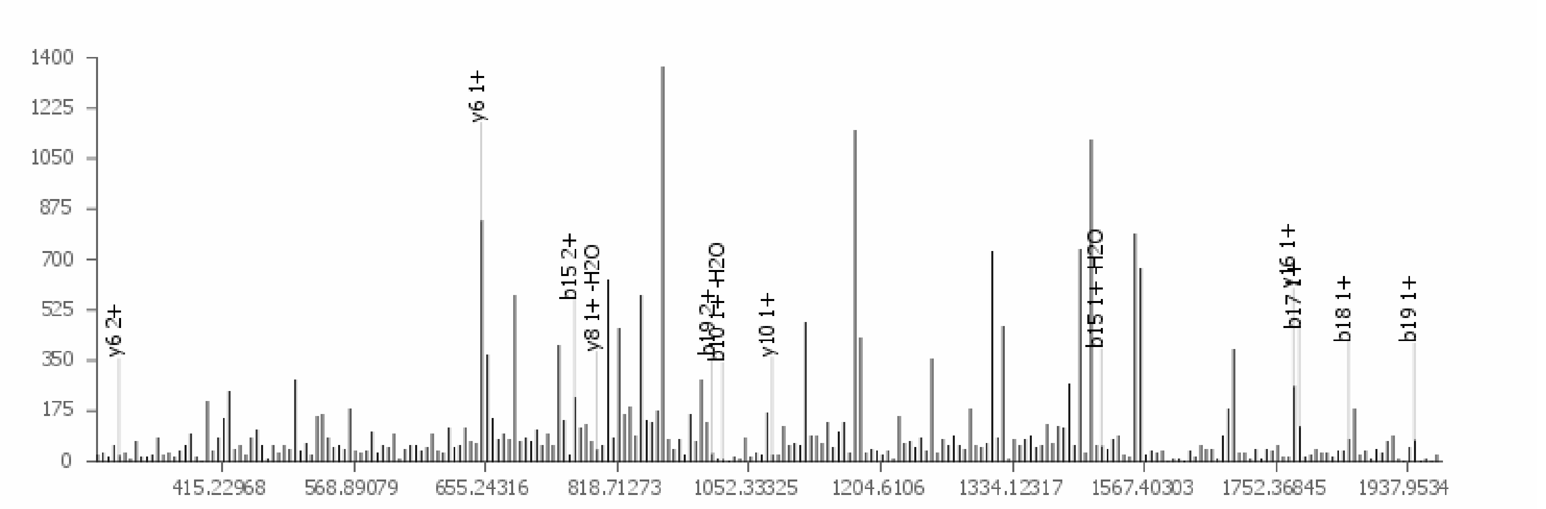

AT1G01830.1 - MMVSYGAIG(pY)LK - 706.821733 - Charge:2

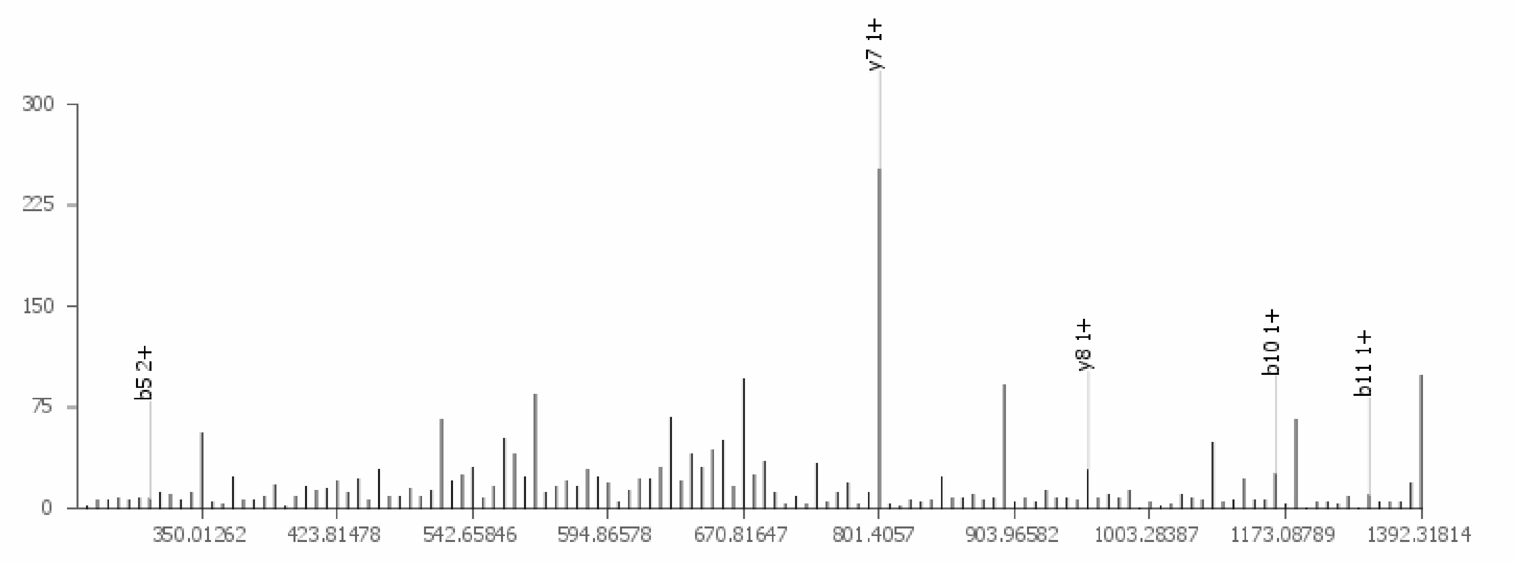

AT1G54640.1 - LNLNE(t)(t)LLK - 619.819022 - Charge:2

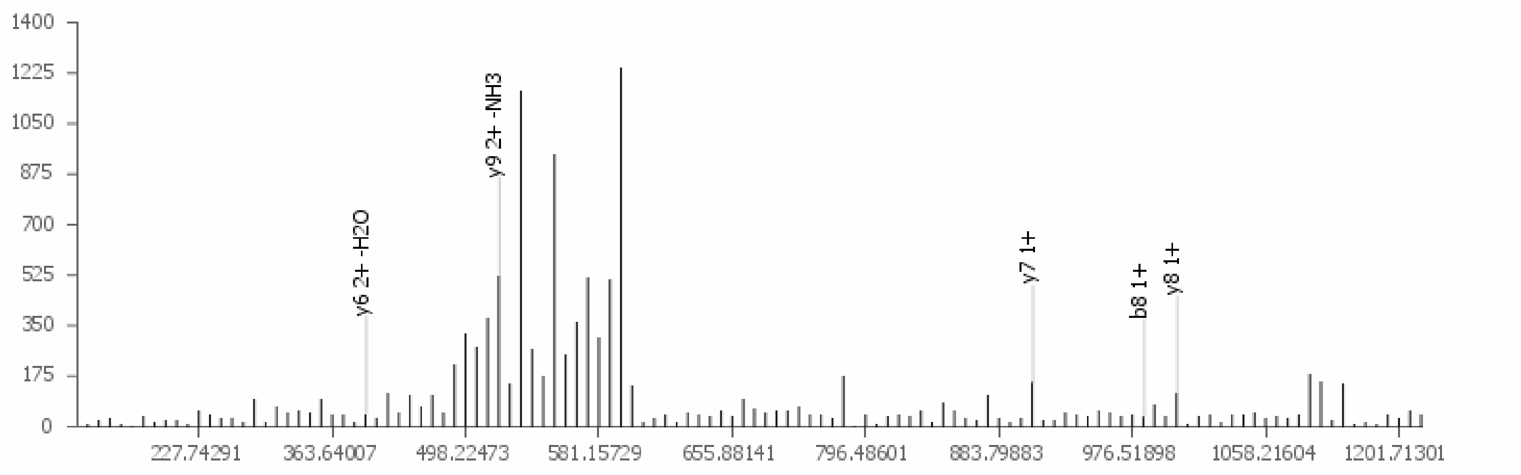

AT5G42030.1 - QTLQF(pS)ETLK - 637.809451 - Charge:2

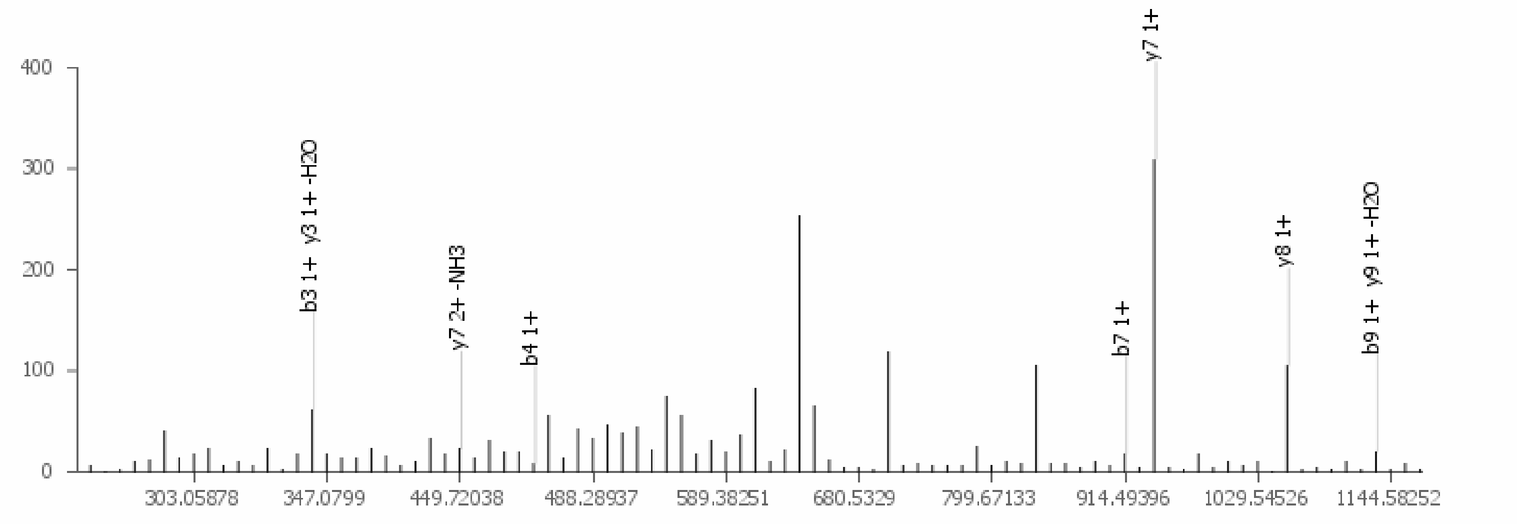

AT5G02900.1 - MSPIKPDVLPSGHKVDS(pS)MK - 1117.033874 - Charge:2

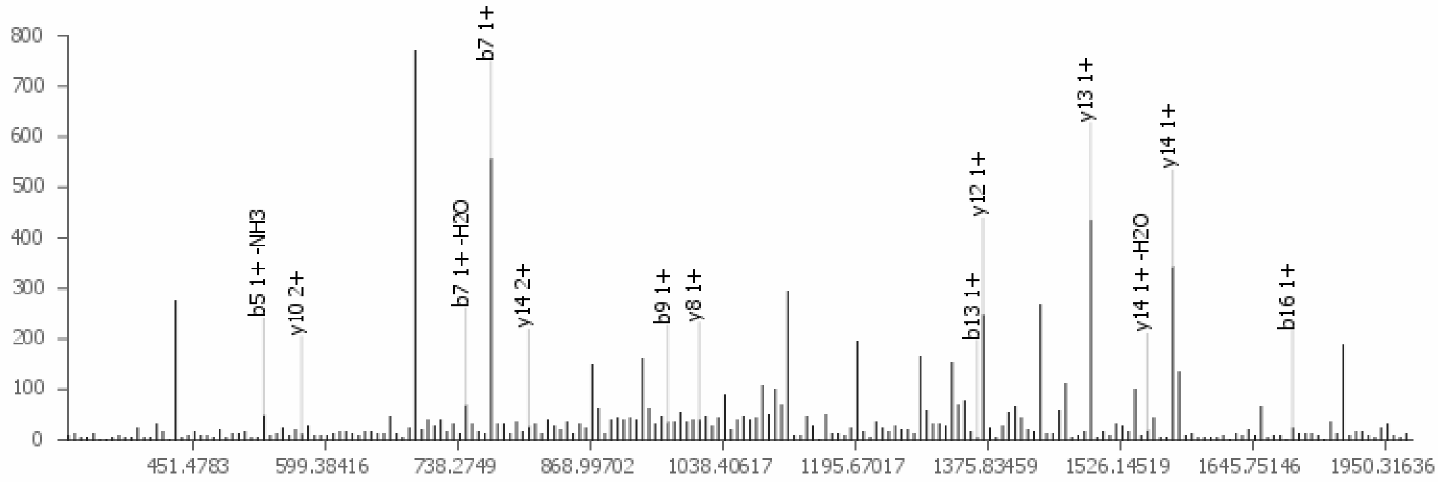

AT4G30190.1 - (s)(s)LEDIKNETVDLEK - 900.420825 - Charge:2

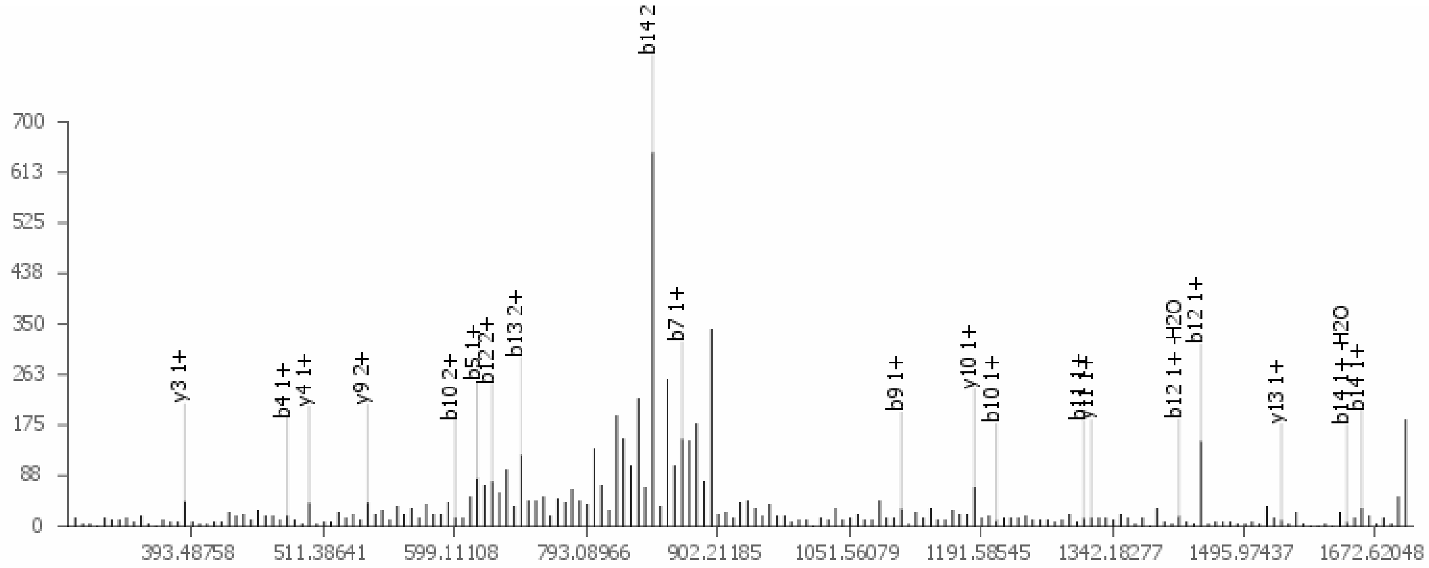

AT5G40405.1 - (oxM)(pY)KNLELGVLASK - 781.39565 - Charge:2

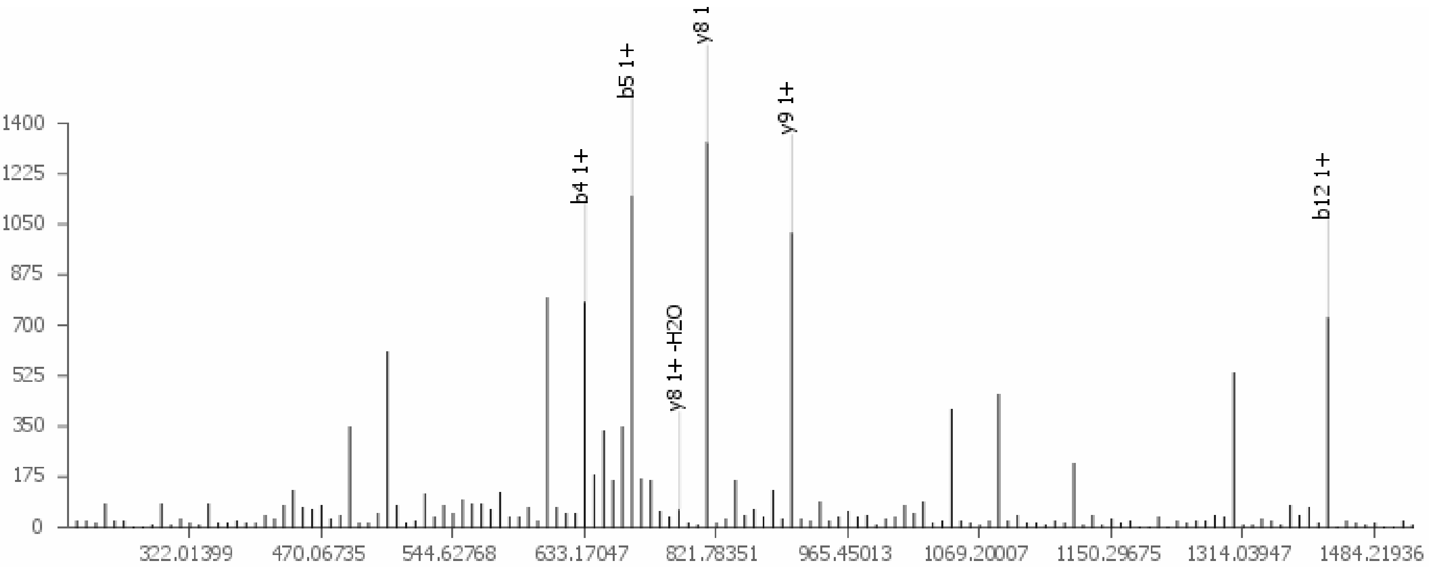

AT1G56380.1 - ITQSFE(pT)LKK - 637.828485 - Charge:2

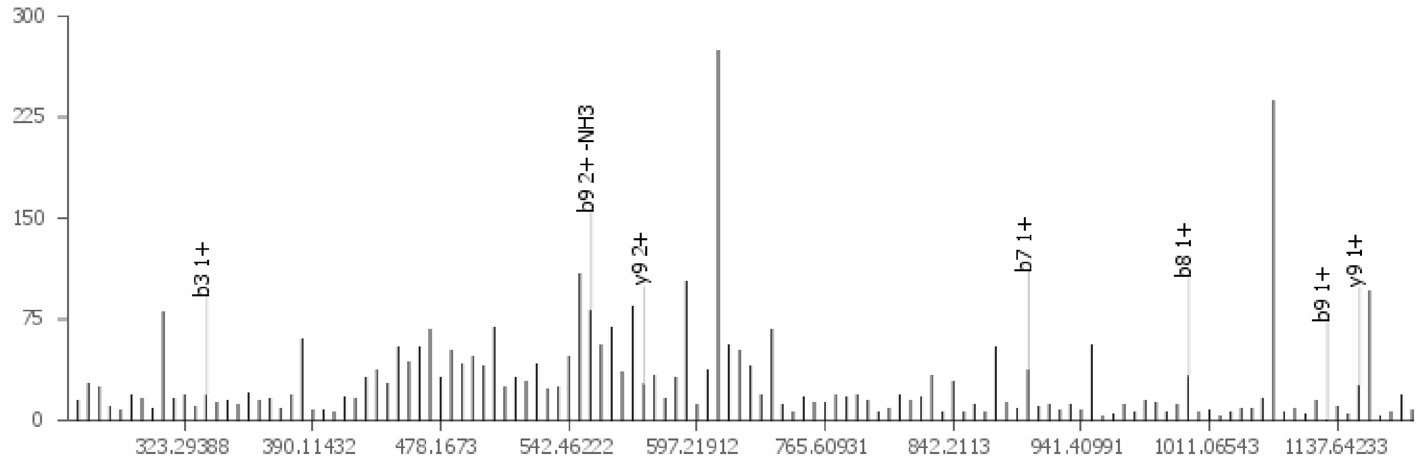

AT1G80870.1 - AKI(pS)DFGLSR - 587.292288 - Charge:2

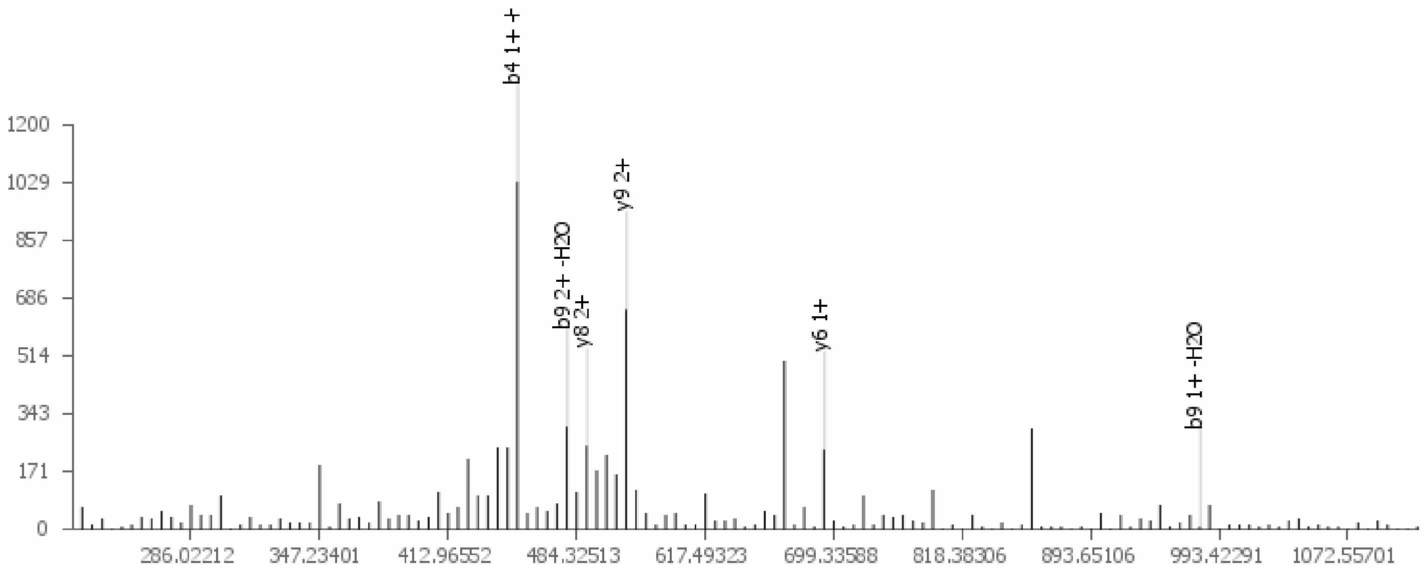

AT5G64940.1 - QVEV(pS)VTPGGR - 604.781566 - Charge:2

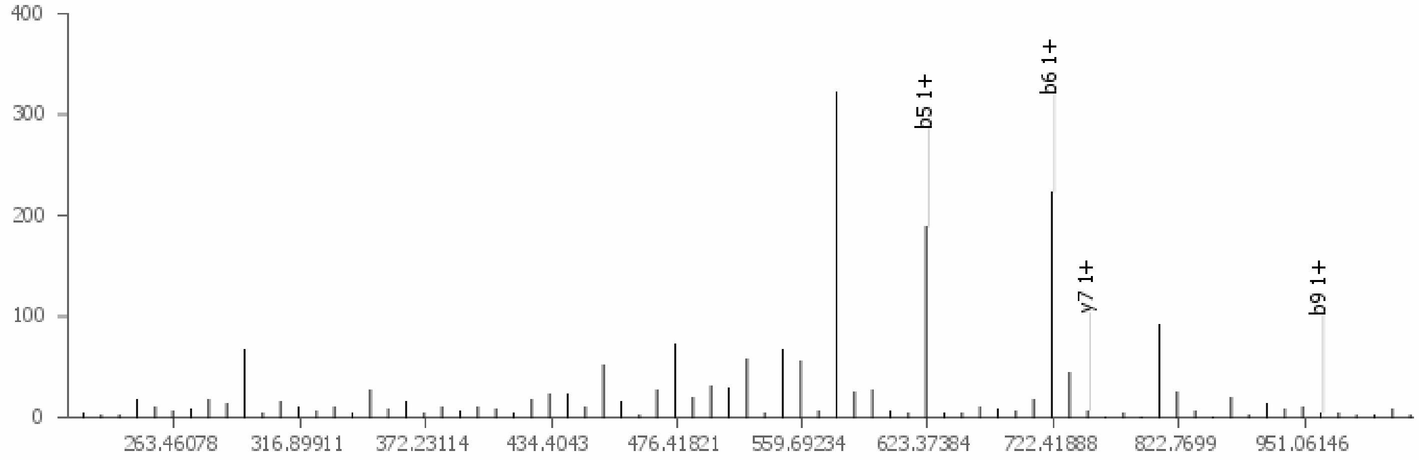

AT2G27630.1 - LF(pT)LEENER - 615.773611 - Charge:2

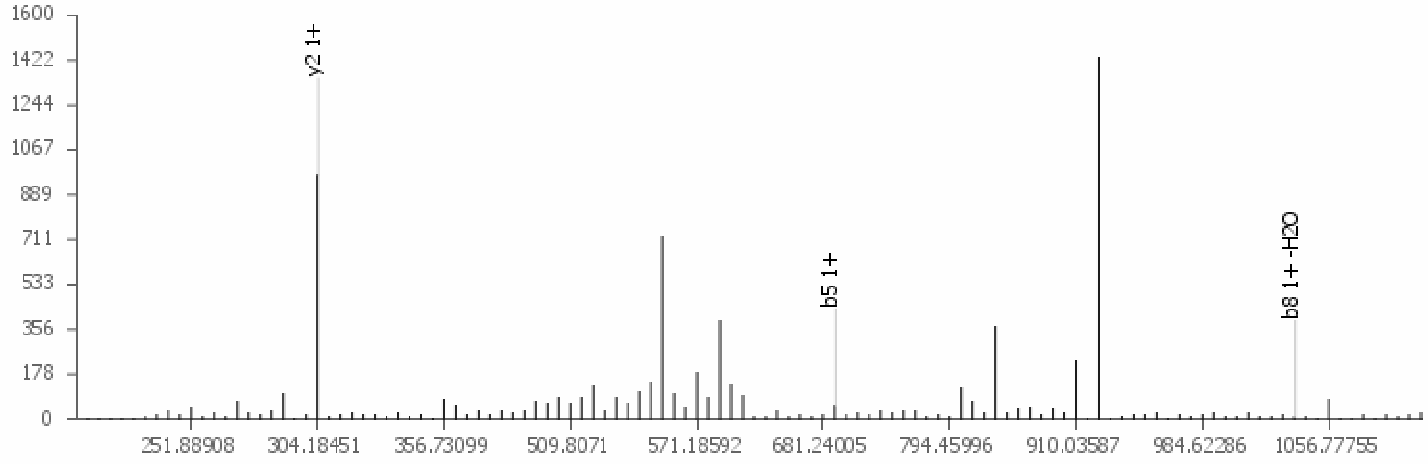

AT2G19190.1 - (pS)ILANGDIR - 519.753064 - Charge:2

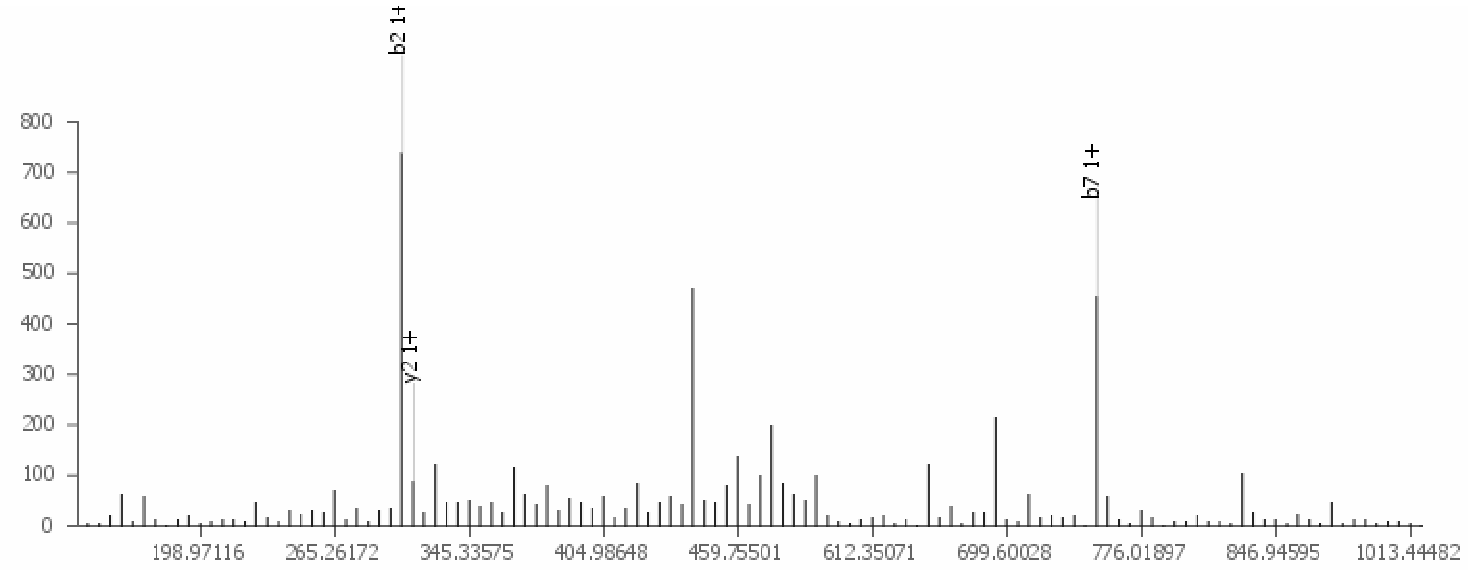

AT5G22700.1 - DDQ(pS)ASFVI(s)NLG(s)SAKADIK - 1157.014049 - Charge:2

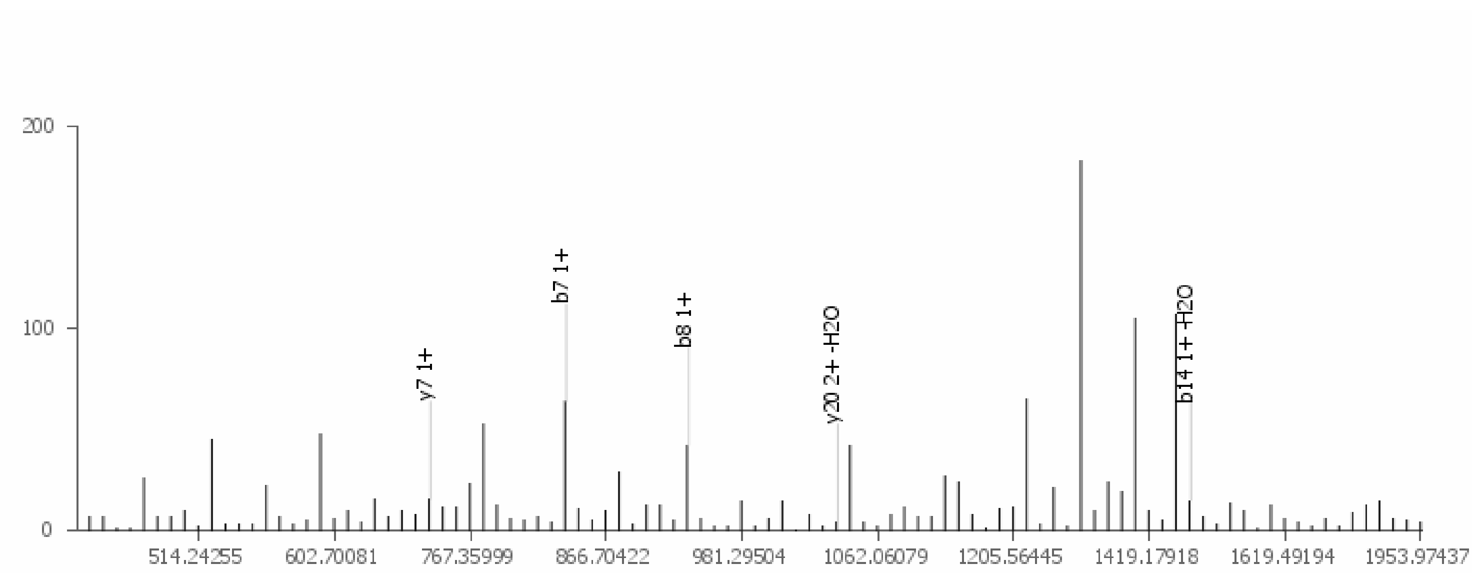

AT3G44200.1 - RTSLIAHQ(pS)R - 624.818786 - Charge:2

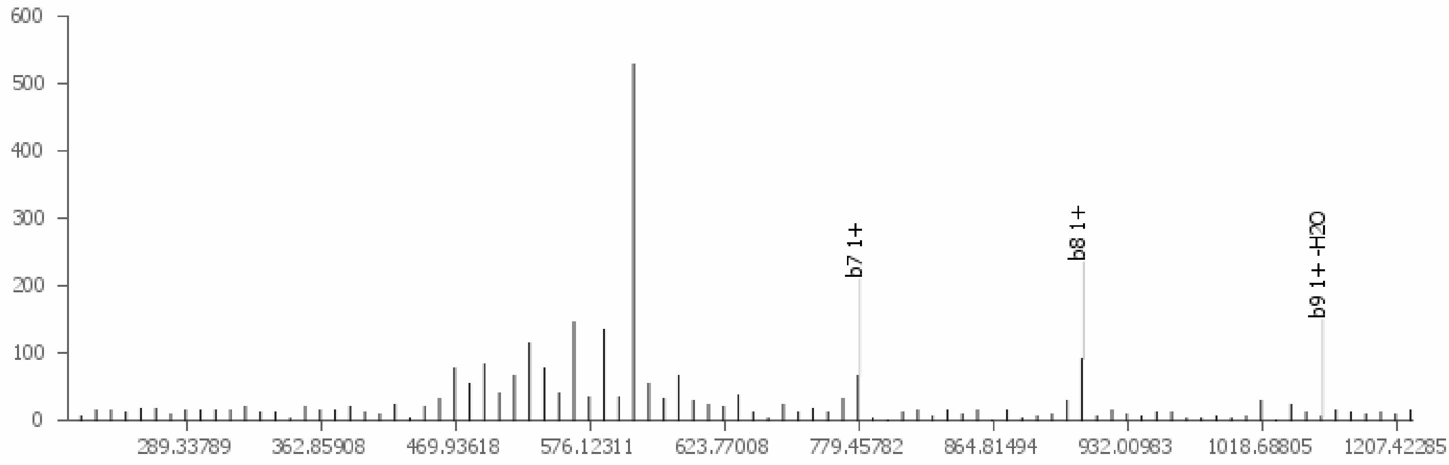

AT1G36730.1 - DKLT(pS)FILK - 572.806503 - Charge:2

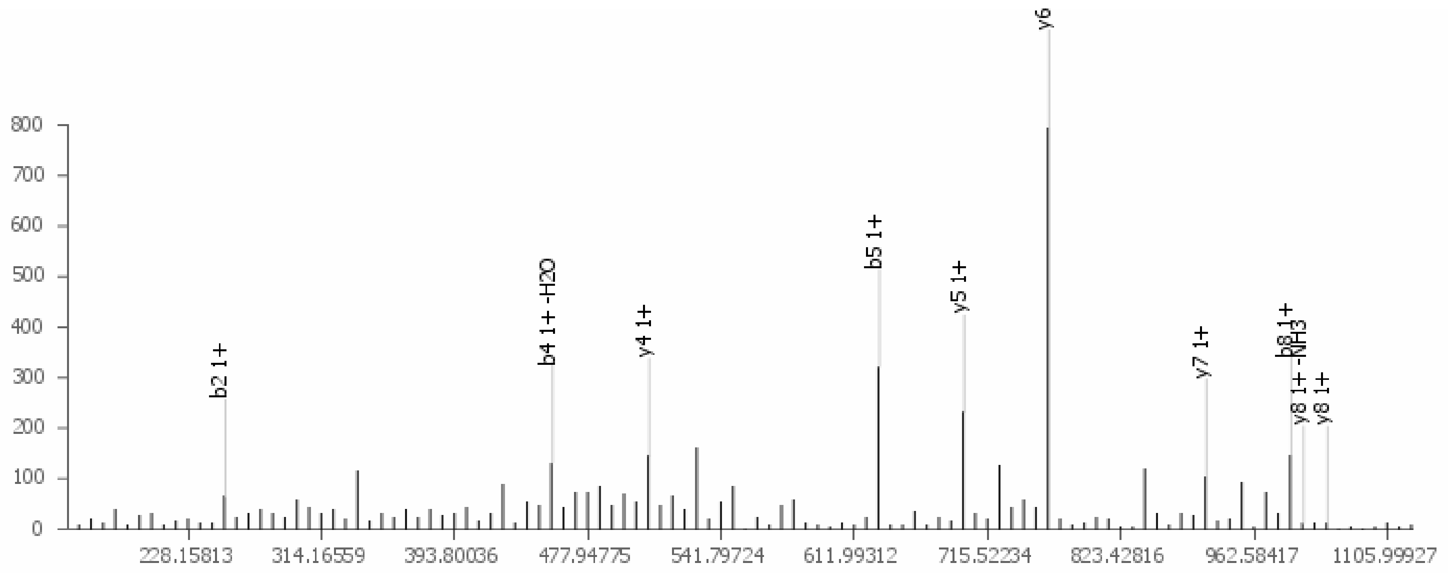

AT1G79990.1 - SVGADEV(pT)DGERLPLAVK - 968.470734 - Charge:2

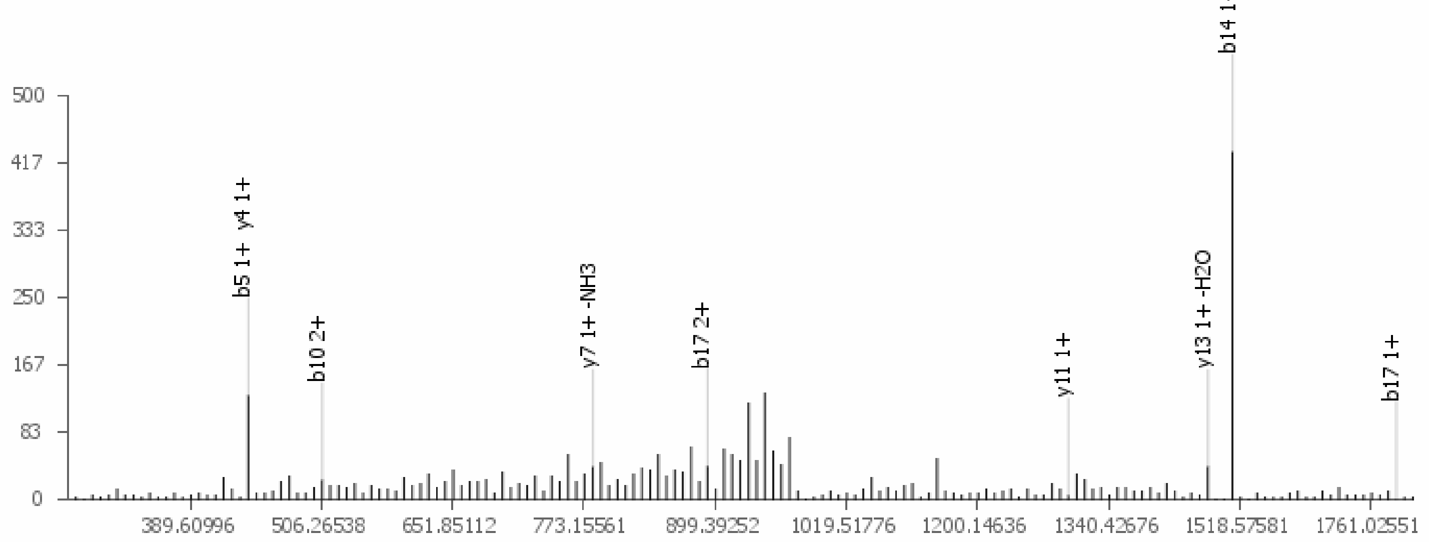

AT4G21430.1 - EIRELTSSD(s)(t)GALR - 857.901955 - Charge:2

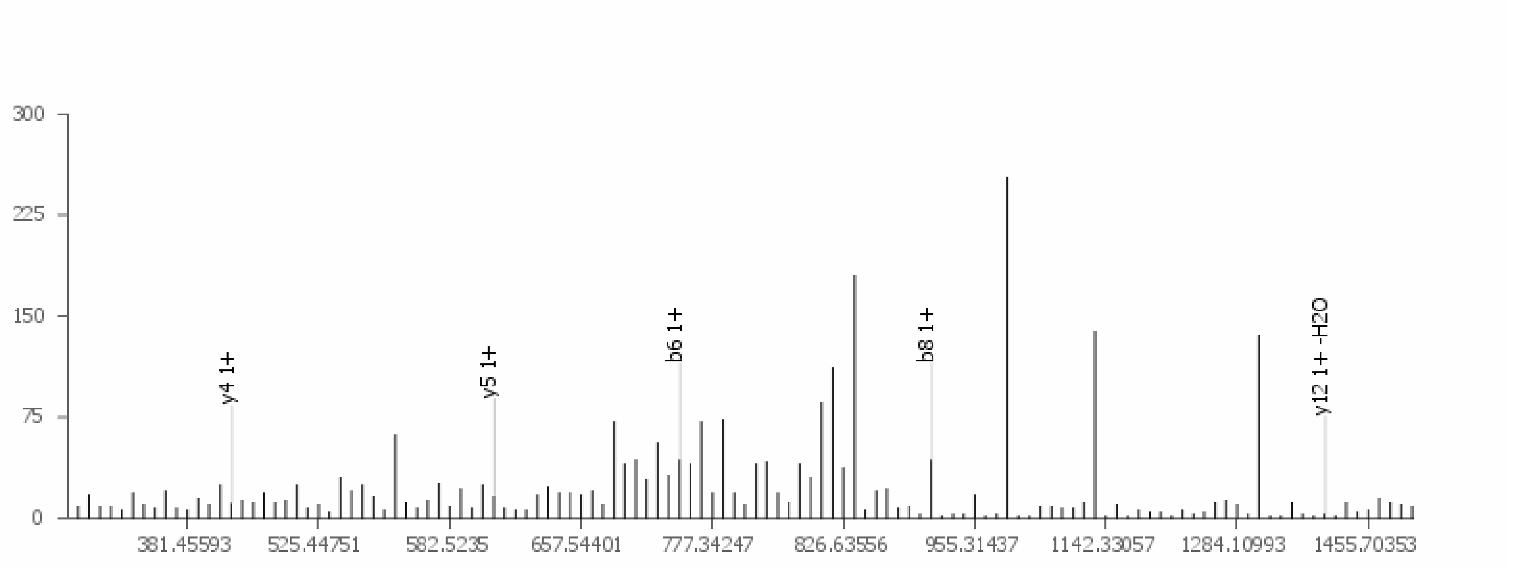

AT5G60660.1 - ALGSFGSFG(pS)FR - 656.794653 - Charge:2

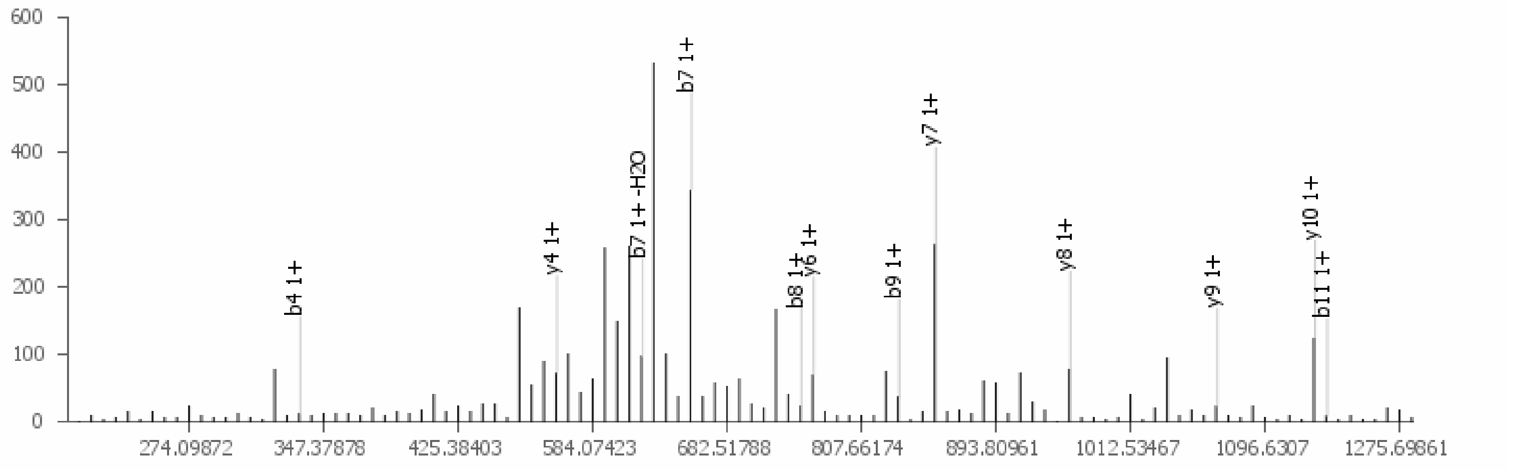

AT2G27410.1 - SI(t)(s)VEPVLHK - 645.329365 - Charge:2

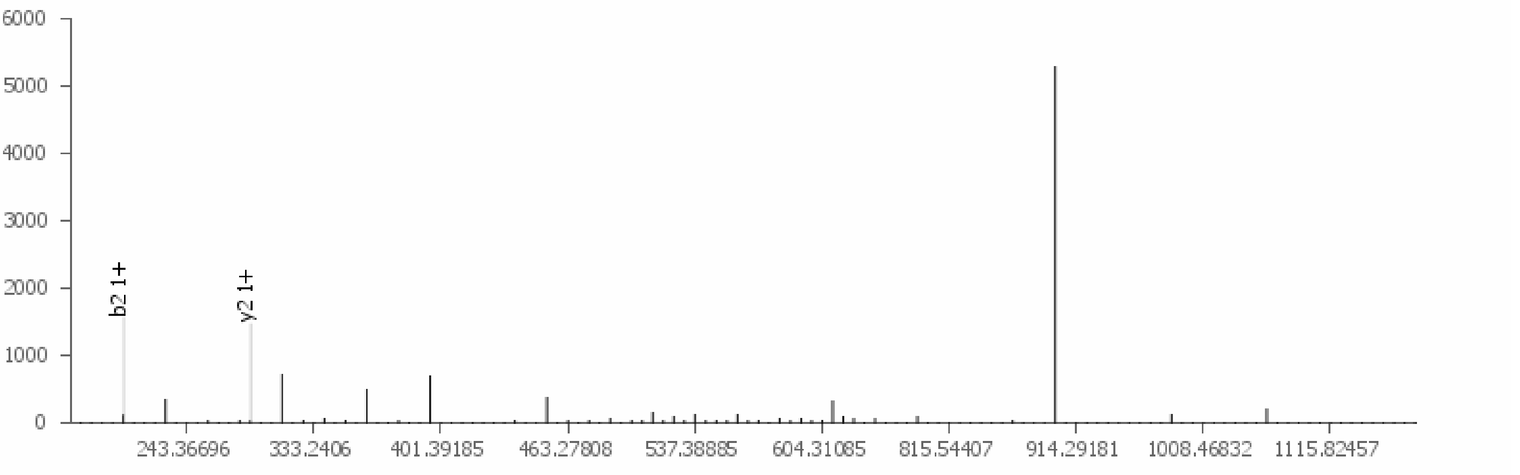

AT3G33073.1 - ERE(pT)LAVYR - 608.785051 - Charge:2

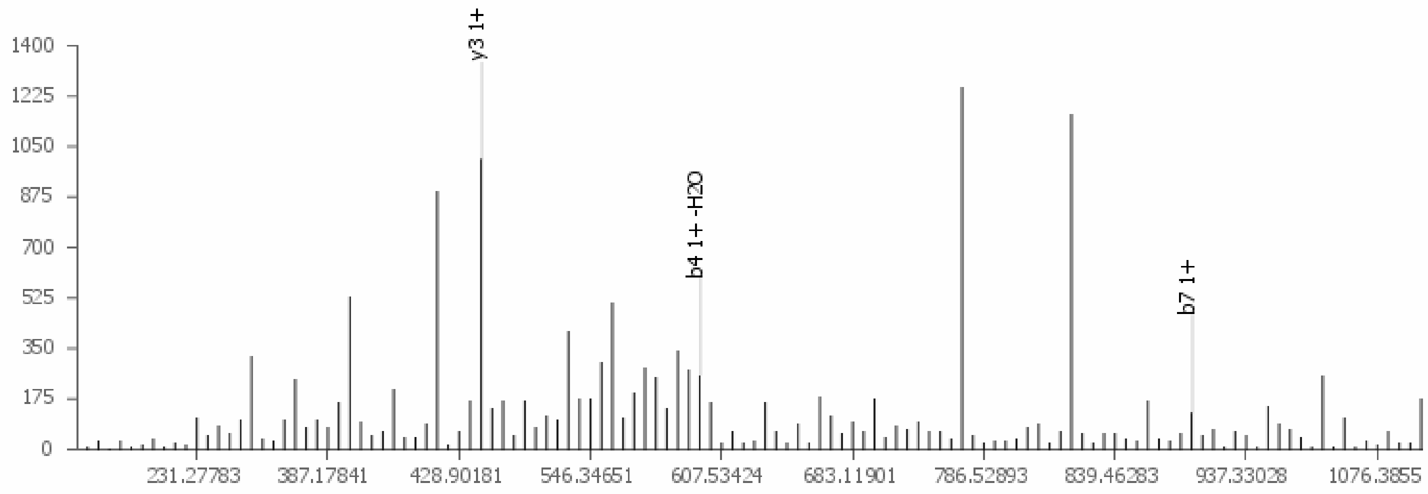

AT1G42970.1 - IVDNE(pT)ISVDGK - 685.320639 - Charge:2

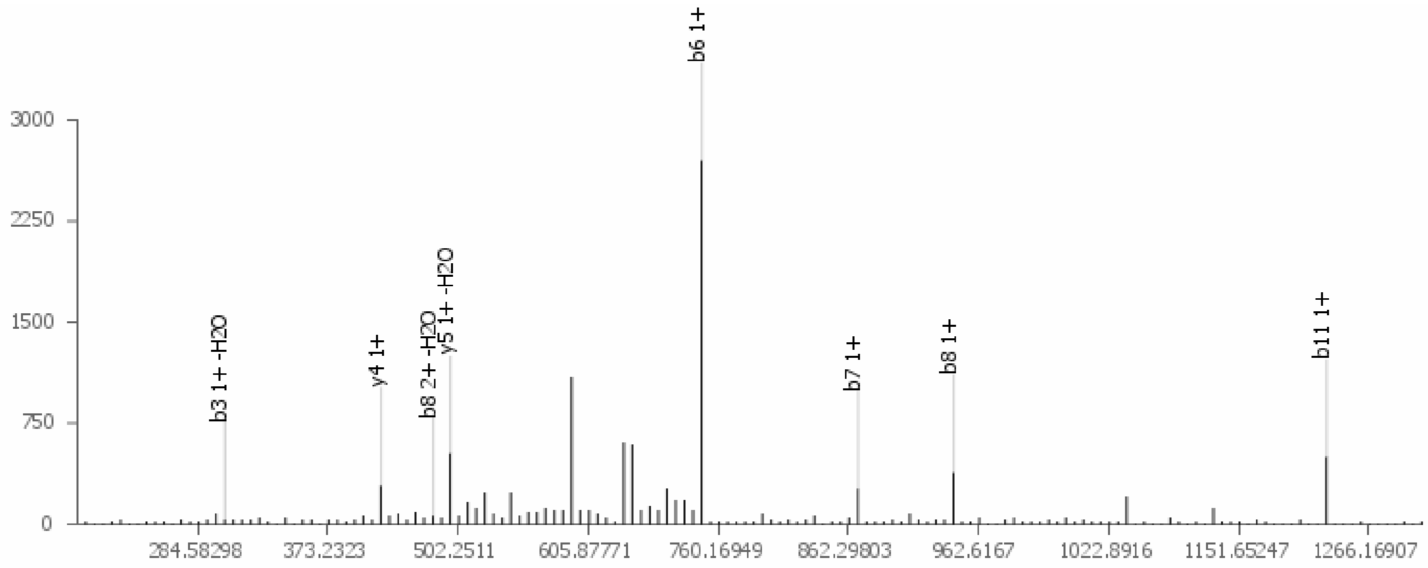

AT3G14970.1 - (pY)IDVVTELIDAILDLKK - 1021.038487 - Charge:2

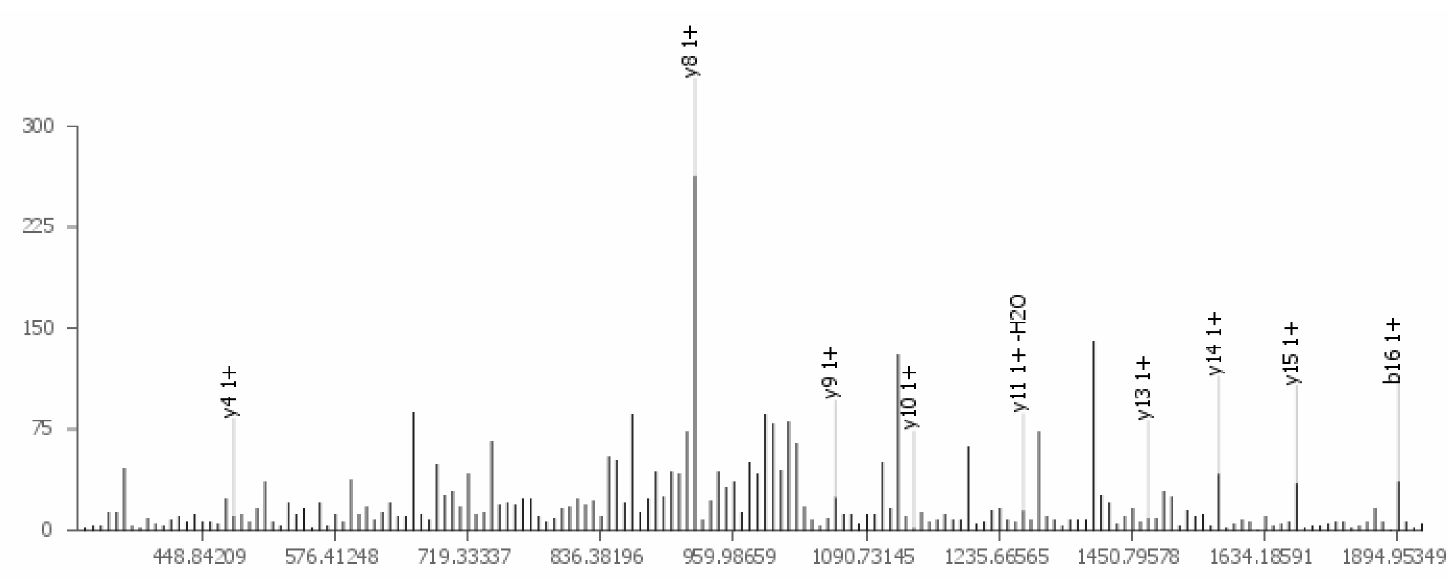

AT5G20320.1 - DIG(s)(s)LSLLPSI(oxM)HR - 574.61178 - Charge:3

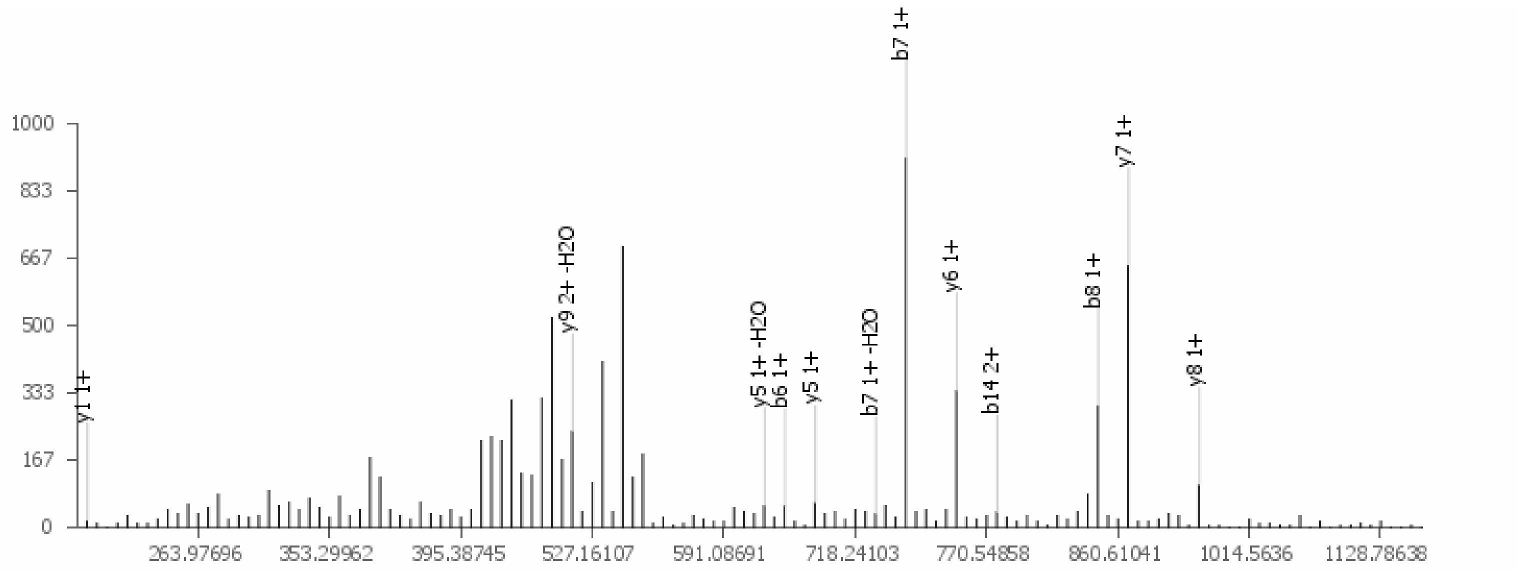

AT2G41240.1 - KINTMF(s)(s)LR - 638.807891 - Charge:2

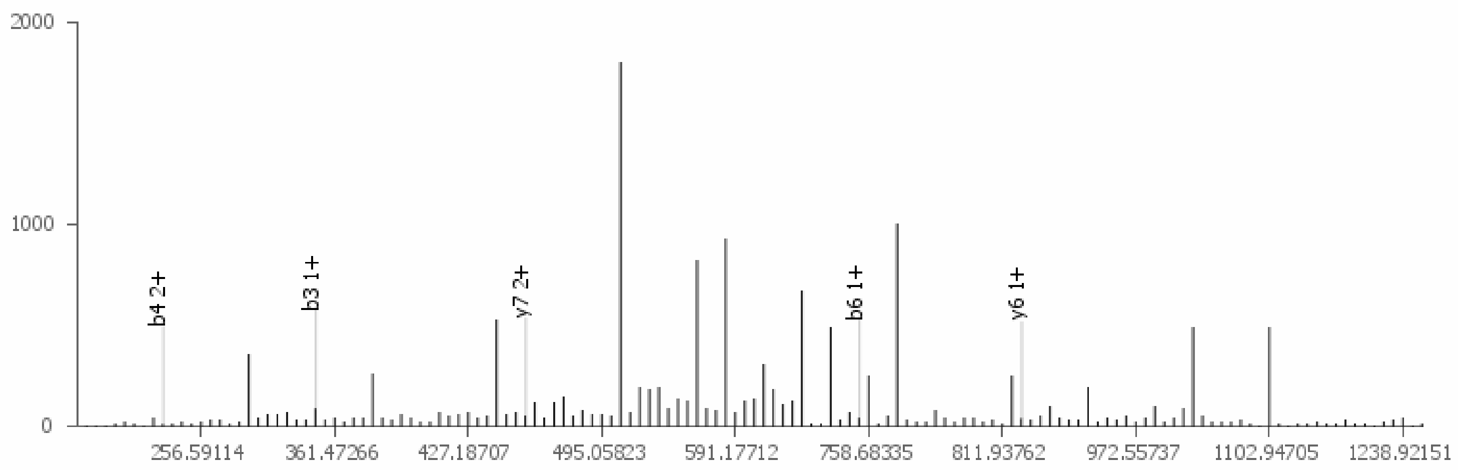

AT2G33740.1 - LSAVIG(pS)RR - 519.771655 - Charge:2

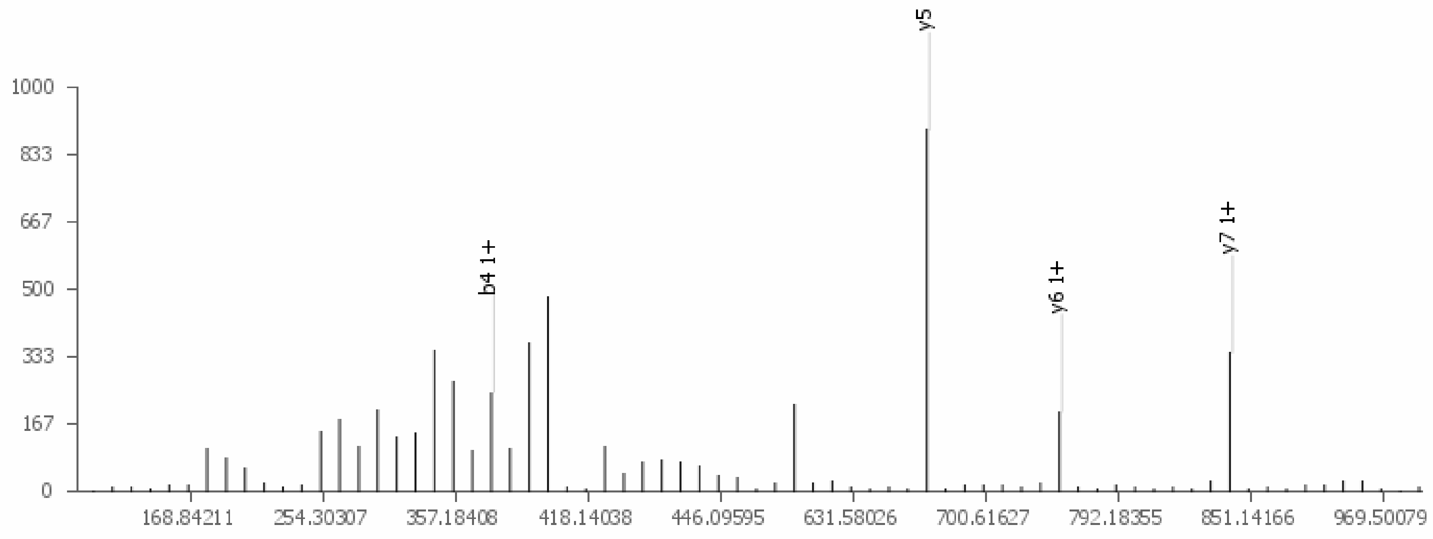

AT5G20610.1 - EVEKI(pS)EALYVNK - 801.388764 - Charge:2

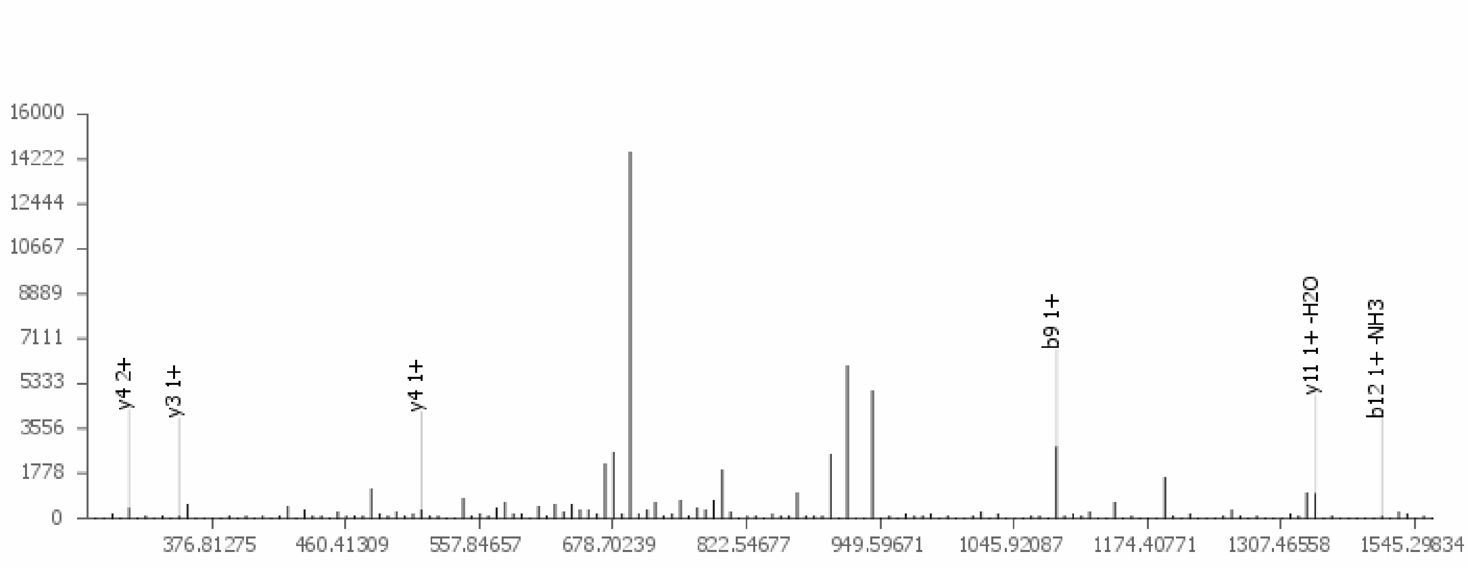

AT1G69870.1 - IS(pS)PGSILDAEK - 648.808803 - Charge:2

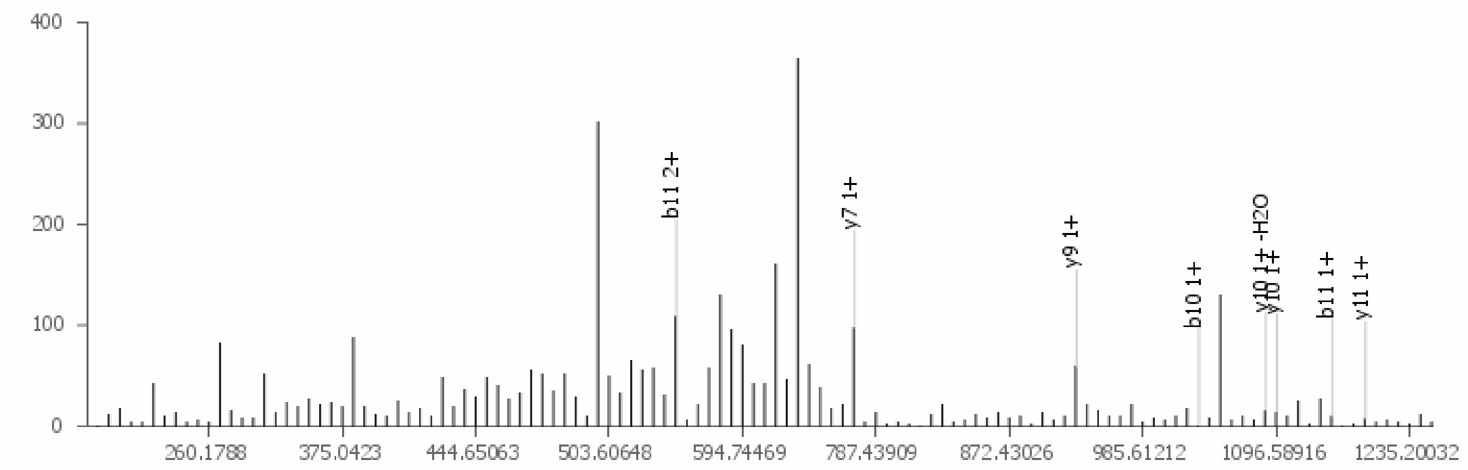

AT3G60680.1 - LRALE(pT)VSNR - 619.817049 - Charge:2

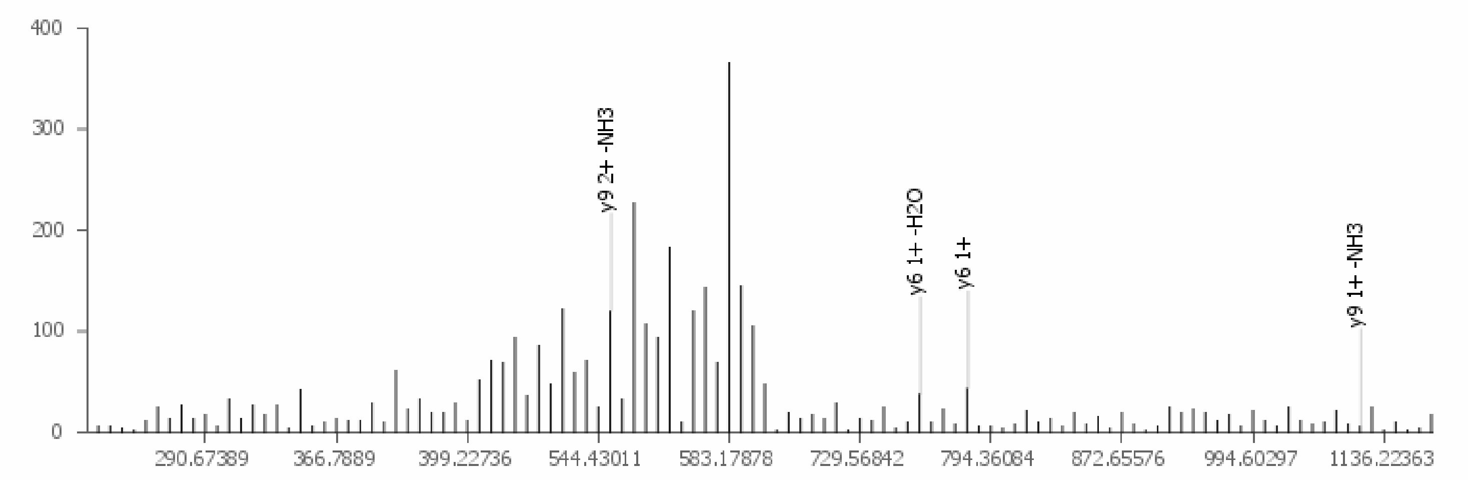

AT3G59190.1 - VNFD(pS)LVEASIGLR - 800.391916 - Charge:2

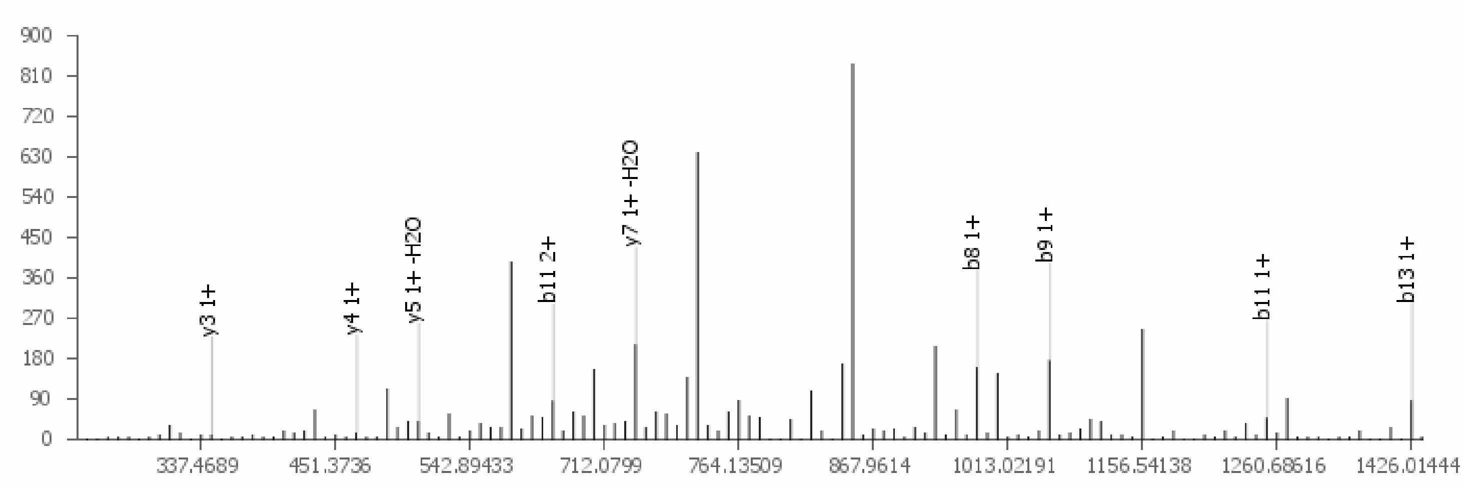

AT1G22830.1 - THDDE(pS)VPQVLFNSFR - 985.941688 - Charge:2

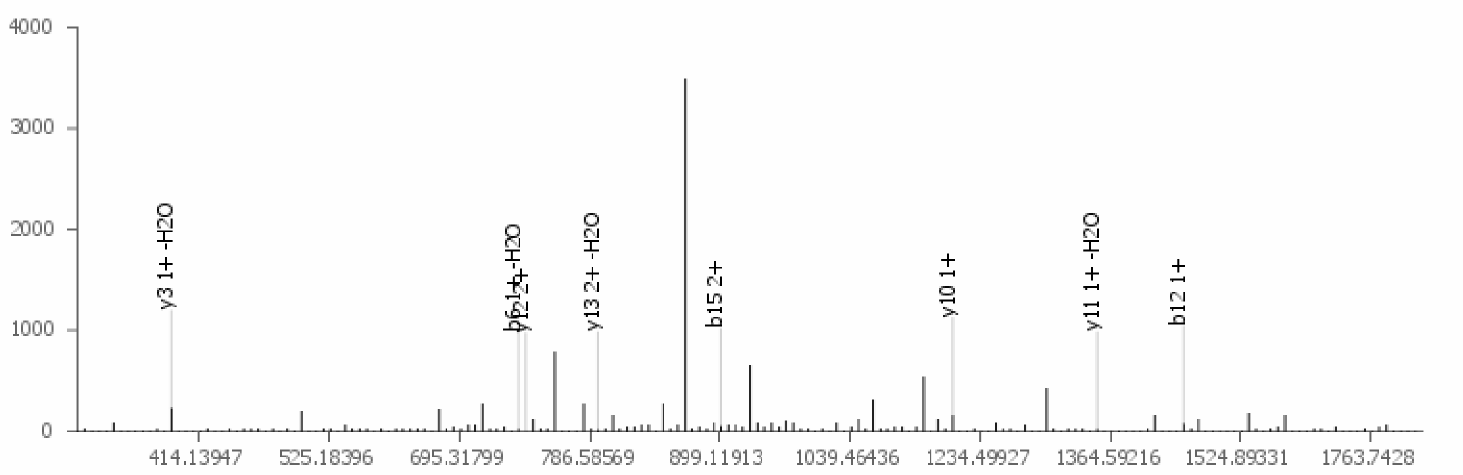

AT1G63660.1 - SLNVFSLVI(pS)G(t)(s)(s)LK - 906.430395 - Charge:2

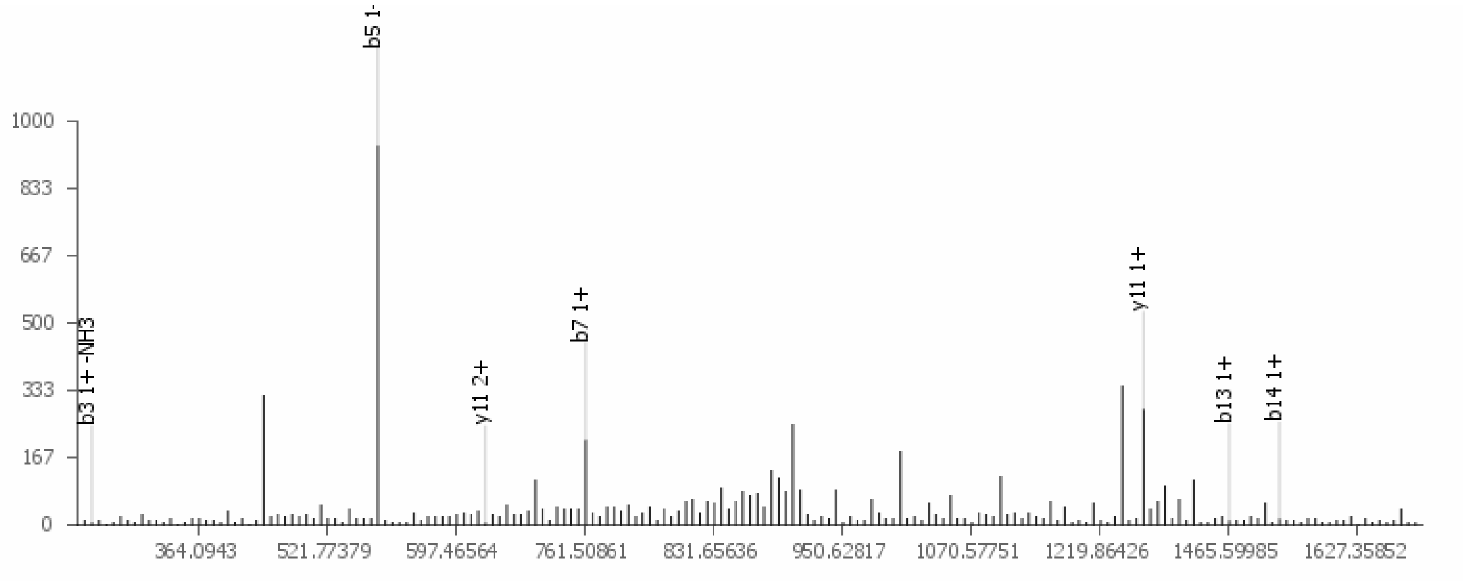

AT3G21100.1 - SLSIG(pS)PVHFSPR - 732.358825 - Charge:2

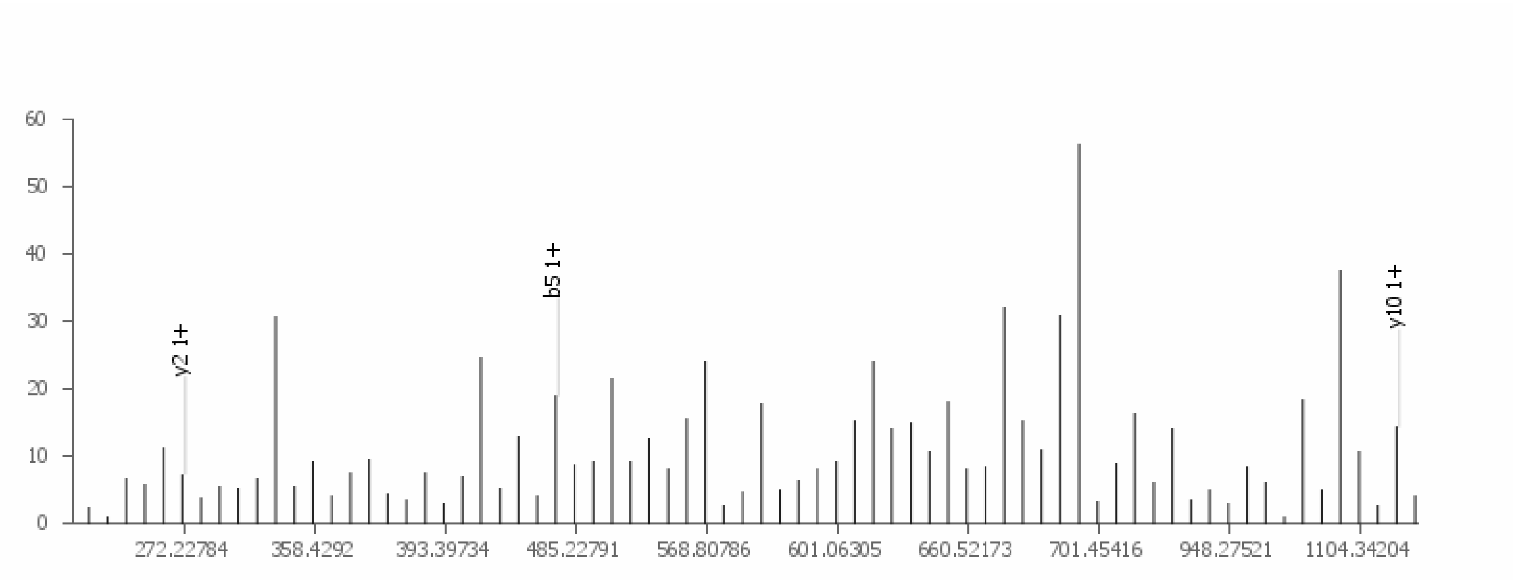

AT1G06690.1 - LGG(pS)DLKVTK - 549.28669 - Charge:2

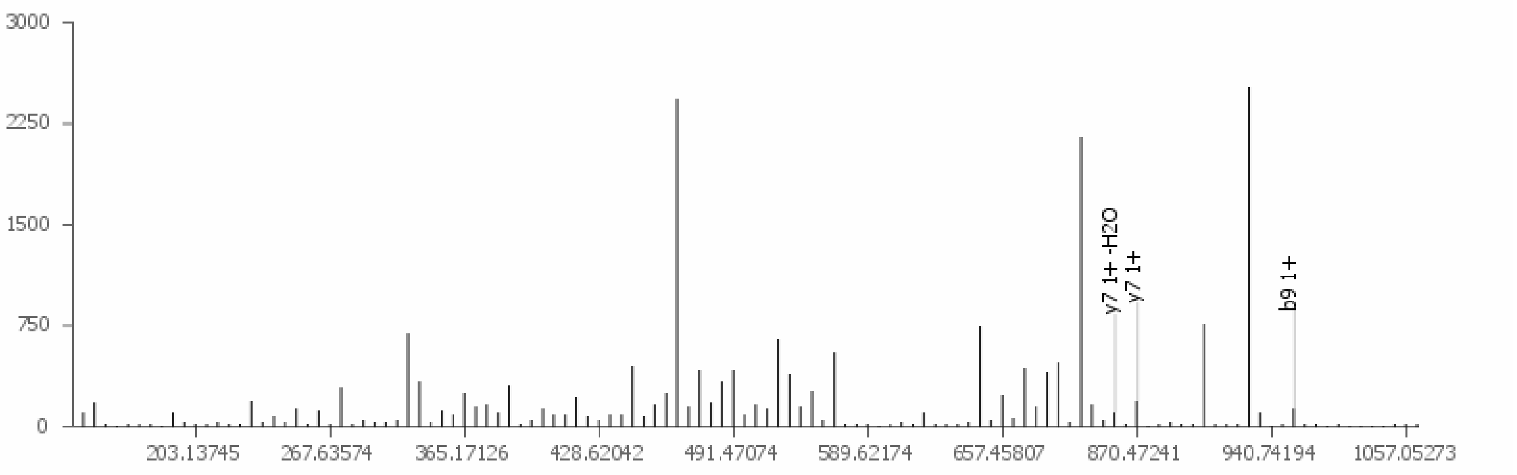

AT1G18680.1 - TVAELLGQ(pS)K - 563.28072 - Charge:2

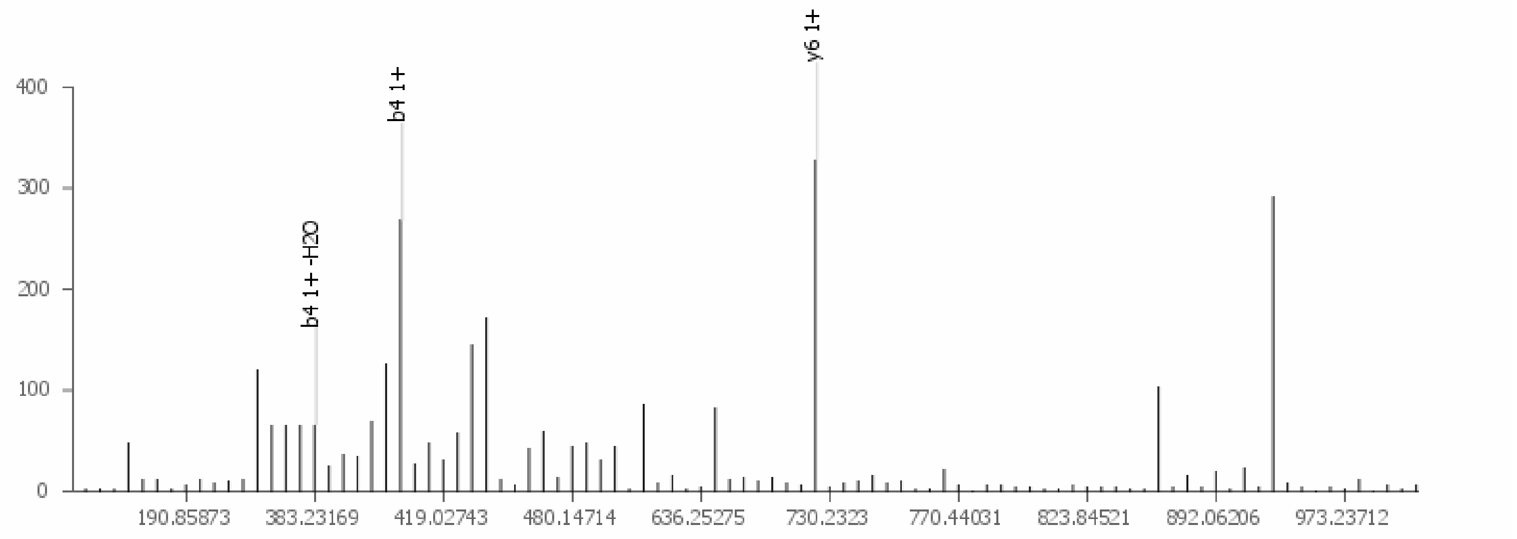

AT1G71710.1 - TVFH(pS)VSALGLPK - 718.373846 - Charge:2

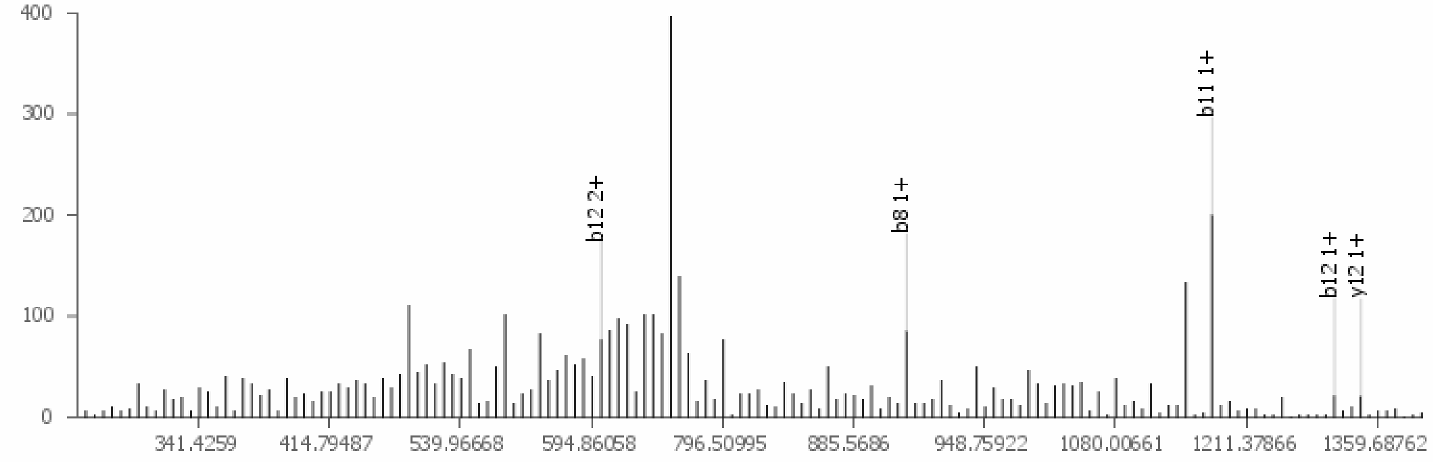

AT1G73920.1 - ISKTNS(pS)K - 472.725385 - Charge:2

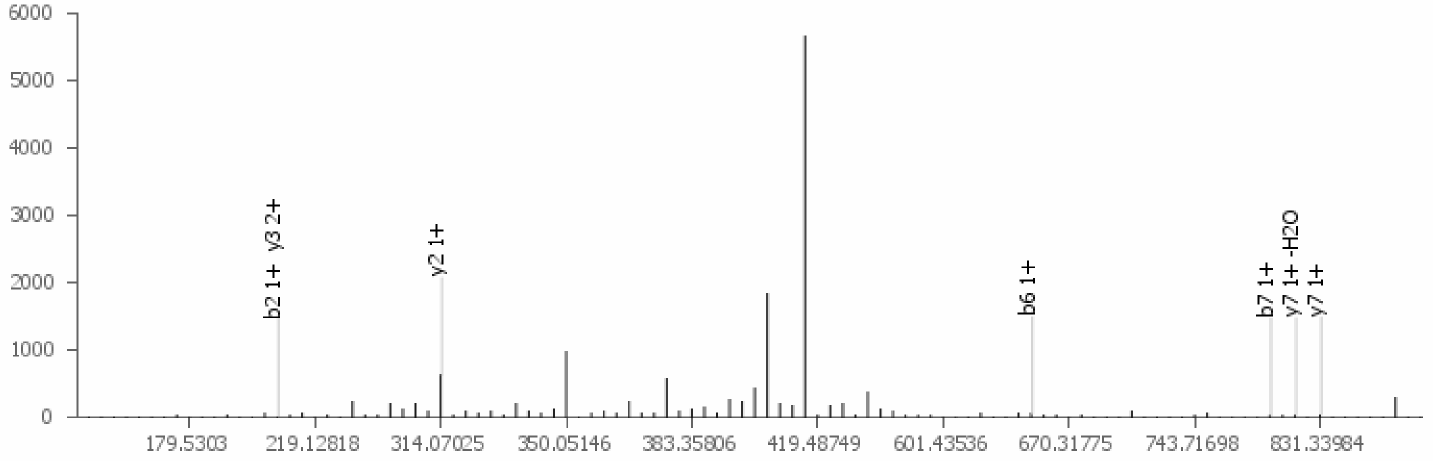

AT1G61970.1 - GHKTISV(pY)(pY)DFIK - 865.884605 - Charge:2

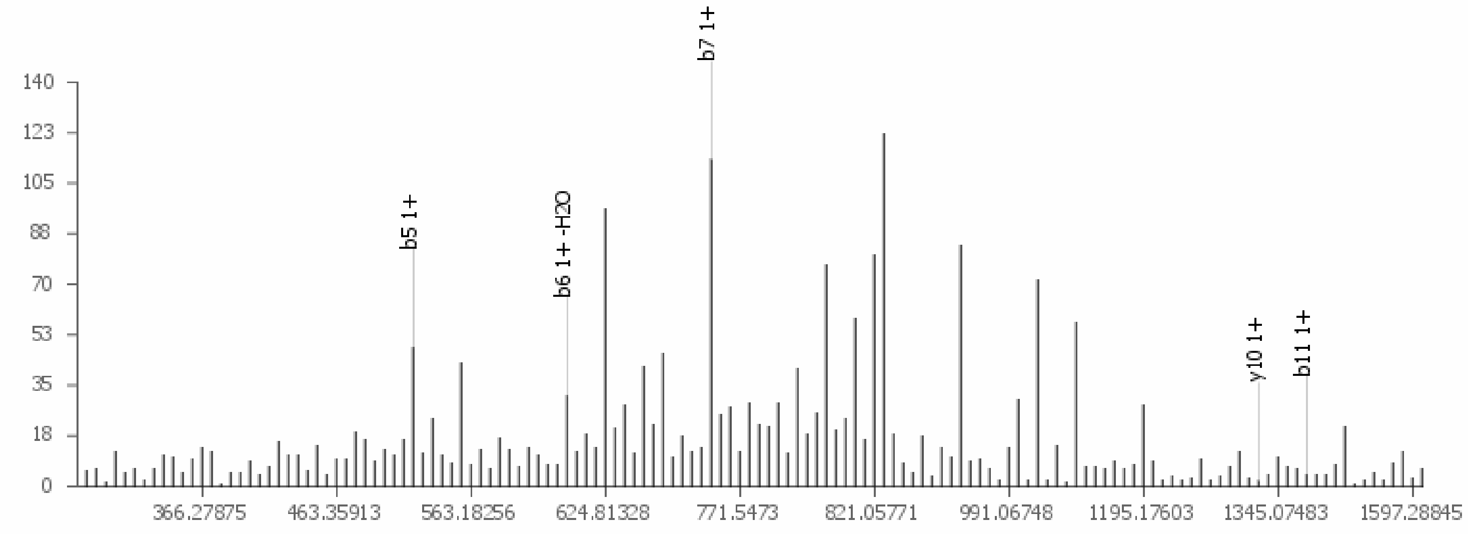

AT1G30820.1 - GLGL(pT)PDILACR - 654.824819 - Charge:2

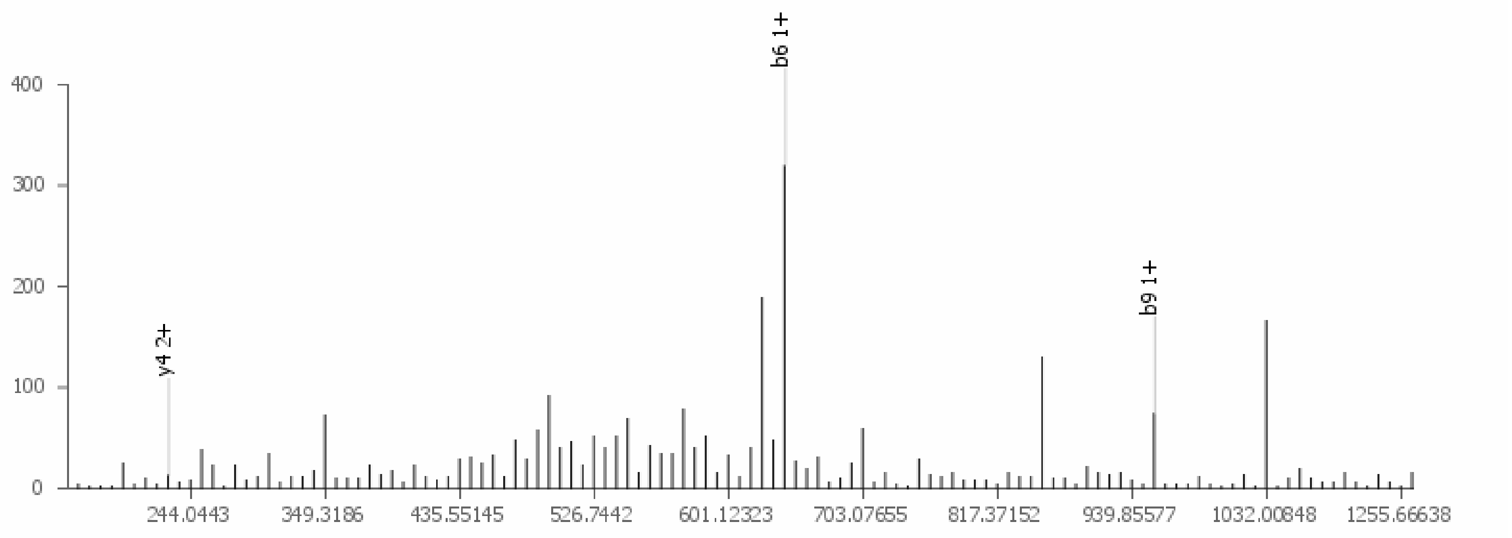

AT1G02220.1 - S(pT)GRPKPIMR - 611.81236 - Charge:2

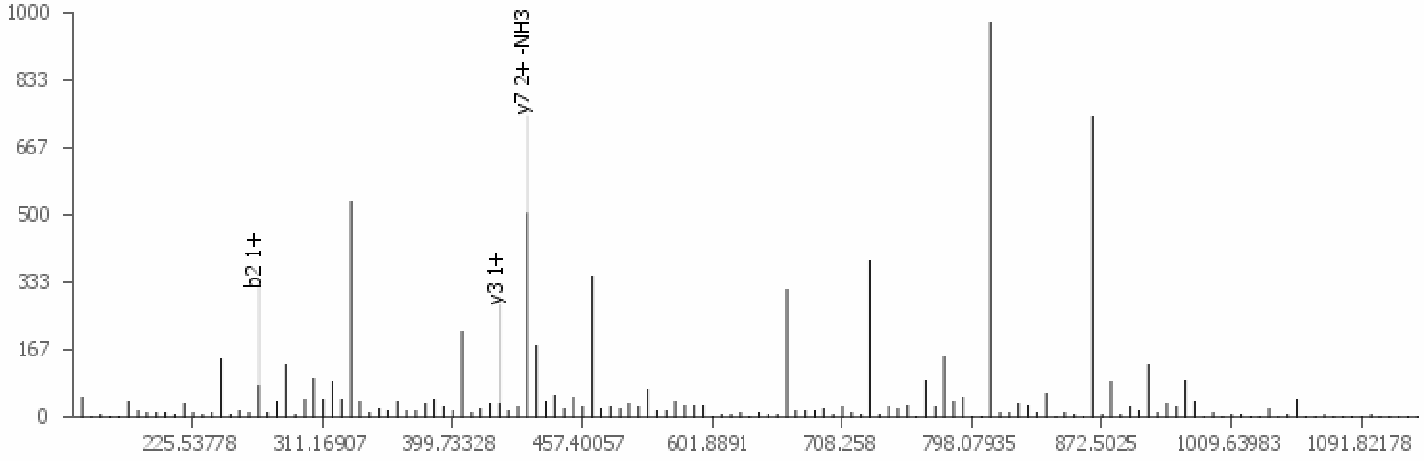

AT1G73960.1 - ILQKII(pS)R - 525.809399 - Charge:2

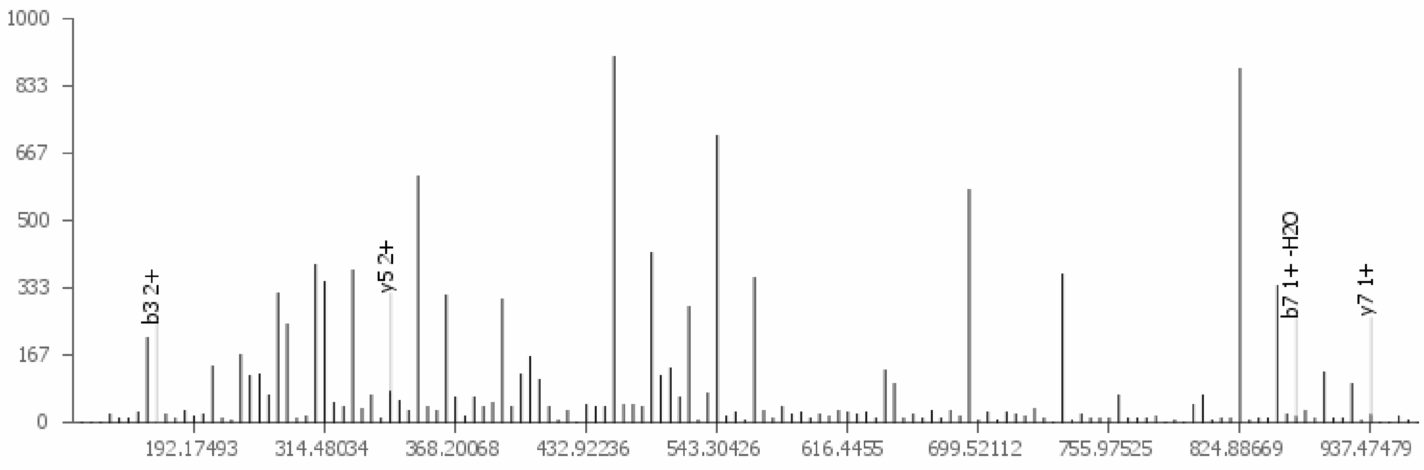

AT1G71880.1 - (pT)AGDLAGPSASVK - 627.294583 - Charge:2

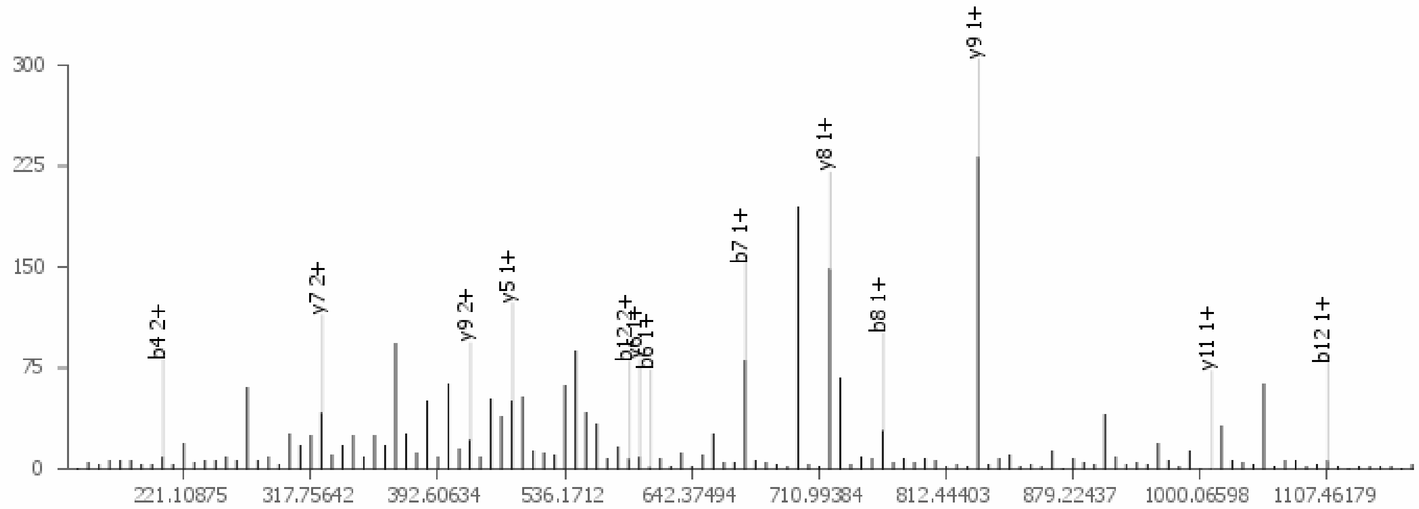

AT5G42120.1 - MPGRL(pS)LAEIK - 647.838118 - Charge:2

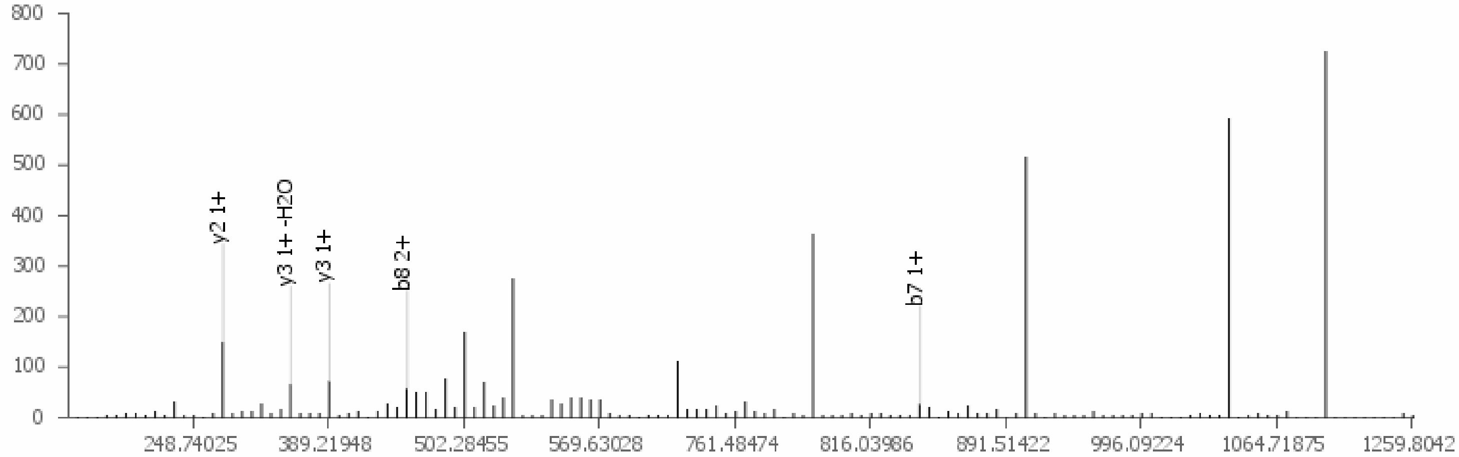

AT1G08060.1 - ML(pT)HIKK - 475.743681 - Charge:2

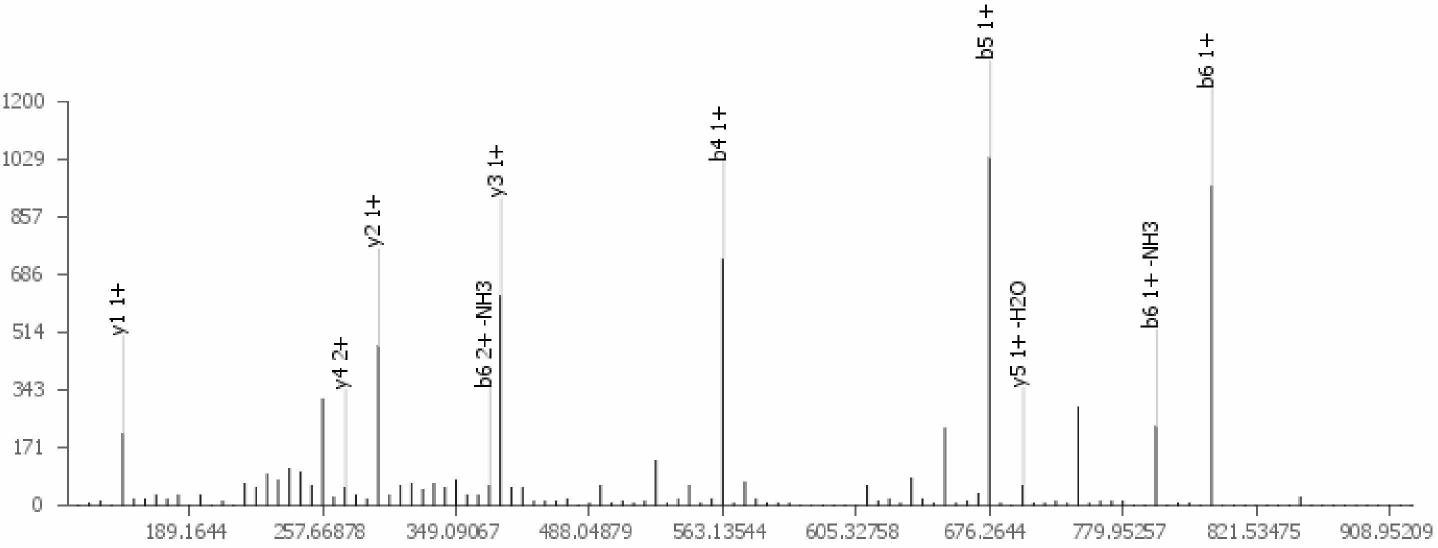

AT2G34660.1 - LASLAEN(pS)LNAVER - 783.880435 - Charge:2

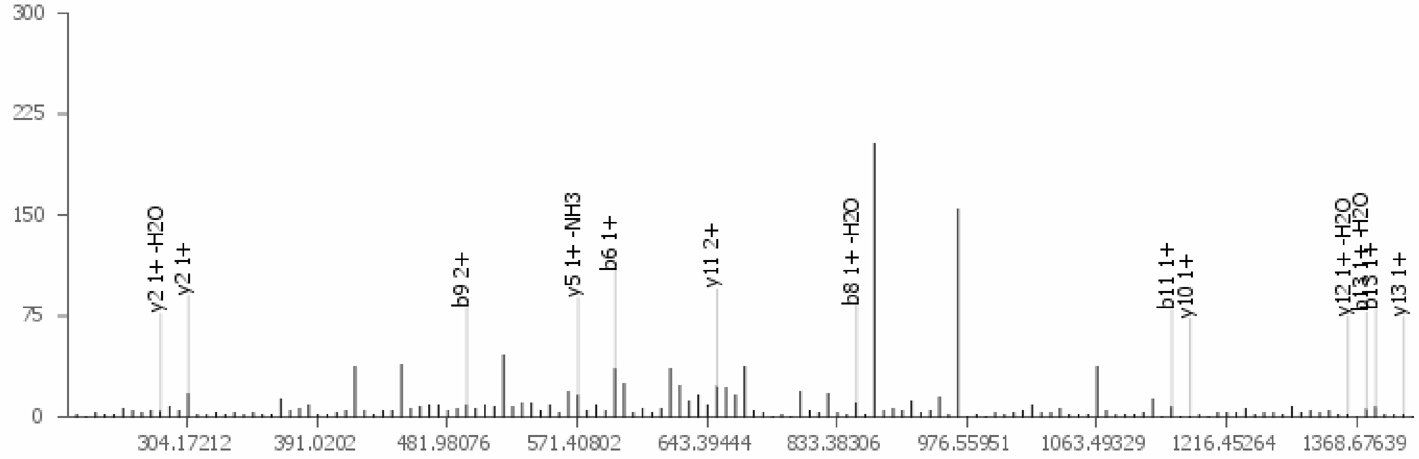

AT3G49850.1 - (pY)LTNVGTLVKK - 658.352893 - Charge:2

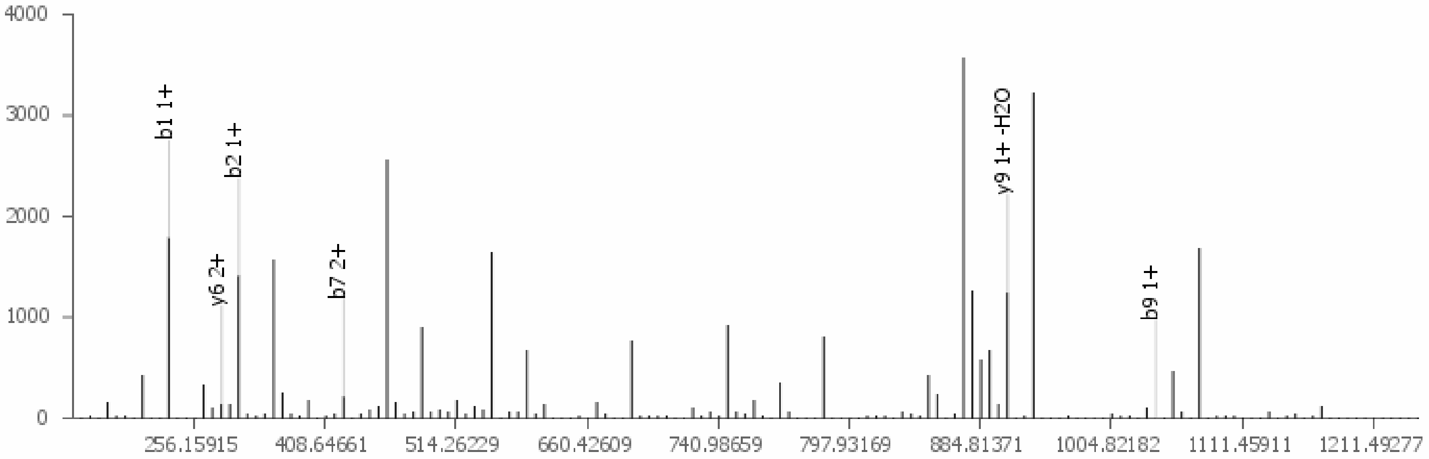

AT5G46020.1 - FSSAADILAG(t)(s)AARPRSFK - 1067.019674 - Charge:2

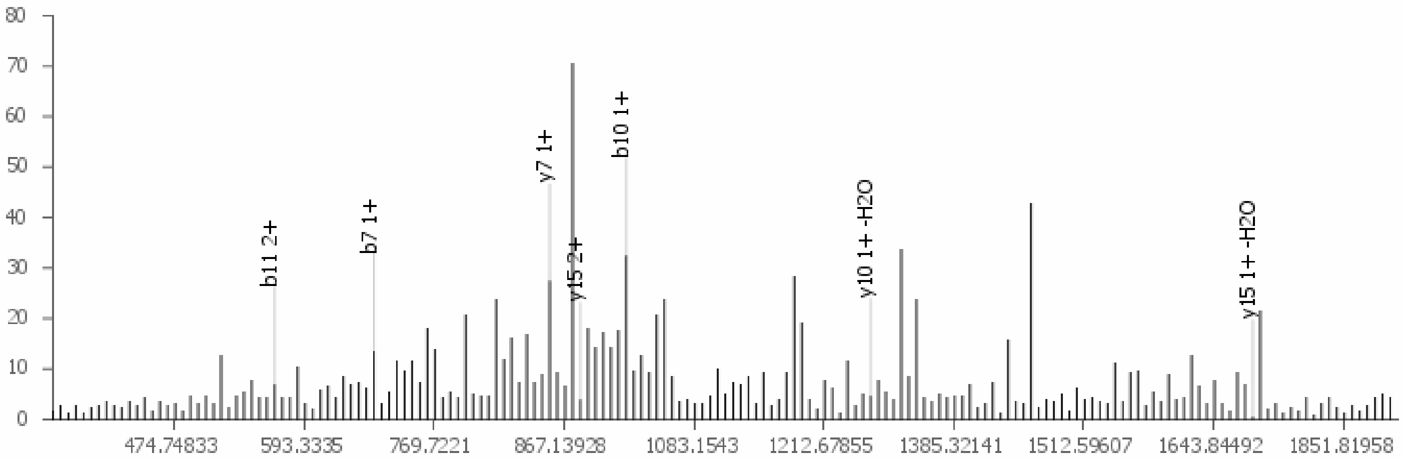

AT1G66600.1 - TIK(pT)SLYQR - 595.2967 - Charge:2

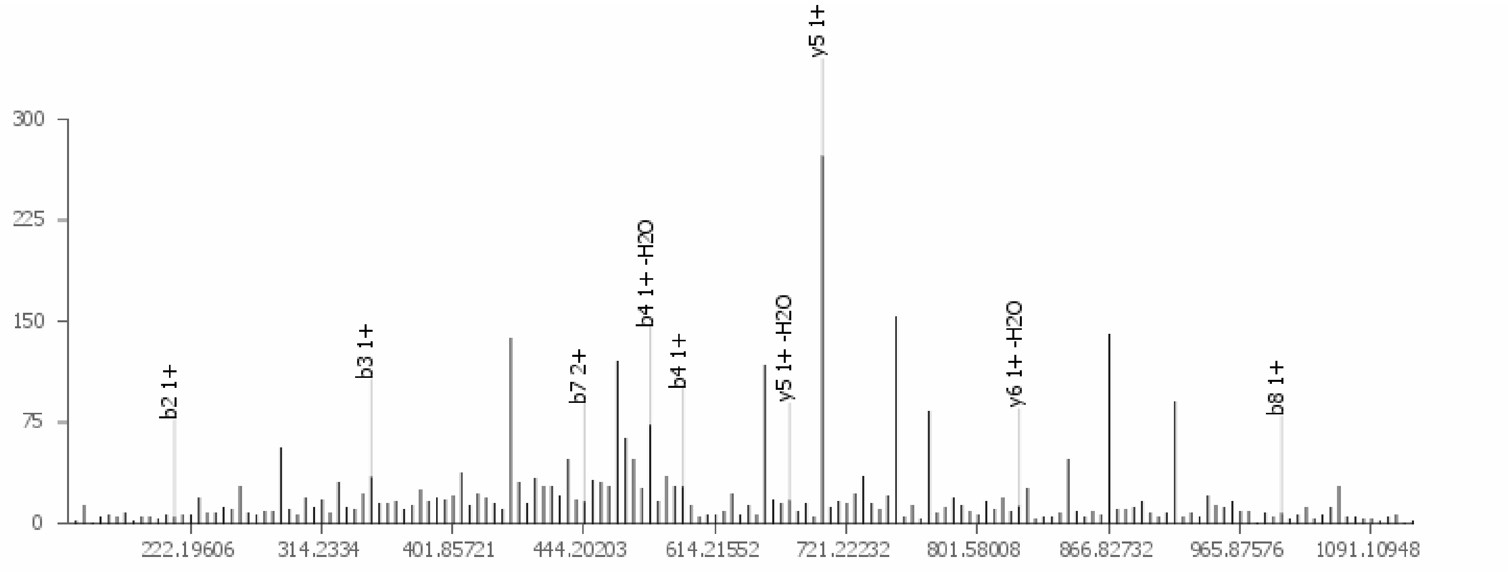

AT4G29170.1 - GL(pS)LEEKR - 506.245041 - Charge:2

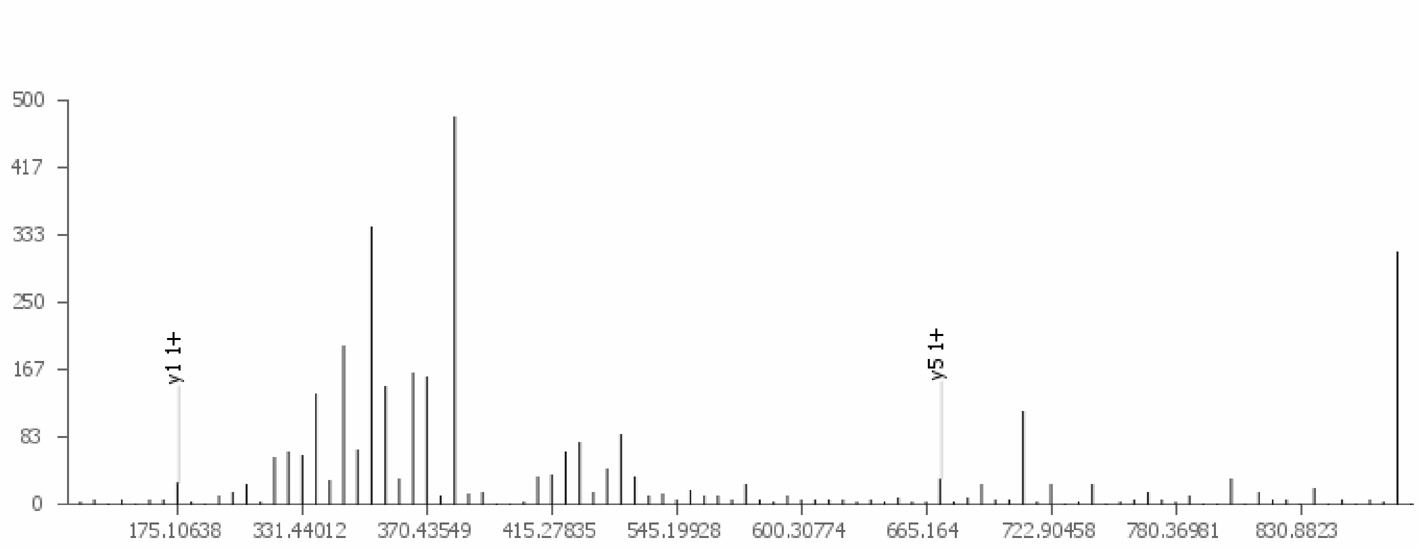

AT3G55360.1 - MKV(pT)VVSR - 500.260923 - Charge:2

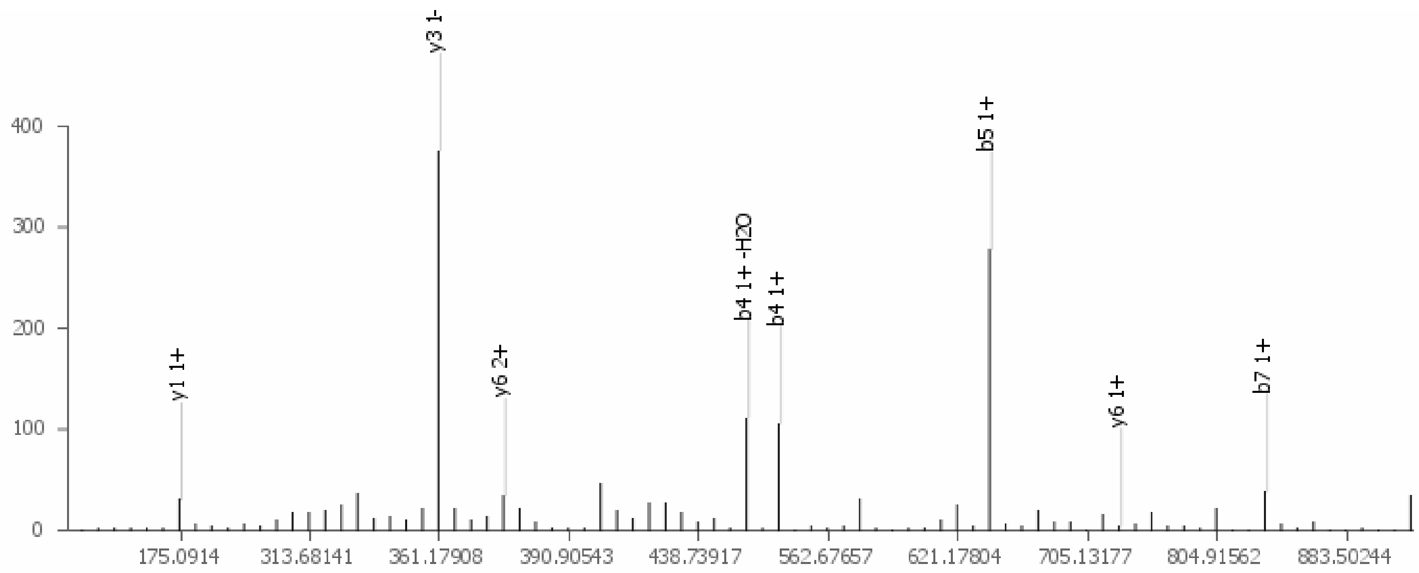

AT3G14040.1 - VTGAKPGGA(pS)LDVK - 690.348052 - Charge:2

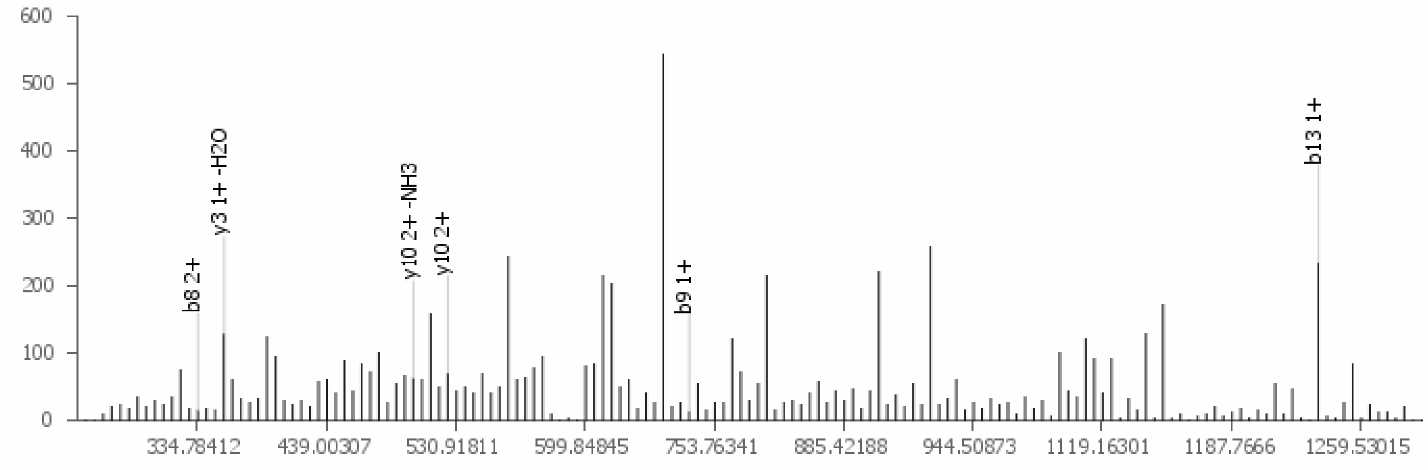

AT2G34680.1 - VKVLDL(pS)FNDFK - 752.871653 - Charge:2

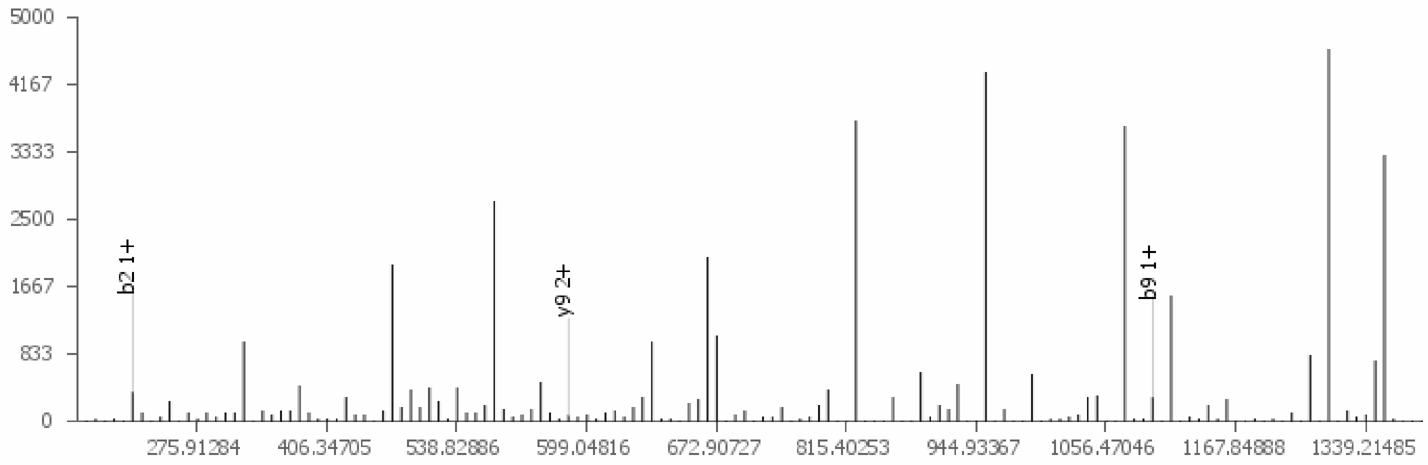

AT5G17820.1 - LASDPQ(pT)RGIVAR - 732.365953 - Charge:2

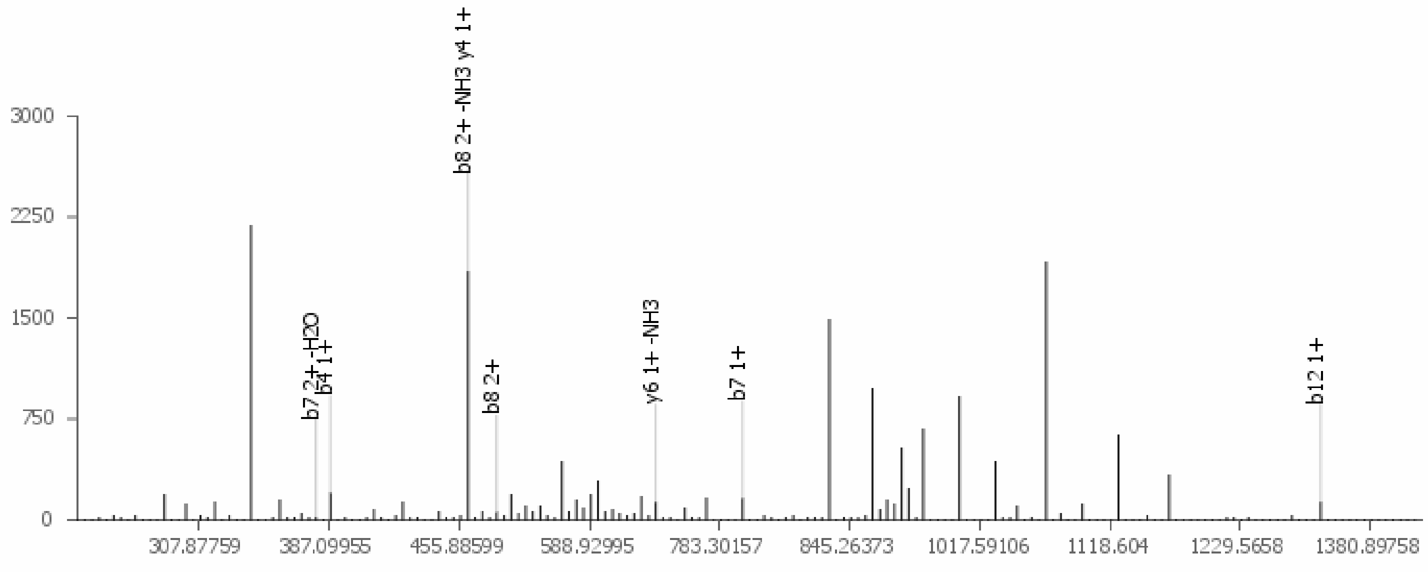

AT3G15354.1 - SKLSSLCWNS(pY)IK - 804.880869 - Charge:2

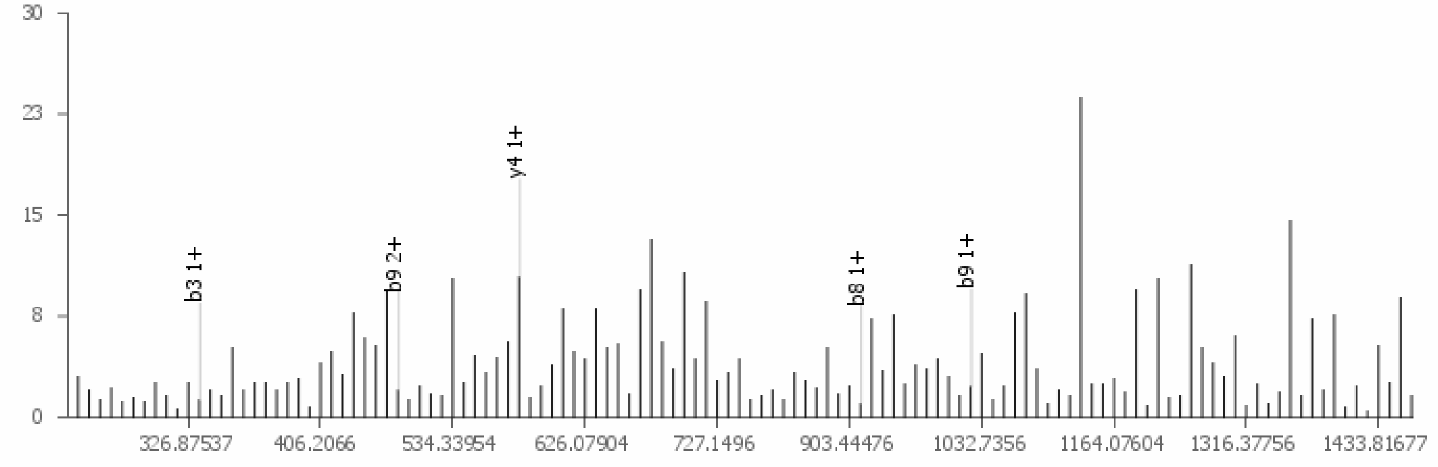

AT5G50900.1 - (pT)VAVI(pS)RISMVESSK - 883.903978 - Charge:2

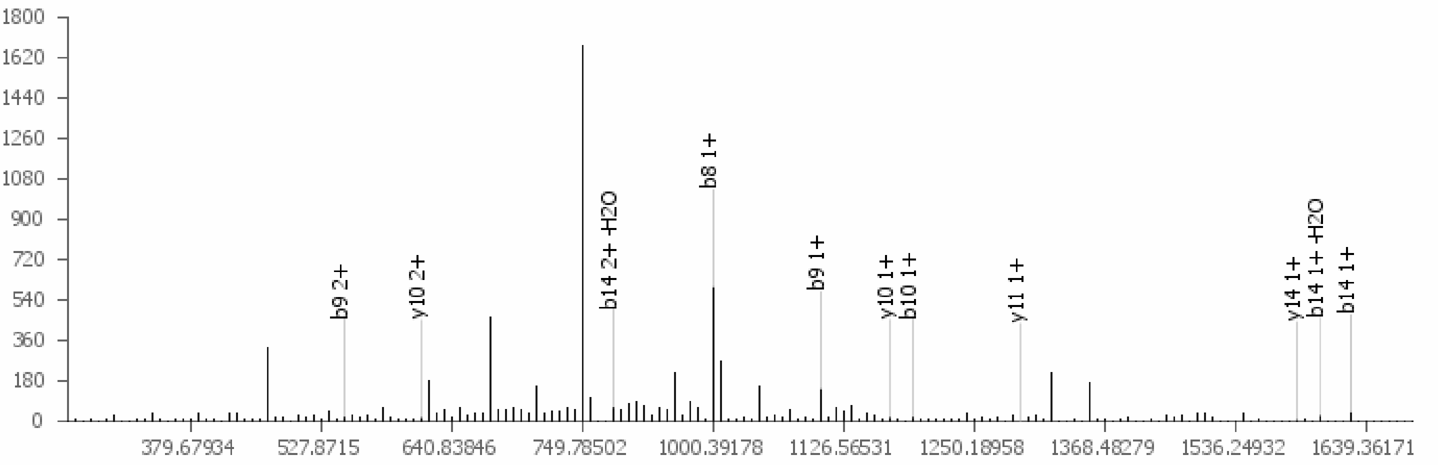

AT2G04330.1 - (oxM)AVLYFL(t)(s)(s)IVVP(pT)K - 972.971342 - Charge:2

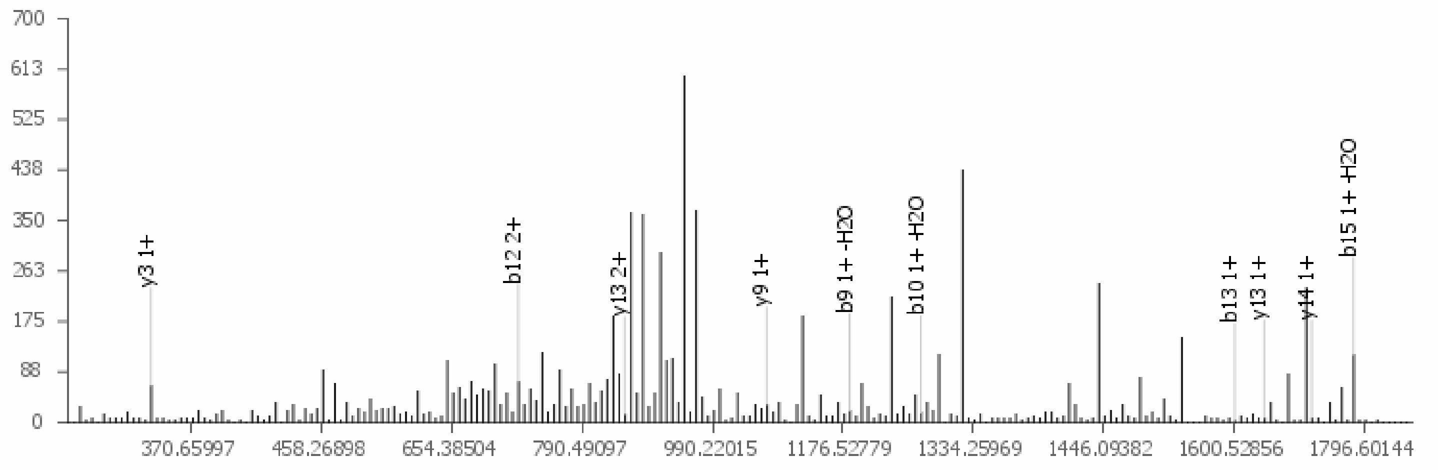

AT4G32880.1 - A(pS)(oxM)LLQNVPPSILLR - 874.459248 - Charge:2

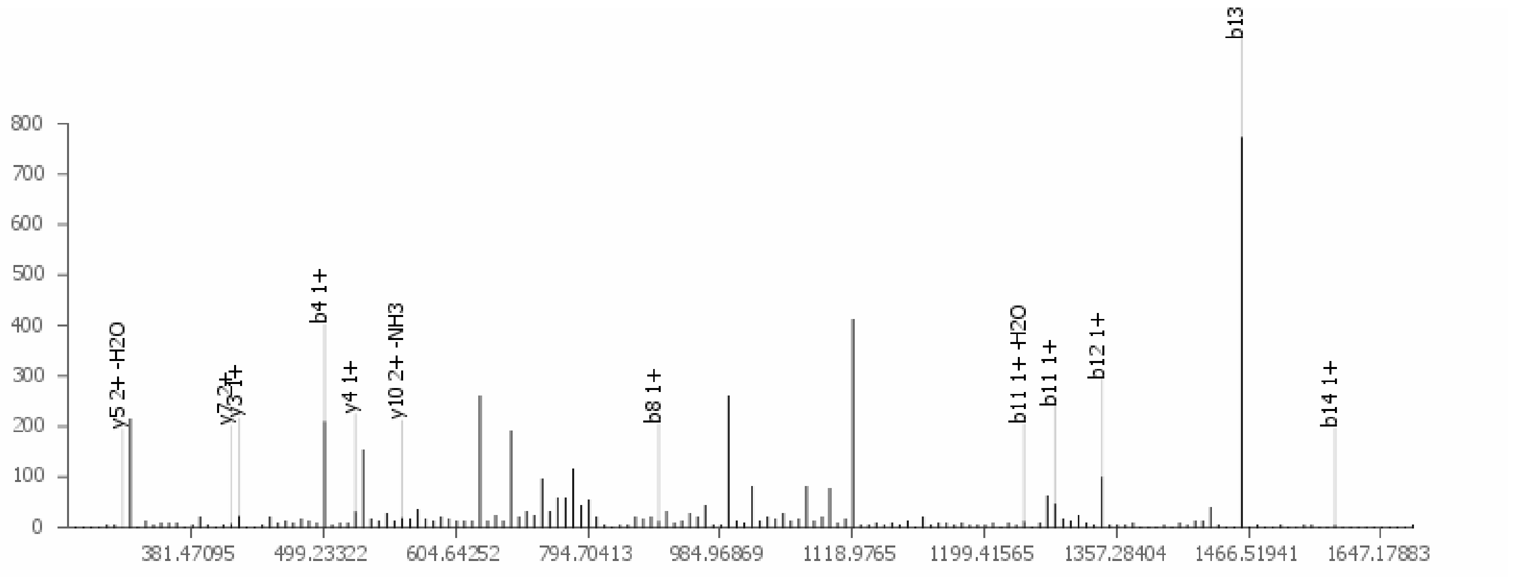

AT3G06770.1 - LSNI(pS)GENIGIAGK - 726.861578 - Charge:2

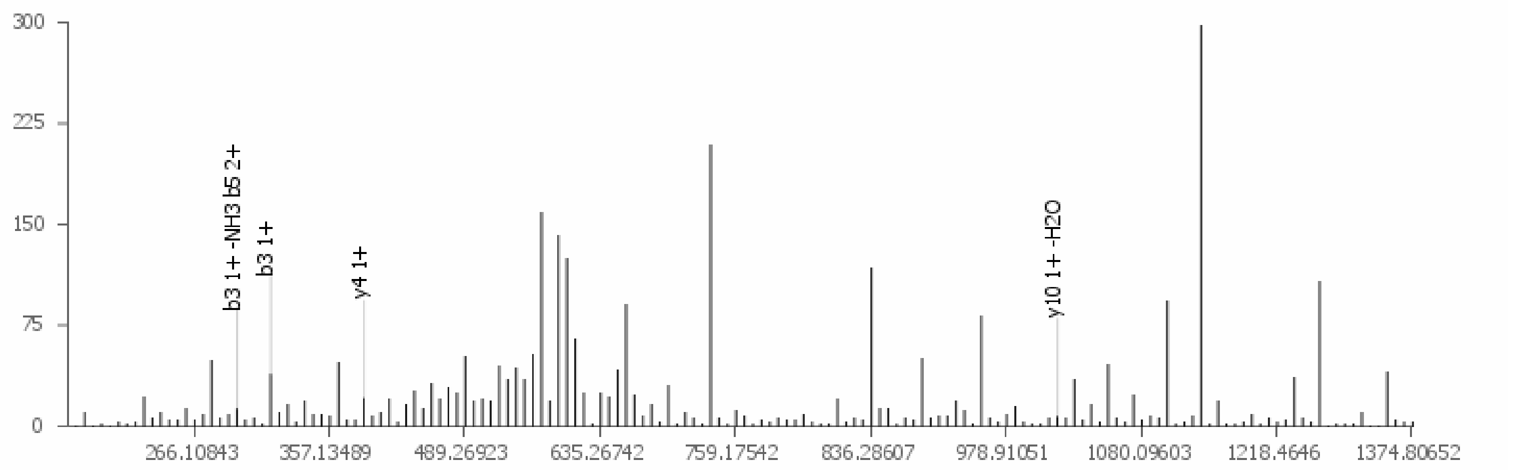

AT1G55200.1 - K(pS)QANWVVLDK - 684.344585 - Charge:2

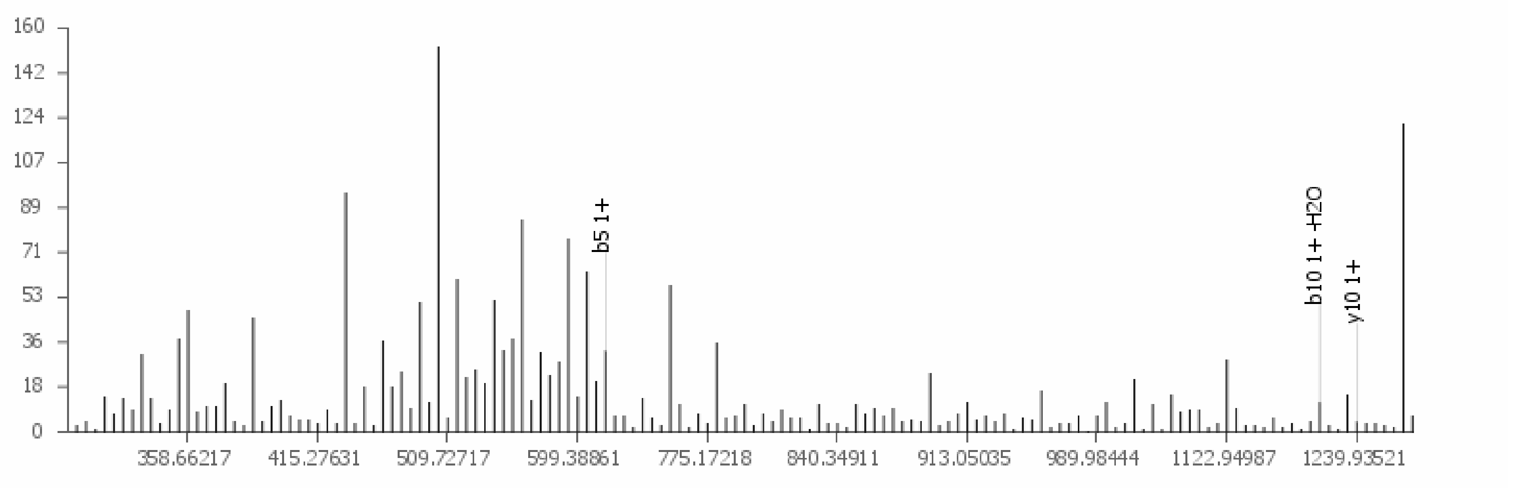

AT4G16890.1 - TG(pT)ETLLGIR - 570.790389 - Charge:2

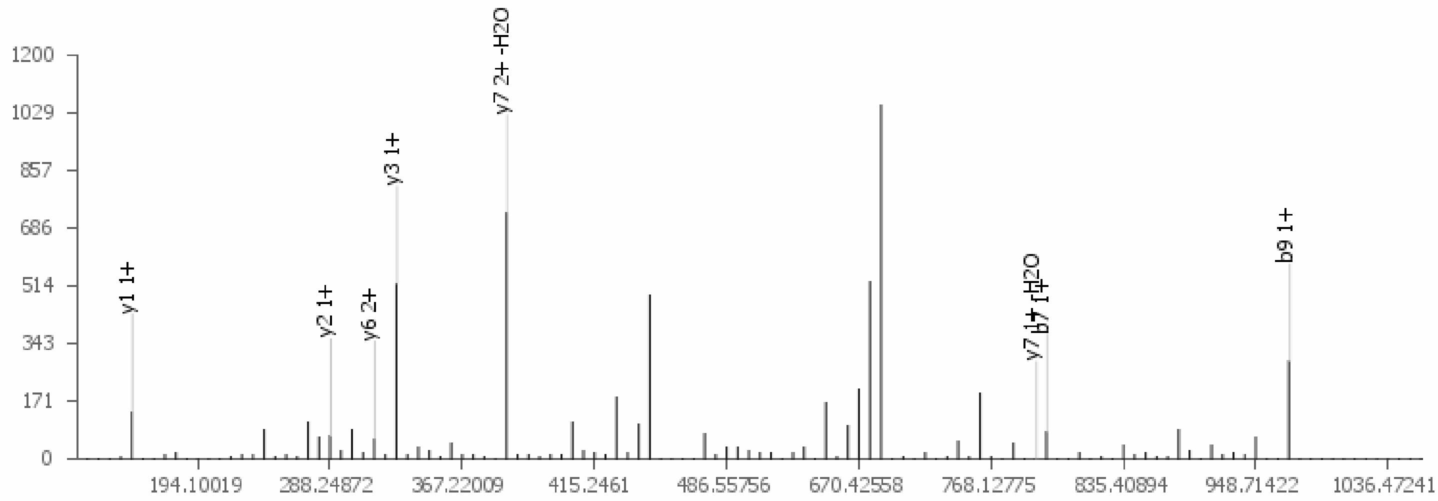

AT2G44980.1 - VLLF(s)QM(t)STLDILQDF(oxM)ELRR - 918.124753 - Charge:3

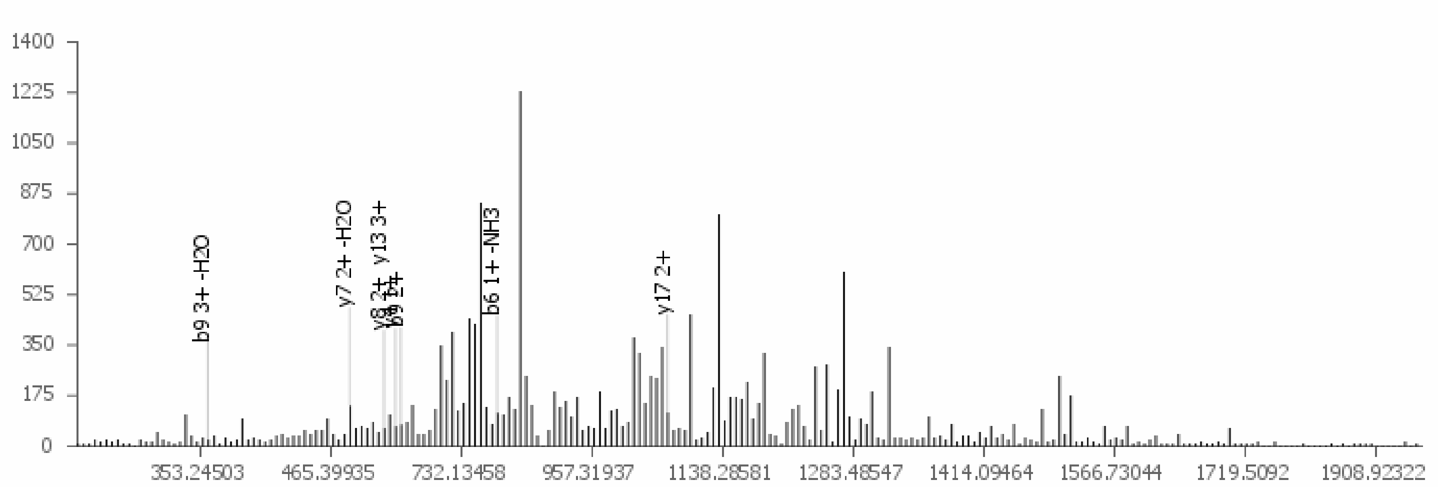

AT4G07400.1 - NIYIF(pS)KEK - 611.303618 - Charge:2

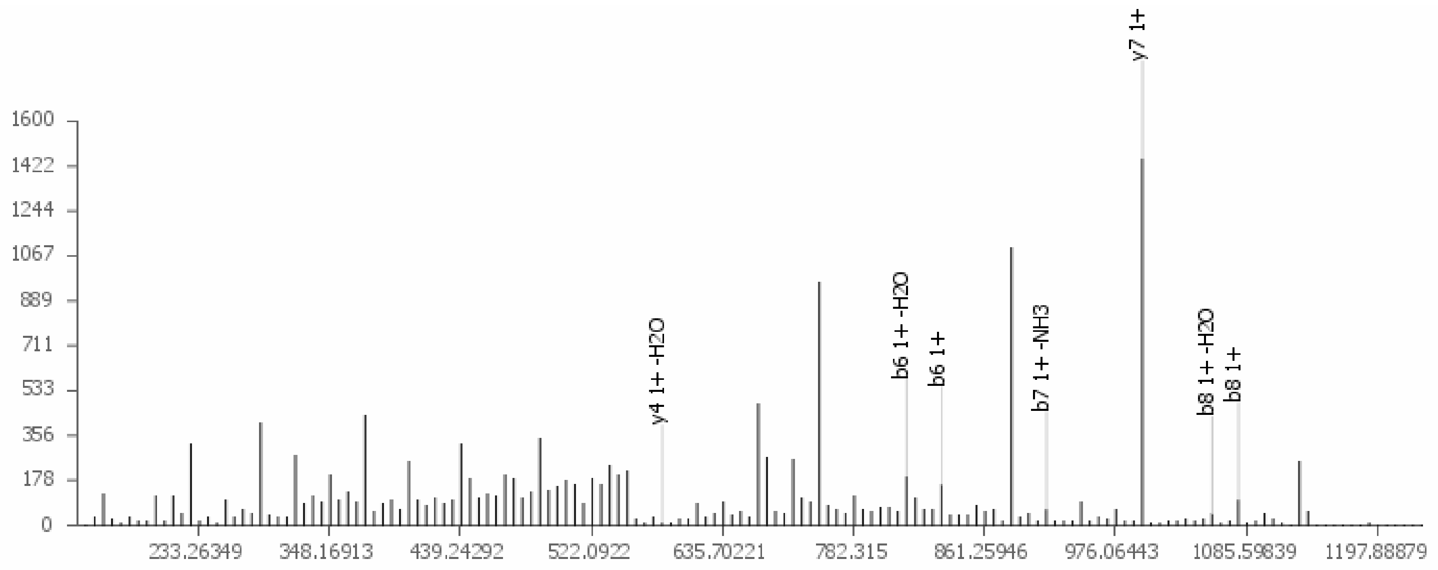

AT5G51960.1 - (oxM)(pS)GEVVLAAR - 564.76518 - Charge:2

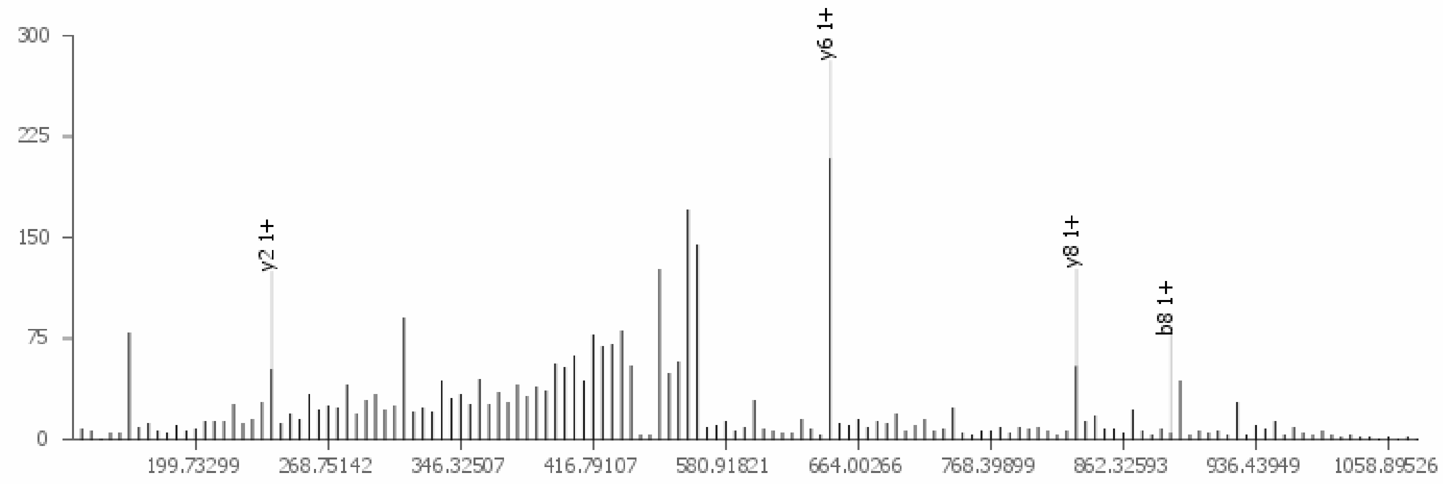

AT2G43470.1 - L(pS)HGARALAK - 552.291168 - Charge:2

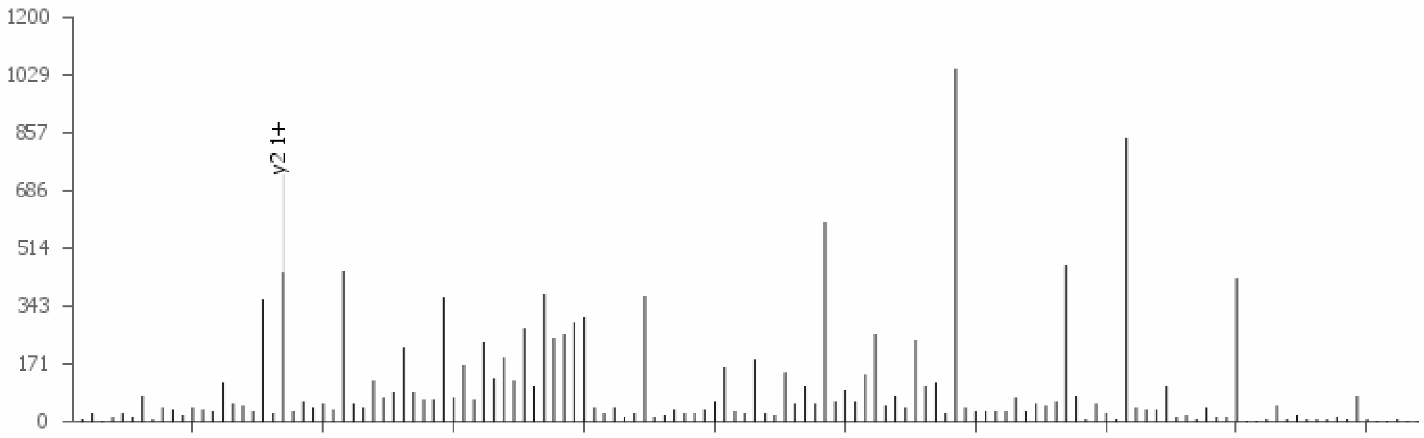

AT2G36325.1 - (pY)GRPR - 364.663171 - Charge:2

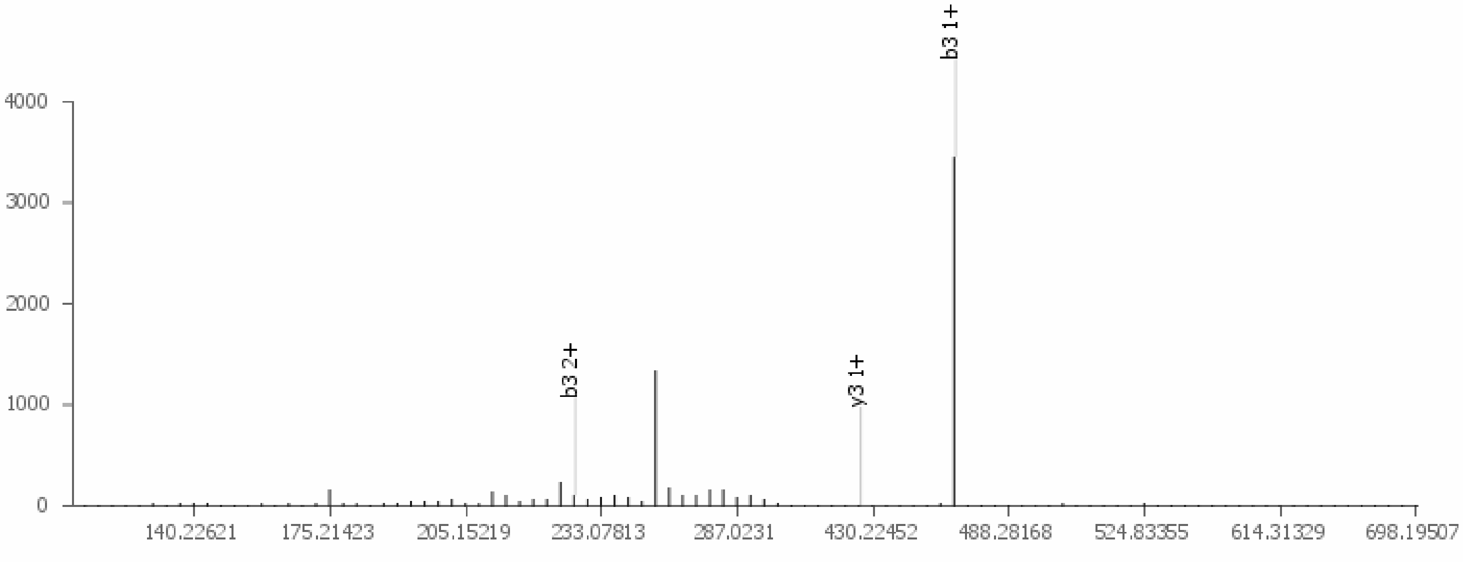

AT4G03150.1 - SSWVCSSV(pT)R - 624.753378 - Charge:2

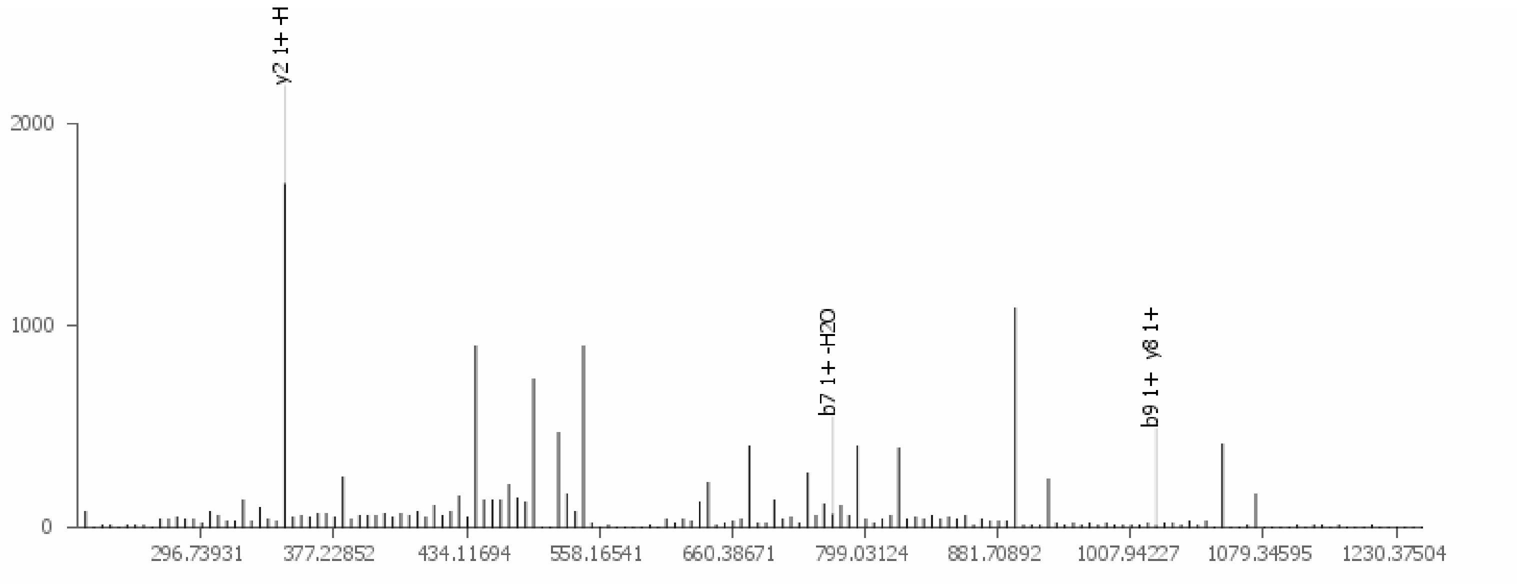

AT4G35390.1 - NKPKPPTII(pT)R - 672.871186 - Charge:2

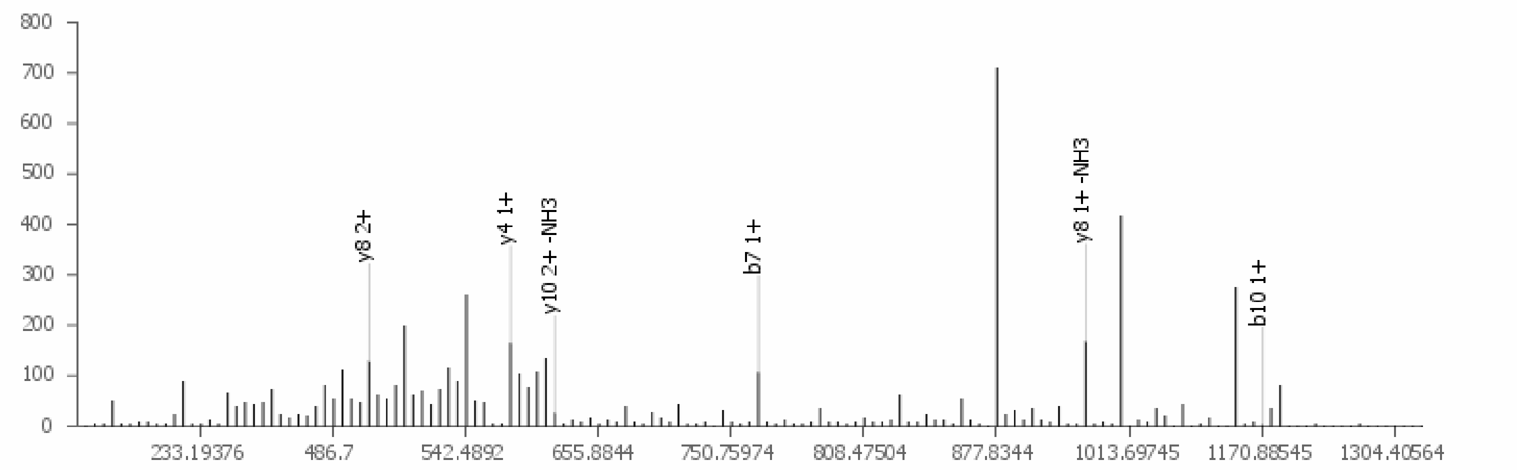

AT5G05990.1 - HLTP(pS)VDR - 502.736282 - Charge:2

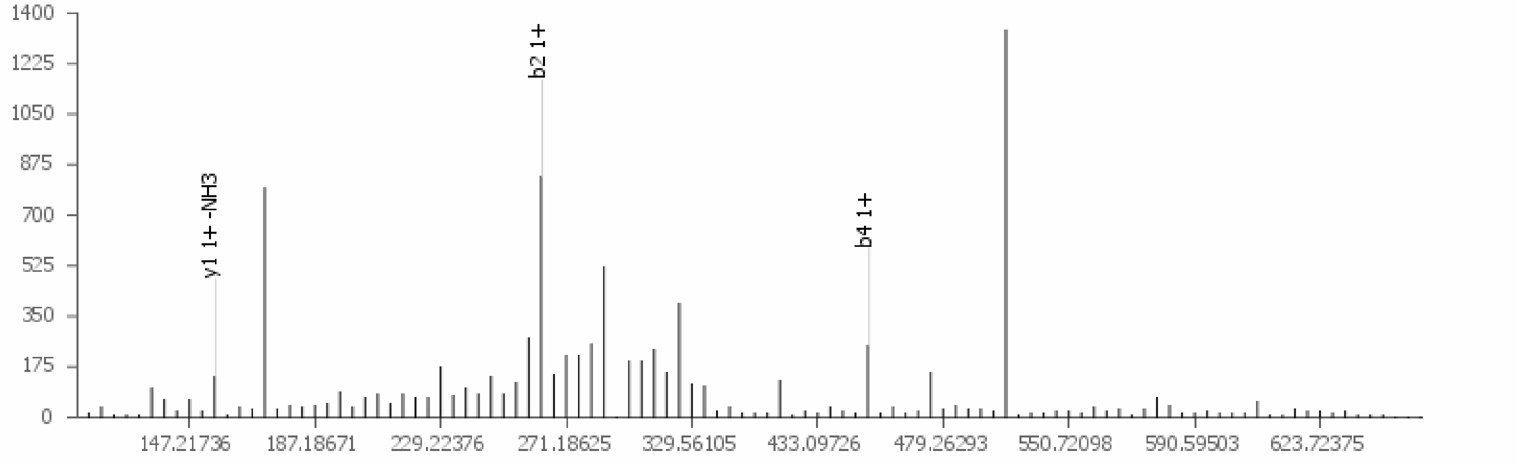

AT5G05140.1 - (pS)IKPSSATDFDGTRRPVK - 1021.513655 - Charge:2

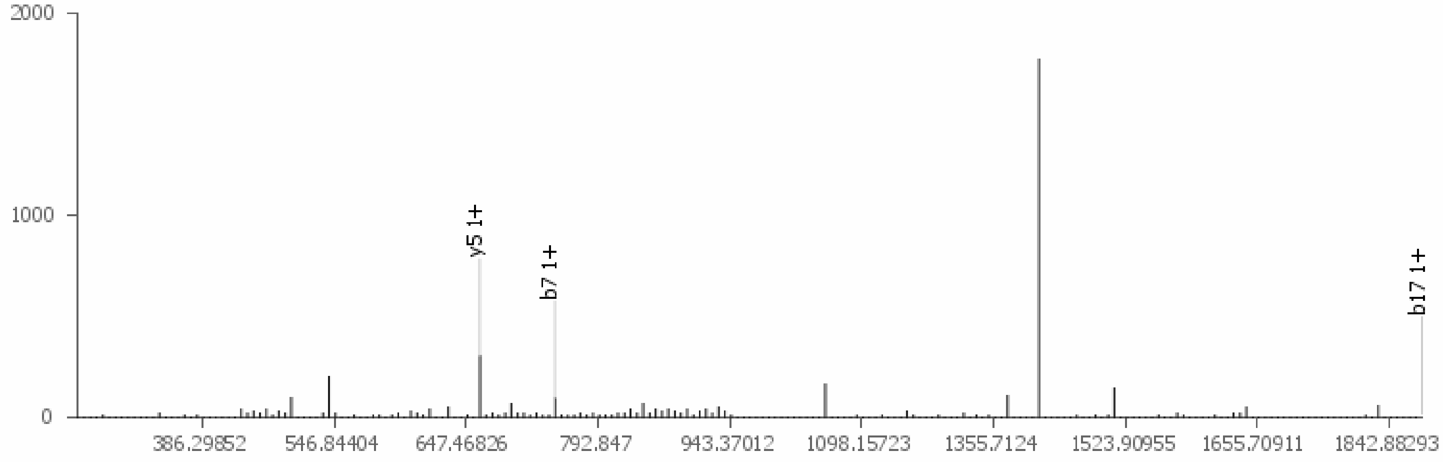

AT1G24250.1 - GF(pT)APR - 364.662878 - Charge:2

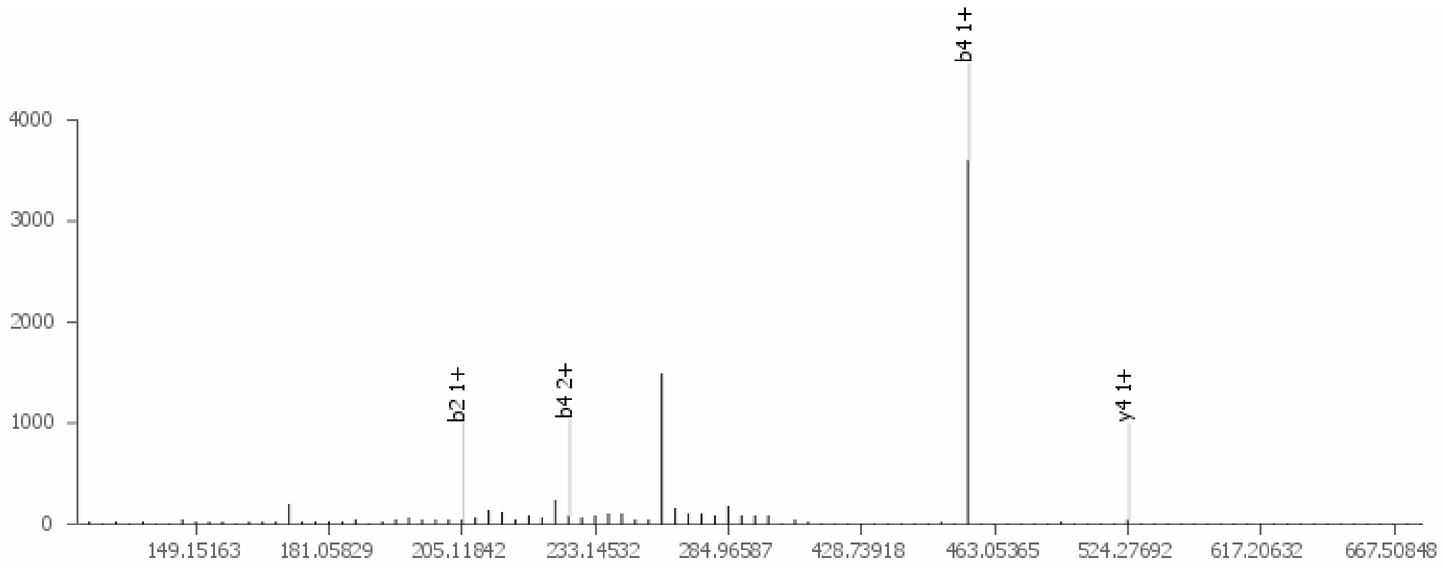

AT1G77180.1 - LSAAQP(pS)NIAR - 604.296681 - Charge:2

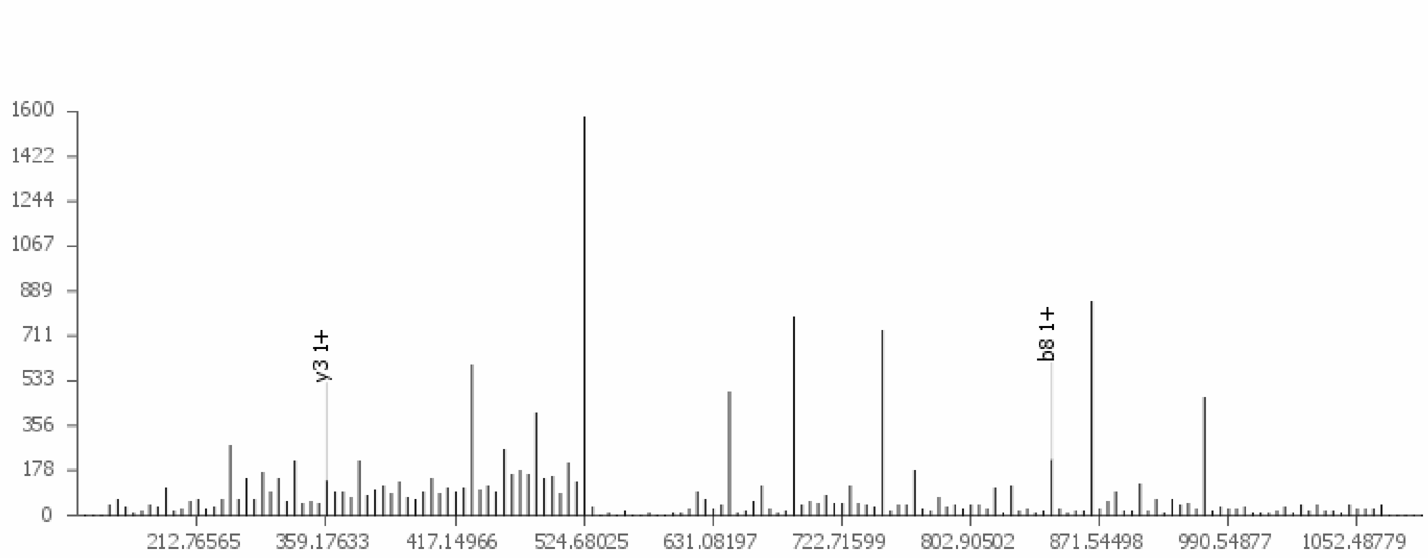

AT1G27000.1 - EIN(pS)QVISAR - 598.78599 - Charge:2

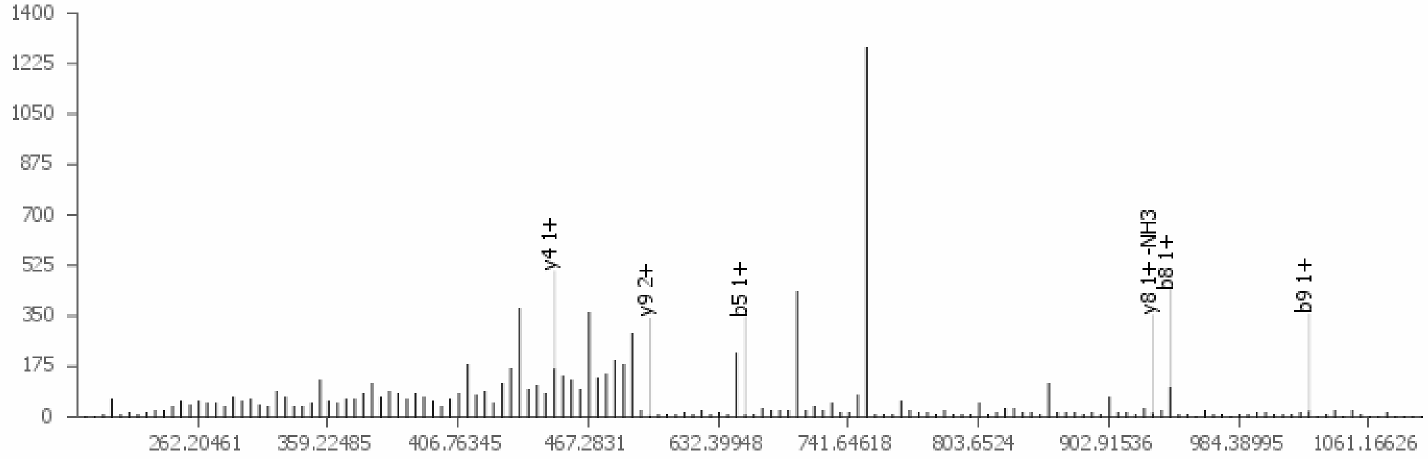

AT5G21030.1 - SSLLPM(pT)RR - 570.786861 - Charge:2

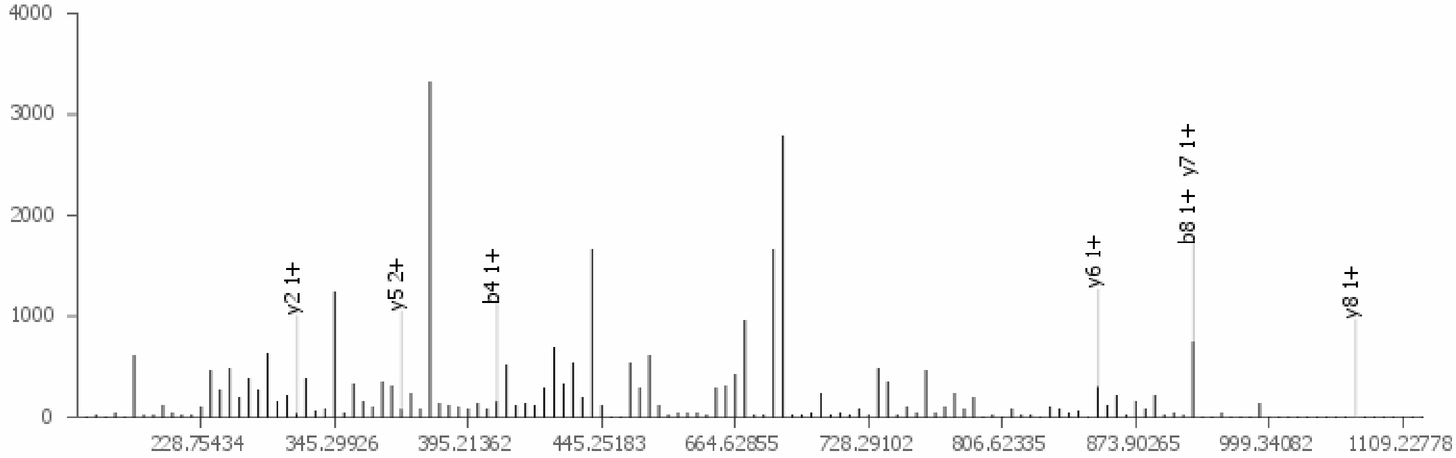

AT5G48830.1 - (pS)VWDVVKVK - 570.292475 - Charge:2

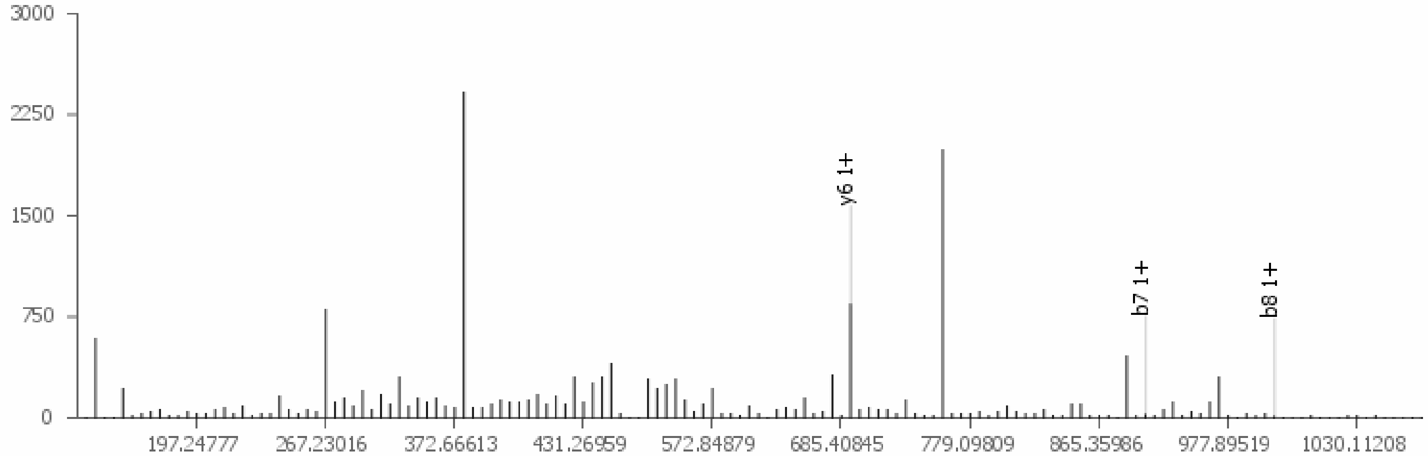

AT2G33490.1 - VSSR(pS)MVALK - 579.294188 - Charge:2

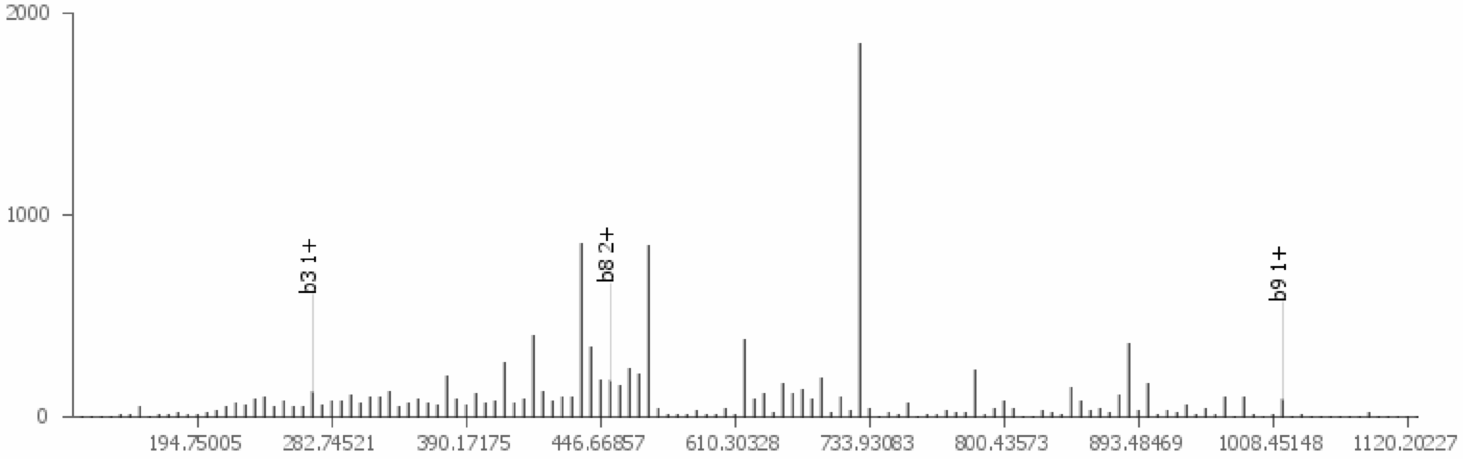

AT5G58840.1 - L(pT)ESERER - 550.242183 - Charge:2

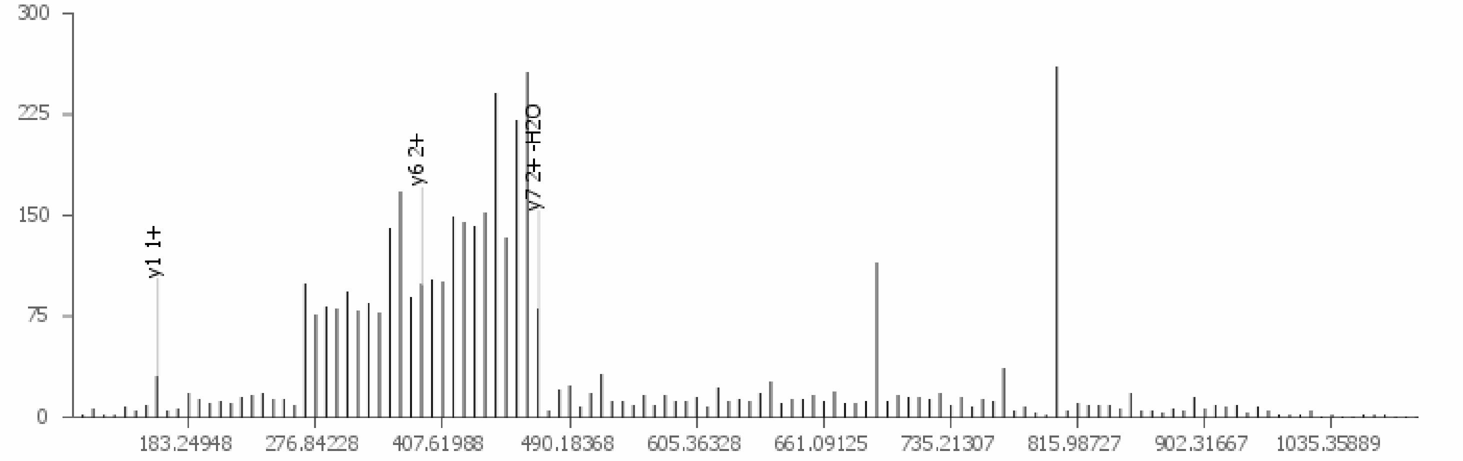

AT2G19160.1 - (pY)(oxM)PEDI(pT)LELIK - 547.565862 - Charge:3

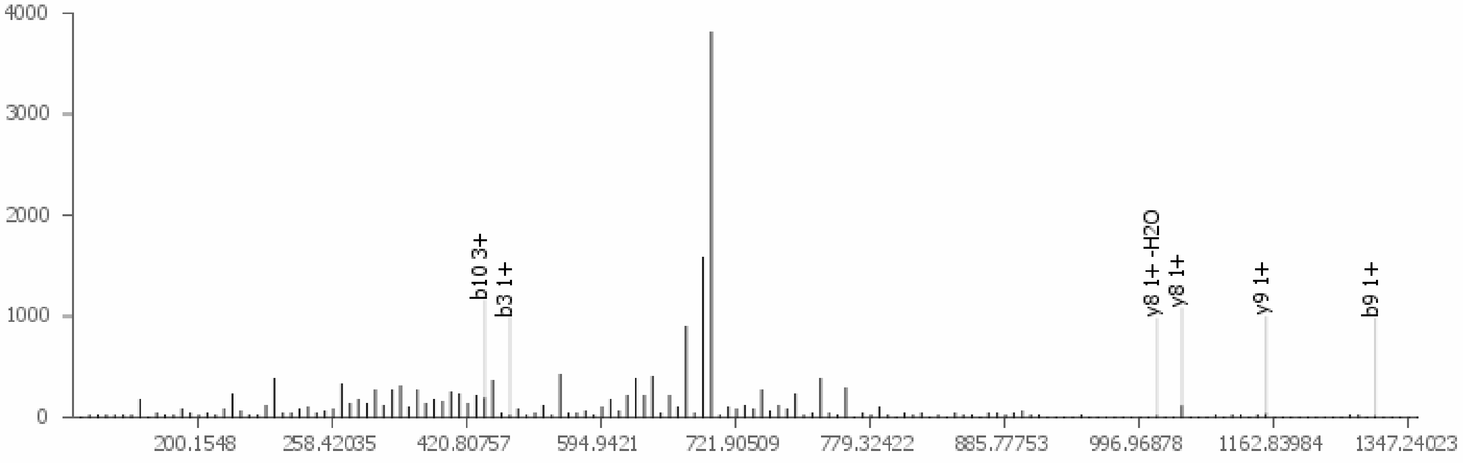

AT5G51500.1 - (pT)VQAAIDVAGR - 590.795287 - Charge:2

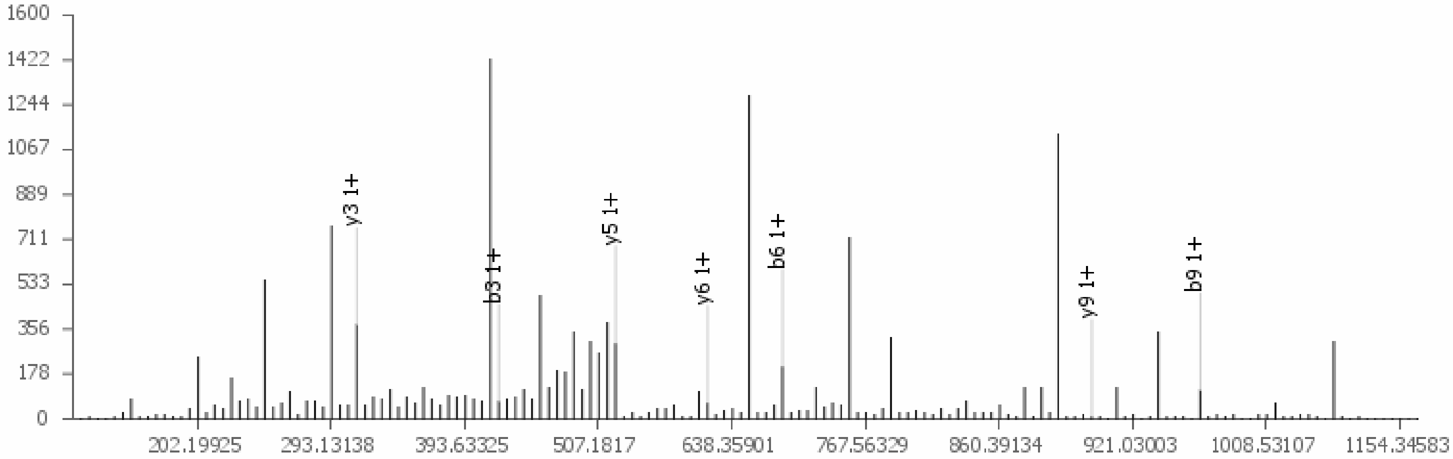

AT4G32230.1 - IR(t)(t)ISPLMGL(pS)HELVR - 1042.010699 - Charge:2

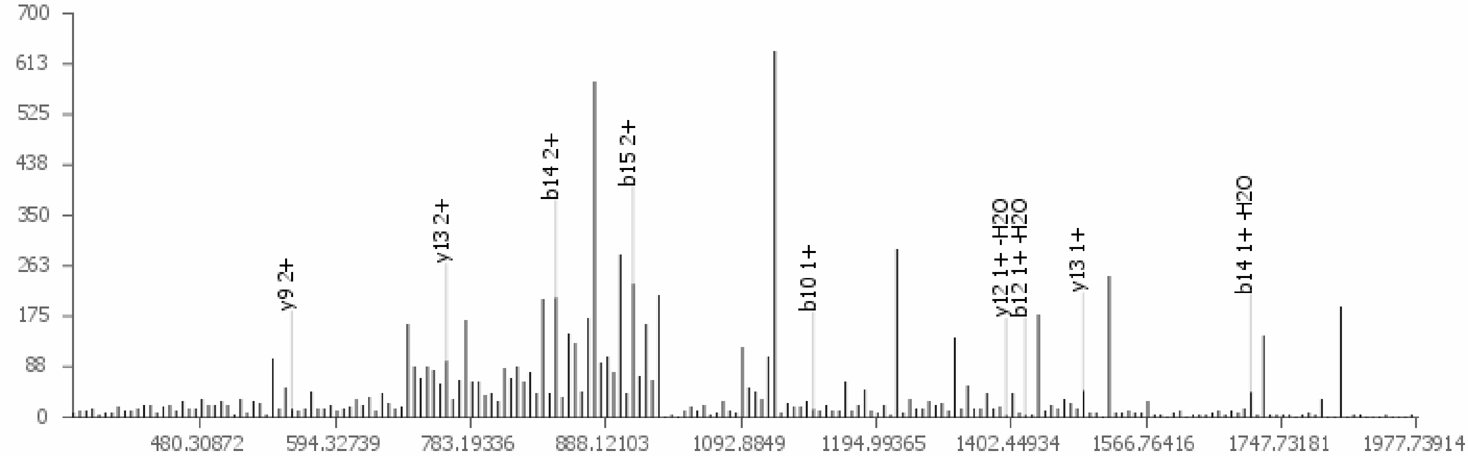

AT5G12090.1 - QASKI(pT)MGIR - 592.797943 - Charge:2

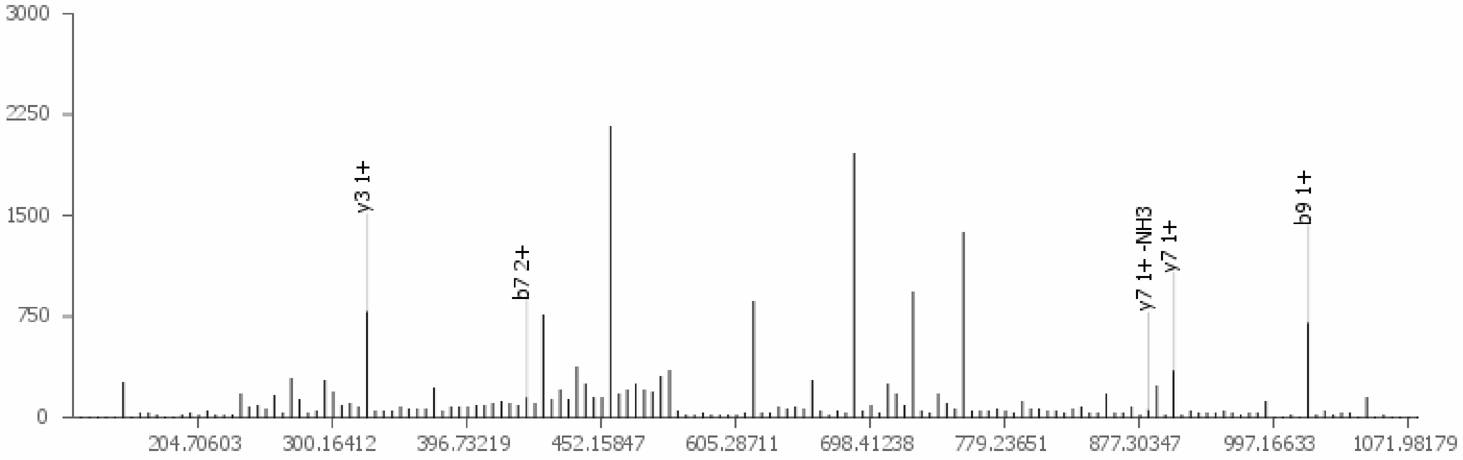

AT2G43470.1 - S(oxM)LL(s)(s)FMLPYKPK - 869.428453 - Charge:2

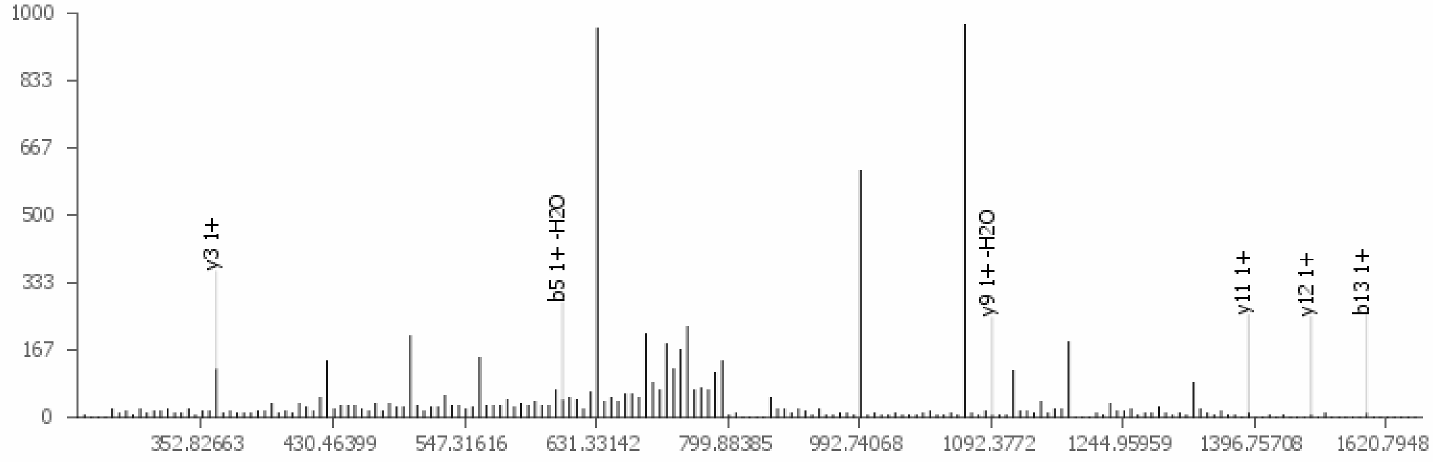

AT2G39370.1 - L(oxM)KQ(s)(s)LGTK - 594.788287 - Charge:2

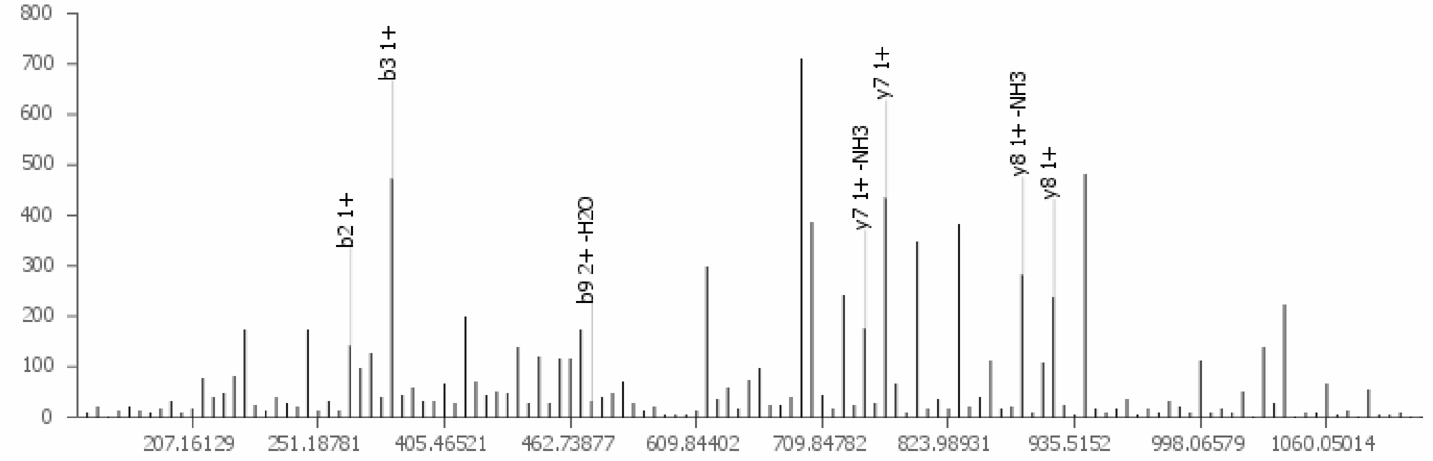

AT1G50480.1 - KGAP(pS)GFVLPIR - 661.360954 - Charge:2

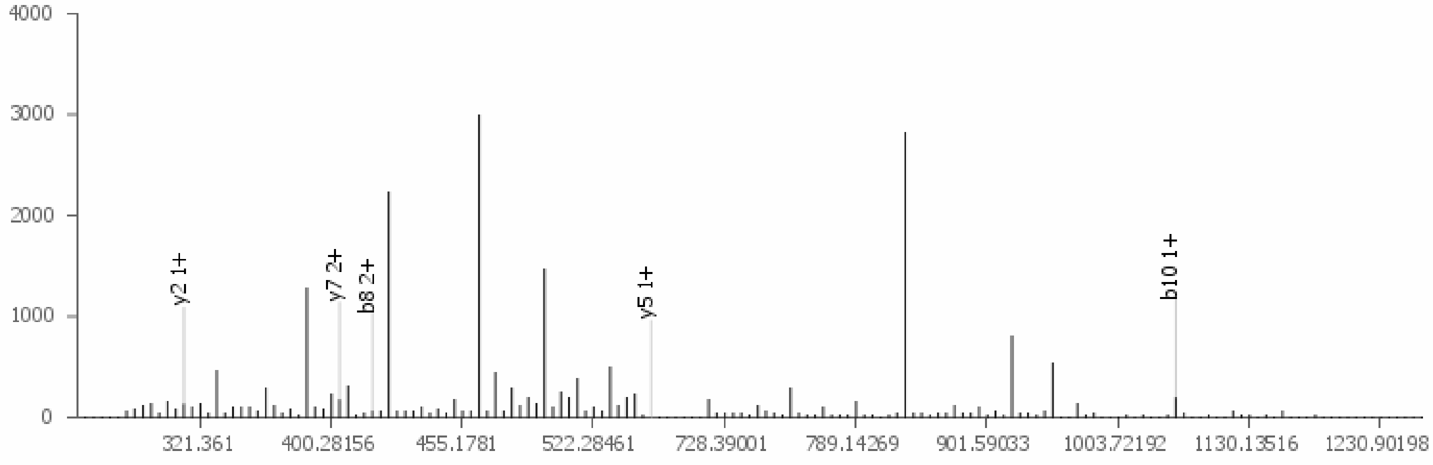

AT5G17980.1 - AYRN(pT)YPLL(oxM)LVNGGVK - 1002.9975 - Charge:2

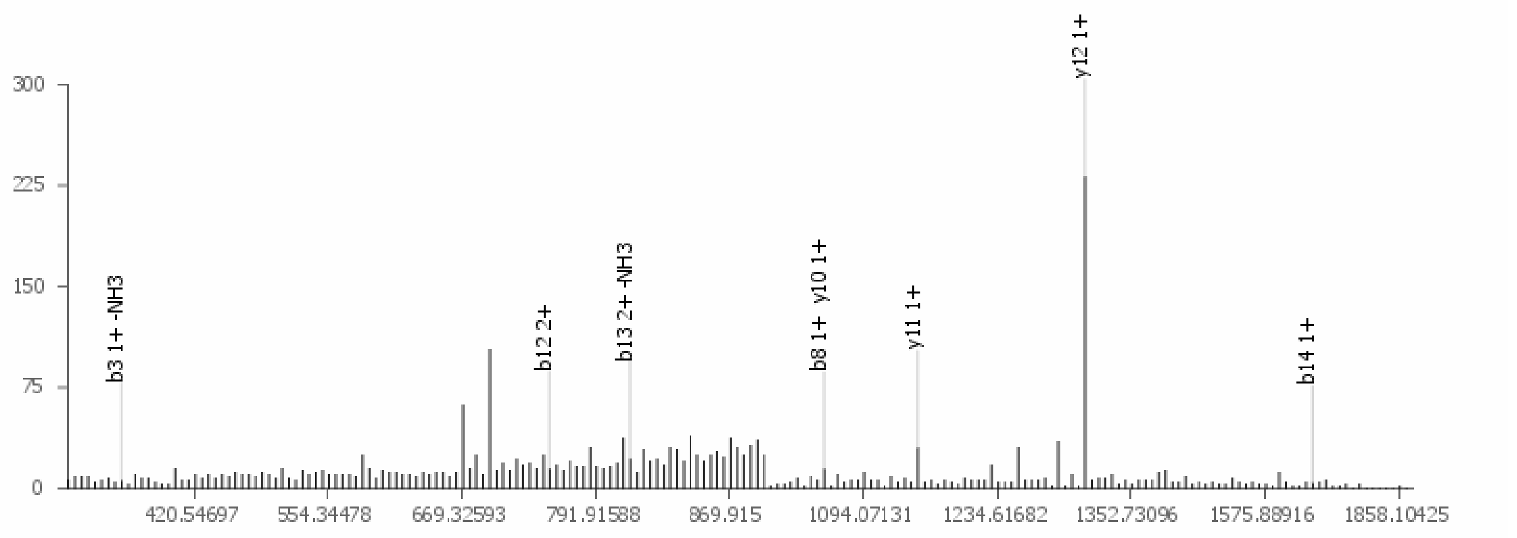

AT1G27070.1 - HRLQQLQSELSSVLH(pS)LR - 737.717499 - Charge:3

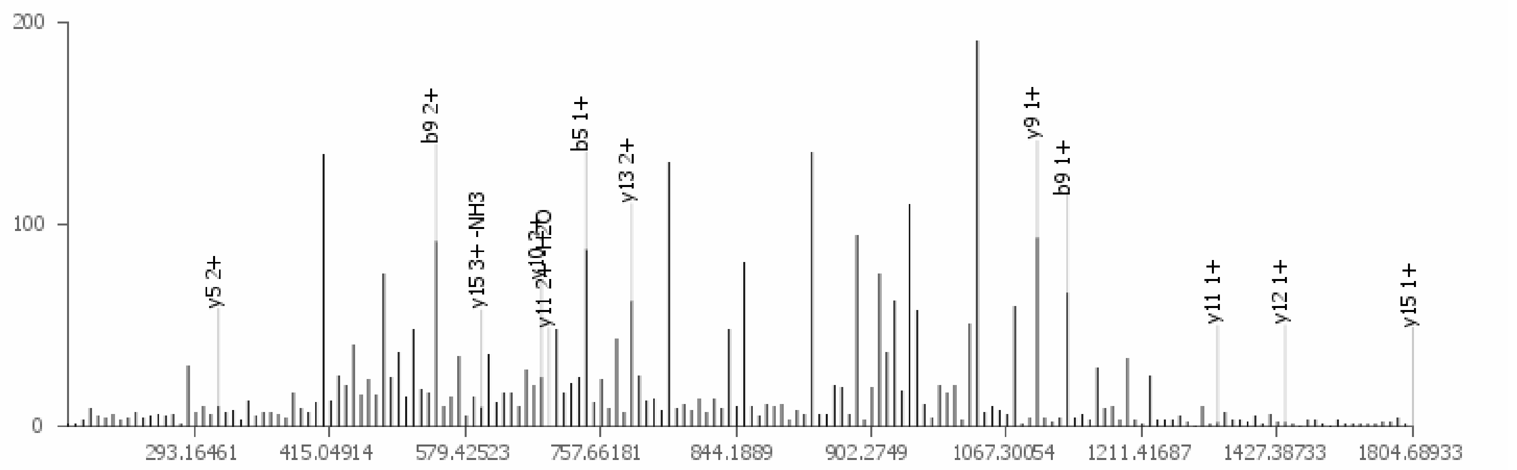

AT3G16730.1 - NSAHRGS(pS)VR - 575.758171 - Charge:2

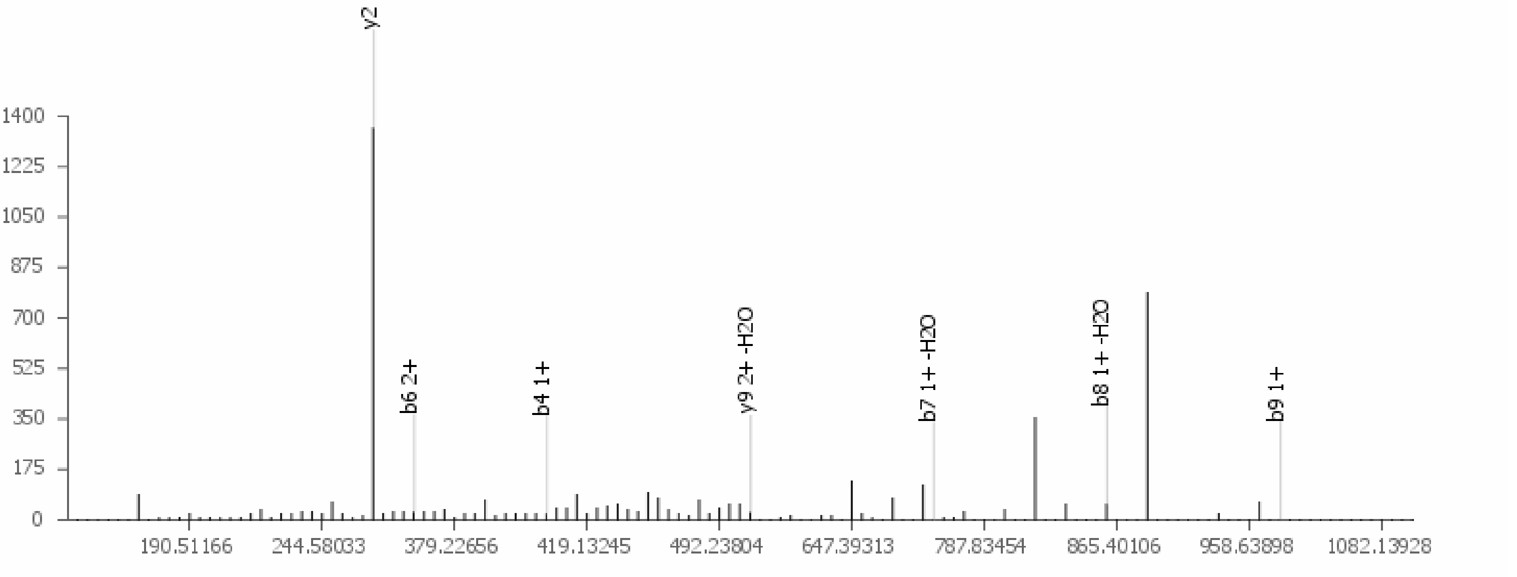

AT4G39850.1 - LIGL(pS)VLQSGASSIIAPSLRHLTQR - 899.840033 - Charge:3

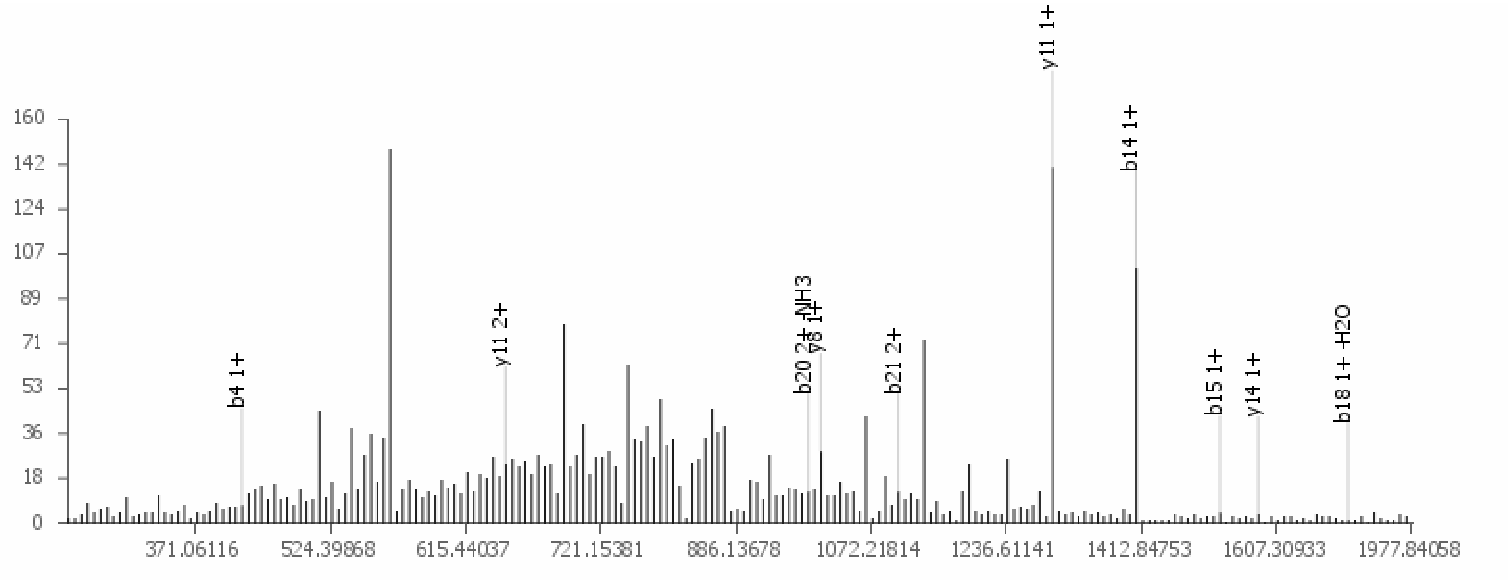

AT4G33080.1 - I(pS)VDDFELL(pT)IIGR - 875.906756 - Charge:2

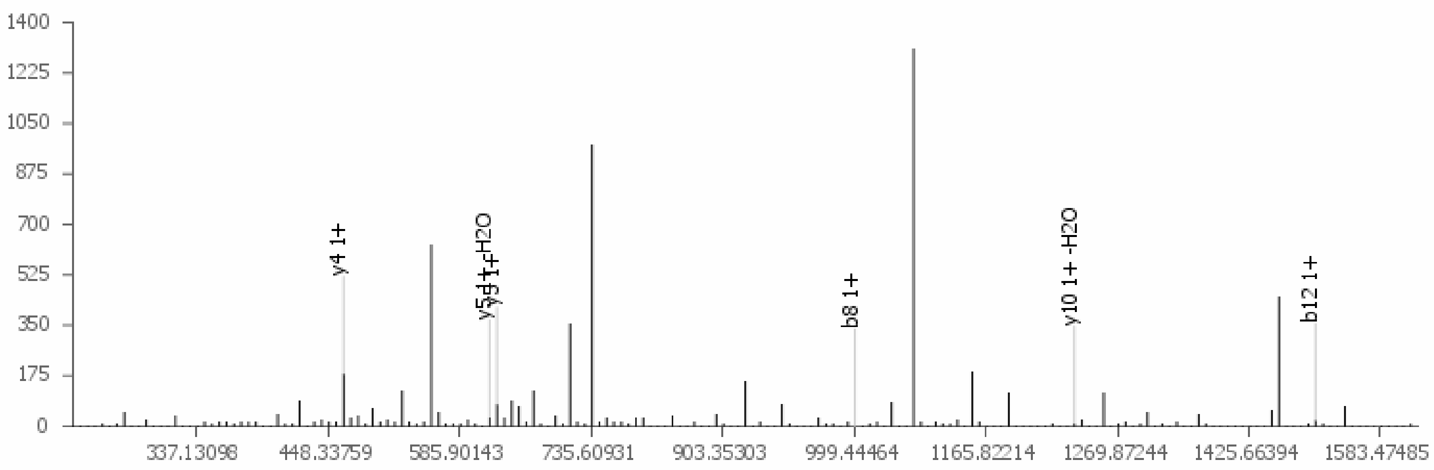

AT2G45750.1 - LNTLGVI(pY)ER - 629.321717 - Charge:2

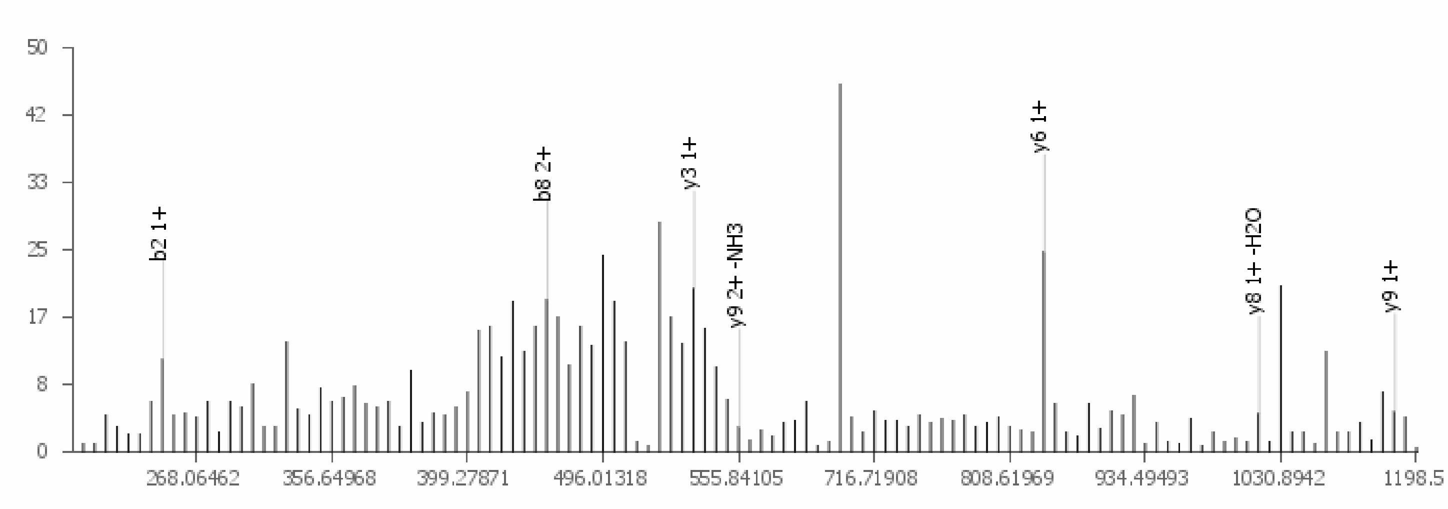

AT2G36070.1 - QGQGVWTEAE(pS)VAKK - 849.393087 - Charge:2

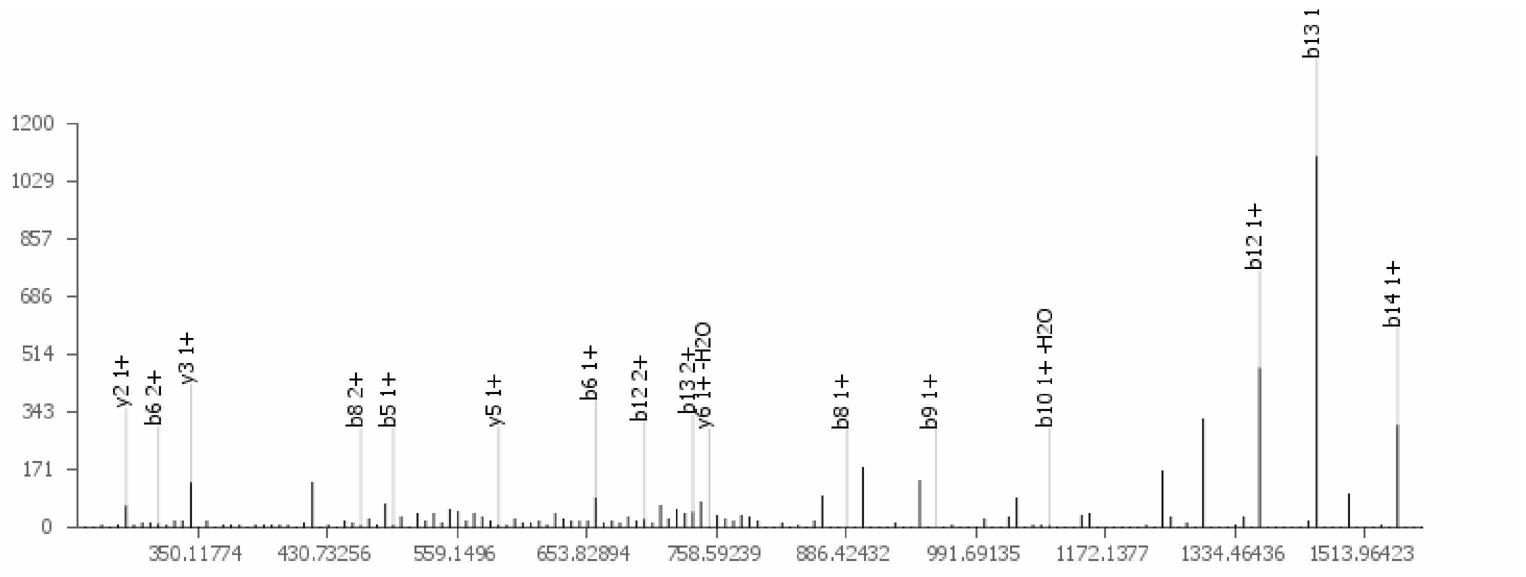

AT4G10120.1 - AADL(t)(s)GKGK - 514.240314 - Charge:2

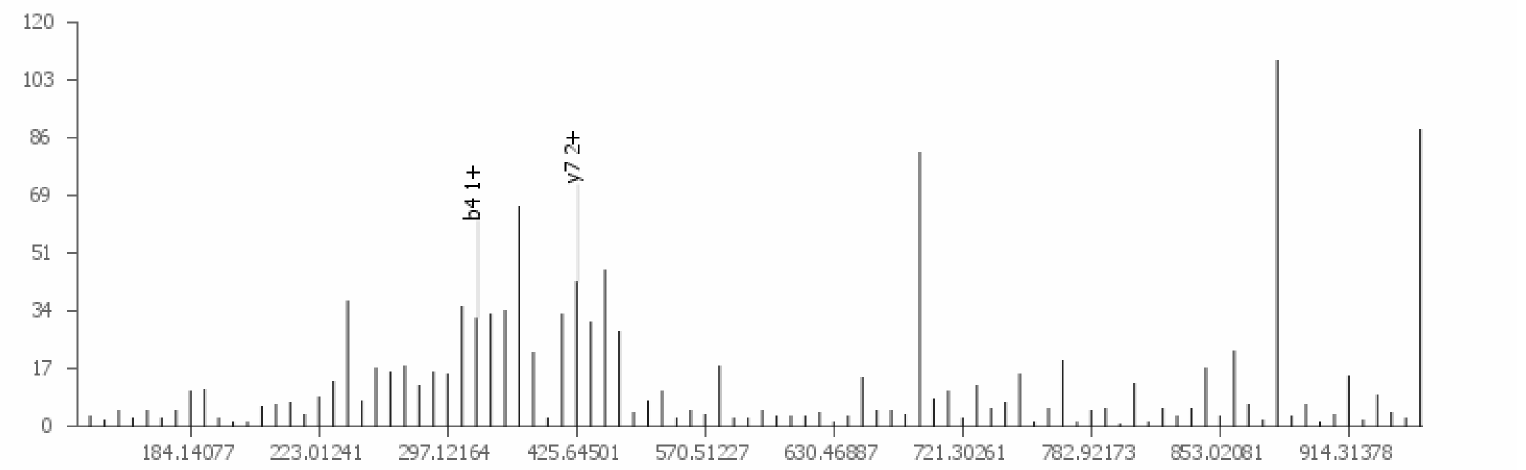

AT5G08370.1 - Q(pT)ADAMVSSGLSAIGYK - 889.912665 - Charge:2

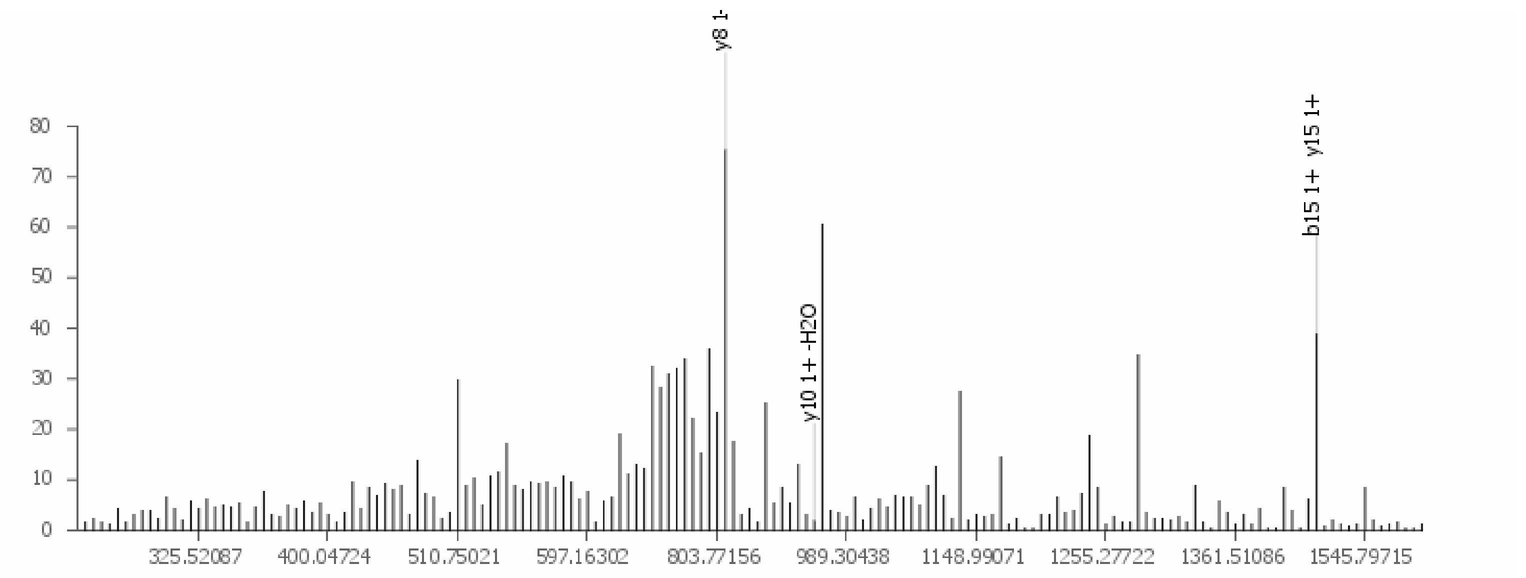

AT1G15280.1 - EK(oxM)PHLD(pS)R - 604.758049 - Charge:2

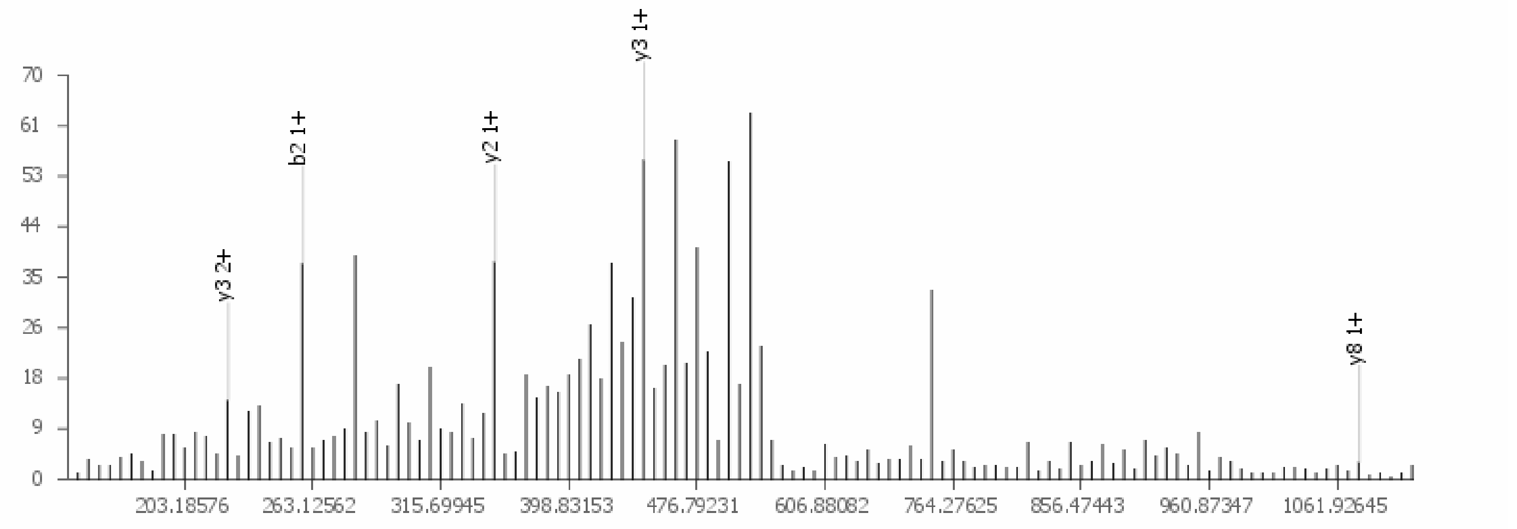

AT4G24900.1 - SSLF(t)(s)LER - 529.249817 - Charge:2

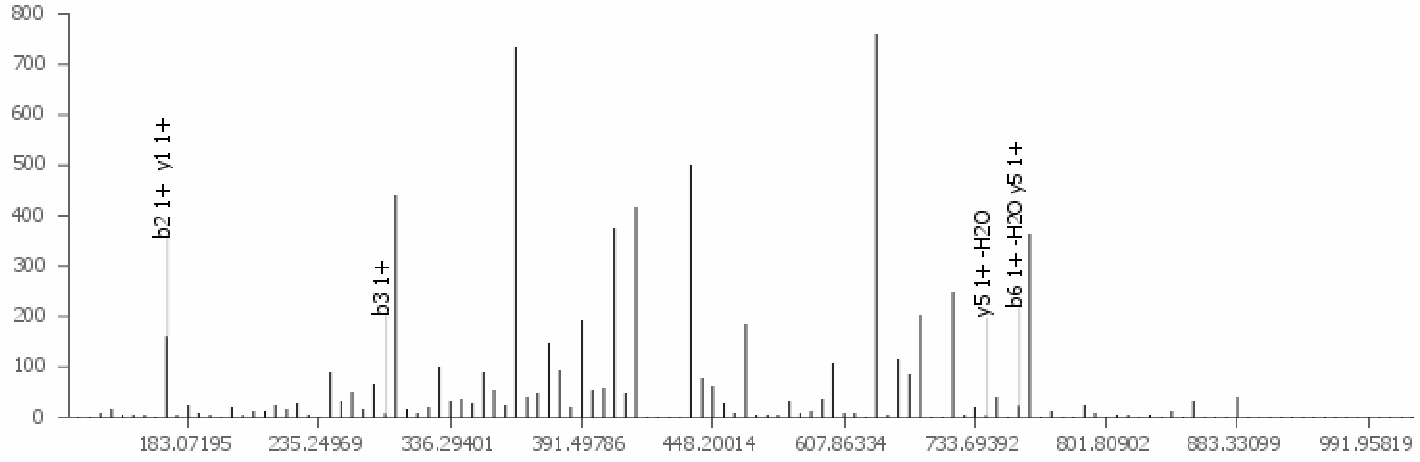

AT5G64380.1 - (oxM)Q(pS)LTLTR - 523.244314 - Charge:2

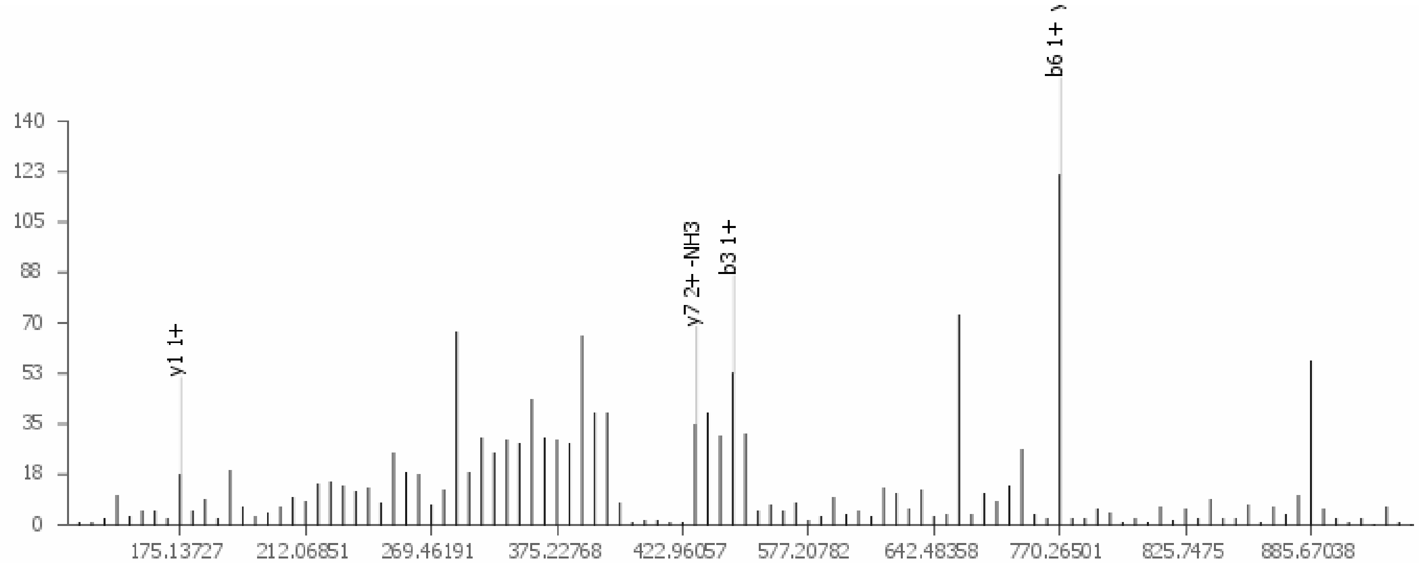

AT4G10100.1 - MPSG(s)(t)TQK - 508.709828 - Charge:2

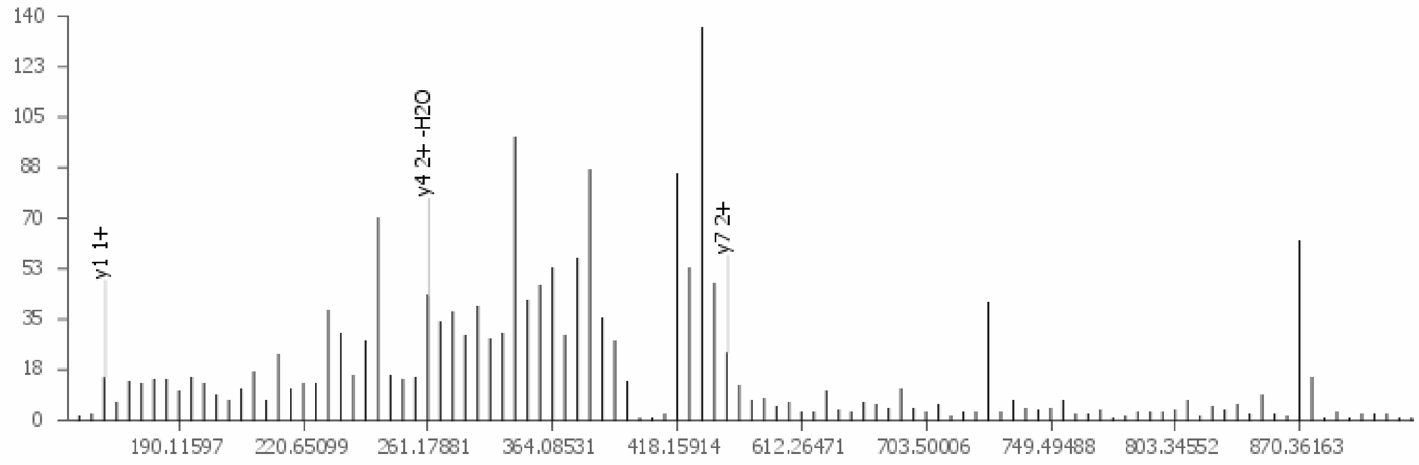

AT4G14040.1 - I(pY)AIDTKTDPK - 672.822496 - Charge:2

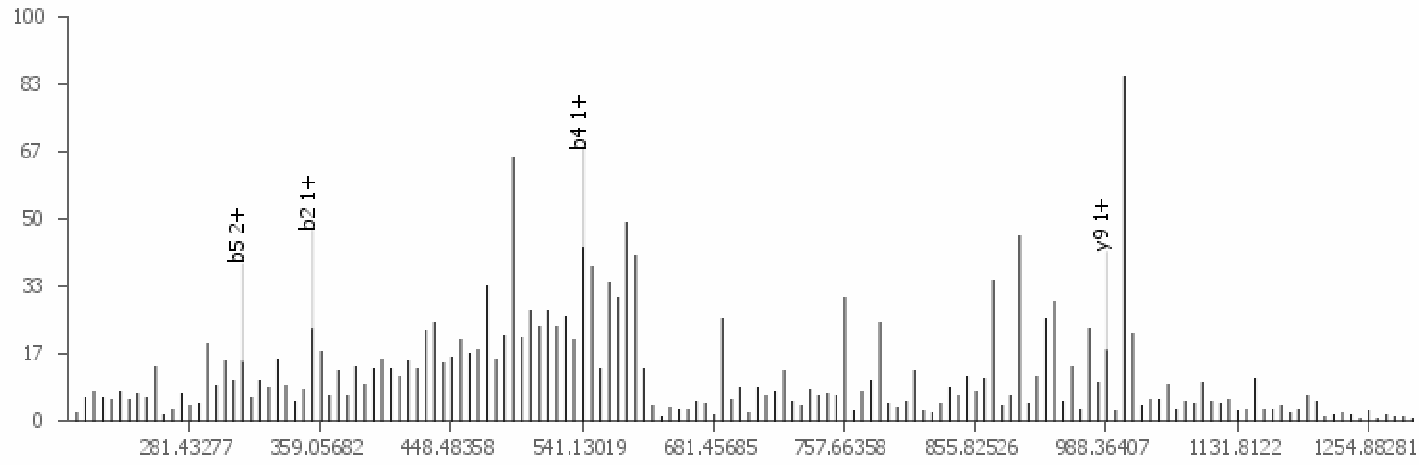

AT2G18960.1 - GLDIDTAGHHY(pT)V - 739.81037 - Charge:2

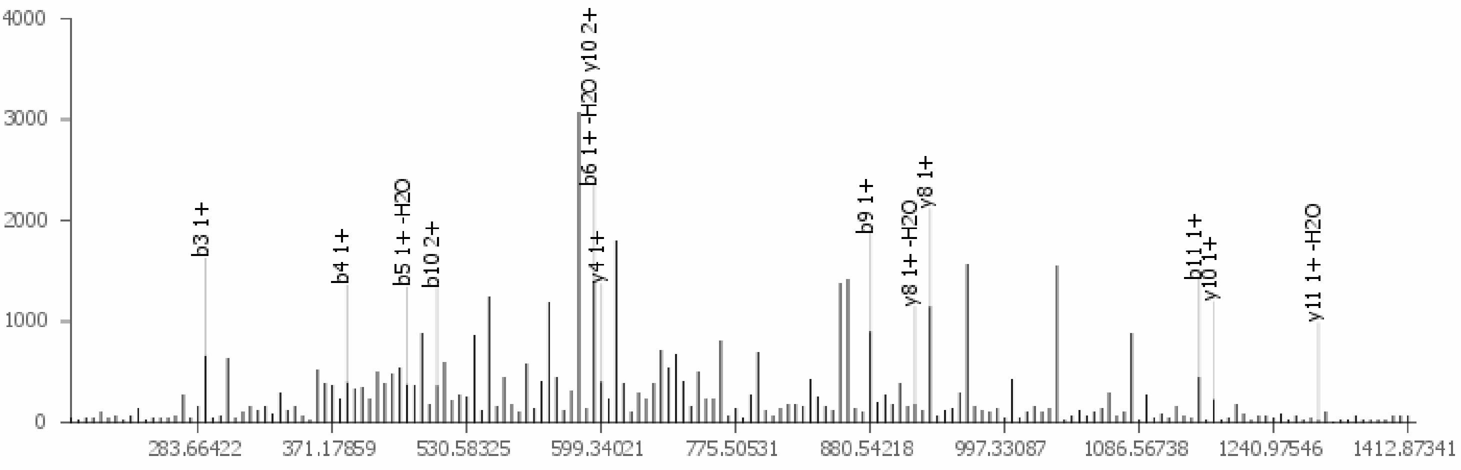

AT4G15180.1 - H(pS)FIEDVVLR - 647.80093 - Charge:2

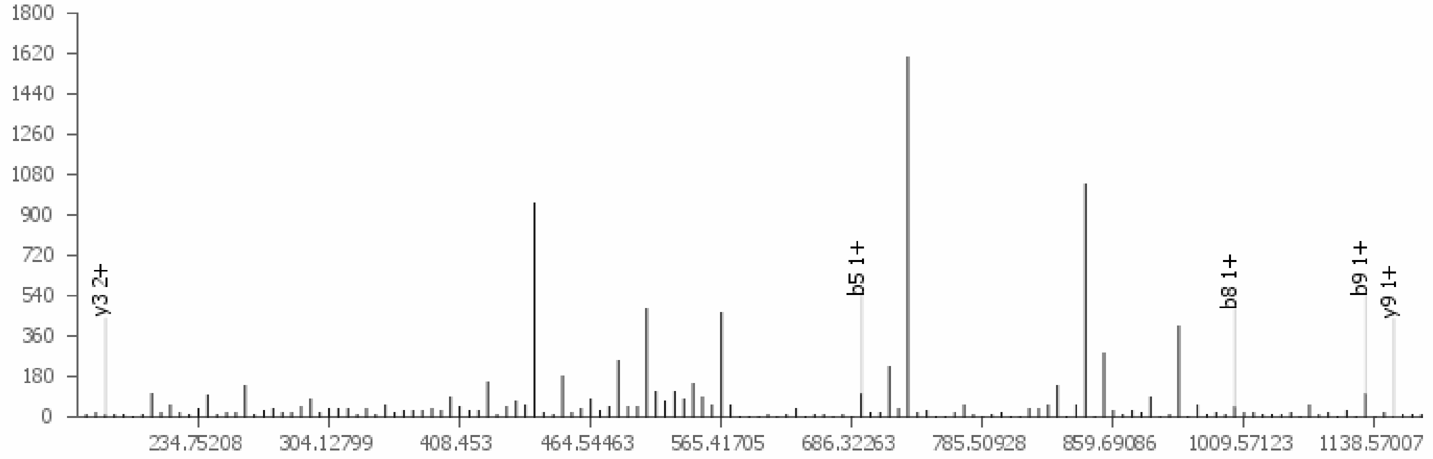

AT4G35600.1 - VG(pS)GMIVAIK - 527.76395 - Charge:2

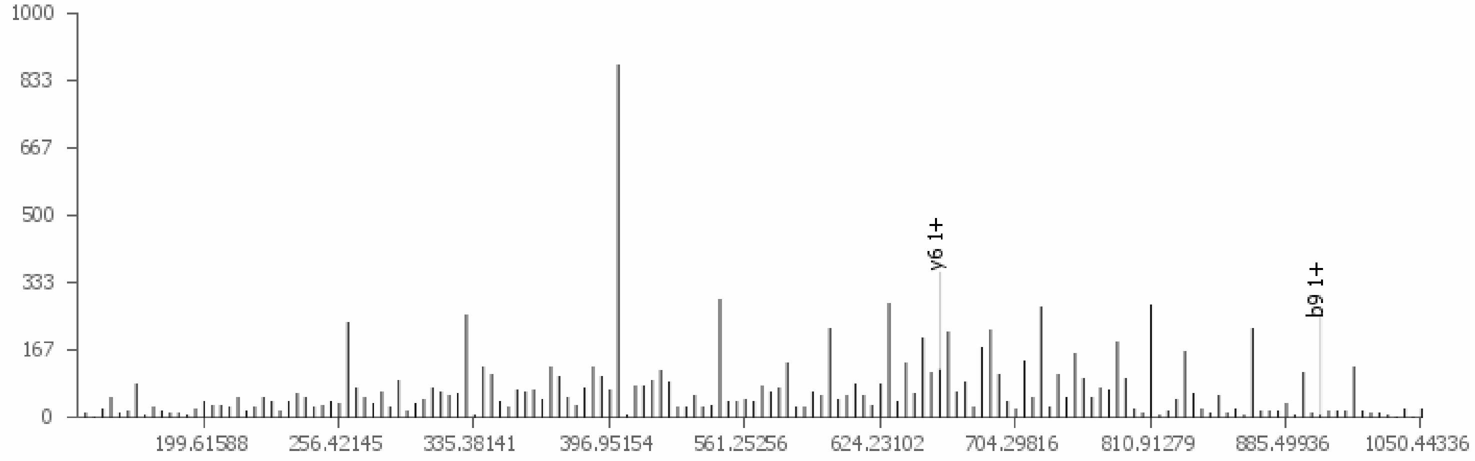

AT1G30360.1 - VAAASAAQ(pS)LHCQMVDKWTVTEAPEPR - 992.78382 - Charge:3

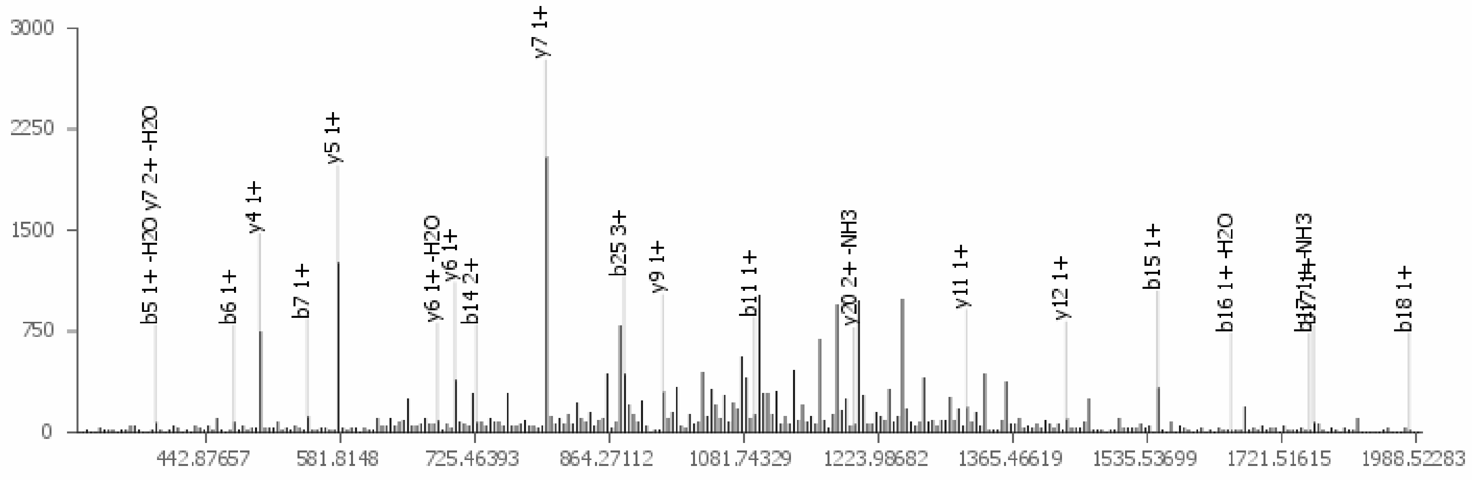

AT1G51800.1 - (pS)HHGFEPPVIAK - 699.82377 - Charge:2

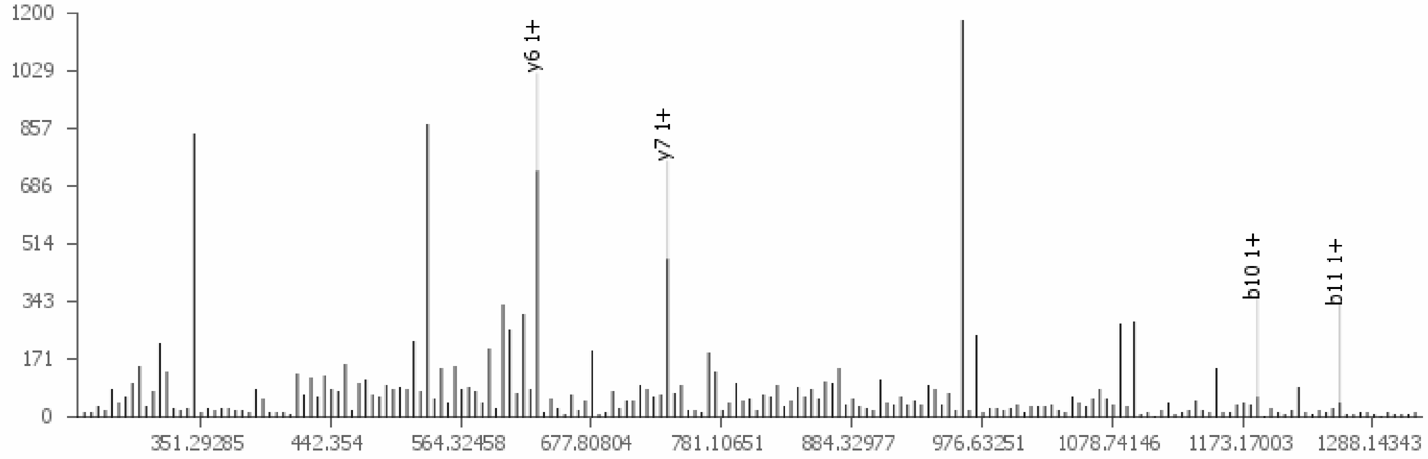

AT4G13510.1 - HGGFAYMYFDDDE(pS)HK - 999.86388 - Charge:2

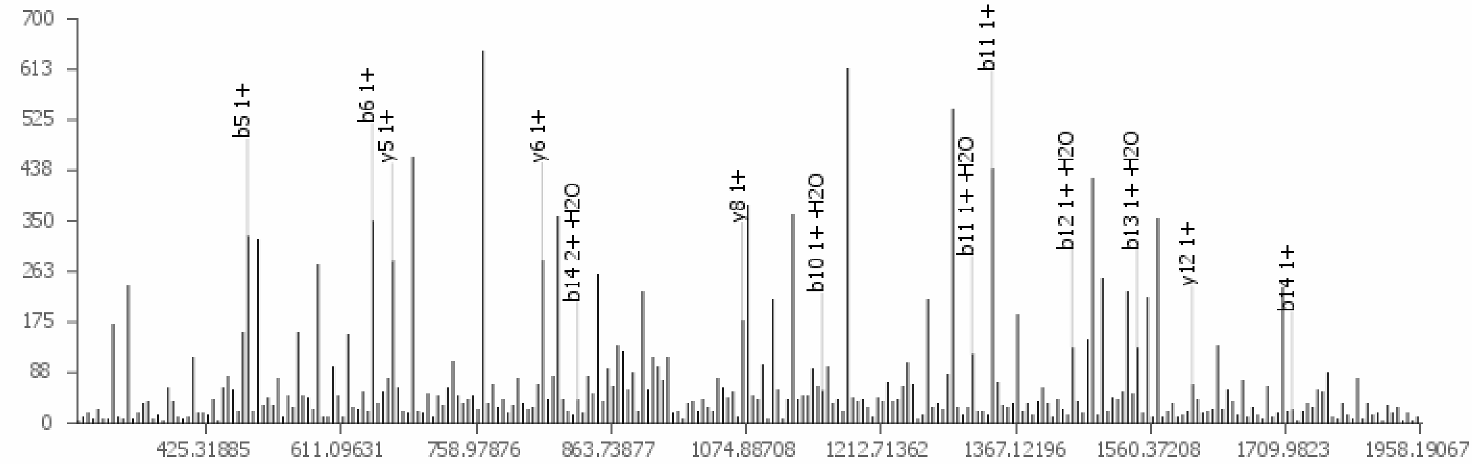

AT4G13510.1 - (pS)P(s)P(s)GANT(t)PTPV - 736.77876 - Charge:2

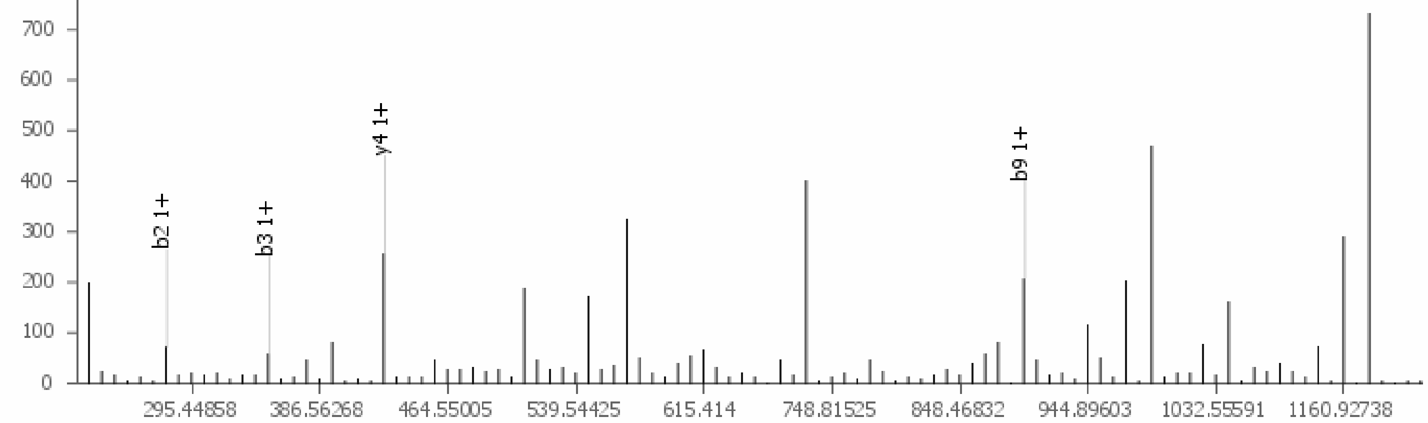

AT1G24220.1 - LG(pS)FR - 330.14069 - Charge:2

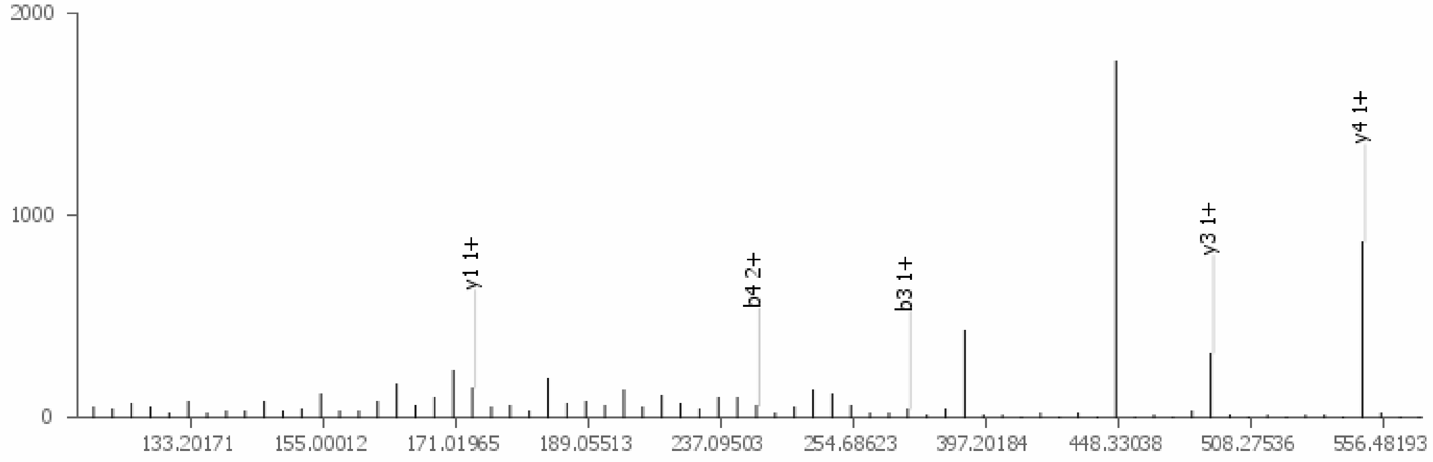

AT2G47000.1 - MSSIE(pS)FKQS(pS)LR - 830.33683 - Charge:2

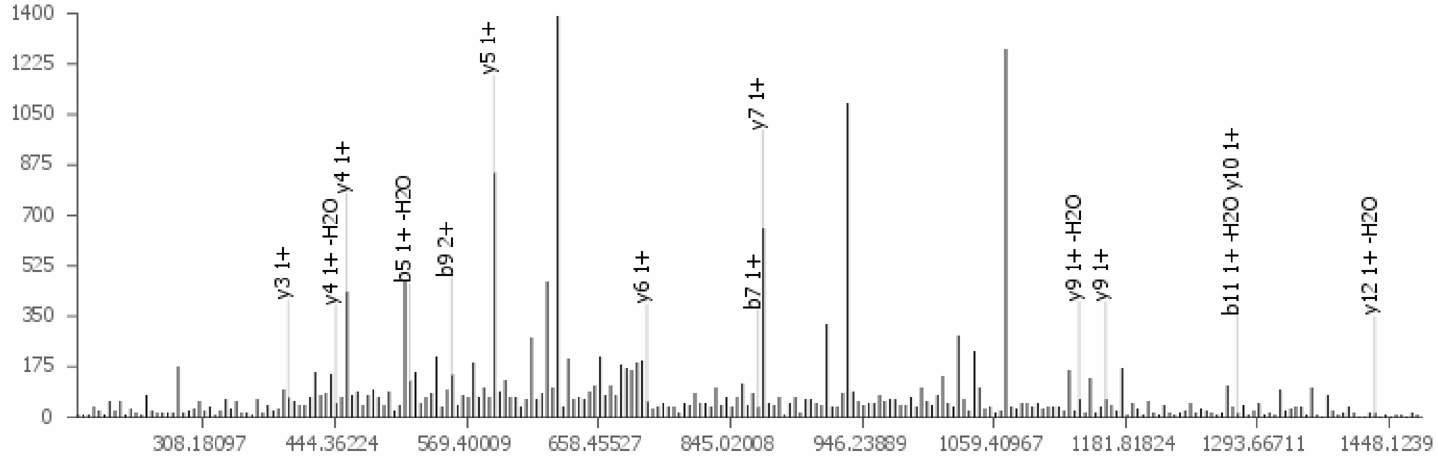

AT1G05150.1 - DNDVPVS(pY)SGSGGPTK - 830.34077 - Charge:2

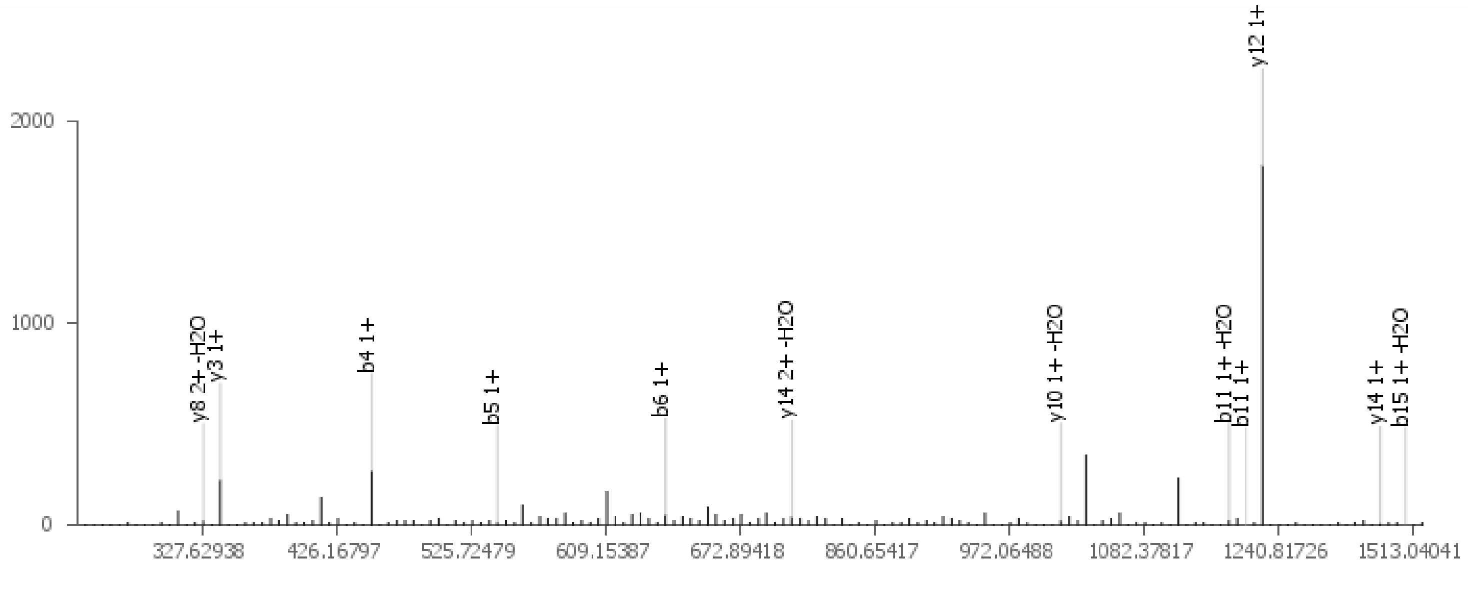

AT4G23640.1 - SI(pS)EANIAGSSR - 636.27466 - Charge:2

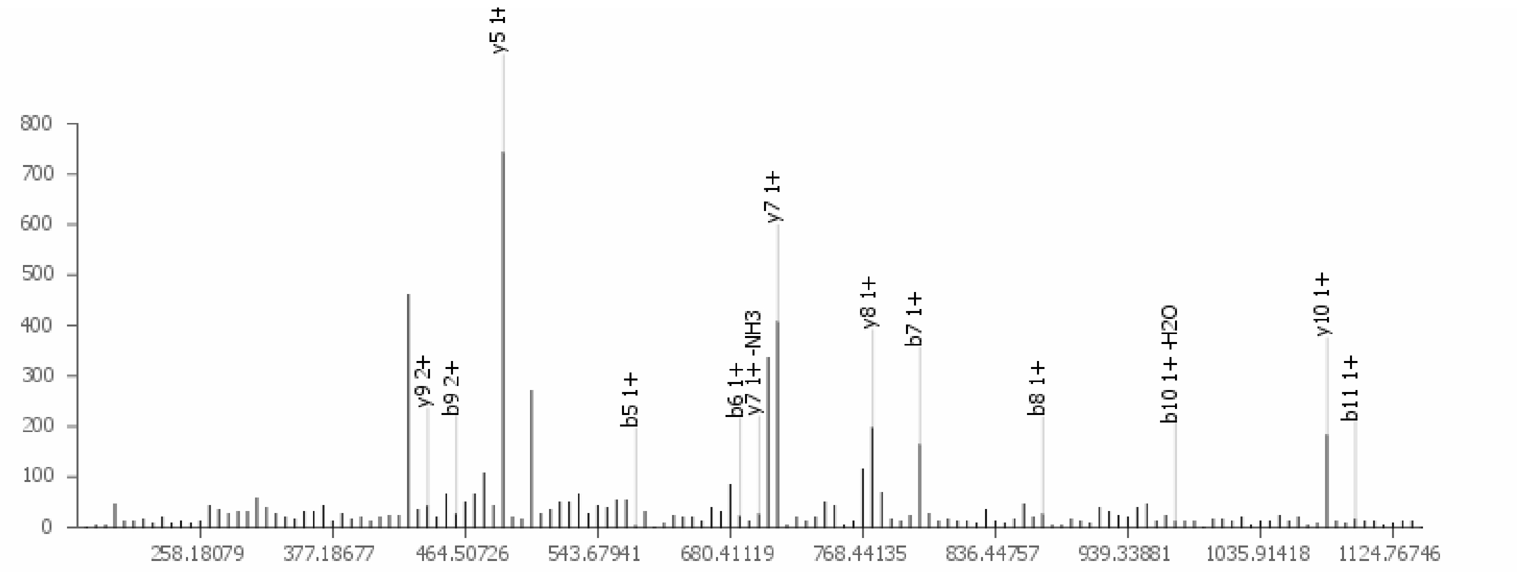

AT3G08510.1 - EVP(pS)FIQR - 528.24097 - Charge:2

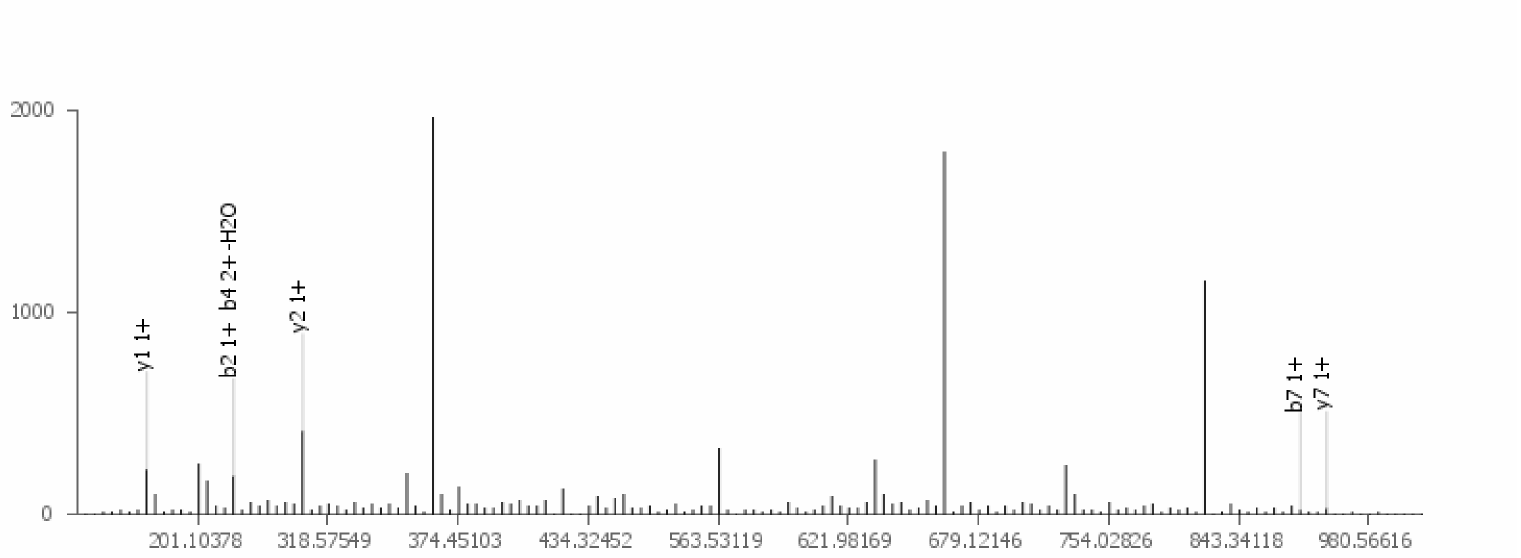

AT2G45820.1 - ALAVVEKPIEEH(pT)PK - 580.96056 - Charge:3

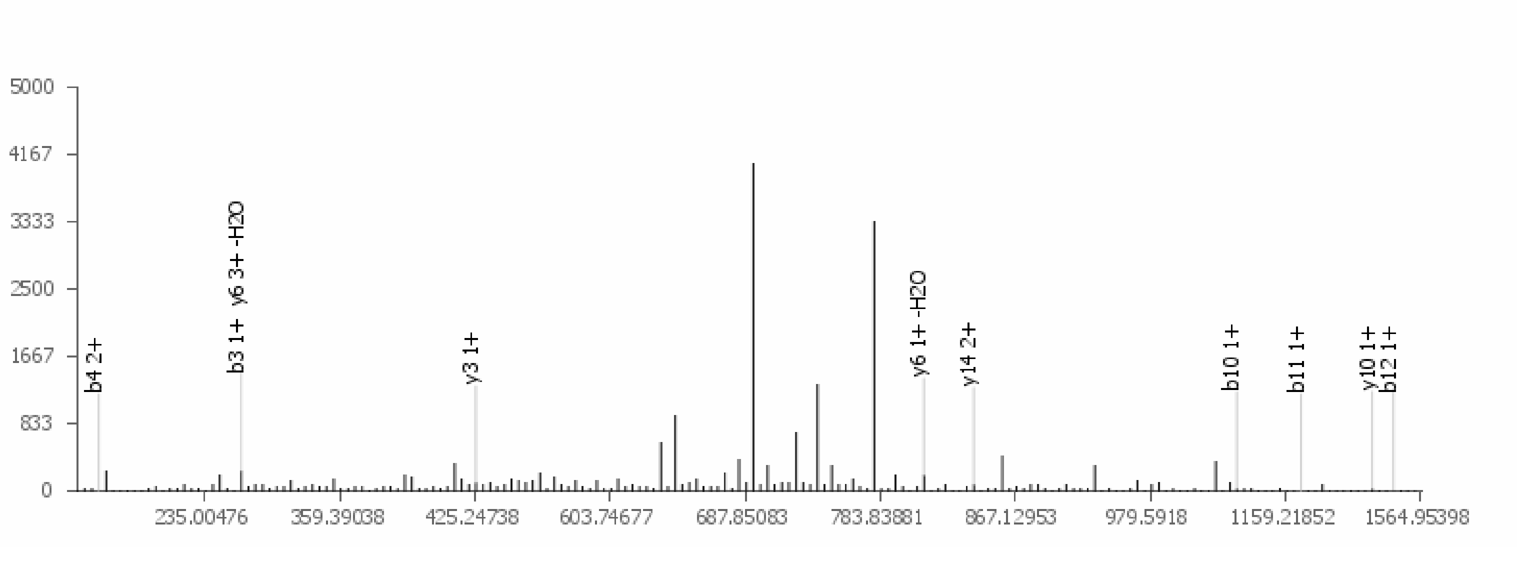

AT5G01490.1 - TV(pS)ASSLIR - 507.24546 - Charge:2

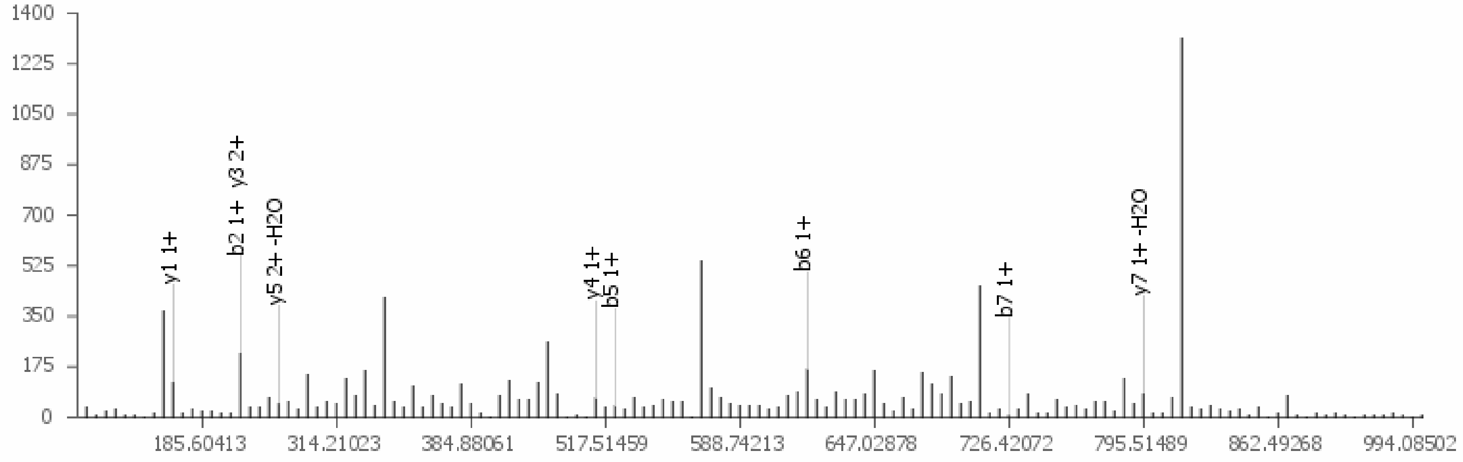

AT1G15400.1 - V(pS)PAVDPP(pS)PR - 641.25749 - Charge:2

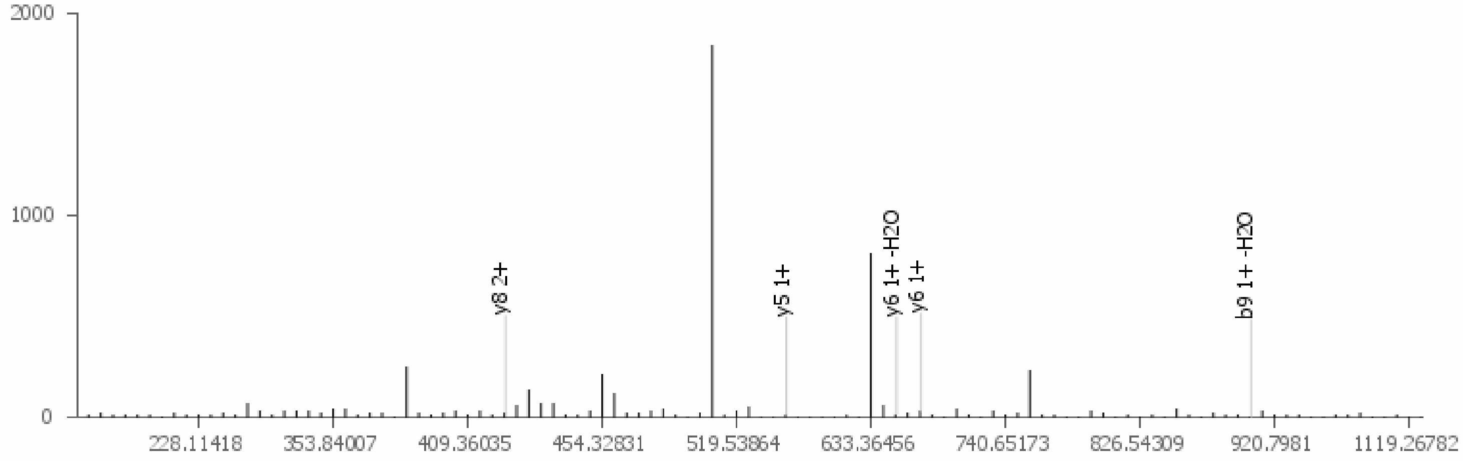

AT3G11820.1 - AS(pS)FIR - 380.66481 - Charge:2

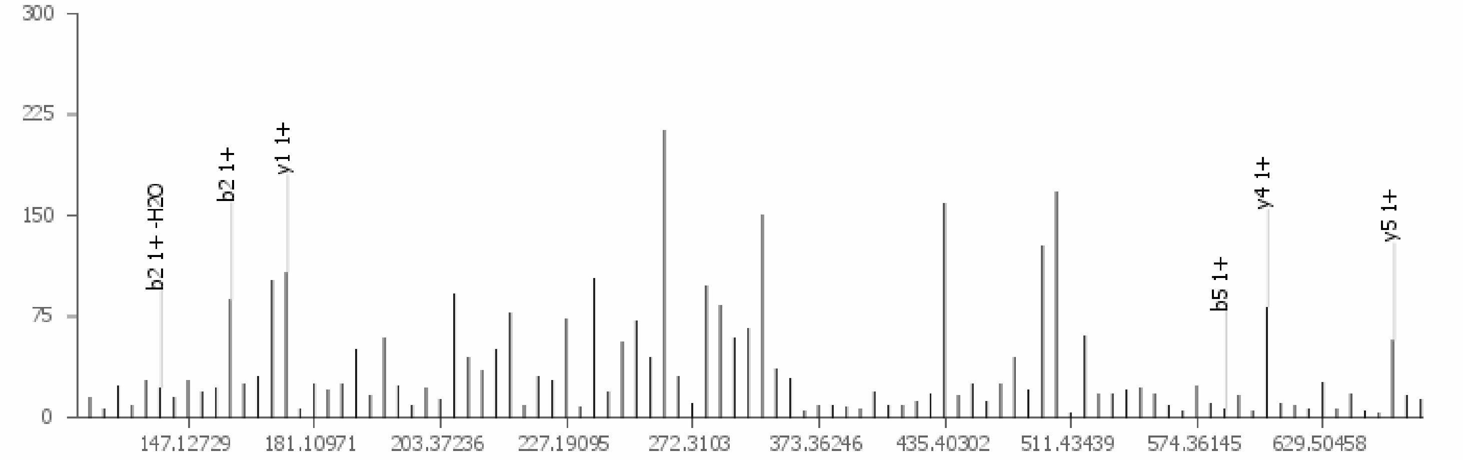

AT2G47000.1 - MSSIE(pS)FKQSSLR - 790.35379 - Charge:2

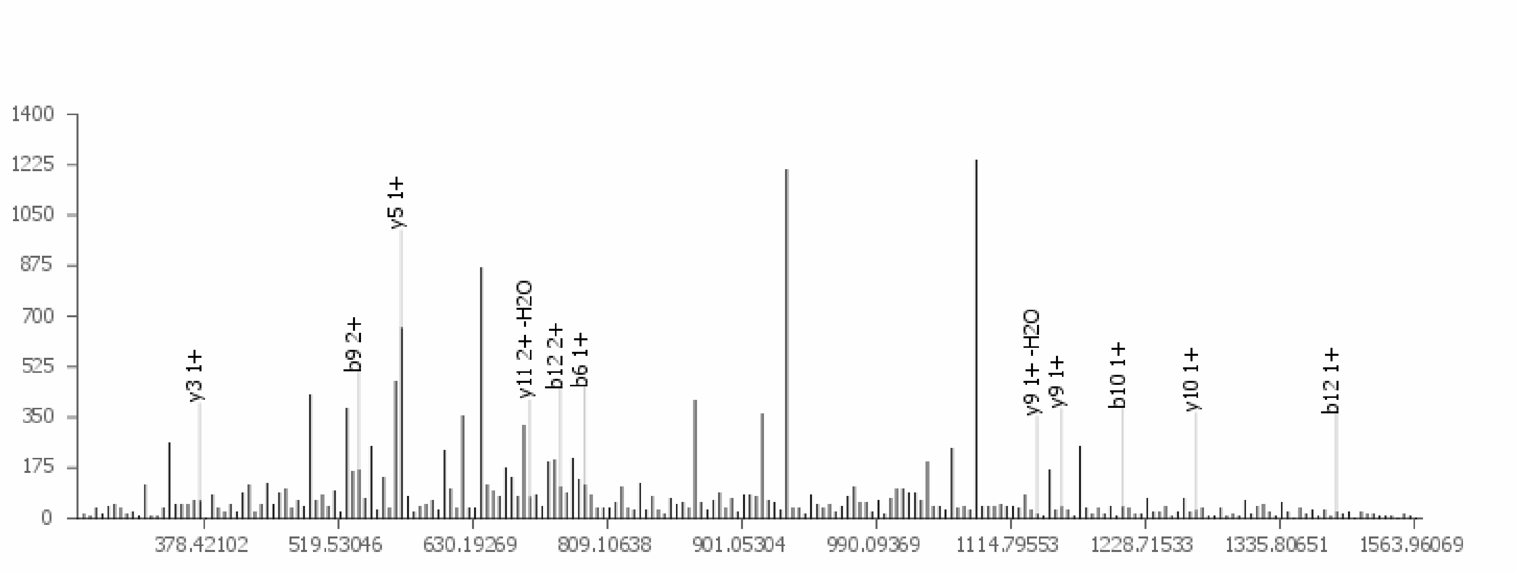

AT3G48740.1 - LGTVS(pS)PEPISVVR - 760.88205 - Charge:2

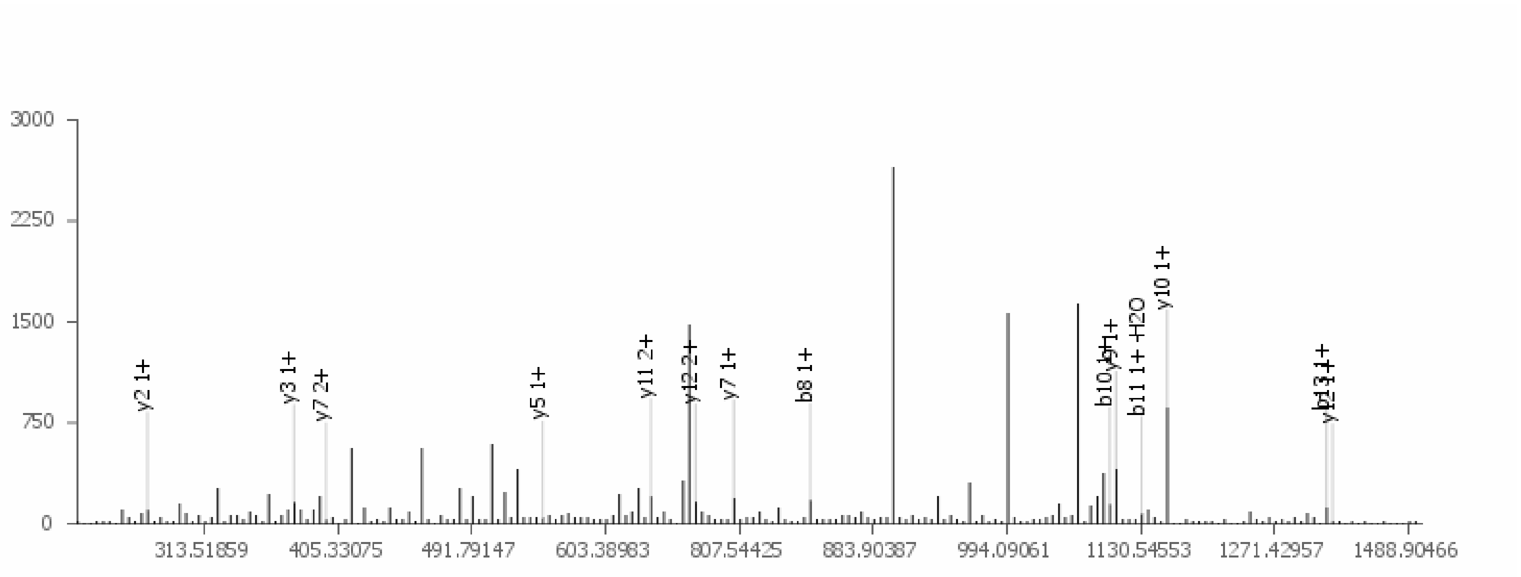

AT3G61260.1 - IALE(s)E(s)PAKVTTPAPADTPAPAPAEIPAPAPAPTPADVTK - 1010.25371 - Cl

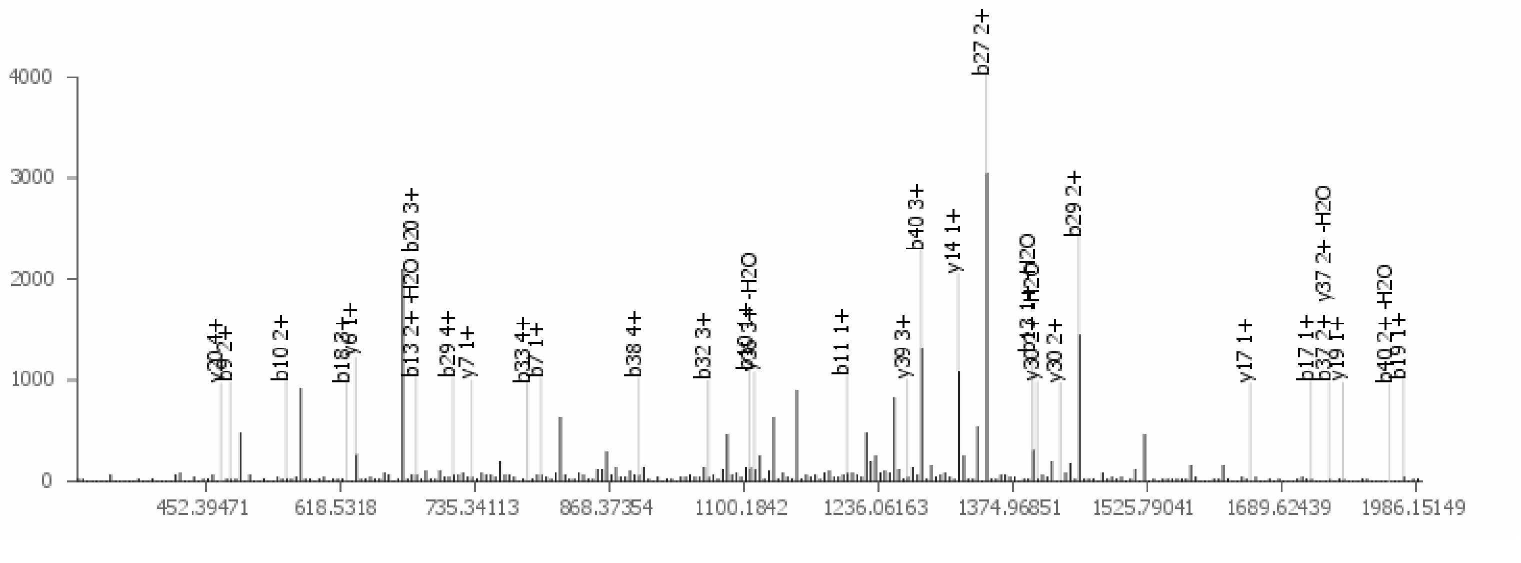

AT4G13510.1 - ISSEDEMAGMDM(pT)R - 826.79485 - Charge:2

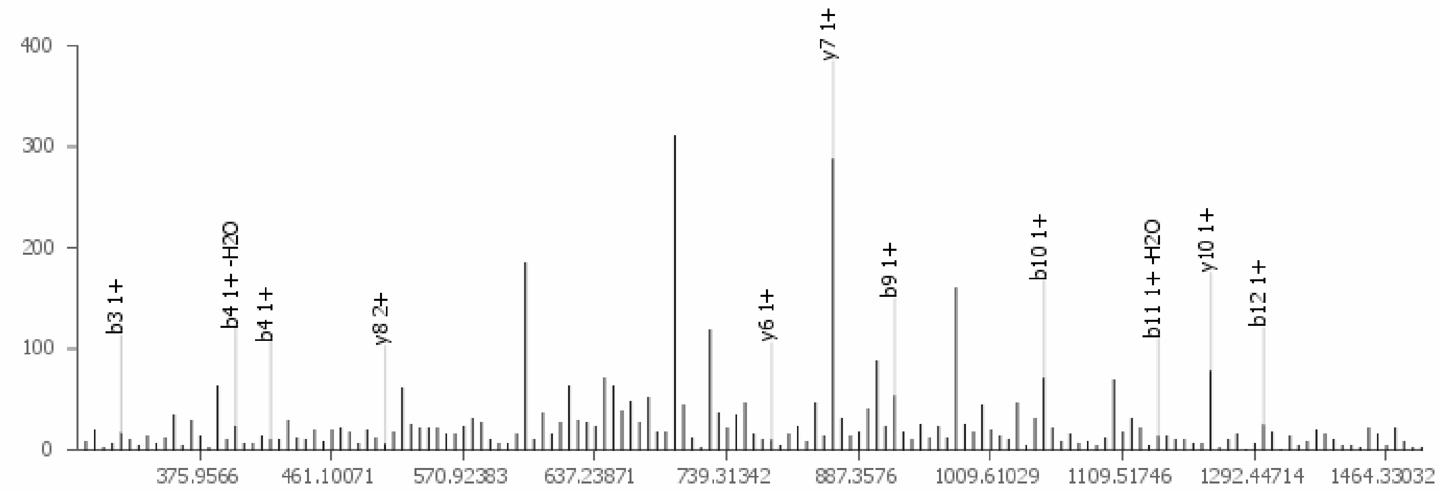

AT1G15400.1 - QG(pS)SGIVFDDR - 630.7562 - Charge:2

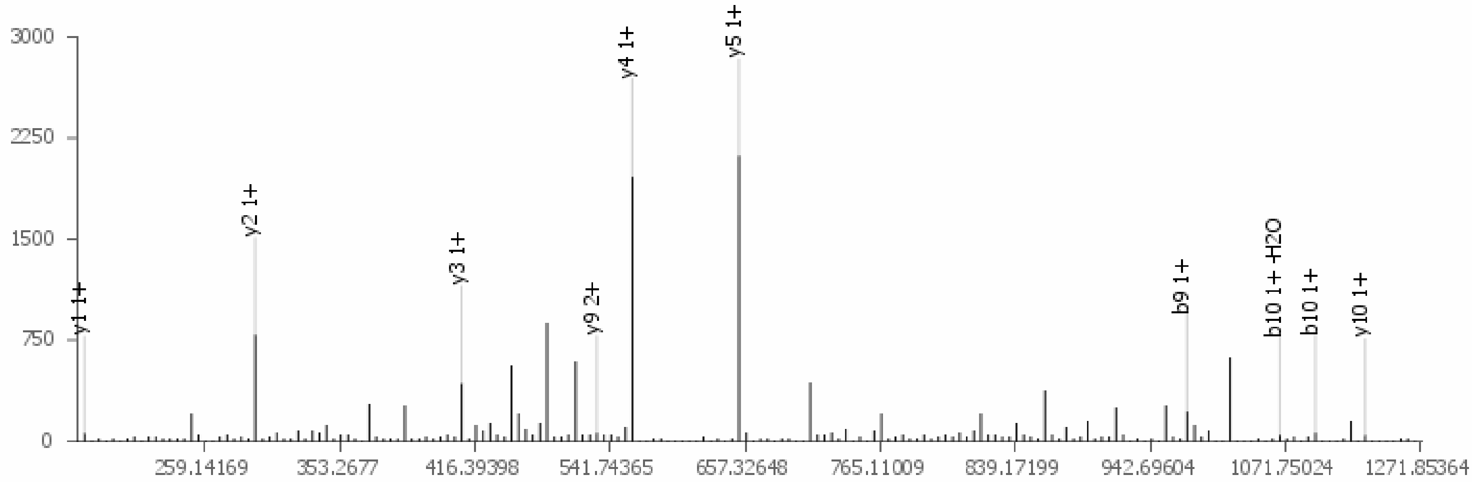

AT3G62700.1 - SI(pS)IESPR - 484.71715 - Charge:2

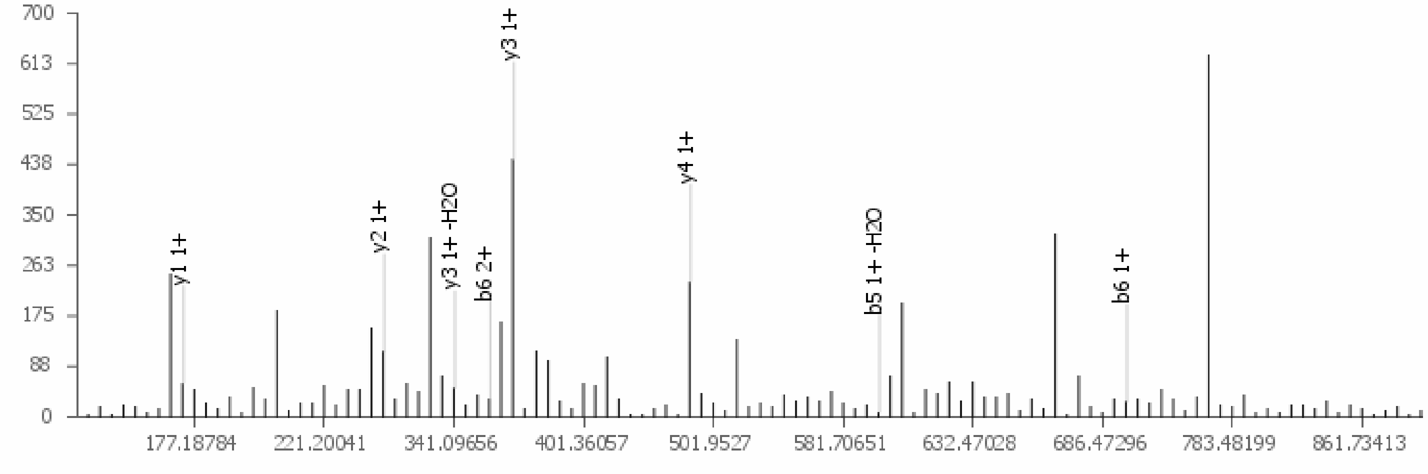

AT4G35230.1 - SY(pS)TNLAYTPPEYLR - 927.90864 - Charge:2

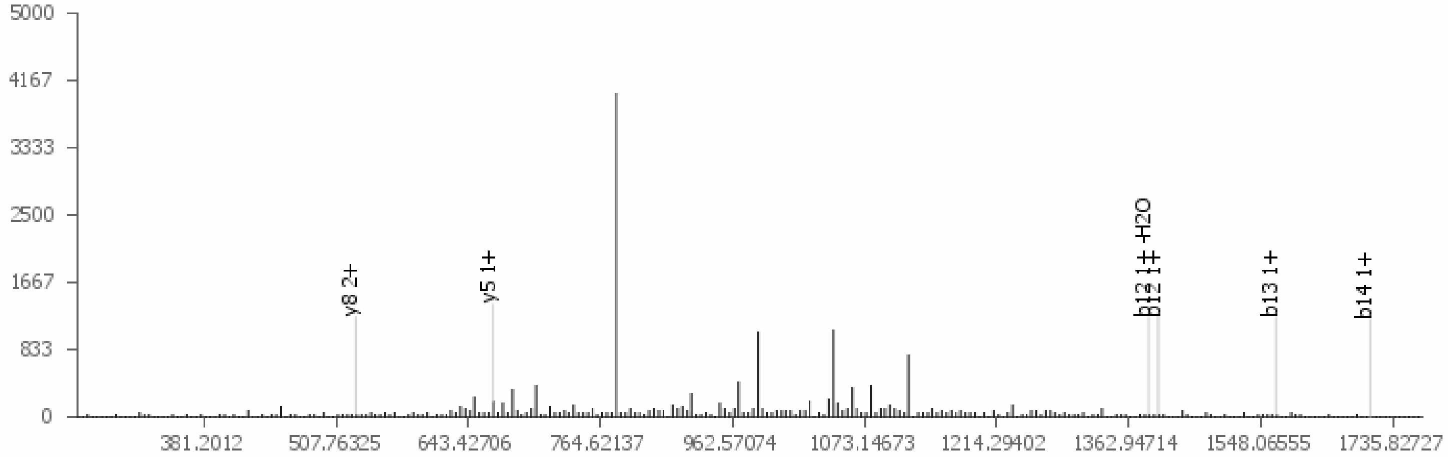

AT4G30190.1 - GLDIETPSHY(pT)V - 706.30322 - Charge:2

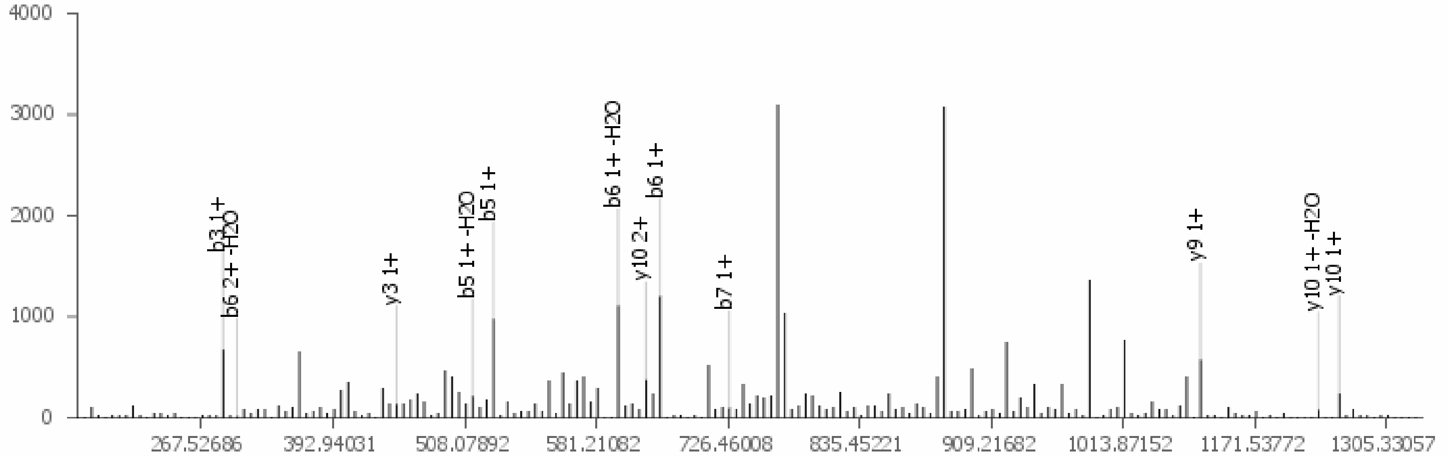

AT3G47780.1 - QG(s)KV(s)VDMEKPDVTHESK - 1090.98906 - Charge:2

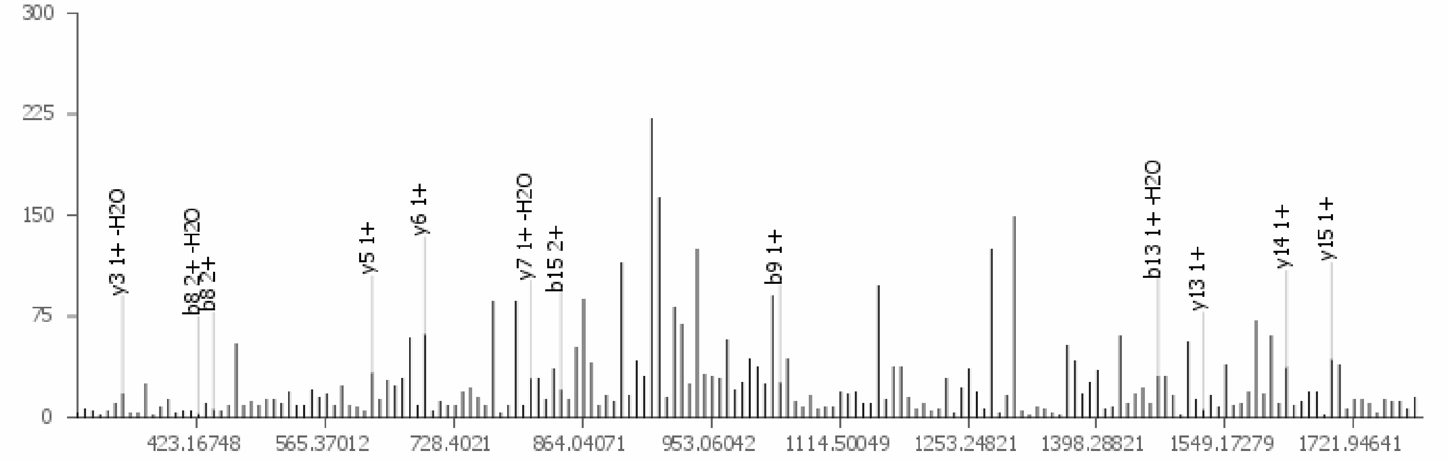

AT4G13510.1 - HGGFAYMYFDDDE(pS)HKAIQLR - 860.69925 - Charge:3

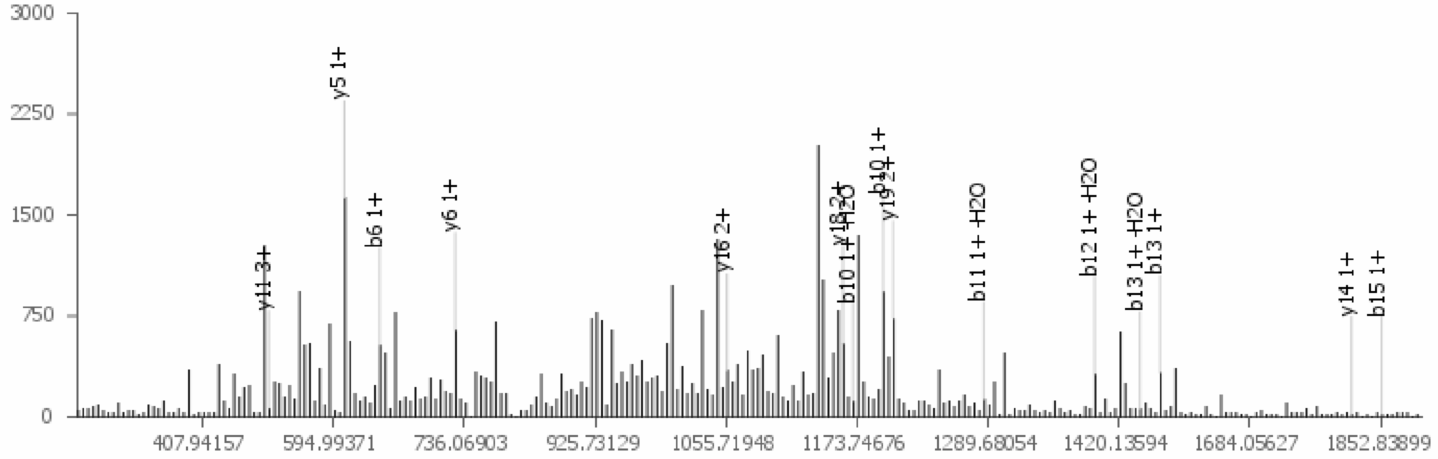

AT3G52400.1 - TSVADG(s)(s)PPHSHNIEMSK - 1030.9326 - Charge:2

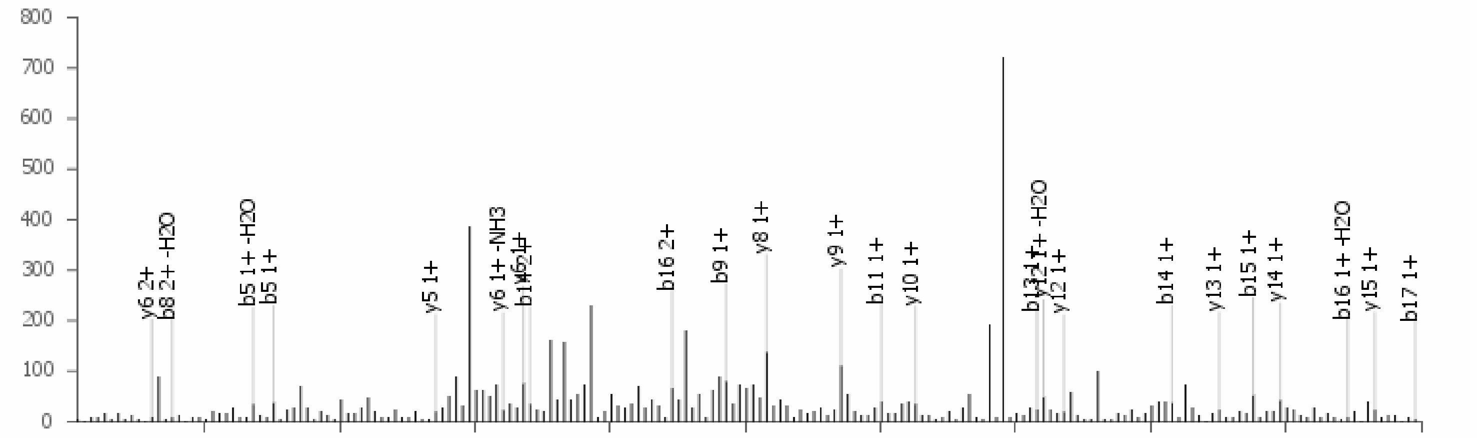

AT5G57110.1 - (pS)EHADSDSDTFYIPSK - 939.86211 - Charge:2

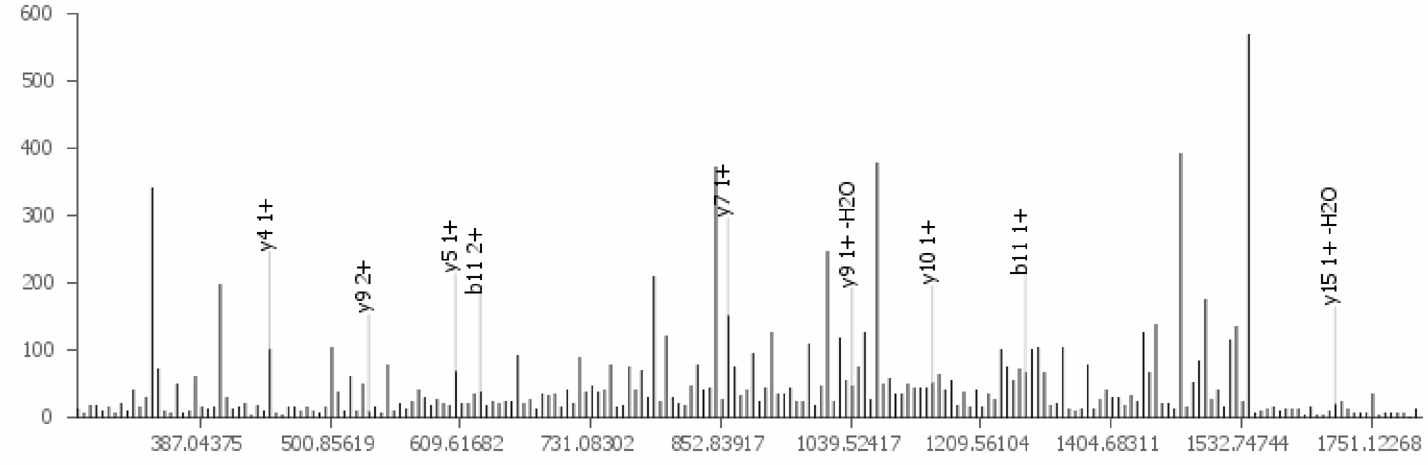

AT3G61260.1 - L(pS)FVR - 351.16463 - Charge:2

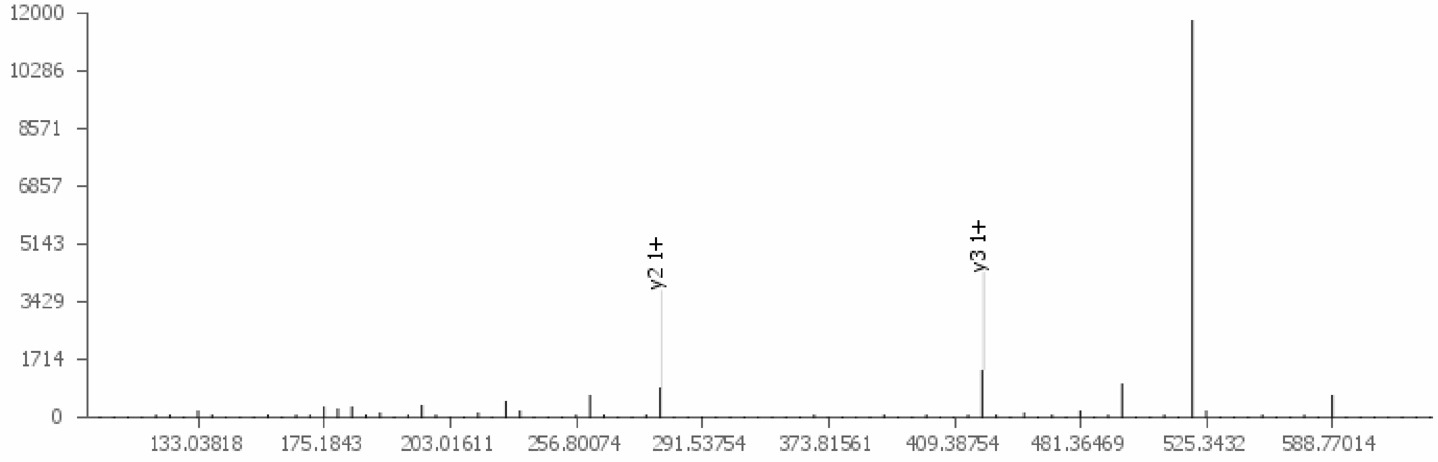

AT3G61260.1 - IALE(s)E(s)PAK - 562.75817 - Charge:2

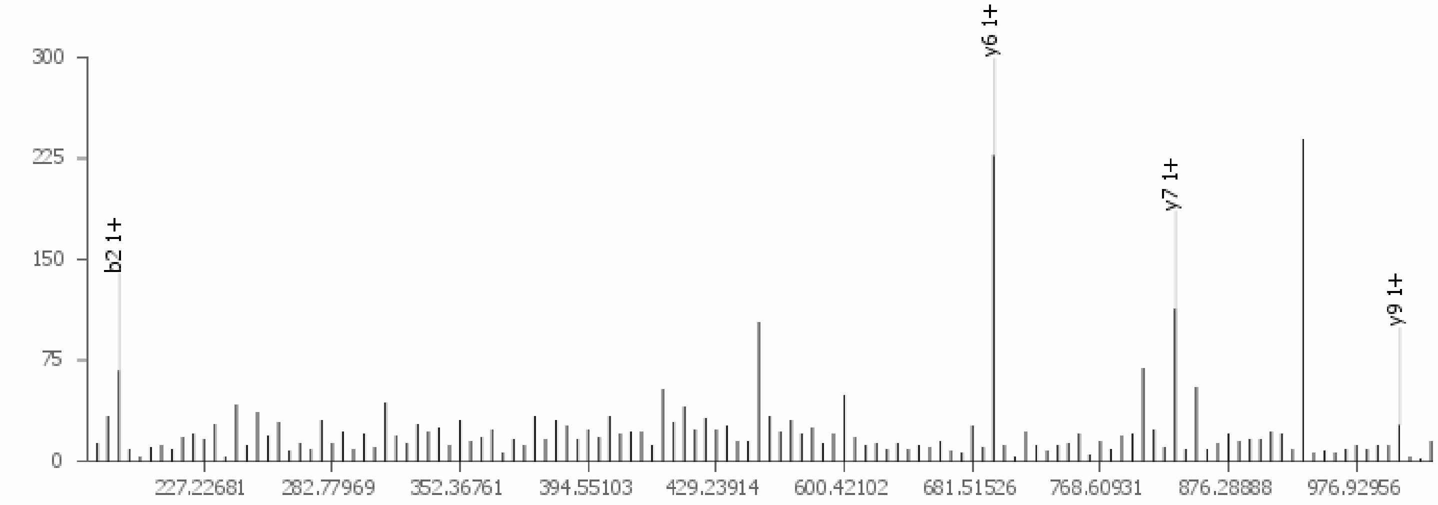

AT1G22280.1 - TDQAILSN(pS)SDLGR - 778.8395 - Charge:2

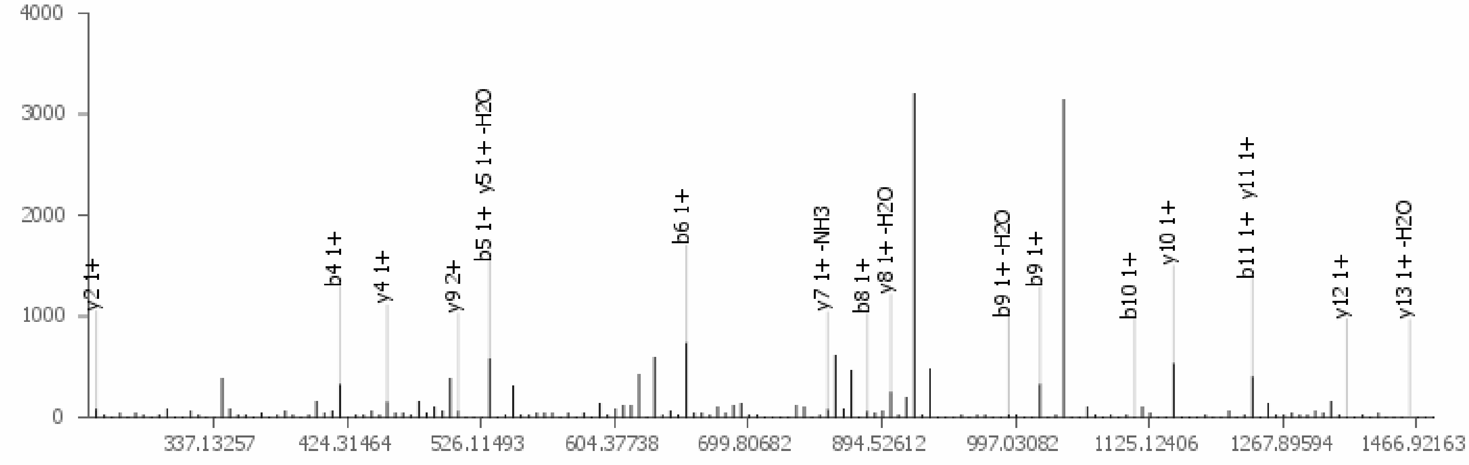

AT2G47800.1 - GITGLVTAE(pT)NSPTKPSDAVSVEK - 827.73437 - Charge:3

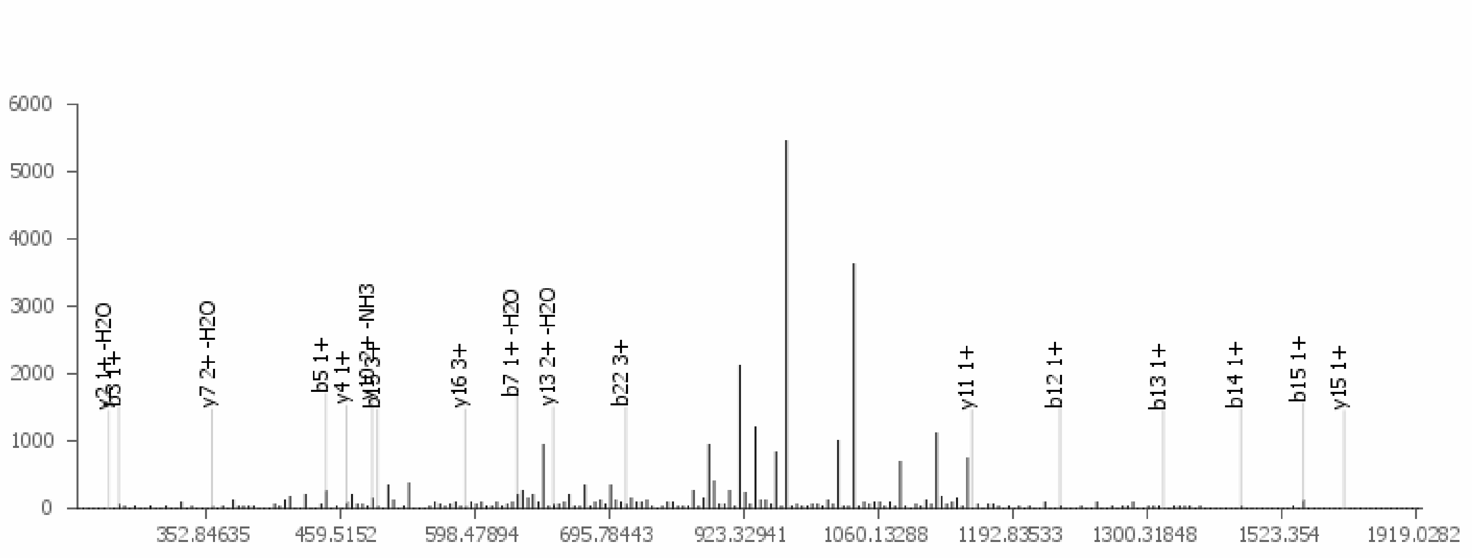

AT3G47780.1 - RP(pS)LQR - 418.70171 - Charge:2

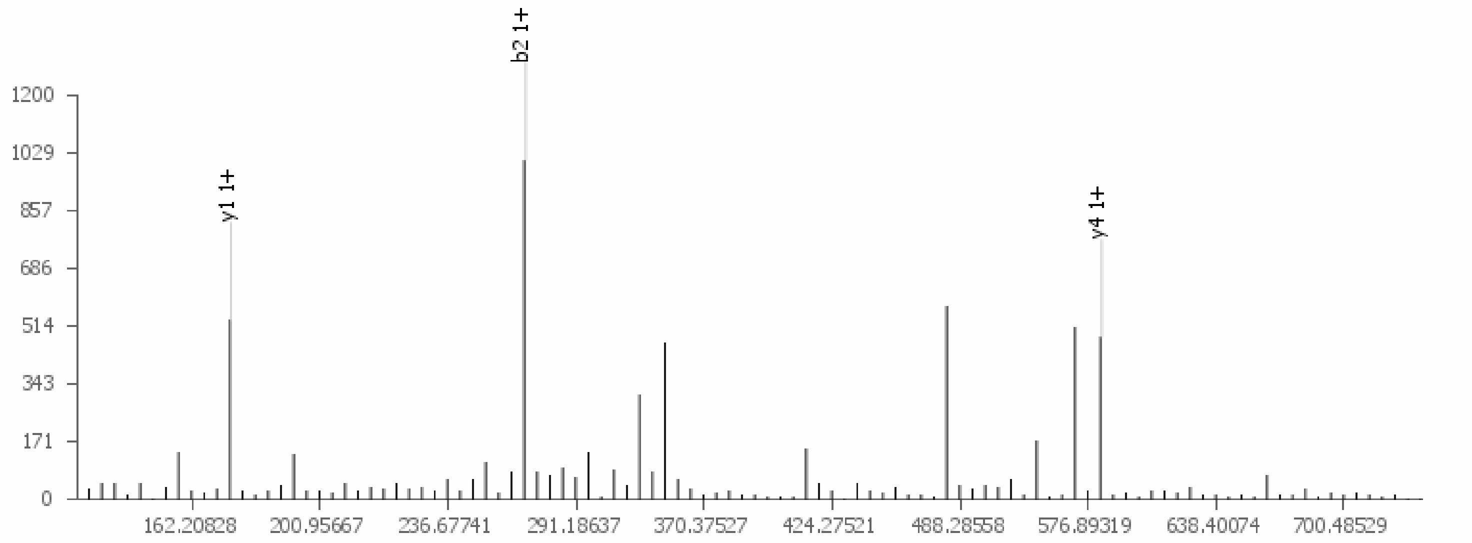

AT3G53420.1 - SLG(pS)FR(pS)AANV - 634.74678 - Charge:2

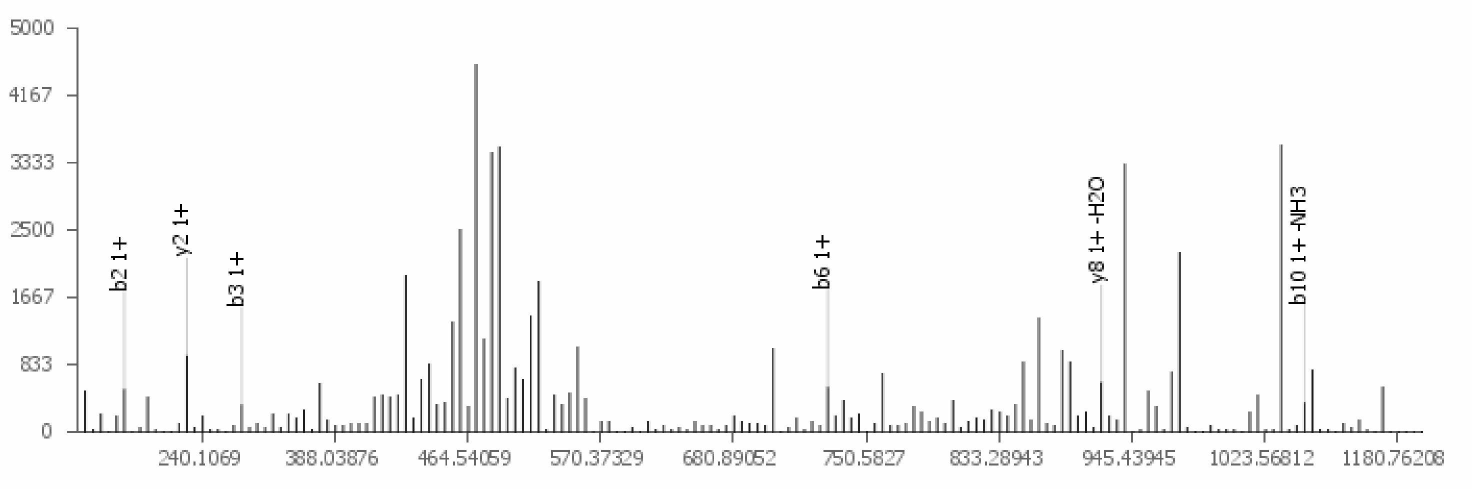

AT2G21880.1 - GQYHD(pS)VTDIIDPDQSR - 1013.42539 - Charge:2

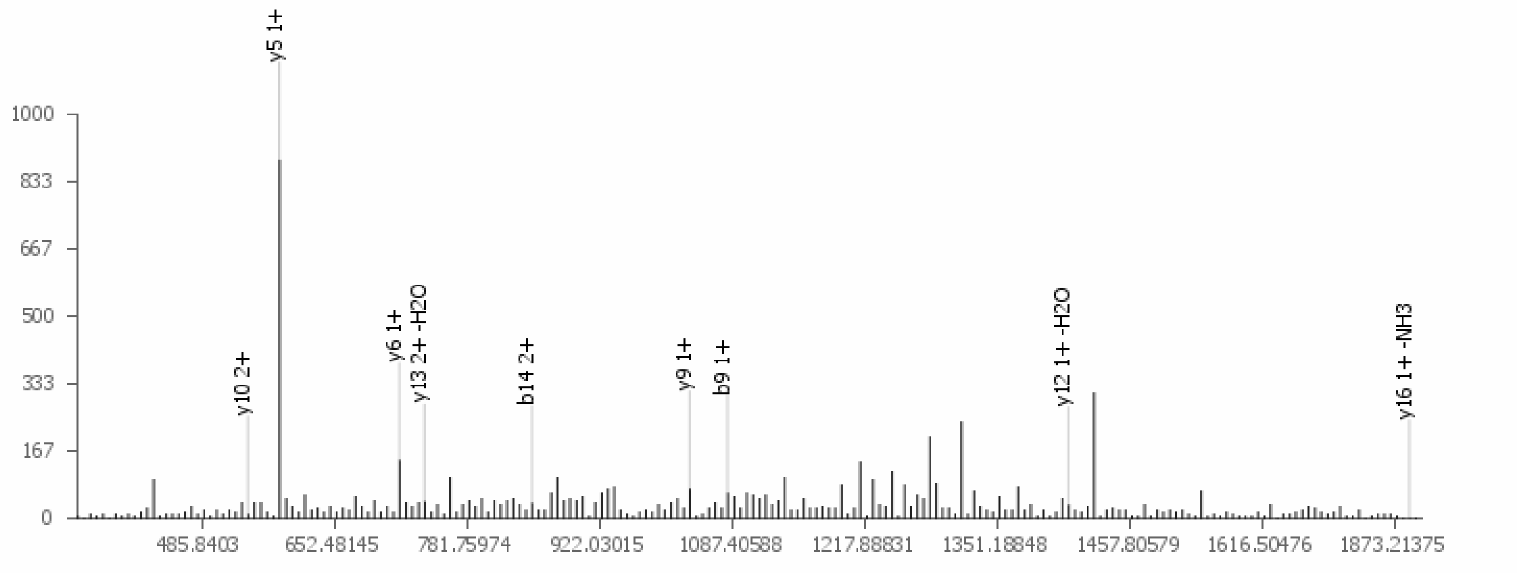

AT4G13510.1 - VEPR(pS)P(pS)P(s)GANT(t)PTPV - 1017.39321 - Charge:2

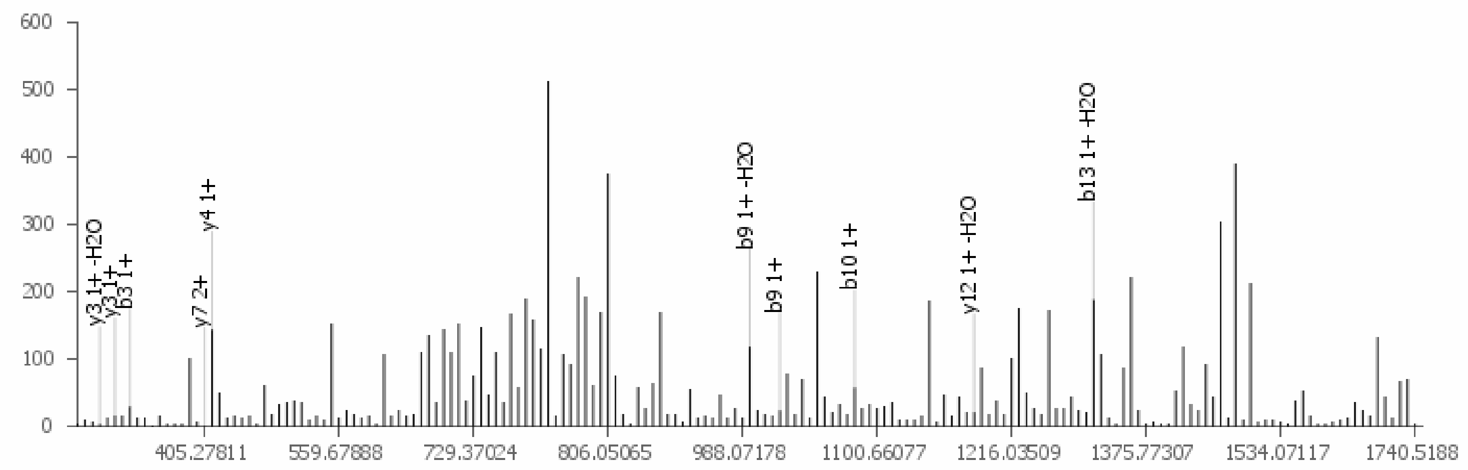

AT3G02880.1 - LIEEVSHSSG(pS)PNPV(pS)D - 957.37123 - Charge:2

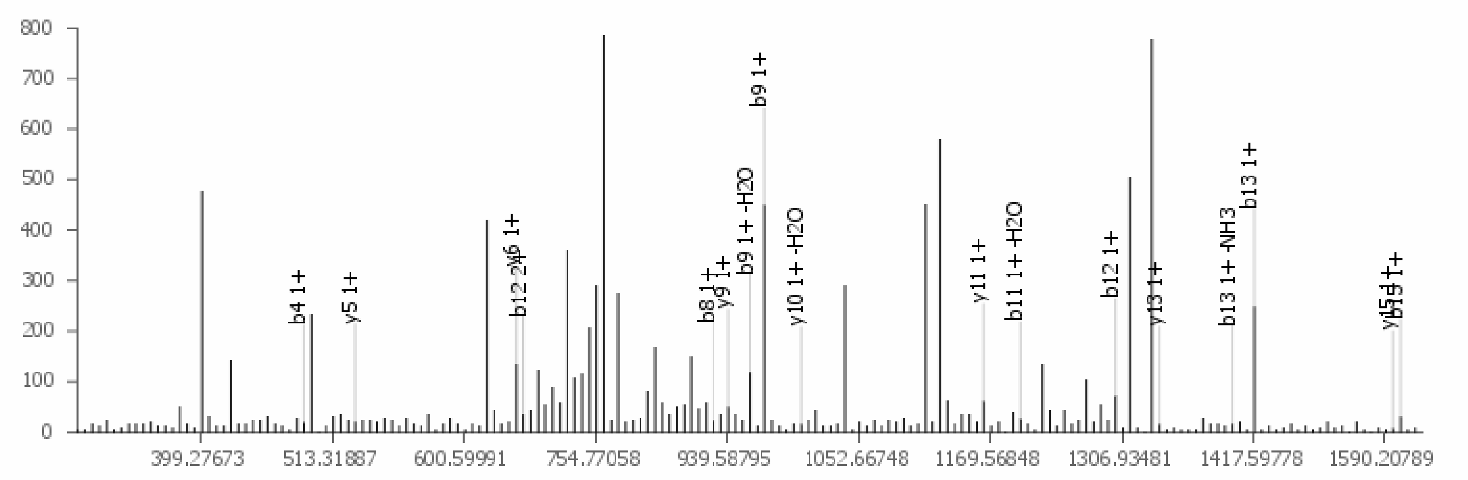

AT3G08710.1 - VTSIIDSVPE(pS)PQRP - 852.90314 - Charge:2

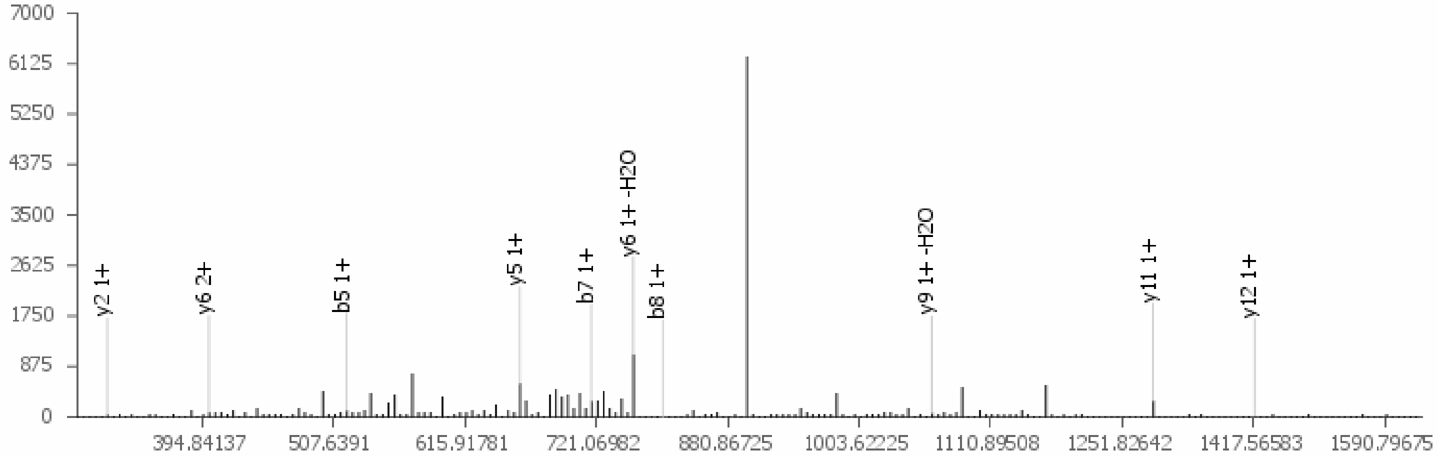

AT1G30360.1 - VAAASAAQ(pS)LHCQMVDK - 905.38875 - Charge:2

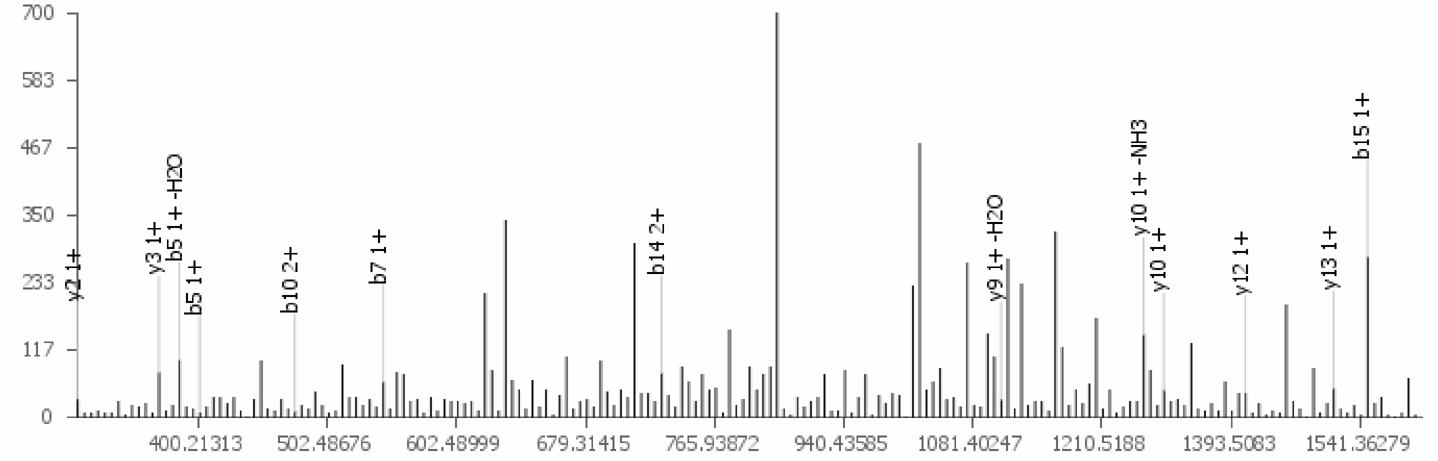

AT5G45380.1 - VVEAYA(pS)GDEDVDVPAEELREEK - 1315.07162 - Charge:3

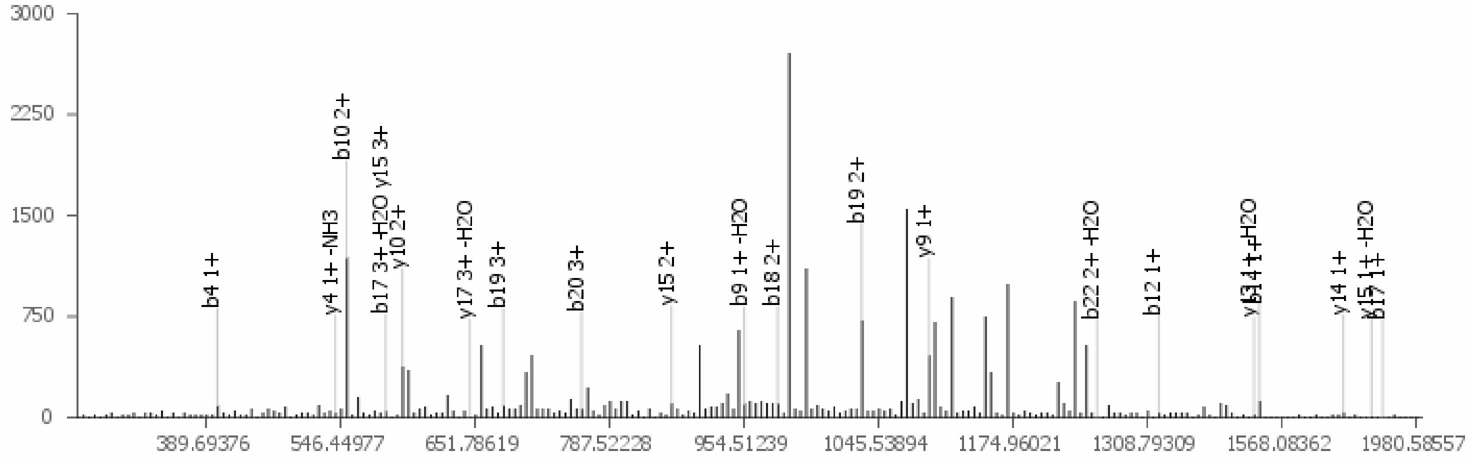

AT3G25070.1 - ADE(pS)PEKVTVVPK - 739.84971 - Charge:2

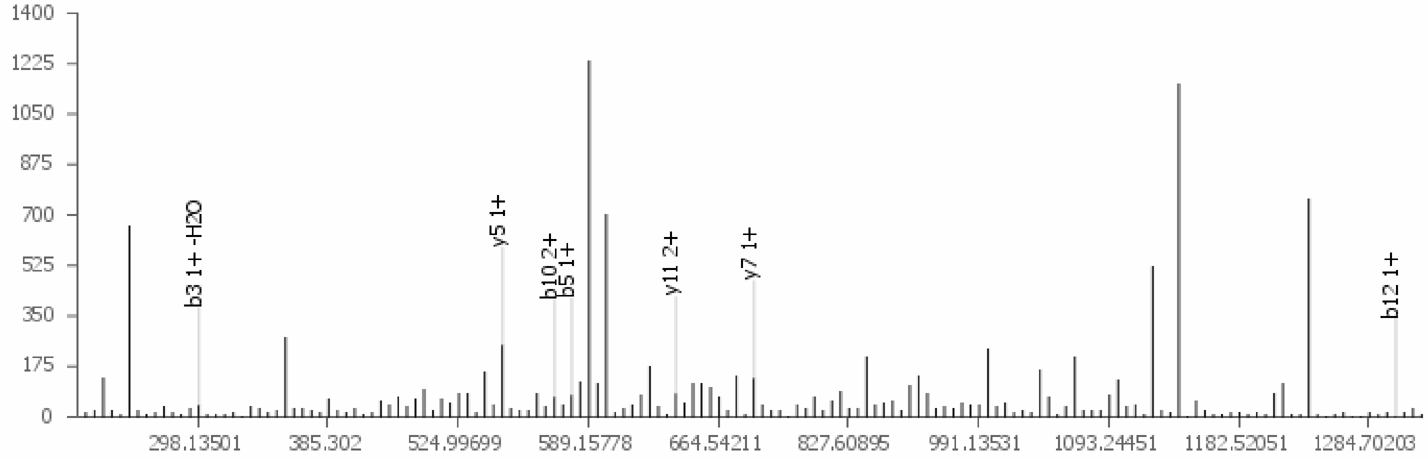

AT3G24300.1 - SA(pT)PPRV - 404.18206 - Charge:2

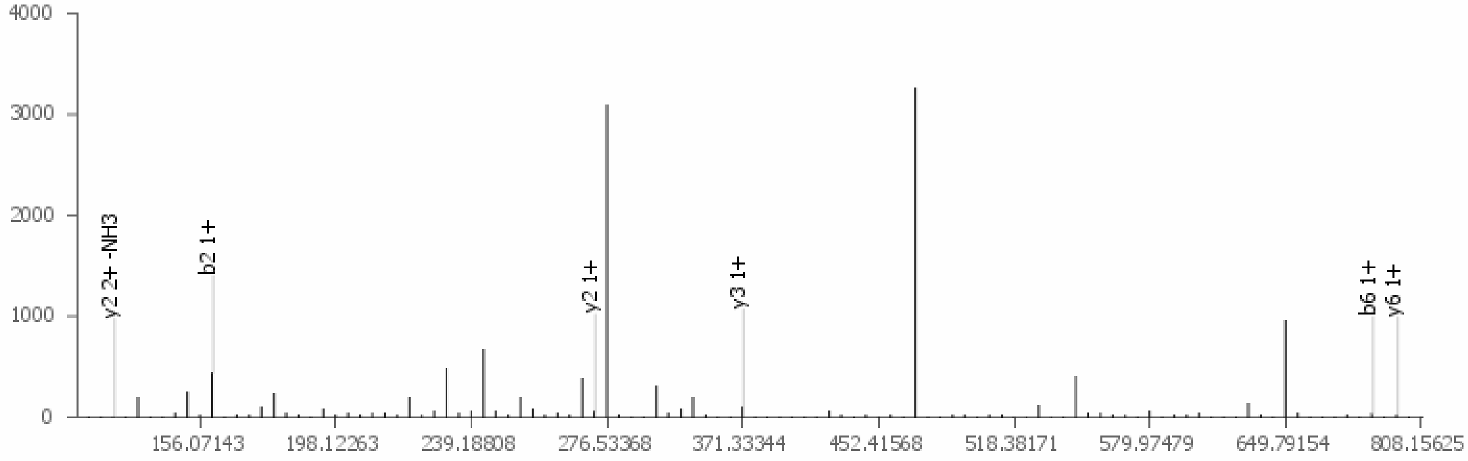

AT1G15400.1 - VSPAVDPP(pS)PR - 601.27314 - Charge:2

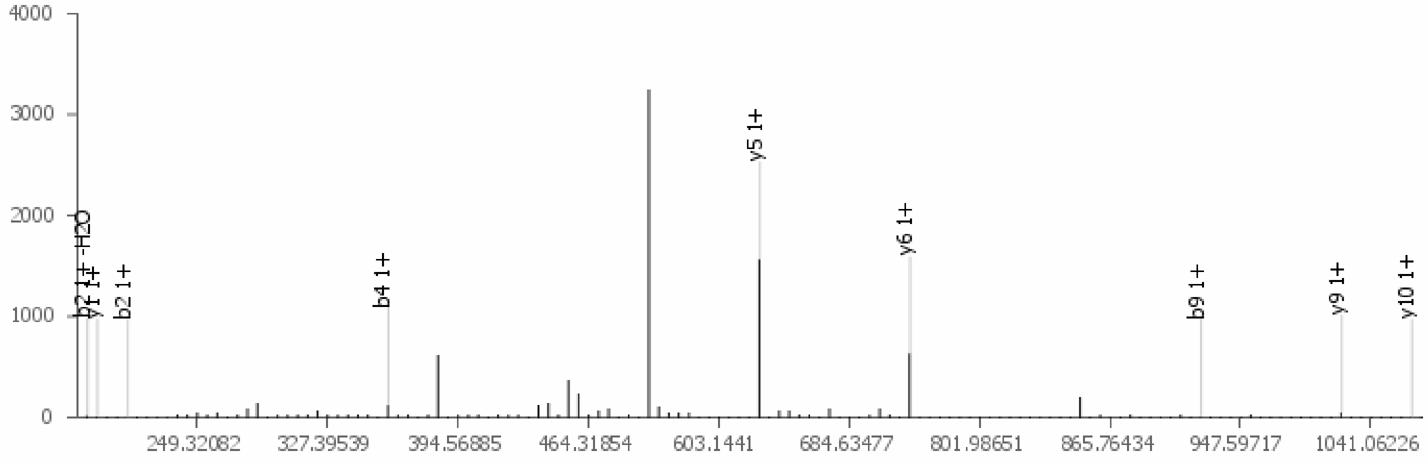

AT1G34750.1 - TDQAILSH(pS)SDLGR - 790.3501 - Charge:2

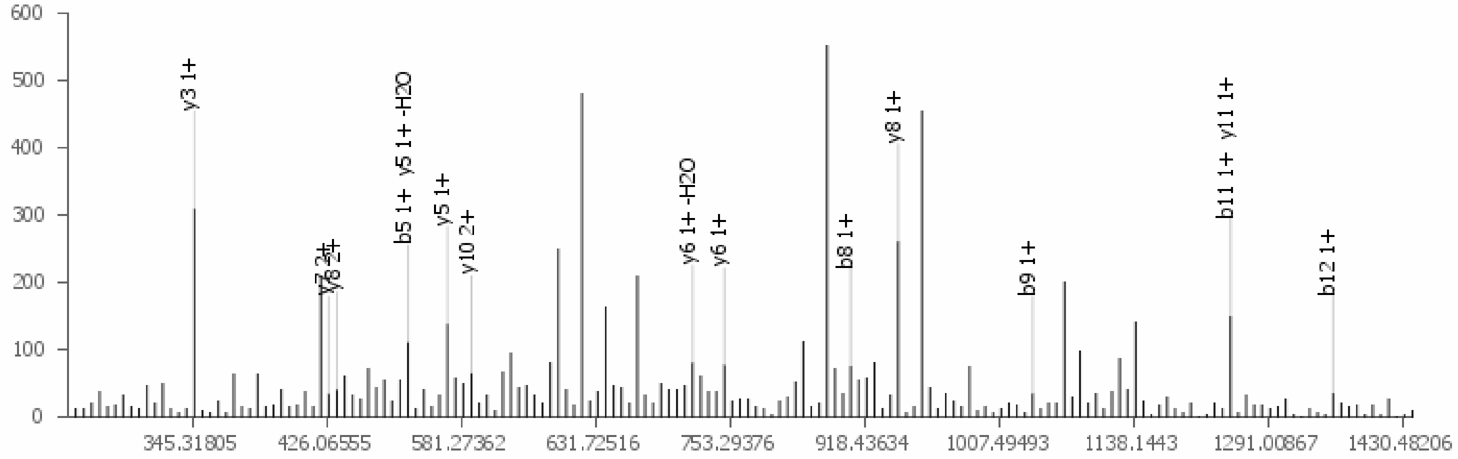

AT5G47910.1 - TS(pS)AAIHALK - 539.75985 - Charge:2

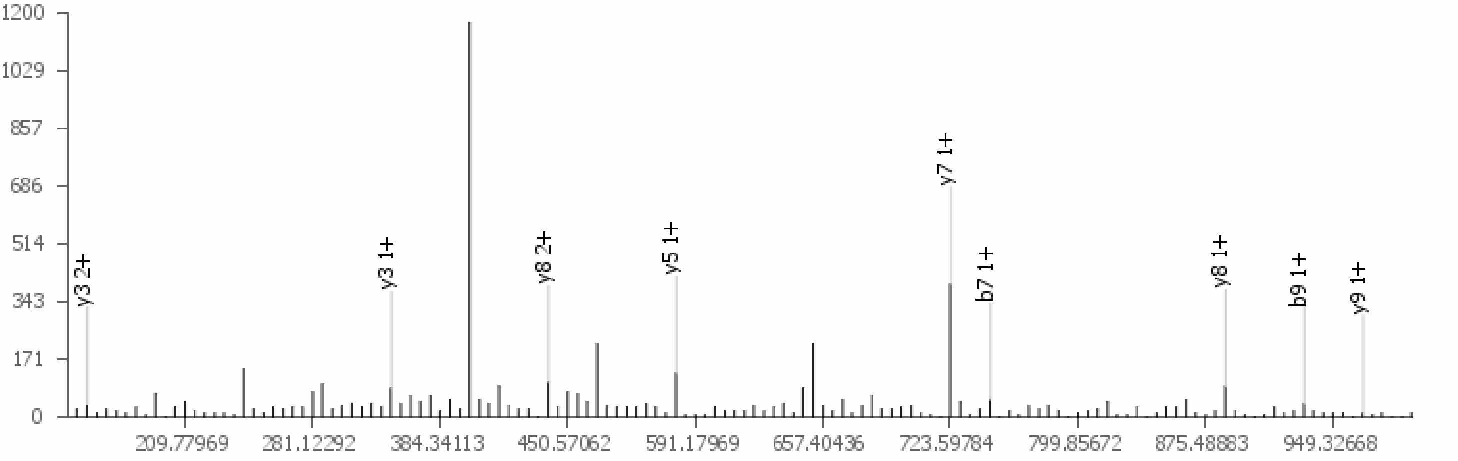

AT2G45820.1 - ALAVVEKPIEEH(pT)PKK - 934.99027 - Charge:3

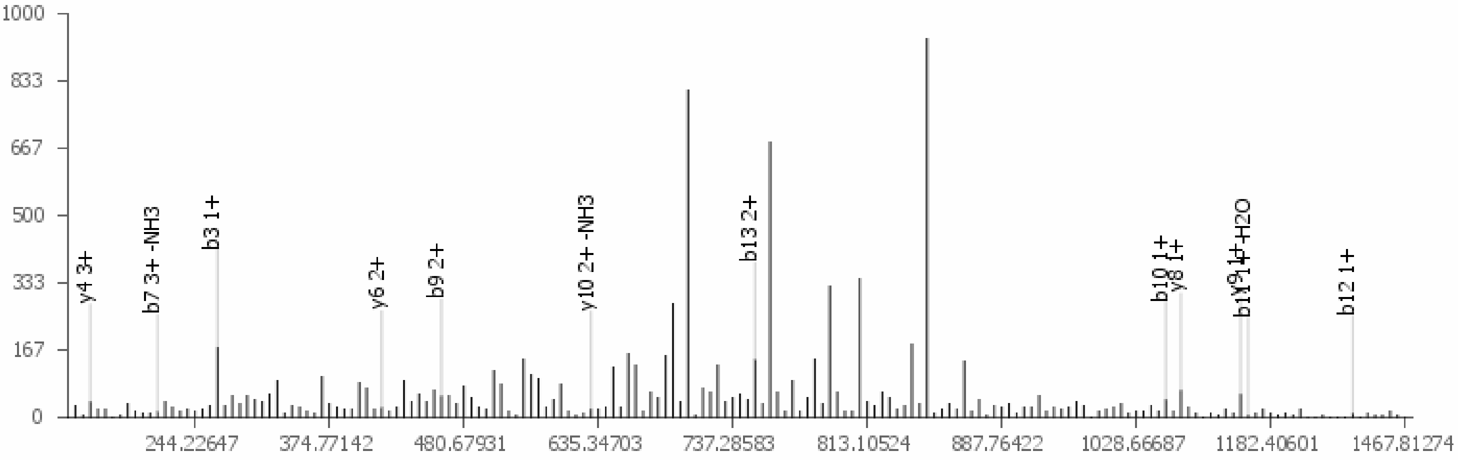

AT2G47000.1 - MSSIE(pS)FK - 504.69979 - Charge:2

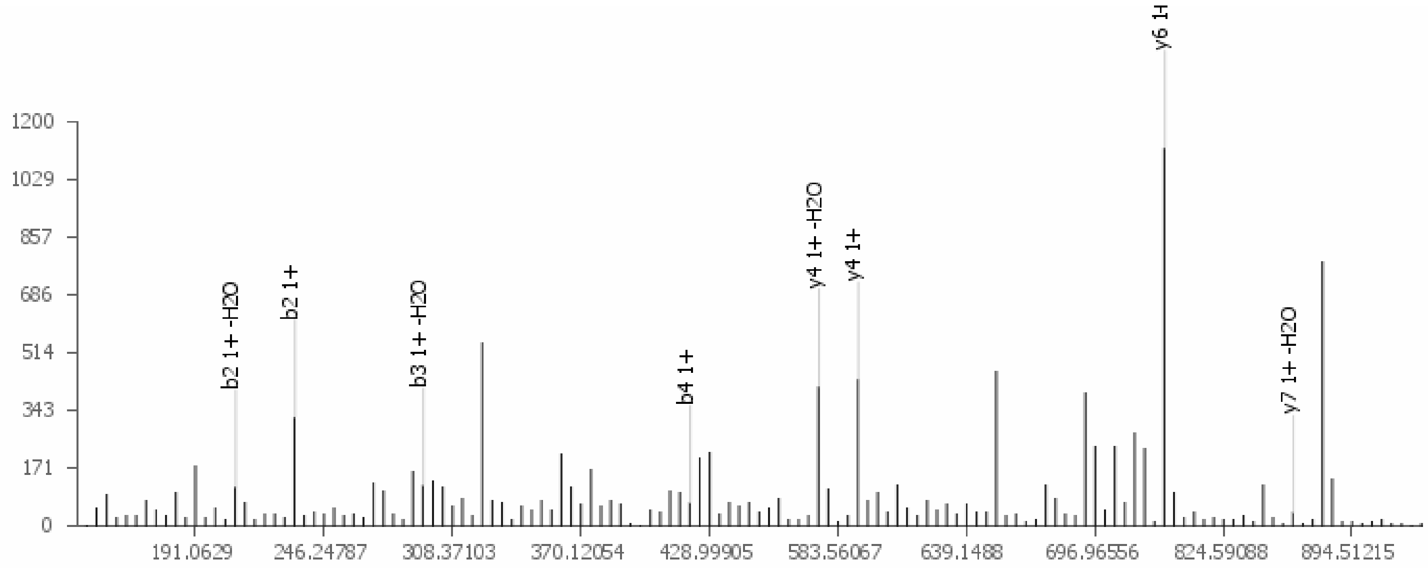

AT1G53350.1 - SL(pS)LQER - 456.70347 - Charge:2

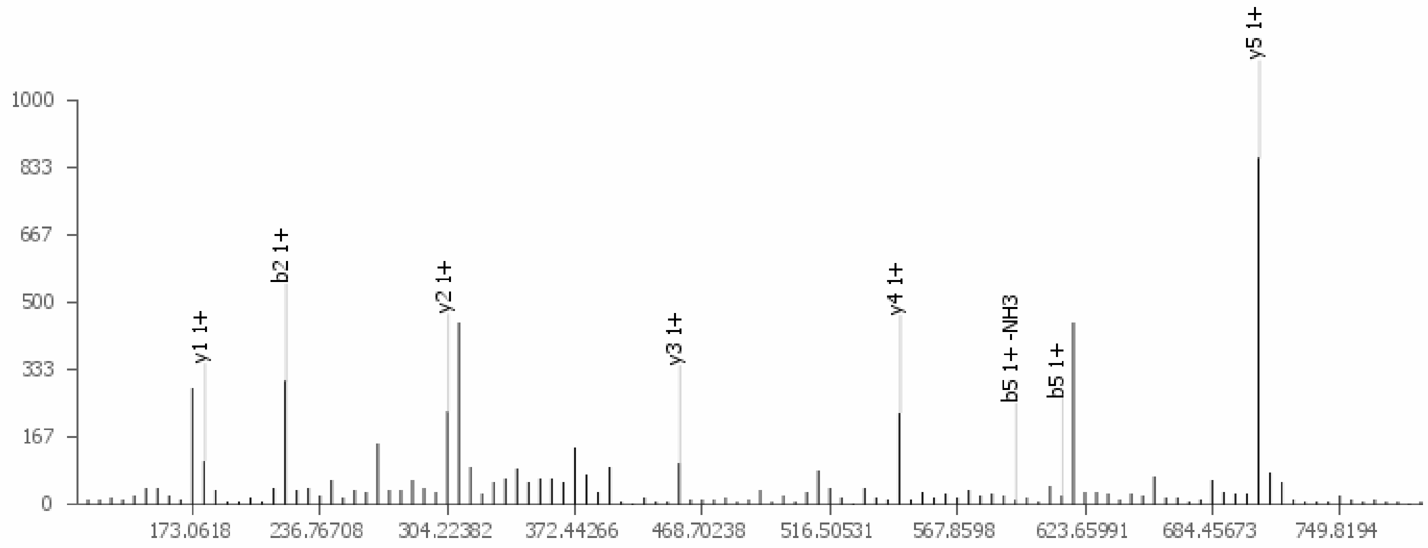

AT5G49890.1 - TTFG(pS)QILR - 551.75915 - Charge:2

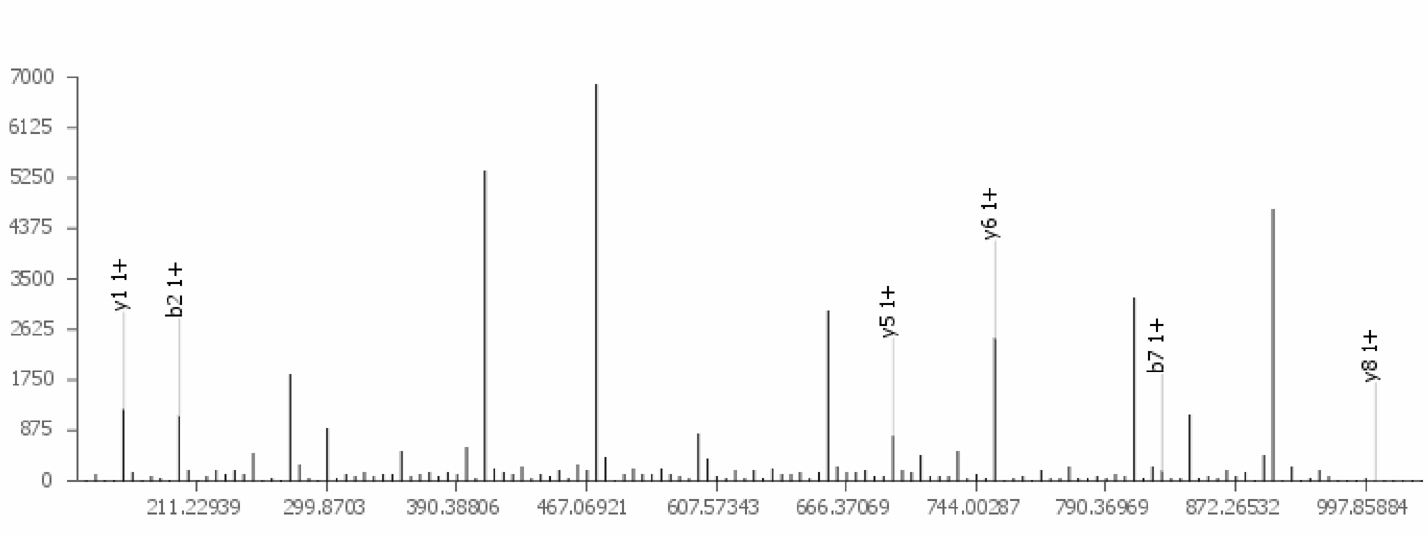

AT1G08090.1 - EQSFAFSVQ(pS)PIVHTDK - 1000.45095 - Charge:2

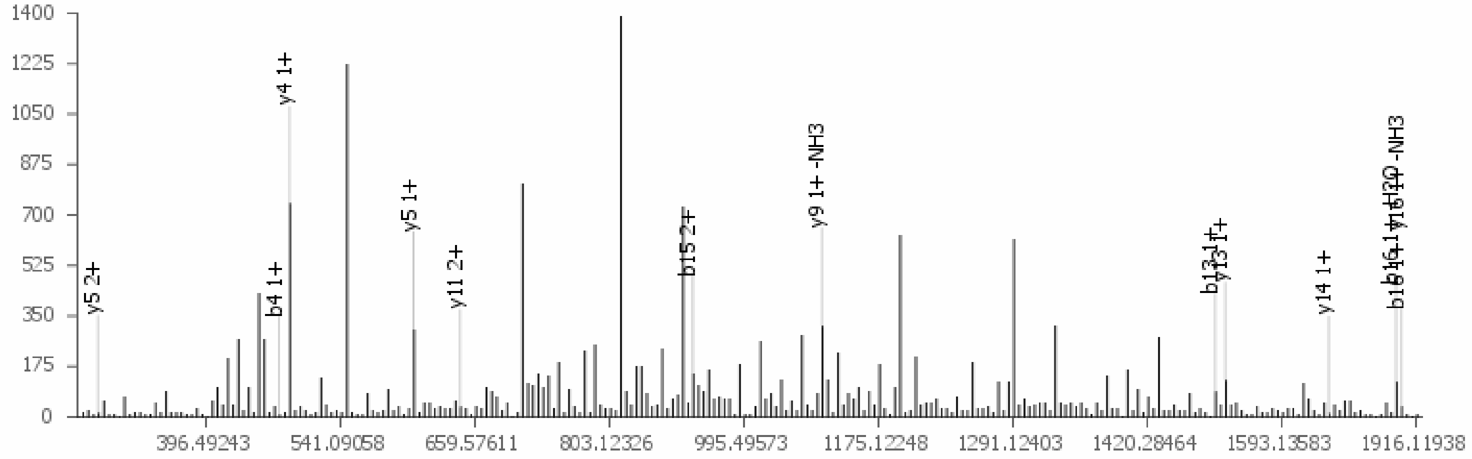

AT1G75220.1 - Q(s)(s)MMGSSQVIR - 695.78561 - Charge:2

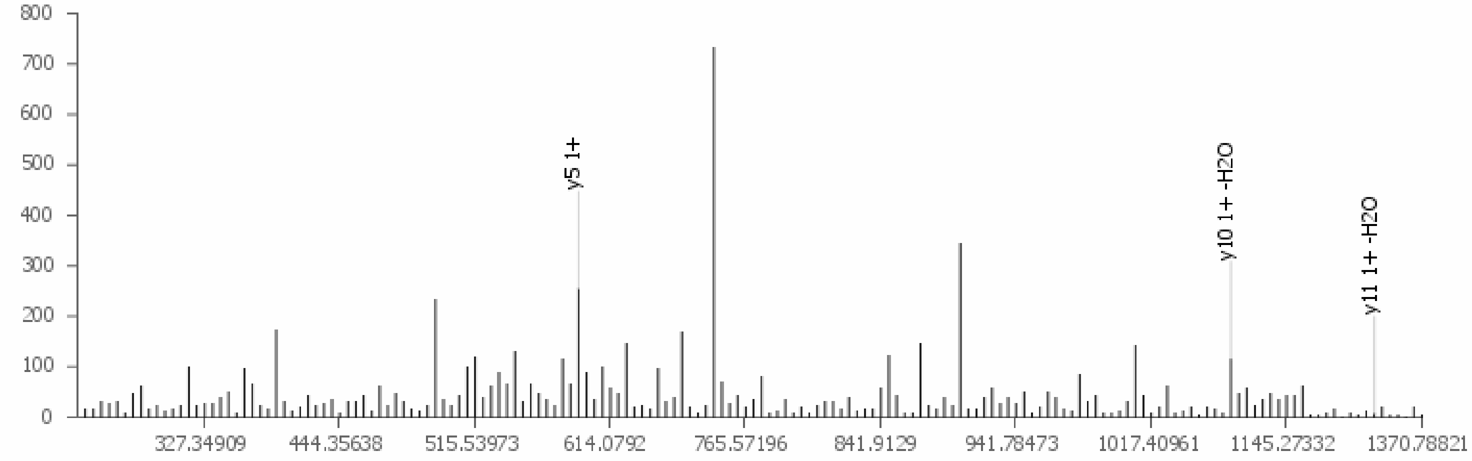

AT3G24300.1 - HGGFAYIYHDNDDE(pS)HRVDPG(pS)PFPR - 787.06151 - Charge:5

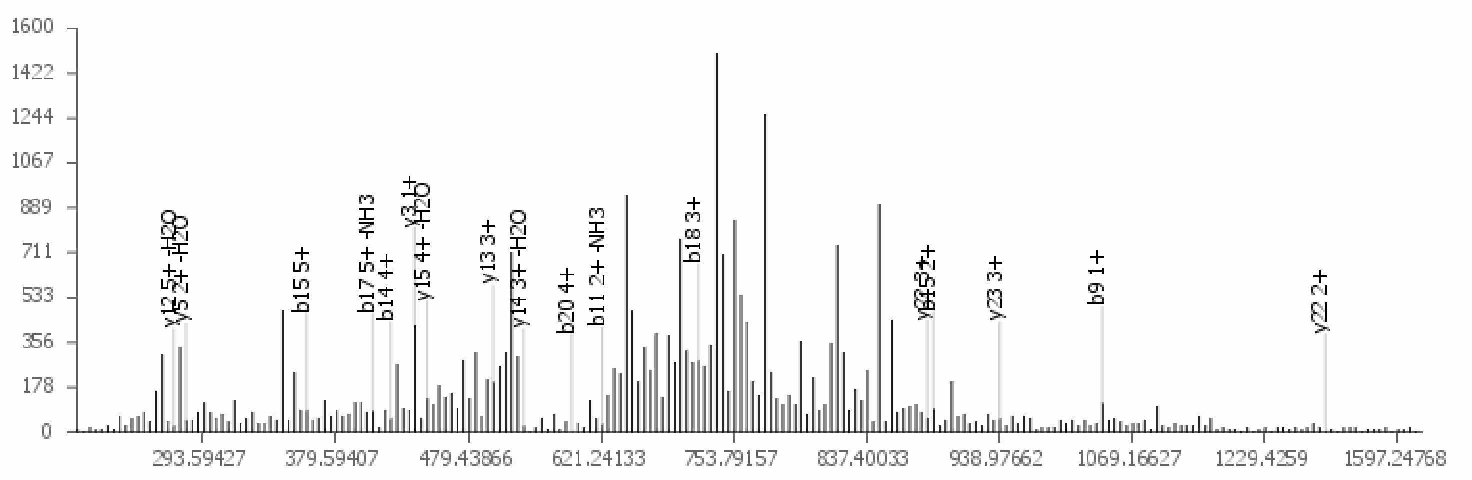

AT5G04930.1 - EVTFGDLG(pS)KR - 644.79055 - Charge:2

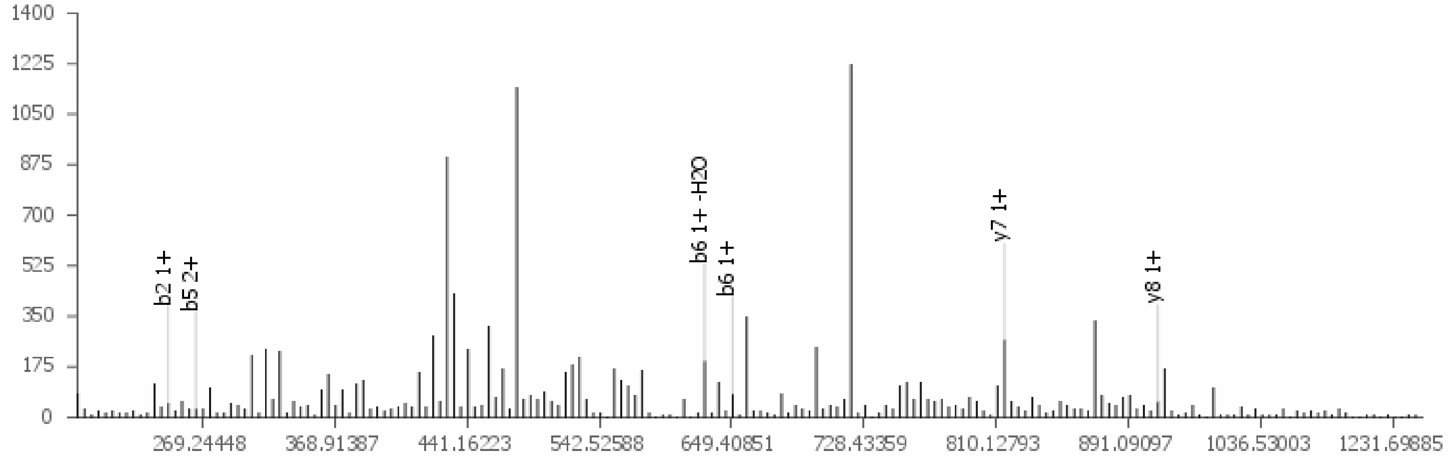

AT3G17420.1 - SNATTLPVTQ(pS)PR - 726.33952 - Charge:2

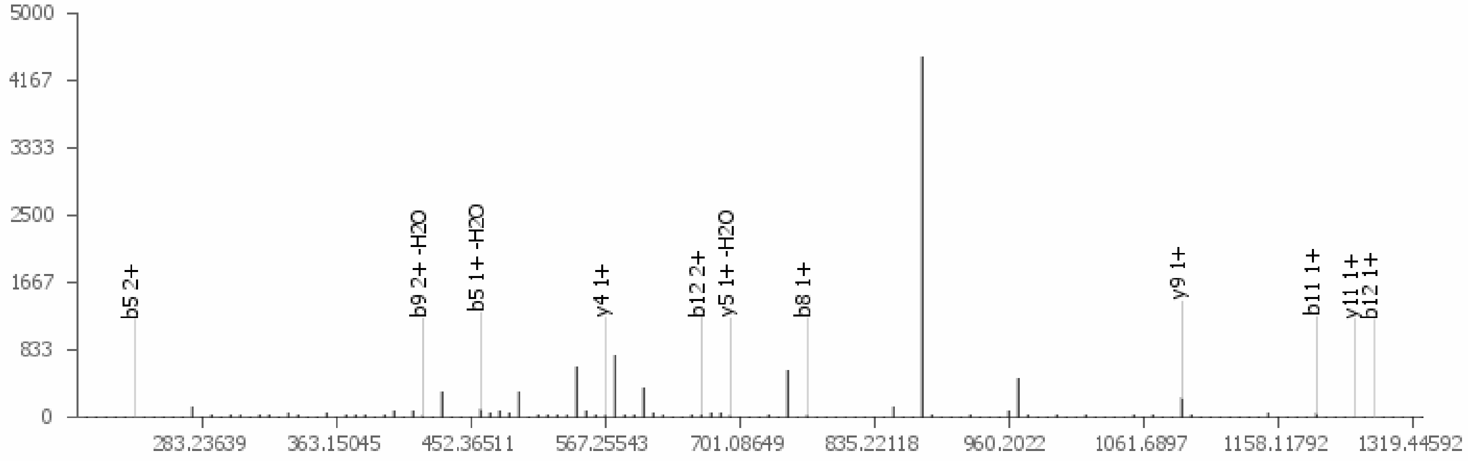

AT5G17010.1 - SSGEI(pS)PEREPLIK - 811.386 - Charge:2

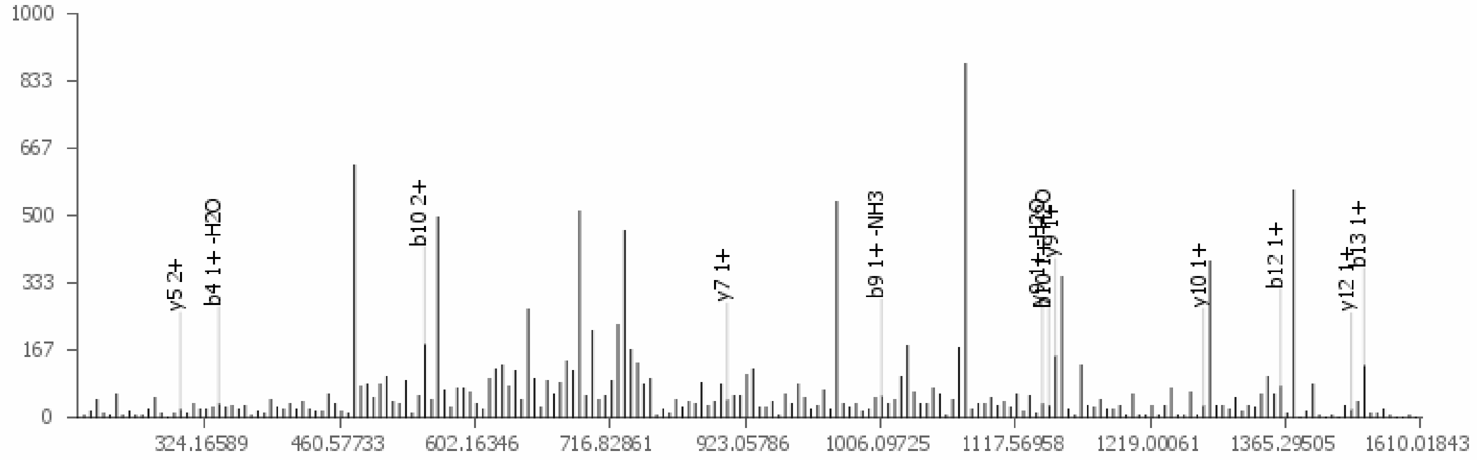

AT3G24300.1 - VDPG(pS)PFPR - 526.22606 - Charge:2

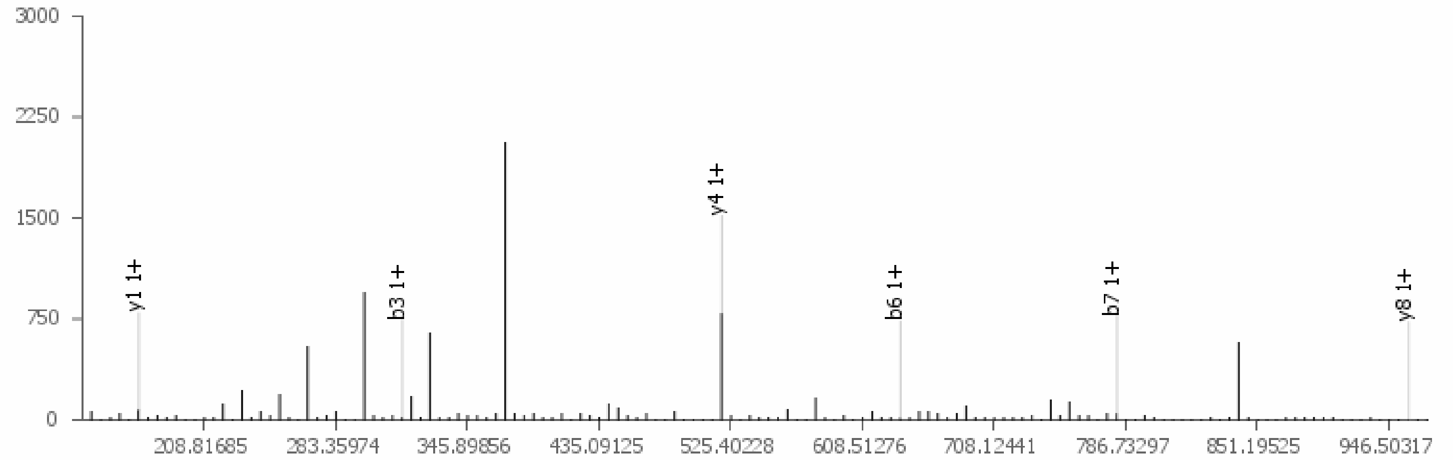

AT5G49890.1 - KI(pS)GILDDGSVGFR - 772.37348 - Charge:2

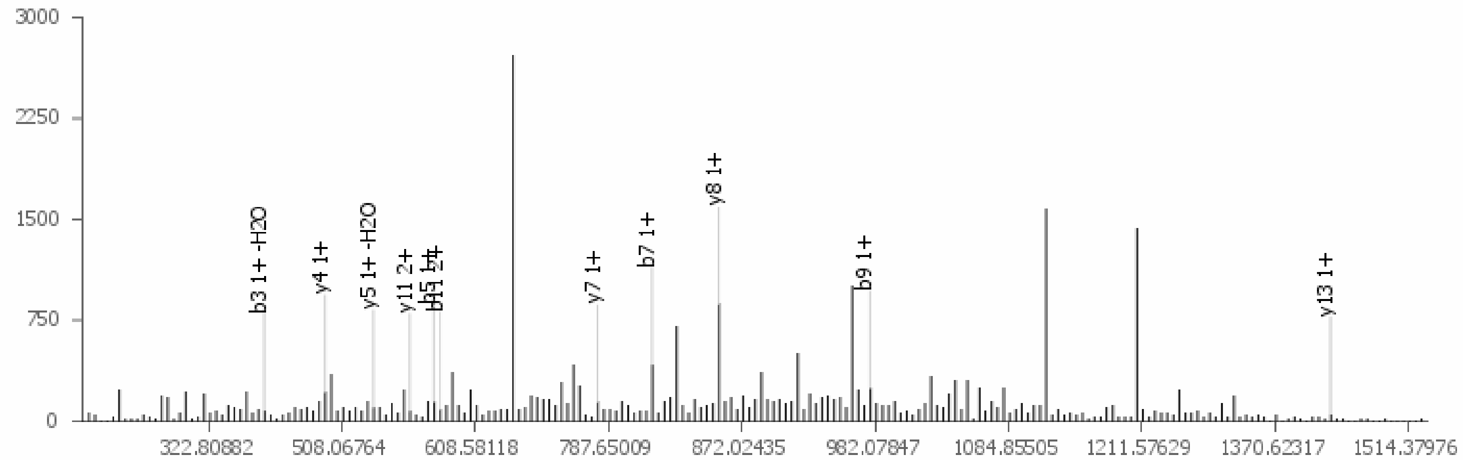

AT2G39010.1 - AYG(pS)VRSQLHELHA - 549.91722 - Charge:3

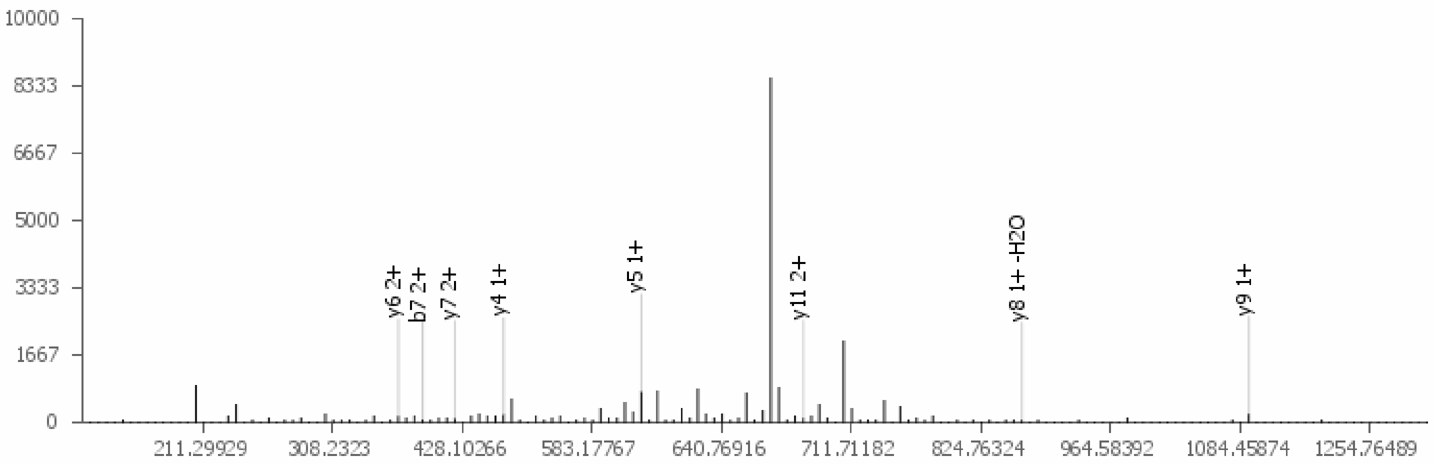

AT1G15400.1 - TTGRV(pS)PAVDPP(pS)PR - 848.86901 - Charge:2

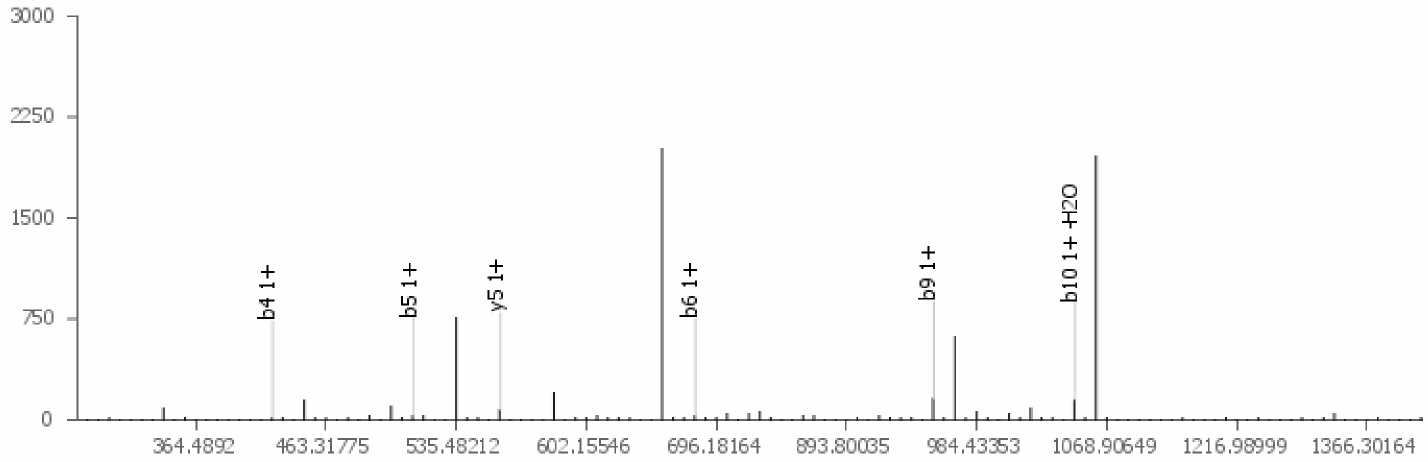

AT5G60660.1 - ALG(pS)FG(pS)FGSFR - 696.76476 - Charge:2

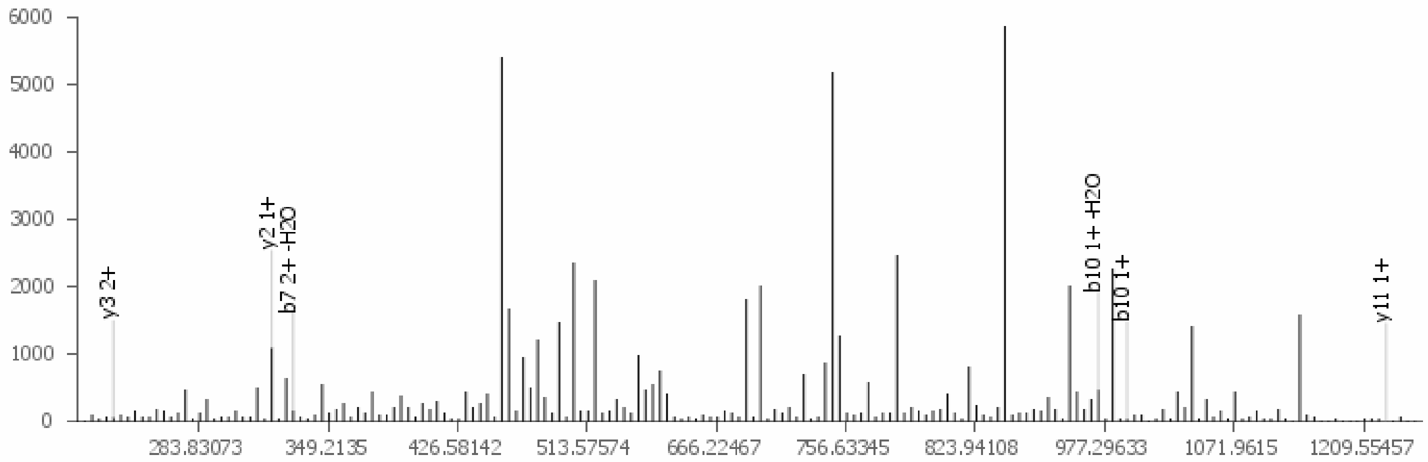

AT3G50150.1 - IG(pY)AR - 330.14056 - Charge:2

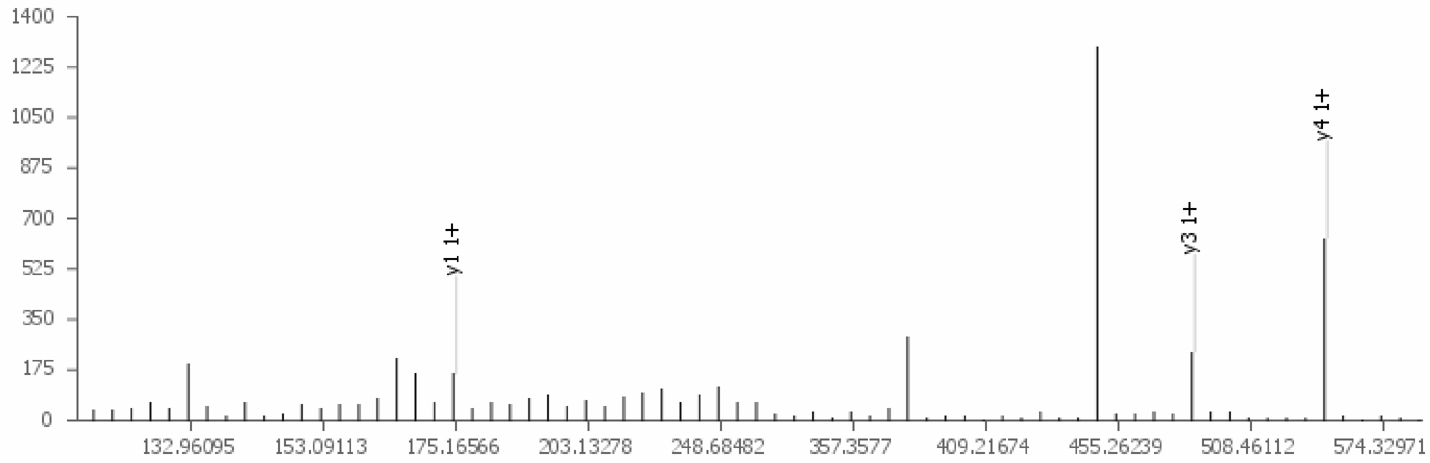

AT1G01100.1 - KDEPAEE(pS)DGDLGFGLFD - 1010.89981 - Charge:2

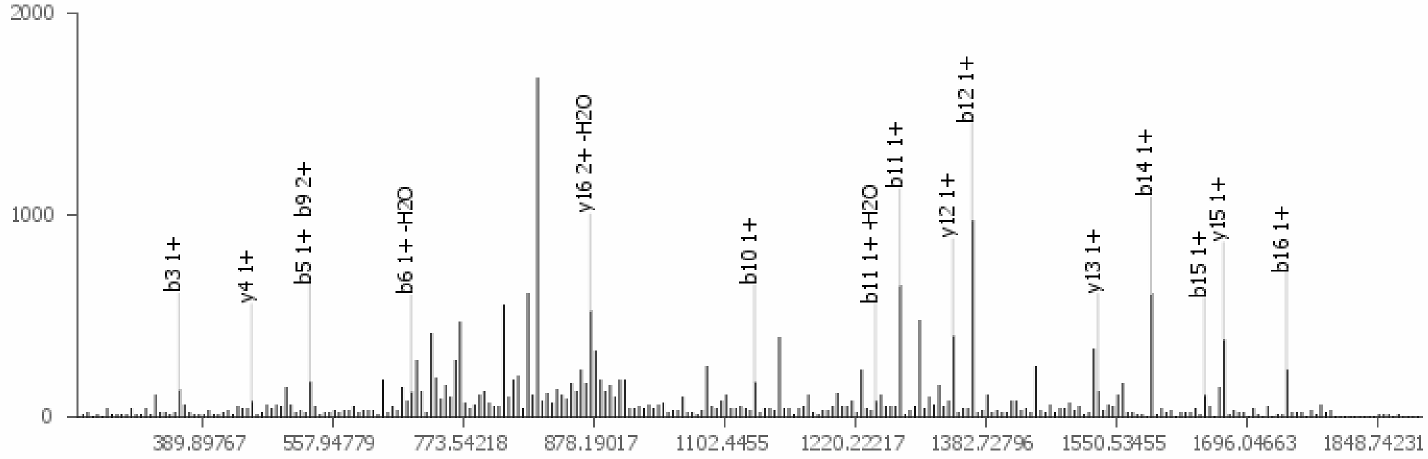

AT5G02310.1 - EIN(oxM)INQSL(pT)R - 707.822352 - Charge:2

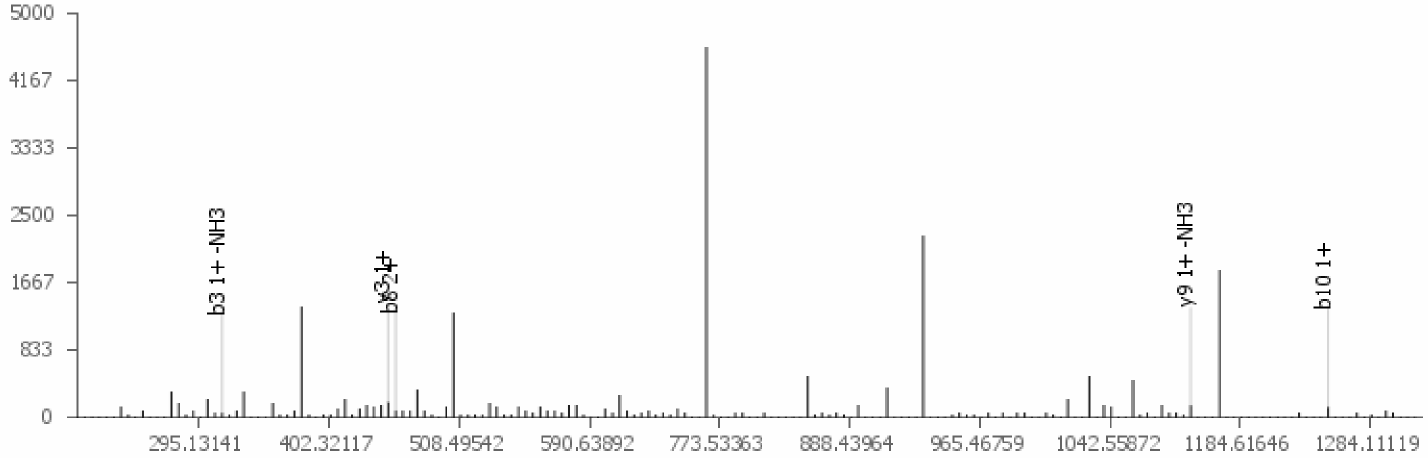

AT5G60100.1 - EDLQ(pS)LEQTLK - 692.32642 - Charge:2

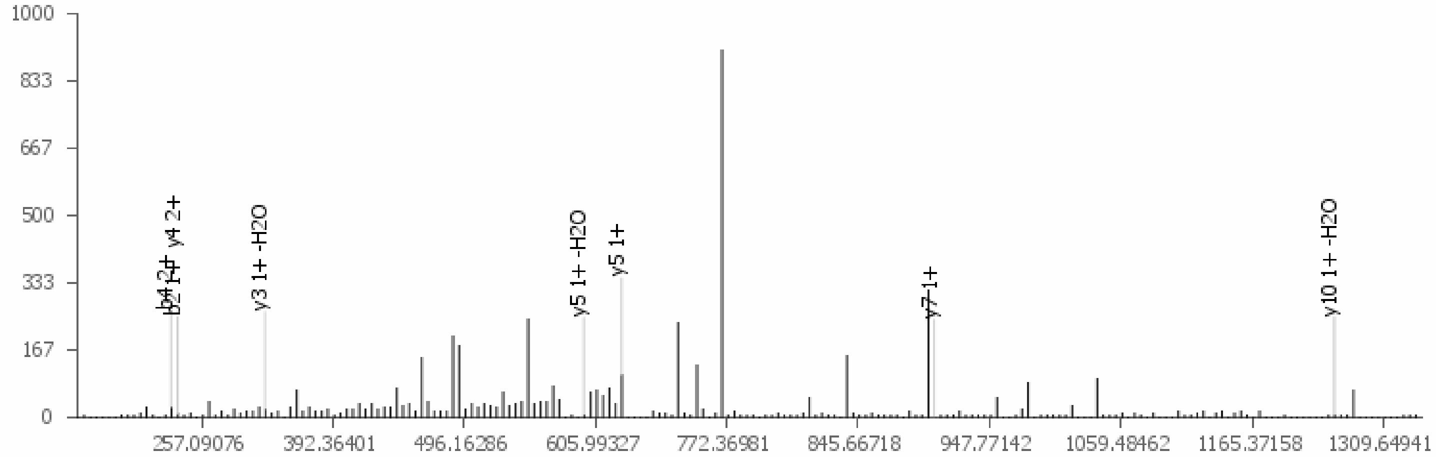

AT1G02280.1 - TIDVLL(pY)VDR - 643.82639 - Charge:2

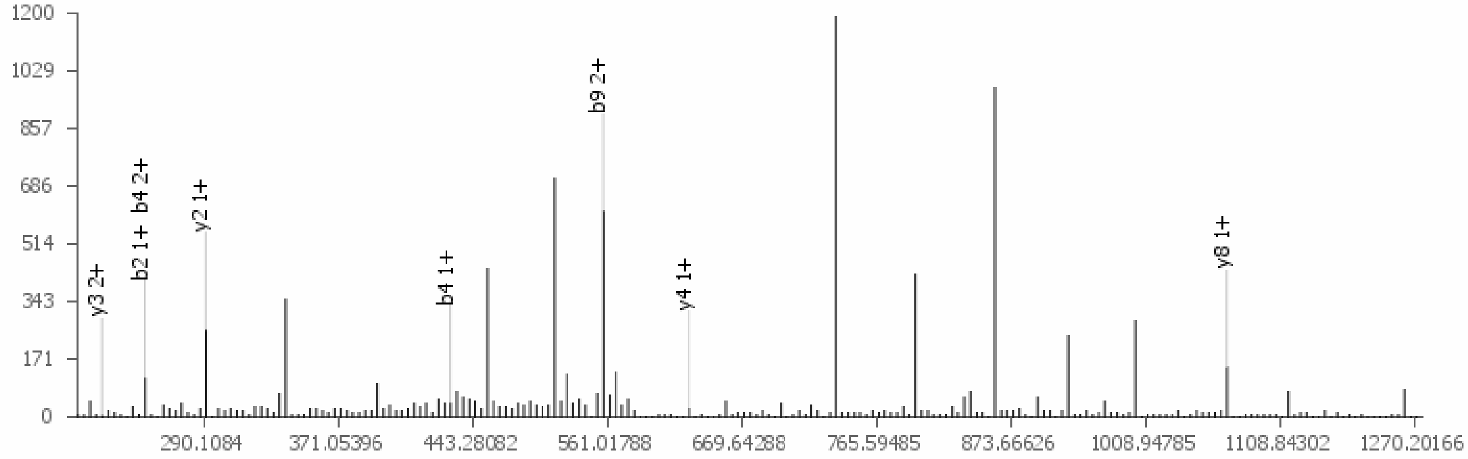

AT5G37600.1 - (s)LV(s)DLINLNLSDSTDK - 957.45013 - Charge:2

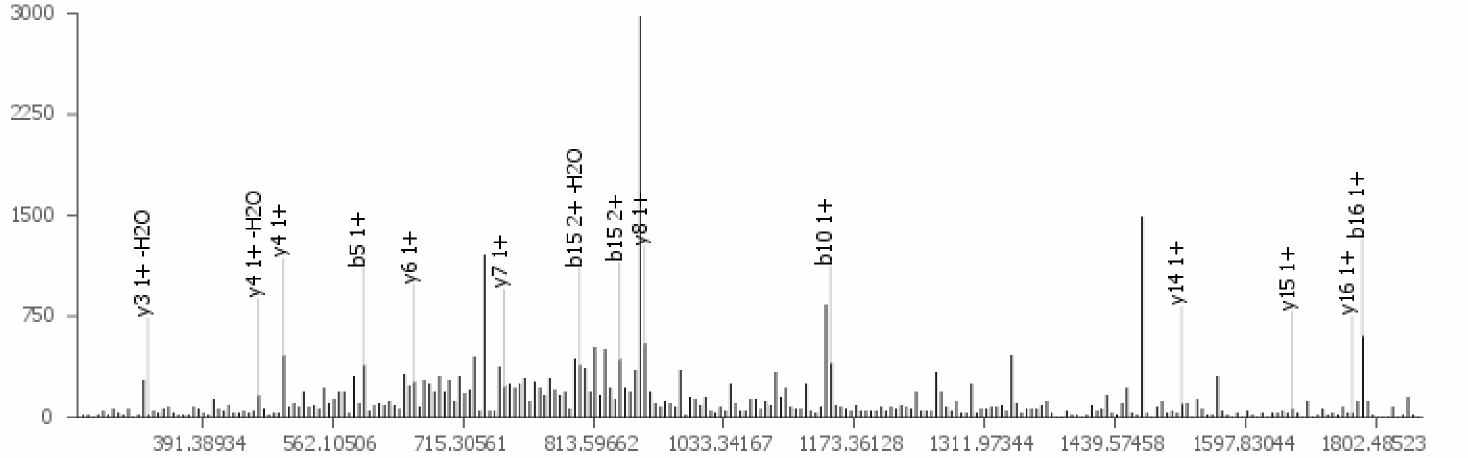

AT1G63070.1 - (pS)QL(pS)LALAILGK - 687.35187 - Charge:2

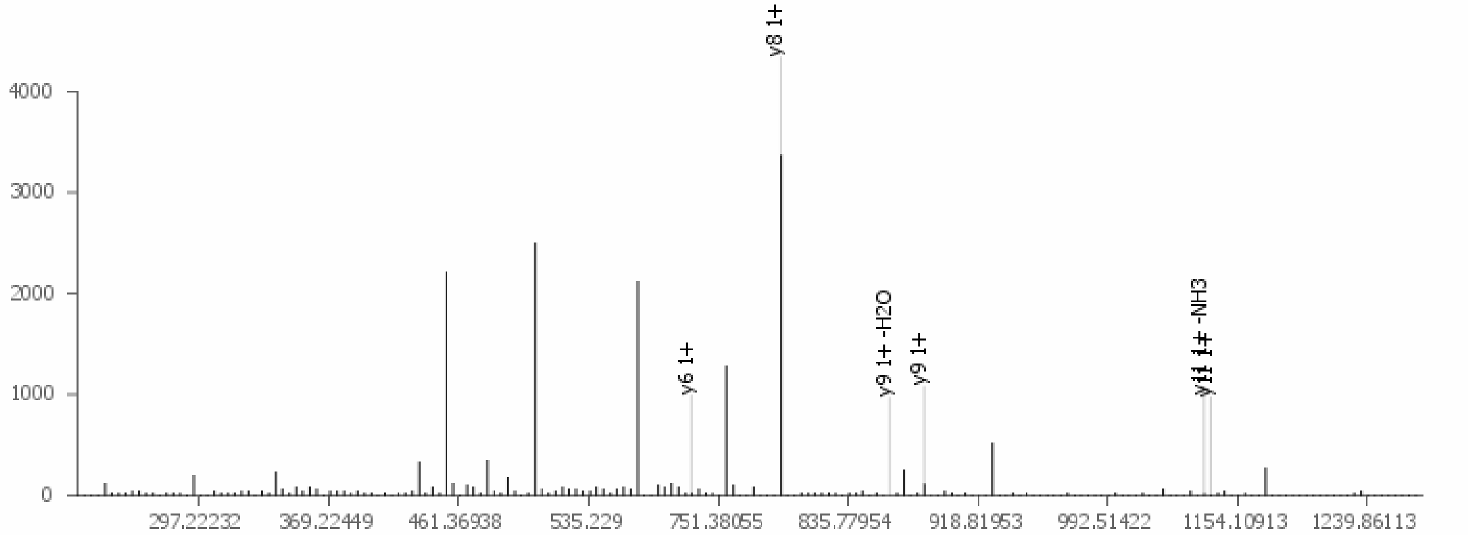

AT3G52390.1 - IIV(pT)GGSLEESR - 670.81999 - Charge:2

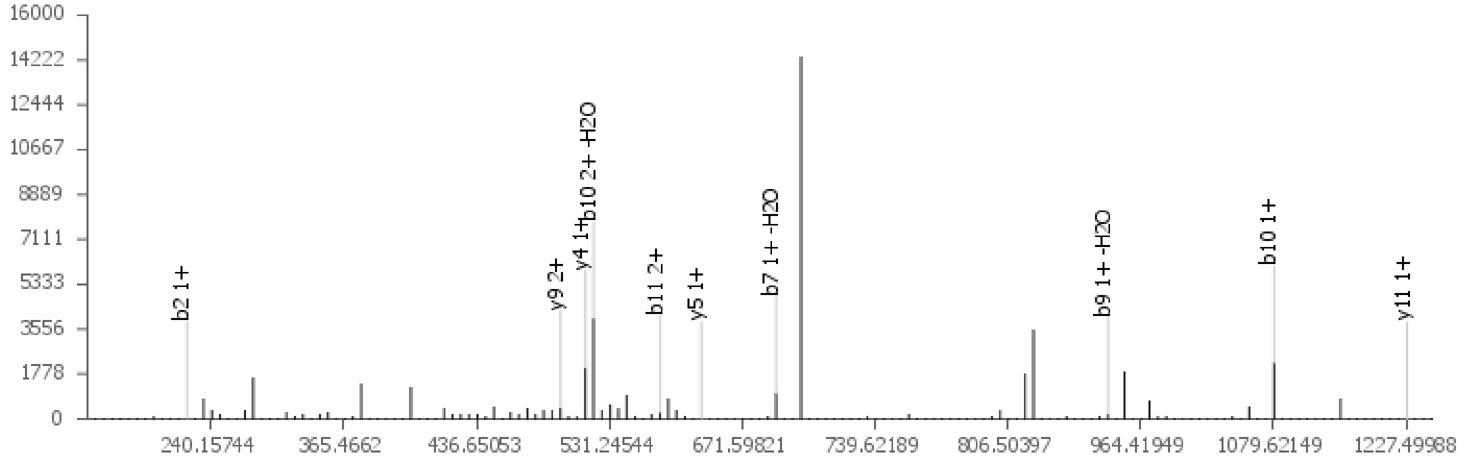

AT5G42800.1 - MTGWMYFVSK(pT)LAEK - 936.43183 - Charge:2

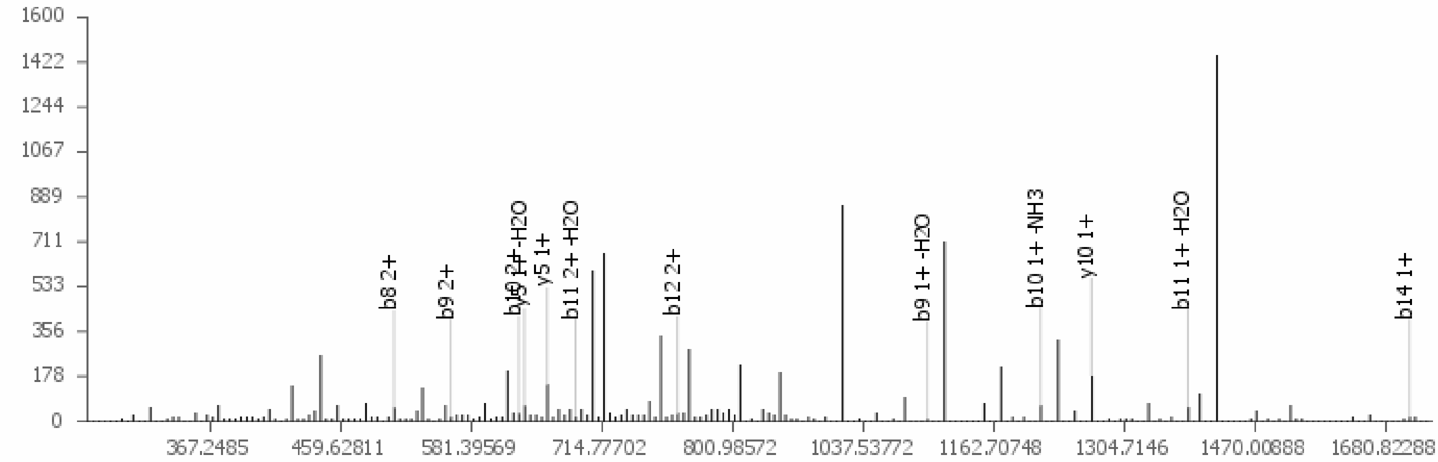

AT5G39320.1 - FDWDHPLHLQPM(s)P(t)(t)VK - 1115.00881 - Charge:2

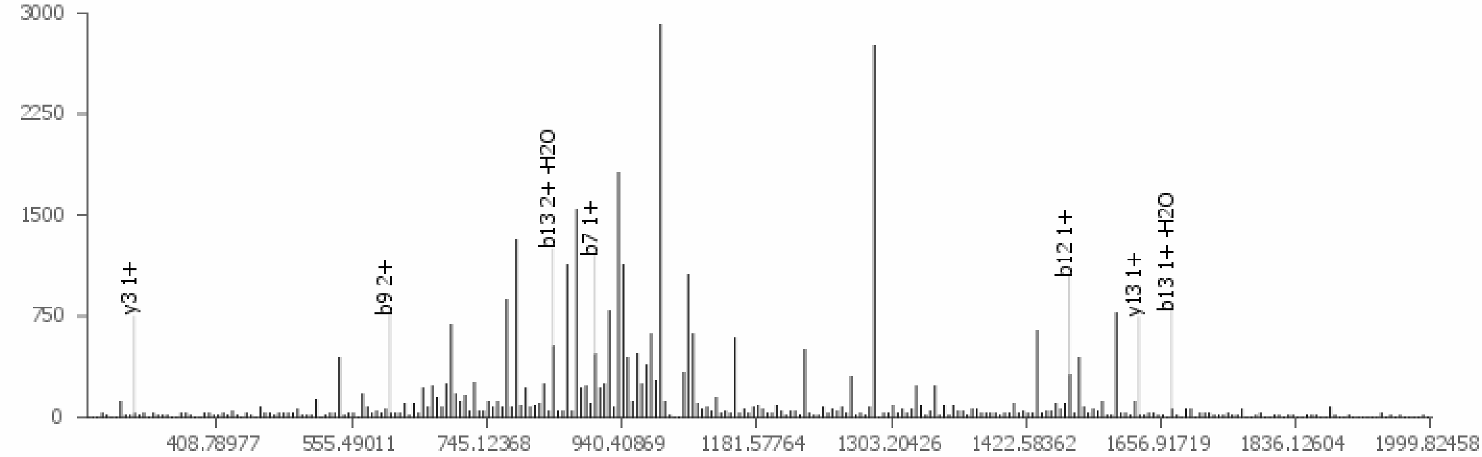

AT5G54820.1 - I(pS)GEVISRIINDLR - 872.92425 - Charge:2

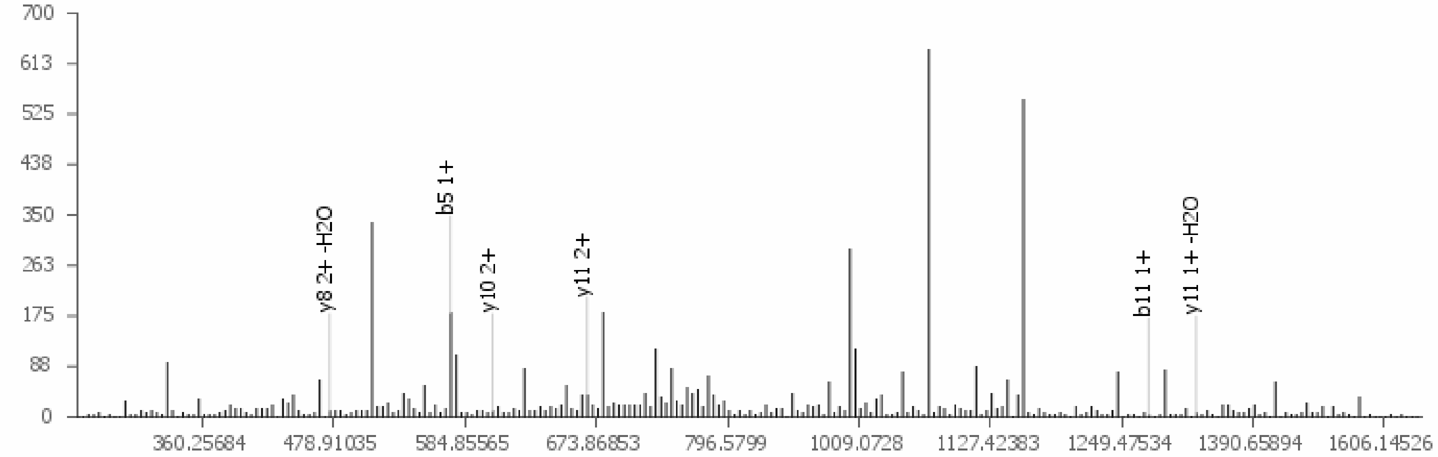

AT4G29810.1 - IISQLEPEVL(pS)PIKPADDQLSLSDLD(oxM)VK - 1097.21354 - Charge:3

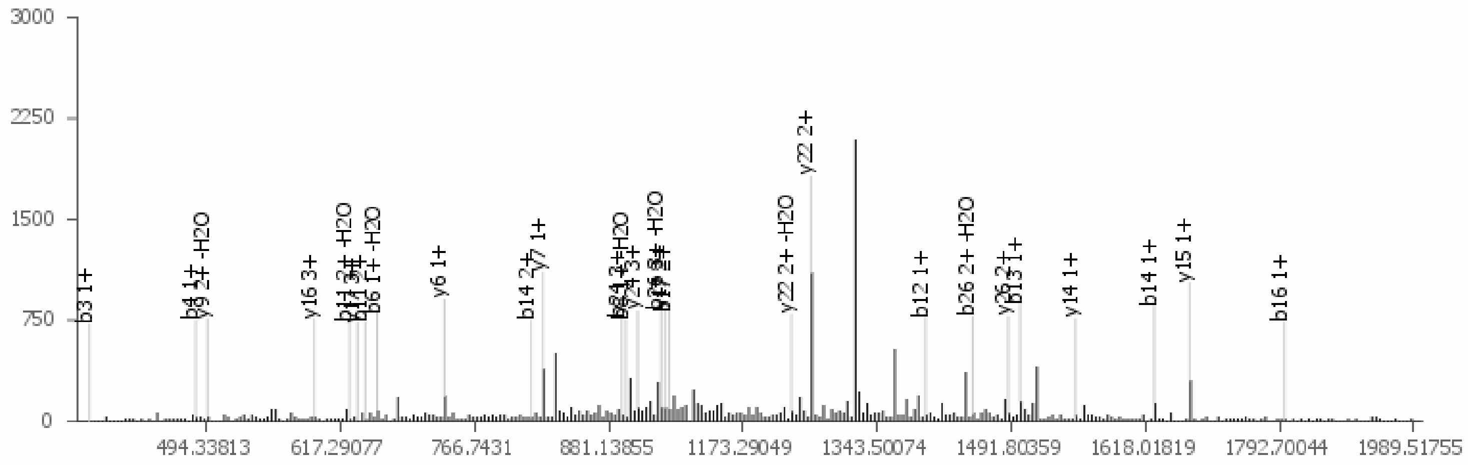

AT5G67385.1 - AEA(pY)LNEVALK - 650.81867 - Charge:2

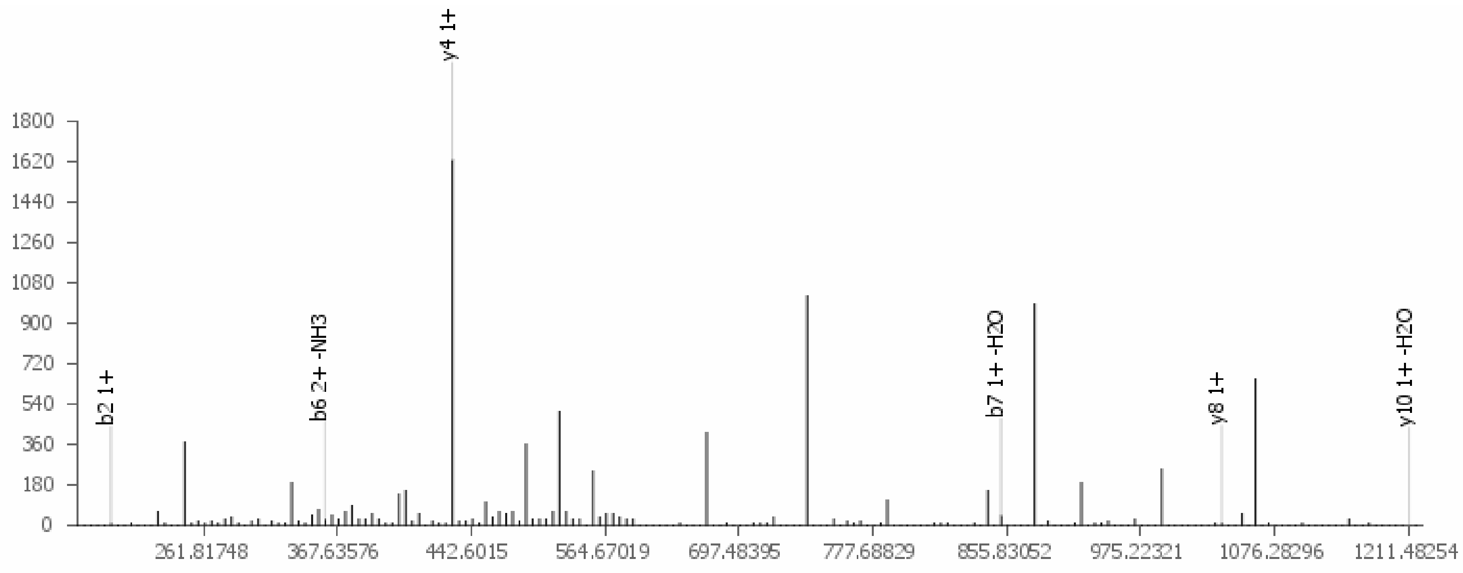

AT1G53310.1 - MA(pS)IDVHLR - 561.2567 - Charge:2

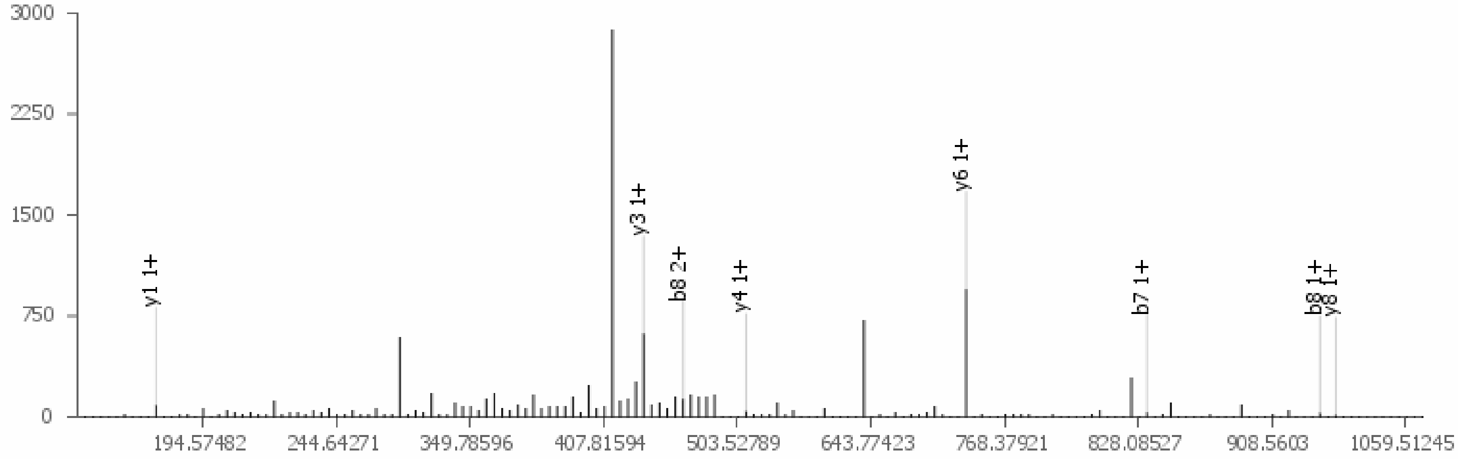

AT2G27710.1 - EE(pS)DDDMGFSLFE - 800.76857 - Charge:2

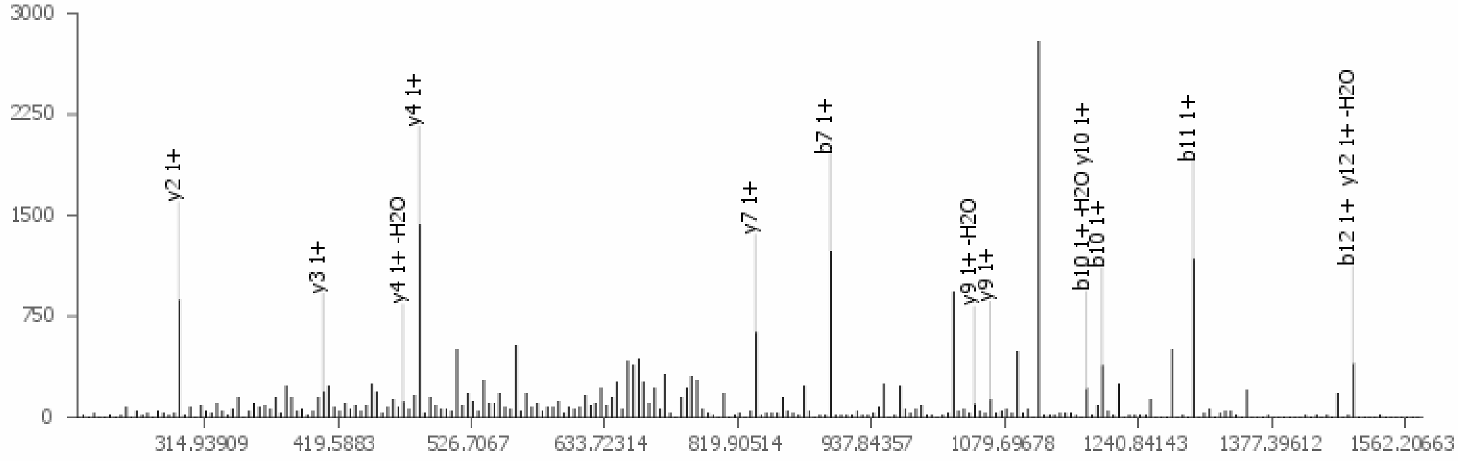

AT1G10520.1 - LLAESKG(pY)R - 558.77327 - Charge:2

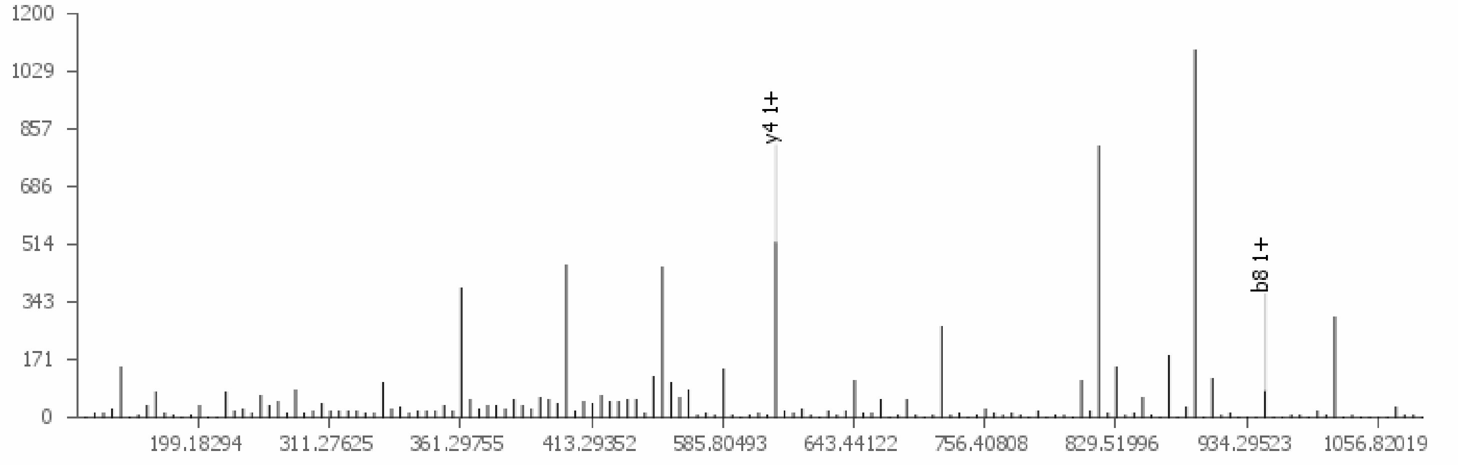

Supplement: Supplementary file 8 [file tpj0069-0978-SD8.pdf]
