# Supplementary material for: Nitrate and ammonium lead to distinct global dynamic phosphorylation patterns when resupplied to nitrogen-starved Arabidopsis seedlings
Source: Plant J. 2012 Jan 20;69(6):978–95. doi: 10.1111/j.1365-313X.2011.04848.x (PMC3380553; doi:10.1111/j.1365-313X.2011.04848.x)
Supplement: Supplementary file 9 [file tpj0069-0978-SD9.rtf]

Supporting Information Legends


Figure S1: Venn diagram of the distribution phosphopeptides to the different treatments. (A) All unique phosphopeptides sequences identified in the different experiments. (B) Unique phosphopeptides that could be quantified at least at three different time points. 

Figure S2: NRT2.1 phosphorylation and nitrate uptake (A) Targeted analysis of NRT2.1 phosphorylation under different concentrations of nitrate resupply for ten minutes. Normalized ion intensities of the phosphorylated form (red) and non-phosphorylated form (gray) are plotted as average of three biological replicates (B) 15N-Nitrate uptake of seedlings resupplied with different concentrations of nitrate under conditions of high- and low-affinity nitrate uptake.

Figure S3: Venn diagram of the identified phosphopeptides under nitrate and ammonium resupply and the respective non-phosphorylated peptides identified under the same conditions in the same respective samples.

Figure S4: Phosphorylation time course profiles for phosphopeptides identified from glutamine synthase 1 isoforms and total GS enzymes activitiy. (A) Phosphorylation time course for GS1.1 (B) phosphorylation time course for GS1.2, (C) phosphorylation time course for GS1.3 (D) Total activity of glutamine synthase in nitrate and ammonium resupplied seedlings.

Figure S5: Boxplot of the relative standard deviation of all quantified peptides averaged over all 12 replicated experiments. 

Figure S6: Overlap of proteins found with change in phosphorylation and with significant changes in transcript levels upon nitrate resupply after nitrogen starvation.

Figure S7: all phosphopeptide spectra as exported from the respective entry in the public database PhosPhAt (http://phosphat.mpimp-golm.mpg.de)

Table S1: List of all phosphopeptides. The first worksheet contains the mass spectrometric evidence for each phosphopeptide by listing mass to charge ratio, MASCOT identification score, PTM score. The second worksheet contains the normalized ion intensities used for quantitation as well as additional information about sequence motifs, or putative MAP-kinase substrates.

Methods S1: Glutamine synthase acitivity; targeted analysis of protein phosphorylation by single reaction monitoring; nitrate uptake assays.
